# Genomic surveillance of SARS-CoV-2 in North Africa: 4 years of GISAID data sharing

**DOI:** 10.1016/j.ijregi.2024.100356

**Published:** 2024-03-19

**Authors:** Zaineb Hamzaoui, Sana Ferjani, Ines Medini, Latifa Charaa, Ichrak Landolsi, Roua Ben Ali, Wissal Khaled, Sarra Chammam, Salma Abid, Lamia Kanzari, Asma Ferjani, Ahmed Fakhfakh, Dhouha Kebaier, Zoubeir Bouslah, Mouna Ben Sassi, Sameh Trabelsi, Ilhem Boutiba-Ben Boubaker

**Affiliations:** 1 Faculty of Medicine of Tunis, University of Tunis El Manar, Tunis, Tunisia; 2 National Center Chalbibelkahia of Pharmacovigilance of Tunis, Laboratory of Clinical Pharmacology, Tunis, Tunisia; 3 Laboratory of Microbiology, Charles Nicolle Hospital, Tunis, Tunisia; 4 University of Tunis El Manar, Faculty of Medicine of Tunis, Tunis Tunisia

**Keywords:** SARS-CoV-2, North Africa, Variants, Lineages, Epidemiological dynamics, Genomic sequences

## Abstract

**Objectives:**

This study aimed to construct geographically, temporally, and epidemiologically representative data sets for SARS-CoV-2 in North Africa, focusing on Variants of Concern (VOCs), Variants of Interest (VOIs), and Variants Under Monitoring (VUMs).

**Methods:**

SARS-CoV-2 genomic sequences and metadata from the EpiCoV database via the Global Initiative on Sharing All Influenza Data platform were analyzed. Data analysis included cases, deaths, demographics, patient status, sequencing technologies, and variant analysis.

**Results:**

A comprehensive analysis of 10,783 viral genomic sequences from six North African countries revealed notable insights. SARS-CoV-2 sampling methods lack standardization, with a majority of countries lacking clear strategies. Over 59% of analyzed genomes lack essential clinical and demographic metadata, including patient age, sex, underlying health conditions, and clinical outcomes, which are essential for comprehensive genomic analysis and epidemiological studies, as submitted to the Global Initiative on Sharing All Influenza Data. Morocco reported the highest number of confirmed COVID-19 cases (1,272,490), whereas Tunisia leads in reported deaths (29,341), emphasizing regional variations in the pandemic's impact. The GRA clade emerged as predominant in North African countries. The lineage analysis showcased a diversity of 190 lineages in Egypt, 26 in Libya, 121 in Tunisia, 90 in Algeria, 146 in Morocco, and 10 in Mauritania. The temporal dynamics of SARS-CoV-2 variants revealed distinct waves driven by different variants.

**Conclusions:**

This study contributes valuable insights into the genomic landscape of SARS-CoV-2 in North Africa, highlighting the importance of genomic surveillance in understanding viral dynamics and informing public health strategies.

## Highlights

• Comprehensive analysis of SARS-CoV-2 genomic epidemiology and lineage dynamics.
• Lack of standardization in SARS-CoV-2 sampling methods in the region.
• Most of analyzed genomes lack essential clinical and demographic metadata.
• Identification of the GRA clade as predominant in North African countries.
• Distinct waves driven by different SARS-CoV-2 variants.

## Introduction

The global impact of the COVID-19 pandemic has not only underscored the interconnectedness of our world but has also necessitated unprecedented collaborative efforts in understanding and combating the causative agent, the SARS-CoV-2. As we delve into the fourth year of this ongoing pandemic, genomic surveillance has emerged as a pivotal tool in tracking the evolution of the virus and informing public health strategies [1].

The SARS-CoV-2 virus, as with other RNA viruses, has a high mutation rate, which has led to the emergence of various mutations and lineages. These mutations can affect the virus's transmissibility, severity, and immune evasion capabilities [2]. Continuous genomic surveillance is crucial to track the emergence and spread of these mutations and lineages to inform public health strategies and interventions [3].

The Global Initiative on Sharing All Influenza Data (GISAID) and other platforms, such as the National Center of Biotechnology Information, the World Health Organization (WHO) database, the COVID-19 Genomics UK Consortium, and Nextstrain, are crucial for sharing genomic data on SARS-CoV-2. GISAID, in particular, has been instrumental during the COVID-19 pandemic, enabling global collaboration and the development of public health strategies. In addition, the Africa Pathogen Genomics Initiative [4] provides genomic sequencing and technical support services to the countries in the region. These platforms and networks play a vital role in sharing genomic data and supporting disease surveillance efforts.

The first SARS-CoV-2 genome sequence was determined in January 2020 [5]. By September 2023, over 16 million genomes have been publicly shared on GISAID. Although the sequencing of cases has risen, high-income countries continue to outpace low- and middle-income nations in genomic surveillance efforts, creating disparities.

Tunisia initially faced challenges in genomic surveillance owing to limited resources. The absence of a national sequencing strategy hindered comprehensive surveillance until June 2020, when Federated Research Projects initiated whole genome sequencing through International Sequencing Partnerships. Despite progress, widespread genomic surveillance aligned with national guidelines remained challenging until January 2021. Triggered by the Alpha variant's emergence in the United Kingdom [6], the Ministry of Health launched a national sequencing strategy supported by international donations. The two-step sequencing involved S gene sequencing, followed by whole genome sequencing, collaborating with various laboratories for broad geographic coverage [7].

As the epidemic evolved, the strategy adapted for identifying variants of concern (VOCs). In 2023, a proactive approach included random sequencing of diverse cases, focusing exclusively on human-hosted viral dynamics and excluding animal or environmental samples.

The situation in Tunisia is not unique. In fact, other African countries have also faced challenges in their genomic surveillance efforts. However, there is evidence that the situation is improving, with more African SARS-CoV-2 genome sequences being submitted to the GISAID database [8,9].

The study aimed to provide a detailed analysis of SARS-CoV-2 genomic epidemiology and lineage dynamics in North Africa over 4 years, emphasizing the need for comprehensive and representative data sets. It investigates the sampling methods, sequencing technologies, patient statuses, and identifies significant virus clades and variants. The findings contribute to understanding the virus’ spread, evolution, and public health impacts in the region. The study also highlights the importance of timely molecular characterization of circulating strains and the challenges faced in North Africa, while contributing to global SARS-CoV-2 understanding and public health strategies.

## Material and methods

### Study design and data collection

On September 15, 2023, the GISAID platform (www.gisaid.org, accessed on September 15, 2023) was assessed. The SARS-CoV-2 genomic sequences and corresponding metadata from the EpiCoV database were downloaded. The following filters were applied: location: Africa/Mauritania, Morocco, Algeria, Tunisia, Libya, and Egypt; host: human; “complete” sequence.” The EpiCoV database defines a “complete genome” as sequences with more than 29,000 nucleotides. The whole genome of the entire data set was already available within the downloaded metadata.

The information collected included gender, age, patient status, specimen, vaccination status, collection date, sampling method, sequencing technology, GISAID clade, and PANGO lineage. The retrieved data were then collated in a Microsoft Excel sheet.

### Data analysis

Data analysis was performed using Microsoft Excel for Microsoft, version 16.0.5404.1002.

All the information regarding the number of cases, deaths, gender, age, patient status, specimen, vaccination status, collection date, sampling method, sequencing technology, and variant analysis was analyzed.

## Results

### Sequencing rates

A total of 10,783 viral genomic sequences obtained from six North African countries—Egypt (5044 sequences), Libya (178 sequences), Tunisia (2455 sequences), Algeria (880 sequences), Morocco (2168 sequences), and Mauritania (58 sequences)—were analyzed.

South Africa was the leading contributor to genomic sequencing in the African region, providing 32.2% of the total sequences, followed by Kenya with 7.6%. However, the North African nations, including Egypt (46.8%), Libya (1.7%), Tunisia (22.8%), Algeria (8.2%), Morocco (20.1%), and Mauritania (0.5%), collectively contributed 6.4% of the genomic sequences from Africa (Supplementary Figure 1).

Notably, the sequencing efforts vary across North African countries (Table 1). In Egypt, approximately 0.98% of COVID-19 samples have undergone sequencing. Algeria has demonstrated a sequencing rate of 0.32%, whereas Tunisia and Morocco follow with rates of 0.21% and 0.17%, respectively. The genomic surveillance efforts in Libya and Mauritania, however, are comparatively lower, with only 0.04% and 0.09% of cases sequenced, respectively. Although Egypt exceeds the suggested threshold of at least 0.5% for routine genomic surveillance [1], the other countries fall below this benchmark.

**Table 1:** Variability and genomic surveillance of SARS-CoV-2 in North Africa (based on data downloaded from Global Initiative on Sharing All Influenza Data per 15 September 2023).

	Sequencing percentages by country			Sampling methods										
	Total number of COVID-19–positive cases	Total number of samples sequenced	Percentage of COVID-19 samples sequenced (%)	Sentinel surveillance (influenza-like illness) (%)	Baseline surveillance (%)	Active surveillance (%)	Non-sentinel-surveillance (hospital) (%)	Random (%)	Breakthrough infection (%)	National SARS-CoV-2 genomic and variants surveillance program (%)	S gene dropout (%)	Same-patient sampling strategy (%)	Outbreak investigation (%)	Not mentioned (%)
Egypt	515,759	5044	0.98	0.4										99.6
Libya	507,187	178	0.04	19.7	0.6									79.8
Tunisia	1,151,126	2455	0.21		19.1	0.04	0.6	59.8						20.4
Algeria	271,494	880	0.32		0.1		0.8		0.1	54	0.8	0.3	43.9	43.9
Morocco	127,2490	2168	0.17		0.9	0.7					0.05	0.05	0.1	98
Mauritania	63,668	58	0.09											100

### Sampling methods

The sampling methods for SARS-CoV-2 in North African countries are not standardized, with the majority of countries lacking a clear strategy (Table 1). In Egypt, Libya, and Morocco, the majority of the reported cases (99.6%, 79.8%, and 98%, respectively) originated from unspecified sources (not mentioned category). In Algeria, 54% of cases were collected through the National SARS-CoV-2 genomic and variant surveillance program. Tunisia applies the baseline surveillance program to 19.1% of sampling cases, whereas 59.8% of cases are sampled randomly. Mauritania lacks detailed sampling information, with 100% of cases categorized as “not mentioned” (Table 1).

### Sequencing technology

In North African countries, SARS-CoV-2 genome sequencing extensively uses two technologies: Illumina and Oxford Nanopore. Illumina dominates in Egypt, Tunisia, and Libya, constituting 94%, 73.6%, and 99.5% of sequencing data, respectively. Conversely, Algeria and Morocco favor Oxford Nanopore, comprising 94.1% and 52.1%, respectively, of their sequencing outputs. Mauritania exclusively relies on Oxford Nanopore, contributing 100% of their data, with no use of Illumina in this context.

### Patient status

Since the onset of the COVID-19 pandemic, over 59% of the genomes from North African countries lack the corresponding clinical and/or demographic metadata submitted to GISAID (Table 2).

**Table 2:** Clinical metadata including patient status, vaccination status, gender distribution, and sex ratio (based on data downloaded from Global Initiative on Sharing All Influenza Data per 15 September 2023).

Country	Patient status					Vaccination status			Gender distribution			Sex ratio
	Hospitalized (%)	Released (%)	Alive (%)	Deceased (%)	Unknown (%)	Vaccinated (%)	Not Vaccinated (%)	Not mentioned (%)	Male (%)	Female (%)	Unknown (%)	
Egypt	22	6.4	23.8	0.06	47.7	6.9		93.1	18.9	17.1	64	1.1
Libya	1.1	0.6	17.9		80.3			100	11.8	7.9	80.3	1.5
Tunisia	2.4	0.2	16.9	0.6	79.7	0.1	13.2	86.7	44.2	53.6	2.2	0.8
Algeria	1.2		63.7		35	0.3		99.7	45.7	52	2.3	0.9
Morocco	2.1	6.5	17.4		74	0.3		99.7	44.5	43.3	12.3	1.0
Mauritania	13.8	6.9	67.2		12.1			100	63.8	36.2		1.8
**Total**	**11.5**	**4.4**	**24.4**	**0.2**	**59.6**	**3.3**	**3**	**93.7**	**32.1**	**33.5**	**34.4**	**1.0**

Assessing the vaccination status of SARS-CoV-2 patients in North African nations reveals a concerning pattern of missing data. Across the region, information regarding vaccination status is notably absent (93.7% of total cases), with a substantial majority of cases remaining unaccounted for (Table 2). In Egypt, only 6.9% of cases mention a vaccination status, leaving a significant 93.1%, in whom the status remains undisclosed. Libya stands out as a unique case, with 100% of cases having no mention of their vaccination status, making the landscape even more enigmatic. Tunisia offers a glimpse, with 0.1% of cases being vaccinated and 13.2% not vaccinated, yet a staggering 86.7% falls into the category of “not mentioned.” Algeria and Morocco share a similar trend, with 0.3% of cases reported as vaccinated, whereas an overwhelming 99.7% lack this crucial information. Mauritania, like Libya, presents a perplexing scenario, with no data available on vaccination status for any of the reported cases (Table 2).

SARS-CoV-2 cases in North African countries show gender disparities. Egypt has a slightly higher incidence of cases in males, with a gender ratio of 1.1, and 64% of cases categorized as “unknown.” Libya has a male predominance, with a gender ratio of 1.5, and 80.3% of cases categorized as unknown. Tunisia, Algeria, and Morocco have a relatively balanced distribution, with similar gender ratios of 0.8, 0.9, and 1, respectively, and low percentages of cases categorized as unknown. Mauritania has a significant male predominance, with a gender ratio of 1.8, and no cases categorized as unknown (Table 2).

The majority of genomes sequenced in Egypt, Tunisia, Algeria, Morocco, and Mauritania were attributed to the 15-44 years age group, followed by the 45-64 years age group as the second largest. However, the situation differs for Libya because a significant portion of these metadata contain unknown categories, representing 80.3% of all Libyan data.

### SARS-CoV-2 epidemiology dynamics in North Africa

Between the onset of the COVID-19 pandemic and September 2023, North African countries have reported varying impacts from SARS-CoV-2. Morocco had the highest number of confirmed COVID-19 cases, with 1,272,490 cases, followed by Tunisia with 1,151,126 cases. In terms of COVID-19–related fatalities, Tunisia reported the highest number of deaths in the region, with 29,341, whereas Egypt had 24,812 deaths (https://covid19.who.int/, Supplementary Figure 2).

### Spatio-temporal distribution of the virus clades

Genomic sequences from North African countries were systematically categorized into GISAID clades. Worldwide, the GRA clade was the predominant lineage, accounting for 48.2% of cases, followed by the GK clade with 15.7% of cases. The other identified clades in the region were GR (10.5%), G (7.7%), GH (7.5%), GRY (6.6%), O (1.8%), GV (0.9%), S (0.8%), L (0.3%), and V (0.02%).

**Figure 1:**
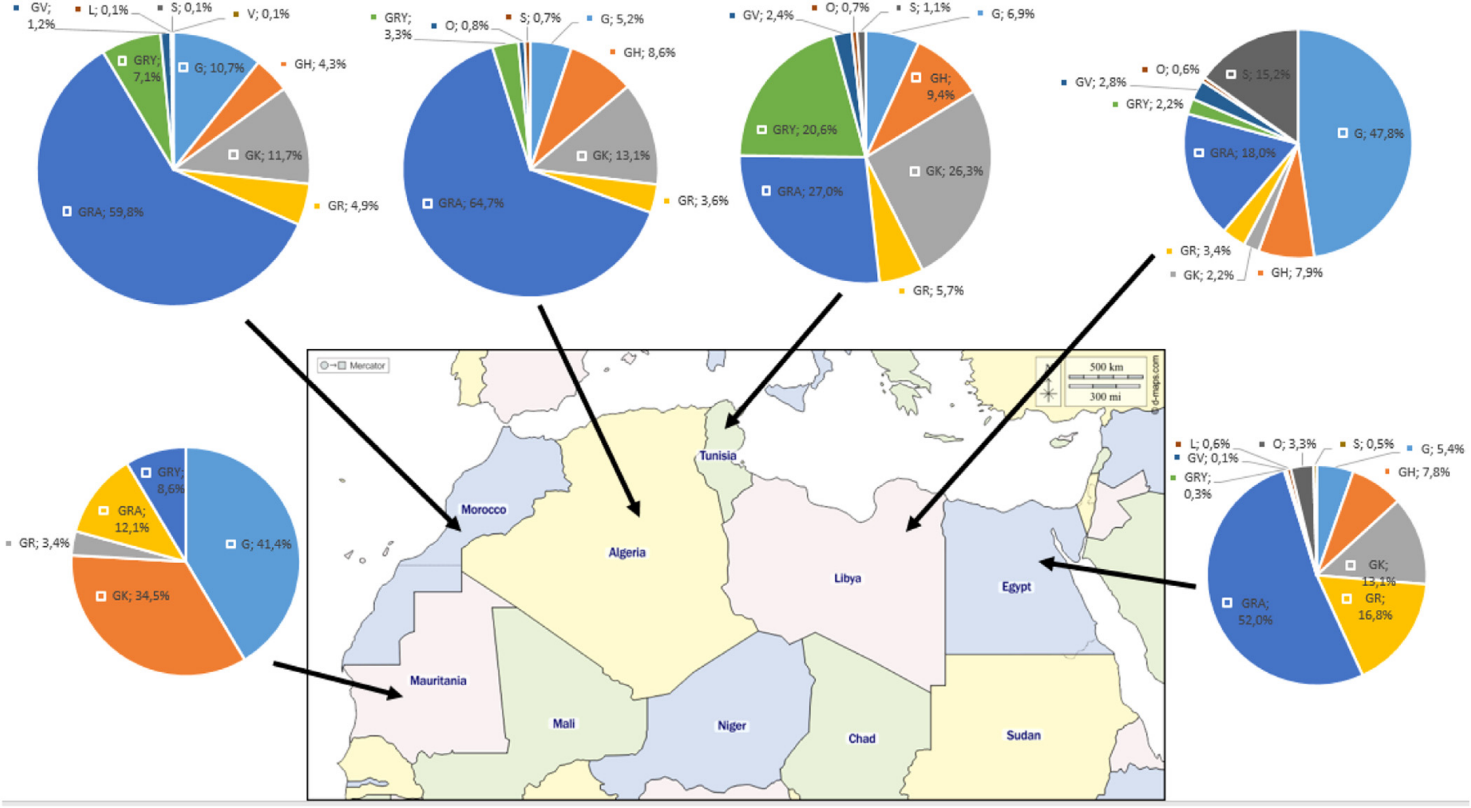
Regional variation in SARS-CoV-2 clade distribution across North African countries (based on data downloaded from Global Initiative on Sharing All Influenza Data per 15 September 2023).

The prevalent clade differed among nations. Notably, the GRA clade was dominant in Algeria (64.7%), Morocco (59.8%), Egypt (52%), and Tunisia (27%). Conversely, Libya and Mauritania exhibited G as the prevailing clade, constituting 47.8% and 41.4%, respectively (Figure 1).

### Distribution of SARS-CoV-2 lineages

**Figure 2:**
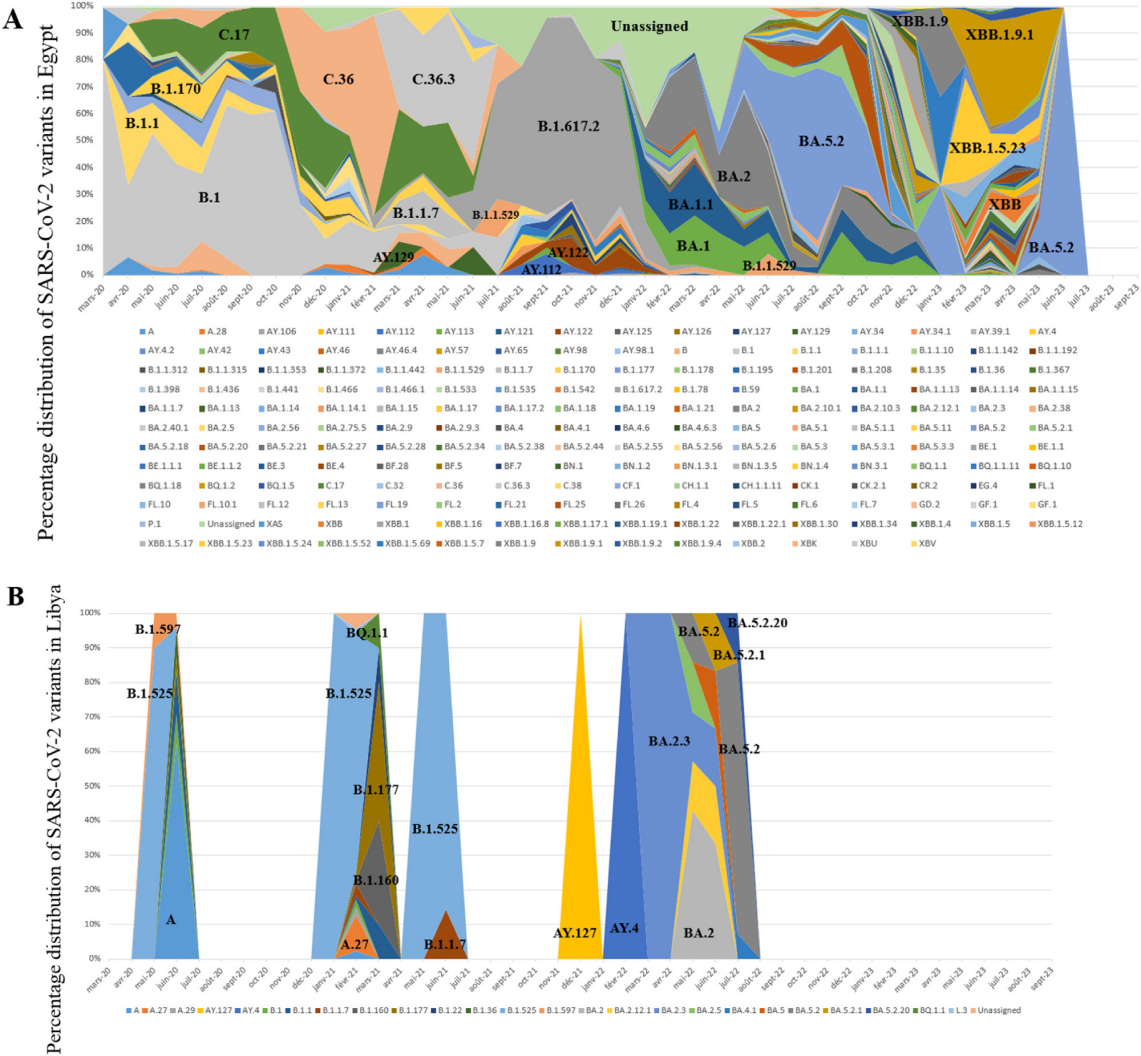

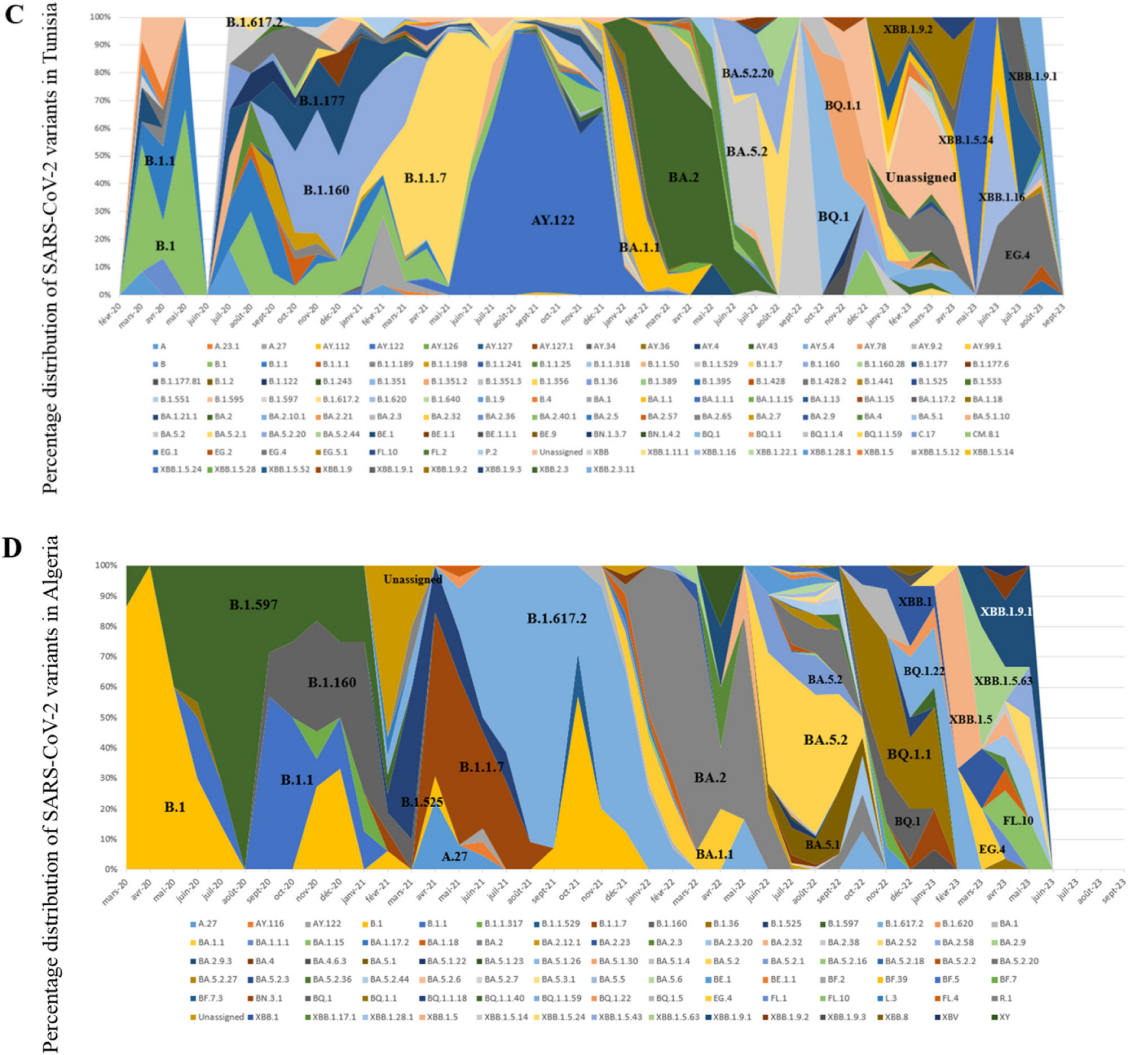

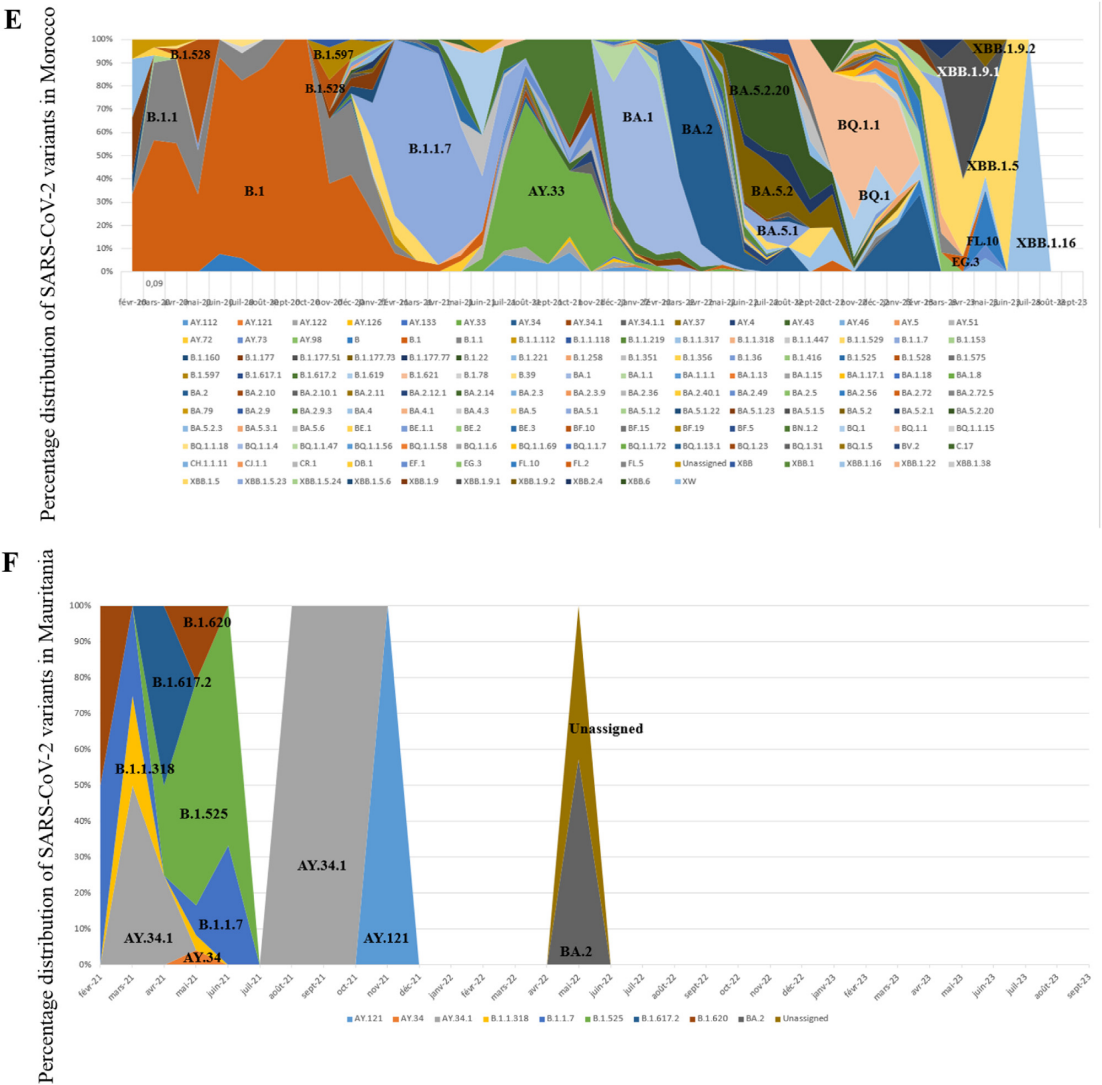
Relative distribution of SARS-CoV-2 lineages in North African countries (February 2020 to September 2023) Based on whole genome sequencing (based on data downloaded from Global Initiative on Sharing All Influenza Data per 15 September 2023). The diagram illustrates the relative distribution (%) of SARS-CoV-2 lineages in North African countries. Different lineages are color-coded, and those with higher proportions are marked in black font for easy identification. (a) Represents the distribution over 5044 sequences obtained in Egypt. (b) Illustrates the distribution over 178 sequences in Libya. (c) Shows the distribution over 2455 sequences in Tunisia. (d) Depicts the distribution over 880 sequences in Algeria. (e) Displays the distribution over 2168 sequences in Morocco. (f) Represents the distribution over 58 sequences in Mauritania.

In this study, all SARS-CoV-2 genome sequences were classified into Pango lineages, and their distribution was analyzed over the study period (Figure 2). The sequences in North African countries can be classified into 190 different lineages in Egypt, 26 in Libya, 121 in Tunisia, 90 in Algeria, 146 in Morocco, and 10 in Mauritania.

Distinct lineage dynamics have unfolded during different phases of the pandemic. In the initial wave, characterized as the pre-VOC phase, the B.1 lineage emerged as the predominant variant in Egypt, Tunisia, Algeria, and Morocco. However, with the advent of the VOC Alpha-driven wave, the genomic landscape witnessed a transition, marked by the dominance of the B.1.1.7 lineage across the region.

Subsequently, during the wave associated with the VOC Delta, a nuanced pattern emerged. In Egypt and Algeria, the B.1.617.2 lineage was the dominant variant. Conversely, during the same period, Libya reported the AY.4 lineage as the dominant variant, Tunisia exhibited dominance of AY.122, Morocco of AY.33, and Mauritania of AY.34.1.

Concerning the Omicron VOC, a significant lineage diversity was observed.

Examining the most abundant lineages, in Egypt, BA.5.2 (13.75%) was the most prevalent, followed by B.1.617.2 (8.74%) (Figure 2a); in Libya, B.525 (44.94%) was the dominant lineage, followed by A (9.55%) (Figure 2b); in Tunisia, AY.122 (24.23%) was the most prevalent, followed by B.1.1.7 (21.67%) (Figure 2c); in Algeria, BA.5.2 (17.27%) was the dominant lineage, followed by B.1.617.2 (12%) (Figure 2d); in Morocco, BA.1 (10.56%) was the dominant lineage, followed by B.1 (10%) (Figure 2e); in Mauritania, B.1.525 (31.03%) was the dominant lineage, followed by AY.34.1 (27.58%) (Figure 2f).

### Temporal dynamics of SARS-CoV-2 variants

**Figure 3:**
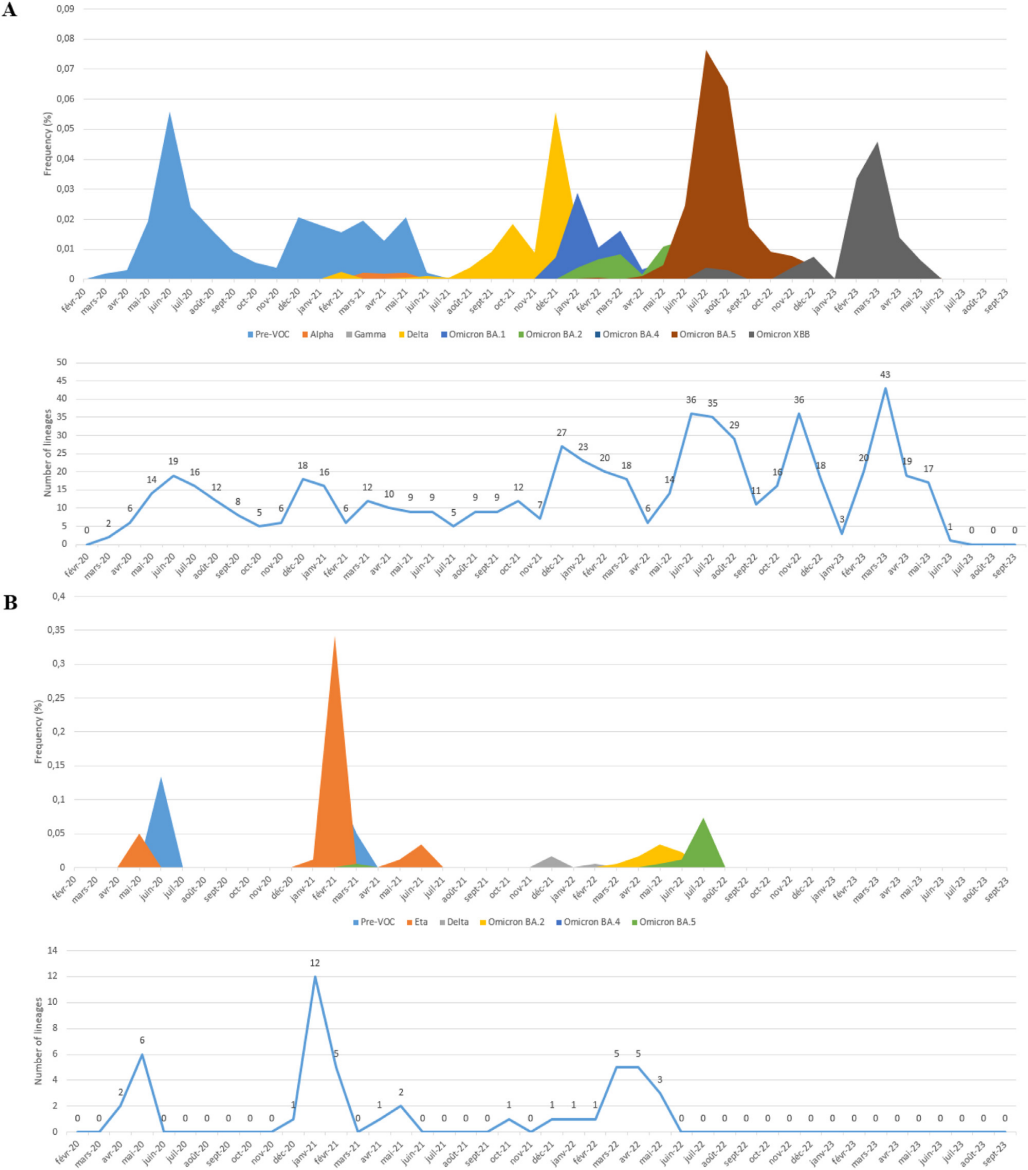

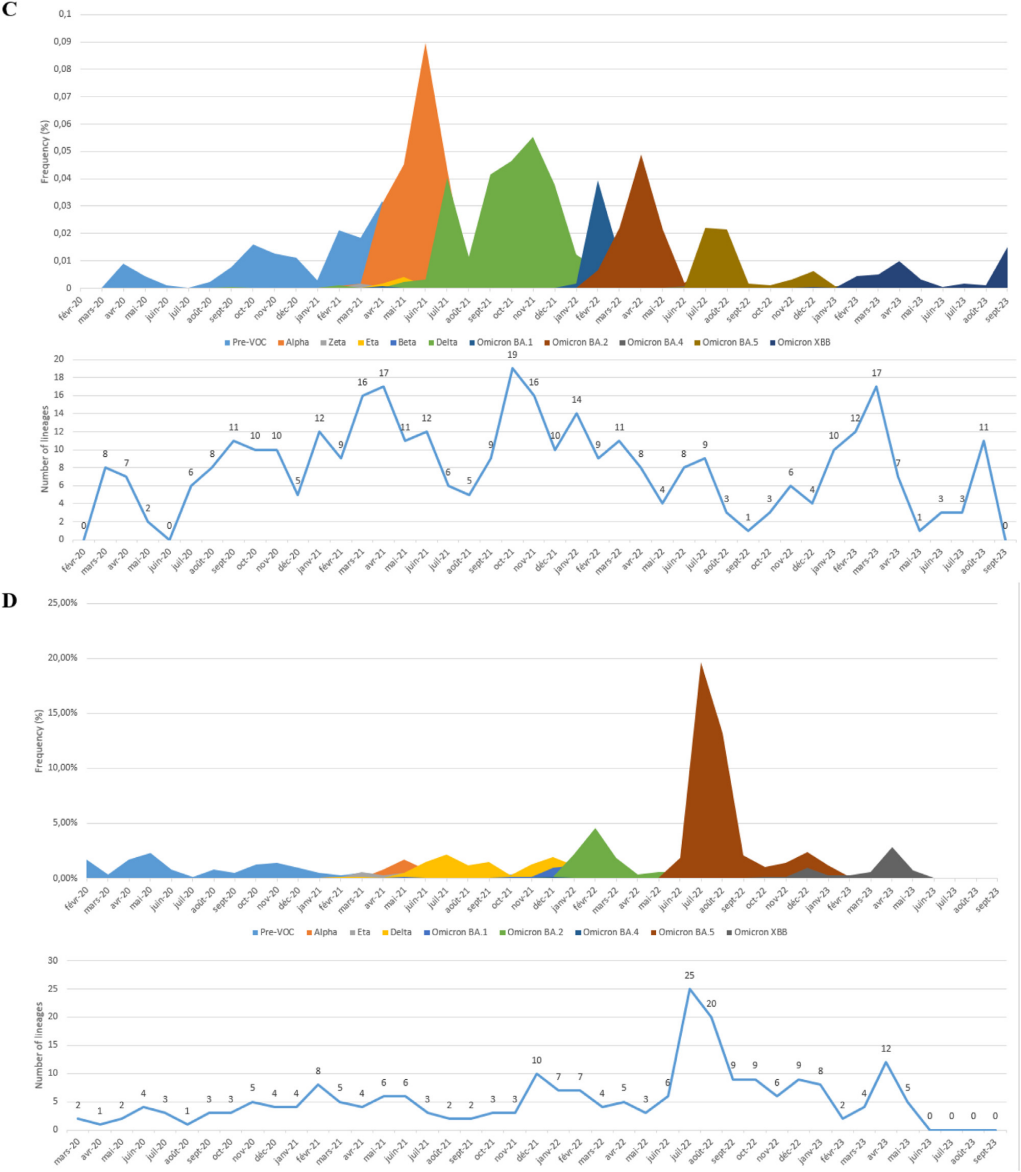

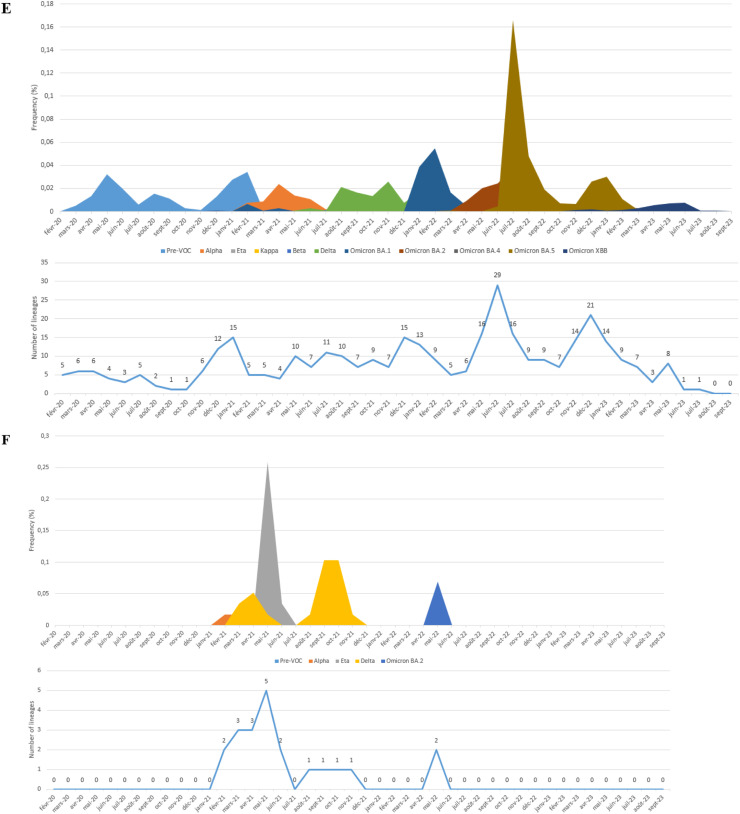
Chronological distribution of Pango lineages among all SARS-CoV-2 genomic sequences from North African countries, retrieved from Global Initiative on Sharing All Influenza Data (accessed on 15 September 2023) spanning from February 2020 to September 2023. The upper graph illustrates the frequency (proportion) of sequences for the most prevalent lineages in North African countries per month). The lower graph presents the absolute number of identified SARS-CoV-2 lineages per month. A. Egypt. B. Libya. C. Tunisia, D. Algeria. E. Morocco. F. Mauritania.

The chronological progression of variants observed across North African countries is illustrated in Figure 3. In these countries, the initial wave, emerging in late 2020, was primarily fueled by pre-variant lineages. However, a shift occurred in Tunisia, Algeria, and Morocco in early 2021 with the advent of the Alpha variant (Figures 3c, d, and e), a VOC characterized by specific point mutations associated with increased transmissibility and disease severity. Interestingly, this variant did not trigger a wave in Egypt (Figure 3a) and did not dominate in Libya and Mauritania (Figures 3b, and f), where the Eta variant was dominant. Toward the close of 2021, a new wave has been driven by the rapid dissemination of the Delta variant, which has completely supplanted the previously circulating VOC. The dominance of the Delta variant endured until the end of 2021 across all North African countries (Figure 3) (for Libya, data availability was limited; Figure 3b). Early in 2022, all North African countries (excluding Mauritania owing to insufficient data; Figure 3f) encountered a fresh wave of infections sparked by the emergence of the Omicron strains. The Omicron BA.1 variant displaced the Delta variant in early 2022, marking the onset of this wave, followed by subsequent waves characterized by the prevalence of the Omicron BA.2 and Omicron BA.5 lineages (Figure 3). The emergence of recombinant virus forms in North Africa toward the end of 2022 marked the start of the endemic phase and new cycles of variant displacement. Throughout 2023, these recombinant forms progressively gained prominence, eventually becoming the predominant lineages by March 2023.

In addition, a meticulous analysis of viral lineages was conducted, revealing distinct patterns through a monthly classification. Notably, with each wave, the number of observed lineages exhibited a consistent trend of initial increase, followed by a subsequent decline after the peak of the wave, only to rise again with the onset of a new variant-driven surge. During the study period, the highest recorded diversity of lineages occurred in Egypt, with 43 different lineages. All were identified during the Omicron XBB variant wave (Figure 3a). In Libya, during the Eta variant surge, 12 distinct lineages were observed (Figure 3b), whereas the Delta variant wave in Tunisia witnessed 19 lineages (Figure 3c). Algeria and Morocco experienced 25 and 29 different lineages, respectively, during the Omicron BA.5 variant surge (Figures 3d, and e). Mauritania saw five distinct lineages during the Eta variant wave (Figure 3f).

### Prevalence of SARS-CoV-2 variants

The genomic sequences of SARS-CoV-2 in North African countries align predominantly with the WHO-defined category of VOCs. In Egypt, Omicron emerges as the dominant VOC, constituting 55.3% of the sequences, followed by Delta at 12.1%. VOCs Alpha and Gamma exhibit frequencies below 1%, with no recorded Beta sequences. Notably, previously circulating Variants of Interest (VOI), such as XBB.1.5 (3.7%) and XBB.1.16 (0.1%), were identified, whereas Kappa, Eta, and Iota were absent among the Egyptian isolates (Supplementary Figure 3).

Conversely, Libya faces data limitations, hindering a comprehensive analysis. Eta, identified as a VOI, prevails at 44.9%, whereas VOCs such as Omicron, Delta, and Alpha constitute 17.4%, 2.2%, and 2.2%, respectively (Supplementary Figure 3). Tunisia exhibits a diverse landscape, with Omicron leading at 28.1%, followed by Delta (26.1%) and Alpha (21.7%). Sequences of Beta are below 1%, whereas Gamma is absent. Additional VOI, such as Zeta, XBB.1.5, and XBB.1.16, along with several Omicron sub-lineages classified as Variants Under Monitoring (VUM), have been identified (Supplementary Figure 3).

In Algeria, Omicron was predominant at 65.3%, followed by Delta at 12.3%. Beta and Gamma sequences are not recorded, whereas Eta and XBB.1.5, as VOIs, are present at 1.8% and 1.5%, respectively (Supplementary Figure 3). Morocco mirrors a similar pattern, with Omicron leading at 61.4%, Delta at 11.9%, and no recorded Gamma sequences. Previously circulating VUM, such as Omicron XBB.1.9.1, is noted at 1.4% (Supplementary Figure 3). Mauritania's data limitations complicate a detailed analysis; however, among the identified VOCs, Delta prevails at 34.5%, followed by Omicron at 12.1% and Alpha at 8.6%. The predominant VOI is Eta at 31%, with no additional available data (Supplementary Figure 3).

## Discussion

Compared with other continents, Africa's overall genomic contribution appears relatively modest (1.06%), with Asia, Europe, and North America accounting for a substantial majority of the global genomic data set at 10.29%, 49.12%, and 35.15%, respectively (www.gisaid.org, accessed on September 15, 2023).

The sequencing rate varies considerably across African countries. In fact, South Africa and Kenya demonstrate a higher sequencing rate relative to their positive cases (32.2% and 7.6%, respectively), whereas other countries have a more moderate contribution [10]. As the COVID-19 landscape continues to evolve, these sequencing rates in North Africa may offer valuable insights into the virus’ spread and adaptation, similar to observations made in other regions [11].

Genomic sequencing is vital for tracking the evolution and spread of SARS-CoV-2; optimizing tests, treatments, and vaccines; and guiding public health responses. Disparities in global genomic surveillance emphasize the need for equitable sharing of pathogen genomic data and resources [1]. In Africa, the expansion of genomic surveillance, including increased domestic sequencing, enables faster detection of new variants and informs tailored public health strategies [12]. However, challenges, such as the lack of a standardized sequencing strategy and incomplete clinical and demographic metadata, need to be addressed to enhance the effectiveness of genomic surveillance in the region. To address these challenges, there is a need for sustained investment in pandemic preparedness, equitable access to scientific technology, and strengthening global health security through geographically representative genomic surveillance [13]. Regional approaches, such as those implemented by the WHO through its regional offices, play a crucial role in supporting countries to strengthen genomic surveillance systems and address knowledge gaps to enhance country-led efforts in pathogen sequencing [13,14].

The lack of a standardized clear strategy for SARS-CoV-2 sequencing in North African countries poses a significant challenge to comprehensive genomic surveillance. The absence of an uniform approach to sampling may lead to inconsistencies in the data collected, hindering the accurate assessment of the virus’ genomic landscape and the identification of emerging variants [11,12,15]. For instance, the high percentage of cases originating from unspecified sources in Egypt, Libya, and Morocco, as well as the lack of detailed sampling information in Mauritania, underscore the need for a more structured and systematic approach to sampling. Without standardized sampling strategies, it becomes increasingly difficult to capture a representative and diverse set of SARS-CoV-2 genomes, which is essential for understanding the virus’ evolution and informing public health responses. The African Union member states have developed practical guidance for implementing genomic SARS-CoV-2 surveillance, including advice on sampling and sample referral logistics, to detect and monitor VOCs [12].

The lack of clinical and demographic metadata submitted to GISAID for over 59% of the genomes from North African countries is a significant concern. The absence of this information hinders the ability to conduct a comprehensive analysis of the virus’ genomic landscape and its impact on different populations. It is highly likely that these metadata are stored within national repositories but are not reported to GISAID to comply with their respective data sharing regulations. Accessing clinical patient information in these countries is further complicated, possibly because of the absence of fully computerized systems in hospitals, making vital information nearly impossible to retrieve [16], [17], [18].

The lack of vaccination status information for a substantial majority of cases in the region, especially in Libya and Mauritania, is concerning. This absence complicates assessing vaccination program effectiveness and its impact on virus spread. A structured and systematic approach to data collection and sharing is crucial to enhance genomic surveillance efforts in the region [19].

The gender distribution variations in SARS-CoV-2 cases in North African countries highlight the potential influence of social and demographic factors on virus spread. The high percentage of cases categorized as unknown in some countries emphasizes the necessity for improved data collection and reporting practices to gain a more comprehensive understanding of the virus’ impact on diverse demographic groups.

The distribution of SARS-CoV-2 clades in North African countries reveals intricate dynamics shaping the virus’ genomic landscape. The diverse clade distribution reflects the complexity of the viral population and highlights unique genetic profiles in the region [20]. Notably, the GRA clade dominates in Algeria, Morocco, Egypt, and Tunisia, emphasizing its regional significance. In contrast, Libya and Mauritania exhibit the G clade as the prevailing lineage, indicating distinct genomic patterns. Analyzing the clade distribution is crucial for understanding SARS-CoV-2 transmission, severity, and potential immune evasion in the region, influenced by factors such as population movements, transmission dynamics, and regional interventions.

The distribution of SARS-CoV-2 lineages in North African countries, as revealed in this study, provides valuable insights into the epidemiological and evolutionary dynamics of the virus in the region. The emergence and dominance of specific lineages during the different phases of the pandemic, such as the pre-VOC phase and the waves associated with the VOC Alpha and VOC Delta, reflect the complex and evolving nature of the virus. The significant diversity of lineages observed concerning the VOC Omicron further underscores the ongoing evolution of SARS-CoV-2. The most prevalent lineages, such as BA.5.2 in Egypt, B.525 in Libya, AY.122 in Tunisia, BA.5.5 in Algeria, BA.1 in Morocco, and B.1.525 in Mauritania, highlight the unique genetic landscape of the virus in each country. These findings are consistent with the broader global trend of the continuous evolution and diversification of SARS-CoV-2 lineages and emphasize the importance of ongoing genomic surveillance to monitor the spread and impact of the virus. This study's results contribute to the growing body of knowledge on the genetic diversity and lineage dynamics of SARS-CoV-2 in North Africa, providing a foundation for further research and public health efforts in the region [11,[21], [22], [23], [24], [25]]. The distribution of lineages in Mauritania and Libya is seriously lacking in information, which highlights the need for improved data collection and reporting practices to ensure a more comprehensive understanding of the virus's impact on different demographic groups. In addition, the absence of COVID-19 cases in Tunisia during June 2020 is a notable finding that may reflect the effectiveness of public health measures implemented during that time.

The monthly classification of SARS-CoV-2 lineages reveals a dynamic pattern, with each successive wave showing an initial increase in observed lineages, followed by a decline after the wave's peak. This cyclic pattern suggests an interplay between viral evolution and population-level immunity, influenced by the introduction and spread of novel variants. It also highlights the continuous evolution and diversification of SARS-CoV-2 lineages, demonstrating the virus’ ability to adapt and generate new variants over time.

## Conclusion

Our comprehensive analysis of SARS-CoV-2 genomic data in North African countries offers valuable insights into the complex dynamics of the virus within the region. Despite Africa's relatively modest contribution to the global genomic data set, the varying sequencing rates across countries underscore the importance of continuous genomic surveillance in understanding the virus’ spread and adaptation.

Countries can strengthen the sampling methodologies by implementing broader and more representative sample collection strategies. In addition, addressing the gaps in metadata sharing through standardized protocols and enhanced collaboration between health care institutions and research entities is crucial. By improving sampling practices and metadata sharing mechanisms, countries can ensure more accurate and comprehensive genomic surveillance, thereby facilitating informed decision-making and effective public health interventions in response to the pandemic.

## Declarations of competing interest

The authors have no competing interests to declare.

## Appendix Supplementary materials

Image, application 1

Image, application 2

Image, application 3

Image, application 4

Image, application 5

Image, application 6

Image, application 7
