# Supplementary material for: Genomic surveillance of SARS-CoV-2 in North Africa: 4 years of GISAID data sharing
Source: IJID Reg. 2024 Mar 19;11:100356. doi: 10.1016/j.ijregi.2024.100356 (PMC11035039; doi:10.1016/j.ijregi.2024.100356)
Supplement: Supplementary file 1 [file mmc1.docx]

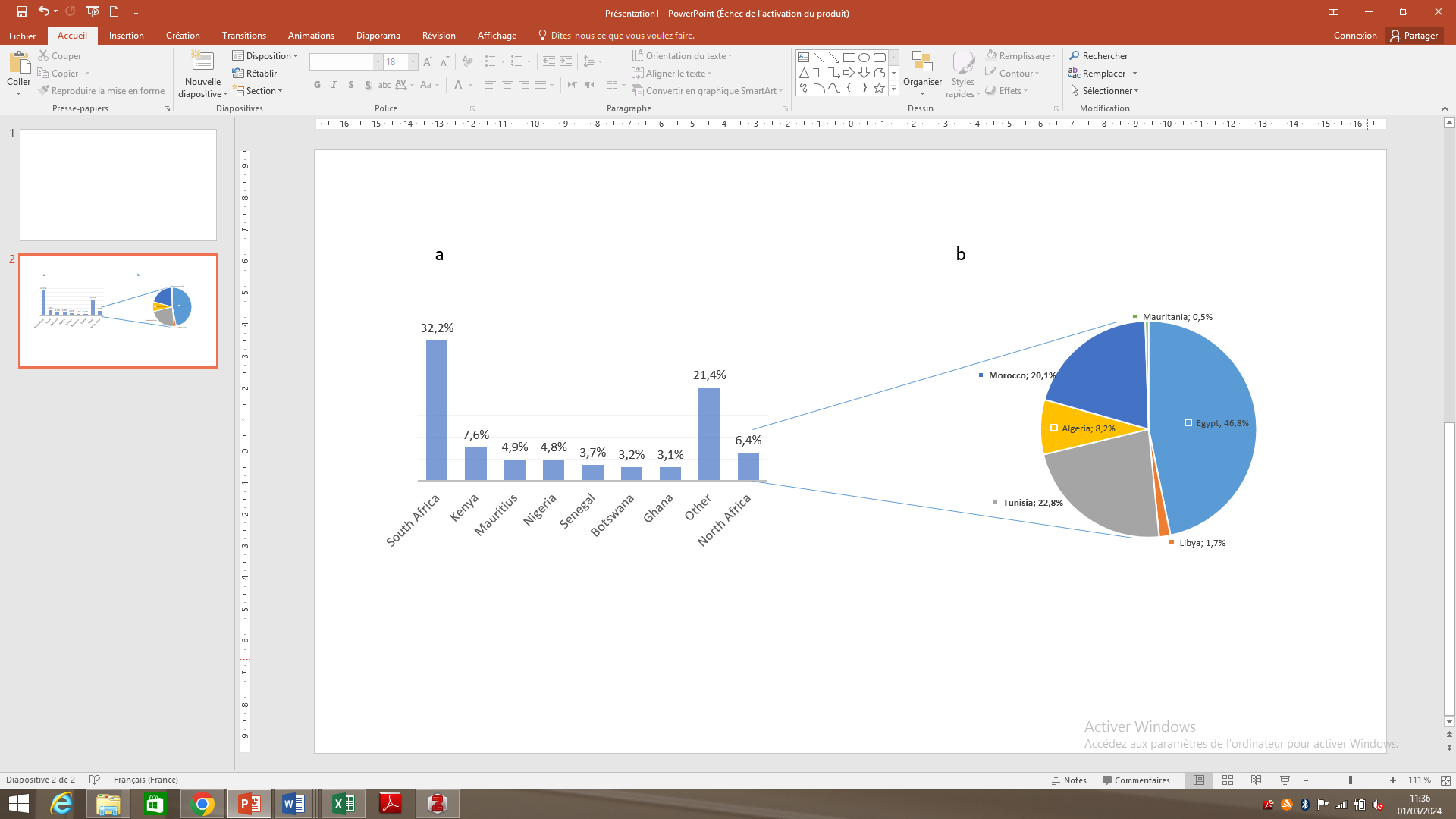


**FigureS1.** Profiles of SARS-CoV-2 Whole Genome Sequencing in (a). African Continent, and in (b). North African countries including Egypt, Libya, Tunisia, Algeria, Morocco, and Mauritania (based on data downloaded from GISAID per 15 September 2023).


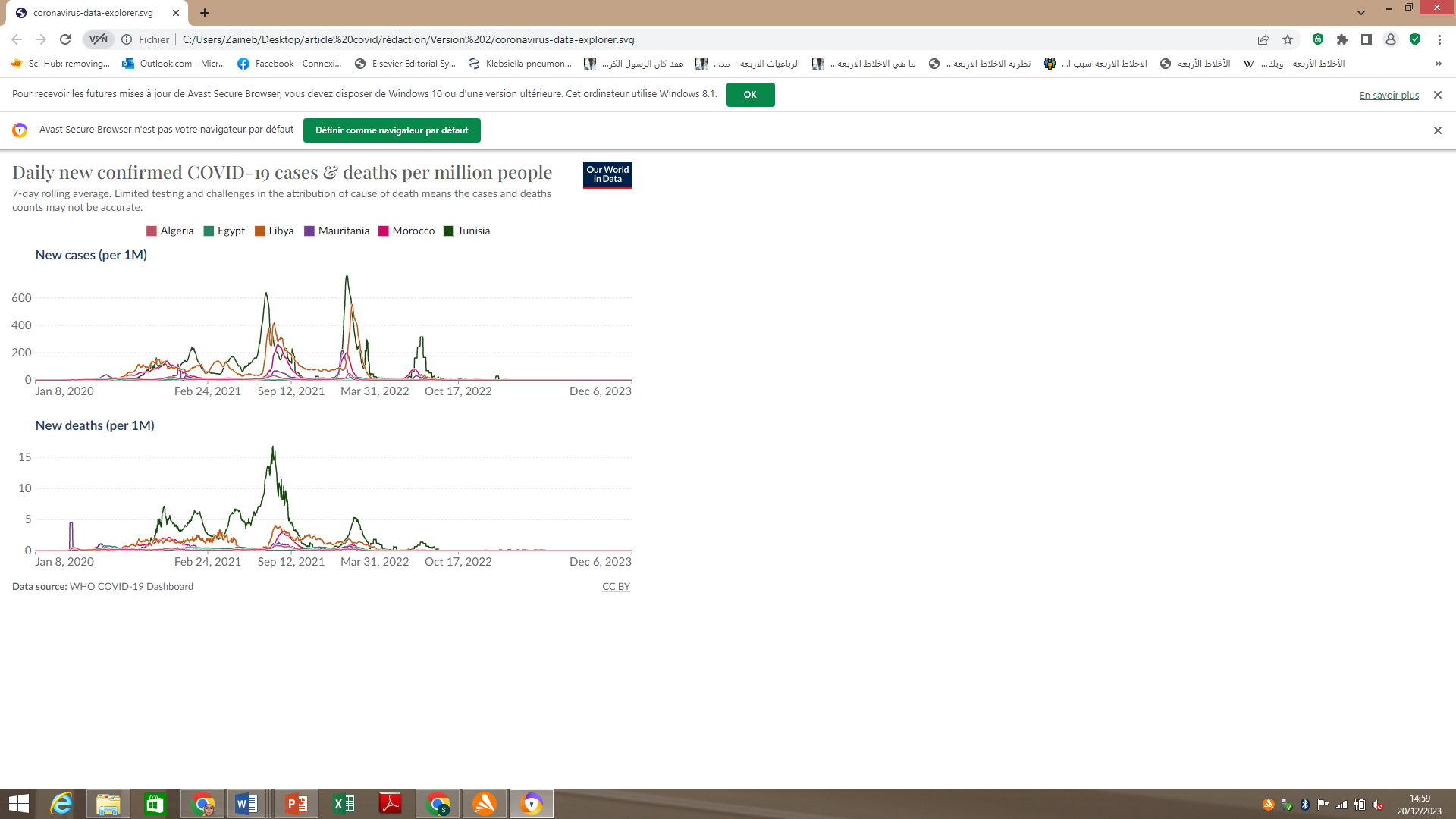


**Figure S2.** Daily new confirmed COVID-19 cases and deaths per million people in North African Countries (<https://covid19.who.int/> ).

**Figure S3.** Distribution of SARS-CoV-2 Variants in North African Countries (February 2020 – September 2023). Overview of the prevalence of SARS-CoV-2 variants in North African countries, highlighting the proportion of Variant of Concern (VOC), Variants of Interest (VOI), and Variants Under Monitoring (VUM) (based on data downloaded from GISAID per 15 September 2023). The chart depicts the percentage distribution of major variants, including Omicron, Delta, Alpha, Eta, and others, providing insights into the genomic landscape and variations across **A.** Egypt, **B.** Libya, **C.** Tunisia, **D.** Algeria, **E.** Morocco, and **F.** Mauritania.
