# Supplementary material for: Genomic surveillance of SARS-CoV-2 in North Africa: 4 years of GISAID data sharing
Source: IJID Reg. 2024 Mar 19;11:100356. doi: 10.1016/j.ijregi.2024.100356 (PMC11035039; doi:10.1016/j.ijregi.2024.100356)
Supplement: Supplementary file 2 [file mmc2.docx]

Table S5. Comprehensive Virus Data from Egypt Including Virus Name, Accession Number, and Clinical Attributes (based on data downloaded from GISAID per 15 September 2023)

| Virus name | Accession ID | Collection date | Location | Host | Sampling strategy | Gender | Patient age (years) | Patient status | Last vaccinated | Sampling strategy | Lineage | Clade |
| --- | --- | --- | --- | --- | --- | --- | --- | --- | --- | --- | --- | --- |
| hCoV-19/Egypt/NRC-03/2020 | EPI_ISL_430819 | 18/03/2020 | Africa / Egypt | Human | unknown | Female | 35 | Hospitalized | unknown | unknown | B.1 | GH |
| hCoV-19/Egypt/NRC-01/2020 | EPI_ISL_430820 | 18/03/2020 | Africa / Egypt | Human | unknown | Male | 5 | Hospitalized | unknown | unknown | B.1 | GH |
| hCoV-19/Egypt/CUNCI-HGC008/2020 | EPI_ISL_468044 | 02/05/2020 | Africa / Egypt | Human | unknown | unknown | unknown | unknown | unknown | unknown | B.1 | GH |
| hCoV-19/Egypt/CUNCI-HGC002/2020 | EPI_ISL_468045 | 02/05/2020 | Africa / Egypt | Human | unknown | unknown | unknown | unknown | unknown | unknown | B.1 | GH |
| hCoV-19/Egypt/CUNCI-HGC007/2020 | EPI_ISL_468046 | 02/05/2020 | Africa / Egypt | Human | unknown | unknown | unknown | unknown | unknown | unknown | B.1 | GH |
| hCoV-19/Egypt/CUNCI-HGC013/2020 | EPI_ISL_468047 | 02/05/2020 | Africa / Egypt | Human | unknown | unknown | unknown | unknown | unknown | unknown | B.1.201 | G |
| hCoV-19/Egypt/CUNCI-HGC004/2020 | EPI_ISL_468048 | 02/05/2020 | Africa / Egypt | Human | unknown | unknown | unknown | unknown | unknown | unknown | B.1 | G |
| hCoV-19/Egypt/CUNCI-HGC003/2020 | EPI_ISL_468049 | 02/05/2020 | Africa / Egypt | Human | unknown | unknown | unknown | unknown | unknown | unknown | B.1 | GH |
| hCoV-19/Egypt/CUNCI-HGC006/2020 | EPI_ISL_468050 | 02/05/2020 | Africa / Egypt | Human | unknown | unknown | unknown | unknown | unknown | unknown | B.1 | GH |
| hCoV-19/Egypt/CUNCI-HGC012/2020 | EPI_ISL_468051 | 02/05/2020 | Africa / Egypt | Human | unknown | unknown | unknown | unknown | unknown | unknown | B.1 | GH |
| hCoV-19/Egypt/CUNCI-HGC005/2020 | EPI_ISL_468052 | 02/05/2020 | Africa / Egypt | Human | unknown | unknown | unknown | unknown | unknown | unknown | B.1 | G |
| hCoV-19/Egypt/CUNCI-HGC009/2020 | EPI_ISL_468053 | 02/05/2020 | Africa / Egypt | Human | unknown | unknown | unknown | unknown | unknown | unknown | B.1 | GH |
| hCoV-19/Egypt/CUNCI-HGC007-2/2020 | EPI_ISL_468054 | 02/05/2020 | Africa / Egypt | Human | unknown | unknown | unknown | unknown | unknown | unknown | B.1 | GH |
| hCoV-19/Egypt/CUNCI-HGC010/2020 | EPI_ISL_468055 | 02/05/2020 | Africa / Egypt | Human | unknown | unknown | unknown | unknown | unknown | unknown | B.1 | GH |
| hCoV-19/Egypt/CUNCI-HGC015/2020 | EPI_ISL_468056 | 02/05/2020 | Africa / Egypt | Human | unknown | unknown | unknown | unknown | unknown | unknown | B.1 | G |
| hCoV-19/Egypt/CUNCI-HGC023/2020 | EPI_ISL_468057 | 02/05/2020 | Africa / Egypt | Human | unknown | unknown | unknown | unknown | unknown | unknown | B.1 | G |
| hCoV-19/Egypt/CUNCI-HGC016/2020 | EPI_ISL_468058 | 02/05/2020 | Africa / Egypt | Human | unknown | unknown | unknown | unknown | unknown | unknown | B.1 | GH |
| hCoV-19/Egypt/CUNCI-HGC011/2020 | EPI_ISL_468059 | 02/05/2020 | Africa / Egypt | Human | unknown | unknown | unknown | unknown | unknown | unknown | B.1 | GH |
| hCoV-19/Egypt/CUNCI-HGC014/2020 | EPI_ISL_468060 | 02/05/2020 | Africa / Egypt | Human | unknown | unknown | unknown | unknown | unknown | unknown | B.1 | G |
| hCoV-19/Egypt/CUNCI-HGC021/2020 | EPI_ISL_468061 | 02/05/2020 | Africa / Egypt | Human | unknown | unknown | unknown | unknown | unknown | unknown | B.1 | G |
| hCoV-19/Egypt/CUNCI-HGC028/2020 | EPI_ISL_468062 | 02/05/2020 | Africa / Egypt | Human | unknown | unknown | unknown | unknown | unknown | unknown | B.1 | O |
| hCoV-19/Egypt/CUNCI-HGC002-2/2020 | EPI_ISL_469275 | 02/05/2020 | Africa / Egypt / Cairo | Human | unknown | Female | 40 | Live | unknown | unknown | B.1 | GH |
| hCoV-19/Egypt/CUNCI-HGC5I015/2020 | EPI_ISL_475722 | 02/05/2020 | Africa / Egypt | Human | unknown | unknown | unknown | unknown | unknown | unknown | B.1 | GH |
| hCoV-19/Egypt/CUNCI-HGC6I016/2020 | EPI_ISL_475723 | 02/06/2020 | Africa / Egypt | Human | unknown | unknown | unknown | unknown | unknown | unknown | B.1.36 | GH |
| hCoV-19/Egypt/CUNCI-HGC6I031/2020 | EPI_ISL_475724 | 02/06/2020 | Africa / Egypt | Human | unknown | unknown | unknown | unknown | unknown | unknown | C.36 | GR |
| hCoV-19/Egypt/MASRI-2/2020 | EPI_ISL_475745 | 2020-05 | Africa / Egypt | Human | unknown | Female | 63 | Live | unknown | unknown | B.1 | GH |
| hCoV-19/Egypt/MASRI-3/2020 | EPI_ISL_475746 | 2020-05 | Africa / Egypt | Human | unknown | Male | 41 | Live | unknown | unknown | A | S |
| hCoV-19/Egypt/MASRI-6/2020 | EPI_ISL_475747 | 2020-05 | Africa / Egypt | Human | unknown | Male | 54 | Live | unknown | unknown | B.1 | GH |
| hCoV-19/Egypt/MASRI-13/2020 | EPI_ISL_475748 | 2020-05 | Africa / Egypt | Human | unknown | Male | 72 | Live | unknown | unknown | B.1 | GH |
| hCoV-19/Egypt/MASRI-14/2020 | EPI_ISL_475749 | 2020-05 | Africa / Egypt | Human | unknown | Female | 35 | Live | unknown | unknown | B.1 | GH |
| hCoV-19/Egypt/MASRI-15/2020 | EPI_ISL_475750 | 2020-05 | Africa / Egypt | Human | unknown | Male | 37 | Live | unknown | unknown | B.1.1 | GR |
| hCoV-19/Egypt/MASRI-9/2020 | EPI_ISL_475751 | 2020-05 | Africa / Egypt | Human | unknown | Male | 50 | Live | unknown | unknown | C.17 | GR |
| hCoV-19/Egypt/MASRI-10/2020 | EPI_ISL_475752 | 2020-05 | Africa / Egypt | Human | unknown | Male | 34 | Live | unknown | unknown | B.1.170 | GH |
| hCoV-19/Egypt/MASRI-11/2020 | EPI_ISL_475753 | 2020-05 | Africa / Egypt | Human | unknown | Female | 70 | Live | unknown | unknown | B.1.36 | GH |
| hCoV-19/Egypt/CUNCI-HGC6I029/2020 | EPI_ISL_477161 | 02/06/2020 | Africa / Egypt | Human | unknown | unknown | unknown | unknown | unknown | unknown | B.1 | GH |
| hCoV-19/Egypt/CUNCI-HGC6I010/2020 | EPI_ISL_478672 | 02/06/2020 | Africa / Egypt | Human | unknown | unknown | unknown | unknown | unknown | unknown | B.1.170 | GH |
| hCoV-19/Egypt/CUNCI-HGC3I02/2020 | EPI_ISL_479686 | 02/06/2020 | Africa / Egypt | Human | unknown | unknown | unknown | unknown | unknown | unknown | B.1 | GH |
| hCoV-19/Egypt/CUNCI-HGC3I03/2020 | EPI_ISL_479687 | 02/06/2020 | Africa / Egypt | Human | unknown | unknown | unknown | unknown | unknown | unknown | B.1 | GH |
| hCoV-19/Egypt/CUNCI-HGC3I06/2020 | EPI_ISL_479688 | 02/06/2020 | Africa / Egypt | Human | unknown | unknown | unknown | unknown | unknown | unknown | B.1 | GH |
| hCoV-19/Egypt/CUNCI-HGC3I07/2020 | EPI_ISL_479689 | 02/06/2020 | Africa / Egypt | Human | unknown | unknown | unknown | unknown | unknown | unknown | B.1 | GH |
| hCoV-19/Egypt/CUNCI-HGC3I05/2020 | EPI_ISL_479690 | 02/06/2020 | Africa / Egypt | Human | unknown | unknown | unknown | unknown | unknown | unknown | B.1 | G |
| hCoV-19/Egypt/CUNCI-HGC3I025/2020 | EPI_ISL_479691 | 02/06/2020 | Africa / Egypt | Human | unknown | unknown | unknown | unknown | unknown | unknown | B.1 | G |
| hCoV-19/Egypt/CUNCI-HGC3I09/2020 | EPI_ISL_479692 | 02/06/2020 | Africa / Egypt | Human | unknown | unknown | unknown | unknown | unknown | unknown | B.1 | GH |
| hCoV-19/Egypt/CUNCI-HGC3I012/2020 | EPI_ISL_479693 | 02/06/2020 | Africa / Egypt | Human | unknown | unknown | unknown | unknown | unknown | unknown | B.1 | GH |
| hCoV-19/Egypt/CUNCI-HGC3I013/2020 | EPI_ISL_479694 | 02/06/2020 | Africa / Egypt | Human | unknown | unknown | unknown | unknown | unknown | unknown | B.1.201 | G |
| hCoV-19/Egypt/CUNCI-HGC3I023/2020 | EPI_ISL_479695 | 02/06/2020 | Africa / Egypt | Human | unknown | unknown | unknown | unknown | unknown | unknown | B.1 | G |
| hCoV-19/Egypt/CUNCI-HGC3I014/2020 | EPI_ISL_479696 | 02/06/2020 | Africa / Egypt | Human | unknown | unknown | unknown | unknown | unknown | unknown | B.1 | G |
| hCoV-19/Egypt/CUNCI-HGC3I011/2020 | EPI_ISL_479697 | 02/06/2020 | Africa / Egypt | Human | unknown | unknown | unknown | unknown | unknown | unknown | B.1 | GH |
| hCoV-19/Egypt/CUNCI-HGC3I08/2020 | EPI_ISL_479698 | 02/06/2020 | Africa / Egypt | Human | unknown | unknown | unknown | unknown | unknown | unknown | B.1 | GH |
| hCoV-19/Egypt/CUNCI-HGC4I003/2020 | EPI_ISL_479699 | 02/06/2020 | Africa / Egypt | Human | unknown | unknown | unknown | unknown | unknown | unknown | B.1.1 | GR |
| hCoV-19/Egypt/CUNCI-HGC4I004/2020 | EPI_ISL_479700 | 02/06/2020 | Africa / Egypt | Human | unknown | unknown | unknown | unknown | unknown | unknown | B.1.1 | GR |
| hCoV-19/Egypt/CUNCI-HGC4I029/2020 | EPI_ISL_479701 | 02/06/2020 | Africa / Egypt | Human | unknown | unknown | unknown | unknown | unknown | unknown | C.36 | GR |
| hCoV-19/Egypt/CUNCI-HGC4I026/2020 | EPI_ISL_479702 | 02/06/2020 | Africa / Egypt | Human | unknown | unknown | unknown | unknown | unknown | unknown | B.1 | GH |
| hCoV-19/Egypt/CUNCI-HGC4I030/2020 | EPI_ISL_479703 | 02/06/2020 | Africa / Egypt | Human | unknown | unknown | unknown | unknown | unknown | unknown | B.1 | GH |
| hCoV-19/Egypt/CUNCI-HGC4I033/2020 | EPI_ISL_479704 | 02/06/2020 | Africa / Egypt | Human | unknown | unknown | unknown | unknown | unknown | unknown | B.1 | GH |
| hCoV-19/Egypt/CUNCI-HGC4I022/2020 | EPI_ISL_479705 | 02/06/2020 | Africa / Egypt | Human | unknown | unknown | unknown | unknown | unknown | unknown | B.1.1 | GR |
| hCoV-19/Egypt/CUNCI-HGC4I025/2020 | EPI_ISL_479706 | 02/06/2020 | Africa / Egypt | Human | unknown | unknown | unknown | unknown | unknown | unknown | B.1 | GH |
| hCoV-19/Egypt/CUNCI-HGC4I031/2020 | EPI_ISL_479707 | 02/06/2020 | Africa / Egypt | Human | unknown | unknown | unknown | unknown | unknown | unknown | C.36 | GR |
| hCoV-19/Egypt/CUNCI-HGC4I034/2020 | EPI_ISL_479708 | 02/06/2020 | Africa / Egypt | Human | unknown | unknown | unknown | unknown | unknown | unknown | B.1 | GH |
| hCoV-19/Egypt/CUNCI-HGC4I032/2020 | EPI_ISL_479709 | 02/06/2020 | Africa / Egypt | Human | unknown | unknown | unknown | unknown | unknown | unknown | B.1.1 | GR |
| hCoV-19/Egypt/CUNCI-HGC5I001/2020 | EPI_ISL_479710 | 02/06/2020 | Africa / Egypt | Human | unknown | unknown | unknown | unknown | unknown | unknown | B.1.1 | GR |
| hCoV-19/Egypt/CUNCI-HGC5I003/2020 | EPI_ISL_479711 | 02/06/2020 | Africa / Egypt | Human | unknown | unknown | unknown | unknown | unknown | unknown | B.1 | G |
| hCoV-19/Egypt/CUNCI-HGC5I011/2020 | EPI_ISL_479712 | 02/06/2020 | Africa / Egypt | Human | unknown | unknown | unknown | unknown | unknown | unknown | B.1.1 | GR |
| hCoV-19/Egypt/CUNCI-HGC5I012/2020 | EPI_ISL_479713 | 02/06/2020 | Africa / Egypt | Human | unknown | unknown | unknown | unknown | unknown | unknown | B.1.1 | GR |
| hCoV-19/Egypt/CUNCI-HGC5I014/2020 | EPI_ISL_479714 | 02/06/2020 | Africa / Egypt | Human | unknown | unknown | unknown | unknown | unknown | unknown | B.1 | GH |
| hCoV-19/Egypt/CUNCI-HGC5I022/2020 | EPI_ISL_479715 | 02/06/2020 | Africa / Egypt | Human | unknown | unknown | unknown | unknown | unknown | unknown | B.1.1 | GR |
| hCoV-19/Egypt/CUNCI-HGC5I025/2020 | EPI_ISL_479716 | 02/06/2020 | Africa / Egypt | Human | unknown | unknown | unknown | unknown | unknown | unknown | A | O |
| hCoV-19/Egypt/CUNCI-HGC5I033/2020 | EPI_ISL_479717 | 02/06/2020 | Africa / Egypt | Human | unknown | unknown | unknown | unknown | unknown | unknown | B.1 | G |
| hCoV-19/Egypt/CUNCI-HGC6I003/2020 | EPI_ISL_479718 | 02/06/2020 | Africa / Egypt | Human | unknown | unknown | unknown | unknown | unknown | unknown | B.1 | G |
| hCoV-19/Egypt/CUNCI-HGC6I002/2020 | EPI_ISL_479719 | 02/06/2020 | Africa / Egypt | Human | unknown | unknown | unknown | unknown | unknown | unknown | C.36 | GR |
| hCoV-19/Egypt/CUNCI-HGC5I034/2020 | EPI_ISL_479720 | 02/06/2020 | Africa / Egypt | Human | unknown | unknown | unknown | unknown | unknown | unknown | B.1 | G |
| hCoV-19/Egypt/CUNCI-HGC5I030/2020 | EPI_ISL_479721 | 02/06/2020 | Africa / Egypt | Human | unknown | unknown | unknown | unknown | unknown | unknown | B.1 | G |
| hCoV-19/Egypt/CUNCI-HGC6I007/2020 | EPI_ISL_479722 | 02/06/2020 | Africa / Egypt | Human | unknown | unknown | unknown | unknown | unknown | unknown | B.1 | G |
| hCoV-19/Egypt/CUNCI-HGC6I033/2020 | EPI_ISL_479723 | 02/06/2020 | Africa / Egypt | Human | unknown | unknown | unknown | unknown | unknown | unknown | C.17 | GR |
| hCoV-19/Egypt/CUNCI-HGC6I026/2020 | EPI_ISL_479724 | 02/06/2020 | Africa / Egypt | Human | unknown | unknown | unknown | unknown | unknown | unknown | C.36 | GR |
| hCoV-19/Egypt/CUNCI-HGC6I015/2020 | EPI_ISL_479725 | 02/06/2020 | Africa / Egypt | Human | unknown | unknown | unknown | unknown | unknown | unknown | B.1.1 | GR |
| hCoV-19/Egypt/CUNCI-HGC6I009/2020 | EPI_ISL_479726 | 02/06/2020 | Africa / Egypt | Human | unknown | unknown | unknown | unknown | unknown | unknown | C.36 | GR |
| hCoV-19/Egypt/CUNCI-HGC6I011/2020 | EPI_ISL_479727 | 02/06/2020 | Africa / Egypt | Human | unknown | unknown | unknown | unknown | unknown | unknown | A | S |
| hCoV-19/Egypt/CUNCI-HGC4I015/2020 | EPI_ISL_479728 | 02/06/2020 | Africa / Egypt | Human | unknown | unknown | unknown | unknown | unknown | unknown | B.1.1 | GR |
| hCoV-19/Egypt/CUNCI-HGC4I023/2020 | EPI_ISL_479729 | 02/06/2020 | Africa / Egypt | Human | unknown | unknown | unknown | unknown | unknown | unknown | B.1.1 | GR |
| hCoV-19/Egypt/CUNCI-HGC5I010/2020 | EPI_ISL_479730 | 02/06/2020 | Africa / Egypt | Human | unknown | unknown | unknown | unknown | unknown | unknown | B.1.1 | GR |
| hCoV-19/Egypt/CUNCI-HGC5I016/2020 | EPI_ISL_479731 | 02/06/2020 | Africa / Egypt | Human | unknown | unknown | unknown | unknown | unknown | unknown | B.1 | GH |
| hCoV-19/Egypt/CUNCI-HGC6I024/2020 | EPI_ISL_479732 | 02/06/2020 | Africa / Egypt | Human | unknown | unknown | unknown | unknown | unknown | unknown | B.1.1 | GR |
| hCoV-19/Egypt/CUNCI-HGC6I032/2020 | EPI_ISL_479733 | 02/06/2020 | Africa / Egypt | Human | unknown | unknown | unknown | unknown | unknown | unknown | C.36 | GR |
| hCoV-19/Egypt/CUNCI-HGC5I029/2020 | EPI_ISL_479734 | 02/06/2020 | Africa / Egypt | Human | unknown | unknown | unknown | unknown | unknown | unknown | B.1 | GH |
| hCoV-19/Egypt/CUNCI-HGC5I026/2020 | EPI_ISL_479735 | 02/06/2020 | Africa / Egypt | Human | unknown | unknown | unknown | unknown | unknown | unknown | B.1 | G |
| hCoV-19/Egypt/MASRI-005/2020 | EPI_ISL_482759 | 30/04/2020 | Africa / Egypt | Human | unknown | Male | 45 | Live | unknown | unknown | B.1.195 | G |
| hCoV-19/Egypt/MASRI-007/2020 | EPI_ISL_482760 | 28/04/2020 | Africa / Egypt | Human | unknown | Male | 35 | Live | unknown | unknown | B.1.195 | G |
| hCoV-19/Egypt/MASRI-008/2020 | EPI_ISL_482761 | 30/04/2020 | Africa / Egypt | Human | unknown | Male | 50 | Live | unknown | unknown | B.1.195 | G |
| hCoV-19/Egypt/MASRI-011/2020 | EPI_ISL_482762 | 05/05/2020 | Africa / Egypt | Human | unknown | Male | 60 | Live | unknown | unknown | B.1.195 | G |
| hCoV-19/Egypt/MASRI-012/2020 | EPI_ISL_482763 | 05/05/2020 | Africa / Egypt | Human | unknown | Female | 45 | Live | unknown | unknown | B.1.195 | G |
| hCoV-19/Egypt/MASRI-013/2020 | EPI_ISL_482764 | 05/05/2020 | Africa / Egypt | Human | unknown | Female | 32 | Live | unknown | unknown | B.1.195 | G |
| hCoV-19/Egypt/MASRI-014/2020 | EPI_ISL_482765 | 30/04/2020 | Africa / Egypt | Human | unknown | Female | 33 | Live | unknown | unknown | B.1.466 | GH |
| hCoV-19/Egypt/MASRI-015/2020 | EPI_ISL_482766 | 11/05/2020 | Africa / Egypt | Human | unknown | Male | 39 | Live | unknown | unknown | B.1 | GH |
| hCoV-19/Egypt/MASRI-016/2020 | EPI_ISL_482767 | 11/05/2020 | Africa / Egypt | Human | unknown | Male | 51 | Live | unknown | unknown | B.1 | GH |
| hCoV-19/Egypt/MASRI-022/2020 | EPI_ISL_482768 | 13/05/2020 | Africa / Egypt | Human | unknown | Female | 50 | Live | unknown | unknown | B.1 | GH |
| hCoV-19/Egypt/MASRI-023/2020 | EPI_ISL_482769 | 11/05/2020 | Africa / Egypt | Human | unknown | Male | 39 | Live | unknown | unknown | B.1 | GH |
| hCoV-19/Egypt/MASRI-024/2020 | EPI_ISL_482770 | 11/05/2020 | Africa / Egypt | Human | unknown | Male | 31 | Live | unknown | unknown | B.1 | GH |
| hCoV-19/Egypt/MASRI-025/2020 | EPI_ISL_482771 | 10/05/2020 | Africa / Egypt | Human | unknown | Female | 39 | Live | unknown | unknown | B.1 | GH |
| hCoV-19/Egypt/MASRI-026/2020 | EPI_ISL_482772 | 10/05/2020 | Africa / Egypt | Human | unknown | Male | 31 | Live | unknown | unknown | B.1 | GH |
| hCoV-19/Egypt/MASRI-027/2020 | EPI_ISL_482773 | 08/05/2020 | Africa / Egypt | Human | unknown | Female | 33 | Live | unknown | unknown | B.1.1 | GR |
| hCoV-19/Egypt/MASRI-029/2020 | EPI_ISL_482774 | 13/05/2020 | Africa / Egypt | Human | unknown | Female | 35 | Live | unknown | unknown | B.1 | GH |
| hCoV-19/Egypt/MASRI-030/2020 | EPI_ISL_482775 | 14/04/2020 | Africa / Egypt | Human | unknown | Female | 0 | Live | unknown | unknown | B.1.1 | GR |
| hCoV-19/Egypt/MASRI-009/2020 | EPI_ISL_483035 | 30/04/2020 | Africa / Egypt / Cairo | Human | unknown | Male | 47 | Live | unknown | unknown | A | S |
| hCoV-19/Egypt/MASRI-018/2020 | EPI_ISL_483036 | 10/05/2020 | Africa / Egypt / Cairo | Human | unknown | Male | 50 | Live | unknown | unknown | A | S |
| hCoV-19/Egypt/MASRI-020/2020 | EPI_ISL_483037 | 13/05/2020 | Africa / Egypt / Kalyoubia | Human | unknown | Male | 35 | Live | unknown | unknown | B.1 | GH |
| hCoV-19/Egypt/MASRI-028/2020 | EPI_ISL_483038 | 13/05/2020 | Africa / Egypt / Cairo / Al Matariyyah | Human | unknown | Male | 39 | Live | unknown | unknown | B.1 | GH |
| hCoV-19/Egypt/Army-MCL001/2020 | EPI_ISL_510526 | 01/06/2020 | Africa / Egypt / Fayoum | Human | unknown | unknown | unknown | unknown | unknown | unknown | B.1 | GH |
| hCoV-19/Egypt/HCoV2-Egy-002/2020 | EPI_ISL_510532 | 02/06/2020 | Africa / Egypt / Fayoum | Human | unknown | unknown | unknown | unknown | unknown | unknown | B.1 | GH |
| hCoV-19/Egypt/CUNCI-7I028/2020 | EPI_ISL_524426 | 19/07/2020 | Africa / Egypt | Human | unknown | unknown | unknown | unknown | unknown | unknown | B.1 | GH |
| hCoV-19/Egypt/CUNCI-7I026/2020 | EPI_ISL_524427 | 19/07/2020 | Africa / Egypt | Human | unknown | unknown | unknown | unknown | unknown | unknown | B.1 | GH |
| hCoV-19/Egypt/EGY-020/2020 | EPI_ISL_526975 | 14/08/2020 | Africa / Egypt | Human | unknown | unknown | unknown | unknown | unknown | unknown | B.1 | GH |
| hCoV-19/Egypt/EGY-021/2020 | EPI_ISL_526976 | 14/08/2020 | Africa / Egypt | Human | unknown | unknown | unknown | unknown | unknown | unknown | B.1 | GH |
| hCoV-19/Egypt/EGY-022/2020 | EPI_ISL_526977 | 14/08/2020 | Africa / Egypt | Human | unknown | unknown | unknown | unknown | unknown | unknown | B.1 | GH |
| hCoV-19/Egypt/EGY-023/2020 | EPI_ISL_526978 | 14/08/2020 | Africa / Egypt | Human | unknown | unknown | unknown | unknown | unknown | unknown | B.1 | GH |
| hCoV-19/Egypt/EGY-024/2020 | EPI_ISL_526979 | 14/08/2020 | Africa / Egypt | Human | unknown | unknown | unknown | unknown | unknown | unknown | B.1 | GH |
| hCoV-19/Egypt/EGY-025/2020 | EPI_ISL_526980 | 14/08/2020 | Africa / Egypt | Human | unknown | unknown | unknown | unknown | unknown | unknown | B.1 | GH |
| hCoV-19/Egypt/EGY-026/2020 | EPI_ISL_526981 | 14/08/2020 | Africa / Egypt | Human | unknown | unknown | unknown | unknown | unknown | unknown | B.1 | GH |
| hCoV-19/Egypt/EGY-027/2020 | EPI_ISL_526982 | 14/08/2020 | Africa / Egypt | Human | unknown | unknown | unknown | unknown | unknown | unknown | B.1 | GH |
| hCoV-19/Egypt/EGY-028/2020 | EPI_ISL_526983 | 14/08/2020 | Africa / Egypt | Human | unknown | unknown | unknown | unknown | unknown | unknown | B.1 | GH |
| hCoV-19/Egypt/EGY-029/2020 | EPI_ISL_526984 | 14/08/2020 | Africa / Egypt | Human | unknown | unknown | unknown | unknown | unknown | unknown | B.1 | GH |
| hCoV-19/Egypt/EGY-030/2020 | EPI_ISL_526985 | 14/08/2020 | Africa / Egypt | Human | unknown | unknown | unknown | unknown | unknown | unknown | B.1 | GH |
| hCoV-19/Egypt/EGY-S031/2020 | EPI_ISL_526986 | 17/08/2020 | Africa / Egypt | Human | unknown | unknown | unknown | unknown | unknown | unknown | B.1 | GH |
| hCoV-19/Egypt/EGY-S032/2020 | EPI_ISL_526987 | 17/08/2020 | Africa / Egypt | Human | unknown | unknown | unknown | unknown | unknown | unknown | B.1 | GH |
| hCoV-19/Egypt/EGY-S033/2020 | EPI_ISL_526988 | 17/08/2020 | Africa / Egypt | Human | unknown | unknown | unknown | unknown | unknown | unknown | B.1 | GH |
| hCoV-19/Egypt/EGY-S034/2020 | EPI_ISL_526989 | 17/08/2020 | Africa / Egypt | Human | unknown | unknown | unknown | unknown | unknown | unknown | B.1 | GH |
| hCoV-19/Egypt/EGY-S035/2020 | EPI_ISL_526990 | 17/08/2020 | Africa / Egypt | Human | unknown | unknown | unknown | unknown | unknown | unknown | B.1 | GH |
| hCoV-19/Egypt/EGY-S036/2020 | EPI_ISL_526991 | 17/08/2020 | Africa / Egypt | Human | unknown | unknown | unknown | unknown | unknown | unknown | B.1 | GH |
| hCoV-19/Egypt/EGY-S037/2020 | EPI_ISL_526992 | 17/08/2020 | Africa / Egypt | Human | unknown | unknown | unknown | unknown | unknown | unknown | B.1 | GH |
| hCoV-19/Egypt/EGY-S038/2020 | EPI_ISL_526993 | 17/08/2020 | Africa / Egypt | Human | unknown | unknown | unknown | unknown | unknown | unknown | B.1 | GH |
| hCoV-19/Egypt/EGY-S039/2020 | EPI_ISL_526994 | 17/08/2020 | Africa / Egypt | Human | unknown | unknown | unknown | unknown | unknown | unknown | B.1 | GH |
| hCoV-19/Egypt/EGY-S040/2020 | EPI_ISL_526995 | 17/08/2020 | Africa / Egypt | Human | unknown | unknown | unknown | unknown | unknown | unknown | B.1 | GH |
| hCoV-19/Egypt/EGY-S030/2020 | EPI_ISL_526996 | 17/08/2020 | Africa / Egypt | Human | unknown | unknown | unknown | unknown | unknown | unknown | B.1 | GH |
| hCoV-19/Egypt/EGY-041/2020 | EPI_ISL_526997 | 22/08/2020 | Africa / Egypt | Human | unknown | unknown | unknown | unknown | unknown | unknown | B.1 | GH |
| hCoV-19/Egypt/EGY-042/2020 | EPI_ISL_526998 | 22/08/2020 | Africa / Egypt | Human | unknown | unknown | unknown | unknown | unknown | unknown | B.1 | GH |
| hCoV-19/Egypt/EGY-044/2020 | EPI_ISL_526999 | 22/08/2020 | Africa / Egypt | Human | unknown | unknown | unknown | unknown | unknown | unknown | B.1 | GH |
| hCoV-19/Egypt/EGY-043/2020 | EPI_ISL_527000 | 22/08/2020 | Africa / Egypt | Human | unknown | unknown | unknown | unknown | unknown | unknown | B.1 | GH |
| hCoV-19/Egypt/EGY-045/2020 | EPI_ISL_527001 | 22/08/2020 | Africa / Egypt | Human | unknown | unknown | unknown | unknown | unknown | unknown | B.1 | GH |
| hCoV-19/Egypt/EGY-046/2020 | EPI_ISL_527002 | 22/08/2020 | Africa / Egypt | Human | unknown | unknown | unknown | unknown | unknown | unknown | B.1 | GH |
| hCoV-19/Egypt/EGY-047/2020 | EPI_ISL_527003 | 22/08/2020 | Africa / Egypt | Human | unknown | unknown | unknown | unknown | unknown | unknown | B.1 | GH |
| hCoV-19/Egypt/EGY-048/2020 | EPI_ISL_527004 | 22/08/2020 | Africa / Egypt | Human | unknown | unknown | unknown | unknown | unknown | unknown | B.1 | GH |
| hCoV-19/Egypt/EGY-049/2020 | EPI_ISL_527005 | 22/08/2020 | Africa / Egypt | Human | unknown | unknown | unknown | unknown | unknown | unknown | B.1 | GH |
| hCoV-19/Egypt/EGY-050/2020 | EPI_ISL_527006 | 22/08/2020 | Africa / Egypt | Human | unknown | unknown | unknown | unknown | unknown | unknown | B.1 | GH |
| hCoV-19/Egypt/EGY-040/2020 | EPI_ISL_527007 | 22/08/2020 | Africa / Egypt | Human | unknown | unknown | unknown | unknown | unknown | unknown | B.1 | GH |
| hCoV-19/Egypt/C-VSVRI-SERVAC/2020 | EPI_ISL_528386 | 22/08/2020 | Africa / Egypt | Human | unknown | unknown | unknown | unknown | unknown | unknown | B.1 | GH |
| hCoV-19/Egypt/C-CU001a-S1/2020 | EPI_ISL_529031 | 19/05/2020 | Africa / Egypt / Cairo | Human | unknown | Male | 35 | Released | unknown | unknown | B.1 | GH |
| hCoV-19/Egypt/C-CU002b-S3/2020 | EPI_ISL_529032 | 19/06/2020 | Africa / Egypt / Cairo | Human | unknown | Female | 40 | Released | unknown | unknown | C.36 | GR |
| hCoV-19/Egypt/CUNCI-HGC7I01/2020 | EPI_ISL_529141 | 19/07/2020 | Africa / Egypt / Cairo | Human | unknown | unknown | unknown | unknown | unknown | unknown | B.1 | GH |
| hCoV-19/Egypt/CUNCI-HGC7I02/2020 | EPI_ISL_529142 | 19/07/2020 | Africa / Egypt / Cairo | Human | unknown | unknown | unknown | unknown | unknown | unknown | A | S |
| hCoV-19/Egypt/CUNCI-HGC7I029/2020 | EPI_ISL_529143 | 19/07/2020 | Africa / Egypt / Cairo | Human | unknown | unknown | unknown | unknown | unknown | unknown | B.1 | GH |
| hCoV-19/Egypt/CUNCI-HGC7I030/2020 | EPI_ISL_529144 | 19/07/2020 | Africa / Egypt / Cairo | Human | unknown | unknown | unknown | unknown | unknown | unknown | B.1 | GH |
| hCoV-19/Egypt/CUNCI-HGC7I07/2020 | EPI_ISL_529145 | 19/07/2020 | Africa / Egypt / Cairo | Human | unknown | unknown | unknown | unknown | unknown | unknown | B.1 | GH |
| hCoV-19/Egypt/CUNCI-HGC7I010/2020 | EPI_ISL_576371 | 19/07/2020 | Africa / Egypt | Human | unknown | unknown | unknown | unknown | unknown | unknown | B.1.1 | GR |
| hCoV-19/Egypt/CUNCI-HGC7I023/2020 | EPI_ISL_576372 | 19/07/2020 | Africa / Egypt | Human | unknown | unknown | unknown | unknown | unknown | unknown | C.36 | GR |
| hCoV-19/Egypt/CUNCI-HGC7I025/2020 | EPI_ISL_576373 | 19/07/2020 | Africa / Egypt | Human | unknown | unknown | unknown | unknown | unknown | unknown | B.1 | GH |
| hCoV-19/Egypt/C-CEIRS-19 MOH/2020 | EPI_ISL_605780 | 13/03/2020 | Africa / Egypt / Cairo | Human | unknown | unknown | unknown | unknown | unknown | unknown | B.1 | GH |
| hCoV-19/Egypt/C-CEIRS-2 MOH/2020 | EPI_ISL_605781 | 02/04/2020 | Africa / Egypt / Cairo | Human | unknown | unknown | unknown | unknown | unknown | unknown | B.1.1 | GR |
| hCoV-19/Egypt/C-CEIRS-8 MOH/2020 | EPI_ISL_605782 | 17/05/2020 | Africa / Egypt / Cairo | Human | unknown | unknown | unknown | unknown | unknown | unknown | B.1 | G |
| hCoV-19/Egypt/PHARCO-ARMY-85/2021 | EPI_ISL_1936369 | 12/02/2021 | Africa / Egypt / Cairo | Human | unknown | unknown | 25-55 | Hospitalized | unknown | unknown | C.36 | GR |
| hCoV-19/Egypt/ARMY-MCL001-2/2020 | EPI_ISL_907077 | 19/11/2020 | Africa / Egypt | Human | unknown | unknown | unknown | unknown | unknown | unknown | B.1 | GH |
| hCoV-19/Egypt/NRC-6735/2020 | EPI_ISL_8193628 | 18/09/2020 | Africa / Egypt | Human | unknown | unknown | unknown | unknown | unknown | unknown | C.17 | G |
| hCoV-19/Egypt/ARMY-MCL012/2020 | EPI_ISL_907085 | 19/11/2020 | Africa / Egypt | Human | unknown | unknown | unknown | unknown | unknown | unknown | B.1 | GH |
| hCoV-19/Egypt/NRC-6450/2020 | EPI_ISL_8193901 | 15/08/2020 | Africa / Egypt | Human | unknown | unknown | unknown | unknown | unknown | unknown | B.1 | G |
| hCoV-19/Egypt/Delta008/2021 | EPI_ISL_4629984 | 04/08/2021 | Africa / Egypt / Cairo | Human | unknown | unknown | unknown | Hospitalized | unknown | unknown | B.1.617.2 | GK |
| hCoV-19/Egypt/NRC-792/2021 | EPI_ISL_8251511 | 13/06/2021 | Africa / Egypt | Human | unknown | unknown | unknown | unknown | unknown | unknown | B.1 | G |
| hCoV-19/Egypt/NRC-678/2021 | EPI_ISL_8251500 | 20/05/2021 | Africa / Egypt | Human | unknown | unknown | unknown | unknown | unknown | unknown | C.36.3 | GR |
| hCoV-19/Egypt/NRC-608/2021 | EPI_ISL_8251489 | 09/05/2021 | Africa / Egypt | Human | unknown | unknown | unknown | unknown | unknown | unknown | C.17 | GR |
| hCoV-19/Egypt/NRC-781/2021 | EPI_ISL_8215725 | 09/06/2021 | Africa / Egypt | Human | unknown | unknown | unknown | unknown | unknown | unknown | C.36.3 | GR |
| hCoV-19/Egypt/NRC-659/2021 | EPI_ISL_8215722 | 19/05/2021 | Africa / Egypt | Human | unknown | unknown | unknown | unknown | unknown | unknown | C.36.3 | GR |
| hCoV-19/Egypt/NRC-657/2021 | EPI_ISL_8215721 | 18/05/2021 | Africa / Egypt | Human | unknown | unknown | unknown | unknown | unknown | unknown | B.1.1.7 | GRY |
| hCoV-19/Egypt/NRC-7615/2021 | EPI_ISL_8215716 | 14/01/2021 | Africa / Egypt | Human | unknown | unknown | unknown | unknown | unknown | unknown | B.1.36 | GH |
| hCoV-19/Egypt/NRC-1093/2021 | EPI_ISL_8215707 | 12/10/2021 | Africa / Egypt | Human | unknown | Female | unknown | unknown | unknown | unknown | B.1.617.2 | GK |
| hCoV-19/Egypt/NRC-1090/2021 | EPI_ISL_8215706 | 12/10/2021 | Africa / Egypt | Human | unknown | Female | unknown | unknown | unknown | unknown | B.1.617.2 | GK |
| hCoV-19/Egypt/NRC-1070/2021 | EPI_ISL_8215705 | 06/10/2021 | Africa / Egypt | Human | unknown | Male | unknown | unknown | unknown | unknown | B.1.617.2 | GK |
| hCoV-19/Egypt/NRC-1018/2021 | EPI_ISL_8215699 | 23/09/2021 | Africa / Egypt | Human | unknown | Female | unknown | unknown | unknown | unknown | B.1.617.2 | GK |
| hCoV-19/Egypt/NRC-936/2021 | EPI_ISL_8215692 | 01/09/2021 | Africa / Egypt | Human | unknown | Female | unknown | unknown | unknown | unknown | B.1.617.2 | GK |
| hCoV-19/Egypt/NRC-1436/2021 | EPI_ISL_8215690 | 03/10/2021 | Africa / Egypt | Human | unknown | Male | unknown | unknown | unknown | unknown | AY.127 | GK |
| hCoV-19/Egypt/CCHE57357-P-43/2020 | EPI_ISL_812871 | 16/07/2020 | Africa / Egypt | Human | unknown | unknown | unknown | unknown | unknown | unknown | B | O |
| hCoV-19/Egypt/CCHE57357_Wave_4_143/2021 | EPI_ISL_6011814 | 16/10/2021 | Africa / Egypt | Human | unknown | unknown | unknown | unknown | unknown | unknown | AY.122 | GK |
| hCoV-19/Egypt/CCHE57357_Wave_4_142/2021 | EPI_ISL_6011813 | 15/10/2021 | Africa / Egypt | Human | unknown | unknown | unknown | unknown | unknown | unknown | B.1.617.2 | GK |
| hCoV-19/Egypt/CCHE57357_Wave_4_141/2021 | EPI_ISL_6011812 | 16/10/2021 | Africa / Egypt | Human | unknown | unknown | unknown | unknown | unknown | unknown | AY.122 | GK |
| hCoV-19/Egypt/CCHE57357_Wave_4_140/2021 | EPI_ISL_6011811 | 16/10/2021 | Africa / Egypt | Human | unknown | unknown | unknown | unknown | unknown | unknown | B.1.617.2 | GK |
| hCoV-19/Egypt/CCHE57357_Wave_4_139/2021 | EPI_ISL_6011810 | 18/10/2021 | Africa / Egypt | Human | unknown | unknown | unknown | unknown | unknown | unknown | B.1.617.2 | GK |
| hCoV-19/Egypt/CCHE57357_Wave_4_138/2021 | EPI_ISL_6011809 | 18/10/2021 | Africa / Egypt | Human | unknown | unknown | unknown | unknown | unknown | unknown | B.1.617.2 | GK |
| hCoV-19/Egypt/CCHE57357_Wave_4_137/2021 | EPI_ISL_6011808 | 18/10/2021 | Africa / Egypt | Human | unknown | unknown | unknown | unknown | unknown | unknown | B.1.617.2 | GK |
| hCoV-19/Egypt/CCHE57357_Wave_4_136/2021 | EPI_ISL_6011807 | 18/10/2021 | Africa / Egypt | Human | unknown | unknown | unknown | unknown | unknown | unknown | B.1.617.2 | GK |
| hCoV-19/Egypt/CCHE57357_Wave_4_135/2021 | EPI_ISL_6011806 | 18/10/2021 | Africa / Egypt | Human | unknown | unknown | unknown | unknown | unknown | unknown | B.1.617.2 | GK |
| hCoV-19/Egypt/CCHE57357_Wave_4_133/2021 | EPI_ISL_6011804 | 18/10/2021 | Africa / Egypt | Human | unknown | unknown | unknown | unknown | unknown | unknown | AY.126 | GK |
| hCoV-19/Egypt/CCHE57357_Wave_4_132/2021 | EPI_ISL_6011803 | 18/10/2021 | Africa / Egypt | Human | unknown | unknown | unknown | unknown | unknown | unknown | AY.4 | GK |
| hCoV-19/Egypt/CCHE57357_Wave_4_129/2021 | EPI_ISL_6011800 | 16/10/2021 | Africa / Egypt | Human | unknown | unknown | unknown | unknown | unknown | unknown | B.1.617.2 | GK |
| hCoV-19/Egypt/CCHE57357_Wave_4_126/2021 | EPI_ISL_6011797 | 16/10/2021 | Africa / Egypt | Human | unknown | unknown | unknown | unknown | unknown | unknown | B.1.617.2 | GK |
| hCoV-19/Egypt/CCHE57357_Wave_4_125/2021 | EPI_ISL_6011796 | 16/10/2021 | Africa / Egypt | Human | unknown | unknown | unknown | unknown | unknown | unknown | B.1.617.2 | GK |
| hCoV-19/Egypt/CCHE57357_Wave_4_124/2021 | EPI_ISL_6011795 | 17/10/2021 | Africa / Egypt | Human | unknown | unknown | unknown | unknown | unknown | unknown | AY.122 | GK |
| hCoV-19/Egypt/CCHE57357_Wave_4_121/2021 | EPI_ISL_6011792 | 19/10/2021 | Africa / Egypt | Human | unknown | unknown | unknown | unknown | unknown | unknown | B.1.617.2 | GK |
| hCoV-19/Egypt/CCHE57357_Wave_4_119/2021 | EPI_ISL_6011790 | 19/10/2021 | Africa / Egypt | Human | unknown | unknown | unknown | unknown | unknown | unknown | B.1.617.2 | GK |
| hCoV-19/Egypt/CCHE57357_Wave_4_118/2021 | EPI_ISL_6011789 | 19/10/2021 | Africa / Egypt | Human | unknown | unknown | unknown | unknown | unknown | unknown | B.1.617.2 | GK |
| hCoV-19/Egypt/CCHE57357_Wave_4_116/2021 | EPI_ISL_6011787 | 19/10/2021 | Africa / Egypt | Human | unknown | unknown | unknown | unknown | unknown | unknown | B.1.617.2 | GK |
| hCoV-19/Egypt/CCHE57357_Wave_4_114/2021 | EPI_ISL_6011785 | 19/10/2021 | Africa / Egypt | Human | unknown | unknown | unknown | unknown | unknown | unknown | B.1.617.2 | GK |
| hCoV-19/Egypt/CCHE57357_Wave_4_112/2021 | EPI_ISL_6011783 | 19/10/2021 | Africa / Egypt | Human | unknown | unknown | unknown | unknown | unknown | unknown | AY.122 | GK |
| hCoV-19/Egypt/CCHE57357_Wave_4_111/2021 | EPI_ISL_6011782 | 19/10/2021 | Africa / Egypt | Human | unknown | unknown | unknown | unknown | unknown | unknown | B.1.617.2 | GK |
| hCoV-19/Egypt/CCHE57357_Wave_4_110/2021 | EPI_ISL_6011781 | 19/10/2021 | Africa / Egypt | Human | unknown | unknown | unknown | unknown | unknown | unknown | B | L |
| hCoV-19/Egypt/CCHE57357_Wave_4_109/2021 | EPI_ISL_6011780 | 19/10/2021 | Africa / Egypt | Human | unknown | unknown | unknown | unknown | unknown | unknown | B.1.617.2 | GK |
| hCoV-19/Egypt/CCHE57357_Wave_4_108/2021 | EPI_ISL_6011779 | 19/10/2021 | Africa / Egypt | Human | unknown | unknown | unknown | unknown | unknown | unknown | B.1.617.2 | GK |
| hCoV-19/Egypt/CCHE57357_Wave_4_107/2021 | EPI_ISL_6011778 | 19/10/2021 | Africa / Egypt | Human | unknown | unknown | unknown | unknown | unknown | unknown | B.1.617.2 | GK |
| hCoV-19/Egypt/CCHE57357_Wave_4_104/2021 | EPI_ISL_6011775 | 20/10/2021 | Africa / Egypt | Human | unknown | unknown | unknown | unknown | unknown | unknown | B.1.617.2 | GK |
| hCoV-19/Egypt/CCHE57357_Wave_4_103/2021 | EPI_ISL_6011774 | 21/10/2021 | Africa / Egypt | Human | unknown | unknown | unknown | unknown | unknown | unknown | AY.126 | GK |
| hCoV-19/Egypt/CCHE57357_Wave_4_102/2021 | EPI_ISL_6011773 | 21/10/2021 | Africa / Egypt | Human | unknown | unknown | unknown | unknown | unknown | unknown | AY.127 | GK |
| hCoV-19/Egypt/CCHE57357_Wave_4_101/2021 | EPI_ISL_6011772 | 21/10/2021 | Africa / Egypt | Human | unknown | unknown | unknown | unknown | unknown | unknown | B.1.617.2 | GK |
| hCoV-19/Egypt/CCHE57357_Wave_4_097/2021 | EPI_ISL_6011768 | 21/10/2021 | Africa / Egypt | Human | unknown | unknown | unknown | unknown | unknown | unknown | B.1.617.2 | GK |
| hCoV-19/Egypt/CCHE57357_Wave_4_096/2021 | EPI_ISL_6011767 | 21/10/2021 | Africa / Egypt | Human | unknown | unknown | unknown | unknown | unknown | unknown | AY.65 | GK |
| hCoV-19/Egypt/CCHE57357_Wave_4_092/2021 | EPI_ISL_6011763 | 21/10/2021 | Africa / Egypt | Human | unknown | unknown | unknown | unknown | unknown | unknown | B.1.617.2 | GK |
| hCoV-19/Egypt/CCHE57357_Wave_4_091/2021 | EPI_ISL_6011762 | 22/10/2021 | Africa / Egypt | Human | unknown | unknown | unknown | unknown | unknown | unknown | B.1.617.2 | GK |
| hCoV-19/Egypt/CCHE57357_Wave_4_088/2021 | EPI_ISL_6011759 | 22/10/2021 | Africa / Egypt | Human | unknown | unknown | unknown | unknown | unknown | unknown | AY.106 | GK |
| hCoV-19/Egypt/CCHE57357_Wave_4_087/2021 | EPI_ISL_6011758 | 22/10/2021 | Africa / Egypt | Human | unknown | unknown | unknown | unknown | unknown | unknown | B.1.617.2 | GK |
| hCoV-19/Egypt/CCHE57357_Wave_4_086/2021 | EPI_ISL_6011757 | 22/10/2021 | Africa / Egypt | Human | unknown | unknown | unknown | unknown | unknown | unknown | B.1.617.2 | GK |
| hCoV-19/Egypt/CCHE57357_Wave_4_085/2021 | EPI_ISL_6011756 | 22/10/2021 | Africa / Egypt | Human | unknown | unknown | unknown | unknown | unknown | unknown | B.1.617.2 | GK |
| hCoV-19/Egypt/CCHE57357_Wave_4_080/2021 | EPI_ISL_6011751 | 23/10/2021 | Africa / Egypt | Human | unknown | unknown | unknown | unknown | unknown | unknown | B.1.617.2 | GK |
| hCoV-19/Egypt/CCHE57357_Wave_4_079/2021 | EPI_ISL_6011750 | 23/10/2021 | Africa / Egypt | Human | unknown | unknown | unknown | unknown | unknown | unknown | B.1.617.2 | GK |
| hCoV-19/Egypt/CCHE57357_Wave_4_078/2021 | EPI_ISL_6011749 | 23/10/2021 | Africa / Egypt | Human | unknown | unknown | unknown | unknown | unknown | unknown | B.1.617.2 | GK |
| hCoV-19/Egypt/CCHE57357_Wave_4_077/2021 | EPI_ISL_6011748 | 23/10/2021 | Africa / Egypt | Human | unknown | unknown | unknown | unknown | unknown | unknown | B.1.617.2 | GK |
| hCoV-19/Egypt/CCHE57357_Wave_4_076/2021 | EPI_ISL_6011747 | 23/10/2021 | Africa / Egypt | Human | unknown | unknown | unknown | unknown | unknown | unknown | B.1.617.2 | GK |
| hCoV-19/Egypt/CCHE57357_Wave_4_075/2021 | EPI_ISL_6011746 | 23/10/2021 | Africa / Egypt | Human | unknown | unknown | unknown | unknown | unknown | unknown | B.1.617.2 | GK |
| hCoV-19/Egypt/CCHE57357_Wave_4_073/2021 | EPI_ISL_6011744 | 23/10/2021 | Africa / Egypt | Human | unknown | unknown | unknown | unknown | unknown | unknown | B.1.617.2 | GK |
| hCoV-19/Egypt/CCHE57357_Wave_4_072/2021 | EPI_ISL_6011743 | 23/10/2021 | Africa / Egypt | Human | unknown | unknown | unknown | unknown | unknown | unknown | AY.112 | GK |
| hCoV-19/Egypt/CCHE57357_Wave_4_070/2021 | EPI_ISL_6011741 | 23/10/2021 | Africa / Egypt | Human | unknown | unknown | unknown | unknown | unknown | unknown | B.1.617.2 | GK |
| hCoV-19/Egypt/NRC-1227/2021 | EPI_ISL_8205826 | 01/11/2021 | Africa / Egypt | Human | unknown | Female | unknown | unknown | unknown | unknown | B.1.617.2 | GK |
| hCoV-19/Egypt/NRC-1342/2021 | EPI_ISL_8205822 | 28/11/2021 | Africa / Egypt | Human | unknown | Female | unknown | unknown | unknown | unknown | AY.43 | GK |
| hCoV-19/Egypt/CCHE57357-P-45/2020 | EPI_ISL_812872 | 26/06/2020 | Africa / Egypt | Human | unknown | unknown | unknown | unknown | unknown | unknown | B.1 | O |
| hCoV-19/Egypt/NRC-6320/2020 | EPI_ISL_8194889 | 14/07/2020 | Africa / Egypt | Human | unknown | unknown | unknown | unknown | unknown | unknown | B.1 | G |
| hCoV-19/Egypt/NRC-6381/2020 | EPI_ISL_8194891 | 28/07/2020 | Africa / Egypt | Human | unknown | unknown | unknown | unknown | unknown | unknown | C.36.3 | G |
| hCoV-19/Egypt/NRC-6351/2020 | EPI_ISL_8194881 | 26/07/2020 | Africa / Egypt | Human | unknown | unknown | unknown | unknown | unknown | unknown | B.1.170 | G |
| hCoV-19/Egypt/NRC-5642OP/2020 | EPI_ISL_8194879 | 07/06/2020 | Africa / Egypt | Human | unknown | unknown | unknown | unknown | unknown | unknown | B.1.533 | GH |
| hCoV-19/Egypt/NRC-5635/2020 | EPI_ISL_8194876 | 06/06/2020 | Africa / Egypt | Human | unknown | unknown | unknown | unknown | unknown | unknown | B.1.1 | GR |
| hCoV-19/Egypt/NRC-5616/2020 | EPI_ISL_8194872 | 07/06/2020 | Africa / Egypt | Human | unknown | unknown | unknown | unknown | unknown | unknown | B.1.1.372 | GR |
| hCoV-19/Egypt/NRC-6469/2020 | EPI_ISL_8194867 | 18/08/2020 | Africa / Egypt | Human | unknown | unknown | unknown | unknown | unknown | unknown | B.1.1.1 | GR |
| hCoV-19/Egypt/NRC-5939/2020 | EPI_ISL_8193923 | 21/06/2020 | Africa / Egypt | Human | unknown | unknown | unknown | unknown | unknown | unknown | B.1.533 | GH |
| hCoV-19/Egypt/NRC-5940/2020 | EPI_ISL_8193922 | 21/06/2020 | Africa / Egypt | Human | unknown | unknown | unknown | unknown | unknown | unknown | C.17 | GR |
| hCoV-19/Egypt/NRC-5953/2020 | EPI_ISL_8193916 | 20/06/2020 | Africa / Egypt | Human | unknown | unknown | unknown | unknown | unknown | unknown | B.1.170 | GH |
| hCoV-19/Egypt/NRC-5955/2020 | EPI_ISL_8193915 | 21/06/2020 | Africa / Egypt | Human | unknown | unknown | unknown | unknown | unknown | unknown | B.1.533 | GH |
| hCoV-19/Egypt/NRC-6017/2020 | EPI_ISL_8193908 | 23/06/2020 | Africa / Egypt | Human | unknown | unknown | unknown | unknown | unknown | unknown | B.1 | GH |
| hCoV-19/Egypt/NRC-6023/2020 | EPI_ISL_8193906 | 23/06/2020 | Africa / Egypt | Human | unknown | unknown | unknown | unknown | unknown | unknown | B.1 | G |
| hCoV-19/Egypt/NRC-6033/2020 | EPI_ISL_8193904 | 23/06/2020 | Africa / Egypt | Human | unknown | unknown | unknown | unknown | unknown | unknown | B.1.1 | GR |
| hCoV-19/Egypt/NRC-6541/2020 | EPI_ISL_8193902 | 30/08/2020 | Africa / Egypt | Human | unknown | unknown | unknown | unknown | unknown | unknown | B.1 | GH |
| hCoV-19/Egypt/NRC-6451/2020 | EPI_ISL_8193900 | 15/08/2020 | Africa / Egypt | Human | unknown | unknown | unknown | unknown | unknown | unknown | B.1 | G |
| hCoV-19/Egypt/NRC-6173/2020 | EPI_ISL_8193891 | 01/07/2020 | Africa / Egypt | Human | unknown | unknown | unknown | unknown | unknown | unknown | B.1 | GH |
| hCoV-19/Egypt/NRC-6174/2020 | EPI_ISL_8193890 | 01/07/2020 | Africa / Egypt | Human | unknown | unknown | unknown | unknown | unknown | unknown | B.1.170 | GH |
| hCoV-19/Egypt/NRC-6292/2020 | EPI_ISL_8193888 | 07/07/2020 | Africa / Egypt | Human | unknown | unknown | unknown | unknown | unknown | unknown | B.1.170 | GH |
| hCoV-19/Egypt/NRC-6301/2020 | EPI_ISL_8193887 | 09/07/2020 | Africa / Egypt | Human | unknown | unknown | unknown | unknown | unknown | unknown | B.1.170 | GH |
| hCoV-19/Egypt/NRC-6167/2020 | EPI_ISL_8193885 | 01/07/2020 | Africa / Egypt | Human | unknown | unknown | unknown | unknown | unknown | unknown | B.1.170 | GH |
| hCoV-19/Egypt/NRC-6172/2020 | EPI_ISL_8193881 | 01/07/2020 | Africa / Egypt | Human | unknown | unknown | unknown | unknown | unknown | unknown | B.1.1 | GR |
| hCoV-19/Egypt/NRC-5445NS/2020 | EPI_ISL_8193873 | 27/05/2020 | Africa / Egypt | Human | unknown | unknown | unknown | unknown | unknown | unknown | C.17 | GR |
| hCoV-19/Egypt/NRC-5726/2020 | EPI_ISL_8193870 | 11/06/2020 | Africa / Egypt | Human | unknown | unknown | unknown | unknown | unknown | unknown | C.17 | GR |
| hCoV-19/Egypt/NRC-5792/2020 | EPI_ISL_8193866 | 14/06/2020 | Africa / Egypt | Human | unknown | unknown | unknown | unknown | unknown | unknown | B.1 | GH |
| hCoV-19/Egypt/NRC-6199/2020 | EPI_ISL_8193864 | 04/07/2020 | Africa / Egypt | Human | unknown | unknown | unknown | unknown | unknown | unknown | B.1.36 | GH |
| hCoV-19/Egypt/NRC-6205/2020 | EPI_ISL_8193862 | 04/07/2020 | Africa / Egypt | Human | unknown | unknown | unknown | unknown | unknown | unknown | B.1.170 | GH |
| hCoV-19/Egypt/NRC-6209/2020 | EPI_ISL_8193859 | 04/07/2020 | Africa / Egypt | Human | unknown | unknown | unknown | unknown | unknown | unknown | B.1.1 | GR |
| hCoV-19/Egypt/NRC-6337/2020 | EPI_ISL_8193853 | 18/07/2020 | Africa / Egypt | Human | unknown | unknown | unknown | unknown | unknown | unknown | B.1.170 | GH |
| hCoV-19/Egypt/NRC-6335/2020 | EPI_ISL_8193851 | 18/07/2020 | Africa / Egypt | Human | unknown | unknown | unknown | unknown | unknown | unknown | B.1.170 | GH |
| hCoV-19/Egypt/NRC-5555NS/2020 | EPI_ISL_8193654 | 03/06/2020 | Africa / Egypt | Human | unknown | unknown | unknown | unknown | unknown | unknown | C.17 | GR |
| hCoV-19/Egypt/NRC-5555OP/2020 | EPI_ISL_8193653 | 03/06/2020 | Africa / Egypt | Human | unknown | unknown | unknown | unknown | unknown | unknown | C.17 | GR |
| hCoV-19/Egypt/NRC-6505/2020 | EPI_ISL_8193651 | 23/08/2020 | Africa / Egypt | Human | unknown | unknown | unknown | unknown | unknown | unknown | C.17 | GR |
| hCoV-19/Egypt/NRC-6506/2020 | EPI_ISL_8193650 | 23/08/2020 | Africa / Egypt | Human | unknown | unknown | unknown | unknown | unknown | unknown | B.1.1.192 | G |
| hCoV-19/Egypt/NRC-6519/2020 | EPI_ISL_8193648 | 24/08/2020 | Africa / Egypt | Human | unknown | unknown | unknown | unknown | unknown | unknown | B.1.1 | G |
| hCoV-19/Egypt/NRC-6602/2020 | EPI_ISL_8193646 | 03/09/2020 | Africa / Egypt | Human | unknown | unknown | unknown | unknown | unknown | unknown | B.1.1.1 | G |
| hCoV-19/Egypt/NRC-7316/2020 | EPI_ISL_8193634 | 15/12/2020 | Africa / Egypt | Human | unknown | unknown | unknown | unknown | unknown | unknown | C.17 | G |
| hCoV-19/Egypt/NRC-5441NS/2020 | EPI_ISL_8193632 | 26/05/2020 | Africa / Egypt | Human | unknown | unknown | unknown | unknown | unknown | unknown | C.17 | G |
| hCoV-19/Egypt/NRC-5435NS/2020 | EPI_ISL_8193631 | 26/05/2020 | Africa / Egypt | Human | unknown | unknown | unknown | unknown | unknown | unknown | B.1.533 | GH |
| hCoV-19/Egypt/NRC-5459NS/2020 | EPI_ISL_8193627 | 30/05/2020 | Africa / Egypt | Human | unknown | unknown | unknown | unknown | unknown | unknown | B.1 | G |
| hCoV-19/Egypt/NRC-6386/2020 | EPI_ISL_8193622 | 29/07/2020 | Africa / Egypt | Human | unknown | unknown | unknown | unknown | unknown | unknown | B.1.1.442 | GH |
| hCoV-19/Egypt/NRC-6912/2020 | EPI_ISL_8193617 | 17/10/2020 | Africa / Egypt | Human | unknown | unknown | unknown | unknown | unknown | unknown | B.1.1.1 | GH |
| hCoV-19/Egypt/NRC-6047/2020 | EPI_ISL_8193614 | 24/06/2020 | Africa / Egypt | Human | unknown | unknown | unknown | unknown | unknown | unknown | B.1 | GH |
| hCoV-19/Egypt/NRC-6056/2020 | EPI_ISL_8193612 | 24/06/2020 | Africa / Egypt | Human | unknown | unknown | unknown | unknown | unknown | unknown | B.1.1.1 | G |
| hCoV-19/Egypt/NRC-6078/2020 | EPI_ISL_8193607 | 28/06/2020 | Africa / Egypt | Human | unknown | unknown | unknown | unknown | unknown | unknown | B.1.1.442 | GR |
| hCoV-19/Egypt/NRC-6081/2020 | EPI_ISL_8193606 | 28/06/2020 | Africa / Egypt | Human | unknown | unknown | unknown | unknown | unknown | unknown | B.1 | G |
| hCoV-19/Egypt/NRC-6108/2020 | EPI_ISL_8193605 | 28/06/2020 | Africa / Egypt | Human | unknown | unknown | unknown | unknown | unknown | unknown | B.1.178 | G |
| hCoV-19/Egypt/NRC-6657/2020 | EPI_ISL_8193599 | 10/09/2020 | Africa / Egypt | Human | unknown | unknown | unknown | unknown | unknown | unknown | B.1.1 | GR |
| hCoV-19/Egypt/NRC-6662/2020 | EPI_ISL_8193593 | 10/09/2020 | Africa / Egypt | Human | unknown | unknown | unknown | unknown | unknown | unknown | B.1.1.442 | GR |
| hCoV-19/Egypt/NRC-6994/2020 | EPI_ISL_8193592 | 27/10/2020 | Africa / Egypt | Human | unknown | unknown | unknown | unknown | unknown | unknown | B.1 | GH |
| hCoV-19/Egypt/NRC-7071/2020 | EPI_ISL_8193589 | 06/11/2020 | Africa / Egypt | Human | unknown | unknown | unknown | unknown | unknown | unknown | B.1.1.1 | GR |
| hCoV-19/Egypt/NRC-7090/2020 | EPI_ISL_8193587 | 06/11/2020 | Africa / Egypt | Human | unknown | unknown | unknown | unknown | unknown | unknown | B.1 | G |
| hCoV-19/Egypt/NRC-6444/2020 | EPI_ISL_8189547 | 14/08/2020 | Africa / Egypt | Human | unknown | unknown | unknown | unknown | unknown | unknown | B.1.1.1 | GR |
| hCoV-19/Egypt/NRC-5545/2020 | EPI_ISL_8189546 | 03/06/2020 | Africa / Egypt | Human | unknown | unknown | unknown | unknown | unknown | unknown | B.1.1 | GR |
| hCoV-19/Egypt/NRC-7302/2020 | EPI_ISL_8189345 | 15/12/2020 | Africa / Egypt | Human | unknown | unknown | unknown | unknown | unknown | unknown | C.17 | GR |
| hCoV-19/Egypt/NRC-5552NS/2020 | EPI_ISL_8189339 | 03/06/2020 | Africa / Egypt | Human | unknown | unknown | unknown | unknown | unknown | unknown | B.1.1.1 | GH |
| hCoV-19/Egypt/CUNCI-HGC10I010/2020 | EPI_ISL_907090 | 25/12/2020 | Africa / Egypt | Human | unknown | unknown | unknown | unknown | unknown | unknown | C.36 | GR |
| hCoV-19/Egypt/ARMY-360/2021 | EPI_ISL_1936290 | 04/04/2021 | Africa / Egypt / Cairo | Human | unknown | unknown | 25-60 | Hospitalized | unknown | unknown | B.1.170 | GH |
| hCoV-19/Egypt/CCHE57357-A-40/2020 | EPI_ISL_812813 | 29/06/2020 | Africa / Egypt | Human | unknown | unknown | unknown | unknown | unknown | unknown | B | L |
| hCoV-19/Egypt/ARMY-EVA-Pharma-Wave4-025/2021 | EPI_ISL_4750220 | 04/09/2021 | Africa / Egypt / Cairo | Human | unknown | unknown | 25-55 | Hospitalized | unknown | unknown | B.1.617.2 | GK |
| hCoV-19/Egypt/ARMY-MCL002/2020 | EPI_ISL_907078 | 19/11/2020 | Africa / Egypt | Human | unknown | unknown | unknown | unknown | unknown | unknown | C.36 | GR |
| hCoV-19/Egypt/NRC-6201/2020 | EPI_ISL_8193863 | 04/07/2020 | Africa / Egypt | Human | unknown | unknown | unknown | unknown | unknown | unknown | B.1 | GH |
| hCoV-19/Egypt/ARMY-231/2021 | EPI_ISL_1936134 | 02/05/2021 | Africa / Egypt / Cairo | Human | unknown | unknown | 25-55 | Hospitalized | unknown | unknown | C.17 | GR |
| hCoV-19/Egypt/ARMY-357/2021 | EPI_ISL_1936287 | 04/04/2021 | Africa / Egypt / Cairo | Human | unknown | unknown | 25-60 | Hospitalized | unknown | unknown | B.1.170 | GH |
| hCoV-19/Egypt/ARMY-359/2021 | EPI_ISL_1936289 | 04/04/2021 | Africa / Egypt / Cairo | Human | unknown | unknown | 25-60 | Hospitalized | unknown | unknown | B.1.170 | GH |
| hCoV-19/Egypt/ARMY-342/2021 | EPI_ISL_1936274 | 04/04/2021 | Africa / Egypt / Cairo | Human | unknown | unknown | 25-60 | Hospitalized | unknown | unknown | C.17 | GR |
| hCoV-19/Egypt/ARMY-343/2021 | EPI_ISL_1936275 | 04/04/2021 | Africa / Egypt / Cairo | Human | unknown | unknown | 25-60 | Hospitalized | unknown | unknown | C.36.3 | GR |
| hCoV-19/Egypt/ARMY-311/2021 | EPI_ISL_1936250 | 22/04/2021 | Africa / Egypt / Cairo | Human | unknown | unknown | 25-60 | Hospitalized | unknown | unknown | A | S |
| hCoV-19/Egypt/ARMY-313/2021 | EPI_ISL_1936252 | 22/04/2021 | Africa / Egypt / Cairo | Human | unknown | unknown | 25-60 | Hospitalized | unknown | unknown | B.1.1 | GR |
| hCoV-19/Egypt/ARMY-42/2021 | EPI_ISL_1936217 | 27/03/2021 | Africa / Egypt / Cairo | Human | unknown | unknown | 25-55 | Hospitalized | unknown | unknown | C.17 | GR |
| hCoV-19/Egypt/ARMY-31/2021 | EPI_ISL_1936205 | 27/03/2021 | Africa / Egypt / Cairo | Human | unknown | unknown | 25-55 | Hospitalized | unknown | unknown | C.17 | GR |
| hCoV-19/Egypt/ARMY-MCL009/2020 | EPI_ISL_907083 | 19/11/2020 | Africa / Egypt | Human | unknown | unknown | unknown | unknown | unknown | unknown | C.36 | GR |
| hCoV-19/Egypt/CCHE57357-P-26/2020 | EPI_ISL_812860 | 30/06/2020 | Africa / Egypt | Human | unknown | unknown | unknown | unknown | unknown | unknown | B.1.1 | L |
| hCoV-19/Egypt/NRC-463/2020 | EPI_ISL_2232406 | 30/05/2020 | Africa / Egypt | Human | unknown | unknown | unknown | unknown | unknown | unknown | B.1.170 | GH |
| hCoV-19/Egypt/NRC-5463/2020 | EPI_ISL_2232405 | 30/05/2020 | Africa / Egypt | Human | unknown | unknown | unknown | unknown | unknown | unknown | B.1.170 | GH |
| hCoV-19/Egypt/NRC-5875/2020 | EPI_ISL_2232402 | 15/06/2020 | Africa / Egypt | Human | unknown | unknown | unknown | unknown | unknown | unknown | B.1.170 | GH |
| hCoV-19/Egypt/NRC-5874/2020 | EPI_ISL_2232401 | 15/06/2020 | Africa / Egypt | Human | unknown | unknown | unknown | unknown | unknown | unknown | B.1.170 | GH |
| hCoV-19/Egypt/NRC-5872/2020 | EPI_ISL_2232400 | 15/06/2020 | Africa / Egypt | Human | unknown | unknown | unknown | unknown | unknown | unknown | B.1.170 | GH |
| hCoV-19/Egypt/NRC-5871/2020 | EPI_ISL_2232399 | 15/06/2020 | Africa / Egypt | Human | unknown | unknown | unknown | unknown | unknown | unknown | B.1.170 | GH |
| hCoV-19/Egypt/NRC-5863/2020 | EPI_ISL_2232397 | 16/06/2020 | Africa / Egypt | Human | unknown | unknown | unknown | unknown | unknown | unknown | C.17 | GR |
| hCoV-19/Egypt/NRC-5884/2020 | EPI_ISL_2232396 | 17/06/2020 | Africa / Egypt | Human | unknown | unknown | unknown | unknown | unknown | unknown | B.1.170 | GH |
| hCoV-19/Egypt/NRC-5877/2020 | EPI_ISL_2232392 | 17/06/2020 | Africa / Egypt | Human | unknown | unknown | unknown | unknown | unknown | unknown | B.1.170 | GH |
| hCoV-19/Egypt/NRC-5892/2020 | EPI_ISL_2232390 | 18/06/2020 | Africa / Egypt | Human | unknown | unknown | unknown | unknown | unknown | unknown | B.1.195 | G |
| hCoV-19/Egypt/NRC-5909/2020 | EPI_ISL_2232389 | 19/06/2020 | Africa / Egypt | Human | unknown | unknown | unknown | unknown | unknown | unknown | B.1 | GH |
| hCoV-19/Egypt/NRC-5981/2020 | EPI_ISL_2232388 | 23/06/2020 | Africa / Egypt | Human | unknown | unknown | unknown | unknown | unknown | unknown | B.1 | GH |
| hCoV-19/Egypt/NRC-6025/2020 | EPI_ISL_2232387 | 23/06/2020 | Africa / Egypt | Human | unknown | unknown | unknown | unknown | unknown | unknown | B.1.170 | GH |
| hCoV-19/Egypt/NRC-5984/2020 | EPI_ISL_2232386 | 23/06/2020 | Africa / Egypt | Human | unknown | unknown | unknown | unknown | unknown | unknown | B.1 | GH |
| hCoV-19/Egypt/NRC-5983/2020 | EPI_ISL_2232385 | 23/06/2020 | Africa / Egypt | Human | unknown | unknown | unknown | unknown | unknown | unknown | B.1 | GH |
| hCoV-19/Egypt/NRC-6048/2020 | EPI_ISL_2232384 | 24/06/2020 | Africa / Egypt | Human | unknown | unknown | unknown | unknown | unknown | unknown | B.1 | GH |
| hCoV-19/Egypt/NRC-6318/2020 | EPI_ISL_8194888 | 14/07/2020 | Africa / Egypt | Human | unknown | unknown | unknown | unknown | unknown | unknown | C.36.3 | GH |
| hCoV-19/Egypt/NRC-6071/2020 | EPI_ISL_2232383 | 27/06/2020 | Africa / Egypt | Human | unknown | unknown | unknown | unknown | unknown | unknown | B.1.1.1 | GR |
| hCoV-19/Egypt/NRC-6158/2020 | EPI_ISL_2232381 | 30/06/2020 | Africa / Egypt | Human | unknown | unknown | unknown | unknown | unknown | unknown | B.1.1.1 | GR |
| hCoV-19/Egypt/NRC-5626/2020 | EPI_ISL_2232380 | 04/06/2020 | Africa / Egypt | Human | unknown | unknown | unknown | unknown | unknown | unknown | B.1.1 | GR |
| hCoV-19/Egypt/NRC-6382/2020 | EPI_ISL_8194887 | 28/07/2020 | Africa / Egypt | Human | unknown | unknown | unknown | unknown | unknown | unknown | B.1 | G |
| hCoV-19/Egypt/NRC-5625/2020 | EPI_ISL_2232379 | 04/06/2020 | Africa / Egypt | Human | unknown | unknown | unknown | unknown | unknown | unknown | B.1.1 | GR |
| hCoV-19/Egypt/NRC-5648/2020 | EPI_ISL_2232378 | 04/06/2020 | Africa / Egypt | Human | unknown | unknown | unknown | unknown | unknown | unknown | B.1.170 | GH |
| hCoV-19/Egypt/NRC-5646/2020 | EPI_ISL_2232377 | 04/06/2020 | Africa / Egypt | Human | unknown | unknown | unknown | unknown | unknown | unknown | B.1.170 | GH |
| hCoV-19/Egypt/NRC-5630/2020 | EPI_ISL_2232376 | 06/06/2020 | Africa / Egypt | Human | unknown | unknown | unknown | unknown | unknown | unknown | B.1.1 | GR |
| hCoV-19/Egypt/NRC-5638/2020 | EPI_ISL_2232373 | 07/06/2020 | Africa / Egypt | Human | unknown | unknown | unknown | unknown | unknown | unknown | B.1.170 | GH |
| hCoV-19/Egypt/NRC-5628/2020 | EPI_ISL_2232375 | 06/06/2020 | Africa / Egypt | Human | unknown | unknown | unknown | unknown | unknown | unknown | B.1.1 | GR |
| hCoV-19/Egypt/NRC-6218/2020 | EPI_ISL_2232372 | 04/07/2020 | Africa / Egypt | Human | unknown | unknown | unknown | unknown | unknown | unknown | C.17 | GR |
| hCoV-19/Egypt/NRC-6216/2020 | EPI_ISL_2232370 | 04/07/2020 | Africa / Egypt | Human | unknown | unknown | unknown | unknown | unknown | unknown | C.17 | GR |
| hCoV-19/Egypt/NRC-6241/2020 | EPI_ISL_2232369 | 06/07/2020 | Africa / Egypt | Human | unknown | unknown | unknown | unknown | unknown | unknown | B.1.1.1 | GR |
| hCoV-19/Egypt/NRC-6449/2020 | EPI_ISL_2232368 | 14/08/2020 | Africa / Egypt | Human | unknown | unknown | unknown | unknown | unknown | unknown | C.17 | GR |
| hCoV-19/Egypt/NRC-6465/2020 | EPI_ISL_2232367 | 17/08/2020 | Africa / Egypt | Human | unknown | unknown | unknown | unknown | unknown | unknown | C.17 | GH |
| hCoV-19/Egypt/NRC-6823/2020 | EPI_ISL_2232358 | 30/09/2020 | Africa / Egypt | Human | unknown | unknown | unknown | unknown | unknown | unknown | B.1 | GH |
| hCoV-19/Egypt/NRC-6942/2020 | EPI_ISL_2232356 | 21/10/2020 | Africa / Egypt | Human | unknown | unknown | unknown | unknown | unknown | unknown | B.1 | GH |
| hCoV-19/Egypt/NRC-6959/2020 | EPI_ISL_2232355 | 23/10/2020 | Africa / Egypt | Human | unknown | unknown | unknown | unknown | unknown | unknown | B.1 | GH |
| hCoV-19/Egypt/NRC-6985/2020 | EPI_ISL_2232354 | 26/10/2020 | Africa / Egypt | Human | unknown | unknown | unknown | unknown | unknown | unknown | B.1 | GH |
| hCoV-19/Egypt/NRC-6976/2020 | EPI_ISL_2232353 | 26/10/2020 | Africa / Egypt | Human | unknown | unknown | unknown | unknown | unknown | unknown | B.1 | GH |
| hCoV-19/Egypt/NRC-7021/2020 | EPI_ISL_2232352 | 29/10/2020 | Africa / Egypt | Human | unknown | unknown | unknown | unknown | unknown | unknown | B.1 | GH |
| hCoV-19/Egypt/NRC-6760/2020 | EPI_ISL_2232366 | 20/09/2020 | Africa / Egypt | Human | unknown | unknown | unknown | unknown | unknown | unknown | B.1 | GH |
| hCoV-19/Egypt/NRC-6817/2020 | EPI_ISL_2232365 | 27/09/2020 | Africa / Egypt | Human | unknown | unknown | unknown | unknown | unknown | unknown | B.1 | GH |
| hCoV-19/Egypt/NRC-6816/2020 | EPI_ISL_2232364 | 27/09/2020 | Africa / Egypt | Human | unknown | unknown | unknown | unknown | unknown | unknown | B.1 | GH |
| hCoV-19/Egypt/NRC-6819/2020 | EPI_ISL_2232363 | 27/09/2020 | Africa / Egypt | Human | unknown | unknown | unknown | unknown | unknown | unknown | B.1 | GH |
| hCoV-19/Egypt/NRC-6818/2020 | EPI_ISL_2232362 | 27/09/2020 | Africa / Egypt | Human | unknown | unknown | unknown | unknown | unknown | unknown | B.1 | GH |
| hCoV-19/Egypt/NRC-5442/2020 | EPI_ISL_2232349 | 27/05/2020 | Africa / Egypt | Human | unknown | Male | unknown | unknown | unknown | unknown | C.17 | GR |
| hCoV-19/Egypt/NRC-5445/2020 | EPI_ISL_2232348 | 27/05/2020 | Africa / Egypt | Human | unknown | Male | unknown | unknown | unknown | unknown | C.17 | GR |
| hCoV-19/Egypt/NRC-6841/2020 | EPI_ISL_2232351 | 04/10/2020 | Africa / Egypt | Human | unknown | unknown | unknown | unknown | unknown | unknown | B.1 | GH |
| hCoV-19/Egypt/NRC-5451/2020 | EPI_ISL_2232346 | 27/05/2020 | Africa / Egypt | Human | unknown | Male | unknown | unknown | unknown | unknown | C.17 | GR |
| hCoV-19/Egypt/NRC-462/2020 | EPI_ISL_2232345 | 30/05/2020 | Africa / Egypt | Human | unknown | Male | unknown | unknown | unknown | unknown | B.1.1 | GR |
| hCoV-19/Egypt/NRC-5462/2020 | EPI_ISL_2232344 | 30/05/2020 | Africa / Egypt | Human | unknown | Male | unknown | unknown | unknown | unknown | B.1.1 | GR |
| hCoV-19/Egypt/NRC-5767/2020 | EPI_ISL_2232342 | 10/06/2020 | Africa / Egypt | Human | unknown | Male | unknown | unknown | unknown | unknown | B.1 | G |
| hCoV-19/Egypt/NRC-5817/2020 | EPI_ISL_2232341 | 14/06/2020 | Africa / Egypt | Human | unknown | Male | unknown | unknown | unknown | unknown | B.1 | GH |
| hCoV-19/Egypt/NRC-5797/2020 | EPI_ISL_2232340 | 15/06/2020 | Africa / Egypt | Human | unknown | Male | unknown | unknown | unknown | unknown | B.1 | GH |
| hCoV-19/Egypt/NRC-5548/2020 | EPI_ISL_2232338 | 03/06/2020 | Africa / Egypt | Human | unknown | Male | unknown | unknown | unknown | unknown | C.17 | GR |
| hCoV-19/Egypt/NRC-6160/2020 | EPI_ISL_2232337 | 30/06/2020 | Africa / Egypt | Human | unknown | Male | unknown | unknown | unknown | unknown | B.1.1.1 | GR |
| hCoV-19/Egypt/NRC-6161/2020 | EPI_ISL_2232336 | 30/06/2020 | Africa / Egypt | Human | unknown | Male | unknown | unknown | unknown | unknown | B.1.1.1 | GR |
| hCoV-19/Egypt/NRC-6215/2020 | EPI_ISL_2232334 | 04/07/2020 | Africa / Egypt | Human | unknown | Male | unknown | unknown | unknown | unknown | C.17 | GR |
| hCoV-19/Egypt/NRC-6611/2020 | EPI_ISL_2232333 | 07/09/2020 | Africa / Egypt | Human | unknown | Male | unknown | unknown | unknown | unknown | C.17 | GR |
| hCoV-19/Egypt/NRC-6894/2020 | EPI_ISL_2232331 | 14/10/2020 | Africa / Egypt | Human | unknown | Male | unknown | unknown | unknown | unknown | C.17 | GR |
| hCoV-19/Egypt/NRC-5447/2020 | EPI_ISL_2232329 | 27/05/2020 | Africa / Egypt | Human | unknown | Female | unknown | unknown | unknown | unknown | C.17 | GR |
| hCoV-19/Egypt/NRC-5446/2020 | EPI_ISL_2232328 | 27/05/2020 | Africa / Egypt | Human | unknown | Female | unknown | unknown | unknown | unknown | C.17 | GR |
| hCoV-19/Egypt/NRC-5456/2020 | EPI_ISL_2232327 | 27/05/2020 | Africa / Egypt | Human | unknown | Female | unknown | unknown | unknown | unknown | B.1.1 | GR |
| hCoV-19/Egypt/NRC-5455/2020 | EPI_ISL_2232326 | 27/05/2020 | Africa / Egypt | Human | unknown | Female | unknown | unknown | unknown | unknown | C.17 | GR |
| hCoV-19/Egypt/NRC-6885/2020 | EPI_ISL_2232332 | 13/10/2020 | Africa / Egypt | Human | unknown | Male | unknown | unknown | unknown | unknown | C.17 | GR |
| hCoV-19/Egypt/NRC-5864/2020 | EPI_ISL_2232322 | 16/06/2020 | Africa / Egypt | Human | unknown | Female | unknown | unknown | unknown | unknown | B.1.170 | GH |
| hCoV-19/Egypt/NRC-5890/2020 | EPI_ISL_2232320 | 18/06/2020 | Africa / Egypt | Human | unknown | Female | unknown | unknown | unknown | unknown | B.1.195 | G |
| hCoV-19/Egypt/NRC-6012/2020 | EPI_ISL_2232318 | 23/06/2020 | Africa / Egypt | Human | unknown | Female | unknown | unknown | unknown | unknown | B.1.1 | GR |
| hCoV-19/Egypt/NRC-5587/2020 | EPI_ISL_2232317 | 04/06/2020 | Africa / Egypt | Human | unknown | Female | unknown | unknown | unknown | unknown | B.1 | GH |
| hCoV-19/Egypt/NRC-5643/2020 | EPI_ISL_2232315 | 07/06/2020 | Africa / Egypt | Human | unknown | Female | unknown | unknown | unknown | unknown | B.1.1 | GR |
| hCoV-19/Egypt/NRC-5611/2020 | EPI_ISL_2232314 | 07/06/2020 | Africa / Egypt | Human | unknown | Female | unknown | unknown | unknown | unknown | C.17 | GR |
| hCoV-19/Egypt/NRC-6374/2020 | EPI_ISL_2232312 | 27/07/2020 | Africa / Egypt | Human | unknown | Female | unknown | unknown | unknown | unknown | C.17 | GR |
| hCoV-19/Egypt/NRC-6504/2020 | EPI_ISL_2232310 | 20/08/2020 | Africa / Egypt | Human | unknown | Female | unknown | unknown | unknown | unknown | C.17 | GR |
| hCoV-19/Egypt/NRC-5464/2020 | EPI_ISL_2232325 | 30/05/2020 | Africa / Egypt | Human | unknown | Female | unknown | unknown | unknown | unknown | C.17 | GR |
| hCoV-19/Egypt/NRC-6410/2020 | EPI_ISL_2232308 | 05/08/2020 | Africa / Egypt | Human | unknown | Female | unknown | unknown | unknown | unknown | C.17 | GR |
| hCoV-19/Egypt/NRC-6834/2020 | EPI_ISL_2232305 | 04/10/2020 | Africa / Egypt | Human | unknown | Female | unknown | unknown | unknown | unknown | C.17 | GR |
| hCoV-19/Egypt/NRC-6837/2020 | EPI_ISL_2232304 | 04/10/2020 | Africa / Egypt | Human | unknown | Female | unknown | unknown | unknown | unknown | B.1 | GH |
| hCoV-19/Egypt/NRC-6117/2020 | EPI_ISL_2232303 | 28/06/2020 | Africa / Egypt | Human | unknown | unknown | unknown | unknown | unknown | unknown | B.1.170 | GH |
| hCoV-19/Egypt/NRC-5467/2020 | EPI_ISL_2232302 | 30/05/2020 | Africa / Egypt | Human | unknown | Female | unknown | unknown | unknown | unknown | B.1.1 | GR |
| hCoV-19/Egypt/NRC-6539/2020 | EPI_ISL_2232309 | 30/08/2020 | Africa / Egypt | Human | unknown | Female | unknown | unknown | unknown | unknown | B.1 | GH |
| hCoV-19/Egypt/NRC-5589/2020 | EPI_ISL_2232300 | 04/06/2020 | Africa / Egypt | Human | unknown | Female | unknown | unknown | unknown | unknown | B.1.1 | GR |
| hCoV-19/Egypt/NRC-5882/2020 | EPI_ISL_2232296 | 17/06/2020 | Africa / Egypt | Human | unknown | unknown | unknown | unknown | unknown | unknown | B.1.170 | GH |
| hCoV-19/Egypt/NRC-6076/2020 | EPI_ISL_2232297 | 27/06/2020 | Africa / Egypt | Human | unknown | Male | unknown | unknown | unknown | unknown | B.1 | GH |
| hCoV-19/Egypt/NRC-5990/2020 | EPI_ISL_2232295 | 22/06/2020 | Africa / Egypt | Human | unknown | unknown | unknown | unknown | unknown | unknown | B.1.195 | G |
| hCoV-19/Egypt/NRC-6001/2020 | EPI_ISL_2232294 | 23/06/2020 | Africa / Egypt | Human | unknown | Female | unknown | unknown | unknown | unknown | C.17 | GR |
| hCoV-19/Egypt/NRC-5912/2020 | EPI_ISL_2232293 | 19/06/2020 | Africa / Egypt | Human | unknown | unknown | unknown | unknown | unknown | unknown | B.1 | GH |
| hCoV-19/Egypt/NRC-5623/2020 | EPI_ISL_2232292 | 07/06/2020 | Africa / Egypt | Human | unknown | Female | unknown | unknown | unknown | unknown | C.17 | GR |
| hCoV-19/Egypt/NRC-5873/2020 | EPI_ISL_2232291 | 15/06/2020 | Africa / Egypt | Human | unknown | unknown | unknown | unknown | unknown | unknown | B.1.170 | GH |
| hCoV-19/Egypt/NRC-5880/2020 | EPI_ISL_2232290 | 17/06/2020 | Africa / Egypt | Human | unknown | unknown | unknown | unknown | unknown | unknown | B.1.170 | GH |
| hCoV-19/Egypt/NRC-5911/2020 | EPI_ISL_2232289 | 19/06/2020 | Africa / Egypt | Human | unknown | unknown | unknown | unknown | unknown | unknown | B.1 | GH |
| hCoV-19/Egypt/NRC-5980/2020 | EPI_ISL_2232287 | 23/06/2020 | Africa / Egypt | Human | unknown | unknown | unknown | unknown | unknown | unknown | B.1 | GH |
| hCoV-19/Egypt/NRC-6051/2020 | EPI_ISL_2232285 | 24/06/2020 | Africa / Egypt | Human | unknown | unknown | unknown | unknown | unknown | unknown | B.1 | GH |
| hCoV-19/Egypt/NRC-5900/2020 | EPI_ISL_2232286 | 23/06/2020 | Africa / Egypt | Human | unknown | unknown | unknown | unknown | unknown | unknown | B.1 | GH |
| hCoV-19/Egypt/NRC-6099/2020 | EPI_ISL_2232284 | 28/06/2020 | Africa / Egypt | Human | unknown | unknown | unknown | unknown | unknown | unknown | B.1 | GH |
| hCoV-19/Egypt/NRC-6141/2020 | EPI_ISL_2232283 | 29/06/2020 | Africa / Egypt | Human | unknown | unknown | unknown | unknown | unknown | unknown | B.1 | GH |
| hCoV-19/Egypt/NRC-6179/2020 | EPI_ISL_2232282 | 30/06/2020 | Africa / Egypt | Human | unknown | unknown | unknown | unknown | unknown | unknown | B.1 | GH |
| hCoV-19/Egypt/NRC-6181/2020 | EPI_ISL_2232280 | 30/06/2020 | Africa / Egypt | Human | unknown | unknown | unknown | unknown | unknown | unknown | B.1 | GH |
| hCoV-19/Egypt/NRC-6180/2020 | EPI_ISL_2232281 | 30/06/2020 | Africa / Egypt | Human | unknown | unknown | unknown | unknown | unknown | unknown | B.1 | GH |
| hCoV-19/Egypt/NRC-6168/2020 | EPI_ISL_2232279 | 01/07/2020 | Africa / Egypt | Human | unknown | unknown | unknown | unknown | unknown | unknown | B.1.1.1 | GR |
| hCoV-19/Egypt/NRC-6375/2020 | EPI_ISL_2232276 | 26/07/2020 | Africa / Egypt | Human | unknown | unknown | unknown | unknown | unknown | unknown | C.17 | GR |
| hCoV-19/Egypt/NRC-6317/2020 | EPI_ISL_2232278 | 14/07/2020 | Africa / Egypt | Human | unknown | unknown | unknown | unknown | unknown | unknown | C.17 | G |
| hCoV-19/Egypt/NRC-6839/2020 | EPI_ISL_2232273 | 04/10/2020 | Africa / Egypt | Human | unknown | unknown | unknown | unknown | unknown | unknown | B.1 | GH |
| hCoV-19/Egypt/NRC-6636/2020 | EPI_ISL_2232275 | 10/09/2020 | Africa / Egypt | Human | unknown | unknown | unknown | unknown | unknown | unknown | C.17 | GR |
| hCoV-19/Egypt/NRC-7011/2020 | EPI_ISL_2232274 | 29/10/2020 | Africa / Egypt | Human | unknown | unknown | unknown | unknown | unknown | unknown | B.1 | GH |
| hCoV-19/Egypt/NRC-6186/2020 | EPI_ISL_2232271 | 01/07/2020 | Africa / Egypt | Human | unknown | Male | unknown | unknown | unknown | unknown | B.1 | GH |
| hCoV-19/Egypt/NRC-6943/2020 | EPI_ISL_2232270 | 21/10/2020 | Africa / Egypt | Human | unknown | Male | unknown | unknown | unknown | unknown | B.1.1.312 | GR |
| hCoV-19/Egypt/NRC-6069/2020 | EPI_ISL_2232272 | 25/06/2020 | Africa / Egypt | Human | unknown | Male | unknown | unknown | unknown | unknown | B.1 | GH |
| hCoV-19/Egypt/NRC-6957/2020 | EPI_ISL_2232269 | 21/10/2020 | Africa / Egypt | Human | unknown | Male | unknown | unknown | unknown | unknown | B.1 | GH |
| hCoV-19/Egypt/NRC-5836/2020 | EPI_ISL_2232268 | 16/06/2020 | Africa / Egypt | Human | unknown | Female | unknown | unknown | unknown | unknown | B.1 | GH |
| hCoV-19/Egypt/NRC-6015/2020 | EPI_ISL_2232266 | 23/06/2020 | Africa / Egypt | Human | unknown | Female | unknown | unknown | unknown | unknown | C.17 | GR |
| hCoV-19/Egypt/NRC-5852/2020 | EPI_ISL_2232267 | 17/06/2020 | Africa / Egypt | Human | unknown | Female | unknown | unknown | unknown | unknown | C.17 | GR |
| hCoV-19/Egypt/NRC-6000/2020 | EPI_ISL_2232265 | 23/06/2020 | Africa / Egypt | Human | unknown | Female | unknown | unknown | unknown | unknown | C.17 | GR |
| hCoV-19/Egypt/NRC-6369/2020 | EPI_ISL_2232263 | 25/07/2020 | Africa / Egypt | Human | unknown | Female | unknown | unknown | unknown | unknown | C.17 | GR |
| hCoV-19/Egypt/NRC-6264/2020 | EPI_ISL_2232262 | 04/07/2020 | Africa / Egypt | Human | unknown | Female | unknown | unknown | unknown | unknown | B.1.1.1 | GR |
| hCoV-19/Egypt/NRC-6089/2020 | EPI_ISL_2232264 | 28/06/2020 | Africa / Egypt | Human | unknown | Female | unknown | unknown | unknown | unknown | C.17 | GR |
| hCoV-19/Egypt/NRC-6883/2020 | EPI_ISL_2232260 | 13/10/2020 | Africa / Egypt | Human | unknown | Female | unknown | unknown | unknown | unknown | C.17 | GR |
| hCoV-19/Egypt/NRC-6737/2020 | EPI_ISL_2232258 | 18/09/2020 | Africa / Egypt | Human | unknown | unknown | unknown | unknown | unknown | unknown | C.17 | GR |
| hCoV-19/Egypt/NRC-6143/2020 | EPI_ISL_2232256 | 29/06/2020 | Africa / Egypt | Human | unknown | unknown | unknown | unknown | unknown | unknown | B.1 | G |
| hCoV-19/Egypt/NRC-6166/2020 | EPI_ISL_2232255 | 01/07/2020 | Africa / Egypt | Human | unknown | unknown | unknown | unknown | unknown | unknown | B.1.1.1 | GR |
| hCoV-19/Egypt/NRC-6177/2020 | EPI_ISL_2232253 | 30/06/2020 | Africa / Egypt | Human | unknown | unknown | unknown | unknown | unknown | unknown | B.1 | GH |
| hCoV-19/Egypt/NRC-5756/2020 | EPI_ISL_2232252 | 10/06/2020 | Africa / Egypt | Human | unknown | unknown | unknown | unknown | unknown | unknown | B.1.1 | GR |
| hCoV-19/Egypt/NRC-488/2021 | EPI_ISL_2227350 | 18/04/2021 | Africa / Egypt | Human | unknown | Male | unknown | unknown | unknown | unknown | C.36.3 | GR |
| hCoV-19/Egypt/NRC-329/2021 | EPI_ISL_2227349 | 15/03/2021 | Africa / Egypt | Human | unknown | Female | unknown | unknown | unknown | unknown | C.36.3 | GR |
| hCoV-19/Egypt/NRC-287/2021 | EPI_ISL_2227348 | 07/03/2021 | Africa / Egypt | Human | unknown | Female | unknown | unknown | unknown | unknown | C.36.3 | GR |
| hCoV-19/Egypt/NRC-337/2021 | EPI_ISL_2227347 | 17/03/2021 | Africa / Egypt | Human | unknown | Female | unknown | unknown | unknown | unknown | C.36.3 | GR |
| hCoV-19/Egypt/NRC-295/2021 | EPI_ISL_2227345 | 08/03/2021 | Africa / Egypt | Human | unknown | Female | unknown | unknown | unknown | unknown | C.36.3 | GR |
| hCoV-19/Egypt/NRC-518/2020 | EPI_ISL_2227344 | 26/04/2020 | Africa / Egypt | Human | unknown | Female | unknown | unknown | unknown | unknown | C.36.3 | GR |
| hCoV-19/Egypt/NRC-407/2021 | EPI_ISL_2227343 | 31/03/2021 | Africa / Egypt | Human | unknown | Female | unknown | unknown | unknown | unknown | C.17 | GR |
| hCoV-19/Egypt/NRC-433/2021 | EPI_ISL_2227342 | 06/04/2021 | Africa / Egypt | Human | unknown | Female | unknown | unknown | unknown | unknown | C.36.3 | GR |
| hCoV-19/Egypt/NRC-369/2021 | EPI_ISL_2227341 | 24/03/2021 | Africa / Egypt | Human | unknown | Female | unknown | unknown | unknown | unknown | C.36.3 | GR |
| hCoV-19/Egypt/NRC-335/2021 | EPI_ISL_2227340 | 16/03/2021 | Africa / Egypt | Human | unknown | Female | unknown | unknown | unknown | unknown | C.17 | GR |
| hCoV-19/Egypt/NRC-434/2021 | EPI_ISL_2227339 | 06/04/2021 | Africa / Egypt | Human | unknown | Female | unknown | unknown | unknown | unknown | C.36.3 | GR |
| hCoV-19/Egypt/NRC-435/2021 | EPI_ISL_2227338 | 07/04/2021 | Africa / Egypt | Human | unknown | Female | unknown | unknown | unknown | unknown | C.36.3 | GR |
| hCoV-19/Egypt/NRC-465/2021 | EPI_ISL_2227337 | 11/04/2021 | Africa / Egypt | Human | unknown | Male | unknown | unknown | unknown | unknown | C.36.3 | GR |
| hCoV-19/Egypt/NRC-291/2021 | EPI_ISL_2227335 | 07/03/2021 | Africa / Egypt | Human | unknown | Male | unknown | unknown | unknown | unknown | C.36.3 | GR |
| hCoV-19/Egypt/NRC-440/2021 | EPI_ISL_2227334 | 07/04/2021 | Africa / Egypt | Human | unknown | Male | unknown | unknown | unknown | unknown | C.36.3 | GR |
| hCoV-19/Egypt/NRC-431/2021 | EPI_ISL_2227333 | 06/04/2021 | Africa / Egypt | Human | unknown | Female | unknown | unknown | unknown | unknown | C.17 | GR |
| hCoV-19/Egypt/NRC-415/2021 | EPI_ISL_2227332 | 04/04/2021 | Africa / Egypt | Human | unknown | Female | unknown | unknown | unknown | unknown | C.36.3 | GR |
| hCoV-19/Egypt/NRC-515/2021 | EPI_ISL_2227331 | 26/04/2021 | Africa / Egypt | Human | unknown | Male | unknown | unknown | unknown | unknown | C.36.3 | GR |
| hCoV-19/Egypt/NRC-289/2021 | EPI_ISL_2227330 | 07/03/2021 | Africa / Egypt | Human | unknown | Male | unknown | unknown | unknown | unknown | C.36.3 | GR |
| hCoV-19/Egypt/NRC-481/2021 | EPI_ISL_2227328 | 18/04/2021 | Africa / Egypt | Human | unknown | Female | unknown | unknown | unknown | unknown | C.36.3 | GR |
| hCoV-19/dog/Egypt/AHRI-5/2020 | EPI_ISL_2932464 | 2020-12 | Africa / Egypt | Human | unknown | unknown | unknown | unknown | unknown | unknown | Unassigned | O |
| hCoV-19/cat/Egypt/AHRI-4/2020 | EPI_ISL_2932463 | 2020-12 | Africa / Egypt | Human | unknown | unknown | unknown | unknown | unknown | unknown | Unassigned | O |
| hCoV-19/Egypt/CCHE57357_Wave_3_A_060/2021 | EPI_ISL_2566528 | 05/03/2021 | Africa / Egypt | Human | unknown | unknown | unknown | unknown | unknown | unknown | B | L |
| hCoV-19/Egypt/NRC-6822/2020 | EPI_ISL_2232359 | 30/09/2020 | Africa / Egypt | Human | unknown | unknown | unknown | unknown | unknown | unknown | B.1 | GH |
| hCoV-19/Egypt/NRC-6821/2020 | EPI_ISL_2232360 | 30/09/2020 | Africa / Egypt | Human | unknown | unknown | unknown | unknown | unknown | unknown | B.1 | GH |
| hCoV-19/Egypt/PHARCO-ARMY-49/2021 | EPI_ISL_1936309 | 12/02/2021 | Africa / Egypt / Cairo | Human | unknown | unknown | 25-55 | Hospitalized | unknown | unknown | C.36 | GR |
| hCoV-19/Egypt/ARMY-363/2021 | EPI_ISL_1936293 | 04/04/2021 | Africa / Egypt / Cairo | Human | unknown | unknown | 25-60 | Hospitalized | unknown | unknown | C.17 | GR |
| hCoV-19/Egypt/CCHE57357-A-13/2020 | EPI_ISL_812793 | 29/04/2020 | Africa / Egypt | Human | unknown | unknown | unknown | unknown | unknown | unknown | B.1.1 | O |
| hCoV-19/Egypt/CPHL-A9/2021 | EPI_ISL_3274164 | 26/03/2021 | Africa / Egypt / Alexandria | Human | Sentinel surveillance (ILI) | Male | 55 | unknown | unknown | Sentinel surveillance (ILI) | B.1 | GH |
| hCoV-19/Egypt/CPHL-A4/2021 | EPI_ISL_3274160 | 06/01/2021 | Africa / Egypt / Sohage | Human | Sentinel surveillance (ILI) | Male | 46 | unknown | unknown | Sentinel surveillance (ILI) | C.36.3 | GR |
| hCoV-19/Egypt/CPHL-S25/2021 | EPI_ISL_3274157 | 08/07/2021 | Africa / Egypt / Damitta | Human | Sentinel surveillance (ILI) | Male | 42 | unknown | unknown | Sentinel surveillance (ILI) | Unassigned | GR |
| hCoV-19/Egypt/CPHL-S7/2021 | EPI_ISL_3274154 | 06/06/2021 | Africa / Egypt / Giza | Human | Sentinel surveillance (ILI) | Male | 32 | unknown | unknown | Sentinel surveillance (ILI) | C.36 | GR |
| hCoV-19/Egypt/ARMY-318/2021 | EPI_ISL_1936257 | 22/04/2021 | Africa / Egypt / Cairo | Human | unknown | unknown | 25-60 | Hospitalized | unknown | unknown | A.28 | S |
| hCoV-19/Egypt/ARMY-65/2021 | EPI_ISL_1936221 | 27/03/2021 | Africa / Egypt / Cairo | Human | unknown | unknown | 25-55 | Hospitalized | unknown | unknown | A | S |
| hCoV-19/Egypt/PHARCO-ARMY-83/2021 | EPI_ISL_1936367 | 12/02/2021 | Africa / Egypt / Cairo | Human | unknown | unknown | 25-55 | Hospitalized | unknown | unknown | C.36 | GR |
| hCoV-19/Egypt/PHARCO-ARMY-33/2021 | EPI_ISL_1936352 | 12/02/2021 | Africa / Egypt / Cairo | Human | unknown | unknown | 25-55 | Hospitalized | unknown | unknown | C.36 | GR |
| hCoV-19/Egypt/PHARCO-ARMY-92/2021 | EPI_ISL_1936356 | 12/02/2021 | Africa / Egypt / Cairo | Human | unknown | unknown | 25-55 | Hospitalized | unknown | unknown | C.36 | GR |
| hCoV-19/Egypt/PHARCO-ARMY-67/2021 | EPI_ISL_1936327 | 12/02/2021 | Africa / Egypt / Cairo | Human | unknown | unknown | 25-55 | Hospitalized | unknown | unknown | C.36 | GR |
| hCoV-19/Egypt/PHARCO-ARMY-55/2021 | EPI_ISL_1936315 | 12/02/2021 | Africa / Egypt / Cairo | Human | unknown | unknown | 25-55 | Hospitalized | unknown | unknown | C.36 | GR |
| hCoV-19/Egypt/ARMY-60/2021 | EPI_ISL_1936236 | 27/03/2021 | Africa / Egypt / Cairo | Human | unknown | unknown | 25-55 | Hospitalized | unknown | unknown | C.17 | GR |
| hCoV-19/Egypt/ARMY-23/2021 | EPI_ISL_1936197 | 27/03/2021 | Africa / Egypt / Cairo | Human | unknown | unknown | 25-55 | Hospitalized | unknown | unknown | B.1.170 | GH |
| hCoV-19/Egypt/ARMY-EVA-Pharma-Wave4-028/2021 | EPI_ISL_4750221 | 04/09/2021 | Africa / Egypt / Cairo | Human | unknown | unknown | 25-55 | Hospitalized | unknown | unknown | B.1.617.2 | O |
| hCoV-19/Egypt/ARMY-EVA-Pharma-Wave4-019/2021 | EPI_ISL_4750219 | 04/09/2021 | Africa / Egypt / Cairo | Human | unknown | unknown | 25-55 | Hospitalized | unknown | unknown | B.1.617.2 | GK |
| hCoV-19/Egypt/ARMY-EVA-Pharma-Wave4-009/2021 | EPI_ISL_4750217 | 04/09/2021 | Africa / Egypt / Cairo | Human | unknown | unknown | 25-55 | Hospitalized | unknown | unknown | B.1.617.2 | GK |
| hCoV-19/Egypt/ARMY-EVA-Pharma-Wave4-026/2021 | EPI_ISL_4748283 | 04/09/2021 | Africa / Egypt / Cairo | Human | unknown | unknown | 25-55 | Hospitalized | unknown | unknown | AY.113 | GK |
| hCoV-19/Egypt/ARMY-EVA-Pharma-Wave4-024/2021 | EPI_ISL_4748282 | 04/09/2021 | Africa / Egypt / Cairo | Human | unknown | unknown | 25-55 | Hospitalized | unknown | unknown | B.1.617.2 | GK |
| hCoV-19/Egypt/ARMY-EVA-Pharma-Wave4-014/2021 | EPI_ISL_4748273 | 04/09/2021 | Africa / Egypt / Cairo | Human | unknown | unknown | 25-55 | Hospitalized | unknown | unknown | B.1.617.2 | GK |
| hCoV-19/Egypt/PHARCO-ARMY-97/2021 | EPI_ISL_1936361 | 12/02/2021 | Africa / Egypt / Cairo | Human | unknown | unknown | 25-55 | Hospitalized | unknown | unknown | C.36 | GR |
| hCoV-19/Egypt/Delta007/2021 | EPI_ISL_4629983 | 04/08/2021 | Africa / Egypt / Cairo | Human | unknown | unknown | unknown | Hospitalized | unknown | unknown | AY.43 | GK |
| hCoV-19/Egypt/Delta002/2021 | EPI_ISL_4629978 | 04/08/2021 | Africa / Egypt / Cairo | Human | unknown | unknown | unknown | Hospitalized | unknown | unknown | B.1.617.2 | GK |
| hCoV-19/Egypt/PHARCO-ARMY-74/2021 | EPI_ISL_1936334 | 12/02/2021 | Africa / Egypt / Cairo | Human | unknown | unknown | 25-55 | Hospitalized | unknown | unknown | B.1 | G |
| hCoV-19/Egypt/CPHL-NRC-6/2020 | EPI_ISL_794606 | 15/03/2020 | Africa / Egypt | Human | unknown | unknown | unknown | unknown | unknown | unknown | B.1 | G |
| hCoV-19/Egypt/CPHL-NRC-9/2020 | EPI_ISL_794605 | 21/05/2020 | Africa / Egypt | Human | unknown | unknown | unknown | unknown | unknown | unknown | Unassigned | G |
| hCoV-19/Egypt/CPHL-NRC-5/2020 | EPI_ISL_794607 | 15/05/2020 | Africa / Egypt | Human | unknown | unknown | unknown | unknown | unknown | unknown | Unassigned | G |
| hCoV-19/Egypt/CPHL-NRC-4/2020 | EPI_ISL_794608 | 07/04/2020 | Africa / Egypt | Human | unknown | unknown | unknown | unknown | unknown | unknown | B.1.1 | GR |
| hCoV-19/Egypt/NRC-5530/2020 | EPI_ISL_8189543 | 02/06/2020 | Africa / Egypt | Human | unknown | unknown | unknown | unknown | unknown | unknown | B.1.170 | GH |
| hCoV-19/Egypt/NRC-7282/2020 | EPI_ISL_8193643 | 14/12/2020 | Africa / Egypt | Human | unknown | unknown | unknown | unknown | unknown | unknown | B.1.170 | G |
| hCoV-19/Egypt/NRC-5878/2020 | EPI_ISL_2232394 | 17/06/2020 | Africa / Egypt | Human | unknown | unknown | unknown | unknown | unknown | unknown | B.1.170 | GH |
| hCoV-19/Egypt/NRC-5844/2020 | EPI_ISL_2232393 | 17/06/2020 | Africa / Egypt | Human | unknown | unknown | unknown | unknown | unknown | unknown | B.1 | GH |
| hCoV-19/Egypt/NRC-5870/2020 | EPI_ISL_2232398 | 15/06/2020 | Africa / Egypt | Human | unknown | unknown | unknown | unknown | unknown | unknown | B.1 | G |
| hCoV-19/Egypt/NRC-6630/2020 | EPI_ISL_2232357 | 09/09/2020 | Africa / Egypt | Human | unknown | unknown | unknown | unknown | unknown | unknown | B.1.195 | G |
| hCoV-19/Egypt/NRC-5886/2020 | EPI_ISL_2232301 | 18/06/2020 | Africa / Egypt | Human | unknown | Female | unknown | unknown | unknown | unknown | B.1.1 | GR |
| hCoV-19/Egypt/NRC-5540/2020 | EPI_ISL_2232319 | 02/06/2020 | Africa / Egypt | Human | unknown | Female | unknown | unknown | unknown | unknown | C.17 | GR |
| hCoV-19/Egypt/NRC-6377/2020 | EPI_ISL_2232277 | 26/07/2020 | Africa / Egypt | Human | unknown | unknown | unknown | unknown | unknown | unknown | C.17 | GR |
| hCoV-19/Egypt/NRC-5982/2020 | EPI_ISL_2232288 | 23/06/2020 | Africa / Egypt | Human | unknown | unknown | unknown | unknown | unknown | unknown | B.1 | GH |
| hCoV-19/Egypt/NRC-6073/2020 | EPI_ISL_2232299 | 27/06/2020 | Africa / Egypt | Human | unknown | unknown | unknown | unknown | unknown | unknown | B.1 | GH |
| hCoV-19/Egypt/NRC-6021/2020 | EPI_ISL_2232298 | 23/06/2020 | Africa / Egypt | Human | unknown | Male | unknown | unknown | unknown | unknown | B.1 | GH |
| hCoV-19/Egypt/CCHE57357-A-55/2020 | EPI_ISL_812828 | 11/08/2020 | Africa / Egypt | Human | unknown | unknown | unknown | unknown | unknown | unknown | B | O |
| hCoV-19/Egypt/ARMY-319/2021 | EPI_ISL_1936258 | 22/04/2021 | Africa / Egypt / Cairo | Human | unknown | unknown | 25-60 | Hospitalized | unknown | unknown | A.28 | S |
| hCoV-19/Egypt/CCHE57357-A-69/2020 | EPI_ISL_812839 | 29/06/2020 | Africa / Egypt | Human | unknown | unknown | unknown | unknown | unknown | unknown | B | L |
| hCoV-19/Egypt/CPHL-NRC-S19/2021 | EPI_ISL_2313050 | 2021-03 | Africa / Egypt | Human | unknown | unknown | unknown | unknown | unknown | unknown | C.36.3 | GR |
| hCoV-19/Egypt/PHARCO-ARMY-99/2021 | EPI_ISL_1936363 | 12/02/2021 | Africa / Egypt / Cairo | Human | unknown | unknown | 25-55 | Hospitalized | unknown | unknown | B.1 | GH |
| hCoV-19/Egypt/CPHL-01/2021 | EPI_ISL_2227195 | 16/03/2021 | Africa / Egypt | Human | Sentinel surveillance (ILI) | unknown | unknown | Live | unknown | Sentinel surveillance (ILI) | C.36.3 | GR |
| hCoV-19/Egypt/PHARCO-ARMY-82/2021 | EPI_ISL_1936366 | 12/02/2021 | Africa / Egypt / Cairo | Human | unknown | unknown | 25-55 | Hospitalized | unknown | unknown | C.36 | GR |
| hCoV-19/cat/Egypt/AHRI/2021 | EPI_ISL_2696281 | 15/06/2021 | Africa / Egypt | Human | unknown | unknown | unknown | unknown | unknown | unknown | Unassigned | O |
| hCoV-19/Egypt/CCHE57357_Wave_3_A_072/2021 | EPI_ISL_2566529 | 09/05/2021 | Africa / Egypt | Human | unknown | unknown | unknown | unknown | unknown | unknown | C.36.3 | GR |
| hCoV-19/Egypt/CCHE57357_Wave_3_A_048/2021 | EPI_ISL_2566525 | 14/03/2021 | Africa / Egypt | Human | unknown | unknown | unknown | unknown | unknown | unknown | C.36.3 | GR |
| hCoV-19/Egypt/CCHE57357_Wave_3_A_037/2021 | EPI_ISL_2566524 | 04/03/2021 | Africa / Egypt | Human | unknown | unknown | unknown | unknown | unknown | unknown | C.36.3 | O |
| hCoV-19/Egypt/CCHE57357_Wave_3_A_027/2021 | EPI_ISL_2566522 | 06/05/2021 | Africa / Egypt | Human | unknown | unknown | unknown | unknown | unknown | unknown | B | O |
| hCoV-19/Egypt/CCHE57357_Wave_3_A_023/2021 | EPI_ISL_2566520 | 08/05/2021 | Africa / Egypt | Human | unknown | unknown | unknown | unknown | unknown | unknown | B | L |
| hCoV-19/Egypt/CCHE57357_Wave_3_A_011/2021 | EPI_ISL_2566519 | 17/05/2021 | Africa / Egypt | Human | unknown | unknown | unknown | unknown | unknown | unknown | B | L |
| hCoV-19/Egypt/CCHE57357_Wave_3_A_009/2021 | EPI_ISL_2566518 | 04/05/2021 | Africa / Egypt | Human | unknown | unknown | unknown | unknown | unknown | unknown | B | L |
| hCoV-19/Egypt/CCHE57357_Wave_3_A008/2021 | EPI_ISL_2566515 | 05/05/2021 | Africa / Egypt | Human | unknown | unknown | unknown | unknown | unknown | unknown | C.38 | O |
| hCoV-19/Egypt/CCHE57357_Wave_3_A074/2021 | EPI_ISL_2566514 | 14/05/2021 | Africa / Egypt | Human | unknown | unknown | unknown | unknown | unknown | unknown | C.36.3 | GR |
| hCoV-19/Egypt/CCHE57357_Wave_3_A073/2021 | EPI_ISL_2566513 | 09/05/2021 | Africa / Egypt | Human | unknown | unknown | unknown | unknown | unknown | unknown | C.36.3 | GR |
| hCoV-19/Egypt/CCHE57357_Wave_3_A067/2021 | EPI_ISL_2566509 | 10/05/2021 | Africa / Egypt | Human | unknown | unknown | unknown | unknown | unknown | unknown | C.36.3 | GR |
| hCoV-19/Egypt/CCHE57357_Wave_3_A066/2021 | EPI_ISL_2566508 | 10/05/2021 | Africa / Egypt | Human | unknown | unknown | unknown | unknown | unknown | unknown | C.36.3 | GR |
| hCoV-19/Egypt/CCHE57357_Wave_3_A064/2021 | EPI_ISL_2566507 | 03/05/2021 | Africa / Egypt | Human | unknown | unknown | unknown | unknown | unknown | unknown | C.17 | GR |
| hCoV-19/Egypt/CCHE57357_Wave_3_A063/2021 | EPI_ISL_2566506 | 17/05/2021 | Africa / Egypt | Human | unknown | unknown | unknown | unknown | unknown | unknown | C.36.3 | GR |
| hCoV-19/Egypt/CCHE57357_Wave_3_A059/2021 | EPI_ISL_2566505 | 07/03/2021 | Africa / Egypt | Human | unknown | unknown | unknown | unknown | unknown | unknown | B.1.1.7 | GR |
| hCoV-19/Egypt/CCHE57357_Wave_3_A058/2021 | EPI_ISL_2566504 | 01/03/2021 | Africa / Egypt | Human | unknown | unknown | unknown | unknown | unknown | unknown | C.17 | GR |
| hCoV-19/Egypt/CCHE57357_Wave_3_A053/2021 | EPI_ISL_2566501 | 04/03/2021 | Africa / Egypt | Human | unknown | unknown | unknown | unknown | unknown | unknown | C.17 | GR |
| hCoV-19/Egypt/CCHE57357_Wave_3_A049/2021 | EPI_ISL_2566498 | 01/03/2021 | Africa / Egypt | Human | unknown | unknown | unknown | unknown | unknown | unknown | C.36.3 | GR |
| hCoV-19/Egypt/CCHE57357_Wave_3_A044/2021 | EPI_ISL_2566495 | 07/03/2021 | Africa / Egypt | Human | unknown | unknown | unknown | unknown | unknown | unknown | C.36.3 | GR |
| hCoV-19/Egypt/CCHE57357_Wave_3_A043/2021 | EPI_ISL_2566494 | 06/03/2021 | Africa / Egypt | Human | unknown | unknown | unknown | unknown | unknown | unknown | C.36.3 | GR |
| hCoV-19/Egypt/CCHE57357_Wave_3_A041/2021 | EPI_ISL_2566493 | 20/03/2021 | Africa / Egypt | Human | unknown | unknown | unknown | unknown | unknown | unknown | C.17 | GR |
| hCoV-19/Egypt/CCHE57357_Wave_3_A038/2021 | EPI_ISL_2566491 | 07/03/2021 | Africa / Egypt | Human | unknown | unknown | unknown | unknown | unknown | unknown | C.36.3 | O |
| hCoV-19/Egypt/CCHE57357_Wave_3_A028/2021 | EPI_ISL_2566485 | 14/05/2021 | Africa / Egypt | Human | unknown | unknown | unknown | unknown | unknown | unknown | C.36.3 | GR |
| hCoV-19/Egypt/CCHE57357_Wave_3_A022/2021 | EPI_ISL_2566482 | 15/05/2021 | Africa / Egypt | Human | unknown | unknown | unknown | unknown | unknown | unknown | B | L |
| hCoV-19/Egypt/CCHE57357_Wave_3_A020/2021 | EPI_ISL_2566480 | 15/05/2021 | Africa / Egypt | Human | unknown | unknown | unknown | unknown | unknown | unknown | C.36.3 | GR |
| hCoV-19/Egypt/CCHE57357_Wave_3_A019/2021 | EPI_ISL_2566479 | 08/05/2021 | Africa / Egypt | Human | unknown | unknown | unknown | unknown | unknown | unknown | B.1.1.7 | GR |
| hCoV-19/Egypt/CCHE57357_Wave_3_A018/2021 | EPI_ISL_2566478 | 07/05/2021 | Africa / Egypt | Human | unknown | unknown | unknown | unknown | unknown | unknown | C.36.3 | O |
| hCoV-19/Egypt/CCHE57357_Wave_3_A013/2021 | EPI_ISL_2566474 | 17/05/2021 | Africa / Egypt | Human | unknown | unknown | unknown | unknown | unknown | unknown | B.1.1.7 | GR |
| hCoV-19/Egypt/CCHE57357_Wave_3_A006/2021 | EPI_ISL_2566471 | 01/03/2021 | Africa / Egypt | Human | unknown | unknown | unknown | unknown | unknown | unknown | C.36.3 | GR |
| hCoV-19/Egypt/CCHE57357_Wave_3_A004/2021 | EPI_ISL_2566470 | 01/05/2021 | Africa / Egypt | Human | unknown | unknown | unknown | unknown | unknown | unknown | C.36.3 | GR |
| hCoV-19/Egypt/CCHE57357_Wave_3_A003/2021 | EPI_ISL_2566469 | 01/05/2021 | Africa / Egypt | Human | unknown | unknown | unknown | unknown | unknown | unknown | C.36.3 | GR |
| hCoV-19/Egypt/CCHE57357_Wave_3_A002/2021 | EPI_ISL_2566468 | 01/05/2021 | Africa / Egypt | Human | unknown | unknown | unknown | unknown | unknown | unknown | C.36.3 | GR |
| hCoV-19/Egypt/CCHE57357_Wave_3_A001/2021 | EPI_ISL_2566467 | 01/05/2021 | Africa / Egypt | Human | unknown | unknown | unknown | unknown | unknown | unknown | C.36.3 | GR |
| hCoV-19/Egypt/PHARCO-ARMY-89/2021 | EPI_ISL_1936373 | 12/02/2021 | Africa / Egypt / Cairo | Human | unknown | unknown | 25-55 | Hospitalized | unknown | unknown | B.1 | GH |
| hCoV-19/Egypt/PHARCO-ARMY-86/2021 | EPI_ISL_1936370 | 12/02/2021 | Africa / Egypt / Cairo | Human | unknown | unknown | 25-55 | Hospitalized | unknown | unknown | C.36 | GR |
| hCoV-19/Egypt/PHARCO-ARMY-100/2021 | EPI_ISL_1936364 | 12/02/2021 | Africa / Egypt / Cairo | Human | unknown | unknown | 25-55 | Hospitalized | unknown | unknown | C.36 | GR |
| hCoV-19/Egypt/PHARCO-ARMY-98/2021 | EPI_ISL_1936362 | 12/02/2021 | Africa / Egypt / Cairo | Human | unknown | unknown | 25-55 | Hospitalized | unknown | unknown | B.1 | GH |
| hCoV-19/Egypt/PHARCO-ARMY-96/2021 | EPI_ISL_1936360 | 12/02/2021 | Africa / Egypt / Cairo | Human | unknown | unknown | 25-55 | Hospitalized | unknown | unknown | C.36 | GR |
| hCoV-19/Egypt/PHARCO-ARMY-95/2021 | EPI_ISL_1936359 | 12/02/2021 | Africa / Egypt / Cairo | Human | unknown | unknown | 25-55 | Hospitalized | unknown | unknown | C.36 | GR |
| hCoV-19/Egypt/PHARCO-ARMY-35/2021 | EPI_ISL_1936354 | 12/02/2021 | Africa / Egypt / Cairo | Human | unknown | unknown | 25-55 | Hospitalized | unknown | unknown | C.36 | GR |
| hCoV-19/Egypt/PHARCO-ARMY-34/2021 | EPI_ISL_1936353 | 12/02/2021 | Africa / Egypt / Cairo | Human | unknown | unknown | 25-55 | Hospitalized | unknown | unknown | C.36 | GR |
| hCoV-19/Egypt/PHARCO-ARMY-30/2021 | EPI_ISL_1936349 | 12/02/2021 | Africa / Egypt / Cairo | Human | unknown | unknown | 25-55 | Hospitalized | unknown | unknown | C.36 | GR |
| hCoV-19/Egypt/PHARCO-ARMY-28/2021 | EPI_ISL_1936347 | 12/02/2021 | Africa / Egypt / Cairo | Human | unknown | unknown | 25-55 | Hospitalized | unknown | unknown | C.36 | GR |
| hCoV-19/Egypt/PHARCO-ARMY-26/2021 | EPI_ISL_1936345 | 12/02/2021 | Africa / Egypt / Cairo | Human | unknown | unknown | 25-55 | Hospitalized | unknown | unknown | C.36 | GR |
| hCoV-19/Egypt/PHARCO-ARMY-23/2021 | EPI_ISL_1936342 | 12/02/2021 | Africa / Egypt / Cairo | Human | unknown | unknown | 25-55 | Hospitalized | unknown | unknown | C.36 | GR |
| hCoV-19/Egypt/PHARCO-ARMY-21/2021 | EPI_ISL_1936340 | 12/02/2021 | Africa / Egypt / Cairo | Human | unknown | unknown | 25-55 | Hospitalized | unknown | unknown | B.1.1 | G |
| hCoV-19/Egypt/PHARCO-ARMY/2021 | EPI_ISL_1936339 | 12/02/2021 | Africa / Egypt / Cairo | Human | unknown | unknown | 25-55 | Hospitalized | unknown | unknown | C.36 | GR |
| hCoV-19/Egypt/PHARCO-ARMY-78/2021 | EPI_ISL_1936338 | 12/02/2021 | Africa / Egypt / Cairo | Human | unknown | unknown | 25-55 | Hospitalized | unknown | unknown | C.36 | GR |
| hCoV-19/Egypt/PHARCO-ARMY-77/2021 | EPI_ISL_1936337 | 12/02/2021 | Africa / Egypt / Cairo | Human | unknown | unknown | 25-55 | Hospitalized | unknown | unknown | C.36 | GR |
| hCoV-19/Egypt/PHARCO-ARMY-70/2021 | EPI_ISL_1936330 | 12/02/2021 | Africa / Egypt / Cairo | Human | unknown | unknown | 25-55 | Hospitalized | unknown | unknown | C.36 | GR |
| hCoV-19/Egypt/PHARCO-ARMY-65/2021 | EPI_ISL_1936325 | 12/02/2021 | Africa / Egypt / Cairo | Human | unknown | unknown | 25-55 | Hospitalized | unknown | unknown | C.36 | GR |
| hCoV-19/Egypt/PHARCO-ARMY-64/2021 | EPI_ISL_1936324 | 12/02/2021 | Africa / Egypt / Cairo | Human | unknown | unknown | 25-55 | Hospitalized | unknown | unknown | C.36 | GR |
| hCoV-19/Egypt/PHARCO-ARMY-63/2021 | EPI_ISL_1936323 | 12/02/2021 | Africa / Egypt / Cairo | Human | unknown | unknown | 25-55 | Hospitalized | unknown | unknown | C.36 | GR |
| hCoV-19/Egypt/PHARCO-ARMY-62/2021 | EPI_ISL_1936322 | 12/02/2021 | Africa / Egypt / Cairo | Human | unknown | unknown | 25-55 | Hospitalized | unknown | unknown | C.36 | GR |
| hCoV-19/Egypt/PHARCO-ARMY-61/2021 | EPI_ISL_1936321 | 12/02/2021 | Africa / Egypt / Cairo | Human | unknown | unknown | 25-55 | Hospitalized | unknown | unknown | C.36 | GR |
| hCoV-19/Egypt/PHARCO-ARMY-59/2021 | EPI_ISL_1936319 | 12/02/2021 | Africa / Egypt / Cairo | Human | unknown | unknown | 25-55 | Hospitalized | unknown | unknown | C.36 | GR |
| hCoV-19/Egypt/PHARCO-ARMY-58/2021 | EPI_ISL_1936318 | 12/02/2021 | Africa / Egypt / Cairo | Human | unknown | unknown | 25-55 | Hospitalized | unknown | unknown | B.1 | G |
| hCoV-19/Egypt/PHARCO-ARMY-56/2021 | EPI_ISL_1936316 | 12/02/2021 | Africa / Egypt / Cairo | Human | unknown | unknown | 25-55 | Hospitalized | unknown | unknown | C.36 | GR |
| hCoV-19/Egypt/PHARCO-ARMY-53/2021 | EPI_ISL_1936313 | 12/02/2021 | Africa / Egypt / Cairo | Human | unknown | unknown | 25-55 | Hospitalized | unknown | unknown | C.36 | GR |
| hCoV-19/Egypt/PHARCO-ARMY-46/2021 | EPI_ISL_1936306 | 12/02/2021 | Africa / Egypt / Cairo | Human | unknown | unknown | 25-55 | Hospitalized | unknown | unknown | C.36 | GR |
| hCoV-19/Egypt/PHARCO-ARMY-45/2021 | EPI_ISL_1936305 | 12/02/2021 | Africa / Egypt / Cairo | Human | unknown | unknown | 25-55 | Hospitalized | unknown | unknown | C.36 | GR |
| hCoV-19/Egypt/PHARCO-ARMY-44/2021 | EPI_ISL_1936304 | 12/02/2021 | Africa / Egypt / Cairo | Human | unknown | unknown | 25-55 | Hospitalized | unknown | unknown | C.36 | GR |
| hCoV-19/Egypt/PHARCO-ARMY-43/2021 | EPI_ISL_1936303 | 12/02/2021 | Africa / Egypt / Cairo | Human | unknown | unknown | 25-55 | Hospitalized | unknown | unknown | C.36 | GR |
| hCoV-19/Egypt/PHARCO-ARMY-41/2021 | EPI_ISL_1936301 | 12/02/2021 | Africa / Egypt / Cairo | Human | unknown | unknown | 25-55 | Hospitalized | unknown | unknown | C.36 | GR |
| hCoV-19/Egypt/PHARCO-ARMY-39/2021 | EPI_ISL_1936299 | 12/02/2021 | Africa / Egypt / Cairo | Human | unknown | unknown | 25-55 | Hospitalized | unknown | unknown | C.36 | GR |
| hCoV-19/Egypt/PHARCO-ARMY-36/2021 | EPI_ISL_1936296 | 12/02/2021 | Africa / Egypt / Cairo | Human | unknown | unknown | 25-55 | Hospitalized | unknown | unknown | C.36 | GR |
| hCoV-19/Egypt/ARMY-364/2021 | EPI_ISL_1936294 | 04/04/2021 | Africa / Egypt / Cairo | Human | unknown | unknown | 25-60 | Hospitalized | unknown | unknown | C.17 | GR |
| hCoV-19/Egypt/ARMY-362/2021 | EPI_ISL_1936292 | 04/04/2021 | Africa / Egypt / Cairo | Human | unknown | unknown | 25-60 | Hospitalized | unknown | unknown | C.17 | GR |
| hCoV-19/Egypt/ARMY-361/2021 | EPI_ISL_1936291 | 04/04/2021 | Africa / Egypt / Cairo | Human | unknown | unknown | 25-60 | Hospitalized | unknown | unknown | C.17 | GR |
| hCoV-19/Egypt/ARMY-358/2021 | EPI_ISL_1936288 | 04/04/2021 | Africa / Egypt / Cairo | Human | unknown | unknown | 25-60 | Hospitalized | unknown | unknown | B.1.170 | GH |
| hCoV-19/Egypt/ARMY-354/2021 | EPI_ISL_1936284 | 04/04/2021 | Africa / Egypt / Cairo | Human | unknown | unknown | 25-60 | Hospitalized | unknown | unknown | B.1.1.7 | GR |
| hCoV-19/Egypt/ARMY-349/2021 | EPI_ISL_1936279 | 04/04/2021 | Africa / Egypt / Cairo | Human | unknown | unknown | 25-60 | Hospitalized | unknown | unknown | A | S |
| hCoV-19/Egypt/ARMY-348/2021 | EPI_ISL_1936278 | 04/04/2021 | Africa / Egypt / Cairo | Human | unknown | unknown | 25-60 | Hospitalized | unknown | unknown | A | S |
| hCoV-19/Egypt/ARMY-347/2021 | EPI_ISL_1936277 | 04/04/2021 | Africa / Egypt / Cairo | Human | unknown | unknown | 25-60 | Hospitalized | unknown | unknown | A | S |
| hCoV-19/Egypt/ARMY-335/2021 | EPI_ISL_1936272 | 22/04/2021 | Africa / Egypt / Cairo | Human | unknown | unknown | 25-60 | Hospitalized | unknown | unknown | C.36.3 | GR |
| hCoV-19/Egypt/ARMY-334/2021 | EPI_ISL_1936271 | 22/04/2021 | Africa / Egypt / Cairo | Human | unknown | unknown | 25-60 | Hospitalized | unknown | unknown | C.36.3 | GR |
| hCoV-19/Egypt/ARMY-332/2021 | EPI_ISL_1936269 | 22/04/2021 | Africa / Egypt / Cairo | Human | unknown | unknown | 25-60 | Hospitalized | unknown | unknown | C.36.3 | GR |
| hCoV-19/Egypt/ARMY-331/2021 | EPI_ISL_1936268 | 22/04/2021 | Africa / Egypt / Cairo | Human | unknown | unknown | 25-60 | Hospitalized | unknown | unknown | C.36.3 | GR |
| hCoV-19/Egypt/ARMY-330/2021 | EPI_ISL_1936267 | 22/04/2021 | Africa / Egypt / Cairo | Human | unknown | unknown | 25-60 | Hospitalized | unknown | unknown | C.36.3 | GR |
| hCoV-19/Egypt/ARMY-328/2021 | EPI_ISL_1936265 | 22/04/2021 | Africa / Egypt / Cairo | Human | unknown | unknown | 25-60 | Hospitalized | unknown | unknown | C.38 | GR |
| hCoV-19/Egypt/ARMY-327/2021 | EPI_ISL_1936264 | 22/04/2021 | Africa / Egypt / Cairo | Human | unknown | unknown | 25-60 | Hospitalized | unknown | unknown | C.38 | GR |
| hCoV-19/Egypt/ARMY-326/2021 | EPI_ISL_1936263 | 22/04/2021 | Africa / Egypt / Cairo | Human | unknown | unknown | 25-60 | Hospitalized | unknown | unknown | C.38 | GR |
| hCoV-19/Egypt/ARMY-325/2021 | EPI_ISL_1936262 | 22/04/2021 | Africa / Egypt / Cairo | Human | unknown | unknown | 25-60 | Hospitalized | unknown | unknown | C.38 | GR |
| hCoV-19/Egypt/ARMY-324/2021 | EPI_ISL_1936261 | 22/04/2021 | Africa / Egypt / Cairo | Human | unknown | unknown | 25-60 | Hospitalized | unknown | unknown | C.38 | GR |
| hCoV-19/Egypt/ARMY-321/2021 | EPI_ISL_1936260 | 22/04/2021 | Africa / Egypt / Cairo | Human | unknown | unknown | 25-60 | Hospitalized | unknown | unknown | C.17 | G |
| hCoV-19/Egypt/ARMY-317/2021 | EPI_ISL_1936256 | 22/04/2021 | Africa / Egypt / Cairo | Human | unknown | unknown | 25-60 | Hospitalized | unknown | unknown | C.17 | GR |
| hCoV-19/Egypt/ARMY-314/2021 | EPI_ISL_1936253 | 22/04/2021 | Africa / Egypt / Cairo | Human | unknown | unknown | 25-60 | Hospitalized | unknown | unknown | B.1.1 | GR |
| hCoV-19/Egypt/ARMY-310/2021 | EPI_ISL_1936249 | 22/04/2021 | Africa / Egypt / Cairo | Human | unknown | unknown | 25-60 | Hospitalized | unknown | unknown | A | S |
| hCoV-19/Egypt/ARMY-309/2021 | EPI_ISL_1936248 | 22/04/2021 | Africa / Egypt / Cairo | Human | unknown | unknown | 25-60 | Hospitalized | unknown | unknown | C.38 | GR |
| hCoV-19/Egypt/ARMY-307/2021 | EPI_ISL_1936246 | 22/04/2021 | Africa / Egypt / Cairo | Human | unknown | unknown | 25-60 | Hospitalized | unknown | unknown | C.36.3 | GR |
| hCoV-19/Egypt/ARMY-306/2021 | EPI_ISL_1936245 | 22/04/2021 | Africa / Egypt / Cairo | Human | unknown | unknown | 25-60 | Hospitalized | unknown | unknown | C.36.3 | GR |
| hCoV-19/Egypt/ARMY-303/2021 | EPI_ISL_1936242 | 22/04/2021 | Africa / Egypt / Cairo | Human | unknown | unknown | 25-60 | Hospitalized | unknown | unknown | C.36.3 | GR |
| hCoV-19/Egypt/ARMY-300/2021 | EPI_ISL_1936240 | 22/04/2021 | Africa / Egypt / Cairo | Human | unknown | unknown | 25-60 | Hospitalized | unknown | unknown | C.36.3 | GR |
| hCoV-19/Egypt/ARMY-61/2021 | EPI_ISL_1936237 | 27/03/2021 | Africa / Egypt / Cairo | Human | unknown | unknown | 25-55 | Hospitalized | unknown | unknown | C.17 | GR |
| hCoV-19/Egypt/ARMY-58/2021 | EPI_ISL_1936234 | 27/03/2021 | Africa / Egypt / Cairo | Human | unknown | unknown | 25-55 | Hospitalized | unknown | unknown | B.1 | GH |
| hCoV-19/Egypt/ARMY-56/2021 | EPI_ISL_1936232 | 27/03/2021 | Africa / Egypt / Cairo | Human | unknown | unknown | 25-55 | Hospitalized | unknown | unknown | C.17 | GR |
| hCoV-19/Egypt/ARMY-53/2021 | EPI_ISL_1936229 | 27/03/2021 | Africa / Egypt / Cairo | Human | unknown | unknown | 25-55 | Hospitalized | unknown | unknown | C.17 | GR |
| hCoV-19/Egypt/ARMY-52/2021 | EPI_ISL_1936228 | 27/03/2021 | Africa / Egypt / Cairo | Human | unknown | unknown | 25-55 | Hospitalized | unknown | unknown | C.17 | GR |
| hCoV-19/Egypt/ARMY-50/2021 | EPI_ISL_1936226 | 27/03/2021 | Africa / Egypt / Cairo | Human | unknown | unknown | 25-55 | Hospitalized | unknown | unknown | C.17 | GR |
| hCoV-19/Egypt/ARMY-46/2021 | EPI_ISL_1936222 | 27/03/2021 | Africa / Egypt / Cairo | Human | unknown | unknown | 25-55 | Hospitalized | unknown | unknown | A | S |
| hCoV-19/Egypt/ARMY-44/2021 | EPI_ISL_1936219 | 27/03/2021 | Africa / Egypt / Cairo | Human | unknown | unknown | 25-55 | Hospitalized | unknown | unknown | C.36.3 | GR |
| hCoV-19/Egypt/ARMY-43/2021 | EPI_ISL_1936218 | 27/03/2021 | Africa / Egypt / Cairo | Human | unknown | unknown | 25-55 | Hospitalized | unknown | unknown | C.17 | GR |
| hCoV-19/Egypt/ARMY-41/2021 | EPI_ISL_1936216 | 27/03/2021 | Africa / Egypt / Cairo | Human | unknown | unknown | 25-55 | Hospitalized | unknown | unknown | C.17 | GR |
| hCoV-19/Egypt/ARMY-39/2021 | EPI_ISL_1936214 | 27/03/2021 | Africa / Egypt / Cairo | Human | unknown | unknown | 25-55 | Hospitalized | unknown | unknown | C.17 | GR |
| hCoV-19/Egypt/ARMY-38/2021 | EPI_ISL_1936213 | 27/03/2021 | Africa / Egypt / Cairo | Human | unknown | unknown | 25-55 | Hospitalized | unknown | unknown | C.17 | GR |
| hCoV-19/Egypt/ARMY-37/2021 | EPI_ISL_1936212 | 27/03/2021 | Africa / Egypt / Cairo | Human | unknown | unknown | 25-55 | Hospitalized | unknown | unknown | C.17 | GR |
| hCoV-19/Egypt/ARMY-36/2021 | EPI_ISL_1936211 | 27/03/2021 | Africa / Egypt / Cairo | Human | unknown | unknown | 25-55 | Hospitalized | unknown | unknown | C.17 | GR |
| hCoV-19/Egypt/ARMY-63/2021 | EPI_ISL_1936209 | 27/03/2021 | Africa / Egypt / Cairo | Human | unknown | unknown | 25-55 | Hospitalized | unknown | unknown | C.17 | GR |
| hCoV-19/Egypt/ARMY-34/2021 | EPI_ISL_1936208 | 27/03/2021 | Africa / Egypt / Cairo | Human | unknown | unknown | 25-55 | Hospitalized | unknown | unknown | C.17 | GR |
| hCoV-19/Egypt/ARMY-29/2021 | EPI_ISL_1936203 | 27/03/2021 | Africa / Egypt / Cairo | Human | unknown | unknown | 25-55 | Hospitalized | unknown | unknown | B.1.1.7 | GR |
| hCoV-19/Egypt/ARMY-27/2021 | EPI_ISL_1936201 | 27/03/2021 | Africa / Egypt / Cairo | Human | unknown | unknown | 25-55 | Hospitalized | unknown | unknown | B.1.1.7 | GR |
| hCoV-19/Egypt/ARMY-25/2021 | EPI_ISL_1936199 | 27/03/2021 | Africa / Egypt / Cairo | Human | unknown | unknown | 25-55 | Hospitalized | unknown | unknown | B.1.170 | GH |
| hCoV-19/Egypt/ARMY-22/2021 | EPI_ISL_1936196 | 27/03/2021 | Africa / Egypt / Cairo | Human | unknown | unknown | 25-55 | Hospitalized | unknown | unknown | C.38 | GR |
| hCoV-19/Egypt/ARMY-21/2021 | EPI_ISL_1936195 | 27/03/2021 | Africa / Egypt / Cairo | Human | unknown | unknown | 25-55 | Hospitalized | unknown | unknown | C.17 | GR |
| hCoV-19/Egypt/ARMY-20/2021 | EPI_ISL_1936194 | 27/03/2021 | Africa / Egypt / Cairo | Human | unknown | unknown | 25-55 | Hospitalized | unknown | unknown | C.17 | GR |
| hCoV-19/Egypt/ARMY-218/2021 | EPI_ISL_1936145 | 02/05/2021 | Africa / Egypt / Cairo | Human | unknown | unknown | 25-55 | Hospitalized | unknown | unknown | C.17 | GR |
| hCoV-19/Egypt/ARMY-212/2021 | EPI_ISL_1936141 | 02/05/2021 | Africa / Egypt / Cairo | Human | unknown | unknown | 25-55 | Hospitalized | unknown | unknown | B.1 | GH |
| hCoV-19/Egypt/ARMY-211/2021 | EPI_ISL_1936140 | 02/05/2021 | Africa / Egypt / Cairo | Human | unknown | unknown | 25-55 | Hospitalized | unknown | unknown | C.17 | GR |
| hCoV-19/Egypt/ARMY-205/2021 | EPI_ISL_1936138 | 02/05/2021 | Africa / Egypt / Cairo | Human | unknown | unknown | 25-55 | Hospitalized | unknown | unknown | C.17 | GR |
| hCoV-19/Egypt/ARMY-297/2021 | EPI_ISL_1936135 | 02/05/2021 | Africa / Egypt / Cairo | Human | unknown | unknown | 25-55 | Hospitalized | unknown | unknown | C.36.3 | GR |
| hCoV-19/Egypt/ARMY-274/2021 | EPI_ISL_1936129 | 02/05/2021 | Africa / Egypt / Cairo | Human | unknown | unknown | 25-55 | Hospitalized | unknown | unknown | A | S |
| hCoV-19/Egypt/ARMY-273/2021 | EPI_ISL_1936128 | 02/05/2021 | Africa / Egypt / Cairo | Human | unknown | unknown | 25-55 | Hospitalized | unknown | unknown | A | S |
| hCoV-19/Egypt/ARMY-266/2021 | EPI_ISL_1936126 | 02/05/2021 | Africa / Egypt / Cairo | Human | unknown | unknown | 25-55 | Hospitalized | unknown | unknown | C.17 | GR |
| hCoV-19/Egypt/ARMY-265/2021 | EPI_ISL_1936125 | 02/05/2021 | Africa / Egypt / Cairo | Human | unknown | unknown | 25-55 | Hospitalized | unknown | unknown | C.17 | GR |
| hCoV-19/Egypt/ARMY-264/2021 | EPI_ISL_1936124 | 02/05/2021 | Africa / Egypt / Cairo | Human | unknown | unknown | 25-55 | Hospitalized | unknown | unknown | C.17 | GR |
| hCoV-19/Egypt/ARMY-262/2021 | EPI_ISL_1936123 | 02/05/2021 | Africa / Egypt / Cairo | Human | unknown | unknown | 25-55 | Hospitalized | unknown | unknown | C.17 | GR |
| hCoV-19/Egypt/ARMY-258/2021 | EPI_ISL_1936120 | 02/05/2021 | Africa / Egypt / Cairo | Human | unknown | unknown | 25-55 | Hospitalized | unknown | unknown | C.17 | GR |
| hCoV-19/Egypt/ARMY-256/2021 | EPI_ISL_1936118 | 02/05/2021 | Africa / Egypt / Cairo | Human | unknown | unknown | 25-55 | Hospitalized | unknown | unknown | C.17 | GR |
| hCoV-19/Egypt/ARMY-290/2021 | EPI_ISL_1936114 | 02/05/2021 | Africa / Egypt / Cairo | Human | unknown | unknown | 25-55 | Hospitalized | unknown | unknown | C.17 | GR |
| hCoV-19/Egypt/ARMY-251/2021 | EPI_ISL_1936113 | 02/05/2021 | Africa / Egypt / Cairo | Human | unknown | unknown | 25-55 | Hospitalized | unknown | unknown | C.17 | GR |
| hCoV-19/Egypt/ARMY-259/2021 | EPI_ISL_1936111 | 02/05/2021 | Africa / Egypt / Cairo | Human | unknown | unknown | 25-55 | Hospitalized | unknown | unknown | B.1.1.7 | GR |
| hCoV-19/Egypt/ARMY-249/2021 | EPI_ISL_1936110 | 02/05/2021 | Africa / Egypt / Cairo | Human | unknown | unknown | 25-55 | Hospitalized | unknown | unknown | B.1.1.7 | GR |
| hCoV-19/Egypt/ARMY-206/2021 | EPI_ISL_1936109 | 02/05/2021 | Africa / Egypt / Cairo | Human | unknown | unknown | 25-55 | Hospitalized | unknown | unknown | B.1.1.7 | GR |
| hCoV-19/Egypt/ARMY-213/2021 | EPI_ISL_1936104 | 02/05/2021 | Africa / Egypt / Cairo | Human | unknown | unknown | 25-55 | Hospitalized | unknown | unknown | C.38 | GR |
| hCoV-19/Egypt/ARMY-210/2021 | EPI_ISL_1936103 | 02/05/2021 | Africa / Egypt / Cairo | Human | unknown | unknown | 25-55 | Hospitalized | unknown | unknown | C.17 | GR |
| hCoV-19/Egypt/ARMY-208/2021 | EPI_ISL_1936102 | 02/05/2021 | Africa / Egypt / Cairo | Human | unknown | unknown | 25-55 | Hospitalized | unknown | unknown | C.17 | GR |
| hCoV-19/Egypt/NRC-6572/2020 | EPI_ISL_2380098 | 30/08/2020 | Africa / Egypt | Human | unknown | unknown | unknown | unknown | unknown | unknown | B.1.1 | GR |
| hCoV-19/Egypt/NRC-6599/2020 | EPI_ISL_2380097 | 03/09/2020 | Africa / Egypt | Human | unknown | unknown | unknown | unknown | unknown | unknown | B.1 | G |
| hCoV-19/Egypt/NRC-6637/2020 | EPI_ISL_2380096 | 10/09/2020 | Africa / Egypt | Human | unknown | unknown | unknown | unknown | unknown | unknown | B.1 | G |
| hCoV-19/Egypt/NRC-6503/2020 | EPI_ISL_2380095 | 20/08/2020 | Africa / Egypt | Human | unknown | Male | unknown | unknown | unknown | unknown | C.17 | GR |
| hCoV-19/Egypt/NRC-6639/2020 | EPI_ISL_2380094 | 10/09/2020 | Africa / Egypt | Human | unknown | unknown | unknown | unknown | unknown | unknown | B.1 | GH |
| hCoV-19/Egypt/NRC-6496/2020 | EPI_ISL_2380093 | 20/08/2020 | Africa / Egypt | Human | unknown | unknown | unknown | unknown | unknown | unknown | C.17 | GR |
| hCoV-19/Egypt/NRC-6478/2020 | EPI_ISL_2380092 | 20/08/2020 | Africa / Egypt | Human | unknown | Female | unknown | unknown | unknown | unknown | C.17 | G |
| hCoV-19/Egypt/NRC-5883/2020 | EPI_ISL_2380091 | 17/06/2020 | Africa / Egypt | Human | unknown | unknown | unknown | unknown | unknown | unknown | B.1.170 | GH |
| hCoV-19/Egypt/NRC-6792/2020 | EPI_ISL_2380090 | 23/09/2020 | Africa / Egypt | Human | unknown | unknown | unknown | unknown | unknown | unknown | B.1 | GH |
| hCoV-19/Egypt/NRC-6743/2020 | EPI_ISL_2380089 | 18/09/2020 | Africa / Egypt | Human | unknown | unknown | unknown | unknown | unknown | unknown | B.1 | G |
| hCoV-19/Egypt/NRC-6030/2020 | EPI_ISL_2380088 | 23/06/2020 | Africa / Egypt | Human | unknown | unknown | unknown | unknown | unknown | unknown | B.1.170 | GH |
| hCoV-19/Egypt/NRC-6596/2020 | EPI_ISL_2380087 | 03/09/2020 | Africa / Egypt | Human | unknown | unknown | unknown | unknown | unknown | unknown | B.1 | GH |
| hCoV-19/Egypt/NRC-5972/2020 | EPI_ISL_2380086 | 23/06/2020 | Africa / Egypt | Human | unknown | unknown | unknown | unknown | unknown | unknown | B.1.170 | GH |
| hCoV-19/Egypt/NRC-6082/2020 | EPI_ISL_2380085 | 28/06/2020 | Africa / Egypt | Human | unknown | Male | unknown | unknown | unknown | unknown | B.1 | GH |
| hCoV-19/Egypt/NRC-6513/2020 | EPI_ISL_2380084 | 23/08/2020 | Africa / Egypt | Human | unknown | unknown | unknown | unknown | unknown | unknown | C.17 | GR |
| hCoV-19/Egypt/NRC-6378/2020 | EPI_ISL_2380083 | 28/07/2020 | Africa / Egypt | Human | unknown | Male | unknown | unknown | unknown | unknown | B.1.170 | GH |
| hCoV-19/Egypt/NRC-6102/2020 | EPI_ISL_2380081 | 28/06/2020 | Africa / Egypt | Human | unknown | Female | unknown | unknown | unknown | unknown | B.1.1.1 | GR |
| hCoV-19/Egypt/NRC-6766/2020 | EPI_ISL_2380080 | 20/09/2020 | Africa / Egypt | Human | unknown | unknown | unknown | unknown | unknown | unknown | B.1 | G |
| hCoV-19/Egypt/NRC-6037/2020 | EPI_ISL_2380079 | 24/06/2020 | Africa / Egypt | Human | unknown | unknown | unknown | unknown | unknown | unknown | B.1.195 | G |
| hCoV-19/Egypt/NRC-6761/2020 | EPI_ISL_2380078 | 20/09/2020 | Africa / Egypt | Human | unknown | unknown | unknown | unknown | unknown | unknown | B.1 | GH |
| hCoV-19/Egypt/NRC-6773/2020 | EPI_ISL_2380076 | 23/09/2020 | Africa / Egypt | Human | unknown | unknown | unknown | unknown | unknown | unknown | B.1 | G |
| hCoV-19/Egypt/NRC-6050/2020 | EPI_ISL_2380075 | 24/06/2020 | Africa / Egypt | Human | unknown | unknown | unknown | unknown | unknown | unknown | B.1 | GH |
| hCoV-19/Egypt/NRC-6775/2020 | EPI_ISL_2380074 | 23/09/2020 | Africa / Egypt | Human | unknown | unknown | unknown | unknown | unknown | unknown | B.1 | GH |
| hCoV-19/Egypt/NRC-5868/2020 | EPI_ISL_2380073 | 16/06/2020 | Africa / Egypt | Human | unknown | unknown | unknown | unknown | unknown | unknown | B.1.1 | GR |
| hCoV-19/Egypt/NRC-6751/2020 | EPI_ISL_2380072 | 20/09/2020 | Africa / Egypt | Human | unknown | unknown | unknown | unknown | unknown | unknown | B.1 | GH |
| hCoV-19/Egypt/NRC-6781/2020 | EPI_ISL_2380071 | 23/09/2020 | Africa / Egypt | Human | unknown | unknown | unknown | unknown | unknown | unknown | B.1 | G |
| hCoV-19/Egypt/NRC-6746/2020 | EPI_ISL_2380070 | 18/09/2020 | Africa / Egypt | Human | unknown | unknown | unknown | unknown | unknown | unknown | B.1 | GH |
| hCoV-19/Egypt/NRC-6581/2020 | EPI_ISL_2380069 | 31/08/2020 | Africa / Egypt | Human | unknown | unknown | unknown | unknown | unknown | unknown | B.1 | GH |
| hCoV-19/Egypt/NRC-6183/2020 | EPI_ISL_2380068 | 30/06/2020 | Africa / Egypt | Human | unknown | Male | unknown | unknown | unknown | unknown | B.1 | GH |
| hCoV-19/Egypt/NRC-6184/2020 | EPI_ISL_2380067 | 01/07/2020 | Africa / Egypt | Human | unknown | Male | unknown | unknown | unknown | unknown | B.1 | GH |
| hCoV-19/Egypt/NRC-6649/2020 | EPI_ISL_2380066 | 10/09/2020 | Africa / Egypt | Human | unknown | Male | unknown | unknown | unknown | unknown | B.1 | G |
| hCoV-19/Egypt/NRC-6840/2020 | EPI_ISL_2380065 | 04/10/2020 | Africa / Egypt | Human | unknown | unknown | unknown | unknown | unknown | unknown | B.1 | GH |
| hCoV-19/Egypt/NRC-6165/2020 | EPI_ISL_2380064 | 01/07/2020 | Africa / Egypt | Human | unknown | unknown | unknown | unknown | unknown | unknown | B.1.1.1 | GR |
| hCoV-19/Egypt/NRC-6185/2020 | EPI_ISL_2380063 | 01/07/2020 | Africa / Egypt | Human | unknown | Male | unknown | unknown | unknown | unknown | B.1 | GH |
| hCoV-19/Egypt/NRC-6651/2020 | EPI_ISL_2380062 | 10/09/2020 | Africa / Egypt | Human | unknown | unknown | unknown | unknown | unknown | unknown | B.1 | GH |
| hCoV-19/Egypt/NRC-6663/2020 | EPI_ISL_2380061 | 10/09/2020 | Africa / Egypt | Human | unknown | unknown | unknown | unknown | unknown | unknown | B.1.35 | G |
| hCoV-19/Egypt/NRC-6848/2020 | EPI_ISL_2380060 | 05/10/2020 | Africa / Egypt | Human | unknown | unknown | unknown | unknown | unknown | unknown | B.1 | GH |
| hCoV-19/Egypt/NRC-6170/2020 | EPI_ISL_2380059 | 01/07/2020 | Africa / Egypt | Human | unknown | unknown | unknown | unknown | unknown | unknown | B.1 | GH |
| hCoV-19/Egypt/NRC-6849/2020 | EPI_ISL_2380058 | 05/10/2020 | Africa / Egypt | Human | unknown | unknown | unknown | unknown | unknown | unknown | B.1 | GH |
| hCoV-19/Egypt/NRC-6634/2020 | EPI_ISL_2380057 | 09/09/2020 | Africa / Egypt | Human | unknown | Male | unknown | unknown | unknown | unknown | C.17 | GR |
| hCoV-19/Egypt/NRC-6176/2020 | EPI_ISL_2380056 | 30/06/2020 | Africa / Egypt | Human | unknown | unknown | unknown | unknown | unknown | unknown | B.1 | GH |
| hCoV-19/Egypt/NRC-6853/2020 | EPI_ISL_2380055 | 06/10/2020 | Africa / Egypt | Human | unknown | unknown | unknown | unknown | unknown | unknown | B.1 | GH |
| hCoV-19/Egypt/NRC-6631/2020 | EPI_ISL_2380053 | 09/09/2020 | Africa / Egypt | Human | unknown | unknown | unknown | unknown | unknown | unknown | C.17 | GR |
| hCoV-19/Egypt/NRC-6408/2020 | EPI_ISL_2380052 | 05/08/2020 | Africa / Egypt | Human | unknown | Female | unknown | unknown | unknown | unknown | C.17 | GR |
| hCoV-19/Egypt/NRC-6182/2020 | EPI_ISL_2380051 | 30/06/2020 | Africa / Egypt | Human | unknown | Female | unknown | unknown | unknown | unknown | B.1 | GH |
| hCoV-19/Egypt/NRC-5448NS/2020 | EPI_ISL_8193872 | 27/05/2020 | Africa / Egypt | Human | unknown | unknown | unknown | unknown | unknown | unknown | B.1.533 | GH |
| hCoV-19/Egypt/CPHL-NRC-S24/2021 | EPI_ISL_2313069 | 2021-03 | Africa / Egypt | Human | unknown | unknown | unknown | unknown | unknown | unknown | C.36.3 | GR |
| hCoV-19/Egypt/CPHL-NRC-S1/2021 | EPI_ISL_2313068 | 2021-03 | Africa / Egypt | Human | unknown | unknown | unknown | unknown | unknown | unknown | C.36.3 | GR |
| hCoV-19/Egypt/CPHL-NRC-S2/2021 | EPI_ISL_2313067 | 2021-03 | Africa / Egypt | Human | unknown | unknown | unknown | unknown | unknown | unknown | C.36.3 | GR |
| hCoV-19/Egypt/CPHL-NRC-S3/2021 | EPI_ISL_2313066 | 2021-03 | Africa / Egypt | Human | unknown | unknown | unknown | unknown | unknown | unknown | C.36.3 | GR |
| hCoV-19/Egypt/CPHL-NRC-S4/2021 | EPI_ISL_2313065 | 2021-03 | Africa / Egypt | Human | unknown | unknown | unknown | unknown | unknown | unknown | C.36.3 | GR |
| hCoV-19/Egypt/CPHL-NRC-S5/2021 | EPI_ISL_2313064 | 2021-03 | Africa / Egypt | Human | unknown | unknown | unknown | unknown | unknown | unknown | C.17 | GR |
| hCoV-19/Egypt/CPHL-NRC-S6/2021 | EPI_ISL_2313063 | 2021-03 | Africa / Egypt | Human | unknown | unknown | unknown | unknown | unknown | unknown | C.36.3 | GR |
| hCoV-19/Egypt/CPHL-NRC-S10/2021 | EPI_ISL_2313059 | 2021-03 | Africa / Egypt | Human | unknown | unknown | unknown | unknown | unknown | unknown | C.36.3 | GR |
| hCoV-19/Egypt/CPHL-NRC-S11/2021 | EPI_ISL_2313058 | 2021-03 | Africa / Egypt | Human | unknown | unknown | unknown | unknown | unknown | unknown | C.17 | GR |
| hCoV-19/Egypt/CPHL-NRC-S12/2021 | EPI_ISL_2313057 | 2021-03 | Africa / Egypt | Human | unknown | unknown | unknown | unknown | unknown | unknown | C.36.3 | GR |
| hCoV-19/Egypt/CPHL-NRC-S13/2021 | EPI_ISL_2313056 | 2021-03 | Africa / Egypt | Human | unknown | unknown | unknown | unknown | unknown | unknown | C.17 | GR |
| hCoV-19/Egypt/CPHL-NRC-S14/2021 | EPI_ISL_2313055 | 2021-03 | Africa / Egypt | Human | unknown | unknown | unknown | unknown | unknown | unknown | C.36.3 | GR |
| hCoV-19/Egypt/CPHL-NRC-S15/2021 | EPI_ISL_2313054 | 2021-03 | Africa / Egypt | Human | unknown | unknown | unknown | unknown | unknown | unknown | C.36.3 | GR |
| hCoV-19/Egypt/CPHL-NRC-S16/2021 | EPI_ISL_2313053 | 2021-03 | Africa / Egypt | Human | unknown | unknown | unknown | unknown | unknown | unknown | C.36.3 | GR |
| hCoV-19/Egypt/CPHL-NRC-S17/2021 | EPI_ISL_2313052 | 2021-03 | Africa / Egypt | Human | unknown | unknown | unknown | unknown | unknown | unknown | C.36.3 | GR |
| hCoV-19/Egypt/CPHL-NRC-S18/2021 | EPI_ISL_2313051 | 2021-03 | Africa / Egypt | Human | unknown | unknown | unknown | unknown | unknown | unknown | C.36.3 | GR |
| hCoV-19/Egypt/CPHL-NRC-S20/2021 | EPI_ISL_2313049 | 2021-03 | Africa / Egypt | Human | unknown | unknown | unknown | unknown | unknown | unknown | C.36.3 | GR |
| hCoV-19/Egypt/CPHL-NRC-S21/2021 | EPI_ISL_2313048 | 2021-03 | Africa / Egypt | Human | unknown | unknown | unknown | unknown | unknown | unknown | C.36.3 | GR |
| hCoV-19/Egypt/CPHL-NRC-S22/2021 | EPI_ISL_2313047 | 2021-03 | Africa / Egypt | Human | unknown | unknown | unknown | unknown | unknown | unknown | C.36.3 | GR |
| hCoV-19/Egypt/CPHL-NRC-S23/2021 | EPI_ISL_2313046 | 2021-03 | Africa / Egypt | Human | unknown | unknown | unknown | unknown | unknown | unknown | C.36.3 | GR |
| hCoV-19/Egypt/CPHL-A6/2021 | EPI_ISL_3274162 | 18/01/2021 | Africa / Egypt / Qulyiubia | Human | Sentinel surveillance (ILI) | Female | 44 | unknown | unknown | Sentinel surveillance (ILI) | B.1.466 | GH |
| hCoV-19/cat/Egypt/AHRI-3/2020 | EPI_ISL_2932462 | 2020-12 | Africa / Egypt | Human | unknown | unknown | unknown | unknown | unknown | unknown | Unassigned | O |
| hCoV-19/Egypt/CCHE57357_Wave_3_A068/2021 | EPI_ISL_2566510 | 14/05/2021 | Africa / Egypt | Human | unknown | unknown | unknown | unknown | unknown | unknown | C.36.3 | GR |
| hCoV-19/Egypt/CCHE57357_Wave_3_A_005/2021 | EPI_ISL_2510688 | 01/05/2021 | Africa / Egypt | Human | unknown | unknown | unknown | unknown | unknown | unknown | B.1.1.7 | GR |
| hCoV-19/Egypt/NRC-5799/2020 | EPI_ISL_2232323 | 15/06/2020 | Africa / Egypt | Human | unknown | Female | unknown | unknown | unknown | unknown | B.1 | G |
| hCoV-19/Egypt/CPHL-NRC-S9/2021 | EPI_ISL_2313060 | 2021-03 | Africa / Egypt | Human | unknown | unknown | unknown | unknown | unknown | unknown | C.36.3 | GR |
| hCoV-19/Egypt/ARMY-308/2021 | EPI_ISL_1936247 | 22/04/2021 | Africa / Egypt / Cairo | Human | unknown | unknown | 25-60 | Hospitalized | unknown | unknown | C.38 | GR |
| hCoV-19/Egypt/CPHL-NRC-16/2020 | EPI_ISL_794596 | 06/05/2020 | Africa / Egypt | Human | unknown | unknown | unknown | unknown | unknown | unknown | B.1 | GH |
| hCoV-19/Egypt/CPHL-NRC-21/2020 | EPI_ISL_794604 | 13/03/2020 | Africa / Egypt | Human | unknown | unknown | unknown | unknown | unknown | unknown | B.1 | GH |
| hCoV-19/Egypt/CCHE57357-A-10/2020 | EPI_ISL_812791 | 15/05/2020 | Africa / Egypt | Human | unknown | unknown | unknown | unknown | unknown | unknown | B.1 | G |
| hCoV-19/Egypt/CCHE57357-A-80/2020 | EPI_ISL_812844 | 02/08/2020 | Africa / Egypt | Human | unknown | unknown | unknown | unknown | unknown | unknown | B | G |
| hCoV-19/Egypt/CCHE57357-A-93/2020 | EPI_ISL_812849 | 16/07/2020 | Africa / Egypt | Human | unknown | unknown | unknown | unknown | unknown | unknown | B.1 | GH |
| hCoV-19/Egypt/CUNCI-HGC11I035/2021 | EPI_ISL_862804 | 03/01/2021 | Africa / Egypt | Human | unknown | unknown | unknown | unknown | unknown | unknown | B.1.170 | GH |
| hCoV-19/Egypt/CUNCI-HGC11I006/2021 | EPI_ISL_862788 | 03/01/2021 | Africa / Egypt | Human | unknown | unknown | unknown | unknown | unknown | unknown | B.1.466 | GH |
| hCoV-19/Egypt/CUNCI-HGC11I027/2021 | EPI_ISL_862797 | 03/01/2021 | Africa / Egypt | Human | unknown | unknown | unknown | unknown | unknown | unknown | B.1.466 | GH |
| hCoV-19/Egypt/CUNCI-HGC09I030/2020 | EPI_ISL_907095 | 25/12/2020 | Africa / Egypt | Human | unknown | unknown | unknown | unknown | unknown | unknown | C.36 | GR |
| hCoV-19/Egypt/NRC-6032/2020 | EPI_ISL_8193905 | 23/06/2020 | Africa / Egypt | Human | unknown | unknown | unknown | unknown | unknown | unknown | B.1.1.1 | GR |
| hCoV-19/Egypt/CUNCI-HGC12I049/2021 | EPI_ISL_1040916 | 03/01/2021 | Africa / Egypt | Human | unknown | unknown | unknown | unknown | unknown | unknown | A.28 | S |
| hCoV-19/Egypt/ARMY-24/2021 | EPI_ISL_1936198 | 27/03/2021 | Africa / Egypt / Cairo | Human | unknown | unknown | 25-55 | Hospitalized | unknown | unknown | B.1.170 | GH |
| hCoV-19/Egypt/PHARCO-ARMY-38/2021 | EPI_ISL_1936298 | 12/02/2021 | Africa / Egypt / Cairo | Human | unknown | unknown | 25-55 | Hospitalized | unknown | unknown | C.36 | GR |
| hCoV-19/Egypt/NRC-6888/2020 | EPI_ISL_2232330 | 14/10/2020 | Africa / Egypt | Human | unknown | Male | unknown | unknown | unknown | unknown | C.17 | GR |
| hCoV-19/Egypt/MASRI-C4-041/2020 | EPI_ISL_1109486 | 21/05/2020 | Africa / Egypt / Cairo | Human | unknown | Female | unknown | unknown | unknown | unknown | B.1.1.1 | GR |
| hCoV-19/Egypt/NRC1-2/2020 | EPI_ISL_1273103 | 01/05/2020 | Africa / Egypt | Human | unknown | unknown | unknown | unknown | unknown | unknown | B.1 | GH |
| hCoV-19/Egypt/NRC-748/2021 | EPI_ISL_8251508 | 01/06/2021 | Africa / Egypt | Human | unknown | unknown | unknown | unknown | unknown | unknown | C.36.3 | GR |
| hCoV-19/Egypt/NRC-6074/2020 | EPI_ISL_8193608 | 27/06/2020 | Africa / Egypt | Human | unknown | unknown | unknown | unknown | unknown | unknown | B.1.195 | G |
| hCoV-19/Egypt/CCHE57357_Wave_3_A051/2021 | EPI_ISL_2566500 | 23/03/2021 | Africa / Egypt | Human | unknown | unknown | unknown | unknown | unknown | unknown | C.36.3 | GR |
| hCoV-19/Egypt/CCHE57357_Wave_3_A055/2021 | EPI_ISL_2566517 | 10/03/2021 | Africa / Egypt | Human | unknown | unknown | unknown | unknown | unknown | unknown | C.36.3 | GR |
| hCoV-19/Egypt/CCHE57357_Wave_3_A_056/2021 | EPI_ISL_2566527 | 10/03/2021 | Africa / Egypt | Human | unknown | unknown | unknown | unknown | unknown | unknown | C.36.3 | GR |
| hCoV-19/Egypt/CCHE57357_Wave_3_A_052/2021 | EPI_ISL_2566526 | 04/03/2021 | Africa / Egypt | Human | unknown | unknown | unknown | unknown | unknown | unknown | B.1.1.7 | GR |
| hCoV-19/Egypt/CCHE57357_Wave_3_A_076/2021 | EPI_ISL_2566530 | 10/05/2021 | Africa / Egypt | Human | unknown | unknown | unknown | unknown | unknown | unknown | C.36.3 | GR |
| hCoV-19/Egypt/CCHE57357_Wave_3_A015/2021 | EPI_ISL_2566475 | 05/05/2021 | Africa / Egypt | Human | unknown | unknown | unknown | unknown | unknown | unknown | C.36.3 | GR |
| hCoV-19/Egypt/CCHE57357_Wave_3_A016/2021 | EPI_ISL_2566476 | 06/05/2021 | Africa / Egypt | Human | unknown | unknown | unknown | unknown | unknown | unknown | C.36.3 | O |
| hCoV-19/Egypt/CCHE57357_Wave_3_A012/2021 | EPI_ISL_2566473 | 17/05/2021 | Africa / Egypt | Human | unknown | unknown | unknown | unknown | unknown | unknown | C.36.3 | GR |
| hCoV-19/Egypt/CCHE57357_Wave_3_A007/2021 | EPI_ISL_2566472 | 05/03/2021 | Africa / Egypt | Human | unknown | unknown | unknown | unknown | unknown | unknown | C.17 | O |
| hCoV-19/Egypt/CCHE57357_Wave_3_A033/2021 | EPI_ISL_2566489 | 04/05/2021 | Africa / Egypt | Human | unknown | unknown | unknown | unknown | unknown | unknown | C.36.3 | GR |
| hCoV-19/Egypt/CCHE57357_Wave_3_A029/2021 | EPI_ISL_2566486 | 11/05/2021 | Africa / Egypt | Human | unknown | unknown | unknown | unknown | unknown | unknown | C.36.3 | GR |
| hCoV-19/Egypt/CCHE57357_Wave_3_A032/2021 | EPI_ISL_2566488 | 07/05/2021 | Africa / Egypt | Human | unknown | unknown | unknown | unknown | unknown | unknown | C.36.3 | GR |
| hCoV-19/Egypt/CCHE57357_Wave_3_A030/2021 | EPI_ISL_2566487 | 11/05/2021 | Africa / Egypt | Human | unknown | unknown | unknown | unknown | unknown | unknown | C.36.3 | GR |
| hCoV-19/Egypt/CCHE57357_Wave_3_A021/2021 | EPI_ISL_2566481 | 14/05/2021 | Africa / Egypt | Human | unknown | unknown | unknown | unknown | unknown | unknown | C.36.3 | GR |
| hCoV-19/Egypt/CCHE57357_Wave_3_A025/2021 | EPI_ISL_2566484 | 08/05/2021 | Africa / Egypt | Human | unknown | unknown | unknown | unknown | unknown | unknown | C.36.3 | GR |
| hCoV-19/Egypt/CCHE57357_Wave_3_A047/2021 | EPI_ISL_2566497 | 04/03/2021 | Africa / Egypt | Human | unknown | unknown | unknown | unknown | unknown | unknown | C.36.3 | GR |
| hCoV-19/Egypt/CCHE57357_Wave_3_A045/2021 | EPI_ISL_2566496 | 15/03/2021 | Africa / Egypt | Human | unknown | unknown | unknown | unknown | unknown | unknown | C.36.3 | O |
| hCoV-19/Egypt/CCHE57357_Wave_3_A050/2021 | EPI_ISL_2566499 | 01/03/2021 | Africa / Egypt | Human | unknown | unknown | unknown | unknown | unknown | unknown | C.36.3 | GR |
| hCoV-19/Egypt/CCHE57357_Wave_3_A039/2021 | EPI_ISL_2566492 | 07/03/2021 | Africa / Egypt | Human | unknown | unknown | unknown | unknown | unknown | unknown | C.36.3 | GR |
| hCoV-19/Egypt/CCHE57357_Wave_3_A036/2021 | EPI_ISL_2566490 | 12/05/2021 | Africa / Egypt | Human | unknown | unknown | unknown | unknown | unknown | unknown | C.36.3 | GR |
| hCoV-19/Egypt/NRC-784/2021 | EPI_ISL_8251509 | 10/06/2021 | Africa / Egypt | Human | unknown | unknown | unknown | unknown | unknown | unknown | C.36.3 | GH |
| hCoV-19/Egypt/NRC-6944/2020 | EPI_ISL_8193910 | 21/10/2020 | Africa / Egypt | Human | unknown | unknown | unknown | unknown | unknown | unknown | B.1.1.312 | GR |
| hCoV-19/Egypt/NRC-785/2021 | EPI_ISL_8251510 | 11/06/2021 | Africa / Egypt | Human | unknown | unknown | unknown | unknown | unknown | unknown | C.36.3 | G |
| hCoV-19/Egypt/NRC-1099/2021 | EPI_ISL_8215710 | 13/10/2021 | Africa / Egypt | Human | unknown | Male | unknown | unknown | unknown | unknown | AY.122 | GK |
| hCoV-19/Egypt/MASRI-C5-042/2020 | EPI_ISL_1167197 | 15/07/2020 | Africa / Egypt / Cairo | Human | unknown | Female | unknown | unknown | unknown | unknown | C.17 | GR |
| hCoV-19/Egypt/MASRI-C5-039/2020 | EPI_ISL_1167196 | 19/07/2020 | Africa / Egypt / Cairo | Human | unknown | Male | 56 | unknown | unknown | unknown | B.1.1.1 | G |
| hCoV-19/Egypt/MASRI-C5-027/2020 | EPI_ISL_1167195 | 24/06/2020 | Africa / Egypt / Cairo | Human | unknown | Female | unknown | unknown | unknown | unknown | B.1.1.1 | GH |
| hCoV-19/Egypt/MASRI-C5-026/2020 | EPI_ISL_1167194 | 24/06/2020 | Africa / Egypt / Cairo | Human | unknown | Female | unknown | unknown | unknown | unknown | B.1 | GH |
| hCoV-19/Egypt/MASRI-C5-021/2020 | EPI_ISL_1167193 | 04/07/2020 | Africa / Egypt / Cairo | Human | unknown | Male | 11 | unknown | unknown | unknown | C.17 | GR |
| hCoV-19/Egypt/MASRI-C5-020/2020 | EPI_ISL_1167192 | 13/09/2020 | Africa / Egypt / Cairo | Human | unknown | Female | 7 months | unknown | unknown | unknown | B.1 | GH |
| hCoV-19/Egypt/MASRI-C5-017/2020 | EPI_ISL_1167191 | 25/05/2020 | Africa / Egypt / Cairo | Human | unknown | Male | 11 | unknown | unknown | unknown | B.1.1.142 | O |
| hCoV-19/Egypt/MASRI-C5-009/2020 | EPI_ISL_1167189 | 29/06/2020 | Africa / Egypt / Cairo | Human | unknown | Female | 2 | unknown | unknown | unknown | B.1.1.1 | GR |
| hCoV-19/Egypt/MASRI-C5-007/2020 | EPI_ISL_1167188 | 01/07/2020 | Africa / Egypt / Cairo | Human | unknown | Female | 10 | unknown | unknown | unknown | B.1.542 | G |
| hCoV-19/Egypt/MASRI-C5-005/2020 | EPI_ISL_1167187 | 13/06/2020 | Africa / Egypt / Cairo | Human | unknown | Female | 3 | unknown | unknown | unknown | Unassigned | O |
| hCoV-19/Egypt/MASRI-C5-003/2020 | EPI_ISL_1167186 | 25/05/2020 | Africa / Egypt / Cairo | Human | unknown | Male | 1 | unknown | unknown | unknown | B.1.1 | G |
| hCoV-19/Egypt/ARMY-340/2021 | EPI_ISL_1936273 | 04/04/2021 | Africa / Egypt / Cairo | Human | unknown | unknown | 25-60 | Hospitalized | unknown | unknown | C.17 | GR |
| hCoV-19/Egypt/MASRI-C4-018/2020 | EPI_ISL_1165087 | 01/07/2020 | Africa / Egypt / Cairo | Human | unknown | Male | 0 | unknown | unknown | unknown | B.1 | GH |
| hCoV-19/Egypt/MASRI-C4-014/2020 | EPI_ISL_1165086 | 08/06/2020 | Africa / Egypt / Cairo | Human | unknown | Female | 0 | unknown | unknown | unknown | B.1 | GH |
| hCoV-19/Egypt/MASRI-C4-012/2020 | EPI_ISL_1165085 | 08/06/2020 | Africa / Egypt / Cairo | Human | unknown | Female | 6 | unknown | unknown | unknown | B.1 | GH |
| hCoV-19/Egypt/MASRI-C4-004/2020 | EPI_ISL_1165084 | 08/06/2020 | Africa / Egypt / Cairo | Human | unknown | Female | 0 | unknown | unknown | unknown | C.17 | G |
| hCoV-19/Egypt/MASRI-C4-023/2020 | EPI_ISL_1165083 | 06/05/2020 | Africa / Egypt / Cairo | Human | unknown | Female | 44 | Dead | unknown | unknown | B.1 | GH |
| hCoV-19/Egypt/MASRI-C4-022/2020 | EPI_ISL_1165082 | 01/05/2020 | Africa / Egypt / Cairo | Human | unknown | Female | 52 | Dead | unknown | unknown | B.1 | GH |
| hCoV-19/Egypt/MASRI-C4-038/2020 | EPI_ISL_1165081 | 17/11/2020 | Africa / Egypt / Cairo | Human | unknown | Female | 78 | unknown | unknown | unknown | C.17 | GR |
| hCoV-19/Egypt/MASRI-C4-037/2020 | EPI_ISL_1165080 | 17/11/2020 | Africa / Egypt / Cairo | Human | unknown | Female | 53 | unknown | unknown | unknown | C.17 | GR |
| hCoV-19/Egypt/MASRI-C4-033/2020 | EPI_ISL_1165079 | 25/11/2020 | Africa / Egypt / Cairo | Human | unknown | Female | 74 | unknown | unknown | unknown | C.17 | GR |
| hCoV-19/Egypt/MASRI-C4-029/2020 | EPI_ISL_1165078 | 28/06/2020 | Africa / Egypt / Cairo | Human | unknown | Female | unknown | unknown | unknown | unknown | C.17 | GR |
| hCoV-19/Egypt/NRC-5943/2020 | EPI_ISL_8193921 | 21/06/2020 | Africa / Egypt | Human | unknown | unknown | unknown | unknown | unknown | unknown | B.1 | GH |
| hCoV-19/Egypt/MASRI-C4-011/2020 | EPI_ISL_1141525 | 08/06/2020 | Africa / Egypt / Cairo | Human | unknown | Male | 0 | unknown | unknown | unknown | C.17 | GR |
| hCoV-19/Egypt/CCHE57357_Wave_3_A057/2021 | EPI_ISL_2566503 | 20/03/2021 | Africa / Egypt | Human | unknown | unknown | unknown | unknown | unknown | unknown | B.1.1.7 | GR |
| hCoV-19/Egypt/CCHE57357_Wave_3_A040/2021 | EPI_ISL_2566516 | 01/03/2021 | Africa / Egypt | Human | unknown | unknown | unknown | unknown | unknown | unknown | B.1.1.7 | GR |
| hCoV-19/Egypt/CCHE57357_Wave_3_A070/2021 | EPI_ISL_2566512 | 10/05/2021 | Africa / Egypt | Human | unknown | unknown | unknown | unknown | unknown | unknown | B.1.1.7 | GR |
| hCoV-19/Egypt/ARMY-215/2021 | EPI_ISL_1936142 | 02/05/2021 | Africa / Egypt / Cairo | Human | unknown | unknown | 25-55 | Hospitalized | unknown | unknown | B.1 | GH |
| hCoV-19/Egypt/NRC-802/2021 | EPI_ISL_8251512 | 15/06/2021 | Africa / Egypt | Human | unknown | unknown | unknown | unknown | unknown | unknown | C.38 | GR |
| hCoV-19/Egypt/MASRI-C5-019/2020 | EPI_ISL_1109630 | 06/06/2020 | Africa / Egypt / Cairo | Human | unknown | Male | 3 | unknown | unknown | unknown | C.17 | GR |
| hCoV-19/Egypt/MASRI-C5-002/2020 | EPI_ISL_1109629 | 06/06/2020 | Africa / Egypt / Cairo | Human | unknown | Male | 3 | unknown | unknown | unknown | B.1 | GH |
| hCoV-19/Egypt/MASRI-C5-013/2020 | EPI_ISL_1109628 | 08/06/2020 | Africa / Egypt / Cairo | Human | unknown | Male | 1 | unknown | unknown | unknown | B.1.36 | GH |
| hCoV-19/Egypt/MASRI-C5-015/2020 | EPI_ISL_1109627 | 13/06/2020 | Africa / Egypt / Cairo | Human | unknown | Male | 13 | unknown | unknown | unknown | B.1 | GH |
| hCoV-19/Egypt/MASRI-C5-016/2020 | EPI_ISL_1109625 | 01/07/2020 | Africa / Egypt / Cairo | Human | unknown | Male | 15 | unknown | unknown | unknown | C.17 | GR |
| hCoV-19/Egypt/MASRI-C4-036/2020 | EPI_ISL_1109485 | 17/11/2020 | Africa / Egypt / Cairo | Human | unknown | Male | 59 | unknown | unknown | unknown | B.1.367 | GH |
| hCoV-19/Egypt/MASRI-C4-035/2020 | EPI_ISL_1109484 | 19/11/2020 | Africa / Egypt / Cairo | Human | unknown | Male | 47 | unknown | unknown | unknown | C.17 | GR |
| hCoV-19/Egypt/EMC-2/2021 | EPI_ISL_2955336 | 16/04/2021 | Africa / Egypt | Human | unknown | unknown | unknown | unknown | unknown | unknown | B.1.1.7 | GRY |
| hCoV-19/Egypt/NRC-5455NS/2020 | EPI_ISL_8193871 | 27/05/2020 | Africa / Egypt | Human | unknown | unknown | unknown | unknown | unknown | unknown | B.1.170 | G |
| hCoV-19/Egypt/ARMY-257/2021 | EPI_ISL_1936119 | 02/05/2021 | Africa / Egypt / Cairo | Human | unknown | unknown | 25-55 | Hospitalized | unknown | unknown | C.17 | GR |
| hCoV-19/Egypt/NRC-1159/2021 | EPI_ISL_8205813 | 18/10/2021 | Africa / Egypt | Human | unknown | Female | unknown | unknown | unknown | unknown | B.1.617.2 | GK |
| hCoV-19/Egypt/MASRI-C4-025/2020 | EPI_ISL_1098839 | 10/07/2020 | Africa / Egypt / Cairo | Human | unknown | Female | 69 | Deceased | unknown | unknown | C.17 | GR |
| hCoV-19/Egypt/MASRI-018/2020 | EPI_ISL_1097029 | 01/06/2020 | Africa / Egypt / Cairo | Human | unknown | Male | 0 | Live | unknown | unknown | B.1 | GH |
| hCoV-19/Egypt/MASRI-014/2020 | EPI_ISL_1097028 | 08/06/2020 | Africa / Egypt / Cairo | Human | unknown | Female | 0 | Live | unknown | unknown | B.1 | GH |
| hCoV-19/Egypt/MASRI-012/2020 | EPI_ISL_1097027 | 08/06/2020 | Africa / Egypt / Cairo | Human | unknown | Female | 6 | Live | unknown | unknown | B.1 | GH |
| hCoV-19/Egypt/MASRI-011/2020 | EPI_ISL_1097026 | 08/06/2020 | Africa / Egypt / Cairo | Human | unknown | Male | 0 | Live | unknown | unknown | C.17 | GR |
| hCoV-19/Egypt/ARMY-239/2021 | EPI_ISL_1936108 | 02/05/2021 | Africa / Egypt / Cairo | Human | unknown | unknown | 25-55 | Hospitalized | unknown | unknown | B.1.1.7 | GR |
| hCoV-19/Egypt/CUNCI-HGC12I046/2021 | EPI_ISL_907118 | 03/01/2021 | Africa / Egypt | Human | unknown | unknown | unknown | unknown | unknown | unknown | B.1 | G |
| hCoV-19/Egypt/CUNCI-HGC12I040/2021 | EPI_ISL_907117 | 03/01/2021 | Africa / Egypt | Human | unknown | unknown | unknown | unknown | unknown | unknown | C.36 | GR |
| hCoV-19/Egypt/CUNCI-HGC12I025/2021 | EPI_ISL_907116 | 03/01/2021 | Africa / Egypt | Human | unknown | unknown | unknown | unknown | unknown | unknown | C.36 | GR |
| hCoV-19/Egypt/CUNCI-HGC12I024/2021 | EPI_ISL_907115 | 03/01/2021 | Africa / Egypt | Human | unknown | unknown | unknown | unknown | unknown | unknown | C.36 | GR |
| hCoV-19/Egypt/CUNCI-HGC12I002/2021 | EPI_ISL_907114 | 03/01/2021 | Africa / Egypt | Human | unknown | unknown | unknown | unknown | unknown | unknown | B.1.170 | G |
| hCoV-19/Egypt/CUNCI-HGC11I051/2021 | EPI_ISL_907113 | 03/01/2021 | Africa / Egypt | Human | unknown | unknown | unknown | unknown | unknown | unknown | C.36 | GR |
| hCoV-19/Egypt/CUNCI-HGC11I039/2021 | EPI_ISL_907110 | 03/01/2021 | Africa / Egypt | Human | unknown | unknown | unknown | unknown | unknown | unknown | C.36 | GR |
| hCoV-19/Egypt/CUNCI-HGC11I038/2021 | EPI_ISL_907109 | 03/01/2021 | Africa / Egypt | Human | unknown | unknown | unknown | unknown | unknown | unknown | B.1.1 | GR |
| hCoV-19/Egypt/CUNCI-HGC11I030/2021 | EPI_ISL_907108 | 03/01/2021 | Africa / Egypt | Human | unknown | unknown | unknown | unknown | unknown | unknown | B.1.466 | GH |
| hCoV-19/Egypt/CUNCI-HGC11I025/2021 | EPI_ISL_907107 | 03/01/2021 | Africa / Egypt | Human | unknown | unknown | unknown | unknown | unknown | unknown | A.28 | S |
| hCoV-19/Egypt/CUNCI-HGC11I024/2021 | EPI_ISL_907106 | 03/01/2021 | Africa / Egypt | Human | unknown | unknown | unknown | unknown | unknown | unknown | B.1 | GH |
| hCoV-19/Egypt/CUNCI-HGC11I023/2021 | EPI_ISL_907105 | 03/01/2021 | Africa / Egypt | Human | unknown | unknown | unknown | unknown | unknown | unknown | A | O |
| hCoV-19/Egypt/CUNCI-HGC11I021/2021 | EPI_ISL_907104 | 03/01/2021 | Africa / Egypt | Human | unknown | unknown | unknown | unknown | unknown | unknown | C.36 | GR |
| hCoV-19/Egypt/CUNCI-HGC11I011/2021 | EPI_ISL_907102 | 03/01/2021 | Africa / Egypt | Human | unknown | unknown | unknown | unknown | unknown | unknown | C.36 | GR |
| hCoV-19/Egypt/CUNCI-HGC11I007/2021 | EPI_ISL_907101 | 03/01/2021 | Africa / Egypt | Human | unknown | unknown | unknown | unknown | unknown | unknown | A.28 | S |
| hCoV-19/Egypt/CUNCI-HGC10I027/2020 | EPI_ISL_907100 | 25/12/2020 | Africa / Egypt | Human | unknown | unknown | unknown | unknown | unknown | unknown | B.1 | G |
| hCoV-19/Egypt/CUNCI-HGC10I021/2020 | EPI_ISL_907099 | 25/12/2020 | Africa / Egypt | Human | unknown | unknown | unknown | unknown | unknown | unknown | C.36 | GR |
| hCoV-19/Egypt/CUNCI-HGC10I009/2020 | EPI_ISL_907098 | 25/12/2020 | Africa / Egypt | Human | unknown | unknown | unknown | unknown | unknown | unknown | B.1 | GH |
| hCoV-19/Egypt/CUNCI-HGC09I041/2020 | EPI_ISL_907097 | 25/12/2020 | Africa / Egypt | Human | unknown | unknown | unknown | unknown | unknown | unknown | B.1 | GH |
| hCoV-19/Egypt/CUNCI-HGC09I025/2020 | EPI_ISL_907094 | 25/12/2020 | Africa / Egypt | Human | unknown | unknown | unknown | unknown | unknown | unknown | C.36 | GR |
| hCoV-19/Egypt/CUNCI-HGC11I003/2021 | EPI_ISL_907093 | 03/01/2021 | Africa / Egypt | Human | unknown | unknown | unknown | unknown | unknown | unknown | C.36 | GR |
| hCoV-19/Egypt/CUNCI-HGC10I014/2020 | EPI_ISL_907092 | 25/12/2020 | Africa / Egypt | Human | unknown | unknown | unknown | unknown | unknown | unknown | C.36 | GR |
| hCoV-19/Egypt/CUNCI-HGC10I015/2020 | EPI_ISL_907091 | 25/12/2020 | Africa / Egypt | Human | unknown | unknown | unknown | unknown | unknown | unknown | C.36 | GR |
| hCoV-19/Egypt/CUNCI-HGC9I040/2020 | EPI_ISL_907089 | 25/12/2020 | Africa / Egypt | Human | unknown | unknown | unknown | unknown | unknown | unknown | C.36 | GR |
| hCoV-19/Egypt/CUNCI-HGC9I015/2020 | EPI_ISL_907088 | 25/12/2020 | Africa / Egypt | Human | unknown | unknown | unknown | unknown | unknown | unknown | C.36 | GR |
| hCoV-19/Egypt/CUNCI-HGC8I033/2020 | EPI_ISL_907086 | 25/12/2020 | Africa / Egypt | Human | unknown | unknown | unknown | unknown | unknown | unknown | C.36 | GR |
| hCoV-19/Egypt/ARMY-MCL011/2020 | EPI_ISL_907084 | 19/11/2020 | Africa / Egypt | Human | unknown | unknown | unknown | unknown | unknown | unknown | B.1 | GH |
| hCoV-19/Egypt/ARMY-MCL0018/2020 | EPI_ISL_907082 | 19/11/2020 | Africa / Egypt | Human | unknown | unknown | unknown | unknown | unknown | unknown | C.36 | GR |
| hCoV-19/Egypt/ARMY-MCL008/2020 | EPI_ISL_907081 | 19/11/2020 | Africa / Egypt | Human | unknown | unknown | unknown | unknown | unknown | unknown | C.36 | GR |
| hCoV-19/Egypt/ARMY-MCL005/2020 | EPI_ISL_907080 | 19/11/2020 | Africa / Egypt | Human | unknown | unknown | unknown | unknown | unknown | unknown | C.36 | GR |
| hCoV-19/Egypt/ARMY/2020 | EPI_ISL_907076 | 01/11/2020 | Africa / Egypt | Human | unknown | unknown | unknown | unknown | unknown | unknown | C.17 | GR |
| hCoV-19/Egypt/CUNCI-HGC12I053/2021 | EPI_ISL_890231 | 09/01/2021 | Africa / Egypt | Human | unknown | unknown | unknown | unknown | unknown | unknown | C.17 | GR |
| hCoV-19/Egypt/CUNCI-HGC12I048/2021 | EPI_ISL_890228 | 09/01/2021 | Africa / Egypt | Human | unknown | unknown | unknown | unknown | unknown | unknown | C.36 | GR |
| hCoV-19/Egypt/CUNCI-HGC12I047/2021 | EPI_ISL_890227 | 09/01/2021 | Africa / Egypt | Human | unknown | unknown | unknown | unknown | unknown | unknown | C.36 | GR |
| hCoV-19/Egypt/CUNCI-HGC12I045/2021 | EPI_ISL_890226 | 09/01/2021 | Africa / Egypt | Human | unknown | unknown | unknown | unknown | unknown | unknown | C.36 | GR |
| hCoV-19/Egypt/CUNCI-HGC12I042/2021 | EPI_ISL_890224 | 09/01/2021 | Africa / Egypt | Human | unknown | unknown | unknown | unknown | unknown | unknown | B.1.466 | GH |
| hCoV-19/Egypt/CUNCI-HGC12I041/2021 | EPI_ISL_890223 | 09/01/2021 | Africa / Egypt | Human | unknown | unknown | unknown | unknown | unknown | unknown | B.1 | G |
| hCoV-19/Egypt/CUNCI-HGC12I039/2021 | EPI_ISL_890222 | 09/01/2021 | Africa / Egypt | Human | unknown | unknown | unknown | unknown | unknown | unknown | B.1 | GH |
| hCoV-19/Egypt/CUNCI-HGC12I038/2021 | EPI_ISL_890221 | 09/01/2021 | Africa / Egypt | Human | unknown | unknown | unknown | unknown | unknown | unknown | C.17 | GR |
| hCoV-19/Egypt/CUNCI-HGC12I037/2021 | EPI_ISL_890220 | 09/01/2021 | Africa / Egypt | Human | unknown | unknown | unknown | unknown | unknown | unknown | B.1.466 | GH |
| hCoV-19/Egypt/CUNCI-HGC12I036/2021 | EPI_ISL_890219 | 09/01/2021 | Africa / Egypt | Human | unknown | unknown | unknown | unknown | unknown | unknown | B.1 | G |
| hCoV-19/Egypt/CUNCI-HGC12I034/2021 | EPI_ISL_890217 | 09/01/2021 | Africa / Egypt | Human | unknown | unknown | unknown | unknown | unknown | unknown | C.36 | GR |
| hCoV-19/Egypt/CUNCI-HGC12I033/2021 | EPI_ISL_890216 | 09/01/2021 | Africa / Egypt | Human | unknown | unknown | unknown | unknown | unknown | unknown | B.1 | G |
| hCoV-19/Egypt/CUNCI-HGC12I032/2021 | EPI_ISL_890215 | 09/01/2021 | Africa / Egypt | Human | unknown | unknown | unknown | unknown | unknown | unknown | C.17 | GR |
| hCoV-19/Egypt/CUNCI-HGC12I031/2021 | EPI_ISL_890214 | 09/01/2021 | Africa / Egypt | Human | unknown | unknown | unknown | unknown | unknown | unknown | C.36 | GR |
| hCoV-19/Egypt/CUNCI-HGC12I030/2021 | EPI_ISL_890213 | 09/01/2021 | Africa / Egypt | Human | unknown | unknown | unknown | unknown | unknown | unknown | B.1.1 | G |
| hCoV-19/Egypt/CUNCI-HGC12I029/2021 | EPI_ISL_890212 | 09/01/2021 | Africa / Egypt | Human | unknown | unknown | unknown | unknown | unknown | unknown | C.36 | GR |
| hCoV-19/Egypt/CUNCI-HGC12I026/2021 | EPI_ISL_890211 | 09/01/2021 | Africa / Egypt | Human | unknown | unknown | unknown | unknown | unknown | unknown | C.36 | GR |
| hCoV-19/Egypt/CUNCI-HGC12I021/2021 | EPI_ISL_890208 | 09/01/2021 | Africa / Egypt | Human | unknown | unknown | unknown | unknown | unknown | unknown | C.36 | GR |
| hCoV-19/Egypt/CUNCI-HGC12I016/2021 | EPI_ISL_890207 | 09/01/2021 | Africa / Egypt | Human | unknown | unknown | unknown | unknown | unknown | unknown | C.36 | GR |
| hCoV-19/Egypt/CUNCI-HGC12I014/2021 | EPI_ISL_890206 | 09/01/2021 | Africa / Egypt | Human | unknown | unknown | unknown | unknown | unknown | unknown | C.36 | GR |
| hCoV-19/Egypt/CUNCI-HGC12I013/2021 | EPI_ISL_890205 | 09/01/2021 | Africa / Egypt | Human | unknown | unknown | unknown | unknown | unknown | unknown | C.17 | GR |
| hCoV-19/Egypt/CUNCI-HGC12I012/2021 | EPI_ISL_890204 | 09/01/2021 | Africa / Egypt | Human | unknown | unknown | unknown | unknown | unknown | unknown | C.17 | GR |
| hCoV-19/Egypt/CUNCI-HGC12I010/2021 | EPI_ISL_890202 | 09/01/2021 | Africa / Egypt | Human | unknown | unknown | unknown | unknown | unknown | unknown | C.17 | GR |
| hCoV-19/Egypt/CUNCI-HGC12I009/2021 | EPI_ISL_890201 | 09/01/2021 | Africa / Egypt | Human | unknown | unknown | unknown | unknown | unknown | unknown | C.36 | GR |
| hCoV-19/Egypt/CUNCI-HGC12I008/2021 | EPI_ISL_890200 | 09/01/2021 | Africa / Egypt | Human | unknown | unknown | unknown | unknown | unknown | unknown | C.36 | GR |
| hCoV-19/Egypt/CUNCI-HGC12I007/2021 | EPI_ISL_890199 | 09/01/2021 | Africa / Egypt | Human | unknown | unknown | unknown | unknown | unknown | unknown | C.36 | GR |
| hCoV-19/Egypt/CUNCI-HGC12I004/2021 | EPI_ISL_890197 | 09/01/2021 | Africa / Egypt | Human | unknown | unknown | unknown | unknown | unknown | unknown | B.1 | G |
| hCoV-19/Egypt/CUNCI-HGC12I003/2021 | EPI_ISL_890196 | 09/01/2021 | Africa / Egypt | Human | unknown | unknown | unknown | unknown | unknown | unknown | B.1.398 | G |
| hCoV-19/Egypt/CUNCI-HGC12I001/2021 | EPI_ISL_890195 | 09/01/2021 | Africa / Egypt | Human | unknown | unknown | unknown | unknown | unknown | unknown | B.1.170 | GH |
| hCoV-19/Egypt/CUNCI-HGC11I053/2021 | EPI_ISL_862813 | 03/01/2021 | Africa / Egypt | Human | unknown | unknown | unknown | unknown | unknown | unknown | C.36 | GR |
| hCoV-19/Egypt/CUNCI-HGC11I049/2021 | EPI_ISL_862811 | 03/01/2021 | Africa / Egypt | Human | unknown | unknown | unknown | unknown | unknown | unknown | B.1 | G |
| hCoV-19/Egypt/CUNCI-HGC11I047/2021 | EPI_ISL_862809 | 03/01/2021 | Africa / Egypt | Human | unknown | unknown | unknown | unknown | unknown | unknown | C.36 | GR |
| hCoV-19/Egypt/CUNCI-HGC11I040/2021 | EPI_ISL_862806 | 03/01/2021 | Africa / Egypt | Human | unknown | unknown | unknown | unknown | unknown | unknown | B.1.441 | G |
| hCoV-19/Egypt/CUNCI-HGC11I034/2021 | EPI_ISL_862803 | 03/01/2021 | Africa / Egypt | Human | unknown | unknown | unknown | unknown | unknown | unknown | C.36 | GR |
| hCoV-19/Egypt/CUNCI-HGC11I033/2021 | EPI_ISL_862802 | 03/01/2021 | Africa / Egypt | Human | unknown | unknown | unknown | unknown | unknown | unknown | C.36 | GR |
| hCoV-19/Egypt/CUNCI-HGC11I032/2021 | EPI_ISL_862801 | 03/01/2021 | Africa / Egypt | Human | unknown | unknown | unknown | unknown | unknown | unknown | B.1.170 | GH |
| hCoV-19/Egypt/CUNCI-HGC11I031/2021 | EPI_ISL_862800 | 03/01/2021 | Africa / Egypt | Human | unknown | unknown | unknown | unknown | unknown | unknown | C.36 | GR |
| hCoV-19/Egypt/CUNCI-HGC11I029/2021 | EPI_ISL_862799 | 03/01/2021 | Africa / Egypt | Human | unknown | unknown | unknown | unknown | unknown | unknown | B.1.466 | GH |
| hCoV-19/Egypt/CUNCI-HGC11I028/2021 | EPI_ISL_862798 | 03/01/2021 | Africa / Egypt | Human | unknown | unknown | unknown | unknown | unknown | unknown | C.36 | GR |
| hCoV-19/Egypt/CUNCI-HGC11I026/2021 | EPI_ISL_862796 | 03/01/2021 | Africa / Egypt | Human | unknown | unknown | unknown | unknown | unknown | unknown | B.1.398 | G |
| hCoV-19/Egypt/CUNCI-HGC11I016/2021 | EPI_ISL_862794 | 03/01/2021 | Africa / Egypt | Human | unknown | unknown | unknown | unknown | unknown | unknown | B.1 | G |
| hCoV-19/Egypt/CUNCI-HGC11I014/2021 | EPI_ISL_862793 | 03/01/2021 | Africa / Egypt | Human | unknown | unknown | unknown | unknown | unknown | unknown | B.1.398 | G |
| hCoV-19/Egypt/CUNCI-HGC11I013/2021 | EPI_ISL_862792 | 03/01/2021 | Africa / Egypt | Human | unknown | unknown | unknown | unknown | unknown | unknown | B.1.398 | G |
| hCoV-19/Egypt/CUNCI-HGC11I010/2021 | EPI_ISL_862791 | 03/01/2021 | Africa / Egypt | Human | unknown | unknown | unknown | unknown | unknown | unknown | C.36 | GR |
| hCoV-19/Egypt/CUNCI-HGC11I009/2021 | EPI_ISL_862790 | 03/01/2021 | Africa / Egypt | Human | unknown | unknown | unknown | unknown | unknown | unknown | C.36 | GR |
| hCoV-19/Egypt/CUNCI-HGC11I008/2021 | EPI_ISL_862789 | 03/01/2021 | Africa / Egypt | Human | unknown | unknown | unknown | unknown | unknown | unknown | B.1.177 | G |
| hCoV-19/Egypt/CUNCI-HGC9I014/2020 | EPI_ISL_907087 | 25/12/2020 | Africa / Egypt | Human | unknown | unknown | unknown | unknown | unknown | unknown | C.36 | GR |
| hCoV-19/Egypt/CUNCI-HGC9I025/2020 | EPI_ISL_862784 | 25/12/2020 | Africa / Egypt | Human | unknown | unknown | unknown | unknown | unknown | unknown | C.36 | GR |
| hCoV-19/Egypt/CUNCI-HGC9I030/2020 | EPI_ISL_862783 | 25/12/2020 | Africa / Egypt | Human | unknown | unknown | unknown | unknown | unknown | unknown | C.36 | GR |
| hCoV-19/Egypt/CUNCI-HGC11I005/2021 | EPI_ISL_862787 | 03/01/2021 | Africa / Egypt | Human | unknown | unknown | unknown | unknown | unknown | unknown | B.1.170 | GH |
| hCoV-19/Egypt/CUNCI-HGC11I004/2021 | EPI_ISL_862786 | 03/01/2021 | Africa / Egypt | Human | unknown | unknown | unknown | unknown | unknown | unknown | C.36 | GR |
| hCoV-19/Egypt/CUNCI-HGC10I024/2020 | EPI_ISL_862785 | 25/12/2020 | Africa / Egypt | Human | unknown | unknown | unknown | unknown | unknown | unknown | C.36 | GR |
| hCoV-19/Egypt/CUNCI-HGC9I043/2020 | EPI_ISL_857346 | 25/12/2020 | Africa / Egypt | Human | unknown | unknown | unknown | unknown | unknown | unknown | B.1.1 | GR |
| hCoV-19/Egypt/CUNCI-HGC9I038/2020 | EPI_ISL_857347 | 25/12/2020 | Africa / Egypt | Human | unknown | unknown | unknown | unknown | unknown | unknown | C.36 | GR |
| hCoV-19/Egypt/CUNCI-HGC9I034/2020 | EPI_ISL_857343 | 25/12/2020 | Africa / Egypt | Human | unknown | unknown | unknown | unknown | unknown | unknown | C.36 | GR |
| hCoV-19/Egypt/CUNCI-HGC9I016/2020 | EPI_ISL_857342 | 25/12/2020 | Africa / Egypt | Human | unknown | unknown | unknown | unknown | unknown | unknown | B.1.1 | GR |
| hCoV-19/Egypt/CUNCI-HGC9I024/2020 | EPI_ISL_857341 | 25/12/2020 | Africa / Egypt | Human | unknown | unknown | unknown | unknown | unknown | unknown | B.1.466.1 | GH |
| hCoV-19/Egypt/CUNCI-HGC9I033/2020 | EPI_ISL_857340 | 25/12/2020 | Africa / Egypt | Human | unknown | unknown | unknown | unknown | unknown | unknown | C.36 | GR |
| hCoV-19/Egypt/CUNCI-HGC9I021/2020 | EPI_ISL_857339 | 25/12/2020 | Africa / Egypt | Human | unknown | unknown | unknown | unknown | unknown | unknown | C.36 | GR |
| hCoV-19/Egypt/CUNCI-HGC9I031/2020 | EPI_ISL_857338 | 25/12/2020 | Africa / Egypt | Human | unknown | unknown | unknown | unknown | unknown | unknown | C.36 | GR |
| hCoV-19/Egypt/CUNCI-HGC9I037/2020 | EPI_ISL_857337 | 25/12/2020 | Africa / Egypt | Human | unknown | unknown | unknown | unknown | unknown | unknown | B.1.466 | GH |
| hCoV-19/Egypt/CUNCI-HGC9I036/2020 | EPI_ISL_857336 | 25/12/2020 | Africa / Egypt | Human | unknown | unknown | unknown | unknown | unknown | unknown | A.28 | S |
| hCoV-19/Egypt/CUNCI-HGC9I028/2020 | EPI_ISL_857334 | 25/12/2020 | Africa / Egypt | Human | unknown | unknown | unknown | unknown | unknown | unknown | C.36 | GR |
| hCoV-19/Egypt/CUNCI-HGC9I022/2020 | EPI_ISL_857332 | 25/12/2020 | Africa / Egypt | Human | unknown | unknown | unknown | unknown | unknown | unknown | A | GH |
| hCoV-19/Egypt/CUNCI-HGC9I023/2020 | EPI_ISL_857330 | 25/12/2020 | Africa / Egypt | Human | unknown | unknown | unknown | unknown | unknown | unknown | B.1.398 | G |
| hCoV-19/Egypt/CUNCI-HGC9I013/2020 | EPI_ISL_857328 | 25/12/2020 | Africa / Egypt | Human | unknown | unknown | unknown | unknown | unknown | unknown | C.36 | GR |
| hCoV-19/Egypt/CUNCI-HGC9I011/2020 | EPI_ISL_857327 | 25/12/2020 | Africa / Egypt | Human | unknown | unknown | unknown | unknown | unknown | unknown | B.1 | G |
| hCoV-19/Egypt/CUNCI-HGC9I007/2020 | EPI_ISL_857325 | 25/12/2020 | Africa / Egypt | Human | unknown | unknown | unknown | unknown | unknown | unknown | B.1.170 | GH |
| hCoV-19/Egypt/CUNCI-HGC9I004/2020 | EPI_ISL_857324 | 25/12/2020 | Africa / Egypt | Human | unknown | unknown | unknown | unknown | unknown | unknown | C.36 | GR |
| hCoV-19/Egypt/CUNCI-HGC8I039/2020 | EPI_ISL_857323 | 25/12/2020 | Africa / Egypt | Human | unknown | unknown | unknown | unknown | unknown | unknown | C.36 | GR |
| hCoV-19/Egypt/CUNCI-HGC8I040/2020 | EPI_ISL_857322 | 25/12/2020 | Africa / Egypt | Human | unknown | unknown | unknown | unknown | unknown | unknown | C.36 | GR |
| hCoV-19/Egypt/CUNCI-HGC8I037/2020 | EPI_ISL_857321 | 25/12/2020 | Africa / Egypt | Human | unknown | unknown | unknown | unknown | unknown | unknown | C.36 | GR |
| hCoV-19/Egypt/CUNCI-HGC8I036/2020 | EPI_ISL_857320 | 25/12/2020 | Africa / Egypt | Human | unknown | unknown | unknown | unknown | unknown | unknown | C.36 | GR |
| hCoV-19/Egypt/CUNCI-HGC8I034/2020 | EPI_ISL_857319 | 25/12/2020 | Africa / Egypt | Human | unknown | unknown | unknown | unknown | unknown | unknown | C.36 | GR |
| hCoV-19/Egypt/CUNCI-HGC8I035/2020 | EPI_ISL_857318 | 25/12/2020 | Africa / Egypt | Human | unknown | unknown | unknown | unknown | unknown | unknown | C.36 | GR |
| hCoV-19/Egypt/CUNCI-HGC8I032/2020 | EPI_ISL_857317 | 25/12/2020 | Africa / Egypt | Human | unknown | unknown | unknown | unknown | unknown | unknown | C.36 | GR |
| hCoV-19/Egypt/CUNCI-HGC8I025/2020 | EPI_ISL_857315 | 25/12/2020 | Africa / Egypt | Human | unknown | unknown | unknown | unknown | unknown | unknown | B.1.36 | GH |
| hCoV-19/Egypt/CUNCI-HGC9I032/2020 | EPI_ISL_857344 | 25/12/2020 | Africa / Egypt | Human | unknown | unknown | unknown | unknown | unknown | unknown | C.36 | GR |
| hCoV-19/Egypt/CCHE57357-P-39/2020 | EPI_ISL_812869 | 28/07/2020 | Africa / Egypt | Human | unknown | unknown | unknown | unknown | unknown | unknown | B.1.1.1 | GR |
| hCoV-19/Egypt/CCHE57357-P-38/2020 | EPI_ISL_812868 | 30/06/2020 | Africa / Egypt | Human | unknown | unknown | unknown | unknown | unknown | unknown | B.1.1 | GR |
| hCoV-19/Egypt/CCHE57357-P-37/2020 | EPI_ISL_812867 | 28/07/2020 | Africa / Egypt | Human | unknown | unknown | unknown | unknown | unknown | unknown | B | L |
| hCoV-19/Egypt/CCHE57357-P-36/2020 | EPI_ISL_812866 | 04/07/2020 | Africa / Egypt | Human | unknown | unknown | unknown | unknown | unknown | unknown | B.1.1 | O |
| hCoV-19/Egypt/CCHE57357-P-33/2020 | EPI_ISL_812864 | 12/07/2020 | Africa / Egypt | Human | unknown | unknown | unknown | unknown | unknown | unknown | C.36 | GR |
| hCoV-19/Egypt/CCHE57357-P-32/2020 | EPI_ISL_812863 | 30/06/2020 | Africa / Egypt | Human | unknown | unknown | unknown | unknown | unknown | unknown | B.1.1.1 | GR |
| hCoV-19/Egypt/CCHE57357-P-30/2020 | EPI_ISL_812862 | 04/07/2020 | Africa / Egypt | Human | unknown | unknown | unknown | unknown | unknown | unknown | B.1 | GH |
| hCoV-19/Egypt/CCHE57357-P-27/2020 | EPI_ISL_812861 | 12/07/2020 | Africa / Egypt | Human | unknown | unknown | unknown | unknown | unknown | unknown | B | L |
| hCoV-19/Egypt/CCHE57357-P-22/2020 | EPI_ISL_812857 | 24/08/2020 | Africa / Egypt | Human | unknown | unknown | unknown | unknown | unknown | unknown | B.1 | GH |
| hCoV-19/Egypt/CCHE57357-P-20/2020 | EPI_ISL_812856 | 26/06/2020 | Africa / Egypt | Human | unknown | unknown | unknown | unknown | unknown | unknown | B.1 | GH |
| hCoV-19/Egypt/CCHE57357-P-17/2020 | EPI_ISL_812855 | 05/07/2020 | Africa / Egypt | Human | unknown | unknown | unknown | unknown | unknown | unknown | B.1 | GH |
| hCoV-19/Egypt/CCHE57357-P-16/2020 | EPI_ISL_812854 | 22/06/2020 | Africa / Egypt | Human | unknown | unknown | unknown | unknown | unknown | unknown | B.1 | GH |
| hCoV-19/Egypt/CCHE57357-P-10/2020 | EPI_ISL_812852 | 03/08/2020 | Africa / Egypt | Human | unknown | unknown | unknown | unknown | unknown | unknown | B.1 | G |
| hCoV-19/Egypt/CCHE57357-A-91/2020 | EPI_ISL_812848 | 30/06/2020 | Africa / Egypt | Human | unknown | unknown | unknown | unknown | unknown | unknown | B.1 | G |
| hCoV-19/Egypt/CCHE57357-A-90/2020 | EPI_ISL_812847 | 26/06/2020 | Africa / Egypt | Human | unknown | unknown | unknown | unknown | unknown | unknown | C.36 | GR |
| hCoV-19/Egypt/CCHE57357-A-89/2020 | EPI_ISL_812846 | 02/08/2020 | Africa / Egypt | Human | unknown | unknown | unknown | unknown | unknown | unknown | B.1 | G |
| hCoV-19/Egypt/CCHE57357-A-75/2020 | EPI_ISL_812843 | 28/07/2020 | Africa / Egypt | Human | unknown | unknown | unknown | unknown | unknown | unknown | A | O |
| hCoV-19/Egypt/CCHE57357-A-71/2020 | EPI_ISL_812841 | 23/07/2020 | Africa / Egypt | Human | unknown | unknown | unknown | unknown | unknown | unknown | B | L |
| hCoV-19/Egypt/CCHE57357-A-68/2020 | EPI_ISL_812838 | 29/06/2020 | Africa / Egypt | Human | unknown | unknown | unknown | unknown | unknown | unknown | B.1 | O |
| hCoV-19/Egypt/CCHE57357-A-67/2020 | EPI_ISL_812837 | 08/07/2020 | Africa / Egypt | Human | unknown | unknown | unknown | unknown | unknown | unknown | B.1.1 | O |
| hCoV-19/Egypt/CCHE57357-P-14/2020 | EPI_ISL_812853 | 11/06/2020 | Africa / Egypt | Human | unknown | unknown | unknown | unknown | unknown | unknown | B | G |
| hCoV-19/Egypt/CCHE57357-A-63/2020 | EPI_ISL_812835 | 25/07/2020 | Africa / Egypt | Human | unknown | unknown | unknown | unknown | unknown | unknown | C.36 | GR |
| hCoV-19/Egypt/CCHE57357-A-62/2020 | EPI_ISL_812834 | 03/08/2020 | Africa / Egypt | Human | unknown | unknown | unknown | unknown | unknown | unknown | C.36 | GR |
| hCoV-19/Egypt/CCHE57357-A-57/2020 | EPI_ISL_812830 | 02/08/2020 | Africa / Egypt | Human | unknown | unknown | unknown | unknown | unknown | unknown | B.1.1 | GR |
| hCoV-19/Egypt/CCHE57357-A-52/2020 | EPI_ISL_812825 | 16/07/2020 | Africa / Egypt | Human | unknown | unknown | unknown | unknown | unknown | unknown | C.17 | GR |
| hCoV-19/Egypt/CCHE57357-A-51/2020 | EPI_ISL_812824 | 11/07/2020 | Africa / Egypt | Human | unknown | unknown | unknown | unknown | unknown | unknown | C.17 | GR |
| hCoV-19/Egypt/CCHE57357-A-66/2020 | EPI_ISL_812836 | 11/06/2020 | Africa / Egypt | Human | unknown | unknown | unknown | unknown | unknown | unknown | B | L |
| hCoV-19/Egypt/CCHE57357-A-49/2020 | EPI_ISL_812822 | 01/07/2020 | Africa / Egypt | Human | unknown | unknown | unknown | unknown | unknown | unknown | C.36 | GR |
| hCoV-19/Egypt/CCHE57357-A-47/2020 | EPI_ISL_812820 | 30/06/2020 | Africa / Egypt | Human | unknown | unknown | unknown | unknown | unknown | unknown | B.1 | O |
| hCoV-19/Egypt/CCHE57357-A-48/2020 | EPI_ISL_812821 | 28/07/2020 | Africa / Egypt | Human | unknown | unknown | unknown | unknown | unknown | unknown | A.28 | S |
| hCoV-19/Egypt/CCHE57357-A-45/2020 | EPI_ISL_812818 | 20/06/2020 | Africa / Egypt | Human | unknown | unknown | unknown | unknown | unknown | unknown | B.1.1 | GR |
| hCoV-19/Egypt/CCHE57357-A-43/2020 | EPI_ISL_812816 | 12/07/2020 | Africa / Egypt | Human | unknown | unknown | unknown | unknown | unknown | unknown | C.17 | GR |
| hCoV-19/Egypt/CCHE57357-A-42/2020 | EPI_ISL_812815 | 23/07/2020 | Africa / Egypt | Human | unknown | unknown | unknown | unknown | unknown | unknown | B.1.1 | GR |
| hCoV-19/Egypt/CCHE57357-A-46/2020 | EPI_ISL_812819 | 03/08/2020 | Africa / Egypt | Human | unknown | unknown | unknown | unknown | unknown | unknown | B.1 | GH |
| hCoV-19/Egypt/CCHE57357-A-41/2020 | EPI_ISL_812814 | 02/08/2020 | Africa / Egypt | Human | unknown | unknown | unknown | unknown | unknown | unknown | B.1.1 | GR |
| hCoV-19/Egypt/CCHE57357-A-36/2020 | EPI_ISL_812810 | 02/08/2020 | Africa / Egypt | Human | unknown | unknown | unknown | unknown | unknown | unknown | B | G |
| hCoV-19/Egypt/CCHE57357-A-38/2020 | EPI_ISL_812811 | 19/07/2020 | Africa / Egypt | Human | unknown | unknown | unknown | unknown | unknown | unknown | B | L |
| hCoV-19/Egypt/CCHE57357-A-34/2020 | EPI_ISL_812808 | 11/07/2020 | Africa / Egypt | Human | unknown | unknown | unknown | unknown | unknown | unknown | C.36 | GR |
| hCoV-19/Egypt/CCHE57357-A-35/2020 | EPI_ISL_812809 | 16/07/2020 | Africa / Egypt | Human | unknown | unknown | unknown | unknown | unknown | unknown | B.1.1.1 | GR |
| hCoV-19/Egypt/CCHE57357-A-30/2020 | EPI_ISL_812806 | 30/06/2020 | Africa / Egypt | Human | unknown | unknown | unknown | unknown | unknown | unknown | B.1 | G |
| hCoV-19/Egypt/CCHE57357-A-29/2020 | EPI_ISL_812805 | 28/07/2020 | Africa / Egypt | Human | unknown | unknown | unknown | unknown | unknown | unknown | B.1 | L |
| hCoV-19/Egypt/CCHE57357-A-27/2020 | EPI_ISL_812803 | 05/07/2020 | Africa / Egypt | Human | unknown | unknown | unknown | unknown | unknown | unknown | B.1.1 | G |
| hCoV-19/Egypt/CCHE57357-A-25/2020 | EPI_ISL_812801 | 27/05/2020 | Africa / Egypt | Human | unknown | unknown | unknown | unknown | unknown | unknown | B.1 | G |
| hCoV-19/Egypt/CCHE57357-A-24/2020 | EPI_ISL_812800 | 25/05/2020 | Africa / Egypt | Human | unknown | unknown | unknown | unknown | unknown | unknown | B.1.1 | GR |
| hCoV-19/Egypt/CCHE57357-A-23/2020 | EPI_ISL_812799 | 21/05/2020 | Africa / Egypt | Human | unknown | unknown | unknown | unknown | unknown | unknown | C.36 | GR |
| hCoV-19/Egypt/CCHE57357-A-22/2020 | EPI_ISL_812798 | 04/07/2020 | Africa / Egypt | Human | unknown | unknown | unknown | unknown | unknown | unknown | B.1 | G |
| hCoV-19/Egypt/CCHE57357-A-19/2020 | EPI_ISL_812797 | 04/07/2020 | Africa / Egypt | Human | unknown | unknown | unknown | unknown | unknown | unknown | C.36 | GR |
| hCoV-19/Egypt/CCHE57357-A-26/2020 | EPI_ISL_812802 | 02/08/2020 | Africa / Egypt | Human | unknown | unknown | unknown | unknown | unknown | unknown | B | L |
| hCoV-19/Egypt/CCHE57357-A-18/2020 | EPI_ISL_812796 | 03/07/2020 | Africa / Egypt | Human | unknown | unknown | unknown | unknown | unknown | unknown | C.36 | GR |
| hCoV-19/Egypt/CCHE57357-A-12/2020 | EPI_ISL_812792 | 20/05/2020 | Africa / Egypt | Human | unknown | unknown | unknown | unknown | unknown | unknown | B.1 | GH |
| hCoV-19/Egypt/CCHE57357-A-09/2020 | EPI_ISL_812790 | 14/05/2020 | Africa / Egypt | Human | unknown | unknown | unknown | unknown | unknown | unknown | B.1 | GH |
| hCoV-19/Egypt/CCHE57357-A-15/2020 | EPI_ISL_812795 | 09/06/2020 | Africa / Egypt | Human | unknown | unknown | unknown | unknown | unknown | unknown | C.36 | GR |
| hCoV-19/Egypt/CCHE57357-A-06/2020 | EPI_ISL_812787 | 30/05/2020 | Africa / Egypt | Human | unknown | unknown | unknown | unknown | unknown | unknown | C.36 | GR |
| hCoV-19/Egypt/CCHE57357-A-05/2020 | EPI_ISL_812786 | 24/05/2020 | Africa / Egypt | Human | unknown | unknown | unknown | unknown | unknown | unknown | B.1.1.1 | GR |
| hCoV-19/Egypt/CCHE57357-A-03/2020 | EPI_ISL_812785 | 14/05/2020 | Africa / Egypt | Human | unknown | unknown | unknown | unknown | unknown | unknown | B | G |
| hCoV-19/Egypt/CCHE57357-A-01/2020 | EPI_ISL_812783 | 24/05/2020 | Africa / Egypt | Human | unknown | unknown | unknown | unknown | unknown | unknown | C.36 | GR |
| hCoV-19/Egypt/CUNCI-HGC11I022/2021 | EPI_ISL_862795 | 03/01/2021 | Africa / Egypt | Human | unknown | unknown | unknown | unknown | unknown | unknown | C.36 | GR |
| hCoV-19/Egypt/NRC-7475/2020 | EPI_ISL_8189551 | 24/12/2020 | Africa / Egypt | Human | unknown | unknown | unknown | unknown | unknown | unknown | C.17 | GR |
| hCoV-19/Egypt/ARMY-MCL003/2020 | EPI_ISL_907079 | 19/11/2020 | Africa / Egypt | Human | unknown | unknown | unknown | unknown | unknown | unknown | C.36 | GR |
| hCoV-19/Egypt/CPHL-NRC-22/2020 | EPI_ISL_794603 | 13/03/2020 | Africa / Egypt | Human | unknown | unknown | unknown | unknown | unknown | unknown | B.1 | GH |
| hCoV-19/Egypt/CPHL-NRC-23/2020 | EPI_ISL_794602 | 13/03/2020 | Africa / Egypt | Human | unknown | unknown | unknown | unknown | unknown | unknown | B.1 | GH |
| hCoV-19/Egypt/CPHL-NRC-1/2020 | EPI_ISL_794601 | 10/06/2020 | Africa / Egypt | Human | unknown | unknown | unknown | unknown | unknown | unknown | B.1 | GH |
| hCoV-19/Egypt/CPHL-NRC-3/2020 | EPI_ISL_794600 | 05/04/2020 | Africa / Egypt | Human | unknown | unknown | unknown | unknown | unknown | unknown | B.1 | GH |
| hCoV-19/Egypt/CPHL-NRC-10/2020 | EPI_ISL_794599 | 12/04/2020 | Africa / Egypt | Human | unknown | unknown | unknown | unknown | unknown | unknown | B.1 | GH |
| hCoV-19/Egypt/CCHE57357-A-08/2020 | EPI_ISL_812789 | 07/05/2020 | Africa / Egypt | Human | unknown | unknown | unknown | unknown | unknown | unknown | B.1 | GH |
| hCoV-19/Egypt/CCHE57357-A-07/2020 | EPI_ISL_812788 | 08/06/2020 | Africa / Egypt | Human | unknown | unknown | unknown | unknown | unknown | unknown | B.1 | GH |
| hCoV-19/Egypt/CPHL-NRC-17/2020 | EPI_ISL_794595 | 25/03/2020 | Africa / Egypt | Human | unknown | unknown | unknown | unknown | unknown | unknown | B.1.535 | G |
| hCoV-19/Egypt/CPHL-NRC-18/2020 | EPI_ISL_794594 | 25/03/2020 | Africa / Egypt | Human | unknown | unknown | unknown | unknown | unknown | unknown | B.1.535 | G |
| hCoV-19/Egypt/CPHL-NRC-20/2020 | EPI_ISL_794593 | 13/03/2020 | Africa / Egypt | Human | unknown | unknown | unknown | unknown | unknown | unknown | B.1 | GH |
| hCoV-19/Egypt/MASRI-C5-010/2020 | EPI_ISL_1167190 | 06/06/2020 | Africa / Egypt / Cairo | Human | unknown | Female | 6 | unknown | unknown | unknown | B.1 | G |
| hCoV-19/Egypt/NRC2/2020 | EPI_ISL_1315065 | 26/04/2020 | Africa / Egypt / Cairo / Giza | Human | unknown | Female | 55 | Hospitalized | unknown | unknown | B.1 | GH |
| hCoV-19/Egypt/CCHE57357-P-24/2020 | EPI_ISL_812858 | 21/06/2020 | Africa / Egypt | Human | unknown | unknown | unknown | unknown | unknown | unknown | B.1.1 | GR |
| hCoV-19/Egypt/CCHE57357-A-14/2020 | EPI_ISL_812794 | 20/05/2020 | Africa / Egypt | Human | unknown | unknown | unknown | unknown | unknown | unknown | B.1 | GH |
| hCoV-19/Egypt/CCHE57357_Wave_4_071/2021 | EPI_ISL_6011742 | 22/10/2021 | Africa / Egypt | Human | unknown | unknown | unknown | unknown | unknown | unknown | B.1.617.2 | GK |
| hCoV-19/Egypt/CCHE57357_Wave_4_115/2021 | EPI_ISL_6011786 | 19/10/2021 | Africa / Egypt | Human | unknown | unknown | unknown | unknown | unknown | unknown | AY.65 | GK |
| hCoV-19/Egypt/CCHE57357_Wave_4_117/2021 | EPI_ISL_6011788 | 18/10/2021 | Africa / Egypt | Human | unknown | unknown | unknown | unknown | unknown | unknown | AY.122 | GK |
| hCoV-19/Egypt/CCHE57357_Wave_4_106/2021 | EPI_ISL_6011777 | 19/10/2021 | Africa / Egypt | Human | unknown | unknown | unknown | unknown | unknown | unknown | B.1.617.2 | GK |
| hCoV-19/Egypt/NRC-5578OP/2020 | EPI_ISL_8193660 | 03/06/2020 | Africa / Egypt | Human | unknown | unknown | unknown | unknown | unknown | unknown | B.1.1.192 | GH |
| hCoV-19/Egypt/NRC-6207/2020 | EPI_ISL_8193860 | 04/07/2020 | Africa / Egypt | Human | unknown | unknown | unknown | unknown | unknown | unknown | B.1.1 | GR |
| hCoV-19/Egypt/NRC-5616OP/2020 | EPI_ISL_8194873 | 07/06/2020 | Africa / Egypt | Human | unknown | unknown | unknown | unknown | unknown | unknown | C.17 | GR |
| hCoV-19/Egypt/CCHE57357_Wave_4_130/2021 | EPI_ISL_6011801 | 18/10/2021 | Africa / Egypt | Human | unknown | unknown | unknown | unknown | unknown | unknown | B.1.617.2 | GK |
| hCoV-19/Egypt/NRC-533/2021 | EPI_ISL_8251483 | 27/04/2021 | Africa / Egypt | Human | unknown | unknown | unknown | unknown | unknown | unknown | C.17 | GR |
| hCoV-19/Egypt/NRC-5443NS/2020 | EPI_ISL_8193874 | 27/05/2020 | Africa / Egypt | Human | unknown | unknown | unknown | unknown | unknown | unknown | B.1.170 | G |
| hCoV-19/dog/Egypt/AHRI-6/2020 | EPI_ISL_2932465 | 2020-12 | Africa / Egypt | Human | unknown | unknown | unknown | unknown | unknown | unknown | Unassigned | O |
| hCoV-19/cat/Egypt/AHRI-1/2020 | EPI_ISL_2932460 | 2020-12 | Africa / Egypt | Human | unknown | unknown | unknown | unknown | unknown | unknown | Unassigned | O |
| hCoV-19/cat/Egypt/AHRI-2/2020 | EPI_ISL_2932461 | 2020-12 | Africa / Egypt | Human | unknown | unknown | unknown | unknown | unknown | unknown | Unassigned | O |
| hCoV-19/Egypt/NRC-5627/2020 | EPI_ISL_8194875 | 06/06/2020 | Africa / Egypt | Human | unknown | unknown | unknown | unknown | unknown | unknown | B.1 | GH |
| hCoV-19/Egypt/NRC-536/2021 | EPI_ISL_8251484 | 28/04/2021 | Africa / Egypt | Human | unknown | unknown | unknown | unknown | unknown | unknown | C.36.3 | GR |
| hCoV-19/Egypt/NRC-6357/2020 | EPI_ISL_8194884 | 22/07/2020 | Africa / Egypt | Human | unknown | unknown | unknown | unknown | unknown | unknown | B.1.1 | G |
| hCoV-19/Egypt/NRC-5552OP/2020 | EPI_ISL_8193584 | 03/06/2020 | Africa / Egypt | Human | unknown | unknown | unknown | unknown | unknown | unknown | B.1 | G |
| hCoV-19/Egypt/NRC-7269/2020 | EPI_ISL_8193644 | 14/12/2020 | Africa / Egypt | Human | unknown | unknown | unknown | unknown | unknown | unknown | B.1.1 | G |
| hCoV-19/Egypt/NRC-6376/2020 | EPI_ISL_8194885 | 26/07/2020 | Africa / Egypt | Human | unknown | unknown | unknown | unknown | unknown | unknown | B.1.1.1 | GR |
| hCoV-19/Egypt/NRC-921/2021 | EPI_ISL_8250585 | 25/08/2021 | Africa / Egypt | Human | unknown | Female | unknown | unknown | unknown | unknown | B.1.617.2 | GK |
| hCoV-19/Egypt/NRC-5448OP/2020 | EPI_ISL_8193876 | 27/05/2020 | Africa / Egypt | Human | unknown | unknown | unknown | unknown | unknown | unknown | C.17 | GR |
| hCoV-19/Egypt/CCHE57357_Wave_4_082/2021 | EPI_ISL_6011753 | 22/10/2021 | Africa / Egypt | Human | unknown | unknown | unknown | unknown | unknown | unknown | B.1.617.2 | GK |
| hCoV-19/Egypt/CCHE57357_Wave_4_093/2021 | EPI_ISL_6011764 | 21/10/2021 | Africa / Egypt | Human | unknown | unknown | unknown | unknown | unknown | unknown | B.1.617.2 | GK |
| hCoV-19/Egypt/CCHE57357_Wave_4_099/2021 | EPI_ISL_6011770 | 20/10/2021 | Africa / Egypt | Human | unknown | unknown | unknown | unknown | unknown | unknown | AY.126 | GK |
| hCoV-19/Egypt/CCHE57357_Wave_4_100/2021 | EPI_ISL_6011771 | 21/10/2021 | Africa / Egypt | Human | unknown | unknown | unknown | unknown | unknown | unknown | B.1.617.2 | GK |
| hCoV-19/Egypt/CCHE57357_Wave_4_105/2021 | EPI_ISL_6011776 | 19/10/2021 | Africa / Egypt | Human | unknown | unknown | unknown | unknown | unknown | unknown | B.1.617.2 | GK |
| hCoV-19/Egypt/CCHE57357_Wave_4_094/2021 | EPI_ISL_6011765 | 21/10/2021 | Africa / Egypt | Human | unknown | unknown | unknown | unknown | unknown | unknown | AY.127 | GK |
| hCoV-19/Egypt/CCHE57357_Wave_4_089/2021 | EPI_ISL_6011760 | 22/10/2021 | Africa / Egypt | Human | unknown | unknown | unknown | unknown | unknown | unknown | B.1.617.2 | GK |
| hCoV-19/Egypt/CCHE57357_Wave_4_090/2021 | EPI_ISL_6011761 | 22/10/2021 | Africa / Egypt | Human | unknown | unknown | unknown | unknown | unknown | unknown | AY.112 | GK |
| hCoV-19/Egypt/CCHE57357_Wave_4_074/2021 | EPI_ISL_6011745 | 23/10/2021 | Africa / Egypt | Human | unknown | unknown | unknown | unknown | unknown | unknown | B.1.617.2 | GK |
| hCoV-19/Egypt/CCHE57357_Wave_4_120/2021 | EPI_ISL_6011791 | 19/10/2021 | Africa / Egypt | Human | unknown | unknown | unknown | unknown | unknown | unknown | AY.65 | GK |
| hCoV-19/Egypt/CCHE57357_Wave_4_081/2021 | EPI_ISL_6011752 | 23/10/2021 | Africa / Egypt | Human | unknown | unknown | unknown | unknown | unknown | unknown | B.1.617.2 | GK |
| hCoV-19/Egypt/CCHE57357_Wave_4_084/2021 | EPI_ISL_6011755 | 21/10/2021 | Africa / Egypt | Human | unknown | unknown | unknown | unknown | unknown | unknown | B.1.617.2 | GK |
| hCoV-19/Egypt/CCHE57357_Wave_4_083/2021 | EPI_ISL_6011754 | 22/10/2021 | Africa / Egypt | Human | unknown | unknown | unknown | unknown | unknown | unknown | B.1.617.2 | GK |
| hCoV-19/Egypt/CCHE57357_Wave_4_127/2021 | EPI_ISL_6011798 | 16/10/2021 | Africa / Egypt | Human | unknown | unknown | unknown | unknown | unknown | unknown | AY.122 | GK |
| hCoV-19/Egypt/CCHE57357_Wave_4_122/2021 | EPI_ISL_6011793 | 19/10/2021 | Africa / Egypt | Human | unknown | unknown | unknown | unknown | unknown | unknown | AY.122 | GK |
| hCoV-19/Egypt/CCHE57357_Wave_4_123/2021 | EPI_ISL_6011794 | 16/10/2021 | Africa / Egypt | Human | unknown | unknown | unknown | unknown | unknown | unknown | B.1.617.2 | GK |
| hCoV-19/Egypt/CCHE57357_Wave_4_131/2021 | EPI_ISL_6011802 | 18/10/2021 | Africa / Egypt | Human | unknown | unknown | unknown | unknown | unknown | unknown | AY.122 | GK |
| hCoV-19/Egypt/NRC-6285/2020 | EPI_ISL_8193880 | 07/07/2020 | Africa / Egypt | Human | unknown | unknown | unknown | unknown | unknown | unknown | B.1.1 | GR |
| hCoV-19/Egypt/NRC-6902/2020 | EPI_ISL_8193618 | 12/10/2020 | Africa / Egypt | Human | unknown | unknown | unknown | unknown | unknown | unknown | B.1.1.1 | GH |
| hCoV-19/Egypt/NRC-637/2021 | EPI_ISL_8215720 | 17/05/2021 | Africa / Egypt | Human | unknown | unknown | unknown | unknown | unknown | unknown | C.36.3 | GR |
| hCoV-19/Egypt/NRC-6445/2020 | EPI_ISL_8193854 | 14/08/2020 | Africa / Egypt | Human | unknown | unknown | unknown | unknown | unknown | unknown | B.1.170 | GH |
| hCoV-19/Egypt/NRC-6231/2020 | EPI_ISL_8193886 | 06/07/2020 | Africa / Egypt | Human | unknown | unknown | unknown | unknown | unknown | unknown | B.1.170 | GH |
| hCoV-19/Egypt/NRC-6575/2020 | EPI_ISL_8194886 | 30/08/2020 | Africa / Egypt | Human | unknown | unknown | unknown | unknown | unknown | unknown | B.1 | GH |
| hCoV-19/Egypt/NRC-922/2021 | EPI_ISL_8250586 | 25/08/2021 | Africa / Egypt | Human | unknown | Male | unknown | unknown | unknown | unknown | B.1.617.2 | GK |
| hCoV-19/Egypt/NRC-5451NS/2020 | EPI_ISL_8189550 | 27/05/2020 | Africa / Egypt | Human | unknown | unknown | unknown | unknown | unknown | unknown | C.17 | GR |
| hCoV-19/Egypt/ARMY-356/2021 | EPI_ISL_1936286 | 04/04/2021 | Africa / Egypt / Cairo | Human | unknown | unknown | 25-60 | Hospitalized | unknown | unknown | B.1.1.7 | GR |
| hCoV-19/Egypt/ARMY-344/2021 | EPI_ISL_1936276 | 04/04/2021 | Africa / Egypt / Cairo | Human | unknown | unknown | 25-60 | Hospitalized | unknown | unknown | B.1.1.7 | GR |
| hCoV-19/Egypt/ARMY-216/2021 | EPI_ISL_1936143 | 02/05/2021 | Africa / Egypt / Cairo | Human | unknown | unknown | 25-55 | Hospitalized | unknown | unknown | B.1 | GH |
| hCoV-19/Egypt/ARMY-315/2021 | EPI_ISL_1936254 | 22/04/2021 | Africa / Egypt / Cairo | Human | unknown | unknown | 25-60 | Hospitalized | unknown | unknown | C.17 | GR |
| hCoV-19/Egypt/ARMY-304/2021 | EPI_ISL_1936243 | 22/04/2021 | Africa / Egypt / Cairo | Human | unknown | unknown | 25-60 | Hospitalized | unknown | unknown | C.36.3 | GR |
| hCoV-19/Egypt/ARMY-267/2021 | EPI_ISL_1936127 | 02/05/2021 | Africa / Egypt / Cairo | Human | unknown | unknown | 25-55 | Hospitalized | unknown | unknown | C.36.3 | GR |
| hCoV-19/Egypt/ARMY-EVA-Pharma-Wave4-011/2021 | EPI_ISL_4748271 | 04/09/2021 | Africa / Egypt / Cairo | Human | unknown | unknown | 25-55 | Hospitalized | unknown | unknown | B.1.617.2 | GK |
| hCoV-19/Egypt/NRC-596/2021 | EPI_ISL_8251487 | 09/05/2021 | Africa / Egypt | Human | unknown | unknown | unknown | unknown | unknown | unknown | C.17 | G |
| hCoV-19/Egypt/NRC-7313/2020 | EPI_ISL_8193636 | 15/12/2020 | Africa / Egypt | Human | unknown | unknown | unknown | unknown | unknown | unknown | B.1.1 | G |
| hCoV-19/Egypt/CUNCI-HGC9I035/2020 | EPI_ISL_857331 | 25/12/2020 | Africa / Egypt | Human | unknown | unknown | unknown | unknown | unknown | unknown | C.36 | GR |
| hCoV-19/Egypt/ARMY-281/2021 | EPI_ISL_1936132 | 02/05/2021 | Africa / Egypt / Cairo | Human | unknown | unknown | 25-55 | Hospitalized | unknown | unknown | B | L |
| hCoV-19/Egypt/NRC-5159/2020 | EPI_ISL_2232407 | 02/04/2020 | Africa / Egypt | Human | unknown | unknown | unknown | unknown | unknown | unknown | B.1.1.1 | GR |
| hCoV-19/Egypt/NRC-6365/2020 | EPI_ISL_8194880 | 25/07/2020 | Africa / Egypt | Human | unknown | unknown | unknown | unknown | unknown | unknown | B.1 | GH |
| hCoV-19/Egypt/ARMY-353/2021 | EPI_ISL_1936283 | 04/04/2021 | Africa / Egypt / Cairo | Human | unknown | unknown | 25-60 | Hospitalized | unknown | unknown | B | L |
| hCoV-19/Egypt/ARMY-280/2021 | EPI_ISL_1936131 | 02/05/2021 | Africa / Egypt / Cairo | Human | unknown | unknown | 25-55 | Hospitalized | unknown | unknown | B | L |
| hCoV-19/Egypt/NRC-6178/2020 | EPI_ISL_8193889 | 30/06/2020 | Africa / Egypt | Human | unknown | unknown | unknown | unknown | unknown | unknown | B.1.170 | GH |
| hCoV-19/Egypt/NRC-6350/2020 | EPI_ISL_8194864 | 24/06/2020 | Africa / Egypt | Human | unknown | unknown | unknown | unknown | unknown | unknown | B.1 | GH |
| hCoV-19/Egypt/NRC-699/2021 | EPI_ISL_8251504 | 24/05/2021 | Africa / Egypt | Human | unknown | unknown | unknown | unknown | unknown | unknown | C.36.3 | G |
| hCoV-19/Egypt/NRC-5558OP/2020 | EPI_ISL_8193657 | 03/06/2020 | Africa / Egypt | Human | unknown | unknown | unknown | unknown | unknown | unknown | C.17 | GR |
| hCoV-19/Egypt/NRC-1216/2021 | EPI_ISL_8205831 | 28/10/2021 | Africa / Egypt | Human | unknown | Male | unknown | unknown | unknown | unknown | B.1.617.2 | GK |
| hCoV-19/Egypt/NRC-1231/2021 | EPI_ISL_8207957 | 02/11/2021 | Africa / Egypt | Human | unknown | Male | unknown | unknown | unknown | unknown | B.1.617.2 | GK |
| hCoV-19/Egypt/ARMY-351/2021 | EPI_ISL_1936281 | 04/04/2021 | Africa / Egypt / Cairo | Human | unknown | unknown | 25-60 | Hospitalized | unknown | unknown | B | L |
| hCoV-19/Egypt/NRC-7068/2020 | EPI_ISL_8193590 | 08/11/2020 | Africa / Egypt | Human | unknown | unknown | unknown | unknown | unknown | unknown | B.1 | GH |
| hCoV-19/Egypt/NRC-613/2021 | EPI_ISL_8251490 | 10/05/2021 | Africa / Egypt | Human | unknown | unknown | unknown | unknown | unknown | unknown | B.1.170 | GH |
| hCoV-19/Egypt/ARMY-47/2021 | EPI_ISL_1936223 | 27/03/2021 | Africa / Egypt / Cairo | Human | unknown | unknown | 25-55 | Hospitalized | unknown | unknown | B | L |
| hCoV-19/Egypt/NRC-7307/2020 | EPI_ISL_8193637 | 15/12/2020 | Africa / Egypt | Human | unknown | unknown | unknown | unknown | unknown | unknown | C.17 | GH |
| hCoV-19/Egypt/NRC-6999/2020 | EPI_ISL_8193591 | 27/10/2020 | Africa / Egypt | Human | unknown | unknown | unknown | unknown | unknown | unknown | B.1.170 | GH |
| hCoV-19/Egypt/NRC-5772/2020 | EPI_ISL_8193892 | 13/06/2020 | Africa / Egypt | Human | unknown | unknown | unknown | unknown | unknown | unknown | B.1.170 | GH |
| hCoV-19/Egypt/NRC-6380/2020 | EPI_ISL_8194892 | 28/07/2020 | Africa / Egypt | Human | unknown | unknown | unknown | unknown | unknown | unknown | B.1.1.372 | GR |
| hCoV-19/Egypt/NRC-6219/2020 | EPI_ISL_2232335 | 04/07/2020 | Africa / Egypt | Human | unknown | Male | unknown | unknown | unknown | unknown | B.1.1.1 | GR |
| hCoV-19/Egypt/NRC-623/2021 | EPI_ISL_8251492 | 10/05/2021 | Africa / Egypt | Human | unknown | unknown | unknown | unknown | unknown | unknown | C.36.3 | GR |
| hCoV-19/Egypt/NRC-5548OP/2020 | EPI_ISL_8189341 | 03/06/2020 | Africa / Egypt | Human | unknown | unknown | unknown | unknown | unknown | unknown | B.1.1.1 | GH |
| hCoV-19/Egypt/NRC-6206/2020 | EPI_ISL_8193861 | 04/07/2020 | Africa / Egypt | Human | unknown | unknown | unknown | unknown | unknown | unknown | B.1.1.1 | GR |
| hCoV-19/Egypt/CPHL-S9/2021 | EPI_ISL_3274156 | 08/07/2021 | Africa / Egypt / Sohag | Human | Sentinel surveillance (ILI) | Female | 70 | unknown | unknown | Sentinel surveillance (ILI) | C.36 | GR |
| hCoV-19/Egypt/NRC-972/2021 | EPI_ISL_8215693 | 12/09/2021 | Africa / Egypt | Human | unknown | Female | unknown | unknown | unknown | unknown | AY.112 | GK |
| hCoV-19/Egypt/NRC-635/2021 | EPI_ISL_8251493 | 17/05/2021 | Africa / Egypt | Human | unknown | unknown | unknown | unknown | unknown | unknown | C.36.3 | GR |
| hCoV-19/Egypt/NRC-6352/2020 | EPI_ISL_8194882 | 26/07/2020 | Africa / Egypt | Human | unknown | unknown | unknown | unknown | unknown | unknown | B.1 | GH |
| hCoV-19/Egypt/NRC-6237/2020 | EPI_ISL_8193883 | 06/07/2020 | Africa / Egypt | Human | unknown | unknown | unknown | unknown | unknown | unknown | B.1.170 | GH |
| hCoV-19/Egypt/NRC-5556OP/2020 | EPI_ISL_8189346 | 03/06/2020 | Africa / Egypt | Human | unknown | unknown | unknown | unknown | unknown | unknown | C.17 | GR |
| hCoV-19/Egypt/NRC-6169/2020 | EPI_ISL_8193894 | 01/07/2020 | Africa / Egypt | Human | unknown | unknown | unknown | unknown | unknown | unknown | B.1.533 | GH |
| hCoV-19/Egypt/CUNCI-HGC11I036/2021 | EPI_ISL_862805 | 03/01/2021 | Africa / Egypt | Human | unknown | unknown | unknown | unknown | unknown | unknown | B.1.466 | GH |
| hCoV-19/Egypt/NRC-639/2021 | EPI_ISL_8251494 | 17/05/2021 | Africa / Egypt | Human | unknown | unknown | unknown | unknown | unknown | unknown | C.36.3 | GR |
| hCoV-19/Egypt/NRC-6590/2020 | EPI_ISL_8193647 | 03/09/2020 | Africa / Egypt | Human | unknown | unknown | unknown | unknown | unknown | unknown | B.1.1.1 | GR |
| hCoV-19/Egypt/NRC-5794/2020 | EPI_ISL_8189549 | 14/06/2020 | Africa / Egypt | Human | unknown | unknown | unknown | unknown | unknown | unknown | B.1 | G |
| hCoV-19/Egypt/Delta005/2021 | EPI_ISL_4629981 | 04/08/2021 | Africa / Egypt / Cairo | Human | unknown | unknown | unknown | Hospitalized | unknown | unknown | B.1.617.2 | GK |
| hCoV-19/Egypt/Delta001/2021 | EPI_ISL_4629977 | 04/08/2021 | Africa / Egypt / Cairo | Human | unknown | unknown | unknown | Hospitalized | unknown | unknown | B.1.617.2 | GK |
| hCoV-19/Egypt/Delta006/2021 | EPI_ISL_4629982 | 04/08/2021 | Africa / Egypt / Cairo | Human | unknown | unknown | unknown | Hospitalized | unknown | unknown | AY.34.1 | GK |
| hCoV-19/Egypt/Delta004/2021 | EPI_ISL_4629980 | 04/08/2021 | Africa / Egypt / Cairo | Human | unknown | unknown | unknown | Hospitalized | unknown | unknown | B.1.617.2 | GK |
| hCoV-19/Egypt/Delta003/2021 | EPI_ISL_4629979 | 04/08/2021 | Africa / Egypt / Cairo | Human | unknown | unknown | unknown | Hospitalized | unknown | unknown | AY.112 | GK |
| hCoV-19/Egypt/NRC-6661/2020 | EPI_ISL_8193595 | 10/09/2020 | Africa / Egypt | Human | unknown | unknown | unknown | unknown | unknown | unknown | B.1.195 | G |
| hCoV-19/Egypt/NRC-987/2021 | EPI_ISL_8215695 | 14/09/2021 | Africa / Egypt | Human | unknown | Female | unknown | unknown | unknown | unknown | B.1.617.2 | GK |
| hCoV-19/Egypt/NRC-650/2021 | EPI_ISL_8251495 | 18/05/2021 | Africa / Egypt | Human | unknown | unknown | unknown | unknown | unknown | unknown | C.17 | GR |
| hCoV-19/Egypt/NRC-5954/2020 | EPI_ISL_8189552 | 21/06/2020 | Africa / Egypt | Human | unknown | unknown | unknown | unknown | unknown | unknown | B.1.170 | GH |
| hCoV-19/Egypt/NRC-651/2021 | EPI_ISL_8251496 | 18/05/2021 | Africa / Egypt | Human | unknown | unknown | unknown | unknown | unknown | unknown | C.36.3 | G |
| hCoV-19/Egypt/NRC-6164/2020 | EPI_ISL_8193896 | 01/07/2020 | Africa / Egypt | Human | unknown | unknown | unknown | unknown | unknown | unknown | B.1 | G |
| hCoV-19/Egypt/NRC-6163/2020 | EPI_ISL_8193897 | 01/07/2020 | Africa / Egypt | Human | unknown | unknown | unknown | unknown | unknown | unknown | B.1 | G |
| hCoV-19/Egypt/NRC-998/2021 | EPI_ISL_8215697 | 19/09/2021 | Africa / Egypt | Human | unknown | Female | unknown | unknown | unknown | unknown | B.1.617.2 | GK |
| hCoV-19/Egypt/NRC-6659/2020 | EPI_ISL_8193597 | 10/09/2020 | Africa / Egypt | Human | unknown | unknown | unknown | unknown | unknown | unknown | B.1.1 | GR |
| hCoV-19/Egypt/ARMY-EVA-Pharma-Wave4-013/2021 | EPI_ISL_4748272 | 04/09/2021 | Africa / Egypt / Cairo | Human | unknown | unknown | 25-55 | Hospitalized | unknown | unknown | B.1.617.2 | GK |
| hCoV-19/Egypt/ARMY-EVA-Pharma-Wave4-015/2021 | EPI_ISL_4748274 | 04/09/2021 | Africa / Egypt / Cairo | Human | unknown | unknown | 25-55 | Hospitalized | unknown | unknown | B.1.617.2 | GK |
| hCoV-19/Egypt/ARMY-EVA-Pharma-Wave4-016/2021 | EPI_ISL_4748275 | 04/09/2021 | Africa / Egypt / Cairo | Human | unknown | unknown | 25-55 | Hospitalized | unknown | unknown | B.1.617.2 | GK |
| hCoV-19/Egypt/ARMY-EVA-Pharma-Wave4-017/2021 | EPI_ISL_4748276 | 04/09/2021 | Africa / Egypt / Cairo | Human | unknown | unknown | 25-55 | Hospitalized | unknown | unknown | AY.43 | GK |
| hCoV-19/Egypt/ARMY-EVA-Pharma-Wave4-018/2021 | EPI_ISL_4748277 | 04/09/2021 | Africa / Egypt / Cairo | Human | unknown | unknown | 25-55 | Hospitalized | unknown | unknown | AY.112 | GK |
| hCoV-19/Egypt/ARMY-EVA-Pharma-Wave4-020/2021 | EPI_ISL_4748278 | 04/09/2021 | Africa / Egypt / Cairo | Human | unknown | unknown | 25-55 | Hospitalized | unknown | unknown | B.1.617.2 | GK |
| hCoV-19/Egypt/ARMY-EVA-Pharma-Wave4-021/2021 | EPI_ISL_4748279 | 04/09/2021 | Africa / Egypt / Cairo | Human | unknown | unknown | 25-55 | Hospitalized | unknown | unknown | AY.65 | GK |
| hCoV-19/Egypt/ARMY-EVA-Pharma-Wave4-022/2021 | EPI_ISL_4748280 | 04/09/2021 | Africa / Egypt / Cairo | Human | unknown | unknown | 25-55 | Hospitalized | unknown | unknown | B.1.617.2 | GK |
| hCoV-19/Egypt/ARMY-EVA-Pharma-Wave4-023/2021 | EPI_ISL_4748281 | 04/09/2021 | Africa / Egypt / Cairo | Human | unknown | unknown | 25-55 | Hospitalized | unknown | unknown | AY.65 | GK |
| hCoV-19/Egypt/ARMY-EVA-Pharma-Wave4-027/2021 | EPI_ISL_4748284 | 04/09/2021 | Africa / Egypt / Cairo | Human | unknown | unknown | 25-55 | Hospitalized | unknown | unknown | B.1.617.2 | O |
| hCoV-19/Egypt/ARMY-EVA-Pharma-Wave4-010/2021 | EPI_ISL_4748270 | 04/09/2021 | Africa / Egypt / Cairo | Human | unknown | unknown | 25-55 | Hospitalized | unknown | unknown | B.1.617.2 | GK |
| hCoV-19/Egypt/NRC-537/2021 | EPI_ISL_8251485 | 28/04/2021 | Africa / Egypt | Human | unknown | unknown | unknown | unknown | unknown | unknown | C.38 | GR |
| hCoV-19/Egypt/ARMY-EVA-Pharma-Wave4-012/2021 | EPI_ISL_4750218 | 04/09/2021 | Africa / Egypt / Cairo | Human | unknown | unknown | 25-55 | Hospitalized | unknown | unknown | B.1.617.2 | GK |
| hCoV-19/Egypt/NRC-6658/2020 | EPI_ISL_8193598 | 10/09/2020 | Africa / Egypt | Human | unknown | unknown | unknown | unknown | unknown | unknown | B.1.1.1 | GR |
| hCoV-19/Egypt/NRC-6553/2020 | EPI_ISL_8193898 | 27/08/2020 | Africa / Egypt | Human | unknown | unknown | unknown | unknown | unknown | unknown | B.1.170 | GH |
| hCoV-19/Egypt/NRC-6373/2020 | EPI_ISL_2232311 | 27/07/2020 | Africa / Egypt | Human | unknown | Female | unknown | unknown | unknown | unknown | C.17 | GR |
| hCoV-19/Egypt/ARMY-282/2021 | EPI_ISL_1936133 | 02/05/2021 | Africa / Egypt / Cairo | Human | unknown | unknown | 25-55 | Hospitalized | unknown | unknown | B | L |
| hCoV-19/Egypt/ARMY-48/2021 | EPI_ISL_1936224 | 27/03/2021 | Africa / Egypt / Cairo | Human | unknown | unknown | 25-55 | Hospitalized | unknown | unknown | B | L |
| hCoV-19/Egypt/ARMY-40/2021 | EPI_ISL_1936215 | 27/03/2021 | Africa / Egypt / Cairo | Human | unknown | unknown | 25-55 | Hospitalized | unknown | unknown | C.17 | GR |
| hCoV-19/Egypt/PHARCO-ARMY-50/2021 | EPI_ISL_1936310 | 12/02/2021 | Africa / Egypt / Cairo | Human | unknown | unknown | 25-55 | Hospitalized | unknown | unknown | B.1 | G |
| hCoV-19/Egypt/PHARCO-ARMY-84/2021 | EPI_ISL_1936368 | 12/02/2021 | Africa / Egypt / Cairo | Human | unknown | unknown | 25-55 | Hospitalized | unknown | unknown | C.36 | GR |
| hCoV-19/Egypt/CUNCI-HGC12I022/2021 | EPI_ISL_890209 | 09/01/2021 | Africa / Egypt | Human | unknown | unknown | unknown | unknown | unknown | unknown | C.36 | GR |
| hCoV-19/Egypt/ARMY-28/2021 | EPI_ISL_1936202 | 27/03/2021 | Africa / Egypt / Cairo | Human | unknown | unknown | 25-55 | Hospitalized | unknown | unknown | B.1.1.7 | GR |
| hCoV-19/Egypt/ARMY-33/2021 | EPI_ISL_1936207 | 27/03/2021 | Africa / Egypt / Cairo | Human | unknown | unknown | 25-55 | Hospitalized | unknown | unknown | C.17 | GR |
| hCoV-19/Egypt/PHARCO-ARMY-37/2021 | EPI_ISL_1936297 | 12/02/2021 | Africa / Egypt / Cairo | Human | unknown | unknown | 25-55 | Hospitalized | unknown | unknown | C.36 | GR |
| hCoV-19/Egypt/ARMY-26/2021 | EPI_ISL_1936200 | 27/03/2021 | Africa / Egypt / Cairo | Human | unknown | unknown | 25-55 | Hospitalized | unknown | unknown | B.1.1.7 | GR |
| hCoV-19/Egypt/ARMY-355/2021 | EPI_ISL_1936285 | 04/04/2021 | Africa / Egypt / Cairo | Human | unknown | unknown | 25-60 | Hospitalized | unknown | unknown | B.1.1.7 | GR |
| hCoV-19/Egypt/ARMY-59/2021 | EPI_ISL_1936235 | 27/03/2021 | Africa / Egypt / Cairo | Human | unknown | unknown | 25-55 | Hospitalized | unknown | unknown | B.1 | GH |
| hCoV-19/Egypt/ARMY-352/2021 | EPI_ISL_1936282 | 04/04/2021 | Africa / Egypt / Cairo | Human | unknown | unknown | 25-60 | Hospitalized | unknown | unknown | B | L |
| hCoV-19/Egypt/CCHE57357-A-54/2020 | EPI_ISL_812827 | 19/07/2020 | Africa / Egypt | Human | unknown | unknown | unknown | unknown | unknown | unknown | B | O |
| hCoV-19/Egypt/ARMY-228/2021 | EPI_ISL_1936107 | 02/05/2021 | Africa / Egypt / Cairo | Human | unknown | unknown | 25-55 | Hospitalized | unknown | unknown | B.1.170 | GH |
| hCoV-19/Egypt/NRC-5771/2020 | EPI_ISL_8193867 | 13/06/2020 | Africa / Egypt | Human | unknown | unknown | unknown | unknown | unknown | unknown | B.1.78 | G |
| hCoV-19/Egypt/ARMY-350/2021 | EPI_ISL_1936280 | 04/04/2021 | Africa / Egypt / Cairo | Human | unknown | unknown | 25-60 | Hospitalized | unknown | unknown | B | L |
| hCoV-19/Egypt/NRC-1001/2021 | EPI_ISL_8215698 | 20/09/2021 | Africa / Egypt | Human | unknown | Male | unknown | unknown | unknown | unknown | B.1.617.2 | GK |
| hCoV-19/Egypt/CCHE57357-P-25/2020 | EPI_ISL_812859 | 16/07/2020 | Africa / Egypt | Human | unknown | unknown | unknown | unknown | unknown | unknown | B | L |
| hCoV-19/Egypt/NRC-5755/2020 | EPI_ISL_8193868 | 10/06/2020 | Africa / Egypt | Human | unknown | unknown | unknown | unknown | unknown | unknown | B.1.170 | GH |
| hCoV-19/Egypt/NRC-6453/2020 | EPI_ISL_8194868 | 17/08/2020 | Africa / Egypt | Human | unknown | unknown | unknown | unknown | unknown | unknown | B.1.533 | GH |
| hCoV-19/Egypt/NRC-5574OP/2020 | EPI_ISL_8189342 | 03/06/2020 | Africa / Egypt | Human | unknown | unknown | unknown | unknown | unknown | unknown | C.17 | GR |
| hCoV-19/Egypt/NRC-628/2021 | EPI_ISL_8215719 | 10/05/2021 | Africa / Egypt | Human | unknown | unknown | unknown | unknown | unknown | unknown | C.17 | GR |
| hCoV-19/Egypt/CPHL-A3/2021 | EPI_ISL_3274159 | 01/08/2021 | Africa / Egypt / Cairo | Human | Sentinel surveillance (ILI) | Male | 21 | unknown | unknown | Sentinel surveillance (ILI) | B.1.1 | GR |
| hCoV-19/Egypt/CPHL-A5/2021 | EPI_ISL_3274161 | 02/03/2021 | Africa / Egypt / Giza | Human | Sentinel surveillance (ILI) | Female | 34 | unknown | unknown | Sentinel surveillance (ILI) | C.36 | GR |
| hCoV-19/Egypt/CPHL-A2/2021 | EPI_ISL_3274158 | 18/01/2021 | Africa / Egypt / Cairo | Human | Sentinel surveillance (ILI) | Male | 25 | unknown | unknown | Sentinel surveillance (ILI) | C.36 | GR |
| hCoV-19/Egypt/ARMY-RLEUH-Omi058/2021 | EPI_ISL_7952324 | 09/12/2021 | Africa / Egypt / Suez | Human | unknown | Male | 67 | Hospitalized | Sinopharm | unknown | BA.1 | GRA |
| hCoV-19/Egypt/NRC-5754/2020 | EPI_ISL_8193869 | 10/06/2020 | Africa / Egypt | Human | unknown | unknown | unknown | unknown | unknown | unknown | B.1.170 | GH |
| hCoV-19/Egypt/ARMY-35/2021 | EPI_ISL_1936210 | 27/03/2021 | Africa / Egypt / Cairo | Human | unknown | unknown | 25-55 | Hospitalized | unknown | unknown | C.17 | GR |
| hCoV-19/Egypt/CCHE57357-A-28/2020 | EPI_ISL_812804 | 04/07/2020 | Africa / Egypt | Human | unknown | unknown | unknown | unknown | unknown | unknown | B | L |
| hCoV-19/Egypt/ARMY-241/2021 | EPI_ISL_1936106 | 02/05/2021 | Africa / Egypt / Cairo | Human | unknown | unknown | 25-55 | Hospitalized | unknown | unknown | B.1.170 | GH |
| hCoV-19/Egypt/ARMY-219/2021 | EPI_ISL_1936105 | 02/05/2021 | Africa / Egypt / Cairo | Human | unknown | unknown | 25-55 | Hospitalized | unknown | unknown | B.1.170 | GH |
| hCoV-19/Egypt/NRC-6072/2020 | EPI_ISL_8193609 | 27/06/2020 | Africa / Egypt | Human | unknown | unknown | unknown | unknown | unknown | unknown | B.1.195 | G |
| hCoV-19/Egypt/NRC-5466NS/2020 | EPI_ISL_8193626 | 30/05/2020 | Africa / Egypt | Human | unknown | unknown | unknown | unknown | unknown | unknown | B.1.1 | GR |
| hCoV-19/Egypt/NRC-5520/2020 | EPI_ISL_2232404 | 01/06/2020 | Africa / Egypt | Human | unknown | unknown | unknown | unknown | unknown | unknown | B.1.1 | GR |
| hCoV-19/Egypt/NRC-5450OP/2020 | EPI_ISL_8193875 | 27/05/2020 | Africa / Egypt | Human | unknown | unknown | unknown | unknown | unknown | unknown | B.1.170 | G |
| hCoV-19/Egypt/ARMY-402/2021 | EPI_ISL_1969080 | 24/04/2021 | Africa / Egypt / Cairo | Human | unknown | unknown | 45 | Hospitalized | unknown | unknown | B.1.1.7 | GR |
| hCoV-19/Egypt/NRC-6333/2020 | EPI_ISL_8193857 | 15/07/2020 | Africa / Egypt | Human | unknown | unknown | unknown | unknown | unknown | unknown | B.1.170 | GH |
| hCoV-19/Egypt/NRC-6187/2020 | EPI_ISL_8193884 | 01/07/2020 | Africa / Egypt | Human | unknown | unknown | unknown | unknown | unknown | unknown | B.1.170 | GH |
| hCoV-19/Egypt/NRC-5586/2020 | EPI_ISL_2232316 | 04/06/2020 | Africa / Egypt | Human | unknown | Female | unknown | unknown | unknown | unknown | B.1 | GH |
| hCoV-19/Egypt/CCHE57357-A-44/2020 | EPI_ISL_812817 | 12/06/2020 | Africa / Egypt | Human | unknown | unknown | unknown | unknown | unknown | unknown | C.36 | GR |
| hCoV-19/Egypt/NRC-6036/2020 | EPI_ISL_8193616 | 24/06/2020 | Africa / Egypt | Human | unknown | unknown | unknown | unknown | unknown | unknown | B.1.1 | G |
| hCoV-19/Egypt/ARMY-255/2021 | EPI_ISL_1936117 | 02/05/2021 | Africa / Egypt / Cairo | Human | unknown | unknown | 25-55 | Hospitalized | unknown | unknown | C.17 | GR |
| hCoV-19/Egypt/NRC-6828/2020 | EPI_ISL_2232307 | 30/09/2020 | Africa / Egypt | Human | unknown | Female | unknown | unknown | unknown | unknown | B.1 | GH |
| hCoV-19/Egypt/CUNCI-HGC12I023/2021 | EPI_ISL_890210 | 09/01/2021 | Africa / Egypt | Human | unknown | unknown | unknown | unknown | unknown | unknown | C.36 | GR |
| hCoV-19/Egypt/PHARCO-ARMY-51/2021 | EPI_ISL_1936311 | 12/02/2021 | Africa / Egypt / Cairo | Human | unknown | unknown | 25-55 | Hospitalized | unknown | unknown | C.36 | GR |
| hCoV-19/Egypt/ARMY-291/2021 | EPI_ISL_1936115 | 02/05/2021 | Africa / Egypt / Cairo | Human | unknown | unknown | 25-55 | Hospitalized | unknown | unknown | C.17 | GR |
| hCoV-19/Egypt/NRC-5632/2020 | EPI_ISL_8194877 | 06/06/2020 | Africa / Egypt | Human | unknown | unknown | unknown | unknown | unknown | unknown | B.1.170 | GH |
| hCoV-19/Egypt/NRC-603/2021 | EPI_ISL_8251488 | 09/05/2021 | Africa / Egypt | Human | unknown | unknown | unknown | unknown | unknown | unknown | C.36.3 | GR |
| hCoV-19/Egypt/NRC-5445OP/2020 | EPI_ISL_8193877 | 27/05/2020 | Africa / Egypt | Human | unknown | unknown | unknown | unknown | unknown | unknown | C.17 | GR |
| hCoV-19/Egypt/ARMY-54/2021 | EPI_ISL_1936230 | 27/03/2021 | Africa / Egypt / Cairo | Human | unknown | unknown | 25-55 | Hospitalized | unknown | unknown | C.17 | GR |
| hCoV-19/Egypt/CCHE57357-A-59/2020 | EPI_ISL_812832 | 12/07/2020 | Africa / Egypt | Human | unknown | unknown | unknown | unknown | unknown | unknown | B.1 | GH |
| hCoV-19/Egypt/PHARCO-ARMY-73/2021 | EPI_ISL_1936333 | 12/02/2021 | Africa / Egypt / Cairo | Human | unknown | unknown | 25-55 | Hospitalized | unknown | unknown | C.36 | GR |
| hCoV-19/Egypt/NRC-308/2021 | EPI_ISL_2227336 | 10/03/2021 | Africa / Egypt | Human | unknown | Male | unknown | unknown | unknown | unknown | C.17 | GR |
| hCoV-19/Egypt/ARMY-207/2021 | EPI_ISL_1936139 | 02/05/2021 | Africa / Egypt / Cairo | Human | unknown | unknown | 25-55 | Hospitalized | unknown | unknown | C.17 | GR |
| hCoV-19/Egypt/NRC-6004/2020 | EPI_ISL_2232339 | 23/06/2020 | Africa / Egypt | Human | unknown | Male | unknown | unknown | unknown | unknown | B.1 | G |
| hCoV-19/Egypt/NRC-5551NS/2020 | EPI_ISL_8189340 | 03/06/2020 | Africa / Egypt | Human | unknown | unknown | unknown | unknown | unknown | unknown | B.1.170 | GH |
| hCoV-19/Egypt/NRC-6559/2020 | EPI_ISL_8194866 | 29/08/2020 | Africa / Egypt | Human | unknown | unknown | unknown | unknown | unknown | unknown | B.1.1.1 | GR |
| hCoV-19/Egypt/NRC-314/2021 | EPI_ISL_2227346 | 10/03/2021 | Africa / Egypt | Human | unknown | Male | unknown | unknown | unknown | unknown | C.36.3 | GR |
| hCoV-19/Egypt/NRC-5581OP/2020 | EPI_ISL_8193658 | 03/06/2020 | Africa / Egypt | Human | unknown | unknown | unknown | unknown | unknown | unknown | B.1.1 | GR |
| hCoV-19/Egypt/CCHE57357-P-03/2020 | EPI_ISL_812851 | 11/08/2020 | Africa / Egypt | Human | unknown | unknown | unknown | unknown | unknown | unknown | B.1 | GH |
| hCoV-19/Egypt/CPHL-S5/2021 | EPI_ISL_3274152 | 29/05/2021 | Africa / Egypt / Cairo | Human | Sentinel surveillance (ILI) | Male | 45 | unknown | unknown | Sentinel surveillance (ILI) | C.36.3 | GR |
| hCoV-19/Egypt/CPHL-NRC-S7/2021 | EPI_ISL_2313062 | 2021-03 | Africa / Egypt | Human | unknown | unknown | unknown | unknown | unknown | unknown | C.36.3 | GR |
| hCoV-19/Egypt/NRC-5853/2020 | EPI_ISL_2232321 | 17/06/2020 | Africa / Egypt | Human | unknown | Female | unknown | unknown | unknown | unknown | C.17 | GR |
| hCoV-19/Egypt/ARMY-260/2021 | EPI_ISL_1936121 | 02/05/2021 | Africa / Egypt / Cairo | Human | unknown | unknown | 25-55 | Hospitalized | unknown | unknown | C.17 | GR |
| hCoV-19/Egypt/CUNCI-HGC9I010/2020 | EPI_ISL_857326 | 25/12/2020 | Africa / Egypt | Human | unknown | unknown | unknown | unknown | unknown | unknown | B.1.1.1 | GR |
| hCoV-19/Egypt/MASRI-C5-008/2020 | EPI_ISL_1109626 | 28/06/2020 | Africa / Egypt / Cairo | Human | unknown | Female | 13 | unknown | unknown | unknown | C.17 | GR |
| hCoV-19/Egypt/CCHE57357-P-41/2020 | EPI_ISL_812870 | 23/07/2020 | Africa / Egypt | Human | unknown | unknown | unknown | unknown | unknown | unknown | B | L |
| hCoV-19/Egypt/PHARCO-ARMY-88/2021 | EPI_ISL_1936372 | 12/02/2021 | Africa / Egypt / Cairo | Human | unknown | unknown | 25-55 | Hospitalized | unknown | unknown | B.1 | GH |
| hCoV-19/Egypt/CCHE57357-A-02/2020 | EPI_ISL_812784 | 16/05/2020 | Africa / Egypt | Human | unknown | unknown | unknown | unknown | unknown | unknown | B.1 | G |
| hCoV-19/Egypt/NRC-1151/2021 | EPI_ISL_8205830 | 17/10/2021 | Africa / Egypt | Human | unknown | Male | unknown | unknown | unknown | unknown | B.1.617.2 | GK |
| hCoV-19/Egypt/CUNCI-HGC09I039/2020 | EPI_ISL_907096 | 25/12/2020 | Africa / Egypt | Human | unknown | unknown | unknown | unknown | unknown | unknown | A.28 | S |
| hCoV-19/Egypt/NRC-6157/2020 | EPI_ISL_2232382 | 30/06/2020 | Africa / Egypt | Human | unknown | unknown | unknown | unknown | unknown | unknown | B.1.1.1 | GR |
| hCoV-19/Egypt/NRC-5443OP/2020 | EPI_ISL_8193878 | 27/05/2020 | Africa / Egypt | Human | unknown | unknown | unknown | unknown | unknown | unknown | B.1.1 | GR |
| hCoV-19/Egypt/NRC-5918/2020 | EPI_ISL_2380082 | 19/06/2020 | Africa / Egypt | Human | unknown | unknown | unknown | unknown | unknown | unknown | B.1 | GH |
| hCoV-19/Egypt/ARMY-329/2021 | EPI_ISL_1936266 | 22/04/2021 | Africa / Egypt / Cairo | Human | unknown | unknown | 25-60 | Hospitalized | unknown | unknown | C.36.3 | GR |
| hCoV-19/Egypt/CUNCI-HGC12I035/2021 | EPI_ISL_890218 | 09/01/2021 | Africa / Egypt | Human | unknown | unknown | unknown | unknown | unknown | unknown | B.1 | G |
| hCoV-19/Egypt/NRC-5531/2020 | EPI_ISL_8189544 | 02/06/2020 | Africa / Egypt | Human | unknown | unknown | unknown | unknown | unknown | unknown | B.1.170 | GH |
| hCoV-19/Egypt/NRC-5525/2020 | EPI_ISL_8189545 | 01/06/2020 | Africa / Egypt | Human | unknown | unknown | unknown | unknown | unknown | unknown | B.1.170 | GH |
| hCoV-19/Egypt/NRC-5795/2020 | EPI_ISL_8189548 | 14/06/2020 | Africa / Egypt | Human | unknown | unknown | unknown | unknown | unknown | unknown | B.1.1.372 | GR |
| hCoV-19/Egypt/NRC-5930/2020 | EPI_ISL_8189553 | 21/06/2020 | Africa / Egypt | Human | unknown | unknown | unknown | unknown | unknown | unknown | C.17 | GR |
| hCoV-19/Egypt/NRC-6354/2020 | EPI_ISL_8189308 | 26/07/2020 | Africa / Egypt | Human | unknown | unknown | unknown | unknown | unknown | unknown | B.1 | GH |
| hCoV-19/Egypt/NRC-6363/2020 | EPI_ISL_8189307 | 22/07/2020 | Africa / Egypt | Human | unknown | unknown | unknown | unknown | unknown | unknown | B.1 | GH |
| hCoV-19/Egypt/NRC-7332/2020 | EPI_ISL_8189344 | 13/12/2020 | Africa / Egypt | Human | unknown | unknown | unknown | unknown | unknown | unknown | B.1 | GH |
| hCoV-19/Egypt/NRC-6128/2020 | EPI_ISL_8189343 | 28/06/2020 | Africa / Egypt | Human | unknown | unknown | unknown | unknown | unknown | unknown | C.17 | GR |
| hCoV-19/Egypt/NRC-6283/2020 | EPI_ISL_8193882 | 07/07/2020 | Africa / Egypt | Human | unknown | unknown | unknown | unknown | unknown | unknown | B.1.533 | GH |
| hCoV-19/Egypt/NRC-6334/2020 | EPI_ISL_8193856 | 18/07/2020 | Africa / Egypt | Human | unknown | unknown | unknown | unknown | unknown | unknown | B.1.170 | GH |
| hCoV-19/Egypt/NRC-6332/2020 | EPI_ISL_8193858 | 15/07/2020 | Africa / Egypt | Human | unknown | unknown | unknown | unknown | unknown | unknown | B.1.170 | GH |
| hCoV-19/Egypt/NRC-6336/2020 | EPI_ISL_8193852 | 18/07/2020 | Africa / Egypt | Human | unknown | unknown | unknown | unknown | unknown | unknown | C.17 | GR |
| hCoV-19/Egypt/NRC-6446/2020 | EPI_ISL_8193855 | 14/08/2020 | Africa / Egypt | Human | unknown | unknown | unknown | unknown | unknown | unknown | B.1.170 | GH |
| hCoV-19/Egypt/NRC-5575OP/2020 | EPI_ISL_8193659 | 03/06/2020 | Africa / Egypt | Human | unknown | unknown | unknown | unknown | unknown | unknown | C.17 | GR |
| hCoV-19/Egypt/NRC-5556NS/2020 | EPI_ISL_8193655 | 03/06/2020 | Africa / Egypt | Human | unknown | unknown | unknown | unknown | unknown | unknown | C.17 | GR |
| hCoV-19/Egypt/NRC-5558NS/2020 | EPI_ISL_8193656 | 03/06/2020 | Africa / Egypt | Human | unknown | unknown | unknown | unknown | unknown | unknown | C.17 | GR |
| hCoV-19/Egypt/NRC-7306/2020 | EPI_ISL_8193638 | 15/12/2020 | Africa / Egypt | Human | unknown | unknown | unknown | unknown | unknown | unknown | C.17 | G |
| hCoV-19/Egypt/NRC-5438NS/2020 | EPI_ISL_8193630 | 26/05/2020 | Africa / Egypt | Human | unknown | unknown | unknown | unknown | unknown | unknown | B.1 | GH |
| hCoV-19/Egypt/NRC-7314/2020 | EPI_ISL_8193635 | 15/12/2020 | Africa / Egypt | Human | unknown | unknown | unknown | unknown | unknown | unknown | B.1 | GH |
| hCoV-19/Egypt/NRC-6509/2020 | EPI_ISL_8193649 | 23/08/2020 | Africa / Egypt | Human | unknown | unknown | unknown | unknown | unknown | unknown | B.1.1 | G |
| hCoV-19/Egypt/NRC-7297/2020 | EPI_ISL_8193640 | 15/12/2020 | Africa / Egypt | Human | unknown | unknown | unknown | unknown | unknown | unknown | C.32 | G |
| hCoV-19/Egypt/NRC-7292/2020 | EPI_ISL_8193641 | 14/12/2020 | Africa / Egypt | Human | unknown | unknown | unknown | unknown | unknown | unknown | B.1 | G |
| hCoV-19/Egypt/NRC-7283/2020 | EPI_ISL_8193642 | 14/12/2020 | Africa / Egypt | Human | unknown | unknown | unknown | unknown | unknown | unknown | C.17 | GR |
| hCoV-19/Egypt/NRC-6612/2020 | EPI_ISL_8193645 | 07/09/2020 | Africa / Egypt | Human | unknown | unknown | unknown | unknown | unknown | unknown | C.17 | G |
| hCoV-19/Egypt/NRC-6043/2020 | EPI_ISL_8193615 | 24/06/2020 | Africa / Egypt | Human | unknown | unknown | unknown | unknown | unknown | unknown | B.1.1 | G |
| hCoV-19/Egypt/NRC-6398/2020 | EPI_ISL_8193619 | 04/08/2020 | Africa / Egypt | Human | unknown | unknown | unknown | unknown | unknown | unknown | B.1.170 | GH |
| hCoV-19/Egypt/NRC-6063/2020 | EPI_ISL_8193610 | 24/06/2020 | Africa / Egypt | Human | unknown | unknown | unknown | unknown | unknown | unknown | B.1.1.1 | GR |
| hCoV-19/Egypt/NRC-6057/2020 | EPI_ISL_8193611 | 24/06/2020 | Africa / Egypt | Human | unknown | unknown | unknown | unknown | unknown | unknown | B.1.1.1 | GH |
| hCoV-19/Egypt/NRC-6049/2020 | EPI_ISL_8193613 | 24/06/2020 | Africa / Egypt | Human | unknown | unknown | unknown | unknown | unknown | unknown | B.1 | GH |
| hCoV-19/Egypt/NRC-5486/2020 | EPI_ISL_8193625 | 30/05/2020 | Africa / Egypt | Human | unknown | unknown | unknown | unknown | unknown | unknown | B.1.1.1 | GH |
| hCoV-19/Egypt/NRC-6725/2020 | EPI_ISL_8193629 | 18/09/2020 | Africa / Egypt | Human | unknown | unknown | unknown | unknown | unknown | unknown | B.1 | G |
| hCoV-19/Egypt/NRC-6379/2020 | EPI_ISL_8193620 | 28/07/2020 | Africa / Egypt | Human | unknown | unknown | unknown | unknown | unknown | unknown | B.1 | G |
| hCoV-19/Egypt/NRC-6390/2020 | EPI_ISL_8193621 | 01/08/2020 | Africa / Egypt | Human | unknown | unknown | unknown | unknown | unknown | unknown | B.1.436 | GH |
| hCoV-19/Egypt/NRC-5538OP/2020 | EPI_ISL_8193623 | 03/06/2020 | Africa / Egypt | Human | unknown | unknown | unknown | unknown | unknown | unknown | B.1 | GH |
| hCoV-19/Egypt/NRC-5521NS/2020 | EPI_ISL_8193624 | 01/06/2020 | Africa / Egypt | Human | unknown | unknown | unknown | unknown | unknown | unknown | B.1.195 | G |
| hCoV-19/Egypt/NRC-6129/2020 | EPI_ISL_8193603 | 28/06/2020 | Africa / Egypt | Human | unknown | unknown | unknown | unknown | unknown | unknown | B.1.1 | GR |
| hCoV-19/Egypt/NRC-6109/2020 | EPI_ISL_8193604 | 28/06/2020 | Africa / Egypt | Human | unknown | unknown | unknown | unknown | unknown | unknown | B.1.195 | G |
| hCoV-19/Egypt/NRC-6645/2020 | EPI_ISL_8193600 | 10/09/2020 | Africa / Egypt | Human | unknown | unknown | unknown | unknown | unknown | unknown | B.1 | G |
| hCoV-19/Egypt/NRC-6641/2020 | EPI_ISL_8193601 | 10/09/2020 | Africa / Egypt | Human | unknown | unknown | unknown | unknown | unknown | unknown | B.1.208 | G |
| hCoV-19/Egypt/NRC-6040/2020 | EPI_ISL_8193602 | 24/06/2020 | Africa / Egypt | Human | unknown | unknown | unknown | unknown | unknown | unknown | B.1.1.1 | G |
| hCoV-19/Egypt/NRC-6660/2020 | EPI_ISL_8193596 | 10/09/2020 | Africa / Egypt | Human | unknown | unknown | unknown | unknown | unknown | unknown | B.1 | G |
| hCoV-19/Egypt/NRC-5551OP/2020 | EPI_ISL_8193585 | 03/06/2020 | Africa / Egypt | Human | unknown | unknown | unknown | unknown | unknown | unknown | C.32 | G |
| hCoV-19/Egypt/NRC-5550OP/2020 | EPI_ISL_8193586 | 03/06/2020 | Africa / Egypt | Human | unknown | unknown | unknown | unknown | unknown | unknown | B.1.1.1 | GH |
| hCoV-19/Egypt/NRC-7076/2020 | EPI_ISL_8193588 | 06/11/2020 | Africa / Egypt | Human | unknown | unknown | unknown | unknown | unknown | unknown | B.1.170 | GH |
| hCoV-19/Egypt/NRC-6353/2020 | EPI_ISL_8194883 | 26/07/2020 | Africa / Egypt | Human | unknown | unknown | unknown | unknown | unknown | unknown | B.1 | GH |
| hCoV-19/Egypt/NRC-6701/2020 | EPI_ISL_8194870 | 15/09/2020 | Africa / Egypt | Human | unknown | unknown | unknown | unknown | unknown | unknown | B.1 | G |
| hCoV-19/Egypt/NRC-6359/2020 | EPI_ISL_8194869 | 22/07/2020 | Africa / Egypt | Human | unknown | unknown | unknown | unknown | unknown | unknown | B.1.533 | GH |
| hCoV-19/Egypt/NRC-6064/2020 | EPI_ISL_8194865 | 24/06/2020 | Africa / Egypt | Human | unknown | unknown | unknown | unknown | unknown | unknown | B.1.1.1 | GR |
| hCoV-19/Egypt/NRC-5621/2020 | EPI_ISL_8194874 | 07/06/2020 | Africa / Egypt | Human | unknown | unknown | unknown | unknown | unknown | unknown | B.1 | G |
| hCoV-19/Egypt/NRC-5949/2020 | EPI_ISL_8193919 | 20/06/2020 | Africa / Egypt | Human | unknown | unknown | unknown | unknown | unknown | unknown | B.1.170 | GH |
| hCoV-19/Egypt/NRC-5965/2020 | EPI_ISL_8193911 | 21/06/2020 | Africa / Egypt | Human | unknown | unknown | unknown | unknown | unknown | unknown | B.1.170 | GH |
| hCoV-19/Egypt/NRC-5964/2020 | EPI_ISL_8193912 | 21/06/2020 | Africa / Egypt | Human | unknown | unknown | unknown | unknown | unknown | unknown | B.1 | GH |
| hCoV-19/Egypt/NRC-5962/2020 | EPI_ISL_8193913 | 21/06/2020 | Africa / Egypt | Human | unknown | unknown | unknown | unknown | unknown | unknown | B.1 | GH |
| hCoV-19/Egypt/NRC-5961/2020 | EPI_ISL_8193914 | 21/06/2020 | Africa / Egypt | Human | unknown | unknown | unknown | unknown | unknown | unknown | B.1.1 | GR |
| hCoV-19/Egypt/NRC-5952/2020 | EPI_ISL_8193917 | 20/06/2020 | Africa / Egypt | Human | unknown | unknown | unknown | unknown | unknown | unknown | B.1.170 | GH |
| hCoV-19/Egypt/NRC-5950/2020 | EPI_ISL_8193918 | 20/06/2020 | Africa / Egypt | Human | unknown | unknown | unknown | unknown | unknown | unknown | B.1.1 | GR |
| hCoV-19/Egypt/NRC-5938/2020 | EPI_ISL_8193924 | 21/06/2020 | Africa / Egypt | Human | unknown | unknown | unknown | unknown | unknown | unknown | B.1 | GH |
| hCoV-19/Egypt/NRC-5936/2020 | EPI_ISL_8193925 | 21/06/2020 | Africa / Egypt | Human | unknown | unknown | unknown | unknown | unknown | unknown | B.1 | GH |
| hCoV-19/Egypt/NRC-5944/2020 | EPI_ISL_8193920 | 21/06/2020 | Africa / Egypt | Human | unknown | unknown | unknown | unknown | unknown | unknown | B.1.1 | GR |
| hCoV-19/Egypt/NRC-6006/2020 | EPI_ISL_8193909 | 23/06/2020 | Africa / Egypt | Human | unknown | unknown | unknown | unknown | unknown | unknown | B.1 | G |
| hCoV-19/Egypt/NRC-6538/2020 | EPI_ISL_8193903 | 30/08/2020 | Africa / Egypt | Human | unknown | unknown | unknown | unknown | unknown | unknown | C.36.3 | GH |
| hCoV-19/Egypt/NRC-6031/2020 | EPI_ISL_8193907 | 23/06/2020 | Africa / Egypt | Human | unknown | unknown | unknown | unknown | unknown | unknown | B.1 | GH |
| hCoV-19/Egypt/NRC-6171/2020 | EPI_ISL_8193893 | 01/07/2020 | Africa / Egypt | Human | unknown | unknown | unknown | unknown | unknown | unknown | B.1.1 | GH |
| hCoV-19/Egypt/NRC-6135/2020 | EPI_ISL_8193895 | 29/06/2020 | Africa / Egypt | Human | unknown | unknown | unknown | unknown | unknown | unknown | B.1 | G |
| hCoV-19/Egypt/CCHE57357-A-58/2020 | EPI_ISL_812831 | 23/07/2020 | Africa / Egypt | Human | unknown | unknown | unknown | unknown | unknown | unknown | B | L |
| hCoV-19/Egypt/ARMY-333/2021 | EPI_ISL_1936270 | 22/04/2021 | Africa / Egypt / Cairo | Human | unknown | unknown | 25-60 | Hospitalized | unknown | unknown | C.36.3 | GR |
| hCoV-19/Egypt/NRC-1246/2021 | EPI_ISL_8205815 | 04/11/2021 | Africa / Egypt | Human | unknown | Female | unknown | unknown | unknown | unknown | B.1.617.2 | GK |
| hCoV-19/Egypt/NRC-1218/2021 | EPI_ISL_8205814 | 28/10/2021 | Africa / Egypt | Human | unknown | Male | unknown | unknown | unknown | unknown | B.1.617.2 | GK |
| hCoV-19/Egypt/NRC-1292/2021 | EPI_ISL_8205819 | 11/11/2021 | Africa / Egypt | Human | unknown | unknown | unknown | unknown | unknown | unknown | B.1.617.2 | GK |
| hCoV-19/Egypt/NRC-1289/2021 | EPI_ISL_8205818 | 11/11/2021 | Africa / Egypt | Human | unknown | unknown | unknown | unknown | unknown | unknown | B.1.617.2 | GK |
| hCoV-19/Egypt/NRC-1248/2021 | EPI_ISL_8205817 | 04/11/2021 | Africa / Egypt | Human | unknown | Male | unknown | unknown | unknown | unknown | B.1.617.2 | GK |
| hCoV-19/Egypt/NRC-1247/2021 | EPI_ISL_8205816 | 04/11/2021 | Africa / Egypt | Human | unknown | Female | unknown | unknown | unknown | unknown | B.1.617.2 | GK |
| hCoV-19/Egypt/NRC-1229/2021 | EPI_ISL_8205825 | 02/11/2021 | Africa / Egypt | Human | unknown | Female | unknown | unknown | unknown | unknown | B.1.617.2 | GK |
| hCoV-19/Egypt/NRC-1245/2021 | EPI_ISL_8205824 | 04/11/2021 | Africa / Egypt | Human | unknown | Male | unknown | unknown | unknown | unknown | B.1.617.2 | GK |
| hCoV-19/Egypt/NRC-1261/2021 | EPI_ISL_8205823 | 07/11/2021 | Africa / Egypt | Human | unknown | Male | unknown | unknown | unknown | unknown | B.1.617.2 | GK |
| hCoV-19/Egypt/NRC-1324/2021 | EPI_ISL_8205821 | 17/11/2021 | Africa / Egypt | Human | unknown | Male | unknown | unknown | unknown | unknown | B.1.617.2 | GK |
| hCoV-19/Egypt/NRC-1293/2021 | EPI_ISL_8205820 | 11/11/2021 | Africa / Egypt | Human | unknown | unknown | unknown | unknown | unknown | unknown | B.1.617.2 | GK |
| hCoV-19/Egypt/NRC-1341/2021 | EPI_ISL_8205829 | 28/11/2021 | Africa / Egypt | Human | unknown | Male | unknown | unknown | unknown | unknown | B.1.617.2 | GK |
| hCoV-19/Egypt/NRC-1323/2021 | EPI_ISL_8205828 | 17/11/2021 | Africa / Egypt | Human | unknown | Female | unknown | unknown | unknown | unknown | B.1.617.2 | GK |
| hCoV-19/Egypt/NRC-1316/2021 | EPI_ISL_8205827 | 16/11/2021 | Africa / Egypt | Human | unknown | Female | unknown | unknown | unknown | unknown | B.1.617.2 | GK |
| hCoV-19/Egypt/NRC-1230/2021 | EPI_ISL_8205833 | 02/11/2021 | Africa / Egypt | Human | unknown | Female | unknown | unknown | unknown | unknown | B.1.617.2 | GK |
| hCoV-19/Egypt/NRC-1328/2021 | EPI_ISL_8205832 | 18/11/2021 | Africa / Egypt | Human | unknown | Female | unknown | unknown | unknown | unknown | B.1.617.2 | GK |
| hCoV-19/Egypt/PHARCO-ARMY-87/2021 | EPI_ISL_1936371 | 12/02/2021 | Africa / Egypt / Cairo | Human | unknown | unknown | 25-55 | Hospitalized | unknown | unknown | C.36 | GR |
| hCoV-19/Egypt/NRC-6217/2020 | EPI_ISL_2232371 | 04/07/2020 | Africa / Egypt | Human | unknown | unknown | unknown | unknown | unknown | unknown | C.17 | GR |
| hCoV-19/Egypt/NRC-1291/2021 | EPI_ISL_8207956 | 11/11/2021 | Africa / Egypt | Human | unknown | unknown | unknown | unknown | unknown | unknown | B.1.617.2 | GK |
| hCoV-19/Egypt/NRC-1044/2021 | EPI_ISL_8215702 | 30/09/2021 | Africa / Egypt | Human | unknown | Male | unknown | unknown | unknown | unknown | AY.112 | GK |
| hCoV-19/Egypt/NRC-1039/2021 | EPI_ISL_8215701 | 29/09/2021 | Africa / Egypt | Human | unknown | Female | unknown | unknown | unknown | unknown | B.1.617.2 | GK |
| hCoV-19/Egypt/NRC-1055/2021 | EPI_ISL_8215704 | 03/10/2021 | Africa / Egypt | Human | unknown | Female | unknown | unknown | unknown | unknown | B.1.617.2 | GK |
| hCoV-19/Egypt/NRC-1045/2021 | EPI_ISL_8215703 | 30/09/2021 | Africa / Egypt | Human | unknown | Female | unknown | unknown | unknown | unknown | B.1.617.2 | GK |
| hCoV-19/Egypt/NRC-1037/2021 | EPI_ISL_8215700 | 29/09/2021 | Africa / Egypt | Human | unknown | Male | unknown | unknown | unknown | unknown | B.1.617.2 | GK |
| hCoV-19/Egypt/NRC-1126/2021 | EPI_ISL_8215713 | 14/10/2021 | Africa / Egypt | Human | unknown | Male | unknown | unknown | unknown | unknown | B.1.617.2 | GK |
| hCoV-19/Egypt/NRC-1102/2021 | EPI_ISL_8215712 | 13/10/2021 | Africa / Egypt | Human | unknown | Male | unknown | unknown | unknown | unknown | B.1.617.2 | GK |
| hCoV-19/Egypt/NRC-1100/2021 | EPI_ISL_8215711 | 13/10/2021 | Africa / Egypt | Human | unknown | Female | unknown | unknown | unknown | unknown | C.36.3 | GR |
| hCoV-19/Egypt/NRC-1098/2021 | EPI_ISL_8215709 | 13/10/2021 | Africa / Egypt | Human | unknown | Female | unknown | unknown | unknown | unknown | B.1.617.2 | GK |
| hCoV-19/Egypt/NRC-1095/2021 | EPI_ISL_8215708 | 12/10/2021 | Africa / Egypt | Human | unknown | Male | unknown | unknown | unknown | unknown | B.1.617.2 | GK |
| hCoV-19/Egypt/NRC-690/2021 | EPI_ISL_8215724 | 23/05/2021 | Africa / Egypt | Human | unknown | unknown | unknown | unknown | unknown | unknown | C.36.3 | G |
| hCoV-19/Egypt/NRC-681/2021 | EPI_ISL_8215723 | 20/05/2021 | Africa / Egypt | Human | unknown | unknown | unknown | unknown | unknown | unknown | C.36.3 | GR |
| hCoV-19/Egypt/NRC-791/2021 | EPI_ISL_8215726 | 13/06/2021 | Africa / Egypt | Human | unknown | unknown | unknown | unknown | unknown | unknown | C.36.3 | G |
| hCoV-19/Egypt/NRC-581/2021 | EPI_ISL_8215717 | 09/05/2021 | Africa / Egypt | Human | unknown | unknown | unknown | unknown | unknown | unknown | C.36.3 | GR |
| hCoV-19/Egypt/NRC-627/2021 | EPI_ISL_8215718 | 10/05/2021 | Africa / Egypt | Human | unknown | unknown | unknown | unknown | unknown | unknown | C.36.3 | GR |
| hCoV-19/Egypt/NRC-974/2021 | EPI_ISL_8215694 | 12/09/2021 | Africa / Egypt | Human | unknown | Female | unknown | unknown | unknown | unknown | B.1.617.2 | GK |
| hCoV-19/Egypt/NRC-989/2021 | EPI_ISL_8215696 | 15/09/2021 | Africa / Egypt | Human | unknown | Female | unknown | unknown | unknown | unknown | B.1.617.2 | GK |
| hCoV-19/Egypt/NRC-1437/2021 | EPI_ISL_8215691 | 03/10/2021 | Africa / Egypt | Human | unknown | Female | unknown | unknown | unknown | unknown | AY.127 | GK |
| hCoV-19/Egypt/NRC-5645/2020 | EPI_ISL_8194878 | 04/06/2020 | Africa / Egypt | Human | unknown | unknown | unknown | unknown | unknown | unknown | B.1.170 | GH |
| hCoV-19/Egypt/NRC-5641/2020 | EPI_ISL_2232374 | 07/06/2020 | Africa / Egypt | Human | unknown | unknown | unknown | unknown | unknown | unknown | B.1.170 | GH |
| hCoV-19/Egypt/NRC-6003/2020 | EPI_ISL_2380077 | 23/06/2020 | Africa / Egypt | Human | unknown | Male | unknown | unknown | unknown | unknown | C.17 | GR |
| hCoV-19/Egypt/NRC-652/2021 | EPI_ISL_8251497 | 18/05/2021 | Africa / Egypt | Human | unknown | unknown | unknown | unknown | unknown | unknown | B.1.1.7 | GR |
| hCoV-19/Egypt/NRC-617/2021 | EPI_ISL_8251491 | 10/05/2021 | Africa / Egypt | Human | unknown | unknown | unknown | unknown | unknown | unknown | B.1 | G |
| hCoV-19/Egypt/NRC-589/2021 | EPI_ISL_8251486 | 09/05/2021 | Africa / Egypt | Human | unknown | unknown | unknown | unknown | unknown | unknown | C.36.3 | GR |
| hCoV-19/Egypt/NRC-697/2021 | EPI_ISL_8251503 | 24/05/2021 | Africa / Egypt | Human | unknown | unknown | unknown | unknown | unknown | unknown | C.17 | G |
| hCoV-19/Egypt/NRC-704/2021 | EPI_ISL_8251505 | 24/05/2021 | Africa / Egypt | Human | unknown | unknown | unknown | unknown | unknown | unknown | C.36.3 | GR |
| hCoV-19/Egypt/NRC-772/2021 | EPI_ISL_8251506 | 07/06/2021 | Africa / Egypt | Human | unknown | unknown | unknown | unknown | unknown | unknown | C.36.3 | GR |
| hCoV-19/Egypt/NRC-682/2021 | EPI_ISL_8251501 | 20/05/2021 | Africa / Egypt | Human | unknown | unknown | unknown | unknown | unknown | unknown | B.1 | G |
| hCoV-19/Egypt/NRC-694/2021 | EPI_ISL_8251502 | 23/05/2021 | Africa / Egypt | Human | unknown | unknown | unknown | unknown | unknown | unknown | C.36.3 | G |
| hCoV-19/Egypt/NRC-731/2021 | EPI_ISL_8251507 | 31/05/2021 | Africa / Egypt | Human | unknown | unknown | unknown | unknown | unknown | unknown | C.17 | GR |
| hCoV-19/Egypt/NRC-7082/2021 | EPI_ISL_8251514 | 06/01/2021 | Africa / Egypt | Human | unknown | unknown | unknown | unknown | unknown | unknown | B.1 | GH |
| hCoV-19/Egypt/NRC-7088/2021 | EPI_ISL_8251513 | 06/01/2021 | Africa / Egypt | Human | unknown | unknown | unknown | unknown | unknown | unknown | B.1.170 | GH |
| hCoV-19/Egypt/CCHE57357_Wave_3_A017/2021 | EPI_ISL_2566477 | 03/05/2021 | Africa / Egypt | Human | unknown | unknown | unknown | unknown | unknown | unknown | C.38 | GR |
| hCoV-19/Egypt/ARMY-217/2021 | EPI_ISL_1936144 | 02/05/2021 | Africa / Egypt / Cairo | Human | unknown | unknown | 25-55 | Hospitalized | unknown | unknown | C.17 | GR |
| hCoV-19/Egypt/CPHL-S1/2021 | EPI_ISL_3274150 | 16/06/2021 | Africa / Egypt / South Sinai | Human | Sentinel surveillance (ILI) | Male | 56 | unknown | unknown | Sentinel surveillance (ILI) | Unassigned | GR |
| hCoV-19/Egypt/CPHL-A1/2021 | EPI_ISL_3262210 | 11/01/2021 | Africa / Egypt / Cairo | Human | unknown | Male | 23 | Released | unknown | unknown | C.36.3 | G |
| hCoV-19/Egypt/NRC-6290/2020 | EPI_ISL_8193879 | 07/07/2020 | Africa / Egypt | Human | unknown | unknown | unknown | unknown | unknown | unknown | B.1.1 | GR |
| hCoV-19/Egypt/CPHL-A10/2021 | EPI_ISL_3274165 | 26/03/2021 | Africa / Egypt / Cairo | Human | Sentinel surveillance (ILI) | Female | 38 | unknown | unknown | Sentinel surveillance (ILI) | A.28 | S |
| hCoV-19/Egypt/CPHL-A8/2020 | EPI_ISL_3274163 | 01/12/2020 | Africa / Egypt / South Sinai | Human | Sentinel surveillance (ILI) | Male | 39 | unknown | unknown | Sentinel surveillance (ILI) | C.17 | GR |
| hCoV-19/Egypt/CPHL-S4/2021 | EPI_ISL_3274151 | 16/06/2021 | Africa / Egypt / Cairo | Human | Sentinel surveillance (ILI) | Male | 35 | unknown | unknown | Sentinel surveillance (ILI) | C.36.3 | GR |
| hCoV-19/Egypt/CPHL-S8/2021 | EPI_ISL_3274155 | 02/07/2021 | Africa / Egypt / Cairo | Human | Sentinel surveillance (ILI) | Male | 24 | unknown | unknown | Sentinel surveillance (ILI) | B.1 | GH |
| hCoV-19/Egypt/CPHL-S6/2021 | EPI_ISL_3274153 | 05/06/2021 | Africa / Egypt / Red Sea | Human | Sentinel surveillance (ILI) | Female | 44 | unknown | unknown | Sentinel surveillance (ILI) | C.17 | GR |
| hCoV-19/Egypt/NRC-7340/2020 | EPI_ISL_8193633 | 16/12/2020 | Africa / Egypt | Human | unknown | unknown | unknown | unknown | unknown | unknown | B.1.170 | GH |
| hCoV-19/Egypt/CCHE57357_Wave_4_095/2021 | EPI_ISL_6011766 | 21/10/2021 | Africa / Egypt | Human | unknown | unknown | unknown | unknown | unknown | unknown | B.1.617.2 | GK |
| hCoV-19/Egypt/CCHE57357_Wave_4_134/2021 | EPI_ISL_6011805 | 18/10/2021 | Africa / Egypt | Human | unknown | unknown | unknown | unknown | unknown | unknown | AY.127 | GK |
| hCoV-19/Egypt/CCHE57357_Wave_4_128/2021 | EPI_ISL_6011799 | 17/10/2021 | Africa / Egypt | Human | unknown | unknown | unknown | unknown | unknown | unknown | AY.125 | GK |
| hCoV-19/Egypt/CCHE57357_Wave_4_113/2021 | EPI_ISL_6011784 | 19/10/2021 | Africa / Egypt | Human | unknown | unknown | unknown | unknown | unknown | unknown | B.1.617.2 | GK |
| hCoV-19/Egypt/CCHE57357_Wave_4_098/2021 | EPI_ISL_6011769 | 21/10/2021 | Africa / Egypt | Human | unknown | unknown | unknown | unknown | unknown | unknown | AY.122 | GK |
| hCoV-19/Egypt/NRC-5781/2020 | EPI_ISL_2232403 | 13/06/2020 | Africa / Egypt | Human | unknown | unknown | unknown | unknown | unknown | unknown | B.1.170 | GH |
| hCoV-19/Egypt/NRC-5879/2020 | EPI_ISL_2232391 | 17/06/2020 | Africa / Egypt | Human | unknown | unknown | unknown | unknown | unknown | unknown | B.1.170 | GH |
| hCoV-19/Egypt/CCHE57357-A-56/2020 | EPI_ISL_812829 | 29/06/2020 | Africa / Egypt | Human | unknown | unknown | unknown | unknown | unknown | unknown | B | O |
| hCoV-19/Egypt/CCHE57357_Wave_3_A054/2021 | EPI_ISL_2566502 | 07/03/2021 | Africa / Egypt | Human | unknown | unknown | unknown | unknown | unknown | unknown | C.17 | GR |
| hCoV-19/Egypt/NRC-6452/2020 | EPI_ISL_8193899 | 15/08/2020 | Africa / Egypt | Human | unknown | unknown | unknown | unknown | unknown | unknown | B.1.195 | G |
| hCoV-19/Egypt/ARMY-49/2021 | EPI_ISL_1936225 | 27/03/2021 | Africa / Egypt / Cairo | Human | unknown | unknown | 25-55 | Hospitalized | unknown | unknown | B | L |
| hCoV-19/Egypt/PHARCO-ARMY-81/2021 | EPI_ISL_1936365 | 12/02/2021 | Africa / Egypt / Cairo | Human | unknown | unknown | 25-55 | Hospitalized | unknown | unknown | B.1 | GH |
| hCoV-19/Egypt/PHARCO-ARMY-91/2021 | EPI_ISL_1936355 | 12/02/2021 | Africa / Egypt / Cairo | Human | unknown | unknown | 25-55 | Hospitalized | unknown | unknown | B.1 | GH |
| hCoV-19/Egypt/PHARCO-ARMY-94/2021 | EPI_ISL_1936358 | 12/02/2021 | Africa / Egypt / Cairo | Human | unknown | unknown | 25-55 | Hospitalized | unknown | unknown | C.36 | GR |
| hCoV-19/Egypt/PHARCO-ARMY-27/2021 | EPI_ISL_1936346 | 12/02/2021 | Africa / Egypt / Cairo | Human | unknown | unknown | 25-55 | Hospitalized | unknown | unknown | C.36 | GR |
| hCoV-19/Egypt/PHARCO-ARMY-71/2021 | EPI_ISL_1936331 | 12/02/2021 | Africa / Egypt / Cairo | Human | unknown | unknown | 25-55 | Hospitalized | unknown | unknown | C.36 | GR |
| hCoV-19/Egypt/PHARCO-ARMY-72/2021 | EPI_ISL_1936332 | 12/02/2021 | Africa / Egypt / Cairo | Human | unknown | unknown | 25-55 | Hospitalized | unknown | unknown | C.36 | GR |
| hCoV-19/Egypt/PHARCO-ARMY-75/2021 | EPI_ISL_1936335 | 12/02/2021 | Africa / Egypt / Cairo | Human | unknown | unknown | 25-55 | Hospitalized | unknown | unknown | C.36 | GR |
| hCoV-19/Egypt/PHARCO-ARMY-76/2021 | EPI_ISL_1936336 | 12/02/2021 | Africa / Egypt / Cairo | Human | unknown | unknown | 25-55 | Hospitalized | unknown | unknown | C.36 | GR |
| hCoV-19/Egypt/PHARCO-ARMY-60/2021 | EPI_ISL_1936320 | 12/02/2021 | Africa / Egypt / Cairo | Human | unknown | unknown | 25-55 | Hospitalized | unknown | unknown | C.36 | GR |
| hCoV-19/Egypt/PHARCO-ARMY-69/2021 | EPI_ISL_1936329 | 12/02/2021 | Africa / Egypt / Cairo | Human | unknown | unknown | 25-55 | Hospitalized | unknown | unknown | C.36 | GR |
| hCoV-19/Egypt/PHARCO-ARMY-52/2021 | EPI_ISL_1936312 | 12/02/2021 | Africa / Egypt / Cairo | Human | unknown | unknown | 25-55 | Hospitalized | unknown | unknown | C.36 | GR |
| hCoV-19/Egypt/PHARCO-ARMY-40/2021 | EPI_ISL_1936300 | 12/02/2021 | Africa / Egypt / Cairo | Human | unknown | unknown | 25-55 | Hospitalized | unknown | unknown | C.36 | GR |
| hCoV-19/Egypt/PHARCO-ARMY-42/2021 | EPI_ISL_1936302 | 12/02/2021 | Africa / Egypt / Cairo | Human | unknown | unknown | 25-55 | Hospitalized | unknown | unknown | B.1 | G |
| hCoV-19/Egypt/PHARCO-ARMY-48/2021 | EPI_ISL_1936308 | 12/02/2021 | Africa / Egypt / Cairo | Human | unknown | unknown | 25-55 | Hospitalized | unknown | unknown | C.36 | GR |
| hCoV-19/Egypt/MASRI-C5-040/2020 | EPI_ISL_1586895 | 2020 | Africa / Egypt / Cairo | Human | unknown | Male | 56 | unknown | unknown | unknown | C.17 | GR |
| hCoV-19/Egypt/NRC-660/2021 | EPI_ISL_8251498 | 19/05/2021 | Africa / Egypt | Human | unknown | unknown | unknown | unknown | unknown | unknown | C.36.3 | G |
| hCoV-19/Egypt/NRC-5885/2020 | EPI_ISL_2232395 | 17/06/2020 | Africa / Egypt | Human | unknown | unknown | unknown | unknown | unknown | unknown | B.1.170 | GH |
| hCoV-19/Egypt/NRC-662/2021 | EPI_ISL_8251499 | 19/05/2021 | Africa / Egypt | Human | unknown | unknown | unknown | unknown | unknown | unknown | C.36.3 | G |
| hCoV-19/Egypt/CPHL-S26/2021 | EPI_ISL_3600721 | 15/07/2021 | Africa / Egypt / Cairo | Human | unknown | Female | 37 | Released | unknown | unknown | B.1.617.2 | GK |
| hCoV-19/Egypt/CPHL-NRC-15/2020 | EPI_ISL_794597 | 04/06/2020 | Africa / Egypt | Human | unknown | unknown | unknown | unknown | unknown | unknown | B.1 | G |
| hCoV-19/Egypt/ARMY-203/2021 | EPI_ISL_1936136 | 02/05/2021 | Africa / Egypt / Cairo | Human | unknown | unknown | 25-55 | Hospitalized | unknown | unknown | C.17 | GR |
| hCoV-19/Egypt/PHARCO-ARMY-80/2021 | EPI_ISL_1936374 | 12/02/2021 | Africa / Egypt / Cairo | Human | unknown | unknown | 25-55 | Hospitalized | unknown | unknown | C.36 | GR |
| hCoV-19/Egypt/ARMY-400/2021 | EPI_ISL_1969078 | 28/03/2021 | Africa / Egypt / Cairo | Human | unknown | unknown | 29 | Hospitalized | unknown | unknown | B.1.1.7 | GR |
| hCoV-19/Egypt/ARMY-401/2021 | EPI_ISL_1969079 | 28/03/2021 | Africa / Egypt / Cairo | Human | unknown | unknown | 55 | Hospitalized | unknown | unknown | B.1.1.7 | GR |
| hCoV-19/Egypt/ARMY-404/2021 | EPI_ISL_1969081 | 24/04/2021 | Africa / Egypt / Cairo | Human | unknown | unknown | 59 | Hospitalized | unknown | unknown | B.1.1.7 | GR |
| hCoV-19/Egypt/CUNCI-HGC12I005/2021 | EPI_ISL_890198 | 09/01/2021 | Africa / Egypt | Human | unknown | unknown | unknown | unknown | unknown | unknown | B.1 | G |
| hCoV-19/Egypt/CCHE57357_Wave_3_A024/2021 | EPI_ISL_2566483 | 08/05/2021 | Africa / Egypt | Human | unknown | unknown | unknown | unknown | unknown | unknown | B.1.1.7 | GR |
| hCoV-19/Egypt/NRC-6367/2020 | EPI_ISL_8194871 | 25/07/2020 | Africa / Egypt | Human | unknown | unknown | unknown | unknown | unknown | unknown | C.17 | GR |
| hCoV-19/Egypt/NRC-7305/2020 | EPI_ISL_8193639 | 15/12/2020 | Africa / Egypt | Human | unknown | unknown | unknown | unknown | unknown | unknown | B.1.1 | G |
| hCoV-19/Egypt/EMC-1/2021 | EPI_ISL_2960150 | 21/04/2021 | Africa / Egypt | Human | unknown | unknown | unknown | unknown | unknown | unknown | B.1.1.7 | GRY |
| hCoV-19/Egypt/CUNCI-HGC8I031/2020 | EPI_ISL_857316 | 25/12/2020 | Africa / Egypt | Human | unknown | unknown | unknown | unknown | unknown | unknown | C.36 | GR |
| hCoV-19/Egypt/ARMY-252/2021 | EPI_ISL_1936116 | 02/05/2021 | Africa / Egypt / Cairo | Human | unknown | unknown | 25-55 | Hospitalized | unknown | unknown | C.17 | GR |
| hCoV-19/Egypt/PHARCO-ARMY-57/2021 | EPI_ISL_1936317 | 12/02/2021 | Africa / Egypt / Cairo | Human | unknown | unknown | 25-55 | Hospitalized | unknown | unknown | C.36 | GR |
| hCoV-19/Egypt/CUNCI-HGC12I011/2021 | EPI_ISL_890203 | 09/01/2021 | Africa / Egypt | Human | unknown | unknown | unknown | unknown | unknown | unknown | C.36 | GR |
| hCoV-19/Egypt/ARMY-30/2021 | EPI_ISL_1936204 | 27/03/2021 | Africa / Egypt / Cairo | Human | unknown | unknown | 25-55 | Hospitalized | unknown | unknown | C.17 | GR |
| hCoV-19/Egypt/ARMY-32/2021 | EPI_ISL_1936206 | 27/03/2021 | Africa / Egypt / Cairo | Human | unknown | unknown | 25-55 | Hospitalized | unknown | unknown | C.17 | GR |
| hCoV-19/Egypt/NRC-6991/2020 | EPI_ISL_2232306 | 26/10/2020 | Africa / Egypt | Human | unknown | Female | unknown | unknown | unknown | unknown | C.17 | GR |
| hCoV-19/Egypt/CCHE57357-A-31/2020 | EPI_ISL_812807 | 28/07/2020 | Africa / Egypt | Human | unknown | unknown | unknown | unknown | unknown | unknown | C.36 | GR |
| hCoV-19/Egypt/PHARCO-ARMY-47/2021 | EPI_ISL_1936307 | 12/02/2021 | Africa / Egypt / Cairo | Human | unknown | unknown | 25-55 | Hospitalized | unknown | unknown | C.36 | GR |
| hCoV-19/Egypt/CUNCI-HGC11I041/2021 | EPI_ISL_862807 | 03/01/2021 | Africa / Egypt | Human | unknown | unknown | unknown | unknown | unknown | unknown | B.1.1.353 | GR |
| hCoV-19/Egypt/CUNCI-HGC11I042/2021 | EPI_ISL_862808 | 03/01/2021 | Africa / Egypt | Human | unknown | unknown | unknown | unknown | unknown | unknown | C.36 | GR |
| hCoV-19/Egypt/CUNCI-HGC11I048/2021 | EPI_ISL_862810 | 03/01/2021 | Africa / Egypt | Human | unknown | unknown | unknown | unknown | unknown | unknown | B.1 | G |
| hCoV-19/Egypt/CUNCI-HGC11I046/2021 | EPI_ISL_907111 | 03/01/2021 | Africa / Egypt | Human | unknown | unknown | unknown | unknown | unknown | unknown | B.1 | G |
| hCoV-19/Egypt/CCHE57357_Wave_3_A069/2021 | EPI_ISL_2566511 | 14/05/2021 | Africa / Egypt | Human | unknown | unknown | unknown | unknown | unknown | unknown | C.17 | GR |
| hCoV-19/Egypt/ARMY-250/2021 | EPI_ISL_1936112 | 02/05/2021 | Africa / Egypt / Cairo | Human | unknown | unknown | 25-55 | Hospitalized | unknown | unknown | C.17 | GR |
| hCoV-19/Egypt/NRC-5610/2020 | EPI_ISL_2232313 | 07/06/2020 | Africa / Egypt | Human | unknown | Female | unknown | unknown | unknown | unknown | C.17 | GR |
| hCoV-19/Egypt/ARMY-45/2021 | EPI_ISL_1936220 | 27/03/2021 | Africa / Egypt / Cairo | Human | unknown | unknown | 25-55 | Hospitalized | unknown | unknown | A | S |
| hCoV-19/Egypt/CCHE57357_Wave_3_A_026/2021 | EPI_ISL_2566521 | 15/05/2021 | Africa / Egypt | Human | unknown | unknown | unknown | unknown | unknown | unknown | C.36.3 | O |
| hCoV-19/Egypt/CCHE57357-A-50/2020 | EPI_ISL_812823 | 23/07/2020 | Africa / Egypt | Human | unknown | unknown | unknown | unknown | unknown | unknown | B.1 | G |
| hCoV-19/Egypt/MASRI-C5-001/2020 | EPI_ISL_1109624 | 18/07/2020 | Africa / Egypt / Cairo | Human | unknown | Male | unknown | unknown | unknown | unknown | C.17 | GR |
| hCoV-19/Egypt/NRC-6895/2020 | EPI_ISL_2232324 | 14/06/2020 | Africa / Egypt | Human | unknown | Female | unknown | unknown | unknown | unknown | C.17 | GR |
| hCoV-19/Egypt/MASRI-004/2020 | EPI_ISL_1097025 | 08/06/2020 | Africa / Egypt / Cairo | Human | unknown | Female | 0 | Live | unknown | unknown | C.17 | G |
| hCoV-19/Egypt/CUNCI-HGC12I043/2021 | EPI_ISL_890225 | 09/01/2021 | Africa / Egypt | Human | unknown | unknown | unknown | unknown | unknown | unknown | C.36 | GR |
| hCoV-19/Egypt/CCHE57357-A-53/2020 | EPI_ISL_812826 | 18/06/2020 | Africa / Egypt | Human | unknown | unknown | unknown | unknown | unknown | unknown | B.1 | GH |
| hCoV-19/Egypt/ARMY-51/2021 | EPI_ISL_1936227 | 27/03/2021 | Africa / Egypt / Cairo | Human | unknown | unknown | 25-55 | Hospitalized | unknown | unknown | C.36.3 | GR |
| hCoV-19/Egypt/PHARCO-ARMY-68/2021 | EPI_ISL_1936328 | 12/02/2021 | Africa / Egypt / Cairo | Human | unknown | unknown | 25-55 | Hospitalized | unknown | unknown | C.36 | GR |
| hCoV-19/Egypt/CUNCI-HGC9I012/2020 | EPI_ISL_857329 | 25/12/2020 | Africa / Egypt | Human | unknown | unknown | unknown | unknown | unknown | unknown | C.36 | GR |
| hCoV-19/Egypt/CUNCI-HGC12I050/2021 | EPI_ISL_890229 | 09/01/2021 | Africa / Egypt | Human | unknown | unknown | unknown | unknown | unknown | unknown | C.36 | GR |
| hCoV-19/Egypt/NRC-479/2021 | EPI_ISL_2227329 | 15/04/2021 | Africa / Egypt | Human | unknown | Female | unknown | unknown | unknown | unknown | C.36.3 | GR |
| hCoV-19/Egypt/CUNCI-HGC12I052/2021 | EPI_ISL_890230 | 09/01/2021 | Africa / Egypt | Human | unknown | unknown | unknown | unknown | unknown | unknown | B.1 | GH |
| hCoV-19/Egypt/ARMY-275/2021 | EPI_ISL_1936130 | 02/05/2021 | Africa / Egypt / Cairo | Human | unknown | unknown | 25-55 | Hospitalized | unknown | unknown | A | S |
| hCoV-19/Egypt/ARMY-55/2021 | EPI_ISL_1936231 | 27/03/2021 | Africa / Egypt / Cairo | Human | unknown | unknown | 25-55 | Hospitalized | unknown | unknown | C.17 | GR |
| hCoV-19/Egypt/CCHE57357-A-60/2020 | EPI_ISL_812833 | 12/06/2020 | Africa / Egypt | Human | unknown | unknown | unknown | unknown | unknown | unknown | B | L |
| hCoV-19/Egypt/CUNCI-HGC9I029/2020 | EPI_ISL_857333 | 25/12/2020 | Africa / Egypt | Human | unknown | unknown | unknown | unknown | unknown | unknown | C.36 | GR |
| hCoV-19/Egypt/ARMY-57/2021 | EPI_ISL_1936233 | 27/03/2021 | Africa / Egypt / Cairo | Human | unknown | unknown | 25-55 | Hospitalized | unknown | unknown | B.1 | GH |
| hCoV-19/Egypt/EGY-Cairo/2020 | EPI_ISL_794634 | 01/12/2020 | Africa / Egypt | Human | unknown | unknown | unknown | unknown | unknown | unknown | B.1 | GH |
| hCoV-19/Egypt/CUNCI-HGC9I042/2020 | EPI_ISL_857335 | 25/12/2020 | Africa / Egypt | Human | unknown | unknown | unknown | unknown | unknown | unknown | B.1 | G |
| hCoV-19/Egypt/ARMY-204/2021 | EPI_ISL_1936137 | 02/05/2021 | Africa / Egypt / Cairo | Human | unknown | unknown | 25-55 | Hospitalized | unknown | unknown | C.17 | GR |
| hCoV-19/Egypt/CUNCI-HGC11I015/2021 | EPI_ISL_907103 | 03/01/2021 | Africa / Egypt | Human | unknown | unknown | unknown | unknown | unknown | unknown | B.1 | G |
| hCoV-19/Egypt/CUNCI-HGC11I050/2021 | EPI_ISL_907112 | 03/01/2021 | Africa / Egypt | Human | unknown | unknown | unknown | unknown | unknown | unknown | C.36 | GR |
| hCoV-19/Egypt/CCHE57357-A-70/2020 | EPI_ISL_812840 | 02/07/2020 | Africa / Egypt | Human | unknown | unknown | unknown | unknown | unknown | unknown | B | O |
| hCoV-19/Egypt/PHARCO-ARMY-22/2021 | EPI_ISL_1936341 | 12/02/2021 | Africa / Egypt / Cairo | Human | unknown | unknown | 25-55 | Hospitalized | unknown | unknown | C.36 | GR |
| hCoV-19/Egypt/ARMY-301/2021 | EPI_ISL_1936241 | 22/04/2021 | Africa / Egypt / Cairo | Human | unknown | unknown | 25-60 | Hospitalized | unknown | unknown | C.36.3 | GR |
| hCoV-19/Egypt/CCHE57357-A-72/2020 | EPI_ISL_812842 | 30/06/2020 | Africa / Egypt | Human | unknown | unknown | unknown | unknown | unknown | unknown | B.1.1 | GR |
| hCoV-19/Egypt/NRC-6322/2020 | EPI_ISL_8194890 | 15/07/2020 | Africa / Egypt | Human | unknown | unknown | unknown | unknown | unknown | unknown | B.59 | GR |
| hCoV-19/Egypt/PHARCO-ARMY-24/2021 | EPI_ISL_1936343 | 12/02/2021 | Africa / Egypt / Cairo | Human | unknown | unknown | 25-55 | Hospitalized | unknown | unknown | C.36 | GR |
| hCoV-19/Egypt/PHARCO-ARMY-25/2021 | EPI_ISL_1936344 | 12/02/2021 | Africa / Egypt / Cairo | Human | unknown | unknown | 25-55 | Hospitalized | unknown | unknown | B.1 | GH |
| hCoV-19/Egypt/ARMY-305/2021 | EPI_ISL_1936244 | 22/04/2021 | Africa / Egypt / Cairo | Human | unknown | unknown | 25-60 | Hospitalized | unknown | unknown | C.36.3 | GR |
| hCoV-19/Egypt/CCHE57357-A-88/2020 | EPI_ISL_812845 | 16/07/2020 | Africa / Egypt | Human | unknown | unknown | unknown | unknown | unknown | unknown | B | L |
| hCoV-19/Egypt/CUNCI-HGC9I027/2020 | EPI_ISL_857345 | 25/12/2020 | Africa / Egypt | Human | unknown | unknown | unknown | unknown | unknown | unknown | C.36 | GR |
| hCoV-19/Egypt/NRC-5444/2020 | EPI_ISL_2232347 | 27/05/2020 | Africa / Egypt | Human | unknown | Male | unknown | unknown | unknown | unknown | C.17 | GR |
| hCoV-19/Egypt/PHARCO-ARMY-29/2021 | EPI_ISL_1936348 | 12/02/2021 | Africa / Egypt / Cairo | Human | unknown | unknown | 25-55 | Hospitalized | unknown | unknown | B.1 | GH |
| hCoV-19/Egypt/PHARCO-ARMY-31/2021 | EPI_ISL_1936350 | 12/02/2021 | Africa / Egypt / Cairo | Human | unknown | unknown | 25-55 | Hospitalized | unknown | unknown | C.36 | GR |
| hCoV-19/Egypt/NRC-6838/2020 | EPI_ISL_2232350 | 04/10/2020 | Africa / Egypt | Human | unknown | unknown | unknown | unknown | unknown | unknown | B.1 | GH |
| hCoV-19/Egypt/NRC-6407/2020 | EPI_ISL_2380050 | 05/08/2020 | Africa / Egypt | Human | unknown | Male | unknown | unknown | unknown | unknown | C.17 | GR |
| hCoV-19/Egypt/CCHE57357-P-01/2020 | EPI_ISL_812850 | 18/06/2020 | Africa / Egypt | Human | unknown | unknown | unknown | unknown | unknown | unknown | B.1 | GH |
| hCoV-19/Egypt/PHARCO-ARMY-32/2021 | EPI_ISL_1936351 | 12/02/2021 | Africa / Egypt / Cairo | Human | unknown | unknown | 25-55 | Hospitalized | unknown | unknown | C.36 | GR |
| hCoV-19/Egypt/ARMY-312/2021 | EPI_ISL_1936251 | 22/04/2021 | Africa / Egypt / Cairo | Human | unknown | unknown | 25-60 | Hospitalized | unknown | unknown | A | S |
| hCoV-19/Egypt/NRC-6638/2020 | EPI_ISL_2232254 | 10/09/2020 | Africa / Egypt | Human | unknown | unknown | unknown | unknown | unknown | unknown | B.1.35 | G |
| hCoV-19/Egypt/NRC-6856/2020 | EPI_ISL_2380054 | 06/10/2020 | Africa / Egypt | Human | unknown | unknown | unknown | unknown | unknown | unknown | B.1 | GH |
| hCoV-19/Egypt/ARMY-316/2021 | EPI_ISL_1936255 | 22/04/2021 | Africa / Egypt / Cairo | Human | unknown | unknown | 25-60 | Hospitalized | unknown | unknown | C.17 | GR |
| hCoV-19/Egypt/PHARCO-ARMY-93/2021 | EPI_ISL_1936357 | 12/02/2021 | Africa / Egypt / Cairo | Human | unknown | unknown | 25-55 | Hospitalized | unknown | unknown | C.36 | GR |
| hCoV-19/Egypt/NRC-6632/2020 | EPI_ISL_2232257 | 09/09/2020 | Africa / Egypt | Human | unknown | unknown | unknown | unknown | unknown | unknown | C.17 | GR |
| hCoV-19/Egypt/ARMY-320/2021 | EPI_ISL_1936259 | 22/04/2021 | Africa / Egypt / Cairo | Human | unknown | unknown | 25-60 | Hospitalized | unknown | unknown | C.17 | G |
| hCoV-19/Egypt/CPHL-NRC-S8/2021 | EPI_ISL_2313061 | 2021-03 | Africa / Egypt | Human | unknown | unknown | unknown | unknown | unknown | unknown | C.17 | GR |
| hCoV-19/Egypt/NRC-6411/2020 | EPI_ISL_2232261 | 05/08/2020 | Africa / Egypt | Human | unknown | Female | unknown | unknown | unknown | unknown | C.17 | GR |
| hCoV-19/Egypt/NRC-6820/2020 | EPI_ISL_2232361 | 30/09/2020 | Africa / Egypt | Human | unknown | unknown | unknown | unknown | unknown | unknown | B.1 | GH |
| hCoV-19/Egypt/NRC-5461/2020 | EPI_ISL_2232343 | 30/05/2020 | Africa / Egypt | Human | unknown | Male | unknown | unknown | unknown | unknown | B.1.1 | GR |
| hCoV-19/Egypt/NRC-5876/2020 | EPI_ISL_2232259 | 15/06/2020 | Africa / Egypt | Human | unknown | unknown | unknown | unknown | unknown | unknown | B.1.170 | GH |
| hCoV-19/Egypt/NRC1/2020 | EPI_ISL_1315064 | 26/04/2020 | Africa / Egypt / Cairo / Giza | Human | unknown | Male | 45 | Hospitalized | unknown | unknown | B.1 | GH |
| hCoV-19/Egypt/CUNCI-HGC11I052/2021 | EPI_ISL_862812 | 03/01/2021 | Africa / Egypt | Human | unknown | unknown | unknown | unknown | unknown | unknown | C.36 | GR |
| hCoV-19/Egypt/CCHE57357-A-39/2020 | EPI_ISL_812812 | 11/08/2020 | Africa / Egypt | Human | unknown | unknown | unknown | unknown | unknown | unknown | B | O |
| hCoV-19/Egypt/PHARCO-ARMY-54/2021 | EPI_ISL_1936314 | 12/02/2021 | Africa / Egypt / Cairo | Human | unknown | unknown | 25-55 | Hospitalized | unknown | unknown | C.36 | GR |
| hCoV-19/Egypt/ARMY-261/2021 | EPI_ISL_1936122 | 02/05/2021 | Africa / Egypt / Cairo | Human | unknown | unknown | 25-55 | Hospitalized | unknown | unknown | C.17 | GR |
| hCoV-19/Egypt/CCHE57357_Wave_3_A_035/2021 | EPI_ISL_2566523 | 07/05/2021 | Africa / Egypt | Human | unknown | unknown | unknown | unknown | unknown | unknown | C.36.3 | GR |
| hCoV-19/Egypt/PHARCO-ARMY-66/2021 | EPI_ISL_1936326 | 12/02/2021 | Africa / Egypt / Cairo | Human | unknown | unknown | 25-55 | Hospitalized | unknown | unknown | B.1 | G |
| hCoV-19/Egypt/CCHE57357-P-34/2020 | EPI_ISL_812865 | 02/08/2020 | Africa / Egypt | Human | unknown | unknown | unknown | unknown | unknown | unknown | B.1 | G |
| hCoV-19/Egypt/CPHL-A7/2020 | EPI_ISL_12571632 | 01/12/2020 | Africa / Egypt / Cairo | Human | Sentinel surveillance (ILI) | Male | 22 | unknown | unknown | Sentinel surveillance (ILI) | Unassigned | GR |
| hCoV-19/Egypt/CPHL-NRC-14/2020 | EPI_ISL_794598 | 02/06/2020 | Africa / Egypt | Human | unknown | unknown | unknown | unknown | unknown | unknown | B.1 | GH |
| hCoV-19/Egypt/USC-5/2021 | EPI_ISL_8470239 | 2021-01 | Africa / Egypt / Cairo | Human | unknown | unknown | unknown | unknown | unknown | unknown | Unassigned | O |
| hCoV-19/Egypt/CPHL-EGY21476/2021 | EPI_ISL_9047805 | 29/09/2021 | Africa / Egypt / Aswan governorate | Human | unknown | Female | 43 | Hospitalized | unknown | unknown | AY.122 | GK |
| hCoV-19/Egypt/CPHL-EGY21473/2021 | EPI_ISL_9047802 | 28/09/2021 | Africa / Egypt / Aswan governorate | Human | unknown | Male | 27 | Hospitalized | unknown | unknown | AY.112 (consensus call) | GK |
| hCoV-19/Egypt/CPHL-EGY21472/2021 | EPI_ISL_9047801 | 27/09/2021 | Africa / Egypt / Aswan governorate | Human | unknown | Female | 19 | Hospitalized | unknown | unknown | AY.42 | GK |
| hCoV-19/Egypt/CPHL-EGY21471/2021 | EPI_ISL_9047800 | 27/09/2021 | Africa / Egypt / Aswan governorate | Human | unknown | Female | 29 | Hospitalized | unknown | unknown | B.1.617.2 | GK |
| hCoV-19/Egypt/CPHL-EGY21468/2021 | EPI_ISL_9047797 | 25/09/2021 | Africa / Egypt / Aswan governorate | Human | unknown | Male | 38 | Hospitalized | unknown | unknown | B.1.617.2 | GK |
| hCoV-19/Egypt/CPHL-EGY21466/2021 | EPI_ISL_9047795 | 22/09/2021 | Africa / Egypt / Aswan governorate | Human | unknown | Female | 62 | Hospitalized | unknown | unknown | Unassigned | GK |
| hCoV-19/Egypt/CPHL-EGY21465/2021 | EPI_ISL_9047794 | 22/09/2021 | Africa / Egypt / Aswan governorate | Human | unknown | Female | 66 | Hospitalized | unknown | unknown | B.1.617.2 | GK |
| hCoV-19/Egypt/CPHL-EGY21463/2021 | EPI_ISL_9047792 | 20/09/2021 | Africa / Egypt / Aswan governorate | Human | unknown | Male | 53 | Hospitalized | unknown | unknown | B.1.617.2 | GK |
| hCoV-19/Egypt/CPHL-EGY21461/2021 | EPI_ISL_9047790 | 19/09/2021 | Africa / Egypt / Aswan governorate | Human | unknown | Male | 24 | Hospitalized | unknown | unknown | B.1.617.2 | GK |
| hCoV-19/Egypt/CPHL-EGY21459/2021 | EPI_ISL_9047788 | 17/11/2021 | Africa / Egypt / Aswan governorate | Human | unknown | Male | 25 | Hospitalized | unknown | unknown | B.1.617.2 | GK |
| hCoV-19/Egypt/CPHL-EGY21458/2021 | EPI_ISL_9047787 | 16/11/2021 | Africa / Egypt / Aswan governorate | Human | unknown | Male | 84 | Hospitalized | unknown | unknown | B.1.617.2 | GK |
| hCoV-19/Egypt/CPHL-EGY21448/2021 | EPI_ISL_9047777 | 01/12/2021 | Africa / Egypt / Aswan governorate | Human | unknown | Male | 45 | Hospitalized | unknown | unknown | B.1.617.2 | GK |
| hCoV-19/Egypt/CPHL-EGY21445/2022 | EPI_ISL_9047774 | 07/01/2022 | Africa / Egypt / Red sea governorate / Hurgada | Human | unknown | Female | 25 | Hotel isolation | unknown | unknown | AY.122 | GK |
| hCoV-19/Egypt/CPHL-EGY21444/2022 | EPI_ISL_9047773 | 08/01/2022 | Africa / Egypt / Red sea governorate / Hurgada | Human | unknown | Female | 36 | Hotel isolation | unknown | unknown | B.1.617.2 | GK |
| hCoV-19/Egypt/CPHL-EGY21438/2022 | EPI_ISL_9047767 | 08/01/2022 | Africa / Egypt / Red sea governorate / Hurgada | Human | unknown | Female | 24 | Hotel isolation | unknown | unknown | B.1.617.2 | GK |
| hCoV-19/Egypt/CPHL-EGY21428/2022 | EPI_ISL_9047757 | 02/01/2022 | Africa / Egypt / Red sea governorate / Hurgada | Human | unknown | Male | 11 | Hotel isolation | unknown | unknown | Unassigned | GK |
| hCoV-19/Egypt/USC-4/2021 | EPI_ISL_8469870 | 2021-01 | Africa / Egypt / Cairo | Human | unknown | unknown | unknown | unknown | unknown | unknown | Unassigned | O |
| hCoV-19/Egypt/CPHL-EGY21427/2022 | EPI_ISL_9047756 | 02/01/2022 | Africa / Egypt / Red sea governorate / Hurgada | Human | unknown | Male | 24 | Hotel isolation | unknown | unknown | Unassigned | GK |
| hCoV-19/Egypt/CPHL-EGY21422/2022 | EPI_ISL_9047753 | 02/01/2022 | Africa / Egypt / Red sea governorate / Hurgada | Human | unknown | Female | 36 | Hotel isolation | unknown | unknown | Unassigned | G |
| hCoV-19/Egypt/CPHL-EGY21420/2022 | EPI_ISL_9047751 | 02/01/2022 | Africa / Egypt / Red sea governorate / Hurgada | Human | unknown | Female | 24 | Hotel isolation | unknown | unknown | B.1.617.2 | GK |
| hCoV-19/Egypt/CPHL-EGY21419/2022 | EPI_ISL_9047750 | 02/01/2022 | Africa / Egypt / Red sea governorate / Hurgada | Human | unknown | Male | 23 | Hotel isolation | unknown | unknown | B.1.617.2 | GK |
| hCoV-19/Egypt/CPHL-EGY21417/2022 | EPI_ISL_9047748 | 02/01/2022 | Africa / Egypt / Red sea governorate / Hurgada | Human | unknown | Female | 22 | Hotel isolation | unknown | unknown | B.1.617.2 | GK |
| hCoV-19/Egypt/CPHL-EGY21415/2022 | EPI_ISL_9047746 | 02/01/2022 | Africa / Egypt / Red sea governorate / Hurgada | Human | unknown | Female | 28 | Hotel isolation | unknown | unknown | Unassigned | G |
| hCoV-19/Egypt/CPHL-EGY21412/2022 | EPI_ISL_9047743 | 02/01/2022 | Africa / Egypt / Red sea governorate / Hurgada | Human | unknown | Male | 37 | Hotel isolation | unknown | unknown | Unassigned | GK |
| hCoV-19/Egypt/CPHL-EGY21411/2022 | EPI_ISL_9047742 | 02/01/2022 | Africa / Egypt / Aswan governorate | Human | unknown | Male | 48 | Hospitalized | unknown | unknown | Unassigned | GRA |
| hCoV-19/Egypt/CPHL-EGY21407/2021 | EPI_ISL_9047739 | 31/12/2021 | Africa / Egypt / Red sea governorate / Hurgada | Human | unknown | Female | 46 | Hotel isolation | unknown | unknown | B.1.617.2 | GK |
| hCoV-19/Egypt/CPHL-EGY21406/2021 | EPI_ISL_9047738 | 31/12/2021 | Africa / Egypt / Red sea governorate / Hurgada | Human | unknown | Female | 48 | Hotel isolation | unknown | unknown | B.1.617.2 | GK |
| hCoV-19/Egypt/CPHL-EGY21401/2021 | EPI_ISL_9047733 | 31/12/2021 | Africa / Egypt / Red sea governorate / Hurgada | Human | unknown | Female | 66 | Hotel isolation | unknown | unknown | B.1.617.2 | GK |
| hCoV-19/Egypt/CPHL-EGY21400/2022 | EPI_ISL_9047732 | 02/01/2022 | Africa / Egypt / Red sea governorate / Hurgada | Human | unknown | Female | 44 | Hotel isolation | unknown | unknown | Unassigned | GK |
| hCoV-19/Egypt/CPHL-EGY21398/2021 | EPI_ISL_9047731 | 31/12/2021 | Africa / Egypt / Red sea governorate / Hurgada | Human | unknown | Male | 57 | Hotel isolation | unknown | unknown | B.1.617.2 | GK |
| hCoV-19/Egypt/CPHL-EGY21397/2021 | EPI_ISL_9047730 | 31/12/2021 | Africa / Egypt / Red sea governorate / Hurgada | Human | unknown | Male | 56 | Hotel isolation | unknown | unknown | B.1.617.2 | GK |
| hCoV-19/Egypt/CPHL-EGY21396/2022 | EPI_ISL_9047729 | 01/01/2022 | Africa / Egypt / Red sea governorate / Hurgada | Human | unknown | Female | 46 | Hotel isolation | unknown | unknown | Unassigned | G |
| hCoV-19/Egypt/CPHL-EGY21394/2021 | EPI_ISL_9047727 | 31/12/2021 | Africa / Egypt / Red sea governorate / Hurgada | Human | unknown | Male | 45 | Hotel isolation | unknown | unknown | B.1.617.2 | GK |
| hCoV-19/Egypt/CPHL-EGY21393/2022 | EPI_ISL_9047726 | 01/01/2022 | Africa / Egypt / Red sea governorate / Hurgada | Human | unknown | Male | 12 | Hotel isolation | unknown | unknown | B.1.617.2 | GK |
| hCoV-19/Egypt/CPHL-EGY21392/2021 | EPI_ISL_9047725 | 31/12/2021 | Africa / Egypt / Red sea governorate / Hurgada | Human | unknown | Male | 24 | Hotel isolation | unknown | unknown | B.1.617.2 | GK |
| hCoV-19/Egypt/CPHL-EGY21391/2021 | EPI_ISL_9047724 | 31/12/2021 | Africa / Egypt / Red sea governorate / Hurgada | Human | unknown | Male | 26 | Hotel isolation | unknown | unknown | B.1.617.2 | GK |
| hCoV-19/Egypt/CPHL-EGY21390/2022 | EPI_ISL_9047723 | 01/01/2022 | Africa / Egypt / Red sea governorate / Hurgada | Human | unknown | Female | 23 | Hotel isolation | unknown | unknown | B.1.617.2 | GK |
| hCoV-19/Egypt/CPHL-EGY21389/2021 | EPI_ISL_9047722 | 31/12/2021 | Africa / Egypt / Red sea governorate / Hurgada | Human | unknown | Female | 52 | Hotel isolation | unknown | unknown | Unassigned | GK |
| hCoV-19/Egypt/CPHL-EGY21388/2021 | EPI_ISL_9047721 | 31/12/2021 | Africa / Egypt / Red sea governorate / Hurgada | Human | unknown | Male | 48 | Hotel isolation | unknown | unknown | Unassigned | GK |
| hCoV-19/Egypt/CPHL-EGY21384/2022 | EPI_ISL_9047718 | 01/01/2022 | Africa / Egypt / Red sea governorate / Hurgada | Human | unknown | Male | 26 | Hotel isolation | unknown | unknown | B.1.617.2 | GK |
| hCoV-19/Egypt/CPHL-EGY21379/2021 | EPI_ISL_9047714 | 31/12/2021 | Africa / Egypt / Red sea governorate / Hurgada | Human | unknown | Male | 26 | Hotel isolation | unknown | unknown | B.1.617.2 | GK |
| hCoV-19/Egypt/CPHL-EGY21378/2021 | EPI_ISL_9047713 | 31/12/2021 | Africa / Egypt / Red sea governorate / Hurgada | Human | unknown | Female | 23 | Hotel isolation | unknown | unknown | AY.127 | GK |
| hCoV-19/Egypt/CPHL-EGY21377/2021 | EPI_ISL_9047712 | 31/12/2021 | Africa / Egypt / Aswan governorate | Human | unknown | Female | 52 | Hospitalized | unknown | unknown | Unassigned | GRA |
| hCoV-19/Egypt/CPHL-EGY21376/2021 | EPI_ISL_9047711 | 01/12/2021 | Africa / Egypt / Aswan governorate | Human | unknown | Male | 48 | Hospitalized | unknown | unknown | Unassigned | GRA |
| hCoV-19/Egypt/CPHL-EGY21375/2021 | EPI_ISL_9047710 | 01/12/2021 | Africa / Egypt / Red sea governorate / Hurgada | Human | unknown | Female | 23 | Hotel isolation | unknown | unknown | B.1.617.2 | GK |
| hCoV-19/Egypt/CPHL-EGY21374/2021 | EPI_ISL_9047709 | 31/12/2021 | Africa / Egypt / Red sea governorate / Hurgada | Human | unknown | Male | 29 | Hotel isolation | unknown | unknown | B.1.617.2 | GK |
| hCoV-19/Egypt/CPHL-EGY21373/2021 | EPI_ISL_9047708 | 31/12/2021 | Africa / Egypt / Red sea governorate / Hurgada | Human | unknown | Male | 25 | Hotel isolation | unknown | unknown | B.1.617.2 | GK |
| hCoV-19/Egypt/CPHL-EGY21372/2021 | EPI_ISL_9047707 | 31/12/2021 | Africa / Egypt / Red sea governorate / Hurgada | Human | unknown | Female | 23 | Hotel isolation | unknown | unknown | B.1.617.2 | GK |
| hCoV-19/Egypt/CPHL-EGY21371/2021 | EPI_ISL_9047706 | 31/12/2021 | Africa / Egypt / Red sea governorate / Hurgada | Human | unknown | Female | 27 | Hotel isolation | unknown | unknown | B.1.617.2 | GK |
| hCoV-19/Egypt/CPHL-EGY21370/2021 | EPI_ISL_9047705 | 31/12/2021 | Africa / Egypt / Red sea governorate / Hurgada | Human | unknown | Female | 29 | Hotel isolation | unknown | unknown | AY.46 | GK |
| hCoV-19/Egypt/CPHL-EGY21368/2021 | EPI_ISL_9047703 | 31/12/2021 | Africa / Egypt / Red sea governorate / Hurgada | Human | unknown | Male | 23 | Hotel isolation | unknown | unknown | B.1.617.2 | GK |
| hCoV-19/Egypt/CPHL-EGY21367/2021 | EPI_ISL_9047702 | 31/12/2021 | Africa / Egypt / Red sea governorate / Hurgada | Human | unknown | Female | 44 | Hotel isolation | unknown | unknown | B.1.617.2 | GK |
| hCoV-19/Egypt/CPHL-EGY21366/2021 | EPI_ISL_9047701 | 31/12/2021 | Africa / Egypt / Red sea governorate / Hurgada | Human | unknown | Female | 54 | Hotel isolation | unknown | unknown | B.1.617.2 | GK |
| hCoV-19/Egypt/CPHL-EGY21365/2021 | EPI_ISL_9047700 | 31/12/2021 | Africa / Egypt / Red sea governorate / Hurgada | Human | unknown | Female | 56 | Hotel isolation | unknown | unknown | AY.122 | GK |
| hCoV-19/Egypt/CPHL-EGY21363/2021 | EPI_ISL_9047698 | 31/12/2021 | Africa / Egypt / Red sea governorate / Hurgada | Human | unknown | Female | 56 | Hotel isolation | unknown | unknown | B.1.617.2 | GK |
| hCoV-19/Egypt/CPHL-EGY21362/2021 | EPI_ISL_9047697 | 31/12/2021 | Africa / Egypt / Red sea governorate / Hurgada | Human | unknown | Male | 54 | Hotel isolation | unknown | unknown | B.1.617.2 | GK |
| hCoV-19/Egypt/CPHL-EGY21359/2021 | EPI_ISL_9047695 | 31/12/2021 | Africa / Egypt / Red sea governorate / Hurgada | Human | unknown | Female | 54 | Hotel isolation | unknown | unknown | AY.122 (consensus call) | GK |
| hCoV-19/Egypt/CPHL-EGY21358/2021 | EPI_ISL_9047694 | 31/12/2021 | Africa / Egypt / Red sea governorate / Hurgada | Human | unknown | Female | 56 | Hotel isolation | unknown | unknown | AY.46 | GK |
| hCoV-19/Egypt/CPHL-EGY21356/2021 | EPI_ISL_9047693 | 31/12/2021 | Africa / Egypt / Red sea governorate / Hurgada | Human | unknown | Female | 52 | Hotel isolation | unknown | unknown | B.1.617.2 | O |
| hCoV-19/Egypt/CPHL-EGY21355/2021 | EPI_ISL_9047692 | 31/12/2021 | Africa / Egypt / Red sea governorate / Hurgada | Human | unknown | Male | 53 | Hotel isolation | unknown | unknown | B.1.617.2 | GK |
| hCoV-19/Egypt/CPHL-EGY21349/2021 | EPI_ISL_9047686 | 31/12/2021 | Africa / Egypt / Red sea governorate / Hurgada | Human | unknown | Female | 46 | Hotel isolation | unknown | unknown | B.1.617.2 | O |
| hCoV-19/Egypt/CPHL-EGY21341/2022 | EPI_ISL_9047678 | 04/01/2022 | Africa / Egypt / Red sea governorate / Hurgada | Human | unknown | Male | 29 | Hotel isolation | unknown | unknown | AY.122 | GK |
| hCoV-19/Egypt/CPHL-EGY21333/2021 | EPI_ISL_9047671 | 29/12/2021 | Africa / Egypt / Red sea governorate / Hurgada | Human | unknown | Female | 39 | Hotel isolation | unknown | unknown | B.1.617.2 | GK |
| hCoV-19/Egypt/CPHL-EGY21332/2021 | EPI_ISL_9047670 | 29/12/2021 | Africa / Egypt / Red sea governorate / Hurgada | Human | unknown | Female | 20 | Hotel isolation | unknown | unknown | B.1.617.2 | GK |
| hCoV-19/Egypt/CPHL-EGY21329/2021 | EPI_ISL_9047667 | 29/12/2021 | Africa / Egypt / Red sea governorate / Hurgada | Human | unknown | Male | 56 | Hotel isolation | unknown | unknown | AY.122 | GK |
| hCoV-19/Egypt/CPHL-EGY21328/2021 | EPI_ISL_9047666 | 29/12/2021 | Africa / Egypt / Red sea governorate / Hurgada | Human | unknown | Male | 24 | Hotel isolation | unknown | unknown | AY.122 | GH |
| hCoV-19/Egypt/CPHL-EGY21327/2021 | EPI_ISL_9047665 | 29/12/2021 | Africa / Egypt / Red sea governorate / Hurgada | Human | unknown | Female | 34 | Hotel isolation | unknown | unknown | B.1.617.2 | GK |
| hCoV-19/Egypt/CPHL-EGY21323/2021 | EPI_ISL_9047661 | 28/12/2021 | Africa / Egypt / Red sea governorate / Hurgada | Human | unknown | Female | 42 | Hotel isolation | unknown | unknown | B.1.617.2 | GK |
| hCoV-19/Egypt/CPHL-EGY21322/2021 | EPI_ISL_9047660 | 28/12/2021 | Africa / Egypt / Aswan governorate | Human | unknown | Female | 21 | Hotel isolation | unknown | unknown | BA.1 | GRA |
| hCoV-19/Egypt/CPHL-EGY21320/2021 | EPI_ISL_9047659 | 28/12/2021 | Africa / Egypt / Red sea governorate / Hurgada | Human | unknown | Male | 36 | Hotel isolation | unknown | unknown | B.1.617.2 | GK |
| hCoV-19/Egypt/CPHL-EGY21319/2021 | EPI_ISL_9047658 | 28/12/2021 | Africa / Egypt / Red sea governorate / Hurgada | Human | unknown | Male | 27 | Hotel isolation | unknown | unknown | B.1.617.2 | GK |
| hCoV-19/Egypt/CPHL-EGY21318/2021 | EPI_ISL_9047657 | 28/12/2021 | Africa / Egypt / Red sea governorate / Hurgada | Human | unknown | Male | 28 | Hotel isolation | unknown | unknown | AY.122 (consensus call) | GK |
| hCoV-19/Egypt/CPHL-EGY21315/2021 | EPI_ISL_9047654 | 28/12/2021 | Africa / Egypt / Red sea governorate / Hurgada | Human | unknown | MALE+L316:N366 | 31 | Hotel isolation | unknown | unknown | AY.122 | GK |
| hCoV-19/Egypt/CPHL-EGY21312/2021 | EPI_ISL_9047652 | 28/12/2021 | Africa / Egypt / Red sea governorate / Hurgada | Human | unknown | Male | 49 | Hotel isolation | unknown | unknown | B.1.617.2 | GK |
| hCoV-19/Egypt/CPHL-EGY21311/2021 | EPI_ISL_9047651 | 27/12/2021 | Africa / Egypt / Red sea governorate / Hurgada | Human | unknown | Female | 48 | Hotel isolation | unknown | unknown | AY.112 | GK |
| hCoV-19/Egypt/CPHL-EGY21309/2021 | EPI_ISL_9047649 | 27/12/2021 | Africa / Egypt / Red sea governorate / Hurgada | Human | unknown | Male | 45 | Hotel isolation | unknown | unknown | B.1.617.2 | GK |
| hCoV-19/Egypt/CPHL-EGY21308/2021 | EPI_ISL_9047648 | 27/12/2021 | Africa / Egypt / Red sea governorate / Hurgada | Human | unknown | Female | 45 | Hotel isolation | unknown | unknown | B.1.617.2 | GK |
| hCoV-19/Egypt/CPHL-EGY21306/2021 | EPI_ISL_9047646 | 04/12/2021 | Africa / Egypt / Red sea governorate / Hurgada | Human | unknown | Female | 44 | Hotel isolation | unknown | unknown | AY.122 | GK |
| hCoV-19/Egypt/CPHL-EGY21305/2021 | EPI_ISL_9047645 | 15/12/2021 | Africa / Egypt / Red sea governorate / Hurgada | Human | unknown | Female | 36 | Hotel isolation | unknown | unknown | Unassigned | GK |
| hCoV-19/Egypt/CPHL-EGY21304/2021 | EPI_ISL_9047644 | 15/12/2021 | Africa / Egypt / Red sea governorate / Hurgada | Human | unknown | Female | 59 | Hotel isolation | unknown | unknown | AY.121 | GK |
| hCoV-19/Egypt/CPHL-EGY21301/2021 | EPI_ISL_9047642 | 15/12/2021 | Africa / Egypt / Red sea governorate / Hurgada | Human | unknown | Female | 29 | Hotel isolation | unknown | unknown | B.1.617.2 | GK |
| hCoV-19/Egypt/CPHL-EGY21300/2021 | EPI_ISL_9047641 | 15/12/2021 | Africa / Egypt / Red sea governorate / Hurgada | Human | unknown | Female | 17 | Hotel isolation | unknown | unknown | B.1.617.2 | GK |
| hCoV-19/Egypt/CPHL-EGY21298/2021 | EPI_ISL_9047640 | 12/12/2021 | Africa / Egypt / Red sea governorate / Hurgada | Human | unknown | Female | 42 | Hotel isolation | unknown | unknown | AY.122 | GK |
| hCoV-19/Egypt/CPHL-EGY21297/2021 | EPI_ISL_9047639 | 12/12/2021 | Africa / Egypt / Red sea governorate / Hurgada | Human | unknown | Female | 48 | Hotel isolation | unknown | unknown | AY.4 | GK |
| hCoV-19/Egypt/CPHL-EGY21296/2021 | EPI_ISL_9047638 | 12/12/2021 | Africa / Egypt / Red sea governorate / Hurgada | Human | unknown | Female | 53 | Hotel isolation | unknown | unknown | B.1.617.2 | GK |
| hCoV-19/Egypt/CPHL-EGY21295/2021 | EPI_ISL_9047637 | 01/12/2021 | Africa / Egypt / Red sea governorate / Hurgada | Human | unknown | Female | 57 | Hotel isolation | unknown | unknown | B.1.617.2 | GK |
| hCoV-19/Egypt/CPHL-EGY21292/2021 | EPI_ISL_9047635 | 13/12/2021 | Africa / Egypt / Red sea governorate / Safaga | Human | unknown | Male | 30 | Hotel isolation | unknown | unknown | BA.1.1 | GRA |
| hCoV-19/Egypt/CPHL-EGY21291/2021 | EPI_ISL_9047634 | 24/12/2021 | Africa / Egypt / Red sea governorate / Hurgada | Human | unknown | Female | 56 | Hotel isolation | unknown | unknown | AY.122 (consensus call) | GK |
| hCoV-19/Egypt/CPHL-EGY21288/2021 | EPI_ISL_9047633 | 02/12/2021 | Africa / Egypt / Red sea governorate / Hurgada | Human | unknown | Female | 44 | Hotel isolation | unknown | unknown | B.1.617.2 | GK |
| hCoV-19/Egypt/CPHL-EGY21287/2021 | EPI_ISL_9047632 | 02/12/2021 | Africa / Egypt / Red sea governorate / Hurgada | Human | unknown | Female | 45 | Hotel isolation | unknown | unknown | AY.46 | GK |
| hCoV-19/Egypt/CPHL-EGY21286/2021 | EPI_ISL_9047631 | 02/12/2021 | Africa / Egypt / Red sea governorate / Hurgada | Human | unknown | Female | 70 | Hotel isolation | unknown | unknown | AY.122 | O |
| hCoV-19/Egypt/CPHL-EGY21285/2021 | EPI_ISL_9047630 | 27/12/2021 | Africa / Egypt / Red sea governorate / Hurgada | Human | unknown | Female | 65 | Hotel isolation | unknown | unknown | BA.1.19 | GRA |
| hCoV-19/Egypt/CPHL-EGY21283/2021 | EPI_ISL_9047628 | 04/12/2021 | Africa / Egypt / Red sea governorate / Hurgada | Human | unknown | Female | 28 | Hotel isolation | unknown | unknown | Unassigned | GK |
| hCoV-19/Egypt/CPHL-EGY21282/2021 | EPI_ISL_9047627 | 02/12/2021 | Africa / Egypt / Red sea governorate / Hurgada | Human | unknown | Female | 24 | Hotel isolation | unknown | unknown | AY.4.2 | GV |
| hCoV-19/Egypt/CPHL-EGY21281/2021 | EPI_ISL_9047626 | 02/12/2021 | Africa / Egypt / Red sea governorate / Hurgada | Human | unknown | Female | 23 | Hotel isolation | unknown | unknown | AY.122 | GK |
| hCoV-19/Egypt/CPHL-EGY21280/2021 | EPI_ISL_9047625 | 23/12/2021 | Africa / Egypt / Red sea governorate / Hurgada | Human | unknown | Female | 36 | Hotel isolation | unknown | unknown | Unassigned | GRA |
| hCoV-19/Egypt/CPHL-EGY21279/2021 | EPI_ISL_9047624 | 12/12/2021 | Africa / Egypt / Red sea governorate / Hurgada | Human | unknown | Female | 45 | Hotel isolation | unknown | unknown | BA.1 | GRA |
| hCoV-19/Egypt/CPHL-EGY21278/2021 | EPI_ISL_9047623 | 11/12/2021 | Africa / Egypt / Red sea governorate / Hurgada | Human | unknown | Female | 35 | Hotel isolation | unknown | unknown | B.1.617.2 | GK |
| hCoV-19/Egypt/CPHL-EGY21276/2021 | EPI_ISL_9047622 | 11/12/2021 | Africa / Egypt / Red sea governorate / Hurgada | Human | unknown | Female | 63 | Hotel isolation | unknown | unknown | B.1.617.2 | GK |
| hCoV-19/Egypt/CPHL-EGY21274/2021 | EPI_ISL_9047620 | 11/12/2021 | Africa / Egypt / Red sea governorate / Hurgada | Human | unknown | Female | 58 | Hotel isolation | unknown | unknown | AY.127 | GK |
| hCoV-19/Egypt/CPHL-EGY21273/2021 | EPI_ISL_9047619 | 11/12/2021 | Africa / Egypt / Red sea governorate / Hurgada | Human | unknown | Female | 44 | Hotel isolation | unknown | unknown | AY.127 | GK |
| hCoV-19/Egypt/CPHL-EGY21270/2021 | EPI_ISL_9047617 | 10/12/2021 | Africa / Egypt / Red sea governorate / Hurgada | Human | unknown | Female | 56 | Hotel isolation | unknown | unknown | AY.43 (consensus call) | GK |
| hCoV-19/Egypt/CPHL-EGY21268/2021 | EPI_ISL_9047615 | 14/12/2021 | Africa / Egypt / Red sea governorate / Hurgada | Human | unknown | Female | 49 | Hotel isolation | unknown | unknown | B.1.617.2 | GK |
| hCoV-19/Egypt/CPHL-EGY21267/2021 | EPI_ISL_9047614 | 10/12/2021 | Africa / Egypt / Red sea governorate / Hurgada | Human | unknown | Female | 39 | Hotel isolation | unknown | unknown | B.1.617.2 | GK |
| hCoV-19/Egypt/CPHL-EGY21266/2021 | EPI_ISL_9047613 | 04/12/2021 | Africa / Egypt / Red sea governorate / Hurgada | Human | unknown | Female | 29 | Hotel isolation | unknown | unknown | B.1.617.2 | GK |
| hCoV-19/Egypt/CPHL-EGY21265/2021 | EPI_ISL_9047612 | 09/12/2021 | Africa / Egypt / Red sea governorate / Hurgada | Human | unknown | Female | 16 | Hotel isolation | unknown | unknown | B.1.617.2 | GK |
| hCoV-19/Egypt/CPHL-EGY21264/2021 | EPI_ISL_9047611 | 09/12/2021 | Africa / Egypt / Red sea governorate / Hurgada | Human | unknown | Female | 36 | Hotel isolation | unknown | unknown | B.1.617.2 | GK |
| hCoV-19/Egypt/CPHL-EGY21261/2021 | EPI_ISL_9047608 | 08/12/2021 | Africa / Egypt / Red sea governorate / Hurgada | Human | unknown | Female | 22 | Hotel isolation | unknown | unknown | B.1.617.2 | GK |
| hCoV-19/Egypt/CPHL-EGY21259/2021 | EPI_ISL_9047606 | 08/12/2021 | Africa / Egypt / Red sea governorate / Hurgada | Human | unknown | Female | 66 | Hotel isolation | unknown | unknown | Unassigned | GK |
| hCoV-19/Egypt/CPHL-EGY21258/2021 | EPI_ISL_9047605 | 07/12/2021 | Africa / Egypt / Red sea governorate / Hurgada | Human | unknown | Female | 52 | Hotel isolation | unknown | unknown | B.1.617.2 | GK |
| hCoV-19/Egypt/CPHL-EGY21257/2021 | EPI_ISL_9047604 | 17/12/2021 | Africa / Egypt / Red sea governorate / Hurgada | Human | unknown | Female | 48 | Hotel isolation | unknown | unknown | Unassigned | GK |
| hCoV-19/Egypt/CPHL-EGY21256/2021 | EPI_ISL_9047603 | 06/12/2021 | Africa / Egypt / Red sea governorate / Hurgada | Human | unknown | Female | 33 | Hotel isolation | unknown | unknown | AY.122 | GK |
| hCoV-19/Egypt/CPHL-EGY21255/2021 | EPI_ISL_9047602 | 06/12/2021 | Africa / Egypt / Red sea governorate / Hurgada | Human | unknown | Female | 44 | Hotel isolation | unknown | unknown | Unassigned | GK |
| hCoV-19/Egypt/CPHL-EGY21253/2021 | EPI_ISL_9047600 | 05/12/2021 | Africa / Egypt / Red sea governorate / Hurgada | Human | unknown | Female | 22 | Hotel isolation | unknown | unknown | B.1.617.2 | GK |
| hCoV-19/Egypt/CPHL-EGY21248/2021 | EPI_ISL_9047595 | 23/12/2021 | Africa / Egypt / Red sea governorate / Hurgada | Human | unknown | Female | 28 | Hotel isolation | unknown | unknown | Unassigned | GK |
| hCoV-19/Egypt/CPHL-EGY21244/2021 | EPI_ISL_9047591 | 19/12/2021 | Africa / Egypt / Red sea governorate / Hurgada | Human | unknown | Male | 39 | Hotel isolation | unknown | unknown | Unassigned | GK |
| hCoV-19/Egypt/CPHL-EGY21242/2021 | EPI_ISL_9047589 | 19/12/2021 | Africa / Egypt / Red sea governorate / Hurgada | Human | unknown | Male | 12 | Hotel isolation | unknown | unknown | AY.98 | GK |
| hCoV-19/Egypt/CPHL-EGY21241/2021 | EPI_ISL_9047588 | 19/12/2021 | Africa / Egypt / Red sea governorate / Hurgada | Human | unknown | Male | 71 | Hotel isolation | unknown | unknown | B.1.617.2 | GK |
| hCoV-19/Egypt/CPHL-EGY21235/2021 | EPI_ISL_9047582 | 17/12/2021 | Africa / Egypt / Red sea governorate / Hurgada | Human | unknown | Male | 77 | Hotel isolation | unknown | unknown | B.1.617.2 | GK |
| hCoV-19/Egypt/CPHL-EGY21234/2021 | EPI_ISL_9047581 | 13/12/2021 | Africa / Egypt / Red sea governorate / Hurgada | Human | unknown | Male | 59 | Hotel isolation | unknown | unknown | Unassigned | GRA |
| hCoV-19/Egypt/CPHL-EGY21232/2021 | EPI_ISL_9047579 | 01/12/2021 | Africa / Egypt / Red sea governorate / Safaga | Human | unknown | Male | 23 | Hotel isolation | unknown | unknown | Unassigned | GK |
| hCoV-19/Egypt/CPHL-EGY21231/2021 | EPI_ISL_9047578 | 01/12/2021 | Africa / Egypt / Red sea governorate / Safaga | Human | unknown | Male | 26 | Hotel isolation | unknown | unknown | B.1.617.2 | GK |
| hCoV-19/Egypt/CPHL-EGY21226/2021 | EPI_ISL_9047574 | 01/12/2021 | Africa / Egypt / Red sea governorate / Safaga | Human | unknown | Male | 66 | Hotel isolation | unknown | unknown | Unassigned | GK |
| hCoV-19/Egypt/CPHL-EGY21222/2021 | EPI_ISL_9047571 | 25/11/2021 | Africa / Egypt / Red sea governorate / Safaga | Human | unknown | Male | 29 | Hospitalized | unknown | unknown | Unassigned | G |
| hCoV-19/Egypt/CPHL-EGY21219/2021 | EPI_ISL_9047568 | 12/02/2021 | Africa / Egypt / Red sea governorate / Safaga | Human | unknown | Male | 36 | Hospitalized | unknown | unknown | Unassigned | GK |
| hCoV-19/Egypt/CPHL-EGY21217/2021 | EPI_ISL_9047566 | 12/02/2021 | Africa / Egypt / Red sea governorate / Safaga | Human | unknown | Male | 34 | Hospitalized | unknown | unknown | B.1.617.2 | GK |
| hCoV-19/Egypt/CPHL-EGY21214/2021 | EPI_ISL_9047563 | 10/12/2021 | Africa / Egypt / Red sea governorate / Safaga | Human | unknown | Male | 23 | Hospitalized | unknown | unknown | B.1.617.2 | GK |
| hCoV-19/Egypt/CPHL-EGY21213/2021 | EPI_ISL_9047562 | 08/12/2021 | Africa / Egypt / Red sea governorate / Safaga | Human | unknown | Male | 22 | Hospitalized | unknown | unknown | Unassigned | GK |
| hCoV-19/Egypt/CPHL-EGY21210/2021 | EPI_ISL_9047559 | 02/12/2021 | Africa / Egypt / Red sea governorate / Safaga | Human | unknown | Male | 45 | Hospitalized | unknown | unknown | B.1.617.2 | GK |
| hCoV-19/Egypt/CPHL-EGY21209/2021 | EPI_ISL_9047558 | 02/12/2021 | Africa / Egypt / Red sea governorate / Safaga | Human | unknown | Male | 36 | Hospitalized | unknown | unknown | B.1.617.2 | GK |
| hCoV-19/Egypt/CPHL-EGY21208/2021 | EPI_ISL_9047557 | 30/11/2021 | Africa / Egypt / Red sea governorate / Safaga | Human | unknown | Male | 25 | Hospitalized | unknown | unknown | B.1.617.2 | GK |
| hCoV-19/Egypt/CPHL-EGY21206/2021 | EPI_ISL_9047555 | 10/08/2021 | Africa / Egypt / Alexandria governorate | Human | unknown | Female | 34 | Hospitalized | unknown | unknown | B.1.617.2 | GK |
| hCoV-19/Egypt/CPHL-EGY21204/2021 | EPI_ISL_9047553 | 17/08/2021 | Africa / Egypt / Alexandria governorate | Human | unknown | Female | 39 | Hospitalized | unknown | unknown | B.1.617.2 | GK |
| hCoV-19/Egypt/CPHL-EGY21202/2021 | EPI_ISL_9047551 | 22/08/2021 | Africa / Egypt / Alexandria governorate | Human | unknown | Female | 22 | Hospitalized | unknown | unknown | B.1.617.2 | GK |
| hCoV-19/Egypt/CPHL-EGY21199/2021 | EPI_ISL_9047548 | 27/08/2021 | Africa / Egypt / Alexandria governorate | Human | unknown | Male | 38 | Hospitalized | unknown | unknown | B.1.617.2 | GK |
| hCoV-19/Egypt/CPHL-EGY21198/2021 | EPI_ISL_9047547 | 27/08/2021 | Africa / Egypt / Alexandria governorate | Human | unknown | Male | 35 | Hospitalized | unknown | unknown | Unassigned | GK |
| hCoV-19/Egypt/CPHL-EGY21196/2021 | EPI_ISL_9047545 | 10/08/2021 | Africa / Egypt / Alexandria governorate | Human | unknown | Female | 28 | Hospitalized | unknown | unknown | Unassigned | G |
| hCoV-19/Egypt/CPHL-EGY21194/2021 | EPI_ISL_9047543 | 12/11/2021 | Africa / Egypt / Red sea governorate / Safaga | Human | unknown | Male | 32 | Hospitalized | unknown | unknown | AY.127 | GK |
| hCoV-19/Egypt/CPHL-EGY21193/2021 | EPI_ISL_9047542 | 12/11/2021 | Africa / Egypt / Red sea governorate / Safaga | Human | unknown | Female | 28 | Hospitalized | unknown | unknown | B.1.617.2 | GK |
| hCoV-19/Egypt/CPHL-EGY21192/2021 | EPI_ISL_9047541 | 10/08/2021 | Africa / Egypt / Alexandria governorate | Human | unknown | Male | 15 | Hospitalized | unknown | unknown | B.1.617.2 | GK |
| hCoV-19/Egypt/CPHL-EGY21191/2021 | EPI_ISL_9047540 | 12/10/2021 | Africa / Egypt / Red sea governorate / Safaga | Human | unknown | Female | 29 | Hospitalized | unknown | unknown | B.1.617.2 | GK |
| hCoV-19/Egypt/CPHL-EGY21188/2021 | EPI_ISL_9047537 | 12/11/2021 | Africa / Egypt / Red sea governorate / Safaga | Human | unknown | Male | 32 | Hospitalized | unknown | unknown | B.1.617.2 | GK |
| hCoV-19/Egypt/CPHL-EGY21187/2021 | EPI_ISL_9047536 | 12/11/2021 | Africa / Egypt / Red sea governorate / Safaga | Human | unknown | Female | 33 | Hospitalized | unknown | unknown | AY.43 | GK |
| hCoV-19/Egypt/CPHL-EGY21185/2021 | EPI_ISL_9047534 | 14/11/2021 | Africa / Egypt / Red sea governorate / Safaga | Human | unknown | Female | 62 | Hospitalized | unknown | unknown | B.1.617.2 | GK |
| hCoV-19/Egypt/CPHL-EGY21184/2021 | EPI_ISL_9047533 | 13/11/2021 | Africa / Egypt / Red sea governorate / Safaga | Human | unknown | Female | 64 | Hospitalized | unknown | unknown | B.1.1.529 | G |
| hCoV-19/Egypt/CPHL-EGY21183/2021 | EPI_ISL_9047532 | 13/12/2021 | Africa / Egypt / Red sea governorate / Safaga | Human | unknown | Male | 45 | Hotel isolation | unknown | unknown | B.1.1.529 | GRA |
| hCoV-19/Egypt/CPHL-EGY21177/2021 | EPI_ISL_9047527 | 10/12/2021 | Africa / Egypt / Red sea governorate / Safaga | Human | unknown | Male | 45 | Hotel isolation | unknown | unknown | B.1.617.2 | GK |
| hCoV-19/Egypt/CPHL-EGY21176/2021 | EPI_ISL_9047526 | 10/12/2021 | Africa / Egypt / Red sea governorate / Safaga | Human | unknown | Female | 48 | Hotel isolation | unknown | unknown | Unassigned | GK |
| hCoV-19/Egypt/CPHL-EGY21175/2021 | EPI_ISL_9047525 | 10/12/2021 | Africa / Egypt / Red sea governorate / Safaga | Human | unknown | Male | 45 | Hotel isolation | unknown | unknown | Unassigned | G |
| hCoV-19/Egypt/CPHL-EGY21174/2021 | EPI_ISL_9047524 | 10/12/2021 | Africa / Egypt / Red sea governorate / Safaga | Human | unknown | Female | 47 | Hotel isolation | unknown | unknown | AY.43 | GK |
| hCoV-19/Egypt/CPHL-EGY21172/2021 | EPI_ISL_9047522 | 08/12/2021 | Africa / Egypt / Red sea governorate / Safaga | Human | unknown | Male | 34 | Hotel isolation | unknown | unknown | B.1.617.2 | GK |
| hCoV-19/Egypt/CPHL-EGY21171/2021 | EPI_ISL_9047521 | 08/12/2021 | Africa / Egypt / Red sea governorate / Safaga | Human | unknown | Female | 22 | Hotel isolation | unknown | unknown | B.1.617.2 | GK |
| hCoV-19/Egypt/CPHL-EGY21170/2021 | EPI_ISL_9047520 | 08/12/2021 | Africa / Egypt / Red sea governorate / Safaga | Human | unknown | Female | 22 | Hotel isolation | unknown | unknown | B.1.617.2 | GK |
| hCoV-19/Egypt/CPHL-EGY21169/2021 | EPI_ISL_9047519 | 08/12/2021 | Africa / Egypt / Red sea governorate / Safaga | Human | unknown | Male | 34 | Hotel isolation | unknown | unknown | B.1.617.2 | GK |
| hCoV-19/Egypt/CPHL-EGY21168/2021 | EPI_ISL_9047518 | 07/12/2021 | Africa / Egypt / Dammita governorate | Human | unknown | Female | 67 | Hospitalized | unknown | unknown | B.1.617.2 | GK |
| hCoV-19/Egypt/CPHL-EGY21167/2021 | EPI_ISL_9047517 | 06/12/2021 | Africa / Egypt / Red sea governorate / Safaga | Human | unknown | Male | 55 | Hotel isolation | unknown | unknown | AY.122 | GK |
| hCoV-19/Egypt/CPHL-EGY21166/2021 | EPI_ISL_9047516 | 06/12/2021 | Africa / Egypt / Red sea governorate / Safaga | Human | unknown | Male | 56 | Hotel isolation | unknown | unknown | AY.127 | GK |
| hCoV-19/Egypt/CPHL-EGY21164/2021 | EPI_ISL_9047514 | 05/12/2021 | Africa / Egypt / Red sea governorate / Safaga | Human | unknown | Male | 26 | Hotel isolation | unknown | unknown | AY.122 | GK |
| hCoV-19/Egypt/CPHL-EGY21163/2021 | EPI_ISL_9047513 | 05/12/2021 | Africa / Egypt / Red sea governorate / Safaga | Human | unknown | Female | 43 | Hotel isolation | unknown | unknown | Unassigned | GK |
| hCoV-19/Egypt/CPHL-EGY21162/2021 | EPI_ISL_9047512 | 04/12/2021 | Africa / Egypt / Red sea governorate / Safaga | Human | unknown | Male | 65 | Hotel isolation | unknown | unknown | B.1.617.2 | O |
| hCoV-19/Egypt/CPHL-EGY21161/2021 | EPI_ISL_9047511 | 04/12/2021 | Africa / Egypt / Red sea governorate / Safaga | Human | unknown | Female | 67 | Hotel isolation | unknown | unknown | Unassigned | GK |
| hCoV-19/Egypt/CPHL-EGY21159/2021 | EPI_ISL_9047509 | 04/12/2021 | Africa / Egypt / Red sea governorate / Safaga | Human | unknown | Female | 66 | Hotel isolation | unknown | unknown | Unassigned | G |
| hCoV-19/Egypt/CPHL-EGY21158/2021 | EPI_ISL_9047508 | 04/12/2021 | Africa / Egypt / Red sea governorate / Safaga | Human | unknown | Female | 48 | Hotel isolation | unknown | unknown | B.1.617.2 | GK |
| hCoV-19/Egypt/CPHL-EGY21157/2021 | EPI_ISL_9047507 | 04/12/2021 | Africa / Egypt / Red sea governorate / Safaga | Human | unknown | Female | 30 | Hotel isolation | unknown | unknown | B.1.617.2 | GK |
| hCoV-19/Egypt/CPHL-EGY21156/2021 | EPI_ISL_9047506 | 04/12/2021 | Africa / Egypt / Red sea governorate / Safaga | Human | unknown | Female | 56 | Hotel isolation | unknown | unknown | B.1.617.2 | GK |
| hCoV-19/Egypt/CPHL-EGY21155/2021 | EPI_ISL_9047505 | 04/12/2021 | Africa / Egypt / Dammita governorate | Human | unknown | Female | 56 | Hospitalized | unknown | unknown | B.1.617.2 | GK |
| hCoV-19/Egypt/CPHL-EGY21154/2021 | EPI_ISL_9047504 | 20/11/2021 | Africa / Egypt / Red sea governorate / Safaga | Human | unknown | Male | 57 | Hotel isolation | unknown | unknown | B.1.617.2 | GK |
| hCoV-19/Egypt/CPHL-EGY21151/2021 | EPI_ISL_9047501 | 25/10/2021 | Africa / Egypt / Red sea governorate / Safaga | Human | unknown | Female | 44 | Hotel isolation | unknown | unknown | AY.122 | GK |
| hCoV-19/Egypt/CPHL-EGY21150/2021 | EPI_ISL_9047500 | 25/10/2021 | Africa / Egypt / Red sea governorate / Safaga | Human | unknown | Female | 45 | Hotel isolation | unknown | unknown | Unassigned | GK |
| hCoV-19/Egypt/CPHL-EGY21149/2021 | EPI_ISL_9047499 | 13/12/2021 | Africa / Egypt / Red sea governorate / Safaga | Human | unknown | Male | 45 | Hotel isolation | unknown | unknown | B.1.1.529 | GRA |
| hCoV-19/Egypt/CPHL-EGY21147/2021 | EPI_ISL_9047497 | 25/10/2021 | Africa / Egypt / Red sea governorate / Safaga | Human | unknown | Female | 12 | Hotel isolation | unknown | unknown | B.1.617.2 | GK |
| hCoV-19/Egypt/CPHL-EGY21145/2021 | EPI_ISL_9047495 | 25/10/2021 | Africa / Egypt / Red sea governorate / Safaga | Human | unknown | Female | 56 | Hotel isolation | unknown | unknown | B.1.617.2 | GK |
| hCoV-19/Egypt/CPHL-EGY21141/2021 | EPI_ISL_9047491 | 05/10/2021 | Africa / Egypt / Red sea governorate / Safaga | Human | unknown | Male | 66 | Hotel isolation | unknown | unknown | B.1.617.2 | GK |
| hCoV-19/Egypt/CPHL-EGY21138/2021 | EPI_ISL_9047488 | 01/12/2021 | Africa / Egypt / Cairo | Human | unknown | Male | 25 | Hospitalized | unknown | unknown | Unassigned | GRA |
| hCoV-19/Egypt/CPHL-EGY21137/2021 | EPI_ISL_9047487 | 14/12/2021 | Africa / Egypt / Red sea governorate / Safaga | Human | unknown | Male | 56 | Hotel isolation | unknown | unknown | B.1.617.2 | GK |
| hCoV-19/Egypt/CPHL-EGY21136/2021 | EPI_ISL_9047486 | 13/12/2021 | Africa / Egypt / Red sea governorate / Safaga | Human | unknown | Male | 42 | Hotel isolation | unknown | unknown | Unassigned | GK |
| hCoV-19/Egypt/CPHL-EGY21128/2021 | EPI_ISL_9047480 | 15/12/2021 | Africa / Egypt / Red sea governorate / Safaga | Human | unknown | Male | 22 | Hotel isolation | unknown | unknown | Unassigned | GK |
| hCoV-19/Egypt/CPHL-EGY21122/2021 | EPI_ISL_9047474 | 05/12/2021 | Africa / Egypt / Red sea governorate / Safaga | Human | unknown | Female | 39 | Hotel isolation | unknown | unknown | B.1.617.2 | GK |
| hCoV-19/Egypt/CPHL-EGY21119/2021 | EPI_ISL_9047471 | 05/12/2021 | Africa / Egypt / Red sea governorate / Safaga | Human | unknown | Male | 42 | Hotel isolation | unknown | unknown | AY.4 | GK |
| hCoV-19/Egypt/CPHL-EGY21117/2021 | EPI_ISL_9047469 | 05/12/2021 | Africa / Egypt / Red sea governorate / Safaga | Human | unknown | Male | 6 | Hotel isolation | unknown | unknown | Unassigned | GK |
| hCoV-19/Egypt/CPHL-EGY21116/2021 | EPI_ISL_9047468 | 05/12/2021 | Africa / Egypt / Red sea governorate / Safaga | Human | unknown | Female | 48 | Hotel isolation | unknown | unknown | Unassigned | GK |
| hCoV-19/Egypt/CPHL-EGY21114/2021 | EPI_ISL_9047466 | 23/12/2021 | Africa / Egypt / Red sea governorate / Safaga | Human | unknown | Male | 30 | Hotel isolation | unknown | unknown | B.1.617.2 | GK |
| hCoV-19/Egypt/CPHL-EGY21112/2021 | EPI_ISL_9047464 | 19/12/2021 | Africa / Egypt / Red sea governorate / Safaga | Human | unknown | Male | 58 | Hotel isolation | unknown | unknown | B.1.617.2 | GK |
| hCoV-19/Egypt/CPHL-EGY21109/2021 | EPI_ISL_9047461 | 19/12/2021 | Africa / Egypt / Red sea governorate / Safaga | Human | unknown | Female | 36 | Hotel isolation | unknown | unknown | B.1.617.2 | GK |
| hCoV-19/Egypt/CPHL-EGY21105/2021 | EPI_ISL_9047457 | 18/12/2021 | Africa / Egypt / Red sea governorate / Safaga | Human | unknown | Male | 6 | Hotel isolation | unknown | unknown | Unassigned | GR |
| hCoV-19/Egypt/CPHL-EGY21102/2021 | EPI_ISL_9047454 | 17/12/2021 | Africa / Egypt / Red sea governorate / Safaga | Human | unknown | Female | 67 | Hotel isolation | unknown | unknown | Unassigned | G |
| hCoV-19/Egypt/CPHL-EGY21098/2021 | EPI_ISL_9047450 | 16/12/2021 | Africa / Egypt / Red sea governorate / Safaga | Human | unknown | Female | 78 | Hotel isolation | unknown | unknown | AY.127 | GK |
| hCoV-19/Egypt/CPHL-EGY21095/2021 | EPI_ISL_9047447 | 14/12/2021 | Africa / Egypt / Red sea governorate / Safaga | Human | unknown | Male | 31 | Hotel isolation | unknown | unknown | B.1.617.2 | GK |
| hCoV-19/Egypt/CPHL-EGY21094/2021 | EPI_ISL_9047446 | 14/12/2021 | Africa / Egypt / Red sea governorate / Safaga | Human | unknown | Male | 23 | Hotel isolation | unknown | unknown | B.1.617.2 | GK |
| hCoV-19/Egypt/CPHL-EGY21093/2021 | EPI_ISL_9047445 | 14/12/2021 | Africa / Egypt / Red sea governorate / Safaga | Human | unknown | Female | 32 | Hotel isolation | unknown | unknown | AY.122 | GK |
| hCoV-19/Egypt/CPHL-EGY21092/2021 | EPI_ISL_9047444 | 14/12/2021 | Africa / Egypt / Cairo | Human | unknown | Male | 23 | Hospitalized | unknown | unknown | BA.1 | GRA |
| hCoV-19/Egypt/CPHL-EGY21091/2021 | EPI_ISL_9047443 | 14/12/2021 | Africa / Egypt / Red sea governorate / Safaga | Human | unknown | Female | 34 | Hotel isolation | unknown | unknown | AY.46 | GK |
| hCoV-19/Egypt/CPHL-EGY21088/2021 | EPI_ISL_9047440 | 15/12/2021 | Africa / Egypt / Red sea governorate / Safaga | Human | unknown | Female | 45 | Hotel isolation | unknown | unknown | AY.126 | GK |
| hCoV-19/Egypt/CPHL-EGY21086/2021 | EPI_ISL_9047438 | 13/12/2021 | Africa / Egypt / Red sea governorate / Safaga | Human | unknown | Male | 24 | Hospitalized | unknown | unknown | AY.122 (consensus call) | GRA |
| hCoV-19/Egypt/CPHL-EGY21085/2021 | EPI_ISL_9047437 | 13/12/2021 | Africa / Egypt / Red sea governorate / Safaga | Human | unknown | Male | 22 | Hotel isolation | unknown | unknown | B.1.617.2 | GK |
| hCoV-19/Egypt/CPHL-EGY21082/2021 | EPI_ISL_9047434 | 19/12/2021 | Africa / Egypt / Red sea governorate / Safaga | Human | unknown | Female | 44 | Hotel isolation | unknown | unknown | B.1.1.529 | GRA |
| hCoV-19/Egypt/CPHL-EGY21081/2021 | EPI_ISL_9047433 | 18/12/2021 | Africa / Egypt / Red sea governorate / Safaga | Human | unknown | Male | 56 | Hotel isolation | unknown | unknown | B.1.617.2 | GK |
| hCoV-19/Egypt/CPHL-EGY21079/2021 | EPI_ISL_9047431 | 16/12/2021 | Africa / Egypt / Red sea governorate / Safaga | Human | unknown | Female | 67 | Hotel isolation | unknown | unknown | AY.46 | GK |
| hCoV-19/Egypt/CPHL-EGY21078/2021 | EPI_ISL_9047430 | 15/12/2021 | Africa / Egypt / Red sea governorate / Safaga | Human | unknown | Female | 45 | Hotel isolation | unknown | unknown | AY.112 | GK |
| hCoV-19/Egypt/CPHL-EGY21077/2021 | EPI_ISL_9047429 | 14/12/2021 | Africa / Egypt / Red sea governorate / Safaga | Human | unknown | Male | 34 | Hotel isolation | unknown | unknown | B.1.617.2 | GK |
| hCoV-19/Egypt/CPHL-EGY21076/2021 | EPI_ISL_9047428 | 13/12/2021 | Africa / Egypt / Red sea governorate / Safaga | Human | unknown | Female | 23 | Hotel isolation | unknown | unknown | B.1.617.2 | GK |
| hCoV-19/Egypt/CPHL-EGY21075/2021 | EPI_ISL_9047427 | 13/12/2021 | Africa / Egypt / Red sea governorate / Safaga | Human | unknown | Male | 66 | Hotel isolation | unknown | unknown | B.1.617.2 | GK |
| hCoV-19/Egypt/CPHL-EGY21074/2021 | EPI_ISL_9047426 | 18/12/2021 | Africa / Egypt / Red sea governorate / Safaga | Human | unknown | Female | 72 | Hotel isolation | unknown | unknown | B.1.617.2 | GK |
| hCoV-19/Egypt/CPHL-EGY21073/2021 | EPI_ISL_9047425 | 18/12/2021 | Africa / Egypt / Red sea governorate / Safaga | Human | unknown | Male | 33 | Hotel isolation | unknown | unknown | B.1.617.2 | GK |
| hCoV-19/Egypt/CPHL-EGY21070/2021 | EPI_ISL_9047422 | 17/12/2021 | Africa / Egypt / Red sea governorate / Safaga | Human | unknown | Male | 33 | Hotel isolation | unknown | unknown | B.1.617.2 | GK |
| hCoV-19/Egypt/CPHL-EGY21069/2021 | EPI_ISL_9047421 | 17/12/2021 | Africa / Egypt / Red sea governorate / Safaga | Human | unknown | Female | 67 | Hotel isolation | unknown | unknown | B.1.617.2 | GK |
| hCoV-19/Egypt/CPHL-EGY21068/2021 | EPI_ISL_9047420 | 17/12/2021 | Africa / Egypt / Red sea governorate / Safaga | Human | unknown | Female | 56 | Hotel isolation | unknown | unknown | B.1.617.2 | GK |
| hCoV-19/Egypt/CPHL-EGY21067/2021 | EPI_ISL_9047419 | 17/12/2021 | Africa / Egypt / Red sea governorate / Safaga | Human | unknown | Male | 50 | Hotel isolation | unknown | unknown | B.1.617.2 | GK |
| hCoV-19/Egypt/CPHL-EGY21066/2021 | EPI_ISL_9047418 | 17/12/2021 | Africa / Egypt / Red sea governorate / Safaga | Human | unknown | Female | 30 | Hotel isolation | unknown | unknown | AY.127 | GK |
| hCoV-19/Egypt/CPHL-EGY21065/2021 | EPI_ISL_9047417 | 16/12/2021 | Africa / Egypt / Red sea governorate / Safaga | Human | unknown | Female | 40 | Hotel isolation | unknown | unknown | B.1.617.2 | GK |
| hCoV-19/Egypt/CPHL-EGY21064/2021 | EPI_ISL_9047416 | 01/12/2021 | Africa / Egypt / Cairo | Human | unknown | Female | 52 | Hospitalized | unknown | unknown | BA.1 | GRA |
| hCoV-19/Egypt/CPHL-EGY21063/2021 | EPI_ISL_9047415 | 15/12/2021 | Africa / Egypt / Red sea governorate / Safaga | Human | unknown | Female | 34 | Hotel isolation | unknown | unknown | B.1.617.2 | GK |
| hCoV-19/Egypt/CPHL-EGY21062/2021 | EPI_ISL_9047414 | 15/12/2021 | Africa / Egypt / Red sea governorate / Safaga | Human | unknown | Male | 48 | Hotel isolation | unknown | unknown | B.1.617.2 | GK |
| hCoV-19/Egypt/CPHL-EGY21061/2021 | EPI_ISL_9047413 | 15/12/2021 | Africa / Egypt / Red sea governorate / Safaga | Human | unknown | Male | 33 | Hotel isolation | unknown | unknown | B.1.1.529 | GRA |
| hCoV-19/Egypt/CPHL-EGY21060/2021 | EPI_ISL_9047412 | 14/12/2021 | Africa / Egypt / Red sea governorate / Safaga | Human | unknown | Female | 26 | Hotel isolation | unknown | unknown | AY.106 | GK |
| hCoV-19/Egypt/CPHL-EGY21059/2021 | EPI_ISL_9047411 | 14/12/2021 | Africa / Egypt / Red sea governorate / Safaga | Human | unknown | Female | 24 | Hotel isolation | unknown | unknown | B.1.617.2 | GK |
| hCoV-19/Egypt/CPHL-EGY21058/2021 | EPI_ISL_9047410 | 14/12/2021 | Africa / Egypt / Red sea governorate / Safaga | Human | unknown | Female | 22 | Hotel isolation | unknown | unknown | B.1.617.2 | GK |
| hCoV-19/Egypt/CPHL-EGY21057/2021 | EPI_ISL_9047409 | 14/12/2021 | Africa / Egypt / Red sea governorate / Safaga | Human | unknown | Male | 5 | Hotel isolation | unknown | unknown | B.1.617.2 | GK |
| hCoV-19/Egypt/CPHL-EGY21056/2021 | EPI_ISL_9047408 | 14/12/2021 | Africa / Egypt / Red sea governorate / Safaga | Human | unknown | Female | 25 | Hotel isolation | unknown | unknown | B.1.617.2 | GK |
| hCoV-19/Egypt/CPHL-EGY21054/2021 | EPI_ISL_9047406 | 13/12/2021 | Africa / Egypt / Red sea governorate / Safaga | Human | unknown | Male | 39 | Hotel isolation | unknown | unknown | AY.34 | GK |
| hCoV-19/Egypt/CPHL-EGY21053/2021 | EPI_ISL_9047405 | 06/12/2021 | Africa / Egypt / Cairo / Shoubrai | Human | unknown | Male | 52 | Hospitalized | unknown | unknown | BA.1 | GRA |
| hCoV-19/Egypt/CPHL-EGY21052/2021 | EPI_ISL_9047404 | 06/12/2021 | Africa / Egypt / Cairo / Shoubrai | Human | unknown | Male | 44 | Hospitalized | unknown | unknown | BA.1 | GRA |
| hCoV-19/Egypt/CPHL-EGY21051/2021 | EPI_ISL_9047403 | 06/12/2021 | Africa / Egypt / Cairo / Shoubrai | Human | unknown | Male | 46 | Hospitalized | unknown | unknown | BA.1.21 | GRA |
| hCoV-19/Egypt/CPHL-EGY21050/2021 | EPI_ISL_9047402 | 06/12/2021 | Africa / Egypt / Cairo / Shoubrai | Human | unknown | Male | 42 | Hospitalized | unknown | unknown | BA.1.17.2 | GRA |
| hCoV-19/Egypt/CPHL-EGY21049/2021 | EPI_ISL_9047401 | 06/12/2021 | Africa / Egypt / Cairo / Shoubrai | Human | unknown | Male | 45 | Hospitalized | unknown | unknown | BA.1.17 | GRA |
| hCoV-19/Egypt/CPHL-EGY21047/2021 | EPI_ISL_9047399 | 13/12/2021 | Africa / Egypt / Red sea governorate / Safaga | Human | unknown | Male | 7 | Hospitalized | unknown | unknown | C.17 | GR |
| hCoV-19/Egypt/CPHL-EGY21046/2021 | EPI_ISL_9047398 | 13/12/2021 | Africa / Egypt / Menofia governorate / Shebin el kom | Human | unknown | Female | 50 | Hospitalized | unknown | unknown | B.1 | G |
| hCoV-19/Egypt/CPHL-EGY21044/2021 | EPI_ISL_9047396 | 13/12/2021 | Africa / Egypt / Menofia governorate / Shebin el kom | Human | unknown | Female | 22 | Hospitalized | unknown | unknown | B.1.1.7 | GRA |
| hCoV-19/Egypt/CPHL-EGY21043/2021 | EPI_ISL_9047395 | 13/12/2021 | Africa / Egypt / Menofia governorate / Shebin el kom | Human | unknown | Male | 62 | Hospitalized | unknown | unknown | C.36.3 | GR |
| hCoV-19/Egypt/CPHL-EGY21040/2021 | EPI_ISL_9047392 | 12/12/2021 | Africa / Egypt / Menofia governorate / Shebin el kom | Human | unknown | Male | 72 | Hospitalized | unknown | unknown | C.36.3 | GR |
| hCoV-19/Egypt/CPHL-EGY21039/2021 | EPI_ISL_9047391 | 12/12/2021 | Africa / Egypt / Menofia governorate / Shebin el kom | Human | unknown | Female | 15 | Hospitalized | unknown | unknown | C.36.3 | GR |
| hCoV-19/Egypt/CPHL-EGY21038/2021 | EPI_ISL_9047390 | 12/12/2021 | Africa / Egypt / Menofia governorate / Shebin el kom | Human | unknown | Male | 25 | Hospitalized | unknown | unknown | C.36.3 | GR |
| hCoV-19/Egypt/CPHL-EGY21037/2021 | EPI_ISL_9047389 | 12/12/2021 | Africa / Egypt / Menofia governorate / Shebin el kom | Human | unknown | Male | 40 | Hospitalized | unknown | unknown | AY.122 | GK |
| hCoV-19/Egypt/CPHL-EGY21035/2021 | EPI_ISL_9047387 | 12/12/2021 | Africa / Egypt / Menofia governorate / Shebin el kom | Human | unknown | Male | 52 | Hospitalized | unknown | unknown | AY.43 | GK |
| hCoV-19/Egypt/CPHL-EGY21033/2021 | EPI_ISL_9047385 | 12/12/2021 | Africa / Egypt / Menofia governorate / Shebin el kom | Human | unknown | Female | 50 | Hospitalized | unknown | unknown | B.1.1.529 | GRA |
| hCoV-19/Egypt/CPHL-EGY21029/2021 | EPI_ISL_9047381 | 04/12/2021 | Africa / Egypt / Menofia governorate / Shebin el kom | Human | unknown | Female | 25 | Hospitalized | unknown | unknown | B.1.617.2 | GK |
| hCoV-19/Egypt/CPHL-EGY21028/2021 | EPI_ISL_9047380 | 04/12/2021 | Africa / Egypt / Menofia governorate / Shebin el kom | Human | unknown | Male | 69 | Hospitalized | unknown | unknown | B.1.1.529 | GR |
| hCoV-19/Egypt/CPHL-EGY21025/2021 | EPI_ISL_9047377 | 03/12/2021 | Africa / Egypt / Menofia governorate / Shebin el kom | Human | unknown | Male | 55 | Hospitalized | unknown | unknown | B.1.617.2 | GK |
| hCoV-19/Egypt/CPHL-EGY21021/2021 | EPI_ISL_9047373 | 01/12/2021 | Africa / Egypt / Menofia governorate / Shebin el kom | Human | unknown | Female | 32 | Hospitalized | unknown | unknown | AY.122 | GK |
| hCoV-19/Egypt/CPHL-EGY21019/2021 | EPI_ISL_9047371 | 01/12/2021 | Africa / Egypt / Menofia governorate / Shebin el kom | Human | unknown | Female | 45 | Hospitalized | unknown | unknown | B.1.617.2 | GK |
| hCoV-19/Egypt/CPHL-EGY21018/2021 | EPI_ISL_9047370 | 01/12/2021 | Africa / Egypt / Menofia governorate / Shebin el kom | Human | unknown | Female | 68 | Hospitalized | unknown | unknown | B.1.617.2 | GK |
| hCoV-19/Egypt/CPHL-EGY21013/2021 | EPI_ISL_9047365 | 01/12/2021 | Africa / Egypt / Menofia governorate / Shebin el kom | Human | unknown | Male | 39 | Hospitalized | unknown | unknown | AY.57 | GK |
| hCoV-19/Egypt/CPHL-EGY21010/2021 | EPI_ISL_9047362 | 01/12/2021 | Africa / Egypt / Menofia governorate / Shebin el kom | Human | unknown | Female | 45 | Hospitalized | unknown | unknown | B.1.617.2 | GK |
| hCoV-19/Egypt/CPHL-EGY21006/2021 | EPI_ISL_9047359 | 01/12/2021 | Africa / Egypt / Dammita governorate | Human | unknown | Female | 44 | Hospitalized | unknown | unknown | BA.1 | GRA |
| hCoV-19/Egypt/CPHL-EGY21005/2021 | EPI_ISL_9047358 | 09/12/2021 | Africa / Egypt / Cairo | Human | unknown | Male | 45 | Hospitalized | unknown | unknown | BA.1.14 | GRA |
| hCoV-19/Egypt/CPHL-EGY21004/2021 | EPI_ISL_9047357 | 07/12/2021 | Africa / Egypt / Gharbia / Tanta | Human | unknown | Male | 41 | Hospitalized | unknown | unknown | BA.1 | GRA |
| hCoV-19/Egypt/CPHL-EGY21003/2021 | EPI_ISL_9047356 | 07/12/2021 | Africa / Egypt / Cairo | Human | unknown | Male | 48 | Hospitalized | unknown | unknown | BA.1 | GRA |
| hCoV-19/Egypt/CPHL-EGY21002/2021 | EPI_ISL_9047355 | 05/12/2021 | Africa / Egypt / Cairo | Human | unknown | Male | 50 | Hospitalized | unknown | unknown | BA.1.17.2 | GRA |
| hCoV-19/Egypt/CPHL-EGY21001/2021 | EPI_ISL_9047354 | 09/12/2021 | Africa / Egypt / Cairo | Human | unknown | Female | 60 | Hospitalized | unknown | unknown | BA.1 | GRA |
| hCoV-19/Egypt/NRC-1483/2021 | EPI_ISL_8267202 | 22/06/2021 | Africa / Egypt | Human | unknown | unknown | unknown | unknown | unknown | unknown | B.1.617.2 | GK |
| hCoV-19/Egypt/CPHL-EGY21072/2021 | EPI_ISL_9047424 | 17/12/2021 | Africa / Egypt / Red sea governorate / Safaga | Human | unknown | Female | 38 | Hotel isolation | unknown | unknown | B.1.1.529 | GRA |
| hCoV-19/Egypt/CPHL-EGY21110/2021 | EPI_ISL_9047462 | 19/12/2021 | Africa / Egypt / Red sea governorate / Safaga | Human | unknown | Female | 34 | Hotel isolation | unknown | unknown | AY.126 | GK |
| hCoV-19/Egypt/CPHL-EGY21414/2022 | EPI_ISL_9047745 | 02/01/2022 | Africa / Egypt / Red sea governorate / Hurgada | Human | unknown | Female | 36 | Hotel isolation | unknown | unknown | B.1.617.2 | GK |
| hCoV-19/Egypt/CPHL-EGY21418/2022 | EPI_ISL_9047749 | 02/01/2022 | Africa / Egypt / Red sea governorate / Hurgada | Human | unknown | Female | 26 | Hotel isolation | unknown | unknown | B.1.617.2 | GK |
| hCoV-19/Egypt/CPHL-EGY21011/2021 | EPI_ISL_9047363 | 01/12/2021 | Africa / Egypt / Menofia governorate / Shebin el kom | Human | unknown | Female | 46 | Hospitalized | unknown | unknown | B.1.617.2 | GK |
| hCoV-19/Egypt/CPHL-EGY21344/2021 | EPI_ISL_9047681 | 30/12/2021 | Africa / Egypt / Red sea governorate / Hurgada | Human | unknown | Female | 39 | Hotel isolation | unknown | unknown | B.1.617.2 | GK |
| hCoV-19/Egypt/CPHL-EGY21310/2021 | EPI_ISL_9047650 | 27/12/2021 | Africa / Egypt / Red sea governorate / Hurgada | Human | unknown | Male | 42 | Hotel isolation | unknown | unknown | AY.43 | GK |
| hCoV-19/Egypt/CPHL-EGY21089/2021 | EPI_ISL_9047441 | 05/12/2021 | Africa / Egypt / Cairo | Human | unknown | Male | 59 | Hospitalized | unknown | unknown | B.1.1.529 | GRA |
| hCoV-19/Egypt/CPHL-EGY21090/2021 | EPI_ISL_9047442 | 14/12/2021 | Africa / Egypt / Cairo | Human | unknown | Female | 55 | Hotel isolation | unknown | unknown | B.1.617.2 | GK |
| hCoV-19/Egypt/USC-3/2021 | EPI_ISL_8469597 | 2021-01 | Africa / Egypt / Cairo | Human | unknown | unknown | unknown | unknown | unknown | unknown | Unassigned | O |
| hCoV-19/Egypt/ARMY-ECRRM0087/2021 | EPI_ISL_14593733 | 19/12/2021 | Africa / Egypt / Cairo | Human | unknown | Female | 32 | Hospitalized | Sinopharm | unknown | C.17 | GR |
| hCoV-19/Egypt/CPHL-EGY22637/2022 | EPI_ISL_16299749 | 14/09/2022 | Africa / Egypt | Human | unknown | unknown | unknown | non hospitalized | unknown | unknown | BA.5.2 | GRA |
| hCoV-19/Egypt/USC-2/2021 | EPI_ISL_8466472 | 2021-01 | Africa / Egypt / Cairo | Human | unknown | unknown | unknown | unknown | unknown | unknown | Unassigned | O |
| hCoV-19/Egypt/USC-1/2021 | EPI_ISL_8464608 | 2021-01 | Africa / Egypt / Cairo | Human | unknown | unknown | unknown | unknown | unknown | unknown | Unassigned | O |
| hCoV-19/Egypt/ARMY-BPD0013/2022 | EPI_ISL_15932106 | 09/02/2022 | Africa / Egypt / Cairo | Human | unknown | Male | 23 | unknown | Sinopharm | unknown | BA.1.1 (consensus call) | GRA |
| hCoV-19/Egypt/CPHL-EGY21118/2021 | EPI_ISL_9047470 | 05/12/2021 | Africa / Egypt / Red sea governorate / Safaga | Human | unknown | Male | 45 | Hotel isolation | unknown | unknown | B.1.617.2 | GK |
| hCoV-19/Egypt/CPHL-EGY21263/2021 | EPI_ISL_9047610 | 06/12/2021 | Africa / Egypt / Red sea governorate / Hurgada | Human | unknown | Female | 28 | Hotel isolation | unknown | unknown | B.1.617.2 | GK |
| hCoV-19/Egypt/CPHL-EGY21284/2021 | EPI_ISL_9047629 | 02/12/2021 | Africa / Egypt / Red sea governorate / Hurgada | Human | unknown | Female | 39 | Hotel isolation | unknown | unknown | B.1.617.2 | GK |
| hCoV-19/Egypt/UC-003/2021 | EPI_ISL_16871428 | 12/02/2021 | Africa / Egypt / Giza | Human | unknown | unknown | unknown | unknown | unknown | unknown | B.1.617.2 | GK |
| hCoV-19/Egypt/UC-004/2021 | EPI_ISL_16871427 | 23/02/2021 | Africa / Egypt / Giza | Human | unknown | unknown | unknown | unknown | unknown | unknown | B.1.617.2 | GK |
| hCoV-19/Egypt/UC-005/2021 | EPI_ISL_16871426 | 05/03/2021 | Africa / Egypt / Giza | Human | unknown | unknown | unknown | unknown | unknown | unknown | B.1.617.2 | GK |
| hCoV-19/Egypt/UC-007/2021 | EPI_ISL_16871424 | 23/04/2021 | Africa / Egypt / Giza | Human | unknown | unknown | unknown | unknown | unknown | unknown | B.1.617.2 | GK |
| hCoV-19/Egypt/UC-009/2021 | EPI_ISL_16871423 | 11/05/2021 | Africa / Egypt / Giza | Human | unknown | unknown | unknown | unknown | unknown | unknown | B.1.617.2 | GK |
| hCoV-19/Egypt/UC-010/2021 | EPI_ISL_16871422 | 21/05/2021 | Africa / Egypt / Giza | Human | unknown | unknown | unknown | unknown | unknown | unknown | B.1.617.2 | GK |
| hCoV-19/Egypt/UC-011/2021 | EPI_ISL_16871421 | 03/06/2021 | Africa / Egypt / Giza | Human | unknown | unknown | unknown | unknown | unknown | unknown | B.1.617.2 | GK |
| hCoV-19/Egypt/UC-013/2021 | EPI_ISL_16871419 | 26/07/2021 | Africa / Egypt / Giza | Human | unknown | unknown | unknown | unknown | unknown | unknown | B.1.617.2 | GK |
| hCoV-19/Egypt/UC-014/2021 | EPI_ISL_16871418 | 28/07/2021 | Africa / Egypt / Giza | Human | unknown | unknown | unknown | unknown | unknown | unknown | B.1.617.2 | GK |
| hCoV-19/Egypt/UC-015/2021 | EPI_ISL_16871417 | 09/08/2021 | Africa / Egypt / Giza | Human | unknown | unknown | unknown | unknown | unknown | unknown | B.1.617.2 | GK |
| hCoV-19/Egypt/CPHL-EGY22735/2022 | EPI_ISL_16299843 | 21/11/2022 | Africa / Egypt | Human | unknown | unknown | unknown | non hospitalized | unknown | unknown | BA.2 | GR |
| hCoV-19/Egypt/CPHL-EGY22733/2022 | EPI_ISL_16299842 | 23/08/2022 | Africa / Egypt | Human | unknown | unknown | unknown | non hospitalized | unknown | unknown | BA.2 | GRA |
| hCoV-19/Egypt/CPHL-EGY22732/2022 | EPI_ISL_16299841 | 23/08/2022 | Africa / Egypt | Human | unknown | unknown | unknown | non hospitalized | unknown | unknown | BA.5.2 | GRA |
| hCoV-19/Egypt/CPHL-EGY22731/2022 | EPI_ISL_16299840 | 23/08/2022 | Africa / Egypt | Human | unknown | unknown | unknown | non hospitalized | unknown | unknown | BA.5.1 | GRA |
| hCoV-19/Egypt/CPHL-EGY22730/2022 | EPI_ISL_16299839 | 23/08/2022 | Africa / Egypt | Human | unknown | unknown | unknown | non hospitalized | unknown | unknown | BA.5.2 | GRA |
| hCoV-19/Egypt/CPHL-EGY22729/2022 | EPI_ISL_16299838 | 23/08/2022 | Africa / Egypt | Human | unknown | unknown | unknown | non hospitalized | unknown | unknown | BA.5.2 | GRA |
| hCoV-19/Egypt/CPHL-EGY22728/2022 | EPI_ISL_16299837 | 23/08/2022 | Africa / Egypt | Human | unknown | unknown | unknown | non hospitalized | unknown | unknown | BA.5.2 | GRA |
| hCoV-19/Egypt/CPHL-EGY22727/2022 | EPI_ISL_16299836 | 23/08/2022 | Africa / Egypt | Human | unknown | unknown | unknown | non hospitalized | unknown | unknown | BA.5.2 | GRA |
| hCoV-19/Egypt/CPHL-EGY22726/2022 | EPI_ISL_16299835 | 17/08/2022 | Africa / Egypt | Human | unknown | unknown | unknown | non hospitalized | unknown | unknown | BA.5.2 | GRA |
| hCoV-19/Egypt/CPHL-EGY22725/2022 | EPI_ISL_16299834 | 23/11/2022 | Africa / Egypt | Human | unknown | unknown | unknown | non hospitalized | unknown | unknown | BA.5.2.20 | GRA |
| hCoV-19/Egypt/CPHL-EGY22724/2022 | EPI_ISL_16299833 | 16/11/2022 | Africa / Egypt | Human | unknown | unknown | unknown | non hospitalized | unknown | unknown | BN.1.3.1 | GRA |
| hCoV-19/Egypt/CPHL-EGY22723/2022 | EPI_ISL_16299832 | 17/08/2022 | Africa / Egypt | Human | unknown | unknown | unknown | non hospitalized | unknown | unknown | BQ.1.1 | GRA |
| hCoV-19/Egypt/CPHL-EGY22722/2022 | EPI_ISL_16299831 | 17/08/2022 | Africa / Egypt | Human | unknown | unknown | unknown | non hospitalized | unknown | unknown | BA.5.3.1 | GRA |
| hCoV-19/Egypt/CPHL-EGY22721/2022 | EPI_ISL_16299830 | 18/08/2022 | Africa / Egypt | Human | unknown | unknown | unknown | non hospitalized | unknown | unknown | BA.5.2.1 | GRA |
| hCoV-19/Egypt/CPHL-EGY22720/2022 | EPI_ISL_16299829 | 18/08/2022 | Africa / Egypt | Human | unknown | unknown | unknown | non hospitalized | unknown | unknown | BA.5.2 | GRA |
| hCoV-19/Egypt/CPHL-EGY22719/2022 | EPI_ISL_16299828 | 18/08/2022 | Africa / Egypt | Human | unknown | unknown | unknown | non hospitalized | unknown | unknown | BA.5.2 | GRA |
| hCoV-19/Egypt/CPHL-EGY22718/2022 | EPI_ISL_16299827 | 18/08/2022 | Africa / Egypt | Human | unknown | unknown | unknown | non hospitalized | unknown | unknown | BA.5.3.1 | GRA |
| hCoV-19/Egypt/CPHL-EGY22717/2022 | EPI_ISL_16299826 | 13/11/2022 | Africa / Egypt | Human | unknown | unknown | unknown | non hospitalized | unknown | unknown | BA.5.2 | GRA |
| hCoV-19/Egypt/CPHL-EGY22716/2022 | EPI_ISL_16299825 | 17/11/2022 | Africa / Egypt | Human | unknown | unknown | unknown | non hospitalized | unknown | unknown | BF.7 | GRA |
| hCoV-19/Egypt/CPHL-EGY22715/2022 | EPI_ISL_16299824 | 13/11/2022 | Africa / Egypt | Human | unknown | unknown | unknown | non hospitalized | unknown | unknown | BQ.1.5 | GRA |
| hCoV-19/Egypt/CPHL-EGY22714/2022 | EPI_ISL_16299823 | 20/11/2022 | Africa / Egypt | Human | unknown | unknown | unknown | non hospitalized | unknown | unknown | XBB.1.4 | GRA |
| hCoV-19/Egypt/CPHL-EGY22713/2022 | EPI_ISL_16299822 | 20/11/2022 | Africa / Egypt | Human | unknown | unknown | unknown | non hospitalized | unknown | unknown | BQ.1.18 | GRA |
| hCoV-19/Egypt/CPHL-EGY22712/2022 | EPI_ISL_16299821 | 20/11/2022 | Africa / Egypt | Human | unknown | unknown | unknown | non hospitalized | unknown | unknown | BA.4.6.3 | GRA |
| hCoV-19/Egypt/CPHL-EGY22711/2022 | EPI_ISL_16299820 | 21/11/2022 | Africa / Egypt | Human | unknown | unknown | unknown | non hospitalized | unknown | unknown | BA.1.1 | GRA |
| hCoV-19/Egypt/CPHL-EGY22710/2022 | EPI_ISL_16299819 | 23/11/2022 | Africa / Egypt | Human | unknown | unknown | unknown | non hospitalized | unknown | unknown | CH.1.1.11 | GRA |
| hCoV-19/Egypt/CPHL-EGY22709/2022 | EPI_ISL_16299818 | 27/11/2022 | Africa / Egypt | Human | unknown | unknown | unknown | non hospitalized | unknown | unknown | BQ.1.1.11 | GRA |
| hCoV-19/Egypt/CPHL-EGY22708/2022 | EPI_ISL_16299817 | 23/11/2022 | Africa / Egypt | Human | unknown | unknown | unknown | non hospitalized | unknown | unknown | XBB.1 | GRA |
| hCoV-19/Egypt/CPHL-EGY22707/2022 | EPI_ISL_16299816 | 23/11/2022 | Africa / Egypt | Human | unknown | unknown | unknown | non hospitalized | unknown | unknown | XBB.1 | GRA |
| hCoV-19/Egypt/CPHL-EGY22706/2022 | EPI_ISL_16299815 | 13/11/2022 | Africa / Egypt | Human | unknown | unknown | unknown | non hospitalized | unknown | unknown | BQ.1.2 | GRA |
| hCoV-19/Egypt/CPHL-EGY22705/2022 | EPI_ISL_16299814 | 17/11/2022 | Africa / Egypt | Human | unknown | unknown | unknown | non hospitalized | unknown | unknown | BA.5.2.6 | GRA |
| hCoV-19/Egypt/CPHL-EGY22704/2022 | EPI_ISL_16299813 | 29/11/2022 | Africa / Egypt | Human | unknown | unknown | unknown | non hospitalized | unknown | unknown | XBB.1.9 | GRA |
| hCoV-19/Egypt/CPHL-EGY22703/2022 | EPI_ISL_16299812 | 19/11/2022 | Africa / Egypt | Human | unknown | unknown | unknown | non hospitalized | unknown | unknown | BQ.1.1 | GRA |
| hCoV-19/Egypt/CPHL-EGY22702/2022 | EPI_ISL_16299811 | 19/11/2022 | Africa / Egypt | Human | unknown | unknown | unknown | non hospitalized | unknown | unknown | BA.4.6.3 | GRA |
| hCoV-19/Egypt/CPHL-EGY22701/2022 | EPI_ISL_16299810 | 23/11/2022 | Africa / Egypt | Human | unknown | unknown | unknown | non hospitalized | unknown | unknown | BQ.1.2 | GRA |
| hCoV-19/Egypt/CPHL-EGY22700/2022 | EPI_ISL_16299809 | 29/11/2022 | Africa / Egypt | Human | unknown | unknown | unknown | non hospitalized | unknown | unknown | BE.1.1 | GRA |
| hCoV-19/Egypt/CPHL-EGY22699/2022 | EPI_ISL_16299808 | 23/11/2022 | Africa / Egypt | Human | unknown | unknown | unknown | non hospitalized | unknown | unknown | BQ.1.5 | GRA |
| hCoV-19/Egypt/CPHL-EGY22698/2022 | EPI_ISL_16299807 | 25/11/2022 | Africa / Egypt | Human | unknown | unknown | unknown | non hospitalized | unknown | unknown | BE.1.1.1 | GRA |
| hCoV-19/Egypt/CPHL-EGY22697/2022 | EPI_ISL_16299806 | 25/11/2022 | Africa / Egypt | Human | unknown | unknown | unknown | non hospitalized | unknown | unknown | BQ.1.1 | GRA |
| hCoV-19/Egypt/CPHL-EGY22696/2022 | EPI_ISL_16299805 | 26/10/2022 | Africa / Egypt | Human | unknown | unknown | unknown | non hospitalized | unknown | unknown | BA.2 | GRA |
| hCoV-19/Egypt/CPHL-EGY22695/2022 | EPI_ISL_16299804 | 27/11/2022 | Africa / Egypt | Human | unknown | unknown | unknown | non hospitalized | unknown | unknown | BQ.1.1 | GRA |
| hCoV-19/Egypt/CPHL-EGY22694/2022 | EPI_ISL_16299803 | 25/11/2022 | Africa / Egypt | Human | unknown | unknown | unknown | non hospitalized | unknown | unknown | XBB.1 | GRA |
| hCoV-19/Egypt/CPHL-EGY22693/2022 | EPI_ISL_16299802 | 28/11/2022 | Africa / Egypt | Human | unknown | unknown | unknown | non hospitalized | unknown | unknown | XBB.2 | GRA |
| hCoV-19/Egypt/CPHL-EGY22691/2022 | EPI_ISL_16299801 | 26/11/2022 | Africa / Egypt | Human | unknown | unknown | unknown | non hospitalized | unknown | unknown | XBB.1.4 | GRA |
| hCoV-19/Egypt/CPHL-EGY22690/2022 | EPI_ISL_16299800 | 15/11/2022 | Africa / Egypt | Human | unknown | unknown | unknown | non hospitalized | unknown | unknown | BA.5.2 | GRA |
| hCoV-19/Egypt/CPHL-EGY22689/2022 | EPI_ISL_16299799 | 15/11/2022 | Africa / Egypt | Human | unknown | unknown | unknown | non hospitalized | unknown | unknown | CK.1 | GRA |
| hCoV-19/Egypt/CPHL-EGY22687/2022 | EPI_ISL_16299798 | 01/12/2022 | Africa / Egypt | Human | unknown | unknown | unknown | non hospitalized | unknown | unknown | BQ.1.2 | GRA |
| hCoV-19/Egypt/CPHL-EGY22686/2022 | EPI_ISL_16299797 | 06/09/2022 | Africa / Egypt | Human | unknown | unknown | unknown | non hospitalized | unknown | unknown | BA.5.2.20 | GRA |
| hCoV-19/Egypt/CPHL-EGY22685/2022 | EPI_ISL_16299796 | 06/09/2022 | Africa / Egypt | Human | unknown | unknown | unknown | non hospitalized | unknown | unknown | BA.5.3.1 | GRA |
| hCoV-19/Egypt/CPHL-EGY22684/2022 | EPI_ISL_16299795 | 06/09/2022 | Africa / Egypt | Human | unknown | unknown | unknown | non hospitalized | unknown | unknown | BA.5.2 | GRA |
| hCoV-19/Egypt/CPHL-EGY22683/2022 | EPI_ISL_16299794 | 06/09/2022 | Africa / Egypt | Human | unknown | unknown | unknown | non hospitalized | unknown | unknown | BA.5.2.20 | GRA |
| hCoV-19/Egypt/CPHL-EGY22682/2022 | EPI_ISL_16299793 | 06/09/2022 | Africa / Egypt | Human | unknown | unknown | unknown | non hospitalized | unknown | unknown | BA.5.2 | GRA |
| hCoV-19/Egypt/CPHL-EGY22681/2022 | EPI_ISL_16299792 | 06/09/2022 | Africa / Egypt | Human | unknown | unknown | unknown | non hospitalized | unknown | unknown | BA.5.2 | GRA |
| hCoV-19/Egypt/CPHL-EGY22680/2022 | EPI_ISL_16299791 | 06/09/2022 | Africa / Egypt | Human | unknown | unknown | unknown | non hospitalized | unknown | unknown | BA.5.2 | GRA |
| hCoV-19/Egypt/CPHL-EGY22679/2022 | EPI_ISL_16299790 | 06/09/2022 | Africa / Egypt | Human | unknown | unknown | unknown | non hospitalized | unknown | unknown | BA.5.2 | GRA |
| hCoV-19/Egypt/CPHL-EGY22678/2022 | EPI_ISL_16299789 | 06/09/2022 | Africa / Egypt | Human | unknown | unknown | unknown | non hospitalized | unknown | unknown | BA.5.2.55 | GRA |
| hCoV-19/Egypt/CPHL-EGY22677/2022 | EPI_ISL_16299788 | 06/09/2022 | Africa / Egypt | Human | unknown | unknown | unknown | non hospitalized | unknown | unknown | BA.5.2.1 | GRA |
| hCoV-19/Egypt/CPHL-EGY22676/2022 | EPI_ISL_16299787 | 06/09/2022 | Africa / Egypt | Human | unknown | unknown | unknown | non hospitalized | unknown | unknown | BA.5.2 | GRA |
| hCoV-19/Egypt/CPHL-EGY22675/2022 | EPI_ISL_16299786 | 06/09/2022 | Africa / Egypt | Human | unknown | unknown | unknown | non hospitalized | unknown | unknown | BA.5.2.20 | GRA |
| hCoV-19/Egypt/CPHL-EGY22674/2022 | EPI_ISL_16299785 | 06/09/2022 | Africa / Egypt | Human | unknown | unknown | unknown | non hospitalized | unknown | unknown | BA.5.2 | GRA |
| hCoV-19/Egypt/CPHL-EGY22673/2022 | EPI_ISL_16299784 | 06/09/2022 | Africa / Egypt | Human | unknown | unknown | unknown | non hospitalized | unknown | unknown | BA.5.2 | GRA |
| hCoV-19/Egypt/CPHL-EGY22672/2022 | EPI_ISL_16299783 | 06/09/2022 | Africa / Egypt | Human | unknown | unknown | unknown | non hospitalized | unknown | unknown | BA.5.2 | GRA |
| hCoV-19/Egypt/CPHL-EGY22671/2022 | EPI_ISL_16299782 | 06/09/2022 | Africa / Egypt | Human | unknown | unknown | unknown | non hospitalized | unknown | unknown | BA.5.2 | GRA |
| hCoV-19/Egypt/CPHL-EGY22670/2022 | EPI_ISL_16299781 | 06/09/2022 | Africa / Egypt | Human | unknown | unknown | unknown | non hospitalized | unknown | unknown | BA.5.2 | GRA |
| hCoV-19/Egypt/CPHL-EGY22669/2022 | EPI_ISL_16299780 | 06/09/2022 | Africa / Egypt | Human | unknown | unknown | unknown | non hospitalized | unknown | unknown | BA.5.2 | GRA |
| hCoV-19/Egypt/CPHL-EGY22668/2022 | EPI_ISL_16299779 | 06/09/2022 | Africa / Egypt | Human | unknown | unknown | unknown | non hospitalized | unknown | unknown | BA.5.2 | GRA |
| hCoV-19/Egypt/CPHL-EGY22667/2022 | EPI_ISL_16299778 | 06/09/2022 | Africa / Egypt | Human | unknown | unknown | unknown | non hospitalized | unknown | unknown | BA.5.2 | GRA |
| hCoV-19/Egypt/CPHL-EGY22666/2022 | EPI_ISL_16299777 | 06/09/2022 | Africa / Egypt | Human | unknown | unknown | unknown | non hospitalized | unknown | unknown | BA.5.2 | GRA |
| hCoV-19/Egypt/CPHL-EGY22665/2022 | EPI_ISL_16299776 | 06/09/2022 | Africa / Egypt | Human | unknown | unknown | unknown | non hospitalized | unknown | unknown | BA.5.2 | GRA |
| hCoV-19/Egypt/CPHL-EGY22664/2022 | EPI_ISL_16299775 | 14/09/2022 | Africa / Egypt | Human | unknown | unknown | unknown | non hospitalized | unknown | unknown | BA.5.2 | GRA |
| hCoV-19/Egypt/CPHL-EGY22663/2022 | EPI_ISL_16299774 | 06/09/2022 | Africa / Egypt | Human | unknown | unknown | unknown | non hospitalized | unknown | unknown | BA.5.2 | GRA |
| hCoV-19/Egypt/CPHL-EGY22662/2022 | EPI_ISL_16299773 | 01/09/2022 | Africa / Egypt | Human | unknown | unknown | unknown | non hospitalized | unknown | unknown | BA.5.2 | GRA |
| hCoV-19/Egypt/CPHL-EGY22661/2022 | EPI_ISL_16299772 | 06/09/2022 | Africa / Egypt | Human | unknown | unknown | unknown | non hospitalized | unknown | unknown | BA.5.2 | GRA |
| hCoV-19/Egypt/CPHL-EGY22660/2022 | EPI_ISL_16299771 | 06/09/2022 | Africa / Egypt | Human | unknown | unknown | unknown | non hospitalized | unknown | unknown | BA.5.2 | GRA |
| hCoV-19/Egypt/CPHL-EGY22659/2022 | EPI_ISL_16299770 | 06/09/2022 | Africa / Egypt | Human | unknown | unknown | unknown | non hospitalized | unknown | unknown | BA.5.2 | GRA |
| hCoV-19/Egypt/CPHL-EGY22658/2022 | EPI_ISL_16299769 | 06/09/2022 | Africa / Egypt | Human | unknown | unknown | unknown | non hospitalized | unknown | unknown | BA.5.2 | GRA |
| hCoV-19/Egypt/CPHL-EGY22657/2022 | EPI_ISL_16299768 | 05/09/2022 | Africa / Egypt | Human | unknown | unknown | unknown | non hospitalized | unknown | unknown | BA.1.1 | GRA |
| hCoV-19/Egypt/CPHL-EGY22656/2022 | EPI_ISL_16299767 | 05/09/2022 | Africa / Egypt | Human | unknown | unknown | unknown | non hospitalized | unknown | unknown | BA.1.1 | GRA |
| hCoV-19/Egypt/CPHL-EGY22655/2022 | EPI_ISL_16299766 | 05/09/2022 | Africa / Egypt | Human | unknown | unknown | unknown | non hospitalized | unknown | unknown | BA.5.2.1 | GR |
| hCoV-19/Egypt/CPHL-EGY22654/2022 | EPI_ISL_16299765 | 05/09/2022 | Africa / Egypt | Human | unknown | unknown | unknown | non hospitalized | unknown | unknown | BA.1.1 | GRA |
| hCoV-19/Egypt/CPHL-EGY22653/2022 | EPI_ISL_16299764 | 05/09/2022 | Africa / Egypt | Human | unknown | unknown | unknown | non hospitalized | unknown | unknown | BA.5.2.1 | GRA |
| hCoV-19/Egypt/CPHL-EGY22652/2022 | EPI_ISL_16299763 | 05/09/2022 | Africa / Egypt | Human | unknown | unknown | unknown | non hospitalized | unknown | unknown | BA.5.2.1 | GRA |
| hCoV-19/Egypt/CPHL-EGY22651/2022 | EPI_ISL_16299762 | 05/09/2022 | Africa / Egypt | Human | unknown | unknown | unknown | non hospitalized | unknown | unknown | BF.5 | GRA |
| hCoV-19/Egypt/CPHL-EGY22650/2022 | EPI_ISL_16299761 | 06/09/2022 | Africa / Egypt | Human | unknown | unknown | unknown | non hospitalized | unknown | unknown | BA.5.2.1 | GRA |
| hCoV-19/Egypt/CPHL-EGY22649/2022 | EPI_ISL_16299760 | 06/09/2022 | Africa / Egypt | Human | unknown | unknown | unknown | non hospitalized | unknown | unknown | BA.5.2 | GRA |
| hCoV-19/Egypt/CPHL-EGY22648/2022 | EPI_ISL_16299759 | 05/09/2022 | Africa / Egypt | Human | unknown | unknown | unknown | non hospitalized | unknown | unknown | BA.5.2.1 | GRA |
| hCoV-19/Egypt/CPHL-EGY22647/2022 | EPI_ISL_16299758 | 05/09/2022 | Africa / Egypt | Human | unknown | unknown | unknown | non hospitalized | unknown | unknown | BA.5.2.1 | GRA |
| hCoV-19/Egypt/CPHL-EGY22646/2022 | EPI_ISL_16299757 | 14/09/2022 | Africa / Egypt | Human | unknown | unknown | unknown | non hospitalized | unknown | unknown | BA.5.2.20 | GRA |
| hCoV-19/Egypt/CPHL-EGY22645/2022 | EPI_ISL_16299756 | 14/09/2022 | Africa / Egypt | Human | unknown | unknown | unknown | non hospitalized | unknown | unknown | BA.5.2 | GRA |
| hCoV-19/Egypt/CPHL-EGY22643/2022 | EPI_ISL_16299755 | 05/09/2022 | Africa / Egypt | Human | unknown | unknown | unknown | non hospitalized | unknown | unknown | BA.2 | GRA |
| hCoV-19/Egypt/CPHL-EGY22642/2022 | EPI_ISL_16299754 | 05/09/2022 | Africa / Egypt | Human | unknown | unknown | unknown | non hospitalized | unknown | unknown | BA.5.2.1 | GRA |
| hCoV-19/Egypt/CPHL-EGY22641/2022 | EPI_ISL_16299753 | 05/09/2022 | Africa / Egypt | Human | unknown | unknown | unknown | non hospitalized | unknown | unknown | BA.1.1 | GRA |
| hCoV-19/Egypt/CPHL-EGY22640/2022 | EPI_ISL_16299752 | 05/09/2022 | Africa / Egypt | Human | unknown | unknown | unknown | non hospitalized | unknown | unknown | BA.5.2 | GRA |
| hCoV-19/Egypt/CPHL-EGY22639/2022 | EPI_ISL_16299751 | 14/09/2022 | Africa / Egypt | Human | unknown | unknown | unknown | non hospitalized | unknown | unknown | BA.5.2.20 | GRA |
| hCoV-19/Egypt/CPHL-EGY22638/2022 | EPI_ISL_16299750 | 14/09/2022 | Africa / Egypt | Human | unknown | unknown | unknown | non hospitalized | unknown | unknown | BA.5.2 | GRA |
| hCoV-19/Egypt/CPHL-EGY22636/2022 | EPI_ISL_16299748 | 14/09/2022 | Africa / Egypt | Human | unknown | unknown | unknown | non hospitalized | unknown | unknown | BA.5.2.1 | GRA |
| hCoV-19/Egypt/CPHL-EGY22635/2022 | EPI_ISL_16299747 | 14/09/2022 | Africa / Egypt | Human | unknown | unknown | unknown | non hospitalized | unknown | unknown | BA.5.3.1 | GRA |
| hCoV-19/Egypt/CPHL-EGY22634/2022 | EPI_ISL_16299746 | 14/09/2022 | Africa / Egypt | Human | unknown | unknown | unknown | non hospitalized | unknown | unknown | BA.5.2 | GRA |
| hCoV-19/Egypt/CPHL-EGY22633/2022 | EPI_ISL_16299745 | 22/08/2022 | Africa / Egypt | Human | unknown | unknown | unknown | non hospitalized | unknown | unknown | BF.5 | GRA |
| hCoV-19/Egypt/CPHL-EGY22632/2022 | EPI_ISL_16299744 | 22/08/2022 | Africa / Egypt | Human | unknown | unknown | unknown | non hospitalized | unknown | unknown | BA.5.2 | GRA |
| hCoV-19/Egypt/CPHL-EGY22631/2022 | EPI_ISL_16299743 | 22/08/2022 | Africa / Egypt | Human | unknown | unknown | unknown | non hospitalized | unknown | unknown | BF.5 | GRA |
| hCoV-19/Egypt/CPHL-EGY22630/2022 | EPI_ISL_16299742 | 22/08/2022 | Africa / Egypt | Human | unknown | unknown | unknown | non hospitalized | unknown | unknown | BA.5.2.20 | GRA |
| hCoV-19/Egypt/CPHL-EGY22629/2022 | EPI_ISL_16299741 | 22/08/2022 | Africa / Egypt | Human | unknown | unknown | unknown | non hospitalized | unknown | unknown | BA.5.2 | GRA |
| hCoV-19/Egypt/CPHL-EGY22628/2022 | EPI_ISL_16299740 | 22/08/2022 | Africa / Egypt | Human | unknown | unknown | unknown | non hospitalized | unknown | unknown | BF.5 | GR |
| hCoV-19/Egypt/CPHL-EGY22627/2022 | EPI_ISL_16299739 | 22/08/2022 | Africa / Egypt | Human | unknown | unknown | unknown | non hospitalized | unknown | unknown | BA.2 | GRA |
| hCoV-19/Egypt/CPHL-EGY22626/2022 | EPI_ISL_16299738 | 22/08/2022 | Africa / Egypt | Human | unknown | unknown | unknown | non hospitalized | unknown | unknown | BA.5.2 | GRA |
| hCoV-19/Egypt/CPHL-EGY22625/2022 | EPI_ISL_16299737 | 14/09/2022 | Africa / Egypt | Human | unknown | unknown | unknown | non hospitalized | unknown | unknown | BA.5.2 | GRA |
| hCoV-19/Egypt/CPHL-EGY22624/2022 | EPI_ISL_16299736 | 22/08/2022 | Africa / Egypt | Human | unknown | unknown | unknown | non hospitalized | unknown | unknown | BF.5 | GRA |
| hCoV-19/Egypt/ARMY-BPD0087/2022 | EPI_ISL_15932130 | 11/05/2022 | Africa / Egypt / Alexandria | Human | unknown | Male | 36 | unknown | Sinopharm | unknown | BA.1 | GRA |
| hCoV-19/Egypt/ARMY-BPD0086/2022 | EPI_ISL_15932129 | 11/05/2022 | Africa / Egypt / Alexandria | Human | unknown | Female | 38 | unknown | Sinopharm | unknown | BA.1.1 (consensus call) | GRA |
| hCoV-19/Egypt/ARMY-BPD0085/2022 | EPI_ISL_15932128 | 11/05/2022 | Africa / Egypt / Alexandria | Human | unknown | Male | 42 | unknown | Sinopharm | unknown | BA.2 | GR |
| hCoV-19/Egypt/ARMY-BPD0084/2022 | EPI_ISL_15932127 | 11/05/2022 | Africa / Egypt / Alexandria | Human | unknown | Male | 55 | unknown | Sinopharm | unknown | BA.2 | GR |
| hCoV-19/Egypt/ARMY-BPD0083/2022 | EPI_ISL_15932126 | 11/05/2022 | Africa / Egypt / Alexandria | Human | unknown | Male | 54 | unknown | Sinopharm | unknown | BA.2 | GR |
| hCoV-19/Egypt/ARMY-BPD0082/2022 | EPI_ISL_15932125 | 11/05/2022 | Africa / Egypt / Alexandria | Human | unknown | Male | 54 | unknown | Sinopharm | unknown | BA.1 | GRA |
| hCoV-19/Egypt/ARMY-BPD0081/2022 | EPI_ISL_15932124 | 11/05/2022 | Africa / Egypt / Alexandria | Human | unknown | Male | 44 | unknown | Sinopharm | unknown | BA.1.1 (consensus call) | GRA |
| hCoV-19/Egypt/ARMY-BPD0103/2022 | EPI_ISL_15932123 | 24/06/2022 | Africa / Egypt / Cairo | Human | unknown | Male | 46 | unknown | Sinopharm | unknown | BA.1 | GRA |
| hCoV-19/Egypt/ARMY-BPD0102/2022 | EPI_ISL_15932122 | 24/06/2022 | Africa / Egypt / Cairo | Human | unknown | Female | 36 | unknown | Sinopharm | unknown | BA.1 | GRA |
| hCoV-19/Egypt/ARMY-BPD0101/2022 | EPI_ISL_15932121 | 24/06/2022 | Africa / Egypt / Cairo | Human | unknown | Male | 38 | unknown | Sinopharm | unknown | BA.1 | GRA |
| hCoV-19/Egypt/ARMY-BPD0100/2022 | EPI_ISL_15932120 | 24/06/2022 | Africa / Egypt / Cairo | Human | unknown | Male | 46 | unknown | Sinopharm | unknown | BA.1.1 (consensus call) | GRA |
| hCoV-19/Egypt/ARMY-BPD0099/2022 | EPI_ISL_15932119 | 24/06/2022 | Africa / Egypt / Cairo | Human | unknown | Male | 47 | unknown | Sinopharm | unknown | BA.1.1 (consensus call) | GRA |
| hCoV-19/Egypt/ARMY-BPD0098/2022 | EPI_ISL_15932118 | 24/06/2022 | Africa / Egypt / Cairo | Human | unknown | Female | 41 | unknown | Sinopharm | unknown | BA.1 | GRA |
| hCoV-19/Egypt/ARMY-BPD0097/2022 | EPI_ISL_15932117 | 24/06/2022 | Africa / Egypt / Cairo | Human | unknown | Male | 36 | unknown | Sinopharm | unknown | BA.1.1 | GRA |
| hCoV-19/Egypt/ARMY-BPD0096/2022 | EPI_ISL_15932116 | 24/06/2022 | Africa / Egypt / Cairo | Human | unknown | Male | 35 | unknown | Sinopharm | unknown | BA.1 | GRA |
| hCoV-19/Egypt/ARMY-BPD0095/2022 | EPI_ISL_15932115 | 24/06/2022 | Africa / Egypt / Cairo | Human | unknown | Male | 33 | unknown | Sinopharm | unknown | BA.1.1 (consensus call) | GRA |
| hCoV-19/Egypt/ARMY-BPD0094/2022 | EPI_ISL_15932114 | 11/05/2022 | Africa / Egypt / Cairo | Human | unknown | Male | 32 | unknown | Sinopharm | unknown | BA.1 | GRA |
| hCoV-19/Egypt/ARMY-BPD0093/2022 | EPI_ISL_15932113 | 11/05/2022 | Africa / Egypt / Cairo | Human | unknown | Female | 28 | unknown | Sinopharm | unknown | BA.2 | GR |
| hCoV-19/Egypt/ARMY-BPD0019/2022 | EPI_ISL_15932112 | 09/02/2022 | Africa / Egypt / Cairo | Human | unknown | Male | 43 | unknown | Sinopharm | unknown | BA.1.1 (consensus call) | GRA |
| hCoV-19/Egypt/ARMY-BPD0018/2022 | EPI_ISL_15932111 | 09/02/2022 | Africa / Egypt / Cairo | Human | unknown | Male | 54 | unknown | Sinopharm | unknown | BA.2 | GR |
| hCoV-19/Egypt/ARMY-BPD0017/2022 | EPI_ISL_15932110 | 09/02/2022 | Africa / Egypt / Cairo | Human | unknown | Male | 34 | unknown | Sinopharm | unknown | BA.1.1 (consensus call) | GRA |
| hCoV-19/Egypt/ARMY-BPD0016/2022 | EPI_ISL_15932109 | 09/02/2022 | Africa / Egypt / Cairo | Human | unknown | Male | 32 | unknown | Sinopharm | unknown | BA.2 | GR |
| hCoV-19/Egypt/ARMY-BPD0015/2022 | EPI_ISL_15932108 | 09/02/2022 | Africa / Egypt / Cairo | Human | unknown | Male | 71 | unknown | Sinopharm | unknown | BA.1 | GRA |
| hCoV-19/Egypt/ARMY-BPD0014/2022 | EPI_ISL_15932107 | 09/02/2022 | Africa / Egypt / Cairo | Human | unknown | Male | 27 | unknown | Sinopharm | unknown | BA.2 | GR |
| hCoV-19/Egypt/ARMY-BPD0011/2022 | EPI_ISL_15932105 | 09/02/2022 | Africa / Egypt / Cairo | Human | unknown | Male | 45 | unknown | Sinopharm | unknown | BA.1 | GRA |
| hCoV-19/Egypt/ARMY-BPD0010/2022 | EPI_ISL_15932104 | 09/02/2022 | Africa / Egypt / Cairo | Human | unknown | Female | 43 | unknown | Sinopharm | unknown | BA.1 | GRA |
| hCoV-19/Egypt/ARMY-BPD0009/2022 | EPI_ISL_15932103 | 09/02/2022 | Africa / Egypt / Cairo | Human | unknown | Male | 34 | unknown | Sinopharm | unknown | BA.1 | GRA |
| hCoV-19/Egypt/ARMY-BPD0046/2022 | EPI_ISL_15932102 | 15/03/2022 | Africa / Egypt / Fayoum | Human | unknown | Male | 28 | unknown | Sinopharm | unknown | BA.1 | GRA |
| hCoV-19/Egypt/ARMY-BPD0045/2022 | EPI_ISL_15932101 | 15/03/2022 | Africa / Egypt / Fayoum | Human | unknown | Male | 32 | unknown | Sinopharm | unknown | BA.2 | GR |
| hCoV-19/Egypt/ARMY-BPD0043/2022 | EPI_ISL_15932100 | 15/03/2022 | Africa / Egypt / Fayoum | Human | unknown | Male | 45 | unknown | Sinopharm | unknown | BA.1 | GRA |
| hCoV-19/Egypt/ARMY-BPD0042/2022 | EPI_ISL_15932099 | 15/03/2022 | Africa / Egypt / Fayoum | Human | unknown | Female | 51 | unknown | Sinopharm | unknown | BA.1 | GRA |
| hCoV-19/Egypt/ARMY-BPD0031/2022 | EPI_ISL_15932098 | 15/03/2022 | Africa / Egypt / Fayoum | Human | unknown | Female | 53 | unknown | Sinopharm | unknown | BA.2 | GR |
| hCoV-19/Egypt/ARMY-BPD0030/2022 | EPI_ISL_15932097 | 15/03/2022 | Africa / Egypt / Fayoum | Human | unknown | Female | 32 | unknown | Sinopharm | unknown | BA.2 | GR |
| hCoV-19/Egypt/ARMY-BPD0029/2022 | EPI_ISL_15932096 | 15/03/2022 | Africa / Egypt / Fayoum | Human | unknown | Female | 41 | unknown | Sinopharm | unknown | BA.1.1 | GRA |
| hCoV-19/Egypt/ARMY-BPD0028/2022 | EPI_ISL_15932095 | 15/03/2022 | Africa / Egypt / Fayoum | Human | unknown | Male | 34 | unknown | Sinopharm | unknown | BA.2 | GR |
| hCoV-19/Egypt/ARMY-BPD0027/2022 | EPI_ISL_15932094 | 15/03/2022 | Africa / Egypt / Fayoum | Human | unknown | Male | 37 | unknown | Sinopharm | unknown | BA.1 | GRA |
| hCoV-19/Egypt/ARMY-BPD0026/2022 | EPI_ISL_15932093 | 15/03/2022 | Africa / Egypt / Fayoum | Human | unknown | Male | 47 | unknown | Sinopharm | unknown | BA.2 | GR |
| hCoV-19/Egypt/ARMY-BPD0025/2022 | EPI_ISL_15932092 | 15/03/2022 | Africa / Egypt / Fayoum | Human | unknown | Male | 43 | unknown | Sinopharm | unknown | BA.1 | GRA |
| hCoV-19/Egypt/ARMY-BPD0022/2022 | EPI_ISL_15932091 | 15/03/2022 | Africa / Egypt / Fayoum | Human | unknown | Male | 44 | unknown | Sinopharm | unknown | BA.2 | GR |
| hCoV-19/Egypt/ARMY-BPD0021/2022 | EPI_ISL_15932090 | 15/03/2022 | Africa / Egypt / Fayoum | Human | unknown | Male | 34 | unknown | Sinopharm | unknown | BA.2 | GR |
| hCoV-19/Egypt/ARMY-BPD0020/2022 | EPI_ISL_15932089 | 15/03/2022 | Africa / Egypt / Fayoum | Human | unknown | Male | 24 | unknown | Sinopharm | unknown | BA.1 | GRA |
| hCoV-19/Egypt/ARMY-BPD0092/2022 | EPI_ISL_15932088 | 11/05/2022 | Africa / Egypt / Giza | Human | unknown | Male | 25 | unknown | Sinopharm | unknown | BA.2 | GR |
| hCoV-19/Egypt/ARMY-BPD0091/2022 | EPI_ISL_15932087 | 11/05/2022 | Africa / Egypt / Giza | Human | unknown | Male | 22 | unknown | Sinopharm | unknown | BA.1.1 | GRA |
| hCoV-19/Egypt/ARMY-BPD0090/2022 | EPI_ISL_15932086 | 11/05/2022 | Africa / Egypt / Giza | Human | unknown | Male | 19 | unknown | Sinopharm | unknown | BA.2 | GR |
| hCoV-19/Egypt/ARMY-BPD0089/2022 | EPI_ISL_15932085 | 11/05/2022 | Africa / Egypt / Giza | Human | unknown | Male | 24 | unknown | Sinopharm | unknown | BA.1 | GRA |
| hCoV-19/Egypt/ARMY-BPD0088/2022 | EPI_ISL_15932084 | 11/05/2022 | Africa / Egypt / Giza | Human | unknown | Female | 25 | unknown | Sinopharm | unknown | BA.2 | GR |
| hCoV-19/Egypt/ARMY-BPD0007/2022 | EPI_ISL_15932083 | 22/01/2022 | Africa / Egypt / Giza | Human | unknown | Female | 43 | unknown | Sinopharm | unknown | BA.1.1 (consensus call) | GRA |
| hCoV-19/Egypt/ARMY-BPD0006/2022 | EPI_ISL_15932082 | 22/01/2022 | Africa / Egypt / Giza | Human | unknown | Female | 55 | unknown | Sinopharm | unknown | BA.1.1 (consensus call) | GRA |
| hCoV-19/Egypt/ARMY-BPD0005/2022 | EPI_ISL_15932081 | 22/01/2022 | Africa / Egypt / Giza | Human | unknown | Female | 65 | unknown | Sinopharm | unknown | BA.1 | GRA |
| hCoV-19/Egypt/ARMY-BPD0004/2022 | EPI_ISL_15932080 | 22/01/2022 | Africa / Egypt / Giza | Human | unknown | Female | 56 | unknown | Sinopharm | unknown | BA.1.1 | GRA |
| hCoV-19/Egypt/ARMY-BPD0002/2022 | EPI_ISL_15932079 | 22/01/2022 | Africa / Egypt / Giza | Human | unknown | Male | 61 | unknown | Sinopharm | unknown | BA.1.1 (consensus call) | GRA |
| hCoV-19/Egypt/ARMY-BPD0054/2022 | EPI_ISL_15932078 | 14/04/2022 | Africa / Egypt / Menofiya | Human | unknown | Male | 45 | unknown | Sinopharm | unknown | BA.2 | GR |
| hCoV-19/Egypt/ARMY-BPD0053/2022 | EPI_ISL_15932077 | 14/04/2022 | Africa / Egypt / Menofiya | Human | unknown | Male | 43 | unknown | Sinopharm | unknown | BA.2 | GR |
| hCoV-19/Egypt/ARMY-BPD0052/2022 | EPI_ISL_15932076 | 14/04/2022 | Africa / Egypt / Menofiya | Human | unknown | Male | 42 | unknown | Sinopharm | unknown | BA.2 | GR |
| hCoV-19/Egypt/ARMY-BPD0049/2022 | EPI_ISL_15932075 | 14/04/2022 | Africa / Egypt / Menofiya | Human | unknown | Male | 54 | unknown | Sinopharm | unknown | BA.2 | GR |
| hCoV-19/Egypt/ARMY-BPD0048/2022 | EPI_ISL_15932074 | 14/04/2022 | Africa / Egypt / Menofiya | Human | unknown | Male | 63 | unknown | Sinopharm | unknown | BA.1.1 (consensus call) | GRA |
| hCoV-19/Egypt/ARMY-BPD0047/2022 | EPI_ISL_15932073 | 14/04/2022 | Africa / Egypt / Menofiya | Human | unknown | Female | 61 | unknown | Sinopharm | unknown | BA.2 | GR |
| hCoV-19/Egypt/ARMY-BPD0079/2022 | EPI_ISL_15932072 | 11/05/2022 | Africa / Egypt / Sharqia | Human | unknown | Female | 42 | unknown | Sinopharm | unknown | BA.1.1 (consensus call) | GRA |
| hCoV-19/Egypt/ARMY-BPD0078/2022 | EPI_ISL_15932071 | 11/05/2022 | Africa / Egypt / Sharqia | Human | unknown | Female | 43 | unknown | Sinopharm | unknown | BA.2 | GR |
| hCoV-19/Egypt/ARMY-BPD0076/2022 | EPI_ISL_15932070 | 11/05/2022 | Africa / Egypt / Sharqia | Human | unknown | Female | 45 | unknown | Sinopharm | unknown | BA.2 | GR |
| hCoV-19/Egypt/ARMY-BPD0074/2022 | EPI_ISL_15932069 | 11/05/2022 | Africa / Egypt / Sharqia | Human | unknown | Female | 33 | unknown | Sinopharm | unknown | BA.1 | GRA |
| hCoV-19/Egypt/ARMY-BPD0073/2022 | EPI_ISL_15932068 | 11/05/2022 | Africa / Egypt / Sharqia | Human | unknown | Female | 32 | unknown | Sinopharm | unknown | BA.1 | GRA |
| hCoV-19/Egypt/ARMY-BPD0072/2022 | EPI_ISL_15932067 | 11/05/2022 | Africa / Egypt / Sharqia | Human | unknown | Female | 31 | unknown | Sinopharm | unknown | BA.1 | GRA |
| hCoV-19/Egypt/ARMY-BPD0070/2022 | EPI_ISL_15932066 | 14/04/2022 | Africa / Egypt / Sharqia | Human | unknown | Male | 34 | unknown | Sinopharm | unknown | BA.1 | GRA |
| hCoV-19/Egypt/ARMY-BPD0069/2022 | EPI_ISL_15932065 | 14/04/2022 | Africa / Egypt / Sharqia | Human | unknown | Female | 43 | unknown | Sinopharm | unknown | BA.1.1 (consensus call) | GRA |
| hCoV-19/Egypt/ARMY-BPD0068/2022 | EPI_ISL_15932064 | 14/04/2022 | Africa / Egypt / Suez | Human | unknown | Male | 47 | unknown | Sinopharm | unknown | BA.1.1 (consensus call) | GRA |
| hCoV-19/Egypt/ARMY-BPD0067/2022 | EPI_ISL_15932063 | 14/04/2022 | Africa / Egypt / Suez | Human | unknown | Female | 47 | unknown | Sinopharm | unknown | BA.1 | GRA |
| hCoV-19/Egypt/ARMY-BPD0066/2022 | EPI_ISL_15932062 | 14/04/2022 | Africa / Egypt / Suez | Human | unknown | Male | 46 | unknown | Sinopharm | unknown | BA.1.1 | GRA |
| hCoV-19/Egypt/ARMY-BPD0065/2022 | EPI_ISL_15932061 | 14/04/2022 | Africa / Egypt / Suez | Human | unknown | Female | 45 | unknown | Sinopharm | unknown | BA.1 | GRA |
| hCoV-19/Egypt/ARMY-BPD0064/2022 | EPI_ISL_15932060 | 14/04/2022 | Africa / Egypt / Suez | Human | unknown | Male | 55 | unknown | Sinopharm | unknown | BA.1.1 (consensus call) | GRA |
| hCoV-19/Egypt/ARMY-BPD0080/2022 | EPI_ISL_15931225 | 11/05/2022 | Africa / Egypt / Alexandria | Human | unknown | Female | 55 | unknown | Sinopharm | unknown | BA.2 | GR |
| hCoV-19/Egypt/ARMY-BPD0012/2022 | EPI_ISL_15931224 | 09/02/2022 | Africa / Egypt / Cairo | Human | unknown | Male | 67 | unknown | Sinopharm | unknown | BA.1 | GRA |
| hCoV-19/Egypt/ARMY-BPD0008/2022 | EPI_ISL_15931223 | 09/03/2022 | Africa / Egypt / Cairo | Human | unknown | Male | 23 | unknown | Sinopharm | unknown | BA.1 | GRA |
| hCoV-19/Egypt/ARMY-BPD0044/2022 | EPI_ISL_15931222 | 15/03/2022 | Africa / Egypt / Fayoum | Human | unknown | Female | 66 | unknown | Sinopharm | unknown | BA.1.1 (consensus call) | GRA |
| hCoV-19/Egypt/ARMY-BPD0041/2022 | EPI_ISL_15931221 | 15/03/2022 | Africa / Egypt / Fayoum | Human | unknown | Male | 21 | unknown | Sinopharm | unknown | BA.1 | GRA |
| hCoV-19/Egypt/ARMY-BPD0040/2022 | EPI_ISL_15931220 | 15/03/2022 | Africa / Egypt / Fayoum | Human | unknown | Male | 22 | unknown | Sinopharm | unknown | BA.1 | GRA |
| hCoV-19/Egypt/ARMY-BPD0039/2022 | EPI_ISL_15931219 | 15/03/2022 | Africa / Egypt / Fayoum | Human | unknown | Male | 18 | unknown | Sinopharm | unknown | BA.1 | GRA |
| hCoV-19/Egypt/ARMY-BPD0038/2022 | EPI_ISL_15931218 | 15/03/2022 | Africa / Egypt / Fayoum | Human | unknown | Male | 17 | unknown | Sinopharm | unknown | BA.1.1 (consensus call) | GRA |
| hCoV-19/Egypt/ARMY-BPD0037/2022 | EPI_ISL_15931217 | 15/03/2022 | Africa / Egypt / Fayoum | Human | unknown | Male | 19 | unknown | Sinopharm | unknown | BA.1.1 (consensus call) | GRA |
| hCoV-19/Egypt/ARMY-BPD0036/2022 | EPI_ISL_15931216 | 15/03/2022 | Africa / Egypt / Fayoum | Human | unknown | Male | 44 | unknown | Sinopharm | unknown | BA.1 | GRA |
| hCoV-19/Egypt/ARMY-BPD0035/2022 | EPI_ISL_15931215 | 15/03/2022 | Africa / Egypt / Fayoum | Human | unknown | Male | 54 | unknown | Sinopharm | unknown | BA.1.1 | GRA |
| hCoV-19/Egypt/ARMY-BPD0034/2022 | EPI_ISL_15931214 | 15/03/2022 | Africa / Egypt / Fayoum | Human | unknown | Male | 33 | unknown | Sinopharm | unknown | BA.1 | GRA |
| hCoV-19/Egypt/ARMY-BPD0033/2022 | EPI_ISL_15931213 | 15/03/2022 | Africa / Egypt / Fayoum | Human | unknown | Female | 43 | unknown | Sinopharm | unknown | BA.1.1 (consensus call) | GRA |
| hCoV-19/Egypt/ARMY-BPD0032/2022 | EPI_ISL_15931212 | 15/03/2022 | Africa / Egypt / Fayoum | Human | unknown | Female | 54 | unknown | Sinopharm | unknown | BA.1 | GRA |
| hCoV-19/Egypt/ARMY-BPD0024/2022 | EPI_ISL_15931211 | 15/03/2022 | Africa / Egypt / Fayoum | Human | unknown | Male | 41 | unknown | Sinopharm | unknown | BA.1.1 (consensus call) | GRA |
| hCoV-19/Egypt/ARMY-BPD0023/2022 | EPI_ISL_15931210 | 15/03/2022 | Africa / Egypt / Fayoum | Human | unknown | Male | 45 | unknown | Sinopharm | unknown | BA.2 | GR |
| hCoV-19/Egypt/ARMY-BPD0003/2022 | EPI_ISL_15931209 | 22/01/2022 | Africa / Egypt / Giza | Human | unknown | Female | 45 | unknown | Sinopharm | unknown | BA.1 | GRA |
| hCoV-19/Egypt/ARMY-BPD0001/2022 | EPI_ISL_15931208 | 22/01/2022 | Africa / Egypt / Giza | Human | unknown | Male | 55 | unknown | Sinopharm | unknown | BA.1 | GRA |
| hCoV-19/Egypt/ARMY-BPD0060/2022 | EPI_ISL_15931207 | 14/04/2022 | Africa / Egypt / Menofiya | Human | unknown | Male | 55 | unknown | Sinopharm | unknown | BA.1.1 | GRA |
| hCoV-19/Egypt/ARMY-BPD0059/2022 | EPI_ISL_15931206 | 14/04/2022 | Africa / Egypt / Menofiya | Human | unknown | Male | 43 | unknown | Sinopharm | unknown | BA.2 | GR |
| hCoV-19/Egypt/ARMY-BPD0058/2022 | EPI_ISL_15931205 | 14/04/2022 | Africa / Egypt / Menofiya | Human | unknown | Male | 42 | unknown | Sinopharm | unknown | BA.1 | GRA |
| hCoV-19/Egypt/ARMY-BPD0057/2022 | EPI_ISL_15931204 | 14/04/2022 | Africa / Egypt / Menofiya | Human | unknown | Male | 44 | unknown | Sinopharm | unknown | BA.2 | GR |
| hCoV-19/Egypt/ARMY-BPD0056/2022 | EPI_ISL_15931203 | 14/04/2022 | Africa / Egypt / Menofiya | Human | unknown | Male | 47 | unknown | Sinopharm | unknown | BA.1 | GRA |
| hCoV-19/Egypt/ARMY-BPD0055/2022 | EPI_ISL_15931202 | 14/04/2022 | Africa / Egypt / Menofiya | Human | unknown | Male | 45 | unknown | Sinopharm | unknown | BA.1.1 (consensus call) | GRA |
| hCoV-19/Egypt/ARMY-BPD0051/2022 | EPI_ISL_15931201 | 14/04/2022 | Africa / Egypt / Menofiya | Human | unknown | Male | 56 | unknown | Sinopharm | unknown | BA.1 | GRA |
| hCoV-19/Egypt/ARMY-BPD0050/2022 | EPI_ISL_15931200 | 14/04/2022 | Africa / Egypt / Menofiya | Human | unknown | Male | 55 | unknown | Sinopharm | unknown | BA.1.1 (consensus call) | GRA |
| hCoV-19/Egypt/ARMY-BPD0077/2022 | EPI_ISL_15931199 | 11/05/2022 | Africa / Egypt / Sharqia | Human | unknown | Male | 46 | unknown | Sinopharm | unknown | BA.1 | GRA |
| hCoV-19/Egypt/ARMY-BPD0075/2022 | EPI_ISL_15931198 | 11/05/2022 | Africa / Egypt / Sharqia | Human | unknown | Male | 44 | unknown | Sinopharm | unknown | BA.1.1 (consensus call) | GRA |
| hCoV-19/Egypt/ARMY-BPD0071/2022 | EPI_ISL_15931197 | 14/04/2022 | Africa / Egypt / Sharqia | Human | unknown | Female | 33 | unknown | Sinopharm | unknown | BA.1 | GRA |
| hCoV-19/Egypt/ARMY-BPD0063/2022 | EPI_ISL_15931196 | 14/04/2022 | Africa / Egypt / Suez | Human | unknown | Male | 54 | unknown | Sinopharm | unknown | BA.1 | GRA |
| hCoV-19/Egypt/ARMY-BPD0062/2022 | EPI_ISL_15931195 | 14/04/2022 | Africa / Egypt / Suez | Human | unknown | Male | 45 | unknown | Sinopharm | unknown | BA.2 | GR |
| hCoV-19/Egypt/ARMY-BPD0061/2022 | EPI_ISL_15931194 | 14/04/2022 | Africa / Egypt / Suez | Human | unknown | Male | 45 | unknown | Sinopharm | unknown | BA.2 | GR |
| hCoV-19/Egypt/CPHL-EGY21012/2021 | EPI_ISL_9047364 | 01/12/2021 | Africa / Egypt / Menofia governorate / Shebin el kom | Human | unknown | Male | 45 | Hospitalized | unknown | unknown | AY.122 | GK |
| hCoV-19/Egypt/CPHL-EGY21230/2021 | EPI_ISL_9047577 | 01/12/2021 | Africa / Egypt / Red sea governorate / Safaga | Human | unknown | Female | 14 | Hotel isolation | unknown | unknown | B.1.617.2 | GK |
| hCoV-19/Egypt/CPHL-EGY21233/2021 | EPI_ISL_9047580 | 02/12/2021 | Africa / Egypt / Red sea governorate / Safaga | Human | unknown | Male | 29 | Hotel isolation | unknown | unknown | B.1.617.2 | GK |
| hCoV-19/Egypt/CPHL-EGY21178/2021 | EPI_ISL_9047528 | 10/12/2021 | Africa / Egypt / Red sea governorate / Safaga | Human | unknown | Female | 59 | Hotel isolation | unknown | unknown | B.1.617.2 | GK |
| hCoV-19/Egypt/CPHL-EGY21182/2021 | EPI_ISL_9047531 | 10/12/2021 | Africa / Egypt / Red sea governorate / Safaga | Human | unknown | Male | 56 | Hotel isolation | unknown | unknown | B.1.617.2 | GK |
| hCoV-19/Egypt/CPHL-EGY21189/2021 | EPI_ISL_9047538 | 12/11/2021 | Africa / Egypt / Red sea governorate / Safaga | Human | unknown | Male | 29 | Hospitalized | unknown | unknown | AY.122 | GK |
| hCoV-19/Egypt/CPHL-EGY21197/2021 | EPI_ISL_9047546 | 27/08/2021 | Africa / Egypt / Alexandria governorate | Human | unknown | Male | 50 | Hospitalized | unknown | unknown | AY.122 | GK |
| hCoV-19/Egypt/CPHL-EGY21160/2021 | EPI_ISL_9047510 | 04/12/2021 | Africa / Egypt / Red sea governorate / Safaga | Human | unknown | Female | 67 | Hotel isolation | unknown | unknown | B.1.617.2 | GK |
| hCoV-19/Egypt/CPHL-EGY21364/2021 | EPI_ISL_9047699 | 31/12/2021 | Africa / Egypt / Red sea governorate / Hurgada | Human | unknown | Male | 56 | Hotel isolation | unknown | unknown | AY.122 (consensus call) | GK |
| hCoV-19/Egypt/CPHL-EGY21339/2021 | EPI_ISL_9047676 | 01/12/2021 | Africa / Egypt / Red sea governorate / Hurgada | Human | unknown | Male | 25 | Hotel isolation | unknown | unknown | B.1.617.2 | GK |
| hCoV-19/Egypt/CPHL-EGY21317/2021 | EPI_ISL_9047656 | 28/12/2021 | Africa / Egypt / Red sea governorate / Hurgada | Human | unknown | Male | 26 | Hotel isolation | unknown | unknown | AY.43 | GK |
| hCoV-19/Egypt/CPHL-EGY21303/2021 | EPI_ISL_9047643 | 15/12/2021 | Africa / Egypt / Red sea governorate / Hurgada | Human | unknown | Female | 55 | Hotel isolation | unknown | unknown | B.1.617.2 | GK |
| hCoV-19/Egypt/CPHL-EGY21262/2021 | EPI_ISL_9047609 | 08/12/2021 | Africa / Egypt / Red sea governorate / Hurgada | Human | unknown | Female | 24 | Hotel isolation | unknown | unknown | B.1.617.2 | GK |
| hCoV-19/Egypt/CPHL-EGY21369/2021 | EPI_ISL_9047704 | 31/12/2021 | Africa / Egypt / Red sea governorate / Hurgada | Human | unknown | Female | 25 | Hotel isolation | unknown | unknown | AY.122 (consensus call) | GK |
| hCoV-19/Egypt/CPHL-EGY21383/2021 | EPI_ISL_9047717 | 31/12/2021 | Africa / Egypt / Red sea governorate / Hurgada | Human | unknown | Female | 21 | Hotel isolation | unknown | unknown | AY.122 | GK |
| hCoV-19/Egypt/CPHL-EGY21385/2021 | EPI_ISL_9047719 | 31/12/2021 | Africa / Egypt / Red sea governorate / Hurgada | Human | unknown | Male | 25 | Hotel isolation | unknown | unknown | B.1.617.2 | GK |
| hCoV-19/Egypt/CPHL-EGY21380/2021 | EPI_ISL_9047715 | 31/12/2021 | Africa / Egypt / Red sea governorate / Hurgada | Human | unknown | Male | 24 | Hotel isolation | unknown | unknown | AY.122 | GK |
| hCoV-19/Egypt/CPHL-EGY21387/2022 | EPI_ISL_9047720 | 01/01/2022 | Africa / Egypt / Red sea governorate / Hurgada | Human | unknown | Female | 23 | Hotel isolation | unknown | unknown | B.1.617.2 | GV |
| hCoV-19/Egypt/CPHL-EGY21456/2021 | EPI_ISL_9047785 | 15/11/2021 | Africa / Egypt / Aswan governorate | Human | unknown | Female | 48 | Hospitalized | unknown | unknown | AY.122 | GK |
| hCoV-19/Egypt/CPHL-EGY21421/2022 | EPI_ISL_9047752 | 02/01/2022 | Africa / Egypt / Red sea governorate / Hurgada | Human | unknown | Female | 23 | Hotel isolation | unknown | unknown | B.1.617.2 | GK |
| hCoV-19/Egypt/CPHL-EGY21430/2022 | EPI_ISL_9047759 | 02/01/2022 | Africa / Egypt / Red sea governorate / Hurgada | Human | unknown | Male | 14 | Hotel isolation | unknown | unknown | B.1.617.2 | GK |
| hCoV-19/Egypt/CPHL-EGY21431/2022 | EPI_ISL_9047760 | 02/01/2022 | Africa / Egypt / Red sea governorate / Hurgada | Human | unknown | Male | 23 | Hotel isolation | unknown | unknown | AY.4 | GK |
| hCoV-19/Egypt/CPHL-EGY21395/2021 | EPI_ISL_9047728 | 31/12/2021 | Africa / Egypt / Red sea governorate / Hurgada | Human | unknown | Female | 48 | Hotel isolation | unknown | unknown | B.1.617.2 | GK |
| hCoV-19/Egypt/CPHL-EGY21403/2021 | EPI_ISL_9047735 | 31/12/2021 | Africa / Egypt / Red sea governorate / Hurgada | Human | unknown | Female | 23 | Hotel isolation | unknown | unknown | B.1.617.2 | GK |
| hCoV-19/Egypt/CPHL-EGY21404/2021 | EPI_ISL_9047736 | 31/12/2021 | Africa / Egypt / Red sea governorate / Hurgada | Human | unknown | Female | 29 | Hotel isolation | unknown | unknown | B.1.617.2 | GK |
| hCoV-19/Egypt/CPHL-EGY21409/2022 | EPI_ISL_9047741 | 02/01/2022 | Africa / Egypt / Red sea governorate / Hurgada | Human | unknown | Female | 42 | Hotel isolation | unknown | unknown | AY.112 | GK |
| hCoV-19/Egypt/CPHL-EGY21413/2022 | EPI_ISL_9047744 | 02/01/2022 | Africa / Egypt / Red sea governorate / Hurgada | Human | unknown | Male | 25 | Hotel isolation | unknown | unknown | B.1.617.2 | GK |
| hCoV-19/Egypt/CPHL-EGY21408/2022 | EPI_ISL_9047740 | 02/01/2022 | Africa / Egypt / Red sea governorate / Hurgada | Human | unknown | Male | 44 | Hotel isolation | unknown | unknown | B.1.617.2 | GK |
| hCoV-19/Egypt/CPHL-EGY21474/2021 | EPI_ISL_9047803 | 28/09/2021 | Africa / Egypt / Aswan governorate | Human | unknown | Female | 36 | Hospitalized | unknown | unknown | B.1.617.2 | GK |
| hCoV-19/Egypt/CPHL-EGY21041/2021 | EPI_ISL_9047393 | 04/12/2021 | Africa / Egypt / Dammita governorate | Human | unknown | Male | 41 | Hospitalized | unknown | unknown | B.1.1.529 | GRA |
| hCoV-19/Egypt/CPHL-EGY21007/2021 | EPI_ISL_9047360 | 01/12/2021 | Africa / Egypt / Menofia governorate / Shebin el kom | Human | unknown | Male | 39 | Hospitalized | unknown | unknown | B.1.617.2 | GK |
| hCoV-19/Egypt/CPHL-EGY21127/2021 | EPI_ISL_9047479 | 15/12/2021 | Africa / Egypt / Red sea governorate / Safaga | Human | unknown | Male | 8 | Hotel isolation | unknown | unknown | B.1.1.529 | GRA |
| hCoV-19/Egypt/CPHL-EGY21097/2021 | EPI_ISL_9047449 | 16/12/2021 | Africa / Egypt / Red sea governorate / Safaga | Human | unknown | Male | 29 | Hotel isolation | unknown | unknown | B.1.617.2 | GK |
| hCoV-19/Egypt/CPHL-EGY21104/2021 | EPI_ISL_9047456 | 17/12/2021 | Africa / Egypt / Red sea governorate / Safaga | Human | unknown | Female | 58 | Hotel isolation | unknown | unknown | AY.122 | GK |
| hCoV-19/Egypt/CPHL-EGY21100/2021 | EPI_ISL_9047452 | 17/12/2021 | Africa / Egypt / Red sea governorate / Safaga | Human | unknown | Female | 48 | Hotel isolation | unknown | unknown | B.1.617.2 | G |
| hCoV-19/Egypt/CPHL-EGY21115/2021 | EPI_ISL_9047467 | 05/12/2021 | Africa / Egypt / Red sea governorate / Safaga | Human | unknown | Male | 48 | Hotel isolation | unknown | unknown | B.1.1.529 | G |
| hCoV-19/Egypt/CPHL-EGY21113/2021 | EPI_ISL_9047465 | 23/12/2021 | Africa / Egypt / Red sea governorate / Safaga | Human | unknown | Male | 32 | Hotel isolation | unknown | unknown | B.1.617.2 | GK |
| hCoV-19/Egypt/CPHL-EGY21084/2021 | EPI_ISL_9047436 | 01/12/2021 | Africa / Egypt / Cairo | Human | unknown | Male | 44 | Hospitalized | unknown | unknown | BA.1 | GRA |
| hCoV-19/Egypt/CPHL-EGY21083/2021 | EPI_ISL_9047435 | 20/12/2021 | Africa / Egypt / Red sea governorate / Safaga | Human | unknown | Female | 22 | Hotel isolation | unknown | unknown | B.1.617.2 | GK |
| hCoV-19/Egypt/CPHL-EGY21080/2021 | EPI_ISL_9047432 | 17/12/2021 | Africa / Egypt / Red sea governorate / Safaga | Human | unknown | Male | 22 | Hotel isolation | unknown | unknown | B.1.617.2 | GK |
| hCoV-19/Egypt/CPHL-EGY21087/2021 | EPI_ISL_9047439 | 04/12/2021 | Africa / Egypt / Cairo | Human | unknown | Male | 44 | Hospitalized | unknown | unknown | BA.1 | GRA |
| hCoV-19/Egypt/CPHL-EGY21048/2021 | EPI_ISL_9047400 | 04/12/2021 | Africa / Egypt / Cairo / Shoubrai | Human | unknown | Male | 50 | Hospitalized | unknown | unknown | BA.1.14 | GRA |
| hCoV-19/Egypt/CPHL-EGY21201/2021 | EPI_ISL_9047550 | 27/08/2021 | Africa / Egypt / Alexandria governorate | Human | unknown | Female | 45 | Hospitalized | unknown | unknown | Unassigned | GR |
| hCoV-19/Egypt/CPHL-EGY21195/2021 | EPI_ISL_9047544 | 12/11/2021 | Africa / Egypt / Red sea governorate / Safaga | Human | unknown | Male | 32 | Hospitalized | unknown | unknown | Unassigned | GK |
| hCoV-19/Egypt/CPHL-EGY21467/2021 | EPI_ISL_9047796 | 23/09/2021 | Africa / Egypt / Aswan governorate | Human | unknown | Female | 35 | Hospitalized | unknown | unknown | Unassigned | GK |
| hCoV-19/Egypt/CPHL-EGY21216/2021 | EPI_ISL_9047565 | 12/02/2021 | Africa / Egypt / Red sea governorate / Safaga | Human | unknown | Male | 26 | Hospitalized | unknown | unknown | Unassigned | GK |
| hCoV-19/Egypt/CPHL-EGY21179/2021 | EPI_ISL_9047529 | 10/12/2021 | Africa / Egypt / Red sea governorate / Safaga | Human | unknown | Female | 69 | Hotel isolation | unknown | unknown | Unassigned | GK |
| hCoV-19/Egypt/CPHL-EGY21447/2021 | EPI_ISL_9047776 | 18/11/2021 | Africa / Egypt / Aswan governorate | Human | unknown | Male | 55 | Hospitalized | unknown | unknown | Unassigned | GK |
| hCoV-19/Egypt/CPHL-EGY21453/2021 | EPI_ISL_9047782 | 13/11/2021 | Africa / Egypt / Aswan governorate | Human | unknown | Male | 39 | Hospitalized | unknown | unknown | Unassigned | GK |
| hCoV-19/Egypt/CPHL-EGY21207/2021 | EPI_ISL_9047556 | 10/08/2021 | Africa / Egypt / Alexandria governorate | Human | unknown | Female | 26 | Hospitalized | unknown | unknown | Unassigned | GK |
| hCoV-19/Egypt/CPHL-EGY21254/2021 | EPI_ISL_9047601 | 05/12/2021 | Africa / Egypt / Red sea governorate / Hurgada | Human | unknown | Female | 78 | Hotel isolation | unknown | unknown | Unassigned | GK |
| hCoV-19/Egypt/CPHL-EGY21246/2021 | EPI_ISL_9047593 | 19/12/2021 | Africa / Egypt / Red sea governorate / Hurgada | Human | unknown | Male | 17 | Hotel isolation | unknown | unknown | Unassigned | GK |
| hCoV-19/Egypt/CPHL-EGY21436/2022 | EPI_ISL_9047765 | 07/01/2022 | Africa / Egypt / Red sea governorate / Hurgada | Human | unknown | Female | 25 | Hotel isolation | unknown | unknown | Unassigned | GK |
| hCoV-19/Egypt/CPHL-EGY21425/2022 | EPI_ISL_9047755 | 02/01/2022 | Africa / Egypt / Red sea governorate / Hurgada | Human | unknown | Male | 36 | Hotel isolation | unknown | unknown | Unassigned | GK |
| hCoV-19/Egypt/CPHL-EGY21455/2021 | EPI_ISL_9047784 | 14/11/2021 | Africa / Egypt / Aswan governorate | Human | unknown | Female | 52 | Hospitalized | unknown | unknown | Unassigned | GK |
| hCoV-19/Egypt/CPHL-EGY21275/2021 | EPI_ISL_9047621 | 11/12/2021 | Africa / Egypt / Red sea governorate / Hurgada | Human | unknown | Female | 57 | Hotel isolation | unknown | unknown | Unassigned | GK |
| hCoV-19/Egypt/CPHL-EGY21173/2021 | EPI_ISL_9047523 | 08/12/2021 | Africa / Egypt / Red sea governorate / Safaga | Human | unknown | Male | 33 | Hotel isolation | unknown | unknown | Unassigned | G |
| hCoV-19/Egypt/CPHL-EGY21221/2021 | EPI_ISL_9047570 | 13/02/2021 | Africa / Egypt / Red sea governorate / Safaga | Human | unknown | Female | 52 | Hospitalized | unknown | unknown | Unassigned | GK |
| hCoV-19/Egypt/CPHL-EGY21434/2022 | EPI_ISL_9047763 | 02/01/2022 | Africa / Egypt / Red sea governorate / Hurgada | Human | unknown | Female | 26 | Hotel isolation | unknown | unknown | Unassigned | GK |
| hCoV-19/Egypt/CPHL-EGY21433/2022 | EPI_ISL_9047762 | 02/01/2022 | Africa / Egypt / Red sea governorate / Hurgada | Human | unknown | Male | 25 | Hotel isolation | unknown | unknown | Unassigned | GK |
| hCoV-19/Egypt/CPHL-EGY21238/2021 | EPI_ISL_9047585 | 18/12/2021 | Africa / Egypt / Red sea governorate / Hurgada | Human | unknown | Female | 12 | Hotel isolation | unknown | unknown | Unassigned | GK |
| hCoV-19/Egypt/CPHL-EGY21402/2021 | EPI_ISL_9047734 | 31/12/2021 | Africa / Egypt / Red sea governorate / Hurgada | Human | unknown | Female | 23 | Hotel isolation | unknown | unknown | Unassigned | GK |
| hCoV-19/Egypt/CPHL-EGY21405/2021 | EPI_ISL_9047737 | 31/12/2021 | Africa / Egypt / Red sea governorate / Hurgada | Human | unknown | Male | 45 | Hotel isolation | unknown | unknown | Unassigned | GK |
| hCoV-19/Egypt/CPHL-EGY21152/2021 | EPI_ISL_9047502 | 07/11/2021 | Africa / Egypt / Red sea governorate / Safaga | Human | unknown | Female | 46 | Hotel isolation | unknown | unknown | Unassigned | GK |
| hCoV-19/Egypt/CPHL-EGY21142/2021 | EPI_ISL_9047492 | 05/10/2021 | Africa / Egypt / Red sea governorate / Safaga | Human | unknown | Male | 78 | Hotel isolation | unknown | unknown | Unassigned | GK |
| hCoV-19/Egypt/CPHL-EGY21140/2021 | EPI_ISL_9047490 | 01/12/2021 | Africa / Egypt / Red sea governorate / Safaga | Human | unknown | Male | 72 | Hotel isolation | unknown | unknown | Unassigned | G |
| hCoV-19/Egypt/CPHL-EGY21027/2021 | EPI_ISL_9047379 | 03/12/2021 | Africa / Egypt / Dammita governorate | Human | unknown | Female | 42 | Hospitalized | unknown | unknown | Unassigned | G |
| hCoV-19/Egypt/CPHL-EGY21148/2021 | EPI_ISL_9047498 | 25/10/2021 | Africa / Egypt / Red sea governorate / Safaga | Human | unknown | Female | 19 | Hotel isolation | unknown | unknown | Unassigned | GK |
| hCoV-19/Egypt/CPHL-EGY21131/2021 | EPI_ISL_9047482 | 25/11/2021 | Africa / Egypt / Red sea governorate / Safaga | Human | unknown | Female | 56 | Hotel isolation | unknown | unknown | Unassigned | G |
| hCoV-19/Egypt/CPHL-EGY21125/2021 | EPI_ISL_9047477 | 15/12/2021 | Africa / Egypt / Red sea governorate / Safaga | Human | unknown | Male | 39 | Hotel isolation | unknown | unknown | Unassigned | GK |
| hCoV-19/Egypt/CPHL-EGY21008/2021 | EPI_ISL_9047361 | 01/12/2021 | Africa / Egypt / Menofia governorate / Shebin el kom | Human | unknown | Male | 44 | Hospitalized | unknown | unknown | Unassigned | GK |
| hCoV-19/Egypt/CPHL-EGY21123/2021 | EPI_ISL_9047475 | 15/12/2021 | Africa / Egypt / Red sea governorate / Safaga | Human | unknown | Female | 42 | Hotel isolation | unknown | unknown | Unassigned | GK |
| hCoV-19/Egypt/CPHL-EGY21130/2021 | EPI_ISL_9047481 | 24/11/2021 | Africa / Egypt / Red sea governorate / Safaga | Human | unknown | Female | 45 | Hotel isolation | unknown | unknown | Unassigned | GK |
| hCoV-19/Egypt/CPHL-EGY21099/2021 | EPI_ISL_9047451 | 16/12/2021 | Africa / Egypt / Red sea governorate / Safaga | Human | unknown | Female | 49 | Hotel isolation | unknown | unknown | Unassigned | GK |
| hCoV-19/Egypt/CPHL-EGY21055/2021 | EPI_ISL_9047407 | 01/12/2021 | Africa / Egypt / Red sea governorate / Hurgada | Human | unknown | Male | 45 | Hotel isolation | unknown | unknown | Unassigned | G |
| hCoV-19/Egypt/CPHL-EGY21454/2021 | EPI_ISL_9047783 | 13/11/2021 | Africa / Egypt / Aswan governorate | Human | unknown | Male | 45 | Hospitalized | unknown | unknown | B.1.617.2 | GK |
| hCoV-19/Egypt/CPHL-EGY21133/2021 | EPI_ISL_9047483 | 28/11/2021 | Africa / Egypt / Red sea governorate / Safaga | Human | unknown | Female | 45 | Hotel isolation | unknown | unknown | B.1.617.2 | GK |
| hCoV-19/Egypt/CPHL-EGY21346/2021 | EPI_ISL_9047683 | 30/12/2021 | Africa / Egypt / Red sea governorate / Hurgada | Human | unknown | Male | 46 | Hotel isolation | unknown | unknown | B.1.617.2 | GK |
| hCoV-19/Egypt/CPHL-EGY21324/2021 | EPI_ISL_9047662 | 29/12/2021 | Africa / Egypt / Red sea governorate / Hurgada | Human | unknown | Male | 41 | Hotel isolation | unknown | unknown | B.1.617.2 | GK |
| hCoV-19/Egypt/CPHL-EGY21446/2021 | EPI_ISL_9047775 | 19/11/2021 | Africa / Egypt / Red sea governorate / Hurgada | Human | unknown | Male | 25 | Hotel isolation | unknown | unknown | B.1.617.2 | GK |
| hCoV-19/Egypt/CPHL-EGY21032/2021 | EPI_ISL_9047384 | 05/12/2021 | Africa / Egypt / Menofia governorate / Shebin el kom | Human | unknown | Female | 41 | Hospitalized | unknown | unknown | AY.122 | GK |
| hCoV-19/Egypt/CPHL-EGY21347/2021 | EPI_ISL_9047684 | 31/12/2021 | Africa / Egypt / Red sea governorate / Hurgada | Human | unknown | Male | 50 | Hotel isolation | unknown | unknown | B.1.617.2 | GK |
| hCoV-19/Egypt/CPHL-EGY21325/2021 | EPI_ISL_9047663 | 29/12/2021 | Africa / Egypt / Red sea governorate / Hurgada | Human | unknown | Male | 25 | Hotel isolation | unknown | unknown | B.1.617.2 | GK |
| hCoV-19/Egypt/CPHL-EGY21135/2021 | EPI_ISL_9047485 | 28/11/2021 | Africa / Egypt / Red sea governorate / Safaga | Human | unknown | Female | 43 | Hotel isolation | unknown | unknown | B.1.617.2 | GK |
| hCoV-19/Egypt/CPHL-EGY21423/2022 | EPI_ISL_9047754 | 02/01/2022 | Africa / Egypt / Red sea governorate / Hurgada | Human | unknown | Female | 25 | Hotel isolation | unknown | unknown | B.1.617.2 | GK |
| hCoV-19/Egypt/CPHL-EGY21071/2021 | EPI_ISL_9047423 | 17/12/2021 | Africa / Egypt / Red sea governorate / Safaga | Human | unknown | Female | 44 | Hotel isolation | unknown | unknown | B.1.617.2 | GK |
| hCoV-19/Egypt/CPHL-EGY21165/2021 | EPI_ISL_9047515 | 06/12/2021 | Africa / Egypt / Red sea governorate / Safaga | Human | unknown | Female | 57 | Hotel isolation | unknown | unknown | AY.127 | GK |
| hCoV-19/Egypt/CPHL-EGY21034/2021 | EPI_ISL_9047386 | 12/12/2021 | Africa / Egypt / Menofia governorate / Shebin el kom | Human | unknown | Male | 65 | Hospitalized | unknown | unknown | AY.127 | GK |
| hCoV-19/Egypt/CPHL-EGY21239/2021 | EPI_ISL_9047586 | 19/12/2021 | Africa / Egypt / Red sea governorate / Hurgada | Human | unknown | Male | 19 | Hotel isolation | unknown | unknown | B.1.617.2 | GK |
| hCoV-19/Egypt/CPHL-EGY21215/2021 | EPI_ISL_9047564 | 12/02/2021 | Africa / Egypt / Dammita governorate | Human | unknown | Female | 28 | Hospitalized | unknown | unknown | AY.4 | GK |
| hCoV-19/Egypt/CPHL-EGY21101/2021 | EPI_ISL_9047453 | 17/12/2021 | Africa / Egypt / Red sea governorate / Safaga | Human | unknown | Female | 24 | Hotel isolation | unknown | unknown | B.1.617.2 | GK |
| hCoV-19/Egypt/CPHL-EGY21240/2021 | EPI_ISL_9047587 | 19/12/2021 | Africa / Egypt / Red sea governorate / Hurgada | Human | unknown | Male | 8 | Hotel isolation | unknown | unknown | AY.122 | GK |
| hCoV-19/Egypt/CPHL-EGY21350/2021 | EPI_ISL_9047687 | 31/12/2021 | Africa / Egypt / Red sea governorate / Hurgada | Human | unknown | Male | 44 | Hotel isolation | unknown | unknown | B.1.617.2 | GK |
| hCoV-19/Egypt/CPHL-EGY21260/2021 | EPI_ISL_9047607 | 08/12/2021 | Africa / Egypt / Red sea governorate / Hurgada | Human | unknown | Female | 69 | Hotel isolation | unknown | unknown | AY.122 | GK |
| hCoV-19/Egypt/CPHL-EGY21314/2021 | EPI_ISL_9047653 | 28/12/2021 | Africa / Egypt / Aswan governorate | Human | unknown | Female | 52 | Hospitalized | unknown | unknown | Unassigned | G |
| hCoV-19/Egypt/CPHL-EGY21036/2021 | EPI_ISL_9047388 | 12/12/2021 | Africa / Egypt / Menofia governorate / Shebin el kom | Human | unknown | Male | 73 | Hospitalized | unknown | unknown | AY.43 (consensus call) | GK |
| hCoV-19/Egypt/CPHL-EGY21351/2021 | EPI_ISL_9047688 | 31/12/2021 | Africa / Egypt / Red sea governorate / Hurgada | Human | unknown | Male | 46 | Hotel isolation | unknown | unknown | AY.122 (consensus call) | GK |
| hCoV-19/Egypt/UC-006/2021 | EPI_ISL_16871425 | 19/03/2021 | Africa / Egypt / Giza | Human | unknown | unknown | unknown | unknown | unknown | unknown | B.1.617.2 | GK |
| hCoV-19/Egypt/UC-012/2021 | EPI_ISL_16871420 | 09/06/2021 | Africa / Egypt / Giza | Human | unknown | unknown | unknown | unknown | unknown | unknown | B.1.617.2 | GK |
| hCoV-19/Egypt/CPHL-EGY21153/2021 | EPI_ISL_9047503 | 07/11/2021 | Africa / Egypt / Red sea governorate / Safaga | Human | unknown | Male | 56 | Hotel isolation | unknown | unknown | B.1.617.2 | GK |
| hCoV-19/Egypt/CPHL-EGY21139/2021 | EPI_ISL_9047489 | 30/11/2021 | Africa / Egypt / Red sea governorate / Safaga | Human | unknown | Female | 56 | Hotel isolation | unknown | unknown | B.1.617.2 | GK |
| hCoV-19/Egypt/CPHL-EGY21352/2021 | EPI_ISL_9047689 | 31/12/2021 | Africa / Egypt / Red sea governorate / Hurgada | Human | unknown | Male | 65 | Hotel isolation | unknown | unknown | B.1.617.2 | GK |
| hCoV-19/Egypt/CPHL-EGY21460/2021 | EPI_ISL_9047789 | 20/11/2021 | Africa / Egypt / Aswan governorate | Human | unknown | Male | 36 | Hospitalized | unknown | unknown | B.1.617.2 | GK |
| hCoV-19/Egypt/CPHL-EGY21353/2021 | EPI_ISL_9047690 | 31/12/2021 | Africa / Egypt / Red sea governorate / Hurgada | Human | unknown | Female | 58 | Hotel isolation | unknown | unknown | AY.122 (consensus call) | GK |
| hCoV-19/Egypt/CPHL-EGY21293/2021 | EPI_ISL_9047636 | 01/12/2021 | Africa / Egypt / Red sea governorate / Hurgada | Human | unknown | Female | 62 | Hotel isolation | unknown | unknown | B.1.617.2 | GK |
| hCoV-19/Egypt/CPHL-EGY21030/2021 | EPI_ISL_9047382 | 04/12/2021 | Africa / Egypt / Menofia governorate / Shebin el kom | Human | unknown | Female | 65 | Hospitalized | unknown | unknown | AY.43 | GK |
| hCoV-19/Egypt/CPHL-EGY21345/2021 | EPI_ISL_9047682 | 30/12/2021 | Africa / Egypt / Red sea governorate / Hurgada | Human | unknown | Female | 49 | Hotel isolation | unknown | unknown | B.1.617.2 | O |
| hCoV-19/Egypt/CPHL-EGY21462/2021 | EPI_ISL_9047791 | 20/09/2021 | Africa / Egypt / Aswan governorate | Human | unknown | Female | 55 | Hospitalized | unknown | unknown | B.1.617.2 | GK |
| hCoV-19/Egypt/CPHL-EGY21243/2021 | EPI_ISL_9047590 | 19/12/2021 | Africa / Egypt / Red sea governorate / Hurgada | Human | unknown | Female | 25 | Hotel isolation | unknown | unknown | B.1.617.2 | GK |
| hCoV-19/Egypt/CPHL-EGY21103/2021 | EPI_ISL_9047455 | 17/12/2021 | Africa / Egypt / Red sea governorate / Safaga | Human | unknown | Female | 24 | Hotel isolation | unknown | unknown | AY.122 | GK |
| hCoV-19/Egypt/CPHL-EGY21024/2021 | EPI_ISL_9047376 | 03/12/2021 | Africa / Egypt / Menofia governorate / Shebin el kom | Human | unknown | Female | 42 | Hospitalized | unknown | unknown | B.1.617.2 | GK |
| hCoV-19/Egypt/CPHL-EGY21205/2021 | EPI_ISL_9047554 | 10/08/2021 | Africa / Egypt / Alexandria governorate | Human | unknown | Female | 34 | Hospitalized | unknown | unknown | AY.4 | GK |
| hCoV-19/Egypt/CPHL-EGY21475/2021 | EPI_ISL_9047804 | 29/09/2021 | Africa / Egypt / Aswan governorate | Human | unknown | Male | 41 | Hospitalized | unknown | unknown | B.1.617.2 | GK |
| hCoV-19/Egypt/CPHL-EGY21143/2021 | EPI_ISL_9047493 | 10/05/2021 | Africa / Egypt / Red sea governorate / Safaga | Human | unknown | Female | 72 | Hotel isolation | unknown | unknown | B.1.617.2 | GK |
| hCoV-19/Egypt/CPHL-EGY21464/2021 | EPI_ISL_9047793 | 21/09/2021 | Africa / Egypt / Aswan governorate | Human | unknown | Female | 48 | Hospitalized | unknown | unknown | B.1.617.2 | GK |
| hCoV-19/Egypt/CPHL-EGY21247/2021 | EPI_ISL_9047594 | 23/12/2021 | Africa / Egypt / Red sea governorate / Hurgada | Human | unknown | Female | 26 | Hotel isolation | unknown | unknown | AY.122 | GK |
| hCoV-19/Egypt/CPHL-EGY21144/2020 | EPI_ISL_9047494 | 13/12/2020 | Africa / Egypt / Red sea governorate / Safaga | Human | unknown | Female | 71 | Hotel isolation | unknown | unknown | B.1.617.2 | GK |
| hCoV-19/Egypt/CPHL-EGY21042/2021 | EPI_ISL_9047394 | 12/12/2021 | Africa / Egypt / Menofia governorate / Shebin el kom | Human | unknown | Female | 65 | Hospitalized | unknown | unknown | C.36.3 | GR |
| hCoV-19/Egypt/CPHL-EGY21307/2021 | EPI_ISL_9047647 | 05/12/2021 | Africa / Egypt / Red sea governorate / Hurgada | Human | unknown | Male | 55 | Hotel isolation | unknown | unknown | B.1.617.2 | GK |
| hCoV-19/Egypt/CPHL-EGY21437/2022 | EPI_ISL_9047766 | 08/01/2022 | Africa / Egypt / Red sea governorate / Hurgada | Human | unknown | Male | 26 | Hotel isolation | unknown | unknown | B.1.617.2 | GK |
| hCoV-19/Egypt/CPHL-EGY21382/2021 | EPI_ISL_9047716 | 31/12/2021 | Africa / Egypt / Red sea governorate / Hurgada | Human | unknown | Female | 19 | Hotel isolation | unknown | unknown | Unassigned | GV |
| hCoV-19/Egypt/CPHL-EGY21245/2021 | EPI_ISL_9047592 | 13/12/2021 | Africa / Egypt / Red sea governorate / Hurgada | Human | unknown | Male | 56 | Hotel isolation | unknown | unknown | Unassigned | GRA |
| hCoV-19/Egypt/CPHL-EGY21223/2021 | EPI_ISL_9047572 | 13/12/2021 | Africa / Egypt / Red sea governorate / Safaga | Human | unknown | Male | 49 | Hotel isolation | unknown | unknown | Unassigned | GRA |
| hCoV-19/Egypt/CPHL-EGY21340/2022 | EPI_ISL_9047677 | 03/01/2022 | Africa / Egypt / Aswan governorate | Human | unknown | Female | 55 | Hospitalized | unknown | unknown | Unassigned | GRA |
| hCoV-19/Egypt/CPHL-EGY21449/2021 | EPI_ISL_9047778 | 08/11/2021 | Africa / Egypt / Aswan governorate | Human | unknown | Male | 49 | Hospitalized | unknown | unknown | Unassigned | GRA |
| hCoV-19/Egypt/CPHL-EGY21014/2021 | EPI_ISL_9047366 | 01/12/2021 | Africa / Egypt / Menofia governorate / Shebin el kom | Human | unknown | Female | 54 | Hospitalized | unknown | unknown | B.1.617.2 | GK |
| hCoV-19/Egypt/CPHL-EGY21249/2021 | EPI_ISL_9047596 | 04/12/2021 | Africa / Egypt / Red sea governorate / Hurgada | Human | unknown | Male | 34 | Hotel isolation | unknown | unknown | B.1.617.2 | GK |
| hCoV-19/Egypt/CPHL-EGY21237/2021 | EPI_ISL_9047584 | 17/12/2021 | Africa / Egypt / Red sea governorate / Hurgada | Human | unknown | Male | 36 | Hotel isolation | unknown | unknown | B.1.617.2 | GK |
| hCoV-19/Egypt/CPHL-EGY21361/2021 | EPI_ISL_9047696 | 31/12/2021 | Africa / Egypt / Red sea governorate / Hurgada | Human | unknown | Female | 54 | Hotel isolation | unknown | unknown | B.1.617.2 | GK |
| hCoV-19/Egypt/CPHL-EGY21146/2021 | EPI_ISL_9047496 | 26/08/2021 | Africa / Egypt / Red sea governorate / Safaga | Human | unknown | Female | 44 | Hotel isolation | unknown | unknown | AY.98.1 | GK |
| hCoV-19/Egypt/ARMY-BPD-ECRRM0134/2022 | EPI_ISL_14594552 | 28/07/2022 | Africa / Egypt / Suez | Human | unknown | Female | 43 | Hospitalized | Sinopharm | unknown | BA.2 | O |
| hCoV-19/Egypt/ARMY-BPD-ECRRM0133/2022 | EPI_ISL_14594551 | 20/07/2022 | Africa / Egypt / Fayoum | Human | unknown | Female | 44 | Hospitalized | Sinopharm | unknown | BA.2 | O |
| hCoV-19/Egypt/ARMY-BPD-ECRRM0112/2022 | EPI_ISL_14594530 | 19/06/2022 | Africa / Egypt / Cairo | Human | unknown | Male | 55 | Hospitalized | Sinopharm | unknown | BA.5.2 | O |
| hCoV-19/Egypt/CPHL-EGY21450/2021 | EPI_ISL_9047779 | 09/11/2021 | Africa / Egypt / Aswan governorate | Human | unknown | Female | 46 | Hospitalized | unknown | unknown | B.1.617.2 | GK |
| hCoV-19/Egypt/CPHL-EGY21045/2021 | EPI_ISL_9047397 | 12/12/2021 | Africa / Egypt / Menofia governorate / Shebin el kom | Human | unknown | Female | 24 | Hospitalized | unknown | unknown | B.1.1.7 | GRY |
| hCoV-19/Egypt/CPHL-EGY21250/2021 | EPI_ISL_9047597 | 04/12/2021 | Africa / Egypt / Red sea governorate / Hurgada | Human | unknown | Male | 39 | Hotel isolation | unknown | unknown | B.1.617.2 | GK |
| hCoV-19/Egypt/USC-6/2021 | EPI_ISL_8470721 | 2021-01 | Africa / Egypt / Cairo | Human | unknown | unknown | unknown | unknown | unknown | unknown | Unassigned | O |
| hCoV-19/Egypt/CPHL-EGY21348/2021 | EPI_ISL_9047685 | 31/12/2021 | Africa / Egypt / Red sea governorate / Hurgada | Human | unknown | Male | 45 | Hotel isolation | unknown | unknown | AY.122 | GK |
| hCoV-19/Egypt/CPHL-EGY21120/2021 | EPI_ISL_9047472 | 05/12/2021 | Africa / Egypt / Red sea governorate / Safaga | Human | unknown | Male | 44 | Hotel isolation | unknown | unknown | B.1.617.2 | GK |
| hCoV-19/Egypt/CPHL-EGY21251/2021 | EPI_ISL_9047598 | 04/12/2021 | Africa / Egypt / Red sea governorate / Hurgada | Human | unknown | Male | 65 | Hotel isolation | unknown | unknown | B.1.617.2 | GK |
| hCoV-19/Egypt/CPHL-EGY21469/2021 | EPI_ISL_9047798 | 26/09/2021 | Africa / Egypt / Aswan governorate | Human | unknown | Male | 39 | Hospitalized | unknown | unknown | B.1.617.2 | GK |
| hCoV-19/Egypt/ARMY-ECRRM0129/2021 | EPI_ISL_14594263 | 24/12/2021 | Africa / Egypt / Fayoum | Human | unknown | Female | 43 | Hospitalized | Sinopharm | unknown | B.1.617.2 | GK |
| hCoV-19/Egypt/CPHL-EGY21218/2021 | EPI_ISL_9047567 | 12/02/2021 | Africa / Egypt / Red sea governorate / Safaga | Human | unknown | Male | 39 | Hospitalized | unknown | unknown | B.1.617.2 | GK |
| hCoV-19/Egypt/UC-016/2021 | EPI_ISL_16871416 | 18/08/2021 | Africa / Egypt / Giza | Human | unknown | unknown | unknown | unknown | unknown | unknown | Unassigned | O |
| hCoV-19/Egypt/ARMY-ECRRM0084/2021 | EPI_ISL_14593731 | 19/12/2021 | Africa / Egypt / Cairo | Human | unknown | Female | 43 | Hospitalized | Sinopharm | unknown | C.36.3 | GR |
| hCoV-19/Egypt/ARMY-ECRRM0082/2021 | EPI_ISL_14593730 | 19/12/2021 | Africa / Egypt / Cairo | Human | unknown | Female | 44 | Hospitalized | Sinopharm | unknown | C.36.3 | GR |
| hCoV-19/Egypt/ARMY-ECRRM0081/2021 | EPI_ISL_14593729 | 19/12/2021 | Africa / Egypt / Cairo | Human | unknown | Female | 76 | Hospitalized | Sinopharm | unknown | C.36.3 | GR |
| hCoV-19/Egypt/ARMY-ECRRM0085/2021 | EPI_ISL_14593732 | 19/12/2021 | Africa / Egypt / Cairo | Human | unknown | Female | 41 | Hospitalized | Sinopharm | unknown | C.36.3 | GR |
| hCoV-19/Egypt/ARMY-ECRRM0079/2021 | EPI_ISL_14593728 | 19/12/2021 | Africa / Egypt / Cairo | Human | unknown | Female | 67 | Hospitalized | Sinopharm | unknown | C.36.3 | GR |
| hCoV-19/Egypt/ARMY-ECRRM0078/2021 | EPI_ISL_14593727 | 19/12/2021 | Africa / Egypt / Cairo | Human | unknown | Female | 56 | Hospitalized | Sinopharm | unknown | C.36.3 | GR |
| hCoV-19/Egypt/ARMY-ECRRM0058/2021 | EPI_ISL_14593714 | 09/12/2021 | Africa / Egypt / Cairo | Human | unknown | Male | 33 | Hospitalized | Sinopharm | unknown | B.1.617.2 | GK |
| hCoV-19/Egypt/CPHL-EGY21330/2021 | EPI_ISL_9047668 | 29/12/2021 | Africa / Egypt / Red sea governorate / Hurgada | Human | unknown | Female | 33 | Hotel isolation | unknown | unknown | AY.122 | GK |
| hCoV-19/Egypt/CPHL-EGY21016/2021 | EPI_ISL_9047368 | 01/12/2021 | Africa / Egypt / Menofia governorate / Shebin el kom | Human | unknown | Male | 58 | Hospitalized | unknown | unknown | AY.106 | GK |
| hCoV-19/Egypt/CPHL-EGY21220/2021 | EPI_ISL_9047569 | 12/02/2021 | Africa / Egypt / Red sea governorate / Safaga | Human | unknown | Female | 55 | Hospitalized | unknown | unknown | AY.122 | GK |
| hCoV-19/Egypt/CPHL-EGY21017/2021 | EPI_ISL_9047369 | 01/12/2021 | Africa / Egypt / Menofia governorate / Shebin el kom | Human | unknown | Male | 65 | Hospitalized | unknown | unknown | B.1.617.2 | GK |
| hCoV-19/Egypt/CPHL-EGY21440/2022 | EPI_ISL_9047769 | 08/01/2022 | Africa / Egypt / Red sea governorate / Hurgada | Human | unknown | Male | 30 | Hotel isolation | unknown | unknown | B.1.617.2 | GK |
| hCoV-19/Egypt/CPHL-EGY21331/2021 | EPI_ISL_9047669 | 29/12/2021 | Africa / Egypt / Red sea governorate / Hurgada | Human | unknown | Male | 30 | Hotel isolation | unknown | unknown | B.1.617.2 | GK |
| hCoV-19/Egypt/CPHL-EGY21020/2021 | EPI_ISL_9047372 | 01/12/2021 | Africa / Egypt / Menofia governorate / Shebin el kom | Human | unknown | Male | 55 | Hospitalized | unknown | unknown | AY.127 | GK |
| hCoV-19/Egypt/CPHL-EGY21443/2022 | EPI_ISL_9047772 | 08/01/2022 | Africa / Egypt / Red sea governorate / Hurgada | Human | unknown | Female | 26 | Hotel isolation | unknown | unknown | AY.111 | GK |
| hCoV-19/Egypt/CPHL-EGY21336/2021 | EPI_ISL_9047673 | 31/12/2021 | Africa / Egypt / Red sea governorate / Hurgada | Human | unknown | Male | 48 | Hotel isolation | unknown | unknown | B.1.617.2 | GK |
| hCoV-19/Egypt/CPHL-EGY21121/2021 | EPI_ISL_9047473 | 05/12/2021 | Africa / Egypt / Red sea governorate / Safaga | Human | unknown | Female | 40 | Hotel isolation | unknown | unknown | B.1.617.2 | GK |
| hCoV-19/Egypt/CPHL-EGY21337/2022 | EPI_ISL_9047674 | 01/01/2022 | Africa / Egypt / Red sea governorate / Hurgada | Human | unknown | Male | 58 | Hotel isolation | unknown | unknown | AY.122 (consensus call) | GK |
| hCoV-19/Egypt/CPHL-EGY21022/2021 | EPI_ISL_9047374 | 03/12/2021 | Africa / Egypt / Menofia governorate / Shebin el kom | Human | unknown | Male | 39 | Hospitalized | unknown | unknown | AY.43 | GK |
| hCoV-19/Egypt/CPHL-EGY21023/2021 | EPI_ISL_9047375 | 03/12/2021 | Africa / Egypt / Menofia governorate / Shebin el kom | Human | unknown | Male | 72 | Hospitalized | unknown | unknown | AY.122 | GK |
| hCoV-19/Egypt/CPHL-EGY21338/2022 | EPI_ISL_9047675 | 02/01/2022 | Africa / Egypt / Red sea governorate / Hurgada | Human | unknown | Male | 65 | Hotel isolation | unknown | unknown | B.1.617.2 | GK |
| hCoV-19/Egypt/CPHL-EGY21229/2021 | EPI_ISL_9047576 | 01/12/2021 | Africa / Egypt / Red sea governorate / Safaga | Human | unknown | Female | 48 | Hotel isolation | unknown | unknown | B.1.617.2 | GK |
| hCoV-19/Egypt/CPHL-EGY21124/2021 | EPI_ISL_9047476 | 15/12/2021 | Africa / Egypt / Red sea governorate / Safaga | Human | unknown | Female | 42 | Hotel isolation | unknown | unknown | B.1.617.2 | GK |
| hCoV-19/Egypt/CPHL-EGY21316/2021 | EPI_ISL_9047655 | 28/12/2021 | Africa / Egypt / Red sea governorate / Hurgada | Human | unknown | Female | 21 | Hotel isolation | unknown | unknown | B.1.617.2 | O |
| hCoV-19/Egypt/CPHL-EGY21026/2021 | EPI_ISL_9047378 | 03/12/2021 | Africa / Egypt / Menofia governorate / Shebin el kom | Human | unknown | Female | 67 | Hospitalized | unknown | unknown | AY.43 | GK |
| hCoV-19/Egypt/CPHL-EGY22177/2022 | EPI_ISL_14584143 | 17/07/2022 | Africa / Egypt | Human | unknown | Female | unknown | non hospitalized | unknown | unknown | BE.4 | GRA |
| hCoV-19/Egypt/CPHL-EGY22163/2022 | EPI_ISL_14584130 | 16/07/2022 | Africa / Egypt | Human | unknown | Female | unknown | non hospitalized | unknown | unknown | C.17 | GR |
| hCoV-19/Egypt/CPHL-EGY22178/2022 | EPI_ISL_14584144 | 17/07/2022 | Africa / Egypt | Human | unknown | Male | unknown | non hospitalized | unknown | unknown | BA.5.2 | GRA |
| hCoV-19/Egypt/CPHL-EGY22161/2022 | EPI_ISL_14584128 | 16/07/2022 | Africa / Egypt | Human | unknown | Female | unknown | non hospitalized | unknown | unknown | C.17 | GR |
| hCoV-19/Egypt/CPHL-EGY22129/2022 | EPI_ISL_14584099 | 04/07/2022 | Africa / Egypt | Human | unknown | Male | 49 | hospitalized | unknown | unknown | BE.4 | GRA |
| hCoV-19/Egypt/CPHL-EGY22124/2022 | EPI_ISL_14584094 | 03/07/2022 | Africa / Egypt | Human | unknown | Female | 41 | hospitalized | unknown | unknown | BA.5.2.21 | GRA |
| hCoV-19/Egypt/CPHL-EGY22123/2022 | EPI_ISL_14584093 | 04/07/2022 | Africa / Egypt | Human | unknown | NA | 21 | hospitalized | unknown | unknown | BA.5.2 | GRA |
| hCoV-19/Egypt/CPHL-EGY22106/2022 | EPI_ISL_14584080 | 29/06/2022 | Africa / Egypt | Human | unknown | Male | 43 | hospitalized | unknown | unknown | BA.5.2.20 | GRA |
| hCoV-19/Egypt/CPHL-EGY22162/2022 | EPI_ISL_14584129 | 16/07/2022 | Africa / Egypt | Human | unknown | Female | unknown | non hospitalized | unknown | unknown | BA.5.2.21 | GRA |
| hCoV-19/Egypt/CPHL-EGY22096/2022 | EPI_ISL_14584071 | 29/06/2022 | Africa / Egypt | Human | unknown | NA | unknown | non hospitalized | unknown | unknown | BA.5.2 | GRA |
| hCoV-19/Egypt/CPHL-EGY22085/2022 | EPI_ISL_14584062 | 29/06/2022 | Africa / Egypt | Human | unknown | Female | unknown | non hospitalized | unknown | unknown | BA.5.2 | GRA |
| hCoV-19/Egypt/CPHL-EGY21342/2022 | EPI_ISL_9047679 | 05/01/2022 | Africa / Egypt / Red sea governorate / Hurgada | Human | unknown | Male | 31 | Hotel isolation | unknown | unknown | B.1.617.2 | GK |
| hCoV-19/Egypt/CPHL-EGY21343/2022 | EPI_ISL_9047680 | 06/01/2022 | Africa / Egypt / Red sea governorate / Hurgada | Human | unknown | Male | 32 | Hotel isolation | unknown | unknown | AY.122 | GK |
| hCoV-19/Egypt/CPHL-EGY21451/2021 | EPI_ISL_9047780 | 09/11/2021 | Africa / Egypt / Aswan governorate | Human | unknown | Female | 48 | Hospitalized | unknown | unknown | B.1.617.2 | GK |
| hCoV-19/Egypt/CPHL-EGY21252/2021 | EPI_ISL_9047599 | 04/12/2021 | Africa / Egypt / Red sea governorate / Hurgada | Human | unknown | Male | 64 | Hotel isolation | unknown | unknown | B.1.617.2 | GK |
| hCoV-19/Egypt/CPHL-EGY21470/2021 | EPI_ISL_9047799 | 26/09/2021 | Africa / Egypt / Aswan governorate | Human | unknown | Female | 25 | Hospitalized | unknown | unknown | B.1.617.2 | GK |
| hCoV-19/Egypt/CPHL-EGY21429/2022 | EPI_ISL_9047758 | 02/01/2022 | Africa / Egypt / Red sea governorate / Hurgada | Human | unknown | Female | 26 | Hotel isolation | unknown | unknown | Unassigned | GK |
| hCoV-19/Egypt/CPHL-EGY21126/2021 | EPI_ISL_9047478 | 15/12/2021 | Africa / Egypt / Red sea governorate / Safaga | Human | unknown | Female | 52 | Hotel isolation | unknown | unknown | Unassigned | G |
| hCoV-19/Egypt/CPHL-EGY21227/2021 | EPI_ISL_9047575 | 01/12/2021 | Africa / Egypt / Red sea governorate / Safaga | Human | unknown | Male | 22 | Hotel isolation | unknown | unknown | Unassigned | GK |
| hCoV-19/Egypt/CPHL-EGY21203/2021 | EPI_ISL_9047552 | 17/08/2021 | Africa / Egypt / Alexandria governorate | Human | unknown | Female | 34 | Hospitalized | unknown | unknown | Unassigned | GK |
| hCoV-19/Egypt/CPHL-EGY21211/2021 | EPI_ISL_9047560 | 04/12/2021 | Africa / Egypt / Red sea governorate / Safaga | Human | unknown | Female | 45 | Hospitalized | unknown | unknown | Unassigned | G |
| hCoV-19/Egypt/CPHL-EGY21457/2021 | EPI_ISL_9047786 | 15/11/2021 | Africa / Egypt / Aswan governorate | Human | unknown | Male | 63 | Hospitalized | unknown | unknown | Unassigned | GK |
| hCoV-19/Egypt/CPHL-EGY21452/2021 | EPI_ISL_9047781 | 09/11/2021 | Africa / Egypt / Aswan governorate | Human | unknown | Male | 21 | Hospitalized | unknown | unknown | Unassigned | GK |
| hCoV-19/Egypt/CPHL-EGY21269/2021 | EPI_ISL_9047616 | 10/12/2021 | Africa / Egypt / Red sea governorate / Hurgada | Human | unknown | Female | 52 | Hotel isolation | unknown | unknown | Unassigned | G |
| hCoV-19/Egypt/CPHL-EGY21106/2021 | EPI_ISL_9047458 | 19/12/2021 | Africa / Egypt / Red sea governorate / Safaga | Human | unknown | Male | 18 | Hotel isolation | unknown | unknown | B.1.617.2 | GK |
| hCoV-19/Egypt/CPHL-EGY21134/2021 | EPI_ISL_9047484 | 28/11/2021 | Africa / Egypt / Red sea governorate / Safaga | Human | unknown | Male | 45 | Hotel isolation | unknown | unknown | B.1.617.2 | O |
| hCoV-19/Egypt/CPHL-EGY21108/2021 | EPI_ISL_9047460 | 19/12/2021 | Africa / Egypt / Red sea governorate / Safaga | Human | unknown | Male | 37 | Hotel isolation | unknown | unknown | B.1.617.2 | GK |
| hCoV-19/Egypt/CPHL-EGY21180/2021 | EPI_ISL_9047530 | 10/12/2021 | Africa / Egypt / Red sea governorate / Safaga | Human | unknown | Female | 22 | Hotel isolation | unknown | unknown | B.1.617.2 | GK |
| hCoV-19/Egypt/CPHL-EGY21096/2021 | EPI_ISL_9047448 | 14/12/2021 | Africa / Egypt / Red sea governorate / Safaga | Human | unknown | Male | 10 | Hotel isolation | unknown | unknown | B.1.617.2 | GK |
| hCoV-19/Egypt/CPHL-EGY21435/2022 | EPI_ISL_9047764 | 02/01/2022 | Africa / Egypt / Red sea governorate / Hurgada | Human | unknown | Female | 28 | Hotel isolation | unknown | unknown | B.1.617.2 | GK |
| hCoV-19/Egypt/CPHL-EGY21224/2021 | EPI_ISL_9047573 | 01/12/2021 | Africa / Egypt / Red sea governorate / Safaga | Human | unknown | Female | 58 | Hotel isolation | unknown | unknown | B.1.617.2 | GK |
| hCoV-19/Egypt/CPHL-EGY21200/2021 | EPI_ISL_9047549 | 27/08/2021 | Africa / Egypt / Alexandria governorate | Human | unknown | Female | 33 | Hospitalized | unknown | unknown | B.1.617.2 | GK |
| hCoV-19/Egypt/CPHL-EGY21439/2022 | EPI_ISL_9047768 | 07/01/2022 | Africa / Egypt / Red sea governorate / Hurgada | Human | unknown | Female | 28 | Hotel isolation | unknown | unknown | B.1.617.2 | GK |
| hCoV-19/Egypt/CPHL-EGY21186/2021 | EPI_ISL_9047535 | 15/11/2021 | Africa / Egypt / Red sea governorate / Safaga | Human | unknown | Female | 44 | Hospitalized | unknown | unknown | B.1.617.2 | GV |
| hCoV-19/Egypt/CPHL-EGY21354/2021 | EPI_ISL_9047691 | 31/12/2021 | Africa / Egypt / Red sea governorate / Hurgada | Human | unknown | Male | 54 | Hotel isolation | unknown | unknown | B.1.617.2 | GK |
| hCoV-19/Egypt/CPHL-EGY21432/2022 | EPI_ISL_9047761 | 02/01/2022 | Africa / Egypt / Red sea governorate / Hurgada | Human | unknown | Male | 26 | Hotel isolation | unknown | unknown | B.1.617.2 | GK |
| hCoV-19/Egypt/CPHL-EGY21111/2021 | EPI_ISL_9047463 | 19/12/2021 | Africa / Egypt / Red sea governorate / Safaga | Human | unknown | Female | 60 | Hotel isolation | unknown | unknown | B.1.617.2 | GK |
| hCoV-19/Egypt/CPHL-EGY21107/2021 | EPI_ISL_9047459 | 19/12/2021 | Africa / Egypt / Red sea governorate / Safaga | Human | unknown | Female | 17 | Hotel isolation | unknown | unknown | B.1.617.2 | GK |
| hCoV-19/Egypt/CPHL-EGY21326/2021 | EPI_ISL_9047664 | 29/12/2021 | Africa / Egypt / Red sea governorate / Hurgada | Human | unknown | Male | 29 | Hotel isolation | unknown | unknown | B.1.617.2 | GK |
| hCoV-19/Egypt/CPHL-EGY21190/2021 | EPI_ISL_9047539 | 12/11/2021 | Africa / Egypt / Red sea governorate / Safaga | Human | unknown | Female | 10 | Hospitalized | unknown | unknown | B.1.617.2 | GK |
| hCoV-19/Egypt/CPHL-EGY21031/2021 | EPI_ISL_9047383 | 04/12/2021 | Africa / Egypt / Menofia governorate / Shebin el kom | Human | unknown | Male | 44 | Hospitalized | unknown | unknown | AY.43 | GK |
| hCoV-19/Egypt/CPHL-EGY21212/2021 | EPI_ISL_9047561 | 06/12/2021 | Africa / Egypt / Red sea governorate / Safaga | Human | unknown | Female | 44 | Hospitalized | unknown | unknown | AY.43 (consensus call) | GK |
| hCoV-19/Egypt/CPHL-EGY21416/2022 | EPI_ISL_9047747 | 02/01/2022 | Africa / Egypt / Red sea governorate / Hurgada | Human | unknown | Male | 24 | Hotel isolation | unknown | unknown | AY.111 | GK |
| hCoV-19/Egypt/CPHL-EGY21015/2021 | EPI_ISL_9047367 | 01/12/2021 | Africa / Egypt / Menofia governorate / Shebin el kom | Human | unknown | Female | 56 | Hospitalized | unknown | unknown | B.1.617.2 | GK |
| hCoV-19/Egypt/CPHL-EGY21272/2021 | EPI_ISL_9047618 | 11/12/2021 | Africa / Egypt / Red sea governorate / Hurgada | Human | unknown | Female | 39 | Hotel isolation | unknown | unknown | Unassigned | GK |
| hCoV-19/Egypt/CPHL-EGY21441/2022 | EPI_ISL_9047770 | 08/01/2022 | Africa / Egypt / Red sea governorate / Hurgada | Human | unknown | Male | 32 | Hotel isolation | unknown | unknown | B.1.617.2 | GK |
| hCoV-19/Egypt/CPHL-EGY21442/2022 | EPI_ISL_9047771 | 07/01/2022 | Africa / Egypt / Red sea governorate / Hurgada | Human | unknown | Female | 28 | Hotel isolation | unknown | unknown | AY.122 | GK |
| hCoV-19/Egypt/CPHL-EGY21335/2021 | EPI_ISL_9047672 | 30/12/2021 | Africa / Egypt / Red sea governorate / Hurgada | Human | unknown | Female | 47 | Hotel isolation | unknown | unknown | AY.122 | GK |
| hCoV-19/Egypt/CPHL-EGY21236/2021 | EPI_ISL_9047583 | 17/12/2021 | Africa / Egypt / Red sea governorate / Hurgada | Human | unknown | Male | 64 | Hotel isolation | unknown | unknown | Unassigned | G |
| hCoV-19/Egypt/CPHL-EGY22622/2022 | EPI_ISL_15670140 | 02/10/2022 | Africa / Egypt | Human | unknown | unknown | unknown | non hospitalized | unknown | unknown | C.36.3 | GR |
| hCoV-19/Egypt/CPHL-EGY22621/2022 | EPI_ISL_15670139 | 23/08/2022 | Africa / Egypt | Human | unknown | unknown | unknown | non hospitalized | unknown | unknown | BA.5.2 | GRA |
| hCoV-19/Egypt/CPHL-EGY22620/2022 | EPI_ISL_15670138 | 21/09/2022 | Africa / Egypt | Human | unknown | unknown | unknown | non hospitalized | unknown | unknown | BA.5.2.34 | GRA |
| hCoV-19/Egypt/CPHL-EGY22619/2022 | EPI_ISL_15670137 | 14/09/2022 | Africa / Egypt | Human | unknown | unknown | unknown | non hospitalized | unknown | unknown | C.17 | GR |
| hCoV-19/Egypt/CPHL-EGY22618/2022 | EPI_ISL_15670136 | 14/09/2022 | Africa / Egypt | Human | unknown | unknown | unknown | non hospitalized | unknown | unknown | BA.5.2 | GRA |
| hCoV-19/Egypt/CPHL-EGY22617/2022 | EPI_ISL_15670135 | 02/10/2022 | Africa / Egypt | Human | unknown | unknown | unknown | non hospitalized | unknown | unknown | BA.5.2 | GRA |
| hCoV-19/Egypt/CPHL-EGY22615/2022 | EPI_ISL_15670134 | 14/09/2022 | Africa / Egypt | Human | unknown | unknown | unknown | non hospitalized | unknown | unknown | BA.5.2 | GRA |
| hCoV-19/Egypt/CPHL-EGY22614/2022 | EPI_ISL_15670133 | 02/10/2022 | Africa / Egypt | Human | unknown | unknown | unknown | non hospitalized | unknown | unknown | BA.5.2 | GRA |
| hCoV-19/Egypt/CPHL-EGY22613/2022 | EPI_ISL_15670132 | 14/09/2022 | Africa / Egypt | Human | unknown | unknown | unknown | non hospitalized | unknown | unknown | BA.5.2 | GRA |
| hCoV-19/Egypt/CPHL-EGY22612/2022 | EPI_ISL_15670131 | 29/09/2022 | Africa / Egypt | Human | unknown | unknown | unknown | non hospitalized | unknown | unknown | C.17 | GR |
| hCoV-19/Egypt/CPHL-EGY22611/2022 | EPI_ISL_15670130 | 02/10/2022 | Africa / Egypt | Human | unknown | unknown | unknown | non hospitalized | unknown | unknown | BA.5.2 | GRA |
| hCoV-19/Egypt/CPHL-EGY22609/2022 | EPI_ISL_15670129 | 14/09/2022 | Africa / Egypt | Human | unknown | unknown | unknown | non hospitalized | unknown | unknown | BA.2 | GRA |
| hCoV-19/Egypt/CPHL-EGY22608/2022 | EPI_ISL_15670128 | 02/10/2022 | Africa / Egypt | Human | unknown | unknown | unknown | non hospitalized | unknown | unknown | BA.5.2 | GRA |
| hCoV-19/Egypt/CPHL-EGY22607/2022 | EPI_ISL_15670127 | 15/08/2022 | Africa / Egypt | Human | unknown | unknown | unknown | non hospitalized | unknown | unknown | BA.5.2 | GRA |
| hCoV-19/Egypt/CPHL-EGY22606/2022 | EPI_ISL_15670126 | 08/08/2022 | Africa / Egypt | Human | unknown | unknown | unknown | non hospitalized | unknown | unknown | BA.5.2 | GRA |
| hCoV-19/Egypt/CPHL-EGY22605/2022 | EPI_ISL_15670125 | 08/08/2022 | Africa / Egypt | Human | unknown | unknown | unknown | non hospitalized | unknown | unknown | BA.5.2 | GRA |
| hCoV-19/Egypt/CPHL-EGY22603/2022 | EPI_ISL_15670124 | 08/08/2022 | Africa / Egypt | Human | unknown | unknown | unknown | non hospitalized | unknown | unknown | BA.5.2 | GRA |
| hCoV-19/Egypt/CPHL-EGY22602/2022 | EPI_ISL_15670123 | 22/08/2022 | Africa / Egypt | Human | unknown | unknown | unknown | non hospitalized | unknown | unknown | BA.5.2 | GRA |
| hCoV-19/Egypt/CPHL-EGY22601/2022 | EPI_ISL_15670122 | 08/08/2022 | Africa / Egypt | Human | unknown | unknown | unknown | non hospitalized | unknown | unknown | B.1.1.529 | GR |
| hCoV-19/Egypt/CPHL-EGY22600/2022 | EPI_ISL_15670121 | 08/08/2022 | Africa / Egypt | Human | unknown | unknown | unknown | non hospitalized | unknown | unknown | BA.5.2.18 | GRA |
| hCoV-19/Egypt/CPHL-EGY22599/2022 | EPI_ISL_15670120 | 14/09/2022 | Africa / Egypt | Human | unknown | unknown | unknown | non hospitalized | unknown | unknown | C.17 | GR |
| hCoV-19/Egypt/CPHL-EGY22598/2022 | EPI_ISL_15670119 | 22/08/2022 | Africa / Egypt | Human | unknown | unknown | unknown | non hospitalized | unknown | unknown | BA.5.2 | GRA |
| hCoV-19/Egypt/CPHL-EGY22597/2022 | EPI_ISL_15670118 | 22/08/2022 | Africa / Egypt | Human | unknown | unknown | unknown | non hospitalized | unknown | unknown | BA.5.2 | GRA |
| hCoV-19/Egypt/CPHL-EGY22596/2022 | EPI_ISL_15670117 | 22/08/2022 | Africa / Egypt | Human | unknown | unknown | unknown | non hospitalized | unknown | unknown | BA.1.1 | GRA |
| hCoV-19/Egypt/CPHL-EGY22595/2022 | EPI_ISL_15670116 | 29/08/2022 | Africa / Egypt | Human | unknown | unknown | unknown | non hospitalized | unknown | unknown | BA.1.1 | GRA |
| hCoV-19/Egypt/CPHL-EGY22594/2022 | EPI_ISL_15670115 | 08/08/2022 | Africa / Egypt | Human | unknown | unknown | unknown | non hospitalized | unknown | unknown | BA.5.2.20 | GRA |
| hCoV-19/Egypt/CPHL-EGY22593/2022 | EPI_ISL_15670114 | 15/08/2022 | Africa / Egypt | Human | unknown | unknown | unknown | non hospitalized | unknown | unknown | BA.5.2.1 | GRA |
| hCoV-19/Egypt/CPHL-EGY22592/2022 | EPI_ISL_15670113 | 22/08/2022 | Africa / Egypt | Human | unknown | unknown | unknown | non hospitalized | unknown | unknown | BA.5.2 | GRA |
| hCoV-19/Egypt/CPHL-EGY22591/2022 | EPI_ISL_15670112 | 29/08/2022 | Africa / Egypt | Human | unknown | unknown | unknown | non hospitalized | unknown | unknown | BA.1.1 | GRA |
| hCoV-19/Egypt/CPHL-EGY22590/2022 | EPI_ISL_15670111 | 22/08/2022 | Africa / Egypt | Human | unknown | unknown | unknown | non hospitalized | unknown | unknown | BF.5 | GRA |
| hCoV-19/Egypt/CPHL-EGY22589/2022 | EPI_ISL_15670110 | 06/08/2022 | Africa / Egypt | Human | unknown | unknown | unknown | non hospitalized | unknown | unknown | BA.5.2 | GRA |
| hCoV-19/Egypt/CPHL-EGY22588/2022 | EPI_ISL_15670109 | 29/08/2022 | Africa / Egypt | Human | unknown | unknown | unknown | non hospitalized | unknown | unknown | BA.1.1 | GRA |
| hCoV-19/Egypt/CPHL-EGY22587/2022 | EPI_ISL_15670108 | 22/08/2022 | Africa / Egypt | Human | unknown | unknown | unknown | non hospitalized | unknown | unknown | BA.5.2 | GRA |
| hCoV-19/Egypt/CPHL-EGY22586/2022 | EPI_ISL_15670107 | 04/08/2022 | Africa / Egypt | Human | unknown | unknown | unknown | non hospitalized | unknown | unknown | BA.5.2 | GRA |
| hCoV-19/Egypt/CPHL-EGY22585/2022 | EPI_ISL_15670106 | 04/08/2022 | Africa / Egypt | Human | unknown | unknown | unknown | non hospitalized | unknown | unknown | BA.2 | GRA |
| hCoV-19/Egypt/CPHL-EGY22584/2022 | EPI_ISL_15670105 | 04/08/2022 | Africa / Egypt | Human | unknown | unknown | unknown | non hospitalized | unknown | unknown | B.1.1.529 | GRA |
| hCoV-19/Egypt/CPHL-EGY22583/2022 | EPI_ISL_15670104 | 04/08/2022 | Africa / Egypt | Human | unknown | unknown | unknown | non hospitalized | unknown | unknown | BA.5.2 | GRA |
| hCoV-19/Egypt/CPHL-EGY22582/2022 | EPI_ISL_15670103 | 18/08/2022 | Africa / Egypt | Human | unknown | unknown | unknown | non hospitalized | unknown | unknown | BA.5.3.1 | GRA |
| hCoV-19/Egypt/CPHL-EGY22581/2022 | EPI_ISL_15670102 | 05/09/2022 | Africa / Egypt | Human | unknown | unknown | unknown | non hospitalized | unknown | unknown | BA.5.2 | GRA |
| hCoV-19/Egypt/CPHL-EGY22580/2022 | EPI_ISL_15670101 | 20/10/2022 | Africa / Egypt | Human | unknown | unknown | unknown | non hospitalized | unknown | unknown | BA.4.6.3 | GRA |
| hCoV-19/Egypt/CPHL-EGY22579/2022 | EPI_ISL_15670100 | 20/10/2022 | Africa / Egypt | Human | unknown | unknown | unknown | non hospitalized | unknown | unknown | CK.2.1 | GRA |
| hCoV-19/Egypt/CPHL-EGY22578/2022 | EPI_ISL_15670099 | 29/08/2022 | Africa / Egypt | Human | unknown | unknown | unknown | non hospitalized | unknown | unknown | BA.1.1 | GRA |
| hCoV-19/Egypt/CPHL-EGY22577/2022 | EPI_ISL_15670098 | 29/08/2022 | Africa / Egypt | Human | unknown | unknown | unknown | non hospitalized | unknown | unknown | C.36.3 | GR |
| hCoV-19/Egypt/CPHL-EGY22576/2022 | EPI_ISL_15670097 | 03/10/2022 | Africa / Egypt | Human | unknown | unknown | unknown | non hospitalized | unknown | unknown | BA.5.3.1 | GRA |
| hCoV-19/Egypt/CPHL-EGY22575/2022 | EPI_ISL_15670096 | 03/10/2022 | Africa / Egypt | Human | unknown | unknown | unknown | non hospitalized | unknown | unknown | BA.5.3.1 | GRA |
| hCoV-19/Egypt/CPHL-EGY22574/2022 | EPI_ISL_15670095 | 03/10/2022 | Africa / Egypt | Human | unknown | unknown | unknown | non hospitalized | unknown | unknown | BA.5.2.1 | GRA |
| hCoV-19/Egypt/CPHL-EGY22573/2022 | EPI_ISL_15670094 | 03/10/2022 | Africa / Egypt | Human | unknown | unknown | unknown | non hospitalized | unknown | unknown | BA.5.3.1 | GRA |
| hCoV-19/Egypt/CPHL-EGY22572/2022 | EPI_ISL_15670093 | 02/10/2022 | Africa / Egypt | Human | unknown | unknown | unknown | non hospitalized | unknown | unknown | BA.5.2 | GRA |
| hCoV-19/Egypt/CPHL-EGY22571/2022 | EPI_ISL_15670092 | 02/10/2022 | Africa / Egypt | Human | unknown | unknown | unknown | non hospitalized | unknown | unknown | BA.5.2 | GRA |
| hCoV-19/Egypt/CPHL-EGY22570/2022 | EPI_ISL_15670091 | 02/10/2022 | Africa / Egypt | Human | unknown | unknown | unknown | non hospitalized | unknown | unknown | BA.5.2 | GRA |
| hCoV-19/Egypt/CPHL-EGY22569/2022 | EPI_ISL_15670090 | 02/10/2022 | Africa / Egypt | Human | unknown | unknown | unknown | non hospitalized | unknown | unknown | BA.5.2 | GRA |
| hCoV-19/Egypt/CPHL-EGY22568/2022 | EPI_ISL_15670089 | 02/10/2022 | Africa / Egypt | Human | unknown | unknown | unknown | non hospitalized | unknown | unknown | BA.5.2 | GRA |
| hCoV-19/Egypt/CPHL-EGY22567/2022 | EPI_ISL_15670088 | 02/10/2022 | Africa / Egypt | Human | unknown | unknown | unknown | non hospitalized | unknown | unknown | BA.2 | GRA |
| hCoV-19/Egypt/CPHL-EGY22566/2022 | EPI_ISL_15670087 | 02/10/2022 | Africa / Egypt | Human | unknown | unknown | unknown | non hospitalized | unknown | unknown | C.17 | GR |
| hCoV-19/Egypt/CPHL-EGY22565/2022 | EPI_ISL_15670086 | 02/10/2022 | Africa / Egypt | Human | unknown | unknown | unknown | non hospitalized | unknown | unknown | C.17 | GR |
| hCoV-19/Egypt/CPHL-EGY22564/2022 | EPI_ISL_15670085 | 02/10/2022 | Africa / Egypt | Human | unknown | unknown | unknown | non hospitalized | unknown | unknown | C.36.3 | GR |
| hCoV-19/Egypt/CPHL-EGY22563/2022 | EPI_ISL_15670084 | 02/10/2022 | Africa / Egypt | Human | unknown | unknown | unknown | non hospitalized | unknown | unknown | BA.5.2.20 | GRA |
| hCoV-19/Egypt/CPHL-EGY22562/2022 | EPI_ISL_15670083 | 02/10/2022 | Africa / Egypt | Human | unknown | unknown | unknown | non hospitalized | unknown | unknown | BA.5.2 | GRA |
| hCoV-19/Egypt/CPHL-EGY22560/2022 | EPI_ISL_15670082 | 02/10/2022 | Africa / Egypt | Human | unknown | unknown | unknown | non hospitalized | unknown | unknown | BA.5.2 | GRA |
| hCoV-19/Egypt/CPHL-EGY22558/2022 | EPI_ISL_15670081 | 02/10/2022 | Africa / Egypt | Human | unknown | unknown | unknown | non hospitalized | unknown | unknown | BA.5.1 | GRA |
| hCoV-19/Egypt/CPHL-EGY22557/2022 | EPI_ISL_15670080 | 02/10/2022 | Africa / Egypt | Human | unknown | unknown | unknown | non hospitalized | unknown | unknown | BA.5.2 | GRA |
| hCoV-19/Egypt/CPHL-EGY22556/2022 | EPI_ISL_15670079 | 02/10/2022 | Africa / Egypt | Human | unknown | unknown | unknown | non hospitalized | unknown | unknown | BA.5.2 | GRA |
| hCoV-19/Egypt/CPHL-EGY22555/2022 | EPI_ISL_15670078 | 02/10/2022 | Africa / Egypt | Human | unknown | unknown | unknown | non hospitalized | unknown | unknown | BA.5.2 | GRA |
| hCoV-19/Egypt/CPHL-EGY22553/2022 | EPI_ISL_15670077 | 02/10/2022 | Africa / Egypt | Human | unknown | unknown | unknown | non hospitalized | unknown | unknown | BA.5.2 | GRA |
| hCoV-19/Egypt/CPHL-EGY22552/2022 | EPI_ISL_15670076 | 02/10/2022 | Africa / Egypt | Human | unknown | unknown | unknown | non hospitalized | unknown | unknown | BA.5.2 | GRA |
| hCoV-19/Egypt/CPHL-EGY22551/2022 | EPI_ISL_15670075 | 02/10/2022 | Africa / Egypt | Human | unknown | unknown | unknown | non hospitalized | unknown | unknown | BA.5.2 | GRA |
| hCoV-19/Egypt/CPHL-EGY22550/2022 | EPI_ISL_15270743 | 28/06/2022 | Africa / Egypt | Human | unknown | unknown | unknown | non hospitalized | unknown | unknown | B.1.1.529 | GR |
| hCoV-19/Egypt/CPHL-EGY22548/2022 | EPI_ISL_15270742 | 28/06/2022 | Africa / Egypt | Human | unknown | unknown | unknown | non hospitalized | unknown | unknown | B.1.1.529 | GRA |
| hCoV-19/Egypt/CPHL-EGY22547/2022 | EPI_ISL_15270741 | 28/06/2022 | Africa / Egypt | Human | unknown | unknown | unknown | non hospitalized | unknown | unknown | BA.2 | GRA |
| hCoV-19/Egypt/CPHL-EGY22545/2022 | EPI_ISL_15270740 | 28/06/2022 | Africa / Egypt | Human | unknown | unknown | unknown | non hospitalized | unknown | unknown | BA.2 | GRA |
| hCoV-19/Egypt/CPHL-EGY22544/2022 | EPI_ISL_15270739 | 28/06/2022 | Africa / Egypt | Human | unknown | unknown | unknown | non hospitalized | unknown | unknown | BA.5.2 | GRA |
| hCoV-19/Egypt/CPHL-EGY22543/2022 | EPI_ISL_15270738 | 28/06/2022 | Africa / Egypt | Human | unknown | unknown | unknown | non hospitalized | unknown | unknown | BA.2.10.3 | GRA |
| hCoV-19/Egypt/CPHL-EGY22542/2022 | EPI_ISL_15270737 | 28/06/2022 | Africa / Egypt | Human | unknown | unknown | unknown | non hospitalized | unknown | unknown | BA.5 | GRA |
| hCoV-19/Egypt/CPHL-EGY22541/2022 | EPI_ISL_15270736 | 28/06/2022 | Africa / Egypt | Human | unknown | unknown | unknown | non hospitalized | unknown | unknown | BA.5.2 | GRA |
| hCoV-19/Egypt/CPHL-EGY22540/2022 | EPI_ISL_15270735 | 28/06/2022 | Africa / Egypt | Human | unknown | unknown | unknown | non hospitalized | unknown | unknown | BA.5 | GRA |
| hCoV-19/Egypt/CPHL-EGY22539/2022 | EPI_ISL_15270734 | 27/06/2022 | Africa / Egypt | Human | unknown | unknown | unknown | non hospitalized | unknown | unknown | BA.5.2 | GRA |
| hCoV-19/Egypt/CPHL-EGY22538/2022 | EPI_ISL_15270733 | 27/06/2022 | Africa / Egypt | Human | unknown | unknown | unknown | non hospitalized | unknown | unknown | BF.28 | GRA |
| hCoV-19/Egypt/CPHL-EGY22537/2022 | EPI_ISL_15270732 | 28/06/2022 | Africa / Egypt | Human | unknown | unknown | unknown | non hospitalized | unknown | unknown | BA.2 | GRA |
| hCoV-19/Egypt/CPHL-EGY22535/2022 | EPI_ISL_15270731 | 28/06/2022 | Africa / Egypt | Human | unknown | unknown | unknown | non hospitalized | unknown | unknown | BA.2 | GRA |
| hCoV-19/Egypt/CPHL-EGY22534/2022 | EPI_ISL_15270730 | 28/06/2022 | Africa / Egypt | Human | unknown | unknown | unknown | non hospitalized | unknown | unknown | BA.5.2 | GRA |
| hCoV-19/Egypt/CPHL-EGY22533/2022 | EPI_ISL_15270729 | 28/06/2022 | Africa / Egypt | Human | unknown | unknown | unknown | non hospitalized | unknown | unknown | BA.5.2 | GRA |
| hCoV-19/Egypt/CPHL-EGY22532/2022 | EPI_ISL_15270728 | 28/06/2022 | Africa / Egypt | Human | unknown | unknown | unknown | non hospitalized | unknown | unknown | BA.5.2 | GRA |
| hCoV-19/Egypt/CPHL-EGY22531/2022 | EPI_ISL_15270727 | 27/06/2022 | Africa / Egypt | Human | unknown | unknown | unknown | non hospitalized | unknown | unknown | BA.4.1 | GRA |
| hCoV-19/Egypt/CPHL-EGY22530/2022 | EPI_ISL_15270726 | 27/06/2022 | Africa / Egypt | Human | unknown | unknown | unknown | non hospitalized | unknown | unknown | BA.2.56 | GRA |
| hCoV-19/Egypt/CPHL-EGY22529/2022 | EPI_ISL_15270725 | 28/06/2022 | Africa / Egypt | Human | unknown | unknown | unknown | non hospitalized | unknown | unknown | BA.5.2 | GR |
| hCoV-19/Egypt/CPHL-EGY22528/2022 | EPI_ISL_15270724 | 28/06/2022 | Africa / Egypt | Human | unknown | unknown | unknown | non hospitalized | unknown | unknown | BA.5.2 | GRA |
| hCoV-19/Egypt/CPHL-EGY22527/2022 | EPI_ISL_15270723 | 28/06/2022 | Africa / Egypt | Human | unknown | unknown | unknown | non hospitalized | unknown | unknown | BA.5.2 | GRA |
| hCoV-19/Egypt/CPHL-EGY22526/2022 | EPI_ISL_15270722 | 28/06/2022 | Africa / Egypt | Human | unknown | unknown | unknown | non hospitalized | unknown | unknown | B.1.1.529 | GR |
| hCoV-19/Egypt/CPHL-EGY22525/2022 | EPI_ISL_15270721 | 28/06/2022 | Africa / Egypt | Human | unknown | unknown | unknown | non hospitalized | unknown | unknown | BA.5.2 | GRA |
| hCoV-19/Egypt/CPHL-EGY22524/2022 | EPI_ISL_15270720 | 27/06/2022 | Africa / Egypt | Human | unknown | unknown | unknown | non hospitalized | unknown | unknown | BE.4 | GRA |
| hCoV-19/Egypt/CPHL-EGY22523/2022 | EPI_ISL_15270719 | 27/06/2022 | Africa / Egypt | Human | unknown | unknown | unknown | non hospitalized | unknown | unknown | BA.4.1 | GRA |
| hCoV-19/Egypt/CPHL-EGY22522/2022 | EPI_ISL_15270718 | 28/06/2022 | Africa / Egypt | Human | unknown | unknown | unknown | non hospitalized | unknown | unknown | BA.5.2.1 | GRA |
| hCoV-19/Egypt/CPHL-EGY22521/2022 | EPI_ISL_15270717 | 28/06/2022 | Africa / Egypt | Human | unknown | unknown | unknown | non hospitalized | unknown | unknown | BA.5.2 | GRA |
| hCoV-19/Egypt/CPHL-EGY22520/2022 | EPI_ISL_15270716 | 28/06/2022 | Africa / Egypt | Human | unknown | unknown | unknown | non hospitalized | unknown | unknown | BA.5.2 | GRA |
| hCoV-19/Egypt/CPHL-EGY22519/2022 | EPI_ISL_15270715 | 28/06/2022 | Africa / Egypt | Human | unknown | unknown | unknown | non hospitalized | unknown | unknown | BA.5.2 | GRA |
| hCoV-19/Egypt/CPHL-EGY22518/2022 | EPI_ISL_15270714 | 28/06/2022 | Africa / Egypt | Human | unknown | unknown | unknown | non hospitalized | unknown | unknown | BA.2.56 | GRA |
| hCoV-19/Egypt/CPHL-EGY22517/2022 | EPI_ISL_15270713 | 27/06/2022 | Africa / Egypt | Human | unknown | unknown | unknown | non hospitalized | unknown | unknown | BF.28 | GRA |
| hCoV-19/Egypt/CPHL-EGY22516/2022 | EPI_ISL_15270712 | 27/06/2022 | Africa / Egypt | Human | unknown | unknown | unknown | non hospitalized | unknown | unknown | BA.5.2 | GRA |
| hCoV-19/Egypt/CPHL-EGY22514/2022 | EPI_ISL_15270711 | 27/06/2022 | Africa / Egypt | Human | unknown | unknown | unknown | non hospitalized | unknown | unknown | BA.5.3 | GRA |
| hCoV-19/Egypt/CPHL-EGY22513/2022 | EPI_ISL_15270710 | 27/06/2022 | Africa / Egypt | Human | unknown | unknown | unknown | non hospitalized | unknown | unknown | BA.5.2 | GRA |
| hCoV-19/Egypt/CPHL-EGY22511/2022 | EPI_ISL_15270709 | 27/06/2022 | Africa / Egypt | Human | unknown | unknown | unknown | non hospitalized | unknown | unknown | BA.5.2 | GRA |
| hCoV-19/Egypt/CPHL-EGY22510/2022 | EPI_ISL_15270708 | 27/06/2022 | Africa / Egypt | Human | unknown | unknown | unknown | non hospitalized | unknown | unknown | BA.5 | GRA |
| hCoV-19/Egypt/CPHL-EGY22509/2022 | EPI_ISL_15270707 | 27/06/2022 | Africa / Egypt | Human | unknown | unknown | unknown | non hospitalized | unknown | unknown | BA.5.2 | GRA |
| hCoV-19/Egypt/CPHL-EGY22508/2022 | EPI_ISL_15270706 | 27/06/2022 | Africa / Egypt | Human | unknown | unknown | unknown | non hospitalized | unknown | unknown | BA.5.2.20 | GRA |
| hCoV-19/Egypt/CPHL-EGY22507/2022 | EPI_ISL_15270705 | 27/06/2022 | Africa / Egypt | Human | unknown | unknown | unknown | non hospitalized | unknown | unknown | BA.5 | GRA |
| hCoV-19/Egypt/CPHL-EGY22506/2022 | EPI_ISL_15270704 | 04/07/2022 | Africa / Egypt | Human | unknown | unknown | unknown | non hospitalized | unknown | unknown | BA.5 | GRA |
| hCoV-19/Egypt/CPHL-EGY22505/2022 | EPI_ISL_15270703 | 04/07/2022 | Africa / Egypt | Human | unknown | unknown | unknown | non hospitalized | unknown | unknown | BA.5.2 | GRA |
| hCoV-19/Egypt/CPHL-EGY22504/2022 | EPI_ISL_15270702 | 04/07/2022 | Africa / Egypt | Human | unknown | unknown | unknown | non hospitalized | unknown | unknown | BA.5.2 | GRA |
| hCoV-19/Egypt/CPHL-EGY22503/2022 | EPI_ISL_15270701 | 04/07/2022 | Africa / Egypt | Human | unknown | unknown | unknown | non hospitalized | unknown | unknown | BA.5.2.1 | GRA |
| hCoV-19/Egypt/CPHL-EGY22502/2022 | EPI_ISL_15270700 | 04/07/2022 | Africa / Egypt | Human | unknown | unknown | unknown | non hospitalized | unknown | unknown | BA.5.2 | GRA |
| hCoV-19/Egypt/CPHL-EGY22501/2022 | EPI_ISL_15270699 | 04/07/2022 | Africa / Egypt | Human | unknown | unknown | unknown | non hospitalized | unknown | unknown | BA.5.3.1 | GRA |
| hCoV-19/Egypt/CPHL-EGY22500/2022 | EPI_ISL_15270698 | 04/07/2022 | Africa / Egypt | Human | unknown | unknown | unknown | non hospitalized | unknown | unknown | BA.5.2 | GRA |
| hCoV-19/Egypt/CPHL-EGY22499/2022 | EPI_ISL_15270697 | 27/06/2022 | Africa / Egypt | Human | unknown | unknown | unknown | non hospitalized | unknown | unknown | BF.28 | GRA |
| hCoV-19/Egypt/CPHL-EGY22498/2022 | EPI_ISL_15270696 | 29/06/2022 | Africa / Egypt | Human | unknown | unknown | unknown | non hospitalized | unknown | unknown | BA.5.2 | GRA |
| hCoV-19/Egypt/CPHL-EGY22497/2022 | EPI_ISL_15270695 | 04/07/2022 | Africa / Egypt | Human | unknown | unknown | unknown | non hospitalized | unknown | unknown | BA.5.2.20 | GRA |
| hCoV-19/Egypt/CPHL-EGY22496/2022 | EPI_ISL_15270694 | 04/07/2022 | Africa / Egypt | Human | unknown | unknown | unknown | non hospitalized | unknown | unknown | BA.2.12.1 | GRA |
| hCoV-19/Egypt/CPHL-EGY22495/2022 | EPI_ISL_15270693 | 04/07/2022 | Africa / Egypt | Human | unknown | unknown | unknown | non hospitalized | unknown | unknown | BA.5.2 | GRA |
| hCoV-19/Egypt/CPHL-EGY22494/2022 | EPI_ISL_15270692 | 04/07/2022 | Africa / Egypt | Human | unknown | unknown | unknown | non hospitalized | unknown | unknown | BA.5.2 | GRA |
| hCoV-19/Egypt/CPHL-EGY22493/2022 | EPI_ISL_15270691 | 04/07/2022 | Africa / Egypt | Human | unknown | unknown | unknown | non hospitalized | unknown | unknown | B.1.1.529 | GR |
| hCoV-19/Egypt/CPHL-EGY22492/2022 | EPI_ISL_15270690 | 04/07/2022 | Africa / Egypt | Human | unknown | unknown | unknown | non hospitalized | unknown | unknown | BA.5.2.1 | GRA |
| hCoV-19/Egypt/CPHL-EGY22491/2022 | EPI_ISL_15270689 | 04/07/2022 | Africa / Egypt | Human | unknown | unknown | unknown | non hospitalized | unknown | unknown | BA.5.2.1 | GRA |
| hCoV-19/Egypt/CPHL-EGY22490/2022 | EPI_ISL_15270688 | 04/07/2022 | Africa / Egypt | Human | unknown | unknown | unknown | non hospitalized | unknown | unknown | BA.5.2 | GRA |
| hCoV-19/Egypt/CPHL-EGY22489/2022 | EPI_ISL_15270687 | 04/07/2022 | Africa / Egypt | Human | unknown | unknown | unknown | non hospitalized | unknown | unknown | BA.5.2 | GRA |
| hCoV-19/Egypt/CPHL-EGY22487/2022 | EPI_ISL_15270686 | 04/07/2022 | Africa / Egypt | Human | unknown | unknown | unknown | non hospitalized | unknown | unknown | BF.5 | GRA |
| hCoV-19/Egypt/CPHL-EGY22486/2022 | EPI_ISL_15270685 | 04/07/2022 | Africa / Egypt | Human | unknown | unknown | unknown | non hospitalized | unknown | unknown | BA.5.2 | GRA |
| hCoV-19/Egypt/CPHL-EGY22485/2022 | EPI_ISL_15270684 | 04/07/2022 | Africa / Egypt | Human | unknown | unknown | unknown | non hospitalized | unknown | unknown | BA.5.2 | GRA |
| hCoV-19/Egypt/CPHL-EGY22484/2022 | EPI_ISL_15270683 | 04/07/2022 | Africa / Egypt | Human | unknown | unknown | unknown | non hospitalized | unknown | unknown | BA.5.3 | GRA |
| hCoV-19/Egypt/CPHL-EGY22482/2022 | EPI_ISL_15270682 | 04/07/2022 | Africa / Egypt | Human | unknown | unknown | unknown | non hospitalized | unknown | unknown | BA.5.3 | GRA |
| hCoV-19/Egypt/CPHL-EGY22481/2022 | EPI_ISL_15270681 | 04/07/2022 | Africa / Egypt | Human | unknown | unknown | unknown | non hospitalized | unknown | unknown | BF.5 | GRA |
| hCoV-19/Egypt/CPHL-EGY22480/2022 | EPI_ISL_15270680 | 04/07/2022 | Africa / Egypt | Human | unknown | unknown | unknown | non hospitalized | unknown | unknown | BA.5.2 | GRA |
| hCoV-19/Egypt/CPHL-EGY22479/2022 | EPI_ISL_15270679 | 04/07/2022 | Africa / Egypt | Human | unknown | unknown | unknown | non hospitalized | unknown | unknown | BA.2.56 | GRA |
| hCoV-19/Egypt/CPHL-EGY22478/2022 | EPI_ISL_15270678 | 03/07/2022 | Africa / Egypt | Human | unknown | unknown | unknown | non hospitalized | unknown | unknown | BA.5.2.20 | GRA |
| hCoV-19/Egypt/CPHL-EGY22476/2022 | EPI_ISL_15270677 | 04/07/2022 | Africa / Egypt | Human | unknown | unknown | unknown | non hospitalized | unknown | unknown | BA.5 | GRA |
| hCoV-19/Egypt/CPHL-EGY22475/2022 | EPI_ISL_15270676 | 03/07/2022 | Africa / Egypt | Human | unknown | unknown | unknown | non hospitalized | unknown | unknown | BA.5.2.18 | GRA |
| hCoV-19/Egypt/CPHL-EGY22474/2022 | EPI_ISL_15270675 | 04/07/2022 | Africa / Egypt | Human | unknown | unknown | unknown | non hospitalized | unknown | unknown | BA.5.2 | GRA |
| hCoV-19/Egypt/CPHL-EGY22473/2022 | EPI_ISL_15270674 | 04/07/2022 | Africa / Egypt | Human | unknown | unknown | unknown | non hospitalized | unknown | unknown | BA.5.2.20 | GR |
| hCoV-19/Egypt/CPHL-EGY22472/2022 | EPI_ISL_15270673 | 28/06/2022 | Africa / Egypt | Human | unknown | unknown | unknown | non hospitalized | unknown | unknown | BA.2 | GRA |
| hCoV-19/Egypt/CPHL-EGY22470/2022 | EPI_ISL_15270672 | 28/06/2022 | Africa / Egypt | Human | unknown | unknown | unknown | non hospitalized | unknown | unknown | BA.5.2.20 | GR |
| hCoV-19/Egypt/CPHL-EGY22469/2022 | EPI_ISL_15270671 | 28/06/2022 | Africa / Egypt | Human | unknown | unknown | unknown | non hospitalized | unknown | unknown | BA.5.2.20 | GRA |
| hCoV-19/Egypt/CPHL-EGY22468/2022 | EPI_ISL_15270670 | 27/06/2022 | Africa / Egypt | Human | unknown | unknown | unknown | non hospitalized | unknown | unknown | BA.5.2 | GRA |
| hCoV-19/Egypt/CPHL-EGY22467/2022 | EPI_ISL_15270669 | 27/06/2022 | Africa / Egypt | Human | unknown | unknown | unknown | non hospitalized | unknown | unknown | BA.2 | GRA |
| hCoV-19/Egypt/CPHL-EGY22466/2022 | EPI_ISL_15270668 | 28/06/2022 | Africa / Egypt | Human | unknown | unknown | unknown | non hospitalized | unknown | unknown | BA.5.2 | GRA |
| hCoV-19/Egypt/CPHL-EGY22464/2022 | EPI_ISL_15270667 | 28/06/2022 | Africa / Egypt | Human | unknown | unknown | unknown | non hospitalized | unknown | unknown | BA.5 | GRA |
| hCoV-19/Egypt/CPHL-EGY22463/2022 | EPI_ISL_15270666 | 28/06/2022 | Africa / Egypt | Human | unknown | unknown | unknown | non hospitalized | unknown | unknown | BA.5.2 | GRA |
| hCoV-19/Egypt/CPHL-EGY22461/2022 | EPI_ISL_15270665 | 28/06/2022 | Africa / Egypt | Human | unknown | unknown | unknown | non hospitalized | unknown | unknown | BA.5.2 | GRA |
| hCoV-19/Egypt/CPHL-EGY22460/2022 | EPI_ISL_15270664 | 27/06/2022 | Africa / Egypt | Human | unknown | unknown | unknown | non hospitalized | unknown | unknown | BA.5.2.20 | GRA |
| hCoV-19/Egypt/CPHL-EGY22459/2022 | EPI_ISL_15270663 | 27/06/2022 | Africa / Egypt | Human | unknown | unknown | unknown | non hospitalized | unknown | unknown | BA.5.2 | GRA |
| hCoV-19/Egypt/CPHL-EGY22458/2022 | EPI_ISL_15270662 | 27/06/2022 | Africa / Egypt | Human | unknown | unknown | unknown | non hospitalized | unknown | unknown | BF.5 | GRA |
| hCoV-19/Egypt/CPHL-EGY22457/2022 | EPI_ISL_15270661 | 27/06/2022 | Africa / Egypt | Human | unknown | unknown | unknown | non hospitalized | unknown | unknown | BA.5.2.1 | GRA |
| hCoV-19/Egypt/CPHL-EGY22456/2022 | EPI_ISL_15270660 | 29/06/2022 | Africa / Egypt | Human | unknown | unknown | unknown | non hospitalized | unknown | unknown | BA.5.2 | GRA |
| hCoV-19/Egypt/CPHL-EGY22455/2022 | EPI_ISL_15270659 | 29/06/2022 | Africa / Egypt | Human | unknown | unknown | unknown | non hospitalized | unknown | unknown | BA.5.3.1 | GRA |
| hCoV-19/Egypt/CPHL-EGY22454/2022 | EPI_ISL_15270658 | 29/06/2022 | Africa / Egypt | Human | unknown | unknown | unknown | non hospitalized | unknown | unknown | BA.2 | GRA |
| hCoV-19/Egypt/CPHL-EGY22453/2022 | EPI_ISL_15270657 | 29/06/2022 | Africa / Egypt | Human | unknown | unknown | unknown | non hospitalized | unknown | unknown | BA.5.2.1 | GRA |
| hCoV-19/Egypt/CPHL-EGY22452/2022 | EPI_ISL_15270656 | 29/06/2022 | Africa / Egypt | Human | unknown | unknown | unknown | non hospitalized | unknown | unknown | BA.5.2.1 | GRA |
| hCoV-19/Egypt/CPHL-EGY22451/2022 | EPI_ISL_15270655 | 29/06/2022 | Africa / Egypt | Human | unknown | unknown | unknown | non hospitalized | unknown | unknown | BA.5.2.1 | GRA |
| hCoV-19/Egypt/CPHL-EGY22450/2022 | EPI_ISL_15270654 | 14/06/2022 | Africa / Egypt | Human | unknown | unknown | unknown | non hospitalized | unknown | unknown | BA.5.2 | GRA |
| hCoV-19/Egypt/CPHL-EGY22449/2022 | EPI_ISL_15270653 | 19/06/2022 | Africa / Egypt | Human | unknown | unknown | unknown | non hospitalized | unknown | unknown | B.1.1.529 | GR |
| hCoV-19/Egypt/CPHL-EGY22448/2022 | EPI_ISL_15270652 | 06/06/2022 | Africa / Egypt | Human | unknown | unknown | unknown | non hospitalized | unknown | unknown | BA.2.38 | GRA |
| hCoV-19/Egypt/CPHL-EGY22447/2022 | EPI_ISL_15270651 | 06/06/2022 | Africa / Egypt | Human | unknown | unknown | unknown | non hospitalized | unknown | unknown | BA.5.2.1 | GRA |
| hCoV-19/Egypt/CPHL-EGY22446/2022 | EPI_ISL_15270650 | 06/06/2022 | Africa / Egypt | Human | unknown | unknown | unknown | non hospitalized | unknown | unknown | B.1.1.529 | GR |
| hCoV-19/Egypt/CPHL-EGY22445/2022 | EPI_ISL_15270649 | 06/06/2022 | Africa / Egypt | Human | unknown | unknown | unknown | non hospitalized | unknown | unknown | BA.5.2 | GRA |
| hCoV-19/Egypt/CPHL-EGY22444/2022 | EPI_ISL_15270648 | 06/06/2022 | Africa / Egypt | Human | unknown | unknown | unknown | non hospitalized | unknown | unknown | BA.5.2 | GRA |
| hCoV-19/Egypt/CPHL-EGY22443/2022 | EPI_ISL_15270647 | 02/06/2022 | Africa / Egypt | Human | unknown | unknown | unknown | non hospitalized | unknown | unknown | BA.5.2 | GRA |
[truncated: 479,695 more chars]
