# Supplementary material for: Genomic surveillance of SARS-CoV-2 in North Africa: 4 years of GISAID data sharing
Source: IJID Reg. 2024 Mar 19;11:100356. doi: 10.1016/j.ijregi.2024.100356 (PMC11035039; doi:10.1016/j.ijregi.2024.100356)
Supplement: Supplementary file 3 [file mmc3.docx]

Table S2. Comprehensive Virus Data from Libya Including Virus Name, Accession Number, and Clinical Attributes (based on data downloaded from GISAID per 15 September 2023)

| Virus name | Accession ID | Collection date | Location | Host | Sampling strategy | Gender | Patient age (years) | Patient status | Last vaccinated | Sampling strategy | Lineage | Clade |
| --- | --- | --- | --- | --- | --- | --- | --- | --- | --- | --- | --- | --- |
| hCoV-19/Libya/79742-96/2022 | EPI_ISL_13505286 | 23/03/2022 | Africa / Libya / Tripoli | Human | Sentinel surveillance (SARI) | Female | 14 | Released | unknown | Sentinel surveillance (SARI) | BA.2.3 | GRA |
| hCoV-19/Libya/81133-89/2022 | EPI_ISL_13505287 | 22/04/2022 | Africa / Libya / Tripoli | Human | Sentinel surveillance (SARI) | Male | 18 | Hospitalized | unknown | Sentinel surveillance (SARI) | BA.2.3 | GRA |
| hCoV-19/Libya/80903-91/2022 | EPI_ISL_13505288 | 02/04/2022 | Africa / Libya / Tripoli | Human | Sentinel surveillance (SARI) | Male | 59 | Live | unknown | Sentinel surveillance (SARI) | BA.2.3 | GRA |
| hCoV-19/Libya/80894-93/2022 | EPI_ISL_13505289 | 02/04/2022 | Africa / Libya / Tripoli | Human | Sentinel surveillance (SARI) | Male | 21 | Live | unknown | Sentinel surveillance (SARI) | BA.2.3 | GRA |
| hCoV-19/Libya/59772-19/2022 | EPI_ISL_14014989 | 16/02/2022 | Africa / Libya / Tripoli | Human | Sentinel surveillance (SARI) | Female | 38 | Live | unknown | Sentinel surveillance (SARI) | AY.4 | GK |
| hCoV-19/Libya/85811-25/2022 | EPI_ISL_14014990 | 30/05/2022 | Africa / Libya / Tripoli | Human | Sentinel surveillance (SARI) | Female | 80 | Live | unknown | Sentinel surveillance (SARI) | BA.2.12.1 | GRA |
| hCoV-19/Libya/86043-26/2022 | EPI_ISL_14014991 | 01/06/2022 | Africa / Libya / Tripoli | Human | Sentinel surveillance (SARI) | Male | 39 | Live | unknown | Sentinel surveillance (SARI) | BA.2.12.1 | GRA |
| hCoV-19/Libya/86174-27/2022 | EPI_ISL_14014992 | 02/06/2022 | Africa / Libya / Tripoli | Human | Sentinel surveillance (SARI) | Male | 32 | Hospitalized | unknown | Sentinel surveillance (SARI) | BA.2.3 | GRA |
| hCoV-19/Libya/86368-28/2022 | EPI_ISL_14014993 | 05/06/2022 | Africa / Libya / Tripoli | Human | Sentinel surveillance (SARI) | Female | 52 | Live | unknown | Sentinel surveillance (SARI) | BA.2 | GRA |
| hCoV-19/Libya/86436-29/2022 | EPI_ISL_14014994 | 06/06/2022 | Africa / Libya / Tripoli | Human | Sentinel surveillance (SARI) | Male | 42 | Live | unknown | Sentinel surveillance (SARI) | BA.2 | GRA |
| hCoV-19/Libya/86412a-34/2021 | EPI_ISL_14014995 | 29/12/2021 | Africa / Libya / Tripoli | Human | Sentinel surveillance (SARI) | Male | 53 | Live | unknown | Sentinel surveillance (SARI) | AY.127 | GK |
| hCoV-19/Libya/86442a-35/2021 | EPI_ISL_14014996 | 29/12/2021 | Africa / Libya / Tripoli | Human | Sentinel surveillance (SARI) | Female | 43 | Live | unknown | Sentinel surveillance (SARI) | AY.127 | GK |
| hCoV-19/Libya/86574a-37/2021 | EPI_ISL_14014997 | 30/12/2021 | Africa / Libya / Tripoli | Human | Sentinel surveillance (SARI) | Male | 20 | Live | unknown | Sentinel surveillance (SARI) | AY.127 | GK |
| hCoV-19/Libya/84331-7/2022 | EPI_ISL_14327797 | 14/05/2022 | Africa / Libya / Tripoli | Human | Sentinel surveillance (SARI) | Male | 63 | Live | unknown | Sentinel surveillance (SARI) | BA.2.3 | GRA |
| hCoV-19/Libya/84332-8/2022 | EPI_ISL_14327798 | 14/05/2022 | Africa / Libya / Tripoli | Human | Sentinel surveillance (SARI) | Female | 63 | Live | unknown | Sentinel surveillance (SARI) | BA.2 | GRA |
| hCoV-19/Libya/85148-12/2022 | EPI_ISL_14327799 | 23/05/2022 | Africa / Libya / Tripoli | Human | Sentinel surveillance (SARI) | Male | 40 | Live | unknown | Sentinel surveillance (SARI) | BA.2.5 | GRA |
| hCoV-19/Libya/85189-14/2022 | EPI_ISL_14327800 | 23/05/2022 | Africa / Libya / Tripoli | Human | Sentinel surveillance (SARI) | Male | 62 | Live | unknown | Sentinel surveillance (SARI) | BA.2 | GRA |
| hCoV-19/Libya/85006-15/2022 | EPI_ISL_14327801 | 22/05/2022 | Africa / Libya / Tripoli | Human | Sentinel surveillance (SARI) | Male | 65 | Live | unknown | Sentinel surveillance (SARI) | BA.2 | GRA |
| hCoV-19/Libya/85065-16/2022 | EPI_ISL_14327802 | 22/05/2022 | Africa / Libya / Tripoli | Human | Sentinel surveillance (SARI) | Male | 52 | Live | unknown | Sentinel surveillance (SARI) | BA.5.2 | GRA |
| hCoV-19/Libya/88160-41/2022 | EPI_ISL_14358718 | 25/06/2022 | Africa / Libya / Tripoli | Human | Sentinel surveillance (SARI) | Female | 33 | Live | unknown | Sentinel surveillance (SARI) | BA.5.2.1 | GRA |
| hCoV-19/Libya/88159-42/2022 | EPI_ISL_14358719 | 25/06/2022 | Africa / Libya / Tripoli | Human | Sentinel surveillance (SARI) | Female | 31 | Live | unknown | Sentinel surveillance (SARI) | BA.5 | GRA |
| hCoV-19/Libya/89391-43/2022 | EPI_ISL_14358720 | 04/07/2022 | Africa / Libya / Tripoli | Human | Sentinel surveillance (SARI) | Male | 50 | Live | unknown | Sentinel surveillance (SARI) | BA.5.2 | GRA |
| hCoV-19/Libya/89643-44/2022 | EPI_ISL_14358721 | 06/07/2022 | Africa / Libya / Tripoli | Human | Sentinel surveillance (SARI) | Female | 39 | Live | unknown | Sentinel surveillance (SARI) | BA.5.2 | GRA |
| hCoV-19/Libya/89713-45/2022 | EPI_ISL_14358722 | 06/07/2022 | Africa / Libya / Tripoli | Human | Sentinel surveillance (SARI) | Male | 31 | Live | unknown | Sentinel surveillance (SARI) | BA.5.2 | GRA |
| hCoV-19/Libya/89811-46/2022 | EPI_ISL_14358723 | 11/07/2022 | Africa / Libya / Tripoli | Human | Sentinel surveillance (SARI) | Female | 60 | Live | unknown | Sentinel surveillance (SARI) | BA.4.1 | GRA |
| hCoV-19/Libya/89812-47/2022 | EPI_ISL_14358724 | 11/07/2022 | Africa / Libya / Tripoli | Human | Sentinel surveillance (SARI) | Male | 27 | Live | unknown | Sentinel surveillance (SARI) | BA.5.2.20 | GRA |
| hCoV-19/Libya/89833-48/2022 | EPI_ISL_14358725 | 11/07/2022 | Africa / Libya / Tripoli | Human | Sentinel surveillance (SARI) | Male | 69 | Live | unknown | Sentinel surveillance (SARI) | BA.5.2 | GRA |
| hCoV-19/Libya/89834-49/2022 | EPI_ISL_14358726 | 11/07/2022 | Africa / Libya / Tripoli | Human | Sentinel surveillance (SARI) | Female | 35 | Live | unknown | Sentinel surveillance (SARI) | BA.5.2 | GRA |
| hCoV-19/Libya/89836-50/2022 | EPI_ISL_14358727 | 11/07/2022 | Africa / Libya / Tripoli | Human | Sentinel surveillance (SARI) | Female | 43 | Live | unknown | Sentinel surveillance (SARI) | BA.5.2 | GRA |
| hCoV-19/Libya/89850-51/2022 | EPI_ISL_14358728 | 11/07/2022 | Africa / Libya / Tripoli | Human | Sentinel surveillance (SARI) | Female | 32 | Live | unknown | Sentinel surveillance (SARI) | BA.5.2 | GRA |
| hCoV-19/Libya/89851-52/2022 | EPI_ISL_14358729 | 11/07/2022 | Africa / Libya / Tripoli | Human | Sentinel surveillance (SARI) | Male | 38 | Live | unknown | Sentinel surveillance (SARI) | BA.5.2 | GRA |
| hCoV-19/Libya/89852-53/2022 | EPI_ISL_14358730 | 11/07/2022 | Africa / Libya / Tripoli | Human | Sentinel surveillance (SARI) | Male | 55 | Live | unknown | Sentinel surveillance (SARI) | BA.5.2 | GRA |
| hCoV-19/Libya/89867-54/2022 | EPI_ISL_14358731 | 12/07/2022 | Africa / Libya / Tripoli | Human | Sentinel surveillance (SARI) | Female | 49 | Live | unknown | Sentinel surveillance (SARI) | BA.5.2 | GRA |
| hCoV-19/Libya/89879-55/2022 | EPI_ISL_14358732 | 12/07/2022 | Africa / Libya / Tripoli | Human | Sentinel surveillance (SARI) | Male | 56 | Live | unknown | Sentinel surveillance (SARI) | BA.5.2 | GRA |
| hCoV-19/Libya/89880-56/2022 | EPI_ISL_14358733 | 12/07/2022 | Africa / Libya / Tripoli | Human | Sentinel surveillance (SARI) | Male | 56 | Live | unknown | Sentinel surveillance (SARI) | BA.5.2.20 | GRA |
| hCoV-19/Libya/BTRC-WGS-32/2021 | EPI_ISL_14569344 | 25/02/2021 | Africa / Libya | Human | unknown | unknown | unknown | unknown | unknown | unknown | B.1.525 | G |
| hCoV-19/Libya/BTRC-WGS-21/2021 | EPI_ISL_14569345 | 25/02/2021 | Africa / Libya | Human | unknown | unknown | unknown | unknown | unknown | unknown | A | L |
| hCoV-19/Libya/BTRC-WGS-8/2021 | EPI_ISL_14569346 | 25/02/2021 | Africa / Libya | Human | unknown | unknown | unknown | unknown | unknown | unknown | B.1.1.7 | GRY |
| hCoV-19/Libya/421067094/2020 | EPI_ISL_16648336 | 20/05/2020 | Africa / Libya / Tripoli | Human | unknown | unknown | unknown | unknown | unknown | unknown | B.1.525 | G |
| hCoV-19/Libya/421067082/2020 | EPI_ISL_16648337 | 30/05/2020 | Africa / Libya / Tripoli | Human | unknown | unknown | unknown | unknown | unknown | unknown | B.1.525 | G |
| hCoV-19/Libya/421067084/2020 | EPI_ISL_16648338 | 30/05/2020 | Africa / Libya / Tripoli | Human | unknown | unknown | unknown | unknown | unknown | unknown | B.1.525 | G |
| hCoV-19/Libya/421067087/2020 | EPI_ISL_16648339 | 30/05/2020 | Africa / Libya / Tripoli | Human | unknown | unknown | unknown | unknown | unknown | unknown | B.1.525 | G |
| hCoV-19/Libya/421067088/2020 | EPI_ISL_16648340 | 30/05/2020 | Africa / Libya / Tripoli | Human | unknown | unknown | unknown | unknown | unknown | unknown | B.1.525 | G |
| hCoV-19/Libya/421067089/2020 | EPI_ISL_16648341 | 30/05/2020 | Africa / Libya / Tripoli | Human | unknown | unknown | unknown | unknown | unknown | unknown | B.1.525 | G |
| hCoV-19/Libya/421067069/2021 | EPI_ISL_16648342 | 19/01/2021 | Africa / Libya / Tripoli | Human | unknown | unknown | unknown | unknown | unknown | unknown | B.1.525 | G |
| hCoV-19/Libya/421067070/2021 | EPI_ISL_16648343 | 19/01/2021 | Africa / Libya / Tripoli | Human | unknown | unknown | unknown | unknown | unknown | unknown | B.1.525 | G |
| hCoV-19/Libya/421067093/2021 | EPI_ISL_16648344 | 19/02/2021 | Africa / Libya / Tripoli | Human | unknown | unknown | unknown | unknown | unknown | unknown | B.1.525 | G |
| hCoV-19/Libya/421067095/2021 | EPI_ISL_16648345 | 19/02/2021 | Africa / Libya / Tripoli | Human | unknown | unknown | unknown | unknown | unknown | unknown | B.1.525 | G |
| hCoV-19/Libya/421067096/2021 | EPI_ISL_16648346 | 19/02/2021 | Africa / Libya / Tripoli | Human | unknown | unknown | unknown | unknown | unknown | unknown | B.1.525 | G |
| hCoV-19/Libya/421067071/2021 | EPI_ISL_16648347 | 20/02/2021 | Africa / Libya / Tripoli | Human | unknown | unknown | unknown | unknown | unknown | unknown | B.1.525 | G |
| hCoV-19/Libya/421067072/2021 | EPI_ISL_16648348 | 20/02/2021 | Africa / Libya / Tripoli | Human | unknown | unknown | unknown | unknown | unknown | unknown | B.1.525 | G |
| hCoV-19/Libya/421067076/2021 | EPI_ISL_16648349 | 20/02/2021 | Africa / Libya / Tripoli | Human | unknown | unknown | unknown | unknown | unknown | unknown | B.1.525 | G |
| hCoV-19/Libya/421067077/2021 | EPI_ISL_16648350 | 20/02/2021 | Africa / Libya / Tripoli | Human | unknown | unknown | unknown | unknown | unknown | unknown | B.1.525 | G |
| hCoV-19/Libya/421067078/2021 | EPI_ISL_16648351 | 20/02/2021 | Africa / Libya / Tripoli | Human | unknown | unknown | unknown | unknown | unknown | unknown | B.1.525 | G |
| hCoV-19/Libya/421067079/2021 | EPI_ISL_16648352 | 20/02/2021 | Africa / Libya / Tripoli | Human | unknown | unknown | unknown | unknown | unknown | unknown | B.1.525 | G |
| hCoV-19/Libya/421067080/2021 | EPI_ISL_16648353 | 20/02/2021 | Africa / Libya / Tripoli | Human | unknown | unknown | unknown | unknown | unknown | unknown | B.1.525 | G |
| hCoV-19/Libya/421067081/2021 | EPI_ISL_16648354 | 20/02/2021 | Africa / Libya / Tripoli | Human | unknown | unknown | unknown | unknown | unknown | unknown | B.1.525 | G |
| hCoV-19/Libya/421067090/2021 | EPI_ISL_16648355 | 21/02/2021 | Africa / Libya / Tripoli | Human | unknown | unknown | unknown | unknown | unknown | unknown | B.1.525 | G |
| hCoV-19/Libya/421067002/2021 | EPI_ISL_16648356 | 25/02/2021 | Africa / Libya / Tripoli | Human | unknown | unknown | unknown | unknown | unknown | unknown | A.29 | S |
| hCoV-19/Libya/421067007/2021 | EPI_ISL_16648357 | 25/02/2021 | Africa / Libya / Tripoli | Human | unknown | unknown | unknown | unknown | unknown | unknown | Unassigned | G |
| hCoV-19/Libya/421067010/2021 | EPI_ISL_16648358 | 25/02/2021 | Africa / Libya / Tripoli | Human | unknown | unknown | unknown | unknown | unknown | unknown | B.1.525 | G |
| hCoV-19/Libya/421067012/2021 | EPI_ISL_16648359 | 25/02/2021 | Africa / Libya / Tripoli | Human | unknown | unknown | unknown | unknown | unknown | unknown | B.1.525 | G |
| hCoV-19/Libya/421067016/2021 | EPI_ISL_16648360 | 25/02/2021 | Africa / Libya / Tripoli | Human | unknown | unknown | unknown | unknown | unknown | unknown | B.1.525 | G |
| hCoV-19/Libya/421067027/2021 | EPI_ISL_16648361 | 25/02/2021 | Africa / Libya / Tripoli | Human | unknown | unknown | unknown | unknown | unknown | unknown | Unassigned | G |
| hCoV-19/Libya/421067029/2021 | EPI_ISL_16648362 | 25/02/2021 | Africa / Libya / Tripoli | Human | unknown | unknown | unknown | unknown | unknown | unknown | B.1.525 | G |
| hCoV-19/Libya/421067030/2021 | EPI_ISL_16648363 | 25/02/2021 | Africa / Libya / Tripoli | Human | unknown | unknown | unknown | unknown | unknown | unknown | B.1.525 | G |
| hCoV-19/Libya/421067036/2021 | EPI_ISL_16648364 | 25/02/2021 | Africa / Libya / Tripoli | Human | unknown | unknown | unknown | unknown | unknown | unknown | A.29 | S |
| hCoV-19/Libya/421067038/2021 | EPI_ISL_16648365 | 25/02/2021 | Africa / Libya / Tripoli | Human | unknown | unknown | unknown | unknown | unknown | unknown | B.1.525 | G |
| hCoV-19/Libya/421067045/2021 | EPI_ISL_16648366 | 25/02/2021 | Africa / Libya / Tripoli | Human | unknown | unknown | unknown | unknown | unknown | unknown | B.1.525 | G |
| hCoV-19/Libya/421067047/2021 | EPI_ISL_16648367 | 25/02/2021 | Africa / Libya / Tripoli | Human | unknown | unknown | unknown | unknown | unknown | unknown | B.1.525 | G |
| hCoV-19/Libya/421067048/2021 | EPI_ISL_16648368 | 25/02/2021 | Africa / Libya / Tripoli | Human | unknown | unknown | unknown | unknown | unknown | unknown | Unassigned | GH |
| hCoV-19/Libya/421067050/2021 | EPI_ISL_16648369 | 25/02/2021 | Africa / Libya / Tripoli | Human | unknown | unknown | unknown | unknown | unknown | unknown | B.1.525 | G |
| hCoV-19/Libya/421067051/2021 | EPI_ISL_16648370 | 25/02/2021 | Africa / Libya / Tripoli | Human | unknown | unknown | unknown | unknown | unknown | unknown | B.1.525 | G |
| hCoV-19/Libya/421067052/2021 | EPI_ISL_16648371 | 25/02/2021 | Africa / Libya / Tripoli | Human | unknown | unknown | unknown | unknown | unknown | unknown | B.1.525 | G |
| hCoV-19/Libya/421067054/2021 | EPI_ISL_16648372 | 25/02/2021 | Africa / Libya / Tripoli | Human | unknown | unknown | unknown | unknown | unknown | unknown | B.1.525 | G |
| hCoV-19/Libya/421067056/2021 | EPI_ISL_16648373 | 25/02/2021 | Africa / Libya / Tripoli | Human | unknown | unknown | unknown | unknown | unknown | unknown | B.1.525 | G |
| hCoV-19/Libya/421067063/2021 | EPI_ISL_16648374 | 25/02/2021 | Africa / Libya / Tripoli | Human | unknown | unknown | unknown | unknown | unknown | unknown | B.1.525 | G |
| hCoV-19/Libya/421067066/2021 | EPI_ISL_16648375 | 25/02/2021 | Africa / Libya / Tripoli | Human | unknown | unknown | unknown | unknown | unknown | unknown | B.1.525 | G |
| hCoV-19/Libya/421067067/2021 | EPI_ISL_16648376 | 25/02/2021 | Africa / Libya / Tripoli | Human | unknown | unknown | unknown | unknown | unknown | unknown | B.1.525 | G |
| hCoV-19/Libya/421067068/2021 | EPI_ISL_16648377 | 25/02/2021 | Africa / Libya / Tripoli | Human | unknown | unknown | unknown | unknown | unknown | unknown | B.1.525 | G |
| hCoV-19/Libya/421067075/2021 | EPI_ISL_16652173 | 20/02/2021 | Africa / Libya / Tripoli | Human | unknown | unknown | unknown | unknown | unknown | unknown | B.1.525 | G |
| hCoV-19/Libya/421067004/2021 | EPI_ISL_16652174 | 25/02/2021 | Africa / Libya / Tripoli | Human | unknown | unknown | unknown | unknown | unknown | unknown | B.1.525 | G |
| hCoV-19/Libya/421067011/2021 | EPI_ISL_16652175 | 25/02/2021 | Africa / Libya / Tripoli | Human | unknown | unknown | unknown | unknown | unknown | unknown | B.1.36 | GH |
| hCoV-19/Libya/421067015/2021 | EPI_ISL_16652176 | 25/02/2021 | Africa / Libya / Tripoli | Human | unknown | unknown | unknown | unknown | unknown | unknown | A.27 | S |
| hCoV-19/Libya/421067018/2021 | EPI_ISL_16652177 | 25/02/2021 | Africa / Libya / Tripoli | Human | unknown | unknown | unknown | unknown | unknown | unknown | A.27 | S |
| hCoV-19/Libya/421067020/2021 | EPI_ISL_16652178 | 25/02/2021 | Africa / Libya / Tripoli | Human | unknown | unknown | unknown | unknown | unknown | unknown | B.1.525 | G |
| hCoV-19/Libya/421067023/2021 | EPI_ISL_16652179 | 25/02/2021 | Africa / Libya / Tripoli | Human | unknown | unknown | unknown | unknown | unknown | unknown | A | S |
| hCoV-19/Libya/421067025/2021 | EPI_ISL_16652180 | 25/02/2021 | Africa / Libya / Tripoli | Human | unknown | unknown | unknown | unknown | unknown | unknown | A.27 | S |
| hCoV-19/Libya/421067026/2021 | EPI_ISL_16652181 | 25/02/2021 | Africa / Libya / Tripoli | Human | unknown | unknown | unknown | unknown | unknown | unknown | A.27 | S |
| hCoV-19/Libya/421067028/2021 | EPI_ISL_16652182 | 25/02/2021 | Africa / Libya / Tripoli | Human | unknown | unknown | unknown | unknown | unknown | unknown | A.27 | S |
| hCoV-19/Libya/421067032/2021 | EPI_ISL_16652183 | 25/02/2021 | Africa / Libya / Tripoli | Human | unknown | unknown | unknown | unknown | unknown | unknown | A.27 | S |
| hCoV-19/Libya/421067039/2021 | EPI_ISL_16652184 | 25/02/2021 | Africa / Libya / Tripoli | Human | unknown | unknown | unknown | unknown | unknown | unknown | B.1.525 | G |
| hCoV-19/Libya/421067041/2021 | EPI_ISL_16652185 | 25/02/2021 | Africa / Libya / Tripoli | Human | unknown | unknown | unknown | unknown | unknown | unknown | A.27 | S |
| hCoV-19/Libya/421067042/2021 | EPI_ISL_16652186 | 25/02/2021 | Africa / Libya / Tripoli | Human | unknown | unknown | unknown | unknown | unknown | unknown | A.27 | S |
| hCoV-19/Libya/421067044/2021 | EPI_ISL_16652187 | 25/02/2021 | Africa / Libya / Tripoli | Human | unknown | unknown | unknown | unknown | unknown | unknown | A.27 | S |
| hCoV-19/Libya/421067120/2021 | EPI_ISL_17682070 | 03/03/2021 | Africa / Libya / Tripoli | Human | unknown | unknown | unknown | unknown | unknown | unknown | B.1.160 | GH |
| hCoV-19/Libya/421067121/2021 | EPI_ISL_17682071 | 03/03/2021 | Africa / Libya / Tripoli | Human | unknown | unknown | unknown | unknown | unknown | unknown | B.1.177 | GV |
| hCoV-19/Libya/421067123/2021 | EPI_ISL_17682072 | 03/03/2021 | Africa / Libya / Tripoli | Human | unknown | unknown | unknown | unknown | unknown | unknown | B.1.160 | GH |
| hCoV-19/Libya/421067124/2021 | EPI_ISL_17682073 | 03/03/2021 | Africa / Libya / Tripoli | Human | unknown | unknown | unknown | unknown | unknown | unknown | B.1.177 | G |
| hCoV-19/Libya/421067097/2021 | EPI_ISL_17682074 | 19/02/2021 | Africa / Libya / Tripoli | Human | unknown | unknown | unknown | unknown | unknown | unknown | B.1.160 | GH |
| hCoV-19/Libya/421067098/2020 | EPI_ISL_17682075 | 20/05/2020 | Africa / Libya / Tripoli | Human | unknown | unknown | unknown | unknown | unknown | unknown | B.1.597 | GH |
| hCoV-19/Libya/421067099/2021 | EPI_ISL_17682076 | 19/02/2021 | Africa / Libya / Tripoli | Human | unknown | unknown | unknown | unknown | unknown | unknown | B.1.177 | GV |
| hCoV-19/Libya/421067100/2020 | EPI_ISL_17682077 | 11/06/2020 | Africa / Libya / Tripoli | Human | unknown | unknown | unknown | unknown | unknown | unknown | B.1.1 | GR |
| hCoV-19/Libya/421067101/2020 | EPI_ISL_17682078 | 12/06/2020 | Africa / Libya / Tripoli | Human | unknown | unknown | unknown | unknown | unknown | unknown | B.1.177 | GV |
| hCoV-19/Libya/421067103/2020 | EPI_ISL_17682079 | 11/06/2020 | Africa / Libya / Tripoli | Human | unknown | unknown | unknown | unknown | unknown | unknown | A | S |
| hCoV-19/Libya/421067104/2020 | EPI_ISL_17682080 | 11/06/2020 | Africa / Libya / Tripoli | Human | unknown | unknown | unknown | unknown | unknown | unknown | A | S |
| hCoV-19/Libya/421067105/2020 | EPI_ISL_17682081 | 14/06/2020 | Africa / Libya / Tripoli | Human | unknown | unknown | unknown | unknown | unknown | unknown | A | S |
| hCoV-19/Libya/421067106/2020 | EPI_ISL_17682082 | 14/06/2020 | Africa / Libya / Tripoli | Human | unknown | unknown | unknown | unknown | unknown | unknown | A | S |
| hCoV-19/Libya/421067107/2020 | EPI_ISL_17682083 | 22/06/2020 | Africa / Libya / Tripoli | Human | unknown | unknown | unknown | unknown | unknown | unknown | A | S |
| hCoV-19/Libya/421067108/2020 | EPI_ISL_17682084 | 22/06/2020 | Africa / Libya / Tripoli | Human | unknown | unknown | unknown | unknown | unknown | unknown | B.1.36 | GH |
| hCoV-19/Libya/421067109/2020 | EPI_ISL_17682085 | 22/06/2020 | Africa / Libya / Tripoli | Human | unknown | unknown | unknown | unknown | unknown | unknown | B.1.1 | GR |
| hCoV-19/Libya/421067110/2020 | EPI_ISL_17682086 | 22/06/2020 | Africa / Libya / Tripoli | Human | unknown | unknown | unknown | unknown | unknown | unknown | A | S |
| hCoV-19/Libya/421067111/2020 | EPI_ISL_17682087 | 22/06/2020 | Africa / Libya / Tripoli | Human | unknown | unknown | unknown | unknown | unknown | unknown | B.1.597 | GH |
| hCoV-19/Libya/421067112/2020 | EPI_ISL_17682088 | 22/06/2020 | Africa / Libya / Tripoli | Human | unknown | unknown | unknown | unknown | unknown | unknown | B.1.1 | GR |
| hCoV-19/Libya/421067113/2020 | EPI_ISL_17682089 | 22/06/2020 | Africa / Libya / Tripoli | Human | unknown | unknown | unknown | unknown | unknown | unknown | B.1 | GH |
| hCoV-19/Libya/421067114/2020 | EPI_ISL_17682090 | 18/06/2020 | Africa / Libya / Tripoli | Human | unknown | unknown | unknown | unknown | unknown | unknown | B.1 | GH |
| hCoV-19/Libya/421067115/2021 | EPI_ISL_17682091 | 03/03/2021 | Africa / Libya / Tripoli | Human | unknown | unknown | unknown | unknown | unknown | unknown | B.1.177 | GV |
| hCoV-19/Libya/421067116/2021 | EPI_ISL_17682092 | 03/03/2021 | Africa / Libya / Tripoli | Human | unknown | unknown | unknown | unknown | unknown | unknown | B.1.22 | GH |
| hCoV-19/Libya/421067117/2021 | EPI_ISL_17682093 | 03/03/2021 | Africa / Libya / Tripoli | Human | unknown | unknown | unknown | unknown | unknown | unknown | BQ.1.1 | GRA |
| hCoV-19/Libya/421067118/2021 | EPI_ISL_17690240 | 03/03/2021 | Africa / Libya / Tripoli | Human | unknown | unknown | unknown | unknown | unknown | unknown | B.1.1 | GR |
| hCoV-19/Libya/421067119/2021 | EPI_ISL_17690241 | 03/03/2021 | Africa / Libya / Tripoli | Human | unknown | unknown | unknown | unknown | unknown | unknown | B.1.177 | GV |
| hCoV-19/Libya/421067122/2021 | EPI_ISL_17690242 | 03/03/2021 | Africa / Libya / Tripoli | Human | unknown | unknown | unknown | unknown | unknown | unknown | B.1.160 | GH |
| hCoV-19/Libya/12371/2020 | EPI_ISL_2649891 | 2020-06 | Africa / Libya | Human | unknown | unknown | unknown | unknown | unknown | unknown | A | S |
| hCoV-19/Libya/12156/2020 | EPI_ISL_2649892 | 2020-06 | Africa / Libya | Human | unknown | unknown | unknown | unknown | unknown | unknown | A | S |
| hCoV-19/Libya/11106/2020 | EPI_ISL_2649893 | 2020-06 | Africa / Libya | Human | unknown | unknown | unknown | unknown | unknown | unknown | A | S |
| hCoV-19/Libya/11040/2020 | EPI_ISL_2649894 | 2020-06 | Africa / Libya | Human | unknown | unknown | unknown | unknown | unknown | unknown | A | S |
| hCoV-19/Libya/10298/2020 | EPI_ISL_2649895 | 2020-06 | Africa / Libya | Human | unknown | unknown | unknown | unknown | unknown | unknown | A | S |
| hCoV-19/Libya/10230/2020 | EPI_ISL_2649896 | 2020-06 | Africa / Libya | Human | unknown | unknown | unknown | unknown | unknown | unknown | B.1.36 | GH |
| hCoV-19/Libya/958/2020 | EPI_ISL_2649897 | 2020-06 | Africa / Libya | Human | unknown | unknown | unknown | unknown | unknown | unknown | A | S |
| hCoV-19/Libya/7700/2020 | EPI_ISL_2649898 | 2020-06 | Africa / Libya | Human | unknown | unknown | unknown | unknown | unknown | unknown | A | S |
| hCoV-19/Libya/449/2020 | EPI_ISL_2649899 | 2020-06 | Africa / Libya | Human | unknown | unknown | unknown | unknown | unknown | unknown | A | S |
| hCoV-19/Libya/75/2020 | EPI_ISL_2649900 | 2020-06 | Africa / Libya | Human | unknown | unknown | unknown | unknown | unknown | unknown | A | S |
| hCoV-19/Libya/EMC-1/2021 | EPI_ISL_2860636 | 18/02/2021 | Africa / Libya | Human | unknown | unknown | unknown | unknown | unknown | unknown | B.1.525 | G |
| hCoV-19/Libya/EMC-2/2021 | EPI_ISL_2860637 | 20/02/2021 | Africa / Libya | Human | unknown | unknown | unknown | unknown | unknown | unknown | B.1.525 | G |
| hCoV-19/Libya/EMC-3/2021 | EPI_ISL_2860638 | 20/02/2021 | Africa / Libya | Human | unknown | unknown | unknown | unknown | unknown | unknown | B.1.525 | G |
| hCoV-19/Libya/EMC-4/2021 | EPI_ISL_2860639 | 30/05/2021 | Africa / Libya | Human | unknown | unknown | unknown | unknown | unknown | unknown | B.1.525 | G |
| hCoV-19/Libya/EMC-5/2021 | EPI_ISL_2860640 | 30/05/2021 | Africa / Libya | Human | unknown | unknown | unknown | unknown | unknown | unknown | B.1.525 | G |
| hCoV-19/Libya/EMC-6/2021 | EPI_ISL_2860641 | 02/06/2021 | Africa / Libya | Human | unknown | unknown | unknown | unknown | unknown | unknown | B.1.525 | G |
| hCoV-19/Libya/EMC-7/2021 | EPI_ISL_2860642 | 05/06/2021 | Africa / Libya | Human | unknown | unknown | unknown | unknown | unknown | unknown | B.1.525 | G |
| hCoV-19/Libya/EMC-8/2021 | EPI_ISL_2860643 | 05/06/2021 | Africa / Libya | Human | unknown | unknown | unknown | unknown | unknown | unknown | B.1.525 | G |
| hCoV-19/Libya/EMC-9/2021 | EPI_ISL_2860644 | 07/06/2021 | Africa / Libya | Human | unknown | unknown | unknown | unknown | unknown | unknown | B.1.525 | G |
| hCoV-19/Libya/EMC-10/2021 | EPI_ISL_2860645 | 07/06/2021 | Africa / Libya | Human | unknown | unknown | unknown | unknown | unknown | unknown | B.1.525 | G |
| hCoV-19/Libya/EMC-11/2021 | EPI_ISL_2860646 | 05/06/2021 | Africa / Libya | Human | unknown | unknown | unknown | unknown | unknown | unknown | B.1.1.7 | GRY |
| hCoV-19/Libya/EMC-12/2021 | EPI_ISL_2860647 | 05/06/2021 | Africa / Libya | Human | unknown | unknown | unknown | unknown | unknown | unknown | B.1.525 | G |
| hCoV-19/Libya/MZ054864/2021 | EPI_ISL_8064591 | 17/02/2021 | Africa / Libya / Tripoli | Human | Baseline surveillance | unknown | unknown | unknown | unknown | Baseline surveillance | B.1.1.7 | GRY |
| hCoV-19/Libya/MZ054857/2021 | EPI_ISL_8295636 | 25/02/2021 | Africa / Libya / Tripoli | Human | unknown | unknown | unknown | unknown | unknown | unknown | B.1.525 | G |
| hCoV-19/Libya/MZ054858/2021 | EPI_ISL_8295637 | 25/02/2021 | Africa / Libya / Tripoli | Human | unknown | unknown | unknown | unknown | unknown | unknown | B.1.525 | G |
| hCoV-19/Libya/MZ054859/2021 | EPI_ISL_8295638 | 25/02/2021 | Africa / Libya / Tripoli | Human | unknown | unknown | unknown | unknown | unknown | unknown | B.1.525 | G |
| hCoV-19/Libya/MZ054860/2021 | EPI_ISL_8295639 | 25/02/2021 | Africa / Libya / Tripoli | Human | unknown | unknown | unknown | unknown | unknown | unknown | B.1.525 | G |
| hCoV-19/Libya/MZ054861/2021 | EPI_ISL_8295640 | 25/02/2021 | Africa / Libya / Tripoli | Human | unknown | unknown | unknown | unknown | unknown | unknown | B.1.525 | G |
| hCoV-19/Libya/MZ054862/2020 | EPI_ISL_8295641 | 30/05/2020 | Africa / Libya / Tripoli | Human | unknown | unknown | unknown | unknown | unknown | unknown | B.1.525 | G |
| hCoV-19/Libya/MZ054863/2021 | EPI_ISL_8295642 | 25/02/2021 | Africa / Libya / Tripoli | Human | unknown | unknown | unknown | unknown | unknown | unknown | B.1.525 | G |
| hCoV-19/Libya/MZ054865/2021 | EPI_ISL_8295643 | 25/02/2021 | Africa / Libya / Tripoli | Human | unknown | unknown | unknown | unknown | unknown | unknown | Unassigned | G |
| hCoV-19/Libya/MZ054866/2021 | EPI_ISL_8295644 | 25/02/2021 | Africa / Libya / Tripoli | Human | unknown | unknown | unknown | unknown | unknown | unknown | B.1 | G |
| hCoV-19/Libya/MZ054867/2021 | EPI_ISL_8295645 | 25/02/2021 | Africa / Libya / Tripoli | Human | unknown | unknown | unknown | unknown | unknown | unknown | B.1.525 | G |
| hCoV-19/Libya/MZ054868/2021 | EPI_ISL_8295646 | 25/02/2021 | Africa / Libya / Tripoli | Human | unknown | unknown | unknown | unknown | unknown | unknown | B.1.525 | G |
| hCoV-19/Libya/MZ054869/2021 | EPI_ISL_8295647 | 25/02/2021 | Africa / Libya / Tripoli | Human | unknown | unknown | unknown | unknown | unknown | unknown | B.1.525 | G |
| hCoV-19/Libya/MZ054870/2021 | EPI_ISL_8295648 | 25/02/2021 | Africa / Libya / Tripoli | Human | unknown | unknown | unknown | unknown | unknown | unknown | B.1.525 | G |
| hCoV-19/Libya/MZ054871/2021 | EPI_ISL_8295649 | 25/02/2021 | Africa / Libya / Tripoli | Human | unknown | unknown | unknown | unknown | unknown | unknown | B.1.525 | G |
| hCoV-19/Libya/MZ054872/2021 | EPI_ISL_8295650 | 25/02/2021 | Africa / Libya / Tripoli | Human | unknown | unknown | unknown | unknown | unknown | unknown | B.1.525 | G |
| hCoV-19/Libya/MZ054873/2021 | EPI_ISL_8295651 | 25/02/2021 | Africa / Libya / Tripoli | Human | unknown | unknown | unknown | unknown | unknown | unknown | B.1.525 | G |
| hCoV-19/Libya/MZ054874/2021 | EPI_ISL_8295652 | 25/02/2021 | Africa / Libya / Tripoli | Human | unknown | unknown | unknown | unknown | unknown | unknown | B.1.525 | GH |
| hCoV-19/Libya/MZ054875/2021 | EPI_ISL_8295653 | 25/02/2021 | Africa / Libya / Tripoli | Human | unknown | unknown | unknown | unknown | unknown | unknown | B.1.525 | G |
| hCoV-19/Libya/MZ054876/2020 | EPI_ISL_8295654 | 30/05/2020 | Africa / Libya / Tripoli | Human | unknown | unknown | unknown | unknown | unknown | unknown | B.1.525 | G |
| hCoV-19/Libya/MZ054878/2021 | EPI_ISL_8295655 | 25/02/2021 | Africa / Libya / Tripoli | Human | unknown | unknown | unknown | unknown | unknown | unknown | B.1.525 | G |
| hCoV-19/Libya/MZ054879/2021 | EPI_ISL_8295656 | 25/02/2021 | Africa / Libya / Tripoli | Human | unknown | unknown | unknown | unknown | unknown | unknown | B.1 | G |
| hCoV-19/Libya/MZ054880/2021 | EPI_ISL_8295657 | 25/02/2021 | Africa / Libya / Tripoli | Human | unknown | unknown | unknown | unknown | unknown | unknown | B.1.525 | G |
| hCoV-19/Libya/MZ054881/2021 | EPI_ISL_8295658 | 25/02/2021 | Africa / Libya / Tripoli | Human | unknown | unknown | unknown | unknown | unknown | unknown | B.1.1.7 | GRY |
| hCoV-19/Libya/MZ054882/2021 | EPI_ISL_8295659 | 25/02/2021 | Africa / Libya / Tripoli | Human | unknown | unknown | unknown | unknown | unknown | unknown | B.1.525 | G |
| hCoV-19/Libya/MZ054883/2021 | EPI_ISL_8295660 | 25/02/2021 | Africa / Libya / Tripoli | Human | unknown | unknown | unknown | unknown | unknown | unknown | B.1.525 | G |
| hCoV-19/Libya/MZ054884/2021 | EPI_ISL_8295661 | 25/02/2021 | Africa / Libya / Tripoli | Human | unknown | unknown | unknown | unknown | unknown | unknown | B.1.525 | G |
| hCoV-19/Libya/MZ054885/2021 | EPI_ISL_8295662 | 25/02/2021 | Africa / Libya / Tripoli | Human | unknown | unknown | unknown | unknown | unknown | unknown | B.1.525 | G |
| hCoV-19/Libya/MZ054886/2021 | EPI_ISL_8295663 | 25/02/2021 | Africa / Libya / Tripoli | Human | unknown | unknown | unknown | unknown | unknown | unknown | B.1.525 | G |
| hCoV-19/Libya/MZ054887/2020 | EPI_ISL_8295664 | 30/05/2020 | Africa / Libya / Tripoli | Human | unknown | unknown | unknown | unknown | unknown | unknown | B.1.525 | G |
| hCoV-19/Libya/MZ054889/2021 | EPI_ISL_8295665 | 25/02/2021 | Africa / Libya / Tripoli | Human | unknown | unknown | unknown | unknown | unknown | unknown | L.3 | GR |
| hCoV-19/Libya/MZ054890/2021 | EPI_ISL_8295666 | 25/02/2021 | Africa / Libya / Tripoli | Human | unknown | unknown | unknown | unknown | unknown | unknown | B.1.525 | G |
| hCoV-19/Libya/MZ054891/2021 | EPI_ISL_8295667 | 25/02/2021 | Africa / Libya / Tripoli | Human | unknown | unknown | unknown | unknown | unknown | unknown | B.1.1 | GR |
| hCoV-19/Libya/MZ054892/2021 | EPI_ISL_8295668 | 25/02/2021 | Africa / Libya / Tripoli | Human | unknown | unknown | unknown | unknown | unknown | unknown | B.1.525 | G |
