# Supplementary material for: Genomic surveillance of SARS-CoV-2 in North Africa: 4 years of GISAID data sharing
Source: IJID Reg. 2024 Mar 19;11:100356. doi: 10.1016/j.ijregi.2024.100356 (PMC11035039; doi:10.1016/j.ijregi.2024.100356)
Supplement: Supplementary file 4 [file mmc4.docx]

Table S3. Comprehensive Virus Data from Tunisia Including Virus Name, Accession Number, and Clinical Attributes (based on data downloaded from GISAID per 15 September 2023)

| Virus name | Accession ID | Collection date | Location | Host | Sampling strategy | Gender | Patient age (years) | Patient status | Last vaccinated | Sampling strategy | Lineage | Clade |
| --- | --- | --- | --- | --- | --- | --- | --- | --- | --- | --- | --- | --- |
| hCoV-19/Tunisia/H-0350/2021 | EPI_ISL_10101346 | 26/11/2021 | Africa / Tunisia | Human | unknown | Female | 35 | unknown | unknown | unknown | B.1.160 | GH |
| hCoV-19/Tunisia/F-3452/2021 | EPI_ISL_8298536 | 16/09/2021 | Africa / Tunisia / Tunis | Human | Random | Female | 31 | unknown | unknown | Random | AY.122 | GK |
| hCoV-19/Tunisia/S1579/2021 | EPI_ISL_9058825 | 02/12/2021 | Africa / Tunisia | Human | Active surveillance | Male | 23 | Live | unknown | Active surveillance | BA.1 | GRA |
| hCoV-19/Tunisia/Y-6757/2021 | EPI_ISL_10101267 | 20/10/2021 | Africa / Tunisia | Human | unknown | Male | 40 | unknown | unknown | unknown | Unassigned | G |
| hCoV-19/Tunisia/F-4003/2021 | EPI_ISL_8309619 | 27/09/2021 | Africa / Tunisia / Tunis | Human | Random | Female | 28 | unknown | unknown | Random | AY.122 | GK |
| hCoV-19/Tunisia/V-2179/2021 | EPI_ISL_8309620 | 28/09/2021 | Africa / Tunisia / Tunis | Human | Random | Female | 48 | unknown | unknown | Random | AY.122 | GK |
| hCoV-19/Tunisia/F-2729/2021 | EPI_ISL_8309617 | 03/09/2021 | Africa / Tunisia / Tunis | Human | Random | Male | 32 | unknown | unknown | Random | AY.122 | GK |
| hCoV-19/Tunisia/F-0683/2021 | EPI_ISL_8309615 | 05/08/2021 | Africa / Tunisia / Tunis | Human | Random | Male | 45 | unknown | unknown | Random | AY.122 | GK |
| hCoV-19/Tunisia/S-1039/2021 | EPI_ISL_8309614 | 15/09/2021 | Africa / Tunisia / Sousse | Human | Random | Male | 47 | unknown | unknown | Random | AY.122 | GK |
| hCoV-19/Tunisia/V-0679/2021 | EPI_ISL_8298552 | 02/08/2021 | Africa / Tunisia / Tunis | Human | Random | Male | 57 | unknown | unknown | Random | AY.122 | GK |
| hCoV-19/Tunisia/F-4000/2021 | EPI_ISL_8298551 | 27/09/2021 | Africa / Tunisia / Tunis | Human | Random | Female | 46 | unknown | unknown | Random | AY.122 | GK |
| hCoV-19/Tunisia/F-3965/2021 | EPI_ISL_8298550 | 27/09/2021 | Africa / Tunisia / Tunis | Human | Random | Male | 38 | unknown | unknown | Random | AY.122 | GK |
| hCoV-19/Tunisia/F-3746/2021 | EPI_ISL_8298547 | 21/09/2021 | Africa / Tunisia / Tunis | Human | Random | Female | 48 | unknown | unknown | Random | AY.122 | GK |
| hCoV-19/Tunisia/F-3710/2021 | EPI_ISL_8298545 | 21/09/2021 | Africa / Tunisia / Tunis | Human | Random | Male | 29 | unknown | unknown | Random | AY.122 | GK |
| hCoV-19/Tunisia/F-3693/2021 | EPI_ISL_8298544 | 21/09/2021 | Africa / Tunisia / Tunis | Human | Random | Female | 32 | unknown | unknown | Random | AY.122 | GK |
| hCoV-19/Tunisia/F-3651/2021 | EPI_ISL_8298543 | 20/09/2021 | Africa / Tunisia / Tunis | Human | Random | Female | 64 | unknown | unknown | Random | AY.122 | GK |
| hCoV-19/Tunisia/F-3627/2021 | EPI_ISL_8298542 | 20/09/2021 | Africa / Tunisia / Tunis | Human | Random | Female | 44 | unknown | unknown | Random | AY.122 | GK |
| hCoV-19/Tunisia/F-3577/2021 | EPI_ISL_8298541 | 18/09/2021 | Africa / Tunisia / Tunis | Human | Random | Female | 21 | unknown | unknown | Random | AY.122 | GK |
| hCoV-19/Tunisia/F-2978/2021 | EPI_ISL_8309618 | 07/09/2021 | Africa / Tunisia / Tunis | Human | Random | Male | 38 | unknown | unknown | Random | AY.122 | GK |
| hCoV-19/Tunisia/F-3469/2021 | EPI_ISL_8298538 | 16/09/2021 | Africa / Tunisia / Tunis | Human | Random | Female | 53 | unknown | unknown | Random | AY.122 | GK |
| hCoV-19/Tunisia/F-3486/2021 | EPI_ISL_8298539 | 16/09/2021 | Africa / Tunisia / Tunis | Human | Random | Female | 46 | unknown | unknown | Random | AY.122 | GK |
| hCoV-19/Tunisia/F-3464/2021 | EPI_ISL_8298537 | 16/09/2021 | Africa / Tunisia / Tunis | Human | Random | Female | 38 | unknown | unknown | Random | AY.122 | GK |
| hCoV-19/Tunisia/F-3422/2021 | EPI_ISL_8298535 | 15/09/2021 | Africa / Tunisia / Tunis | Human | Random | Female | 58 | unknown | unknown | Random | AY.122 | GK |
| hCoV-19/Tunisia/F-3411/2021 | EPI_ISL_8298534 | 15/09/2021 | Africa / Tunisia / Tunis | Human | Random | Male | 44 | unknown | unknown | Random | AY.122 | GK |
| hCoV-19/Tunisia/F-3399/2021 | EPI_ISL_8298533 | 15/09/2021 | Africa / Tunisia / Tunis | Human | Random | Female | 32 | unknown | unknown | Random | AY.122 | GK |
| hCoV-19/Tunisia/F-3350/2021 | EPI_ISL_8298532 | 14/09/2021 | Africa / Tunisia / Tunis | Human | Random | Female | 32 | unknown | unknown | Random | AY.122 | GK |
| hCoV-19/Tunisia/F-3344/2021 | EPI_ISL_8298531 | 14/09/2021 | Africa / Tunisia / Tunis | Human | Random | Female | 21 | unknown | unknown | Random | AY.122 | GK |
| hCoV-19/Tunisia/F-3277/2021 | EPI_ISL_8298529 | 13/09/2021 | Africa / Tunisia / Tunis | Human | Random | Male | 14 | unknown | unknown | Random | AY.122 | GK |
| hCoV-19/Tunisia/F-3266/2021 | EPI_ISL_8298528 | 13/09/2021 | Africa / Tunisia / Tunis | Human | Random | Male | 28 | unknown | unknown | Random | AY.122 | GK |
| hCoV-19/Tunisia/F-3243/2021 | EPI_ISL_8298527 | 12/09/2021 | Africa / Tunisia / Tunis | Human | Random | Female | 33 | unknown | unknown | Random | AY.122 | GK |
| hCoV-19/Tunisia/F-3214/2021 | EPI_ISL_8298526 | 11/09/2021 | Africa / Tunisia / Tunis | Human | Random | Female | 44 | unknown | unknown | Random | AY.122 | GK |
| hCoV-19/Tunisia/F-3173/2021 | EPI_ISL_8298524 | 10/09/2021 | Africa / Tunisia / Tunis | Human | Random | Male | 34 | unknown | unknown | Random | AY.122 | GK |
| hCoV-19/Tunisia/F-3160/2021 | EPI_ISL_8298522 | 10/09/2021 | Africa / Tunisia / Tunis | Human | Random | Female | 27 | unknown | unknown | Random | AY.122 | GK |
| hCoV-19/Tunisia/F-3162/2021 | EPI_ISL_8298523 | 10/09/2021 | Africa / Tunisia / Tunis | Human | Random | Female | 44 | unknown | unknown | Random | AY.122 | GK |
| hCoV-19/Tunisia/F-3138/2021 | EPI_ISL_8298521 | 10/09/2021 | Africa / Tunisia / Tunis | Human | Random | Male | 34 | unknown | unknown | Random | AY.122 | GK |
| hCoV-19/Tunisia/F-3082/2021 | EPI_ISL_8298519 | 09/09/2021 | Africa / Tunisia / Tunis | Human | Random | Female | 42 | unknown | unknown | Random | AY.122 | GK |
| hCoV-19/Tunisia/F-3081/2021 | EPI_ISL_8298518 | 09/09/2021 | Africa / Tunisia / Tunis | Human | Random | Female | 68 | unknown | unknown | Random | AY.122 | GK |
| hCoV-19/Tunisia/F-3057/2021 | EPI_ISL_8298517 | 08/09/2021 | Africa / Tunisia / Tunis | Human | Random | Male | 62 | unknown | unknown | Random | AY.122 | GK |
| hCoV-19/Tunisia/F-3053/2021 | EPI_ISL_8298516 | 08/09/2021 | Africa / Tunisia / Tunis | Human | Random | Male | 48 | unknown | unknown | Random | AY.122 | GK |
| hCoV-19/Tunisia/F-3050/2021 | EPI_ISL_8298515 | 08/09/2021 | Africa / Tunisia / Tunis | Human | Random | Male | 32 | unknown | unknown | Random | AY.122 | GK |
| hCoV-19/Tunisia/F-3036/2021 | EPI_ISL_8298514 | 08/09/2021 | Africa / Tunisia / Tunis | Human | Random | Female | 56 | unknown | unknown | Random | AY.122 | GK |
| hCoV-19/Tunisia/F-2998/2021 | EPI_ISL_8298513 | 07/09/2021 | Africa / Tunisia / Tunis | Human | Random | Female | 55 | unknown | unknown | Random | AY.122 | GK |
| hCoV-19/Tunisia/F-2994/2021 | EPI_ISL_8298512 | 07/09/2021 | Africa / Tunisia / Tunis | Human | Random | Female | 71 | unknown | unknown | Random | AY.122 | GK |
| hCoV-19/Tunisia/F-2976/2021 | EPI_ISL_8298511 | 07/09/2021 | Africa / Tunisia / Tunis | Human | Random | Female | 33 | unknown | unknown | Random | AY.122 | GK |
| hCoV-19/Tunisia/F-2962/2021 | EPI_ISL_8298509 | 07/09/2021 | Africa / Tunisia / Tunis | Human | Random | Female | 25 | unknown | unknown | Random | AY.122 | GK |
| hCoV-19/Tunisia/F-2899/2021 | EPI_ISL_8298508 | 06/09/2021 | Africa / Tunisia / Tunis | Human | Random | Female | 16 | unknown | unknown | Random | AY.122 | GK |
| hCoV-19/Tunisia/F-2894/2021 | EPI_ISL_8298507 | 06/09/2021 | Africa / Tunisia / Tunis | Human | Random | Male | 29 | unknown | unknown | Random | AY.122 | GK |
| hCoV-19/Tunisia/F-2884/2021 | EPI_ISL_8298505 | 06/09/2021 | Africa / Tunisia / Tunis | Human | Random | Male | 42 | unknown | unknown | Random | AY.122 | GK |
| hCoV-19/Tunisia/F-2878/2021 | EPI_ISL_8298504 | 06/09/2021 | Africa / Tunisia / Tunis | Human | Random | Male | 27 | unknown | unknown | Random | AY.122 | GK |
| hCoV-19/Tunisia/F-2857/2021 | EPI_ISL_8298503 | 06/09/2021 | Africa / Tunisia / Tunis | Human | Random | Male | 46 | unknown | unknown | Random | AY.122 | GK |
| hCoV-19/Tunisia/F-2763/2021 | EPI_ISL_8298502 | 04/09/2021 | Africa / Tunisia / Tunis | Human | Random | Female | 44 | unknown | unknown | Random | AY.122 | GK |
| hCoV-19/Tunisia/F-2715/2021 | EPI_ISL_8298500 | 03/09/2021 | Africa / Tunisia / Tunis | Human | Random | Female | 33 | unknown | unknown | Random | AY.122 | GK |
| hCoV-19/Tunisia/F-2703/2021 | EPI_ISL_8298499 | 03/09/2021 | Africa / Tunisia / Tunis | Human | Random | Male | 34 | unknown | unknown | Random | AY.122 | GK |
| hCoV-19/Tunisia/F-2616/2021 | EPI_ISL_8298498 | 02/09/2021 | Africa / Tunisia / Tunis | Human | Random | Male | 23 | unknown | unknown | Random | AY.122 | GK |
| hCoV-19/Tunisia/F-2581/2021 | EPI_ISL_8298495 | 01/09/2021 | Africa / Tunisia / Tunis | Human | Random | Male | 23 | unknown | unknown | Random | AY.122 | GK |
| hCoV-19/Tunisia/F-2575/2021 | EPI_ISL_8298494 | 01/09/2021 | Africa / Tunisia / Tunis | Human | Random | Female | 52 | unknown | unknown | Random | AY.122 | GK |
| hCoV-19/Tunisia/F-2334/2021 | EPI_ISL_8298493 | 29/08/2021 | Africa / Tunisia / Tunis | Human | Random | Male | 73 | unknown | unknown | Random | AY.122 | GK |
| hCoV-19/Tunisia/F-2108/2021 | EPI_ISL_8298492 | 26/08/2021 | Africa / Tunisia / Tunis | Human | Random | Female | 32 | unknown | unknown | Random | AY.122 | GK |
| hCoV-19/Tunisia/F-2013/2021 | EPI_ISL_8298491 | 25/08/2021 | Africa / Tunisia / Tunis | Human | Random | Female | 46 | unknown | unknown | Random | AY.122 | GK |
| hCoV-19/Tunisia/F-1989/2021 | EPI_ISL_8298489 | 25/08/2021 | Africa / Tunisia / Tunis | Human | Random | Female | 30 | unknown | unknown | Random | AY.122 | GK |
| hCoV-19/Tunisia/F-1978/2021 | EPI_ISL_8298487 | 25/08/2021 | Africa / Tunisia / Tunis | Human | Random | Female | 40 | unknown | unknown | Random | AY.122 | GK |
| hCoV-19/Tunisia/F-1912/2021 | EPI_ISL_8298484 | 24/08/2021 | Africa / Tunisia / Tunis | Human | Random | Male | 27 | unknown | unknown | Random | AY.122 | GK |
| hCoV-19/Tunisia/F-1878/2021 | EPI_ISL_8298482 | 23/08/2021 | Africa / Tunisia / Tunis | Human | Random | Female | 56 | unknown | unknown | Random | AY.122 | GK |
| hCoV-19/Tunisia/F-1874/2021 | EPI_ISL_8298481 | 23/08/2021 | Africa / Tunisia / Tunis | Human | Random | Female | 34 | unknown | unknown | Random | AY.122 | GK |
| hCoV-19/Tunisia/F-1871/2021 | EPI_ISL_8298480 | 23/08/2021 | Africa / Tunisia / Tunis | Human | Random | Female | 40 | unknown | unknown | Random | AY.122 | GK |
| hCoV-19/Tunisia/F-1856/2021 | EPI_ISL_8298479 | 23/08/2021 | Africa / Tunisia / Tunis | Human | Random | Male | 37 | unknown | unknown | Random | AY.122 | GK |
| hCoV-19/Tunisia/F-1851/2021 | EPI_ISL_8298477 | 23/08/2021 | Africa / Tunisia / Tunis | Human | Random | Female | 47 | unknown | unknown | Random | AY.122 | GK |
| hCoV-19/Tunisia/F-1697/2021 | EPI_ISL_8298474 | 21/08/2021 | Africa / Tunisia / Tunis | Human | Random | Female | 38 | unknown | unknown | Random | AY.122 | GK |
| hCoV-19/Tunisia/F-1342/2021 | EPI_ISL_8298468 | 16/08/2021 | Africa / Tunisia / Tunis | Human | Random | Female | 54 | unknown | unknown | Random | AY.122 | GK |
| hCoV-19/Tunisia/F-1237/2021 | EPI_ISL_8298466 | 14/08/2021 | Africa / Tunisia / Tunis | Human | Random | Female | 16 | unknown | unknown | Random | AY.122 | GH |
| hCoV-19/Tunisia/F-1211/2021 | EPI_ISL_8298465 | 14/08/2021 | Africa / Tunisia / Tunis | Human | Random | Male | 17 | unknown | unknown | Random | AY.122 | GK |
| hCoV-19/Tunisia/F-1167/2021 | EPI_ISL_8298463 | 13/08/2021 | Africa / Tunisia / Tunis | Human | Random | Female | 38 | unknown | unknown | Random | AY.122 | GK |
| hCoV-19/Tunisia/F-1128/2021 | EPI_ISL_8298461 | 12/08/2021 | Africa / Tunisia / Tunis | Human | Random | Male | 16 | unknown | unknown | Random | AY.122 | GK |
| hCoV-19/Tunisia/F-1068/2021 | EPI_ISL_8298460 | 11/08/2021 | Africa / Tunisia / Tunis | Human | Random | Female | 26 | unknown | unknown | Random | AY.122 | GK |
| hCoV-19/Tunisia/F-0985/2021 | EPI_ISL_8298459 | 11/08/2021 | Africa / Tunisia / Tunis | Human | Random | Male | 45 | unknown | unknown | Random | AY.122 | GK |
| hCoV-19/Tunisia/F-0982/2021 | EPI_ISL_8298458 | 11/08/2021 | Africa / Tunisia / Tunis | Human | Random | Male | 44 | unknown | unknown | Random | AY.122 | GK |
| hCoV-19/Tunisia/F-0980/2021 | EPI_ISL_8298457 | 11/08/2021 | Africa / Tunisia / Tunis | Human | Random | Female | 62 | unknown | unknown | Random | AY.122 | GK |
| hCoV-19/Tunisia/F-0971/2021 | EPI_ISL_8298456 | 10/08/2021 | Africa / Tunisia / Tunis | Human | Random | Male | 37 | unknown | unknown | Random | AY.122 | GK |
| hCoV-19/Tunisia/F-0957/2021 | EPI_ISL_8298455 | 09/08/2021 | Africa / Tunisia / Tunis | Human | Random | Female | 49 | unknown | unknown | Random | AY.122 | GK |
| hCoV-19/Tunisia/F-0797/2021 | EPI_ISL_8298453 | 06/08/2021 | Africa / Tunisia / Tunis | Human | Random | Male | 21 | unknown | unknown | Random | AY.122 | GK |
| hCoV-19/Tunisia/F-0679/2021 | EPI_ISL_8298452 | 05/08/2021 | Africa / Tunisia / Tunis | Human | Random | Female | 30 | unknown | unknown | Random | AY.122 | GK |
| hCoV-19/Tunisia/F-0512/2021 | EPI_ISL_8298451 | 03/08/2021 | Africa / Tunisia / Tunis | Human | Random | Male | 35 | unknown | unknown | Random | AY.122 | GK |
| hCoV-19/Tunisia/F-0491/2021 | EPI_ISL_8298450 | 03/08/2021 | Africa / Tunisia / Tunis | Human | Random | Female | 19 | unknown | unknown | Random | AY.122 | GK |
| hCoV-19/Tunisia/F-0428/2021 | EPI_ISL_8298448 | 03/08/2021 | Africa / Tunisia / Tunis | Human | Random | Female | 12 | unknown | unknown | Random | AY.122 | GK |
| hCoV-19/Tunisia/F-0398/2021 | EPI_ISL_8298447 | 03/08/2021 | Africa / Tunisia / Tunis | Human | Random | Female | 61 | unknown | unknown | Random | AY.122 | GK |
| hCoV-19/Tunisia/F-0382/2021 | EPI_ISL_8298446 | 02/08/2021 | Africa / Tunisia / Tunis | Human | Random | Female | 38 | unknown | unknown | Random | AY.122 | GK |
| hCoV-19/Tunisia/S-1038/2021 | EPI_ISL_8298441 | 14/09/2021 | Africa / Tunisia / Sousse | Human | Random | Male | 51 | unknown | unknown | Random | AY.34 | GK |
| hCoV-19/Tunisia/V-2116/2021 | EPI_ISL_8298439 | 24/09/2021 | Africa / Tunisia / Nabeul | Human | Random | Female | 33 | unknown | unknown | Random | AY.122 | GH |
| hCoV-19/Tunisia/S-1036/2021 | EPI_ISL_8298437 | 19/08/2021 | Africa / Tunisia / Nabeul | Human | Random | Female | 34 | unknown | unknown | Random | AY.122 | GK |
| hCoV-19/Tunisia/S-1035/2021 | EPI_ISL_8298436 | 07/08/2021 | Africa / Tunisia / Nabeul | Human | Random | Male | 49 | unknown | unknown | Random | AY.122 | GK |
| hCoV-19/Tunisia/F-1470/2021 | EPI_ISL_8298435 | 18/08/2021 | Africa / Tunisia / Manouba | Human | Random | Female | 46 | unknown | unknown | Random | AY.122 | GK |
| hCoV-19/Tunisia/V-1052/2021 | EPI_ISL_8298433 | 16/08/2021 | Africa / Tunisia / Kairouan | Human | Random | Female | 27 | unknown | unknown | Random | AY.122 | GK |
| hCoV-19/Tunisia/V-1932/2021 | EPI_ISL_8298431 | 15/09/2021 | Africa / Tunisia / Ben Arous | Human | Random | Female | 63 | unknown | unknown | Random | AY.122 | GK |
| hCoV-19/Tunisia/V-1853/2021 | EPI_ISL_8298430 | 13/09/2021 | Africa / Tunisia / Ben Arous | Human | Random | Male | 17 | unknown | unknown | Random | AY.122 | GK |
| hCoV-19/Tunisia/V-0793/2021 | EPI_ISL_8298429 | 04/08/2021 | Africa / Tunisia / Ben Arous | Human | Random | Female | 31 | unknown | unknown | Random | AY.122 | GK |
| hCoV-19/Tunisia/F-2908/2021 | EPI_ISL_8298428 | 06/09/2021 | Africa / Tunisia / Ben Arous | Human | Random | Male | 36 | unknown | unknown | Random | AY.122 | GK |
| hCoV-19/Tunisia/F-2831/2021 | EPI_ISL_8298427 | 04/09/2021 | Africa / Tunisia / Ben Arous | Human | Random | Female | 52 | unknown | unknown | Random | AY.122 | GK |
| hCoV-19/Tunisia/F-2382/2021 | EPI_ISL_8298426 | 30/08/2021 | Africa / Tunisia / Ben Arous | Human | Random | Female | 46 | unknown | unknown | Random | AY.122 | GK |
| hCoV-19/Tunisia/F-2106/2021 | EPI_ISL_8298424 | 26/08/2021 | Africa / Tunisia / Ben Arous | Human | Random | Male | 26 | unknown | unknown | Random | AY.122 | GK |
| hCoV-19/Tunisia/F-1640/2021 | EPI_ISL_8298423 | 20/08/2021 | Africa / Tunisia / Ben Arous | Human | Random | Male | 69 | unknown | unknown | Random | AY.122 | GK |
| hCoV-19/Tunisia/F-1352/2021 | EPI_ISL_8298422 | 16/08/2021 | Africa / Tunisia / Ben Arous | Human | Random | Female | 39 | unknown | unknown | Random | AY.122 | GK |
| hCoV-19/Tunisia/F-1340/2021 | EPI_ISL_8298421 | 16/08/2021 | Africa / Tunisia / Ben Arous | Human | Random | Female | 35 | unknown | unknown | Random | AY.122 | GK |
| hCoV-19/Tunisia/F-1184/2021 | EPI_ISL_8298420 | 14/08/2021 | Africa / Tunisia / Ben Arous | Human | Random | Female | 53 | unknown | unknown | Random | AY.122 | GK |
| hCoV-19/Tunisia/F-1182/2021 | EPI_ISL_8298419 | 14/08/2021 | Africa / Tunisia / Ben Arous | Human | Random | Male | 18 | unknown | unknown | Random | AY.122 | GK |
| hCoV-19/Tunisia/F-1091/2021 | EPI_ISL_8298418 | 12/08/2021 | Africa / Tunisia / Ben Arous | Human | Random | Female | 22 | unknown | unknown | Random | AY.122 | GK |
| hCoV-19/Tunisia/F-0811/2021 | EPI_ISL_8298417 | 07/08/2021 | Africa / Tunisia / Ben Arous | Human | Random | Female | 36 | unknown | unknown | Random | AY.122 | GK |
| hCoV-19/Tunisia/F-0689/2021 | EPI_ISL_8298416 | 05/08/2021 | Africa / Tunisia / Ben Arous | Human | Random | Female | 34 | unknown | unknown | Random | AY.122 | GK |
| hCoV-19/Tunisia/F-3571/2021 | EPI_ISL_8298415 | 18/09/2021 | Africa / Tunisia / Ariana | Human | Random | Male | 43 | unknown | unknown | Random | AY.122 | GK |
| hCoV-19/Tunisia/F-3530/2021 | EPI_ISL_8298414 | 17/09/2021 | Africa / Tunisia / Ariana | Human | Random | Female | 39 | unknown | unknown | Random | AY.122 | GK |
| hCoV-19/Tunisia/F-3263/2021 | EPI_ISL_8298413 | 13/09/2021 | Africa / Tunisia / Ariana | Human | Random | Female | 59 | unknown | unknown | Random | AY.122 | GK |
| hCoV-19/Tunisia/F-2997/2021 | EPI_ISL_8298412 | 07/09/2021 | Africa / Tunisia / Ariana | Human | Random | Male | 48 | unknown | unknown | Random | AY.122 | GK |
| hCoV-19/Tunisia/F-2916/2021 | EPI_ISL_8298411 | 06/09/2021 | Africa / Tunisia / Ariana | Human | Random | Female | 48 | unknown | unknown | Random | AY.122 | GK |
| hCoV-19/Tunisia/F-2682/2021 | EPI_ISL_8298410 | 02/09/2021 | Africa / Tunisia / Ariana | Human | Random | Male | 60 | unknown | unknown | Random | AY.122 | GK |
| hCoV-19/Tunisia/F-2470/2021 | EPI_ISL_8298409 | 31/08/2021 | Africa / Tunisia / Ariana | Human | Random | Male | 36 | unknown | unknown | Random | AY.122 | GK |
| hCoV-19/Tunisia/F-2464/2021 | EPI_ISL_8298408 | 31/08/2021 | Africa / Tunisia / Ariana | Human | Random | Male | 18 | unknown | unknown | Random | AY.122 | GK |
| hCoV-19/Tunisia/F-2101/2021 | EPI_ISL_8298407 | 26/08/2021 | Africa / Tunisia / Ariana | Human | Random | Female | 26 | unknown | unknown | Random | AY.122 | GK |
| hCoV-19/Tunisia/F-1952/2021 | EPI_ISL_8298406 | 25/08/2021 | Africa / Tunisia / Ariana | Human | Random | Male | 23 | unknown | unknown | Random | AY.122 | GK |
| hCoV-19/Tunisia/F-1902/2021 | EPI_ISL_8298405 | 24/08/2021 | Africa / Tunisia / Ariana | Human | Random | Female | 52 | unknown | unknown | Random | AY.122 | GK |
| hCoV-19/Tunisia/F-1691/2021 | EPI_ISL_8298402 | 21/08/2021 | Africa / Tunisia / Ariana | Human | Random | Male | 41 | unknown | unknown | Random | AY.122 | GK |
| hCoV-19/Tunisia/F-1686/2021 | EPI_ISL_8298400 | 21/08/2021 | Africa / Tunisia / Ariana | Human | Random | Male | 25 | unknown | unknown | Random | AY.122 | GK |
| hCoV-19/Tunisia/F-1270/2021 | EPI_ISL_8298398 | 18/08/2021 | Africa / Tunisia / Ariana | Human | Random | Male | 4 | unknown | unknown | Random | AY.122 | GK |
| hCoV-19/Tunisia/F-0978/2021 | EPI_ISL_8298396 | 11/08/2021 | Africa / Tunisia / Ariana | Human | Random | Female | 46 | unknown | unknown | Random | AY.122 | GK |
| hCoV-19/Tunisia/F-0821/2021 | EPI_ISL_8298394 | 07/08/2021 | Africa / Tunisia / Ariana | Human | Random | Female | 32 | unknown | unknown | Random | AY.122 | GK |
| hCoV-19/Tunisia/F-0817/2021 | EPI_ISL_8298392 | 07/08/2021 | Africa / Tunisia / Ariana | Human | Random | Female | 43 | unknown | unknown | Random | AY.122 | GK |
| hCoV-19/Tunisia/F-0708/2021 | EPI_ISL_8298391 | 06/08/2021 | Africa / Tunisia / Ariana | Human | Random | Female | 23 | unknown | unknown | Random | AY.122 | GK |
| hCoV-19/Tunisia/F-0672/2021 | EPI_ISL_8298390 | 05/08/2021 | Africa / Tunisia / Ariana | Human | Random | Female | 16 | unknown | unknown | Random | AY.122 | GK |
| hCoV-19/Tunisia/F-0422/2021 | EPI_ISL_8298388 | 03/08/2021 | Africa / Tunisia / Ariana | Human | Random | Male | 35 | unknown | unknown | Random | AY.122 | GK |
| hCoV-19/Tunisia/11-MHT_18/2020 | EPI_ISL_855570 | 15/09/2020 | Africa / Tunisia / Tunis / Tunis | Human | unknown | Male | 81 | unknown | unknown | unknown | B.1.177 | GV |
| hCoV-19/Tunisia/F-6654/2022 | EPI_ISL_16185798 | 01/03/2022 | Africa / Tunisia / Tunis | Human | Random | Female | 48 | unknown | unknown | Random | BA.1 | GRA |
| hCoV-19/Tunisia/F-6677/2022 | EPI_ISL_16185799 | 04/01/2022 | Africa / Tunisia / Tunis | Human | Random | Female | 58 | unknown | unknown | Random | BA.1.1 | GRA |
| hCoV-19/Tunisia/F-6784/2022 | EPI_ISL_16185800 | 04/01/2022 | Africa / Tunisia / Tunis | Human | Random | Female | 76 | unknown | unknown | Random | BA.1.1 | GRA |
| hCoV-19/Tunisia/F-7054/2022 | EPI_ISL_16185801 | 06/01/2022 | Africa / Tunisia / Tunis | Human | Random | Female | 61 | unknown | unknown | Random | BA.1.1 | GRA |
| hCoV-19/Tunisia/F-7088/2022 | EPI_ISL_16185802 | 06/01/2022 | Africa / Tunisia / Tunis | Human | Random | Male | 45 | unknown | unknown | Random | BA.1.1 | GRA |
| hCoV-19/Tunisia/F-7262/2022 | EPI_ISL_16185804 | 10/01/2022 | Africa / Tunisia / Tunis | Human | Random | Female | 42 | unknown | unknown | Random | BA.1.1 | GRA |
| hCoV-19/Tunisia/G-0944/2022 | EPI_ISL_16185829 | 22/01/2022 | Africa / Tunisia / Tunis | Human | Random | Male | 32 | unknown | unknown | Random | BA.1 | GRA |
| hCoV-19/Tunisia/F-7548/2022 | EPI_ISL_16185805 | 10/01/2022 | Africa / Tunisia / Tunis | Human | Random | Female | 30 | unknown | unknown | Random | BA.1.1.1 | GRA |
| hCoV-19/Tunisia/F-8151/2022 | EPI_ISL_16185807 | 12/01/2022 | Africa / Tunisia / Tunis | Human | Random | Female | 57 | unknown | unknown | Random | BA.1.1 | GRA |
| hCoV-19/Tunisia/F-7810/2022 | EPI_ISL_16185806 | 11/01/2022 | Africa / Tunisia / Tunis | Human | Random | Male | 13 | unknown | unknown | Random | BA.1.1 | GRA |
| hCoV-19/Tunisia/F-8800/2022 | EPI_ISL_16185813 | 14/01/2022 | Africa / Tunisia / Tunis | Human | Random | Female | 69 | unknown | unknown | Random | BA.1.1 | GRA |
| hCoV-19/Tunisia/F-8180/2022 | EPI_ISL_16185808 | 12/01/2022 | Africa / Tunisia / Tunis | Human | Random | Male | 62 | unknown | unknown | Random | BA.1 | GRA |
| hCoV-19/Tunisia/F-8398/2022 | EPI_ISL_16185809 | 13/01/2022 | Africa / Tunisia / Tunis | Human | Random | Male | 25 | unknown | unknown | Random | BA.1.1.1 | GRA |
| hCoV-19/Tunisia/G-0123/2022 | EPI_ISL_16185821 | 19/01/2022 | Africa / Tunisia / Tunis | Human | Random | Female | 43 | unknown | unknown | Random | BA.1.1 | GRA |
| hCoV-19/Tunisia/F-8426/2022 | EPI_ISL_16185811 | 13/01/2022 | Africa / Tunisia / Tunis | Human | Random | Female | 29 | unknown | unknown | Random | BA.1.17.2 | GRA |
| hCoV-19/Tunisia/F-8745/2022 | EPI_ISL_16185812 | 14/01/2022 | Africa / Tunisia / Tunis | Human | Random | Male | 46 | unknown | unknown | Random | BA.1.1 | GRA |
| hCoV-19/Tunisia/F-9008/2022 | EPI_ISL_16185814 | 15/01/2022 | Africa / Tunisia / Tunis | Human | Random | Male | 27 | unknown | unknown | Random | BA.1.1 | GRA |
| hCoV-19/Tunisia/F-9037/2022 | EPI_ISL_16185815 | 01/01/2022 | Africa / Tunisia / Tunis | Human | Random | Female | 21 | unknown | unknown | Random | BA.1.1 | GRA |
| hCoV-19/Tunisia/F-9322/2022 | EPI_ISL_16185816 | 17/01/2022 | Africa / Tunisia / Tunis | Human | Random | Female | 42 | unknown | unknown | Random | BA.1.1 | GRA |
| hCoV-19/Tunisia/G-0434/2022 | EPI_ISL_16185824 | 20/01/2022 | Africa / Tunisia / Tunis | Human | Random | Male | 64 | unknown | unknown | Random | BA.1.17.2 | GRA |
| hCoV-19/Tunisia/F-9390/2022 | EPI_ISL_16185817 | 17/01/2022 | Africa / Tunisia / Tunis | Human | Random | Female | 51 | unknown | unknown | Random | BA.1.1 | GRA |
| hCoV-19/Tunisia/F-9509/2022 | EPI_ISL_16185818 | 17/01/2022 | Africa / Tunisia / Tunis | Human | Random | Female | 27 | unknown | unknown | Random | BA.1.1 | GRA |
| hCoV-19/Tunisia/F-9803/2022 | EPI_ISL_16185819 | 18/01/2022 | Africa / Tunisia / Tunis | Human | Random | Male | 67 | unknown | unknown | Random | BA.1.1 | GRA |
| hCoV-19/Tunisia/G-0564/2022 | EPI_ISL_16185825 | 21/01/2022 | Africa / Tunisia / Tunis | Human | Random | Male | 51 | unknown | unknown | Random | BA.1.1 | GRA |
| hCoV-19/Tunisia/F-9806/2022 | EPI_ISL_16185820 | 18/01/2022 | Africa / Tunisia / Tunis | Human | Random | Female | 37 | unknown | unknown | Random | BA.1.1 | GRA |
| hCoV-19/Tunisia/G-0162/2022 | EPI_ISL_16185822 | 19/01/2022 | Africa / Tunisia / Tunis | Human | Random | Male | 61 | unknown | unknown | Random | BA.2 | GRA |
| hCoV-19/Tunisia/G-1088/2022 | EPI_ISL_16185830 | 24/01/2022 | Africa / Tunisia / Tunis | Human | Random | Male | 48 | unknown | unknown | Random | BA.2 | GRA |
| hCoV-19/Tunisia/G-0418/2022 | EPI_ISL_16185823 | 19/01/2022 | Africa / Tunisia / Tunis | Human | Random | Male | 52 | unknown | unknown | Random | BA.2 | GRA |
| hCoV-19/Tunisia/G-1160/2022 | EPI_ISL_16185832 | 25/01/2022 | Africa / Tunisia / Tunis | Human | Random | Male | 30 | unknown | unknown | Random | BA.1.1 | GRA |
| hCoV-19/Tunisia/G-0605/2022 | EPI_ISL_16185826 | 21/01/2022 | Africa / Tunisia / Tunis | Human | Random | Female | 29 | unknown | unknown | Random | BA.1.1 | GRA |
| hCoV-19/Tunisia/G-0833/2022 | EPI_ISL_16185827 | 22/01/2022 | Africa / Tunisia / Tunis | Human | Random | Female | 39 | unknown | unknown | Random | BA.2 | GRA |
| hCoV-19/Tunisia/G-0847/2022 | EPI_ISL_16185828 | 22/01/2022 | Africa / Tunisia / Tunis | Human | Random | Female | 55 | unknown | unknown | Random | BA.1.1 | GRA |
| hCoV-19/Tunisia/G-1119/2022 | EPI_ISL_16185831 | 24/01/2022 | Africa / Tunisia / Tunis | Human | Random | Male | 32 | unknown | unknown | Random | BA.1.1 | GRA |
| hCoV-19/Tunisia/G-1190/2022 | EPI_ISL_16185833 | 25/01/2022 | Africa / Tunisia / Tunis | Human | Random | Male | 38 | unknown | unknown | Random | BA.1.1 | GRA |
| hCoV-19/Tunisia/G-1207/2022 | EPI_ISL_16185834 | 25/01/2022 | Africa / Tunisia / Tunis | Human | Random | Female | 34 | unknown | unknown | Random | BA.1.1 | GRA |
| hCoV-19/Tunisia/G-1281/2022 | EPI_ISL_16185835 | 27/01/2022 | Africa / Tunisia / Tunis | Human | Random | Male | 52 | unknown | unknown | Random | BA.1.1 | GRA |
| hCoV-19/Tunisia/G-1462/2022 | EPI_ISL_16185838 | 28/01/2022 | Africa / Tunisia / Tunis | Human | Random | Male | 44 | unknown | unknown | Random | BA.1.1 | GRA |
| hCoV-19/Tunisia/G-1446/2022 | EPI_ISL_16185836 | 27/01/2022 | Africa / Tunisia / Tunis | Human | Random | Male | 37 | unknown | unknown | Random | BA.1.1 | GRA |
| hCoV-19/Tunisia/G-1447/2022 | EPI_ISL_16185837 | 28/01/2022 | Africa / Tunisia / Tunis | Human | Random | Female | 71 | unknown | unknown | Random | BA.1.18 | GRA |
| hCoV-19/Tunisia/G-1572/2022 | EPI_ISL_16185839 | 28/01/2022 | Africa / Tunisia / Tunis | Human | Random | Male | 64 | unknown | unknown | Random | BA.2 | GRA |
| hCoV-19/Tunisia/G-2668/2022 | EPI_ISL_16185842 | 15/02/2022 | Africa / Tunisia / Tunis | Human | Random | Female | 44 | unknown | unknown | Random | BA.1.1 (consensus call) | GRA |
| hCoV-19/Tunisia/G-2662/2022 | EPI_ISL_16185840 | 15/02/2022 | Africa / Tunisia / Tunis | Human | Random | Female | 55 | unknown | unknown | Random | BA.2 | GRA |
| hCoV-19/Tunisia/G-2665/2022 | EPI_ISL_16185841 | 15/02/2022 | Africa / Tunisia / Tunis | Human | Random | Female | 56 | unknown | unknown | Random | BA.2 | GRA |
| hCoV-19/Tunisia/G-2679/2022 | EPI_ISL_16185843 | 15/02/2022 | Africa / Tunisia / Tunis | Human | Random | Female | 50 | unknown | unknown | Random | BA.2 | GRA |
| hCoV-19/Tunisia/G-2687/2022 | EPI_ISL_16185844 | 15/02/2022 | Africa / Tunisia / Tunis | Human | Random | Male | 37 | unknown | unknown | Random | BA.2 | GRA |
| hCoV-19/Tunisia/G-2690/2022 | EPI_ISL_16185845 | 15/02/2022 | Africa / Tunisia / Tunis | Human | Random | Female | 25 | unknown | unknown | Random | BA.2 | GRA |
| hCoV-19/Tunisia/G-2694/2022 | EPI_ISL_16185846 | 16/02/2022 | Africa / Tunisia / Tunis | Human | Random | Male | 54 | unknown | unknown | Random | BA.2 | GRA |
| hCoV-19/Tunisia/G-2706/2022 | EPI_ISL_16185847 | 16/02/2022 | Africa / Tunisia / Tunis | Human | Random | Female | 27 | unknown | unknown | Random | BA.1.1 | GRA |
| hCoV-19/Tunisia/G-2760/2022 | EPI_ISL_16185852 | 17/02/2022 | Africa / Tunisia / Tunis | Human | Random | Female | 45 | unknown | unknown | Random | BA.2 | GRA |
| hCoV-19/Tunisia/G-2818/2022 | EPI_ISL_16185856 | 19/02/2022 | Africa / Tunisia / Tunis | Human | Random | Female | 36 | unknown | unknown | Random | BA.2 | GRA |
| hCoV-19/Tunisia/G-2719/2022 | EPI_ISL_16185848 | 16/02/2022 | Africa / Tunisia / Tunis | Human | Random | Female | 22 | unknown | unknown | Random | BA.2 | GRA |
| hCoV-19/Tunisia/G-2720/2022 | EPI_ISL_16185849 | 17/02/2022 | Africa / Tunisia / Tunis | Human | Random | Male | 59 | unknown | unknown | Random | BA.2.21 | GRA |
| hCoV-19/Tunisia/G-2745/2022 | EPI_ISL_16185850 | 17/02/2022 | Africa / Tunisia / Tunis | Human | Random | Female | 52 | unknown | unknown | Random | BA.2 | GRA |
| hCoV-19/Tunisia/G-2758/2022 | EPI_ISL_16185851 | 17/02/2022 | Africa / Tunisia / Tunis | Human | Random | Male | 45 | unknown | unknown | Random | BA.2 | GRA |
| hCoV-19/Tunisia/G-2778/2022 | EPI_ISL_16185854 | 17/02/2022 | Africa / Tunisia / Tunis | Human | Random | Female | 34 | unknown | unknown | Random | BA.2 | GRA |
| hCoV-19/Tunisia/G-2765/2022 | EPI_ISL_16185853 | 17/02/2022 | Africa / Tunisia / Tunis | Human | Random | Female | 46 | unknown | unknown | Random | BA.2 | GRA |
| hCoV-19/Tunisia/G-2966/2022 | EPI_ISL_16185863 | 24/02/2022 | Africa / Tunisia / Tunis | Human | Random | Male | 49 | unknown | unknown | Random | BA.2 | GRA |
| hCoV-19/Tunisia/G-3053/2022 | EPI_ISL_16185872 | 01/03/2022 | Africa / Tunisia / Tunis | Human | Random | Female | 35 | unknown | unknown | Random | BA.2 | GRA |
| hCoV-19/Tunisia/G-2801/2022 | EPI_ISL_16185855 | 18/02/2022 | Africa / Tunisia / Tunis | Human | Random | Female | 26 | unknown | unknown | Random | BA.2 | GRA |
| hCoV-19/Tunisia/G-2899/2022 | EPI_ISL_16185857 | 22/02/2022 | Africa / Tunisia / Tunis | Human | Random | Female | 16 | unknown | unknown | Random | BA.2 | GRA |
| hCoV-19/Tunisia/G-2904/2022 | EPI_ISL_16185858 | 22/02/2022 | Africa / Tunisia / Tunis | Human | Random | Female | 60 | unknown | unknown | Random | BA.1.1 | GRA |
| hCoV-19/Tunisia/G-2912/2022 | EPI_ISL_16185859 | 22/02/2022 | Africa / Tunisia / Tunis | Human | Random | Female | 54 | unknown | unknown | Random | BA.2.9 | GRA |
| hCoV-19/Tunisia/G-2956/2022 | EPI_ISL_16185861 | 24/02/2022 | Africa / Tunisia / Tunis | Human | Random | Female | 51 | unknown | unknown | Random | BA.1.1 | GRA |
| hCoV-19/Tunisia/G-2937/2022 | EPI_ISL_16185860 | 23/02/2022 | Africa / Tunisia / Tunis | Human | Random | Male | 25 | unknown | unknown | Random | BA.1.1 | GRA |
| hCoV-19/Tunisia/G-2967/2022 | EPI_ISL_16185864 | 24/02/2022 | Africa / Tunisia / Tunis | Human | Random | Female | 47 | unknown | unknown | Random | BA.2 | GRA |
| hCoV-19/Tunisia/G-2959/2022 | EPI_ISL_16185862 | 24/02/2022 | Africa / Tunisia / Tunis | Human | Random | Female | 53 | unknown | unknown | Random | BA.2 | GRA |
| hCoV-19/Tunisia/G-2970/2022 | EPI_ISL_16185865 | 24/02/2022 | Africa / Tunisia / Tunis | Human | Random | Female | 19 | unknown | unknown | Random | BA.2 | GRA |
| hCoV-19/Tunisia/G-2979/2022 | EPI_ISL_16185866 | 25/02/2022 | Africa / Tunisia / Tunis | Human | Random | Female | 28 | unknown | unknown | Random | BA.2 | GRA |
| hCoV-19/Tunisia/G-3005/2022 | EPI_ISL_16185867 | 26/02/2022 | Africa / Tunisia / Tunis | Human | Random | Female | 11 | unknown | unknown | Random | BA.2 | GRA |
| hCoV-19/Tunisia/G-3040/2022 | EPI_ISL_16185869 | 01/03/2022 | Africa / Tunisia / Tunis | Human | Random | Female | 47 | unknown | unknown | Random | BA.2 | GRA |
| hCoV-19/Tunisia/G-3031/2022 | EPI_ISL_16185868 | 28/02/2022 | Africa / Tunisia / Tunis | Human | Random | Male | 30 | unknown | unknown | Random | BA.2 | GRA |
| hCoV-19/Tunisia/G-3049/2022 | EPI_ISL_16185870 | 01/03/2022 | Africa / Tunisia / Tunis | Human | Random | Male | 45 | unknown | unknown | Random | BA.1.1 | GRA |
| hCoV-19/Tunisia/G-3051/2022 | EPI_ISL_16185871 | 01/03/2022 | Africa / Tunisia / Tunis | Human | Random | Male | 14 | unknown | unknown | Random | BA.2 | GRA |
| hCoV-19/Tunisia/G-3061/2022 | EPI_ISL_16185873 | 01/03/2022 | Africa / Tunisia / Tunis | Human | Random | Female | 52 | unknown | unknown | Random | BA.2 | GRA |
| hCoV-19/Tunisia/G-3213/2022 | EPI_ISL_16185880 | 08/03/2022 | Africa / Tunisia / Tunis | Human | Random | Female | 55 | unknown | unknown | Random | BA.2 | GRA |
| hCoV-19/Tunisia/G-3070/2022 | EPI_ISL_16185874 | 01/03/2022 | Africa / Tunisia / Tunis | Human | Random | Female | 32 | unknown | unknown | Random | BA.2 | GRA |
| hCoV-19/Tunisia/G-3247/2022 | EPI_ISL_16185882 | 10/03/2022 | Africa / Tunisia / Tunis | Human | Random | Male | 43 | unknown | unknown | Random | BA.2 | GRA |
| hCoV-19/Tunisia/G-3085/2022 | EPI_ISL_16185875 | 01/03/2022 | Africa / Tunisia / Tunis | Human | Random | Male | 51 | unknown | unknown | Random | BA.2 | GRA |
| hCoV-19/Tunisia/G-3089/2022 | EPI_ISL_16185876 | 02/03/2022 | Africa / Tunisia / Tunis | Human | Random | Female | 76 | unknown | unknown | Random | BA.2 | GRA |
| hCoV-19/Tunisia/G-3118/2022 | EPI_ISL_16185877 | 02/03/2022 | Africa / Tunisia / Tunis | Human | Random | Male | 32 | unknown | unknown | Random | BA.2 | GRA |
| hCoV-19/Tunisia/G-3161/2022 | EPI_ISL_16185878 | 05/03/2022 | Africa / Tunisia / Tunis | Human | Random | Female | 43 | unknown | unknown | Random | BA.2 | GRA |
| hCoV-19/Tunisia/G-3201/2022 | EPI_ISL_16185879 | 08/03/2022 | Africa / Tunisia / Tunis | Human | Random | Female | 68 | unknown | unknown | Random | BA.2 | GRA |
| hCoV-19/Tunisia/G-3215/2022 | EPI_ISL_16185881 | 08/03/2022 | Africa / Tunisia / Tunis | Human | Random | Female | 54 | unknown | unknown | Random | BA.1.1 | GRA |
| hCoV-19/Tunisia/G-3249/2022 | EPI_ISL_16185883 | 10/03/2022 | Africa / Tunisia / Tunis | Human | Random | Male | 41 | unknown | unknown | Random | BA.2 | GRA |
| hCoV-19/Tunisia/G-3252/2022 | EPI_ISL_16185884 | 10/03/2022 | Africa / Tunisia / Tunis | Human | Random | Male | 50 | unknown | unknown | Random | BA.2 | GRA |
| hCoV-19/Tunisia/G-3317/2022 | EPI_ISL_16185891 | 15/03/2022 | Africa / Tunisia / Tunis | Human | Random | Male | 61 | unknown | unknown | Random | BA.2 | GRA |
| hCoV-19/Tunisia/G-3257/2022 | EPI_ISL_16185885 | 10/03/2022 | Africa / Tunisia / Tunis | Human | Random | Male | 45 | unknown | unknown | Random | BA.2 | GRA |
| hCoV-19/Tunisia/G-3280/2022 | EPI_ISL_16185886 | 11/03/2022 | Africa / Tunisia / Tunis | Human | Random | Female | 32 | unknown | unknown | Random | BA.2 | GRA |
| hCoV-19/Tunisia/G-3328/2022 | EPI_ISL_16185892 | 16/03/2022 | Africa / Tunisia / Tunis | Human | Random | Male | 44 | unknown | unknown | Random | BA.2 | GRA |
| hCoV-19/Tunisia/S-2498/2022 | EPI_ISL_16185955 | 14/01/2022 | Africa / Tunisia / Manouba | Human | Random | Male | 35 | unknown | unknown | Random | BA.1.1 | GRA |
| hCoV-19/Tunisia/G-3281/2022 | EPI_ISL_16185887 | 11/03/2022 | Africa / Tunisia / Tunis | Human | Random | Male | 26 | unknown | unknown | Random | BA.2 | GRA |
| hCoV-19/Tunisia/G-3370/2022 | EPI_ISL_16185897 | 18/03/2022 | Africa / Tunisia / Tunis | Human | Random | Male | 60 | unknown | unknown | Random | BA.2.7 | GRA |
| hCoV-19/Tunisia/G-3294/2022 | EPI_ISL_16185888 | 14/03/2022 | Africa / Tunisia / Tunis | Human | Random | Female | 10 | unknown | unknown | Random | BA.2 | GRA |
| hCoV-19/Tunisia/G-3434/2022 | EPI_ISL_16185901 | 25/03/2022 | Africa / Tunisia / Tunis | Human | Random | Female | 25 | unknown | unknown | Random | BA.2 | GRA |
| hCoV-19/Tunisia/S-2317/2022 | EPI_ISL_16185947 | 11/01/2022 | Africa / Tunisia / Tunis | Human | Random | Male | 48 | unknown | unknown | Random | BA.1.1 | GRA |
| hCoV-19/Tunisia/G-3298/2022 | EPI_ISL_16185889 | 11/03/2022 | Africa / Tunisia / Tunis | Human | Random | Female | 70 | unknown | unknown | Random | BA.2 | GRA |
| hCoV-19/Tunisia/G-3301/2022 | EPI_ISL_16185890 | 14/03/2022 | Africa / Tunisia / Tunis | Human | Random | Male | 29 | unknown | unknown | Random | BA.2 | GRA |
| hCoV-19/Tunisia/G-3401/2022 | EPI_ISL_16185899 | 22/03/2022 | Africa / Tunisia / Tunis | Human | Random | Male | 49 | unknown | unknown | Random | BA.2 | GRA |
| hCoV-19/Tunisia/S-2218/2022 | EPI_ISL_16185942 | 12/01/2022 | Africa / Tunisia / Kairouan | Human | Random | Male | 66 | unknown | unknown | Random | B.1.1.529 | GRA |
| hCoV-19/Tunisia/G-3331/2022 | EPI_ISL_16185894 | 16/03/2022 | Africa / Tunisia / Tunis | Human | Random | Female | 38 | unknown | unknown | Random | BA.2.9 | GRA |
| hCoV-19/Tunisia/G-3372/2022 | EPI_ISL_16185898 | 18/03/2022 | Africa / Tunisia / Tunis | Human | Random | Male | 28 | unknown | unknown | Random | BA.2 | GRA |
| hCoV-19/Tunisia/S-2533/2022 | EPI_ISL_16185971 | 19/01/2022 | Africa / Tunisia / Sousse | Human | Random | Male | 37 | unknown | unknown | Random | BA.1.18 | GRA |
| hCoV-19/Tunisia/G-3334/2022 | EPI_ISL_16185895 | 16/03/2022 | Africa / Tunisia / Tunis | Human | Random | Male | 62 | unknown | unknown | Random | BA.2 | GRA |
| hCoV-19/Tunisia/G-3352/2022 | EPI_ISL_16185896 | 17/03/2022 | Africa / Tunisia / Tunis | Human | Random | Female | 35 | unknown | unknown | Random | BA.2 | GRA |
| hCoV-19/Tunisia/G-3402/2022 | EPI_ISL_16185900 | 22/03/2022 | Africa / Tunisia / Tunis | Human | Random | Male | 60 | unknown | unknown | Random | BA.2 | GRA |
| hCoV-19/Tunisia/G-4027/2022 | EPI_ISL_16185902 | 22/07/2022 | Africa / Tunisia / Tunis | Human | Random | Male | 29 | unknown | unknown | Random | BA.5.2 | GRA |
| hCoV-19/Tunisia/S-2135/2022 | EPI_ISL_16185934 | 10/01/2022 | Africa / Tunisia / Ben Arous | Human | Random | Female | 31 | unknown | unknown | Random | BA.1.1 | GRA |
| hCoV-19/Tunisia/S-2136/2022 | EPI_ISL_16185935 | 10/01/2022 | Africa / Tunisia / Ben Arous | Human | Random | Female | 61 | unknown | unknown | Random | BA.1 | GRA |
| hCoV-19/Tunisia/S-2305/2022 | EPI_ISL_16185945 | 12/01/2022 | Africa / Tunisia / Tunis | Human | Random | Female | 21 | unknown | unknown | Random | BA.1 | GRA |
| hCoV-19/Tunisia/S-2142/2022 | EPI_ISL_16185936 | 10/01/2022 | Africa / Tunisia / Ben Arous | Human | Random | Male | 70 | unknown | unknown | Random | BA.1.18 | GRA |
| hCoV-19/Tunisia/S-2164/2022 | EPI_ISL_16185938 | 10/01/2022 | Africa / Tunisia / Ben Arous | Human | Random | Female | 60 | unknown | unknown | Random | BA.1.17.2 | GRA |
| hCoV-19/Tunisia/S-2160/2022 | EPI_ISL_16185937 | 10/01/2022 | Africa / Tunisia / Ben Arous | Human | Random | Male | 98 | unknown | unknown | Random | BA.1.1 | GRA |
| hCoV-19/Tunisia/S-2234/2022 | EPI_ISL_16185944 | 12/01/2022 | Africa / Tunisia / Kairouan | Human | Random | Female | 58 | unknown | unknown | Random | BA.1.17.2 | GRA |
| hCoV-19/Tunisia/S-2318/2022 | EPI_ISL_16185948 | 11/01/2022 | Africa / Tunisia / Tunis | Human | Random | Female | 12 | unknown | unknown | Random | BA.1.1 | GRA |
| hCoV-19/Tunisia/S-2324/2022 | EPI_ISL_16185949 | 11/01/2022 | Africa / Tunisia / Tunis | Human | Random | Female | 41 | unknown | unknown | Random | BA.1.1 | GRA |
| hCoV-19/Tunisia/S-2332/2022 | EPI_ISL_16185950 | 11/01/2022 | Africa / Tunisia / Tunis | Human | Random | Male | 32 | unknown | unknown | Random | BA.1.18 | GRA |
| hCoV-19/Tunisia/S-2528/2022 | EPI_ISL_16185968 | 19/01/2022 | Africa / Tunisia / Sousse | Human | Random | Male | 13 | unknown | unknown | Random | BA.1.1 | GRA |
| hCoV-19/Tunisia/S-2348/2022 | EPI_ISL_16185951 | 11/01/2022 | Africa / Tunisia / Tunis | Human | Random | Female | 50 | unknown | unknown | Random | BA.1.1 | GRA |
| hCoV-19/Tunisia/S-2495/2022 | EPI_ISL_16185953 | 14/01/2022 | Africa / Tunisia / Manouba | Human | Random | Female | 45 | unknown | unknown | Random | BA.1.17.2 | GRA |
| hCoV-19/Tunisia/S-2496/2022 | EPI_ISL_16185954 | 14/01/2022 | Africa / Tunisia / Manouba | Human | Random | Male | 47 | unknown | unknown | Random | BA.1.1.1 | GRA |
| hCoV-19/Tunisia/S-2352/2022 | EPI_ISL_16185952 | 14/01/2022 | Africa / Tunisia / Tunis | Human | Random | Male | 18 | unknown | unknown | Random | BA.1.13 | GRA |
| hCoV-19/Tunisia/S-2505/2022 | EPI_ISL_16185958 | 14/01/2022 | Africa / Tunisia / Manouba | Human | Random | Female | 29 | unknown | unknown | Random | BA.1.15 | GRA |
| hCoV-19/Tunisia/S-2507/2022 | EPI_ISL_16185959 | 14/01/2022 | Africa / Tunisia / Manouba | Human | Random | Male | 24 | unknown | unknown | Random | BA.1.1 | GRA |
| hCoV-19/Tunisia/S-2500/2022 | EPI_ISL_16185956 | 14/01/2022 | Africa / Tunisia / Manouba | Human | Random | Female | 63 | unknown | unknown | Random | BA.1 | GRA |
| hCoV-19/Tunisia/S-2596/2022 | EPI_ISL_16185978 | 28/01/2022 | Africa / Tunisia / Kef | Human | Random | Male | 70 | unknown | unknown | Random | BA.2 | GRA |
| hCoV-19/Tunisia/S-2613/2022 | EPI_ISL_16185984 | 23/03/2022 | Africa / Tunisia / Tunis | Human | Random | Female | 38 | unknown | unknown | Random | BA.2.3 | GRA |
| hCoV-19/Tunisia/S-2616/2022 | EPI_ISL_16185987 | 23/03/2022 | Africa / Tunisia / Tunis | Human | Random | Female | 66 | unknown | unknown | Random | BA.2.3 | GRA |
| hCoV-19/Tunisia/S-2504/2022 | EPI_ISL_16185957 | 14/01/2022 | Africa / Tunisia / Manouba | Human | Random | Male | 48 | unknown | unknown | Random | BA.1.1 | GRA |
| hCoV-19/Tunisia/S-2509/2022 | EPI_ISL_16185960 | 14/01/2022 | Africa / Tunisia / Manouba | Human | Random | Female | 40 | unknown | unknown | Random | BA.1.1 | GRA |
| hCoV-19/Tunisia/S-2512/2022 | EPI_ISL_16185961 | 14/01/2022 | Africa / Tunisia / Manouba | Human | Random | Male | 27 | unknown | unknown | Random | BA.1 | GRA |
| hCoV-19/Tunisia/S-2532/2022 | EPI_ISL_16185970 | 19/01/2022 | Africa / Tunisia / Sousse | Human | Random | Female | 57 | unknown | unknown | Random | BA.2 | GRA |
| hCoV-19/Tunisia/S-2514/2022 | EPI_ISL_16185962 | 14/01/2022 | Africa / Tunisia / Manouba | Human | Random | Female | 25 | unknown | unknown | Random | BA.1.15 | GRA |
| hCoV-19/Tunisia/S-2523/2022 | EPI_ISL_16185965 | 19/01/2022 | Africa / Tunisia / Sousse | Human | Random | Male | unknown | unknown | unknown | Random | BA.1.1 | GRA |
| hCoV-19/Tunisia/S-2521/2022 | EPI_ISL_16185964 | 19/01/2022 | Africa / Tunisia / Sousse | Human | Random | Male | 16 | unknown | unknown | Random | BA.1 | GRA |
| hCoV-19/Tunisia/S-2526/2022 | EPI_ISL_16185966 | 19/01/2022 | Africa / Tunisia / Sousse | Human | Random | Male | 12 | unknown | unknown | Random | BA.1.1 | GRA |
| hCoV-19/Tunisia/S-2593/2022 | EPI_ISL_16185977 | 28/01/2022 | Africa / Tunisia / Kef | Human | Random | Female | 73 | unknown | unknown | Random | BA.2 | GRA |
| hCoV-19/Tunisia/S-2536/2022 | EPI_ISL_16185973 | 25/01/2022 | Africa / Tunisia / Kairouan | Human | Random | Female | 39 | unknown | unknown | Random | BA.1.1 | GRA |
| hCoV-19/Tunisia/S-2537/2022 | EPI_ISL_16185974 | 25/01/2022 | Africa / Tunisia / Kairouan | Human | Random | Male | 64 | unknown | unknown | Random | BA.1.17.2 | GRA |
| hCoV-19/Tunisia/S-2617/2022 | EPI_ISL_16185988 | 23/03/2022 | Africa / Tunisia / Tunis | Human | Random | Female | 62 | unknown | unknown | Random | BA.2.3 | GRA |
| hCoV-19/Tunisia/S-2539/2022 | EPI_ISL_16185975 | 25/01/2022 | Africa / Tunisia / Kairouan | Human | Random | Male | unknown | unknown | unknown | Random | BA.1.13 | GRA |
| hCoV-19/Tunisia/S-2612/2022 | EPI_ISL_16185983 | 23/03/2022 | Africa / Tunisia / Tunis | Human | Random | Female | 67 | unknown | unknown | Random | BA.2.3 | GRA |
| hCoV-19/Tunisia/S-2592/2022 | EPI_ISL_16185976 | 28/01/2022 | Africa / Tunisia / Kef | Human | Random | Female | 24 | unknown | unknown | Random | BA.2 | GRA |
| hCoV-19/Tunisia/S-2607/2022 | EPI_ISL_16185981 | 23/03/2022 | Africa / Tunisia / Tunis | Human | Random | Female | 57 | unknown | unknown | Random | BA.2 | GRA |
| hCoV-19/Tunisia/S-2601/2022 | EPI_ISL_16185979 | 26/02/2022 | Africa / Tunisia / Kef | Human | Random | Female | 29 | unknown | unknown | Random | BA.2 | GRA |
| hCoV-19/Tunisia/S-2606/2022 | EPI_ISL_16185980 | 23/03/2022 | Africa / Tunisia / Tunis | Human | Random | Female | unknown | unknown | unknown | Random | BA.2.3 | GRA |
| hCoV-19/Tunisia/S-2625/2022 | EPI_ISL_16185992 | 23/03/2022 | Africa / Tunisia / Tunis | Human | Random | Female | 37 | unknown | unknown | Random | BA.2 | GRA |
| hCoV-19/Tunisia/S-2634/2022 | EPI_ISL_16185998 | 23/03/2022 | Africa / Tunisia / Tunis | Human | Random | Female | 45 | unknown | unknown | Random | BA.2 | GRA |
| hCoV-19/Tunisia/S-2611/2022 | EPI_ISL_16185982 | 23/03/2022 | Africa / Tunisia / Tunis | Human | Random | Female | 84 | unknown | unknown | Random | BA.2 | GRA |
| hCoV-19/Tunisia/S-2614/2022 | EPI_ISL_16185985 | 23/03/2022 | Africa / Tunisia / Tunis | Human | Random | Female | 57 | unknown | unknown | Random | BA.2.3 | GRA |
| hCoV-19/Tunisia/S-2647/2022 | EPI_ISL_16186006 | 28/02/2022 | Africa / Tunisia / Kef | Human | Random | Male | 21 | unknown | unknown | Random | BA.2 | GRA |
| hCoV-19/Tunisia/S-2615/2022 | EPI_ISL_16185986 | 23/03/2022 | Africa / Tunisia / Tunis | Human | Random | Female | 65 | unknown | unknown | Random | BA.2.3 | GRA |
| hCoV-19/Tunisia/S-2619/2022 | EPI_ISL_16185989 | 23/03/2022 | Africa / Tunisia / Tunis | Human | Random | Female | 54 | unknown | unknown | Random | BA.2 | GRA |
| hCoV-19/Tunisia/S-2624/2022 | EPI_ISL_16185991 | 23/03/2022 | Africa / Tunisia / Tunis | Human | Random | Female | 66 | unknown | unknown | Random | BA.1.1 | GRA |
| hCoV-19/Tunisia/S-2620/2022 | EPI_ISL_16185990 | 23/03/2022 | Africa / Tunisia / Tunis | Human | Random | Female | 64 | unknown | unknown | Random | BA.2 | GRA |
| hCoV-19/Tunisia/S-2626/2022 | EPI_ISL_16185993 | 23/03/2022 | Africa / Tunisia / Tunis | Human | Random | Female | 61 | unknown | unknown | Random | BA.2 | GRA |
| hCoV-19/Tunisia/S-2630/2022 | EPI_ISL_16185996 | 23/03/2022 | Africa / Tunisia / Tunis | Human | Random | Female | 70 | unknown | unknown | Random | BA.2.40.1 | GRA |
| hCoV-19/Tunisia/S-2627/2022 | EPI_ISL_16185994 | 23/03/2022 | Africa / Tunisia / Tunis | Human | Random | Female | 66 | unknown | unknown | Random | BA.2 | GRA |
| hCoV-19/Tunisia/S-2629/2022 | EPI_ISL_16185995 | 23/03/2022 | Africa / Tunisia / Tunis | Human | Random | Female | 51 | unknown | unknown | Random | BA.2 | GRA |
| hCoV-19/Tunisia/S-2633/2022 | EPI_ISL_16185997 | 23/03/2022 | Africa / Tunisia / Tunis | Human | Random | Male | 62 | unknown | unknown | Random | BA.2.3 | GRA |
| hCoV-19/Tunisia/S-2635/2022 | EPI_ISL_16185999 | 23/03/2022 | Africa / Tunisia / Tunis | Human | Random | Female | 49 | unknown | unknown | Random | BA.2 | GRA |
| hCoV-19/Tunisia/S-2636/2022 | EPI_ISL_16186000 | 23/03/2022 | Africa / Tunisia / Tunis | Human | Random | Female | 60 | unknown | unknown | Random | BA.2 | GRA |
| hCoV-19/Tunisia/S-2638/2022 | EPI_ISL_16186002 | 23/03/2022 | Africa / Tunisia / Tunis | Human | Random | Male | 78 | unknown | unknown | Random | BA.2 | GRA |
| hCoV-19/Tunisia/S-2637/2022 | EPI_ISL_16186001 | 23/03/2022 | Africa / Tunisia / Tunis | Human | Random | Female | 83 | unknown | unknown | Random | BA.2.5 | GRA |
| hCoV-19/Tunisia/S-2686/2022 | EPI_ISL_16186007 | 21/02/2022 | Africa / Tunisia / Zaghouan | Human | Random | Male | 9 | unknown | unknown | Random | BA.2 | GRA |
| hCoV-19/Tunisia/S-2724/2022 | EPI_ISL_16186016 | 17/01/2022 | Africa / Tunisia / Tunis | Human | Random | Female | 14 | unknown | unknown | Random | BA.1.1.1 | GRA |
| hCoV-19/Tunisia/S-2640/2022 | EPI_ISL_16186003 | 23/03/2022 | Africa / Tunisia / Tunis | Human | Random | Male | 64 | unknown | unknown | Random | BA.2 | GRA |
| hCoV-19/Tunisia/S-2642/2022 | EPI_ISL_16186005 | 23/03/2022 | Africa / Tunisia / Tunis | Human | Random | Female | 53 | unknown | unknown | Random | BA.2.3 | GRA |
| hCoV-19/Tunisia/S-2718/2022 | EPI_ISL_16186013 | 22/01/2022 | Africa / Tunisia / Silina | Human | Random | Male | 8 | unknown | unknown | Random | BA.2 | GRA |
| hCoV-19/Tunisia/S-2687/2022 | EPI_ISL_16186008 | 28/02/2022 | Africa / Tunisia / Beja | Human | Random | Female | 9 | unknown | unknown | Random | BA.2 | GRA |
| hCoV-19/Tunisia/S-2696/2022 | EPI_ISL_16186009 | 24/01/2022 | Africa / Tunisia / Tunis | Human | Random | Female | 13 | unknown | unknown | Random | BA.1.1 | GRA |
| hCoV-19/Tunisia/S-2701/2022 | EPI_ISL_16186010 | 08/02/2022 | Africa / Tunisia / Tunis | Human | Random | Female | 11 | unknown | unknown | Random | BA.2 | GRA |
| hCoV-19/Tunisia/S-2708/2022 | EPI_ISL_16186011 | 11/02/2022 | Africa / Tunisia / Tunis | Human | Random | Female | 1 | unknown | unknown | Random | BA.1.1 | GRA |
| hCoV-19/Tunisia/S-2711/2022 | EPI_ISL_16186012 | 24/01/2022 | Africa / Tunisia / Tunis | Human | Random | Male | 14 | unknown | unknown | Random | BA.1.1 | GRA |
| hCoV-19/Tunisia/S-2721/2022 | EPI_ISL_16186014 | 22/01/2022 | Africa / Tunisia / Tunis | Human | Random | Male | 2 | unknown | unknown | Random | BA.2 | GRA |
| hCoV-19/Tunisia/S-2722/2022 | EPI_ISL_16186015 | 01/02/2022 | Africa / Tunisia / Nabeul | Human | Random | Male | 10 | unknown | unknown | Random | BA.2 | GRA |
| hCoV-19/Tunisia/S-2726/2022 | EPI_ISL_16186017 | 08/02/2022 | Africa / Tunisia / Tunis | Human | Random | Female | 1 | unknown | unknown | Random | BA.1.1 | GRA |
| hCoV-19/Tunisia/S-2775/2022 | EPI_ISL_16186025 | 07/03/2022 | Africa / Tunisia / Sfax | Human | Random | Female | 22 | unknown | unknown | Random | BA.2 | GRA |
| hCoV-19/Tunisia/V-5478/2022 | EPI_ISL_16186041 | 18/02/2022 | Africa / Tunisia / Tunis | Human | Random | Male | 80 | unknown | unknown | Random | BA.2 | GRA |
| hCoV-19/Tunisia/S-2727/2022 | EPI_ISL_16186018 | 17/01/2022 | Africa / Tunisia / Tunis | Human | Random | Female | 10 | unknown | unknown | Random | BA.1.1 | GRA |
| hCoV-19/Tunisia/S-2729/2022 | EPI_ISL_16186019 | 25/01/2022 | Africa / Tunisia / Jendouba | Human | Random | Female | 2 | unknown | unknown | Random | BA.2 | GRA |
| hCoV-19/Tunisia/V-5526/2022 | EPI_ISL_16186044 | 21/02/2022 | Africa / Tunisia / Tunis | Human | Random | Male | 59 | unknown | unknown | Random | BA.1.1 | GRA |
| hCoV-19/Tunisia/S-2766/2022 | EPI_ISL_16186020 | 17/03/2022 | Africa / Tunisia / Sousse | Human | Random | Male | 47 | unknown | unknown | Random | BA.2 | GRA |
| hCoV-19/Tunisia/S-2770/2022 | EPI_ISL_16186021 | 14/03/2022 | Africa / Tunisia / Sousse | Human | Random | Male | 1 | unknown | unknown | Random | BA.2.3 | GRA |
| hCoV-19/Tunisia/S-2783/2022 | EPI_ISL_16186029 | 25/03/2022 | Africa / Tunisia / Sfax | Human | Random | Female | 4 | unknown | unknown | Random | BA.1.1.1 | GRA |
| hCoV-19/Tunisia/S-2771/2022 | EPI_ISL_16186022 | 14/03/2022 | Africa / Tunisia / Sousse | Human | Random | Female | 26 | unknown | unknown | Random | BA.2 | GRA |
| hCoV-19/Tunisia/V-4875/2022 | EPI_ISL_16186031 | 24/03/2022 | Africa / Tunisia / Tunis | Human | Random | Male | 44 | unknown | unknown | Random | BA.1.1 | GRA |
| hCoV-19/Tunisia/S-2772/2022 | EPI_ISL_16186023 | 15/03/2022 | Africa / Tunisia / Sousse | Human | Random | Female | 2 months | unknown | unknown | Random | BA.2 | GRA |
| hCoV-19/Tunisia/S-2776/2022 | EPI_ISL_16186026 | 03/03/2022 | Africa / Tunisia / Sfax | Human | Random | Female | 22 | unknown | unknown | Random | BA.2 | GRA |
| hCoV-19/Tunisia/S-2782/2022 | EPI_ISL_16186028 | 11/03/2022 | Africa / Tunisia / Sfax | Human | Random | Female | 56 | unknown | unknown | Random | BA.2 | GRA |
| hCoV-19/Tunisia/S-2773/2022 | EPI_ISL_16186024 | 18/03/2022 | Africa / Tunisia / Sousse | Human | Random | Male | 82 | unknown | unknown | Random | BA.2 | GRA |
| hCoV-19/Tunisia/S-2777/2022 | EPI_ISL_16186027 | 04/03/2022 | Africa / Tunisia / Sfax | Human | Random | Male | unknown | unknown | unknown | Random | BA.2 | GRA |
| hCoV-19/Tunisia/V-4895/2022 | EPI_ISL_16186035 | 24/01/2022 | Africa / Tunisia / Tunis | Human | Random | Male | 27 | unknown | unknown | Random | BA.2 | GRA |
| hCoV-19/Tunisia/S-2794/2022 | EPI_ISL_16186030 | 23/03/2022 | Africa / Tunisia / Sfax | Human | Random | Female | 48 | unknown | unknown | Random | BA.2 | GRA |
| hCoV-19/Tunisia/V-5457/2022 | EPI_ISL_16186040 | 17/02/2022 | Africa / Tunisia / Tunis | Human | Random | Male | 25 | unknown | unknown | Random | BA.2 | GRA |
| hCoV-19/Tunisia/V-5625/2022 | EPI_ISL_16186049 | 25/02/2022 | Africa / Tunisia / Tunis | Human | Random | Female | 31 | unknown | unknown | Random | BA.2 | GRA |
| hCoV-19/Tunisia/V-4882/2022 | EPI_ISL_16186032 | 24/01/2022 | Africa / Tunisia / Tunis | Human | Random | Male | 32 | unknown | unknown | Random | BA.1.17.2 | GRA |
| hCoV-19/Tunisia/V-4884/2022 | EPI_ISL_16186033 | 24/01/2022 | Africa / Tunisia / Tunis | Human | Random | Female | 33 | unknown | unknown | Random | BA.2 | GRA |
| hCoV-19/Tunisia/V-4902/2022 | EPI_ISL_16186037 | 24/01/2022 | Africa / Tunisia / Tunis | Human | Random | Female | 45 | unknown | unknown | Random | BA.2 | GRA |
| hCoV-19/Tunisia/V-4888/2022 | EPI_ISL_16186034 | 24/01/2022 | Africa / Tunisia / Tunis | Human | Random | Female | 16 | unknown | unknown | Random | BA.1.18 | GRA |
| hCoV-19/Tunisia/V-4898/2022 | EPI_ISL_16186036 | 24/01/2022 | Africa / Tunisia / Tunis | Human | Random | Male | 72 | unknown | unknown | Random | BA.1.1 | GRA |
| hCoV-19/Tunisia/V-5520/2022 | EPI_ISL_16186043 | 21/02/2022 | Africa / Tunisia / Tunis | Human | Random | Female | 33 | unknown | unknown | Random | BA.1.1 | GRA |
| hCoV-19/Tunisia/V-5017/2022 | EPI_ISL_16186038 | 27/01/2022 | Africa / Tunisia / Tunis | Human | Random | Male | 29 | unknown | unknown | Random | BA.2 | GRA |
| hCoV-19/Tunisia/V-5519/2022 | EPI_ISL_16186042 | 21/02/2022 | Africa / Tunisia / Tunis | Human | Random | Male | 33 | unknown | unknown | Random | BA.2 | GRA |
| hCoV-19/Tunisia/V-5455/2022 | EPI_ISL_16186039 | 16/02/2022 | Africa / Tunisia / Tunis | Human | Random | Female | 37 | unknown | unknown | Random | BA.2 | GRA |
| hCoV-19/Tunisia/V-5540/2022 | EPI_ISL_16186045 | 21/02/2022 | Africa / Tunisia / Tunis | Human | Random | Male | 33 | unknown | unknown | Random | BA.2 | GRA |
| hCoV-19/Tunisia/V-5541/2022 | EPI_ISL_16186046 | 21/02/2022 | Africa / Tunisia / Tunis | Human | Random | Female | 26 | unknown | unknown | Random | BA.2 | GRA |
| hCoV-19/Tunisia/V-5542/2022 | EPI_ISL_16186047 | 21/02/2022 | Africa / Tunisia / Tunis | Human | Random | Female | 28 | unknown | unknown | Random | BA.2 | GRA |
| hCoV-19/Tunisia/V-5565/2022 | EPI_ISL_16186048 | 23/02/2022 | Africa / Tunisia / Tunis | Human | Random | Male | 69 | unknown | unknown | Random | BA.2 | GRA |
| hCoV-19/Tunisia/V-5640/2022 | EPI_ISL_16186050 | 28/02/2022 | Africa / Tunisia / Tunis | Human | Random | Female | 26 | unknown | unknown | Random | BA.2.7 | GRA |
| hCoV-19/Tunisia/V-6929/2022 | EPI_ISL_16186065 | 11/08/2022 | Africa / Tunisia / Tunis | Human | Random | Female | 77 | unknown | unknown | Random | BA.5.2.44 | GRA |
| hCoV-19/Tunisia/G-3338/2022 | EPI_ISL_16377407 | 16/03/2022 | Africa / Tunisia / Tunis | Human | Random | Male | 68 | unknown | unknown | Random | BA.2 | GRA |
| hCoV-19/Tunisia/V-5661/2022 | EPI_ISL_16186051 | 02/03/2022 | Africa / Tunisia / Tunis | Human | Random | Female | 31 | unknown | unknown | Random | BA.2 | GRA |
| hCoV-19/Tunisia/V-5750/2022 | EPI_ISL_16186059 | 09/03/2022 | Africa / Tunisia / Tunis | Human | Random | Female | 62 | unknown | unknown | Random | BA.2 | GRA |
| hCoV-19/Tunisia/V-5676/2022 | EPI_ISL_16186053 | 02/03/2022 | Africa / Tunisia / Tunis | Human | Random | Male | 19 | unknown | unknown | Random | BA.2 | GRA |
| hCoV-19/Tunisia/V-5680/2022 | EPI_ISL_16186054 | 03/03/2022 | Africa / Tunisia / Tunis | Human | Random | Male | 44 | unknown | unknown | Random | BA.2 | GRA |
| hCoV-19/Tunisia/G-4024/2022 | EPI_ISL_16377408 | 21/07/2022 | Africa / Tunisia / Tunis | Human | Random | Male | 1 | unknown | unknown | Random | BA.5.2 | GRA |
| hCoV-19/Tunisia/V-5593/2022 | EPI_ISL_16377413 | 24/02/2022 | Africa / Tunisia / Tunis | Human | Random | Male | 61 | unknown | unknown | Random | BA.1.1 (consensus call) | GRA |
| hCoV-19/Tunisia/V-5681/2022 | EPI_ISL_16186055 | 03/03/2022 | Africa / Tunisia / Tunis | Human | Random | Female | 38 | unknown | unknown | Random | BA.2 | GRA |
| hCoV-19/Tunisia/G-3048/2022 | EPI_ISL_16377406 | 01/03/2022 | Africa / Tunisia / Tunis | Human | Random | Female | 53 | unknown | unknown | Random | BA.2 | GRA |
| hCoV-19/Tunisia/V-5682/2022 | EPI_ISL_16186056 | 03/03/2022 | Africa / Tunisia / Tunis | Human | Random | Female | 39 | unknown | unknown | Random | BA.2 | GRA |
| hCoV-19/Tunisia/V-5699/2022 | EPI_ISL_16186057 | 03/03/2022 | Africa / Tunisia / Tunis | Human | Random | Male | 41 | unknown | unknown | Random | BA.2 | GRA |
| hCoV-19/Tunisia/G-1286/2022 | EPI_ISL_16377404 | 25/01/2022 | Africa / Tunisia / Tunis | Human | Random | Male | 37 | unknown | unknown | Random | BA.1.1 | GRA |
| hCoV-19/Tunisia/V-5746/2022 | EPI_ISL_16186058 | 09/03/2022 | Africa / Tunisia / Tunis | Human | Random | Male | 33 | unknown | unknown | Random | BA.2 | GRA |
| hCoV-19/Tunisia/V-5776/2022 | EPI_ISL_16186060 | 11/03/2022 | Africa / Tunisia / Tunis | Human | Random | Male | 64 | unknown | unknown | Random | BA.2 | GRA |
| hCoV-19/Tunisia/V-6869/2022 | EPI_ISL_16377417 | 01/08/2022 | Africa / Tunisia / Tunis | Human | Random | Male | 53 | unknown | unknown | Random | BA.5.2.1 | GRA |
| hCoV-19/Tunisia/V-5802/2022 | EPI_ISL_16186061 | 16/03/2022 | Africa / Tunisia / Tunis | Human | Random | Female | 60 | unknown | unknown | Random | BA.2 | GRA |
| hCoV-19/Tunisia/V-5845/2022 | EPI_ISL_16186062 | 22/03/2022 | Africa / Tunisia / Tunis | Human | Random | Female | 26 | unknown | unknown | Random | BA.2 | GRA |
| hCoV-19/Tunisia/V-5859/2022 | EPI_ISL_16186063 | 22/03/2022 | Africa / Tunisia / Tunis | Human | Random | Male | 66 | unknown | unknown | Random | BA.2 | GRA |
| hCoV-19/Tunisia/V-6802/2022 | EPI_ISL_16186064 | 22/07/2022 | Africa / Tunisia / Tunis | Human | Random | Male | 58 | unknown | unknown | Random | BA.5.2.20 | GRA |
| hCoV-19/Tunisia/G-2968/2022 | EPI_ISL_16377405 | 24/02/2022 | Africa / Tunisia / Tunis | Human | Random | Male | 59 | unknown | unknown | Random | BA.2 | GRA |
| hCoV-19/Tunisia/S-2881/2022 | EPI_ISL_16377412 | 06/04/2022 | Africa / Tunisia / Tunis | Human | Random | Female | unknown | unknown | unknown | Random | BA.2 | GRA |
| hCoV-19/Tunisia/V-6826/2022 | EPI_ISL_16377415 | 27/07/2022 | Africa / Tunisia / Tunis | Human | Random | Female | 26 | unknown | unknown | Random | BA.5.2 | GRA |
| hCoV-19/Tunisia/S-2713/2022 | EPI_ISL_16377411 | 04/02/2022 | Africa / Tunisia / Manouba | Human | Random | Female | 13 | unknown | unknown | Random | BA.2 | GRA |
| hCoV-19/Tunisia/V-6810/2022 | EPI_ISL_16377414 | 26/07/2022 | Africa / Tunisia / Tunis | Human | Random | Male | 79 | unknown | unknown | Random | BA.5.1.10 | GRA |
| hCoV-19/Tunisia/V-6834/2022 | EPI_ISL_16377416 | 28/07/2022 | Africa / Tunisia / Tunis | Human | Random | Male | 54 | unknown | unknown | Random | BA.5.2 | GRA |
| hCoV-19/Tunisia/V-6901/2022 | EPI_ISL_16377418 | 04/08/2022 | Africa / Tunisia / Tunis | Human | Random | Male | 23 | unknown | unknown | Random | BA.5.2.1 | GRA |
| hCoV-19/Tunisia/59879/2023 | EPI_ISL_18161875 | 15/08/2023 | Africa / Tunisia / Tunis | Human | Baseline surveillance | Female | 58 | unknown | unknown | Baseline surveillance | EG.4 | G |
| hCoV-19/Tunisia/59881/2023 | EPI_ISL_18161876 | 18/08/2023 | Africa / Tunisia / Tunis | Human | Baseline surveillance | Female | 36 | unknown | unknown | Baseline surveillance | EG.2 | GRA |
| hCoV-19/Tunisia/G-4671/2023 | EPI_ISL_18161884 | 26/06/2023 | Africa / Tunisia / Tunis | Human | Baseline surveillance | Male | 76 | unknown | unknown | Baseline surveillance | EG.4 | GRA |
| hCoV-19/Tunisia/D-3775/2021 | EPI_ISL_18161883 | 26/05/2021 | Africa / Tunisia / Tunis | Human | Baseline surveillance | Female | 42 | unknown | unknown | Baseline surveillance | B.1.525 | G |
| hCoV-19/Tunisia/V-7332/2023 | EPI_ISL_18161885 | 08/06/2023 | Africa / Tunisia / Nabeul | Human | Baseline surveillance | Male | 41 | unknown | unknown | Baseline surveillance | XBB.1.16 | GRA |
| hCoV-19/Tunisia/S-474/2021 | EPI_ISL_18161880 | 07/06/2021 | Africa / Tunisia / Medenine | Human | Baseline surveillance | Female | unknown | unknown | unknown | Baseline surveillance | B.1.525 | G |
| hCoV-19/Tunisia/S-484/2021 | EPI_ISL_18161881 | 07/06/2021 | Africa / Tunisia / Medenine | Human | Baseline surveillance | Female | 73 | unknown | unknown | Baseline surveillance | B.1 | G |
| hCoV-19/Tunisia/S-1651/2021 | EPI_ISL_18161882 | 09/12/2021 | Africa / Tunisia / Nabeul | Human | Baseline surveillance | Female | 1 | unknown | unknown | Baseline surveillance | B.1.640 | GH |
| hCoV-19/Tunisia/S-411/2021 | EPI_ISL_18161879 | 18/05/2021 | Africa / Tunisia / Medenine | Human | Baseline surveillance | Male | unknown | unknown | unknown | Baseline surveillance | B.1.525 | G |
| hCoV-19/Tunisia/M-4988/2021 | EPI_ISL_10101224 | 05/11/2021 | Africa / Tunisia | Human | unknown | Male | 75 | unknown | unknown | unknown | B.1 | G |
| hCoV-19/Tunisia/M-5569/2021 | EPI_ISL_10101253 | 10/11/2021 | Africa / Tunisia | Human | unknown | Female | 17 | unknown | unknown | unknown | B.1 | G |
| hCoV-19/Tunisia/H-9332/2021 | EPI_ISL_10101247 | 17/01/2021 | Africa / Tunisia | Human | unknown | Male | 89 | unknown | unknown | unknown | B.1 | G |
| hCoV-19/Tunisia/H-8036/2021 | EPI_ISL_10101231 | 12/01/2021 | Africa / Tunisia | Human | unknown | Female | 15 | unknown | unknown | unknown | AY.34 | GK |
| hCoV-19/Tunisia/H-8060/2021 | EPI_ISL_10101229 | 13/01/2021 | Africa / Tunisia | Human | unknown | Female | 64 | unknown | unknown | unknown | B.1 | G |
| hCoV-19/Tunisia/Z-3664/2021 | EPI_ISL_10101291 | 06/11/2021 | Africa / Tunisia | Human | unknown | Female | 1 month | unknown | unknown | unknown | B.1 | G |
| hCoV-19/Tunisia/34230/2021 | EPI_ISL_2909086 | 20/05/2021 | Africa / Tunisia / Ariana | Human | unknown | Female | 86 | unknown | unknown | unknown | Unassigned | G |
| hCoV-19/Tunisia/X-1742/2020 | EPI_ISL_10141514 | 25/08/2020 | Africa / Tunisia / Tunis | Human | unknown | Female | 27 | Asymptomatic | No | unknown | B.1.22 | GH |
| hCoV-19/Tunisia/F-5539/2021 | EPI_ISL_18168268 | 22/12/2021 | Africa / Tunisia / Tunis | Human | Baseline surveillance | Male | unknown | unknown | unknown | Baseline surveillance | Unassigned | GR |
| hCoV-19/Tunisia/V-7331/2023 | EPI_ISL_18168269 | 05/06/2023 | Africa / Tunisia / Tunis | Human | Baseline surveillance | Female | 47 | unknown | unknown | Baseline surveillance | XBB.1.16 | GRA |
| hCoV-19/Tunisia/G-4693/2023 | EPI_ISL_18168267 | 10/08/2023 | Africa / Tunisia / Tunis | Human | Baseline surveillance | Female | 43 | unknown | unknown | Baseline surveillance | XBB.1.28.1 | GRA |
| hCoV-19/Tunisia/S-421/2021 | EPI_ISL_18168266 | 18/05/2021 | Africa / Tunisia / Medenine | Human | Baseline surveillance | Male | 55 | unknown | unknown | Baseline surveillance | B.1 | GR |
| hCoV-19/Tunisia/TUN_ADAGE_4870/2020 | EPI_ISL_6973518 | 07/07/2020 | Africa / Tunisia / Sfax | Human | unknown | Female | 68 | Hospitalized | unknown | unknown | A | S |
| hCoV-19/Tunisia/TUN_ADAGE_4681/2020 | EPI_ISL_6972396 | 07/07/2020 | Africa / Tunisia / Sfax | Human | unknown | Male | 21 | Hospitalized | unknown | unknown | B.1.36 | GH |
| hCoV-19/Tunisia/TUN_ADAGE_EZ432/2020 | EPI_ISL_7054876 | 30/05/2020 | Africa / Tunisia / Monastir | Human | unknown | Female | 20 | Hospitalized | unknown | unknown | B.1 | GH |
| hCoV-19/Tunisia/SP-0382/2021 | EPI_ISL_2035988 | 08/03/2021 | Africa / Tunisia / Ben Arous | Human | unknown | Male | 49 | unknown | unknown | unknown | B.1.160 | GH |
| hCoV-19/Tunisia/TUN_ADAGE_EZ224/2020 | EPI_ISL_7054620 | 27/05/2020 | Africa / Tunisia / Monastir | Human | unknown | Male | 26 | Hospitalized | unknown | unknown | B.1.1 | GR |
| hCoV-19/Tunisia/202129459/2021 | EPI_ISL_2154331 | 26/04/2021 | Africa / Tunisia / Gafsa | Human | Baseline surveillance | Male | 61 | Live | unknown | Baseline surveillance | B.1.351.2 | GH |
| hCoV-19/Tunisia/29100/2021 | EPI_ISL_2153433 | 24/04/2021 | Africa / Tunisia / Nabeul / Bni Khiar | Human | Baseline surveillance | Male | 38 | Live | unknown | Baseline surveillance | B.1.351 | GH |
| hCoV-19/Tunisia/H-8926/2021 | EPI_ISL_10101223 | 15/01/2021 | Africa / Tunisia | Human | unknown | Male | 15 | unknown | unknown | unknown | B.1 | G |
| hCoV-19/Tunisia/M-8004/2021 | EPI_ISL_10101260 | 24/11/2021 | Africa / Tunisia | Human | unknown | Male | 83 | unknown | unknown | unknown | B.1.177 | GV |
| hCoV-19/Tunisia/M-5827/2021 | EPI_ISL_10101228 | 12/11/2021 | Africa / Tunisia | Human | unknown | Male | 69 | unknown | unknown | unknown | B.1.1 | GR |
| hCoV-19/Tunisia/H-1830/2021 | EPI_ISL_10101292 | 05/12/2021 | Africa / Tunisia | Human | unknown | Male | 57 | unknown | unknown | unknown | B.1.177 | GV |
| hCoV-19/Tunisia/P3-0826/2021 | EPI_ISL_10101303 | 05/04/2021 | Africa / Tunisia | Human | unknown | Male | 62 | unknown | unknown | unknown | B.1 | G |
| hCoV-19/Tunisia/M-5092/2021 | EPI_ISL_10101363 | 05/11/2021 | Africa / Tunisia | Human | unknown | Male | 31 | unknown | unknown | unknown | B.1.1.1 | GR |
| hCoV-19/Tunisia/S-1040/2021 | EPI_ISL_8298442 | 20/09/2021 | Africa / Tunisia / Sousse | Human | Random | Female | 28 | unknown | unknown | Random | AY.122 | GK |
| hCoV-19/Tunisia/Tunis_7266/2020 | EPI_ISL_450493 | 02/04/2020 | Africa / Tunisia | Human | unknown | Female | unknown | unknown | unknown | unknown | Unassigned | O |
| hCoV-19/Tunisia/Tunis7266/2020 | EPI_ISL_450482 | 02/04/2020 | Africa / Tunisia / Tunis | Human | unknown | unknown | unknown | unknown | unknown | unknown | Unassigned | O |
| hCoV-19/Tunisia/S-2520/2022 | EPI_ISL_16185963 | 19/01/2022 | Africa / Tunisia / Sousse | Human | Random | Male | unknown | unknown | unknown | Random | BA.1.1 | GRA |
| hCoV-19/Tunisia/S-2531/2022 | EPI_ISL_16185969 | 19/01/2022 | Africa / Tunisia / Sousse | Human | Random | Female | 26 | unknown | unknown | Random | BA.1.18 | GRA |
| hCoV-19/Tunisia/30390/2021 | EPI_ISL_2916656 | 30/04/2021 | Africa / Tunisia / Ariana | Human | unknown | Male | unknown | unknown | unknown | unknown | B.1 | G |
| hCoV-19/Tunisia/Q6575/2021 | EPI_ISL_1207287 | 07/02/2021 | Africa / Tunisia / Tunis | Human | unknown | Male | 17 | Live | unknown | unknown | A.27 | S |
| hCoV-19/Tunisia/32751/2021 | EPI_ISL_2915969 | 11/05/2021 | Africa / Tunisia / Ariana | Human | unknown | Male | 26 | unknown | unknown | unknown | Unassigned | G |
| hCoV-19/Tunisia/32990/2021 | EPI_ISL_2915294 | 11/05/2021 | Africa / Tunisia / Ariana | Human | unknown | Male | 68 | unknown | unknown | unknown | B.1.1.7 | GH |
| hCoV-19/Tunisia/33940/2021 | EPI_ISL_2911581 | 19/05/2021 | Africa / Tunisia / Ariana | Human | unknown | Female | 69 | unknown | unknown | unknown | B.1.1.7 | G |
| hCoV-19/Tunisia/34229/2021 | EPI_ISL_2910524 | 20/05/2021 | Africa / Tunisia / Ariana | Human | unknown | Male | 59 | unknown | unknown | unknown | B.1.1.7 | G |
| hCoV-19/Tunisia/TUN_ADAGE_4761/2020 | EPI_ISL_6972817 | 07/07/2020 | Africa / Tunisia / Sfax | Human | unknown | Female | 44 | Hospitalized | unknown | unknown | B.1.1 | GR |
| hCoV-19/Tunisia/A-0208/2021 | EPI_ISL_8298444 | 19/08/2021 | Africa / Tunisia / Tunis | Human | Random | unknown | 21 | unknown | unknown | Random | AY.122 | GH |
| hCoV-19/Tunisia/36107/2021 | EPI_ISL_2896981 | 28/05/2021 | Africa / Tunisia / Tunis | Human | unknown | Male | 47 | Live | unknown | unknown | B.1.351.3 | GH |
| hCoV-19/Tunisia/36108/2021 | EPI_ISL_2896943 | 28/05/2021 | Africa / Tunisia / Tunis | Human | unknown | Male | 35 | Live | unknown | unknown | Unassigned | GH |
| hCoV-19/Tunisia/B-0367/2021 | EPI_ISL_10101287 | 02/03/2021 | Africa / Tunisia | Human | unknown | Female | 67 | unknown | unknown | unknown | B.1.177 | GV |
| hCoV-19/Tunisia/ADAGE-24755/2020 | EPI_ISL_712062 | 08/11/2020 | Africa / Tunisia / Sfax | Human | unknown | unknown | unknown | unknown | unknown | unknown | B.1.428.2 | GH |
| hCoV-19/Tunisia/SP-0055/2021 | EPI_ISL_2035720 | 28/01/2021 | Africa / Tunisia / Tunis | Human | unknown | Male | 40 | unknown | unknown | unknown | B.1.160 | GH |
| hCoV-19/Tunisia/SP-0378/2021 | EPI_ISL_2035949 | 08/03/2021 | Africa / Tunisia / Ben Arous | Human | unknown | Female | 87 | unknown | unknown | unknown | B.1.160 | GH |
| hCoV-19/Tunisia/SP-0362/2021 | EPI_ISL_2035947 | 05/03/2021 | Africa / Tunisia / Tunks | Human | unknown | Male | unknown | unknown | unknown | unknown | B.1.525 | G |
| hCoV-19/Tunisia/SP-0347/2021 | EPI_ISL_2035946 | 03/03/2021 | Africa / Tunisia / Tunis | Human | unknown | Female | 77 | unknown | unknown | unknown | A.27 | S |
| hCoV-19/Tunisia/SP-0393/2021 | EPI_ISL_2036077 | 08/03/2021 | Africa / Tunisia / Tunis | Human | unknown | Female | 38 | unknown | unknown | unknown | B.1.1.7 | GRY |
| hCoV-19/Tunisia/SP-0157/2021 | EPI_ISL_2035942 | 07/02/2021 | Africa / Tunisia / Tunis | Human | unknown | Male | 17 | unknown | unknown | unknown | A.27 | S |
| hCoV-19/Tunisia/SP-0105/2021 | EPI_ISL_2035940 | 01/02/2021 | Africa / Tunisia / Tunis | Human | unknown | Male | 79 | unknown | unknown | unknown | B.1.160 | GH |
| hCoV-19/Tunisia/SP-0089/2021 | EPI_ISL_2035753 | 29/01/2021 | Africa / Tunisia / Tunis | Human | unknown | Male | 37 | unknown | unknown | unknown | B.1.160 | GH |
| hCoV-19/Tunisia/SP-0084/2021 | EPI_ISL_2035752 | 31/01/2021 | Africa / Tunisia / Tunis | Human | unknown | Male | unknown | unknown | unknown | unknown | B.1.160 | GH |
| hCoV-19/Tunisia/SP-0083/2021 | EPI_ISL_2035734 | 30/01/2021 | Africa / Tunisia / Tunis | Human | unknown | Female | unknown | unknown | unknown | unknown | B.1.177 | GV |
| hCoV-19/Tunisia/SP-0036/2021 | EPI_ISL_2035563 | 25/01/2021 | Africa / Tunisia / Tunis | Human | unknown | Female | 35 | unknown | unknown | unknown | B.1.177 | GV |
| hCoV-19/Tunisia/SP-0210/2021 | EPI_ISL_2035944 | 10/02/2021 | Africa / Tunisia / Tunis | Human | unknown | Male | 44 | unknown | unknown | unknown | B.1.177 | GV |
| hCoV-19/Tunisia/A-0028/2021 | EPI_ISL_16943972 | 25/05/2021 | Africa / Tunisia | Human | Random | unknown | unknown | unknown | unknown | Random | B.1.1.7 | GRY |
| hCoV-19/Tunisia/B-3362/2021 | EPI_ISL_16943984 | 18/03/2021 | Africa / Tunisia | Human | Random | unknown | unknown | Severe | unknown | Random | B.1.1.7 | GRY |
| hCoV-19/Tunisia/B-3367/2021 | EPI_ISL_16943986 | 18/03/2021 | Africa / Tunisia | Human | Random | unknown | unknown | Severe | unknown | Random | B.1.1.7 | GRY |
| hCoV-19/Tunisia/B-3364/2021 | EPI_ISL_16943985 | 18/03/2021 | Africa / Tunisia | Human | Random | unknown | unknown | Severe | unknown | Random | B.1.1.7 | GRY |
| hCoV-19/Tunisia/A-0109/2021 | EPI_ISL_16943978 | 25/05/2021 | Africa / Tunisia | Human | Random | unknown | unknown | unknown | unknown | Random | B.1.1.7 | GRY |
| hCoV-19/Tunisia/E-4451/2021 | EPI_ISL_10141455 | 05/07/2021 | Africa / Tunisia / Tunis | Human | unknown | Female | 36 | unknown | No | unknown | B.1 | GK |
| hCoV-19/Tunisia/Y-478/2020 | EPI_ISL_10141445 | 26/08/2020 | Africa / Tunisia / Jendouba | Human | unknown | Female | unknown | unknown | No | unknown | B.1.1 | GR |
| hCoV-19/Tunisia/7930/2023 | EPI_ISL_18220370 | 03/08/2023 | Africa / Tunisia / Tunis | Human | Baseline surveillance | Male | 2 months | unknown | unknown | Baseline surveillance | Unassigned | GR |
| hCoV-19/Tunisia/7593/2023 | EPI_ISL_18220369 | 20/07/2023 | Africa / Tunisia / Ariana | Human | Baseline surveillance | Female | 44 | unknown | unknown | Baseline surveillance | XBB.1.5.52 | GRA |
| hCoV-19/Tunisia/159/2023 | EPI_ISL_18220419 | 30/01/2023 | Africa / Tunisia / Ariana | Human | Baseline surveillance | Female | 44 | unknown | unknown | Baseline surveillance | XBB.1.11.1 | GRA |
| hCoV-19/Tunisia/7947/2023 | EPI_ISL_18220371 | 03/08/2023 | Africa / Tunisia / Tunis | Human | Baseline surveillance | Female | 30 | unknown | unknown | Baseline surveillance | XBB.2.3.11 | GRA |
| hCoV-19/Tunisia/186/2023 | EPI_ISL_18220421 | 03/02/2023 | Africa / Tunisia / Ariana | Human | Baseline surveillance | Female | 56 | unknown | unknown | Baseline surveillance | XBB.1.9.2 | GRA |
| hCoV-19/Tunisia/169/2023 | EPI_ISL_18220420 | 31/01/2023 | Africa / Tunisia / Ariana | Human | Baseline surveillance | Female | 48 | unknown | unknown | Baseline surveillance | XBB.1.9.2 | GRA |
| hCoV-19/Tunisia/8451/2022 | EPI_ISL_18220423 | 08/12/2022 | Africa / Tunisia / Ariana | Human | Baseline surveillance | Female | unknown | unknown | unknown | Baseline surveillance | BQ.1.1 | GRA |
| hCoV-19/Tunisia/156/2023 | EPI_ISL_18220418 | 30/01/2023 | Africa / Tunisia / Ariana | Human | Baseline surveillance | Female | 37 | unknown | unknown | Baseline surveillance | XBB.1.9.2 | GRA |
| hCoV-19/Tunisia/8453/2022 | EPI_ISL_18220424 | 08/12/2022 | Africa / Tunisia / Ariana | Human | Baseline surveillance | Male | unknown | unknown | unknown | Baseline surveillance | B.1 | G |
| hCoV-19/Tunisia/134/2023 | EPI_ISL_18220416 | 26/01/2023 | Africa / Tunisia / Ariana | Human | Baseline surveillance | Male | 67 | unknown | unknown | Baseline surveillance | B.1.1.529 | G |
| hCoV-19/Tunisia/153/2023 | EPI_ISL_18220417 | 28/01/2023 | Africa / Tunisia / Ariana | Human | Baseline surveillance | Female | 27 | unknown | unknown | Baseline surveillance | XBB.1.9.2 | GRA |
| hCoV-19/Tunisia/528/2023 | EPI_ISL_18220470 | 30/03/2023 | Africa / Tunisia / Ariana | Human | Baseline surveillance | Male | 70 | unknown | unknown | Baseline surveillance | FL.10 | GRA |
| hCoV-19/Tunisia/589/2023 | EPI_ISL_18220484 | 09/01/2023 | Africa / Tunisia / Ariana | Human | Baseline surveillance | Male | 81 | unknown | unknown | Baseline surveillance | XBB.1.5.14 | G |
| hCoV-19/Tunisia/199/2023 | EPI_ISL_18220463 | 04/02/2023 | Africa / Tunisia / Ariana | Human | Baseline surveillance | Female | 58 | unknown | unknown | Baseline surveillance | CM.8.1 | GRA |
| hCoV-19/Tunisia/665/2023 | EPI_ISL_18220485 | 28/01/2023 | Africa / Tunisia / Ariana | Human | Baseline surveillance | Female | 27 | unknown | unknown | Baseline surveillance | FL.2 | GRA |
| hCoV-19/Tunisia/298/2023 | EPI_ISL_18220464 | 18/02/2023 | Africa / Tunisia / Ariana | Human | Baseline surveillance | Female | 86 | unknown | unknown | Baseline surveillance | XBB.1.9.1 | GRA |
| hCoV-19/Tunisia/741/2023 | EPI_ISL_18220486 | 30/01/2023 | Africa / Tunisia / Ariana | Human | Baseline surveillance | Female | 37 | unknown | unknown | Baseline surveillance | XBB.1.5.14 | GRA |
| hCoV-19/Tunisia/301/2023 | EPI_ISL_18220465 | 18/02/2023 | Africa / Tunisia / Ariana | Human | Baseline surveillance | Female | 47 | unknown | unknown | Baseline surveillance | XBB | GRA |
| hCoV-19/Tunisia/760/2023 | EPI_ISL_18220487 | 30/01/2023 | Africa / Tunisia / Ariana | Human | Baseline surveillance | Female | 44 | unknown | unknown | Baseline surveillance | XBB.1.9.2 | GRA |
| hCoV-19/Tunisia/346/2023 | EPI_ISL_18220466 | 24/02/2023 | Africa / Tunisia / Ariana | Human | Baseline surveillance | Female | 25 | unknown | unknown | Baseline surveillance | XBB.1.5.14 | GRA |
| hCoV-19/Tunisia/835/2023 | EPI_ISL_18220488 | 31/01/2023 | Africa / Tunisia / Ariana | Human | Baseline surveillance | Female | 48 | unknown | unknown | Baseline surveillance | EG.4 | GRA |
| hCoV-19/Tunisia/430/2023 | EPI_ISL_18220467 | 10/03/2023 | Africa / Tunisia / Ariana | Human | Baseline surveillance | Male | 95 | unknown | unknown | Baseline surveillance | XBB.1.5.52 | GRA |
| hCoV-19/Tunisia/491/2023 | EPI_ISL_18220468 | 22/03/2023 | Africa / Tunisia / Ariana | Human | Baseline surveillance | Male | 81 | unknown | unknown | Baseline surveillance | XBB.1.9.2 | GRA |
| hCoV-19/Tunisia/518/2023 | EPI_ISL_18220469 | 28/03/2023 | Africa / Tunisia / Ariana | Human | Baseline surveillance | Male | 64 | unknown | unknown | Baseline surveillance | XBB.1.5.12 | GRA |
| hCoV-19/Tunisia/F-0943/2021 | EPI_ISL_8309616 | 07/08/2021 | Africa / Tunisia / Tunis | Human | Random | Female | 61 | unknown | unknown | Random | AY.122 | GK |
| hCoV-19/Tunisia/S-0759/2021 | EPI_ISL_10141516 | 29/06/2021 | Africa / Tunisia / Medenine | Human | unknown | Female | 26 | unknown | No | unknown | AY.122 | GK |
| hCoV-19/Tunisia/C-5773/2021 | EPI_ISL_10141517 | 26/04/2021 | Africa / Tunisia / Rades | Human | unknown | Female | 70 | Mild | No | unknown | B.1.1.7 | GRY |
| hCoV-19/Tunisia/11-MHT_10/2020 | EPI_ISL_855561 | 13/09/2020 | Africa / Tunisia / Tunis / Tunis | Human | unknown | Female | 83 | unknown | unknown | unknown | B.1.1 | GR |
| hCoV-19/Tunisia/X-3240/2020 | EPI_ISL_10141403 | 29/08/2020 | Africa / Tunisia / Ariana | Human | unknown | Male | 25 | Mild | No | unknown | B.1.617.2 | GK |
| hCoV-19/Tunisia/11-MHT_24/2020 | EPI_ISL_855572 | 15/09/2020 | Africa / Tunisia / Tunis / Tunis | Human | unknown | Male | 56 | unknown | unknown | unknown | B.1.177 | GV |
| hCoV-19/Tunisia/F-3760/2021 | EPI_ISL_8298548 | 21/09/2021 | Africa / Tunisia / Tunis | Human | Random | Male | 90 | unknown | unknown | Random | AY.122 | GK |
| hCoV-19/Tunisia/14670/2021 | EPI_ISL_1208403 | 09/02/2021 | Africa / Tunisia / Sousse | Human | unknown | Male | unknown | Live | unknown | unknown | A.27 | S |
| hCoV-19/Tunisia/Q8734/2021 | EPI_ISL_1197037 | 20/02/2021 | Africa / Tunisia / Tunis | Human | unknown | Male | 26 | Live | unknown | unknown | B.1.1.7 | GRY |
| hCoV-19/Tunisia/5516/2021 | EPI_ISL_1208399 | 02/02/2021 | Africa / Tunisia / Tunis | Human | unknown | Female | 34 | Live | unknown | unknown | A | O |
| hCoV-19/Tunisia/5509/2021 | EPI_ISL_1208398 | 02/02/2021 | Africa / Tunisia / Tunis | Human | unknown | Female | 26 | Live | unknown | unknown | A.27 | S |
| hCoV-19/Tunisia/20216575/2021 | EPI_ISL_1208160 | 07/02/2021 | Africa / Tunisia / Tunis | Human | unknown | Male | 70 | unknown | unknown | unknown | A.27 | S |
| hCoV-19/Tunisia/F-2969/2021 | EPI_ISL_8298510 | 07/09/2021 | Africa / Tunisia / Tunis | Human | Random | Male | 17 | unknown | unknown | Random | AY.122 | GK |
| hCoV-19/Tunisia/C-5376/2021 | EPI_ISL_10141511 | 23/04/2021 | Africa / Tunisia / Tunis | Human | unknown | Male | unknown | Hospitalized | No | unknown | B.1.1.7 | GRY |
| hCoV-19/Tunisia/S-1923-2/2021 | EPI_ISL_16185920 | 24/12/2021 | Africa / Tunisia / Sousse | Human | Random | Female | 55 | unknown | unknown | Random | AY.122 | GK |
| hCoV-19/Tunisia/S-1932-2/2021 | EPI_ISL_16185921 | 24/12/2021 | Africa / Tunisia / Sousse | Human | Random | Male | 50 | unknown | unknown | Random | AY.122 | GK |
| hCoV-19/Tunisia/S-1915-2/2021 | EPI_ISL_16185919 | 24/12/2021 | Africa / Tunisia / Sousse | Human | Random | Male | 39 | unknown | unknown | Random | AY.122 | GK |
| hCoV-19/Tunisia/38266QC/2021 | EPI_ISL_1138747 | 10/01/2021 | Africa / Tunisia / Sfax | Human | unknown | Male | 3 months | unknown | unknown | unknown | B.1.160 | GH |
| hCoV-19/Tunisia/NC9560/2020 | EPI_ISL_1137609 | 31/12/2020 | Africa / Tunisia / Sousse | Human | unknown | Female | 36 | unknown | unknown | unknown | B.1.160 | GH |
| hCoV-19/Tunisia/40795/2021 | EPI_ISL_1118923 | 28/01/2021 | Africa / Tunisia / Sfax | Human | unknown | Female | 31 | unknown | unknown | unknown | B.1.160 | GH |
| hCoV-19/Tunisia/34855/2020 | EPI_ISL_1118884 | 19/12/2020 | Africa / Tunisia / Sfax | Human | unknown | Female | 32 | unknown | unknown | unknown | B.1.160 | GH |
| hCoV-19/Tunisia/S-0781/2021 | EPI_ISL_10141424 | 30/06/2021 | Africa / Tunisia / Beja | Human | unknown | Male | 32 | unknown | No | unknown | B.1.1 | GR |
| hCoV-19/Tunisia/15833/2020 | EPI_ISL_1118675 | 21/09/2020 | Africa / Tunisia / Sfax | Human | unknown | Female | 32 | unknown | unknown | unknown | B.1.428.2 | GH |
| hCoV-19/Tunisia/33265/2021 | EPI_ISL_2914585 | 17/05/2021 | Africa / Tunisia / Ariana | Human | unknown | Female | 74 | unknown | unknown | unknown | B.1.1.7 | G |
| hCoV-19/Tunisia/37994/2021 | EPI_ISL_1116469 | 07/01/2021 | Africa / Tunisia / Sfax | Human | unknown | Female | 36 | unknown | unknown | unknown | B.1.160 | GH |
| hCoV-19/Tunisia/9066/2020 | EPI_ISL_1116468 | 22/08/2020 | Africa / Tunisia / Sfax | Human | unknown | Female | 36 | unknown | unknown | unknown | B.1 | GH |
| hCoV-19/Tunisia/35507/2020 | EPI_ISL_1116464 | 21/12/2020 | Africa / Tunisia / Sfax | Human | unknown | Female | 41 | Hospitalized | unknown | unknown | B.1.160 | GH |
| hCoV-19/Tunisia/11-MHT_12/2020 | EPI_ISL_855569 | 14/09/2020 | Africa / Tunisia / Tunis / Tunis | Human | unknown | Male | 58 | unknown | unknown | unknown | B.1.160 | GH |
| hCoV-19/Tunisia/11-MHT_6/2020 | EPI_ISL_855568 | 13/09/2020 | Africa / Tunisia / Tunis / Tunis | Human | unknown | Male | 30 | unknown | unknown | unknown | B.1.1 | GR |
| hCoV-19/Tunisia/11-MHT_5/2020 | EPI_ISL_855567 | 11/09/2020 | Africa / Tunisia / Tunis / Bouchoucha | Human | unknown | Male | 62 | unknown | unknown | unknown | B.1.1.241 | GR |
| hCoV-19/Tunisia/23-MHT_22/2020 | EPI_ISL_855565 | 15/09/2020 | Africa / Tunisia / Bizerte / Corniche | Human | unknown | Female | 58 | unknown | unknown | unknown | B.1.597 | GH |
| hCoV-19/Tunisia/43-MHT_19/2020 | EPI_ISL_855564 | 15/09/2020 | Africa / Tunisia / Sidi Bouzid / Sidi Bouzid | Human | unknown | Female | 48 | unknown | unknown | unknown | B.1.160 | GH |
| hCoV-19/Tunisia/11-MHT_17/2020 | EPI_ISL_855563 | 15/09/2020 | Africa / Tunisia / Tunis / Tunis | Human | unknown | Female | 65 | unknown | unknown | unknown | B.1.177 | GV |
| hCoV-19/Tunisia/23-MHT_15/2020 | EPI_ISL_855562 | 14/09/2020 | Africa / Tunisia / Bizerte / Cobbala | Human | unknown | Female | 31 | unknown | unknown | unknown | B.1.428.2 | GH |
| hCoV-19/Tunisia/11-MHT_8/2020 | EPI_ISL_855559 | 13/09/2020 | Africa / Tunisia / Tunis / Ben Arous | Human | unknown | Male | 43 | unknown | unknown | unknown | B.1.1.198 | GR |
| hCoV-19/Tunisia/11-MHT_7/2020 | EPI_ISL_855558 | 11/09/2020 | Africa / Tunisia / Tunis / Tunis | Human | unknown | Female | 33 | unknown | unknown | unknown | B.1.1.198 | GR |
| hCoV-19/Tunisia/23-MHT_14/2020 | EPI_ISL_855557 | 14/09/2020 | Africa / Tunisia / Bizerte / Cobbala | Human | unknown | Male | 42 | unknown | unknown | unknown | B.1.160 | GH |
| hCoV-19/Tunisia/475/2023 | EPI_ISL_18226708 | 18/03/2023 | Africa / Tunisia / Ariana | Human | Baseline surveillance | Male | 95 | unknown | unknown | Baseline surveillance | BA.2.10.1 | GR |
| hCoV-19/Tunisia/402/2023 | EPI_ISL_18226687 | 07/03/2023 | Africa / Tunisia / Ariana | Human | Baseline surveillance | Female | 58 | unknown | unknown | Baseline surveillance | XBB.1.9.2 | GRA |
| hCoV-19/Tunisia/314/2023 | EPI_ISL_18226701 | 21/02/2023 | Africa / Tunisia / Ariana | Human | Baseline surveillance | Female | 33 | unknown | unknown | Baseline surveillance | EG.4 | GR |
| hCoV-19/Tunisia/564/2023 | EPI_ISL_18226710 | 07/04/2023 | Africa / Tunisia / Ariana | Human | Baseline surveillance | Male | 43 | unknown | unknown | Baseline surveillance | XBB.1.9.3 | GRA |
| hCoV-19/Tunisia/399/2023 | EPI_ISL_18226704 | 07/03/2023 | Africa / Tunisia / Ariana | Human | Baseline surveillance | Male | 59 | unknown | unknown | Baseline surveillance | XBB | GR |
| hCoV-19/Tunisia/436/2023 | EPI_ISL_18226706 | 11/03/2023 | Africa / Tunisia / Ariana | Human | Baseline surveillance | Male | 70 | unknown | unknown | Baseline surveillance | BA.2 | GR |
| hCoV-19/Tunisia/310/2023 | EPI_ISL_18226700 | 20/02/2023 | Africa / Tunisia / Ariana | Human | Baseline surveillance | Female | 33 | unknown | unknown | Baseline surveillance | XBB.1.5 | GRA |
| hCoV-19/Tunisia/405/2023 | EPI_ISL_18226705 | 08/03/2023 | Africa / Tunisia / Ariana | Human | Baseline surveillance | Female | 37 | unknown | unknown | Baseline surveillance | XBB | GRA |
| hCoV-19/Tunisia/380/2023 | EPI_ISL_18226703 | 02/03/2023 | Africa / Tunisia / Ariana | Human | Baseline surveillance | Male | 68 | unknown | unknown | Baseline surveillance | Unassigned | GR |
| hCoV-19/Tunisia/560/2023 | EPI_ISL_18226709 | 06/04/2023 | Africa / Tunisia / Ariana | Human | Baseline surveillance | Male | 57 | unknown | unknown | Baseline surveillance | BA.2.10.1 | GR |
| hCoV-19/Tunisia/461/2023 | EPI_ISL_18226707 | 15/03/2023 | Africa / Tunisia / Ariana | Human | Baseline surveillance | Male | 74 | unknown | unknown | Baseline surveillance | EG.4 | G |
| hCoV-19/Tunisia/44/2023 | EPI_ISL_18226714 | 09/01/2023 | Africa / Tunisia / Ariana | Human | Baseline surveillance | Male | 81 | unknown | unknown | Baseline surveillance | BQ.1 | G |
| hCoV-19/Tunisia/8530/2022 | EPI_ISL_18226716 | 28/12/2022 | Africa / Tunisia / Ariana | Human | Baseline surveillance | Female | unknown | unknown | unknown | Baseline surveillance | BA.5.2.20 | GRA |
| hCoV-19/Tunisia/500327/2023 | EPI_ISL_18226721 | 09/08/2023 | Africa / Tunisia / Tunis | Human | Baseline surveillance | Female | 76 | unknown | unknown | Baseline surveillance | XBB.2.3.11 | GRA |
| hCoV-19/Tunisia/8508/2022 | EPI_ISL_18226715 | 20/12/2022 | Africa / Tunisia / Ariana | Human | Baseline surveillance | Female | unknown | unknown | unknown | Baseline surveillance | Unassigned | G |
| hCoV-19/Tunisia/500329/2023 | EPI_ISL_18226722 | 09/08/2023 | Africa / Tunisia / Tunis | Human | Baseline surveillance | Male | unknown | unknown | unknown | Baseline surveillance | EG.4 | GRA |
| hCoV-19/Tunisia/234687/2023 | EPI_ISL_18226720 | 31/07/2023 | Africa / Tunisia / Tunis | Human | Baseline surveillance | Male | 64 | unknown | unknown | Baseline surveillance | XBB.1.9.1 | GRA |
| hCoV-19/Tunisia/8270/2023 | EPI_ISL_18226842 | 17/08/2023 | Africa / Tunisia / Tunis | Human | Baseline surveillance | Male | 39 | unknown | unknown | Baseline surveillance | EG.2 | GRA |
| hCoV-19/Tunisia/8237/2023 | EPI_ISL_18226843 | 16/08/2023 | Africa / Tunisia / Tunis | Human | Baseline surveillance | Male | 18 months | unknown | unknown | Baseline surveillance | EG.5.1 | GRA |
| hCoV-19/Tunisia/8272/2023 | EPI_ISL_18226841 | 18/08/2023 | Africa / Tunisia / Nabeul | Human | Baseline surveillance | Male | unknown | unknown | unknown | Baseline surveillance | EG.4 | GRA |
| hCoV-19/Tunisia/8274/2023 | EPI_ISL_18226837 | 16/08/2023 | Africa / Tunisia / Nabeul | Human | Baseline surveillance | Male | unknown | unknown | unknown | Baseline surveillance | XBB.2.3 | GRA |
| hCoV-19/Tunisia/8259/2023 | EPI_ISL_18226836 | 17/08/2023 | Africa / Tunisia / Tunis | Human | Baseline surveillance | Male | unknown | unknown | unknown | Baseline surveillance | XBB.2.3.11 | GRA |
| hCoV-19/Tunisia/8456/2023 | EPI_ISL_18226834 | 23/08/2023 | Africa / Tunisia / Ariana | Human | Baseline surveillance | Female | 12 | unknown | unknown | Baseline surveillance | XBB.2.3.11 | GRA |
| hCoV-19/Tunisia/8214/2023 | EPI_ISL_18226839 | 15/08/2023 | Africa / Tunisia / Tunis | Human | Baseline surveillance | Female | 3 months | unknown | unknown | Baseline surveillance | EG.4 | GRA |
| hCoV-19/Tunisia/8327/2023 | EPI_ISL_18226840 | 18/08/2023 | Africa / Tunisia / Tunis | Human | Baseline surveillance | Female | unknown | unknown | unknown | Baseline surveillance | XBB.2.3.11 | GRA |
| hCoV-19/Tunisia/8215/2023 | EPI_ISL_18226838 | 15/08/2023 | Africa / Tunisia / Tunis | Human | Baseline surveillance | Male | 2 months | unknown | unknown | Baseline surveillance | EG.4 | GRA |
| hCoV-19/Tunisia/8326/2023 | EPI_ISL_18226833 | 19/08/2023 | Africa / Tunisia / Tunis | Human | Baseline surveillance | Male | unknown | unknown | unknown | Baseline surveillance | XBB.2.3.11 | GRA |
| hCoV-19/Tunisia/8289/2023 | EPI_ISL_18226832 | 18/08/2023 | Africa / Tunisia / Ariana | Human | Baseline surveillance | Female | 82 | unknown | unknown | Baseline surveillance | XBB.1.9.2 | GRA |
| hCoV-19/Tunisia/8519/2023 | EPI_ISL_18226835 | 25/08/2023 | Africa / Tunisia / Tunis | Human | Baseline surveillance | Female | unknown | unknown | unknown | Baseline surveillance | XBB.2.3.11 | GRA |
| hCoV-19/Tunisia/403/2023 | EPI_ISL_18227529 | 17/08/2023 | Africa / Tunisia / Sousse | Human | Baseline surveillance | Female | 27 | unknown | unknown | Baseline surveillance | XBB.2.3.11 | GRA |
| hCoV-19/Tunisia/388/2023 | EPI_ISL_18227528 | 15/08/2023 | Africa / Tunisia / Sousse | Human | Baseline surveillance | Male | 84 | unknown | unknown | Baseline surveillance | XBB.2.3.11 | GRA |
| hCoV-19/Tunisia/363/2023 | EPI_ISL_18227525 | 11/08/2023 | Africa / Tunisia / Sousse | Human | Baseline surveillance | Male | 16 | unknown | unknown | Baseline surveillance | XBB.2.3 | GRA |
| hCoV-19/Tunisia/458/2023 | EPI_ISL_18227533 | 23/08/2023 | Africa / Tunisia / Sousse | Human | Baseline surveillance | Male | 75 | unknown | unknown | Baseline surveillance | EG.4 | GRA |
| hCoV-19/Tunisia/374/2023 | EPI_ISL_18227527 | 12/08/2023 | Africa / Tunisia / Monastir | Human | Baseline surveillance | Male | unknown | unknown | unknown | Baseline surveillance | XBB.1.16 | GRA |
| hCoV-19/Tunisia/428/2023 | EPI_ISL_18227531 | 21/08/2023 | Africa / Tunisia / Sousse | Human | Baseline surveillance | Female | 58 | unknown | unknown | Baseline surveillance | XBB.2.3.11 | GRA |
| hCoV-19/Tunisia/459/2023 | EPI_ISL_18227534 | 24/08/2023 | Africa / Tunisia / Sousse | Human | Baseline surveillance | Female | 30 | unknown | unknown | Baseline surveillance | XBB.2.3.11 | GRA |
| hCoV-19/Tunisia/416/2023 | EPI_ISL_18227530 | 18/08/2023 | Africa / Tunisia / Sousse | Human | Baseline surveillance | Female | 48 | unknown | unknown | Baseline surveillance | XBB.2.3.11 | GRA |
| hCoV-19/Tunisia/434/2023 | EPI_ISL_18227532 | 21/08/2023 | Africa / Tunisia / Sousse | Human | Baseline surveillance | Female | 56 | unknown | unknown | Baseline surveillance | EG.1 | GRA |
| hCoV-19/Tunisia/483/2023 | EPI_ISL_18227535 | 26/08/2023 | Africa / Tunisia / Sousse | Human | Baseline surveillance | Female | 27 | unknown | unknown | Baseline surveillance | EG.1 | GRA |
| hCoV-19/Tunisia/373/2023 | EPI_ISL_18227526 | 12/08/2023 | Africa / Tunisia / Sousse | Human | Baseline surveillance | Male | 1 month | unknown | unknown | Baseline surveillance | XBB.1.16 | GRA |
| hCoV-19/Tunisia/59836/2023 | EPI_ISL_18227868 | 27/08/2023 | Africa / Tunisia / Tunis | Human | Baseline surveillance | Female | 68 | unkwown | unknown | Baseline surveillance | XBB.2.3.11 | G |
| hCoV-19/Tunisia/59897/2023 | EPI_ISL_18227869 | 24/08/2023 | Africa / Tunisia / Tunis | Human | Baseline surveillance | Male | 63 | unkwown | unknown | Baseline surveillance | XBB.2.3.11 | GRA |
| hCoV-19/Tunisia/8303/2022 | EPI_ISL_18220422 | 01/11/2022 | Africa / Tunisia / Ariana | Human | Baseline surveillance | Female | unknown | unknown | unknown | Baseline surveillance | Unassigned | G |
| hCoV-19/Tunisia/8517/2022 | EPI_ISL_18220425 | 27/12/2022 | Africa / Tunisia / Ariana | Human | Baseline surveillance | Female | unknown | unknown | unknown | Baseline surveillance | Unassigned | GR |
| hCoV-19/Tunisia/607/2023 | EPI_ISL_18226712 | 26/01/2023 | Africa / Tunisia / Ariana | Human | Baseline surveillance | Male | 67 | unknown | unknown | Baseline surveillance | Unassigned | GR |
| hCoV-19/Tunisia/361/2023 | EPI_ISL_18226702 | 27/02/2023 | Africa / Tunisia / Ariana | Human | Baseline surveillance | Female | 37 | unknown | unknown | Baseline surveillance | BA.2 | GRA |
| hCoV-19/Tunisia/8533/2022 | EPI_ISL_18226717 | 29/12/2022 | Africa / Tunisia / Ariana | Human | Baseline surveillance | Female | unknown | unknown | unknown | Baseline surveillance | Unassigned | G |
| hCoV-19/Tunisia/3833/2023 | EPI_ISL_17771482 | 16/03/2023 | Africa / Tunisia / Sfax | Human | Baseline surveillance | Male | 62 | unknown | unknown | Baseline surveillance | BA.2.3 | GR |
| hCoV-19/Tunisia/3604/2023 | EPI_ISL_17771481 | 16/03/2023 | Africa / Tunisia / Ben Arous | Human | Baseline surveillance | Male | 64 | unknown | unknown | Baseline surveillance | Unassigned | GR |
| hCoV-19/Tunisia/3600/2023 | EPI_ISL_17771480 | 16/03/2023 | Africa / Tunisia / Tunis | Human | Baseline surveillance | Female | 37 | unknown | unknown | Baseline surveillance | Unassigned | GR |
| hCoV-19/Tunisia/3592/2023 | EPI_ISL_17771479 | 16/03/2023 | Africa / Tunisia / Tunis | Human | Baseline surveillance | Female | 74 | unknown | unknown | Baseline surveillance | BA.2.10.1 | GR |
| hCoV-19/Tunisia/3589/2023 | EPI_ISL_17771478 | 16/03/2023 | Africa / Tunisia / Tunis | Human | Baseline surveillance | Female | 72 | unknown | unknown | Baseline surveillance | Unassigned | GR |
| hCoV-19/Tunisia/3582/2023 | EPI_ISL_17771477 | 16/03/2023 | Africa / Tunisia / Tunis | Human | Baseline surveillance | Female | 55 | unknown | unknown | Baseline surveillance | Unassigned | GR |
| hCoV-19/Tunisia/3580/2023 | EPI_ISL_17771476 | 16/03/2023 | Africa / Tunisia / Tunis | Human | Baseline surveillance | Female | 51 | unknown | unknown | Baseline surveillance | Unassigned | GR |
| hCoV-19/Tunisia/3328/2023 | EPI_ISL_17764926 | 10/03/2023 | Africa / Tunisia / Nabeul | Human | Baseline surveillance | Female | 29 | unknown | unknown | Baseline surveillance | Unassigned | GR |
| hCoV-19/Tunisia/3275/2023 | EPI_ISL_17764924 | 09/03/2023 | Africa / Tunisia / Tunis | Human | Baseline surveillance | Male | 70 | unknown | unknown | Baseline surveillance | EG.4 | GR |
| hCoV-19/Tunisia/3264/2023 | EPI_ISL_17764923 | 09/03/2023 | Africa / Tunisia / Kef | Human | Baseline surveillance | Female | 59 | unknown | unknown | Baseline surveillance | XBB.1.5.14 | GRA |
| hCoV-19/Tunisia/4812/2023 | EPI_ISL_17764824 | 19/04/2023 | Africa / Tunisia / Sfax | Human | Baseline surveillance | Female | unknown | unknown | unknown | Baseline surveillance | Unassigned | G |
| hCoV-19/Tunisia/5024/2023 | EPI_ISL_17764815 | 28/04/2023 | Africa / Tunisia / Mahdia | Human | Baseline surveillance | Male | 42 | unknown | unknown | Baseline surveillance | Unassigned | G |
| hCoV-19/Tunisia/4659/2023 | EPI_ISL_17764812 | 14/04/2023 | Africa / Tunisia / Tunis | Human | Baseline surveillance | Male | 1 | unknown | unknown | Baseline surveillance | EG.4 | GRA |
| hCoV-19/Tunisia/5010/2023 | EPI_ISL_17764798 | 28/04/2023 | Africa / Tunisia / Gabes | Human | Baseline surveillance | Male | 50 | unknown | unknown | Baseline surveillance | EG.4 | GRA |
| hCoV-19/Tunisia/4800/2023 | EPI_ISL_17764797 | 19/04/2023 | Africa / Tunisia / Ariana | Human | Baseline surveillance | Female | 72 | unknown | unknown | Baseline surveillance | XBB.1.5.14 | G |
| hCoV-19/Tunisia/3848/2023 | EPI_ISL_17764794 | 24/03/2023 | Africa / Tunisia / Sfax | Human | Baseline surveillance | Female | 70 | unknown | unknown | Baseline surveillance | EG.4 | GRA |
| hCoV-19/Tunisia/3834/2023 | EPI_ISL_17764793 | 24/03/2023 | Africa / Tunisia / Bizerte | Human | Baseline surveillance | Male | 19 | unknown | unknown | Baseline surveillance | EG.4 | GRA |
| hCoV-19/Tunisia/4160/2023 | EPI_ISL_17764792 | 24/03/2023 | Africa / Tunisia / Medenine | Human | Baseline surveillance | Male | 55 | unknown | unknown | Baseline surveillance | BN.1.4.2 | GRA |
| hCoV-19/Tunisia/3327/2023 | EPI_ISL_17764155 | 10/03/2023 | Africa / Tunisia / Nabeul | Human | Baseline surveillance | Male | 83 | unknown | unknown | Baseline surveillance | XBB.1.22.1 | GRA |
| hCoV-19/Tunisia/3208/2023 | EPI_ISL_17764151 | 09/03/2023 | Africa / Tunisia / Ariana | Human | Baseline surveillance | Female | 26 | unknown | unknown | Baseline surveillance | Unassigned | GR |
| hCoV-19/Tunisia/3198/2023 | EPI_ISL_17764150 | 09/03/2023 | Africa / Tunisia / Manouba | Human | Baseline surveillance | Female | 47 | unknown | unknown | Baseline surveillance | EG.4 | GRA |
| hCoV-19/Tunisia/3195/2023 | EPI_ISL_17764149 | 09/03/2023 | Africa / Tunisia / Sousse | Human | Baseline surveillance | Male | 80 | unknown | unknown | Baseline surveillance | B.1.1.7 | GR |
| hCoV-19/Tunisia/3184/2023 | EPI_ISL_17764148 | 09/03/2023 | Africa / Tunisia / Gabes | Human | Baseline surveillance | Female | 20 | unknown | unknown | Baseline surveillance | EG.4 | GRA |
| hCoV-19/Tunisia/4408/2023 | EPI_ISL_17764145 | 07/04/2023 | Africa / Tunisia / Sfax | Human | Baseline surveillance | Male | 75 | unknown | unknown | Baseline surveillance | Unassigned | G |
| hCoV-19/Tunisia/4181/2023 | EPI_ISL_17764143 | 31/03/2023 | Africa / Tunisia / Zaghouan | Human | Baseline surveillance | Male | 75 | unknown | unknown | Baseline surveillance | EG.4 | G |
| hCoV-19/Tunisia/3005/2023 | EPI_ISL_17762992 | 04/03/2023 | Africa / Tunisia / Zaghouan | Human | Baseline surveillance | Female | 54 | unknown | unknown | Baseline surveillance | Unassigned | GR |
| hCoV-19/Tunisia/2907/2023 | EPI_ISL_17762991 | 02/03/2023 | Africa / Tunisia / Nabeul | Human | Baseline surveillance | Male | 40 | unknown | unknown | Baseline surveillance | FL.2 | GRA |
| hCoV-19/Tunisia/2905/2023 | EPI_ISL_17762990 | 02/03/2023 | Africa / Tunisia / Tunis | Human | Baseline surveillance | Female | 14 | unknown | unknown | Baseline surveillance | Unassigned | GR |
| hCoV-19/Tunisia/2877/2023 | EPI_ISL_17762989 | 02/03/2023 | Africa / Tunisia / Sfax | Human | Baseline surveillance | Female | 67 | unknown | unknown | Baseline surveillance | Unassigned | GR |
| hCoV-19/Tunisia/2872/2023 | EPI_ISL_17762988 | 02/03/2023 | Africa / Tunisia / Gabes | Human | Baseline surveillance | Male | 66 | unknown | unknown | Baseline surveillance | Unassigned | G |
| hCoV-19/Tunisia/2859/2023 | EPI_ISL_17762987 | 02/03/2023 | Africa / Tunisia / Sousse | Human | Baseline surveillance | Female | 58 | unknown | unknown | Baseline surveillance | Unassigned | G |
| hCoV-19/Tunisia/2847/2023 | EPI_ISL_17762985 | 02/03/2023 | Africa / Tunisia / Sfax | Human | Baseline surveillance | Male | 36 | unknown | unknown | Baseline surveillance | BE.9 | GRA |
| hCoV-19/Tunisia/2666/2023 | EPI_ISL_17762984 | 24/02/2023 | Africa / Tunisia / Nabeul | Human | Baseline surveillance | Male | 53 | unknown | unknown | Baseline surveillance | BQ.1.1 | GRA |
| hCoV-19/Tunisia/2587/2023 | EPI_ISL_17762982 | 24/02/2023 | Africa / Tunisia / Ariana | Human | Baseline surveillance | Female | 75 | unknown | unknown | Baseline surveillance | Unassigned | G |
| hCoV-19/Tunisia/2400/2023 | EPI_ISL_17762981 | 17/02/2023 | Africa / Tunisia / Bizerte | Human | Baseline surveillance | Female | unknown | unknown | unknown | Baseline surveillance | Unassigned | GR |
| hCoV-19/Tunisia/2385/2023 | EPI_ISL_17762980 | 17/02/2023 | Africa / Tunisia / Sfax | Human | Baseline surveillance | Male | unknown | unknown | unknown | Baseline surveillance | Unassigned | G |
| hCoV-19/Tunisia/2378/2023 | EPI_ISL_17762868 | 17/02/2023 | Africa / Tunisia / Tunis | Human | Baseline surveillance | Male | 1 | unknown | unknown | Baseline surveillance | Unassigned | GR |
| hCoV-19/Tunisia/2331/2023 | EPI_ISL_17762867 | 16/02/2023 | Africa / Tunisia / Tunis | Human | Baseline surveillance | Female | 68 | unknown | unknown | Baseline surveillance | Unassigned | GR |
| hCoV-19/Tunisia/2320/2023 | EPI_ISL_17762865 | 16/02/2023 | Africa / Tunisia / Nabeul | Human | Baseline surveillance | Male | 20 | unknown | unknown | Baseline surveillance | Unassigned | GR |
| hCoV-19/Tunisia/2307/2023 | EPI_ISL_17762864 | 16/02/2023 | Africa / Tunisia / Nabeul | Human | Baseline surveillance | Male | 45 | unknown | unknown | Baseline surveillance | Unassigned | G |
| hCoV-19/Tunisia/2274/2023 | EPI_ISL_17762859 | 16/02/2023 | Africa / Tunisia / Mahdia | Human | Baseline surveillance | Male | 36 | unknown | unknown | Baseline surveillance | Unassigned | GR |
| hCoV-19/Tunisia/2269/2023 | EPI_ISL_17762858 | 16/02/2023 | Africa / Tunisia / Sfax | Human | Baseline surveillance | Male | 63 | unknown | unknown | Baseline surveillance | XBB.1.5 | GRA |
| hCoV-19/Tunisia/2253/2023 | EPI_ISL_17762857 | 16/02/2023 | Africa / Tunisia / Gabes | Human | Baseline surveillance | Male | 69 | unknown | unknown | Baseline surveillance | Unassigned | GR |
| hCoV-19/Tunisia/2252/2023 | EPI_ISL_17762856 | 16/02/2023 | Africa / Tunisia / Sfax | Human | Baseline surveillance | Male | 75 | unknown | unknown | Baseline surveillance | XBB.1.5.52 | GRA |
| hCoV-19/Tunisia/2243/2023 | EPI_ISL_17762855 | 16/02/2023 | Africa / Tunisia / Tunis | Human | Baseline surveillance | Female | 1 | unknown | unknown | Baseline surveillance | Unassigned | G |
| hCoV-19/Tunisia/2237/2023 | EPI_ISL_17762854 | 16/02/2023 | Africa / Tunisia / Sousse | Human | Baseline surveillance | Female | 61 | unknown | unknown | Baseline surveillance | Unassigned | GR |
| hCoV-19/Tunisia/2213/2023 | EPI_ISL_17762508 | 16/02/2023 | Africa / Tunisia / Ariana | Human | Baseline surveillance | Female | 46 | unknown | unknown | Baseline surveillance | Unassigned | GR |
| hCoV-19/Tunisia/IPT-S-2889/2022 | EPI_ISL_17731640 | 06/04/2022 | Africa / Tunisia / Gafsa | Human | Random | Male | 73 | unknown | unknown | Random | BA.2 | GRA |
| hCoV-19/Tunisia/IPT-S-2887/2022 | EPI_ISL_17731639 | 06/04/2022 | Africa / Tunisia / Tunis | Human | Random | Female | 59 | unknown | unknown | Random | BA.2 | GRA |
| hCoV-19/Tunisia/IPT-S-2886/2022 | EPI_ISL_17731638 | 06/04/2022 | Africa / Tunisia / Tunis | Human | Random | Female | unknown | unknown | unknown | Random | BA.2.3 | GRA |
| hCoV-19/Tunisia/IPT-S-2885/2022 | EPI_ISL_17731637 | 06/04/2022 | Africa / Tunisia / Tunis | Human | Random | Male | 44 | unknown | unknown | Random | BA.2.3 | GRA |
| hCoV-19/Tunisia/IPT-S-2884/2022 | EPI_ISL_17731636 | 06/04/2022 | Africa / Tunisia / Tunis | Human | Random | Male | unknown | unknown | unknown | Random | BA.2.32 | GRA |
| hCoV-19/Tunisia/IPT-S-2883/2022 | EPI_ISL_17731635 | 06/04/2022 | Africa / Tunisia / Tunis | Human | Random | Female | 51 | unknown | unknown | Random | BA.2 | GRA |
| hCoV-19/Tunisia/IPT-S-2877/2022 | EPI_ISL_17731634 | 06/04/2022 | Africa / Tunisia / Tunis | Human | Random | Female | 42 | unknown | unknown | Random | BA.2.65 | GRA |
| hCoV-19/Tunisia/IPT-S-2876/2022 | EPI_ISL_17731633 | 06/04/2022 | Africa / Tunisia / Tunis | Human | Random | Female | 53 | unknown | unknown | Random | BA.2 | GRA |
| hCoV-19/Tunisia/IPT-S-2873/2022 | EPI_ISL_17731632 | 06/04/2022 | Africa / Tunisia / Tunis | Human | Random | Female | 78 | unknown | unknown | Random | BA.2.57 | GRA |
| hCoV-19/Tunisia/IPT-S-2872/2022 | EPI_ISL_17731631 | 06/04/2022 | Africa / Tunisia / Tunis | Human | Random | Male | 62 | unknown | unknown | Random | BA.2 | GRA |
| hCoV-19/Tunisia/IPT-S-2871/2022 | EPI_ISL_17731630 | 06/04/2022 | Africa / Tunisia / Sfax | Human | Random | Female | 54 | unknown | unknown | Random | BA.2.3 | GRA |
| hCoV-19/Tunisia/IPT-S-2870/2022 | EPI_ISL_17731629 | 06/04/2022 | Africa / Tunisia / Sfax | Human | Random | Female | unknown | unknown | unknown | Random | BA.2 | GRA |
| hCoV-19/Tunisia/IPT-S-2869/2022 | EPI_ISL_17731628 | 06/04/2022 | Africa / Tunisia / Gafsa | Human | Random | Female | 73 | unknown | unknown | Random | BA.2 | GRA |
| hCoV-19/Tunisia/IPT-S-2868/2022 | EPI_ISL_17731627 | 06/04/2022 | Africa / Tunisia / Gafsa | Human | Random | Male | 81 | unknown | unknown | Random | BA.2 | GRA |
| hCoV-19/Tunisia/IPT-S-2867/2022 | EPI_ISL_17731626 | 06/04/2022 | Africa / Tunisia / Mahdia | Human | Random | Male | 87 | unknown | unknown | Random | BA.2 | GRA |
| hCoV-19/Tunisia/IPT-S-2866/2022 | EPI_ISL_17731625 | 06/04/2022 | Africa / Tunisia / Sfax | Human | Random | Male | 66 | unknown | unknown | Random | BA.2.57 | GRA |
| hCoV-19/Tunisia/IPT-S-2865/2022 | EPI_ISL_17731624 | 06/04/2022 | Africa / Tunisia / Sfax | Human | Random | Female | 75 | unknown | unknown | Random | BA.2 | GRA |
| hCoV-19/Tunisia/IPT-S-2864/2022 | EPI_ISL_17731623 | 06/04/2022 | Africa / Tunisia / Monastir | Human | Random | Female | 54 | unknown | unknown | Random | BA.2.3 | GRA |
| hCoV-19/Tunisia/IPT-S-2863/2022 | EPI_ISL_17731622 | 06/04/2022 | Africa / Tunisia / Gabes | Human | Random | Female | 61 | unknown | unknown | Random | BA.2 | GRA |
| hCoV-19/Tunisia/IPT-S-2862/2022 | EPI_ISL_17731621 | 06/04/2022 | Africa / Tunisia / Sfax | Human | Random | Male | 74 | unknown | unknown | Random | BA.2 | GRA |
| hCoV-19/Tunisia/IPT-S-2861/2022 | EPI_ISL_17731620 | 06/04/2022 | Africa / Tunisia / Bizerte | Human | Random | Female | 64 | unknown | unknown | Random | BA.2 | GRA |
| hCoV-19/Tunisia/IPT-S-2860/2022 | EPI_ISL_17731619 | 06/04/2022 | Africa / Tunisia / Gafsa | Human | Random | Female | 51 | unknown | unknown | Random | BA.2.3 | GRA |
| hCoV-19/Tunisia/IPT-S-2859/2022 | EPI_ISL_17731618 | 06/04/2022 | Africa / Tunisia / Silina | Human | Random | Male | 84 | unknown | unknown | Random | BA.2.40.1 | GRA |
| hCoV-19/Tunisia/IPT-S-2858/2022 | EPI_ISL_17731617 | 06/04/2022 | Africa / Tunisia / Tunis | Human | Random | Male | 66 | unknown | unknown | Random | BA.2 | GRA |
| hCoV-19/Tunisia/IPT-S-2857/2022 | EPI_ISL_17731616 | 06/04/2022 | Africa / Tunisia / Gafsa | Human | Random | Female | unknown | unknown | unknown | Random | BA.1.1.15 | GRA |
| hCoV-19/Tunisia/IPT-S-2856/2022 | EPI_ISL_17731615 | 06/04/2022 | Africa / Tunisia / Sfax | Human | Random | Male | unknown | unknown | unknown | Random | BA.2 | GRA |
| hCoV-19/Tunisia/IPT-S-2855/2022 | EPI_ISL_17731614 | 06/04/2022 | Africa / Tunisia / Tunis | Human | Random | Female | 51 | unknown | unknown | Random | BA.2 | GRA |
| hCoV-19/Tunisia/IPT-S-2854/2022 | EPI_ISL_17731613 | 06/04/2022 | Africa / Tunisia / Tunis | Human | Random | Female | 64 | unknown | unknown | Random | BA.2 | GRA |
| hCoV-19/Tunisia/IPT-S-2853/2022 | EPI_ISL_17731612 | 06/04/2022 | Africa / Tunisia / Tunis | Human | Random | Female | 62 | unknown | unknown | Random | BA.2 | GRA |
| hCoV-19/Tunisia/IPT-S-2851/2022 | EPI_ISL_17731611 | 06/04/2022 | Africa / Tunisia / Bizerte | Human | Random | Female | 53 | unknown | unknown | Random | BA.2.3 | GRA |
| hCoV-19/Tunisia/IPT-S-2850/2022 | EPI_ISL_17731610 | 06/04/2022 | Africa / Tunisia / Beja | Human | Random | Female | 58 | unknown | unknown | Random | BA.2 | GRA |
| hCoV-19/Tunisia/IPT-S-2849/2022 | EPI_ISL_17731609 | 06/04/2022 | Africa / Tunisia / Sidi Bouzid | Human | Random | Female | 58 | unknown | unknown | Random | BA.2 | GRA |
| hCoV-19/Tunisia/IPT-S-2847/2022 | EPI_ISL_17731608 | 06/04/2022 | Africa / Tunisia / Monastir | Human | Random | Female | 77 | unknown | unknown | Random | BA.2 | GRA |
| hCoV-19/Tunisia/IPT-S-2846/2022 | EPI_ISL_17731607 | 06/04/2022 | Africa / Tunisia / Monastir | Human | Random | Female | 42 | unknown | unknown | Random | BA.2 | GRA |
| hCoV-19/Tunisia/IPT-S-2845/2022 | EPI_ISL_17731606 | 06/04/2022 | Africa / Tunisia / Gafsa | Human | Random | Male | unknown | unknown | unknown | Random | BA.1.1.15 | GRA |
| hCoV-19/Tunisia/IPT-S-2842/2022 | EPI_ISL_17731605 | 06/04/2022 | Africa / Tunisia / Tunis | Human | Random | Female | 87 | unknown | unknown | Random | BA.2 | GRA |
| hCoV-19/Tunisia/IPT-S-2841/2022 | EPI_ISL_17731604 | 06/04/2022 | Africa / Tunisia / Kairouan | Human | Random | Female | 67 | unknown | unknown | Random | BA.2 | GRA |
| hCoV-19/Tunisia/IPT-S-2840/2022 | EPI_ISL_17731603 | 06/04/2022 | Africa / Tunisia / Tunis | Human | Random | Female | 75 | unknown | unknown | Random | BA.2.3 | GRA |
| hCoV-19/Tunisia/IPT-S-2839/2022 | EPI_ISL_17731602 | 06/04/2022 | Africa / Tunisia / Nabeul | Human | Random | Female | 56 | unknown | unknown | Random | BA.2.40.1 | GRA |
| hCoV-19/Tunisia/IPT-S-2838/2022 | EPI_ISL_17731601 | 06/04/2022 | Africa / Tunisia / Manouba | Human | Random | Male | 69 | unknown | unknown | Random | BA.2.40.1 | GRA |
| hCoV-19/Tunisia/IPT-S-2837/2022 | EPI_ISL_17731600 | 06/04/2022 | Africa / Tunisia / Nabeul | Human | Random | Male | 53 | unknown | unknown | Random | BA.2 | GRA |
| hCoV-19/Tunisia/IPT-S-2836/2022 | EPI_ISL_17731599 | 06/04/2022 | Africa / Tunisia / Tunis | Human | Random | Male | 78 | unknown | unknown | Random | BA.2.3 | GRA |
| hCoV-19/Tunisia/IPT-S-2835/2022 | EPI_ISL_17731598 | 06/04/2022 | Africa / Tunisia / Ben Arous | Human | Random | Female | 71 | unknown | unknown | Random | BA.2 | GRA |
| hCoV-19/Tunisia/IPT-S-2834/2022 | EPI_ISL_17731597 | 28/03/2022 | Africa / Tunisia / Tunis | Human | Random | Female | 30 | unknown | unknown | Random | BA.2 | GRA |
| hCoV-19/Tunisia/IPT-S-2833/2022 | EPI_ISL_17731596 | 28/03/2022 | Africa / Tunisia / Tunis | Human | Random | Female | 84 | unknown | unknown | Random | BA.2 | GRA |
| hCoV-19/Tunisia/IPT-S-2832/2022 | EPI_ISL_17731595 | 28/03/2022 | Africa / Tunisia / Tunis | Human | Random | Male | unknown | unknown | unknown | Random | BA.2.3 | GRA |
| hCoV-19/Tunisia/IPT-S-2830/2022 | EPI_ISL_17731594 | 28/03/2022 | Africa / Tunisia / Tunis | Human | Random | Female | 47 | unknown | unknown | Random | BA.2 | GRA |
| hCoV-19/Tunisia/IPT-S-2829/2022 | EPI_ISL_17731593 | 28/03/2022 | Africa / Tunisia / Tunis | Human | Random | Female | 24 | unknown | unknown | Random | BA.2 | GRA |
| hCoV-19/Tunisia/IPT-S-2828/2022 | EPI_ISL_17731592 | 28/03/2022 | Africa / Tunisia / Tunis | Human | Random | Female | 72 | unknown | unknown | Random | BA.2.3 | GRA |
| hCoV-19/Tunisia/IPT-S-2827/2022 | EPI_ISL_17731591 | 25/03/2022 | Africa / Tunisia / Tunis | Human | Random | Female | unknown | unknown | unknown | Random | BA.2 | GRA |
| hCoV-19/Tunisia/IPT-S-2826/2022 | EPI_ISL_17731590 | 25/03/2022 | Africa / Tunisia / Tunis | Human | Random | Male | unknown | unknown | unknown | Random | BA.2 | GRA |
| hCoV-19/Tunisia/IPT-S-2824/2022 | EPI_ISL_17731589 | 25/03/2022 | Africa / Tunisia / Tunis | Human | Random | Female | unknown | unknown | unknown | Random | BA.2 | GRA |
| hCoV-19/Tunisia/IPT-S-2823/2022 | EPI_ISL_17731588 | 25/03/2022 | Africa / Tunisia / Tunis | Human | Random | Female | unknown | unknown | unknown | Random | BA.2.3 | GRA |
| hCoV-19/Tunisia/IPT-S-2822/2022 | EPI_ISL_17731587 | 25/03/2022 | Africa / Tunisia / Tunis | Human | Random | Female | unknown | unknown | unknown | Random | BA.2 | GRA |
| hCoV-19/Tunisia/IPT-S-2821/2022 | EPI_ISL_17731586 | 25/03/2022 | Africa / Tunisia / Tunis | Human | Random | Female | unknown | unknown | unknown | Random | BA.2 | GRA |
| hCoV-19/Tunisia/IPT-S-2820/2022 | EPI_ISL_17731585 | 25/03/2022 | Africa / Tunisia / Tunis | Human | Random | Female | unknown | unknown | unknown | Random | BA.2 | GRA |
| hCoV-19/Tunisia/IPT-S-2819/2022 | EPI_ISL_17731584 | 25/03/2022 | Africa / Tunisia / Tunis | Human | Random | Female | unknown | unknown | unknown | Random | BA.2 | GRA |
| hCoV-19/Tunisia/IPT-S-2817/2022 | EPI_ISL_17731583 | 25/03/2022 | Africa / Tunisia / Tunis | Human | Random | Female | unknown | unknown | unknown | Random | BA.2 | GRA |
| hCoV-19/Tunisia/IPT-S-2813/2022 | EPI_ISL_17731582 | 25/03/2022 | Africa / Tunisia / Bizerte | Human | Random | Female | 74 | unknown | unknown | Random | BA.2 | GRA |
| hCoV-19/Tunisia/IPT-S-2812/2022 | EPI_ISL_17731581 | 25/03/2022 | Africa / Tunisia / Gabes | Human | Random | Female | 47 | unknown | unknown | Random | BA.2 | GRA |
| hCoV-19/Tunisia/IPT-S-2807/2022 | EPI_ISL_17731580 | 25/03/2022 | Africa / Tunisia / Gbeli | Human | Random | Male | 22 | unknown | unknown | Random | BA.2 | GRA |
| hCoV-19/Tunisia/IPT-S-2806/2022 | EPI_ISL_17731579 | 25/03/2022 | Africa / Tunisia / Tunis | Human | Random | Male | 71 | unknown | unknown | Random | BA.2 | GRA |
| hCoV-19/Tunisia/IPT-S-2803/2022 | EPI_ISL_17731578 | 25/03/2022 | Africa / Tunisia / Kairouan | Human | Random | Male | 64 | unknown | unknown | Random | BA.2 | GRA |
| hCoV-19/Tunisia/IPT-S-2802/2022 | EPI_ISL_17731577 | 25/03/2022 | Africa / Tunisia / Tunis | Human | Random | Female | unknown | unknown | unknown | Random | BA.2 | GRA |
| hCoV-19/Tunisia/IPT-S-2801/2022 | EPI_ISL_17731576 | 25/03/2022 | Africa / Tunisia / Tunis | Human | Random | Female | 55 | unknown | unknown | Random | BA.2 | GRA |
| hCoV-19/Tunisia/IPT-S-2798/2022 | EPI_ISL_17731575 | 25/03/2022 | Africa / Tunisia / Tunis | Human | Random | Female | 60 | unknown | unknown | Random | BA.2 | GRA |
| hCoV-19/Tunisia/IPT-S-2797/2022 | EPI_ISL_17731574 | 25/03/2022 | Africa / Tunisia / Tunis | Human | Random | Female | 60 | unknown | unknown | Random | BA.2 | GRA |
| hCoV-19/Tunisia/IPT-S-2795/2022 | EPI_ISL_17731573 | 25/03/2022 | Africa / Tunisia / Tunis | Human | Random | Female | 59 | unknown | unknown | Random | BA.2 | GRA |
| hCoV-19/Tunisia/2212/2023 | EPI_ISL_17726529 | 16/02/2023 | Africa / Tunisia / Ariana | Human | Baseline surveillance | Male | 29 | unknown | unknown | Baseline surveillance | Unassigned | GR |
| hCoV-19/Tunisia/1990/2023 | EPI_ISL_17726501 | 10/02/2023 | Africa / Tunisia / Ariana | Human | Baseline surveillance | Male | 59 | unknown | unknown | Baseline surveillance | Unassigned | GR |
| hCoV-19/Tunisia/1915/2023 | EPI_ISL_17726500 | 28/02/2023 | Africa / Tunisia / Tunis | Human | Baseline surveillance | Male | unknown | unknown | unknown | Baseline surveillance | Unassigned | G |
| hCoV-19/Tunisia/1914/2023 | EPI_ISL_17726499 | 28/02/2023 | Africa / Tunisia / Tunis | Human | Baseline surveillance | Female | 39 | unknown | unknown | Baseline surveillance | EG.4 | GRA |
| hCoV-19/Tunisia/1806/2023 | EPI_ISL_17726391 | 07/02/2023 | Africa / Tunisia / Mannouba | Human | unknown | Female | 61 | unknown | unknown | unknown | Unassigned | GR |
| hCoV-19/Tunisia/4874/2020 | EPI_ISL_811145 | 24/03/2020 | Africa / Tunisia / Djerba | Human | unknown | Male | 37 | unknown | unknown | unknown | B.1.177 | GV |
| hCoV-19/Tunisia/21-2249/2021 | EPI_ISL_803851 | 08/01/2021 | Africa / Tunisia / Nabeul | Human | unknown | Male | 21 | unknown | unknown | unknown | B.1.177 | GV |
| hCoV-19/Tunisia/12-8274/2020 | EPI_ISL_803817 | 07/04/2020 | Africa / Tunisia / Ariana | Human | unknown | Female | unknown | unknown | unknown | unknown | B.1.428.2 | GH |
| hCoV-19/Tunisia/1890/2021 | EPI_ISL_803430 | 07/01/2021 | Africa / Tunisia / Nabeul | Human | unknown | Female | 50 | unknown | unknown | unknown | B.1.177 | GV |
| hCoV-19/Tunisia/1821/2021 | EPI_ISL_803120 | 07/01/2021 | Africa / Tunisia / Ben Arous | Human | unknown | Female | 22 | unknown | unknown | unknown | B.1.160 | GH |
| hCoV-19/Tunisia/2285/2021 | EPI_ISL_803119 | 08/01/2021 | Africa / Tunisia / Nabeul | Human | unknown | Female | 70 | unknown | unknown | unknown | B.1.177 | GV |
| hCoV-19/Tunisia/IPT-V-7227/2022 | EPI_ISL_17543452 | 28/11/2022 | Africa / Tunisia / Tunis | Human | Random | Female | 36 | unknown | unknown | Random | BQ.1.1 | GRA |
| hCoV-19/Tunisia/IPT-G-4481/2023 | EPI_ISL_17543451 | 13/02/2023 | Africa / Tunisia / Tunis | Human | Random | Female | 57 | unknown | unknown | Random | BA.2.10.1 | GRA |
| hCoV-19/Tunisia/IPT-G-4457/2023 | EPI_ISL_17543450 | 02/02/2023 | Africa / Tunisia / Tunis | Human | Random | Female | 45 | unknown | unknown | Random | EG.4 | GRA |
| hCoV-19/Tunisia/IPT-G-4445/2023 | EPI_ISL_17543449 | 26/01/2023 | Africa / Tunisia / Tunis | Human | Random | Female | 42 | unknown | unknown | Random | BQ.1.1.59 | GRA |
| hCoV-19/Tunisia/IPT-G-4214/2022 | EPI_ISL_17543447 | 27/09/2022 | Africa / Tunisia / Tunis | Human | Random | Female | 80 | unknown | unknown | Random | BA.5.2 | GRA |
| hCoV-19/Tunisia/69681/2020 | EPI_ISL_796782 | 24/12/2020 | Africa / Tunisia / Ksar Hellal | Human | unknown | Male | unknown | unknown | unknown | unknown | B.1.177.6 | GV |
| hCoV-19/Tunisia/1267/2021 | EPI_ISL_794738 | 06/01/2021 | Africa / Tunisia / Tunis | Human | unknown | Female | 25 | Live | unknown | unknown | B.1.177 | GV |
| hCoV-19/Tunisia/1241/2021 | EPI_ISL_794737 | 06/01/2021 | Africa / Tunisia / Nabeul | Human | unknown | Female | 57 | unknown | unknown | unknown | B.1.177 | GV |
| hCoV-19/Tunisia/1268/2021 | EPI_ISL_794736 | 06/01/2021 | Africa / Tunisia / Tunis | Human | unknown | Female | 33 | Live | unknown | unknown | B.1.160 | GH |
| hCoV-19/Tunisia/1280/2021 | EPI_ISL_794735 | 06/01/2021 | Africa / Tunisia / Tunis | Human | unknown | Male | 59 | Live | unknown | unknown | B.1.1 | GR |
| hCoV-19/Tunisia/V-7227/2022 | EPI_ISL_17383739 | 28/11/2022 | Africa / Tunisia / Tunis | Human | Random | Female | 36 | unknown | unknown | Random | BQ.1.1 | GRA |
| hCoV-19/Tunisia/G-4481/2023 | EPI_ISL_17383738 | 13/02/2023 | Africa / Tunisia / Tunis | Human | Random | Female | 57 | unknown | unknown | Random | BA.2.10.1 | GRA |
| hCoV-19/Tunisia/G-4457/2023 | EPI_ISL_17383737 | 02/02/2023 | Africa / Tunisia / Tunis | Human | Random | Female | 45 | unknown | unknown | Random | EG.4 | GRA |
| hCoV-19/Tunisia/G-4445/2023 | EPI_ISL_17383736 | 26/01/2023 | Africa / Tunisia / Tunis | Human | Random | Female | 42 | unknown | unknown | Random | BQ.1.1.59 | GRA |
| hCoV-19/Tunisia/G-4414/2023 | EPI_ISL_17383735 | 12/01/2023 | Africa / Tunisia / Tunis | Human | Random | Male | 71 | unknown | unknown | Random | XBB.1.5.52 | GRA |
| hCoV-19/Tunisia/G-4214/2022 | EPI_ISL_17383734 | 27/09/2022 | Africa / Tunisia / Tunis | Human | Random | Female | 80 | unknown | unknown | Random | BA.5.2 | GRA |
| hCoV-19/Tunisia/IPT-0001/2021 | EPI_ISL_17036536 | 01/03/2021 | Africa / Tunisia / Kairouan | Human | Random | Female | 60 | unknown | unknown | Random | B.1.243 | G |
| hCoV-19/Tunisia/IPT-0880/2021 | EPI_ISL_17036520 | 08/06/2021 | Africa / Tunisia / Tunis | Human | Random | unknown | unknown | unknown | unknown | Random | B.1.160.28 | GH |
| hCoV-19/Tunisia/IPT-0796/2021 | EPI_ISL_17036519 | 29/06/2021 | Africa / Tunisia / Tunis | Human | Random | Female | 65 | unknown | unknown | Random | AY.122 | GK |
| hCoV-19/Tunisia/IPT-0791/2021 | EPI_ISL_17036518 | 29/06/2021 | Africa / Tunisia / Tunis | Human | Random | Female | 36 | unknown | unknown | Random | B.1.177 | GV |
| hCoV-19/Tunisia/IPT-0723/2021 | EPI_ISL_17036517 | 23/03/2021 | Africa / Tunisia / Tunis | Human | Random | Male | 52 | unknown | unknown | Random | B.1.525 | G |
| hCoV-19/Tunisia/IPT-0606/2021 | EPI_ISL_17036516 | 08/03/2021 | Africa / Tunisia / Monastir | Human | Random | Female | 70 | unknown | unknown | Random | B.1.160 | GH |
| hCoV-19/Tunisia/IPT-0605/2021 | EPI_ISL_17036515 | 08/03/2021 | Africa / Tunisia / Monastir | Human | Random | Male | 66 | unknown | unknown | Random | B.1.160 | GH |
| hCoV-19/Tunisia/IPT-0431/2021 | EPI_ISL_17036514 | 09/04/2021 | Africa / Tunisia / Medenine | Human | Random | Male | 57 | unknown | unknown | Random | AY.122 | GK |
| hCoV-19/Tunisia/IPT-0414/2021 | EPI_ISL_17036513 | 11/04/2021 | Africa / Tunisia / Medenine | Human | Random | Male | 61 | unknown | unknown | Random | AY.122 | GK |
| hCoV-19/Tunisia/IPT-0407/2021 | EPI_ISL_17036512 | 05/04/2021 | Africa / Tunisia / Medenine | Human | Random | Male | 76 | unknown | unknown | Random | AY.122 | GK |
| hCoV-19/Tunisia/IPT-0394/2021 | EPI_ISL_17036511 | 28/04/2021 | Africa / Tunisia / Medenine | Human | Random | Male | 86 | unknown | unknown | Random | B.1.525 | G |
| hCoV-19/Tunisia/IPT-0393/2021 | EPI_ISL_17036510 | 28/04/2021 | Africa / Tunisia / Medenine | Human | Random | Female | 55 | unknown | unknown | Random | B.1.525 | G |
| hCoV-19/Tunisia/IPT-0311/2021 | EPI_ISL_17036509 | 24/04/2021 | Africa / Tunisia / Medenine | Human | Random | Male | 87 | unknown | unknown | Random | B.1.525 | G |
| hCoV-19/Tunisia/IPT-0195/2021 | EPI_ISL_17036508 | 22/03/2021 | Africa / Tunisia / Sfax | Human | Random | Female | unknown | unknown | unknown | Random | A.23.1 | S |
| hCoV-19/Tunisia/IPT-0191/2021 | EPI_ISL_17036507 | 19/03/2021 | Africa / Tunisia / Sfax | Human | Random | Female | 51 | unknown | unknown | Random | B.1.160 | GH |
| hCoV-19/Tunisia/IPT-0036/2021 | EPI_ISL_17036506 | 05/03/2021 | Africa / Tunisia / Tunis | Human | Random | Male | unknown | unknown | unknown | Random | BA.1.1 | GRA |
| hCoV-19/Tunisia/IPT-0024/2021 | EPI_ISL_17036505 | 11/03/2021 | Africa / Tunisia / Manouba | Human | Random | Female | 39 | unknown | unknown | Random | B.1.160.28 | GH |
| hCoV-19/Tunisia/IPT-0017/2021 | EPI_ISL_17036504 | 11/03/2021 | Africa / Tunisia / Manouba | Human | Random | Female | 20 | unknown | unknown | Random | B.1.160 | GH |
| hCoV-19/Tunisia/IPT-0011/2021 | EPI_ISL_17036503 | 04/03/2021 | Africa / Tunisia / Kebili | Human | Random | Male | 74 | unknown | unknown | Random | B.1.160 | GH |
| hCoV-19/Tunisia/IPT-0010/2021 | EPI_ISL_17036502 | 04/03/2021 | Africa / Tunisia / Kebili | Human | Random | Female | 52 | unknown | unknown | Random | B.1.160 | GH |
| hCoV-19/Tunisia/IPT-0002/2021 | EPI_ISL_17036501 | 01/03/2021 | Africa / Tunisia / Kairouan | Human | Random | Male | unknown | unknown | unknown | Random | B.1.160 | GH |
| hCoV-19/Tunisia/IPT-3894/2021 | EPI_ISL_17036500 | 27/05/2021 | Africa / Tunisia / Tunis | Human | Random | Male | 60 | unknown | unknown | Random | AY.122 | GK |
| hCoV-19/Tunisia/IPT-4064/2021 | EPI_ISL_17036499 | 27/05/2021 | Africa / Tunisia / Tunis | Human | Random | Female | 16 | unknown | unknown | Random | B.1.160 | GH |
| hCoV-19/Tunisia/IPT-3339/2021 | EPI_ISL_17036498 | 18/03/2021 | Africa / Tunisia | Human | Random | unknown | unknown | unknown | unknown | Random | B.1.160 | GH |
| hCoV-19/Tunisia/IPT-3152/2021 | EPI_ISL_17036497 | 18/03/2021 | Africa / Tunisia | Human | Random | unknown | unknown | unknown | unknown | Random | B.1.177 | GV |
| hCoV-19/Tunisia/IPT-1552/2021 | EPI_ISL_17036496 | 10/03/2021 | Africa / Tunisia / Tunis | Human | Random | Male | 20 | unknown | unknown | Random | B.1.160 | GH |
| hCoV-19/Tunisia/IPT-1503/2021 | EPI_ISL_17036495 | 09/03/2021 | Africa / Tunisia | Human | Random | unknown | unknown | unknown | unknown | Random | B.1.160 | GH |
| hCoV-19/Tunisia/B-1552/2021 | EPI_ISL_17018835 | 10/03/2021 | Africa / Tunisia / Tunis | Human | Random | Male | 20 | unknown | unknown | Random | B.1.160 | GH |
| hCoV-19/Tunisia/S-0036/2021 | EPI_ISL_17018834 | 12/03/2021 | Africa / Tunisia / Tunis | Human | Random | Male | unknown | unknown | unknown | Random | BA.1.1 | GRA |
| hCoV-19/Tunisia/D-3894/2021 | EPI_ISL_17018833 | 27/05/2021 | Africa / Tunisia / Tunis | Human | Random | Male | 60 | unknown | unknown | Random | AY.122 | GK |
| hCoV-19/Tunisia/S-0723/2021 | EPI_ISL_17018832 | 23/03/2021 | Africa / Tunisia / Tunis | Human | Random | Male | 52 | unknown | unknown | Random | B.1.525 | G |
| hCoV-19/Tunisia/S-0024/2021 | EPI_ISL_17018831 | 11/03/2021 | Africa / Tunisia / Manouba | Human | Random | Female | 39 | unknown | unknown | Random | B.1.160.28 | GH |
| hCoV-19/Tunisia/S-0393/2021 | EPI_ISL_17018830 | 28/04/2021 | Africa / Tunisia / Medenine | Human | Random | Female | 55 | unknown | unknown | Random | B.1.525 | G |
| hCoV-19/Tunisia/S-0311/2021 | EPI_ISL_17018829 | 24/04/2021 | Africa / Tunisia / Medenine | Human | Random | Male | 87 | unknown | unknown | Random | B.1.525 | G |
| hCoV-19/Tunisia/S-0002/2021 | EPI_ISL_17018828 | 01/03/2021 | Africa / Tunisia / Kairouan | Human | Random | Male | unknown | unknown | unknown | Random | B.1.160 | GH |
| hCoV-19/Tunisia/S-0191/2021 | EPI_ISL_17018827 | 19/03/2021 | Africa / Tunisia / Sfax | Human | Random | Female | 51 | unknown | unknown | Random | B.1.160 | GH |
| hCoV-19/Tunisia/S-0796/2021 | EPI_ISL_17018826 | 29/06/2021 | Africa / Tunisia / Tunis | Human | Random | Female | 65 | unknown | unknown | Random | AY.122 | GK |
| hCoV-19/Tunisia/S-0394/2021 | EPI_ISL_17018825 | 28/04/2021 | Africa / Tunisia / Medenine | Human | Random | Male | 86 | unknown | unknown | Random | B.1.525 | G |
| hCoV-19/Tunisia/S-0431/2021 | EPI_ISL_17018824 | 09/04/2021 | Africa / Tunisia / Medenine | Human | Random | Male | 57 | unknown | unknown | Random | AY.122 | GK |
| hCoV-19/Tunisia/S-0414/2021 | EPI_ISL_17018823 | 11/04/2021 | Africa / Tunisia / Medenine | Human | Random | Male | 61 | unknown | unknown | Random | AY.122 | GK |
| hCoV-19/Tunisia/S-0407/2021 | EPI_ISL_17018822 | 05/04/2021 | Africa / Tunisia / Medenine | Human | Random | Male | 76 | unknown | unknown | Random | AY.122 | GK |
| hCoV-19/Tunisia/S-0880/2021 | EPI_ISL_17018821 | 08/06/2021 | Africa / Tunisia / Tunis | Human | Random | unknown | unknown | unknown | unknown | Random | B.1.160.28 | GH |
| hCoV-19/Tunisia/S-0605/2021 | EPI_ISL_17018818 | 08/03/2021 | Africa / Tunisia / Monastir | Human | Random | Male | 66 | unknown | unknown | Random | B.1.160 | GH |
| hCoV-19/Tunisia/S-0195/2021 | EPI_ISL_17018817 | 22/03/2021 | Africa / Tunisia / Sfax | Human | Random | Female | unknown | unknown | unknown | Random | A.23.1 | S |
| hCoV-19/Tunisia/S-0017/2021 | EPI_ISL_17018816 | 11/03/2021 | Africa / Tunisia / Manouba | Human | Random | Female | 20 | unknown | unknown | Random | B.1.160 | GH |
| hCoV-19/Tunisia/S-0011/2021 | EPI_ISL_17018815 | 04/03/2021 | Africa / Tunisia / Kebili | Human | Random | Male | 74 | unknown | unknown | Random | B.1.160 | GH |
| hCoV-19/Tunisia/S-0010/2021 | EPI_ISL_17018814 | 04/03/2021 | Africa / Tunisia / Kebili | Human | Random | Female | 52 | unknown | unknown | Random | B.1.160 | GH |
| hCoV-19/Tunisia/B-4064/2021 | EPI_ISL_17018812 | 27/05/2021 | Africa / Tunisia / Tunis | Human | Random | Female | 16 | unknown | unknown | Random | B.1.160 | GH |
| hCoV-19/Tunisia/B-3339/2021 | EPI_ISL_17018811 | 18/03/2021 | Africa / Tunisia | Human | Random | unknown | unknown | unknown | unknown | Random | B.1.160 | GH |
| hCoV-19/Tunisia/B-1503/2021 | EPI_ISL_17018809 | 09/03/2021 | Africa / Tunisia | Human | Random | unknown | unknown | unknown | unknown | Random | B.1.160 | GH |
| hCoV-19/Tunisia/D-1592-b/2021 | EPI_ISL_16981072 | 27/05/2021 | Africa / Tunisia / Tunis | Human | Random | Female | 18 | unknown | unknown | Random | B.1.1.7 | GRY |
| hCoV-19/Tunisia/D-1879-b/2021 | EPI_ISL_16981071 | 27/05/2021 | Africa / Tunisia / Tunis | Human | Random | Male | 15 | unknown | unknown | Random | B.1.1.7 | GRY |
| hCoV-19/Tunisia/D-4661/2021 | EPI_ISL_16967982 | 01/06/2021 | Africa / Tunisia / Tunis | Human | Random | Female | 32 | unknown | unknown | Random | B.1.1.7 | GRY |
| hCoV-19/Tunisia/D-1153/2021 | EPI_ISL_16967981 | 10/05/2021 | Africa / Tunisia / Tunis | Human | Random | Male | 18 | unknown | unknown | Random | B.1.1.7 | GRY |
| hCoV-19/Tunisia/D-3019/2021 | EPI_ISL_16967980 | 22/05/2021 | Africa / Tunisia / Tunis | Human | Random | Male | 16 | unknown | unknown | Random | B.1.1.7 | GR |
| hCoV-19/Tunisia/D-3220/2021 | EPI_ISL_16967979 | 21/05/2021 | Africa / Tunisia / Tunis | Human | Random | Male | 44 | unknown | unknown | Random | B.1.1.7 | GRY |
| hCoV-19/Tunisia/7899/2020 | EPI_ISL_763067 | 06/04/2020 | Africa / Tunisia / Tunis | Human | unknown | Female | 79 | unknown | unknown | unknown | B.4 | O |
| hCoV-19/Tunisia/6895/2020 | EPI_ISL_763065 | 01/04/2020 | Africa / Tunisia / Tunis | Human | unknown | Male | 60 | unknown | unknown | unknown | B.1.1 | GR |
| hCoV-19/Tunisia/SP-0343/2021 | EPI_ISL_2035945 | 03/03/2021 | Africa / Tunisia / Tunis | Human | unknown | Male | unknown | unknown | unknown | unknown | B.1.1.7 | GRY |
| hCoV-19/Tunisia/S-0914/2021 | EPI_ISL_16955532 | 09/10/2021 | Africa / Tunisia / Mahdia | Human | Random | Female | 82 | unknown | unknown | Random | B.1.1.7 | GRY |
| hCoV-19/Tunisia/S-0638/2021 | EPI_ISL_16955534 | 24/06/2021 | Africa / Tunisia / Sousse | Human | Random | unknown | unknown | unknown | unknown | Random | B.1.1.7 | GR |
| hCoV-19/Tunisia/S-0473/2021 | EPI_ISL_16955528 | 01/05/2021 | Africa / Tunisia / Medenine | Human | Random | Male | 57 | unknown | unknown | Random | B.1.1.7 | GRY |
| hCoV-19/Tunisia/S-0467/2021 | EPI_ISL_16955527 | 07/05/2021 | Africa / Tunisia / Medenine | Human | Random | Male | 61 | unknown | unknown | Random | B.1.1.7 | GRY |
| hCoV-19/Tunisia/S-0428/2021 | EPI_ISL_16955525 | 03/04/2021 | Africa / Tunisia / Medenine | Human | Random | Male | 41 | unknown | unknown | Random | B.1.1.7 | GRY |
| hCoV-19/Tunisia/S-0092/2021 | EPI_ISL_16955524 | 14/03/2021 | Africa / Tunisia / Sousse | Human | Random | Female | 22 | unknown | unknown | Random | B.1.1.7 | GRY |
| hCoV-19/Tunisia/D-6064/2021 | EPI_ISL_16955523 | 07/06/2021 | Africa / Tunisia / Tunis | Human | Random | Female | 49 | Moderate | unknown | Random | B.1.1.7 | GRY |
| hCoV-19/Tunisia/D-4841/2021 | EPI_ISL_16955522 | 01/06/2021 | Africa / Tunisia / Tunis | Human | Random | Female | 51 | unknown | unknown | Random | B.1.1.7 | GRY |
| hCoV-19/Tunisia/D-4765/2021 | EPI_ISL_16955521 | 01/06/2021 | Africa / Tunisia / Tunis | Human | Random | Female | 30 | unknown | unknown | Random | B.1.1.7 | GRY |
| hCoV-19/Tunisia/D-3875/2021 | EPI_ISL_16955520 | 27/05/2021 | Africa / Tunisia / Tunis | Human | Random | Female | 67 | Moderate | unknown | Random | B.1.1.7 | GRY |
| hCoV-19/Tunisia/D-3840/2021 | EPI_ISL_16955519 | 27/05/2021 | Africa / Tunisia / Tunis | Human | Random | Female | 43 | unknown | unknown | Random | B.1.1.7 | GRY |
| hCoV-19/Tunisia/D-3710/2021 | EPI_ISL_16955518 | 26/05/2021 | Africa / Tunisia / Tunis | Human | Random | Female | 53 | Moderate | unknown | Random | B.1.1.7 | GRY |
| hCoV-19/Tunisia/Ariana/S-0387/2021 | EPI_ISL_16944358 | 08/05/2021 | Africa / Tunisia / Ariana | Human | Random | Female | unknown | unknown | unknown | Random | B.1.1.7 | GRY |
| hCoV-19/Tunisia/Jendouba/S-0947/2021 | EPI_ISL_16944357 | 09/10/2021 | Africa / Tunisia / Jendouba | Human | Random | Male | 61 | unknown | unknown | Random | B.1.1.7 | GRY |
| hCoV-19/Tunisia/Kasserine/S-0931/2021 | EPI_ISL_16944356 | 09/10/2021 | Africa / Tunisia / Kasserine | Human | Random | Female | unknown | unknown | unknown | Random | B.1.1.7 | GRY |
| hCoV-19/Tunisia/Mahdia/S-0920/2021 | EPI_ISL_16944355 | 09/10/2021 | Africa / Tunisia / Mahdia | Human | Random | Female | 47 | unknown | unknown | Random | B.1.1.7 | GRY |
| hCoV-19/Tunisia/Mahdia/S-0912/2021 | EPI_ISL_16944354 | 09/10/2021 | Africa / Tunisia / Mahdia | Human | Random | Female | 55 | unknown | unknown | Random | B.1.1.7 | GRY |
| hCoV-19/Tunisia/Gafsa/S-0884/2021 | EPI_ISL_16944353 | 30/08/2021 | Africa / Tunisia / Gafsa | Human | Random | Female | 15 | unknown | unknown | Random | B.1.1.7 | GRY |
| hCoV-19/Tunisia/Gafsa/S-0840/2021 | EPI_ISL_16944352 | 30/08/2021 | Africa / Tunisia / Gafsa | Human | Random | Male | 52 | unknown | unknown | Random | B.1.1.7 | GRY |
| hCoV-19/Tunisia/Manouba/S-0813/2021 | EPI_ISL_16944351 | 08/05/2021 | Africa / Tunisia / Manouba | Human | Random | Female | unknown | unknown | unknown | Random | B.1.1.7 | GR |
| hCoV-19/Tunisia/Zaghouan/S-0801/2021 | EPI_ISL_16944350 | 08/05/2021 | Africa / Tunisia / Zaghouan | Human | Random | Male | 48 | unknown | unknown | Random | B.1.1.7 | GRY |
| hCoV-19/Tunisia/Beja/S-0792/2021 | EPI_ISL_16944349 | 30/06/2021 | Africa / Tunisia / Beja | Human | Random | Female | unknown | unknown | unknown | Random | B.1.1.7 | GRY |
| hCoV-19/Tunisia/Beja/S-0788/2021 | EPI_ISL_16944348 | 30/08/2021 | Africa / Tunisia / Beja | Human | Random | Female | 47 | unknown | unknown | Random | B.1.1.7 | GRY |
| hCoV-19/Tunisia/Beja/S-0786/2021 | EPI_ISL_16944347 | 05/05/2021 | Africa / Tunisia / Beja | Human | Random | Female | 53 | unknown | unknown | Random | B.1.1.7 | GRY |
| hCoV-19/Tunisia/Medenine/S-0774/2021 | EPI_ISL_16944346 | 27/05/2021 | Africa / Tunisia / Medenine | Human | Random | Male | 46 | unknown | unknown | Random | B.1.1.7 | GRY |
| hCoV-19/Tunisia/Medenine/S-0773/2021 | EPI_ISL_16944345 | 27/05/2021 | Africa / Tunisia / Medenine | Human | Random | Female | 32 | unknown | unknown | Random | B.1.1.7 | GRY |
| hCoV-19/Tunisia/Medenine/S-0772/2021 | EPI_ISL_16944344 | 01/06/2021 | Africa / Tunisia / Medenine | Human | Random | Male | 31 | unknown | unknown | Random | B.1.1.7 | GRY |
| hCoV-19/Tunisia/Medenine/S-0757/2021 | EPI_ISL_16944343 | 29/06/2021 | Africa / Tunisia / Medenine | Human | Random | Female | 53 | unknown | unknown | Random | B.1.1.7 | GRY |
| hCoV-19/Tunisia/Medenine/S-0751/2021 | EPI_ISL_16944342 | 07/06/2021 | Africa / Tunisia / Medenine | Human | Random | Male | 46 | unknown | unknown | Random | B.1.1.7 | GR |
| hCoV-19/Tunisia/Sousse/S-0743/2021 | EPI_ISL_16944341 | 17/06/2021 | Africa / Tunisia / Sousse | Human | Random | Female | 69 | unknown | unknown | Random | B.1.1.7 | GRY |
| hCoV-19/Tunisia/Nabeul/S-0717/2021 | EPI_ISL_16944340 | 27/05/2021 | Africa / Tunisia / Nabeul | Human | Random | Female | 50 | unknown | unknown | Random | B.1.1.7 | GRY |
| hCoV-19/Tunisia/Sfax/S-0697/2021 | EPI_ISL_16944339 | 30/04/2021 | Africa / Tunisia / Sfax | Human | Random | Male | unknown | unknown | unknown | Random | B.1.1.7 | GRY |
| hCoV-19/Tunisia/Sfax/S-0696/2021 | EPI_ISL_16944338 | 06/05/2021 | Africa / Tunisia / Sfax | Human | Random | Female | 20 | unknown | unknown | Random | B.1.1.7 | GRY |
| hCoV-19/Tunisia/Sfax/S-0687/2021 | EPI_ISL_16944337 | 01/04/2021 | Africa / Tunisia / Sfax | Human | Random | Male | 52 | unknown | unknown | Random | B.1.1.7 | GRY |
| hCoV-19/Tunisia/Sfax/S-0685/2021 | EPI_ISL_16944336 | 26/06/2021 | Africa / Tunisia / Sfax | Human | Random | Female | unknown | unknown | unknown | Random | B.1.1.7 | GRY |
| hCoV-19/Tunisia/Sfax/S-0682/2021 | EPI_ISL_16944335 | 14/03/2021 | Africa / Tunisia / Sfax | Human | Random | Female | 53 | unknown | unknown | Random | B.1.1.7 | GRY |
| hCoV-19/Tunisia/Sfax/S-0680/2021 | EPI_ISL_16944334 | 25/03/2021 | Africa / Tunisia / Sfax | Human | Random | Male | 52 | unknown | unknown | Random | B.1.1.7 | GRY |
| hCoV-19/Tunisia/Manouba/S-0660/2021 | EPI_ISL_16944333 | 25/05/2021 | Africa / Tunisia / Manouba | Human | Random | Female | 55 | unknown | unknown | Random | B.1.1.7 | GRY |
| hCoV-19/Tunisia/Sousse/S-0637/2021 | EPI_ISL_16944332 | 24/06/2021 | Africa / Tunisia / Sousse | Human | Random | unknown | unknown | unknown | unknown | Random | B.1.1.7 | GRY |
| hCoV-19/Tunisia/Sousse/S-0636/2021 | EPI_ISL_16944331 | 24/06/2021 | Africa / Tunisia / Sousse | Human | Random | unknown | unknown | unknown | unknown | Random | B.1.1.7 | GRY |
| hCoV-19/Tunisia/Sousse/S-0635/2021 | EPI_ISL_16944330 | 24/06/2021 | Africa / Tunisia / Sousse | Human | Random | unknown | unknown | unknown | unknown | Random | B.1.1.7 | GRY |
| hCoV-19/Tunisia/Sousse/S-0634/2021 | EPI_ISL_16944329 | 24/06/2021 | Africa / Tunisia / Sousse | Human | Random | unknown | unknown | unknown | unknown | Random | B.1.1.7 | GRY |
| hCoV-19/Tunisia/Sousse/S-0633/2021 | EPI_ISL_16944328 | 24/06/2021 | Africa / Tunisia / Sousse | Human | Random | unknown | unknown | unknown | unknown | Random | B.1.1.7 | GRY |
| hCoV-19/Tunisia/Sousse/S-0632/2021 | EPI_ISL_16944327 | 24/06/2021 | Africa / Tunisia / Sousse | Human | Random | unknown | unknown | unknown | unknown | Random | B.1.1.7 | GRY |
| hCoV-19/Tunisia/Sousse/S-0631/2021 | EPI_ISL_16944326 | 24/06/2021 | Africa / Tunisia / Sousse | Human | Random | unknown | unknown | unknown | unknown | Random | B.1.1.7 | GRY |
| hCoV-19/Tunisia/Sousse/S-0630/2021 | EPI_ISL_16944325 | 24/06/2021 | Africa / Tunisia / Sousse | Human | Random | unknown | unknown | unknown | unknown | Random | B.1.1.7 | GRY |
| hCoV-19/Tunisia/Sousse/S-0629/2021 | EPI_ISL_16944324 | 24/06/2021 | Africa / Tunisia / Sousse | Human | Random | unknown | unknown | unknown | unknown | Random | B.1.1.7 | GRY |
| hCoV-19/Tunisia/Sousse/S-0625/2021 | EPI_ISL_16944323 | 24/06/2021 | Africa / Tunisia / Sousse | Human | Random | unknown | unknown | unknown | unknown | Random | B.1.1.7 | GR |
| hCoV-19/Tunisia/Sousse/S-0624/2021 | EPI_ISL_16944322 | 24/06/2021 | Africa / Tunisia / Sousse | Human | Random | unknown | unknown | unknown | unknown | Random | B.1.1.7 | GRY |
| hCoV-19/Tunisia/Monastir/S-0623/2021 | EPI_ISL_16944321 | 10/05/2021 | Africa / Tunisia / Monastir | Human | Random | Female | 66 | unknown | unknown | Random | B.1.1.7 | GRY |
| hCoV-19/Tunisia/Monastir/S-0621/2021 | EPI_ISL_16944320 | 01/05/2021 | Africa / Tunisia / Monastir | Human | Random | Female | 25 | unknown | unknown | Random | B.1.1.7 | GRY |
| hCoV-19/Tunisia/Monastir/S-0620/2021 | EPI_ISL_16944319 | 17/05/2021 | Africa / Tunisia / Monastir | Human | Random | Male | 20 | unknown | unknown | Random | B.1.1.7 | GRY |
| hCoV-19/Tunisia/Monastir/S-0619/2021 | EPI_ISL_16944318 | 20/04/2021 | Africa / Tunisia / Monastir | Human | Random | Male | 73 | unknown | unknown | Random | B.1.1.7 | GRY |
| hCoV-19/Tunisia/Monastir/S-0614/2021 | EPI_ISL_16944317 | 15/04/2021 | Africa / Tunisia / Monastir | Human | Random | Female | 53 | unknown | unknown | Random | B.1.1.7 | GRY |
| hCoV-19/Tunisia/Monastir/S-0613/2021 | EPI_ISL_16944316 | 15/04/2021 | Africa / Tunisia / Monastir | Human | Random | Male | 47 | unknown | unknown | Random | B.1.1.7 | GRY |
| hCoV-19/Tunisia/Monastir/S-0612/2021 | EPI_ISL_16944315 | 12/04/2021 | Africa / Tunisia / Monastir | Human | Random | Male | 52 | unknown | unknown | Random | B.1.1.7 | GRY |
| hCoV-19/Tunisia/Sousse/S-0565/2021 | EPI_ISL_16944314 | 18/05/2021 | Africa / Tunisia / Sousse | Human | Random | Female | 31 | unknown | unknown | Random | B.1.1.7 | GRY |
| hCoV-19/Tunisia/Sousse/S-0564/2021 | EPI_ISL_16944313 | 17/05/2021 | Africa / Tunisia / Sousse | Human | Random | Female | 15 | unknown | unknown | Random | B.1.1.7 | GRY |
| hCoV-19/Tunisia/Bizerte/S-0547/2021 | EPI_ISL_16944312 | 21/05/2021 | Africa / Tunisia / Bizerte | Human | Random | Female | 87 | unknown | unknown | Random | B.1.1.7 | GRY |
| hCoV-19/Tunisia/Bizerte/S-0545/2021 | EPI_ISL_16944311 | 25/03/2021 | Africa / Tunisia / Bizerte | Human | Random | Male | 26 | unknown | unknown | Random | B.1.1.7 | GRY |
| hCoV-19/Tunisia/Kebili/S-0539/2021 | EPI_ISL_16944310 | 17/05/2021 | Africa / Tunisia / Kebili | Human | Random | Female | 36 | unknown | unknown | Random | B.1.1.7 | GRY |
| hCoV-19/Tunisia/Sousse/S-0518/2021 | EPI_ISL_16944309 | 16/06/2021 | Africa / Tunisia / Sousse | Human | Random | Female | unknown | unknown | unknown | Random | B.1.1.7 | GRY |
| hCoV-19/Tunisia/Sousse/S-0516/2021 | EPI_ISL_16944308 | 05/06/2021 | Africa / Tunisia / Sousse | Human | Random | Female | 50 | unknown | unknown | Random | B.1.1.7 | GRY |
| hCoV-19/Tunisia/Sousse/S-0515/2021 | EPI_ISL_16944307 | 09/06/2021 | Africa / Tunisia / Sousse | Human | Random | Male | 39 | unknown | unknown | Random | B.1.1.7 | GRY |
| hCoV-19/Tunisia/Tunis/S-0493/2021 | EPI_ISL_16944306 | 08/06/2021 | Africa / Tunisia / Tunis | Human | Random | Female | 50 | unknown | unknown | Random | B.1.1.7 | GRY |
| hCoV-19/Tunisia/Medenine/S-0483/2021 | EPI_ISL_16944305 | 03/05/2021 | Africa / Tunisia / Medenine | Human | Random | Male | 56 | unknown | unknown | Random | B.1.1.7 | GRY |
| hCoV-19/Tunisia/Medenine/S-0475/2021 | EPI_ISL_16944304 | 02/05/2021 | Africa / Tunisia / Medenine | Human | Random | Male | 29 | unknown | unknown | Random | B.1.1.7 | GRY |
| hCoV-19/Tunisia/Medenine/S-0472/2021 | EPI_ISL_16944303 | 06/05/2021 | Africa / Tunisia / Medenine | Human | Random | Male | 39 | unknown | unknown | Random | B.1.1.7 | GRY |
| hCoV-19/Tunisia/Medenine/S-0469/2021 | EPI_ISL_16944302 | 01/05/2021 | Africa / Tunisia / Medenine | Human | Random | Male | 76 | unknown | unknown | Random | B.1.1.7 | GRY |
| hCoV-19/Tunisia/Medenine/S-0468/2021 | EPI_ISL_16944301 | 02/05/2021 | Africa / Tunisia / Medenine | Human | Random | Male | 42 | unknown | unknown | Random | B.1.1.7 | GRY |
| hCoV-19/Tunisia/Medenine/S-0465/2021 | EPI_ISL_16944300 | 05/05/2021 | Africa / Tunisia / Medenine | Human | Random | Female | 59 | unknown | unknown | Random | B.1.1.7 | GRY |
| hCoV-19/Tunisia/Nabeul/S-0461/2021 | EPI_ISL_16944299 | 07/06/2021 | Africa / Tunisia / Nabeul | Human | Random | Female | 53 | unknown | unknown | Random | B.1.1.7 | GRY |
| hCoV-19/Tunisia/Kairouan/S-0451/2021 | EPI_ISL_16944298 | 18/05/2021 | Africa / Tunisia / Kairouan | Human | Random | Male | 42 | unknown | unknown | Random | B.1.1.7 | GRY |
| hCoV-19/Tunisia/Sousse/S-0445/2021 | EPI_ISL_16944297 | 18/05/2021 | Africa / Tunisia / Sousse | Human | Random | Male | 3 months | unknown | unknown | Random | B.1.1.7 | GRY |
| hCoV-19/Tunisia/Sousse/S-0443/2021 | EPI_ISL_16944296 | 08/05/2021 | Africa / Tunisia / Sousse | Human | Random | Male | unknown | unknown | unknown | Random | B.1.1.7 | GRY |
| hCoV-19/Tunisia/Medenine/S-0439/2021 | EPI_ISL_16944295 | 02/04/2021 | Africa / Tunisia / Medenine | Human | Random | Female | 40 | unknown | unknown | Random | B.1.1.7 | GRY |
| hCoV-19/Tunisia/Medenine/S-0436/2021 | EPI_ISL_16944294 | 30/03/2021 | Africa / Tunisia / Medenine | Human | Random | Female | 65 | unknown | unknown | Random | B.1.1.7 | GRY |
| hCoV-19/Tunisia/Medenine/S-0435/2021 | EPI_ISL_16944293 | 01/04/2021 | Africa / Tunisia / Medenine | Human | Random | Male | 71 | unknown | unknown | Random | B.1.1.7 | GRY |
| hCoV-19/Tunisia/Medenine/S-0434/2021 | EPI_ISL_16944292 | 10/04/2021 | Africa / Tunisia / Medenine | Human | Random | Female | 78 | unknown | unknown | Random | B.1.1.7 | GRY |
| hCoV-19/Tunisia/Medenine/S-0432/2021 | EPI_ISL_16944291 | 05/04/2021 | Africa / Tunisia / Medenine | Human | Random | Female | 41 | unknown | unknown | Random | B.1.1.7 | GRY |
| hCoV-19/Tunisia/Medenine/S-0429/2021 | EPI_ISL_16944290 | 05/04/2021 | Africa / Tunisia / Medenine | Human | Random | Female | 68 | unknown | unknown | Random | B.1.1.7 | GRY |
| hCoV-19/Tunisia/Medenine/S-0419/2021 | EPI_ISL_16944289 | 28/04/2021 | Africa / Tunisia / Medenine | Human | Random | Male | 33 | unknown | unknown | Random | B.1.1.7 | GRY |
| hCoV-19/Tunisia/Medenine/S-0402/2021 | EPI_ISL_16944288 | 05/01/2021 | Africa / Tunisia / Medenine | Human | Random | Male | 56 | unknown | unknown | Random | B.1.1.7 | GRY |
| hCoV-19/Tunisia/Medenine/S-0401/2021 | EPI_ISL_16944287 | 05/03/2021 | Africa / Tunisia / Medenine | Human | Random | Female | 61 | unknown | unknown | Random | B.1.1.7 | GRY |
| hCoV-19/Tunisia/Medenine/S-0400/2021 | EPI_ISL_16944286 | 28/04/2021 | Africa / Tunisia / Medenine | Human | Random | Female | 73 | unknown | unknown | Random | B.1.1.7 | GRY |
| hCoV-19/Tunisia/Medenine/S-0396/2021 | EPI_ISL_16944285 | 24/04/2021 | Africa / Tunisia / Medenine | Human | Random | Male | 66 | unknown | unknown | Random | B.1.1.7 | GRY |
| hCoV-19/Tunisia/Ben Arous/S-0384/2021 | EPI_ISL_16944284 | 07/05/2021 | Africa / Tunisia / Ben Arous | Human | Random | Female | unknown | unknown | unknown | Random | B.1.1.7 | GRY |
| hCoV-19/Tunisia/Ben Arous/S-0383/2021 | EPI_ISL_16944283 | 07/05/2021 | Africa / Tunisia / Ben Arous | Human | Random | Female | unknown | unknown | unknown | Random | B.1.1.7 | GRY |
| hCoV-19/Tunisia/Ben Arous/S-0382/2021 | EPI_ISL_16944282 | 07/05/2021 | Africa / Tunisia / Ariana | Human | Random | Male | unknown | unknown | unknown | Random | B.1.1.7 | GRY |
| hCoV-19/Tunisia/Ariana/S-0381/2021 | EPI_ISL_16944281 | 07/05/2021 | Africa / Tunisia / Ariana | Human | Random | Male | unknown | unknown | unknown | Random | B.1.1.7 | GRY |
| hCoV-19/Tunisia/Ben Arous/S-0380/2021 | EPI_ISL_16944280 | 07/05/2021 | Africa / Tunisia / Ben Arous | Human | Random | Male | unknown | unknown | unknown | Random | B.1.1.7 | GRY |
| hCoV-19/Tunisia/Ben Arous/S-0379/2021 | EPI_ISL_16944279 | 07/05/2021 | Africa / Tunisia / Ben Arous | Human | Random | Female | unknown | unknown | unknown | Random | B.1.1.7 | GRY |
| hCoV-19/Tunisia/Ariana/S-0377/2021 | EPI_ISL_16944278 | 07/05/2021 | Africa / Tunisia / Ariana | Human | Random | Female | unknown | unknown | unknown | Random | B.1.1.7 | GRY |
| hCoV-19/Tunisia/Ben Arous/S-0376/2021 | EPI_ISL_16944277 | 07/05/2021 | Africa / Tunisia / Ben Arous | Human | Random | Male | unknown | unknown | unknown | Random | B.1.1.7 | GRY |
| hCoV-19/Tunisia/Ben Arous/S-0375/2021 | EPI_ISL_16944276 | 07/05/2021 | Africa / Tunisia / Ben Arous | Human | Random | Male | unknown | unknown | unknown | Random | B.1.1.7 | GRY |
| hCoV-19/Tunisia/Ariana/S-0374/2021 | EPI_ISL_16944275 | 07/05/2021 | Africa / Tunisia / Ariana | Human | Random | Female | unknown | unknown | unknown | Random | B.1.1.7 | GRY |
| hCoV-19/Tunisia/Ariana/S-0372/2021 | EPI_ISL_16944274 | 07/05/2021 | Africa / Tunisia / Ariana | Human | Random | Male | unknown | unknown | unknown | Random | B.1.1.7 | GRY |
| hCoV-19/Tunisia/Ariana/S-0371/2021 | EPI_ISL_16944273 | 07/05/2021 | Africa / Tunisia / Ariana | Human | Random | Male | unknown | unknown | unknown | Random | B.1.1.7 | GRY |
| hCoV-19/Tunisia/Ben Arous/S-0370/2021 | EPI_ISL_16944272 | 07/05/2021 | Africa / Tunisia / Ben Arous | Human | Random | Female | unknown | unknown | unknown | Random | B.1.1.7 | GRY |
| hCoV-19/Tunisia/Ben Arous/S-0369/2021 | EPI_ISL_16944271 | 07/05/2021 | Africa / Tunisia / Ben Arous | Human | Random | Male | unknown | unknown | unknown | Random | B.1.1.7 | GRY |
| hCoV-19/Tunisia/Ben Arous/S-0364/2021 | EPI_ISL_16944270 | 07/05/2021 | Africa / Tunisia / Ben Arous | Human | Random | Female | unknown | unknown | unknown | Random | B.1.1.7 | GRY |
| hCoV-19/Tunisia/Ben Arous/S-0363/2021 | EPI_ISL_16944269 | 07/05/2021 | Africa / Tunisia / Ben Arous | Human | Random | Male | unknown | unknown | unknown | Random | B.1.1.7 | GRY |
| hCoV-19/Tunisia/Ariana/S-0361/2021 | EPI_ISL_16944268 | 07/05/2021 | Africa / Tunisia / Ariana | Human | Random | Male | unknown | unknown | unknown | Random | B.1.1.7 | GRY |
| hCoV-19/Tunisia/Ariana/S-0360/2021 | EPI_ISL_16944267 | 07/05/2021 | Africa / Tunisia / Ariana | Human | Random | Female | unknown | unknown | unknown | Random | B.1.1.7 | GRY |
| hCoV-19/Tunisia/Ariana/S-0359/2021 | EPI_ISL_16944266 | 07/05/2021 | Africa / Tunisia / Ariana | Human | Random | Male | unknown | unknown | unknown | Random | B.1.1.7 | GRY |
| hCoV-19/Tunisia/Ariana/S-0358/2021 | EPI_ISL_16944265 | 07/05/2021 | Africa / Tunisia / Ariana | Human | Random | Male | unknown | unknown | unknown | Random | B.1.1.7 | GRY |
| hCoV-19/Tunisia/Ben Arous/S-0354/2021 | EPI_ISL_16944264 | 07/05/2021 | Africa / Tunisia / Ben Arous | Human | Random | Male | unknown | unknown | unknown | Random | B.1.1.7 | GRY |
| hCoV-19/Tunisia/Ben Arous/S-0353/2021 | EPI_ISL_16944263 | 07/05/2021 | Africa / Tunisia / Ben Arous | Human | Random | Male | unknown | unknown | unknown | Random | B.1.1.7 | GRY |
| hCoV-19/Tunisia/Ben Arous/S-0352/2021 | EPI_ISL_16944262 | 07/05/2021 | Africa / Tunisia / Ben Arous | Human | Random | Male | unknown | unknown | unknown | Random | B.1.1.7 | GRY |
| hCoV-19/Tunisia/Ben Arous/S-0350/2021 | EPI_ISL_16944261 | 07/05/2021 | Africa / Tunisia / Ben Arous | Human | Random | Female | unknown | unknown | unknown | Random | B.1.1.7 | GRY |
| hCoV-19/Tunisia/Ben Arous/S-0348/2021 | EPI_ISL_16944260 | 07/05/2021 | Africa / Tunisia / Ben Arous | Human | Random | Female | unknown | unknown | unknown | Random | B.1.1.7 | GRY |
| hCoV-19/Tunisia/Ben Arous/S-0346/2021 | EPI_ISL_16944259 | 07/05/2021 | Africa / Tunisia / Ben Arous | Human | Random | Female | unknown | unknown | unknown | Random | B.1.1.7 | GRY |
| hCoV-19/Tunisia/Ariana/S-0345/2021 | EPI_ISL_16944258 | 07/05/2021 | Africa / Tunisia / Ariana | Human | Random | Female | unknown | unknown | unknown | Random | B.1.1.7 | GRY |
| hCoV-19/Tunisia/Ariana/S-0343/2021 | EPI_ISL_16944257 | 07/05/2021 | Africa / Tunisia / Ariana | Human | Random | Male | unknown | unknown | unknown | Random | B.1.1.7 | GRY |
| hCoV-19/Tunisia/Ariana/S-0341/2021 | EPI_ISL_16944256 | 07/05/2021 | Africa / Tunisia / Ariana | Human | Random | Male | unknown | unknown | unknown | Random | B.1.1.7 | GRY |
| hCoV-19/Tunisia/Ariana/S-0340/2021 | EPI_ISL_16944255 | 07/05/2021 | Africa / Tunisia / Ariana | Human | Random | Male | unknown | unknown | unknown | Random | B.1.1.7 | GRY |
| hCoV-19/Tunisia/Ariana/S-0339/2021 | EPI_ISL_16944254 | 07/05/2021 | Africa / Tunisia / Ariana | Human | Random | Male | unknown | unknown | unknown | Random | B.1.1.7 | GRY |
| hCoV-19/Tunisia/Sousse/S-0315/2021 | EPI_ISL_16944253 | 17/04/2021 | Africa / Tunisia / Sousse | Human | Random | Male | 1 month | unknown | unknown | Random | B.1.1.7 | GRY |
| hCoV-19/Tunisia/Sousse/S-0312/2021 | EPI_ISL_16944252 | 30/04/2021 | Africa / Tunisia / Sousse | Human | Random | Male | 65 | unknown | unknown | Random | B.1.1.7 | GRY |
| hCoV-19/Tunisia/Medenine/S-0303/2021 | EPI_ISL_16944251 | 15/04/2021 | Africa / Tunisia / Medenine | Human | Random | Male | 28 | unknown | unknown | Random | B.1.1.7 | GRY |
| hCoV-19/Tunisia/Medenine/S-0301/2021 | EPI_ISL_16944250 | 15/04/2021 | Africa / Tunisia / Medenine | Human | Random | Male | 38 | unknown | unknown | Random | B.1.1.7 | GRY |
| hCoV-19/Tunisia/Medenine/S-0292/2021 | EPI_ISL_16944249 | 07/04/2021 | Africa / Tunisia / Medenine | Human | Random | Female | unknown | unknown | unknown | Random | B.1.1.7 | GRY |
| hCoV-19/Tunisia/Medenine/S-0286/2021 | EPI_ISL_16944248 | 08/04/2021 | Africa / Tunisia / Medenine | Human | Random | Male | 65 | unknown | unknown | Random | B.1.1.7 | GRY |
| hCoV-19/Tunisia/Kairouan/S-0276/2021 | EPI_ISL_16944247 | 03/04/2021 | Africa / Tunisia / Kairouan | Human | Random | Female | unknown | unknown | unknown | Random | B.1.1.7 | GRY |
| hCoV-19/Tunisia/Kairouan/S-0275/2021 | EPI_ISL_16944246 | 26/03/2021 | Africa / Tunisia / Kairouan | Human | Random | Male | 51 | unknown | unknown | Random | B.1.1.7 | GRY |
| hCoV-19/Tunisia/Kairouan/S-0274/2021 | EPI_ISL_16944245 | 01/04/2021 | Africa / Tunisia / Kairouan | Human | Random | Male | 41 | unknown | unknown | Random | B.1.1.7 | GRY |
| hCoV-19/Tunisia/Sousse/S-0273/2021 | EPI_ISL_16944244 | 08/04/2021 | Africa / Tunisia / Sousse | Human | Random | Male | 17 | unknown | unknown | Random | B.1.1.7 | GRY |
| hCoV-19/Tunisia/Sousse/S-0272/2021 | EPI_ISL_16944243 | 16/04/2021 | Africa / Tunisia / Sousse | Human | Random | Female | unknown | unknown | unknown | Random | B.1.1.7 | GRY |
| hCoV-19/Tunisia/Sousse/S-0271/2021 | EPI_ISL_16944242 | 12/04/2021 | Africa / Tunisia / Sousse | Human | Random | Male | 44 | unknown | unknown | Random | B.1.1.7 | GRY |
| hCoV-19/Tunisia/Sousse/S-0270/2021 | EPI_ISL_16944241 | 14/04/2021 | Africa / Tunisia / Sousse | Human | Random | Female | 25 | unknown | unknown | Random | B.1.1.7 | GRY |
| hCoV-19/Tunisia/Tozeur/S-0210/2021 | EPI_ISL_16944240 | 04/04/2021 | Africa / Tunisia / Tozeur | Human | Random | Male | 40 | unknown | unknown | Random | B.1.1.7 | GRY |
| hCoV-19/Tunisia/Tozeur/S-0208/2021 | EPI_ISL_16944239 | 04/04/2021 | Africa / Tunisia / Tozeur | Human | Random | Male | 51 | unknown | unknown | Random | B.1.1.7 | GRY |
| hCoV-19/Tunisia/Sousse/S-0204/2021 | EPI_ISL_16944238 | 01/04/2021 | Africa / Tunisia / Sousse | Human | Random | Male | 54 | unknown | unknown | Random | B.1.1.7 | GRY |
| hCoV-19/Tunisia/Sousse/S-0202/2021 | EPI_ISL_16944237 | 27/03/2021 | Africa / Tunisia / Sousse | Human | Random | Male | 61 | unknown | unknown | Random | B.1.1.7 | GRY |
| hCoV-19/Tunisia/Sfax/S-0194/2021 | EPI_ISL_16944236 | 30/03/2021 | Africa / Tunisia / Sfax | Human | Random | Male | 54 | unknown | unknown | Random | B.1.1.7 | GRY |
| hCoV-19/Tunisia/Sfax/S-0192/2021 | EPI_ISL_16944235 | 18/03/2021 | Africa / Tunisia / Sfax | Human | Random | Female | 27 | unknown | unknown | Random | B.1.1.7 | GRY |
| hCoV-19/Tunisia/Sfax/S-0190/2021 | EPI_ISL_16944234 | 27/03/2021 | Africa / Tunisia / Sfax | Human | Random | Female | 71 | unknown | unknown | Random | B.1.1.7 | GRY |
| hCoV-19/Tunisia/Sfax/S-0187/2021 | EPI_ISL_16944233 | 18/03/2021 | Africa / Tunisia / Sfax | Human | Random | Male | 73 | unknown | unknown | Random | B.1.1.7 | GRY |
| hCoV-19/Tunisia/Sfax/S-0186/2021 | EPI_ISL_16944232 | 19/03/2021 | Africa / Tunisia / Sfax | Human | Random | Female | 67 | unknown | unknown | Random | B.1.1.7 | GRY |
| hCoV-19/Tunisia/Kairouan/S-0172/2021 | EPI_ISL_16944231 | 01/04/2021 | Africa / Tunisia / Kairouan | Human | Random | Female | unknown | unknown | unknown | Random | B.1.1.7 | GRY |
| hCoV-19/Tunisia/Ariana/S-0164/2021 | EPI_ISL_16944230 | 01/04/2021 | Africa / Tunisia / Ariana | Human | Random | Male | unknown | unknown | unknown | Random | B.1.1.7 | GRY |
| hCoV-19/Tunisia/Manouba/S-0147/2021 | EPI_ISL_16944229 | 29/03/2021 | Africa / Tunisia / Manouba | Human | Random | Female | 47 | unknown | unknown | Random | B.1.1.7 | GRY |
| hCoV-19/Tunisia/Manouba/S-0145/2021 | EPI_ISL_16944228 | 29/03/2021 | Africa / Tunisia / Manouba | Human | Random | Male | 55 | unknown | unknown | Random | B.1.1.7 | GRY |
| hCoV-19/Tunisia/Manouba/S-0144/2021 | EPI_ISL_16944227 | 29/03/2021 | Africa / Tunisia / Manouba | Human | Random | Female | 61 | unknown | unknown | Random | B.1.1.7 | GRY |
| hCoV-19/Tunisia/Manouba/S-0143/2021 | EPI_ISL_16944226 | 29/03/2021 | Africa / Tunisia / Manouba | Human | Random | Female | 42 | unknown | unknown | Random | B.1.1.7 | GRY |
| hCoV-19/Tunisia/Manouba/S-0142/2021 | EPI_ISL_16944225 | 29/03/2021 | Africa / Tunisia / Manouba | Human | Random | Male | 71 | unknown | unknown | Random | B.1.1.7 | GRY |
| hCoV-19/Tunisia/Manouba/S-0139/2021 | EPI_ISL_16944224 | 29/03/2021 | Africa / Tunisia / Manouba | Human | Random | Male | 14 | unknown | unknown | Random | B.1.1.7 | GRY |
| hCoV-19/Tunisia/Manouba/S-0138/2021 | EPI_ISL_16944223 | 29/03/2021 | Africa / Tunisia / Manouba | Human | Random | Male | 35 | unknown | unknown | Random | B.1.1.7 | GRY |
| hCoV-19/Tunisia/Manouba/S-0137/2021 | EPI_ISL_16944222 | 29/03/2021 | Africa / Tunisia / Manouba | Human | Random | Female | 42 | unknown | unknown | Random | B.1.1.7 | GRY |
| hCoV-19/Tunisia/Manouba/S-0135/2021 | EPI_ISL_16944221 | 29/03/2021 | Africa / Tunisia / Manouba | Human | Random | Female | 57 | unknown | unknown | Random | B.1.1.7 | GRY |
| hCoV-19/Tunisia/Manouba/S-0134/2021 | EPI_ISL_16944220 | 29/03/2021 | Africa / Tunisia / Manouba | Human | Random | Male | 60 | unknown | unknown | Random | B.1.1.7 | GRY |
| hCoV-19/Tunisia/Manouba/S-0133/2021 | EPI_ISL_16944219 | 29/03/2021 | Africa / Tunisia / Manouba | Human | Random | Male | 51 | unknown | unknown | Random | B.1.1.7 | GRY |
| hCoV-19/Tunisia/Manouba/S-0132/2021 | EPI_ISL_16944218 | 29/03/2021 | Africa / Tunisia / Manouba | Human | Random | Male | 53 | unknown | unknown | Random | B.1.1.7 | GRY |
| hCoV-19/Tunisia/Manouba/S-0131/2021 | EPI_ISL_16944217 | 29/03/2021 | Africa / Tunisia / Manouba | Human | Random | Female | 53 | unknown | unknown | Random | B.1.1.7 | GRY |
| hCoV-19/Tunisia/Manouba/S-0130/2021 | EPI_ISL_16944216 | 29/03/2021 | Africa / Tunisia / Manouba | Human | Random | Male | 43 | unknown | unknown | Random | B.1.1.7 | GRY |
| hCoV-19/Tunisia/Tunis/S-0099/2021 | EPI_ISL_16944215 | 30/03/2021 | Africa / Tunisia / Tunis | Human | Random | Female | unknown | unknown | unknown | Random | B.1.1.7 | GRY |
| hCoV-19/Tunisia/Tunis/S-0098/2021 | EPI_ISL_16944214 | 02/03/2021 | Africa / Tunisia / Tunis | Human | Random | Male | 33 | unknown | unknown | Random | B.1.1.7 | GRY |
| hCoV-19/Tunisia/Tunis/S-0095/2021 | EPI_ISL_16944213 | 12/03/2021 | Africa / Tunisia / Tunis | Human | Random | Female | 17 | unknown | unknown | Random | B.1.1.7 | GRY |
| hCoV-19/Tunisia/Sousse/S-0094/2021 | EPI_ISL_16944212 | 22/03/2021 | Africa / Tunisia / Sousse | Human | Random | Male | unknown | unknown | unknown | Random | B.1.1.7 | GRY |
| hCoV-19/Tunisia/Kebili/S-0088/2021 | EPI_ISL_16944211 | 11/03/2021 | Africa / Tunisia / Kebili | Human | Random | Female | 70 | unknown | unknown | Random | B.1.1.7 | GRY |
| hCoV-19/Tunisia/Kebili/S-0085/2021 | EPI_ISL_16944210 | 07/03/2021 | Africa / Tunisia / Kebili | Human | Random | Female | 32 | unknown | unknown | Random | B.1.1.7 | GRY |
| hCoV-19/Tunisia/Kebili/S-0084/2021 | EPI_ISL_16944209 | 06/03/2021 | Africa / Tunisia / Kebili | Human | Random | Female | 41 | unknown | unknown | Random | B.1.1.7 | GRY |
| hCoV-19/Tunisia/Kebili/S-0082/2021 | EPI_ISL_16944208 | 25/03/2021 | Africa / Tunisia / Kebili | Human | Random | Male | 33 | unknown | unknown | Random | B.1.1.7 | GRY |
| hCoV-19/Tunisia/Kebili/S-0079/2021 | EPI_ISL_16944207 | 25/03/2021 | Africa / Tunisia / Kebili | Human | Random | Male | 53 | unknown | unknown | Random | B.1.1.7 | GRY |
| hCoV-19/Tunisia/Ariana/S-0077/2021 | EPI_ISL_16944206 | 23/03/2021 | Africa / Tunisia / Ariana | Human | Random | Male | unknown | unknown | unknown | Random | B.1.1.7 | GRY |
| hCoV-19/Tunisia/Ariana/S-0076/2021 | EPI_ISL_16944205 | 23/03/2021 | Africa / Tunisia / Ariana | Human | Random | Female | unknown | unknown | unknown | Random | B.1.1.7 | GRY |
| hCoV-19/Tunisia/Ariana/S-0064/2021 | EPI_ISL_16944204 | 23/03/2021 | Africa / Tunisia / Ariana | Human | Random | Female | unknown | unknown | unknown | Random | B.1.1.7 | GRY |
| hCoV-19/Tunisia/Sfax/S-0050/2021 | EPI_ISL_16944203 | 13/03/2021 | Africa / Tunisia / Sfax | Human | Random | Male | 52 | unknown | unknown | Random | B.1.1.7 | GRY |
| hCoV-19/Tunisia/Sfax/S-0048/2021 | EPI_ISL_16944202 | 06/03/2021 | Africa / Tunisia / Sfax | Human | Random | Female | 48 | unknown | unknown | Random | B.1.1.7 | GRY |
| hCoV-19/Tunisia/Sfax/S-0047/2021 | EPI_ISL_16944201 | 11/03/2021 | Africa / Tunisia / Sfax | Human | Random | Male | 60 | unknown | unknown | Random | B.1.1.7 | GRY |
| hCoV-19/Tunisia/Sfax/S-0046/2021 | EPI_ISL_16944200 | 11/03/2021 | Africa / Tunisia / Sfax | Human | Random | Female | 28 | unknown | unknown | Random | B.1.1.7 | GRY |
| hCoV-19/Tunisia/Manouba/S-0025/2021 | EPI_ISL_16944199 | 11/03/2021 | Africa / Tunisia / Manouba | Human | Random | Male | 45 | unknown | unknown | Random | B.1.1.7 | GRY |
| hCoV-19/Tunisia/Tunis/D-7118/2021 | EPI_ISL_16944198 | 11/06/2021 | Africa / Tunisia / Tunis | Human | Random | Male | 18 | unknown | unknown | Random | B.1.1.7 | GR |
| hCoV-19/Tunisia/Tunis/D-6366/2021 | EPI_ISL_16944197 | 08/06/2021 | Africa / Tunisia / Tunis | Human | Random | Male | 58 | unknown | unknown | Random | B.1.1.7 | GRY |
| hCoV-19/Tunisia/Tunis/D-6356/2021 | EPI_ISL_16944196 | 08/06/2021 | Africa / Tunisia / Tunis | Human | Random | Female | 42 | Moderate | unknown | Random | B.1.1.7 | GRY |
| hCoV-19/Tunisia/Tunis/D-6306/2021 | EPI_ISL_16944195 | 08/06/2021 | Africa / Tunisia / Tunis | Human | Random | Female | 45 | Moderate | unknown | Random | B.1.1.7 | GRY |
| hCoV-19/Tunisia/Tunis/D-6299/2021 | EPI_ISL_16944193 | 08/06/2021 | Africa / Tunisia / Tunis | Human | Random | Female | 60 | Moderate | unknown | Random | B.1.1.7 | GRY |
| hCoV-19/Tunisia/Tunis/D-6282/2021 | EPI_ISL_16944192 | 08/06/2021 | Africa / Tunisia / Tunis | Human | Random | Female | 37 | Moderate | unknown | Random | B.1.1.7 | GRY |
| hCoV-19/Tunisia/Tunis/D-6279/2021 | EPI_ISL_16944191 | 08/06/2021 | Africa / Tunisia / Tunis | Human | Random | Male | 56 | Moderate | unknown | Random | B.1.1.7 | GRY |
| hCoV-19/Tunisia/Tunis/D-6265/2021 | EPI_ISL_16944190 | 08/06/2021 | Africa / Tunisia / Tunis | Human | Random | Female | 50 | Moderate | unknown | Random | B.1.1.7 | GRY |
| hCoV-19/Tunisia/Tunis/D-6077/2021 | EPI_ISL_16944189 | 07/06/2021 | Africa / Tunisia / Tunis | Human | Random | Male | 56 | unknown | unknown | Random | B.1.1.7 | GRY |
| hCoV-19/Tunisia/Tunis/D-6076/2021 | EPI_ISL_16944188 | 07/06/2021 | Africa / Tunisia / Tunis | Human | Random | Male | 35 | Moderate | unknown | Random | B.1.1.7 | GRY |
| hCoV-19/Tunisia/Tunis/D-6075/2021 | EPI_ISL_16944187 | 07/06/2021 | Africa / Tunisia / Tunis | Human | Random | Female | 54 | Moderate | unknown | Random | B.1.1.7 | GRY |
| hCoV-19/Tunisia/Tunis/D-6072/2021 | EPI_ISL_16944186 | 07/06/2021 | Africa / Tunisia / Tunis | Human | Random | Female | 33 | unknown | unknown | Random | B.1.1.7 | GRY |
| hCoV-19/Tunisia/Tunis/D-6071/2021 | EPI_ISL_16944185 | 07/06/2021 | Africa / Tunisia / Tunis | Human | Random | Male | 40 | Moderate | unknown | Random | B.1.1.7 | GRY |
| hCoV-19/Tunisia/Tunis/D-6070/2021 | EPI_ISL_16944184 | 07/06/2021 | Africa / Tunisia / Tunis | Human | Random | Male | 28 | Moderate | unknown | Random | B.1.1.7 | GRY |
| hCoV-19/Tunisia/Tunis/D-6069/2021 | EPI_ISL_16944183 | 07/06/2021 | Africa / Tunisia / Tunis | Human | Random | Male | 54 | Moderate | unknown | Random | B.1.1.7 | GRY |
| hCoV-19/Tunisia/Tunis/D-6061/2021 | EPI_ISL_16944182 | 07/06/2021 | Africa / Tunisia / Tunis | Human | Random | Male | 43 | Moderate | unknown | Random | B.1.1.7 | GRY |
| hCoV-19/Tunisia/Tunis/D-6057/2021 | EPI_ISL_16944181 | 07/06/2021 | Africa / Tunisia / Tunis | Human | Random | Female | 24 | Moderate | unknown | Random | B.1.1.7 | GRY |
| hCoV-19/Tunisia/Tunis/D-6055/2021 | EPI_ISL_16944180 | 07/06/2021 | Africa / Tunisia / Tunis | Human | Random | Male | 48 | Moderate | unknown | Random | B.1.1.7 | GRY |
| hCoV-19/Tunisia/Tunis/D-6051/2021 | EPI_ISL_16944179 | 07/06/2021 | Africa / Tunisia / Tunis | Human | Random | Female | 54 | unknown | unknown | Random | B.1.1.7 | GRY |
| hCoV-19/Tunisia/Tunis/D-6037/2021 | EPI_ISL_16944178 | 07/06/2021 | Africa / Tunisia / Tunis | Human | Random | Female | 32 | Moderate | unknown | Random | B.1.1.7 | GRY |
| hCoV-19/Tunisia/Tunis/D-6033/2021 | EPI_ISL_16944177 | 07/06/2021 | Africa / Tunisia / Tunis | Human | Random | Female | 45 | unknown | unknown | Random | B.1.1.7 | GRY |
| hCoV-19/Tunisia/Tunis/D-6025/2021 | EPI_ISL_16944176 | 07/06/2021 | Africa / Tunisia / Tunis | Human | Random | Male | 43 | Moderate | unknown | Random | B.1.1.7 | GRY |
| hCoV-19/Tunisia/Tunis/D-6022/2021 | EPI_ISL_16944175 | 07/06/2021 | Africa / Tunisia / Tunis | Human | Random | Female | 54 | unknown | unknown | Random | B.1.1.7 | GRY |
| hCoV-19/Tunisia/Tunis/D-6010/2021 | EPI_ISL_16944174 | 07/06/2021 | Africa / Tunisia / Tunis | Human | Random | Male | 35 | Moderate | unknown | Random | B.1.1.7 | GRY |
| hCoV-19/Tunisia/Tunis/D-6005/2021 | EPI_ISL_16944173 | 07/06/2021 | Africa / Tunisia / Tunis | Human | Random | Female | 33 | Moderate | unknown | Random | B.1.1.7 | GRY |
| hCoV-19/Tunisia/Tunis/D-5994/2021 | EPI_ISL_16944172 | 07/06/2021 | Africa / Tunisia / Tunis | Human | Random | Female | 44 | Moderate | unknown | Random | B.1.1.7 | GRY |
| hCoV-19/Tunisia/Tunis/D-5987/2021 | EPI_ISL_16944171 | 07/06/2021 | Africa / Tunisia / Tunis | Human | Random | Male | 17 | unknown | unknown | Random | B.1.1.7 | GRY |
| hCoV-19/Tunisia/Tunis/D-5978/2021 | EPI_ISL_16944170 | 07/06/2021 | Africa / Tunisia / Tunis | Human | Random | Female | 68 | Moderate | unknown | Random | B.1.1.7 | GRY |
| hCoV-19/Tunisia/Tunis/D-5965/2021 | EPI_ISL_16944169 | 07/06/2021 | Africa / Tunisia / Tunis | Human | Random | Male | 61 | Moderate | unknown | Random | B.1.1.7 | GRY |
| hCoV-19/Tunisia/Tunis/D-5963/2021 | EPI_ISL_16944168 | 07/06/2021 | Africa / Tunisia / Tunis | Human | Random | Male | 42 | Moderate | unknown | Random | B.1.1.7 | GRY |
| hCoV-19/Tunisia/Tunis/D-4853/2021 | EPI_ISL_16944167 | 01/06/2021 | Africa / Tunisia / Tunis | Human | Random | Female | 70 | Moderate | unknown | Random | B.1.1.7 | GRY |
| hCoV-19/Tunisia/Tunis/D-4849/2021 | EPI_ISL_16944166 | 01/06/2021 | Africa / Tunisia / Tunis | Human | Random | Female | 31 | Moderate | unknown | Random | B.1.1.7 | GRY |
| hCoV-19/Tunisia/Tunis/D-4840/2021 | EPI_ISL_16944165 | 01/06/2021 | Africa / Tunisia / Tunis | Human | Random | Female | 37 | Moderate | unknown | Random | B.1.1.7 | GRY |
| hCoV-19/Tunisia/Tunis/D-4836/2021 | EPI_ISL_16944164 | 01/06/2021 | Africa / Tunisia / Tunis | Human | Random | Male | 40 | Moderate | unknown | Random | B.1.1.7 | GRY |
| hCoV-19/Tunisia/Tunis/D-4834/2021 | EPI_ISL_16944163 | 01/06/2021 | Africa / Tunisia / Tunis | Human | Random | Female | 48 | unknown | unknown | Random | B.1.1.7 | GRY |
| hCoV-19/Tunisia/Tunis/D-4829/2021 | EPI_ISL_16944162 | 01/06/2021 | Africa / Tunisia / Tunis | Human | Random | Male | 23 | Moderate | unknown | Random | B.1.1.7 | GRY |
| hCoV-19/Tunisia/Tunis/D-4825/2021 | EPI_ISL_16944161 | 01/06/2021 | Africa / Tunisia / Tunis | Human | Random | Female | 30 | Moderate | unknown | Random | B.1.1.7 | GRY |
| hCoV-19/Tunisia/Tunis/D-4823/2021 | EPI_ISL_16944160 | 01/06/2021 | Africa / Tunisia / Tunis | Human | Random | Male | 41 | Moderate | unknown | Random | B.1.1.7 | GRY |
| hCoV-19/Tunisia/Tunis/D-4822/2021 | EPI_ISL_16944159 | 01/06/2021 | Africa / Tunisia / Tunis | Human | Random | Female | 39 | Moderate | unknown | Random | B.1.1.7 | GRY |
| hCoV-19/Tunisia/Tunis/D-4821/2021 | EPI_ISL_16944158 | 01/06/2021 | Africa / Tunisia / Tunis | Human | Random | Female | 23 | unknown | unknown | Random | B.1.1.7 | GRY |
| hCoV-19/Tunisia/Tunis/D-4815/2021 | EPI_ISL_16944157 | 01/06/2021 | Africa / Tunisia / Tunis | Human | Random | Female | 44 | unknown | unknown | Random | B.1.1.7 | GRY |
| hCoV-19/Tunisia/Tunis/D-4810/2021 | EPI_ISL_16944156 | 01/06/2021 | Africa / Tunisia / Tunis | Human | Random | Male | 22 | Moderate | unknown | Random | B.1.1.7 | GRY |
| hCoV-19/Tunisia/Tunis/D-4807/2021 | EPI_ISL_16944155 | 01/06/2021 | Africa / Tunisia / Tunis | Human | Random | Male | 53 | Moderate | unknown | Random | B.1.1.7 | GRY |
| hCoV-19/Tunisia/Tunis/D-4803/2021 | EPI_ISL_16944154 | 01/06/2021 | Africa / Tunisia / Tunis | Human | Random | Female | 21 | unknown | unknown | Random | B.1.1.7 | GRY |
| hCoV-19/Tunisia/Tunis/D-4802/2021 | EPI_ISL_16944153 | 01/06/2021 | Africa / Tunisia / Tunis | Human | Random | Female | 35 | unknown | unknown | Random | B.1.1.7 | GRY |
| hCoV-19/Tunisia/Tunis/D-4801/2021 | EPI_ISL_16944152 | 01/06/2021 | Africa / Tunisia / Tunis | Human | Random | Female | 49 | Moderate | unknown | Random | B.1.1.7 | GRY |
| hCoV-19/Tunisia/Tunis/D-4790/2021 | EPI_ISL_16944151 | 01/06/2021 | Africa / Tunisia / Tunis | Human | Random | Female | 51 | Moderate | unknown | Random | B.1.1.7 | GRY |
| hCoV-19/Tunisia/Tunis/D-4788/2021 | EPI_ISL_16944150 | 01/06/2021 | Africa / Tunisia / Tunis | Human | Random | Female | 40 | Moderate | unknown | Random | B.1.1.7 | GRY |
| hCoV-19/Tunisia/Tunis/D-4785/2021 | EPI_ISL_16944149 | 01/06/2021 | Africa / Tunisia / Tunis | Human | Random | Female | 31 | unknown | unknown | Random | B.1.1.7 | GRY |
| hCoV-19/Tunisia/Tunis/D-4777/2021 | EPI_ISL_16944148 | 01/06/2021 | Africa / Tunisia / Tunis | Human | Random | Male | 32 | Moderate | unknown | Random | B.1.1.7 | GRY |
| hCoV-19/Tunisia/Tunis/D-4770/2021 | EPI_ISL_16944147 | 01/06/2021 | Africa / Tunisia / Tunis | Human | Random | Female | 42 | Moderate | unknown | Random | B.1.1.7 | GRY |
| hCoV-19/Tunisia/Tunis/D-4761/2021 | EPI_ISL_16944146 | 01/06/2021 | Africa / Tunisia / Tunis | Human | Random | Male | 57 | Moderate | unknown | Random | B.1.1.7 | GRY |
| hCoV-19/Tunisia/Tunis/D-4757/2021 | EPI_ISL_16944145 | 01/06/2021 | Africa / Tunisia / Tunis | Human | Random | Male | 57 | Severe | unknown | Random | B.1.1.7 | GRY |
| hCoV-19/Tunisia/Tunis/D-4744/2021 | EPI_ISL_16944144 | 01/06/2021 | Africa / Tunisia / Tunis | Human | Random | Female | 38 | unknown | unknown | Random | B.1.1.7 | GRY |
| hCoV-19/Tunisia/Tunis/D-4741/2021 | EPI_ISL_16944143 | 01/06/2021 | Africa / Tunisia / Tunis | Human | Random | Male | 44 | Moderate | unknown | Random | B.1.1.7 | GRY |
| hCoV-19/Tunisia/Tunis/D-4739/2021 | EPI_ISL_16944142 | 01/06/2021 | Africa / Tunisia / Tunis | Human | Random | Female | 70 | Moderate | unknown | Random | B.1.1.7 | GRY |
| hCoV-19/Tunisia/Tunis/D-4737/2021 | EPI_ISL_16944141 | 01/06/2021 | Africa / Tunisia / Tunis | Human | Random | Male | 27 | unknown | unknown | Random | B.1.1.7 | GRY |
| hCoV-19/Tunisia/Tunis/D-4736/2021 | EPI_ISL_16944140 | 01/06/2021 | Africa / Tunisia / Tunis | Human | Random | Female | 42 | unknown | unknown | Random | B.1.1.7 | GRY |
| hCoV-19/Tunisia/Tunis/D-4727/2021 | EPI_ISL_16944139 | 01/06/2021 | Africa / Tunisia / Tunis | Human | Random | Female | 43 | unknown | unknown | Random | B.1.1.7 | GRY |
| hCoV-19/Tunisia/Tunis/D-4724/2021 | EPI_ISL_16944138 | 01/06/2021 | Africa / Tunisia / Tunis | Human | Random | Female | 33 | unknown | unknown | Random | B.1.1.7 | GRY |
| hCoV-19/Tunisia/Tunis/D-4722/2021 | EPI_ISL_16944137 | 01/06/2021 | Africa / Tunisia / Tunis | Human | Random | Male | 63 | Moderate | unknown | Random | B.1.1.7 | GRY |
| hCoV-19/Tunisia/Tunis/D-4720/2021 | EPI_ISL_16944136 | 01/06/2021 | Africa / Tunisia / Tunis | Human | Random | Female | 22 | unknown | unknown | Random | B.1.1.7 | GRY |
| hCoV-19/Tunisia/Tunis/D-4711/2021 | EPI_ISL_16944135 | 01/06/2021 | Africa / Tunisia / Tunis | Human | Random | Male | 52 | unknown | unknown | Random | B.1.1.7 | GRY |
| hCoV-19/Tunisia/Tunis/D-4693/2021 | EPI_ISL_16944134 | 01/06/2021 | Africa / Tunisia / Tunis | Human | Random | Male | 52 | unknown | unknown | Random | B.1.1.7 | GRY |
| hCoV-19/Tunisia/Tunis/D-4689/2021 | EPI_ISL_16944133 | 01/06/2021 | Africa / Tunisia / Tunis | Human | Random | Female | 55 | unknown | unknown | Random | B.1.1.7 | GRY |
| hCoV-19/Tunisia/Tunis/D-4684/2021 | EPI_ISL_16944132 | 01/06/2021 | Africa / Tunisia / Tunis | Human | Random | Female | 45 | unknown | unknown | Random | B.1.1.7 | GRY |
| hCoV-19/Tunisia/Tunis/D-4683/2021 | EPI_ISL_16944131 | 01/06/2021 | Africa / Tunisia / Tunis | Human | Random | Male | 50 | Moderate | unknown | Random | B.1.1.7 | GRY |
| hCoV-19/Tunisia/Tunis/D-4680/2021 | EPI_ISL_16944130 | 01/06/2021 | Africa / Tunisia / Tunis | Human | Random | Female | 63 | Severe | unknown | Random | B.1.1.7 | GRY |
| hCoV-19/Tunisia/Tunis/D-4676/2021 | EPI_ISL_16944129 | 01/06/2021 | Africa / Tunisia / Tunis | Human | Random | Male | 66 | unknown | unknown | Random | B.1.1.7 | GRY |
| hCoV-19/Tunisia/Tunis/D-4674/2021 | EPI_ISL_16944128 | 01/06/2021 | Africa / Tunisia / Tunis | Human | Random | Male | 57 | Moderate | unknown | Random | B.1.1.7 | GRY |
| hCoV-19/Tunisia/Tunis/D-4672/2021 | EPI_ISL_16944127 | 01/06/2021 | Africa / Tunisia / Tunis | Human | Random | Male | 67 | Moderate | unknown | Random | B.1.1.7 | GRY |
| hCoV-19/Tunisia/Tunis/D-4665/2021 | EPI_ISL_16944126 | 01/06/2021 | Africa / Tunisia / Tunis | Human | Random | Female | 31 | unknown | unknown | Random | B.1.1.7 | GRY |
| hCoV-19/Tunisia/Tunis/D-4144/2021 | EPI_ISL_16944125 | 28/05/2021 | Africa / Tunisia / Tunis | Human | Random | Female | 61 | Moderate | unknown | Random | B.1.1.7 | GRY |
| hCoV-19/Tunisia/Tunis/D-4121/2021 | EPI_ISL_16944124 | 28/05/2021 | Africa / Tunisia / Tunis | Human | Random | Female | 38 | Moderate | unknown | Random | B.1.1.7 | GRY |
| hCoV-19/Tunisia/Tunis/D-4111/2021 | EPI_ISL_16944123 | 28/05/2021 | Africa / Tunisia / Tunis | Human | Random | Male | 31 | Moderate | unknown | Random | B.1.1.7 | GRY |
| hCoV-19/Tunisia/Tunis/D-4074/2021 | EPI_ISL_16944122 | 28/05/2021 | Africa / Tunisia / Tunis | Human | Random | Male | 44 | unknown | unknown | Random | B.1.1.7 | GRY |
| hCoV-19/Tunisia/Tunis/D-4069/2021 | EPI_ISL_16944121 | 28/05/2021 | Africa / Tunisia / Tunis | Human | Random | Female | 31 | Moderate | unknown | Random | B.1.1.7 | GRY |
| hCoV-19/Tunisia/Tunis/D-4065/2021 | EPI_ISL_16944120 | 28/05/2021 | Africa / Tunisia / Tunis | Human | Random | Female | 56 | Moderate | unknown | Random | B.1.1.7 | GRY |
| hCoV-19/Tunisia/Tunis/D-4062/2021 | EPI_ISL_16944119 | 28/05/2021 | Africa / Tunisia / Tunis | Human | Random | Female | 33 | Moderate | unknown | Random | B.1.1.7 | GRY |
| hCoV-19/Tunisia/Tunis/D-4060/2021 | EPI_ISL_16944118 | 28/05/2021 | Africa / Tunisia / Tunis | Human | Random | Female | 25 | unknown | unknown | Random | B.1.1.7 | GRY |
| hCoV-19/Tunisia/Tunis/D-4055/2021 | EPI_ISL_16944117 | 28/05/2021 | Africa / Tunisia / Tunis | Human | Random | Female | 59 | Moderate | unknown | Random | B.1.1.7 | GRY |
| hCoV-19/Tunisia/Tunis/D-4054/2021 | EPI_ISL_16944116 | 28/05/2021 | Africa / Tunisia / Tunis | Human | Random | Female | 60 | Moderate | unknown | Random | B.1.1.7 | GRY |
| hCoV-19/Tunisia/Tunis/D-4026/2021 | EPI_ISL_16944115 | 28/05/2021 | Africa / Tunisia / Tunis | Human | Random | Male | 60 | unknown | unknown | Random | B.1.1.7 | GRY |
| hCoV-19/Tunisia/Tunis/D-4020/2021 | EPI_ISL_16944114 | 28/05/2021 | Africa / Tunisia / Tunis | Human | Random | Female | 23 | Moderate | unknown | Random | B.1.1.7 | GRY |
| hCoV-19/Tunisia/Tunis/D-4018/2021 | EPI_ISL_16944113 | 28/05/2021 | Africa / Tunisia / Tunis | Human | Random | Male | 34 | Moderate | unknown | Random | B.1.1.7 | GRY |
| hCoV-19/Tunisia/Tunis/D-4014/2021 | EPI_ISL_16944112 | 28/05/2021 | Africa / Tunisia / Tunis | Human | Random | Male | 43 | Moderate | unknown | Random | B.1.1.7 | GRY |
| hCoV-19/Tunisia/Tunis/D-4009/2021 | EPI_ISL_16944111 | 28/05/2021 | Africa / Tunisia / Tunis | Human | Random | Female | 21 | Moderate | unknown | Random | B.1.1.7 | GRY |
| hCoV-19/Tunisia/Tunis/D-4008/2021 | EPI_ISL_16944110 | 28/05/2021 | Africa / Tunisia / Tunis | Human | Random | Male | 32 | Moderate | unknown | Random | B.1.1.7 | GRY |
| hCoV-19/Tunisia/Tunis/D-4006/2021 | EPI_ISL_16944109 | 28/05/2021 | Africa / Tunisia / Tunis | Human | Random | Male | 29 | Moderate | unknown | Random | B.1.1.7 | GRY |
| hCoV-19/Tunisia/Tunis/D-3979/2021 | EPI_ISL_16944108 | 28/05/2021 | Africa / Tunisia / Tunis | Human | Random | Female | 30 | Moderate | unknown | Random | B.1.1.7 | GRY |
| hCoV-19/Tunisia/Tunis/D-3941/2021 | EPI_ISL_16944107 | 27/05/2021 | Africa / Tunisia / Tunis | Human | Random | Male | 33 | Moderate | unknown | Random | B.1.1.7 | GRY |
| hCoV-19/Tunisia/Tunis/D-3939/2021 | EPI_ISL_16944106 | 27/05/2021 | Africa / Tunisia / Tunis | Human | Random | Male | 26 | Moderate | unknown | Random | B.1.1.7 | GRY |
| hCoV-19/Tunisia/Tunis/D-3936/2021 | EPI_ISL_16944105 | 27/05/2021 | Africa / Tunisia / Tunis | Human | Random | Female | 32 | Moderate | unknown | Random | B.1.1.7 | GRY |
| hCoV-19/Tunisia/Tunis/D-3933/2021 | EPI_ISL_16944104 | 27/05/2021 | Africa / Tunisia / Tunis | Human | Random | Female | 39 | Moderate | unknown | Random | B.1.1.7 | GRY |
| hCoV-19/Tunisia/Tunis/D-3932/2021 | EPI_ISL_16944103 | 27/05/2021 | Africa / Tunisia / Tunis | Human | Random | Female | 43 | Moderate | unknown | Random | B.1.1.7 | GRY |
| hCoV-19/Tunisia/Tunis/D-3924/2021 | EPI_ISL_16944102 | 27/05/2021 | Africa / Tunisia / Tunis | Human | Random | Female | 43 | Moderate | unknown | Random | B.1.1.7 | GRY |
| hCoV-19/Tunisia/Tunis/D-3921/2021 | EPI_ISL_16944101 | 27/05/2021 | Africa / Tunisia / Tunis | Human | Random | Male | 40 | Moderate | unknown | Random | B.1.1.7 | GR |
| hCoV-19/Tunisia/Tunis/D-3920/2021 | EPI_ISL_16944100 | 27/05/2021 | Africa / Tunisia / Tunis | Human | Random | Male | 15 | Moderate | unknown | Random | B.1.1.7 | GRY |
| hCoV-19/Tunisia/Tunis/D-3918/2021 | EPI_ISL_16944099 | 27/05/2021 | Africa / Tunisia / Tunis | Human | Random | Female | 40 | Moderate | unknown | Random | B.1.1.7 | GRY |
| hCoV-19/Tunisia/Tunis/D-3917/2021 | EPI_ISL_16944098 | 27/05/2021 | Africa / Tunisia / Tunis | Human | Random | Male | 34 | Moderate | unknown | Random | B.1.1.7 | GRY |
| hCoV-19/Tunisia/Tunis/D-3916/2021 | EPI_ISL_16944097 | 27/05/2021 | Africa / Tunisia / Tunis | Human | Random | Male | 49 | Moderate | unknown | Random | B.1.1.7 | GRY |
| hCoV-19/Tunisia/Tunis/D-3915/2021 | EPI_ISL_16944096 | 27/05/2021 | Africa / Tunisia / Tunis | Human | Random | Male | 34 | Moderate | unknown | Random | B.1.1.7 | GRY |
| hCoV-19/Tunisia/Tunis/D-3909/2021 | EPI_ISL_16944095 | 27/05/2021 | Africa / Tunisia / Tunis | Human | Random | Female | 35 | Moderate | unknown | Random | B.1.1.7 | GRY |
| hCoV-19/Tunisia/Tunis/D-3908/2021 | EPI_ISL_16944094 | 27/05/2021 | Africa / Tunisia / Tunis | Human | Random | Female | 38 | Moderate | unknown | Random | B.1.1.7 | GRY |
| hCoV-19/Tunisia/Tunis/D-3907/2021 | EPI_ISL_16944093 | 27/05/2021 | Africa / Tunisia / Tunis | Human | Random | Male | 40 | Moderate | unknown | Random | B.1.1.7 | GRY |
| hCoV-19/Tunisia/Tunis/D-3903/2021 | EPI_ISL_16944092 | 27/05/2021 | Africa / Tunisia / Tunis | Human | Random | Female | 28 | Moderate | unknown | Random | B.1.1.7 | GRY |
| hCoV-19/Tunisia/Tunis/D-3902/2021 | EPI_ISL_16944091 | 27/05/2021 | Africa / Tunisia / Tunis | Human | Random | Female | 20 | Moderate | unknown | Random | B.1.1.7 | GRY |
| hCoV-19/Tunisia/Tunis/D-3898/2021 | EPI_ISL_16944090 | 27/05/2021 | Africa / Tunisia / Tunis | Human | Random | Female | 28 | Moderate | unknown | Random | B.1.1.7 | GRY |
| hCoV-19/Tunisia/Tunis/D-3897/2021 | EPI_ISL_16944089 | 27/05/2021 | Africa / Tunisia / Tunis | Human | Random | Female | 34 | unknown | unknown | Random | B.1.1.7 | GRY |
| hCoV-19/Tunisia/Tunis/D-3896/2021 | EPI_ISL_16944088 | 27/05/2021 | Africa / Tunisia / Tunis | Human | Random | Female | 23 | Moderate | unknown | Random | B.1.1.7 | GRY |
| hCoV-19/Tunisia/Tunis/D-3892/2021 | EPI_ISL_16944087 | 27/05/2021 | Africa / Tunisia / Tunis | Human | Random | Female | 59 | Moderate | unknown | Random | B.1.1.7 | GRY |
| hCoV-19/Tunisia/Tunis/D-3888/2021 | EPI_ISL_16944086 | 27/05/2021 | Africa / Tunisia / Tunis | Human | Random | Female | 69 | Severe | unknown | Random | B.1.1.7 | GRY |
| hCoV-19/Tunisia/Tunis/D-3880/2021 | EPI_ISL_16944085 | 27/05/2021 | Africa / Tunisia / Tunis | Human | Random | Female | 24 | Moderate | unknown | Random | B.1.1.7 | GRY |
| hCoV-19/Tunisia/Tunis/D-3876/2021 | EPI_ISL_16944084 | 27/05/2021 | Africa / Tunisia / Tunis | Human | Random | Male | 19 | Moderate | unknown | Random | B.1.1.7 | GRY |
| hCoV-19/Tunisia/Tunis/D-3874/2021 | EPI_ISL_16944083 | 27/05/2021 | Africa / Tunisia / Tunis | Human | Random | Male | 61 | Asymptomatic | unknown | Random | B.1.1.7 | GRY |
| hCoV-19/Tunisia/Tunis/D-3873/2021 | EPI_ISL_16944082 | 27/05/2021 | Africa / Tunisia / Tunis | Human | Random | Female | 64 | Moderate | unknown | Random | B.1.1.7 | GRY |
| hCoV-19/Tunisia/Tunis/D-3867/2021 | EPI_ISL_16944081 | 27/05/2021 | Africa / Tunisia / Tunis | Human | Random | Female | 73 | Moderate | unknown | Random | B.1.1.7 | GRY |
| hCoV-19/Tunisia/Tunis/D-3866/2021 | EPI_ISL_16944080 | 27/05/2021 | Africa / Tunisia / Tunis | Human | Random | Female | 63 | unknown | unknown | Random | B.1.1.7 | GRY |
| hCoV-19/Tunisia/Tunis/D-3864/2021 | EPI_ISL_16944079 | 27/05/2021 | Africa / Tunisia / Tunis | Human | Random | Female | 86 | Moderate | unknown | Random | B.1.1.7 | GRY |
| hCoV-19/Tunisia/Tunis/D-3861/2021 | EPI_ISL_16944078 | 27/05/2021 | Africa / Tunisia / Tunis | Human | Random | Female | 41 | Moderate | unknown | Random | B.1.1.7 | GRY |
| hCoV-19/Tunisia/Tunis/D-3859/2021 | EPI_ISL_16944077 | 27/05/2021 | Africa / Tunisia / Tunis | Human | Random | Male | 58 | Moderate | unknown | Random | B.1.1.7 | GRY |
| hCoV-19/Tunisia/Tunis/D-3855/2021 | EPI_ISL_16944076 | 27/05/2021 | Africa / Tunisia / Tunis | Human | Random | Male | 1 | Moderate | unknown | Random | B.1.1.7 | GRY |
| hCoV-19/Tunisia/Tunis/D-3852/2021 | EPI_ISL_16944075 | 27/05/2021 | Africa / Tunisia / Tunis | Human | Random | Female | 43 | Moderate | unknown | Random | B.1.1.7 | GRY |
| hCoV-19/Tunisia/Tunis/D-3851/2021 | EPI_ISL_16944074 | 27/05/2021 | Africa / Tunisia / Tunis | Human | Random | Female | 58 | Moderate | unknown | Random | B.1.1.7 | GRY |
| hCoV-19/Tunisia/Tunis/D-3848/2021 | EPI_ISL_16944073 | 27/05/2021 | Africa / Tunisia / Tunis | Human | Random | Male | 27 | Moderate | unknown | Random | B.1.1.7 | GRY |
| hCoV-19/Tunisia/Tunis/D-3844/2021 | EPI_ISL_16944072 | 27/05/2021 | Africa / Tunisia / Tunis | Human | Random | Male | 42 | unknown | unknown | Random | B.1.1.7 | GRY |
| hCoV-19/Tunisia/Tunis/D-3811/2021 | EPI_ISL_16944071 | 27/05/2021 | Africa / Tunisia / Tunis | Human | Random | Male | 66 | unknown | unknown | Random | B.1.1.7 | GR |
| hCoV-19/Tunisia/Tunis/D-3808/2021 | EPI_ISL_16944070 | 27/05/2021 | Africa / Tunisia / Tunis | Human | Random | Female | 55 | unknown | unknown | Random | B.1.1.7 | GRY |
| hCoV-19/Tunisia/Tunis/D-3807/2021 | EPI_ISL_16944069 | 27/05/2021 | Africa / Tunisia / Tunis | Human | Random | Male | 77 | unknown | unknown | Random | B.1.1.7 | GRY |
| hCoV-19/Tunisia/Tunis/D-3786/2021 | EPI_ISL_16944068 | 26/05/2021 | Africa / Tunisia / Tunis | Human | Random | Male | 35 | Moderate | unknown | Random | B.1.1.7 | GRY |
| hCoV-19/Tunisia/Tunis/D-3784/2021 | EPI_ISL_16944067 | 26/05/2021 | Africa / Tunisia / Tunis | Human | Random | Female | 35 | Moderate | unknown | Random | B.1.1.7 | GRY |
| hCoV-19/Tunisia/Tunis/D-3779/2021 | EPI_ISL_16944066 | 26/05/2021 | Africa / Tunisia / Tunis | Human | Random | Female | 41 | Moderate | unknown | Random | B.1.1.7 | GRY |
| hCoV-19/Tunisia/Tunis/D-3774/2021 | EPI_ISL_16944065 | 26/05/2021 | Africa / Tunisia / Tunis | Human | Random | Female | 25 | unknown | unknown | Random | B.1.1.7 | GRY |
| hCoV-19/Tunisia/Tunis/D-3768/2021 | EPI_ISL_16944064 | 26/05/2021 | Africa / Tunisia / Tunis | Human | Random | Female | 37 | Moderate | unknown | Random | B.1.1.7 | GRY |
| hCoV-19/Tunisia/Tunis/D-3760/2021 | EPI_ISL_16944063 | 26/05/2021 | Africa / Tunisia / Tunis | Human | Random | Male | 33 | unknown | unknown | Random | B.1.1.7 | GRY |
| hCoV-19/Tunisia/Tunis/D-3756/2021 | EPI_ISL_16944062 | 26/05/2021 | Africa / Tunisia / Tunis | Human | Random | Male | 33 | unknown | unknown | Random | B.1.1.7 | GRY |
| hCoV-19/Tunisia/Tunis/D-3752/2021 | EPI_ISL_16944061 | 26/05/2021 | Africa / Tunisia / Tunis | Human | Random | Female | 49 | Moderate | unknown | Random | B.1.1.7 | GRY |
| hCoV-19/Tunisia/Tunis/D-3746/2021 | EPI_ISL_16944060 | 26/05/2021 | Africa / Tunisia / Tunis | Human | Random | Male | 40 | Moderate | unknown | Random | B.1.1.7 | GRY |
| hCoV-19/Tunisia/Tunis/D-3745/2021 | EPI_ISL_16944059 | 26/05/2021 | Africa / Tunisia / Tunis | Human | Random | Female | 50 | Moderate | unknown | Random | B.1.1.7 | GRY |
| hCoV-19/Tunisia/Tunis/D-3738/2021 | EPI_ISL_16944058 | 26/05/2021 | Africa / Tunisia / Tunis | Human | Random | Female | 57 | Moderate | unknown | Random | B.1.1.7 | GRY |
| hCoV-19/Tunisia/Tunis/D-3737/2021 | EPI_ISL_16944057 | 26/05/2021 | Africa / Tunisia / Tunis | Human | Random | Female | 45 | unknown | unknown | Random | B.1.1.7 | GRY |
| hCoV-19/Tunisia/Tunis/D-3726/2021 | EPI_ISL_16944056 | 26/05/2021 | Africa / Tunisia / Tunis | Human | Random | Male | 63 | unknown | unknown | Random | B.1.1.7 | GRY |
| hCoV-19/Tunisia/Tunis/D-3725/2021 | EPI_ISL_16944055 | 26/05/2021 | Africa / Tunisia / Tunis | Human | Random | Female | 80 | Moderate | unknown | Random | B.1.1.7 | GRY |
| hCoV-19/Tunisia/Tunis/D-3716/2021 | EPI_ISL_16944054 | 26/05/2021 | Africa / Tunisia / Tunis | Human | Random | Female | 24 | Moderate | unknown | Random | B.1.1.7 | GRY |
| hCoV-19/Tunisia/Tunis/D-3711/2021 | EPI_ISL_16944053 | 26/05/2021 | Africa / Tunisia / Tunis | Human | Random | Female | 57 | Moderate | unknown | Random | B.1.1.7 | GRY |
| hCoV-19/Tunisia/Tunis/D-3691/2021 | EPI_ISL_16944052 | 26/05/2021 | Africa / Tunisia / Tunis | Human | Random | Male | 36 | Moderate | unknown | Random | B.1.1.7 | GRY |
| hCoV-19/Tunisia/Tunis/D-3688/2021 | EPI_ISL_16944051 | 26/05/2021 | Africa / Tunisia / Tunis | Human | Random | Male | 36 | Moderate | unknown | Random | B.1.1.7 | GRY |
| hCoV-19/Tunisia/Tunis/D-3687/2021 | EPI_ISL_16944050 | 26/05/2021 | Africa / Tunisia / Tunis | Human | Random | Female | 32 | unknown | unknown | Random | B.1.1.7 | GRY |
| hCoV-19/Tunisia/Tunis/D-3682/2021 | EPI_ISL_16944049 | 26/05/2021 | Africa / Tunisia / Tunis | Human | Random | Male | 81 | unknown | unknown | Random | B.1.1.7 | GRY |
| hCoV-19/Tunisia/Tunis/D-3680/2021 | EPI_ISL_16944048 | 26/05/2021 | Africa / Tunisia / Tunis | Human | Random | Female | 71 | unknown | unknown | Random | B.1.1.7 | GRY |
| hCoV-19/Tunisia/Tunis/D-3679/2021 | EPI_ISL_16944047 | 26/05/2021 | Africa / Tunisia / Tunis | Human | Random | Male | 60 | Moderate | unknown | Random | B.1.1.7 | GRY |
| hCoV-19/Tunisia/Tunis/D-3678/2021 | EPI_ISL_16944046 | 26/05/2021 | Africa / Tunisia / Tunis | Human | Random | Male | 65 | Moderate | unknown | Random | B.1.1.7 | GRY |
| hCoV-19/Tunisia/Tunis/D-3677/2021 | EPI_ISL_16944045 | 26/05/2021 | Africa / Tunisia / Tunis | Human | Random | Female | 49 | Moderate | unknown | Random | B.1.1.7 | GRY |
| hCoV-19/Tunisia/Tunis/D-3676/2021 | EPI_ISL_16944044 | 26/05/2021 | Africa / Tunisia / Tunis | Human | Random | Female | 85 | Moderate | unknown | Random | B.1.1.7 | GRY |
| hCoV-19/Tunisia/Tunis/D-3674/2021 | EPI_ISL_16944043 | 26/05/2021 | Africa / Tunisia / Tunis | Human | Random | Female | 29 | unknown | unknown | Random | B.1.1.7 | GRY |
| hCoV-19/Tunisia/Tunis/D-3672/2021 | EPI_ISL_16944042 | 26/05/2021 | Africa / Tunisia / Tunis | Human | Random | Female | 50 | Moderate | unknown | Random | B.1.1.7 | GRY |
| hCoV-19/Tunisia/Tunis/D-3671/2021 | EPI_ISL_16944041 | 26/05/2021 | Africa / Tunisia / Tunis | Human | Random | Female | 53 | Moderate | unknown | Random | B.1.1.7 | GH |
| hCoV-19/Tunisia/Tunis/D-3667/2021 | EPI_ISL_16944040 | 26/05/2021 | Africa / Tunisia / Tunis | Human | Random | Female | 31 | unknown | unknown | Random | B.1.1.7 | GRY |
| hCoV-19/Tunisia/Tunis/D-3664/2021 | EPI_ISL_16944039 | 26/05/2021 | Africa / Tunisia / Tunis | Human | Random | Female | 31 | Moderate | unknown | Random | B.1.1.7 | GRY |
| hCoV-19/Tunisia/Tunis/D-3663/2021 | EPI_ISL_16944038 | 26/05/2021 | Africa / Tunisia / Tunis | Human | Random | Female | 37 | unknown | unknown | Random | B.1.1.7 | GRY |
| hCoV-19/Tunisia/Tunis/D-3654/2021 | EPI_ISL_16944037 | 26/05/2021 | Africa / Tunisia / Tunis | Human | Random | Male | 35 | Moderate | unknown | Random | B.1.1.7 | GRY |
| hCoV-19/Tunisia/Tunis/D-3650/2021 | EPI_ISL_16944036 | 26/05/2021 | Africa / Tunisia / Tunis | Human | Random | Male | 68 | Moderate | unknown | Random | B.1.1.7 | GRY |
| hCoV-19/Tunisia/Tunis/D-3648/2021 | EPI_ISL_16944035 | 26/05/2021 | Africa / Tunisia / Tunis | Human | Random | Female | 32 | Moderate | unknown | Random | B.1.1.7 | GRY |
| hCoV-19/Tunisia/Tunis/D-3641/2021 | EPI_ISL_16944034 | 26/05/2021 | Africa / Tunisia / Tunis | Human | Random | Male | 30 | unknown | unknown | Random | B.1.1.7 | GRY |
| hCoV-19/Tunisia/Tunis/D-3632/2021 | EPI_ISL_16944033 | 26/05/2021 | Africa / Tunisia / Tunis | Human | Random | Male | 52 | Moderate | unknown | Random | B.1.1.7 | GRY |
| hCoV-19/Tunisia/Tunis/D-3631/2021 | EPI_ISL_16944032 | 26/05/2021 | Africa / Tunisia / Tunis | Human | Random | Female | 20 | Moderate | unknown | Random | B.1.1.7 | GRY |
| hCoV-19/Tunisia/Tunis/D-3623/2021 | EPI_ISL_16944031 | 26/05/2021 | Africa / Tunisia / Tunis | Human | Random | Female | 28 | Moderate | unknown | Random | B.1.1.7 | GRY |
| hCoV-19/Tunisia/Tunis/D-3613/2021 | EPI_ISL_16944030 | 26/05/2021 | Africa / Tunisia / Tunis | Human | Random | Female | 41 | Moderate | unknown | Random | B.1.1.7 | GRY |
| hCoV-19/Tunisia/Tunis/D-3611/2021 | EPI_ISL_16944029 | 26/05/2021 | Africa / Tunisia / Tunis | Human | Random | Female | 42 | unknown | unknown | Random | B.1.1.7 | GRY |
| hCoV-19/Tunisia/Tunis/D-3603/2021 | EPI_ISL_16944028 | 26/05/2021 | Africa / Tunisia / Tunis | Human | Random | Male | 73 | unknown | unknown | Random | B.1.1.7 | GRY |
| hCoV-19/Tunisia/Tunis/D-3588/2021 | EPI_ISL_16944027 | 25/05/2021 | Africa / Tunisia / Tunis | Human | Random | Male | 30 | Moderate | unknown | Random | B.1.1.7 | GRY |
| hCoV-19/Tunisia/Tunis/D-3585/2021 | EPI_ISL_16944026 | 25/05/2021 | Africa / Tunisia / Tunis | Human | Random | Female | 32 | unknown | unknown | Random | B.1.1.7 | GRY |
| hCoV-19/Tunisia/Tunis/D-3584/2021 | EPI_ISL_16944025 | 25/05/2021 | Africa / Tunisia / Tunis | Human | Random | Male | 36 | unknown | unknown | Random | B.1.1.7 | GRY |
| hCoV-19/Tunisia/Tunis/D-3567/2021 | EPI_ISL_16944024 | 25/05/2021 | Africa / Tunisia / Tunis | Human | Random | Female | 36 | unknown | unknown | Random | B.1.1.7 | GRY |
| hCoV-19/Tunisia/Tunis/D-3564/2021 | EPI_ISL_16944023 | 25/05/2021 | Africa / Tunisia / Tunis | Human | Random | Male | 54 | Moderate | unknown | Random | B.1.1.7 | GRY |
| hCoV-19/Tunisia/Tunis/D-3563/2021 | EPI_ISL_16944022 | 25/05/2021 | Africa / Tunisia / Tunis | Human | Random | Female | 54 | Moderate | unknown | Random | B.1.1.7 | GRY |
| hCoV-19/Tunisia/Tunis/D-3561/2021 | EPI_ISL_16944021 | 25/05/2021 | Africa / Tunisia / Tunis | Human | Random | Female | 24 | Moderate | unknown | Random | B.1.1.7 | GRY |
| hCoV-19/Tunisia/Tunis/D-3544/2021 | EPI_ISL_16944020 | 25/05/2021 | Africa / Tunisia / Tunis | Human | Random | Female | 85 | Moderate | unknown | Random | B.1.1.7 | GRY |
| hCoV-19/Tunisia/Tunis/D-3540/2021 | EPI_ISL_16944019 | 25/05/2021 | Africa / Tunisia / Tunis | Human | Random | Female | 23 | unknown | unknown | Random | B.1.1.7 | GRY |
| hCoV-19/Tunisia/Tunis/D-3533/2021 | EPI_ISL_16944018 | 25/05/2021 | Africa / Tunisia / Tunis | Human | Random | Male | 38 | Moderate | unknown | Random | B.1.1.7 | GRY |
| hCoV-19/Tunisia/Tunis/D-3527/2021 | EPI_ISL_16944017 | 25/05/2021 | Africa / Tunisia / Tunis | Human | Random | Female | 46 | Moderate | unknown | Random | B.1.1.7 | GRY |
| hCoV-19/Tunisia/Tunis/D-3525/2021 | EPI_ISL_16944016 | 25/05/2021 | Africa / Tunisia / Tunis | Human | Random | Female | 58 | Moderate | unknown | Random | B.1.1.7 | GRY |
| hCoV-19/Tunisia/Tunis/D-3524/2021 | EPI_ISL_16944015 | 25/05/2021 | Africa / Tunisia / Tunis | Human | Random | Male | 29 | Moderate | unknown | Random | B.1.1.7 | GRY |
| hCoV-19/Tunisia/Tunis/D-3523/2021 | EPI_ISL_16944014 | 25/05/2021 | Africa / Tunisia / Tunis | Human | Random | Male | 25 | Moderate | unknown | Random | B.1.1.7 | GRY |
| hCoV-19/Tunisia/Tunis/D-3522/2021 | EPI_ISL_16944013 | 25/05/2021 | Africa / Tunisia / Tunis | Human | Random | Male | 25 | Moderate | unknown | Random | B.1.1.7 | GRY |
| hCoV-19/Tunisia/Tunis/D-3521/2021 | EPI_ISL_16944012 | 25/05/2021 | Africa / Tunisia / Tunis | Human | Random | Female | 32 | unknown | unknown | Random | B.1.1.7 | GRY |
| hCoV-19/Tunisia/Tunis/D-3507/2021 | EPI_ISL_16944011 | 25/05/2021 | Africa / Tunisia / Tunis | Human | Random | Female | 53 | unknown | unknown | Random | B.1.1.7 | GRY |
| hCoV-19/Tunisia/Tunis/D-3506/2021 | EPI_ISL_16944010 | 25/05/2021 | Africa / Tunisia / Tunis | Human | Random | Male | 53 | Moderate | unknown | Random | B.1.1.7 | GRY |
| hCoV-19/Tunisia/Tunis/D-3505/2021 | EPI_ISL_16944009 | 25/05/2021 | Africa / Tunisia / Tunis | Human | Random | Female | 37 | unknown | unknown | Random | B.1.1.7 | GRY |
| hCoV-19/Tunisia/Tunis/D-3499/2021 | EPI_ISL_16944008 | 25/05/2021 | Africa / Tunisia / Tunis | Human | Random | Female | 57 | Moderate | unknown | Random | B.1.1.7 | GRY |
| hCoV-19/Tunisia/Tunis/D-3497/2021 | EPI_ISL_16944007 | 25/05/2021 | Africa / Tunisia / Tunis | Human | Random | Female | 29 | Moderate | unknown | Random | B.1.1.7 | GRY |
| hCoV-19/Tunisia/Tunis/D-3486/2021 | EPI_ISL_16944006 | 25/05/2021 | Africa / Tunisia / Tunis | Human | Random | Male | 27 | unknown | unknown | Random | B.1.1.7 | GRY |
| hCoV-19/Tunisia/Tunis/D-3471/2021 | EPI_ISL_16944005 | 25/05/2021 | Africa / Tunisia / Tunis | Human | Random | Male | 19 | unknown | unknown | Random | B.1.1.7 | GRY |
| hCoV-19/Tunisia/Tunis/D-3468/2021 | EPI_ISL_16944004 | 25/05/2021 | Africa / Tunisia / Tunis | Human | Random | Female | 44 | unknown | unknown | Random | B.1.1.7 | GRY |
| hCoV-19/Tunisia/Tunis/D-3461/2021 | EPI_ISL_16944003 | 25/05/2021 | Africa / Tunisia / Tunis | Human | Random | Female | 34 | unknown | unknown | Random | B.1.1.7 | GRY |
| hCoV-19/Tunisia/Tunis/D-3460/2021 | EPI_ISL_16944002 | 25/05/2021 | Africa / Tunisia / Tunis | Human | Random | Female | 42 | unknown | unknown | Random | B.1.1.7 | GRY |
| hCoV-19/Tunisia/Tunis/D-3458/2021 | EPI_ISL_16944001 | 25/05/2021 | Africa / Tunisia / Tunis | Human | Random | Male | 63 | unknown | unknown | Random | B.1.1.7 | GRY |
| hCoV-19/Tunisia/Tunis/D-3440/2021 | EPI_ISL_16944000 | 25/05/2021 | Africa / Tunisia / Tunis | Human | Random | Female | 50 | unknown | unknown | Random | B.1.1.7 | GRY |
| hCoV-19/Tunisia/Tunis/D-3435/2021 | EPI_ISL_16943999 | 25/05/2021 | Africa / Tunisia / Tunis | Human | Random | Male | 52 | Moderate | unknown | Random | B.1.1.7 | GRY |
| hCoV-19/Tunisia/Tunis/D-3434/2021 | EPI_ISL_16943998 | 25/05/2021 | Africa / Tunisia / Tunis | Human | Random | Male | 52 | Moderate | unknown | Random | B.1.1.7 | GRY |
| hCoV-19/Tunisia/Tunis/D-3421/2021 | EPI_ISL_16943997 | 25/05/2021 | Africa / Tunisia / Tunis | Human | Random | Male | 22 | Moderate | unknown | Random | B.1.1.7 | GRY |
| hCoV-19/Tunisia/Tunis/D-3224/2021 | EPI_ISL_16943996 | 24/05/2021 | Africa / Tunisia / Tunis | Human | Random | Female | 22 | unknown | unknown | Random | B.1.1.7 | GRY |
| hCoV-19/Tunisia/Tunis/D-3222/2021 | EPI_ISL_16943995 | 24/05/2021 | Africa / Tunisia / Tunis | Human | Random | Female | 53 | Moderate | unknown | Random | B.1.1.7 | GRY |
| hCoV-19/Tunisia/Tunis/B-1988/2021 | EPI_ISL_16943982 | 11/03/2021 | Africa / Tunisia / Tunis | Human | Random | Female | 56 | Moderate | unknown | Random | B.1.1.7 | GRY |
| hCoV-19/Tunisia/Tunis/B-1547/2021 | EPI_ISL_16943981 | 10/03/2021 | Africa / Tunisia / Tunis | Human | Random | Female | 28 | Moderate | unknown | Random | B.1.1.7 | GRY |
| hCoV-19/Tunisia/Tunis/B-1319/2021 | EPI_ISL_16943980 | 09/03/2021 | Africa / Tunisia / Tunis | Human | Random | Female | 27 | Moderate | unknown | Random | B.1.1.7 | GRY |
| hCoV-19/Tunisia/IPT-S340/2020 | EPI_ISL_16913952 | 31/10/2020 | Africa / Tunisia / Tunis | Human | Random | Male | 5 | unknown | unknown | Random | B.1.160 | GH |
| hCoV-19/Tunisia/S-0562/2021 | EPI_ISL_16955531 | 22/06/2021 | Africa / Tunisia / Bizerte | Human | Random | Female | 49 | unknown | unknown | Random | B.1.1.7 | GRY |
| hCoV-19/Tunisia/S-0481/2021 | EPI_ISL_16955530 | 04/05/2021 | Africa / Tunisia / Medenine | Human | Random | Male | 51 | unknown | unknown | Random | B.1.1.7 | GRY |
| hCoV-19/Tunisia/S1021/2022 | EPI_ISL_16862262 | 24/11/2022 | Africa / Tunisia / Sfax | Human | Baseline surveillance | Female | 74 | unknown | unknown | Baseline surveillance | BN.1.3.7 | GRA |
| hCoV-19/Tunisia/S1020/2022 | EPI_ISL_16862261 | 19/11/2022 | Africa / Tunisia / Sfax | Human | Baseline surveillance | Male | 63 | unknown | unknown | Baseline surveillance | XBB.1.9 | GRA |
| hCoV-19/Tunisia/S1019/2022 | EPI_ISL_16862260 | 17/11/2022 | Africa / Tunisia / Sfax | Human | Baseline surveillance | Male | 59 | unknown | unknown | Baseline surveillance | BE.1.1.1 | GRA |
| hCoV-19/Tunisia/S1018/2022 | EPI_ISL_16862259 | 16/11/2022 | Africa / Tunisia / Sfax | Human | Baseline surveillance | Female | 58 | unknown | unknown | Baseline surveillance | BQ.1.1 | GR |
| hCoV-19/Tunisia/S1017/2022 | EPI_ISL_16862258 | 15/11/2022 | Africa / Tunisia / Sfax | Human | Baseline surveillance | Male | 51 | unknown | unknown | Baseline surveillance | BQ.1 | GRA |
| hCoV-19/Tunisia/S1016/2022 | EPI_ISL_16862257 | 14/11/2022 | Africa / Tunisia / Sfax | Human | Baseline surveillance | Male | 28 | unknown | unknown | Baseline surveillance | BQ.1 | GRA |
| hCoV-19/Tunisia/S1015/2022 | EPI_ISL_16862256 | 15/11/2022 | Africa / Tunisia / Sfax | Human | Baseline surveillance | Female | 31 | unknown | unknown | Baseline surveillance | BQ.1.1 | GRA |
| hCoV-19/Tunisia/S-0480/2021 | EPI_ISL_16955529 | 04/05/2021 | Africa / Tunisia / Medenine | Human | Random | Female | 45 | unknown | unknown | Random | B.1.1.7 | GRY |
| hCoV-19/Tunisia/S1012/2022 | EPI_ISL_16862253 | 13/11/2022 | Africa / Tunisia / Sfax | Human | Baseline surveillance | Female | 25 | unknown | unknown | Baseline surveillance | Unassigned | G |
| hCoV-19/Tunisia/S1014/2022 | EPI_ISL_16862255 | 12/11/2022 | Africa / Tunisia / Sfax | Human | Baseline surveillance | Female | 58 | unknown | unknown | Baseline surveillance | BQ.1.1 | GRA |
| hCoV-19/Tunisia/S1013/2022 | EPI_ISL_16862254 | 14/11/2022 | Africa / Tunisia / Sfax | Human | Baseline surveillance | Male | 36 | unknown | unknown | Baseline surveillance | BQ.1.1 | GRA |
| hCoV-19/Tunisia/S1006/2022 | EPI_ISL_16862249 | 08/11/2022 | Africa / Tunisia / Sfax | Human | Baseline surveillance | Female | 28 | unknown | unknown | Baseline surveillance | BQ.1 | GRA |
| hCoV-19/Tunisia/S1005/2022 | EPI_ISL_16862248 | 08/11/2022 | Africa / Tunisia / Sfax | Human | Baseline surveillance | Female | 34 | unknown | unknown | Baseline surveillance | BQ.1.1 | GRA |
| hCoV-19/Tunisia/S1004/2022 | EPI_ISL_16862247 | 04/11/2022 | Africa / Tunisia / Sfax | Human | Baseline surveillance | Male | 32 | unknown | unknown | Baseline surveillance | BQ.1 | GRA |
| hCoV-19/Tunisia/S1003/2022 | EPI_ISL_16862246 | 29/10/2022 | Africa / Tunisia / Sfax | Human | Baseline surveillance | Female | 55 | unknown | unknown | Baseline surveillance | BQ.1 | GRA |
| hCoV-19/Tunisia/S1002/2022 | EPI_ISL_16862245 | 30/10/2022 | Africa / Tunisia / Sfax | Human | Baseline surveillance | Male | 35 | unknown | unknown | Baseline surveillance | BQ.1 | GRA |
| hCoV-19/Tunisia/S1001/2022 | EPI_ISL_16862244 | 29/10/2022 | Africa / Tunisia / Sfax | Human | Baseline surveillance | Male | 51 | unknown | unknown | Baseline surveillance | BQ.1 | GRA |
| hCoV-19/Tunisia/S1000/2022 | EPI_ISL_16862243 | 25/10/2022 | Africa / Tunisia / Sfax | Human | Baseline surveillance | Female | 55 | unknown | unknown | Baseline surveillance | BQ.1.1 | GRA |
| hCoV-19/Tunisia/S998/2022 | EPI_ISL_16862242 | 24/10/2022 | Africa / Tunisia / Sfax | Human | Baseline surveillance | Female | 49 | unknown | unknown | Baseline surveillance | BQ.1 | GRA |
| hCoV-19/Tunisia/S997/2022 | EPI_ISL_16862241 | 23/10/2022 | Africa / Tunisia / Sfax | Human | Baseline surveillance | Male | 66 | unknown | unknown | Baseline surveillance | BQ.1 | GRA |
| hCoV-19/Tunisia/S996/2022 | EPI_ISL_16862240 | 21/10/2022 | Africa / Tunisia / Sfax | Human | Baseline surveillance | Female | 31 | unknown | unknown | Baseline surveillance | BQ.1.1.4 | GRA |
| hCoV-19/Tunisia/S995/2022 | EPI_ISL_16862239 | 20/10/2022 | Africa / Tunisia / Sfax | Human | Baseline surveillance | Female | 42 | unknown | unknown | Baseline surveillance | BQ.1 | GRA |
| hCoV-19/Tunisia/S987/2022 | EPI_ISL_16862238 | 15/09/2022 | Africa / Tunisia / Sfax | Human | Baseline surveillance | Male | 18 | unknown | unknown | Baseline surveillance | BA.5.2 | GRA |
| hCoV-19/Tunisia/S1011/2022 | EPI_ISL_16862252 | 12/11/2022 | Africa / Tunisia / Sfax | Human | Baseline surveillance | Female | 27 | unknown | unknown | Baseline surveillance | BE.1.1.1 | G |
| hCoV-19/Tunisia/S1010/2022 | EPI_ISL_16862251 | 12/11/2022 | Africa / Tunisia / Sfax | Human | Baseline surveillance | Male | 63 | Deceaded | unknown | Baseline surveillance | BQ.1.1 | GRA |
| hCoV-19/Tunisia/S1009/2022 | EPI_ISL_16862250 | 11/11/2022 | Africa / Tunisia / Sfax | Human | Baseline surveillance | Male | 28 | unknown | unknown | Baseline surveillance | BQ.1 | GRA |
| hCoV-19/Tunisia/U-3636/2021 | EPI_ISL_16847411 | 23/02/2021 | Africa / Tunisia / Tunis | Human | Random | Female | 6 | unknown | unknown | Random | B.1.160 | GH |
| hCoV-19/Tunisia/D-2972/2021 | EPI_ISL_16854727 | 22/05/2021 | Africa / Tunisia / Tunis | Human | Random | Female | 16 | unknown | unknown | Random | AY.122 | GK |
| hCoV-19/Tunisia/A179/2021 | EPI_ISL_16854726 | 28/07/2021 | Africa / Tunisia / Tunis | Human | Random | Female | 19 | unknown | unknown | Random | AY.122 | GK |
| hCoV-19/Tunisia/S387/2021 | EPI_ISL_16847408 | 17/06/2021 | Africa / Tunisia / Tunis | Human | Random | Male | 12 | unknown | unknown | Random | B.1.1.7 | GRY |
| hCoV-19/Tunisia/F-7846/2022 | EPI_ISL_16847407 | 11/01/2022 | Africa / Tunisia / Tunis | Human | Random | Female | 13 | unknown | unknown | Random | BA.1.1.1 | GRA |
| hCoV-19/Tunisia/C-8565/2021 | EPI_ISL_16847406 | 21/06/2021 | Africa / Tunisia / Tunis | Human | Random | Female | 18 | unknown | unknown | Random | AY.122 | GK |
| hCoV-19/Tunisia/TD-2920/2021 | EPI_ISL_16847405 | 21/05/2021 | Africa / Tunisia / Tunis | Human | Random | Female | 18 | unknown | unknown | Random | B.1.1.7 | GRY |
| hCoV-19/Tunisia/D-6300/2021 | EPI_ISL_16847404 | 08/06/2021 | Africa / Tunisia / Tunis | Human | Random | Female | 13 | unknown | unknown | Random | B.1.1.7 | GR |
| hCoV-19/Tunisia/E-494/2021 | EPI_ISL_16847403 | 23/06/2021 | Africa / Tunisia / Tunis | Human | Random | Female | 11 months | unknown | unknown | Random | AY.122 | GK |
| hCoV-19/Tunisia/A104/2021 | EPI_ISL_16847402 | 16/06/2021 | Africa / Tunisia / Tunis | Human | Random | Male | 18 | unknown | unknown | Random | AY.122 | GK |
| hCoV-19/Tunisia/A127/2021 | EPI_ISL_16847401 | 23/06/2021 | Africa / Tunisia / Tunis | Human | Random | Female | 18 | unknown | unknown | Random | AY.122 | GK |
| hCoV-19/Tunisia/E-9220/2021 | EPI_ISL_16847400 | 23/07/2021 | Africa / Tunisia / Tunis | Human | Random | Female | 18 | unknown | unknown | Random | AY.122 | GK |
| hCoV-19/Tunisia/E-843/2021 | EPI_ISL_10141452 | 24/06/2021 | Africa / Tunisia / Tunis | Human | unknown | Male | 26 | unknown | No | unknown | AY.122 | GK |
| hCoV-19/Tunisia/19695/2020 | EPI_ISL_733500 | 12/07/2020 | Africa / Tunisia / Ben Arous | Human | unknown | Female | 49 | unknown | unknown | unknown | B.1.177 | GV |
| hCoV-19/Tunisia/63686/2020 | EPI_ISL_733499 | 01/12/2020 | Africa / Tunisia / Nabeul | Human | unknown | Male | 37 | unknown | unknown | unknown | B.1.177 | GV |
| hCoV-19/Tunisia/S-1598/2021 | EPI_ISL_16398507 | 25/11/2021 | Africa / Tunisia / Sousse | Human | Random | Female | 50 | unknown | unknown | Random | AY.43 | GK |
| hCoV-19/Tunisia/S-1613/2021 | EPI_ISL_16398506 | 05/12/2021 | Africa / Tunisia / Sousse | Human | Random | Female | 45 | unknown | unknown | Random | AY.122 | GK |
| hCoV-19/Tunisia/S-1690/2021 | EPI_ISL_16398505 | 10/12/2021 | Africa / Tunisia / Bizerte | Human | Random | Female | 25 | unknown | unknown | Random | AY.122 | GK |
| hCoV-19/Tunisia/S-1688/2021 | EPI_ISL_16398504 | 10/12/2021 | Africa / Tunisia / Bizerte | Human | Random | Female | 26 | unknown | unknown | Random | AY.122 | GK |
| hCoV-19/Tunisia/S-1676/2021 | EPI_ISL_16398503 | 09/12/2021 | Africa / Tunisia / Manouba | Human | Random | Female | 47 | unknown | unknown | Random | AY.122 | GK |
| hCoV-19/Tunisia/S-1632/2021 | EPI_ISL_16398502 | 02/12/2021 | Africa / Tunisia / Tataouine | Human | Random | Female | 17 | unknown | unknown | Random | AY.122 | GK |
| hCoV-19/Tunisia/S-1631/2021 | EPI_ISL_16398501 | 01/12/2021 | Africa / Tunisia / Tataouine | Human | Random | Female | 2 | unknown | unknown | Random | AY.122 | GK |
| hCoV-19/Tunisia/S-1629/2021 | EPI_ISL_16398500 | 02/12/2021 | Africa / Tunisia / Tataouine | Human | Random | Female | 43 | unknown | unknown | Random | AY.122 | GK |
| hCoV-19/Tunisia/S-1625/2021 | EPI_ISL_16398499 | 02/12/2021 | Africa / Tunisia / Tataouine | Human | Random | Male | 24 | unknown | unknown | Random | AY.122 | GK |
| hCoV-19/Tunisia/S-1624/2021 | EPI_ISL_16398498 | 07/12/2021 | Africa / Tunisia / Tataouine | Human | Random | Female | 56 | unknown | unknown | Random | AY.122 | GK |
| hCoV-19/Tunisia/S-1621/2021 | EPI_ISL_16398497 | 02/12/2021 | Africa / Tunisia / Tataouine | Human | Random | Male | 59 | unknown | unknown | Random | AY.122 | GK |
| hCoV-19/Tunisia/S-1783/2021 | EPI_ISL_16398496 | 20/12/2021 | Africa / Tunisia / Ariana | Human | Random | Female | 38 | unknown | unknown | Random | AY.122 | GK |
| hCoV-19/Tunisia/S-1764/2021 | EPI_ISL_16398495 | 27/12/2021 | Africa / Tunisia / Sousse | Human | Random | Female | 48 | unknown | unknown | Random | AY.122 | GK |
| hCoV-19/Tunisia/S-1732/2021 | EPI_ISL_16398494 | 16/12/2021 | Africa / Tunisia / Bizerte | Human | Random | Female | 39 | unknown | unknown | Random | AY.122 | GK |
| hCoV-19/Tunisia/S-1915/2021 | EPI_ISL_16398493 | 09/12/2021 | Africa / Tunisia / Sousse | Human | Random | Male | 39 | Mild infection | unknown | Random | AY.122 | GK |
| hCoV-19/Tunisia/S-1911/2021 | EPI_ISL_16398492 | 07/12/2021 | Africa / Tunisia / Sousse | Human | Random | Male | 55 | Mild infection | unknown | Random | AY.122 | GK |
| hCoV-19/Tunisia/S-1923/2021 | EPI_ISL_16398491 | 12/12/2021 | Africa / Tunisia / Sousse | Human | Random | Female | 55 | Mild infection | unknown | Random | AY.122 | GK |
| hCoV-19/Tunisia/S-1932/2021 | EPI_ISL_16398490 | 14/12/2021 | Africa / Tunisia / Sousse | Human | Random | Male | 50 | unknown | unknown | Random | AY.122 | GK |
| hCoV-19/Tunisia/S-1963/2021 | EPI_ISL_16398489 | 23/12/2021 | Africa / Tunisia / Sousse | Human | Random | Female | 88 | Mild infection | unknown | Random | AY.122 | GK |
| hCoV-19/Tunisia/S-2000/2022 | EPI_ISL_16398488 | 04/01/2022 | Africa / Tunisia / Sousse | Human | Random | Female | 29 | Mild infection | unknown | Random | AY.122 | GK |
| hCoV-19/Tunisia/S-2074/2022 | EPI_ISL_16398487 | 03/01/2022 | Africa / Tunisia / Ariana | Human | Random | Female | 35 | unknown | unknown | Random | AY.122 | GK |
| hCoV-19/Tunisia/S-2084/2021 | EPI_ISL_16398486 | 18/12/2021 | Africa / Tunisia / Bizerte | Human | Random | Female | 63 | unknown | unknown | Random | AY.122 | GK |
| hCoV-19/Tunisia/S-2086/2021 | EPI_ISL_16398485 | 20/12/2021 | Africa / Tunisia / Bizerte | Human | Random | Male | 76 | unknown | unknown | Random | AY.43 | GK |
| hCoV-19/Tunisia/S-2089/2021 | EPI_ISL_16398484 | 20/12/2021 | Africa / Tunisia / Bizerte | Human | Random | Female | 4 | Mild infection | unknown | Random | AY.122 | GK |
| hCoV-19/Tunisia/S-2091/2021 | EPI_ISL_16398483 | 20/12/2021 | Africa / Tunisia / Bizerte | Human | Random | Male | 29 | Severe infection | unknown | Random | AY.122 | GK |
| hCoV-19/Tunisia/S-2101/2021 | EPI_ISL_16398482 | 22/12/2021 | Africa / Tunisia / Bizerte | Human | Random | Female | 37 | Mild infection | unknown | Random | AY.122 | GK |
| hCoV-19/Tunisia/S-2102/2021 | EPI_ISL_16398481 | 23/12/2021 | Africa / Tunisia / Bizerte | Human | Random | Female | 66 | Mild infection | unknown | Random | AY.122 | GK |
| hCoV-19/Tunisia/S-2111/2021 | EPI_ISL_16398480 | 29/12/2021 | Africa / Tunisia / Bizerte | Human | Random | Male | 38 | Mild infection | unknown | Random | AY.122 | GK |
| hCoV-19/Tunisia/S-2119/2021 | EPI_ISL_16398479 | 30/12/2021 | Africa / Tunisia / Bizerte | Human | Random | Female | 67 | Mild infection | unknown | Random | AY.122 | GK |
| hCoV-19/Tunisia/S-2124/2021 | EPI_ISL_16398478 | 31/12/2021 | Africa / Tunisia / Bizerte | Human | Random | Female | 82 | unknown | unknown | Random | AY.122 | GK |
| hCoV-19/Tunisia/S-2207/2022 | EPI_ISL_16398477 | 08/01/2022 | Africa / Tunisia / Kairouan | Human | Random | Male | 83 | Mild infection | unknown | Random | AY.122 | GK |
| hCoV-19/Tunisia/S-2212/2022 | EPI_ISL_16398476 | 08/01/2022 | Africa / Tunisia / Kairouan | Human | Random | Male | 28 | unknown | unknown | Random | AY.122 | GK |
| hCoV-19/Tunisia/S-2215/2022 | EPI_ISL_16398475 | 10/01/2022 | Africa / Tunisia / Kairouan | Human | Random | Male | 61 | unknown | unknown | Random | AY.99.1 | GK |
| hCoV-19/Tunisia/S-2535/2022 | EPI_ISL_16398474 | 07/01/2022 | Africa / Tunisia / Sousse | Human | Random | Male | unknown | unknown | unknown | Random | AY.122 | GK |
| hCoV-19/Tunisia/S-1593/2021 | EPI_ISL_16398473 | 20/11/2021 | Africa / Tunisia / Sousse | Human | Random | Male | 82 | unknown | unknown | Random | AY.122 | GK |
| hCoV-19/Tunisia/S-2525/2022 | EPI_ISL_16377410 | 2022 | Africa / Tunisia / Sousse | Human | Random | Female | 53 | unknown | unknown | Random | AY.122 | GK |
| hCoV-19/Tunisia/S-1605/2021 | EPI_ISL_16377409 | 2021 | Africa / Tunisia / Sousse | Human | Random | Male | 13 | unknown | unknown | Random | AY.4 | GK |
| hCoV-19/Tunisia/S-0711/2021 | EPI_ISL_10141423 | 29/06/2021 | Africa / Tunisia / Kairouen | Human | unknown | Male | 1 month | unknown | No | unknown | B.1 | G |
| hCoV-19/Tunisia/ADAGE-17149/2020 | EPI_ISL_712568 | 29/09/2020 | Africa / Tunisia / Sfax | Human | unknown | unknown | unknown | unknown | unknown | unknown | B.1.36 | GH |
| hCoV-19/Tunisia/ADAGE-24463/2020 | EPI_ISL_712069 | 06/11/2020 | Africa / Tunisia / Sfax | Human | unknown | unknown | unknown | Deceased | unknown | unknown | B.1.177 | G |
| hCoV-19/Tunisia/ADAGE-15425/2020 | EPI_ISL_712068 | 21/09/2020 | Africa / Tunisia / Sfax | Human | unknown | unknown | unknown | Deceased | unknown | unknown | B.1 | G |
| hCoV-19/Tunisia/ADAGE-24804/2020 | EPI_ISL_712067 | 08/11/2020 | Africa / Tunisia / Sfax | Human | unknown | unknown | unknown | unknown | unknown | unknown | B.1.177 | G |
| hCoV-19/Tunisia/ADAGE-24779/2020 | EPI_ISL_712066 | 08/11/2020 | Africa / Tunisia / Sfax | Human | unknown | unknown | unknown | unknown | unknown | unknown | B.1.160 | GH |
| hCoV-19/Tunisia/ADAGE-24763/2020 | EPI_ISL_712064 | 08/11/2020 | Africa / Tunisia / Sfax | Human | unknown | unknown | unknown | unknown | unknown | unknown | B.1.160 | GH |
| hCoV-19/Tunisia/ADAGE-24758/2020 | EPI_ISL_712063 | 08/11/2020 | Africa / Tunisia / Sfax | Human | unknown | unknown | unknown | unknown | unknown | unknown | B.1.597 | GH |
| hCoV-19/Tunisia/ADAGE-18503/2020 | EPI_ISL_711057 | 19/10/2020 | Africa / Tunisia / Monastir | Human | unknown | Female | 50 | Released | unknown | unknown | B.1.177 | GV |
| hCoV-19/Tunisia/ADAGE-17786/2020 | EPI_ISL_710575 | 13/10/2020 | Africa / Tunisia / Monastir | Human | unknown | Female | 48 | Released | unknown | unknown | B.1.177 | GV |
| hCoV-19/Tunisia/ADAGE-18245/2020 | EPI_ISL_710541 | 16/10/2020 | Africa / Tunisia / Monastir | Human | unknown | Male | 37 | Released | unknown | unknown | B.1.1.198 | GR |
| hCoV-19/Tunisia/ADAGE-18212/2020 | EPI_ISL_710540 | 16/10/2020 | Africa / Tunisia / Monastir | Human | unknown | Male | 60 | Post mortem | unknown | unknown | B.1.1.1 | GR |
| hCoV-19/Tunisia/ADAGE-17872/2020 | EPI_ISL_710537 | 14/10/2020 | Africa / Tunisia / Monastir | Human | unknown | Female | 31 | Released | unknown | unknown | B.1.428.2 | GH |
| hCoV-19/Tunisia/ADAGE-17873/2020 | EPI_ISL_710534 | 14/10/2020 | Africa / Tunisia / Monastir | Human | unknown | Female | 33 | Released | unknown | unknown | B.1.1.1 | GR |
| hCoV-19/Tunisia/9111/2020 | EPI_ISL_707793 | 11/04/2020 | Africa / Tunisia / Tunis | Human | unknown | Male | 60 | unknown | unknown | unknown | B.1.1.189 | GR |
| hCoV-19/Tunisia/6736/2020 | EPI_ISL_707792 | 31/03/2020 | Africa / Tunisia / Tunis | Human | unknown | Female | 79 | unknown | unknown | unknown | B.4 | O |
| hCoV-19/Tunisia/2909/2020 | EPI_ISL_707791 | 02/03/2020 | Africa / Tunisia / Gafsa | Human | unknown | Male | 60 | Live | unknown | unknown | B.1 | G |
| hCoV-19/Tunisia/61600/2020 | EPI_ISL_707700 | 23/11/2020 | Africa / Tunisia / Nabeul | Human | unknown | Female | 35 | Live | unknown | unknown | B.1.160 | GH |
| hCoV-19/Tunisia/61624/2020 | EPI_ISL_707699 | 23/11/2020 | Africa / Tunisia / Nabeul | Human | unknown | Male | 40 | Live | unknown | unknown | B.1.1.189 | GR |
| hCoV-19/Tunisia/61627/2020 | EPI_ISL_707698 | 23/11/2020 | Africa / Tunisia / Soliman | Human | unknown | Male | 30 | Live | unknown | unknown | B.1.9 | GH |
| hCoV-19/Tunisia/61628/2020 | EPI_ISL_707697 | 23/11/2020 | Africa / Tunisia / Nabeul | Human | unknown | Male | 45 | Live | unknown | unknown | B.1.356 | GH |
| hCoV-19/Tunisia/3942/2020 | EPI_ISL_699657 | 16/03/2020 | Africa / Tunisia / Monastir | Human | unknown | Female | 33 | Live | unknown | unknown | B.1.177 | GV |
| hCoV-19/Tunisia/5008/2020 | EPI_ISL_699656 | 24/03/2020 | Africa / Tunisia / Tunis | Human | unknown | Male | 1 | Live | unknown | unknown | B.1.597 | GH |
| hCoV-19/Tunisia/61689/2020 | EPI_ISL_699655 | 23/11/2020 | Africa / Tunisia / Nabeul | Human | unknown | Male | 30 | Live | unknown | unknown | B.1 | GH |
| hCoV-19/Tunisia/S-2641/2022 | EPI_ISL_16186004 | 23/03/2022 | Africa / Tunisia / Tunis | Human | Random | Female | 84 | unknown | unknown | Random | BA.2 | GRA |
| hCoV-19/Tunisia/S-2527/2022 | EPI_ISL_16185967 | 19/01/2022 | Africa / Tunisia / Sousse | Human | Random | Male | 39 | unknown | unknown | Random | BA.1.17.2 | GRA |
| hCoV-19/Tunisia/G-3330/2022 | EPI_ISL_16185893 | 16/03/2022 | Africa / Tunisia / Tunis | Human | Random | Male | 36 | unknown | unknown | Random | BA.2.9 | GRA |
| hCoV-19/Tunisia/F-7255/2022 | EPI_ISL_16185803 | 06/01/2022 | Africa / Tunisia / Tunis | Human | Random | Male | 34 | unknown | unknown | Random | BA.1.21.1 | GRA |
| hCoV-19/Tunisia/4107/2020 | EPI_ISL_683329 | 18/03/2020 | Africa / Tunisia / Ben Arous | Human | unknown | Male | 22 | unknown | unknown | unknown | B.1.177 | GV |
| hCoV-19/Tunisia/53873/2020 | EPI_ISL_654020 | 26/10/2020 | Africa / Tunisia / Ben Arous | Human | unknown | Male | 34 | Live | unknown | unknown | B.1.160 | GH |
| hCoV-19/Tunisia/55308/2020 | EPI_ISL_654019 | 02/11/2020 | Africa / Tunisia / Tunis | Human | unknown | Male | 58 | Live | unknown | unknown | B.1.160 | GH |
| hCoV-19/Tunisia/58412/2020 | EPI_ISL_654018 | 02/11/2020 | Africa / Tunisia / Ben Arous | Human | unknown | Female | 23 | Live | unknown | unknown | B.1.160 | GH |
| hCoV-19/Tunisia/55262/2020 | EPI_ISL_654017 | 02/11/2020 | Africa / Tunisia / Nabeul | Human | unknown | Female | 42 | Live | unknown | unknown | B.1.177 | GV |
| hCoV-19/Tunisia/3913/2020 | EPI_ISL_654016 | 16/03/2020 | Africa / Tunisia / Tunis / Marsa | Human | unknown | Female | 45 | Live | unknown | unknown | B.1.1 | GR |
| hCoV-19/Tunisia/55439/2020 | EPI_ISL_635062 | 02/11/2020 | Africa / Tunisia / Tunis | Human | unknown | Female | 24 | Live | unknown | unknown | B.1.160 | GH |
| hCoV-19/Tunisia/55400/2020 | EPI_ISL_635061 | 02/11/2020 | Africa / Tunisia / Nabeul | Human | unknown | Female | 40 | Hospitalized | unknown | unknown | B.1.177 | GV |
| hCoV-19/Tunisia/55319/2020 | EPI_ISL_635060 | 02/11/2020 | Africa / Tunisia / Tunis | Human | unknown | Male | 41 | Live | unknown | unknown | B.1.160 | GH |
| hCoV-19/Tunisia/55304/2020 | EPI_ISL_635059 | 02/11/2020 | Africa / Tunisia / Tunis | Human | unknown | Male | 29 | Live | unknown | unknown | B.1.1 | GR |
| hCoV-19/Tunisia/55153/2020 | EPI_ISL_634977 | 31/10/2020 | Africa / Tunisia / Ben Arous | Human | unknown | Female | 47 | Live | unknown | unknown | B.1.428.2 | GH |
| hCoV-19/Tunisia/55006/2020 | EPI_ISL_632310 | 31/10/2020 | Africa / Tunisia / Ben Arous | Human | unknown | Female | 39 | Live | unknown | unknown | B.1.428.2 | GH |
| hCoV-19/Tunisia/H-3331/2021 | EPI_ISL_10101199 | 14/12/2021 | Africa / Tunisia | Human | unknown | Male | 36 | unknown | unknown | unknown | B.1.160 | GH |
| hCoV-19/Tunisia/COV1482/2020 | EPI_ISL_463006 | 31/03/2020 | Africa / Tunisia | Human | unknown | unknown | unknown | unknown | unknown | unknown | B.1 | GH |
| hCoV-19/Tunisia/COV0425/2020 | EPI_ISL_463005 | 27/03/2020 | Africa / Tunisia | Human | unknown | unknown | unknown | unknown | unknown | unknown | B.1 | GH |
| hCoV-19/Tunisia/COV1663/2020 | EPI_ISL_463004 | 01/04/2020 | Africa / Tunisia | Human | unknown | unknown | unknown | unknown | unknown | unknown | B.1 | G |
| hCoV-19/Tunisia/MHT_2/2020 | EPI_ISL_458286 | 24/03/2020 | Africa / Tunisia / Ben Arous | Human | unknown | Female | 59 | unknown | unknown | unknown | A | S |
| hCoV-19/Tunisia/MHT_1/2020 | EPI_ISL_458285 | 21/03/2020 | Africa / Tunisia / Bizerte | Human | unknown | Male | 32 | unknown | unknown | unknown | B.1 | GH |
| hCoV-19/Tunisia/Tunis_7643-2/2020 | EPI_ISL_450494 | 03/04/2020 | Africa / Tunisia | Human | unknown | Female | unknown | unknown | unknown | unknown | Unassigned | O |
| hCoV-19/Tunisia/Tunis_6401-2/2020 | EPI_ISL_450492 | 29/03/2020 | Africa / Tunisia | Human | unknown | Male | unknown | unknown | unknown | unknown | Unassigned | O |
| hCoV-19/Tunisia/Tunis_7643/2020 | EPI_ISL_450491 | 03/04/2020 | Africa / Tunisia | Human | unknown | Female | unknown | unknown | unknown | unknown | Unassigned | O |
| hCoV-19/Tunisia/GB1373/2020 | EPI_ISL_11172653 | 03/04/2020 | Africa / Tunisia / Medenine | Human | Random | Male | 19 | Mild infection | Not Vaccinated | Random | B.1 | GH |
| hCoV-19/Tunisia/F-1689/2021 | EPI_ISL_8298401 | 21/08/2021 | Africa / Tunisia / Ariana | Human | Random | Male | 49 | unknown | unknown | Random | AY.122 | GK |
| hCoV-19/Tunisia/F-2716/2021 | EPI_ISL_8298501 | 03/09/2021 | Africa / Tunisia / Tunis | Human | Random | Male | 50 | unknown | unknown | Random | AY.122 | GK |
| hCoV-19/Tunisia/B-5241/2021 | EPI_ISL_10141483 | 29/04/2021 | Africa / Tunisia / Tunis | Human | unknown | Female | 50 | Mild | No | unknown | B.1.1.7 | GRY |
| hCoV-19/Tunisia/ADAGE-12622/2020 | EPI_ISL_710532 | 20/09/2020 | Africa / Tunisia / Monastir | Human | unknown | Female | 37 | unknown | unknown | unknown | B.1.1.198 | GR |
| hCoV-19/Tunisia/Q-5328/2021 | EPI_ISL_10101262 | 01/02/2021 | Africa / Tunisia | Human | unknown | Female | 37 | unknown | unknown | unknown | B.1 | GH |
| hCoV-19/Tunisia/F-1828/2021 | EPI_ISL_8298475 | 23/08/2021 | Africa / Tunisia / Tunis | Human | Random | Female | 61 | unknown | unknown | Random | AY.122 | GK |
| hCoV-19/Tunisia/A-9197/2020 | EPI_ISL_10141385 | 24/08/2020 | Africa / Tunisia / Jendouba | Human | unknown | Female | 25 | unknown | No | unknown | B.1.1 | GR |
| hCoV-19/Tunisia/S-1041/2021 | EPI_ISL_8298443 | 20/09/2021 | Africa / Tunisia / Sousse | Human | Random | Female | 69 | unknown | unknown | Random | AY.122 | GK |
| hCoV-19/Tunisia/Q-6798/2021 | EPI_ISL_10101285 | 08/02/2021 | Africa / Tunisia | Human | unknown | Female | 57 | unknown | unknown | unknown | B.1.160 | GH |
| hCoV-19/Tunisia/F-1949/2021 | EPI_ISL_8298485 | 25/08/2021 | Africa / Tunisia / Tunis | Human | Random | Male | 35 | unknown | unknown | Random | AY.122 | GK |
| hCoV-19/Tunisia/V-1490/2021 | EPI_ISL_8298554 | 30/09/2021 | Africa / Tunisia / Tunis | Human | Random | Male | 34 | unknown | unknown | Random | AY.122 | GK |
| hCoV-19/Tunisia/F-1260/2021 | EPI_ISL_8298467 | 16/08/2021 | Africa / Tunisia / Tunis | Human | Random | Male | 83 | unknown | unknown | Random | AY.122 | GK |
| hCoV-19/Tunisia/F-1158/2021 | EPI_ISL_8298462 | 13/08/2021 | Africa / Tunisia / Tunis | Human | Random | Male | 36 | unknown | unknown | Random | AY.122 | GK |
| hCoV-19/Tunisia/F-3334/2021 | EPI_ISL_8298530 | 14/09/2021 | Africa / Tunisia / Tunis | Human | Random | Male | 35 | unknown | unknown | Random | AY.122 | GK |
| hCoV-19/Tunisia/F-1480/2021 | EPI_ISL_8298471 | 18/08/2021 | Africa / Tunisia / Tunis | Human | Random | Male | 58 | unknown | unknown | Random | AY.122 | GK |
| hCoV-19/Tunisia/F-3102/2021 | EPI_ISL_8298520 | 09/09/2021 | Africa / Tunisia / Tunis | Human | Random | Male | 58 | unknown | unknown | Random | AY.122 | GK |
| hCoV-19/Tunisia/F-0816/2021 | EPI_ISL_8298454 | 07/08/2021 | Africa / Tunisia / Tunis | Human | Random | Female | 52 | unknown | unknown | Random | AY.122 | GK |
| hCoV-19/Tunisia/F-3837/2021 | EPI_ISL_8298549 | 23/09/2021 | Africa / Tunisia / Tunis | Human | Random | Female | 44 | unknown | unknown | Random | AY.122 | GK |
| hCoV-19/Tunisia/H-5541/2021 | EPI_ISL_10101248 | 02/01/2021 | Africa / Tunisia | Human | unknown | Male | 62 | unknown | unknown | unknown | B.1 | G |
| hCoV-19/Tunisia/3316/2023 | EPI_ISL_17764925 | 10/03/2023 | Africa / Tunisia / Ariana | Human | Baseline surveillance | Female | 22 | unknown | unknown | Baseline surveillance | XBB.1.9.2 | GR |
| hCoV-19/Tunisia/3579/2023 | EPI_ISL_17764816 | 16/03/2023 | Africa / Tunisia / Tunis | Human | Baseline surveillance | Female | 63 | unknown | unknown | Baseline surveillance | XBB.1.9.2 | GRA |
| hCoV-19/Tunisia/4648/2023 | EPI_ISL_17764811 | 14/04/2023 | Africa / Tunisia / Beja | Human | Baseline surveillance | Female | 2 | unknown | unknown | Baseline surveillance | XBB.1.9.2 | GR |
| hCoV-19/Tunisia/4665/2023 | EPI_ISL_17764796 | 14/04/2023 | Africa / Tunisia / Nabeul | Human | Baseline surveillance | Male | 68 | unknown | unknown | Baseline surveillance | XBB.1.9.2 | GRA |
| hCoV-19/Tunisia/4154/2023 | EPI_ISL_17764795 | 31/03/2023 | Africa / Tunisia / Gabes | Human | Baseline surveillance | Female | 42 | unknown | unknown | Baseline surveillance | XBB.1.9.1 | GRA |
| hCoV-19/Tunisia/4639/2023 | EPI_ISL_17764266 | 14/04/2023 | Africa / Tunisia / Mahdia | Human | Baseline surveillance | Female | 68 | unknown | unknown | Baseline surveillance | XBB.1.9.2 | GR |
| hCoV-19/Tunisia/4420/2023 | EPI_ISL_17764250 | 07/04/2023 | Africa / Tunisia / Gabes | Human | Baseline surveillance | Female | unknown | unknown | unknown | Baseline surveillance | XBB.1.9.1 | GR |
| hCoV-19/Tunisia/3326/2023 | EPI_ISL_17764154 | 10/03/2023 | Africa / Tunisia / Nabeul | Human | Baseline surveillance | Female | 64 | unknown | unknown | Baseline surveillance | XBB.1.9.2 | GRA |
| hCoV-19/Tunisia/3280/2023 | EPI_ISL_17764153 | 09/03/2023 | Africa / Tunisia / Nabeul | Human | Baseline surveillance | Female | 92 | unknown | unknown | Baseline surveillance | XBB.1.9.2 | GRA |
| hCoV-19/Tunisia/3278/2023 | EPI_ISL_17764152 | 09/03/2023 | Africa / Tunisia / Tunis | Human | Baseline surveillance | Female | 53 | unknown | unknown | Baseline surveillance | XBB.1.9.2 | GRA |
| hCoV-19/Tunisia/3026/2023 | EPI_ISL_17764147 | 04/03/2023 | Africa / Tunisia / Nabeul | Human | Baseline surveillance | Male | 32 | unknown | unknown | Baseline surveillance | XBB.1.9.2 | GRA |
| hCoV-19/Tunisia/2298/2023 | EPI_ISL_17762863 | 16/02/2023 | Africa / Tunisia / Tunis | Human | Baseline surveillance | Female | 31 | unknown | unknown | Baseline surveillance | XBB.1.9.2 | GR |
| hCoV-19/Tunisia/Y-4470/2021 | EPI_ISL_10101277 | 06/10/2021 | Africa / Tunisia | Human | unknown | Male | 58 | unknown | unknown | unknown | B.1.597 | GH |
| hCoV-19/Tunisia/F-2176/2021 | EPI_ISL_8298425 | 26/08/2021 | Africa / Tunisia / Ben Arous | Human | Random | Female | 23 | unknown | unknown | Random | AY.122 | GK |
| hCoV-19/Tunisia/34737/2021 | EPI_ISL_2907578 | 21/05/2021 | Africa / Tunisia / Ariana | Human | unknown | Male | unknown | unknown | unknown | unknown | AY.122 | GK |
| hCoV-19/Tunisia/F-0381/2021 | EPI_ISL_8298386 | 03/08/2021 | Africa / Tunisia / Ariana | Human | Random | Male | 38 | unknown | unknown | Random | AY.122 | GK |
| hCoV-19/Tunisia/M-0654/2021 | EPI_ISL_10101249 | 07/10/2021 | Africa / Tunisia | Human | unknown | Female | 52 | unknown | unknown | unknown | B.1 | G |
| hCoV-19/Tunisia/F-1190/2021 | EPI_ISL_8298464 | 14/08/2021 | Africa / Tunisia / Tunis | Human | Random | Male | 35 | unknown | unknown | Random | AY.122 | GK |
| hCoV-19/Tunisia/F-1976/2021 | EPI_ISL_8298486 | 25/08/2021 | Africa / Tunisia / Tunis | Human | Random | Male | 45 | unknown | unknown | Random | AY.122 | GK |
| hCoV-19/Tunisia/Z-3757/2021 | EPI_ISL_10101238 | 09/11/2021 | Africa / Tunisia | Human | unknown | Female | 16 | unknown | unknown | unknown | B.1 | G |
| hCoV-19/Tunisia/S-0830/2021 | EPI_ISL_10141454 | 02/07/2021 | Africa / Tunisia / Tunis | Human | unknown | Female | 57 | unknown | No | unknown | B.1.1.7 | GRY |
| hCoV-19/Tunisia/C-3899/2021 | EPI_ISL_10141486 | 16/04/2021 | Africa / Tunisia / Tunis | Human | unknown | Male | 55 | Hospitalized | No | unknown | B.1.1.7 | GRY |
| hCoV-19/Tunisia/M-1520/2021 | EPI_ISL_10101282 | 13/10/2021 | Africa / Tunisia | Human | unknown | Male | 14 | unknown | unknown | unknown | B.1.1.189 | GR |
| hCoV-19/Tunisia/M-8505/2021 | EPI_ISL_10101299 | 19/12/2021 | Africa / Tunisia | Human | unknown | Male | 40 | unknown | unknown | unknown | B.1 | G |
| hCoV-19/Tunisia/M-1015/2021 | EPI_ISL_10101318 | 08/10/2021 | Africa / Tunisia | Human | unknown | Male | 36 | unknown | unknown | unknown | B.1.160 | GH |
| hCoV-19/Tunisia/F-0407/2021 | EPI_ISL_8298387 | 03/08/2021 | Africa / Tunisia / Ariana | Human | Random | Female | 33 | unknown | unknown | Random | AY.122 | GK |
| hCoV-19/Tunisia/C-5108/2021 | EPI_ISL_10141467 | 22/04/2021 | Africa / Tunisia / Ben Arous | Human | unknown | Male | 25 | Mild | No | unknown | B.1.1.7 | GRY |
| hCoV-19/Tunisia/A-8295/2020 | EPI_ISL_10141456 | 20/08/2020 | Africa / Tunisia / Ben Arous | Human | unknown | Female | 28 | unknown | No | unknown | B.1.428.2 | GH |
| hCoV-19/Tunisia/X-3208/2020 | EPI_ISL_10141461 | 30/08/2020 | Africa / Tunisia / Ariana | Human | unknown | Male | 32 | Mild | No | unknown | B.1.428.2 | GH |
| hCoV-19/Tunisia/S-0714/2021 | EPI_ISL_10141545 | 29/06/2021 | Africa / Tunisia / Kairouen | Human | unknown | Male | 50 | unknown | No | unknown | AY.122 | GK |
| hCoV-19/Tunisia/6211/2023 | EPI_ISL_18108061 | 02/06/2023 | Africa / Tunisia / Sfax | Human | Baseline surveillance | Male | 50 | unknown | unknown | Baseline surveillance | XBB.1.5.14 | GRA |
| hCoV-19/Tunisia/5751/2023 | EPI_ISL_18108058 | 19/05/2023 | Africa / Tunisia / Mahdia | Human | Baseline surveillance | Female | 68 | unknown | unknown | Baseline surveillance | XBB.1.5.24 | G |
| hCoV-19/Tunisia/7946/2023 | EPI_ISL_18110951 | 03/08/2023 | Africa / Tunisia / Tunis | Human | Baseline surveillance | Male | 37 | unknown | unknown | Baseline surveillance | XBB.1.5.28 | GRA |
| hCoV-19/Tunisia/8028/2023 | EPI_ISL_18110955 | 07/08/2023 | Africa / Tunisia / Ariana | Human | Baseline surveillance | Male | 70 | unknown | unknown | Baseline surveillance | EG.4 | GRA |
| hCoV-19/Tunisia/8026/2023 | EPI_ISL_18110953 | 07/08/2023 | Africa / Tunisia / Beja | Human | Baseline surveillance | Male | 51 | unknown | unknown | Baseline surveillance | EG.4 | GRA |
| hCoV-19/Tunisia/7921/2023 | EPI_ISL_18110950 | 03/08/2023 | Africa / Tunisia / Ariana | Human | Baseline surveillance | Male | 2 months | unknown | unknown | Baseline surveillance | EG.4 | GRA |
| hCoV-19/Tunisia/8027/2023 | EPI_ISL_18110954 | 07/08/2023 | Africa / Tunisia / Ariana | Human | Baseline surveillance | Female | unknown | unknown | unknown | Baseline surveillance | EG.4 | GRA |
| hCoV-19/Tunisia/7054/2023 | EPI_ISL_18110949 | 01/07/2023 | Africa / Tunisia / Tunis | Human | Baseline surveillance | Male | unknown | unknown | unknown | Baseline surveillance | EG.4 | GRA |
| hCoV-19/Tunisia/7949/2023 | EPI_ISL_18110952 | 03/08/2023 | Africa / Tunisia / Tunis | Human | Baseline surveillance | Male | 7 months | unknown | unknown | Baseline surveillance | XBB.2.3.11 | GRA |
| hCoV-19/Tunisia/E-1609/2021 | EPI_ISL_10141553 | 27/06/2021 | Africa / Tunisia / Tunis | Human | unknown | Female | 41 | unknown | No | unknown | AY.122 | GK |
| hCoV-19/Tunisia/A-4079/2021 | EPI_ISL_10101188 | 28/07/2021 | Africa / Tunisia | Human | unknown | Female | 43 | unknown | unknown | unknown | B.1 | G |
| hCoV-19/Tunisia/F-1694/2021 | EPI_ISL_8298403 | 21/08/2021 | Africa / Tunisia / Ariana | Human | Random | Male | 23 | unknown | unknown | Random | AY.122 | GK |
| hCoV-19/Tunisia/F-0518/2021 | EPI_ISL_8298389 | 04/08/2021 | Africa / Tunisia / Ariana | Human | Random | Female | 41 | unknown | unknown | Random | AY.122 | GK |
| hCoV-19/Tunisia/Q-7373/2021 | EPI_ISL_10101208 | 11/02/2021 | Africa / Tunisia | Human | unknown | Female | 16 | unknown | unknown | unknown | P.2 | GR |
| hCoV-19/Tunisia/M-3828/2021 | EPI_ISL_10101281 | 02/11/2021 | Africa / Tunisia | Human | unknown | Male | 58 | unknown | unknown | unknown | B.1.597 | GH |
| hCoV-19/Tunisia/Y-6394/2021 | EPI_ISL_10101354 | 19/10/2021 | Africa / Tunisia | Human | unknown | Male | 56 | unknown | unknown | unknown | B.1.177 | GV |
| hCoV-19/Tunisia/A-1244/2021 | EPI_ISL_10101309 | 14/06/2021 | Africa / Tunisia | Human | unknown | Female | 31 | unknown | unknown | unknown | B.1 | G |
| hCoV-19/Tunisia/Q-6797/2021 | EPI_ISL_10101264 | 08/02/2021 | Africa / Tunisia | Human | unknown | Male | 48 | unknown | unknown | unknown | B.1 | G |
| hCoV-19/Tunisia/F-2007/2021 | EPI_ISL_8298490 | 25/08/2021 | Africa / Tunisia / Tunis | Human | Random | Female | 59 | unknown | unknown | Random | AY.122 | GK |
| hCoV-19/Tunisia/B-2284/2021 | EPI_ISL_10101190 | 13/03/2021 | Africa / Tunisia | Human | unknown | Female | 39 | unknown | unknown | unknown | B.1.160 | GH |
| hCoV-19/Tunisia/S-0746/2021 | EPI_ISL_10141390 | 29/06/2021 | Africa / Tunisia / Medenine | Human | unknown | Female | 24 | unknown | No | unknown | AY.122 | GK |
| hCoV-19/Tunisia/M-5563/2021 | EPI_ISL_10101320 | 10/11/2021 | Africa / Tunisia | Human | unknown | Male | 85 | unknown | unknown | unknown | B.1.160 | GH |
| hCoV-19/Tunisia/S-1037/2021 | EPI_ISL_8298438 | 14/08/2021 | Africa / Tunisia / Nabeul | Human | Random | Female | 45 | unknown | unknown | Random | AY.122 | GK |
| hCoV-19/Tunisia/F-1698/2021 | EPI_ISL_8298404 | 21/08/2021 | Africa / Tunisia / Ariana | Human | Random | Female | 48 | unknown | unknown | Random | AY.122 | GK |
| hCoV-19/Tunisia/F-3539/2021 | EPI_ISL_8298540 | 17/09/2021 | Africa / Tunisia / Tunis | Human | Random | Female | 57 | unknown | unknown | Random | AY.122 | GK |
| hCoV-19/Tunisia/M-0793/2021 | EPI_ISL_10101343 | 08/10/2021 | Africa / Tunisia | Human | unknown | Male | 50 | unknown | unknown | unknown | B.1.160 | GH |
| hCoV-19/Tunisia/Q-7965/2021 | EPI_ISL_10101298 | 16/02/2021 | Africa / Tunisia | Human | unknown | Female | 33 | unknown | unknown | unknown | B.1 | G |
| hCoV-19/Tunisia/Q-6809/2021 | EPI_ISL_10101255 | 09/02/2021 | Africa / Tunisia | Human | unknown | Female | 58 | unknown | unknown | unknown | B.1.160 | GH |
| hCoV-19/Tunisia/F-0820/2021 | EPI_ISL_8298393 | 07/08/2021 | Africa / Tunisia / Ariana | Human | Random | Female | 33 | unknown | unknown | Random | AY.122 | GK |
| hCoV-19/Tunisia/GB-1511/2021 | EPI_ISL_10101193 | 02/04/2021 | Africa / Tunisia | Human | unknown | Female | 62 | unknown | unknown | unknown | B.4 | O |
| hCoV-19/Tunisia/M-1151/2021 | EPI_ISL_10101348 | 09/10/2021 | Africa / Tunisia | Human | unknown | Female | 62 | unknown | unknown | unknown | B.1.428.2 | GH |
| hCoV-19/Tunisia/F-1904/2021 | EPI_ISL_8298483 | 24/08/2021 | Africa / Tunisia / Tunis | Human | Random | Female | 27 | unknown | unknown | Random | AY.122 | GK |
| hCoV-19/Tunisia/A-0209/2021 | EPI_ISL_8298445 | 19/08/2021 | Africa / Tunisia / Tunis | Human | Random | unknown | 21 | unknown | unknown | Random | AY.122 | GK |
| hCoV-19/Tunisia/F-3723/2021 | EPI_ISL_8298546 | 21/09/2021 | Africa / Tunisia / Tunis | Human | Random | Female | 49 | unknown | unknown | Random | AY.122 | GK |
| hCoV-19/Tunisia/M-0650/2021 | EPI_ISL_10101352 | 07/10/2021 | Africa / Tunisia | Human | unknown | Male | 55 | unknown | unknown | unknown | B.1 | G |
| hCoV-19/Tunisia/E-1320/2021 | EPI_ISL_10141394 | 26/06/2021 | Africa / Tunisia / Tunis | Human | unknown | Female | 48 | unknown | No | unknown | AY.122 | GK |
| hCoV-19/Tunisia/GB-2285/2021 | EPI_ISL_10101194 | 07/04/2021 | Africa / Tunisia | Human | unknown | Female | 54 | unknown | unknown | unknown | B.1 | GH |
| hCoV-19/Tunisia/Y-6620/2021 | EPI_ISL_10101284 | 20/10/2021 | Africa / Tunisia | Human | unknown | Female | 88 | unknown | unknown | unknown | B.1 | G |
| hCoV-19/Tunisia/V-1165/2021 | EPI_ISL_8298553 | 19/08/2021 | Africa / Tunisia / Tunis | Human | Random | Male | 38 | unknown | unknown | Random | AY.122 | GK |
| hCoV-19/Tunisia/V-1102/2021 | EPI_ISL_8298440 | 18/08/2021 | Africa / Tunisia / Sidi Bouzid | Human | Random | Male | 31 | unknown | unknown | Random | AY.122 | GK |
| hCoV-19/Tunisia/F-0487/2021 | EPI_ISL_8298449 | 03/08/2021 | Africa / Tunisia / Tunis | Human | Random | Male | 19 | unknown | unknown | Random | AY.122 | GK |
| hCoV-19/Tunisia/F-0942/2021 | EPI_ISL_8298395 | 09/08/2021 | Africa / Tunisia / Ariana | Human | Random | Female | 24 | unknown | unknown | Random | AY.122 | GK |
| hCoV-19/Tunisia/GB-0882/2021 | EPI_ISL_10101280 | 28/03/2021 | Africa / Tunisia | Human | unknown | Male | 46 | unknown | unknown | unknown | B.1 | G |
| hCoV-19/Tunisia/F-3175/2021 | EPI_ISL_8298525 | 10/09/2021 | Africa / Tunisia / Tunis | Human | Random | Male | 54 | unknown | unknown | Random | AY.122 | GK |
| hCoV-19/Tunisia/GB-1522/2021 | EPI_ISL_10101252 | 02/04/2021 | Africa / Tunisia | Human | unknown | Female | 72 | unknown | unknown | unknown | B.4 | O |
| hCoV-19/Tunisia/A-3748/2021 | EPI_ISL_10101187 | 25/07/2021 | Africa / Tunisia | Human | unknown | Female | 45 | unknown | unknown | unknown | B.1.1.50 | GR |
| hCoV-19/Tunisia/Z-4222/2021 | EPI_ISL_10101296 | 09/11/2021 | Africa / Tunisia | Human | unknown | Male | 36 | unknown | unknown | unknown | B.1 | G |
| hCoV-19/Tunisia/S-0829/2021 | EPI_ISL_10141396 | 02/07/2021 | Africa / Tunisia / Tunis | Human | unknown | unknown | 42 | unknown | No | unknown | AY.122 | GK |
| hCoV-19/Tunisia/C-4025/2021 | EPI_ISL_10141496 | 17/04/2021 | Africa / Tunisia / Tunis | Human | unknown | Male | 86 | Hospitalized | No | unknown | B.1.1.7 | GRY |
| hCoV-19/Tunisia/F-2587/2021 | EPI_ISL_8298496 | 01/09/2021 | Africa / Tunisia / Tunis | Human | Random | Male | 17 | unknown | unknown | Random | AY.122 | GK |
| hCoV-19/Tunisia/H-0858/2021 | EPI_ISL_10101197 | 30/11/2021 | Africa / Tunisia | Human | unknown | Male | 40 | unknown | unknown | unknown | B.1.160 | GH |
| hCoV-19/Tunisia/F-2892/2021 | EPI_ISL_8298506 | 06/09/2021 | Africa / Tunisia / Tunis | Human | Random | Male | 35 | unknown | unknown | Random | AY.122 | GK |
| hCoV-19/Tunisia/Q-9784/2021 | EPI_ISL_10101297 | 26/02/2021 | Africa / Tunisia | Human | unknown | Male | 21 | unknown | unknown | unknown | B.1.1 | GRY |
| hCoV-19/Tunisia/S-0750/2021 | EPI_ISL_10141397 | 29/06/2021 | Africa / Tunisia / Medenine | Human | unknown | Male | 46 | unknown | No | unknown | AY.122 | GK |
| hCoV-19/Tunisia/C-4073/2021 | EPI_ISL_10141497 | 17/04/2021 | Africa / Tunisia / Ariana | Human | unknown | Female | unknown | Severe | No | unknown | B.1.1.7 | GRY |
| hCoV-19/Tunisia/F-0983/2021 | EPI_ISL_8298397 | 11/08/2021 | Africa / Tunisia / Ariana | Human | Random | Female | 34 | unknown | unknown | Random | AY.122 | GK |
| hCoV-19/Tunisia/F-2602/2021 | EPI_ISL_8298497 | 01/09/2021 | Africa / Tunisia / Tunis | Human | Random | Male | 11 | unknown | unknown | Random | AY.122 | GK |
| hCoV-19/Tunisia/Y-7370/2021 | EPI_ISL_10101216 | 24/10/2021 | Africa / Tunisia | Human | unknown | Male | 5 | unknown | unknown | unknown | B.1.160 | GH |
| hCoV-19/Tunisia/H-0471/2021 | EPI_ISL_10101276 | 26/11/2021 | Africa / Tunisia | Human | unknown | Female | 1 | unknown | unknown | unknown | B.1.1 | GR |
| hCoV-19/Tunisia/E-511/2021 | EPI_ISL_10141462 | 24/06/2021 | Africa / Tunisia / Tunis | Human | unknown | Female | 31 | unknown | No | unknown | B.1 | GK |
| hCoV-19/Tunisia/S-0442/2021 | EPI_ISL_16955526 | 14/05/2021 | Africa / Tunisia / Gabes | Human | Random | Female | 44 | unknown | unknown | Random | B.1.1.7 | GRY |
| hCoV-19/Tunisia/Z-2571/2021 | EPI_ISL_10101217 | 16/10/2021 | Africa / Tunisia | Human | unknown | Female | 35 | unknown | unknown | unknown | B.1.177 | GV |
| hCoV-19/Tunisia/M-7492/2021 | EPI_ISL_10101204 | 20/11/2021 | Africa / Tunisia | Human | unknown | Male | 14 | unknown | unknown | unknown | B.1.177 | GV |
| hCoV-19/Tunisia/F-1482/2021 | EPI_ISL_8298472 | 18/08/2021 | Africa / Tunisia / Tunis | Human | Random | Male | 32 | unknown | unknown | Random | AY.122 | GK |
| hCoV-19/Tunisia/P3-0256/2021 | EPI_ISL_10101317 | 30/03/2021 | Africa / Tunisia | Human | unknown | Male | 80 | unknown | unknown | unknown | B.1.1 | GR |
| hCoV-19/Tunisia/GB-2289/2021 | EPI_ISL_10101293 | 07/04/2021 | Africa / Tunisia | Human | unknown | Male | 5 | unknown | unknown | unknown | B.1.1 | GH |
| hCoV-19/Tunisia/GB-3353/2021 | EPI_ISL_10101259 | 13/04/2021 | Africa / Tunisia | Human | unknown | Male | 23 | unknown | unknown | unknown | B.1.1 | G |
| hCoV-19/Tunisia//S-0001/2021 | EPI_ISL_17018813 | 01/03/2021 | Africa / Tunisia / Kairouan | Human | Random | Female | 60 | unknown | unknown | Random | B.1.243 | G |
| hCoV-19/Tunisia/S-0606/2021 | EPI_ISL_17018819 | 08/03/2021 | Africa / Tunisia / Monastir | Human | Random | Female | 70 | unknown | unknown | Random | B.1.160 | GH |
| hCoV-19/Tunisia/B-3152/2021 | EPI_ISL_17018810 | 18/03/2021 | Africa / Tunisia | Human | Random | unknown | unknown | unknown | unknown | Random | B.1.177 | GV |
| hCoV-19/Tunisia/GB-0528/2021 | EPI_ISL_10101191 | 27/03/2021 | Africa / Tunisia | Human | unknown | Male | 46 | unknown | unknown | unknown | B.1 | GH |
| hCoV-19/Tunisia/C-3243/2021 | EPI_ISL_10141468 | 12/04/2021 | Africa / Tunisia / Sidi Bouzid | Human | unknown | Male | 34 | Mild | No | unknown | B.1.1.7 | GRY |
| hCoV-19/Tunisia/Tunis_6401/2020 | EPI_ISL_450490 | 29/03/2020 | Africa / Tunisia | Human | unknown | Female | unknown | unknown | unknown | unknown | Unassigned | O |
| hCoV-19/Tunisia/A008/2021 | EPI_ISL_16847410 | 26/02/2021 | Africa / Tunisia / Ariana | Human | Random | Male | 15 | unknown | unknown | Random | B.1.160 | GH |
| hCoV-19/Tunisia/S184/2021 | EPI_ISL_16847409 | 24/03/2021 | Africa / Tunisia / Tunis | Human | Random | Male | 1 year 8 months | unknown | unknown | Random | B.1.1.7 | GRY |
| hCoV-19/Tunisia/F-1449/2021 | EPI_ISL_8298470 | 18/08/2021 | Africa / Tunisia / Tunis | Human | Random | Female | 33 | unknown | unknown | Random | AY.122 | GK |
| hCoV-19/Tunisia/F-1370/2021 | EPI_ISL_8298469 | 17/08/2021 | Africa / Tunisia / Tunis | Human | Random | Male | 19 | unknown | unknown | Random | B.1.617.2 | GK |
| hCoV-19/Tunisia/Y-2931/2021 | EPI_ISL_10101212 | 30/09/2021 | Africa / Tunisia | Human | unknown | Female | 41 | unknown | unknown | unknown | B.1.177 | GV |
| hCoV-19/Tunisia/IPT-G-4414/2023 | EPI_ISL_17543448 | 12/01/2023 | Africa / Tunisia / Tunis | Human | Random | Male | 71 | unknown | unknown | Random | XBB.1.5.52 | GRA |
| hCoV-19/Tunisia/B-5037/2021 | EPI_ISL_10141474 | 28/04/2021 | Africa / Tunisia / Ariana | Human | unknown | Female | 2 months | Mild | No | unknown | B.1.1.7 | GRY |
| hCoV-19/Tunisia/A-3568/2021 | EPI_ISL_10101377 | 24/07/2021 | Africa / Tunisia | Human | unknown | Male | 48 | unknown | unknown | unknown | B.1.1.50 | GR |
| hCoV-19/Tunisia/F-1466/2021 | EPI_ISL_8298399 | 18/08/2021 | Africa / Tunisia / Ariana | Human | Random | Female | 65 | unknown | unknown | Random | AY.122 | GK |
| hCoV-19/Tunisia/F-1836/2021 | EPI_ISL_8298476 | 23/08/2021 | Africa / Tunisia / Tunis | Human | Random | Female | 36 | unknown | unknown | Random | AY.122 | GK |
| hCoV-19/Tunisia/S-0791/2021 | EPI_ISL_17018820 | 29/06/2021 | Africa / Tunisia / Tunis | Human | Random | Female | 36 | unknown | unknown | Random | B.1.177 | GV |
| hCoV-19/Tunisia/F-1980/2021 | EPI_ISL_8298488 | 25/08/2021 | Africa / Tunisia / Tunis | Human | Random | Female | 71 | unknown | unknown | Random | AY.122 | GK |
| hCoV-19/Tunisia/TUN_ADAGE_5004/2020 | EPI_ISL_6974314 | 09/07/2020 | Africa / Tunisia / Sfax | Human | unknown | Male | 26 | Hospitalized | unknown | unknown | B.1.597 | GH |
| hCoV-19/Tunisia/C-3632/2021 | EPI_ISL_10141488 | 15/04/2021 | Africa / Tunisia / Tunis | Human | unknown | Female | 57 | Hospitalized | No | unknown | B.1.1.7 | GRY |
| hCoV-19/Tunisia/U-5951/2021 | EPI_ISL_10141489 | 12/04/2021 | Africa / Tunisia / Ariana | Human | unknown | Female | 60 | Deceased | No | unknown | B.1.1.7 | GRY |
| hCoV-19/Tunisia/TUN_ADAGE_EZ504/2020 | EPI_ISL_7055354 | 31/05/2020 | Africa / Tunisia / Monastir | Human | unknown | Male | 22 | Hospitalized | unknown | unknown | B.1 | GH |
| hCoV-19/Tunisia/F-3576/2021 | EPI_ISL_8298432 | 18/09/2021 | Africa / Tunisia / Kairouan | Human | Random | Female | 21 | unknown | unknown | Random | AY.122 | GK |
| hCoV-19/Tunisia/11-MHT_9/2020 | EPI_ISL_855560 | 13/09/2020 | Africa / Tunisia / Tunis / Tunis | Human | unknown | Female | 53 | unknown | unknown | unknown | B.1.160 | GH |
| hCoV-19/Tunisia/20424/2020 | EPI_ISL_1116467 | 19/10/2020 | Africa / Tunisia / Sfax | Human | unknown | Female | 41 | unknown | unknown | unknown | B.1 | GH |
| hCoV-19/Tunisia/H-4647/2021 | EPI_ISL_10101239 | 23/12/2021 | Africa / Tunisia | Human | unknown | Male | 64 | unknown | unknown | unknown | B.1 | G |
| hCoV-19/Tunisia/C-3846/2021 | EPI_ISL_10141478 | 16/04/2021 | Africa / Tunisia / Sousse | Human | unknown | Female | 39 | Mild | No | unknown | B.1.1.7 | GRY |
| hCoV-19/Tunisia/11-MHT_21/2020 | EPI_ISL_855571 | 15/09/2020 | Africa / Tunisia / Tunis / Tunis | Human | unknown | Male | 68 | unknown | unknown | unknown | B.1.1 | GR |
| hCoV-19/Tunisia/23-MHT_23/2020 | EPI_ISL_855566 | 15/09/2020 | Africa / Tunisia / Bizerte / Mateur | Human | unknown | Female | 42 | unknown | unknown | unknown | B.1.177 | GV |
| hCoV-19/Tunisia/F-1853/2021 | EPI_ISL_8298478 | 23/08/2021 | Africa / Tunisia / Tunis | Human | Random | Female | 52 | unknown | unknown | Random | AY.122 | GK |
| hCoV-19/Tunisia/SP-0202/2021 | EPI_ISL_2035943 | 10/02/2021 | Africa / Tunisia / Tunis | Human | unknown | Female | 63 | unknown | unknown | unknown | B.1.160 | GH |
| hCoV-19/Tunisia/C-3275/2021 | EPI_ISL_10141479 | 12/04/2021 | Africa / Tunisia / Sidi Bouzid | Human | unknown | Male | 45 | Mild | No | unknown | B.1.1.7 | GRY |
| hCoV-19/Tunisia/M-7386/2021 | EPI_ISL_10101333 | 19/11/2021 | Africa / Tunisia | Human | unknown | Male | 91 | unknown | unknown | unknown | B.1 | GH |
| hCoV-19/Tunisia/M-4982/2021 | EPI_ISL_10101234 | 05/11/2021 | Africa / Tunisia | Human | unknown | Male | 81 | unknown | unknown | unknown | B.1.1 | GR |
| hCoV-19/Tunisia/M-3530/2021 | EPI_ISL_10101251 | 01/11/2021 | Africa / Tunisia | Human | unknown | Male | 69 | unknown | unknown | unknown | B.1 | G |
| hCoV-19/Tunisia/Q-6821/2021 | EPI_ISL_10101261 | 09/02/2021 | Africa / Tunisia | Human | unknown | Male | 36 | unknown | unknown | unknown | B.1 | GH |
| hCoV-19/Tunisia/33611/2021 | EPI_ISL_2912512 | 18/05/2021 | Africa / Tunisia / Ariana | Human | unknown | Male | 72 | unknown | unknown | unknown | B.1.1.7 | G |
| hCoV-19/Tunisia/F-1063/2021 | EPI_ISL_8298434 | 11/08/2021 | Africa / Tunisia / Manouba | Human | Random | Male | 46 | unknown | unknown | Random | AY.122 | GK |
| hCoV-19/Tunisia/33266/2021 | EPI_ISL_2913937 | 17/05/2021 | Africa / Tunisia / Ariana | Human | unknown | Male | 61 | unknown | unknown | unknown | B.1.1.7 | GRY |
| hCoV-19/Tunisia/U-6706/2021 | EPI_ISL_10141481 | 27/04/2021 | Africa / Tunisia / Mannouba | Human | unknown | Female | 47 | unknown | No | unknown | B.1.1.7 | GRY |
| hCoV-19/Tunisia/U-6808/2021 | EPI_ISL_10141482 | 29/04/2021 | Africa / Tunisia / Tunis | Human | unknown | Female | 49 | Asymptomatic | No | unknown | B.1.1.7 | GRY |
| hCoV-19/Tunisia/F-1695/2021 | EPI_ISL_8298473 | 21/08/2021 | Africa / Tunisia / Tunis | Human | Random | Female | 26 | unknown | unknown | Random | AY.122 | GK |
| hCoV-19/Tunisia/E-1644/2021 | EPI_ISL_10141431 | 27/06/2021 | Africa / Tunisia / Tunis | Human | unknown | Female | 33 | unknown | No | unknown | B.1 | G |
| hCoV-19/Tunisia/B-0147/2021 | EPI_ISL_10101373 | 02/03/2021 | Africa / Tunisia | Human | unknown | Female | 40 | unknown | unknown | unknown | B.1.160 | GH |
| hCoV-19/Tunisia/B-2334/2021 | EPI_ISL_10101370 | 13/03/2021 | Africa / Tunisia | Human | unknown | Female | 91 | unknown | unknown | unknown | B.1.1.7 | GRY |
| hCoV-19/Tunisia/Y-5991/2021 | EPI_ISL_10101350 | 17/10/2021 | Africa / Tunisia | Human | unknown | Female | 18 | unknown | unknown | unknown | B.1.160 | GH |
| hCoV-19/Tunisia/Q-2838/2021 | EPI_ISL_10101349 | 21/01/2021 | Africa / Tunisia | Human | unknown | Female | 28 | unknown | unknown | unknown | B.1.160 | GH |
| hCoV-19/Tunisia/S-0159/2021 | EPI_ISL_10101347 | 16/03/2021 | Africa / Tunisia | Human | unknown | Female | 26 | unknown | unknown | unknown | A.27 | S |
| hCoV-19/Tunisia/H-9711/2021 | EPI_ISL_10101366 | 18/01/2021 | Africa / Tunisia | Human | unknown | Male | 80 | unknown | unknown | unknown | B.1.160 | GH |
| hCoV-19/Tunisia/Z-1847/2021 | EPI_ISL_10101364 | 28/09/2021 | Africa / Tunisia | Human | unknown | Female | 24 | unknown | unknown | unknown | B.1.428.2 | GH |
| hCoV-19/Tunisia/B-2942/2021 | EPI_ISL_10101365 | 16/03/2021 | Africa / Tunisia | Human | unknown | Female | 8 | unknown | unknown | unknown | B.1 | GH |
| hCoV-19/Tunisia/Q-2413/2021 | EPI_ISL_10101361 | 20/01/2021 | Africa / Tunisia | Human | unknown | Female | 17 | unknown | unknown | unknown | B.1.160 | GH |
| hCoV-19/Tunisia/B-2718/2021 | EPI_ISL_10101358 | 15/03/2021 | Africa / Tunisia | Human | unknown | Male | 105 | unknown | unknown | unknown | B.1.1.7 | GRY |
| hCoV-19/Tunisia/C-4468/2021 | EPI_ISL_10141399 | 21/04/2021 | Africa / Tunisia / Ben Arous | Human | unknown | Female | 20 | Mild | No | unknown | A.27 | S |
| hCoV-19/Tunisia/A-1274/2021 | EPI_ISL_10101369 | 14/06/2021 | Africa / Tunisia | Human | unknown | Male | 48 | unknown | unknown | unknown | B.1.441 | GH |
| hCoV-19/Tunisia/C-4564/2021 | EPI_ISL_10141469 | 21/04/2021 | Africa / Tunisia / Mannouba | Human | unknown | Male | 35 | Hospitalized | No | unknown | B.1.1.7 | GRY |
| hCoV-19/Tunisia/S-0408/2021 | EPI_ISL_10141546 | 18/05/2021 | Africa / Tunisia / Medenine | Human | unknown | Female | 59 | unknown | No | unknown | B.1.1.7 | GRY |
| hCoV-19/Tunisia/A-8246/2020 | EPI_ISL_10141409 | 20/08/2020 | Africa / Tunisia / Tunis | Human | unknown | Female | 58 | Mild | No | unknown | B.1 | G |
| hCoV-19/Tunisia/S-0739/2021 | EPI_ISL_10141398 | 29/06/2021 | Africa / Tunisia / Nabeul | Human | unknown | Female | 37 | unknown | No | unknown | AY.122 | GK |
| hCoV-19/Tunisia/C-5524/2021 | EPI_ISL_10141507 | 24/04/2021 | Africa / Tunisia / Tunis | Human | unknown | Male | 72 | Hospitalized | No | unknown | A.27 | S |
| hCoV-19/Tunisia/S-0206/2021 | EPI_ISL_10141505 | 07/04/2021 | Africa / Tunisia / Sousse | Human | unknown | Female | 39 | unknown | No | unknown | B.1.1.7 | GRY |
| hCoV-19/Tunisia/Y-9527/2021 | EPI_ISL_10141510 | 31/03/2021 | Africa / Tunisia / Tunis | Human | unknown | Female | 77 | Severe | No | unknown | B.1.1.7 | GRY |
| hCoV-19/Tunisia/C-3297/2021 | EPI_ISL_10141519 | 14/04/2021 | Africa / Tunisia / Ariana | Human | unknown | Female | 72 | Mild | No | unknown | B.1.1.7 | GRY |
| hCoV-19/Tunisia/C-4063/2021 | EPI_ISL_10141518 | 17/04/2021 | Africa / Tunisia / Tunis | Human | unknown | Male | 51 | Severe | No | unknown | B.1.1.7 | GRY |
| hCoV-19/Tunisia/C-3392/2021 | EPI_ISL_10141515 | 14/04/2021 | Africa / Tunisia / Ben Arous | Human | unknown | Female | 54 | Severe | No | unknown | B.1.177 | GV |
| hCoV-19/Tunisia/C-3248/2021 | EPI_ISL_10141527 | 12/04/2021 | Africa / Tunisia / Sidi Bouzid | Human | unknown | Female | 63 | Mild | No | unknown | B.1.525 | G |
| hCoV-19/Tunisia/C-5365/2021 | EPI_ISL_10141535 | 23/04/2021 | Africa / Tunisia / Ariana | Human | unknown | Male | 47 | Hospitalized | No | unknown | B.1.1.7 | GRY |
| hCoV-19/Tunisia/B-5263/2021 | EPI_ISL_10141533 | 30/04/2021 | Africa / Tunisia / Tunis | Human | unknown | Male | 67 | Mild | No | unknown | B.1.1.7 | GRY |
| hCoV-19/Tunisia/S-0704/2021 | EPI_ISL_10141446 | 29/06/2021 | Africa / Tunisia / Kairouen | Human | unknown | Female | 11 | unknown | No | unknown | AY.122 | GK |
| hCoV-19/Tunisia/S-0716/2021 | EPI_ISL_10141443 | 29/06/2021 | Africa / Tunisia / Kairouen | Human | unknown | Female | 4 months | unknown | No | unknown | AY.122 | GK |
| hCoV-19/Tunisia/X-3429/2020 | EPI_ISL_10141444 | 31/08/2020 | Africa / Tunisia / Ariana | Human | unknown | Male | 33 | Mild | No | unknown | B.1 | G |
| hCoV-19/Tunisia/S-0755/2021 | EPI_ISL_10141441 | 29/06/2021 | Africa / Tunisia / Medenine | Human | unknown | Male | 54 | unknown | No | unknown | B.1.1.7 | GRY |
| hCoV-19/Tunisia/S-0762/2021 | EPI_ISL_10141442 | 29/06/2021 | Africa / Tunisia / Medenine | Human | unknown | Male | 53 | unknown | No | unknown | AY.122 | GK |
| hCoV-19/Tunisia/E-1757/2021 | EPI_ISL_10141449 | 27/06/2021 | Africa / Tunisia / Tunis | Human | unknown | Female | 44 | unknown | No | unknown | AY.122 | GK |
| hCoV-19/Tunisia/C-711/2021 | EPI_ISL_10141458 | 05/04/2021 | Africa / Tunisia / Tunis | Human | unknown | Female | 80 | Severe | No | unknown | B.1 | G |
| hCoV-19/Tunisia/S-0847/2021 | EPI_ISL_10141459 | 05/07/2021 | Africa / Tunisia / Sousse | Human | unknown | Female | 38 | unknown | No | unknown | AY.122 | GK |
| hCoV-19/Tunisia/E-538/2021 | EPI_ISL_10141457 | 24/06/2021 | Africa / Tunisia / Tunis | Human | unknown | Female | 44 | unknown | No | unknown | B.1.617.2 | GK |
| hCoV-19/Tunisia/SP-0377/2021 | EPI_ISL_2035948 | 04/03/2021 | Africa / Tunisia / Silina | Human | unknown | Male | unknown | unknown | unknown | unknown | B.1.177 | GV |
| hCoV-19/Tunisia/COV0880/2020 | EPI_ISL_463002 | 28/03/2020 | Africa / Tunisia | Human | unknown | unknown | unknown | unknown | unknown | unknown | B.4 | O |
| hCoV-19/Tunisia/COV0010-12/2020 | EPI_ISL_463001 | 18/03/2020 | Africa / Tunisia | Human | unknown | unknown | unknown | unknown | unknown | unknown | A | S |
| hCoV-19/Tunisia/16883/2022 | EPI_ISL_15298484 | 08/07/2022 | Africa / Tunisia / Tunis | Human | Baseline surveillance | Male | 37 | unknown | unknown | Baseline surveillance | BA.4 | GRA |
| hCoV-19/Tunisia/COV1339/2020 | EPI_ISL_463003 | 30/03/2020 | Africa / Tunisia | Human | unknown | unknown | unknown | unknown | unknown | unknown | B.1.9 | GH |
| hCoV-19/Tunisia/16644/2022 | EPI_ISL_15298483 | 06/07/2022 | Africa / Tunisia / Tunis | Human | Baseline surveillance | Female | unknown | unknown | unknown | Baseline surveillance | BA.5.2 | GRA |
| hCoV-19/Tunisia/16521/2022 | EPI_ISL_15298482 | 04/07/2022 | Africa / Tunisia / Tunis | Human | Baseline surveillance | Female | 39 | unknown | unknown | Baseline surveillance | BA.5.2 | GRA |
| hCoV-19/Tunisia/15981/2022 | EPI_ISL_15298481 | 25/06/2022 | Africa / Tunisia / Tunis | Human | Baseline surveillance | Female | 77 | unknown | unknown | Baseline surveillance | BA.5.2.20 | GRA |
| hCoV-19/Tunisia/15979/2022 | EPI_ISL_15298480 | 25/06/2022 | Africa / Tunisia / Tunis | Human | Baseline surveillance | Male | 71 | unknown | unknown | Baseline surveillance | BA.5.2 | GRA |
| hCoV-19/Tunisia/53158/2022 | EPI_ISL_15298479 | 22/06/2022 | Africa / Tunisia / Tunis | Human | Baseline surveillance | Male | 5 | unknown | unknown | Baseline surveillance | BA.5.2.20 | GRA |
| hCoV-19/Tunisia/50862/2022 | EPI_ISL_15298478 | 09/02/2022 | Africa / Tunisia / Tunis | Human | Baseline surveillance | Male | 19 | unknown | unknown | Baseline surveillance | BA.1.1 | GRA |
| hCoV-19/Tunisia/15581/2022 | EPI_ISL_15298477 | 17/06/2022 | Africa / Tunisia / Tunis | Human | Baseline surveillance | Male | 69 | unknown | unknown | Baseline surveillance | BA.2 | GRA |
| hCoV-19/Tunisia/14893/2022 | EPI_ISL_15298476 | 31/05/2022 | Africa / Tunisia / Tunis | Human | Baseline surveillance | Female | 54 | unknown | unknown | Baseline surveillance | BA.2 | GRA |
| hCoV-19/Tunisia/14851/2022 | EPI_ISL_15298475 | 30/05/2022 | Africa / Tunisia / Tunis | Human | Baseline surveillance | Male | 14 | unknown | unknown | Baseline surveillance | BA.5.1 | GRA |
| hCoV-19/Tunisia/14635/2022 | EPI_ISL_15298474 | 25/05/2022 | Africa / Tunisia / Tunis | Human | Baseline surveillance | Male | 25 | unknown | unknown | Baseline surveillance | B.1.177 | GV |
| hCoV-19/Tunisia/14085/2022 | EPI_ISL_15298473 | 13/05/2022 | Africa / Tunisia / Tunis | Human | Baseline surveillance | Female | 2 | unknown | unknown | Baseline surveillance | BA.2 | GRA |
| hCoV-19/Tunisia/633/2022 | EPI_ISL_15298472 | 05/07/2022 | Africa / Tunisia / Tunis | Human | Baseline surveillance | Female | 68 | unknown | unknown | Baseline surveillance | BA.5.2 | GRA |
| hCoV-19/Tunisia/15957/2022 | EPI_ISL_15298471 | 25/06/2022 | Africa / Tunisia / Tunis | Human | Baseline surveillance | Female | 1 | unknown | unknown | Baseline surveillance | BA.5.2.20 | GRA |
| hCoV-19/Tunisia/122906/2022 | EPI_ISL_15298470 | 29/06/2022 | Africa / Tunisia / Tunis | Human | Baseline surveillance | Male | 54 | unknown | unknown | Baseline surveillance | BA.2.40.1 | GRA |
| hCoV-19/Tunisia/123006/2022 | EPI_ISL_15298469 | 30/06/2022 | Africa / Tunisia / Tunis | Human | Baseline surveillance | Male | 83 | unknown | unknown | Baseline surveillance | BA.2.40.1 | GRA |
| hCoV-19/Tunisia/112906/2022 | EPI_ISL_15298468 | 29/06/2022 | Africa / Tunisia / Tunis | Human | Baseline surveillance | Female | 36 | unknown | unknown | Baseline surveillance | BA.5.2.20 | GRA |
| hCoV-19/Tunisia/102806/2022 | EPI_ISL_15298467 | 28/06/2022 | Africa / Tunisia / Tunis | Human | Baseline surveillance | Male | 62 | unknown | unknown | Baseline surveillance | BA.5.2 | GRA |
| hCoV-19/Tunisia/92706/2022 | EPI_ISL_15298466 | 27/06/2022 | Africa / Tunisia / Tunis | Human | Baseline surveillance | Male | 88 | unknown | unknown | Baseline surveillance | BA.5.2.20 | GRA |
| hCoV-19/Tunisia/82706 /2022 | EPI_ISL_15298465 | 27/06/2022 | Africa / Tunisia / Tunis | Human | Baseline surveillance | Female | 76 | unknown | unknown | Baseline surveillance | BA.5.2 | GRA |
| hCoV-19/Tunisia/72506/2022 | EPI_ISL_15298464 | 25/06/2022 | Africa / Tunisia / Tunis | Human | Baseline surveillance | Male | 26 | unknown | unknown | Baseline surveillance | BA.5.2.20 | GRA |
| hCoV-19/Tunisia/62106/2022 | EPI_ISL_15298463 | 21/06/2022 | Africa / Tunisia / Tunis | Human | Baseline surveillance | Female | 57 | unknown | unknown | Baseline surveillance | BA.5.2 | GRA |
| hCoV-19/Tunisia/32306/2022 | EPI_ISL_15298462 | 23/06/2022 | Africa / Tunisia / Tunis | Human | Baseline surveillance | Female | unknown | unknown | unknown | Baseline surveillance | BA.2 | GRA |
| hCoV-19/Tunisia/22306/2022 | EPI_ISL_15298461 | 23/06/2022 | Africa / Tunisia / Tunis | Human | Baseline surveillance | Male | 55 | unknown | unknown | Baseline surveillance | Unassigned | G |
| hCoV-19/Tunisia/11405/2022 | EPI_ISL_15298460 | 14/05/2022 | Africa / Tunisia / Tunis | Human | Baseline surveillance | Male | 55 | unknown | unknown | Baseline surveillance | BA.4 | G |
| hCoV-19/Tunisia/15580/2022 | EPI_ISL_15298459 | 12/06/2022 | Africa / Tunisia / Soliman | Human | Baseline surveillance | Male | 13 | unknown | unknown | Baseline surveillance | BA.4 | GRA |
| hCoV-19/Tunisia/16500/2022 | EPI_ISL_15298458 | 04/07/2022 | Africa / Tunisia / Nabeul | Human | Baseline surveillance | Female | 66 | unknown | unknown | Baseline surveillance | BA.5.2 | GRA |
| hCoV-19/Tunisia/16010/2022 | EPI_ISL_15298457 | 25/06/2022 | Africa / Tunisia / Nabeul | Human | Baseline surveillance | Male | unknown | unknown | unknown | Baseline surveillance | BA.5.2.20 | GRA |
| hCoV-19/Tunisia/19006/2022 | EPI_ISL_15298456 | 25/06/2022 | Africa / Tunisia / Nabeul | Human | Baseline surveillance | Male | 89 | unknown | unknown | Baseline surveillance | BA.2 | G |
| hCoV-19/Tunisia/15999/2022 | EPI_ISL_15298455 | 25/06/2022 | Africa / Tunisia / Nabeul | Human | Baseline surveillance | Male | 26 | unknown | unknown | Baseline surveillance | BA.5.2.20 | GRA |
| hCoV-19/Tunisia/15996/2022 | EPI_ISL_15298454 | 25/06/2022 | Africa / Tunisia / Nabeul | Human | Baseline surveillance | Female | 64 | unknown | unknown | Baseline surveillance | BA.2 | GRA |
| hCoV-19/Tunisia/15970/2022 | EPI_ISL_15298453 | 25/06/2022 | Africa / Tunisia / Nabeul | Human | Baseline surveillance | Male | unknown | unknown | unknown | Baseline surveillance | BA.5.2.20 | GRA |
| hCoV-19/Tunisia/8891/2022 | EPI_ISL_15298452 | 24/06/2022 | Africa / Tunisia / Nabeul | Human | Baseline surveillance | Male | 67 | unknown | unknown | Baseline surveillance | BA.2 | GRA |
| hCoV-19/Tunisia/8840/2022 | EPI_ISL_15298451 | 23/06/2022 | Africa / Tunisia / Nabeul | Human | Baseline surveillance | Female | unknown | unknown | unknown | Baseline surveillance | BA.5.2.20 | GRA |
| hCoV-19/Tunisia/8839/2022 | EPI_ISL_15298450 | 23/06/2022 | Africa / Tunisia / Nabeul | Human | Baseline surveillance | Female | 56 | unknown | unknown | Baseline surveillance | BA.2.40.1 | GRA |
| hCoV-19/Tunisia/8827/2022 | EPI_ISL_15298449 | 23/06/2022 | Africa / Tunisia / Nabeul | Human | Baseline surveillance | Male | 40 | unknown | unknown | Baseline surveillance | BA.5.1 | GRA |
| hCoV-19/Tunisia/8797/2022 | EPI_ISL_15298448 | 22/06/2022 | Africa / Tunisia / Nabeul | Human | Baseline surveillance | Female | 63 | unknown | unknown | Baseline surveillance | BA.5.2 | GRA |
| hCoV-19/Tunisia/8792/2022 | EPI_ISL_15298447 | 22/06/2022 | Africa / Tunisia / Nabeul | Human | Baseline surveillance | Male | 35 | unknown | unknown | Baseline surveillance | BA.5.2.20 | GRA |
| hCoV-19/Tunisia/319/2022 | EPI_ISL_15298446 | 21/06/2022 | Africa / Tunisia / Nabeul | Human | Baseline surveillance | Female | 46 | unknown | unknown | Baseline surveillance | BA.5.2 | GRA |
| hCoV-19/Tunisia/293/2022 | EPI_ISL_15298445 | 13/06/2022 | Africa / Tunisia / Nabeul | Human | Baseline surveillance | Female | 28 | unknown | unknown | Baseline surveillance | BA.5.2.20 | GRA |
| hCoV-19/Tunisia/15356/2022 | EPI_ISL_15298444 | 11/06/2022 | Africa / Tunisia / Nabeul | Human | Baseline surveillance | Male | 59 | unknown | unknown | Baseline surveillance | BA.5.2.20 | GRA |
| hCoV-19/Tunisia/14473/2022 | EPI_ISL_15298443 | 21/05/2022 | Africa / Tunisia / Nabeul | Human | Baseline surveillance | Male | 42 | unknown | unknown | Baseline surveillance | BA.2 | GRA |
| hCoV-19/Tunisia/14107/2022 | EPI_ISL_15298442 | 13/05/2022 | Africa / Tunisia / Nabeul | Human | Baseline surveillance | Female | 57 | unknown | unknown | Baseline surveillance | BA.2 | GRA |
| hCoV-19/Tunisia/16849/2022 | EPI_ISL_15298441 | 08/07/2022 | Africa / Tunisia / Nabeul | Human | Baseline surveillance | Female | 71 | unknown | unknown | Baseline surveillance | BA.2 | GRA |
| hCoV-19/Tunisia/16426/2022 | EPI_ISL_15298440 | 02/07/2022 | Africa / Tunisia / Menzel Temime | Human | Baseline surveillance | Male | 60 | unknown | unknown | Baseline surveillance | BA.5.1.10 | GRA |
| hCoV-19/Tunisia/15976/2022 | EPI_ISL_15298439 | 25/06/2022 | Africa / Tunisia / Menzel Bouzelfa | Human | Baseline surveillance | Female | 33 | unknown | unknown | Baseline surveillance | BA.2 | GRA |
| hCoV-19/Tunisia/17071/2022 | EPI_ISL_15298438 | 13/07/2022 | Africa / Tunisia / Menzel Bouzelfa | Human | Baseline surveillance | Male | 48 | unknown | unknown | Baseline surveillance | BA.5.2.20 | GRA |
| hCoV-19/Tunisia/17069/2022 | EPI_ISL_15298437 | 13/07/2022 | Africa / Tunisia / Menzel Bouzelfa | Human | Baseline surveillance | Male | 69 | unknown | unknown | Baseline surveillance | BA.5.2 | GRA |
| hCoV-19/Tunisia/16286/2022 | EPI_ISL_15298436 | 30/06/2022 | Africa / Tunisia / Menzel Bouzelfa | Human | Baseline surveillance | Female | 41 | unknown | unknown | Baseline surveillance | BA.5.2.20 | GRA |
| hCoV-19/Tunisia/16283/2022 | EPI_ISL_15298435 | 30/06/2022 | Africa / Tunisia / Menzel Bouzelfa | Human | Baseline surveillance | Female | 43 | unknown | unknown | Baseline surveillance | BA.2 | GRA |
| hCoV-19/Tunisia/16281/2022 | EPI_ISL_15298434 | 30/06/2022 | Africa / Tunisia / Menzel Bouzelfa | Human | Baseline surveillance | Female | 20 | unknown | unknown | Baseline surveillance | BA.5.2.20 | GRA |
| hCoV-19/Tunisia/16280/2022 | EPI_ISL_15298433 | 30/06/2022 | Africa / Tunisia / Menzel Bouzelfa | Human | Baseline surveillance | Female | 34 | unknown | unknown | Baseline surveillance | BA.5.2 | GRA |
| hCoV-19/Tunisia/53456/2022 | EPI_ISL_15298432 | 02/07/2022 | Africa / Tunisia / Medenine | Human | Baseline surveillance | Male | 19 | unknown | unknown | Baseline surveillance | BA.5.2.20 | GRA |
| hCoV-19/Tunisia/14996/2022 | EPI_ISL_15298431 | 02/06/2022 | Africa / Tunisia / Manzel Temim | Human | Baseline surveillance | Female | 72 | unknown | unknown | Baseline surveillance | BA.2 | GRA |
| hCoV-19/Tunisia/16016/2022 | EPI_ISL_15298430 | 25/06/2022 | Africa / Tunisia / Korba | Human | Baseline surveillance | Female | 44 | unknown | unknown | Baseline surveillance | BA.5.2.20 | GRA |
| hCoV-19/Tunisia/984/2022 | EPI_ISL_15298429 | 15/07/2022 | Africa / Tunisia / Kef | Human | Baseline surveillance | Female | 55 | unknown | unknown | Baseline surveillance | BA.5.2 | GRA |
| hCoV-19/Tunisia/621/2022 | EPI_ISL_15298428 | 04/07/2022 | Africa / Tunisia / Kef | Human | Baseline surveillance | Female | 14 | unknown | unknown | Baseline surveillance | BA.5.2 | GRA |
| hCoV-19/Tunisia/617/2022 | EPI_ISL_15298427 | 02/07/2022 | Africa / Tunisia / Kef | Human | Baseline surveillance | Female | 64 | unknown | unknown | Baseline surveillance | BA.5.2.20 | GRA |
| hCoV-19/Tunisia/610/2022 | EPI_ISL_15298426 | 02/07/2022 | Africa / Tunisia / Kef | Human | Baseline surveillance | Female | 77 | unknown | unknown | Baseline surveillance | BE.1.1 | GRA |
| hCoV-19/Tunisia/604/2022 | EPI_ISL_15298425 | 04/07/2022 | Africa / Tunisia / Kef | Human | Baseline surveillance | Female | 40 | unknown | unknown | Baseline surveillance | BA.2.36 | GRA |
| hCoV-19/Tunisia/603/2022 | EPI_ISL_15298424 | 02/07/2022 | Africa / Tunisia / Kef | Human | Baseline surveillance | Female | 55 | unknown | unknown | Baseline surveillance | BA.2 | GRA |
| hCoV-19/Tunisia/600/2022 | EPI_ISL_15298423 | 02/07/2022 | Africa / Tunisia / Kef | Human | Baseline surveillance | Male | 76 | unknown | unknown | Baseline surveillance | BA.5.2.20 | GRA |
| hCoV-19/Tunisia/593/2022 | EPI_ISL_15298422 | 02/07/2022 | Africa / Tunisia / Kef | Human | Baseline surveillance | Male | 37 | unknown | unknown | Baseline surveillance | BE.1.1 | GRA |
| hCoV-19/Tunisia/591/2022 | EPI_ISL_15298421 | 02/07/2022 | Africa / Tunisia / Kef | Human | Baseline surveillance | Female | 46 | unknown | unknown | Baseline surveillance | BA.5.2 | GRA |
| hCoV-19/Tunisia/588/2022 | EPI_ISL_15298420 | 02/07/2022 | Africa / Tunisia / Kef | Human | Baseline surveillance | Female | 69 | unknown | unknown | Baseline surveillance | BA.5.2.20 | GRA |
| hCoV-19/Tunisia/575/2022 | EPI_ISL_15298419 | 02/07/2022 | Africa / Tunisia / Kef | Human | Baseline surveillance | Female | 117 | unknown | unknown | Baseline surveillance | BA.5.2.20 | GRA |
| hCoV-19/Tunisia/574/2022 | EPI_ISL_15298418 | 02/07/2022 | Africa / Tunisia / Kef | Human | Baseline surveillance | Female | 66 | unknown | unknown | Baseline surveillance | BA.5.2 | GRA |
| hCoV-19/Tunisia/570/2022 | EPI_ISL_15298417 | 02/07/2022 | Africa / Tunisia / Kef | Human | Baseline surveillance | Female | 56 | unknown | unknown | Baseline surveillance | BE.1 | GRA |
| hCoV-19/Tunisia/568/2022 | EPI_ISL_15298416 | 02/07/2022 | Africa / Tunisia / Kef | Human | Baseline surveillance | Female | 31 | unknown | unknown | Baseline surveillance | BA.5.2.20 | GRA |
| hCoV-19/Tunisia/565/2022 | EPI_ISL_15298415 | 02/07/2022 | Africa / Tunisia / Kef | Human | Baseline surveillance | Female | 31 | unknown | unknown | Baseline surveillance | BA.5.2.20 | GRA |
| hCoV-19/Tunisia/562/2022 | EPI_ISL_15298414 | 02/07/2022 | Africa / Tunisia / Kef | Human | Baseline surveillance | Female | 39 | unknown | unknown | Baseline surveillance | BE.1.1 | GRA |
| hCoV-19/Tunisia/561/2022 | EPI_ISL_15298413 | 02/07/2022 | Africa / Tunisia / Kef | Human | Baseline surveillance | Female | 59 | unknown | unknown | Baseline surveillance | BA.5.2 | GRA |
| hCoV-19/Tunisia/542/2022 | EPI_ISL_15298412 | 01/07/2022 | Africa / Tunisia / Kef | Human | Baseline surveillance | Female | 32 | unknown | unknown | Baseline surveillance | BA.5.2.20 | GRA |
| hCoV-19/Tunisia/540/2022 | EPI_ISL_15298411 | 01/07/2022 | Africa / Tunisia / Kef | Human | Baseline surveillance | Female | 52 | unknown | unknown | Baseline surveillance | BA.5.2 | GRA |
| hCoV-19/Tunisia/537/2022 | EPI_ISL_15298410 | 01/07/2022 | Africa / Tunisia / Kef | Human | Baseline surveillance | Male | 71 | unknown | unknown | Baseline surveillance | BA.5.2 | GRA |
| hCoV-19/Tunisia/53528/2022 | EPI_ISL_15298409 | 05/07/2022 | Africa / Tunisia / Kasserine | Human | Baseline surveillance | Female | 32 | unknown | unknown | Baseline surveillance | BA.2 | GRA |
| hCoV-19/Tunisia/53348/2022 | EPI_ISL_15298408 | 29/06/2022 | Africa / Tunisia / Kasserine | Human | Baseline surveillance | Female | 32 | unknown | unknown | Baseline surveillance | BA.5.2.20 | GRA |
| hCoV-19/Tunisia/15359/2022 | EPI_ISL_15298407 | 11/06/2022 | Africa / Tunisia / Grombelia | Human | Baseline surveillance | Female | 73 | unknown | unknown | Baseline surveillance | BA.2 | GRA |
| hCoV-19/Tunisia/14616/2022 | EPI_ISL_15298406 | 25/05/2022 | Africa / Tunisia / Grombelia | Human | Baseline surveillance | Male | 59 | unknown | unknown | Baseline surveillance | BA.2 | GRA |
| hCoV-19/Tunisia/16295/2022 | EPI_ISL_15298405 | 30/06/2022 | Africa / Tunisia / Beni Khalled | Human | Baseline surveillance | Female | 54 | unknown | unknown | Baseline surveillance | BA.5.2.20 | GRA |
| hCoV-19/Tunisia/15862/2022 | EPI_ISL_15298404 | 23/06/2022 | Africa / Tunisia / Beni Khalled | Human | Baseline surveillance | Male | 18 days | unknown | unknown | Baseline surveillance | BA.5.2.20 | GRA |
| hCoV-19/Tunisia/16313/2022 | EPI_ISL_15298403 | 30/06/2022 | Africa / Tunisia / Beni Khalled | Human | Baseline surveillance | Female | 70 | unknown | unknown | Baseline surveillance | BA.5.2 | GRA |
| hCoV-19/Tunisia/16287/2022 | EPI_ISL_15298402 | 30/06/2022 | Africa / Tunisia / Beni Khalled | Human | Baseline surveillance | Female | 31 | unknown | unknown | Baseline surveillance | BA.5.2.20 | GRA |
| hCoV-19/Tunisia/52106/2022 | EPI_ISL_15298401 | 21/06/2022 | Africa / Tunisia / Ben Arous | Human | Baseline surveillance | Male | unknown | unknown | unknown | Baseline surveillance | BA.2 | GRA |
| hCoV-19/Tunisia/53026/2022 | EPI_ISL_15298400 | 20/06/2022 | Africa / Tunisia / Ariana | Human | Baseline surveillance | Female | 33 | unknown | unknown | Baseline surveillance | BA.2 | GRA |
| hCoV-19/Tunisia/52664/2022 | EPI_ISL_15298399 | 05/05/2022 | Africa / Tunisia / Ariana | Human | Baseline surveillance | Male | 35 | unknown | unknown | Baseline surveillance | BA.4 | GRA |
| hCoV-19/Tunisia/51211/2022 | EPI_ISL_15298398 | 15/02/2022 | Africa / Tunisia / Ariana | Human | Baseline surveillance | Male | 50 | unknown | unknown | Baseline surveillance | BA.1.17.2 | GRA |
| hCoV-19/Tunisia/SP-0154/2021 | EPI_ISL_2035941 | 07/02/2021 | Africa / Tunisia / Tunis | Human | unknown | Male | unknown | unknown | unknown | unknown | A.27 | S |
| hCoV-19/Tunisia/S849/2022 | EPI_ISL_15022573 | 27/06/2022 | Africa / Tunisia / Sfax | Human | Baseline surveillance | Female | 33 | unknown | unknown | Baseline surveillance | BA.5.2 | GRA |
| hCoV-19/Tunisia/S841/2022 | EPI_ISL_15022572 | 25/06/2022 | Africa / Tunisia / Sfax | Human | Baseline surveillance | Male | 2 months | Hospitalized | unknown | Baseline surveillance | BA.5.2 | GRA |
| hCoV-19/Tunisia/S839/2022 | EPI_ISL_15022571 | 24/06/2022 | Africa / Tunisia / Sfax | Human | Baseline surveillance | Male | 57 | unknown | unknown | Baseline surveillance | BA.4 | GRA |
| hCoV-19/Tunisia/S823/2022 | EPI_ISL_15022570 | 21/06/2022 | Africa / Tunisia / Sfax | Human | Baseline surveillance | Female | 26 | unknown | Dose 2 :Moderna: 11/09/2021 | Baseline surveillance | BA.4 | GRA |
| hCoV-19/Tunisia/S819/2022 | EPI_ISL_15022569 | 20/06/2022 | Africa / Tunisia / Sfax | Human | Baseline surveillance | Female | 33 | unknown | unknown | Baseline surveillance | BA.5.2 | GRA |
| hCoV-19/Tunisia/S815/2022 | EPI_ISL_15022568 | 17/06/2022 | Africa / Tunisia / Sfax | Human | Baseline surveillance | Female | 62 | unknown | unknown | Baseline surveillance | BA.5.2 | GRA |
| hCoV-19/Tunisia/S814/2022 | EPI_ISL_15022567 | 17/06/2022 | Africa / Tunisia / Sfax | Human | Baseline surveillance | Female | 43 | unknown | unknown | Baseline surveillance | BA.5.2 | GRA |
| hCoV-19/Tunisia/S812/2022 | EPI_ISL_15022566 | 15/06/2022 | Africa / Tunisia / Sfax | Human | Baseline surveillance | Male | 49 | unknown | unknown | Baseline surveillance | BA.5.2 | GRA |
| hCoV-19/Tunisia/S797/2022 | EPI_ISL_15022565 | 08/06/2022 | Africa / Tunisia / Sfax | Human | Baseline surveillance | Female | 48 | unknown | unknown | Baseline surveillance | BA.5.2 | GRA |
| hCoV-19/Tunisia/S796/2022 | EPI_ISL_15022564 | 08/06/2022 | Africa / Tunisia / Sfax | Human | Baseline surveillance | Male | 59 | unknown | unknown | Baseline surveillance | BA.5.2.1 | GRA |
| hCoV-19/Tunisia/S794/2022 | EPI_ISL_15022563 | 07/06/2022 | Africa / Tunisia / Sfax | Human | Baseline surveillance | Female | 54 | unknown | unknown | Baseline surveillance | BA.4 | GRA |
| hCoV-19/Tunisia/S791/2022 | EPI_ISL_15022562 | 06/06/2022 | Africa / Tunisia / Sfax | Human | Baseline surveillance | Female | 57 | unknown | unknown | Baseline surveillance | BA.5.2 | GRA |
| hCoV-19/Tunisia/S724/2022 | EPI_ISL_15022561 | 23/03/2022 | Africa / Tunisia / Sfax | Human | Baseline surveillance | Female | 59 | Hospitalized | unknown | Baseline surveillance | BA.1.1 | GRA |
| hCoV-19/Tunisia/S722/2022 | EPI_ISL_15022560 | 19/06/2022 | Africa / Tunisia / Sfax | Human | Baseline surveillance | Female | 38 | unknown | unknown | Baseline surveillance | BA.5.2 | GRA |
| hCoV-19/Tunisia/S719/2022 | EPI_ISL_15022559 | 23/03/2022 | Africa / Tunisia / Sidi Bouzid | Human | Baseline surveillance | Female | 2 | Hospitalized | unknown | Baseline surveillance | BA.1.1 | GRA |
| hCoV-19/Tunisia/1215/2022 | EPI_ISL_15022558 | 11/01/2022 | Africa / Tunisia / Tunis | Human | Baseline surveillance | Male | 49 | unknown | unknown | Baseline surveillance | B.1.1.529 | GRA |
| hCoV-19/Tunisia/152103/2022 | EPI_ISL_15022557 | 21/03/2022 | Africa / Tunisia / Tunis | Human | Baseline surveillance | Male | 64 | unknown | unknown | Baseline surveillance | AY.122 | GK |
| hCoV-19/Tunisia/54682/2021 | EPI_ISL_15022556 | 27/08/2021 | Africa / Tunisia / Tunis | Human | Baseline surveillance | Female | 25 | unknown | unknown | Baseline surveillance | AY.122 | GK |
| hCoV-19/Tunisia/442/2022 | EPI_ISL_15022555 | 06/01/2022 | Africa / Tunisia / Tunis | Human | Baseline surveillance | Female | 35 | unknown | unknown | Baseline surveillance | AY.122 | GK |
| hCoV-19/Tunisia/430/2022 | EPI_ISL_15022554 | 06/01/2022 | Africa / Tunisia / Tunis | Human | Baseline surveillance | Female | 61 | unknown | unknown | Baseline surveillance | AY.122 | GK |
| hCoV-19/Tunisia/53267/2021 | EPI_ISL_15022553 | 16/08/2021 | Africa / Tunisia / Tunis | Human | Baseline surveillance | Male | 31 | unknown | unknown | Baseline surveillance | AY.122 | O |
| hCoV-19/Tunisia/1289/2022 | EPI_ISL_15022552 | 11/01/2022 | Africa / Tunisia / Tunis | Human | Baseline surveillance | Female | 40 | unknown | unknown | Baseline surveillance | BA.1.1.1 | GRA |
| hCoV-19/Tunisia/64930/2021 | EPI_ISL_15022551 | 31/12/2021 | Africa / Tunisia / Tunis | Human | Baseline surveillance | Male | 56 | unknown | unknown | Baseline surveillance | BA.1.1 | GRA |
| hCoV-19/Tunisia/1441/2022 | EPI_ISL_15022550 | 12/01/2022 | Africa / Tunisia / Tunis | Human | Baseline surveillance | Female | 37 | unknown | unknown | Baseline surveillance | B.1.1.529 | GRA |
| hCoV-19/Tunisia/1426/2022 | EPI_ISL_15022549 | 12/01/2022 | Africa / Tunisia / Tunis | Human | Baseline surveillance | Male | 25 | unknown | unknown | Baseline surveillance | BA.1.1 | GRA |
| hCoV-19/Tunisia/1250/2022 | EPI_ISL_15022548 | 11/01/2022 | Africa / Tunisia / Tunis | Human | Baseline surveillance | Female | 60 | unknown | unknown | Baseline surveillance | AY.78 | GK |
| hCoV-19/Tunisia/3174/2022 | EPI_ISL_15022547 | 17/01/2022 | Africa / Tunisia / Tunis | Human | Baseline surveillance | Female | 44 | unknown | unknown | Baseline surveillance | B.1.617.2 | GK |
| hCoV-19/Tunisia/904/2022 | EPI_ISL_15022546 | 08/01/2022 | Africa / Tunisia / Tunis | Human | Baseline surveillance | Male | 51 | unknown | unknown | Baseline surveillance | AY.122 | GK |
| hCoV-19/Tunisia/325/2022 | EPI_ISL_15022545 | 06/01/2022 | Africa / Tunisia / Tunis | Human | Baseline surveillance | Female | 25 | unknown | unknown | Baseline surveillance | BA.1.1 | GRA |
| hCoV-19/Tunisia/139/2022 | EPI_ISL_15022544 | 04/01/2022 | Africa / Tunisia / Tunis | Human | Baseline surveillance | Female | 28 | unknown | unknown | Baseline surveillance | AY.122 | GK |
| hCoV-19/Tunisia/162103/2022 | EPI_ISL_15022543 | 21/03/2022 | Africa / Tunisia / Tunis | Human | Baseline surveillance | Male | 47 | unknown | unknown | Baseline surveillance | AY.122 | GK |
| hCoV-19/Tunisia/64812/2021 | EPI_ISL_15022542 | 30/12/2021 | Africa / Tunisia / Tunis | Human | Baseline surveillance | Male | 35 | unknown | unknown | Baseline surveillance | BA.1 | GRA |
| hCoV-19/Tunisia/49638/2021 | EPI_ISL_15022541 | 21/07/2021 | Africa / Tunisia / Tunis | Human | Baseline surveillance | Male | 35 | unknown | unknown | Baseline surveillance | AY.122 | GK |
| hCoV-19/Tunisia/1194/2022 | EPI_ISL_15022540 | 11/01/2022 | Africa / Tunisia / Tunis | Human | Baseline surveillance | Female | 39 | unknown | unknown | Baseline surveillance | B.1.1.529 | GRA |
| hCoV-19/Tunisia/7030/2022 | EPI_ISL_15022539 | 01/02/2022 | Africa / Tunisia / Tunis | Human | Baseline surveillance | Female | 28 | unknown | unknown | Baseline surveillance | AY.122 | GK |
| hCoV-19/Tunisia/1244/2022 | EPI_ISL_15022538 | 11/01/2022 | Africa / Tunisia / Tunis | Human | Baseline surveillance | Male | 29 | unknown | unknown | Baseline surveillance | BA.1.1 | GRA |
| hCoV-19/Tunisia/1833/2022 | EPI_ISL_15022537 | 13/01/2022 | Africa / Tunisia / Tunis | Human | Baseline surveillance | Female | 33 | unknown | unknown | Baseline surveillance | AY.122 | GK |
| hCoV-19/Tunisia/4550/2022 | EPI_ISL_15022536 | 20/01/2022 | Africa / Tunisia / Tunis | Human | Baseline surveillance | Male | 60 | unknown | unknown | Baseline surveillance | AY.122 | GK |
| hCoV-19/Tunisia/49637/2021 | EPI_ISL_15022535 | 21/07/2021 | Africa / Tunisia / Tunis | Human | Baseline surveillance | Male | 47 | unknown | unknown | Baseline surveillance | AY.122 | GK |
| hCoV-19/Tunisia/1247/2022 | EPI_ISL_15022534 | 11/01/2022 | Africa / Tunisia / Tunis | Human | Baseline surveillance | Female | 37 | unknown | unknown | Baseline surveillance | AY.122 | GK |
| hCoV-19/Tunisia/48857/2021 | EPI_ISL_15022533 | 16/07/2021 | Africa / Tunisia / Tunis | Human | Baseline surveillance | Male | 37 | unknown | unknown | Baseline surveillance | AY.122 | GK |
| hCoV-19/Tunisia/6072/2022 | EPI_ISL_15022532 | 26/01/2022 | Africa / Tunisia / Tunis | Human | Baseline surveillance | Female | 29 | unknown | unknown | Baseline surveillance | BA.1.1 | GRA |
| hCoV-19/Tunisia/1051/2022 | EPI_ISL_15022531 | 10/01/2022 | Africa / Tunisia / Tunis | Human | Baseline surveillance | Male | 61 | unknown | unknown | Baseline surveillance | BA.1.1 | GRA |
| hCoV-19/Tunisia/8540/2022 | EPI_ISL_15022530 | 11/02/2022 | Africa / Tunisia / Tunis | Human | Baseline surveillance | Male | 57 | unknown | unknown | Baseline surveillance | BA.1.1 | GRA |
| hCoV-19/Tunisia/473/2022 | EPI_ISL_15022529 | 06/01/2022 | Africa / Tunisia / Tunis | Human | Baseline surveillance | Female | 37 | unknown | unknown | Baseline surveillance | BA.1.1 | GRA |
| hCoV-19/Tunisia/320/2022 | EPI_ISL_15022528 | 06/01/2022 | Africa / Tunisia / Tunis | Human | Baseline surveillance | Female | 18 | unknown | unknown | Baseline surveillance | BA.1 | GRA |
| hCoV-19/Tunisia/326/2022 | EPI_ISL_15022527 | 06/01/2022 | Africa / Tunisia / Tunis | Human | Baseline surveillance | Female | 27 | unknown | unknown | Baseline surveillance | B.1.1.529 | GRA |
| hCoV-19/Tunisia/3617/2022 | EPI_ISL_15022526 | 18/01/2022 | Africa / Tunisia / Tunis | Human | Baseline surveillance | Male | 59 | unknown | unknown | Baseline surveillance | B.1.617.2 | GK |
| hCoV-19/Tunisia/4163/2022 | EPI_ISL_15022525 | 14/04/2022 | Africa / Tunisia / Manouba | Human | Baseline surveillance | Female | 60 | unknown | unknown | Baseline surveillance | BA.2 | GRA |
| hCoV-19/Tunisia/2163/2022 | EPI_ISL_15022524 | 14/04/2022 | Africa / Tunisia / Ben Arous | Human | Baseline surveillance | Female | 58 | unknown | unknown | Baseline surveillance | BA.2 | GRA |
| hCoV-19/Tunisia/183/2022 | EPI_ISL_15022523 | 14/04/2022 | Africa / Tunisia / Ben Arous | Human | Baseline surveillance | Female | 51 | unknown | unknown | Baseline surveillance | BA.2 | GRA |
| hCoV-19/Tunisia/13163/2022 | EPI_ISL_15022522 | 26/04/2022 | Africa / Tunisia / Silina | Human | Baseline surveillance | Male | 74 | unknown | unknown | Baseline surveillance | BA.2 | GRA |
| hCoV-19/Tunisia/13153/2022 | EPI_ISL_15022521 | 26/04/2022 | Africa / Tunisia / Jendouba | Human | Baseline surveillance | Female | 25 | unknown | unknown | Baseline surveillance | BA.2 | GRA |
| hCoV-19/Tunisia/12183/2022 | EPI_ISL_15022520 | 26/04/2022 | Africa / Tunisia / Tunis | Human | Baseline surveillance | Female | 37 | unknown | unknown | Baseline surveillance | BA.2 | GRA |
| hCoV-19/Tunisia/9163/2022 | EPI_ISL_15022519 | 26/04/2022 | Africa / Tunisia / Jendouba | Human | Baseline surveillance | Female | 61 | unknown | unknown | Baseline surveillance | BA.1.1 | GRA |
| hCoV-19/Tunisia/8173/2022 | EPI_ISL_15022518 | 26/04/2022 | Africa / Tunisia / Silina | Human | Baseline surveillance | Female | 48 | unknown | unknown | Baseline surveillance | BA.2 | GRA |
| hCoV-19/Tunisia/7313/2022 | EPI_ISL_15022517 | 26/04/2022 | Africa / Tunisia / Jendouba | Human | Baseline surveillance | Female | 45 | unknown | unknown | Baseline surveillance | BA.2 | GRA |
| hCoV-19/Tunisia/4273/2022 | EPI_ISL_15022516 | 26/04/2022 | Africa / Tunisia / Tunis | Human | Baseline surveillance | Male | 40 | unknown | unknown | Baseline surveillance | BA.2 | GRA |
| hCoV-19/Tunisia/614/2022 | EPI_ISL_15022515 | 26/04/2022 | Africa / Tunisia / Manouba | Human | Baseline surveillance | Female | 72 | unknown | unknown | Baseline surveillance | BA.2 | GRA |
| hCoV-19/Tunisia/55765/2022 | EPI_ISL_15022514 | 14/04/2022 | Africa / Tunisia / Tunis | Human | Baseline surveillance | Male | 29 | unknown | unknown | Baseline surveillance | BA.2 | GRA |
| hCoV-19/Tunisia/49838/2022 | EPI_ISL_15022513 | 14/04/2022 | Africa / Tunisia / Tunis | Human | Baseline surveillance | Male | 64 | unknown | unknown | Baseline surveillance | BA.1.1 | GRA |
| hCoV-19/Tunisia/49052/2022 | EPI_ISL_15022512 | 14/04/2022 | Africa / Tunisia / Tunis | Human | Baseline surveillance | Male | 49 | unknown | unknown | Baseline surveillance | BA.1.1 | GRA |
| hCoV-19/Tunisia/49050/2022 | EPI_ISL_15022511 | 14/04/2022 | Africa / Tunisia / Tunis | Human | Baseline surveillance | Female | 31 | unknown | unknown | Baseline surveillance | BA.1.1 | GRA |
| hCoV-19/Tunisia/48833/2022 | EPI_ISL_15022510 | 14/04/2022 | Africa / Tunisia / Tunis | Human | Baseline surveillance | Female | 56 | unknown | unknown | Baseline surveillance | BA.1.1 | GRA |
| hCoV-19/Tunisia/S942/2022 | EPI_ISL_15022237 | 01/08/2022 | Africa / Tunisia / Sfax | Human | Non-sentinel-surveillance (hospital) | Female | 67 | Hospitalized | unknown | Non-sentinel-surveillance (hospital) | BA.5.2.20 | GRA |
| hCoV-19/Tunisia/S939/2022 | EPI_ISL_15022236 | 29/07/2022 | Africa / Tunisia / Sfax | Human | Baseline surveillance | Male | 67 | unknown | unknown | Baseline surveillance | BA.5.2 | GRA |
| hCoV-19/Tunisia/S938/2022 | EPI_ISL_15022235 | 29/07/2022 | Africa / Tunisia / Sfax | Human | Non-sentinel-surveillance (hospital) | Female | 1 | Hospitalized | unknown | Non-sentinel-surveillance (hospital) | BA.5.2 | GRA |
| hCoV-19/Tunisia/S936/2022 | EPI_ISL_15022234 | 29/07/2022 | Africa / Tunisia / Sfax | Human | Non-sentinel-surveillance (hospital) | Female | 76 | Hospitalized | unknown | Non-sentinel-surveillance (hospital) | BA.5.2 | GRA |
| hCoV-19/Tunisia/S932/2022 | EPI_ISL_15022233 | 27/07/2022 | Africa / Tunisia / Sfax | Human | Baseline surveillance | Male | 76 | unknown | unknown | Baseline surveillance | BA.5.2 | GRA |
| hCoV-19/Tunisia/S931/2022 | EPI_ISL_15022232 | 26/07/2022 | Africa / Tunisia / Gabes | Human | Non-sentinel-surveillance (hospital) | Female | 2 | Hospitalized | unknown | Non-sentinel-surveillance (hospital) | BA.4 | GRA |
| hCoV-19/Tunisia/S930/2022 | EPI_ISL_15022231 | 23/07/2022 | Africa / Tunisia / Sfax | Human | Baseline surveillance | Male | 1 | unknown | unknown | Baseline surveillance | BA.5.2 | GRA |
| hCoV-19/Tunisia/S929/2022 | EPI_ISL_15022230 | 21/07/2022 | Africa / Tunisia / Sfax | Human | Baseline surveillance | Male | 122 | unknown | unknown | Baseline surveillance | BA.2 | GRA |
| hCoV-19/Tunisia/S927/2022 | EPI_ISL_15022229 | 21/07/2022 | Africa / Tunisia / Sfax | Human | Baseline surveillance | Female | 25 | unknown | unknown | Baseline surveillance | BA.5.2.20 | GRA |
| hCoV-19/Tunisia/S924/2022 | EPI_ISL_15022228 | 19/07/2022 | Africa / Tunisia / Sfax | Human | Baseline surveillance | Male | 6 | unknown | unknown | Baseline surveillance | BA.5.2 | GRA |
| hCoV-19/Tunisia/S918/2022 | EPI_ISL_15022227 | 16/07/2022 | Africa / Tunisia / Sfax | Human | Non-sentinel-surveillance (hospital) | Female | 16 | unknown | unknown | Non-sentinel-surveillance (hospital) | BA.4 | GRA |
| hCoV-19/Tunisia/S917/2022 | EPI_ISL_15022226 | 16/07/2022 | Africa / Tunisia / Gabes | Human | Non-sentinel-surveillance (hospital) | Female | 7 | Hospitalized | unknown | Non-sentinel-surveillance (hospital) | BA.5.2.20 | GRA |
| hCoV-19/Tunisia/S916/2022 | EPI_ISL_15022225 | 14/07/2022 | Africa / Tunisia / Sfax | Human | Baseline surveillance | Female | 28 | unknown | unknown | Baseline surveillance | BA.5.2 | GRA |
| hCoV-19/Tunisia/S914/2022 | EPI_ISL_15022224 | 16/07/2022 | Africa / Tunisia / Sfax | Human | Non-sentinel-surveillance (hospital) | Male | 43 | Hospitalized | unknown | Non-sentinel-surveillance (hospital) | BA.5.2 | GRA |
| hCoV-19/Tunisia/S912/2022 | EPI_ISL_15022223 | 16/07/2022 | Africa / Tunisia / Sfax | Human | Baseline surveillance | Male | 61 | unknown | unknown | Baseline surveillance | BA.5.2 | GRA |
| hCoV-19/Tunisia/S911/2022 | EPI_ISL_15022222 | 16/07/2022 | Africa / Tunisia / Sfax | Human | Baseline surveillance | Male | 66 | unknown | unknown | Baseline surveillance | BA.5.2 | GRA |
| hCoV-19/Tunisia/S906/2022 | EPI_ISL_15022221 | 14/07/2022 | Africa / Tunisia / Sfax | Human | Baseline surveillance | Female | 70 | unknown | unknown | Baseline surveillance | BA.5.2 | GRA |
| hCoV-19/Tunisia/S904/2022 | EPI_ISL_15022220 | 12/07/2022 | Africa / Tunisia / Sfax | Human | Non-sentinel-surveillance (hospital) | Female | 92 | Hospitalized | unknown | Non-sentinel-surveillance (hospital) | BA.5.2 | GRA |
| hCoV-19/Tunisia/S903/2022 | EPI_ISL_15022219 | 12/07/2022 | Africa / Tunisia / Sfax | Human | Non-sentinel-surveillance (hospital) | Male | 37 | Hospitalized | unknown | Non-sentinel-surveillance (hospital) | BA.5.2 | GRA |
| hCoV-19/Tunisia/S902/2022 | EPI_ISL_15022218 | 13/07/2022 | Africa / Tunisia / Sfax | Human | Non-sentinel-surveillance (hospital) | Male | 27 | Hospitalized | unknown | Non-sentinel-surveillance (hospital) | BA.5.2 | GRA |
| hCoV-19/Tunisia/S900/2022 | EPI_ISL_15022217 | 13/07/2022 | Africa / Tunisia / Sfax | Human | Non-sentinel-surveillance (hospital) | Male | 1 | Hospitalized | unknown | Non-sentinel-surveillance (hospital) | BA.5.2 | GRA |
| hCoV-19/Tunisia/S889/2022 | EPI_ISL_15022216 | 12/07/2022 | Africa / Tunisia / Sfax | Human | Baseline surveillance | Female | 29 | unknown | unknown | Baseline surveillance | BA.4 | GRA |
| hCoV-19/Tunisia/S888/2022 | EPI_ISL_15022215 | 11/07/2022 | Africa / Tunisia / Sfax | Human | Non-sentinel-surveillance (hospital) | Male | 1 | Hospitalized | unknown | Non-sentinel-surveillance (hospital) | BA.5.2 | GRA |
| hCoV-19/Tunisia/S887/2022 | EPI_ISL_15022214 | 11/07/2022 | Africa / Tunisia / Sidi Bouzid | Human | Non-sentinel-surveillance (hospital) | Male | 10 | Hospitalized | unknown | Non-sentinel-surveillance (hospital) | BA.4 | GRA |
| hCoV-19/Tunisia/S878/2022 | EPI_ISL_15022213 | 05/07/2022 | Africa / Tunisia / Sfax | Human | Baseline surveillance | Female | 38 | unknown | unknown | Baseline surveillance | BA.2 | GRA |
| hCoV-19/Tunisia/S874/2022 | EPI_ISL_15022212 | 05/07/2022 | Africa / Tunisia / Sfax | Human | Baseline surveillance | Female | 42 | unknown | unknown | Baseline surveillance | BA.5.2 | GRA |
| hCoV-19/Tunisia/S870/2022 | EPI_ISL_15022211 | 04/07/2022 | Africa / Tunisia / Sfax | Human | Baseline surveillance | Female | 48 | unknown | unknown | Baseline surveillance | BA.4 | GRA |
| hCoV-19/Tunisia/S868/2022 | EPI_ISL_15022210 | 02/07/2022 | Africa / Tunisia / Sfax | Human | Baseline surveillance | Male | 74 | unknown | unknown | Baseline surveillance | BA.5.2.20 | GRA |
| hCoV-19/Tunisia/S867/2022 | EPI_ISL_15022209 | 02/07/2022 | Africa / Tunisia / Sfax | Human | Baseline surveillance | Female | 72 | unknown | unknown | Baseline surveillance | BA.5.2.20 | GRA |
| hCoV-19/Tunisia/S865/2022 | EPI_ISL_15022208 | 30/06/2022 | Africa / Tunisia / Sfax | Human | Baseline surveillance | Male | 47 | unknown | unknown | Baseline surveillance | BA.5.2 | GRA |
| hCoV-19/Tunisia/S864/2022 | EPI_ISL_15022207 | 30/06/2022 | Africa / Tunisia / Sfax | Human | Baseline surveillance | Male | 37 | unknown | unknown | Baseline surveillance | BA.5.2 | GRA |
| hCoV-19/Tunisia/S860/2022 | EPI_ISL_15022206 | 29/06/2022 | Africa / Tunisia / Sfax | Human | Baseline surveillance | Male | 51 | unknown | unknown | Baseline surveillance | BA.5.2 | GRA |
| hCoV-19/Tunisia/S859/2022 | EPI_ISL_15022205 | 29/06/2022 | Africa / Tunisia / Sfax | Human | Non-sentinel-surveillance (hospital) | Male | 1 | Hospitalized | unknown | Non-sentinel-surveillance (hospital) | BA.5.2.1 | GRA |
| hCoV-19/Tunisia/S858/2022 | EPI_ISL_15022204 | 29/06/2022 | Africa / Tunisia / Sfax | Human | Baseline surveillance | Male | 88 | unknown | unknown | Baseline surveillance | BA.5.2 | GRA |
| hCoV-19/Tunisia/S857/2022 | EPI_ISL_15022203 | 29/06/2022 | Africa / Tunisia / Sfax | Human | Baseline surveillance | Female | 68 | unknown | unknown | Baseline surveillance | BA.5.2 | GRA |
| hCoV-19/Tunisia/S856/2022 | EPI_ISL_15022202 | 29/06/2022 | Africa / Tunisia / Sfax | Human | Baseline surveillance | Female | 19 | unknown | unknown | Baseline surveillance | BA.5.2 | GRA |
| hCoV-19/Tunisia/S855/2022 | EPI_ISL_15022201 | 29/06/2022 | Africa / Tunisia / Sfax | Human | Baseline surveillance | Female | 13 | unknown | unknown | Baseline surveillance | BA.5.2 | GRA |
| hCoV-19/Tunisia/S854/2022 | EPI_ISL_15022200 | 29/06/2022 | Africa / Tunisia / Sfax | Human | Baseline surveillance | Female | 39 | unknown | unknown | Baseline surveillance | BA.5.2 | GRA |
| hCoV-19/Tunisia/S848/2022 | EPI_ISL_15022199 | 25/06/2022 | Africa / Tunisia / Sfax | Human | Baseline surveillance | Female | 44 | unknown | unknown | Baseline surveillance | BA.5.2 | GRA |
| hCoV-19/Tunisia/S847/2022 | EPI_ISL_15022198 | 25/06/2022 | Africa / Tunisia / Sfax | Human | Baseline surveillance | Female | 20 | unknown | unknown | Baseline surveillance | BA.5.2 | GRA |
| hCoV-19/Tunisia/S846/2022 | EPI_ISL_15022197 | 25/06/2022 | Africa / Tunisia / Sfax | Human | Baseline surveillance | Female | 43 | unknown | unknown | Baseline surveillance | BA.5.2 | GRA |
| hCoV-19/Tunisia/S843/2022 | EPI_ISL_15022196 | 26/06/2022 | Africa / Tunisia / Sfax | Human | Baseline surveillance | Male | 36 | unknown | unknown | Baseline surveillance | BA.2 | GRA |
| hCoV-19/Tunisia/S840/2022 | EPI_ISL_15022195 | 25/06/2022 | Africa / Tunisia / Sfax | Human | Baseline surveillance | Male | 50 | unknown | unknown | Baseline surveillance | BA.5.2 | GRA |
| hCoV-19/Tunisia/S833/2022 | EPI_ISL_15022194 | 23/06/2022 | Africa / Tunisia / Sfax | Human | Baseline surveillance | Male | 32 | unknown | unknown | Baseline surveillance | BA.5.2 | GRA |
| hCoV-19/Tunisia/S830/2022 | EPI_ISL_15022193 | 22/06/2022 | Africa / Tunisia / Sfax | Human | Baseline surveillance | Female | 26 | unknown | unknown | Baseline surveillance | BA.5.2 | GRA |
| hCoV-19/Tunisia/S885/2022 | EPI_ISL_15022192 | 11/07/2022 | Africa / Tunisia / Sfax | Human | Baseline surveillance | Female | 67 | unknown | unknown | Baseline surveillance | B.1.1.529 | GRA |
| hCoV-19/Tunisia/ADAGE-18505/2020 | EPI_ISL_712060 | 19/10/2020 | Africa / Tunisia / Monastir | Human | unknown | Male | 28 | Released | unknown | unknown | B.1.1.1 | GR |
| hCoV-19/Tunisia/SP-0017/2021 | EPI_ISL_2035560 | 23/01/2021 | Africa / Tunisia / Tunis | Human | unknown | Male | 27 | unknown | unknown | unknown | B.1.160 | GH |
| hCoV-19/Tunisia/24463/2020 | EPI_ISL_1118926 | 07/11/2020 | Africa / Tunisia / Sfax | Human | unknown | Female | 46 | Severe | unknown | unknown | B.1.160 | GH |
| hCoV-19/Tunisia/ADAGE-24776/2020 | EPI_ISL_712065 | 08/11/2020 | Africa / Tunisia / Sfax | Human | unknown | unknown | unknown | unknown | unknown | unknown | B.1.160 | GH |
| hCoV-19/Tunisia/Siliana/Q-9993/2021 | EPI_ISL_12316670 | 01/03/2021 | Africa / Tunisia / Silina | Human | Random | Male | 33 | unknown | Not Vaccinated | Random | A.27 | S |
| hCoV-19/Tunisia/Tunis/Q-9581/2021 | EPI_ISL_12316669 | 25/02/2021 | Africa / Tunisia / Tunis | Human | Random | Male | unknown | unknown | Not Vaccinated | Random | A | G |
| hCoV-19/Tunisia/Tunis/Q-9082/2021 | EPI_ISL_12316668 | 23/02/2021 | Africa / Tunisia / Tunis | Human | Random | Male | 85 | unknown | Not Vaccinated | Random | A.27 | S |
| hCoV-19/Tunisia/Tunis/Q-9081/2021 | EPI_ISL_12316667 | 23/02/2021 | Africa / Tunisia / Tunis | Human | Random | Female | 31 | unknown | Not Vaccinated | Random | A.27 | S |
| hCoV-19/Tunisia/Tunis/Q-9052/2021 | EPI_ISL_12316666 | 23/02/2021 | Africa / Tunisia / Tunis | Human | Random | Male | 78 | unknown | Not Vaccinated | Random | A.27 | S |
| hCoV-19/Tunisia/Tunis/Q-5516/2021 | EPI_ISL_12316665 | 02/02/2021 | Africa / Tunisia / Tunis | Human | Random | Female | 34 | unknown | Not Vaccinated | Random | A.27 | S |
| hCoV-19/Tunisia/S-0042/2021 | EPI_ISL_12280586 | 17/03/2021 | Africa / Tunisia / Mannouba | Human | unknown | Female | 68 | unknown | unknown | unknown | A.27 | S |
| hCoV-19/Tunisia/11800/2022 | EPI_ISL_11986495 | 16/03/2022 | Africa / Tunisia / Ariana | Human | Baseline surveillance | Male | 55 | unknown | unknown | Baseline surveillance | BA.2.3 | GRA |
| hCoV-19/Tunisia/03/2022 | EPI_ISL_11986494 | 28/02/2022 | Africa / Tunisia / Ariana | Human | Baseline surveillance | Male | 71 | unknown | unknown | Baseline surveillance | BA.1.1 | GRA |
| hCoV-19/Tunisia/41756/2021 | EPI_ISL_11986493 | 12/08/2021 | Africa / Tunisia / Ariana | Human | Baseline surveillance | Male | 21 | unknown | unknown | Baseline surveillance | AY.122 | GK |
| hCoV-19/Tunisia/50051/2022 | EPI_ISL_11986492 | 02/01/2022 | Africa / Tunisia / Ariana | Human | Baseline surveillance | Male | 37 | unknown | unknown | Baseline surveillance | BA.1.1 | GRA |
| hCoV-19/Tunisia/47161/2022 | EPI_ISL_11986491 | 12/01/2022 | Africa / Tunisia / Ariana | Human | Baseline surveillance | Male | 37 | unknown | unknown | Baseline surveillance | BA.1.1 | GRA |
| hCoV-19/Tunisia/22/2022 | EPI_ISL_11986490 | 12/03/2022 | Africa / Tunisia / Beja | Human | Baseline surveillance | Male | 62 | unknown | unknown | Baseline surveillance | BA.2 | GRA |
| hCoV-19/Tunisia/1518/2022 | EPI_ISL_11986489 | 19/02/2022 | Africa / Tunisia / Beja | Human | Baseline surveillance | Female | 70 | unknown | unknown | Baseline surveillance | BA.2 | GRA |
| hCoV-19/Tunisia/332022/2022 | EPI_ISL_11986488 | 16/03/2022 | Africa / Tunisia / Ben Arous | Human | Baseline surveillance | Male | 27 | unknown | unknown | Baseline surveillance | BA.2 | GRA |
| hCoV-19/Tunisia/35/2022 | EPI_ISL_11986487 | 02/03/2022 | Africa / Tunisia / Beni Khalled | Human | Baseline surveillance | Male | 9 | unknown | unknown | Baseline surveillance | BA.2 | GRA |
| hCoV-19/Tunisia/8312/2022 | EPI_ISL_11986486 | 10/02/2022 | Africa / Tunisia / Beni Khalled | Human | Baseline surveillance | Male | 40 | unknown | unknown | Baseline surveillance | BA.2 | GRA |
| hCoV-19/Tunisia/10190/2022 | EPI_ISL_11986485 | 28/02/2022 | Africa / Tunisia / Grombalia | Human | Baseline surveillance | Female | 49 | unknown | unknown | Baseline surveillance | BA.1.1 | GRA |
| hCoV-19/Tunisia/01/2022 | EPI_ISL_11986484 | 08/03/2022 | Africa / Tunisia / Grombalia | Human | Baseline surveillance | Female | 34 | unknown | unknown | Baseline surveillance | BA.2 | GRA |
| hCoV-19/Tunisia/8191/2022 | EPI_ISL_11986483 | 09/02/2022 | Africa / Tunisia / Grombalia | Human | Baseline surveillance | Male | 62 | unknown | unknown | Baseline surveillance | BA.2 | GRA |
| hCoV-19/Tunisia/10746/2022 | EPI_ISL_11986482 | 05/03/2022 | Africa / Tunisia / Haouaria | Human | Baseline surveillance | Female | 35 | unknown | unknown | Baseline surveillance | BA.2 | GRA |
| hCoV-19/Tunisia/30/2022 | EPI_ISL_11986481 | 10/03/2022 | Africa / Tunisia / Haouaria | Human | Baseline surveillance | Male | 59 | unknown | unknown | Baseline surveillance | BA.2 | GRA |
| hCoV-19/Tunisia/53957/2022 | EPI_ISL_11986480 | 16/02/2022 | Africa / Tunisia / Haouaria | Human | Baseline surveillance | Female | 13 | unknown | unknown | Baseline surveillance | BA.1.17.2 | GRA |
| hCoV-19/Tunisia/8494/2022 | EPI_ISL_11986479 | 11/02/2022 | Africa / Tunisia / Haouaria | Human | Baseline surveillance | Male | 10 | unknown | unknown | Baseline surveillance | BA.2 | GRA |
| hCoV-19/Tunisia/9611/2022 | EPI_ISL_11986478 | 21/02/2022 | Africa / Tunisia / Korba | Human | Baseline surveillance | Female | 24 | unknown | unknown | Baseline surveillance | BA.2 | GRA |
| hCoV-19/Tunisia/732022/2022 | EPI_ISL_11986477 | 15/03/2022 | Africa / Tunisia / Manouba | Human | Baseline surveillance | Female | 66 | unknown | unknown | Baseline surveillance | BA.2 | GRA |
| hCoV-19/Tunisia/11277/2022 | EPI_ISL_11986476 | 09/03/2022 | Africa / Tunisia / Manouba | Human | Baseline surveillance | Female | 33 | unknown | unknown | Baseline surveillance | BA.2 | GRA |
| hCoV-19/Tunisia/52540/2022 | EPI_ISL_11986475 | 01/02/2022 | Africa / Tunisia / Manouba | Human | Baseline surveillance | Female | 58 | unknown | unknown | Baseline surveillance | BA.1.1 | GRA |
| hCoV-19/Tunisia/12/2022 | EPI_ISL_11986474 | 04/03/2022 | Africa / Tunisia / Manouba | Human | Baseline surveillance | Female | 69 | unknown | unknown | Baseline surveillance | BA.2 | GRA |
| hCoV-19/Tunisia/59966/2021 | EPI_ISL_11986473 | 20/10/2021 | Africa / Tunisia / Manouba | Human | Baseline surveillance | Male | 32 | unknown | unknown | Baseline surveillance | AY.127.1 | GK |
| hCoV-19/Tunisia/53456/2021 | EPI_ISL_11986472 | 18/08/2021 | Africa / Tunisia / Manouba | Human | Baseline surveillance | Male | 45 | unknown | unknown | Baseline surveillance | AY.122 | GK |
| hCoV-19/Tunisia/11275/2022 | EPI_ISL_11986471 | 07/03/2022 | Africa / Tunisia / Menzel Bouzelfa | Human | Baseline surveillance | Male | 16 | unknown | unknown | Baseline surveillance | BA.2 | GRA |
| hCoV-19/Tunisia/8700/2022 | EPI_ISL_11986470 | 12/02/2022 | Africa / Tunisia / Menzel Bouzelfa | Human | Baseline surveillance | Male | 80 | unknown | unknown | Baseline surveillance | BA.1.1.1 | GRA |
| hCoV-19/Tunisia/11280/2022 | EPI_ISL_11986469 | 01/03/2022 | Africa / Tunisia / Menzel Temime | Human | Baseline surveillance | Female | 42 | unknown | unknown | Baseline surveillance | BA.2 | GRA |
| hCoV-19/Tunisia/8861/2022 | EPI_ISL_11986468 | 14/02/2022 | Africa / Tunisia / Menzel Temime | Human | Baseline surveillance | Male | 71 | unknown | unknown | Baseline surveillance | BA.2 | GRA |
| hCoV-19/Tunisia/49918/2022 | EPI_ISL_11986467 | 31/01/2022 | Africa / Tunisia / Monastir | Human | Baseline surveillance | Male | 35 | unknown | unknown | Baseline surveillance | BA.1.1 | GRA |
| hCoV-19/Tunisia/48328/2022 | EPI_ISL_11986466 | 11/01/2022 | Africa / Tunisia / Nabeul | Human | Baseline surveillance | Male | 38 | unknown | unknown | Baseline surveillance | BA.1.1 | GRA |
| hCoV-19/Tunisia/40/2022 | EPI_ISL_11986465 | 24/02/2022 | Africa / Tunisia / Nabeul | Human | Baseline surveillance | Male | unknown | unknown | unknown | Baseline surveillance | BA.1.1 | GRA |
| hCoV-19/Tunisia/8033/2022 | EPI_ISL_11986464 | 07/02/2022 | Africa / Tunisia / Nabeul | Human | Baseline surveillance | Female | unknown | unknown | unknown | Baseline surveillance | BA.2 | GRA |
| hCoV-19/Tunisia/54611/2022 | EPI_ISL_11986463 | 16/02/2022 | Africa / Tunisia / Sidi Hssine | Human | Baseline surveillance | Female | 4 | unknown | unknown | Baseline surveillance | BA.1.1 | GRA |
| hCoV-19/Tunisia/54688/2022 | EPI_ISL_11986462 | 18/02/2022 | Africa / Tunisia / Tunis | Human | Baseline surveillance | Female | 61 | unknown | unknown | Baseline surveillance | BA.1.17.2 | GRA |
| hCoV-19/Tunisia/53954/2022 | EPI_ISL_11986461 | 09/02/2022 | Africa / Tunisia / Tunis | Human | Baseline surveillance | Female | 4 | unknown | unknown | Baseline surveillance | BA.1.1 | GRA |
| hCoV-19/Tunisia/52817/2022 | EPI_ISL_11986460 | 02/02/2022 | Africa / Tunisia / Tunis | Human | Baseline surveillance | Female | 45 | unknown | unknown | Baseline surveillance | BA.1.17.2 | GRA |
| hCoV-19/Tunisia/51214/2022 | EPI_ISL_11986459 | 14/02/2022 | Africa / Tunisia / Tunis | Human | Baseline surveillance | Male | 22 | unknown | unknown | Baseline surveillance | BA.1.18 | GRA |
| hCoV-19/Tunisia/49690/2022 | EPI_ISL_11986458 | 28/01/2022 | Africa / Tunisia / Tunis | Human | Baseline surveillance | Male | 7 | unknown | unknown | Baseline surveillance | BA.1.1 | GRA |
| hCoV-19/Tunisia/11799/2022 | EPI_ISL_11986457 | 15/03/2022 | Africa / Tunisia / Tunis | Human | Baseline surveillance | Male | 55 | unknown | unknown | Baseline surveillance | BA.2.3 | GRA |
| hCoV-19/Tunisia/11276/2022 | EPI_ISL_11986456 | 08/03/2022 | Africa / Tunisia / Tunis | Human | Baseline surveillance | Male | 13 | unknown | unknown | Baseline surveillance | BA.2 | GRA |
| hCoV-19/Tunisia/21/2022 | EPI_ISL_11986455 | 14/03/2022 | Africa / Tunisia / Tunis | Human | Baseline surveillance | Male | 69 | unknown | unknown | Baseline surveillance | BA.1 | GRA |
| hCoV-19/Tunisia/1232022/2022 | EPI_ISL_11986454 | 17/03/2022 | Africa / Tunisia / Tunis | Human | Baseline surveillance | Female | 32 | unknown | unknown | Baseline surveillance | BA.1.18 | GRA |
| hCoV-19/Tunisia/41757/2021 | EPI_ISL_11986453 | 12/08/2021 | Africa / Tunisia / Tunis | Human | Baseline surveillance | Female | 21 | unknown | unknown | Baseline surveillance | AY.122 | GK |
| hCoV-19/Tunisia/11279/2022 | EPI_ISL_11986452 | 28/02/2022 | Africa / Tunisia / Tunis | Human | Baseline surveillance | Female | 42 | unknown | unknown | Baseline surveillance | BA.2 | GRA |
| hCoV-19/Tunisia/11278/2022 | EPI_ISL_11986451 | 08/03/2022 | Africa / Tunisia / Tunis | Human | Baseline surveillance | Female | 32 | unknown | unknown | Baseline surveillance | BA.2 | GRA |
| hCoV-19/Tunisia/51057/2022 | EPI_ISL_11986450 | 22/01/2022 | Africa / Tunisia / Tunis | Human | Baseline surveillance | Female | 29 | unknown | unknown | Baseline surveillance | BA.1.1 | GRA |
| hCoV-19/Tunisia/8182/2022 | EPI_ISL_11986449 | 09/02/2022 | Africa / Tunisia / Tunis | Human | Baseline surveillance | Male | 76 | unknown | unknown | Baseline surveillance | BA.1.1 | GRA |
| hCoV-19/Tunisia/S-0901/2021 | EPI_ISL_11881928 | 15/06/2021 | Africa / Tunisia / Jendouba | Human | Random | Female | 38 | Moderate | unknown | Random | AY.122 | GK |
| hCoV-19/Tunisia/S-0899/2021 | EPI_ISL_11881927 | 05/07/2021 | Africa / Tunisia / Nabeul | Human | Random | Female | 3 months | unknown | unknown | Random | AY.122 | GK |
| hCoV-19/Tunisia/S-0879/2021 | EPI_ISL_11881926 | 09/07/2021 | Africa / Tunisia / Gafsa | Human | Random | Female | unknown | unknown | unknown | Random | AY.122 | GK |
| hCoV-19/Tunisia/S-0875/2021 | EPI_ISL_11881925 | 09/07/2021 | Africa / Tunisia / Gafsa | Human | Random | Female | unknown | unknown | unknown | Random | AY.122 | GK |
| hCoV-19/Tunisia/S-0871/2021 | EPI_ISL_11881924 | 14/06/2021 | Africa / Tunisia / Monastir | Human | Random | Female | 63 | unknown | unknown | Random | AY.122 | GK |
| hCoV-19/Tunisia/S-0862/2021 | EPI_ISL_11881923 | 28/06/2021 | Africa / Tunisia / Nabeul | Human | Random | Male | 45 | Moderate | unknown | Random | AY.122 | GK |
| hCoV-19/Tunisia/S-0853/2021 | EPI_ISL_11881922 | 30/06/2021 | Africa / Tunisia / Sousse | Human | Random | Male | 20 | Moderate | unknown | Random | AY.122 | GK |
| hCoV-19/Tunisia/S-0826/2021 | EPI_ISL_11881921 | 30/06/2021 | Africa / Tunisia / Silina | Human | Random | Female | 27 | Severe | unknown | Random | AY.122 | GK |
| hCoV-19/Tunisia/S-0821/2021 | EPI_ISL_11881920 | 01/07/2021 | Africa / Tunisia / Mannouba | Human | Random | Female | unknown | unknown | unknown | Random | AY.122 | GK |
| hCoV-19/Tunisia/S-0820/2021 | EPI_ISL_11881919 | 01/07/2021 | Africa / Tunisia / Mannouba | Human | Random | Male | unknown | unknown | unknown | Random | AY.122 | GK |
| hCoV-19/Tunisia/S-0819/2021 | EPI_ISL_11881918 | 01/07/2021 | Africa / Tunisia / Mannouba | Human | Random | Male | unknown | unknown | unknown | Random | AY.122 | GK |
| hCoV-19/Tunisia/S-0818/2021 | EPI_ISL_11881917 | 01/07/2021 | Africa / Tunisia / Mannouba | Human | Random | Female | unknown | unknown | unknown | Random | AY.122 | GK |
| hCoV-19/Tunisia/S-0816/2021 | EPI_ISL_11881916 | 01/07/2021 | Africa / Tunisia / Mannouba | Human | Random | Female | unknown | Moderate | unknown | Random | AY.122 | GK |
| hCoV-19/Tunisia/S-0812/2021 | EPI_ISL_11881915 | 23/06/2021 | Africa / Tunisia / Nabeul | Human | Random | Male | 72 | Moderate | unknown | Random | AY.122 | GK |
| hCoV-19/Tunisia/S-0806/2021 | EPI_ISL_11881914 | 29/06/2021 | Africa / Tunisia / Kairouan | Human | Random | Male | 2 months | Moderate | unknown | Random | AY.122 | GK |
| hCoV-19/Tunisia/S-0800/2021 | EPI_ISL_11881913 | 08/06/2021 | Africa / Tunisia / Zaghouen | Human | Random | Female | 56 | Severe | unknown | Random | AY.122 | GK |
| hCoV-19/Tunisia/S-0799/2021 | EPI_ISL_11881912 | 19/06/2021 | Africa / Tunisia / Zaghouen | Human | Random | Male | 14 | Moderate | unknown | Random | AY.122 | GK |
| hCoV-19/Tunisia/S-0797/2021 | EPI_ISL_11881911 | 06/06/2021 | Africa / Tunisia / Zaghouen | Human | Random | Male | 52 | Severe | unknown | Random | AY.122 | GK |
| hCoV-19/Tunisia/S-0748/2021 | EPI_ISL_11881910 | 12/06/2021 | Africa / Tunisia / Medenine | Human | Random | Male | 37 | unknown | unknown | Random | AY.122 | GK |
| hCoV-19/Tunisia/S-0740/2021 | EPI_ISL_11881909 | 07/06/2021 | Africa / Tunisia / Nabeul | Human | Random | Female | 34 | unknown | unknown | Random | AY.122 | GK |
| hCoV-19/Tunisia/S-0733/2021 | EPI_ISL_11881908 | 27/05/2021 | Africa / Tunisia / Nabeul | Human | Random | Male | 29 | unknown | unknown | Random | AY.122 | GK |
| hCoV-19/Tunisia/S-0715/2021 | EPI_ISL_11881907 | 26/06/2021 | Africa / Tunisia / Kairouan | Human | Random | Male | 2 months | Moderate | unknown | Random | AY.122 | GK |
| hCoV-19/Tunisia/S-0676/2021 | EPI_ISL_11881906 | 27/06/2021 | Africa / Tunisia / Sfax | Human | Random | Male | 43 | unknown | unknown | Random | AY.122 | GK |
| hCoV-19/Tunisia/S-0674/2021 | EPI_ISL_11881905 | 26/06/2021 | Africa / Tunisia / Jendouba | Human | Random | Male | 13 | Asymptomatic | unknown | Random | AY.122 | GK |
| hCoV-19/Tunisia/S-0645/2021 | EPI_ISL_11881904 | 24/06/2021 | Africa / Tunisia / Silina | Human | Random | Male | 75 | Severe | unknown | Random | AY.122 | GK |
| hCoV-19/Tunisia/S-0602/2021 | EPI_ISL_11881903 | 22/06/2021 | Africa / Tunisia / Kairouan | Human | Random | Female | 34 | Severe | unknown | Random | AY.122 | GK |
| hCoV-19/Tunisia/S-0600/2021 | EPI_ISL_11881902 | 17/06/2021 | Africa / Tunisia / Kairouan | Human | Random | Male | 43 | Moderate | unknown | Random | AY.122 | GK |
| hCoV-19/Tunisia/S-0599/2021 | EPI_ISL_11881901 | 22/06/2021 | Africa / Tunisia / Kairouan | Human | Random | Male | unknown | Severe | unknown | Random | AY.122 | GK |
| hCoV-19/Tunisia/S-0598/2021 | EPI_ISL_11881900 | 22/06/2021 | Africa / Tunisia / Kairouan | Human | Random | Male | unknown | Moderate | unknown | Random | AY.122 | GK |
| hCoV-19/Tunisia/S-0597/2021 | EPI_ISL_11881899 | 17/06/2021 | Africa / Tunisia / Kairouan | Human | Random | Male | 38 | Severe | unknown | Random | AY.122 | GK |
| hCoV-19/Tunisia/S-0596/2021 | EPI_ISL_11881898 | 22/06/2021 | Africa / Tunisia / Kairouan | Human | Random | Female | 85 | Moderate | unknown | Random | AY.122 | GK |
| hCoV-19/Tunisia/S-0594/2021 | EPI_ISL_11881897 | 16/06/2021 | Africa / Tunisia / Kairouan | Human | Random | Female | 42 | Severe | unknown | Random | AY.122 | GK |
| hCoV-19/Tunisia/S-0591/2021 | EPI_ISL_11881896 | 17/06/2021 | Africa / Tunisia / Kairouan | Human | Random | Female | 30 | Moderate | unknown | Random | AY.122 | GK |
| hCoV-19/Tunisia/S-0589/2021 | EPI_ISL_11881895 | 15/06/2021 | Africa / Tunisia / Kairouan | Human | Random | Male | 4 | Severe | unknown | Random | AY.122 | GK |
| hCoV-19/Tunisia/S-0587/2021 | EPI_ISL_11881894 | 16/06/2021 | Africa / Tunisia / Kairouan | Human | Random | Female | 34 | Severe | unknown | Random | AY.122 | GK |
| hCoV-19/Tunisia/S-0586/2021 | EPI_ISL_11881893 | 14/06/2021 | Africa / Tunisia / Kairouan | Human | Random | Female | 62 | Severe | unknown | Random | AY.122 | GK |
| hCoV-19/Tunisia/S-0585/2021 | EPI_ISL_11881892 | 18/06/2021 | Africa / Tunisia / Kairouan | Human | Random | Female | 8 | Moderate | unknown | Random | AY.122 | GK |
| hCoV-19/Tunisia/S-0584/2021 | EPI_ISL_11881891 | 21/06/2021 | Africa / Tunisia / Kairouan | Human | Random | Female | 1 month | Moderate | unknown | Random | AY.122 | GK |
| hCoV-19/Tunisia/S-0582/2021 | EPI_ISL_11881890 | 17/06/2021 | Africa / Tunisia / Kairouan | Human | Random | Female | 44 | Severe | unknown | Random | AY.122 | GK |
| hCoV-19/Tunisia/S-0581/2021 | EPI_ISL_11881889 | 22/06/2021 | Africa / Tunisia / Kairouan | Human | Random | Male | 32 | Severe | unknown | Random | AY.122 | GK |
| hCoV-19/Tunisia/S-0580/2021 | EPI_ISL_11881888 | 22/06/2021 | Africa / Tunisia / Kairouan | Human | Random | Female | 55 | Moderate | unknown | Random | AY.122 | GK |
| hCoV-19/Tunisia/S-0579/2021 | EPI_ISL_11881887 | 22/06/2021 | Africa / Tunisia / Kairouan | Human | Random | Female | 41 | Severe | unknown | Random | AY.122 | GK |
| hCoV-19/Tunisia/S-0577/2021 | EPI_ISL_11881886 | 17/06/2021 | Africa / Tunisia / Kairouan | Human | Random | Female | 8 | Moderate | unknown | Random | AY.122 | GK |
| hCoV-19/Tunisia/S-0575/2021 | EPI_ISL_11881885 | 22/06/2021 | Africa / Tunisia / Kairouan | Human | Random | Male | 64 | Severe | unknown | Random | AY.122 | GK |
| hCoV-19/Tunisia/S-0573/2021 | EPI_ISL_11881884 | 23/06/2021 | Africa / Tunisia / Kairouan | Human | Random | Male | 44 | Moderate | unknown | Random | AY.122 | GK |
| hCoV-19/Tunisia/S-0572/2021 | EPI_ISL_11881883 | 16/06/2021 | Africa / Tunisia / Kairouan | Human | Random | Female | 33 | Severe | unknown | Random | AY.122 | GK |
| hCoV-19/Tunisia/S-0571/2021 | EPI_ISL_11881882 | 17/06/2021 | Africa / Tunisia / Kairouan | Human | Random | Male | 8 | Moderate | unknown | Random | AY.122 | GK |
| hCoV-19/Tunisia/S-0570/2021 | EPI_ISL_11881881 | 22/06/2021 | Africa / Tunisia / Kairouan | Human | Random | Female | unknown | Severe | unknown | Random | AY.122 | GK |
| hCoV-19/Tunisia/S-0568/2021 | EPI_ISL_11881880 | 17/06/2021 | Africa / Tunisia / Kairouan | Human | Random | Male | 80 | Moderate | unknown | Random | AY.122 | GK |
| hCoV-19/Tunisia/S-0460/2021 | EPI_ISL_11881879 | 28/05/2021 | Africa / Tunisia / Sfax | Human | Random | Female | 19 | Asymptomatic | unknown | Random | B.1.617.2 | GK |
| hCoV-19/Tunisia/Z3136/2020 | EPI_ISL_11782576 | 21/10/2020 | Africa / Tunisia / Silina | Human | Random | Female | unknown | Death | Not Vaccinated | Random | B.1.160 | GH |
| hCoV-19/Tunisia/Z3046/2020 | EPI_ISL_11782575 | 21/10/2020 | Africa / Tunisia / Tunis | Human | Random | Male | unknown | Asymptomatic | Not Vaccinated | Random | B.1.160 | GH |
| hCoV-19/Tunisia/X7385/2020 | EPI_ISL_11782574 | 13/09/2020 | Africa / Tunisia / Tunis | Human | Random | Female | 44 | Severe | Not Vaccinated | Random | B.1.533 | GH |
| hCoV-19/Tunisia/X5561/2020 | EPI_ISL_11782573 | 08/09/2020 | Africa / Tunisia / Sidi Bouzid | Human | Random | Male | 15 | Mild infection | Not Vaccinated | Random | B.1.1.198 | GH |
| hCoV-19/Tunisia/U3382/2021 | EPI_ISL_11782572 | 16/02/2021 | Africa / Tunisia / Tunis | Human | Random | Male | 32 | Asymptomatic | Not Vaccinated | Random | B.1.160 | GH |
| hCoV-19/Tunisia/S0528/2021 | EPI_ISL_11782571 | 15/06/2021 | Africa / Tunisia / Kairouan | Human | Random | Male | 1 | Mild infection | Not Vaccinated | Random | AY.122 | GK |
| hCoV-19/Tunisia/S0522/2021 | EPI_ISL_11782570 | 12/06/2021 | Africa / Tunisia / Sousse | Human | Random | Male | 35 | Severe | Not Vaccinated | Random | AY.122 | GK |
| hCoV-19/Tunisia/S0503/2021 | EPI_ISL_11782569 | 10/06/2021 | Africa / Tunisia / Kairouan | Human | Random | Female | 75 | Death | Not Vaccinated | Random | B | G |
| hCoV-19/Tunisia/S0500/2021 | EPI_ISL_11782568 | 10/06/2021 | Africa / Tunisia / Kairouan | Human | Random | Female | 92 | Death | Not Vaccinated | Random | B.1 | GK |
| hCoV-19/Tunisia/S0062/2021 | EPI_ISL_11782567 | 23/03/2021 | Africa / Tunisia / Ariana | Human | Random | Male | unknown | Severe | Not Vaccinated | Random | B.1 | G |
| hCoV-19/Tunisia/H6630/2021 | EPI_ISL_11782566 | 07/01/2021 | Africa / Tunisia / Tunis | Human | Random | Female | 76 | Asymptomatic | Not Vaccinated | Random | B.1 | G |
| hCoV-19/Tunisia/H6612/2021 | EPI_ISL_11782565 | 07/01/2021 | Africa / Tunisia / Tunis | Human | Random | Male | 39 | Mild infection | Not Vaccinated | Random | B.1.1 | GH |
| hCoV-19/Tunisia/GB0766/2020 | EPI_ISL_11782564 | 28/03/2020 | Africa / Tunisia / Medenine | Human | Random | Female | 64 | Mild infection | Not Vaccinated | Random | B.1 | G |
| hCoV-19/Tunisia/C4075/2021 | EPI_ISL_11782563 | 19/04/2021 | Africa / Tunisia / Tunis | Human | Random | Female | 48 | Severe | Not Vaccinated | Random | B.1.1.7 | GRY |
| hCoV-19/Tunisia/C1620/2021 | EPI_ISL_11782562 | 08/04/2021 | Africa / Tunisia / Tunis | Human | Random | Female | 30 | Asymptomatic | Not Vaccinated | Random | B | G |
| hCoV-19/Tunisia/B1388/2021 | EPI_ISL_11782561 | 09/03/2021 | Africa / Tunisia / Tunis | Human | Random | Female | 31 | Severe | Not Vaccinated | Random | A.27 | S |
| hCoV-19/Tunisia/AR0011/2020 | EPI_ISL_11782560 | 2020 | Africa / Tunisia / Ariana | Human | Random | unknown | unknown | Severe | Not Vaccinated | Random | B.1.1.198 | GR |
| hCoV-19/Tunisia/S-1561/2021 | EPI_ISL_11349066 | 26/11/2021 | Africa / Tunisia / Nabeul | Human | Random | Male | 46 | unknown | unknown | Random | AY.122 | GK |
| hCoV-19/Tunisia/S-1550/2021 | EPI_ISL_11349065 | 24/11/2021 | Africa / Tunisia / Nabeul | Human | Random | Female | 29 | unknown | unknown | Random | AY.122 | GK |
| hCoV-19/Tunisia/S-1548/2021 | EPI_ISL_11349064 | 24/11/2021 | Africa / Tunisia / Nabeul | Human | Random | Male | 8 | unknown | unknown | Random | AY.122 | GK |
| hCoV-19/Tunisia/S-1546/2021 | EPI_ISL_11349063 | 24/11/2021 | Africa / Tunisia / Nabeul | Human | Random | Male | 62 | unknown | unknown | Random | AY.122 | GK |
| hCoV-19/Tunisia/S-1545/2021 | EPI_ISL_11349062 | 22/11/2021 | Africa / Tunisia / Nabeul | Human | Random | Female | 91 | unknown | unknown | Random | AY.122 | GK |
| hCoV-19/Tunisia/S-1544/2021 | EPI_ISL_11349061 | 22/11/2021 | Africa / Tunisia / Nabeul | Human | Random | Female | 45 | unknown | unknown | Random | AY.122 | GK |
| hCoV-19/Tunisia/S-1543/2021 | EPI_ISL_11349060 | 22/11/2021 | Africa / Tunisia / Nabeul | Human | Random | Male | 45 | unknown | unknown | Random | AY.122 | GK |
| hCoV-19/Tunisia/S-1538/2021 | EPI_ISL_11349059 | 16/11/2021 | Africa / Tunisia / Nabeul | Human | Random | Female | 38 | unknown | unknown | Random | AY.122 | GK |
| hCoV-19/Tunisia/S-1535/2021 | EPI_ISL_11349058 | 12/11/2021 | Africa / Tunisia / Nabeul | Human | Random | Female | 53 | unknown | unknown | Random | AY.122 | GK |
| hCoV-19/Tunisia/S-1534/2021 | EPI_ISL_11349057 | 12/11/2021 | Africa / Tunisia / Nabeul | Human | Random | Female | 49 | unknown | unknown | Random | AY.122 | GK |
| hCoV-19/Tunisia/S-1532/2021 | EPI_ISL_11349056 | 12/11/2021 | Africa / Tunisia / Nabeul | Human | Random | Female | 32 | unknown | unknown | Random | B.1.617.2 | GK |
| hCoV-19/Tunisia/S-1529/2021 | EPI_ISL_11349055 | 05/11/2021 | Africa / Tunisia / Nabeul | Human | Random | Male | 52 | unknown | unknown | Random | AY.122 | GK |
| hCoV-19/Tunisia/S-1528/2021 | EPI_ISL_11349054 | 05/11/2021 | Africa / Tunisia / Nabeul | Human | Random | Female | 75 | unknown | unknown | Random | AY.122 | GK |
| hCoV-19/Tunisia/S-1513/2021 | EPI_ISL_11349053 | 17/11/2021 | Africa / Tunisia / Sousse | Human | Random | Female | 10 | unknown | unknown | Random | AY.4 | GK |
| hCoV-19/Tunisia/S-1511/2021 | EPI_ISL_11349052 | 17/11/2021 | Africa / Tunisia / Sousse | Human | Random | Male | 11 | unknown | unknown | Random | B.1.617.2 | GK |
| hCoV-19/Tunisia/S-1510/2021 | EPI_ISL_11349051 | 17/11/2021 | Africa / Tunisia / Sousse | Human | Random | Female | 13 | unknown | unknown | Random | AY.4 | GK |
| hCoV-19/Tunisia/S-1509/2021 | EPI_ISL_11349050 | 17/11/2021 | Africa / Tunisia / Sousse | Human | Random | Female | 12 | unknown | unknown | Random | AY.4 | GK |
| hCoV-19/Tunisia/S-1508/2021 | EPI_ISL_11349049 | 17/11/2021 | Africa / Tunisia / Sousse | Human | Random | Male | 13 | unknown | unknown | Random | AY.4 | GK |
| hCoV-19/Tunisia/S-1470/2021 | EPI_ISL_11349048 | 31/10/2021 | Africa / Tunisia / Sousse | Human | Random | Male | 55 | unknown | unknown | Random | AY.122 | GK |
| hCoV-19/Tunisia/S-1453/2021 | EPI_ISL_11349047 | 06/10/2021 | Africa / Tunisia / Sousse | Human | Random | Female | 61 | unknown | unknown | Random | AY.122 | GK |
| hCoV-19/Tunisia/S-1437/2021 | EPI_ISL_11349046 | 25/09/2021 | Africa / Tunisia / Sousse | Human | Random | Male | 25 | unknown | unknown | Random | AY.122 | GK |
| hCoV-19/Tunisia/S-1379/2021 | EPI_ISL_11349045 | 09/10/2021 | Africa / Tunisia / Kef | Human | Random | Male | 34 | unknown | unknown | Random | AY.122 | GK |
| hCoV-19/Tunisia/S-1366/2021 | EPI_ISL_11349044 | 02/10/2021 | Africa / Tunisia / Kef | Human | Random | Female | 30 | unknown | unknown | Random | AY.122 | GK |
| hCoV-19/Tunisia/S-1343/2021 | EPI_ISL_11349043 | 27/10/2021 | Africa / Tunisia / Nabeul | Human | Random | Male | 65 | unknown | unknown | Random | AY.122 | GK |
| hCoV-19/Tunisia/S-1307/2021 | EPI_ISL_11349042 | 08/10/2021 | Africa / Tunisia / Nabeul | Human | Random | Female | 18 | unknown | unknown | Random | AY.122 | GK |
| hCoV-19/Tunisia/S-1167/2021 | EPI_ISL_11349041 | 29/10/2021 | Africa / Tunisia / Tozeur | Human | Random | Male | 37 | unknown | unknown | Random | AY.122 | GK |
| hCoV-19/Tunisia/S-1103/2021 | EPI_ISL_11349040 | 29/10/2021 | Africa / Tunisia / Tozeur | Human | Random | Male | 33 | unknown | unknown | Random | AY.122 | GK |
| hCoV-19/Tunisia/S-1050/2021 | EPI_ISL_11349039 | 16/09/2021 | Africa / Tunisia / Sfax | Human | Random | Male | 56 | unknown | unknown | Random | AY.122 | GK |
| hCoV-19/Tunisia/S-1044/2021 | EPI_ISL_11349038 | 06/10/2021 | Africa / Tunisia / Sousse | Human | Random | Female | unknown | unknown | unknown | Random | AY.122 | GK |
| hCoV-19/Tunisia/F-4081/2021 | EPI_ISL_11349037 | 29/09/2021 | Africa / Tunisia / Tunis | Human | Random | Female | 28 | unknown | unknown | Random | AY.122 | GK |
| hCoV-19/Tunisia/V-2405/2021 | EPI_ISL_11334083 | 09/10/2021 | Africa / Tunisia / Tunis | Human | Random | Male | 54 | unknown | unknown | Random | AY.122 | GK |
| hCoV-19/Tunisia/S-1579/2021. | EPI_ISL_11334082 | 02/12/2021 | Africa / Tunisia / Tunis | Human | Random | Male | 23 | unknown | unknown | Random | BA.1 | GRA |
| hCoV-19/Tunisia/S-1576/2021 | EPI_ISL_11334081 | 24/11/2021 | Africa / Tunisia / Mednine | Human | Random | Female | 14 | unknown | unknown | Random | AY.122 | GK |
| hCoV-19/Tunisia/S-1568/2021 | EPI_ISL_11334080 | 02/11/2021 | Africa / Tunisia / Gabes | Human | Random | Male | 29 | unknown | unknown | Random | B.1.617.2 | GK |
| hCoV-19/Tunisia/S-1567/2021 | EPI_ISL_11334079 | 29/11/2021 | Africa / Tunisia / Nabeul | Human | Random | Male | 36 | unknown | unknown | Random | AY.122 | GK |
| hCoV-19/Tunisia/S-1566/2021 | EPI_ISL_11334078 | 29/11/2021 | Africa / Tunisia / Nabeul | Human | Random | Male | 42 | unknown | unknown | Random | AY.122 | GK |
| hCoV-19/Tunisia/S-1565/2021 | EPI_ISL_11334077 | 29/11/2021 | Africa / Tunisia / Nabeul | Human | Random | Female | 13 | unknown | unknown | Random | AY.122 | GK |
| hCoV-19/Tunisia/S-1564/2021 | EPI_ISL_11334076 | 29/11/2021 | Africa / Tunisia / Nabeul | Human | Random | Female | 13 | unknown | unknown | Random | AY.122 | GK |
| hCoV-19/Tunisia/S-1563/2021 | EPI_ISL_11334075 | 26/11/2021 | Africa / Tunisia / Nabeul | Human | Random | Male | 23 | unknown | unknown | Random | AY.122 | GK |
| hCoV-19/Tunisia/S-1562/2021 | EPI_ISL_11334074 | 26/11/2021 | Africa / Tunisia / Nabeul | Human | Random | Female | 14 | unknown | unknown | Random | AY.122 | GK |
| hCoV-19/Tunisia/S-1560/2021 | EPI_ISL_11334073 | 26/11/2021 | Africa / Tunisia / Nabeul | Human | Random | Male | 43 | unknown | unknown | Random | AY.122 | GK |
| hCoV-19/Tunisia/S-1559/2021 | EPI_ISL_11334072 | 26/11/2021 | Africa / Tunisia / Nabeul | Human | Random | Male | 11 | unknown | unknown | Random | AY.122 | GK |
| hCoV-19/Tunisia/S-1558/2021 | EPI_ISL_11334071 | 26/11/2021 | Africa / Tunisia / Nabeul | Human | Random | Female | 50 | unknown | unknown | Random | AY.122 | GK |
| hCoV-19/Tunisia/S-1557/2021 | EPI_ISL_11334070 | 26/11/2021 | Africa / Tunisia / Nabeul | Human | Random | Male | 15 | unknown | unknown | Random | AY.122 | GK |
| hCoV-19/Tunisia/S-1556/2021 | EPI_ISL_11334069 | 25/11/2021 | Africa / Tunisia / Nabeul | Human | Random | Female | 53 | unknown | unknown | Random | AY.122 | GK |
| hCoV-19/Tunisia/S-1555/2021 | EPI_ISL_11334068 | 25/11/2021 | Africa / Tunisia / Nabeul | Human | Random | Female | 3 | unknown | unknown | Random | AY.122 | GK |
| hCoV-19/Tunisia/S-1554/2021 | EPI_ISL_11334067 | 25/11/2021 | Africa / Tunisia / Nabeul | Human | Random | Male | 11 | unknown | unknown | Random | AY.122 | GK |
| hCoV-19/Tunisia/S-1552/2021 | EPI_ISL_11334066 | 25/11/2021 | Africa / Tunisia / Nabeul | Human | Random | Male | 50 | unknown | unknown | Random | AY.122 | GK |
| hCoV-19/Tunisia/S-1551/2021 | EPI_ISL_11334065 | 25/11/2021 | Africa / Tunisia / Nabeul | Human | Random | Female | 38 | unknown | unknown | Random | AY.122 | GK |
| hCoV-19/Tunisia/S-1549/2021 | EPI_ISL_11334064 | 24/11/2021 | Africa / Tunisia / Nabeul | Human | Random | Male | 84 | unknown | unknown | Random | AY.122 | GK |
| hCoV-19/Tunisia/S-1547/2021 | EPI_ISL_11334063 | 24/11/2021 | Africa / Tunisia / Nabeul | Human | Random | Female | 13 | unknown | unknown | Random | AY.122 | GK |
| hCoV-19/Tunisia/S-1540/2021 | EPI_ISL_11334062 | 18/11/2021 | Africa / Tunisia / Nabeul | Human | Random | Male | 32 | unknown | unknown | Random | AY.122 | GK |
| hCoV-19/Tunisia/S-1539/2021 | EPI_ISL_11334061 | 16/11/2021 | Africa / Tunisia / Nabeul | Human | Random | Female | 52 | unknown | unknown | Random | AY.122 | GK |
| hCoV-19/Tunisia/S-1537/2021 | EPI_ISL_11334060 | 16/11/2021 | Africa / Tunisia / Nabeul | Human | Random | Male | 41 | unknown | unknown | Random | AY.127 | GK |
| hCoV-19/Tunisia/S-1536/2021 | EPI_ISL_11334059 | 16/11/2021 | Africa / Tunisia / Nabeul | Human | Random | Female | 78 | unknown | unknown | Random | AY.122 | GK |
| hCoV-19/Tunisia/S-1533/2021 | EPI_ISL_11334058 | 12/11/2021 | Africa / Tunisia / Nabeul | Human | Random | Female | 37 | unknown | unknown | Random | AY.122 | GK |
| hCoV-19/Tunisia/S-1531/2021 | EPI_ISL_11334057 | 09/11/2021 | Africa / Tunisia / Tunis | Human | Random | Male | 65 | unknown | unknown | Random | AY.9.2 | GK |
| hCoV-19/Tunisia/S-1530/2021 | EPI_ISL_11334056 | 09/11/2021 | Africa / Tunisia / Tunis | Human | Random | Male | 61 | unknown | unknown | Random | AY.5.4 | GK |
| hCoV-19/Tunisia/S-1527/2021 | EPI_ISL_11334055 | 05/11/2021 | Africa / Tunisia / Nabeul | Human | Random | Female | 74 | unknown | unknown | Random | AY.122 | GK |
| hCoV-19/Tunisia/S-1525/2021 | EPI_ISL_11334054 | 26/11/2021 | Africa / Tunisia / Nabeul | Human | Random | Female | 16 | unknown | unknown | Random | AY.122 | GK |
| hCoV-19/Tunisia/S-1524/2021 | EPI_ISL_11334053 | 26/11/2021 | Africa / Tunisia / Manouba | Human | Random | Female | 16 | unknown | unknown | Random | AY.122 | GK |
| hCoV-19/Tunisia/S-1519/2021 | EPI_ISL_11334052 | 25/11/2021 | Africa / Tunisia / Manouba | Human | Random | Male | 41 | unknown | unknown | Random | AY.122 | GK |
| hCoV-19/Tunisia/S-1516/2021 | EPI_ISL_11334051 | 25/11/2021 | Africa / Tunisia / Manouba | Human | Random | Male | 12 | unknown | unknown | Random | AY.122 | GK |
| hCoV-19/Tunisia/S-1512/2021 | EPI_ISL_11334050 | 17/11/2021 | Africa / Tunisia / Sousse | Human | Random | Female | 13 | unknown | unknown | Random | AY.122 | GK |
| hCoV-19/Tunisia/S-1505/2021 | EPI_ISL_11334049 | 17/11/2021 | Africa / Tunisia / Sousse | Human | Random | Female | 58 | unknown | unknown | Random | AY.43 | GK |
| hCoV-19/Tunisia/S-1489/2021 | EPI_ISL_11334048 | 17/11/2021 | Africa / Tunisia / Sousse | Human | Random | Female | 36 | unknown | unknown | Random | AY.122 | GK |
| hCoV-19/Tunisia/S-1486/2021 | EPI_ISL_11334047 | 17/11/2021 | Africa / Tunisia / Sousse | Human | Random | Male | 58 | unknown | unknown | Random | AY.122 | GK |
| hCoV-19/Tunisia/S-1484/2021 | EPI_ISL_11334046 | 15/11/2021 | Africa / Tunisia / Sousse | Human | Random | Male | 57 | unknown | unknown | Random | AY.122 | GK |
| hCoV-19/Tunisia/S-1482/2021 | EPI_ISL_11334045 | 14/11/2021 | Africa / Tunisia / Sousse | Human | Random | Female | 70 | unknown | unknown | Random | AY.122 | GK |
| hCoV-19/Tunisia/S-1480/2021 | EPI_ISL_11334044 | 10/11/2021 | Africa / Tunisia / Sousse | Human | Random | Male | 37 | unknown | unknown | Random | AY.122 | GK |
| hCoV-19/Tunisia/S-1479/2021 | EPI_ISL_11334043 | 09/11/2021 | Africa / Tunisia / Sousse | Human | Random | Female | 65 | unknown | unknown | Random | AY.122 | GK |
| hCoV-19/Tunisia/S-1478/2021 | EPI_ISL_11334042 | 07/11/2021 | Africa / Tunisia / Sousse | Human | Random | Female | 37 | unknown | unknown | Random | AY.122 | GK |
| hCoV-19/Tunisia/S-1477/2021 | EPI_ISL_11334041 | 07/11/2021 | Africa / Tunisia / Sousse | Human | Random | Female | 60 | unknown | unknown | Random | AY.122 | GK |
| hCoV-19/Tunisia/S-1474/2021 | EPI_ISL_11334040 | 05/11/2021 | Africa / Tunisia / Sousse | Human | Random | Female | 49 | unknown | unknown | Random | AY.122 | GK |
| hCoV-19/Tunisia/S-1473/2021 | EPI_ISL_11334039 | 05/11/2021 | Africa / Tunisia / Sousse | Human | Random | Female | 85 | unknown | unknown | Random | AY.122 | GK |
| hCoV-19/Tunisia/S-1472/2021 | EPI_ISL_11334038 | 05/11/2021 | Africa / Tunisia / Sousse | Human | Random | Female | 83 | unknown | unknown | Random | AY.122 | GK |
| hCoV-19/Tunisia/S-1469/2021 | EPI_ISL_11334037 | 31/10/2021 | Africa / Tunisia / Sousse | Human | Random | Male | 21 | unknown | unknown | Random | AY.122 | GK |
| hCoV-19/Tunisia/S-1465/2021 | EPI_ISL_11334036 | 23/10/2021 | Africa / Tunisia / Sousse | Human | Random | Male | 78 | unknown | unknown | Random | AY.122 | GK |
| hCoV-19/Tunisia/S-1463/2021 | EPI_ISL_11334035 | 21/10/2021 | Africa / Tunisia / Sousse | Human | Random | Female | 58 | unknown | unknown | Random | AY.122 | GK |
| hCoV-19/Tunisia/S-1462/2021 | EPI_ISL_11334034 | 20/10/2021 | Africa / Tunisia / Sousse | Human | Random | Female | 60 | unknown | unknown | Random | AY.122 | GK |
| hCoV-19/Tunisia/S-1460/2021 | EPI_ISL_11334033 | 19/10/2021 | Africa / Tunisia / Sousse | Human | Random | Female | 85 | unknown | unknown | Random | AY.122 | GK |
| hCoV-19/Tunisia/S-1455/2021 | EPI_ISL_11334032 | 07/10/2021 | Africa / Tunisia / Sousse | Human | Random | Female | 29 | unknown | unknown | Random | AY.122 | GK |
| hCoV-19/Tunisia/S-1454/2021 | EPI_ISL_11334031 | 07/10/2021 | Africa / Tunisia / Mahdia | Human | Random | Female | 60 | unknown | unknown | Random | AY.122 | GK |
| hCoV-19/Tunisia/S-1449/2021 | EPI_ISL_11334030 | 01/10/2021 | Africa / Tunisia / Sousse | Human | Random | Female | 27 | unknown | unknown | Random | AY.122 | GK |
| hCoV-19/Tunisia/S-1445/2021 | EPI_ISL_11334029 | 09/11/2021 | Africa / Tunisia / Jendouba | Human | Random | Female | 72 | unknown | unknown | Random | AY.4 | GK |
| hCoV-19/Tunisia/S-1444/2021 | EPI_ISL_11334028 | 09/11/2021 | Africa / Tunisia / Sousse | Human | Random | Female | 68 | unknown | unknown | Random | AY.122 | GK |
| hCoV-19/Tunisia/S-1443/2021 | EPI_ISL_11334027 | 11/10/2021 | Africa / Tunisia / Sousse | Human | Random | Male | 47 | unknown | unknown | Random | AY.122 | GK |
| hCoV-19/Tunisia/S-1442/2021 | EPI_ISL_11334026 | 12/11/2021 | Africa / Tunisia / Sousse | Human | Random | Female | 45 | unknown | unknown | Random | AY.122 | GK |
| hCoV-19/Tunisia/S-1439/2021 | EPI_ISL_11334025 | 08/10/2021 | Africa / Tunisia / Sousse | Human | Random | Male | 25 | unknown | unknown | Random | AY.122 | GK |
| hCoV-19/Tunisia/S-1436/2021 | EPI_ISL_11334024 | 28/10/2021 | Africa / Tunisia / Sousse | Human | Random | Female | 29 | unknown | unknown | Random | AY.126 | GK |
| hCoV-19/Tunisia/S-1435/2021 | EPI_ISL_11334023 | 07/06/2021 | Africa / Tunisia / Sousse | Human | Random | Male | 25 | unknown | unknown | Random | AY.122 | GK |
| hCoV-19/Tunisia/S-1434/2021 | EPI_ISL_11334022 | 23/10/2021 | Africa / Tunisia / Sousse | Human | Random | Male | 1 | unknown | unknown | Random | AY.122 | GK |
| hCoV-19/Tunisia/S-1433/2021 | EPI_ISL_11334021 | 08/11/2021 | Africa / Tunisia / Bizerte | Human | Random | Female | unknown | unknown | unknown | Random | AY.122 | GK |
| hCoV-19/Tunisia/S-1432/2021 | EPI_ISL_11334020 | 09/11/2021 | Africa / Tunisia / Tataouine | Human | Random | Female | 56 | unknown | unknown | Random | AY.122 | GK |
| hCoV-19/Tunisia/S-1431/2021 | EPI_ISL_11334019 | 09/11/2021 | Africa / Tunisia / Tataouine | Human | Random | Female | 91 | unknown | unknown | Random | AY.122 | GK |
| hCoV-19/Tunisia/S-1428/2021 | EPI_ISL_11334018 | 09/11/2021 | Africa / Tunisia / Tataouine | Human | Random | Female | 20 | unknown | unknown | Random | AY.122 | GK |
| hCoV-19/Tunisia/S-1426/2021 | EPI_ISL_11334017 | 02/11/2021 | Africa / Tunisia / Tataouine | Human | Random | Female | 39 | unknown | unknown | Random | AY.122 | GK |
| hCoV-19/Tunisia/S-1425/2021 | EPI_ISL_11334016 | 02/11/2021 | Africa / Tunisia / Tataouine | Human | Random | Male | 59 | unknown | unknown | Random | AY.122 | GK |
| hCoV-19/Tunisia/S-1424/2021 | EPI_ISL_11334015 | 02/11/2021 | Africa / Tunisia / Tataouine | Human | Random | Male | 50 | unknown | unknown | Random | AY.122 | GK |
| hCoV-19/Tunisia/S-1423/2021 | EPI_ISL_11334014 | 02/11/2021 | Africa / Tunisia / Tataouine | Human | Random | Male | 46 | unknown | unknown | Random | AY.122 | GK |
| hCoV-19/Tunisia/S-1419/2021 | EPI_ISL_11334013 | 27/10/2021 | Africa / Tunisia / Kef | Human | Random | Male | 54 | unknown | unknown | Random | AY.122 | GK |
| hCoV-19/Tunisia/S-1415/2021 | EPI_ISL_11334012 | 15/10/2021 | Africa / Tunisia / Kef | Human | Random | Female | 48 | unknown | unknown | Random | AY.122 | GK |
| hCoV-19/Tunisia/S-1414/2021 | EPI_ISL_11334011 | 29/10/2021 | Africa / Tunisia / Kef | Human | Random | Female | 45 | unknown | unknown | Random | AY.122 | GK |
| hCoV-19/Tunisia/S-1412/2021 | EPI_ISL_11334010 | 15/10/2021 | Africa / Tunisia / Kef | Human | Random | Female | 45 | unknown | unknown | Random | AY.122 | GK |
| hCoV-19/Tunisia/S-1410/2021 | EPI_ISL_11334009 | 09/10/2021 | Africa / Tunisia / Kef | Human | Random | Female | 13 | unknown | unknown | Random | AY.122 | GK |
| hCoV-19/Tunisia/S-1408/2021 | EPI_ISL_11334008 | 07/10/2021 | Africa / Tunisia / Kef | Human | Random | Female | 11 | unknown | unknown | Random | AY.122 | GK |
| hCoV-19/Tunisia/S-1407/2021 | EPI_ISL_11334007 | 09/10/2021 | Africa / Tunisia / Kef | Human | Random | Female | 34 | unknown | unknown | Random | AY.122 | GK |
| hCoV-19/Tunisia/S-1399/2021 | EPI_ISL_11334006 | 02/10/2021 | Africa / Tunisia / Kef | Human | Random | Female | 64 | unknown | unknown | Random | AY.122 | GK |
| hCoV-19/Tunisia/S-1397/2021 | EPI_ISL_11334005 | 19/10/2021 | Africa / Tunisia / Kef | Human | Random | Male | 61 | unknown | unknown | Random | AY.122 | GK |
| hCoV-19/Tunisia/S-1390/2021 | EPI_ISL_11334004 | 05/10/2021 | Africa / Tunisia / Kef | Human | Random | Female | 16 | unknown | unknown | Random | AY.122 | GK |
| hCoV-19/Tunisia/S-1386/2021 | EPI_ISL_11334003 | 06/10/2021 | Africa / Tunisia / Kef | Human | Random | Female | 45 | unknown | unknown | Random | AY.122 | GK |
| hCoV-19/Tunisia/S-1385/2021 | EPI_ISL_11334002 | 04/10/2021 | Africa / Tunisia / Kef | Human | Random | Male | 65 | unknown | unknown | Random | AY.122 | GK |
| hCoV-19/Tunisia/S-1383/2021 | EPI_ISL_11334001 | 02/10/2021 | Africa / Tunisia / Kef | Human | Random | Male | 28 | unknown | unknown | Random | B.1.617.2 | GK |
| hCoV-19/Tunisia/S-1382/2021 | EPI_ISL_11334000 | 04/10/2021 | Africa / Tunisia / Kef | Human | Random | Male | 65 | unknown | unknown | Random | AY.122 | GK |
| hCoV-19/Tunisia/S-1381/2021 | EPI_ISL_11333999 | 04/10/2021 | Africa / Tunisia / Kef | Human | Random | Male | 25 | unknown | unknown | Random | AY.122 | GK |
| hCoV-19/Tunisia/S-1380/2021 | EPI_ISL_11333998 | 05/10/2021 | Africa / Tunisia / Kef | Human | Random | Female | 61 | unknown | unknown | Random | AY.122 | GK |
| hCoV-19/Tunisia/S-1378/2021 | EPI_ISL_11333997 | 03/10/2021 | Africa / Tunisia / Kef | Human | Random | Male | 13 | unknown | unknown | Random | AY.122 | GK |
| hCoV-19/Tunisia/S-1376/2021 | EPI_ISL_11333996 | 03/10/2021 | Africa / Tunisia / Kef | Human | Random | Female | 13 | unknown | unknown | Random | AY.122 | GK |
| hCoV-19/Tunisia/S-1371/2021 | EPI_ISL_11333995 | 02/10/2021 | Africa / Tunisia / Kef | Human | Random | Male | 50 | unknown | unknown | Random | AY.122 | GK |
| hCoV-19/Tunisia/S-1370/2021 | EPI_ISL_11333994 | 03/10/2021 | Africa / Tunisia / Kef | Human | Random | Female | unknown | unknown | unknown | Random | AY.122 | GK |
| hCoV-19/Tunisia/S-1367/2021 | EPI_ISL_11333993 | 02/10/2021 | Africa / Tunisia / Kef | Human | Random | Male | 26 | unknown | unknown | Random | B.1.617.2 | GK |
| hCoV-19/Tunisia/S-1364/2021 | EPI_ISL_11333992 | 04/11/2021 | Africa / Tunisia / Kairouan | Human | Random | Female | 13 | unknown | unknown | Random | AY.122 | GK |
| hCoV-19/Tunisia/S-1363/2021 | EPI_ISL_11333991 | 04/11/2021 | Africa / Tunisia / Kairouan | Human | Random | Male | 80 | unknown | unknown | Random | AY.122 | GK |
| hCoV-19/Tunisia/S-1362/2021 | EPI_ISL_11333990 | 04/11/2021 | Africa / Tunisia / Kairouan | Human | Random | Female | 49 | unknown | unknown | Random | AY.122 | GK |
| hCoV-19/Tunisia/S-1361/2021 | EPI_ISL_11333989 | 04/11/2021 | Africa / Tunisia / Kairouan | Human | Random | Male | 37 | unknown | unknown | Random | AY.122 | GK |
| hCoV-19/Tunisia/S-1360/2021 | EPI_ISL_11333988 | 04/11/2021 | Africa / Tunisia / Kairouan | Human | Random | Female | 35 | unknown | unknown | Random | AY.122 | GK |
| hCoV-19/Tunisia/S-1359/2021 | EPI_ISL_11333987 | 27/10/2021 | Africa / Tunisia / Kairouan | Human | Random | Female | 39 | unknown | unknown | Random | AY.122 | GK |
| hCoV-19/Tunisia/S-1358/2021 | EPI_ISL_11333986 | 04/11/2021 | Africa / Tunisia / Kairouan | Human | Random | Male | 76 | unknown | unknown | Random | AY.122 | GK |
| hCoV-19/Tunisia/S-1357/2021 | EPI_ISL_11333985 | 30/10/2021 | Africa / Tunisia / Kairouan | Human | Random | Female | 25 | unknown | unknown | Random | AY.122 | GK |
| hCoV-19/Tunisia/S-1356/2021 | EPI_ISL_11333984 | 25/10/2021 | Africa / Tunisia / Kairouan | Human | Random | Male | 50 | unknown | unknown | Random | AY.122 | GK |
| hCoV-19/Tunisia/S-1354/2021 | EPI_ISL_11333983 | 28/10/2021 | Africa / Tunisia / Kairouan | Human | Random | Female | 34 | unknown | unknown | Random | AY.122 | GK |
| hCoV-19/Tunisia/S-1353/2021 | EPI_ISL_11333982 | 02/11/2021 | Africa / Tunisia / Kairouan | Human | Random | Male | 66 | unknown | unknown | Random | AY.122 | GK |
| hCoV-19/Tunisia/S-1351/2021 | EPI_ISL_11333981 | 02/11/2021 | Africa / Tunisia / Gabes | Human | Random | Male | 29 | unknown | unknown | Random | AY.122 | GK |
| hCoV-19/Tunisia/S-1350/2021 | EPI_ISL_11333980 | 02/11/2021 | Africa / Tunisia / Nabeul | Human | Random | Male | 36 | unknown | unknown | Random | AY.122 | GK |
| hCoV-19/Tunisia/S-1349/2021 | EPI_ISL_11333979 | 02/11/2021 | Africa / Tunisia / Nabeul | Human | Random | Male | 84 | unknown | unknown | Random | AY.122 | GK |
| hCoV-19/Tunisia/S-1347/2021 | EPI_ISL_11333978 | 29/10/2021 | Africa / Tunisia / Nabeul | Human | Random | Female | 30 | unknown | unknown | Random | AY.122 | GK |
| hCoV-19/Tunisia/S-1346/2021 | EPI_ISL_11333977 | 28/10/2021 | Africa / Tunisia / Nabeul | Human | Random | Female | 29 | unknown | unknown | Random | AY.122 | GK |
| hCoV-19/Tunisia/S-1345/2021 | EPI_ISL_11333976 | 27/10/2021 | Africa / Tunisia / Nabeul | Human | Random | Female | 28 | unknown | unknown | Random | AY.122 | GK |
| hCoV-19/Tunisia/S-1342/2021 | EPI_ISL_11333975 | 27/10/2021 | Africa / Tunisia / Nabeul | Human | Random | Male | 76 | unknown | unknown | Random | AY.122 | GK |
| hCoV-19/Tunisia/S-1339/2021 | EPI_ISL_11333974 | 25/10/2021 | Africa / Tunisia / Nabeul | Human | Random | Male | 14 | unknown | unknown | Random | AY.122 | GK |
| hCoV-19/Tunisia/S-1338/2021 | EPI_ISL_11333973 | 25/10/2021 | Africa / Tunisia / Nabeul | Human | Random | Female | 43 | unknown | unknown | Random | AY.122 | GK |
| hCoV-19/Tunisia/S-1336/2021 | EPI_ISL_11333972 | 25/10/2021 | Africa / Tunisia / Nabeul | Human | Random | Male | 38 | unknown | unknown | Random | AY.122 | GK |
| hCoV-19/Tunisia/S-1333/2021 | EPI_ISL_11333971 | 25/10/2021 | Africa / Tunisia / Nabeul | Human | Random | Male | 15 | unknown | unknown | Random | AY.122 | GK |
| hCoV-19/Tunisia/S-1332/2021 | EPI_ISL_11333970 | 22/10/2021 | Africa / Tunisia / Nabeul | Human | Random | Male | 77 | unknown | unknown | Random | AY.122 | GK |
| hCoV-19/Tunisia/S-1327/2021 | EPI_ISL_11333969 | 20/10/2021 | Africa / Tunisia / Nabeul | Human | Random | Male | 80 | unknown | unknown | Random | AY.122 | GK |
| hCoV-19/Tunisia/S-1325/2021 | EPI_ISL_11333968 | 20/10/2021 | Africa / Tunisia / Nabeul | Human | Random | Male | 40 | unknown | unknown | Random | AY.122 | GK |
| hCoV-19/Tunisia/S-1321/2021 | EPI_ISL_11333967 | 14/10/2021 | Africa / Tunisia / Nabeul | Human | Random | Female | 39 | unknown | unknown | Random | AY.122 | GK |
| hCoV-19/Tunisia/S-1319/2021 | EPI_ISL_11333966 | 14/10/2021 | Africa / Tunisia / Nabeul | Human | Random | Male | 17 | unknown | unknown | Random | AY.122 | GK |
| hCoV-19/Tunisia/S-1315/2021 | EPI_ISL_11333965 | 13/10/2021 | Africa / Tunisia / Nabeul | Human | Random | Female | 15 | unknown | unknown | Random | AY.122 | GK |
| hCoV-19/Tunisia/S-1314/2021 | EPI_ISL_11333964 | 13/10/2021 | Africa / Tunisia / Nabeul | Human | Random | Female | 56 | unknown | unknown | Random | AY.122 | GK |
| hCoV-19/Tunisia/S-1313/2021 | EPI_ISL_11333963 | 13/10/2021 | Africa / Tunisia / Nabeul | Human | Random | Female | 24 | unknown | unknown | Random | AY.122 | GK |
| hCoV-19/Tunisia/S-1312/2021 | EPI_ISL_11333962 | 11/10/2021 | Africa / Tunisia / Nabeul | Human | Random | Male | 14 | unknown | unknown | Random | AY.122 | GK |
| hCoV-19/Tunisia/S-1311/2021 | EPI_ISL_11333961 | 11/10/2021 | Africa / Tunisia / Nabeul | Human | Random | Female | 67 | unknown | unknown | Random | AY.122 | GK |
| hCoV-19/Tunisia/S-1309/2021 | EPI_ISL_11333960 | 11/10/2021 | Africa / Tunisia / Nabeul | Human | Random | Male | 30 | unknown | unknown | Random | AY.122 | GK |
| hCoV-19/Tunisia/S-1308/2021 | EPI_ISL_11333959 | 08/10/2021 | Africa / Tunisia / Nabeul | Human | Random | Male | 48 | unknown | unknown | Random | AY.122 | GK |
| hCoV-19/Tunisia/S-1304/2021 | EPI_ISL_11333958 | 08/10/2021 | Africa / Tunisia / Nabeul | Human | Random | Female | 38 | unknown | unknown | Random | AY.122 | GK |
| hCoV-19/Tunisia/S-1303/2021 | EPI_ISL_11333957 | 07/10/2021 | Africa / Tunisia / Nabeul | Human | Random | Female | 3 | unknown | unknown | Random | AY.122 | GK |
| hCoV-19/Tunisia/S-1300/2021 | EPI_ISL_11333956 | 07/10/2021 | Africa / Tunisia / Nabeul | Human | Random | Female | 13 | unknown | unknown | Random | AY.122 | GK |
| hCoV-19/Tunisia/S-1299/2021 | EPI_ISL_11333955 | 25/10/2021 | Africa / Tunisia / Tunis | Human | Random | Male | 49 | unknown | unknown | Random | AY.36 | GK |
| hCoV-19/Tunisia/S-1298/2021 | EPI_ISL_11333954 | 25/10/2021 | Africa / Tunisia / Tunis | Human | Random | Male | 56 | unknown | unknown | Random | AY.127 | GK |
| hCoV-19/Tunisia/S-1294/2021 | EPI_ISL_11333953 | 29/10/2021 | Africa / Tunisia / Tozeur | Human | Random | Male | 65 | unknown | unknown | Random | AY.122 | GK |
| hCoV-19/Tunisia/S-1287/2021 | EPI_ISL_11333952 | 29/10/2021 | Africa / Tunisia / Tozeur | Human | Random | Female | 39 | unknown | unknown | Random | AY.122 | GK |
| hCoV-19/Tunisia/S-1286/2021 | EPI_ISL_11333951 | 29/10/2021 | Africa / Tunisia / Tozeur | Human | Random | Female | 28 | unknown | unknown | Random | AY.122 | GK |
| hCoV-19/Tunisia/S-1285/2021 | EPI_ISL_11333950 | 29/10/2021 | Africa / Tunisia / Monastir | Human | Random | Female | unknown | unknown | unknown | Random | AY.122 | GK |
| hCoV-19/Tunisia/S-1283/2021 | EPI_ISL_11333949 | 29/10/2021 | Africa / Tunisia / Monastir | Human | Random | Male | 68 | unknown | unknown | Random | AY.122 | GK |
| hCoV-19/Tunisia/S-1277/2021 | EPI_ISL_11333948 | 29/10/2021 | Africa / Tunisia / Tozeur | Human | Random | Female | 38 | unknown | unknown | Random | AY.122 | GK |
| hCoV-19/Tunisia/S-1276/2021 | EPI_ISL_11333947 | 29/10/2021 | Africa / Tunisia / Tozeur | Human | Random | Male | 49 | unknown | unknown | Random | AY.122 | GK |
| hCoV-19/Tunisia/S-1275/2021 | EPI_ISL_11333946 | 03/09/2021 | Africa / Tunisia / Monastir | Human | Random | Female | 60 | unknown | unknown | Random | AY.122 | GK |
| hCoV-19/Tunisia/S-1254/2021 | EPI_ISL_11333945 | 29/10/2021 | Africa / Tunisia / Tozeur | Human | Random | Female | 56 | unknown | unknown | Random | AY.122 | GK |
| hCoV-19/Tunisia/S-1244/2021 | EPI_ISL_11333944 | 29/10/2021 | Africa / Tunisia / Tozeur | Human | Random | Male | 45 | unknown | unknown | Random | AY.122 | GK |
| hCoV-19/Tunisia/S-1240/2021 | EPI_ISL_11333943 | 29/10/2021 | Africa / Tunisia / Tozeur | Human | Random | Male | 35 | unknown | unknown | Random | AY.122 | GK |
| hCoV-19/Tunisia/S-1237/2021 | EPI_ISL_11333942 | 29/10/2021 | Africa / Tunisia / Tozeur | Human | Random | Female | 48 | unknown | unknown | Random | AY.122 | GK |
| hCoV-19/Tunisia/S-1235/2021 | EPI_ISL_11333941 | 29/10/2021 | Africa / Tunisia / Tozeur | Human | Random | Male | 64 | unknown | unknown | Random | AY.122 | GK |
| hCoV-19/Tunisia/S-1234/2021 | EPI_ISL_11333940 | 29/09/2021 | Africa / Tunisia / Monastir | Human | Random | Female | 44 | unknown | unknown | Random | AY.122 | GK |
| hCoV-19/Tunisia/S-1224/2021 | EPI_ISL_11333939 | 29/10/2021 | Africa / Tunisia / Tozeur | Human | Random | Female | 40 | unknown | unknown | Random | AY.122 | GK |
| hCoV-19/Tunisia/S-1217/2021 | EPI_ISL_11333938 | 16/09/2021 | Africa / Tunisia / Monastir | Human | Random | Male | 13 | unknown | unknown | Random | AY.122 | GK |
| hCoV-19/Tunisia/S-1213/2021 | EPI_ISL_11333937 | 29/10/2021 | Africa / Tunisia / Tozeur | Human | Random | Female | 31 | unknown | unknown | Random | AY.122 | GK |
| hCoV-19/Tunisia/S-1208/2021 | EPI_ISL_11333936 | 29/10/2021 | Africa / Tunisia / Tozeur | Human | Random | Female | 74 | unknown | unknown | Random | AY.122 | GK |
| hCoV-19/Tunisia/S-1206/2021 | EPI_ISL_11333935 | 29/10/2021 | Africa / Tunisia / Tozeur | Human | Random | Female | 33 | unknown | unknown | Random | AY.122 | GK |
| hCoV-19/Tunisia/S-1205/2021 | EPI_ISL_11333934 | 29/10/2021 | Africa / Tunisia / Tozeur | Human | Random | Female | unknown | unknown | unknown | Random | AY.112 | GK |
| hCoV-19/Tunisia/S-1204/2021 | EPI_ISL_11333933 | 29/10/2021 | Africa / Tunisia / Tozeur | Human | Random | Male | 16 | unknown | unknown | Random | AY.122 | GK |
| hCoV-19/Tunisia/S-1200/2021 | EPI_ISL_11333932 | 29/10/2021 | Africa / Tunisia / Tozeur | Human | Random | Male | 7 | unknown | unknown | Random | AY.122 | GK |
| hCoV-19/Tunisia/S-1196/2021 | EPI_ISL_11333931 | 29/10/2021 | Africa / Tunisia / Monastir | Human | Random | Male | 59 | unknown | unknown | Random | AY.122 | GK |
| hCoV-19/Tunisia/S-1194/2021 | EPI_ISL_11333930 | 29/10/2021 | Africa / Tunisia / Tozeur | Human | Random | Male | 28 | unknown | unknown | Random | AY.122 | GK |
| hCoV-19/Tunisia/S-1192/2021 | EPI_ISL_11333929 | 29/10/2021 | Africa / Tunisia / Tozeur | Human | Random | Male | 71 | unknown | unknown | Random | AY.122 | GK |
| hCoV-19/Tunisia/S-1188/2021 | EPI_ISL_11333928 | 01/09/2021 | Africa / Tunisia / Monastir | Human | Random | Male | 39 | unknown | unknown | Random | AY.122 | GK |
| hCoV-19/Tunisia/S-1180/2021 | EPI_ISL_11333927 | 29/10/2021 | Africa / Tunisia / Monastir | Human | Random | Male | 49 | unknown | unknown | Random | B.1.1.7 | GRY |
| hCoV-19/Tunisia/S-1176/2021 | EPI_ISL_11333926 | 29/10/2021 | Africa / Tunisia / Tozeur | Human | Random | Female | 45 | unknown | unknown | Random | AY.122 | GK |
| hCoV-19/Tunisia/S-1162/2021 | EPI_ISL_11333925 | 29/10/2021 | Africa / Tunisia / Tozeur | Human | Random | Female | 48 | unknown | unknown | Random | B.1.617.2 | GK |
| hCoV-19/Tunisia/S-1154/2021 | EPI_ISL_11333924 | 20/09/2021 | Africa / Tunisia / Monastir | Human | Random | Female | 39 | unknown | unknown | Random | AY.122 | GK |
| hCoV-19/Tunisia/S-1153/2021 | EPI_ISL_11333923 | 20/09/2021 | Africa / Tunisia / Monastir | Human | Random | Female | 21 | unknown | unknown | Random | AY.122 | GK |
| hCoV-19/Tunisia/S-1152/2021 | EPI_ISL_11333922 | 16/09/2021 | Africa / Tunisia / Monastir | Human | Random | Female | 84 | unknown | unknown | Random | AY.122 | GK |
| hCoV-19/Tunisia/S-1142/2021 | EPI_ISL_11333921 | 16/09/2021 | Africa / Tunisia / Monastir | Human | Random | Female | 40 | unknown | unknown | Random | AY.43 | GK |
| hCoV-19/Tunisia/S-1138/2021 | EPI_ISL_11333920 | 16/09/2021 | Africa / Tunisia / Tozeur | Human | Random | Male | unknown | unknown | unknown | Random | AY.112 | GK |
| hCoV-19/Tunisia/S-1137/2021 | EPI_ISL_11333919 | 16/09/2021 | Africa / Tunisia / Monastir | Human | Random | Female | 44 | unknown | unknown | Random | AY.122 | GK |
| hCoV-19/Tunisia/S-1132/2021 | EPI_ISL_11333918 | 29/10/2021 | Africa / Tunisia / Tozeur | Human | Random | Male | 3 | unknown | unknown | Random | AY.122 | GK |
| hCoV-19/Tunisia/S-1130/2021 | EPI_ISL_11333917 | 29/10/2021 | Africa / Tunisia / Tozeur | Human | Random | Male | 41 | unknown | unknown | Random | AY.122 | GK |
| hCoV-19/Tunisia/S-1129/2021 | EPI_ISL_11333916 | 29/10/2021 | Africa / Tunisia / Monastir | Human | Random | Female | 30 | unknown | unknown | Random | AY.122 | GK |
| hCoV-19/Tunisia/S-1126/2021 | EPI_ISL_11333915 | 20/09/2021 | Africa / Tunisia / Monastir | Human | Random | Female | 42 | unknown | unknown | Random | AY.122 | GK |
| hCoV-19/Tunisia/S-1124/2021 | EPI_ISL_11333914 | 18/09/2021 | Africa / Tunisia / Monastir | Human | Random | Male | 61 | unknown | unknown | Random | AY.122 | GK |
| hCoV-19/Tunisia/S-1123/2021 | EPI_ISL_11333913 | 13/09/2021 | Africa / Tunisia / Monastir | Human | Random | Male | 33 | unknown | unknown | Random | AY.122 | GK |
| hCoV-19/Tunisia/S-1121/2021 | EPI_ISL_11333912 | 29/10/2021 | Africa / Tunisia / Tozeur | Human | Random | Female | 38 | unknown | unknown | Random | AY.122 | GK |
| hCoV-19/Tunisia/S-1118/2021 | EPI_ISL_11333911 | 15/09/2021 | Africa / Tunisia / Monastir | Human | Random | Female | 49 | unknown | unknown | Random | AY.122 | GK |
| hCoV-19/Tunisia/S-1115/2021 | EPI_ISL_11333910 | 29/10/2021 | Africa / Tunisia / Tozeur | Human | Random | Male | 24 | unknown | unknown | Random | AY.122 | GK |
| hCoV-19/Tunisia/S-1112/2021 | EPI_ISL_11333909 | 29/10/2021 | Africa / Tunisia / Tozeur | Human | Random | Female | 62 | unknown | unknown | Random | AY.122 | GK |
| hCoV-19/Tunisia/S-1111/2021 | EPI_ISL_11333908 | 29/10/2021 | Africa / Tunisia / Tozeur | Human | Random | Male | 51 | unknown | unknown | Random | AY.122 | GK |
| hCoV-19/Tunisia/S-1110/2021 | EPI_ISL_11333907 | 22/09/2021 | Africa / Tunisia / Monastir | Human | Random | Female | 42 | unknown | unknown | Random | AY.122 | GK |
| hCoV-19/Tunisia/S-1108/2021 | EPI_ISL_11333906 | 29/10/2021 | Africa / Tunisia / Monastir | Human | Random | Male | 78 | unknown | unknown | Random | AY.122 | GK |
| hCoV-19/Tunisia/S-1107/2021 | EPI_ISL_11333905 | 29/10/2021 | Africa / Tunisia / Tozeur | Human | Random | Female | 11 | unknown | unknown | Random | AY.122 | GK |
| hCoV-19/Tunisia/S-1105/2021 | EPI_ISL_11333904 | 28/09/2021 | Africa / Tunisia / Monastir | Human | Random | Female | 21 | unknown | unknown | Random | AY.122 | GK |
| hCoV-19/Tunisia/S-1100/2021 | EPI_ISL_11333903 | 29/10/2021 | Africa / Tunisia / Monastir | Human | Random | Female | 55 | unknown | unknown | Random | AY.43 | GK |
| hCoV-19/Tunisia/S-1097/2021 | EPI_ISL_11333902 | 18/09/2021 | Africa / Tunisia / Monastir | Human | Random | Male | 64 | unknown | unknown | Random | AY.122 | GK |
| hCoV-19/Tunisia/S-1094/2021 | EPI_ISL_11333901 | 29/10/2021 | Africa / Tunisia / Tozeur | Human | Random | Male | 64 | unknown | unknown | Random | AY.122 | GK |
| hCoV-19/Tunisia/S-1088/2021 | EPI_ISL_11333900 | 20/09/2021 | Africa / Tunisia / Monastir | Human | Random | Female | 52 | unknown | unknown | Random | AY.122 | GK |
| hCoV-19/Tunisia/S-1087/2021 | EPI_ISL_11333899 | 22/09/2021 | Africa / Tunisia / Monastir | Human | Random | Female | 72 | unknown | unknown | Random | AY.122 | GK |
| hCoV-19/Tunisia/S-1085/2021 | EPI_ISL_11333898 | 22/09/2021 | Africa / Tunisia / Monastir | Human | Random | Male | 21 | unknown | unknown | Random | AY.122 | GK |
| hCoV-19/Tunisia/S-1083/2021 | EPI_ISL_11333897 | 29/10/2021 | Africa / Tunisia / Tozeur | Human | Random | Female | 35 | unknown | unknown | Random | AY.122 | GK |
| hCoV-19/Tunisia/S-1082/2021 | EPI_ISL_11333896 | 28/10/2021 | Africa / Tunisia / Sousse | Human | Random | Female | 40 | unknown | unknown | Random | AY.122 | GK |
| hCoV-19/Tunisia/S-1081/2021 | EPI_ISL_11333895 | 19/10/2021 | Africa / Tunisia / Sfax | Human | Random | Male | 34 | unknown | unknown | Random | AY.122 | GK |
| hCoV-19/Tunisia/S-1080/2021 | EPI_ISL_11333894 | 19/10/2021 | Africa / Tunisia / Sfax | Human | Random | Male | 35 | unknown | unknown | Random | AY.122 | GK |
| hCoV-19/Tunisia/S-1078/2021 | EPI_ISL_11333893 | 07/10/2021 | Africa / Tunisia / Sfax | Human | Random | Female | 49 | unknown | unknown | Random | AY.122 | GK |
| hCoV-19/Tunisia/S-1077/2021 | EPI_ISL_11333892 | 05/10/2021 | Africa / Tunisia / Sfax | Human | Random | Female | 37 | unknown | unknown | Random | AY.122 | GK |
| hCoV-19/Tunisia/S-1076/2021 | EPI_ISL_11333891 | 05/10/2021 | Africa / Tunisia / Sfax | Human | Random | Male | 47 | unknown | unknown | Random | AY.122 | GK |
| hCoV-19/Tunisia/S-1075/2021 | EPI_ISL_11333890 | 05/10/2021 | Africa / Tunisia / Sfax | Human | Random | Male | 84 | unknown | unknown | Random | AY.122 | GK |
| hCoV-19/Tunisia/S-1074/2021 | EPI_ISL_11333889 | 05/10/2021 | Africa / Tunisia / Sfax | Human | Random | Female | 28 | unknown | unknown | Random | AY.122 | GK |
| hCoV-19/Tunisia/S-1073/2021 | EPI_ISL_11333888 | 05/10/2021 | Africa / Tunisia / Sfax | Human | Random | Male | 40 | unknown | unknown | Random | AY.122 | GK |
| hCoV-19/Tunisia/S-1072/2021 | EPI_ISL_11333887 | 05/10/2021 | Africa / Tunisia / Sfax | Human | Random | Female | 30 | unknown | unknown | Random | AY.122 | GK |
| hCoV-19/Tunisia/S-1071/2021 | EPI_ISL_11333886 | 05/10/2021 | Africa / Tunisia / Sfax | Human | Random | Male | 61 | unknown | unknown | Random | AY.122 | GK |
| hCoV-19/Tunisia/S-1070/2021 | EPI_ISL_11333885 | 21/10/2021 | Africa / Tunisia / Sfax | Human | Random | Male | 38 | unknown | unknown | Random | AY.122 | GK |
| hCoV-19/Tunisia/S-1069/2021 | EPI_ISL_11333884 | 21/10/2021 | Africa / Tunisia / Sfax | Human | Random | Female | 68 | unknown | unknown | Random | AY.122 | GK |
| hCoV-19/Tunisia/S-1068/2021 | EPI_ISL_11333883 | 01/10/2021 | Africa / Tunisia / Sfax | Human | Random | Female | 28 | unknown | unknown | Random | AY.122 | GK |
| hCoV-19/Tunisia/S-1067/2021 | EPI_ISL_11333882 | 26/09/2021 | Africa / Tunisia / Sfax | Human | Random | Female | 24 | unknown | unknown | Random | AY.122 | GK |
| hCoV-19/Tunisia/S-1065/2021 | EPI_ISL_11333881 | 29/09/2021 | Africa / Tunisia / Sfax | Human | Random | Male | 38 | unknown | unknown | Random | AY.122 | GK |
| hCoV-19/Tunisia/S-1064/2021 | EPI_ISL_11333880 | 21/10/2021 | Africa / Tunisia / Sfax | Human | Random | Male | 52 | unknown | unknown | Random | AY.122 | GK |
| hCoV-19/Tunisia/S-1063/2021 | EPI_ISL_11333879 | 21/10/2021 | Africa / Tunisia / Sfax | Human | Random | Female | 54 | unknown | unknown | Random | AY.122 | GK |
| hCoV-19/Tunisia/S-1061/2021 | EPI_ISL_11333878 | 24/09/2021 | Africa / Tunisia / Sfax | Human | Random | Male | 90 | unknown | unknown | Random | AY.122 | GK |
| hCoV-19/Tunisia/S-1060/2021 | EPI_ISL_11333877 | 25/09/2021 | Africa / Tunisia / Sfax | Human | Random | Female | 68 | unknown | unknown | Random | AY.122 | GK |
| hCoV-19/Tunisia/S-1059/2021 | EPI_ISL_11333876 | 23/09/2021 | Africa / Tunisia / Sfax | Human | Random | Male | 63 | unknown | unknown | Random | B.1.617.2 | GK |
| hCoV-19/Tunisia/S-1057/2021 | EPI_ISL_11333875 | 22/09/2021 | Africa / Tunisia / Sfax | Human | Random | Male | 39 | unknown | unknown | Random | AY.122 | GK |
| hCoV-19/Tunisia/S-1056/2021 | EPI_ISL_11333874 | 21/10/2021 | Africa / Tunisia / Sfax | Human | Random | Male | 46 | unknown | unknown | Random | AY.122 | GK |
| hCoV-19/Tunisia/S-1055/2021 | EPI_ISL_11333873 | 21/10/2021 | Africa / Tunisia / Sfax | Human | Random | Female | 52 | unknown | unknown | Random | AY.122 | GK |
| hCoV-19/Tunisia/S-1054/2021 | EPI_ISL_11333872 | 17/09/2021 | Africa / Tunisia / Sfax | Human | Random | Male | 53 | unknown | unknown | Random | AY.122 | GK |
| hCoV-19/Tunisia/S-1053/2021 | EPI_ISL_11333871 | 17/09/2021 | Africa / Tunisia / Sfax | Human | Random | Female | 14 | unknown | unknown | Random | AY.122 | GK |
| hCoV-19/Tunisia/S-1052/2021 | EPI_ISL_11333870 | 16/09/2021 | Africa / Tunisia / Sfax | Human | Random | Female | 57 | unknown | unknown | Random | AY.122 | GK |
| hCoV-19/Tunisia/S-1047/2021 | EPI_ISL_11333869 | 15/09/2021 | Africa / Tunisia / Sfax | Human | Random | Male | 27 | unknown | unknown | Random | AY.122 | GK |
| hCoV-19/Tunisia/S-1046/2021 | EPI_ISL_11333868 | 15/09/2021 | Africa / Tunisia / Sfax | Human | Random | Female | 22 | unknown | unknown | Random | AY.122 | GK |
| hCoV-19/Tunisia/S-1045/2021 | EPI_ISL_11333867 | 15/09/2021 | Africa / Tunisia / Sfax | Human | Random | Female | 54 | unknown | unknown | Random | AY.122 | GK |
| hCoV-19/Tunisia/F-4882/2021 | EPI_ISL_11333866 | 10/11/2021 | Africa / Tunisia / Tunis | Human | Random | Female | 18 | unknown | unknown | Random | AY.122 | GK |
| hCoV-19/Tunisia/F-4881/2021 | EPI_ISL_11333865 | 10/11/2021 | Africa / Tunisia / Tunis | Human | Random | Female | 42 | unknown | unknown | Random | AY.122 | GK |
| hCoV-19/Tunisia/F-4879/2021 | EPI_ISL_11333864 | 10/11/2021 | Africa / Tunisia / Tunis | Human | Random | Female | 52 | unknown | unknown | Random | AY.122 | GK |
| hCoV-19/Tunisia/F-4878/2021 | EPI_ISL_11333863 | 10/11/2021 | Africa / Tunisia / Tunis | Human | Random | Male | 41 | unknown | unknown | Random | AY.122 | GK |
| hCoV-19/Tunisia/F-4820/2021 | EPI_ISL_11333862 | 05/11/2021 | Africa / Tunisia / Tunis | Human | Random | Female | 41 | unknown | unknown | Random | AY.122 | GK |
| hCoV-19/Tunisia/F-4762/2021 | EPI_ISL_11333861 | 02/11/2021 | Africa / Tunisia / Tunis | Human | Random | Male | 25 | unknown | unknown | Random | AY.122 | GK |
| hCoV-19/Tunisia/F-4722/2021 | EPI_ISL_11333860 | 29/10/2021 | Africa / Tunisia / Tunis | Human | Random | Male | 84 | unknown | unknown | Random | AY.122 | GK |
| hCoV-19/Tunisia/F-4697/2021 | EPI_ISL_11333859 | 28/10/2021 | Africa / Tunisia / Manouba | Human | Random | Female | 75 | unknown | unknown | Random | AY.122 | GK |
| hCoV-19/Tunisia/F-4610/2021 | EPI_ISL_11333858 | 22/10/2021 | Africa / Tunisia / Tunis | Human | Random | Male | 49 | unknown | unknown | Random | AY.122 | GK |
| hCoV-19/Tunisia/F-4584/2021 | EPI_ISL_11333857 | 20/10/2021 | Africa / Tunisia / Tunis | Human | Random | Male | 43 | unknown | unknown | Random | AY.122 | GK |
| hCoV-19/Tunisia/F-4572/2021 | EPI_ISL_11333856 | 20/10/2021 | Africa / Tunisia / Tunis | Human | Random | Male | 40 | unknown | unknown | Random | AY.122 | GK |
| hCoV-19/Tunisia/F-4532/2021 | EPI_ISL_11333855 | 17/10/2021 | Africa / Tunisia / Tunis | Human | Random | Female | 46 | unknown | unknown | Random | AY.122 | GK |
| hCoV-19/Tunisia/F-4397/2021 | EPI_ISL_11333854 | 09/10/2021 | Africa / Tunisia / Tunis | Human | Random | Female | 49 | unknown | unknown | Random | AY.122 | GK |
| hCoV-19/Tunisia/F-4392/2021 | EPI_ISL_11333853 | 09/10/2021 | Africa / Tunisia / Tunis | Human | Random | Female | 45 | unknown | unknown | Random | AY.122 | GK |
| hCoV-19/Tunisia/F-4279/2021 | EPI_ISL_11333852 | 05/10/2021 | Africa / Tunisia / Tunis | Human | Random | Female | 39 | unknown | unknown | Random | AY.122 | GK |
| hCoV-19/Tunisia/6719/2021 | EPI_ISL_11266054 | 02/02/2021 | Africa / Tunisia / Tunis | Human | Random | Male | 8 | Mild infection | Not Vaccinated | Random | B.1.1.7 | GRY |
| hCoV-19/Tunisia/B-3544/2021 | EPI_ISL_11266043 | 20/03/2021 | Africa / Tunisia / Tunis | Human | Random | Female | 18 | Mild infection | Not Vaccinated | Random | B.1.160 | GH |
| hCoV-19/Tunisia/B-3226/2021 | EPI_ISL_11266051 | 18/03/2021 | Africa / Tunisia / Tunis | Human | Random | Female | 14 | Mild infection | Not Vaccinated | Random | B.1.1.7 | GRY |
| hCoV-19/Tunisia/C-2510/2021 | EPI_ISL_11266050 | 12/04/2021 | Africa / Tunisia / Ariana | Human | Random | Male | 14 | Mild infection | Not Vaccinated | Random | B.1.1.7 | GRY |
| hCoV-19/Tunisia/C-2001/2021 | EPI_ISL_11266049 | 08/04/2021 | Africa / Tunisia / Tunis | Human | Random | Female | 15 | Mild infection | Not Vaccinated | Random | B.1.620 | G |
| hCoV-19/Tunisia/D-1879/2021 | EPI_ISL_11266048 | 17/05/2021 | Africa / Tunisia / Tunis | Human | Random | Female | 15 | Mild infection | Not Vaccinated | Random | B.1.1.7 | G |
| hCoV-19/Tunisia/A-0052/2021 | EPI_ISL_11266047 | 21/04/2021 | Africa / Tunisia / Ariana | Human | Random | Male | 16 | Mild infection | Not Vaccinated | Random | B.1.1.7 | GRY |
| hCoV-19/Tunisia/B-3757/2021 | EPI_ISL_11266046 | 23/03/2021 | Africa / Tunisia / Ben Arous | Human | Random | Female | 17 | Mild infection | Not Vaccinated | Random | B.1.1.7 | GRY |
| hCoV-19/Tunisia/D-2920/2021 | EPI_ISL_11266045 | 21/05/2021 | Africa / Tunisia / Tunis | Human | Random | Female | 17 | Mild infection | Not Vaccinated | Random | B.1.160 | GH |
| hCoV-19/Tunisia/A-0075/2021 | EPI_ISL_11266044 | 25/05/2021 | Africa / Tunisia / Tunis | Human | Random | Female | 17 | Mild infection | Not Vaccinated | Random | B.1.1.7 | GRY |
| hCoV-19/Tunisia/12412/2021 | EPI_ISL_11266042 | 22/04/2021 | Africa / Tunisia / Tunis | Human | Random | Male | 18 | Mild infection | Not Vaccinated | Random | B.1.1.7 | G |
| hCoV-19/Tunisia/12122/2021 | EPI_ISL_11266041 | 22/04/2021 | Africa / Tunisia / Tunis | Human | Random | Female | 18 | Mild infection | Not Vaccinated | Random | B.1.1.7 | GRY |
| hCoV-19/Tunisia/11686/2021 | EPI_ISL_11266040 | 22/04/2021 | Africa / Tunisia / Tunis | Human | Random | Female | 18 | Mild infection | Not Vaccinated | Random | B.1.1.7 | GRY |
| hCoV-19/Tunisia/11219/2021 | EPI_ISL_11266039 | 22/04/2021 | Africa / Tunisia / Ben Arous | Human | Random | Female | 18 | Mild infection | Not Vaccinated | Random | B.1.1.7 | GRY |
| hCoV-19/Tunisia/11825/2021 | EPI_ISL_11266038 | 22/04/2021 | Africa / Tunisia / Ariana | Human | Random | Female | 18 | Mild infection | Not Vaccinated | Random | B.1.1.7 | GRY |
| hCoV-19/Tunisia/D-1592/2021 | EPI_ISL_11266037 | 15/05/2021 | Africa / Tunisia / Tunis | Human | Random | Female | 18 | Mild infection | Not Vaccinated | Random | B.1.1.7 | G |
| hCoV-19/Tunisia/B-0757/2021 | EPI_ISL_11266036 | 25/05/2021 | Africa / Tunisia / Tunis | Human | Random | Male | 18 | Mild infection | Not Vaccinated | Random | B.1.1.7 | GRY |
| hCoV-19/Tunisia/A-0077/2021 | EPI_ISL_11266035 | 25/05/2021 | Africa / Tunisia / Manouba | Human | Random | Female | 18 | Mild infection | Not Vaccinated | Random | AY.122 | GK |
| hCoV-19/Tunisia/Y-9515/2021 | EPI_ISL_11266034 | 31/03/2021 | Africa / Tunisia / Tunis | Human | Random | Female | 20 | Mild infection | Not Vaccinated | Random | B.1.1.7 | GR |
| hCoV-19/Tunisia/C-0094/2021 | EPI_ISL_11266033 | 02/04/2021 | Africa / Tunisia / Tunis | Human | Random | Female | 28 | Asymptomatic | Not Vaccinated | Random | A.23.1 | S |
| hCoV-19/Tunisia/C-1944/2021 | EPI_ISL_11266032 | 08/04/2021 | Africa / Tunisia / Silina | Human | Random | Female | 30 | Mild infection | Not Vaccinated | Random | B.1 | G |
| hCoV-19/Tunisia/C-2405/2021 | EPI_ISL_11266031 | 10/04/2021 | Africa / Tunisia / Tunis | Human | Random | Male | 33 | Hospitalized | Not Vaccinated | Random | B.1.1.7 | GRY |
| hCoV-19/Tunisia/S-0427/2021 | EPI_ISL_11266030 | 04/05/2021 | Africa / Tunisia / Mednine | Human | Random | Female | 34 | Mild infection | Not Vaccinated | Random | B.1.351 | GH |
| hCoV-19/Tunisia/Y-9546/2021 | EPI_ISL_11266029 | 31/03/2021 | Africa / Tunisia / Silina | Human | Random | Female | 36 | Mild infection | Vaccinated | Random | B.1.1.318 | GR |
| hCoV-19/Tunisia/C-2774/2021 | EPI_ISL_11266028 | 12/04/2021 | Africa / Tunisia / Silina | Human | Random | Male | 48 | Hospitalized | Not Vaccinated | Random | B.1.1.7 | GRY |
| hCoV-19/Tunisia/Y-9517/2021 | EPI_ISL_11266027 | 31/03/2021 | Africa / Tunisia / Tunis | Human | Random | Female | 52 | Hospitalized | Not Vaccinated | Random | B.1.1.7 | GRY |
| hCoV-19/Tunisia/C-2416/2021 | EPI_ISL_11266026 | 10/04/2021 | Africa / Tunisia / Tunis | Human | Random | Male | 53 | Severe infection | Not Vaccinated | Random | B.1.1.7 | GRY |
| hCoV-19/Tunisia/C-2005/2021 | EPI_ISL_11266025 | 08/04/2021 | Africa / Tunisia / Sidi Bouzid | Human | Random | Male | 57 | Mild infection | Not Vaccinated | Random | B.1.620 | G |
| hCoV-19/Tunisia/C-0091/2021 | EPI_ISL_11266024 | 02/04/2021 | Africa / Tunisia / Tunis | Human | Random | Female | 61 | Asymptomatic | Not Vaccinated | Random | B.1.1.7 | GRY |
| hCoV-19/Tunisia/C-2422/2021 | EPI_ISL_11266023 | 10/04/2021 | Africa / Tunisia / Manouba | Human | Random | Female | 61 | Mild infection | Not Vaccinated | Random | B.1.177 | GV |
| hCoV-19/Tunisia/S-0438/2021 | EPI_ISL_11266022 | 02/04/2021 | Africa / Tunisia / Mednine | Human | Random | Female | 64 | Mild infection | Not Vaccinated | Random | B.1.525 | G |
| hCoV-19/Tunisia/C-2408/2021 | EPI_ISL_11266021 | 10/04/2021 | Africa / Tunisia / Ariana | Human | Random | Female | 66 | Mild infection | Not Vaccinated | Random | B.1.160 | GH |
| hCoV-19/Tunisia/S-0420/2021 | EPI_ISL_11266020 | 30/04/2021 | Africa / Tunisia / Mednine | Human | Random | Female | 66 | Mild infection | Not Vaccinated | Random | B.1.525 | G |
| hCoV-19/Tunisia/S-0437/2021 | EPI_ISL_11266019 | 01/04/2021 | Africa / Tunisia / Mednine | Human | Random | Male | 68 | Mild infection | Not Vaccinated | Random | B.1.525 | G |
| hCoV-19/Tunisia/C-1955/2021 | EPI_ISL_11266018 | 08/04/2021 | Africa / Tunisia / Tunis | Human | Random | Female | 73 | Mild infection | Not Vaccinated | Random | B.1 | G |
| hCoV-19/Tunisia/C-1099/2021 | EPI_ISL_11266017 | 06/04/2021 | Africa / Tunisia / Tunis | Human | Random | Female | 79 | Severe infection | Not Vaccinated | Random | B.1.160 | GH |
| hCoV-19/Tunisia/C-2008/2021 | EPI_ISL_11266016 | 08/04/2021 | Africa / Tunisia / Tunis | Human | Random | Female | 81 | Mild infection | Not Vaccinated | Random | B.1 | G |
| hCoV-19/Tunisia/C-1954/2021 | EPI_ISL_11266015 | 08/04/2021 | Africa / Tunisia / Ariana | Human | Random | Female | 83 | Hospitalized | Not Vaccinated | Random | B.1 | G |
| hCoV-19/Tunisia/C-2400/2021 | EPI_ISL_11266014 | 10/04/2021 | Africa / Tunisia / Ariana | Human | Random | Female | 83 | Hospitalized | Not Vaccinated | Random | B.1.1 | GR |
| hCoV-19/Tunisia/B-5033/2021 | EPI_ISL_11266013 | 29/04/2021 | Africa / Tunisia / Ben Arous | Human | Random | Female | 92 | Hospitalized | Not Vaccinated | Random | B.1.1.7 | GRY |
| hCoV-19/Tunisia/Y-9534/2021 | EPI_ISL_11266012 | 31/03/2021 | Africa / Tunisia / Tunis | Human | Random | Male | 1 month | Mild infection | Not Vaccinated | Random | B.1.1.7 | GRY |
| hCoV-19/Tunisia/10820/2021 | EPI_ISL_11266011 | 24/04/2021 | Africa / Tunisia / Tunis | Human | Random | Male | 1 month | Mild infection | Not Vaccinated | Random | B.1 | GH |
| hCoV-19/Tunisia/9385/2021 | EPI_ISL_11266010 | 02/04/2021 | Africa / Tunisia / Tunis | Human | Random | Male | 4 months | Mild infection | Not Vaccinated | Random | B.1.1.7 | GRY |
| hCoV-19/Tunisia/6313/2021 | EPI_ISL_11266009 | 28/01/2021 | Africa / Tunisia / Nabeul | Human | Random | Male | 5 months | Mild infection | Not Vaccinated | Random | B.1.177 | GV |
| hCoV-19/Tunisia/6841/2021 | EPI_ISL_11266008 | 04/02/2021 | Africa / Tunisia / Tunis | Human | Random | Male | 5 months | Mild infection | Not Vaccinated | Random | B.1.160 | GH |
| hCoV-19/Tunisia/6283/2021 | EPI_ISL_11266007 | 27/01/2021 | Africa / Tunisia / Beja | Human | Random | Male | 7 months | Mild infection | Not Vaccinated | Random | B.1.1.7 | GRY |
| hCoV-19/Tunisia/8917/2021 | EPI_ISL_11266006 | 22/03/2021 | Africa / Tunisia / Ariana | Human | Random | Male | 7 months | Mild infection | Not Vaccinated | Random | B.1.1.7 | G |
| hCoV-19/Tunisia/S-0368/2021 | EPI_ISL_11266005 | 07/05/2021 | Africa / Tunisia / Ben Arous | Human | Random | Male | unknown | Mild infection | Not Vaccinated | Random | B.1.620 | G |
| hCoV-19/Tunisia/S-0367/2021 | EPI_ISL_11266004 | 07/05/2021 | Africa / Tunisia / Ben Arous | Human | Random | Female | unknown | Mild infection | Not Vaccinated | Random | B.1.620 | G |
| hCoV-19/Tunisia/Z3148/2020 | EPI_ISL_11172724 | 21/10/2020 | Africa / Tunisia / Tunis | Human | Random | Female | unknown | Severe | Not Vaccinated | Random | B.1.177 | GV |
| hCoV-19/Tunisia/Y6024/2020 | EPI_ISL_11172718 | 16/10/2020 | Africa / Tunisia / Tunis | Human | Random | Female | 64 | Death | Not Vaccinated | Random | B.1.428.2 | GH |
| hCoV-19/Tunisia/X6187/2020 | EPI_ISL_11172707 | 09/09/2020 | Africa / Tunisia / Silina | Human | Random | Male | 52 | Asymptomatic | Not Vaccinated | Random | B.1.1 | GR |
| hCoV-19/Tunisia/X6185/2020 | EPI_ISL_11172706 | 09/09/2020 | Africa / Tunisia / Silina | Human | Random | Male | 52 | Asymptomatic | Not Vaccinated | Random | B.1.1 | GR |
| hCoV-19/Tunisia/X6179/2020 | EPI_ISL_11172705 | 09/09/2020 | Africa / Tunisia / Silina | Human | Random | Male | 11 | Asymptomatic | Not Vaccinated | Random | B.1.1 | GR |
| hCoV-19/Tunisia/Q1935/2020 | EPI_ISL_11172678 | 24/10/2020 | Africa / Tunisia / Tunis | Human | Random | Female | 73 | Severe | Not Vaccinated | Random | B.1.160 | GH |
| hCoV-19/Tunisia/Q1708/2020 | EPI_ISL_11172677 | 13/10/2020 | Africa / Tunisia / Tunis | Human | Random | Female | 65 | Severe | Not Vaccinated | Random | B.1.428.2 | GH |
| hCoV-19/Tunisia/Q1327/2020 | EPI_ISL_11172676 | 10/10/2020 | Africa / Tunisia / Tunis | Human | Random | Male | 67 | Severe | Not Vaccinated | Random | B.1.1.189 | GR |
| hCoV-19/Tunisia/Q0297/2020 | EPI_ISL_11172675 | 25/03/2020 | Africa / Tunisia / Tunis | Human | Random | Male | 86 | Severe | Not Vaccinated | Random | B.1.1 | GH |
| hCoV-19/Tunisia/P1879/2020 | EPI_ISL_11172674 | 25/03/2020 | Africa / Tunisia / Tunis | Human | Random | Male | 66 | Severe | Not Vaccinated | Random | B.1 | G |
| hCoV-19/Tunisia/P1689/2020 | EPI_ISL_11172673 | 13/04/2020 | Africa / Tunisia / Gafsa | Human | Random | Female | 8 | Asymptomatic | Not Vaccinated | Random | B.1.1 | GR |
| hCoV-19/Tunisia/M3579/2020 | EPI_ISL_11172667 | 01/11/2020 | Africa / Tunisia / Tunis | Human | Random | Male | 37 | Moderate | Not Vaccinated | Random | B.1.160 | GH |
| hCoV-19/Tunisia/M2355/2020 | EPI_ISL_11172666 | 27/10/2020 | Africa / Tunisia / Tunis | Human | Random | Female | 75 | Asymptomatic | Not Vaccinated | Random | B.1.177 | GV |
| hCoV-19/Tunisia/H3750/2020 | EPI_ISL_11172660 | 16/12/2020 | Africa / Tunisia / Tunis | Human | Random | Female | 46 | Severe | Not Vaccinated | Random | B.1.177 | G |
| hCoV-19/Tunisia/GB0757/2020 | EPI_ISL_11172659 | 28/03/2020 | Africa / Tunisia / Medenine | Human | Random | Male | 75 | Mild infection | Not Vaccinated | Random | B.1 | G |
| hCoV-19/Tunisia/GB0753/2020 | EPI_ISL_11172658 | 28/03/2020 | Africa / Tunisia / Medenine | Human | Random | Female | 22 | Mild infection | Not Vaccinated | Random | B.1 | GH |
| hCoV-19/Tunisia/GB0726/2020 | EPI_ISL_11172657 | 28/03/2020 | Africa / Tunisia / Ariana | Human | Random | Female | 58 | Mild infection | Not Vaccinated | Random | B.1 | G |
| hCoV-19/Tunisia/GB0406/2020 | EPI_ISL_11172656 | 27/03/2020 | Africa / Tunisia / Kebili | Human | Random | Female | 24 | Mild infection | Not Vaccinated | Random | B.1 | G |
| hCoV-19/Tunisia/GB1412/2020 | EPI_ISL_11172655 | 02/04/2020 | Africa / Tunisia / Sfax | Human | Random | Female | 23 | Mild infection | Not Vaccinated | Random | B.1.1 | GR |
| hCoV-19/Tunisia/GB1410/2020 | EPI_ISL_11172654 | 02/04/2020 | Africa / Tunisia / Sfax | Human | Random | Female | 53 | Mild infection | Not Vaccinated | Random | B.1.1 | GR |
| hCoV-19/Tunisia/AR0009/2020 | EPI_ISL_11172648 | 2020 | Africa / Tunisia / Ariana | Human | Random | unknown | unknown | Severe | Not Vaccinated | Random | B.1.1.198 | G |
| hCoV-19/Tunisia/AR0007/2020 | EPI_ISL_11172647 | 2020 | Africa / Tunisia / Ariana | Human | Random | unknown | unknown | Severe | Not Vaccinated | Random | B.1.595 | GH |
| hCoV-19/Tunisia/Z4214/2020 | EPI_ISL_11172726 | 10/11/2020 | Africa / Tunisia / Tunis | Human | Random | Male | 60 | Asymptomatic | Not Vaccinated | Random | B.1.160 | GH |
| hCoV-19/Tunisia/Z3496/2020 | EPI_ISL_11172725 | 23/10/2020 | Africa / Tunisia / Tunis | Human | Random | Female | unknown | Asymptomatic | Not Vaccinated | Random | B.1.428.2 | GH |
| hCoV-19/Tunisia/Z1405/2020 | EPI_ISL_11172723 | 26/09/2020 | Africa / Tunisia / Tunis | Human | Random | Male | 85 | Death | Not Vaccinated | Random | B.1.160 | GH |
| hCoV-19/Tunisia/Z0672/2020 | EPI_ISL_11172722 | 23/09/2020 | Africa / Tunisia / Tunis | Human | Random | Female | 66 | Mild infection | Not Vaccinated | Random | B.1.1 | GR |
| hCoV-19/Tunisia/Y7052/2020 | EPI_ISL_11172721 | 22/10/2020 | Africa / Tunisia / Tunis | Human | Random | Female | 31 | Mild infection | Not Vaccinated | Random | B.1.160 | GH |
| hCoV-19/Tunisia/Y6620/2020 | EPI_ISL_11172720 | 20/10/2020 | Africa / Tunisia / Tunis | Human | Random | Female | 90 | Death | Not Vaccinated | Random | B.1.177 | GV |
| hCoV-19/Tunisia/Y6032/2020 | EPI_ISL_11172719 | 16/10/2020 | Africa / Tunisia / Tunis | Human | Random | Female | 75 | Severe | Not Vaccinated | Random | B.1.389 | GH |
| hCoV-19/Tunisia/Y5522/2020 | EPI_ISL_11172717 | 13/10/2020 | Africa / Tunisia / Tunis | Human | Random | Female | unknown | Asymptomatic | Not Vaccinated | Random | B.1.160 | GH |
| hCoV-19/Tunisia/Y3940/2020 | EPI_ISL_11172716 | 05/10/2020 | Africa / Tunisia / Tunis | Human | Random | Female | unknown | Severe | Not Vaccinated | Random | B.1.22 | GH |
| hCoV-19/Tunisia/Y2660/2020 | EPI_ISL_11172715 | 29/09/2020 | Africa / Tunisia / Kairouan | Human | Random | Female | 54 | Asymptomatic | Not Vaccinated | Random | B.1 | G |
| hCoV-19/Tunisia/X9556/2020 | EPI_ISL_11172714 | 21/09/2020 | Africa / Tunisia / Tunis | Human | Random | Female | 37 | Asymptomatic | Not Vaccinated | Random | B.1.428.2 | GH |
| hCoV-19/Tunisia/X9466/2020 | EPI_ISL_11172713 | 19/09/2020 | Africa / Tunisia / Manouba | Human | Random | Male | 34 | Mild infection | Not Vaccinated | Random | B.1.22 | GH |
| hCoV-19/Tunisia/X9047/2020 | EPI_ISL_11172712 | 19/09/2020 | Africa / Tunisia / Tunis | Human | Random | Male | 64 | Severe | Not Vaccinated | Random | B.1.22 | O |
| hCoV-19/Tunisia/X8935/2020 | EPI_ISL_11172711 | 18/09/2020 | Africa / Tunisia / Tunis | Human | Random | Male | 77 | Severe | Not Vaccinated | Random | B.1.177 | GV |
| hCoV-19/Tunisia/X6363/2020 | EPI_ISL_11172710 | 09/09/2020 | Africa / Tunisia / Bizerte | Human | Random | Male | 36 | Mild infection | Not Vaccinated | Random | B.1 | G |
| hCoV-19/Tunisia/X6195/2020 | EPI_ISL_11172709 | 09/09/2020 | Africa / Tunisia / Silina | Human | Random | Female | 14 | Asymptomatic | Not Vaccinated | Random | B.1.1 | GR |
| hCoV-19/Tunisia/X6189/2020 | EPI_ISL_11172708 | 09/09/2020 | Africa / Tunisia / Silina | Human | Random | Female | 51 | Asymptomatic | Not Vaccinated | Random | B.1.1 | GR |
| hCoV-19/Tunisia/X5137/2020 | EPI_ISL_11172704 | 07/09/2020 | Africa / Tunisia / Silina | Human | Random | Female | 21 | Asymptomatic | Not Vaccinated | Random | B.1.22 | GH |
| hCoV-19/Tunisia/X4100/2020 | EPI_ISL_11172703 | 04/09/2020 | Africa / Tunisia / Tunis | Human | Random | Female | unknown | Severe | Not Vaccinated | Random | B.1.160 | GH |
| hCoV-19/Tunisia/U4424/2021 | EPI_ISL_11172702 | 16/02/2021 | Africa / Tunisia / Ariana | Human | Random | Female | 32 | Severe | Not Vaccinated | Random | B.1.1.7 | GRY |
| hCoV-19/Tunisia/U3384/2021 | EPI_ISL_11172701 | 16/02/2021 | Africa / Tunisia / Tunis | Human | Random | Male | 72 | Asymptomatic | Not Vaccinated | Random | B.1.160 | GH |
| hCoV-19/Tunisia/U3383/2021 | EPI_ISL_11172700 | 16/02/2021 | Africa / Tunisia / Tunis | Human | Random | Male | 32 | Asymptomatic | Not Vaccinated | Random | B.1.160 | GH |
| hCoV-19/Tunisia/S0644/2021 | EPI_ISL_11172699 | 24/06/2021 | Africa / Tunisia / Silina | Human | Random | Female | 44 | Severe | Not Vaccinated | Random | B.1.1.7 | G |
| hCoV-19/Tunisia/S0544/2021 | EPI_ISL_11172698 | 21/04/2021 | Africa / Tunisia / Bizerte | Human | Random | Male | 61 | Severe | Not Vaccinated | Random | B.1.1.7 | GR |
| hCoV-19/Tunisia/S0543/2021 | EPI_ISL_11172697 | 28/03/2021 | Africa / Tunisia / Nabeul | Human | Random | Female | 75 | Severe | Not Vaccinated | Random | B.1.525 | G |
| hCoV-19/Tunisia/S0529/2021 | EPI_ISL_11172696 | 16/06/2021 | Africa / Tunisia / Kairouan | Human | Random | Female | 37 | Mild infection | Not Vaccinated | Random | AY.122 | GK |
| hCoV-19/Tunisia/S0527/2021 | EPI_ISL_11172695 | 14/06/2021 | Africa / Tunisia / Kairouan | Human | Random | Female | 1 | Mild infection | Not Vaccinated | Random | AY.122 | GK |
| hCoV-19/Tunisia/S0526/2021 | EPI_ISL_11172694 | 12/06/2021 | Africa / Tunisia / Kairouan | Human | Random | Male | 35 | Mild infection | Not Vaccinated | Random | AY.122 | GK |
| hCoV-19/Tunisia/S0525/2021 | EPI_ISL_11172693 | 11/06/2021 | Africa / Tunisia / Kairouan | Human | Random | Female | 1 | Mild infection | Not Vaccinated | Random | AY.122 | GK |
| hCoV-19/Tunisia/S0502/2021 | EPI_ISL_11172692 | 10/06/2021 | Africa / Tunisia / Kairouan | Human | Random | Male | 62 | Death | Not Vaccinated | Random | AY.122 | GK |
| hCoV-19/Tunisia/S0170/2021 | EPI_ISL_11172691 | 01/04/2021 | Africa / Tunisia / Kairouan | Human | Random | Male | unknown | Severe | Not Vaccinated | Random | B.1.177 | O |
| hCoV-19/Tunisia/S0150/2021 | EPI_ISL_11172690 | 24/03/2021 | Africa / Tunisia / Manouba | Human | Random | Male | 27 | Severe | Not Vaccinated | Random | B.1.160 | GH |
| hCoV-19/Tunisia/S0141/2021 | EPI_ISL_11172689 | 29/03/2021 | Africa / Tunisia / Manouba | Human | Random | Male | 68 | Severe | Not Vaccinated | Random | B.1.160 | GH |
| hCoV-19/Tunisia/S0136/2021 | EPI_ISL_11172688 | 29/03/2021 | Africa / Tunisia / Manouba | Human | Random | Female | 52 | Severe | Not Vaccinated | Random | B.1.160 | GH |
| hCoV-19/Tunisia/S0071/2021 | EPI_ISL_11172687 | 23/03/2021 | Africa / Tunisia / Ariana | Human | Random | Male | unknown | Severe | Not Vaccinated | Random | B.1.428 | GH |
| hCoV-19/Tunisia/S0070/2021 | EPI_ISL_11172686 | 23/03/2021 | Africa / Tunisia / Ariana | Human | Random | Male | unknown | Severe | Not Vaccinated | Random | B.1.428 | G |
| hCoV-19/Tunisia/S0069/2021 | EPI_ISL_11172685 | 23/03/2021 | Africa / Tunisia / Ariana | Human | Random | Male | unknown | Severe | Not Vaccinated | Random | B.1.177 | GV |
| hCoV-19/Tunisia/S0066/2021 | EPI_ISL_11172684 | 23/03/2021 | Africa / Tunisia / Ariana | Human | Random | Male | unknown | Severe | Not Vaccinated | Random | B.1.1.198 | GR |
| hCoV-19/Tunisia/S0063/2021 | EPI_ISL_11172683 | 23/03/2021 | Africa / Tunisia / Ariana | Human | Random | Male | unknown | Severe | Not Vaccinated | Random | B.1.1.7 | GRY |
| hCoV-19/Tunisia/S0060/2021 | EPI_ISL_11172682 | 23/03/2021 | Africa / Tunisia / Ariana | Human | Random | Male | unknown | Severe | Not Vaccinated | Random | B.1.1.198 | G |
| hCoV-19/Tunisia/S0059/2021 | EPI_ISL_11172681 | 23/03/2021 | Africa / Tunisia / Ariana | Human | Random | Male | unknown | Severe | Not Vaccinated | Random | B.1.177 | GV |
| hCoV-19/Tunisia/Q2835/2021 | EPI_ISL_11172680 | 21/01/2021 | Africa / Tunisia / Tunis | Human | Random | Male | 25 | Severe | Not Vaccinated | Random | B.1.1 | GR |
| hCoV-19/Tunisia/Q2372/2021 | EPI_ISL_11172679 | 20/01/2021 | Africa / Tunisia / Tunis | Human | Random | Female | 31 | Severe | Not Vaccinated | Random | B.1 | G |
| hCoV-19/Tunisia/P1603/2020 | EPI_ISL_11172672 | 12/04/2020 | Africa / Tunisia / Kebili | Human | Random | Male | unknown | Asymptomatic | Not Vaccinated | Random | B | G |
| hCoV-19/Tunisia/M5685/2020 | EPI_ISL_11172671 | 11/11/2020 | Africa / Tunisia / Tunis | Human | Random | Female | 92 | Severe | Not Vaccinated | Random | B.1 | G |
| hCoV-19/Tunisia/M5067/2020 | EPI_ISL_11172670 | 06/11/2020 | Africa / Tunisia / Tunis | Human | Random | Male | 72 | Asymptomatic | Not Vaccinated | Random | B.1.160 | GH |
| hCoV-19/Tunisia/M5038/2020 | EPI_ISL_11172669 | 06/11/2020 | Africa / Tunisia / Tunis | Human | Random | Female | unknown | Mild infection | Not Vaccinated | Random | B.1.177 | GV |
| hCoV-19/Tunisia/M3581/2020 | EPI_ISL_11172668 | 01/11/2020 | Africa / Tunisia / Tunis | Human | Random | Male | 72 | Severe | Not Vaccinated | Random | B.1.1.198 | GR |
| hCoV-19/Tunisia/M2333/2020 | EPI_ISL_11172665 | 27/10/2020 | Africa / Tunisia / Tunis | Human | Random | Male | 75 | Death | Not Vaccinated | Random | B.1.1.198 | GR |
| hCoV-19/Tunisia/M1938/2020 | EPI_ISL_11172664 | 26/10/2020 | Africa / Tunisia / Tunis | Human | Random | Male | unknown | Severe | Not Vaccinated | Random | B.1.428.2 | GH |
| hCoV-19/Tunisia/H8943/2021 | EPI_ISL_11172663 | 15/01/2021 | Africa / Tunisia / Tozeur | Human | Random | Male | 35 | Asymptomatic | Not Vaccinated | Random | B.1.177.81 | G |
| hCoV-19/Tunisia/H8082/2021 | EPI_ISL_11172662 | 13/01/2021 | Africa / Tunisia / Tunis | Human | Random | Male | unknown | Death | Not Vaccinated | Random | B.1.160 | G |
| hCoV-19/Tunisia/H4141/2020 | EPI_ISL_11172661 | 17/12/2020 | Africa / Tunisia / Tunis | Human | Random | Male | 44 | Asymptomatic | Not Vaccinated | Random | B.1 | G |
| hCoV-19/Tunisia/C4336/2021 | EPI_ISL_11172652 | 19/04/2021 | Africa / Tunisia / Tunis | Human | Random | Male | 72 | Severe | Not Vaccinated | Random | B.1.1.7 | GRY |
| hCoV-19/Tunisia/B4720/2021 | EPI_ISL_11172651 | 27/03/2021 | Africa / Tunisia / Tunis | Human | Random | Male | 15 | Asymptomatic | Not Vaccinated | Random | B.1.160 | GH |
| hCoV-19/Tunisia/B1166/2021 | EPI_ISL_11172650 | 08/03/2021 | Africa / Tunisia / Tunis | Human | Random | Male | 31 | Mild infection | Not Vaccinated | Random | B.1.1.7 | GRY |
| hCoV-19/Tunisia/B1159/2021 | EPI_ISL_11172649 | 08/03/2021 | Africa / Tunisia / Tunis | Human | Random | Male | 36 | Mild infection | Not Vaccinated | Random | B.1.1.7 | GRY |
| hCoV-19/Tunisia/AR0003/2020 | EPI_ISL_11172646 | 2020 | Africa / Tunisia / Ariana | Human | Random | unknown | unknown | Severe | Not Vaccinated | Random | B | G |
| hCoV-19/Tunisia/A9807/2020 | EPI_ISL_11172645 | 28/08/2020 | Africa / Tunisia / Jendouba | Human | Random | Female | 37 | Asymptomatic | Not Vaccinated | Random | B.1.1 | GR |
| hCoV-19/Tunisia/AR0005/2020 | EPI_ISL_11172644 | 2020 | Africa / Tunisia / Ariana | Human | Random | unknown | unknown | Severe | Not Vaccinated | Random | B.1 | G |
| hCoV-19/Tunisia/29198/2021 | EPI_ISL_10863147 | 26/04/2021 | Africa / Tunisia / Ariana | Human | Baseline surveillance | Male | unknown | unknown | unknown | Baseline surveillance | B.1 | G |
| hCoV-19/Tunisia/29197/2021 | EPI_ISL_10863146 | 26/04/2021 | Africa / Tunisia / Ariana | Human | Baseline surveillance | Male | 60 | unknown | unknown | Baseline surveillance | B.1.1.7 | GR |
| hCoV-19/Tunisia/28613/2021 | EPI_ISL_10863145 | 23/04/2021 | Africa / Tunisia / Ariana | Human | Baseline surveillance | Male | 57 | unknown | unknown | Baseline surveillance | B.1 | G |
| hCoV-19/Tunisia/28105/2021 | EPI_ISL_10863144 | 21/04/2021 | Africa / Tunisia / Ariana | Human | Baseline surveillance | Female | 70 | unknown | unknown | Baseline surveillance | B.1.1.7 | GR |
| hCoV-19/Tunisia/23919/2021 | EPI_ISL_10863143 | 05/04/2021 | Africa / Tunisia / Ariana | Human | Baseline surveillance | Male | 74 | unknown | unknown | Baseline surveillance | B.1.1.7 | GRY |
| hCoV-19/Tunisia/18267/2021 | EPI_ISL_10863142 | 01/03/2021 | Africa / Tunisia / Ariana | Human | Baseline surveillance | Female | 21 | unknown | unknown | Baseline surveillance | B.1.1.7 | GR |
| hCoV-19/Tunisia/51470/2021 | EPI_ISL_10863141 | 31/07/2021 | Africa / Tunisia / Ariana | Human | Baseline surveillance | Male | 59 | unknown | unknown | Baseline surveillance | AY.122 | GK |
| hCoV-19/Tunisia/51383/2021 | EPI_ISL_10863140 | 30/07/2021 | Africa / Tunisia / Ariana | Human | Baseline surveillance | Female | 32 | unknown | unknown | Baseline surveillance | AY.122 | GK |
| hCoV-19/Tunisia/51027/2021 | EPI_ISL_10863139 | 29/10/2021 | Africa / Tunisia / Ariana | Human | Baseline surveillance | Female | 50 | unknown | unknown | Baseline surveillance | B.1.1.1 | GR |
| hCoV-19/Tunisia/48535/2021 | EPI_ISL_10863138 | 15/07/2021 | Africa / Tunisia / Ariana | Human | Baseline surveillance | Male | 39 | unknown | unknown | Baseline surveillance | AY.122 | GK |
| hCoV-19/Tunisia/47218/2021 | EPI_ISL_10863137 | 13/01/2021 | Africa / Tunisia / Ariana | Human | Baseline surveillance | Male | 28 | unknown | unknown | Baseline surveillance | AY.122 | GK |
| hCoV-19/Tunisia/45618/2021 | EPI_ISL_10863136 | 13/01/2021 | Africa / Tunisia / Ariana | Human | Baseline surveillance | Female | 44 | unknown | unknown | Baseline surveillance | B.1.1.198 | GR |
| hCoV-19/Tunisia/44788/2021 | EPI_ISL_10863135 | 01/07/2021 | Africa / Tunisia / Ariana | Human | Baseline surveillance | Female | 35 | unknown | unknown | Baseline surveillance | Unassigned | GK |
| hCoV-19/Tunisia/44149/2021 | EPI_ISL_10863134 | 29/06/2021 | Africa / Tunisia / Ariana | Human | Baseline surveillance | Male | 51 | unknown | unknown | Baseline surveillance | AY.122 | GK |
| hCoV-19/Tunisia/43291/2021 | EPI_ISL_10863133 | 25/06/2021 | Africa / Tunisia / Ariana | Human | Baseline surveillance | Male | 36 | unknown | unknown | Baseline surveillance | AY.122 | GK |
| hCoV-19/Tunisia/35510/2021 | EPI_ISL_10863132 | 25/05/2021 | Africa / Tunisia / Ariana | Human | Baseline surveillance | Female | 60 | unknown | unknown | Baseline surveillance | B.1.1.7 | GRY |
| hCoV-19/Tunisia/35311/2021 | EPI_ISL_10863131 | 25/05/2021 | Africa / Tunisia / Ariana | Human | Baseline surveillance | Male | 71 | unknown | unknown | Baseline surveillance | B.1.1.7 | GR |
| hCoV-19/Tunisia/35049/2021 | EPI_ISL_10863130 | 24/05/2021 | Africa / Tunisia / Ariana | Human | Baseline surveillance | Male | 68 | unknown | unknown | Baseline surveillance | B.1.1.7 | GRY |
| hCoV-19/Tunisia/32458/2021 | EPI_ISL_10863129 | 10/05/2021 | Africa / Tunisia / Ariana | Human | Baseline surveillance | Female | 61 | unknown | unknown | Baseline surveillance | B.1.1 | G |
| hCoV-19/Tunisia/6396/2021 | EPI_ISL_10863128 | 18/01/2021 | Africa / Tunisia / Ben Arous | Human | Baseline surveillance | Female | 82 | unknown | unknown | Baseline surveillance | B.1.160 | GH |
| hCoV-19/Tunisia/18671/2021 | EPI_ISL_10863127 | 05/03/2021 | Africa / Tunisia / Bizert Nord | Human | Baseline surveillance | Female | 55 | unknown | unknown | Baseline surveillance | B.1.160 | GH |
| hCoV-19/Tunisia/13679/2021 | EPI_ISL_10863126 | 05/02/2021 | Africa / Tunisia / Bni khaled | Human | Baseline surveillance | Female | 9 | unknown | unknown | Baseline surveillance | B.1.160 | GH |
| hCoV-19/Tunisia/8046/2022 | EPI_ISL_10863125 | 07/02/2022 | Africa / Tunisia / El Haouaria | Human | Baseline surveillance | Male | 2 | unknown | unknown | Baseline surveillance | BA.2 | GRA |
| hCoV-19/Tunisia/8039/2022 | EPI_ISL_10863124 | 07/02/2022 | Africa / Tunisia / El Haouaria | Human | Baseline surveillance | Female | 78 | unknown | unknown | Baseline surveillance | BA.2 | GRA |
| hCoV-19/Tunisia/8037/2022 | EPI_ISL_10863123 | 07/02/2022 | Africa / Tunisia / El Haouaria | Human | Baseline surveillance | Male | 42 | unknown | unknown | Baseline surveillance | BA.2 | GRA |
| hCoV-19/Tunisia/18915/2021 | EPI_ISL_10863122 | 04/03/2021 | Africa / Tunisia / Kasserine | Human | Baseline surveillance | Female | 55 | unknown | unknown | Baseline surveillance | B.1.1.7 | GRY |
| hCoV-19/Tunisia/8029/2022 | EPI_ISL_10863121 | 07/02/2022 | Africa / Tunisia / Korba | Human | Baseline surveillance | Male | 88 | unknown | unknown | Baseline surveillance | BA.1.1 | GRA |
| hCoV-19/Tunisia/61581/2020 | EPI_ISL_10863120 | 23/11/2020 | Africa / Tunisia / Mahdia | Human | Baseline surveillance | Female | 21 | unknown | unknown | Baseline surveillance | B.1 | G |
| hCoV-19/Tunisia/48668/2021 | EPI_ISL_10863119 | 22/07/2021 | Africa / Tunisia / Manouba | Human | Baseline surveillance | Female | 35 | unknown | unknown | Baseline surveillance | AY.122 | GK |
| hCoV-19/Tunisia/47038/2021 | EPI_ISL_10863118 | 08/07/2021 | Africa / Tunisia / Manouba | Human | Baseline surveillance | Female | 35 | unknown | unknown | Baseline surveillance | AY.122 | GK |
| hCoV-19/Tunisia/44262/2021 | EPI_ISL_10863117 | 29/06/2021 | Africa / Tunisia / Manouba | Human | Baseline surveillance | Female | 36 | unknown | unknown | Baseline surveillance | AY.122 | GK |
| hCoV-19/Tunisia/49910/2021 | EPI_ISL_10863116 | 16/07/2021 | Africa / Tunisia / Manouba | Human | Baseline surveillance | Female | 37 | unknown | unknown | Baseline surveillance | B.1.1.7 | GRY |
| hCoV-19/Tunisia/37524/2021 | EPI_ISL_10863115 | 03/06/2021 | Africa / Tunisia / Manouba | Human | Baseline surveillance | Male | 38 | unknown | unknown | Baseline surveillance | B.1.1.7 | GRY |
| hCoV-19/Tunisia/8148/2022 | EPI_ISL_10863114 | 07/02/2022 | Africa / Tunisia / Menzil Tmim | Human | Baseline surveillance | Male | 53 | unknown | unknown | Baseline surveillance | BA.1.17.2 | GRA |
| hCoV-19/Tunisia/19346/2021 | EPI_ISL_10863113 | 03/03/2021 | Africa / Tunisia / Nabeul | Human | Baseline surveillance | Female | 30 | unknown | unknown | Baseline surveillance | B.1.160 | G |
| hCoV-19/Tunisia/21249/2021 | EPI_ISL_10863112 | 08/01/2021 | Africa / Tunisia / Nabeul | Human | Baseline surveillance | Male | 21 | unknown | unknown | Baseline surveillance | B.1.177 | GV |
| hCoV-19/Tunisia/19363/2021 | EPI_ISL_10863111 | 03/03/2021 | Africa / Tunisia / Nabeul | Human | Baseline surveillance | Female | 54 | unknown | unknown | Baseline surveillance | B.1.160 | GH |
| hCoV-19/Tunisia/8034/2022 | EPI_ISL_10863110 | 07/02/2022 | Africa / Tunisia / Nabeul | Human | Baseline surveillance | Male | 42 | unknown | unknown | Baseline surveillance | BA.1.1 | GRA |
| hCoV-19/Tunisia/8021/2022 | EPI_ISL_10863109 | 07/02/2022 | Africa / Tunisia / Nabeul | Human | Baseline surveillance | Male | 81 | unknown | unknown | Baseline surveillance | BA.2 | GRA |
| hCoV-19/Tunisia/18507/2021 | EPI_ISL_10863108 | 02/03/2021 | Africa / Tunisia / Sbitla | Human | Baseline surveillance | Male | 70 | unknown | unknown | Baseline surveillance | B.1.1.7 | GRY |
| hCoV-19/Tunisia/18506/2021 | EPI_ISL_10863107 | 20/03/2021 | Africa / Tunisia / Sbitla | Human | Baseline surveillance | Female | 81 | unknown | unknown | Baseline surveillance | B.1.1.7 | GRY |
| hCoV-19/Tunisia/19286/2021 | EPI_ISL_10863106 | 27/02/2021 | Africa / Tunisia / Sidi Bouzid | Human | Baseline surveillance | Female | 20 | unknown | unknown | Baseline surveillance | B.1.177 | GV |
| hCoV-19/Tunisia/12341/2021 | EPI_ISL_10863105 | 30/01/2021 | Africa / Tunisia / Sousse | Human | Baseline surveillance | Male | 51 | unknown | unknown | Baseline surveillance | B.1.160 | GH |
| hCoV-19/Tunisia/19277/2021 | EPI_ISL_10863104 | 01/03/2021 | Africa / Tunisia / Tataouine | Human | Baseline surveillance | Female | 34 | unknown | unknown | Baseline surveillance | B.1 | GH |
| hCoV-19/Tunisia/5613/2021 | EPI_ISL_10863103 | 24/01/2021 | Africa / Tunisia / Tunis | Human | Baseline surveillance | Male | 22 | unknown | unknown | Baseline surveillance | B.1.428.2 | GH |
| hCoV-19/Tunisia/4044/2021 | EPI_ISL_10863102 | 12/01/2021 | Africa / Tunisia / Tunis | Human | Baseline surveillance | Male | 59 | unknown | unknown | Baseline surveillance | B.1.160 | GH |
| hCoV-19/Tunisia/19152/2021 | EPI_ISL_10863101 | 07/02/2021 | Africa / Tunisia / Tunis | Human | Baseline surveillance | Male | 24 | unknown | unknown | Baseline surveillance | B.1.1.7 | GRY |
| hCoV-19/Tunisia/50753/2020 | EPI_ISL_10863100 | 17/10/2020 | Africa / Tunisia / Tunis | Human | Baseline surveillance | Female | 43 | unknown | unknown | Baseline surveillance | B.1.9 | GH |
| hCoV-19/Tunisia/47035/2021 | EPI_ISL_10863099 | 09/07/2021 | Africa / Tunisia / Tunis | Human | Baseline surveillance | Female | 35 | unknown | unknown | Baseline surveillance | AY.122 | GK |
| hCoV-19/Tunisia/46651/2020 | EPI_ISL_10863098 | 16/10/2020 | Africa / Tunisia / Tunis | Human | Baseline surveillance | Female | 35 | unknown | unknown | Baseline surveillance | B.1.160 | GH |
| hCoV-19/Tunisia/40440/2021 | EPI_ISL_10863097 | 15/06/2021 | Africa / Tunisia / Tunis | Human | Baseline surveillance | Male | 30 | unknown | unknown | Baseline surveillance | AY.122 | GK |
| hCoV-19/Tunisia/39292/2021 | EPI_ISL_10863096 | 10/06/2021 | Africa / Tunisia / Tunis | Human | Baseline surveillance | Female | 46 | unknown | unknown | Baseline surveillance | AY.122 | GK |
| hCoV-19/Tunisia/35750/2021 | EPI_ISL_10863095 | 26/05/2021 | Africa / Tunisia / Tunis | Human | Baseline surveillance | Female | 41 | unknown | unknown | Baseline surveillance | AY.122 | GK |
| hCoV-19/Tunisia/19501/2021 | EPI_ISL_10863094 | 06/03/2021 | Africa / Tunisia / Tunis | Human | Baseline surveillance | Female | 33 | unknown | unknown | Baseline surveillance | C.17 | GR |
| hCoV-19/Tunisia/8100/2022 | EPI_ISL_10863093 | 07/02/2022 | Africa / Tunisia / Tunis | Human | Baseline surveillance | Female | 74 | unknown | unknown | Baseline surveillance | BA.1.1 | GRA |
| hCoV-19/Tunisia/4051/2021 | EPI_ISL_10863092 | 14/01/2021 | Africa / Tunisia / Zaghouane | Human | Baseline surveillance | Female | 39 | unknown | unknown | Baseline surveillance | B.1.177 | G |
| hCoV-19/Tunisia/5610/2020 | EPI_ISL_10863091 | 26/10/2020 | Africa / Tunisia / Zaghouane | Human | Baseline surveillance | Female | 39 | unknown | unknown | Baseline surveillance | B.1.160 | GH |
| hCoV-19/Tunisia/S-0646/2021 | EPI_ISL_10141427 | 25/06/2021 | Africa / Tunisia / Kairouen | Human | unknown | Female | 26 | unknown | No | unknown | B.1 | G |
| hCoV-19/Tunisia/Y-9538/2021 | EPI_ISL_10141386 | 31/03/2021 | Africa / Tunisia / Tunis | Human | unknown | Female | 25 | Hospitalized | No | unknown | B.1.177 | GV |
| hCoV-19/Tunisia/E-1720/2021 | EPI_ISL_10141552 | 27/06/2021 | Africa / Tunisia / Tunis | Human | unknown | Female | 29 | unknown | No | unknown | AY.122 | GK |
| hCoV-19/Tunisia/U-9630/2021 | EPI_ISL_10141551 | 05/07/2021 | Africa / Tunisia / Tunis | Human | unknown | Female | 16 | unknown | No | unknown | AY.122 | GK |
| hCoV-19/Tunisia/E-1674/2021 | EPI_ISL_10141550 | 27/06/2021 | Africa / Tunisia / Tunis | Human | unknown | Female | 48 | unknown | No | unknown | AY.122 | GK |
| hCoV-19/Tunisia/S-0782/2021 | EPI_ISL_10141549 | 30/06/2021 | Africa / Tunisia / Zaghouen | Human | unknown | Female | 42 | unknown | No | unknown | B.1.1.7 | GRY |
| hCoV-19/Tunisia/A-9048/2020 | EPI_ISL_10141548 | 24/08/2020 | Africa / Tunisia / Ariana | Human | unknown | Male | 33 | unknown | No | unknown | B.1.597 | GH |
| hCoV-19/Tunisia/S-0783/2021 | EPI_ISL_10141547 | 30/06/2021 | Africa / Tunisia / Tunis | Human | unknown | Male | 76 | unknown | No | unknown | B.1.1.7 | GRY |
| hCoV-19/Tunisia/E-1712/2021 | EPI_ISL_10141544 | 27/06/2021 | Africa / Tunisia / Tunis | Human | unknown | Male | 46 | unknown | No | unknown | AY.122 | GK |
| hCoV-19/Tunisia/S-0709/2021 | EPI_ISL_10141543 | 29/06/2021 | Africa / Tunisia / Kairouen | Human | unknown | Male | unknown | unknown | No | unknown | AY.122 | GK |
| hCoV-19/Tunisia/E-1738/2021 | EPI_ISL_10141542 | 27/06/2021 | Africa / Tunisia / Tunis | Human | unknown | unknown | unknown | unknown | No | unknown | AY.122 | GK |
| hCoV-19/Tunisia/C-5764/2021 | EPI_ISL_10141541 | 25/04/2021 | Africa / Tunisia / Tunis | Human | unknown | Male | 72 | Hospitalized | No | unknown | B.1.1.7 | GRY |
| hCoV-19/Tunisia/C-5758/2021 | EPI_ISL_10141540 | 26/04/2021 | Africa / Tunisia / Ariana | Human | unknown | Female | 52 | Severe | No | unknown | B.1.1.7 | GRY |
| hCoV-19/Tunisia/S-0705/2021 | EPI_ISL_10141539 | 29/06/2021 | Africa / Tunisia / Kairouen | Human | unknown | Female | 34 | unknown | No | unknown | AY.122 | GK |
| hCoV-19/Tunisia/A-9172/2020 | EPI_ISL_10141538 | 24/08/2020 | Africa / Tunisia / Jendouba | Human | unknown | Female | 21 | unknown | No | unknown | B.1.1 | GR |
| hCoV-19/Tunisia/S-0763/2021 | EPI_ISL_10141537 | 29/06/2021 | Africa / Tunisia / Medenine | Human | unknown | unknown | 49 | unknown | No | unknown | B.1.525 | G |
| hCoV-19/Tunisia/U-6964/2021 | EPI_ISL_10141536 | 04/05/2021 | Africa / Tunisia / Tunis | Human | unknown | Male | 22 | unknown | No | unknown | B.1.1.7 | GRY |
| hCoV-19/Tunisia/C-2861/2021 | EPI_ISL_10141534 | 13/04/2021 | Africa / Tunisia / Tunis | Human | unknown | Female | 50 | Hospitalized | No | unknown | B.1.1.7 | GRY |
| hCoV-19/Tunisia/C-5804/2021 | EPI_ISL_10141532 | 26/04/2021 | Africa / Tunisia / Ariana | Human | unknown | Female | 29 | Mild | No | unknown | B.1.1.7 | GRY |
| hCoV-19/Tunisia/C-2409/2021 | EPI_ISL_10141531 | 10/04/2021 | Africa / Tunisia / Tunis | Human | unknown | Male | 72 | Severe | No | unknown | B.1.1.7 | GRY |
| hCoV-19/Tunisia/S-0802/2021 | EPI_ISL_10141530 | 01/07/2021 | Africa / Tunisia / Kairouen | Human | unknown | Female | 29 | unknown | No | unknown | AY.122 | GK |
| hCoV-19/Tunisia/C-131/2021 | EPI_ISL_10141529 | 02/04/2021 | Africa / Tunisia / Tunis | Human | unknown | Male | 63 | Hospitalized | No | unknown | B.1.1.7 | GRY |
| hCoV-19/Tunisia/LP1-2247/2020 | EPI_ISL_10141528 | 29/07/2020 | Africa / Tunisia / Tunis | Human | unknown | Female | 51 | unknown | No | unknown | B.1.1.50 | GR |
| hCoV-19/Tunisia/C-110/2021 | EPI_ISL_10141526 | 02/04/2021 | Africa / Tunisia / Silina | Human | unknown | Female | 87 | Mild | No | unknown | B.1.2 | GH |
| hCoV-19/Tunisia/C-4735/2021 | EPI_ISL_10141525 | 20/04/2021 | Africa / Tunisia / Tunis | Human | unknown | Female | 58 | Hospitalized | No | unknown | B.1.1.7 | GRY |
| hCoV-19/Tunisia/C-4751/2021 | EPI_ISL_10141524 | 21/04/2021 | Africa / Tunisia / Ariana | Human | unknown | Male | 76 | Mild | No | unknown | B.1.1.7 | GRY |
| hCoV-19/Tunisia/U-6828/2021 | EPI_ISL_10141523 | 29/04/2021 | Africa / Tunisia / Tunis | Human | unknown | Female | 67 | Asymptomatic | No | unknown | B.1.1.7 | GRY |
| hCoV-19/Tunisia/C-2835/2021 | EPI_ISL_10141522 | 12/04/2021 | Africa / Tunisia / Tunis | Human | unknown | Female | 59 | Severe | No | unknown | B.1.1.7 | GRY |
| hCoV-19/Tunisia/C-3393/2021 | EPI_ISL_10141521 | 14/04/2021 | Africa / Tunisia / Tunis | Human | unknown | Female | 63 | Severe | No | unknown | B.1.1.7 | GRY |
| hCoV-19/Tunisia/C-5531/2021 | EPI_ISL_10141520 | 23/04/2021 | Africa / Tunisia / Tunis | Human | unknown | Male | 50 | Severe | No | unknown | B.1.1.7 | GRY |
| hCoV-19/Tunisia/C-3648/2021 | EPI_ISL_10141513 | 16/04/2021 | Africa / Tunisia / Tunis | Human | unknown | Female | 58 | Hospitalized | No | unknown | B.1.1.7 | GRY |
| hCoV-19/Tunisia/C-4469/2021 | EPI_ISL_10141512 | 21/04/2021 | Africa / Tunisia / Ben Arous | Human | unknown | Male | 14 | Mild | No | unknown | A.27 | S |
| hCoV-19/Tunisia/C-5126/2021 | EPI_ISL_10141509 | 22/04/2021 | Africa / Tunisia / Ben Arous | Human | unknown | Male | 72 | Hospitalized | No | unknown | B.1.1.7 | GRY |
| hCoV-19/Tunisia/C-4734/2021 | EPI_ISL_10141508 | 20/04/2021 | Africa / Tunisia / Ariana | Human | unknown | Female | 63 | Hospitalized | No | unknown | B.1.1.7 | GRY |
| hCoV-19/Tunisia/C-3796/2021 | EPI_ISL_10141506 | 15/04/2021 | Africa / Tunisia / Ariana | Human | unknown | Male | 62 | Mild | No | unknown | B.1.1.7 | GRY |
| hCoV-19/Tunisia/E-527/2021 | EPI_ISL_10141504 | 24/06/2021 | Africa / Tunisia / Tunis | Human | unknown | Female | 32 | unknown | No | unknown | AY.122 | GK |
| hCoV-19/Tunisia/C-2974/2021 | EPI_ISL_10141503 | 13/04/2021 | Africa / Tunisia / Ariana | Human | unknown | Male | 77 | Hospitalized | No | unknown | B.1.1.7 | GRY |
| hCoV-19/Tunisia/A-9469/2020 | EPI_ISL_10141502 | 26/08/2020 | Africa / Tunisia / Jendouba | Human | unknown | Female | unknown | unknown | No | unknown | B.1.1.25 | GR |
| hCoV-19/Tunisia/A-9942/2020 | EPI_ISL_10141501 | 29/08/2020 | Africa / Tunisia / Jendouba | Human | unknown | Female | 60 | unknown | No | unknown | B.1.1.25 | GR |
| hCoV-19/Tunisia/U-6310/2021 | EPI_ISL_10141500 | 20/04/2021 | Africa / Tunisia / Ben Arous | Human | unknown | Male | 68 | unknown | No | unknown | B.1.351.2 | GH |
| hCoV-19/Tunisia/X-3209/2020 | EPI_ISL_10141499 | 30/08/2020 | Africa / Tunisia / Ariana | Human | unknown | Male | 41 | unknown | No | unknown | B.1.1.1 | GR |
| hCoV-19/Tunisia/C-5759/2021 | EPI_ISL_10141498 | 26/04/2021 | Africa / Tunisia / Tunis | Human | unknown | Male | 44 | Severe | No | unknown | B.1.1.7 | GRY |
| hCoV-19/Tunisia/C-5769/2021 | EPI_ISL_10141495 | 25/04/2021 | Africa / Tunisia / Tunis | Human | unknown | Female | unknown | Mild | No | unknown | B.1.1.7 | GRY |
| hCoV-19/Tunisia/C-4333/2021 | EPI_ISL_10141494 | 17/04/2021 | Africa / Tunisia / Tunis | Human | unknown | Female | 82 | Severe | No | unknown | B.1.1.7 | GRY |
| hCoV-19/Tunisia/C-3638/2021 | EPI_ISL_10141493 | 15/04/2021 | Africa / Tunisia / Tunis | Human | unknown | Female | 71 | Hospitalized | No | unknown | B.1.1.7 | GRY |
| hCoV-19/Tunisia/C-5114/2021 | EPI_ISL_10141492 | 22/04/2021 | Africa / Tunisia / Tunis | Human | unknown | Female | 69 | Hospitalized | No | unknown | B.1.1.7 | GRY |
| hCoV-19/Tunisia/U-6871/2021 | EPI_ISL_10141491 | 30/04/2021 | Africa / Tunisia / Rades | Human | unknown | Male | 63 | Asymptomatic | No | unknown | B.1.1.7 | GRY |
| hCoV-19/Tunisia/U-6355/2021 | EPI_ISL_10141490 | 21/04/2021 | Africa / Tunisia / Ben Arous | Human | unknown | Female | 62 | Asymptomatic | No | unknown | B.1.1.7 | GRY |
| hCoV-19/Tunisia/C-3227/2021 | EPI_ISL_10141487 | 14/04/2021 | Africa / Tunisia / Tunis | Human | unknown | Male | 57 | Hospitalized | No | unknown | B.1.1.7 | GRY |
| hCoV-19/Tunisia/C-3256/2021 | EPI_ISL_10141485 | 13/04/2021 | Africa / Tunisia / Sidi Bouzid | Human | unknown | Female | 55 | Mild | No | unknown | B.1.1.7 | GRY |
| hCoV-19/Tunisia/C-3251/2021 | EPI_ISL_10141484 | 12/04/2021 | Africa / Tunisia / Sidi Bouzid | Human | unknown | Female | 51 | Mild | No | unknown | B.1.1.7 | GRY |
| hCoV-19/Tunisia/C-3224/2021 | EPI_ISL_10141480 | 14/04/2021 | Africa / Tunisia / Tunis | Human | unknown | Male | 47 | Hospitalized | No | unknown | B.1.1.7 | GRY |
| hCoV-19/Tunisia/C-5761/2021 | EPI_ISL_10141477 | 26/04/2021 | Africa / Tunisia / Ariana | Human | unknown | Male | 34 | Severe | No | unknown | B.1.1.7 | GRY |
| hCoV-19/Tunisia/B-5495/2021 | EPI_ISL_10141476 | 30/04/2021 | Africa / Tunisia / Ben Arous | Human | unknown | Female | 28 | Hospitalized | No | unknown | B.1.1.7 | GRY |
| hCoV-19/Tunisia/C-3273/2021 | EPI_ISL_10141475 | 13/04/2021 | Africa / Tunisia / Sidi Bouzid | Human | unknown | Female | 21 | Mild | No | unknown | B.1.1.7 | GRY |
| hCoV-19/Tunisia/C-3223/2021 | EPI_ISL_10141473 | 14/04/2021 | Africa / Tunisia / Tunis | Human | unknown | Female | 86 | Hospitalized | No | unknown | B.1.1.7 | GRY |
| hCoV-19/Tunisia/C-4563/2021 | EPI_ISL_10141472 | 21/04/2021 | Africa / Tunisia / Mannouba | Human | unknown | Male | 74 | Hospitalized | No | unknown | B.1.1.7 | GRY |
| hCoV-19/Tunisia/B-5488/2021 | EPI_ISL_10141471 | 30/04/2021 | Africa / Tunisia / Ben Arous | Human | unknown | Male | 69 | Mild | No | unknown | B.1.1.7 | GRY |
| hCoV-19/Tunisia/S-0201/2021 | EPI_ISL_10141470 | 07/04/2021 | Africa / Tunisia / Sousse | Human | unknown | Male | 39 | unknown | No | unknown | B.1.1.7 | GRY |
| hCoV-19/Tunisia/C-4450/2021 | EPI_ISL_10141466 | 20/04/2021 | Africa / Tunisia / Tunis | Human | unknown | Male | 22 | Mild | No | unknown | B.1.1.7 | GRY |
| hCoV-19/Tunisia/C-4109/2021 | EPI_ISL_10141465 | 19/04/2021 | Africa / Tunisia / Ariana | Human | unknown | Female | 26 | Mild | No | unknown | B.1.1.318 | GR |
| hCoV-19/Tunisia/C-3650/2021 | EPI_ISL_10141464 | 16/04/2021 | Africa / Tunisia / Ben Arous | Human | unknown | Female | 59 | Hospitalized | No | unknown | B.1.1.7 | GRY |
| hCoV-19/Tunisia/E-1725/2021 | EPI_ISL_10141463 | 27/06/2021 | Africa / Tunisia / Tunis | Human | unknown | Male | unknown | unknown | No | unknown | AY.122 (consensus call) | GK |
| hCoV-19/Tunisia/S-0804/2021 | EPI_ISL_10141460 | 01/07/2021 | Africa / Tunisia / Kairouen | Human | unknown | Female | 46 | unknown | No | unknown | B.1.617.2 | GK |
| hCoV-19/Tunisia/E-1332/2021 | EPI_ISL_10141451 | 26/06/2021 | Africa / Tunisia / Tunis | Human | unknown | Female | 82 | unknown | No | unknown | AY.122 | GK |
| hCoV-19/Tunisia/E-1634/2021 | EPI_ISL_10141450 | 27/06/2021 | Africa / Tunisia / Tunis | Human | unknown | Female | 61 | unknown | No | unknown | AY.122 | GK |
| hCoV-19/Tunisia/E-1359/2021 | EPI_ISL_10141448 | 26/06/2021 | Africa / Tunisia / Tunis | Human | unknown | Male | 23 | unknown | No | unknown | AY.122 | GK |
| hCoV-19/Tunisia/S-0702/2021 | EPI_ISL_10141447 | 29/06/2021 | Africa / Tunisia / Kairouen | Human | unknown | Male | 77 | unknown | No | unknown | AY.122 | GK |
| hCoV-19/Tunisia/E-1663/2021 | EPI_ISL_10141440 | 27/06/2021 | Africa / Tunisia / Tunis | Human | unknown | unknown | 24 | unknown | No | unknown | AY.122 | GK |
| hCoV-19/Tunisia/C-4330/2021 | EPI_ISL_10141439 | 17/04/2021 | Africa / Tunisia / Tunis | Human | unknown | Female | 20 | Mild | No | unknown | Unassigned | GR |
| hCoV-19/Tunisia/S-0849/2021 | EPI_ISL_10141438 | 05/07/2021 | Africa / Tunisia / Sousse | Human | unknown | Male | 49 | unknown | No | unknown | Unassigned | G |
| hCoV-19/Tunisia/S-0877/2021 | EPI_ISL_10141437 | 09/07/2021 | Africa / Tunisia / Gafsa | Human | unknown | Male | 33 | unknown | No | unknown | Unassigned | GR |
| hCoV-19/Tunisia/E-1414/2021 | EPI_ISL_10141436 | 26/06/2021 | Africa / Tunisia / Tunis | Human | unknown | Female | 23 | unknown | No | unknown | AY.122 (consensus call) | GK |
[truncated: 25,518 more chars]
