# Supplementary material for: Genomic surveillance of SARS-CoV-2 in North Africa: 4 years of GISAID data sharing
Source: IJID Reg. 2024 Mar 19;11:100356. doi: 10.1016/j.ijregi.2024.100356 (PMC11035039; doi:10.1016/j.ijregi.2024.100356)
Supplement: Supplementary file 5 [file mmc5.docx]

Table S4. Comprehensive Virus Data from Algeria Including Virus Name, Accession Number, and Clinical Attributes (based on data downloaded from GISAID per 15 September 2023)

| Virus name | Accession ID | Collection date | Location | Host | Sampling strategy | Gender | Patient age (years) | Patient status | Last vaccinated | Sampling strategy | Lineage | Clade |
| --- | --- | --- | --- | --- | --- | --- | --- | --- | --- | --- | --- | --- |
| hCoV-19/Algeria/12463/2022 | EPI_ISL_10588933 | 30/01/2022 | Africa / Algeria / Laghouat | Human | unknown | Female | 46 | unknown | unknown | unknown | BA.2 | GRA |
| hCoV-19/Algeria/12467/2022 | EPI_ISL_10588934 | 30/01/2022 | Africa / Algeria / Laghouat | Human | unknown | Male | 25 | unknown | unknown | unknown | BA.2 | GRA |
| hCoV-19/Algeria/12571/2022 | EPI_ISL_10588935 | 30/01/2022 | Africa / Algeria / Bouira | Human | unknown | Male | 31 | unknown | unknown | unknown | BA.2 | GRA |
| hCoV-19/Algeria/12601/2022 | EPI_ISL_10588936 | 30/01/2022 | Africa / Algeria / Bouira | Human | unknown | Female | 84 | unknown | unknown | unknown | BA.2 | GRA |
| hCoV-19/Algeria/12606/2022 | EPI_ISL_10588937 | 30/01/2022 | Africa / Algeria / Laghouat | Human | unknown | Female | 47 | unknown | unknown | unknown | BA.2 | GRA |
| hCoV-19/Algeria/12614/2022 | EPI_ISL_10588938 | 30/01/2022 | Africa / Algeria / Bouira | Human | unknown | Male | 93 | unknown | unknown | unknown | BA.2 | GRA |
| hCoV-19/Algeria/12654/2022 | EPI_ISL_10588939 | 31/01/2022 | Africa / Algeria / Algiers | Human | unknown | Female | 61 | unknown | unknown | unknown | BA.2 | GRA |
| hCoV-19/Algeria/12662/2022 | EPI_ISL_10588940 | 31/01/2022 | Africa / Algeria / Algiers | Human | unknown | Female | 1 | unknown | unknown | unknown | BA.2 | GRA |
| hCoV-19/Algeria/12664/2022 | EPI_ISL_10588941 | 31/01/2022 | Africa / Algeria / Algiers | Human | unknown | Female | 88 | unknown | unknown | unknown | BA.2 | GRA |
| hCoV-19/Algeria/12679/2022 | EPI_ISL_10588942 | 31/01/2022 | Africa / Algeria / Algiers | Human | unknown | Male | 46 | unknown | unknown | unknown | BA.2 | GRA |
| hCoV-19/Algeria/12689/2022 | EPI_ISL_10588943 | 31/01/2022 | Africa / Algeria / Algiers | Human | unknown | Male | 29 | unknown | unknown | unknown | BA.2 | GRA |
| hCoV-19/Algeria/12608/2022 | EPI_ISL_10640211 | 30/01/2022 | Africa / Algeria / Bouira | Human | unknown | Female | 58 | unknown | unknown | unknown | BA.2 | GRA |
| hCoV-19/Algeria/12955/2022 | EPI_ISL_10752628 | 01/02/2022 | Africa / Algeria / Laghouat | Human | unknown | Male | 41 | unknown | unknown | unknown | BA.2 | GRA |
| hCoV-19/Algeria/13637/2022 | EPI_ISL_10752629 | 03/02/2022 | Africa / Algeria / Laghouat | Human | unknown | Male | 87 | unknown | unknown | unknown | B.1.617.2 | GK |
| hCoV-19/Algeria/13777/2022 | EPI_ISL_10752630 | 05/02/2022 | Africa / Algeria / Bouira | Human | unknown | Male | 72 | unknown | unknown | unknown | BA.2 | GRA |
| hCoV-19/Algeria/13780/2022 | EPI_ISL_10752631 | 05/02/2022 | Africa / Algeria / Bouira | Human | unknown | Female | 38 | unknown | unknown | unknown | B.1.617.2 | GK |
| hCoV-19/Algeria/13809/2022 | EPI_ISL_10752632 | 05/02/2022 | Africa / Algeria / Laghouat | Human | unknown | Male | 67 | unknown | unknown | unknown | BA.2 | GRA |
| hCoV-19/Algeria/13820/2022 | EPI_ISL_10752633 | 05/02/2022 | Africa / Algeria / Laghouat | Human | unknown | Male | 57 | unknown | unknown | unknown | BA.2 | GRA |
| hCoV-19/Algeria/13816/2022 | EPI_ISL_10752634 | 05/02/2022 | Africa / Algeria / Laghouat | Human | unknown | Male | 54 | unknown | unknown | unknown | BA.2 | GRA |
| hCoV-19/Algeria/13828/2022 | EPI_ISL_10752635 | 05/02/2022 | Africa / Algeria / Laghouat | Human | unknown | Female | 62 | unknown | unknown | unknown | BA.2 | GRA |
| hCoV-19/Algeria/14265/2022 | EPI_ISL_10752636 | 07/02/2022 | Africa / Algeria / Blida | Human | unknown | Male | 62 | unknown | unknown | unknown | BA.1.1 | GRA |
| hCoV-19/Algeria/14387/2022 | EPI_ISL_10752637 | 07/02/2022 | Africa / Algeria / Bouira | Human | unknown | Female | 84 | unknown | unknown | unknown | BA.2 | GRA |
| hCoV-19/Algeria/15053/2022 | EPI_ISL_10752638 | 10/02/2022 | Africa / Algeria / Algiers | Human | unknown | Male | 79 | unknown | unknown | unknown | BA.2 | GRA |
| hCoV-19/Algeria/13447-44FR/2021 | EPI_ISL_1093427 | 11/02/2021 | Africa / Algeria / Algiers | Human | unknown | Female | 43 | Live | unknown | unknown | Unassigned | O |
| hCoV-19/Algeria/13447-47FR/2021 | EPI_ISL_1093428 | 11/02/2021 | Africa / Algeria / Algiers | Human | unknown | Female | 43 | Live | unknown | unknown | Unassigned | G |
| hCoV-19/Algeria/13442-44FR/2021 | EPI_ISL_1093429 | 11/02/2021 | Africa / Algeria / Algiers | Human | unknown | Female | 30 | unknown | unknown | unknown | Unassigned | O |
| hCoV-19/Algeria/13442-47FR/2021 | EPI_ISL_1093430 | 11/02/2021 | Africa / Algeria / Algiers | Human | unknown | Female | 30 | unknown | unknown | unknown | Unassigned | G |
| hCoV-19/Algeria/98101/2021 | EPI_ISL_10969510 | 21/12/2021 | Africa / Algeria / Algiers | Human | unknown | Male | 69 | unknown | unknown | unknown | BA.1.1 | GRA |
| hCoV-19/Algeria/97421/2021 | EPI_ISL_10969511 | 20/12/2021 | Africa / Algeria / Algiers | Human | unknown | Female | 23 | unknown | unknown | unknown | BA.1.18 | GRA |
| hCoV-19/Algeria/98321/2021 | EPI_ISL_10969512 | 21/12/2021 | Africa / Algeria / Algiers | Human | unknown | Male | 47 | unknown | unknown | unknown | BA.1.17.2 | GRA |
| hCoV-19/Algeria/96000/2021 | EPI_ISL_10969513 | 14/12/2021 | Africa / Algeria / Blida | Human | unknown | Male | 29 | unknown | unknown | unknown | B.1.617.2 | GK |
| hCoV-19/Algeria/96318/2021 | EPI_ISL_10969514 | 14/12/2021 | Africa / Algeria / Bouira | Human | unknown | Female | 45 | unknown | unknown | unknown | B.1.617.2 | GK |
| hCoV-19/Algeria/96449/2021 | EPI_ISL_10969515 | 15/12/2021 | Africa / Algeria / Algiers | Human | unknown | Female | 45 | unknown | unknown | unknown | B.1.617.2 | GK |
| hCoV-19/Algeria/96525/2021 | EPI_ISL_10969516 | 15/12/2021 | Africa / Algeria / Bouira | Human | unknown | Male | 48 | unknown | unknown | unknown | B.1.617.2 | GK |
| hCoV-19/Algeria/98051/2021 | EPI_ISL_10969517 | 21/12/2021 | Africa / Algeria / Algiers | Human | unknown | Male | 39 | unknown | unknown | unknown | B.1.617.2 | GK |
| hCoV-19/Algeria/95992/2021 | EPI_ISL_10969518 | 14/12/2021 | Africa / Algeria / Blida | Human | unknown | Female | 21 | unknown | unknown | unknown | B.1.617.2 | GK |
| hCoV-19/Algeria/96377/2021 | EPI_ISL_10969519 | 15/12/2021 | Africa / Algeria / Laghouat | Human | unknown | Male | 54 | unknown | unknown | unknown | B.1.617.2 | GK |
| hCoV-19/Algeria/I1/2021 | EPI_ISL_10969614 | 31/12/2021 | Africa / Algeria / Constantine | Human | unknown | Male | 50 | unknown | unknown | unknown | BA.1.1.1 | GRA |
| hCoV-19/Algeria/882/2022 | EPI_ISL_10969615 | 03/01/2022 | Africa / Algeria / Algiers | Human | unknown | Male | 42 | unknown | unknown | unknown | BA.1.1 | GRA |
| hCoV-19/Algeria/99781/2021 | EPI_ISL_10969616 | 26/12/2021 | Africa / Algeria / Algiers | Human | unknown | Male | unknown | unknown | unknown | unknown | BA.1 | GRA |
| hCoV-19/Algeria/I2/2021 | EPI_ISL_10969617 | 31/12/2021 | Africa / Algeria / Constantine | Human | unknown | Male | 32 | unknown | unknown | unknown | BA.1.18 | GRA |
| hCoV-19/Algeria/325/2022 | EPI_ISL_10969618 | 02/01/2022 | Africa / Algeria / Algiers | Human | unknown | Female | 37 | unknown | unknown | unknown | B.1.617.2 | GK |
| hCoV-19/Algeria/100322/2021 | EPI_ISL_10969619 | 29/12/2021 | Africa / Algeria / Algiers | Human | unknown | Male | 42 | unknown | unknown | unknown | B.1.617.2 | GK |
| hCoV-19/Algeria/100313/2021 | EPI_ISL_10969620 | 29/12/2021 | Africa / Algeria / Algiers | Human | unknown | Male | 74 | unknown | unknown | unknown | B.1.617.2 | GK |
| hCoV-19/Algeria/100308/2021 | EPI_ISL_10969621 | 29/12/2021 | Africa / Algeria / Algiers | Human | unknown | Female | 54 | unknown | unknown | unknown | B.1.617.2 | GK |
| hCoV-19/Algeria/100307/2021 | EPI_ISL_10969622 | 29/12/2021 | Africa / Algeria / Algiers | Human | unknown | Female | 76 | unknown | unknown | unknown | B.1.617.2 | GK |
| hCoV-19/Algeria/98751/2021 | EPI_ISL_10969623 | 26/12/2021 | Africa / Algeria / Algiers | Human | unknown | Male | 29 | unknown | unknown | unknown | BA.2 | GRA |
| hCoV-19/Algeria/99824/2021 | EPI_ISL_10969624 | 28/12/2021 | Africa / Algeria / Algiers | Human | unknown | Male | 68 | unknown | unknown | unknown | BA.1.18 | GRA |
| hCoV-19/Algeria/3897/2022 | EPI_ISL_10969625 | 13/01/2022 | Africa / Algeria / Algiers | Human | unknown | Male | 20 | unknown | unknown | unknown | BA.2 | GR |
| hCoV-19/Algeria/7Ibs/2022 | EPI_ISL_10969626 | 06/01/2022 | Africa / Algeria / Constantine | Human | unknown | Male | 62 | unknown | unknown | unknown | BA.1 | GRA |
| hCoV-19/Algeria/3319/2022 | EPI_ISL_10969627 | 11/01/2022 | Africa / Algeria / Algiers | Human | unknown | Female | 25 | unknown | unknown | unknown | BA.2 | GRA |
| hCoV-19/Algeria/3893/2022 | EPI_ISL_10969628 | 13/01/2022 | Africa / Algeria / Algiers | Human | unknown | Female | 48 | unknown | unknown | unknown | BA.2 | GRA |
| hCoV-19/Algeria/5471/2022 | EPI_ISL_10969629 | 17/01/2022 | Africa / Algeria / Blida | Human | unknown | Female | 41 | unknown | unknown | unknown | BA.1.1 | GRA |
| hCoV-19/Algeria/99297/2021 | EPI_ISL_10969630 | 27/12/2021 | Africa / Algeria / Medea | Human | unknown | Male | 78 | unknown | unknown | unknown | B.1.617.2 | GK |
| hCoV-19/Algeria/5Ibs/2022 | EPI_ISL_10969631 | 04/01/2022 | Africa / Algeria / Constantine | Human | unknown | Male | 37 | unknown | unknown | unknown | BA.1.17.2 | GRA |
| hCoV-19/Algeria/2663/2022 | EPI_ISL_10969632 | 10/01/2022 | Africa / Algeria / Algiers | Human | unknown | Male | 44 | unknown | unknown | unknown | BA.2 | GRA |
| hCoV-19/Algeria/99570/2021 | EPI_ISL_10969633 | 27/12/2021 | Africa / Algeria / Algiers | Human | unknown | Female | 51 | unknown | unknown | unknown | B.1.617.2 | GK |
| hCoV-19/Algeria/42V/2022 | EPI_ISL_10969634 | 06/01/2022 | Africa / Algeria / Algiers | Human | unknown | Female | 60 | unknown | unknown | unknown | BA.2 | GRA |
| hCoV-19/Algeria/3921/2022 | EPI_ISL_10969635 | 13/01/2022 | Africa / Algeria / Algiers | Human | unknown | Female | 22 | unknown | unknown | unknown | BA.2 | GRA |
| hCoV-19/Algeria/95448/2021 | EPI_ISL_10969636 | 11/12/2021 | Africa / Algeria / Algiers | Human | unknown | Female | 50 | unknown | unknown | unknown | BA.1.1 | GRA |
| hCoV-19/Algeria/17145/2022 | EPI_ISL_11290251 | 26/02/2022 | Africa / Algeria / Bouira | Human | unknown | Female | 67 | unknown | unknown | unknown | BA.2 | GRA |
| hCoV-19/Algeria/17245/2022 | EPI_ISL_11290252 | 27/02/2022 | Africa / Algeria / Algiers | Human | unknown | Female | 57 | unknown | unknown | unknown | BA.2 | GRA |
| hCoV-19/Algeria/17302/2022 | EPI_ISL_11290253 | 27/02/2022 | Africa / Algeria / Bouira | Human | unknown | Female | 32 | unknown | unknown | unknown | BA.2 | GRA |
| hCoV-19/Algeria/17305/2022 | EPI_ISL_11290254 | 27/02/2022 | Africa / Algeria / Bouira | Human | unknown | Female | 86 | unknown | unknown | unknown | BA.2 | GRA |
| hCoV-19/Algeria/17360/2022 | EPI_ISL_11290255 | 27/02/2022 | Africa / Algeria / Bouira | Human | unknown | Male | 79 | unknown | unknown | unknown | BA.2 | GRA |
| hCoV-19/Algeria/17368/2022 | EPI_ISL_11290256 | 27/02/2022 | Africa / Algeria / Bouira | Human | unknown | Female | 41 | unknown | unknown | unknown | BA.2 | GRA |
| hCoV-19/Algeria/17459/2022 | EPI_ISL_11290257 | 28/02/2022 | Africa / Algeria / Algiers | Human | unknown | Male | 49 | unknown | unknown | unknown | BA.2 | GRA |
| hCoV-19/Algeria/17575/2022 | EPI_ISL_11290258 | 28/02/2022 | Africa / Algeria / Algiers | Human | unknown | Male | 45 | unknown | unknown | unknown | BA.1.1 | GRA |
| hCoV-19/Algeria/17760/2022 | EPI_ISL_11290259 | 01/03/2022 | Africa / Algeria / Algiers | Human | unknown | Female | 10 | unknown | unknown | unknown | BA.2 | GRA |
| hCoV-19/Algeria/16367/2022 | EPI_ISL_11437936 | 20/02/2022 | Africa / Algeria / Medea | Human | unknown | Male | 69 | unknown | unknown | unknown | BA.1.18 | GRA |
| hCoV-19/Algeria/16383/2022 | EPI_ISL_11437937 | 20/02/2022 | Africa / Algeria / Blida | Human | unknown | Female | 51 | unknown | unknown | unknown | BA.1.1 | GRA |
| hCoV-19/Algeria/16502/2022 | EPI_ISL_11437938 | 21/02/2022 | Africa / Algeria / Algiers | Human | unknown | Male | 77 | unknown | unknown | unknown | BA.1.1 | GRA |
| hCoV-19/Algeria/16509/2022 | EPI_ISL_11437939 | 21/02/2022 | Africa / Algeria / Blida | Human | unknown | Female | 42 | unknown | unknown | unknown | BA.1.1 | GRA |
| hCoV-19/Algeria/16577/2022 | EPI_ISL_11437940 | 21/02/2022 | Africa / Algeria / Algiers | Human | unknown | Male | 57 | unknown | unknown | unknown | BA.2 | GRA |
| hCoV-19/Algeria/16618/2022 | EPI_ISL_11437941 | 22/02/2022 | Africa / Algeria / Algiers | Human | unknown | Male | 43 | unknown | unknown | unknown | BA.2 | GRA |
| hCoV-19/Algeria/16733/2022 | EPI_ISL_11437942 | 22/02/2022 | Africa / Algeria / Medea | Human | unknown | Female | 32 | unknown | unknown | unknown | BA.2 | GRA |
| hCoV-19/Algeria/16351/2022 | EPI_ISL_11437943 | 19/02/2022 | Africa / Algeria / Djelfa | Human | unknown | Male | 81 | unknown | unknown | unknown | BA.2 | GRA |
| hCoV-19/Algeria/18684/2022 | EPI_ISL_11437944 | 09/03/2022 | Africa / Algeria / Algiers | Human | unknown | Male | 17 | unknown | unknown | unknown | BA.2 | GRA |
| hCoV-19/Algeria/18971/2022 | EPI_ISL_11437945 | 10/03/2022 | Africa / Algeria / Algiers | Human | unknown | Male | 55 | unknown | unknown | unknown | BA.2 | GRA |
| hCoV-19/Algeria/40468/2021 | EPI_ISL_11749578 | 25/05/2021 | Africa / Algeria / Algiers | Human | unknown | Female | 63 | unknown | unknown | unknown | B.1.1.7 | GR |
| hCoV-19/Algeria/38096/2021 | EPI_ISL_11749579 | 18/05/2021 | Africa / Algeria / Algiers | Human | unknown | Female | 59 | unknown | unknown | unknown | B.1.1.7 | GR |
| hCoV-19/Algeria/38122/2021 | EPI_ISL_11749580 | 18/05/2021 | Africa / Algeria / Algiers | Human | unknown | Female | 62 | unknown | unknown | unknown | B.1.1.7 | GR |
| hCoV-19/Algeria/7445/2021 | EPI_ISL_11749581 | 17/05/2021 | Africa / Algeria / Mascara | Human | unknown | Female | 37 | unknown | unknown | unknown | B.1.525 | G |
| hCoV-19/Algeria/7220/2021 | EPI_ISL_11749582 | 03/05/2021 | Africa / Algeria / Oran | Human | unknown | Male | 72 | unknown | unknown | unknown | B.1.620 | G |
| hCoV-19/Algeria/42551/2021 | EPI_ISL_11749583 | 01/06/2021 | Africa / Algeria / Bouira | Human | unknown | Female | 85 | unknown | unknown | unknown | B.1.525 | G |
| hCoV-19/Algeria/14564/2021 | EPI_ISL_11905496 | 17/02/2021 | Africa / Algeria / Algiers | Human | unknown | Female | 47 | unknown | unknown | unknown | B.1.1.7 | GRY |
| hCoV-19/Algeria/33699/2021 | EPI_ISL_11905497 | 02/05/2021 | Africa / Algeria / Bouira | Human | unknown | Male | 37 | unknown | unknown | unknown | B.1.1.7 | GRY |
| hCoV-19/Algeria/41388/2021 | EPI_ISL_11905498 | 27/05/2021 | Africa / Algeria / Algiers | Human | unknown | Male | 72 | unknown | unknown | unknown | B.1.1.7 | GRY |
| hCoV-19/Algeria/42269/2021 | EPI_ISL_11905499 | 31/05/2021 | Africa / Algeria / Algiers | Human | unknown | Male | 84 | unknown | unknown | unknown | B.1.1.7 | GRY |
| hCoV-19/Algeria/2023/2021 | EPI_ISL_11905500 | 2021-05 | Africa / Algeria / Ouargla | Human | unknown | Male | 33 | unknown | unknown | unknown | B.1.1.7 | GRY |
| hCoV-19/Algeria/7222/2021 | EPI_ISL_11905501 | 03/05/2021 | Africa / Algeria / Oran | Human | unknown | unknown | unknown | unknown | unknown | unknown | B.1.1.7 | GRY |
| hCoV-19/Algeria/35095/2021 | EPI_ISL_11905502 | 06/05/2021 | Africa / Algeria / Blida | Human | unknown | Female | 74 | unknown | unknown | unknown | B.1.1.7 | GRY |
| hCoV-19/Algeria/36069/2021 | EPI_ISL_11905503 | 10/05/2021 | Africa / Algeria / Algiers | Human | unknown | Female | 37 | unknown | unknown | unknown | B.1.1.7 | GRY |
| hCoV-19/Algeria/42119/2021 | EPI_ISL_11905504 | 31/05/2021 | Africa / Algeria / Tissemsilt | Human | unknown | Male | 83 | unknown | unknown | unknown | B.1.1.7 | GR |
| hCoV-19/Algeria/42210/2021 | EPI_ISL_11905505 | 31/05/2021 | Africa / Algeria / Algiers | Human | unknown | Male | 45 | unknown | unknown | unknown | B.1.525 | G |
| hCoV-19/Algeria/42234/2021 | EPI_ISL_11905506 | 31/05/2021 | Africa / Algeria / Algiers | Human | unknown | Male | 45 | unknown | unknown | unknown | B.1.617.2 | GK |
| hCoV-19/Algeria/42378/2021 | EPI_ISL_11905507 | 31/05/2021 | Africa / Algeria / Algiers | Human | unknown | Male | 55 | unknown | unknown | unknown | B.1.1.7 | GRY |
| hCoV-19/Algeria/42382/2021 | EPI_ISL_11905508 | 31/05/2021 | Africa / Algeria / Algiers | Human | unknown | Female | 34 | unknown | unknown | unknown | B.1.1.7 | GRY |
| hCoV-19/Algeria/40131/2021 | EPI_ISL_11926002 | 24/05/2021 | Africa / Algeria / Algiers | Human | unknown | Female | 50 | unknown | unknown | unknown | A.27 | S |
| hCoV-19/Algeria/40153/2021 | EPI_ISL_11926003 | 24/05/2021 | Africa / Algeria / Algiers | Human | unknown | Male | 56 | unknown | unknown | unknown | A.27 | S |
| hCoV-19/Algeria/30500/2021 | EPI_ISL_11926004 | 19/04/2021 | Africa / Algeria / Bouira | Human | unknown | Male | 55 | unknown | unknown | unknown | A.27 | S |
| hCoV-19/Algeria/30507/2021 | EPI_ISL_11926005 | 19/04/2021 | Africa / Algeria / Bouira | Human | unknown | Male | 81 | unknown | unknown | unknown | A.27 | S |
| hCoV-19/Algeria/32833/2021 | EPI_ISL_11926006 | 27/04/2021 | Africa / Algeria / Bouira | Human | unknown | Female | 58 | unknown | unknown | unknown | A.27 | S |
| hCoV-19/Algeria/16570/2022 | EPI_ISL_12042771 | 21/02/2022 | Africa / Algeria / Algiers | Human | unknown | Female | 75 | unknown | unknown | unknown | BA.1.1 | GRA |
| hCoV-19/Algeria/16580/2022 | EPI_ISL_12042772 | 21/02/2022 | Africa / Algeria / Medea | Human | unknown | Male | 81 | unknown | unknown | unknown | BA.1.1 | GRA |
| hCoV-19/Algeria/16606/2022 | EPI_ISL_12042773 | 21/02/2022 | Africa / Algeria / Algiers | Human | unknown | Female | 38 | unknown | unknown | unknown | BA.2 | GRA |
| hCoV-19/Algeria/17071/2022 | EPI_ISL_12042774 | 24/02/2022 | Africa / Algeria / Algiers | Human | unknown | Female | 29 | unknown | unknown | unknown | BA.2.58 | GRA |
| hCoV-19/Algeria/17072/2022 | EPI_ISL_12042775 | 24/02/2022 | Africa / Algeria / Algiers | Human | unknown | Male | 67 | unknown | unknown | unknown | BA.2 | GRA |
| hCoV-19/Algeria/17304/2022 | EPI_ISL_12042776 | 27/02/2022 | Africa / Algeria / Bouira | Human | unknown | Male | 67 | unknown | unknown | unknown | BA.1.18 | GRA |
| hCoV-19/Algeria/17365/2022 | EPI_ISL_12042777 | 27/02/2022 | Africa / Algeria / Bouira | Human | unknown | Male | 86 | unknown | unknown | unknown | BA.1.18 | GRA |
| hCoV-19/Algeria/18001/2022 | EPI_ISL_12042778 | 02/03/2022 | Africa / Algeria / Blida | Human | unknown | Male | 79 | unknown | unknown | unknown | BA.2 | GRA |
| hCoV-19/Algeria/18190/2022 | EPI_ISL_12042779 | 03/03/2022 | Africa / Algeria / Algiers | Human | unknown | Female | 33 | unknown | unknown | unknown | BA.2.23 | GRA |
| hCoV-19/Algeria/18245/2022 | EPI_ISL_12042780 | 04/03/2022 | Africa / Algeria / Algiers | Human | unknown | Male | unknown | unknown | unknown | unknown | BA.2 | GRA |
| hCoV-19/Algeria/13889/2022 | EPI_ISL_12043095 | 06/02/2022 | Africa / Algeria / Blida | Human | unknown | Male | 73 | unknown | unknown | unknown | BA.2 | GRA |
| hCoV-19/Algeria/13901/2022 | EPI_ISL_12043096 | 06/02/2022 | Africa / Algeria / Algiers | Human | unknown | Female | 30 | unknown | unknown | unknown | BA.2 | GRA |
| hCoV-19/Algeria/13918/2022 | EPI_ISL_12043097 | 06/02/2022 | Africa / Algeria / Algiers | Human | unknown | Female | 39 | unknown | unknown | unknown | BA.2 | GRA |
| hCoV-19/Algeria/14198/2022 | EPI_ISL_12043098 | 06/02/2022 | Africa / Algeria / Bouira | Human | unknown | Male | 54 | unknown | unknown | unknown | BA.2 | GRA |
| hCoV-19/Algeria/14250/2022 | EPI_ISL_12043099 | 07/02/2022 | Africa / Algeria / Algiers | Human | unknown | Male | 36 | unknown | unknown | unknown | BA.2 | GRA |
| hCoV-19/Algeria/14400/2022 | EPI_ISL_12043100 | 07/02/2022 | Africa / Algeria / Medea | Human | unknown | Female | 86 | unknown | unknown | unknown | BA.2 | GRA |
| hCoV-19/Algeria/15105/2022 | EPI_ISL_12043101 | 10/02/2022 | Africa / Algeria / Algiers | Human | unknown | Female | 34 | unknown | unknown | unknown | BA.2 | GRA |
| hCoV-19/Algeria/55951/2021 | EPI_ISL_12156705 | 10/07/2021 | Africa / Algeria / Algiers | Human | unknown | Female | 34 | unknown | unknown | unknown | B.1.617.2 | GK |
| hCoV-19/Algeria/66067/2021 | EPI_ISL_12156706 | 29/07/2021 | Africa / Algeria / El Oued | Human | unknown | Female | 86 | unknown | unknown | unknown | B.1.617.2 | GK |
| hCoV-19/Algeria/65951/2021 | EPI_ISL_12156707 | 30/07/2021 | Africa / Algeria / Bouira | Human | unknown | Male | 63 | unknown | unknown | unknown | B.1.617.2 | GK |
| hCoV-19/Algeria/65936/2021 | EPI_ISL_12156708 | 29/07/2021 | Africa / Algeria / Bouira | Human | unknown | Female | 87 | unknown | unknown | unknown | B.1.617.2 | GK |
| hCoV-19/Algeria/65926/2021 | EPI_ISL_12156709 | 29/07/2021 | Africa / Algeria / Bouira | Human | unknown | Male | 43 | unknown | unknown | unknown | B.1.617.2 | GK |
| hCoV-19/Algeria/65837/2021 | EPI_ISL_12156710 | 01/08/2021 | Africa / Algeria / Blida | Human | unknown | Male | 50 | unknown | unknown | unknown | B.1.1.7 | G |
| hCoV-19/Algeria/65534/2021 | EPI_ISL_12156711 | 01/08/2021 | Africa / Algeria / Algiers | Human | unknown | Female | 38 | unknown | unknown | unknown | B.1.617.2 | GK |
| hCoV-19/Algeria/65367/2021 | EPI_ISL_12156712 | 29/07/2021 | Africa / Algeria / Algiers | Human | unknown | Male | 8 | unknown | unknown | unknown | B.1.617.2 | GK |
| hCoV-19/Algeria/77518/2021 | EPI_ISL_12156713 | 06/09/2021 | Africa / Algeria / Algiers | Human | unknown | Male | 88 | unknown | unknown | unknown | B.1.617.2 | GK |
| hCoV-19/Algeria/76866/2021 | EPI_ISL_12156714 | 03/09/2021 | Africa / Algeria / Algiers | Human | unknown | Male | 90 | unknown | unknown | unknown | B.1.617.2 | GK |
| hCoV-19/Algeria/76454/2021 | EPI_ISL_12156715 | 02/09/2021 | Africa / Algeria / Algiers | Human | unknown | Male | 32 | unknown | unknown | unknown | B.1.617.2 | GK |
| hCoV-19/Algeria/76838/2021 | EPI_ISL_12156716 | 04/09/2021 | Africa / Algeria / Algiers | Human | unknown | Female | 67 | unknown | unknown | unknown | B.1.617.2 | GK |
| hCoV-19/Algeria/66171/2021 | EPI_ISL_12156717 | 01/08/2021 | Africa / Algeria / Algiers | Human | unknown | Female | 88 | unknown | unknown | unknown | B.1.617.2 | GK |
| hCoV-19/Algeria/34044/2021 | EPI_ISL_12156718 | 03/05/2021 | Africa / Algeria / Algiers | Human | unknown | Female | 34 | unknown | unknown | unknown | B.1.1.7 | G |
| hCoV-19/Algeria/126814/2020 | EPI_ISL_12156719 | 19/11/2020 | Africa / Algeria / Algiers | Human | unknown | Male | 72 | unknown | unknown | unknown | B.1 | GH |
| hCoV-19/Algeria/55229/2021 | EPI_ISL_12156720 | 08/07/2021 | Africa / Algeria / Blida | Human | unknown | Female | 43 | unknown | unknown | unknown | B.1.617.2 | GK |
| hCoV-19/Algeria/33352/2021 | EPI_ISL_12156721 | 28/04/2021 | Africa / Algeria / Algiers | Human | unknown | Female | 52 | unknown | unknown | unknown | B.1.1.7 | G |
| hCoV-19/Algeria/122556/2020 | EPI_ISL_12156722 | 14/11/2020 | Africa / Algeria / Bouira | Human | unknown | Male | 80 | unknown | unknown | unknown | B.1 | G |
| hCoV-19/Algeria/11814/2020 | EPI_ISL_12156725 | 02/05/2020 | Africa / Algeria / Medea | Human | unknown | Female | 82 | unknown | unknown | unknown | B.1 | GH |
| hCoV-19/Algeria/12031/2020 | EPI_ISL_12156726 | 16/05/2020 | Africa / Algeria / Blida | Human | unknown | Female | 32 | unknown | unknown | unknown | B.1.597 | GH |
| hCoV-19/Algeria/23675/2020 | EPI_ISL_12156727 | 28/05/2020 | Africa / Algeria / Tiaret | Human | unknown | Female | 35 | unknown | unknown | unknown | B.1 | GK |
| hCoV-19/Algeria/90959/2020 | EPI_ISL_12156728 | 19/09/2020 | Africa / Algeria / Algiers | Human | unknown | Female | 20 | unknown | unknown | unknown | B.1.1 | GR |
| hCoV-19/Algeria/91118/2020 | EPI_ISL_12156729 | 20/09/2020 | Africa / Algeria / Algiers | Human | unknown | Male | 35 | unknown | unknown | unknown | B.1.597 | GH |
| hCoV-19/Algeria/91404/2020 | EPI_ISL_12156730 | 21/09/2020 | Africa / Algeria / Algiers | Human | unknown | Female | 80 | unknown | unknown | unknown | B.1.1 | GR |
| hCoV-19/Algeria/95788/2020 | EPI_ISL_12156731 | 29/09/2020 | Africa / Algeria / Blida | Human | unknown | Female | 51 | unknown | unknown | unknown | B.1.1 | GR |
| hCoV-19/Algeria/96150/2020 | EPI_ISL_12156732 | 01/10/2020 | Africa / Algeria / Bouira | Human | unknown | Male | 92 | unknown | unknown | unknown | B.1.1 | GR |
| hCoV-19/Algeria/96626/2020 | EPI_ISL_12156733 | 03/10/2020 | Africa / Algeria / Bejaia | Human | unknown | Male | 63 | unknown | unknown | unknown | B.1.1 | GR |
| hCoV-19/Algeria/131666/2020 | EPI_ISL_12156734 | 27/11/2020 | Africa / Algeria / Algiers | Human | unknown | Male | 54 | unknown | unknown | unknown | B.1.160 | GH |
| hCoV-19/Algeria/131655/2020 | EPI_ISL_12156735 | 29/11/2020 | Africa / Algeria / Algiers | Human | unknown | Male | 71 | unknown | unknown | unknown | B.1.597 | GH |
| hCoV-19/Algeria/131466/2020 | EPI_ISL_12156736 | 29/11/2020 | Africa / Algeria / Algiers | Human | unknown | Male | 51 | unknown | unknown | unknown | B.1.160 | GH |
| hCoV-19/Algeria/134048/2020 | EPI_ISL_12156737 | 02/12/2020 | Africa / Algeria / Bouira | Human | unknown | Male | 88 | unknown | unknown | unknown | B.1.597 | GH |
| hCoV-19/Algeria/135497/2020 | EPI_ISL_12156738 | 04/12/2020 | Africa / Algeria / Algiers | Human | unknown | Female | 29 | unknown | unknown | unknown | B.1 | G |
| hCoV-19/Algeria/141491/2020 | EPI_ISL_12156739 | 22/12/2020 | Africa / Algeria / Algiers | Human | unknown | Female | 75 | unknown | unknown | unknown | B.1.160 | GH |
| hCoV-19/Algeria/142249/2020 | EPI_ISL_12156740 | 24/12/2020 | Africa / Algeria / Tipaza | Human | unknown | Male | 15 | unknown | unknown | unknown | B.1 | G |
| hCoV-19/Algeria/143920/2020 | EPI_ISL_12156741 | 27/12/2020 | Africa / Algeria / Algiers | Human | unknown | Female | 39 | unknown | unknown | unknown | B.1.597 | GH |
| hCoV-19/Algeria/143901/2020 | EPI_ISL_12156742 | 29/12/2020 | Africa / Algeria / Blida | Human | unknown | Female | 34 | unknown | unknown | unknown | B.1 | G |
| hCoV-19/Algeria/204/2021 | EPI_ISL_12156743 | 02/01/2021 | Africa / Algeria / Laghouat | Human | unknown | Female | 32 | unknown | unknown | unknown | B.1.160 | GH |
| hCoV-19/Algeria/8382/2021 | EPI_ISL_12156744 | 25/01/2021 | Africa / Algeria / El Oued | Human | unknown | Female | 87 | unknown | unknown | unknown | B.1.1 | GR |
| hCoV-19/Algeria/8384/2021 | EPI_ISL_12156745 | 25/01/2021 | Africa / Algeria / El Oued | Human | unknown | Female | 68 | unknown | unknown | unknown | B.1.1.317 | GR |
| hCoV-19/Algeria/17885/2021 | EPI_ISL_12156746 | 01/03/2021 | Africa / Algeria / Algiers | Human | unknown | Male | 60 | unknown | unknown | unknown | B.1.525 | G |
| hCoV-19/Algeria/21179/2021 | EPI_ISL_12156747 | 13/03/2021 | Africa / Algeria / Laghouat | Human | unknown | Male | 40 | unknown | unknown | unknown | B.1.525 | G |
| hCoV-19/Algeria/21542/2021 | EPI_ISL_12156748 | 14/03/2021 | Africa / Algeria / Algiers | Human | unknown | Male | 34 | unknown | unknown | unknown | B.1.525 | G |
| hCoV-19/Algeria/22919/2021 | EPI_ISL_12156749 | 22/03/2021 | Africa / Algeria / Blida | Human | unknown | Female | 46 | unknown | unknown | unknown | R.1 | GR |
| hCoV-19/Algeria/27662/2021 | EPI_ISL_12156750 | 07/04/2021 | Africa / Algeria / Laghouat | Human | unknown | Female | 61 | unknown | unknown | unknown | B.1.525 | G |
| hCoV-19/Algeria/33008/2021 | EPI_ISL_12156751 | 26/04/2021 | Africa / Algeria / Algiers | Human | unknown | Male | 57 | unknown | unknown | unknown | B.1.1.7 | GRY |
| hCoV-19/Algeria/32679/2021 | EPI_ISL_12156752 | 26/04/2021 | Africa / Algeria / Algiers | Human | unknown | Female | 60 | unknown | unknown | unknown | B.1.1.7 | GRY |
| hCoV-19/Algeria/33212/2021 | EPI_ISL_12156753 | 28/04/2021 | Africa / Algeria / Algiers | Human | unknown | Male | 24 | unknown | unknown | unknown | B.1.1.7 | GRY |
| hCoV-19/Algeria/34055/2021 | EPI_ISL_12156754 | 02/05/2021 | Africa / Algeria / Algiers | Human | unknown | Female | 35 | unknown | unknown | unknown | B.1.525 | G |
| hCoV-19/Algeria/35665/2021 | EPI_ISL_12156755 | 09/05/2021 | Africa / Algeria / Algiers | Human | unknown | Female | 43 | unknown | unknown | unknown | B.1.1.7 | GRY |
| hCoV-19/Algeria/36483/2021 | EPI_ISL_12156756 | 11/05/2021 | Africa / Algeria / Algiers | Human | unknown | Female | 43 | unknown | unknown | unknown | B.1.525 | G |
| hCoV-19/Algeria/46827/2021 | EPI_ISL_12156757 | 13/06/2021 | Africa / Algeria / Algiers | Human | unknown | Male | 47 | unknown | unknown | unknown | B.1.1.7 | GRY |
| hCoV-19/Algeria/55605/2021 | EPI_ISL_12156758 | 06/07/2021 | Africa / Algeria / Hassi Messaoud | Human | unknown | Male | 60 | unknown | unknown | unknown | B.1.1.7 | GRY |
| hCoV-19/Algeria/55198/2021 | EPI_ISL_12156759 | 06/07/2021 | Africa / Algeria / Laghouat | Human | unknown | Female | 52 | unknown | unknown | unknown | B.1.1.7 | GRY |
| hCoV-19/Algeria/54697/2021 | EPI_ISL_12156760 | 07/07/2021 | Africa / Algeria / Blida | Human | unknown | Male | 44 | unknown | unknown | unknown | B.1.617.2 | GK |
| hCoV-19/Algeria/77809/2021 | EPI_ISL_12156761 | 07/09/2021 | Africa / Algeria / Algiers | Human | unknown | Female | 46 | unknown | unknown | unknown | B.1.617.2 | GK |
| hCoV-19/Algeria/79040/2021 | EPI_ISL_12156762 | 13/09/2021 | Africa / Algeria / Bouira | Human | unknown | Female | 83 | unknown | unknown | unknown | B.1.617.2 | GK |
| hCoV-19/Algeria/80164/2021 | EPI_ISL_12156763 | 16/09/2021 | Africa / Algeria / Bejaia | Human | unknown | Female | 78 | unknown | unknown | unknown | B.1.617.2 | GK |
| hCoV-19/Algeria/99966/2021 | EPI_ISL_12180503 | 28/12/2021 | Africa / Algeria / Blida | Human | unknown | Male | 41 | unknown | unknown | unknown | B.1.617.2 | GK |
| hCoV-19/Algeria/66076/2021 | EPI_ISL_12180656 | 30/07/2021 | Africa / Algeria / El Oued | Human | unknown | Male | 89 | unknown | unknown | unknown | B.1.617.2 | GK |
| hCoV-19/Algeria/142869/2020 | EPI_ISL_12180657 | 26/12/2020 | Africa / Algeria / Laghouat | Human | unknown | Male | 62 | unknown | unknown | unknown | B.1 | GH |
| hCoV-19/Algeria/188/2021 | EPI_ISL_12180658 | 02/01/2021 | Africa / Algeria / Laghouat | Human | unknown | Female | 26 | unknown | unknown | unknown | B.1.597 | GH |
| hCoV-19/Algeria/22446/2021 | EPI_ISL_12180659 | 18/03/2021 | Africa / Algeria / Algiers | Human | unknown | Male | 73 | unknown | unknown | unknown | B.1.525 | G |
| hCoV-19/Algeria/80177/2021 | EPI_ISL_12180660 | 18/09/2021 | Africa / Algeria / Bejaia | Human | unknown | Male | 62 | unknown | unknown | unknown | B.1.617.2 | GK |
| hCoV-19/Algeria/15356-44FR/2021 | EPI_ISL_1240719 | 21/02/2021 | Africa / Algeria / Algiers | Human | S gene dropout | Male | 47 | Live | unknown | S gene dropout | Unassigned | O |
| hCoV-19/Algeria/17646-44FR/2021 | EPI_ISL_1240720 | 26/02/2021 | Africa / Algeria / Ouargla | Human | S gene dropout | Male | 57 | Hospitalized | unknown | S gene dropout | Unassigned | O |
| hCoV-19/Algeria/17646-47FR/2021 | EPI_ISL_1240721 | 26/02/2021 | Africa / Algeria / Ouargla | Human | S gene dropout | Male | 57 | Hospitalized | unknown | S gene dropout | Unassigned | G |
| hCoV-19/Algeria/18134-44FR/2021 | EPI_ISL_1240722 | 28/02/2021 | Africa / Algeria / Ain Salah | Human | S gene dropout | Male | 87 | Hospitalized | unknown | S gene dropout | Unassigned | O |
| hCoV-19/Algeria/18134-47FR/2021 | EPI_ISL_1240723 | 28/02/2021 | Africa / Algeria / Ain Salah | Human | S gene dropout | Male | 87 | Hospitalized | unknown | S gene dropout | Unassigned | G |
| hCoV-19/Algeria/19101-44FR/2021 | EPI_ISL_1240724 | 04/03/2021 | Africa / Algeria / Algiers | Human | S gene dropout | Female | 38 | Hospitalized | unknown | S gene dropout | Unassigned | O |
| hCoV-19/Algeria/19101-47FR/2021 | EPI_ISL_1240725 | 04/03/2021 | Africa / Algeria / Algiers | Human | S gene dropout | Female | 38 | Hospitalized | unknown | S gene dropout | Unassigned | G |
| hCoV-19/Algeria/17363/2022 | EPI_ISL_12654823 | 27/02/2022 | Africa / Algeria / Bouira | Human | unknown | Male | 68 | unknown | unknown | unknown | B.1.617.2 | GK |
| hCoV-19/Algeria/16564/2022 | EPI_ISL_12654824 | 21/02/2022 | Africa / Algeria / Algiers | Human | unknown | Female | 58 | unknown | unknown | unknown | BA.2 | GRA |
| hCoV-19/Algeria/19994/2022 | EPI_ISL_12654825 | 23/03/2022 | Africa / Algeria / Bouira | Human | unknown | Male | 62 | unknown | unknown | unknown | BA.2 | GRA |
| hCoV-19/Algeria/16372/2022 | EPI_ISL_12654826 | 20/02/2022 | Africa / Algeria / Algiers | Human | unknown | Male | 73 | unknown | unknown | unknown | BA.2 | GRA |
| hCoV-19/Algeria/16723/2022 | EPI_ISL_12654827 | 22/02/2022 | Africa / Algeria / Algiers | Human | unknown | Female | 72 | unknown | unknown | unknown | BA.2 | GRA |
| hCoV-19/Algeria/19947/2022 | EPI_ISL_12654828 | 22/03/2022 | Africa / Algeria / Bouira | Human | unknown | Male | 80 | unknown | unknown | unknown | BA.2 | GRA |
| hCoV-19/Algeria/20423/2022 | EPI_ISL_12654829 | 27/03/2022 | Africa / Algeria / Algiers | Human | unknown | Male | 27 | unknown | unknown | unknown | BA.2.9 | GRA |
| hCoV-19/Algeria/19531/2022 | EPI_ISL_13069001 | 17/03/2022 | Africa / Algeria / Bouira | Human | unknown | Male | 69 | unknown | unknown | unknown | BA.2 | GRA |
| hCoV-19/Algeria/21973/2022 | EPI_ISL_13071427 | 13/04/2022 | Africa / Algeria / Algiers | Human | unknown | Male | 34 | unknown | unknown | unknown | BA.2.3 | GRA |
| hCoV-19/Algeria/22459/2022 | EPI_ISL_13071428 | 20/04/2022 | Africa / Algeria / Algiers | Human | unknown | Female | 57 | unknown | unknown | unknown | XY | GRA |
| hCoV-19/Algeria/2691/2022 | EPI_ISL_13071429 | 18/04/2022 | Africa / Algeria / Algiers | Human | unknown | Female | unknown | unknown | unknown | unknown | BA.2.9.3 | GRA |
| hCoV-19/Algeria/3010/2022 | EPI_ISL_13071430 | 20/04/2022 | Africa / Algeria / Algiers | Human | unknown | unknown | unknown | unknown | unknown | unknown | BA.2 | GRA |
| hCoV-19/Algeria/3055/2022 | EPI_ISL_13071431 | 20/04/2022 | Africa / Algeria / Algiers | Human | unknown | unknown | unknown | unknown | unknown | unknown | BA.1.1 | GRA |
| hCoV-19/Algeria/27344/2022 | EPI_ISL_13080449 | 23/02/2022 | Africa / Algeria / Oran | Human | unknown | Female | 60 | unknown | unknown | unknown | BA.2 | GRA |
| hCoV-19/Algeria/27381/2022 | EPI_ISL_13080450 | 28/02/2022 | Africa / Algeria / Oran | Human | unknown | Male | 48 | unknown | unknown | unknown | BA.2 | GRA |
| hCoV-19/Algeria/27384/2022 | EPI_ISL_13080451 | 28/02/2022 | Africa / Algeria / Oran | Human | unknown | Female | unknown | unknown | unknown | unknown | BA.2 | GRA |
| hCoV-19/Algeria/27392/2022 | EPI_ISL_13080452 | 28/02/2022 | Africa / Algeria / Oran | Human | unknown | Male | 66 | unknown | unknown | unknown | BA.2 | GRA |
| hCoV-19/Algeria/27453/2022 | EPI_ISL_13080453 | 07/03/2022 | Africa / Algeria / Oran | Human | unknown | Female | 33 | unknown | unknown | unknown | BA.2 | GRA |
| hCoV-19/Algeria/27474/2022 | EPI_ISL_13080454 | 08/03/2022 | Africa / Algeria / Oran | Human | unknown | Male | 35 | unknown | unknown | unknown | BA.2 | GRA |
| hCoV-19/Algeria/27475/2022 | EPI_ISL_13080455 | 10/03/2022 | Africa / Algeria / Oran | Human | unknown | Female | 81 | unknown | unknown | unknown | BA.1.1 | GRA |
| hCoV-19/Algeria/27492/2022 | EPI_ISL_13080456 | 14/03/2022 | Africa / Algeria / Oran | Human | unknown | Male | 47 | unknown | unknown | unknown | BA.2 | GRA |
| hCoV-19/Algeria/27521/2022 | EPI_ISL_13080457 | 15/03/2022 | Africa / Algeria / Oran | Human | unknown | Male | 68 | unknown | unknown | unknown | BA.2 | GRA |
| hCoV-19/Algeria/25443/2022 | EPI_ISL_13282958 | 11/01/2022 | Africa / Algeria / Oran | Human | unknown | Female | 32 | unknown | unknown | unknown | BA.1 | GRA |
| hCoV-19/Algeria/26888/2022 | EPI_ISL_13282959 | 31/01/2022 | Africa / Algeria / Oran | Human | unknown | Female | 72 | unknown | unknown | unknown | BA.1.1 | GRA |
| hCoV-19/Algeria/27099/2022 | EPI_ISL_13282960 | 07/02/2022 | Africa / Algeria / Oran | Human | unknown | Male | 72 | unknown | unknown | unknown | BA.2 | GRA |
| hCoV-19/Algeria/27249/2022 | EPI_ISL_13282961 | 15/02/2022 | Africa / Algeria / Oran | Human | unknown | Female | 27 | unknown | unknown | unknown | BA.2 | GRA |
| hCoV-19/Algeria/27259/2022 | EPI_ISL_13282962 | 16/02/2022 | Africa / Algeria / Oran | Human | unknown | Male | 69 | unknown | unknown | unknown | B.1.617.2 | G |
| hCoV-19/Algeria/2364/2022 | EPI_ISL_13311216 | 23/05/2022 | Africa / Algeria / Sidi Bel Abbes | Human | unknown | Female | 25 | unknown | unknown | unknown | BA.2 | GRA |
| hCoV-19/Algeria/25349/2022 | EPI_ISL_13311217 | 10/01/2022 | Africa / Algeria / Oran | Human | unknown | Female | 65 | unknown | unknown | unknown | B.1.617.2 | GK |
| hCoV-19/Algeria/25598/2022 | EPI_ISL_13311218 | 30/05/2022 | Africa / Algeria / Bouira | Human | unknown | Male | 77 | unknown | unknown | unknown | BA.2.32 | GRA |
| hCoV-19/Algeria/25790/2022 | EPI_ISL_13311219 | 01/06/2022 | Africa / Algeria / Algiers | Human | unknown | Male | 33 | unknown | unknown | unknown | BA.2 | GRA |
| hCoV-19/Algeria/25964/2022 | EPI_ISL_13311220 | 04/06/2022 | Africa / Algeria / Algiers | Human | unknown | Male | unknown | unknown | unknown | unknown | BA.2 | GRA |
| hCoV-19/Algeria/26720/2022 | EPI_ISL_13311221 | 25/01/2022 | Africa / Algeria / Oran | Human | unknown | Female | 65 | unknown | unknown | unknown | BA.2 | GRA |
| hCoV-19/Algeria/26932/2022 | EPI_ISL_13371970 | 12/06/2022 | Africa / Algeria / Algiers | Human | unknown | Male | 51 | unknown | unknown | unknown | BA.2 | GRA |
| hCoV-19/Algeria/26941/2022 | EPI_ISL_13371971 | 12/06/2022 | Africa / Algeria / Algiers | Human | unknown | Female | 44 | unknown | unknown | unknown | BA.5.2 | GRA |
| hCoV-19/Algeria/26942/2022 | EPI_ISL_13371972 | 12/06/2022 | Africa / Algeria / Algiers | Human | unknown | Female | 55 | unknown | unknown | unknown | BA.5.2 | GRA |
| hCoV-19/Algeria/26943/2022 | EPI_ISL_13371973 | 12/06/2022 | Africa / Algeria / Algiers | Human | unknown | Male | 64 | unknown | unknown | unknown | BA.5.2 | GRA |
| hCoV-19/Algeria/26944/2022 | EPI_ISL_13371974 | 12/06/2022 | Africa / Algeria / Algiers | Human | unknown | Female | 85 | unknown | unknown | unknown | BA.5.2 | GRA |
| hCoV-19/Algeria/79/2022 | EPI_ISL_13553850 | 13/01/2022 | Africa / Algeria / Annaba | Human | unknown | Male | 64 | unknown | unknown | unknown | B.1.617.2 | GK |
| hCoV-19/Algeria/91/2022 | EPI_ISL_13553851 | 16/01/2022 | Africa / Algeria / Annaba | Human | unknown | Female | 76 | unknown | unknown | unknown | B.1.617.2 | GK |
| hCoV-19/Algeria/147/2022 | EPI_ISL_13553852 | 16/01/2022 | Africa / Algeria / Annaba | Human | unknown | unknown | unknown | unknown | unknown | unknown | B.1.617.2 | GK |
| hCoV-19/Algeria/170/2022 | EPI_ISL_13553853 | 16/01/2022 | Africa / Algeria / Annaba | Human | unknown | unknown | unknown | unknown | unknown | unknown | BA.1.1 | GRA |
| hCoV-19/Algeria/2345/2022 | EPI_ISL_13553854 | 12/05/2022 | Africa / Algeria / Aidi Bel Abbes | Human | unknown | Female | 38 | unknown | unknown | unknown | B.1.617.2 | GK |
| hCoV-19/Algeria/2346/2022 | EPI_ISL_13553855 | 12/05/2022 | Africa / Algeria / Aidi Bel Abbes | Human | unknown | Female | 47 | unknown | unknown | unknown | BA.2 | GRA |
| hCoV-19/Algeria/2355/2022 | EPI_ISL_13553856 | 18/05/2022 | Africa / Algeria / Aidi Bel Abbes | Human | unknown | Male | 34 | unknown | unknown | unknown | BA.2 | GRA |
| hCoV-19/Algeria/23189/2022 | EPI_ISL_13553857 | 26/06/2022 | Africa / Algeria / Thenia | Human | unknown | Male | 38 | unknown | unknown | unknown | BE.1 | GRA |
| hCoV-19/Algeria/27355/2022 | EPI_ISL_13553858 | 24/02/2022 | Africa / Algeria / Oran | Human | unknown | unknown | 66 | unknown | unknown | unknown | BA.2 | GRA |
| hCoV-19/Algeria/2357/2022 | EPI_ISL_13610579 | 18/05/2022 | Africa / Algeria / Aidi Bel Abbes | Human | unknown | Female | 28 | unknown | unknown | unknown | BA.2 | GRA |
| hCoV-19/Algeria/29074/2022 | EPI_ISL_13611587 | 16/06/2022 | Africa / Algeria / Batna | Human | unknown | Male | 31 | Hospitalized | unknown | unknown | BA.2 | GRA |
| hCoV-19/Algeria/27956/2022 | EPI_ISL_13611588 | 16/06/2022 | Africa / Algeria / Algiers | Human | unknown | Male | 30 | Live | unknown | unknown | BA.5.2.1 | GRA |
| hCoV-19/Algeria/443/2022 | EPI_ISL_13611589 | 24/03/2022 | Africa / Algeria / Annaba | Human | unknown | Female | 38 | Live | unknown | unknown | BA.2 | GRA |
| hCoV-19/Algeria/323/2022 | EPI_ISL_13611590 | 15/02/2022 | Africa / Algeria / Annaba | Human | unknown | Male | unknown | Live | unknown | unknown | BA.1.1.1 | GRA |
| hCoV-19/Algeria/28072/2022 | EPI_ISL_13611591 | 23/06/2022 | Africa / Algeria / Algiers | Human | unknown | Female | 30 | Live | unknown | unknown | BA.5.2.1 | GRA |
| hCoV-19/Algeria/UZB_548202207114/2022 | EPI_ISL_13840320 | 08/07/2022 | Africa / Algeria / Laghouat | Human | Baseline surveilance | Male | 6 | unknown | unknown | Baseline surveilance | BA.5.1.23 | GRA |
| hCoV-19/Algeria/0331/2022 | EPI_ISL_13956172 | 01/02/2022 | Africa / Algeria / Annaba | Human | National SARS-CoV-2 genomic and variants surveillance program | Female | 57 | unknown | unknown | National SARS-CoV-2 genomic and variants surveillance program | BA.2 | GRA |
| hCoV-19/Algeria/0269/2022 | EPI_ISL_13956173 | 02/02/2022 | Africa / Algeria / Annaba | Human | National SARS-CoV-2 genomic and variants surveillance program | Female | 35 | unknown | unknown | National SARS-CoV-2 genomic and variants surveillance program | BA.1.1 | GRA |
| hCoV-19/Algeria/0173/2022 | EPI_ISL_13956174 | 23/01/2022 | Africa / Algeria / Annaba | Human | National SARS-CoV-2 genomic and variants surveillance program | Female | 45 | unknown | unknown | National SARS-CoV-2 genomic and variants surveillance program | BA.1.1 | GRA |
| hCoV-19/Algeria/0198/2022 | EPI_ISL_13956175 | 25/01/2022 | Africa / Algeria / Annaba | Human | National SARS-CoV-2 genomic and variants surveillance program | Female | 46 | unknown | unknown | National SARS-CoV-2 genomic and variants surveillance program | BA.1.1 | GRA |
| hCoV-19/Algeria/0417/2022 | EPI_ISL_13956176 | 15/03/2022 | Africa / Algeria / Annaba | Human | National SARS-CoV-2 genomic and variants surveillance program | Female | 2 months | unknown | unknown | National SARS-CoV-2 genomic and variants surveillance program | BA.2 | GRA |
| hCoV-19/Algeria/0213/2022 | EPI_ISL_13956177 | 26/01/2022 | Africa / Algeria / Annaba | Human | National SARS-CoV-2 genomic and variants surveillance program | Female | 31 | unknown | unknown | National SARS-CoV-2 genomic and variants surveillance program | BA.1.18 | GRA |
| hCoV-19/Algeria/D37/2022 | EPI_ISL_13956178 | 07/01/2022 | Africa / Algeria / Annaba | Human | National SARS-CoV-2 genomic and variants surveillance program | Female | 86 | unknown | unknown | National SARS-CoV-2 genomic and variants surveillance program | B.1.617.2 | GK |
| hCoV-19/Algeria/D34/2022 | EPI_ISL_13956179 | 06/01/2022 | Africa / Algeria / Annaba | Human | National SARS-CoV-2 genomic and variants surveillance program | Female | 36 | unknown | unknown | National SARS-CoV-2 genomic and variants surveillance program | B.1.617.2 | GK |
| hCoV-19/Algeria/D30/2022 | EPI_ISL_13956180 | 05/01/2022 | Africa / Algeria / Annaba | Human | National SARS-CoV-2 genomic and variants surveillance program | Female | 28 | unknown | unknown | National SARS-CoV-2 genomic and variants surveillance program | B.1.617.2 | GK |
| hCoV-19/Algeria/D124/2022 | EPI_ISL_13956181 | 20/01/2022 | Africa / Algeria / Annaba | Human | National SARS-CoV-2 genomic and variants surveillance program | Male | unknown | unknown | unknown | National SARS-CoV-2 genomic and variants surveillance program | B.1.617.2 | GK |
| hCoV-19/Algeria/28299/2022 | EPI_ISL_13956182 | 27/06/2022 | Africa / Algeria / Algiers | Human | National SARS-CoV-2 genomic and variants surveillance program | Male | 32 | unknown | unknown | National SARS-CoV-2 genomic and variants surveillance program | BA.5.1 | GRA |
| hCoV-19/Algeria/31168/2022 | EPI_ISL_14497294 | 20/07/2022 | Africa / Algeria / Algiers | Human | unknown | Female | 39 | Live | unknown | unknown | BA.5.2.1 | GRA |
| hCoV-19/Algeria/32593/2022 | EPI_ISL_14497295 | 21/07/2022 | Africa / Algeria / Blida | Human | unknown | Female | 32 | Live | unknown | unknown | BF.5 | GRA |
| hCoV-19/Algeria/30947/2022 | EPI_ISL_14497296 | 18/07/2022 | Africa / Algeria / Blida | Human | unknown | Female | 59 | Live | unknown | unknown | BA.5.2 | GRA |
| hCoV-19/Algeria/31497/2022 | EPI_ISL_14497297 | 21/07/2022 | Africa / Algeria / Algiers | Human | unknown | Male | 28 | Live | unknown | unknown | BA.5.1 | GRA |
| hCoV-19/Algeria/30926/2022 | EPI_ISL_14497298 | 18/07/2022 | Africa / Algeria / Algiers | Human | unknown | Female | 41 | Live | unknown | unknown | BA.5.2.1 | GRA |
| hCoV-19/Algeria/31399/2022 | EPI_ISL_14497299 | 18/07/2022 | Africa / Algeria / Algiers | Human | unknown | Female | 86 | Live | unknown | unknown | BA.5.1.22 | GRA |
| hCoV-19/Algeria/31735/2022 | EPI_ISL_14497300 | 23/07/2022 | Africa / Algeria / Blida | Human | unknown | Female | 75 | Live | unknown | unknown | BE.1 | GRA |
| hCoV-19/Algeria/31697/2022 | EPI_ISL_14497301 | 22/07/2022 | Africa / Algeria / Bouira | Human | unknown | Male | 34 | Live | unknown | unknown | BA.5.2.20 | GRA |
| hCoV-19/Algeria/31487/2022 | EPI_ISL_14497302 | 19/07/2022 | Africa / Algeria / Tebessa | Human | unknown | Male | 77 | Live | unknown | unknown | BA.5.2 | GRA |
| hCoV-19/Algeria/30962/2022 | EPI_ISL_14497303 | 18/07/2022 | Africa / Algeria / Medea | Human | unknown | Female | 61 | Live | unknown | unknown | BA.5.2.27 | GRA |
| hCoV-19/Algeria/31861/2022 | EPI_ISL_14497304 | 24/07/2022 | Africa / Algeria / Bouira | Human | unknown | Male | 57 | Live | unknown | unknown | BF.39 | GRA |
| hCoV-19/Algeria/2705/2022 | EPI_ISL_14497306 | 29/06/2022 | Africa / Algeria / Tlemcen | Human | National SARS-CoV-2 genomic and variants surveillance program | Female | 52 | Live | 2021-09 | National SARS-CoV-2 genomic and variants surveillance program | BE.1 | GRA |
| hCoV-19/Algeria/2707/2022 | EPI_ISL_14497307 | 30/06/2022 | Africa / Algeria / Tlemcen | Human | National SARS-CoV-2 genomic and variants surveillance program | Male | 27 | Live | unknown | National SARS-CoV-2 genomic and variants surveillance program | BA.5.2 | GRA |
| hCoV-19/Algeria/2711/2022 | EPI_ISL_14497308 | 02/07/2022 | Africa / Algeria / Tlemcen | Human | National SARS-CoV-2 genomic and variants surveillance program | Female | 30 | Live | unknown | National SARS-CoV-2 genomic and variants surveillance program | BA.5.2 | GRA |
| hCoV-19/Algeria/27954/2022 | EPI_ISL_14497309 | 04/07/2022 | Africa / Algeria / Oran | Human | National SARS-CoV-2 genomic and variants surveillance program | unknown | 57 | Live | unknown | National SARS-CoV-2 genomic and variants surveillance program | BA.5.1 | GRA |
| hCoV-19/Algeria/27967/2022 | EPI_ISL_14497310 | 10/07/2022 | Africa / Algeria / Oran | Human | National SARS-CoV-2 genomic and variants surveillance program | unknown | 26 | Live | unknown | National SARS-CoV-2 genomic and variants surveillance program | BA.2.12.1 | GRA |
| hCoV-19/Algeria/27975/2022 | EPI_ISL_14497311 | 11/07/2022 | Africa / Algeria / Oran | Human | National SARS-CoV-2 genomic and variants surveillance program | unknown | 18 | Live | unknown | National SARS-CoV-2 genomic and variants surveillance program | BA.5.2 | GRA |
| hCoV-19/Algeria/27978/2022 | EPI_ISL_14497312 | 12/07/2022 | Africa / Algeria / Oran | Human | National SARS-CoV-2 genomic and variants surveillance program | Female | 27 | Live | unknown | National SARS-CoV-2 genomic and variants surveillance program | BA.5.1 | GRA |
| hCoV-19/Algeria/14/2022 | EPI_ISL_14497313 | 29/06/2022 | Africa / Algeria / Ouargla | Human | National SARS-CoV-2 genomic and variants surveillance program | Male | 56 | Live | unknown | National SARS-CoV-2 genomic and variants surveillance program | BA.5.2 | GRA |
| hCoV-19/Algeria/17/2022 | EPI_ISL_14497314 | 17/07/2022 | Africa / Algeria / Touggourt | Human | National SARS-CoV-2 genomic and variants surveillance program | Female | 80 | Live | unknown | National SARS-CoV-2 genomic and variants surveillance program | BA.5.2 | GRA |
| hCoV-19/Algeria/28014/2022 | EPI_ISL_14497315 | 30/06/2022 | Africa / Algeria / Oran | Human | National SARS-CoV-2 genomic and variants surveillance program | Female | 30 | Live | unknown | National SARS-CoV-2 genomic and variants surveillance program | BA.5.2 | GRA |
| hCoV-19/Algeria/28029/2022 | EPI_ISL_14497316 | 17/07/2022 | Africa / Algeria / Oran | Human | National SARS-CoV-2 genomic and variants surveillance program | Female | 43 | Live | 2021-08 | National SARS-CoV-2 genomic and variants surveillance program | BA.2 | GRA |
| hCoV-19/Algeria/31336/2022 | EPI_ISL_14497322 | 20/07/2022 | Africa / Algeria / Algiers | Human | unknown | Male | 22 | Live | unknown | unknown | BA.5.2.20 | GRA |
| hCoV-19/Algeria/31851/2022 | EPI_ISL_14497323 | 24/07/2022 | Africa / Algeria / Medea | Human | unknown | Male | 64 | Live | unknown | unknown | BA.5.2 | GRA |
| hCoV-19/Algeria/32073/2022 | EPI_ISL_14497324 | 26/07/2022 | Africa / Algeria / Algiers | Human | unknown | Male | 86 | Live | unknown | unknown | BA.5.1 | GRA |
| hCoV-19/Algeria/32328/2022 | EPI_ISL_14497325 | 23/07/2022 | Africa / Algeria / Algiers | Human | unknown | Male | 20 | Live | unknown | unknown | BE.1 | GRA |
| hCoV-19/Algeria/32329/2022 | EPI_ISL_14497326 | 27/07/2022 | Africa / Algeria / Algiers | Human | unknown | Female | 23 | Live | unknown | unknown | BA.5.2 | GRA |
| hCoV-19/Algeria/32436/2022 | EPI_ISL_14497327 | 27/07/2022 | Africa / Algeria / Algiers | Human | unknown | Male | 1 | Live | unknown | unknown | BA.5.2 | GRA |
| hCoV-19/Algeria/32438/2022 | EPI_ISL_14497328 | 27/07/2022 | Africa / Algeria / Algiers | Human | unknown | Female | 2 | Live | unknown | unknown | BA.5.2.27 | GRA |
| hCoV-19/Algeria/32446/2022 | EPI_ISL_14497329 | 27/07/2022 | Africa / Algeria / Bouira | Human | unknown | Female | 64 | Live | unknown | unknown | BA.5.6 | GRA |
| hCoV-19/Algeria/31424/2022 | EPI_ISL_14498411 | 20/07/2022 | Africa / Algeria / Algiers | Human | unknown | Female | 30 | Live | unknown | unknown | BA.5.2.20 | GRA |
| hCoV-19/Algeria/31427/2022 | EPI_ISL_14498412 | 20/07/2022 | Africa / Algeria / Algiers | Human | unknown | Male | 21 | Live | unknown | unknown | BA.5.1 | GRA |
| hCoV-19/Algeria/31414/2022 | EPI_ISL_14498413 | 20/07/2022 | Africa / Algeria / Algiers | Human | unknown | Male | 68 | Live | unknown | unknown | BA.5.2 | GRA |
| hCoV-19/Algeria/31419/2022 | EPI_ISL_14498414 | 20/07/2022 | Africa / Algeria / Algiers | Human | unknown | Female | 28 | Live | unknown | unknown | BA.5.2 | GRA |
| hCoV-19/Algeria/30955/2022 | EPI_ISL_14498415 | 16/07/2022 | Africa / Algeria / Tebessa | Human | unknown | Male | 70 | Live | unknown | unknown | BA.5.2 | GRA |
| hCoV-19/Algeria/30532/2022 | EPI_ISL_14498416 | 17/07/2022 | Africa / Algeria / Algiers | Human | unknown | Female | 37 | Live | unknown | unknown | BA.5.2.20 | GRA |
| hCoV-19/Algeria/30906/2022 | EPI_ISL_14498417 | 17/07/2022 | Africa / Algeria / Algiers | Human | unknown | Female | 78 | Live | unknown | unknown | BA.5.1.4 | GRA |
| hCoV-19/Algeria/29140/2022 | EPI_ISL_14498418 | 04/07/2022 | Africa / Algeria / Algiers | Human | unknown | Female | 89 | Hospitalized | unknown | unknown | BA.2.52 | GRA |
| hCoV-19/Algeria/6322/2022 | EPI_ISL_14498419 | 22/06/2022 | Africa / Algeria / Sidi Bel Abbes | Human | National SARS-CoV-2 genomic and variants surveillance program | Female | 64 | Live | unknown | National SARS-CoV-2 genomic and variants surveillance program | BA.2.12.1 | GRA |
| hCoV-19/Algeria/8821/2022 | EPI_ISL_14498420 | 17/07/2022 | Africa / Algeria / Algiers | Human | National SARS-CoV-2 genomic and variants surveillance program | unknown | unknown | Live | unknown | National SARS-CoV-2 genomic and variants surveillance program | BA.5.2 | GRA |
| hCoV-19/Algeria/32903/2022 | EPI_ISL_14625222 | 01/08/2022 | Africa / Algeria / Algiers | Human | National SARS-CoV-2 genomic and variants surveillance program | Female | 59 | Live | unknown | National SARS-CoV-2 genomic and variants surveillance program | BA.5.2 | GRA |
| hCoV-19/Algeria/32912/2022 | EPI_ISL_14625223 | 01/08/2022 | Africa / Algeria / Blida | Human | National SARS-CoV-2 genomic and variants surveillance program | Female | 37 | Live | unknown | National SARS-CoV-2 genomic and variants surveillance program | BA.5.2.1 | GRA |
| hCoV-19/Algeria/32974/2022 | EPI_ISL_14625224 | 01/08/2022 | Africa / Algeria / Algiers | Human | National SARS-CoV-2 genomic and variants surveillance program | Female | 88 | Live | unknown | National SARS-CoV-2 genomic and variants surveillance program | BA.5.2 | GRA |
| hCoV-19/Algeria/33020/2022 | EPI_ISL_14625225 | 02/08/2022 | Africa / Algeria / MEDEA | Human | National SARS-CoV-2 genomic and variants surveillance program | Male | 36 | Live | unknown | National SARS-CoV-2 genomic and variants surveillance program | BA.5.1 | GRA |
| hCoV-19/Algeria/33111/2022 | EPI_ISL_14625226 | 02/08/2022 | Africa / Algeria / MEDEA | Human | National SARS-CoV-2 genomic and variants surveillance program | Female | 67 | Live | unknown | National SARS-CoV-2 genomic and variants surveillance program | BA.5.2 | GRA |
| hCoV-19/Algeria/33118/2022 | EPI_ISL_14625227 | 02/08/2022 | Africa / Algeria / Blida | Human | National SARS-CoV-2 genomic and variants surveillance program | Female | 71 | Live | unknown | National SARS-CoV-2 genomic and variants surveillance program | BA.5.2.27 | GRA |
| hCoV-19/Algeria/33119/2022 | EPI_ISL_14625228 | 03/08/2022 | Africa / Algeria / MEDEA | Human | National SARS-CoV-2 genomic and variants surveillance program | Female | 32 | Live | unknown | National SARS-CoV-2 genomic and variants surveillance program | BA.5.2 | GRA |
| hCoV-19/Algeria/34320/2022 | EPI_ISL_14625229 | 14/08/2022 | Africa / Algeria / Algiers | Human | National SARS-CoV-2 genomic and variants surveillance program | Male | 40 | Live | unknown | National SARS-CoV-2 genomic and variants surveillance program | BA.5.2.20 | GRA |
| hCoV-19/Algeria/32734/2022 | EPI_ISL_14625428 | 01/08/2022 | Africa / Algeria / MEDEA | Human | National SARS-CoV-2 genomic and variants surveillance program | Male | 70 | Live | unknown | National SARS-CoV-2 genomic and variants surveillance program | BA.5.2.44 | GRA |
| hCoV-19/Algeria/32985/2022 | EPI_ISL_14625429 | 01/08/2022 | Africa / Algeria / MEDEA | Human | National SARS-CoV-2 genomic and variants surveillance program | Male | 50 | Live | unknown | National SARS-CoV-2 genomic and variants surveillance program | BA.5.2 | GRA |
| hCoV-19/Algeria/33010/2022 | EPI_ISL_14625430 | 02/08/2022 | Africa / Algeria / MEDEA | Human | National SARS-CoV-2 genomic and variants surveillance program | Male | 61 | Live | unknown | National SARS-CoV-2 genomic and variants surveillance program | BA.5.2 | GRA |
| hCoV-19/Algeria/33007/2022 | EPI_ISL_14625431 | 01/08/2022 | Africa / Algeria / MEDEA | Human | National SARS-CoV-2 genomic and variants surveillance program | Female | 42 | Live | unknown | National SARS-CoV-2 genomic and variants surveillance program | BA.5.2 | GRA |
| hCoV-19/Algeria/33403/2022 | EPI_ISL_14625432 | 04/08/2022 | Africa / Algeria / Algiers | Human | National SARS-CoV-2 genomic and variants surveillance program | Female | 26 | Live | unknown | National SARS-CoV-2 genomic and variants surveillance program | BA.5.2 | GRA |
| hCoV-19/Algeria/33275/2022 | EPI_ISL_14625433 | 04/08/2022 | Africa / Algeria / MEDEA | Human | National SARS-CoV-2 genomic and variants surveillance program | Female | 64 | Live | unknown | National SARS-CoV-2 genomic and variants surveillance program | BA.5.2 | GRA |
| hCoV-19/Algeria/33268/2022 | EPI_ISL_14625434 | 03/08/2022 | Africa / Algeria / TEBESSA | Human | National SARS-CoV-2 genomic and variants surveillance program | Female | 37 | Live | unknown | National SARS-CoV-2 genomic and variants surveillance program | BA.5.2 | GRA |
| hCoV-19/Algeria/33264/2022 | EPI_ISL_14625435 | 04/08/2022 | Africa / Algeria / Bouira | Human | National SARS-CoV-2 genomic and variants surveillance program | Male | 91 | Live | unknown | National SARS-CoV-2 genomic and variants surveillance program | BA.5.2.1 | GRA |
| hCoV-19/Algeria/33263/2022 | EPI_ISL_14625436 | 03/08/2022 | Africa / Algeria / Bouira | Human | National SARS-CoV-2 genomic and variants surveillance program | Female | 34 | Live | unknown | National SARS-CoV-2 genomic and variants surveillance program | BA.5.6 | GRA |
| hCoV-19/Algeria/33262/2022 | EPI_ISL_14685933 | 03/08/2022 | Africa / Algeria / Bouira | Human | National SARS-CoV-2 genomic and variants surveillance program | Female | 46 | Live | unknown | National SARS-CoV-2 genomic and variants surveillance program | BA.5.6 | GRA |
| hCoV-19/Algeria/33261/2022 | EPI_ISL_14685934 | 02/08/2022 | Africa / Algeria / Bouira | Human | National SARS-CoV-2 genomic and variants surveillance program | Male | 42 | Live | unknown | National SARS-CoV-2 genomic and variants surveillance program | BA.5.6 | GRA |
| hCoV-19/Algeria/36601/2022 | EPI_ISL_15112244 | 06/09/2022 | Africa / Algeria / Blida | Human | National SARS-CoV-2 genomic and variants surveillance program | Female | 42 | Live | unknown | National SARS-CoV-2 genomic and variants surveillance program | BA.5.2 | GRA |
| hCoV-19/Algeria/36757/2022 | EPI_ISL_15112245 | 07/09/2022 | Africa / Algeria / Blida | Human | National SARS-CoV-2 genomic and variants surveillance program | Male | 39 | Live | unknown | National SARS-CoV-2 genomic and variants surveillance program | BA.5.2.1 | GRA |
| hCoV-19/Algeria/36759/2022 | EPI_ISL_15112246 | 07/09/2022 | Africa / Algeria / Blida | Human | National SARS-CoV-2 genomic and variants surveillance program | Female | 53 | Live | unknown | National SARS-CoV-2 genomic and variants surveillance program | BA.5.2 | GRA |
| hCoV-19/Algeria/36826/2022 | EPI_ISL_15112247 | 07/09/2022 | Africa / Algeria / Algiers | Human | National SARS-CoV-2 genomic and variants surveillance program | Male | 51 | Live | unknown | National SARS-CoV-2 genomic and variants surveillance program | BF.7.3 | GRA |
| hCoV-19/Algeria/36827/2022 | EPI_ISL_15112248 | 07/09/2022 | Africa / Algeria / Blida | Human | National SARS-CoV-2 genomic and variants surveillance program | Male | 82 | Live | unknown | National SARS-CoV-2 genomic and variants surveillance program | BA.5.2 | GRA |
| hCoV-19/Algeria/36837/2022 | EPI_ISL_15112249 | 07/09/2022 | Africa / Algeria / Tebessa | Human | National SARS-CoV-2 genomic and variants surveillance program | Female | 80 | Live | unknown | National SARS-CoV-2 genomic and variants surveillance program | BA.5.2 | GRA |
| hCoV-19/Algeria/36954/2022 | EPI_ISL_15112250 | 08/09/2022 | Africa / Algeria / Bouira | Human | National SARS-CoV-2 genomic and variants surveillance program | Female | 1 month | Live | unknown | National SARS-CoV-2 genomic and variants surveillance program | BA.5.1 | GRA |
| hCoV-19/Algeria/36955/2022 | EPI_ISL_15112251 | 07/09/2022 | Africa / Algeria / Algiers | Human | National SARS-CoV-2 genomic and variants surveillance program | Female | 47 | Live | unknown | National SARS-CoV-2 genomic and variants surveillance program | BA.5.2.7 | GRA |
| hCoV-19/Algeria/36956/2022 | EPI_ISL_15112252 | 07/09/2022 | Africa / Algeria / Algiers | Human | National SARS-CoV-2 genomic and variants surveillance program | Female | 47 | Live | unknown | National SARS-CoV-2 genomic and variants surveillance program | BA.5.2.20 | GRA |
| hCoV-19/Algeria/37140/2022 | EPI_ISL_15112253 | 11/09/2022 | Africa / Algeria / Algiers | Human | National SARS-CoV-2 genomic and variants surveillance program | Female | 39 | Live | unknown | National SARS-CoV-2 genomic and variants surveillance program | BA.5.2 | GRA |
| hCoV-19/Algeria/12/2022 | EPI_ISL_15235454 | 28/06/2022 | Africa / Algeria / Touggourt | Human | National SARS-CoV-2 genomic and variants surveillance program | Female | 61 | Live | unknown | National SARS-CoV-2 genomic and variants surveillance program | BA.5.2 | GRA |
| hCoV-19/Algeria/13/2022 | EPI_ISL_15235455 | 27/06/2022 | Africa / Algeria / Touggourt | Human | National SARS-CoV-2 genomic and variants surveillance program | Male | 42 | Live | unknown | National SARS-CoV-2 genomic and variants surveillance program | BA.5.2 | GRA |
| hCoV-19/Algeria/2746/2022 | EPI_ISL_15235456 | 14/07/2022 | Africa / Algeria / Tlemcen | Human | National SARS-CoV-2 genomic and variants surveillance program | Male | 64 | Live | unknown | National SARS-CoV-2 genomic and variants surveillance program | BA.5.2.1 | GRA |
| hCoV-19/Algeria/2755/2022 | EPI_ISL_15235457 | 18/07/2022 | Africa / Algeria / Tlemcen | Human | National SARS-CoV-2 genomic and variants surveillance program | Female | 37 | Live | unknown | National SARS-CoV-2 genomic and variants surveillance program | BA.5.2 | GRA |
| hCoV-19/Algeria/2759/2022 | EPI_ISL_15235458 | 19/07/2022 | Africa / Algeria / Tlemcen | Human | National SARS-CoV-2 genomic and variants surveillance program | Male | 34 | Live | unknown | National SARS-CoV-2 genomic and variants surveillance program | BA.5.2 | GRA |
| hCoV-19/Algeria/2760/2022 | EPI_ISL_15235459 | 19/07/2022 | Africa / Algeria / Tlemcen | Human | National SARS-CoV-2 genomic and variants surveillance program | Male | 46 | Live | unknown | National SARS-CoV-2 genomic and variants surveillance program | BA.5.2 | GRA |
| hCoV-19/Algeria/2761/2022 | EPI_ISL_15235460 | 19/07/2022 | Africa / Algeria / Tlemcen | Human | National SARS-CoV-2 genomic and variants surveillance program | Female | 42 | Live | unknown | National SARS-CoV-2 genomic and variants surveillance program | BA.5.2 | GRA |
| hCoV-19/Algeria/2771/2022 | EPI_ISL_15235461 | 20/07/2022 | Africa / Algeria / Tlemcen | Human | National SARS-CoV-2 genomic and variants surveillance program | Male | 42 | Live | unknown | National SARS-CoV-2 genomic and variants surveillance program | BA.5.2.2 | GRA |
| hCoV-19/Algeria/2773/2022 | EPI_ISL_15235462 | 20/07/2022 | Africa / Algeria / Tlemcen | Human | National SARS-CoV-2 genomic and variants surveillance program | Male | 42 | Live | unknown | National SARS-CoV-2 genomic and variants surveillance program | BA.5.2 | GRA |
| hCoV-19/Algeria/2775/2022 | EPI_ISL_15235463 | 20/07/2022 | Africa / Algeria / Tlemcen | Human | National SARS-CoV-2 genomic and variants surveillance program | Male | 42 | Live | unknown | National SARS-CoV-2 genomic and variants surveillance program | BA.5.2 | GRA |
| hCoV-19/Algeria/2778/2022 | EPI_ISL_15235464 | 21/07/2022 | Africa / Algeria / Tlemcen | Human | National SARS-CoV-2 genomic and variants surveillance program | Female | 42 | Live | unknown | National SARS-CoV-2 genomic and variants surveillance program | BA.5.2 | GRA |
| hCoV-19/Algeria/2783/2022 | EPI_ISL_15235465 | 22/07/2022 | Africa / Algeria / Tlemcen | Human | National SARS-CoV-2 genomic and variants surveillance program | Female | 42 | Live | unknown | National SARS-CoV-2 genomic and variants surveillance program | BF.5 | GRA |
| hCoV-19/Algeria/2789/2022 | EPI_ISL_15235466 | 24/07/2022 | Africa / Algeria / Tlemcen | Human | National SARS-CoV-2 genomic and variants surveillance program | Female | 42 | Live | unknown | National SARS-CoV-2 genomic and variants surveillance program | BA.5.2 | GRA |
| hCoV-19/Algeria/2790/2022 | EPI_ISL_15235467 | 24/07/2022 | Africa / Algeria / Tlemcen | Human | National SARS-CoV-2 genomic and variants surveillance program | Female | 42 | Live | unknown | National SARS-CoV-2 genomic and variants surveillance program | BA.5.1.23 | GRA |
| hCoV-19/Algeria/2798/2022 | EPI_ISL_15235468 | 25/07/2022 | Africa / Algeria / Tlemcen | Human | National SARS-CoV-2 genomic and variants surveillance program | Male | 42 | Live | unknown | National SARS-CoV-2 genomic and variants surveillance program | BA.5.2 | GRA |
| hCoV-19/Algeria/2799/2022 | EPI_ISL_15235469 | 25/07/2022 | Africa / Algeria / Tlemcen | Human | National SARS-CoV-2 genomic and variants surveillance program | Female | 42 | Live | unknown | National SARS-CoV-2 genomic and variants surveillance program | BA.5.2.1 | GRA |
| hCoV-19/Algeria/28067/2022 | EPI_ISL_15235470 | 19/07/2022 | Africa / Algeria / Oran | Human | National SARS-CoV-2 genomic and variants surveillance program | Female | 42 | Live | unknown | National SARS-CoV-2 genomic and variants surveillance program | BE.1.1 | GRA |
| hCoV-19/Algeria/28101/2022 | EPI_ISL_15235471 | 24/07/2022 | Africa / Algeria / Oran | Human | National SARS-CoV-2 genomic and variants surveillance program | Female | 42 | Live | unknown | National SARS-CoV-2 genomic and variants surveillance program | BA.5.2 | GRA |
| hCoV-19/Algeria/28103/2022 | EPI_ISL_15235472 | 24/07/2022 | Africa / Algeria / Oran | Human | National SARS-CoV-2 genomic and variants surveillance program | Male | 42 | Live | unknown | National SARS-CoV-2 genomic and variants surveillance program | BA.5.2 | GRA |
| hCoV-19/Algeria/28336/2022 | EPI_ISL_15238171 | 14/08/2022 | Africa / Algeria / Oran | Human | National SARS-CoV-2 genomic and variants surveillance program | Female | 21 | Live | unknown | National SARS-CoV-2 genomic and variants surveillance program | BA.5.2.1 | GRA |
| hCoV-19/Algeria/34703/2022 | EPI_ISL_15252861 | 16/08/2022 | Africa / Algeria / Algiers | Human | National SARS-CoV-2 genomic and variants surveillance program | Male | 1 | Live | unknown | National SARS-CoV-2 genomic and variants surveillance program | BA.5.2.20 | GRA |
| hCoV-19/Algeria/34899/2022 | EPI_ISL_15252862 | 20/08/2022 | Africa / Algeria / Algiers | Human | National SARS-CoV-2 genomic and variants surveillance program | Female | 1 | Live | unknown | National SARS-CoV-2 genomic and variants surveillance program | BQ.1.1 | GRA |
| hCoV-19/Algeria/34824/2022 | EPI_ISL_15252863 | 18/08/2022 | Africa / Algeria / Algiers | Human | National SARS-CoV-2 genomic and variants surveillance program | Male | 21 | Live | unknown | National SARS-CoV-2 genomic and variants surveillance program | BA.5.5 | GRA |
| hCoV-19/Algeria/34938/2022 | EPI_ISL_15252864 | 21/08/2022 | Africa / Algeria / Algiers | Human | National SARS-CoV-2 genomic and variants surveillance program | Female | 27 | Live | unknown | National SARS-CoV-2 genomic and variants surveillance program | BA.5.2 | GRA |
| hCoV-19/Algeria/34960/2022 | EPI_ISL_15252865 | 21/08/2022 | Africa / Algeria / Algiers | Human | National SARS-CoV-2 genomic and variants surveillance program | Female | 27 | Live | unknown | National SARS-CoV-2 genomic and variants surveillance program | BA.5.2.44 | GRA |
| hCoV-19/Algeria/34930/2022 | EPI_ISL_15252866 | 20/08/2022 | Africa / Algeria / Algiers | Human | National SARS-CoV-2 genomic and variants surveillance program | Female | 28 | Live | unknown | National SARS-CoV-2 genomic and variants surveillance program | BA.5.2.27 | GRA |
| hCoV-19/Algeria/34446/2022 | EPI_ISL_15252867 | 15/08/2022 | Africa / Algeria / Algiers | Human | National SARS-CoV-2 genomic and variants surveillance program | Female | 30 | Live | unknown | National SARS-CoV-2 genomic and variants surveillance program | BF.2 | GRA |
| hCoV-19/Algeria/34825/2022 | EPI_ISL_15252868 | 15/08/2022 | Africa / Algeria / Tebessa | Human | National SARS-CoV-2 genomic and variants surveillance program | Male | 30 | Live | unknown | National SARS-CoV-2 genomic and variants surveillance program | BA.5.2 | GRA |
| hCoV-19/Algeria/34838/2022 | EPI_ISL_15252869 | 18/08/2022 | Africa / Algeria / Algiers | Human | National SARS-CoV-2 genomic and variants surveillance program | Female | 31 | Live | unknown | National SARS-CoV-2 genomic and variants surveillance program | BA.5.3.1 | GRA |
| hCoV-19/Algeria/34496/2022 | EPI_ISL_15252870 | 15/08/2022 | Africa / Algeria / Medea | Human | National SARS-CoV-2 genomic and variants surveillance program | Female | 33 | Live | unknown | National SARS-CoV-2 genomic and variants surveillance program | BA.5.2.1 | GRA |
| hCoV-19/Algeria/34818/2022 | EPI_ISL_15252871 | 17/08/2022 | Africa / Algeria / Algiers | Human | National SARS-CoV-2 genomic and variants surveillance program | Female | 34 | Live | unknown | National SARS-CoV-2 genomic and variants surveillance program | BA.5.2.1 | GRA |
| hCoV-19/Algeria/34860/2022 | EPI_ISL_15252872 | 21/08/2022 | Africa / Algeria / Medea | Human | National SARS-CoV-2 genomic and variants surveillance program | Female | 34 | Live | unknown | National SARS-CoV-2 genomic and variants surveillance program | BA.5.2.1 | GRA |
| hCoV-19/Algeria/34907/2022 | EPI_ISL_15252873 | 21/08/2022 | Africa / Algeria / Algiers | Human | National SARS-CoV-2 genomic and variants surveillance program | Female | 34 | Live | unknown | National SARS-CoV-2 genomic and variants surveillance program | BA.5.2.27 | GRA |
| hCoV-19/Algeria/34950/2022 | EPI_ISL_15252874 | 18/08/2022 | Africa / Algeria / Algiers | Human | National SARS-CoV-2 genomic and variants surveillance program | Female | 35 | Live | unknown | National SARS-CoV-2 genomic and variants surveillance program | BA.5.2 | GRA |
| hCoV-19/Algeria/34451/2022 | EPI_ISL_15252875 | 15/08/2022 | Africa / Algeria / Algiers | Human | National SARS-CoV-2 genomic and variants surveillance program | Female | 48 | Live | unknown | National SARS-CoV-2 genomic and variants surveillance program | BA.5.2.6 | GRA |
| hCoV-19/Algeria/35014/2022 | EPI_ISL_15252876 | 21/08/2022 | Africa / Algeria / Tebessa | Human | National SARS-CoV-2 genomic and variants surveillance program | Male | 54 | Live | unknown | National SARS-CoV-2 genomic and variants surveillance program | BA.5.2 | GRA |
| hCoV-19/Algeria/34823/2022 | EPI_ISL_15252877 | 18/08/2022 | Africa / Algeria / Algiers | Human | National SARS-CoV-2 genomic and variants surveillance program | Male | 58 | Live | unknown | National SARS-CoV-2 genomic and variants surveillance program | BA.5.5 | GRA |
| hCoV-19/Algeria/34794/2022 | EPI_ISL_15252878 | 17/08/2022 | Africa / Algeria / Algiers | Human | National SARS-CoV-2 genomic and variants surveillance program | Female | 59 | Live | unknown | National SARS-CoV-2 genomic and variants surveillance program | BA.5.2.1 | GRA |
| hCoV-19/Algeria/34712/2022 | EPI_ISL_15252879 | 16/08/2022 | Africa / Algeria / Bouira | Human | National SARS-CoV-2 genomic and variants surveillance program | Male | 67 | Live | unknown | National SARS-CoV-2 genomic and variants surveillance program | BA.5.2.20 | GRA |
| hCoV-19/Algeria/34450/2022 | EPI_ISL_15252880 | 15/08/2022 | Africa / Algeria / Algiers | Human | National SARS-CoV-2 genomic and variants surveillance program | Male | 68 | Live | unknown | National SARS-CoV-2 genomic and variants surveillance program | BA.5.2 | GRA |
| hCoV-19/Algeria/34828/2022 | EPI_ISL_15252881 | 16/08/2022 | Africa / Algeria / Tebessa | Human | National SARS-CoV-2 genomic and variants surveillance program | Female | 69 | Live | unknown | National SARS-CoV-2 genomic and variants surveillance program | BA.5.2 | GRA |
| hCoV-19/Algeria/34800/2022 | EPI_ISL_15252882 | 16/08/2022 | Africa / Algeria / Bouira | Human | National SARS-CoV-2 genomic and variants surveillance program | Female | 76 | Live | unknown | National SARS-CoV-2 genomic and variants surveillance program | BA.5.2 | GRA |
| hCoV-19/Algeria/35168/2022 | EPI_ISL_15252883 | 22/08/2022 | Africa / Algeria / Algiers | Human | National SARS-CoV-2 genomic and variants surveillance program | Male | 58 | Live | unknown | National SARS-CoV-2 genomic and variants surveillance program | BA.5.2 | GRA |
| hCoV-19/Algeria/34349/2022 | EPI_ISL_15349593 | 14/08/2022 | Africa / Algeria / Algiers | Human | National SARS-CoV-2 genomic and variants surveillance program | Female | 33 | Live | unknown | National SARS-CoV-2 genomic and variants surveillance program | BA.5.2 | GRA |
| hCoV-19/Algeria/34492/2022 | EPI_ISL_15349594 | 15/08/2022 | Africa / Algeria / Algiers | Human | National SARS-CoV-2 genomic and variants surveillance program | Male | 88 | Live | unknown | National SARS-CoV-2 genomic and variants surveillance program | BA.5.2.1 | GRA |
| hCoV-19/Algeria/34341/2022 | EPI_ISL_15349595 | 14/08/2022 | Africa / Algeria / Algiers | Human | National SARS-CoV-2 genomic and variants surveillance program | Male | 15 | Live | unknown | National SARS-CoV-2 genomic and variants surveillance program | BA.5.2 | GRA |
| hCoV-19/Algeria/34319/2022 | EPI_ISL_15349596 | 13/08/2022 | Africa / Algeria / Algiers | Human | National SARS-CoV-2 genomic and variants surveillance program | Female | 29 | Live | unknown | National SARS-CoV-2 genomic and variants surveillance program | BA.5.2.20 | GRA |
| hCoV-19/Algeria/34295/2022 | EPI_ISL_15349597 | 13/08/2022 | Africa / Algeria / Algiers | Human | National SARS-CoV-2 genomic and variants surveillance program | Female | 28 | Live | unknown | National SARS-CoV-2 genomic and variants surveillance program | BA.5.2.20 | GRA |
| hCoV-19/Algeria/34286/2022 | EPI_ISL_15349598 | 13/08/2022 | Africa / Algeria / Algiers | Human | National SARS-CoV-2 genomic and variants surveillance program | Female | 36 | Live | unknown | National SARS-CoV-2 genomic and variants surveillance program | BA.5.3.1 | GRA |
| hCoV-19/Algeria/34278/2022 | EPI_ISL_15349599 | 13/08/2022 | Africa / Algeria / Algiers | Human | National SARS-CoV-2 genomic and variants surveillance program | Female | 28 | Live | unknown | National SARS-CoV-2 genomic and variants surveillance program | BA.5.2 | GRA |
| hCoV-19/Algeria/34425/2022 | EPI_ISL_15349600 | 14/08/2022 | Africa / Algeria / Algiers | Human | National SARS-CoV-2 genomic and variants surveillance program | Female | 21 | Live | unknown | National SARS-CoV-2 genomic and variants surveillance program | BA.5.2 | GRA |
| hCoV-19/Algeria/34723/2022 | EPI_ISL_15349601 | 17/08/2022 | Africa / Algeria / Algiers | Human | National SARS-CoV-2 genomic and variants surveillance program | Male | 75 | Live | unknown | National SARS-CoV-2 genomic and variants surveillance program | BE.1 | GRA |
| hCoV-19/Algeria/34792/2022 | EPI_ISL_15349602 | 17/08/2022 | Africa / Algeria / Algiers | Human | National SARS-CoV-2 genomic and variants surveillance program | Female | 35 | Live | unknown | National SARS-CoV-2 genomic and variants surveillance program | BA.5.2 | GRA |
| hCoV-19/Algeria/34791/2022 | EPI_ISL_15349603 | 17/08/2022 | Africa / Algeria / Algiers | Human | National SARS-CoV-2 genomic and variants surveillance program | Male | 35 | Live | unknown | National SARS-CoV-2 genomic and variants surveillance program | BA.5.2 | GRA |
| hCoV-19/Algeria/34973/2022 | EPI_ISL_15349604 | 21/08/2022 | Africa / Algeria / Algiers | Human | National SARS-CoV-2 genomic and variants surveillance program | Male | 60 | Live | unknown | National SARS-CoV-2 genomic and variants surveillance program | BA.5.2 | GRA |
| hCoV-19/Algeria/34148/2022 | EPI_ISL_15349605 | 11/08/2022 | Africa / Algeria / Algiers | Human | National SARS-CoV-2 genomic and variants surveillance program | Male | 57 | Live | unknown | National SARS-CoV-2 genomic and variants surveillance program | BA.5.2.1 | GRA |
| hCoV-19/Algeria/34890/2022 | EPI_ISL_15349606 | 21/08/2022 | Africa / Algeria / Algiers | Human | National SARS-CoV-2 genomic and variants surveillance program | Male | unknown | Live | unknown | National SARS-CoV-2 genomic and variants surveillance program | BA.5.1.26 | GRA |
| hCoV-19/Algeria/34345/2022 | EPI_ISL_15349607 | 14/08/2022 | Africa / Algeria / Blida | Human | National SARS-CoV-2 genomic and variants surveillance program | Female | 44 | Live | unknown | National SARS-CoV-2 genomic and variants surveillance program | BA.5.2.20 | GRA |
| hCoV-19/Algeria/34196/2022 | EPI_ISL_15349608 | 22/08/2022 | Africa / Algeria / Blida | Human | National SARS-CoV-2 genomic and variants surveillance program | Female | 87 | Live | unknown | National SARS-CoV-2 genomic and variants surveillance program | BA.5.1 | GRA |
| hCoV-19/Algeria/34713/2022 | EPI_ISL_15349609 | 16/08/2022 | Africa / Algeria / Bouira | Human | National SARS-CoV-2 genomic and variants surveillance program | Female | 86 | Live | unknown | National SARS-CoV-2 genomic and variants surveillance program | BA.5.2 | GRA |
| hCoV-19/Algeria/34369/2022 | EPI_ISL_15349610 | 14/08/2022 | Africa / Algeria / Medea | Human | National SARS-CoV-2 genomic and variants surveillance program | Male | 75 | Live | unknown | National SARS-CoV-2 genomic and variants surveillance program | BA.5.2.27 | GRA |
| hCoV-19/Algeria/34327/2022 | EPI_ISL_15349611 | 14/08/2022 | Africa / Algeria / Medea | Human | National SARS-CoV-2 genomic and variants surveillance program | Male | 101 | Live | unknown | National SARS-CoV-2 genomic and variants surveillance program | BA.5.2 | GRA |
| hCoV-19/Algeria/34509/2022 | EPI_ISL_15349612 | 16/08/2022 | Africa / Algeria / Medea | Human | National SARS-CoV-2 genomic and variants surveillance program | Female | 79 | Live | unknown | National SARS-CoV-2 genomic and variants surveillance program | BA.5.2 | GRA |
| hCoV-19/Algeria/34490/2022 | EPI_ISL_15349613 | 15/08/2022 | Africa / Algeria / Medea | Human | National SARS-CoV-2 genomic and variants surveillance program | Male | 86 | Live | unknown | National SARS-CoV-2 genomic and variants surveillance program | BA.5.2.1 | GRA |
| hCoV-19/Algeria/34859/2022 | EPI_ISL_15349614 | 21/08/2022 | Africa / Algeria / Medea | Human | National SARS-CoV-2 genomic and variants surveillance program | Female | 98 | Live | unknown | National SARS-CoV-2 genomic and variants surveillance program | BA.5.1 | GRA |
| hCoV-19/Algeria/34830/2022 | EPI_ISL_15349615 | 16/08/2022 | Africa / Algeria / Tebessa | Human | National SARS-CoV-2 genomic and variants surveillance program | Male | 78 | Live | unknown | National SARS-CoV-2 genomic and variants surveillance program | BA.5.2 | GRA |
| hCoV-19/Algeria/37325/2022 | EPI_ISL_15392092 | 13/09/2022 | Africa / Algeria / Algiers | Human | National SARS-CoV-2 genomic and variants surveillance program | Female | 23 | Live | unknown | National SARS-CoV-2 genomic and variants surveillance program | BA.5.2.20 | GRA |
| hCoV-19/Algeria/38158/2022 | EPI_ISL_15392093 | 20/09/2022 | Africa / Algeria / Algiers | Human | National SARS-CoV-2 genomic and variants surveillance program | Female | 48 | Live | unknown | National SARS-CoV-2 genomic and variants surveillance program | BA.5.1 | GRA |
| hCoV-19/Algeria/38635/2022 | EPI_ISL_15392094 | 26/09/2022 | Africa / Algeria / Algiers | Human | National SARS-CoV-2 genomic and variants surveillance program | Male | 51 | Live | unknown | National SARS-CoV-2 genomic and variants surveillance program | BA.5.2.36 | GRA |
| hCoV-19/Algeria/28188/2022 | EPI_ISL_15393555 | 01/08/2022 | Africa / Algeria / Oran | Human | National SARS-CoV-2 genomic and variants surveillance program | Female | 38 | Live | unknown | National SARS-CoV-2 genomic and variants surveillance program | BA.5.2 | GRA |
| hCoV-19/Algeria/28262/2022 | EPI_ISL_15393556 | 07/08/2022 | Africa / Algeria / Oran | Human | National SARS-CoV-2 genomic and variants surveillance program | Female | 41 | Live | unknown | National SARS-CoV-2 genomic and variants surveillance program | BA.5.2 | GRA |
| hCoV-19/Algeria/28276/2022 | EPI_ISL_15393557 | 08/08/2022 | Africa / Algeria / Oran | Human | National SARS-CoV-2 genomic and variants surveillance program | Female | 59 | Live | unknown | National SARS-CoV-2 genomic and variants surveillance program | BA.5.2.27 | GRA |
| hCoV-19/Algeria/28285/2022 | EPI_ISL_15393558 | 09/08/2022 | Africa / Algeria / Oran | Human | National SARS-CoV-2 genomic and variants surveillance program | Male | 23 | Live | unknown | National SARS-CoV-2 genomic and variants surveillance program | BA.5.2 | GRA |
| hCoV-19/Algeria/28291/2022 | EPI_ISL_15393559 | 09/08/2022 | Africa / Algeria / Oran | Human | National SARS-CoV-2 genomic and variants surveillance program | Female | 25 | Live | unknown | National SARS-CoV-2 genomic and variants surveillance program | BA.5.1 | GRA |
| hCoV-19/Algeria/28300/2022 | EPI_ISL_15393560 | 09/08/2022 | Africa / Algeria / Oran | Human | National SARS-CoV-2 genomic and variants surveillance program | Male | 77 | Live | unknown | National SARS-CoV-2 genomic and variants surveillance program | BA.5.1 | GRA |
| hCoV-19/Algeria/819/2022 | EPI_ISL_15498988 | 31/07/2022 | Africa / Algeria / Mâ€™SILA | Human | National SARS-CoV-2 genomic and variants surveillance program | Female | 51 | Live | unknown | National SARS-CoV-2 genomic and variants surveillance program | BA.5.2.1 | GRA |
| hCoV-19/Algeria/40153/2022 | EPI_ISL_15637960 | 12/10/2022 | Africa / Algeria / Algiers | Human | National SARS-CoV-2 genomic and variants surveillance program | Female | unknown | Live | unknown | National SARS-CoV-2 genomic and variants surveillance program | BA.2.3.20 | GRA |
| hCoV-19/Algeria/39419/2022 | EPI_ISL_15637961 | 05/10/2022 | Africa / Algeria / Algiers | Human | National SARS-CoV-2 genomic and variants surveillance program | Female | 47 | Live | unknown | National SARS-CoV-2 genomic and variants surveillance program | BQ.1 | GRA |
| hCoV-19/Algeria/39226/2022 | EPI_ISL_15637962 | 04/10/2022 | Africa / Algeria / Algiers | Human | National SARS-CoV-2 genomic and variants surveillance program | Male | 64 | Live | unknown | National SARS-CoV-2 genomic and variants surveillance program | BQ.1.1 | GRA |
| hCoV-19/Algeria/40266/2022 | EPI_ISL_15637963 | 15/10/2022 | Africa / Algeria / Algiers | Human | National SARS-CoV-2 genomic and variants surveillance program | Male | 40 | Live | unknown | National SARS-CoV-2 genomic and variants surveillance program | BA.2.3.20 | GRA |
| hCoV-19/Algeria/40231/2022 | EPI_ISL_15637964 | 15/10/2022 | Africa / Algeria / Algiers | Human | National SARS-CoV-2 genomic and variants surveillance program | Male | 69 | Live | unknown | National SARS-CoV-2 genomic and variants surveillance program | XBB.1 | GRA |
| hCoV-19/Algeria/28497/2022 | EPI_ISL_15637965 | 08/09/2022 | Africa / Algeria / Oran | Human | National SARS-CoV-2 genomic and variants surveillance program | Male | 31 | Live | unknown | National SARS-CoV-2 genomic and variants surveillance program | BA.5.1 | GRA |
| hCoV-19/Algeria/28390/2022 | EPI_ISL_15637966 | 18/08/2022 | Africa / Algeria / Oran | Human | National SARS-CoV-2 genomic and variants surveillance program | Male | 42 | Live | unknown | National SARS-CoV-2 genomic and variants surveillance program | BA.5.2 | GRA |
| hCoV-19/Algeria/28376/2022 | EPI_ISL_15637967 | 17/08/2022 | Africa / Algeria / Oran | Human | National SARS-CoV-2 genomic and variants surveillance program | Male | 30 | Live | unknown | National SARS-CoV-2 genomic and variants surveillance program | BA.5.2 | GRA |
| hCoV-19/Algeria/2866/2022 | EPI_ISL_15637968 | 07/08/2022 | Africa / Algeria / Sidi Bel Abbes | Human | National SARS-CoV-2 genomic and variants surveillance program | Male | unknown | Live | unknown | National SARS-CoV-2 genomic and variants surveillance program | BA.5.2 | GRA |
| hCoV-19/Algeria/2827/2022 | EPI_ISL_15637969 | 31/07/2022 | Africa / Algeria / Sidi Bel Abbes | Human | National SARS-CoV-2 genomic and variants surveillance program | Male | unknown | Live | unknown | National SARS-CoV-2 genomic and variants surveillance program | BA.5.2.1 | GRA |
| hCoV-19/Algeria/2795/2022 | EPI_ISL_15637970 | 31/07/2022 | Africa / Algeria / Sidi Bel Abbes | Human | National SARS-CoV-2 genomic and variants surveillance program | Female | 50 | Live | unknown | National SARS-CoV-2 genomic and variants surveillance program | BA.5.1 | GRA |
| hCoV-19/Algeria/02771/2022 | EPI_ISL_15637971 | 27/07/2022 | Africa / Algeria / Sidi Bel Abbes | Human | National SARS-CoV-2 genomic and variants surveillance program | Female | 72 | Live | unknown | National SARS-CoV-2 genomic and variants surveillance program | BA.5.6 | GRA |
| hCoV-19/Algeria/2769/2022 | EPI_ISL_15637972 | 27/07/2022 | Africa / Algeria / Sidi Bel Abbes | Human | National SARS-CoV-2 genomic and variants surveillance program | Female | 27 | Live | unknown | National SARS-CoV-2 genomic and variants surveillance program | BA.5.2 | GRA |
| hCoV-19/Algeria/2864/2022 | EPI_ISL_15637973 | 07/08/2022 | Africa / Algeria / Sidi Bel Abbes | Human | National SARS-CoV-2 genomic and variants surveillance program | Male | 29 | Live | unknown | National SARS-CoV-2 genomic and variants surveillance program | BA.5.1 | GRA |
| hCoV-19/Algeria/2702/2022 | EPI_ISL_15637974 | 24/07/2022 | Africa / Algeria / Sidi Bel Abbes | Human | National SARS-CoV-2 genomic and variants surveillance program | Male | 17 | Live | unknown | National SARS-CoV-2 genomic and variants surveillance program | BA.2.12.1 | GRA |
| hCoV-19/Algeria/39438/2022 | EPI_ISL_15790691 | 04/10/2022 | Africa / Algeria / Blida | Human | National SARS-CoV-2 genomic and variants surveillance program | Male | 70 | Live | unknown | National SARS-CoV-2 genomic and variants surveillance program | BA.5.2 | GRA |
| hCoV-19/Algeria/39718/2022 | EPI_ISL_15790692 | 08/10/2022 | Africa / Algeria / Algiers | Human | National SARS-CoV-2 genomic and variants surveillance program | Male | 57 | Live | unknown | National SARS-CoV-2 genomic and variants surveillance program | BQ.1.1 | GRA |
| hCoV-19/Algeria/39710/2022 | EPI_ISL_15790693 | 08/10/2022 | Africa / Algeria / Algiers | Human | National SARS-CoV-2 genomic and variants surveillance program | Female | 31 | Live | unknown | National SARS-CoV-2 genomic and variants surveillance program | BQ.1.1 | GRA |
| hCoV-19/Algeria/40160/2022 | EPI_ISL_15790694 | 12/10/2022 | Africa / Algeria / Blida | Human | National SARS-CoV-2 genomic and variants surveillance program | Male | 54 | Live | unknown | National SARS-CoV-2 genomic and variants surveillance program | BQ.1.5 | GRA |
| hCoV-19/Algeria/41062/2022 | EPI_ISL_15790695 | 24/10/2022 | Africa / Algeria / Algiers | Human | National SARS-CoV-2 genomic and variants surveillance program | Female | 76 | Live | unknown | National SARS-CoV-2 genomic and variants surveillance program | BA.5.1 | GRA |
| hCoV-19/Algeria/704/2022 | EPI_ISL_15790696 | 08/08/2022 | Africa / Algeria / El Tarf | Human | National SARS-CoV-2 genomic and variants surveillance program | Female | 30 | Live | unknown | National SARS-CoV-2 genomic and variants surveillance program | BA.5.2.44 | GRA |
| hCoV-19/Algeria/706/2022 | EPI_ISL_15790697 | 10/08/2022 | Africa / Algeria / El Tarf | Human | National SARS-CoV-2 genomic and variants surveillance program | Female | 40 | Live | unknown | National SARS-CoV-2 genomic and variants surveillance program | BA.5.2 | GRA |
| hCoV-19/Algeria/709/2022 | EPI_ISL_15790698 | 10/08/2022 | Africa / Algeria / El Tarf | Human | National SARS-CoV-2 genomic and variants surveillance program | Male | 41 | Live | unknown | National SARS-CoV-2 genomic and variants surveillance program | BA.5.1 | GRA |
| hCoV-19/Algeria/710/2022 | EPI_ISL_15790699 | 10/08/2022 | Africa / Algeria / El Tarf | Human | National SARS-CoV-2 genomic and variants surveillance program | Female | 72 | Live | unknown | National SARS-CoV-2 genomic and variants surveillance program | BA.5.2.20 | GRA |
| hCoV-19/Algeria/727/2022 | EPI_ISL_15790700 | 18/08/2022 | Africa / Algeria / El Tarf | Human | National SARS-CoV-2 genomic and variants surveillance program | Female | 41 | Live | unknown | National SARS-CoV-2 genomic and variants surveillance program | BA.2.38 | GRA |
| hCoV-19/Algeria/41915/2022 | EPI_ISL_15887349 | 03/11/2022 | Africa / Algeria / Algiers | Human | unknown | Male | 77 | Live | unknown | unknown | BE.1 | GRA |
| hCoV-19/Algeria/42132/2022 | EPI_ISL_15887350 | 07/11/2022 | Africa / Algeria / Blida | Human | unknown | Female | 78 | Live | unknown | unknown | BQ.1.5 | GRA |
| hCoV-19/Algeria/42133/2022 | EPI_ISL_15887351 | 07/11/2022 | Africa / Algeria / Blida | Human | unknown | Male | 90 | Live | unknown | unknown | BQ.1.1 | GRA |
| hCoV-19/Algeria/603/2022 | EPI_ISL_15887352 | 26/06/2022 | Africa / Algeria / Annaba | Human | unknown | Female | 75 | Live | unknown | unknown | BA.5.2.1 | GRA |
| hCoV-19/Algeria/633/2022 | EPI_ISL_15887353 | 13/07/2022 | Africa / Algeria / El Tarf | Human | unknown | Male | 51 | Live | unknown | unknown | BA.5.2.44 | GRA |
| hCoV-19/Algeria/634/2022 | EPI_ISL_15887354 | 14/07/2022 | Africa / Algeria / El Tarf | Human | unknown | Male | 40 | Live | unknown | unknown | BA.5.2 | GRA |
| hCoV-19/Algeria/639/2022 | EPI_ISL_15887355 | 17/07/2022 | Africa / Algeria / El Tarf | Human | unknown | Female | 41 | Live | unknown | unknown | BA.5.5 | GRA |
| hCoV-19/Algeria/653/2022 | EPI_ISL_15887356 | 20/07/2022 | Africa / Algeria / El Tarf | Human | unknown | Female | 58 | Live | unknown | unknown | BA.5.2 | GRA |
| hCoV-19/Algeria/664/2022 | EPI_ISL_15887357 | 25/07/2022 | Africa / Algeria / El Tarf | Human | unknown | Male | 29 | Live | unknown | unknown | BA.5.2.20 | GRA |
| hCoV-19/Algeria/681/2022 | EPI_ISL_15887358 | 31/07/2022 | Africa / Algeria / El Tarf | Human | unknown | Female | 34 | Live | unknown | unknown | BA.5.1 | GRA |
| hCoV-19/Algeria/683/2022 | EPI_ISL_15887359 | 31/07/2022 | Africa / Algeria / El Tarf | Human | unknown | Female | 34 | Live | unknown | unknown | BA.5.2.20 | GRA |
| hCoV-19/Algeria/685/2022 | EPI_ISL_15887360 | 01/08/2022 | Africa / Algeria / El Tarf | Human | unknown | Female | 75 | Live | unknown | unknown | BA.5.2.44 | GRA |
| hCoV-19/Algeria/692/2022 | EPI_ISL_15887361 | 03/07/2022 | Africa / Algeria / El Tarf | Human | unknown | Female | 30 | Live | unknown | unknown | BA.4 | GRA |
| hCoV-19/Algeria/698/2022 | EPI_ISL_15887362 | 08/08/2022 | Africa / Algeria / El Tarf | Human | unknown | Female | 27 | Live | unknown | unknown | BA.5.2.20 | GRA |
| hCoV-19/Algeria/2820/2022 | EPI_ISL_15887363 | 31/07/2022 | Africa / Algeria / Tlemcen | Human | unknown | Male | 61 | Live | unknown | unknown | BE.1 | GRA |
| hCoV-19/Algeria/2832/2022 | EPI_ISL_15887364 | 04/08/2022 | Africa / Algeria / Tlemcen | Human | unknown | Male | 71 | Live | unknown | unknown | BA.5.2 | GRA |
| hCoV-19/Algeria/950/2022 | EPI_ISL_15887365 | 04/10/2022 | Africa / Algeria / Sidi Bel Abbes | Human | unknown | Male | 45 | Live | unknown | unknown | BQ.1.1 | GRA |
| hCoV-19/Algeria/2839/2022 | EPI_ISL_15887366 | 08/08/2022 | Africa / Algeria / Tlemcen | Human | unknown | Female | 27 | Live | unknown | unknown | BA.5.2.16 | GRA |
| hCoV-19/Algeria/3395/2022 | EPI_ISL_15887367 | 12/10/2022 | Africa / Algeria / Sidi Bel Abbes | Human | unknown | Male | 71 | Live | unknown | unknown | BQ.1.1 | GRA |
| hCoV-19/Algeria/1421/2022 | EPI_ISL_15920753 | 25/09/2022 | Africa / Algeria / Setif | Human | Same-patient sampling strategy | Male | 48 | Live | unknown | Same-patient sampling strategy | BA.2 | GR |
| hCoV-19/Algeria/1409/2022 | EPI_ISL_15920754 | 05/10/2022 | Africa / Algeria / Setif | Human | Same-patient sampling strategy | Male | 48 | Live | unknown | Same-patient sampling strategy | BA.2 | GR |
| hCoV-19/Algeria/1388/2022 | EPI_ISL_15920755 | 20/10/2022 | Africa / Algeria / Setif | Human | Same-patient sampling strategy | Male | 48 | Live | unknown | Same-patient sampling strategy | BA.2 | GR |
| hCoV-19/Algeria/1999/2020 | EPI_ISL_15928044 | 19/03/2020 | Africa / Algeria / Blida | Human | National SARS-CoV-2 genomic and variants surveillance program | Male | 32 | Live | unknown | National SARS-CoV-2 genomic and variants surveillance program | B.1 | GH |
| hCoV-19/Algeria/2002/2020 | EPI_ISL_15928045 | 19/03/2020 | Africa / Algeria / Blida | Human | National SARS-CoV-2 genomic and variants surveillance program | Female | 16 | Live | unknown | National SARS-CoV-2 genomic and variants surveillance program | B.1 | GH |
| hCoV-19/Algeria/2041/2020 | EPI_ISL_15928046 | 19/03/2020 | Africa / Algeria / Blida | Human | National SARS-CoV-2 genomic and variants surveillance program | Female | 43 | Live | unknown | National SARS-CoV-2 genomic and variants surveillance program | B.1.597 | GH |
| hCoV-19/Algeria/2045/2020 | EPI_ISL_15928047 | 19/03/2020 | Africa / Algeria / Blida | Human | National SARS-CoV-2 genomic and variants surveillance program | Female | 38 | Live | unknown | National SARS-CoV-2 genomic and variants surveillance program | B.1 | GH |
| hCoV-19/Algeria/2826/2020 | EPI_ISL_15928048 | 23/03/2020 | Africa / Algeria / Algiers | Human | National SARS-CoV-2 genomic and variants surveillance program | Male | 65 | Live | unknown | National SARS-CoV-2 genomic and variants surveillance program | B.1 | GH |
| hCoV-19/Algeria/2999/2020 | EPI_ISL_15928049 | 24/03/2020 | Africa / Algeria / Blida | Human | National SARS-CoV-2 genomic and variants surveillance program | Female | 48 | Live | unknown | National SARS-CoV-2 genomic and variants surveillance program | B.1 | GH |
| hCoV-19/Algeria/3091/2020 | EPI_ISL_15928050 | 25/03/2020 | Africa / Algeria / Blida | Human | National SARS-CoV-2 genomic and variants surveillance program | Male | 38 | Live | unknown | National SARS-CoV-2 genomic and variants surveillance program | B.1 | GH |
| hCoV-19/Algeria/3449/2020 | EPI_ISL_15928051 | 27/03/2020 | Africa / Algeria / Blida | Human | National SARS-CoV-2 genomic and variants surveillance program | Male | 76 | Live | unknown | National SARS-CoV-2 genomic and variants surveillance program | B.1 | GH |
| hCoV-19/Algeria/3465/2020 | EPI_ISL_15928052 | 27/03/2020 | Africa / Algeria / Blida | Human | National SARS-CoV-2 genomic and variants surveillance program | Male | 61 | Live | unknown | National SARS-CoV-2 genomic and variants surveillance program | B.1 | GH |
| hCoV-19/Algeria/3726/2020 | EPI_ISL_15928053 | 29/03/2020 | Africa / Algeria / Blida | Human | National SARS-CoV-2 genomic and variants surveillance program | Male | 67 | Live | unknown | National SARS-CoV-2 genomic and variants surveillance program | B.1 | GH |
| hCoV-19/Algeria/3731/2020 | EPI_ISL_15928054 | 29/03/2020 | Africa / Algeria / Blida | Human | National SARS-CoV-2 genomic and variants surveillance program | Male | 72 | Live | unknown | National SARS-CoV-2 genomic and variants surveillance program | B.1 | GH |
| hCoV-19/Algeria/3747/2020 | EPI_ISL_15928055 | 29/03/2020 | Africa / Algeria / Blida | Human | National SARS-CoV-2 genomic and variants surveillance program | Female | 80 | Live | unknown | National SARS-CoV-2 genomic and variants surveillance program | B.1.597 | GH |
| hCoV-19/Algeria/9822/2020 | EPI_ISL_15928056 | 26/04/2020 | Africa / Algeria / Blida | Human | National SARS-CoV-2 genomic and variants surveillance program | Female | 60 | Live | unknown | National SARS-CoV-2 genomic and variants surveillance program | B.1 | GH |
| hCoV-19/Algeria/10803/2020 | EPI_ISL_15928057 | 29/04/2020 | Africa / Algeria / Algiers | Human | National SARS-CoV-2 genomic and variants surveillance program | Female | 61 | Live | unknown | National SARS-CoV-2 genomic and variants surveillance program | B.1 | GH |
| hCoV-19/Algeria/11024/2020 | EPI_ISL_15928058 | 01/05/2020 | Africa / Algeria / Algiers | Human | National SARS-CoV-2 genomic and variants surveillance program | Male | 65 | Live | unknown | National SARS-CoV-2 genomic and variants surveillance program | B.1.597 | GH |
| hCoV-19/Algeria/11035/2020 | EPI_ISL_15928059 | 30/04/2020 | Africa / Algeria / Algiers | Human | National SARS-CoV-2 genomic and variants surveillance program | Female | 56 | Live | unknown | National SARS-CoV-2 genomic and variants surveillance program | B.1 | GH |
| hCoV-19/Algeria/11404/2020 | EPI_ISL_15928060 | 01/05/2020 | Africa / Algeria / Algiers | Human | National SARS-CoV-2 genomic and variants surveillance program | Male | 34 | Live | unknown | National SARS-CoV-2 genomic and variants surveillance program | B.1.597 | GH |
| hCoV-19/Algeria/11713/2020 | EPI_ISL_15928061 | 02/05/2020 | Africa / Algeria / Blida | Human | National SARS-CoV-2 genomic and variants surveillance program | Male | 67 | Live | unknown | National SARS-CoV-2 genomic and variants surveillance program | B.1 | GH |
| hCoV-19/Algeria/11755/2020 | EPI_ISL_15928062 | 02/05/2020 | Africa / Algeria / Algiers | Human | National SARS-CoV-2 genomic and variants surveillance program | Male | 60 | Live | unknown | National SARS-CoV-2 genomic and variants surveillance program | B.1.597 | GH |
| hCoV-19/Algeria/12328/2020 | EPI_ISL_15928063 | 04/05/2020 | Africa / Algeria / Tipaza | Human | National SARS-CoV-2 genomic and variants surveillance program | Male | 53 | Live | unknown | National SARS-CoV-2 genomic and variants surveillance program | B.1 | GH |
| hCoV-19/Algeria/13852/2020 | EPI_ISL_15928064 | 08/05/2020 | Africa / Algeria / Blida | Human | National SARS-CoV-2 genomic and variants surveillance program | Male | 36 | Live | unknown | National SARS-CoV-2 genomic and variants surveillance program | B.1.597 | GH |
| hCoV-19/Algeria/15603/2020 | EPI_ISL_15928065 | 12/05/2020 | Africa / Algeria / Algiers | Human | National SARS-CoV-2 genomic and variants surveillance program | Female | 27 | Live | unknown | National SARS-CoV-2 genomic and variants surveillance program | B.1 | G |
| hCoV-19/Algeria/15741/2020 | EPI_ISL_15928066 | 15/05/2020 | Africa / Algeria / Algiers | Human | National SARS-CoV-2 genomic and variants surveillance program | Female | 30 | Live | unknown | National SARS-CoV-2 genomic and variants surveillance program | B.1 | GH |
| hCoV-19/Algeria/16347/2020 | EPI_ISL_15928067 | 14/05/2020 | Africa / Algeria / Blida | Human | National SARS-CoV-2 genomic and variants surveillance program | Male | 29 | Live | unknown | National SARS-CoV-2 genomic and variants surveillance program | B.1 | GH |
| hCoV-19/Algeria/16348/2020 | EPI_ISL_15928068 | 14/05/2020 | Africa / Algeria / Blida | Human | National SARS-CoV-2 genomic and variants surveillance program | Male | 72 | Live | unknown | National SARS-CoV-2 genomic and variants surveillance program | B.1.597 | GH |
| hCoV-19/Algeria/17808/2020 | EPI_ISL_15928069 | 16/05/2020 | Africa / Algeria / Algiers | Human | National SARS-CoV-2 genomic and variants surveillance program | Male | 37 | Live | unknown | National SARS-CoV-2 genomic and variants surveillance program | B.1 | G |
| hCoV-19/Algeria/23488/2020 | EPI_ISL_15928070 | 28/05/2020 | Africa / Algeria / Algiers | Human | National SARS-CoV-2 genomic and variants surveillance program | Female | 46 | Live | unknown | National SARS-CoV-2 genomic and variants surveillance program | B.1 | GH |
| hCoV-19/Algeria/37385/2020 | EPI_ISL_15928071 | 19/06/2020 | Africa / Algeria / Algiers | Human | National SARS-CoV-2 genomic and variants surveillance program | Male | 34 | Live | unknown | National SARS-CoV-2 genomic and variants surveillance program | B.1.1 | GR |
| hCoV-19/Algeria/45522/2020 | EPI_ISL_15928072 | 30/06/2020 | Africa / Algeria / Algiers | Human | National SARS-CoV-2 genomic and variants surveillance program | Male | 40 | Live | unknown | National SARS-CoV-2 genomic and variants surveillance program | B.1.597 | GH |
| hCoV-19/Algeria/45537/2020 | EPI_ISL_15928073 | 30/06/2020 | Africa / Algeria / Algiers | Human | National SARS-CoV-2 genomic and variants surveillance program | Male | 45 | Live | unknown | National SARS-CoV-2 genomic and variants surveillance program | B.1.597 | GH |
| hCoV-19/Algeria/45542/2020 | EPI_ISL_15928074 | 30/06/2020 | Africa / Algeria / Algiers | Human | National SARS-CoV-2 genomic and variants surveillance program | Female | 23 | Live | unknown | National SARS-CoV-2 genomic and variants surveillance program | B.1.597 | GH |
| hCoV-19/Algeria/45555/2020 | EPI_ISL_15928075 | 30/06/2020 | Africa / Algeria / Algiers | Human | National SARS-CoV-2 genomic and variants surveillance program | Male | 25 | Live | unknown | National SARS-CoV-2 genomic and variants surveillance program | B.1.597 | GH |
| hCoV-19/Algeria/45562/2020 | EPI_ISL_15928076 | 30/06/2020 | Africa / Algeria / Algiers | Human | National SARS-CoV-2 genomic and variants surveillance program | Male | 40 | Live | unknown | National SARS-CoV-2 genomic and variants surveillance program | B.1.597 | GH |
| hCoV-19/Algeria/45953/2020 | EPI_ISL_15928077 | 01/07/2020 | Africa / Algeria / Algiers | Human | National SARS-CoV-2 genomic and variants surveillance program | Female | 67 | Live | unknown | National SARS-CoV-2 genomic and variants surveillance program | B.1.597 | GH |
| hCoV-19/Algeria/48642/2020 | EPI_ISL_15928078 | 05/07/2020 | Africa / Algeria / Algiers | Human | National SARS-CoV-2 genomic and variants surveillance program | Female | 4 | Live | unknown | National SARS-CoV-2 genomic and variants surveillance program | B.1 | GH |
| hCoV-19/Algeria/49081/2020 | EPI_ISL_15928079 | 03/07/2020 | Africa / Algeria / Blida | Human | National SARS-CoV-2 genomic and variants surveillance program | Male | 59 | Live | unknown | National SARS-CoV-2 genomic and variants surveillance program | B.1.597 | GH |
| hCoV-19/Algeria/49082/2020 | EPI_ISL_15928080 | 03/07/2020 | Africa / Algeria / Blida | Human | National SARS-CoV-2 genomic and variants surveillance program | Male | 35 | Live | unknown | National SARS-CoV-2 genomic and variants surveillance program | B.1.597 | GH |
| hCoV-19/Algeria/50627/2020 | EPI_ISL_15928081 | 06/07/2020 | Africa / Algeria / Algiers | Human | National SARS-CoV-2 genomic and variants surveillance program | Female | 42 | Live | unknown | National SARS-CoV-2 genomic and variants surveillance program | B.1.597 | GH |
| hCoV-19/Algeria/54251/2020 | EPI_ISL_15928082 | 10/07/2020 | Africa / Algeria / Algiers | Human | National SARS-CoV-2 genomic and variants surveillance program | Male | 40 | Live | unknown | National SARS-CoV-2 genomic and variants surveillance program | B.1.597 | GH |
| hCoV-19/Algeria/71348/2020 | EPI_ISL_15928083 | 05/08/2020 | Africa / Algeria / Algiers | Human | National SARS-CoV-2 genomic and variants surveillance program | Male | 41 | Live | unknown | National SARS-CoV-2 genomic and variants surveillance program | B.1.597 | GH |
| hCoV-19/Algeria/91082/2020 | EPI_ISL_15928084 | 20/09/2020 | Africa / Algeria / Bouira | Human | National SARS-CoV-2 genomic and variants surveillance program | Male | 92 | Live | unknown | National SARS-CoV-2 genomic and variants surveillance program | B.1.160 | GH |
| hCoV-19/Algeria/91199/2020 | EPI_ISL_15928085 | 20/09/2020 | Africa / Algeria / Algiers | Human | National SARS-CoV-2 genomic and variants surveillance program | Female | 43 | Live | unknown | National SARS-CoV-2 genomic and variants surveillance program | B.1.1 | GR |
| hCoV-19/Algeria/95763/2020 | EPI_ISL_15928086 | 30/09/2020 | Africa / Algeria / Blida | Human | National SARS-CoV-2 genomic and variants surveillance program | Female | 74 | Live | unknown | National SARS-CoV-2 genomic and variants surveillance program | B.1.597 | GH |
| hCoV-19/Algeria/100749/2020 | EPI_ISL_15928087 | 13/10/2020 | Africa / Algeria / Algiers | Human | National SARS-CoV-2 genomic and variants surveillance program | Male | 38 | Live | unknown | National SARS-CoV-2 genomic and variants surveillance program | B.1.597 | GH |
| hCoV-19/Algeria/100756/2020 | EPI_ISL_15928088 | 13/10/2020 | Africa / Algeria / Algiers | Human | National SARS-CoV-2 genomic and variants surveillance program | Female | 52 | Live | unknown | National SARS-CoV-2 genomic and variants surveillance program | B.1.160 | GH |
| hCoV-19/Algeria/123792/2020 | EPI_ISL_15928089 | 16/11/2020 | Africa / Algeria / Algiers | Human | National SARS-CoV-2 genomic and variants surveillance program | Female | 26 | Live | unknown | National SARS-CoV-2 genomic and variants surveillance program | B.1.160 | GH |
| hCoV-19/Algeria/129498/2020 | EPI_ISL_15928090 | 24/11/2020 | Africa / Algeria / Algiers | Human | National SARS-CoV-2 genomic and variants surveillance program | Male | 60 | Live | unknown | National SARS-CoV-2 genomic and variants surveillance program | B.1.160 | GH |
| hCoV-19/Algeria/129519/2020 | EPI_ISL_15928091 | 24/11/2020 | Africa / Algeria / Algiers | Human | National SARS-CoV-2 genomic and variants surveillance program | Male | 76 | Live | unknown | National SARS-CoV-2 genomic and variants surveillance program | B.1.597 | GH |
| hCoV-19/Algeria/129525/2020 | EPI_ISL_15928092 | 24/11/2020 | Africa / Algeria / Algiers | Human | National SARS-CoV-2 genomic and variants surveillance program | Male | 50 | Live | unknown | National SARS-CoV-2 genomic and variants surveillance program | B.1 | G |
| hCoV-19/Algeria/131369/2020 | EPI_ISL_15928093 | 29/11/2020 | Africa / Algeria / Bouira | Human | National SARS-CoV-2 genomic and variants surveillance program | Male | 72 | Live | unknown | National SARS-CoV-2 genomic and variants surveillance program | B.1.1.317 | GR |
| hCoV-19/Algeria/132094/2020 | EPI_ISL_15928094 | 30/11/2020 | Africa / Algeria / Algiers | Human | National SARS-CoV-2 genomic and variants surveillance program | Female | 30 | Live | unknown | National SARS-CoV-2 genomic and variants surveillance program | B.1.1 | GR |
| hCoV-19/Algeria/134933/2020 | EPI_ISL_15928095 | 06/12/2020 | Africa / Algeria / Algiers | Human | National SARS-CoV-2 genomic and variants surveillance program | Male | 28 | Live | unknown | National SARS-CoV-2 genomic and variants surveillance program | B.1.160 | GH |
| hCoV-19/Algeria/135160/2020 | EPI_ISL_15928096 | 07/12/2020 | Africa / Algeria / Algiers | Human | National SARS-CoV-2 genomic and variants surveillance program | Male | 29 | Live | unknown | National SARS-CoV-2 genomic and variants surveillance program | B.1.1 | GR |
| hCoV-19/Algeria/135656/2020 | EPI_ISL_15928097 | 07/12/2020 | Africa / Algeria / Bouira | Human | National SARS-CoV-2 genomic and variants surveillance program | Female | 26 | Live | unknown | National SARS-CoV-2 genomic and variants surveillance program | B.1.1 | GR |
| hCoV-19/Algeria/135661/2020 | EPI_ISL_15928098 | 07/12/2020 | Africa / Algeria / Bouira | Human | National SARS-CoV-2 genomic and variants surveillance program | Male | 51 | Live | unknown | National SARS-CoV-2 genomic and variants surveillance program | B.1.160 | GH |
| hCoV-19/Algeria/137385/2020 | EPI_ISL_15928099 | 13/12/2020 | Africa / Algeria / Bouira | Human | National SARS-CoV-2 genomic and variants surveillance program | Male | 87 | Live | unknown | National SARS-CoV-2 genomic and variants surveillance program | B.1.597 | GH |
| hCoV-19/Algeria/2347/2021 | EPI_ISL_15928100 | 07/01/2021 | Africa / Algeria / Algiers | Human | National SARS-CoV-2 genomic and variants surveillance program | Female | 13 | Live | unknown | National SARS-CoV-2 genomic and variants surveillance program | B.1.597 | GH |
| hCoV-19/Algeria/8557/2021 | EPI_ISL_15928101 | 27/01/2021 | Africa / Algeria / Algiers | Human | National SARS-CoV-2 genomic and variants surveillance program | Male | 38 | Live | unknown | National SARS-CoV-2 genomic and variants surveillance program | B.1.160 | GH |
| hCoV-19/Algeria/8664/2021 | EPI_ISL_15928102 | 28/01/2021 | Africa / Algeria / Algiers | Human | National SARS-CoV-2 genomic and variants surveillance program | Male | 38 | Live | unknown | National SARS-CoV-2 genomic and variants surveillance program | B.1.160 | GH |
| hCoV-19/Algeria/9791/2021 | EPI_ISL_15928103 | 01/02/2021 | Africa / Algeria / Algiers | Human | National SARS-CoV-2 genomic and variants surveillance program | Female | 73 | Live | unknown | National SARS-CoV-2 genomic and variants surveillance program | B.1 | G |
| hCoV-19/Algeria/9825/2021 | EPI_ISL_15928104 | 01/02/2021 | Africa / Algeria / Algiers | Human | National SARS-CoV-2 genomic and variants surveillance program | Female | 49 | Live | unknown | National SARS-CoV-2 genomic and variants surveillance program | L.3 | GR |
| hCoV-19/Algeria/9902/2021 | EPI_ISL_15928105 | 01/02/2021 | Africa / Algeria / Algiers | Human | National SARS-CoV-2 genomic and variants surveillance program | Female | 54 | Live | unknown | National SARS-CoV-2 genomic and variants surveillance program | B.1.597 | GH |
| hCoV-19/Algeria/13421/2021 | EPI_ISL_15928106 | 11/02/2021 | Africa / Algeria / Algiers | Human | National SARS-CoV-2 genomic and variants surveillance program | Male | 53 | Live | unknown | National SARS-CoV-2 genomic and variants surveillance program | B.1.160 | GH |
| hCoV-19/Algeria/16697/2021 | EPI_ISL_15928107 | 24/02/2021 | Africa / Algeria / Algiers | Human | National SARS-CoV-2 genomic and variants surveillance program | Male | 60 | Live | unknown | National SARS-CoV-2 genomic and variants surveillance program | B.1.525 | G |
| hCoV-19/Algeria/24037/2021 | EPI_ISL_15928108 | 25/03/2021 | Africa / Algeria / Algiers | Human | National SARS-CoV-2 genomic and variants surveillance program | Female | 84 | Live | unknown | National SARS-CoV-2 genomic and variants surveillance program | B.1.525 | G |
| hCoV-19/Algeria/29996/2021 | EPI_ISL_15928109 | 18/04/2021 | Africa / Algeria / Medea | Human | National SARS-CoV-2 genomic and variants surveillance program | Female | 46 | Live | unknown | National SARS-CoV-2 genomic and variants surveillance program | B.1.525 | G |
| hCoV-19/Algeria/31004/2021 | EPI_ISL_15928110 | 21/04/2021 | Africa / Algeria / Algiers | Human | National SARS-CoV-2 genomic and variants surveillance program | Female | 48 | Live | unknown | National SARS-CoV-2 genomic and variants surveillance program | B.1.1.7 | GR |
| hCoV-19/Algeria/31794/2021 | EPI_ISL_15928111 | 25/04/2021 | Africa / Algeria / Algiers | Human | National SARS-CoV-2 genomic and variants surveillance program | Female | 60 | Live | unknown | National SARS-CoV-2 genomic and variants surveillance program | B.1.1.7 | GR |
| hCoV-19/Algeria/49895/2021 | EPI_ISL_15928112 | 24/06/2021 | Africa / Algeria / Algiers | Human | National SARS-CoV-2 genomic and variants surveillance program | Female | 43 | Live | unknown | National SARS-CoV-2 genomic and variants surveillance program | B.1.617.2 | GK |
| hCoV-19/Algeria/20119/2021 | EPI_ISL_15928113 | 24/06/2021 | Africa / Algeria / Algiers | Human | National SARS-CoV-2 genomic and variants surveillance program | Female | 38 | Live | unknown | National SARS-CoV-2 genomic and variants surveillance program | B.1.1.7 | GR |
| hCoV-19/Algeria/50699/2021 | EPI_ISL_15928114 | 27/06/2021 | Africa / Algeria / Algiers | Human | National SARS-CoV-2 genomic and variants surveillance program | Female | 60 | Live | unknown | National SARS-CoV-2 genomic and variants surveillance program | B.1.1.7 | GRY |
| hCoV-19/Algeria/52494/2021 | EPI_ISL_15928115 | 01/07/2021 | Africa / Algeria / Algiers | Human | National SARS-CoV-2 genomic and variants surveillance program | Male | 68 | Live | unknown | National SARS-CoV-2 genomic and variants surveillance program | B.1.1.7 | GRY |
| hCoV-19/Algeria/55073/2021 | EPI_ISL_15928116 | 08/07/2021 | Africa / Algeria / Algiers | Human | National SARS-CoV-2 genomic and variants surveillance program | Female | 70 | Live | unknown | National SARS-CoV-2 genomic and variants surveillance program | B.1.617.2 | GK |
| hCoV-19/Algeria/55474/2021 | EPI_ISL_15928117 | 08/07/2021 | Africa / Algeria / Algiers | Human | National SARS-CoV-2 genomic and variants surveillance program | Male | 43 | Live | unknown | National SARS-CoV-2 genomic and variants surveillance program | B.1.617.2 | GK |
| hCoV-19/Algeria/55544/2021 | EPI_ISL_15928118 | 10/07/2021 | Africa / Algeria / Algiers | Human | National SARS-CoV-2 genomic and variants surveillance program | Male | 67 | Live | unknown | National SARS-CoV-2 genomic and variants surveillance program | B.1.617.2 | GK |
| hCoV-19/Algeria/57718/2021 | EPI_ISL_15928119 | 13/07/2021 | Africa / Algeria / Bouira | Human | National SARS-CoV-2 genomic and variants surveillance program | Female | 87 | Live | unknown | National SARS-CoV-2 genomic and variants surveillance program | B.1.617.2 | GK |
| hCoV-19/Algeria/68015/2021 | EPI_ISL_15928120 | 05/08/2021 | Africa / Algeria / Bouira | Human | National SARS-CoV-2 genomic and variants surveillance program | Female | 93 | Live | unknown | National SARS-CoV-2 genomic and variants surveillance program | B.1.617.2 | GK |
| hCoV-19/Algeria/68099/2021 | EPI_ISL_15928121 | 05/08/2021 | Africa / Algeria / Bouira | Human | National SARS-CoV-2 genomic and variants surveillance program | Male | 89 | Live | unknown | National SARS-CoV-2 genomic and variants surveillance program | B.1.617.2 | GK |
| hCoV-19/Algeria/68952/2021 | EPI_ISL_15928122 | 08/08/2021 | Africa / Algeria / Algiers | Human | National SARS-CoV-2 genomic and variants surveillance program | Male | 93 | Live | unknown | National SARS-CoV-2 genomic and variants surveillance program | B.1.617.2 | GK |
| hCoV-19/Algeria/70103/2021 | EPI_ISL_15928123 | 11/08/2021 | Africa / Algeria / Algiers | Human | National SARS-CoV-2 genomic and variants surveillance program | Female | 47 | Live | unknown | National SARS-CoV-2 genomic and variants surveillance program | B.1.617.2 | GK |
| hCoV-19/Algeria/74749/2021 | EPI_ISL_15928124 | 26/08/2021 | Africa / Algeria / Algiers | Human | National SARS-CoV-2 genomic and variants surveillance program | Male | 1 | Live | unknown | National SARS-CoV-2 genomic and variants surveillance program | B.1.617.2 | GK |
| hCoV-19/Algeria/78902/2021 | EPI_ISL_15928125 | 13/09/2021 | Africa / Algeria / Blida | Human | National SARS-CoV-2 genomic and variants surveillance program | Male | 91 | Live | unknown | National SARS-CoV-2 genomic and variants surveillance program | B.1.617.2 | GK |
| hCoV-19/Algeria/79342/2021 | EPI_ISL_15928126 | 15/09/2021 | Africa / Algeria / Algiers | Human | National SARS-CoV-2 genomic and variants surveillance program | Female | 29 | Live | unknown | National SARS-CoV-2 genomic and variants surveillance program | B.1 | G |
| hCoV-19/Algeria/82249/2021 | EPI_ISL_15928127 | 03/10/2021 | Africa / Algeria / Bouira | Human | National SARS-CoV-2 genomic and variants surveillance program | Male | 87 | Live | unknown | National SARS-CoV-2 genomic and variants surveillance program | B.1 | GK |
| hCoV-19/Algeria/82266/2021 | EPI_ISL_15928128 | 03/10/2021 | Africa / Algeria / Bouira | Human | National SARS-CoV-2 genomic and variants surveillance program | Male | 85 | Live | unknown | National SARS-CoV-2 genomic and variants surveillance program | B.1 | GK |
| hCoV-19/Algeria/82854/2021 | EPI_ISL_15928129 | 06/10/2021 | Africa / Algeria / Algiers | Human | National SARS-CoV-2 genomic and variants surveillance program | Female | 57 | Live | unknown | National SARS-CoV-2 genomic and variants surveillance program | B.1.617.2 | GK |
| hCoV-19/Algeria/83463/2021 | EPI_ISL_15928130 | 10/10/2021 | Africa / Algeria / Algiers | Human | National SARS-CoV-2 genomic and variants surveillance program | Female | 64 | Live | unknown | National SARS-CoV-2 genomic and variants surveillance program | B.1 | G |
| hCoV-19/Algeria/83486/2021 | EPI_ISL_15928131 | 10/10/2021 | Africa / Algeria / Algiers | Human | National SARS-CoV-2 genomic and variants surveillance program | Male | 5mois | Live | unknown | National SARS-CoV-2 genomic and variants surveillance program | B.1.1.529 | G |
| hCoV-19/Algeria/87391/2021 | EPI_ISL_15928132 | 31/10/2021 | Africa / Algeria / Blida | Human | National SARS-CoV-2 genomic and variants surveillance program | Female | 48 | Live | unknown | National SARS-CoV-2 genomic and variants surveillance program | B.1 | GK |
| hCoV-19/Algeria/87396/2021 | EPI_ISL_15928133 | 31/10/2021 | Africa / Algeria / Blida | Human | National SARS-CoV-2 genomic and variants surveillance program | Male | 27 | Live | unknown | National SARS-CoV-2 genomic and variants surveillance program | B.1.617.2 | GK |
| hCoV-19/Algeria/87743/2021 | EPI_ISL_15928134 | 02/11/2021 | Africa / Algeria / Algiers | Human | National SARS-CoV-2 genomic and variants surveillance program | Male | 38 | Live | unknown | National SARS-CoV-2 genomic and variants surveillance program | B.1.617.2 | GK |
| hCoV-19/Algeria/87970/2021 | EPI_ISL_15928135 | 03/11/2021 | Africa / Algeria / Algiers | Human | National SARS-CoV-2 genomic and variants surveillance program | Female | 74 | Live | unknown | National SARS-CoV-2 genomic and variants surveillance program | B.1 | G |
| hCoV-19/Algeria/88041/2021 | EPI_ISL_15928136 | 03/11/2021 | Africa / Algeria / Medea | Human | National SARS-CoV-2 genomic and variants surveillance program | Male | 68 | Live | unknown | National SARS-CoV-2 genomic and variants surveillance program | B.1 | GK |
| hCoV-19/Algeria/88043/2021 | EPI_ISL_15928137 | 03/11/2021 | Africa / Algeria / Medea | Human | National SARS-CoV-2 genomic and variants surveillance program | Male | 80 | Live | unknown | National SARS-CoV-2 genomic and variants surveillance program | B.1.617.2 | GK |
| hCoV-19/Algeria/88187/2021 | EPI_ISL_15928138 | 04/11/2021 | Africa / Algeria / Algiers | Human | National SARS-CoV-2 genomic and variants surveillance program | Male | 33 | Live | unknown | National SARS-CoV-2 genomic and variants surveillance program | B.1.617.2 | GK |
| hCoV-19/Algeria/89226/2021 | EPI_ISL_15928139 | 10/11/2021 | Africa / Algeria / Bouira | Human | National SARS-CoV-2 genomic and variants surveillance program | Male | 51 | Live | unknown | National SARS-CoV-2 genomic and variants surveillance program | B.1.617.2 | GK |
| hCoV-19/Algeria/89238/2021 | EPI_ISL_15928140 | 10/11/2021 | Africa / Algeria / Bouira | Human | National SARS-CoV-2 genomic and variants surveillance program | Male | 64 | Live | unknown | National SARS-CoV-2 genomic and variants surveillance program | B.1.617.2 | GK |
| hCoV-19/Algeria/90207/2021 | EPI_ISL_15928141 | 16/11/2021 | Africa / Algeria / Algiers | Human | National SARS-CoV-2 genomic and variants surveillance program | Female | 42 | Live | unknown | National SARS-CoV-2 genomic and variants surveillance program | B.1 | G |
| hCoV-19/Algeria/92470/2021 | EPI_ISL_15928142 | 28/11/2021 | Africa / Algeria / Algiers | Human | National SARS-CoV-2 genomic and variants surveillance program | Female | 56 | Live | unknown | National SARS-CoV-2 genomic and variants surveillance program | B.1.617.2 | GK |
| hCoV-19/Algeria/92479/2021 | EPI_ISL_15928143 | 28/11/2021 | Africa / Algeria / Algiers | Human | National SARS-CoV-2 genomic and variants surveillance program | Male | 63 | Live | unknown | National SARS-CoV-2 genomic and variants surveillance program | B.1.617.2 | GK |
| hCoV-19/Algeria/95664/2021 | EPI_ISL_15928144 | 12/12/2021 | Africa / Algeria / Algiers | Human | National SARS-CoV-2 genomic and variants surveillance program | Female | 30 | Live | unknown | National SARS-CoV-2 genomic and variants surveillance program | B.1 | G |
| hCoV-19/Algeria/95675/2021 | EPI_ISL_15928145 | 12/12/2021 | Africa / Algeria / Algiers | Human | National SARS-CoV-2 genomic and variants surveillance program | Female | 40 | Live | unknown | National SARS-CoV-2 genomic and variants surveillance program | B.1 | GK |
| hCoV-19/Algeria/95691/2021 | EPI_ISL_15928146 | 12/12/2021 | Africa / Algeria / Algiers | Human | National SARS-CoV-2 genomic and variants surveillance program | Male | 48 | Live | unknown | National SARS-CoV-2 genomic and variants surveillance program | B.1.617.2 | GK |
| hCoV-19/Algeria/95698/2021 | EPI_ISL_15928147 | 12/12/2021 | Africa / Algeria / Algiers | Human | National SARS-CoV-2 genomic and variants surveillance program | Female | 61 | Live | unknown | National SARS-CoV-2 genomic and variants surveillance program | B.1 | GK |
| hCoV-19/Algeria/95707/2021 | EPI_ISL_15928148 | 12/12/2021 | Africa / Algeria / Algiers | Human | National SARS-CoV-2 genomic and variants surveillance program | Female | 22 | Live | unknown | National SARS-CoV-2 genomic and variants surveillance program | BA.4 | GR |
| hCoV-19/Algeria/95708/2021 | EPI_ISL_15928149 | 12/12/2021 | Africa / Algeria / Algiers | Human | National SARS-CoV-2 genomic and variants surveillance program | Male | 52 | Live | unknown | National SARS-CoV-2 genomic and variants surveillance program | B.1 | GK |
| hCoV-19/Algeria/98337/2021 | EPI_ISL_15928150 | 23/12/2021 | Africa / Algeria / Algiers | Human | National SARS-CoV-2 genomic and variants surveillance program | Male | 67 | Live | unknown | National SARS-CoV-2 genomic and variants surveillance program | B.1.617.2 | GK |
| hCoV-19/Algeria/98368/2021 | EPI_ISL_15928151 | 23/12/2021 | Africa / Algeria / Algiers | Human | National SARS-CoV-2 genomic and variants surveillance program | Female | 81 | Live | unknown | National SARS-CoV-2 genomic and variants surveillance program | B.1.617.2 | GK |
| hCoV-19/Algeria/98548/2021 | EPI_ISL_15928152 | 23/12/2021 | Africa / Algeria / Algiers | Human | National SARS-CoV-2 genomic and variants surveillance program | Female | 59 | Live | unknown | National SARS-CoV-2 genomic and variants surveillance program | B.1.617.2 | GK |
| hCoV-19/Algeria/98809/2021 | EPI_ISL_15928153 | 26/12/2021 | Africa / Algeria / Algiers | Human | National SARS-CoV-2 genomic and variants surveillance program | Male | 36 | Live | unknown | National SARS-CoV-2 genomic and variants surveillance program | B.1.617.2 | GK |
| hCoV-19/Algeria/42539/2022 | EPI_ISL_15946128 | 13/11/2022 | Africa / Algeria / Algiers | Human | National SARS-CoV-2 genomic and variants surveillance program | Female | 45 | Live | unknown | National SARS-CoV-2 genomic and variants surveillance program | XBB.1 | GRA |
| hCoV-19/Algeria/631/2022 | EPI_ISL_15946129 | 12/07/2022 | Africa / Algeria / Annaba | Human | National SARS-CoV-2 genomic and variants surveillance program | Male | 33 | Live | unknown | National SARS-CoV-2 genomic and variants surveillance program | BA.5.2.1 | GRA |
| hCoV-19/Algeria/6073/2022 | EPI_ISL_15946130 | 13/07/2022 | Africa / Algeria / Biskra | Human | National SARS-CoV-2 genomic and variants surveillance program | Male | 46 | Live | unknown | National SARS-CoV-2 genomic and variants surveillance program | BA.5.2 | GRA |
| hCoV-19/Algeria/6080/2022 | EPI_ISL_15946131 | 14/07/2022 | Africa / Algeria / Biskra | Human | National SARS-CoV-2 genomic and variants surveillance program | Female | 72 | Live | unknown | National SARS-CoV-2 genomic and variants surveillance program | BA.4 | GRA |
| hCoV-19/Algeria/6090/2022 | EPI_ISL_15946132 | 16/07/2022 | Africa / Algeria / Biskra | Human | National SARS-CoV-2 genomic and variants surveillance program | Male | 82 | Live | unknown | National SARS-CoV-2 genomic and variants surveillance program | BA.5.2 | GRA |
| hCoV-19/Algeria/6094/2022 | EPI_ISL_15946133 | 17/07/2022 | Africa / Algeria / Biskra | Human | National SARS-CoV-2 genomic and variants surveillance program | Female | 24 | Live | unknown | National SARS-CoV-2 genomic and variants surveillance program | BA.5.2 | GRA |
| hCoV-19/Algeria/3W/2022 | EPI_ISL_15946134 | 13/07/2022 | Africa / Algeria / Biskra | Human | National SARS-CoV-2 genomic and variants surveillance program | Male | 40 | Live | unknown | National SARS-CoV-2 genomic and variants surveillance program | BA.5.2.20 | GRA |
| hCoV-19/Algeria/4W/2022 | EPI_ISL_15946135 | 13/07/2022 | Africa / Algeria / Biskra | Human | National SARS-CoV-2 genomic and variants surveillance program | Male | 42 | Live | unknown | National SARS-CoV-2 genomic and variants surveillance program | BA.5.2.20 | GRA |
| hCoV-19/Algeria/5W/2022 | EPI_ISL_15946136 | 13/07/2022 | Africa / Algeria / Biskra | Human | National SARS-CoV-2 genomic and variants surveillance program | Male | 55 | Live | unknown | National SARS-CoV-2 genomic and variants surveillance program | BA.5.2.20 | GRA |
| hCoV-19/Algeria/6W/2022 | EPI_ISL_15946137 | 13/07/2022 | Africa / Algeria / Biskra | Human | National SARS-CoV-2 genomic and variants surveillance program | Male | 57 | Live | unknown | National SARS-CoV-2 genomic and variants surveillance program | BA.5.2.20 | GRA |
| hCoV-19/Algeria/7W/2022 | EPI_ISL_15946138 | 13/07/2022 | Africa / Algeria / Biskra | Human | National SARS-CoV-2 genomic and variants surveillance program | Male | 54 | Live | unknown | National SARS-CoV-2 genomic and variants surveillance program | BA.5.2.20 | GRA |
| hCoV-19/Algeria/9W/2022 | EPI_ISL_15946139 | 13/07/2022 | Africa / Algeria / Biskra | Human | National SARS-CoV-2 genomic and variants surveillance program | Male | 57 | Live | unknown | National SARS-CoV-2 genomic and variants surveillance program | BA.5.2.20 | GRA |
| hCoV-19/Algeria/10W/2022 | EPI_ISL_15946140 | 13/07/2022 | Africa / Algeria / Biskra | Human | National SARS-CoV-2 genomic and variants surveillance program | Male | 54 | Live | unknown | National SARS-CoV-2 genomic and variants surveillance program | BA.5.2.20 | GRA |
| hCoV-19/Algeria/12W/2022 | EPI_ISL_15946141 | 13/07/2022 | Africa / Algeria / Biskra | Human | National SARS-CoV-2 genomic and variants surveillance program | Male | 49 | Live | unknown | National SARS-CoV-2 genomic and variants surveillance program | BA.5.2.20 | GRA |
| hCoV-19/Algeria/13W/2022 | EPI_ISL_15946142 | 13/07/2022 | Africa / Algeria / Biskra | Human | National SARS-CoV-2 genomic and variants surveillance program | Male | 27 | Live | unknown | National SARS-CoV-2 genomic and variants surveillance program | BA.5.2 | GRA |
| hCoV-19/Algeria/17W/2022 | EPI_ISL_15946143 | 19/07/2022 | Africa / Algeria / Biskra | Human | National SARS-CoV-2 genomic and variants surveillance program | Female | 76 | Live | unknown | National SARS-CoV-2 genomic and variants surveillance program | BA.5.2 | GRA |
| hCoV-19/Algeria/6176/2022 | EPI_ISL_15946144 | 31/07/2022 | Africa / Algeria / Biskra | Human | National SARS-CoV-2 genomic and variants surveillance program | Female | 48 | Live | unknown | National SARS-CoV-2 genomic and variants surveillance program | BA.5.2.44 | GRA |
| hCoV-19/Algeria/6180/2022 | EPI_ISL_15946145 | 01/08/2022 | Africa / Algeria / Biskra | Human | National SARS-CoV-2 genomic and variants surveillance program | Female | 46 | Live | unknown | National SARS-CoV-2 genomic and variants surveillance program | BA.5.2 | GRA |
| hCoV-19/Algeria/25R/2022 | EPI_ISL_15946146 | 28/07/2022 | Africa / Algeria / Biskra | Human | National SARS-CoV-2 genomic and variants surveillance program | Male | 80 | Live | unknown | National SARS-CoV-2 genomic and variants surveillance program | BA.5.2 | GRA |
| hCoV-19/Algeria/31W/2022 | EPI_ISL_15946147 | 26/07/2022 | Africa / Algeria / Biskra | Human | National SARS-CoV-2 genomic and variants surveillance program | Male | 55 | Live | unknown | National SARS-CoV-2 genomic and variants surveillance program | BA.5.2.44 | GRA |
| hCoV-19/Algeria/33W/2022 | EPI_ISL_15946148 | 26/07/2022 | Africa / Algeria / Biskra | Human | National SARS-CoV-2 genomic and variants surveillance program | Female | 68 | Live | unknown | National SARS-CoV-2 genomic and variants surveillance program | BA.5.2 | GRA |
| hCoV-19/Algeria/640/2022 | EPI_ISL_15946149 | 18/07/2022 | Africa / Algeria / El Taref | Human | National SARS-CoV-2 genomic and variants surveillance program | Female | 28 | Live | unknown | National SARS-CoV-2 genomic and variants surveillance program | BA.5.1.22 | GRA |
| hCoV-19/Algeria/641/2022 | EPI_ISL_15946150 | 18/07/2022 | Africa / Algeria / El Taref | Human | National SARS-CoV-2 genomic and variants surveillance program | Female | 31 | Live | unknown | National SARS-CoV-2 genomic and variants surveillance program | BA.5.5 | GRA |
| hCoV-19/Algeria/646/2022 | EPI_ISL_15946151 | 19/07/2022 | Africa / Algeria / El Taref | Human | National SARS-CoV-2 genomic and variants surveillance program | Female | 38 | Live | unknown | National SARS-CoV-2 genomic and variants surveillance program | BA.5.2 | GRA |
| hCoV-19/Algeria/651/2022 | EPI_ISL_15946152 | 19/07/2022 | Africa / Algeria / El Taref | Human | National SARS-CoV-2 genomic and variants surveillance program | Female | 39 | Live | unknown | National SARS-CoV-2 genomic and variants surveillance program | BA.5.2.20 | GRA |
| hCoV-19/Algeria/678/2022 | EPI_ISL_15946153 | 28/07/2022 | Africa / Algeria / El Taref | Human | National SARS-CoV-2 genomic and variants surveillance program | Female | 28 | Live | unknown | National SARS-CoV-2 genomic and variants surveillance program | BA.5.2 | GRA |
| hCoV-19/Algeria/697/2022 | EPI_ISL_15946154 | 08/08/2022 | Africa / Algeria / El Taref | Human | National SARS-CoV-2 genomic and variants surveillance program | Female | 45 | Live | unknown | National SARS-CoV-2 genomic and variants surveillance program | BA.5.2.20 | GRA |
| hCoV-19/Algeria/42567/2022 | EPI_ISL_15946155 | 14/11/2022 | Africa / Algeria / Medea | Human | National SARS-CoV-2 genomic and variants surveillance program | Female | 39 | Live | unknown | National SARS-CoV-2 genomic and variants surveillance program | BF.7 | GRA |
| hCoV-19/Algeria/42596/2022 | EPI_ISL_15946156 | 15/11/2022 | Africa / Algeria / Medea | Human | National SARS-CoV-2 genomic and variants surveillance program | Male | 78 | Live | unknown | National SARS-CoV-2 genomic and variants surveillance program | BQ.1 | GRA |
| hCoV-19/Algeria/743/2022 | EPI_ISL_15946157 | 13/07/2022 | Africa / Algeria / Msila | Human | National SARS-CoV-2 genomic and variants surveillance program | Male | 39 | Live | unknown | National SARS-CoV-2 genomic and variants surveillance program | BA.5.2 | GRA |
| hCoV-19/Algeria/744/2022 | EPI_ISL_15946158 | 13/07/2022 | Africa / Algeria / Msila | Human | National SARS-CoV-2 genomic and variants surveillance program | Male | 32 | Live | unknown | National SARS-CoV-2 genomic and variants surveillance program | BA.5.2 | GRA |
| hCoV-19/Algeria/747/2022 | EPI_ISL_15946159 | 14/07/2022 | Africa / Algeria / Msila | Human | National SARS-CoV-2 genomic and variants surveillance program | Male | 38 | Live | unknown | National SARS-CoV-2 genomic and variants surveillance program | BA.5.2.27 | GRA |
| hCoV-19/Algeria/748/2022 | EPI_ISL_15946160 | 14/07/2022 | Africa / Algeria / Msila | Human | National SARS-CoV-2 genomic and variants surveillance program | Female | 22 | Live | unknown | National SARS-CoV-2 genomic and variants surveillance program | BA.4 | GRA |
| hCoV-19/Algeria/761/2022 | EPI_ISL_15946161 | 18/07/2022 | Africa / Algeria / Msila | Human | National SARS-CoV-2 genomic and variants surveillance program | Male | 50 | Live | unknown | National SARS-CoV-2 genomic and variants surveillance program | BA.5.2 | GRA |
| hCoV-19/Algeria/762/2022 | EPI_ISL_15946162 | 18/07/2022 | Africa / Algeria / Msila | Human | National SARS-CoV-2 genomic and variants surveillance program | Male | 60 | Live | unknown | National SARS-CoV-2 genomic and variants surveillance program | BA.5.2 | GRA |
| hCoV-19/Algeria/767/2022 | EPI_ISL_15946163 | 19/07/2022 | Africa / Algeria / Msila | Human | National SARS-CoV-2 genomic and variants surveillance program | Male | 37 | Live | unknown | National SARS-CoV-2 genomic and variants surveillance program | BA.5.1 | GRA |
| hCoV-19/Algeria/768/2022 | EPI_ISL_15946164 | 19/07/2022 | Africa / Algeria / Msila | Human | National SARS-CoV-2 genomic and variants surveillance program | Male | 33 | Live | unknown | National SARS-CoV-2 genomic and variants surveillance program | BA.5.2 | GRA |
| hCoV-19/Algeria/2821/2022 | EPI_ISL_15946165 | 31/07/2022 | Africa / Algeria / Tlemcen | Human | National SARS-CoV-2 genomic and variants surveillance program | Female | 27 | Live | unknown | National SARS-CoV-2 genomic and variants surveillance program | BE.1 | GRA |
| hCoV-19/Algeria/2819/2022 | EPI_ISL_15946166 | 30/07/2022 | Africa / Algeria / Tlemcen | Human | National SARS-CoV-2 genomic and variants surveillance program | Female | 56 | Live | unknown | National SARS-CoV-2 genomic and variants surveillance program | BF.5 | GRA |
| hCoV-19/Algeria/2822/2022 | EPI_ISL_15946167 | 01/08/2022 | Africa / Algeria / Tlemcen | Human | National SARS-CoV-2 genomic and variants surveillance program | Female | 55 | Live | unknown | National SARS-CoV-2 genomic and variants surveillance program | BF.5 | GRA |
| hCoV-19/Algeria/42514/2022 | EPI_ISL_15961761 | 13/11/2022 | Africa / Algeria / Algiers | Human | National SARS-CoV-2 genomic and variants surveillance program | Female | 27 | Live | unknown | National SARS-CoV-2 genomic and variants surveillance program | BQ.1.1 | GRA |
| hCoV-19/Algeria/807/2022 | EPI_ISL_16076505 | 26/07/2022 | Africa / Algeria / Mâ€™Sila | Human | National SARS-CoV-2 genomic and variants surveillance program | Female | 73 | Live | unknown | National SARS-CoV-2 genomic and variants surveillance program | BA.5.2 | GRA |
| hCoV-19/Algeria/2802/2022 | EPI_ISL_16076506 | 26/07/2022 | Africa / Algeria / Tlemcen | Human | National SARS-CoV-2 genomic and variants surveillance program | Female | 62 | Live | unknown | National SARS-CoV-2 genomic and variants surveillance program | BA.5.2.1 | GRA |
| hCoV-19/Algeria/43367/2022 | EPI_ISL_16076507 | 23/11/2022 | Africa / Algeria / Algiers | Human | National SARS-CoV-2 genomic and variants surveillance program | Male | 45 | Live | unknown | National SARS-CoV-2 genomic and variants surveillance program | BQ.1.5 | GRA |
| hCoV-19/Algeria/4750/2022 | EPI_ISL_16076508 | 02/08/2022 | Africa / Algeria / Tizi-Ouzou | Human | National SARS-CoV-2 genomic and variants surveillance program | Female | 69 | Live | unknown | National SARS-CoV-2 genomic and variants surveillance program | BE.1 | GRA |
| hCoV-19/Algeria/4449/2022 | EPI_ISL_16076509 | 17/07/2022 | Africa / Algeria / Tizi-Ouzou | Human | National SARS-CoV-2 genomic and variants surveillance program | Female | 50 | Live | unknown | National SARS-CoV-2 genomic and variants surveillance program | BA.5.1.22 | GRA |
| hCoV-19/Algeria/4526/2022 | EPI_ISL_16076510 | 22/07/2022 | Africa / Algeria / Tizi-Ouzou | Human | National SARS-CoV-2 genomic and variants surveillance program | Female | 56 | Live | unknown | National SARS-CoV-2 genomic and variants surveillance program | BA.5.6 | GRA |
| hCoV-19/Algeria/4673/2022 | EPI_ISL_16076511 | 28/07/2022 | Africa / Algeria / Tizi-Ouzou | Human | National SARS-CoV-2 genomic and variants surveillance program | Female | 23 | Live | unknown | National SARS-CoV-2 genomic and variants surveillance program | BA.5.2.1 | GRA |
| hCoV-19/Algeria/4676/2022 | EPI_ISL_16076512 | 29/07/2022 | Africa / Algeria / Tizi-Ouzou | Human | National SARS-CoV-2 genomic and variants surveillance program | Female | 65 | Live | unknown | National SARS-CoV-2 genomic and variants surveillance program | BA.5.2 | GRA |
| hCoV-19/Algeria/4680/2022 | EPI_ISL_16076513 | 29/07/2022 | Africa / Algeria / Tizi-Ouzou | Human | National SARS-CoV-2 genomic and variants surveillance program | Male | 40 | Live | unknown | National SARS-CoV-2 genomic and variants surveillance program | BE.1 | GRA |
| hCoV-19/Algeria/4837/2022 | EPI_ISL_16076514 | 09/08/2022 | Africa / Algeria / Tizi-Ouzou | Human | National SARS-CoV-2 genomic and variants surveillance program | Male | 27 | Live | unknown | National SARS-CoV-2 genomic and variants surveillance program | BA.5.1 | GRA |
| hCoV-19/Algeria/4871/2022 | EPI_ISL_16076515 | 10/08/2022 | Africa / Algeria / Tizi-Ouzou | Human | National SARS-CoV-2 genomic and variants surveillance program | Male | 87 | Live | unknown | National SARS-CoV-2 genomic and variants surveillance program | BA.5.2.1 | GRA |
| hCoV-19/Algeria/4874/2022 | EPI_ISL_16076516 | 10/08/2022 | Africa / Algeria / Tizi-Ouzou | Human | National SARS-CoV-2 genomic and variants surveillance program | Female | 84 | Live | unknown | National SARS-CoV-2 genomic and variants surveillance program | BA.5.2.1 | GRA |
| hCoV-19/Algeria/4876/2022 | EPI_ISL_16076517 | 10/08/2022 | Africa / Algeria / Tizi-Ouzou | Human | National SARS-CoV-2 genomic and variants surveillance program | Female | 71 | Live | unknown | National SARS-CoV-2 genomic and variants surveillance program | BA.4 | GRA |
| hCoV-19/Algeria/8718/2022 | EPI_ISL_16076518 | 11/08/2022 | Africa / Algeria / Tizi-Ouzou | Human | National SARS-CoV-2 genomic and variants surveillance program | Male | 52 | Live | unknown | National SARS-CoV-2 genomic and variants surveillance program | BA.5.2 | GRA |
| hCoV-19/Algeria/8719/2022 | EPI_ISL_16076519 | 01/08/2022 | Africa / Algeria / Tizi-Ouzou | Human | National SARS-CoV-2 genomic and variants surveillance program | Female | 40 | Live | unknown | National SARS-CoV-2 genomic and variants surveillance program | BA.5.2 | GRA |
| hCoV-19/Algeria/8720/2022 | EPI_ISL_16076520 | 01/08/2022 | Africa / Algeria / Tizi-Ouzou | Human | National SARS-CoV-2 genomic and variants surveillance program | Female | 73 | Live | unknown | National SARS-CoV-2 genomic and variants surveillance program | BA.5.2 | GRA |
| hCoV-19/Algeria/5114/2022 | EPI_ISL_16076521 | 30/08/2022 | Africa / Algeria / Tizi-Ouzou | Human | National SARS-CoV-2 genomic and variants surveillance program | Male | 40 | Live | unknown | National SARS-CoV-2 genomic and variants surveillance program | BA.5.2 | GRA |
| hCoV-19/Algeria/43120/2022 | EPI_ISL_16076522 | 20/11/2022 | Africa / Algeria / Algiers | Human | National SARS-CoV-2 genomic and variants surveillance program | Female | unknown | Live | unknown | National SARS-CoV-2 genomic and variants surveillance program | BQ.1.1 | GRA |
| hCoV-19/Algeria/43068/2022 | EPI_ISL_16076523 | 19/11/2022 | Africa / Algeria / Algiers | Human | National SARS-CoV-2 genomic and variants surveillance program | Male | unknown | Live | unknown | National SARS-CoV-2 genomic and variants surveillance program | BQ.1.1 | GRA |
| hCoV-19/Algeria/43409/2022 | EPI_ISL_16076524 | 23/11/2022 | Africa / Algeria / Algiers | Human | National SARS-CoV-2 genomic and variants surveillance program | Male | 7 | Live | unknown | National SARS-CoV-2 genomic and variants surveillance program | BQ.1.1 | GRA |
| hCoV-19/Algeria/38048/2022 | EPI_ISL_16242278 | 20/09/2022 | Africa / Algeria / Algiers | Human | unknown | Female | 51 | Live | unknown | unknown | BA.5.2 | GRA |
| hCoV-19/Algeria/43899/2022 | EPI_ISL_16242279 | 05/12/2022 | Africa / Algeria / Algiers | Human | unknown | Male | 53 | Live | unknown | unknown | XBB.1 | GRA |
| hCoV-19/Algeria/43883/2022 | EPI_ISL_16242280 | 04/12/2022 | Africa / Algeria / Algiers | Human | unknown | Female | 56 | Live | unknown | unknown | BQ.1.1 | GRA |
| hCoV-19/Algeria/43884/2022 | EPI_ISL_16242281 | 03/12/2022 | Africa / Algeria / Algiers | Human | unknown | Female | 55 | Live | unknown | unknown | BQ.1.1 | GRA |
| hCoV-19/Algeria/44067/2022 | EPI_ISL_16242282 | 07/12/2022 | Africa / Algeria / Algiers | Human | unknown | Female | 42 | Live | unknown | unknown | BQ.1 | GRA |
| hCoV-19/Algeria/HCA976/2022 | EPI_ISL_16242283 | 08/12/2022 | Africa / Algeria / Algiers | Human | unknown | Male | unknown | Live | unknown | unknown | XBB.8 | GRA |
| hCoV-19/Algeria/44064/2022 | EPI_ISL_16242284 | 07/12/2022 | Africa / Algeria / Algiers | Human | unknown | Female | 34 | Live | unknown | unknown | XBB.1.9.3 | GRA |
| hCoV-19/Algeria/38874/2022 | EPI_ISL_16242285 | 29/09/2022 | Africa / Algeria / Blida | Human | unknown | Male | 56 | Live | unknown | unknown | BA.5.2.20 | GRA |
| hCoV-19/Algeria/37286/2022 | EPI_ISL_16242286 | 12/09/2022 | Africa / Algeria / Blida | Human | unknown | Female | 86 | Live | unknown | unknown | BA.5.1 | GRA |
| hCoV-19/Algeria/43906/2022 | EPI_ISL_16242287 | 06/12/2022 | Africa / Algeria / Blida | Human | unknown | Female | 33 | Live | unknown | unknown | BQ.1.1.59 | GRA |
| hCoV-19/Algeria/43323/2022 | EPI_ISL_16242288 | 22/11/2022 | Africa / Algeria / Medea | Human | unknown | Male | 41 | Live | unknown | unknown | BQ.1 | GRA |
| hCoV-19/Algeria/43891/2022 | EPI_ISL_16242289 | 05/12/2022 | Africa / Algeria / Medea | Human | unknown | Female | 50 | Live | unknown | unknown | BQ.1.1 | GRA |
| hCoV-19/Algeria/771/2022 | EPI_ISL_16242290 | 19/07/2022 | Africa / Algeria / Mâ€™sila | Human | unknown | Female | 51 | Live | unknown | unknown | BA.5.3.1 | GRA |
| hCoV-19/Algeria/777/2022 | EPI_ISL_16242291 | 20/07/2022 | Africa / Algeria / Mâ€™sila | Human | unknown | Female | 57 | Live | unknown | unknown | BA.5.2 | GRA |
| hCoV-19/Algeria/801/2022 | EPI_ISL_16242292 | 25/07/2022 | Africa / Algeria / Mâ€™sila | Human | unknown | Female | 12 | Live | unknown | unknown | BA.5.1.30 | GRA |
| hCoV-19/Algeria/805/2022 | EPI_ISL_16242293 | 26/07/2022 | Africa / Algeria / Mâ€™sila | Human | unknown | Female | 75 | Live | unknown | unknown | BA.5.2 | GRA |
| hCoV-19/Algeria/811/2022 | EPI_ISL_16242294 | 27/07/2022 | Africa / Algeria / Mâ€™sila | Human | unknown | Female | 38 | Live | unknown | unknown | BA.5.2 | GRA |
| hCoV-19/Algeria/813/2022 | EPI_ISL_16242295 | 27/07/2022 | Africa / Algeria / Mâ€™sila | Human | unknown | Female | 23 | Live | unknown | unknown | BA.5.2 | GRA |
| hCoV-19/Algeria/815/2022 | EPI_ISL_16242296 | 28/07/2022 | Africa / Algeria / Mâ€™sila | Human | unknown | Female | 73 | Live | unknown | unknown | BA.5.2 | GRA |
| hCoV-19/Algeria/1304/2022 | EPI_ISL_16242297 | 10/08/2022 | Africa / Algeria / Setif | Human | unknown | Female | 81 | Live | unknown | unknown | BA.5.2 | GRA |
| hCoV-19/Algeria/1306/2022 | EPI_ISL_16242298 | 09/08/2022 | Africa / Algeria / Setif | Human | unknown | Male | 85 | Live | unknown | unknown | BA.5.1 | GRA |
| hCoV-19/Algeria/1312/2022 | EPI_ISL_16242299 | 11/08/2022 | Africa / Algeria / Setif | Human | unknown | Female | 70 | Live | unknown | unknown | BA.5.2 | GRA |
| hCoV-19/Algeria/1325/2022 | EPI_ISL_16242300 | 17/08/2022 | Africa / Algeria / Setif | Human | unknown | Female | 45 | Live | unknown | unknown | BA.5.2 | GRA |
| hCoV-19/Algeria/1352/2022 | EPI_ISL_16242301 | 30/08/2022 | Africa / Algeria / Setif | Human | unknown | Male | 83 | Live | unknown | unknown | BA.5.2 | GRA |
| hCoV-19/Algeria/1366/2022 | EPI_ISL_16242302 | 07/09/2022 | Africa / Algeria / Setif | Human | unknown | Male | 63 | Live | unknown | unknown | BA.5.2.44 | GRA |
| hCoV-19/Algeria/1294/2022 | EPI_ISL_16242303 | 07/08/2022 | Africa / Algeria / Setif | Human | unknown | Male | 21 | Live | unknown | unknown | BA.5.2 | GRA |
| hCoV-19/Algeria/2738/2022 | EPI_ISL_16242304 | 18/07/2022 | Africa / Algeria / Sidi Bel Abbes | Human | unknown | Female | 18 | Live | unknown | unknown | BA.5.1 | GRA |
| hCoV-19/Algeria/2742/2022 | EPI_ISL_16242305 | 11/07/2022 | Africa / Algeria / Sidi Bel Abbes | Human | unknown | Female | 39 | Live | unknown | unknown | BA.5.2 | GRA |
| hCoV-19/Algeria/2712/2022 | EPI_ISL_16242306 | 25/07/2022 | Africa / Algeria / Sidi Bel Abbes | Human | unknown | Female | 24 | Live | unknown | unknown | BA.5.2 | GRA |
| hCoV-19/Algeria/2831/2022 | EPI_ISL_16242307 | 02/08/2022 | Africa / Algeria / Sidi Bel Abbes | Human | unknown | Male | 19 | Live | unknown | unknown | BA.5.2 | GRA |
| hCoV-19/Algeria/4711/2022 | EPI_ISL_16242308 | 31/07/2022 | Africa / Algeria / Tizi-Ouzou | Human | unknown | Female | 40 | Live | unknown | unknown | BA.5.2.3 | GRA |
| hCoV-19/Algeria/4835/2022 | EPI_ISL_16242309 | 09/08/2022 | Africa / Algeria / Tizi-Ouzou | Human | unknown | Male | 72 | Live | unknown | unknown | BA.5.2.1 | GRA |
| hCoV-19/Algeria/5042/2022 | EPI_ISL_16242310 | 24/08/2022 | Africa / Algeria / Tizi-Ouzou | Human | unknown | Female | 69 | Live | unknown | unknown | BA.5.2 | GRA |
| hCoV-19/Algeria/5113/2022 | EPI_ISL_16242311 | 30/08/2022 | Africa / Algeria / Tizi-Ouzou | Human | unknown | Female | 9 | Live | unknown | unknown | BA.5.2 | GRA |
| hCoV-19/Algeria/5117/2022 | EPI_ISL_16242312 | 30/08/2022 | Africa / Algeria / Tizi-Ouzou | Human | unknown | Male | 86 | Live | unknown | unknown | BA.5.2 | GRA |
| hCoV-19/Algeria/2842/2022 | EPI_ISL_16242313 | 09/08/2022 | Africa / Algeria / Tlemcen | Human | unknown | Female | 58 | Live | unknown | unknown | BA.5.2 | GRA |
| hCoV-19/Algeria/2850/2022 | EPI_ISL_16242314 | 10/08/2022 | Africa / Algeria / Tlemcen | Human | unknown | Female | 54 | Live | unknown | unknown | BA.5.1 | GRA |
| hCoV-19/Algeria/2852/2022 | EPI_ISL_16242315 | 10/08/2022 | Africa / Algeria / Tlemcen | Human | unknown | Female | 43 | Live | unknown | unknown | BE.1 | GRA |
| hCoV-19/Algeria/6000/2022 | EPI_ISL_16454584 | 03/07/2022 | Africa / Algeria / Biskra | Human | National SARS-CoV-2 genomic and variants surveillance program | Male | 44 | Live | unknown | National SARS-CoV-2 genomic and variants surveillance program | BA.5.2 | GRA |
| hCoV-19/Algeria/6006/2022 | EPI_ISL_16454585 | 04/07/2022 | Africa / Algeria / Biskra | Human | National SARS-CoV-2 genomic and variants surveillance program | Female | 46 | Live | unknown | National SARS-CoV-2 genomic and variants surveillance program | BA.5.2 | GRA |
| hCoV-19/Algeria/6020/2022 | EPI_ISL_16454586 | 07/07/2022 | Africa / Algeria / Biskra | Human | National SARS-CoV-2 genomic and variants surveillance program | Female | 28 | Live | unknown | National SARS-CoV-2 genomic and variants surveillance program | BA.4 | GRA |
| hCoV-19/Algeria/6026/2022 | EPI_ISL_16454587 | 10/07/2022 | Africa / Algeria / Biskra | Human | National SARS-CoV-2 genomic and variants surveillance program | Male | 49 | Live | unknown | National SARS-CoV-2 genomic and variants surveillance program | BA.5.2 | GRA |
| hCoV-19/Algeria/6029/2022 | EPI_ISL_16454588 | 11/07/2022 | Africa / Algeria / Biskra | Human | National SARS-CoV-2 genomic and variants surveillance program | Female | 32 | Live | unknown | National SARS-CoV-2 genomic and variants surveillance program | BA.5.2 | GRA |
| hCoV-19/Algeria/6039/2022 | EPI_ISL_16454589 | 11/07/2022 | Africa / Algeria / Biskra | Human | National SARS-CoV-2 genomic and variants surveillance program | Female | 69 | Live | unknown | National SARS-CoV-2 genomic and variants surveillance program | BA.5.2 | GRA |
| hCoV-19/Algeria/6049/2022 | EPI_ISL_16454590 | 12/07/2022 | Africa / Algeria / Biskra | Human | National SARS-CoV-2 genomic and variants surveillance program | Female | 36 | Live | unknown | National SARS-CoV-2 genomic and variants surveillance program | BA.4 | GRA |
| hCoV-19/Algeria/6050/2022 | EPI_ISL_16454591 | 12/07/2022 | Africa / Algeria / Biskra | Human | National SARS-CoV-2 genomic and variants surveillance program | Male | 69 | Live | unknown | National SARS-CoV-2 genomic and variants surveillance program | BA.5.2 | GRA |
| hCoV-19/Algeria/6197/2022 | EPI_ISL_16454592 | 09/08/2022 | Africa / Algeria / Biskra | Human | National SARS-CoV-2 genomic and variants surveillance program | Female | 51 | Live | unknown | National SARS-CoV-2 genomic and variants surveillance program | BA.5.2 | GRA |
| hCoV-19/Algeria/R26/2022 | EPI_ISL_16454593 | 30/07/2022 | Africa / Algeria / Biskra | Human | National SARS-CoV-2 genomic and variants surveillance program | Male | 52 | Live | unknown | National SARS-CoV-2 genomic and variants surveillance program | BA.5.2 | GRA |
| hCoV-19/Algeria/4387/2022 | EPI_ISL_16454594 | 16/07/2022 | Africa / Algeria / Tizi-Ouzou | Human | National SARS-CoV-2 genomic and variants surveillance program | Female | 29 | Live | unknown | National SARS-CoV-2 genomic and variants surveillance program | BA.5.2.20 | GRA |
| hCoV-19/Algeria/4391/2022 | EPI_ISL_16454595 | 16/07/2022 | Africa / Algeria / Tizi-Ouzou | Human | National SARS-CoV-2 genomic and variants surveillance program | Male | 39 | Live | unknown | National SARS-CoV-2 genomic and variants surveillance program | BA.5.2 | GRA |
| hCoV-19/Algeria/4464/2022 | EPI_ISL_16454596 | 20/07/2022 | Africa / Algeria / Tizi-Ouzou | Human | National SARS-CoV-2 genomic and variants surveillance program | Female | 29 | Live | unknown | National SARS-CoV-2 genomic and variants surveillance program | BA.5.1.30 | GRA |
| hCoV-19/Algeria/4475/2022 | EPI_ISL_16454597 | 20/07/2022 | Africa / Algeria / Tizi-Ouzou | Human | National SARS-CoV-2 genomic and variants surveillance program | Female | 45 | Live | unknown | National SARS-CoV-2 genomic and variants surveillance program | BE.1 | GRA |
| hCoV-19/Algeria/4478/2022 | EPI_ISL_16454598 | 20/07/2022 | Africa / Algeria / Tizi-Ouzou | Human | National SARS-CoV-2 genomic and variants surveillance program | Male | 88 | Live | unknown | National SARS-CoV-2 genomic and variants surveillance program | BA.5.1.22 | GRA |
| hCoV-19/Algeria/4490/2022 | EPI_ISL_16454599 | 20/07/2022 | Africa / Algeria / Tizi-Ouzou | Human | National SARS-CoV-2 genomic and variants surveillance program | Male | 5 | Live | unknown | National SARS-CoV-2 genomic and variants surveillance program | BA.5.2 | GRA |
| hCoV-19/Algeria/4508/2022 | EPI_ISL_16454600 | 22/07/2022 | Africa / Algeria / Tizi-Ouzou | Human | National SARS-CoV-2 genomic and variants surveillance program | Female | 90 | Live | unknown | National SARS-CoV-2 genomic and variants surveillance program | BA.5.1.22 | GRA |
| hCoV-19/Algeria/4515/2022 | EPI_ISL_16454601 | 22/07/2022 | Africa / Algeria / Tizi-Ouzou | Human | National SARS-CoV-2 genomic and variants surveillance program | Female | 33 | Live | unknown | National SARS-CoV-2 genomic and variants surveillance program | BA.5.2.1 | GRA |
| hCoV-19/Algeria/4530/2022 | EPI_ISL_16454602 | 24/07/2022 | Africa / Algeria / Tizi-Ouzou | Human | National SARS-CoV-2 genomic and variants surveillance program | Male | 71 | Live | unknown | National SARS-CoV-2 genomic and variants surveillance program | BA.5.2 | GRA |
| hCoV-19/Algeria/4535/2022 | EPI_ISL_16454603 | 24/07/2022 | Africa / Algeria / Tizi-Ouzou | Human | National SARS-CoV-2 genomic and variants surveillance program | Female | 38 | Live | unknown | National SARS-CoV-2 genomic and variants surveillance program | BA.5.2.1 | GRA |
| hCoV-19/Algeria/4559/2022 | EPI_ISL_16454604 | 25/07/2022 | Africa / Algeria / Tizi-Ouzou | Human | National SARS-CoV-2 genomic and variants surveillance program | Female | 27 | Live | unknown | National SARS-CoV-2 genomic and variants surveillance program | BA.5.2 | GRA |
| hCoV-19/Algeria/4576/2022 | EPI_ISL_16454605 | 26/07/2022 | Africa / Algeria / Tizi-Ouzou | Human | National SARS-CoV-2 genomic and variants surveillance program | Female | 35 | Live | unknown | National SARS-CoV-2 genomic and variants surveillance program | BA.5.1 | GRA |
| hCoV-19/Algeria/43859/2022 | EPI_ISL_17182679 | 04/12/2022 | Africa / Algeria / Algiers | Human | National SARS-CoV-2 genomic and variants surveillance program | Male | unknown | Live | unknown | National SARS-CoV-2 genomic and variants surveillance program | XBB.1 | GRA |
| hCoV-19/Algeria/43973/2022 | EPI_ISL_17182680 | 06/12/2022 | Africa / Algeria / Algiers | Human | National SARS-CoV-2 genomic and variants surveillance program | Female | unknown | Live | unknown | National SARS-CoV-2 genomic and variants surveillance program | BQ.1 | GRA |
| hCoV-19/Algeria/44060/2022 | EPI_ISL_17182681 | 07/12/2022 | Africa / Algeria / Algiers | Human | National SARS-CoV-2 genomic and variants surveillance program | Female | unknown | Live | unknown | National SARS-CoV-2 genomic and variants surveillance program | XBB.1 | GRA |
| hCoV-19/Algeria/44655/2022 | EPI_ISL_17182682 | 22/12/2022 | Africa / Algeria / Algiers | Human | National SARS-CoV-2 genomic and variants surveillance program | Male | unknown | Live | unknown | National SARS-CoV-2 genomic and variants surveillance program | BQ.1.1.59 | GRA |
| hCoV-19/Algeria/44581/2022 | EPI_ISL_17182683 | 21/12/2022 | Africa / Algeria / Algiers | Human | National SARS-CoV-2 genomic and variants surveillance program | Female | 40 | Live | unknown | National SARS-CoV-2 genomic and variants surveillance program | BQ.1.1.59 | GRA |
| hCoV-19/Algeria/44795/2022 | EPI_ISL_17182684 | 25/12/2022 | Africa / Algeria / Medea | Human | National SARS-CoV-2 genomic and variants surveillance program | Male | 94 | Live | unknown | National SARS-CoV-2 genomic and variants surveillance program | BQ.1.1.59 | GRA |
| hCoV-19/Algeria/44799/2022 | EPI_ISL_17182685 | 26/12/2022 | Africa / Algeria / Blida | Human | National SARS-CoV-2 genomic and variants surveillance program | Male | 84 | Live | unknown | National SARS-CoV-2 genomic and variants surveillance program | BQ.1.22 | GRA |
| hCoV-19/Algeria/44777/2022 | EPI_ISL_17182686 | 25/12/2022 | Africa / Algeria / Algiers | Human | National SARS-CoV-2 genomic and variants surveillance program | Female | 23 | Live | unknown | National SARS-CoV-2 genomic and variants surveillance program | BQ.1.1 | GRA |
| hCoV-19/Algeria/44776/2022 | EPI_ISL_17182687 | 24/12/2022 | Africa / Algeria / Algiers | Human | National SARS-CoV-2 genomic and variants surveillance program | Female | 28 | Live | unknown | National SARS-CoV-2 genomic and variants surveillance program | BQ.1 | GRA |
| hCoV-19/Algeria/03_23/2022 | EPI_ISL_17182688 | 30/12/2022 | Africa / Algeria / Algiers | Human | National SARS-CoV-2 genomic and variants surveillance program | Female | 34 | Live | unknown | National SARS-CoV-2 genomic and variants surveillance program | BQ.1 | GRA |
| hCoV-19/Algeria/05_23/2022 | EPI_ISL_17182689 | 29/12/2022 | Africa / Algeria / Algiers | Human | National SARS-CoV-2 genomic and variants surveillance program | Female | 32 | Live | unknown | National SARS-CoV-2 genomic and variants surveillance program | BN.3.1 | GRA |
| hCoV-19/Algeria/06_23/2022 | EPI_ISL_17182690 | 28/12/2022 | Africa / Algeria / Algiers | Human | National SARS-CoV-2 genomic and variants surveillance program | Female | 75 | Live | unknown | National SARS-CoV-2 genomic and variants surveillance program | BQ.1.1.18 | GRA |
| hCoV-19/Algeria/11_23/2022 | EPI_ISL_17182691 | 28/12/2022 | Africa / Algeria / Algiers | Human | National SARS-CoV-2 genomic and variants surveillance program | Female | 67 | Live | unknown | National SARS-CoV-2 genomic and variants surveillance program | XBB.1 | GRA |
| hCoV-19/Algeria/12_23/2022 | EPI_ISL_17182692 | 28/12/2022 | Africa / Algeria / Algiers | Human | National SARS-CoV-2 genomic and variants surveillance program | Male | 91 | Live | unknown | National SARS-CoV-2 genomic and variants surveillance program | XBB.1 | GRA |
| hCoV-19/Algeria/44949/2022 | EPI_ISL_17182693 | 28/12/2022 | Africa / Algeria / Algiers | Human | National SARS-CoV-2 genomic and variants surveillance program | Male | 38 | Live | unknown | National SARS-CoV-2 genomic and variants surveillance program | BQ.1.1.18 | GRA |
| hCoV-19/Algeria/161/2023 | EPI_ISL_17182694 | 03/01/2023 | Africa / Algeria / Algiers | Human | National SARS-CoV-2 genomic and variants surveillance program | Male | 45 | Live | unknown | National SARS-CoV-2 genomic and variants surveillance program | BA.4.6.3 | GRA |
| hCoV-19/Algeria/134/2023 | EPI_ISL_17182695 | 03/01/2023 | Africa / Algeria / Algiers | Human | National SARS-CoV-2 genomic and variants surveillance program | Female | 39 | Live | unknown | National SARS-CoV-2 genomic and variants surveillance program | BQ.1.1 | GRA |
| hCoV-19/Algeria/79/2023 | EPI_ISL_17182696 | 03/01/2023 | Africa / Algeria / Algiers | Human | National SARS-CoV-2 genomic and variants surveillance program | Male | 40 | Live | unknown | National SARS-CoV-2 genomic and variants surveillance program | BQ.1.1 | GRA |
| hCoV-19/Algeria/174/2023 | EPI_ISL_17182697 | 04/01/2023 | Africa / Algeria / Algiers | Human | National SARS-CoV-2 genomic and variants surveillance program | Male | 69 | Live | unknown | National SARS-CoV-2 genomic and variants surveillance program | BQ.1.1 | GRA |
| hCoV-19/Algeria/276/2023 | EPI_ISL_17182698 | 08/01/2023 | Africa / Algeria / Algiers | Human | National SARS-CoV-2 genomic and variants surveillance program | Male | 50 | Live | unknown | National SARS-CoV-2 genomic and variants surveillance program | BQ.1.1.40 | GRA |
| hCoV-19/Algeria/410/2023 | EPI_ISL_17182699 | 10/01/2023 | Africa / Algeria / Algiers | Human | National SARS-CoV-2 genomic and variants surveillance program | Female | unknown | Live | unknown | National SARS-CoV-2 genomic and variants surveillance program | BQ.1.1 | GRA |
| hCoV-19/Algeria/563/2023 | EPI_ISL_17182700 | 12/01/2023 | Africa / Algeria / Algiers | Human | National SARS-CoV-2 genomic and variants surveillance program | Female | 65 | Live | unknown | National SARS-CoV-2 genomic and variants surveillance program | BN.3.1 | GRA |
| hCoV-19/Algeria/599/2023 | EPI_ISL_17182701 | 15/01/2023 | Africa / Algeria / Blida | Human | National SARS-CoV-2 genomic and variants surveillance program | Female | 93 | Live | unknown | National SARS-CoV-2 genomic and variants surveillance program | XBB.1.5.24 | GRA |
| hCoV-19/Algeria/641/2023 | EPI_ISL_17182702 | 17/01/2023 | Africa / Algeria / Medea | Human | National SARS-CoV-2 genomic and variants surveillance program | Male | 54 | Live | unknown | National SARS-CoV-2 genomic and variants surveillance program | BQ.1.1 | GRA |
| hCoV-19/Algeria/747/2023 | EPI_ISL_17182703 | 17/01/2023 | Africa / Algeria / Algiers | Human | National SARS-CoV-2 genomic and variants surveillance program | Male | unknown | Live | unknown | National SARS-CoV-2 genomic and variants surveillance program | XBB.1 | GRA |
| hCoV-19/Algeria/667/2023 | EPI_ISL_17182704 | 17/01/2023 | Africa / Algeria / Algiers | Human | National SARS-CoV-2 genomic and variants surveillance program | Female | 28 | Live | unknown | National SARS-CoV-2 genomic and variants surveillance program | BN.3.1 | GRA |
| hCoV-19/Algeria/1036/2023 | EPI_ISL_17182705 | 23/01/2023 | Africa / Algeria / Algiers | Human | National SARS-CoV-2 genomic and variants surveillance program | Female | 86 | Live | unknown | National SARS-CoV-2 genomic and variants surveillance program | BQ.1.22 | GRA |
| hCoV-19/Algeria/1037/2023 | EPI_ISL_17182706 | 23/01/2023 | Africa / Algeria / Algiers | Human | National SARS-CoV-2 genomic and variants surveillance program | Male | 48 | Live | unknown | National SARS-CoV-2 genomic and variants surveillance program | BQ.1.1.59 | GRA |
| hCoV-19/Algeria/1305/2023 | EPI_ISL_17182707 | 31/01/2023 | Africa / Algeria / Algiers | Human | National SARS-CoV-2 genomic and variants surveillance program | Male | unknown | Live | unknown | National SARS-CoV-2 genomic and variants surveillance program | BQ.1.1.59 | GRA |
| hCoV-19/Algeria/1367/2023 | EPI_ISL_17182708 | 30/01/2023 | Africa / Algeria / Algiers | Human | National SARS-CoV-2 genomic and variants surveillance program | Female | 33 | Live | unknown | National SARS-CoV-2 genomic and variants surveillance program | BQ.1.1.59 | GRA |
| hCoV-19/Algeria/1730/2023 | EPI_ISL_17182709 | 08/02/2023 | Africa / Algeria / Algiers | Human | National SARS-CoV-2 genomic and variants surveillance program | Female | 64 | Live | unknown | National SARS-CoV-2 genomic and variants surveillance program | XBB.1.5 | GRA |
| hCoV-19/Algeria/1952/2023 | EPI_ISL_17182710 | 19/02/2023 | Africa / Algeria / Algiers | Human | National SARS-CoV-2 genomic and variants surveillance program | Female | 42 | Live | unknown | National SARS-CoV-2 genomic and variants surveillance program | BQ.1.1.59 | GRA |
| hCoV-19/Algeria/1733/2023 | EPI_ISL_17182711 | 09/02/2023 | Africa / Algeria / Algiers | Human | National SARS-CoV-2 genomic and variants surveillance program | Female | 3 months | Live | unknown | National SARS-CoV-2 genomic and variants surveillance program | XBB.1.5 | GRA |
| hCoV-19/Algeria/36/2022 | EPI_ISL_17222018 | 21/01/2022 | Africa / Algeria / Bejaia | Human | National SARS-CoV-2 genomic and variants surveillance program | Female | 52 | Live | unknown | National SARS-CoV-2 genomic and variants surveillance program | BA.1.15 | GRA |
| hCoV-19/Algeria/2936/2022 | EPI_ISL_17222019 | 30/06/2022 | Africa / Algeria / Bejaia | Human | National SARS-CoV-2 genomic and variants surveillance program | Female | 74 | Live | unknown | National SARS-CoV-2 genomic and variants surveillance program | BA.5.2.1 | GRA |
| hCoV-19/Algeria/2951/2022 | EPI_ISL_17222020 | 12/07/2022 | Africa / Algeria / Bejaia | Human | National SARS-CoV-2 genomic and variants surveillance program | Female | unknown | Live | unknown | National SARS-CoV-2 genomic and variants surveillance program | BA.5.2 | GRA |
| hCoV-19/Algeria/2953/2022 | EPI_ISL_17222021 | 14/07/2022 | Africa / Algeria / Bejaia | Human | National SARS-CoV-2 genomic and variants surveillance program | Female | 30 | Live | unknown | National SARS-CoV-2 genomic and variants surveillance program | BA.5.2 | GRA |
| hCoV-19/Algeria/2954/2022 | EPI_ISL_17222022 | 14/07/2022 | Africa / Algeria / Bejaia | Human | National SARS-CoV-2 genomic and variants surveillance program | Female | 27 | Live | unknown | National SARS-CoV-2 genomic and variants surveillance program | BA.5.1 | GRA |
| hCoV-19/Algeria/2960/2022 | EPI_ISL_17222023 | 17/07/2022 | Africa / Algeria / Bejaia | Human | National SARS-CoV-2 genomic and variants surveillance program | Female | 29 | Live | unknown | National SARS-CoV-2 genomic and variants surveillance program | BA.5.2 | GRA |
| hCoV-19/Algeria/2961/2022 | EPI_ISL_17222024 | 17/07/2022 | Africa / Algeria / Bejaia | Human | National SARS-CoV-2 genomic and variants surveillance program | Female | 25 | Live | unknown | National SARS-CoV-2 genomic and variants surveillance program | BF.2 | GRA |
| hCoV-19/Algeria/2962/2022 | EPI_ISL_17222025 | 17/07/2022 | Africa / Algeria / Bejaia | Human | National SARS-CoV-2 genomic and variants surveillance program | Female | 48 | Live | unknown | National SARS-CoV-2 genomic and variants surveillance program | BE.1.1 | GRA |
| hCoV-19/Algeria/2966/2022 | EPI_ISL_17222026 | 19/07/2022 | Africa / Algeria / Bejaia | Human | National SARS-CoV-2 genomic and variants surveillance program | unknown | unknown | Live | unknown | National SARS-CoV-2 genomic and variants surveillance program | BA.5.1 | GRA |
| hCoV-19/Algeria/2967/2022 | EPI_ISL_17222027 | 19/07/2022 | Africa / Algeria / Bejaia | Human | National SARS-CoV-2 genomic and variants surveillance program | unknown | unknown | Live | unknown | National SARS-CoV-2 genomic and variants surveillance program | BA.5.1 | GRA |
| hCoV-19/Algeria/2971/2022 | EPI_ISL_17222028 | 19/07/2022 | Africa / Algeria / Bejaia | Human | National SARS-CoV-2 genomic and variants surveillance program | unknown | unknown | Live | unknown | National SARS-CoV-2 genomic and variants surveillance program | BA.5.2.20 | GRA |
| hCoV-19/Algeria/3267/2022 | EPI_ISL_17222029 | 18/12/2022 | Africa / Algeria / Bejaia | Human | National SARS-CoV-2 genomic and variants surveillance program | Male | 89 | Live | unknown | National SARS-CoV-2 genomic and variants surveillance program | BQ.1.1.59 | GRA |
| hCoV-19/Algeria/43980/2022 | EPI_ISL_17675167 | 24/11/2022 | Africa / Algeria / Algiers | Human | National SARS-CoV-2 genomic and variants surveillance program | Female | 222 | Live | unknown | National SARS-CoV-2 genomic and variants surveillance program | BQ.1.1 | GRA |
| hCoV-19/Algeria/44231/2022 | EPI_ISL_17675168 | 12/12/2022 | Africa / Algeria / Algiers | Human | National SARS-CoV-2 genomic and variants surveillance program | Female | 54 | Live | unknown | National SARS-CoV-2 genomic and variants surveillance program | BQ.1.1 | GRA |
| hCoV-19/Algeria/44379/2022 | EPI_ISL_17675169 | 15/12/2022 | Africa / Algeria / Algiers | Human | National SARS-CoV-2 genomic and variants surveillance program | Male | 222 | Live | unknown | National SARS-CoV-2 genomic and variants surveillance program | BQ.1.1 | GRA |
| hCoV-19/Algeria/44404/2022 | EPI_ISL_17675170 | 15/12/2022 | Africa / Algeria / Algiers | Human | National SARS-CoV-2 genomic and variants surveillance program | Female | 85 | Live | unknown | National SARS-CoV-2 genomic and variants surveillance program | BQ.1 | GRA |
| hCoV-19/Algeria/44345/2022 | EPI_ISL_17675171 | 15/12/2022 | Africa / Algeria / Blida | Human | National SARS-CoV-2 genomic and variants surveillance program | Male | 20 | Live | unknown | National SARS-CoV-2 genomic and variants surveillance program | BQ.1.1 | GRA |
| hCoV-19/Algeria/44329/2022 | EPI_ISL_17675172 | 13/12/2022 | Africa / Algeria / Algiers | Human | National SARS-CoV-2 genomic and variants surveillance program | Female | 78 | Live | unknown | National SARS-CoV-2 genomic and variants surveillance program | XBB.1 | GRA |
| hCoV-19/Algeria/44331/2022 | EPI_ISL_17675173 | 13/12/2022 | Africa / Algeria / Algiers | Human | National SARS-CoV-2 genomic and variants surveillance program | Female | 40 | Live | unknown | National SARS-CoV-2 genomic and variants surveillance program | BQ.1.1.59 | GRA |
| hCoV-19/Algeria/6040/2022 | EPI_ISL_17675174 | 11/07/2022 | Africa / Algeria / BISKRA | Human | National SARS-CoV-2 genomic and variants surveillance program | Male | 19 | Live | unknown | National SARS-CoV-2 genomic and variants surveillance program | BA.5.2 | GRA |
| hCoV-19/Algeria/15W/2022 | EPI_ISL_17675175 | 19/07/2022 | Africa / Algeria / BISKRA | Human | National SARS-CoV-2 genomic and variants surveillance program | Female | 23 | Live | unknown | National SARS-CoV-2 genomic and variants surveillance program | BA.5.2 | GRA |
| hCoV-19/Algeria/4388/2022 | EPI_ISL_17675176 | 16/07/2022 | Africa / Algeria / Tizi-Ouzou | Human | National SARS-CoV-2 genomic and variants surveillance program | Female | 31 | Live | unknown | National SARS-CoV-2 genomic and variants surveillance program | BA.5.1 | GRA |
| hCoV-19/Algeria/4469/2022 | EPI_ISL_17675177 | 20/07/2022 | Africa / Algeria / Tizi-Ouzou | Human | National SARS-CoV-2 genomic and variants surveillance program | Male | 43 | Live | unknown | National SARS-CoV-2 genomic and variants surveillance program | BA.5.2 | GRA |
| hCoV-19/Algeria/4472/2022 | EPI_ISL_17675178 | 20/07/2022 | Africa / Algeria / Tizi-Ouzou | Human | National SARS-CoV-2 genomic and variants surveillance program | Male | 71 | Live | unknown | National SARS-CoV-2 genomic and variants surveillance program | BA.5.2 | GRA |
| hCoV-19/Algeria/4474/2022 | EPI_ISL_17675179 | 20/07/2022 | Africa / Algeria / Tizi-Ouzou | Human | National SARS-CoV-2 genomic and variants surveillance program | Male | 33 | Live | unknown | National SARS-CoV-2 genomic and variants surveillance program | BA.5.1 | GRA |
| hCoV-19/Algeria/44793/2022 | EPI_ISL_17675180 | 20/07/2022 | Africa / Algeria / Tizi-Ouzou | Human | National SARS-CoV-2 genomic and variants surveillance program | Female | 47 | Live | unknown | National SARS-CoV-2 genomic and variants surveillance program | BA.5.2 | GRA |
| hCoV-19/Algeria/4506/2022 | EPI_ISL_17675181 | 21/07/2022 | Africa / Algeria / Tizi-Ouzou | Human | National SARS-CoV-2 genomic and variants surveillance program | Female | 78 | Live | unknown | National SARS-CoV-2 genomic and variants surveillance program | BA.5.2 | GRA |
| hCoV-19/Algeria/4542/2022 | EPI_ISL_17675182 | 25/07/2022 | Africa / Algeria / Tizi-Ouzou | Human | National SARS-CoV-2 genomic and variants surveillance program | Male | 31 | Live | unknown | National SARS-CoV-2 genomic and variants surveillance program | BA.5.2 | GRA |
| hCoV-19/Algeria/4543/2022 | EPI_ISL_17675183 | 25/07/2022 | Africa / Algeria / Tizi-Ouzou | Human | National SARS-CoV-2 genomic and variants surveillance program | Male | 02 months | Live | unknown | National SARS-CoV-2 genomic and variants surveillance program | BF.5 | GRA |
| hCoV-19/Algeria/4545/2022 | EPI_ISL_17675184 | 25/07/2022 | Africa / Algeria / Tizi-Ouzou | Human | National SARS-CoV-2 genomic and variants surveillance program | Female | 84 | Live | unknown | National SARS-CoV-2 genomic and variants surveillance program | BA.5.2.1 | GRA |
| hCoV-19/Algeria/4563/2022 | EPI_ISL_17675185 | 25/07/2022 | Africa / Algeria / Tizi-Ouzou | Human | National SARS-CoV-2 genomic and variants surveillance program | Female | 27 | Live | unknown | National SARS-CoV-2 genomic and variants surveillance program | BA.5.2.1 | GRA |
| hCoV-19/Algeria/4575/2022 | EPI_ISL_17675186 | 26/07/2022 | Africa / Algeria / Tizi-Ouzou | Human | National SARS-CoV-2 genomic and variants surveillance program | Male | 37 | Live | unknown | National SARS-CoV-2 genomic and variants surveillance program | BA.5.2 | GRA |
| hCoV-19/Algeria/1154/2022 | EPI_ISL_17675187 | 12/07/2022 | Africa / Algeria / Setif | Human | National SARS-CoV-2 genomic and variants surveillance program | Female | 62 | Live | unknown | National SARS-CoV-2 genomic and variants surveillance program | BA.5.2 | GRA |
| hCoV-19/Algeria/1163/2022 | EPI_ISL_17675188 | 21/07/2022 | Africa / Algeria / Setif | Human | National SARS-CoV-2 genomic and variants surveillance program | Male | 38 | Live | unknown | National SARS-CoV-2 genomic and variants surveillance program | BA.5.2 | GRA |
| hCoV-19/Algeria/1165/2022 | EPI_ISL_17675189 | 21/07/2022 | Africa / Algeria / Setif | Human | National SARS-CoV-2 genomic and variants surveillance program | Female | 26 | Live | unknown | National SARS-CoV-2 genomic and variants surveillance program | BA.5.2 | GRA |
| hCoV-19/Algeria/1175/2022 | EPI_ISL_17675190 | 21/07/2022 | Africa / Algeria / Setif | Human | National SARS-CoV-2 genomic and variants surveillance program | Male | 6 | Live | unknown | National SARS-CoV-2 genomic and variants surveillance program | BA.5.2 | GRA |
| hCoV-19/Algeria/1185/2022 | EPI_ISL_17675191 | 24/07/2022 | Africa / Algeria / Setif | Human | National SARS-CoV-2 genomic and variants surveillance program | Female | 52 | Live | unknown | National SARS-CoV-2 genomic and variants surveillance program | BA.5.2 | GRA |
| hCoV-19/Algeria/1188/2022 | EPI_ISL_17675192 | 25/07/2022 | Africa / Algeria / Setif | Human | National SARS-CoV-2 genomic and variants surveillance program | Female | 30 | Live | unknown | National SARS-CoV-2 genomic and variants surveillance program | BA.5.2.18 | GRA |
| hCoV-19/Algeria/1215/2022 | EPI_ISL_17675193 | 25/07/2022 | Africa / Algeria / Setif | Human | National SARS-CoV-2 genomic and variants surveillance program | Male | 39 | Live | unknown | National SARS-CoV-2 genomic and variants surveillance program | BA.5.2.18 | GRA |
| hCoV-19/Algeria/1216/2022 | EPI_ISL_17675194 | 25/07/2022 | Africa / Algeria / Setif | Human | National SARS-CoV-2 genomic and variants surveillance program | Male | 45 | Live | unknown | National SARS-CoV-2 genomic and variants surveillance program | BA.5.2.18 | GRA |
| hCoV-19/Algeria/1207/2022 | EPI_ISL_17675195 | 25/07/2022 | Africa / Algeria / Setif | Human | National SARS-CoV-2 genomic and variants surveillance program | Male | 80 | Live | unknown | National SARS-CoV-2 genomic and variants surveillance program | BA.5.2.18 | GRA |
| hCoV-19/Algeria/1222/2022 | EPI_ISL_17675196 | 26/07/2022 | Africa / Algeria / Setif | Human | National SARS-CoV-2 genomic and variants surveillance program | Female | 50 | Live | unknown | National SARS-CoV-2 genomic and variants surveillance program | BA.5.1 | GRA |
| hCoV-19/Algeria/1228/2022 | EPI_ISL_17675197 | 26/07/2022 | Africa / Algeria / Setif | Human | National SARS-CoV-2 genomic and variants surveillance program | Male | 39 | Live | unknown | National SARS-CoV-2 genomic and variants surveillance program | BA.5.2 | GRA |
| hCoV-19/Algeria/1231/2022 | EPI_ISL_17675198 | 26/07/2022 | Africa / Algeria / Setif | Human | National SARS-CoV-2 genomic and variants surveillance program | Female | 30 | Live | unknown | National SARS-CoV-2 genomic and variants surveillance program | BA.5.2.27 | GRA |
| hCoV-19/Algeria/1232/2022 | EPI_ISL_17675199 | 26/07/2022 | Africa / Algeria / Setif | Human | National SARS-CoV-2 genomic and variants surveillance program | Female | 32 | Live | unknown | National SARS-CoV-2 genomic and variants surveillance program | BA.5.2 | GRA |
| hCoV-19/Algeria/1240/2022 | EPI_ISL_17675200 | 26/07/2022 | Africa / Algeria / Setif | Human | National SARS-CoV-2 genomic and variants surveillance program | Male | 66 | Live | unknown | National SARS-CoV-2 genomic and variants surveillance program | BA.5.2 | GRA |
| hCoV-19/Algeria/1238/2022 | EPI_ISL_17675201 | 27/07/2022 | Africa / Algeria / Setif | Human | National SARS-CoV-2 genomic and variants surveillance program | Female | 36 | Live | unknown | National SARS-CoV-2 genomic and variants surveillance program | BA.5.2.44 | GRA |
| hCoV-19/Algeria/1246/2022 | EPI_ISL_17675202 | 27/07/2022 | Africa / Algeria / Setif | Human | National SARS-CoV-2 genomic and variants surveillance program | Female | 49 | Live | unknown | National SARS-CoV-2 genomic and variants surveillance program | BA.5.2.20 | GRA |
| hCoV-19/Algeria/1250/2022 | EPI_ISL_17675203 | 27/07/2022 | Africa / Algeria / Setif | Human | National SARS-CoV-2 genomic and variants surveillance program | Female | 34 | Live | unknown | National SARS-CoV-2 genomic and variants surveillance program | BA.5.2 | GRA |
| hCoV-19/Algeria/1255/2022 | EPI_ISL_17675204 | 28/07/2022 | Africa / Algeria / Setif | Human | National SARS-CoV-2 genomic and variants surveillance program | Female | 42 | Live | unknown | National SARS-CoV-2 genomic and variants surveillance program | BA.5.2 | GRA |
| hCoV-19/Algeria/1260/2022 | EPI_ISL_17675205 | 31/07/2022 | Africa / Algeria / Setif | Human | National SARS-CoV-2 genomic and variants surveillance program | Female | 30 | Live | unknown | National SARS-CoV-2 genomic and variants surveillance program | BA.5.2 | GRA |
| hCoV-19/Algeria/1263/2022 | EPI_ISL_17675206 | 01/08/2022 | Africa / Algeria / Setif | Human | National SARS-CoV-2 genomic and variants surveillance program | Female | 62 | Live | unknown | National SARS-CoV-2 genomic and variants surveillance program | BA.5.2.20 | GRA |
| hCoV-19/Algeria/1272/2022 | EPI_ISL_17675207 | 02/08/2022 | Africa / Algeria / Setif | Human | National SARS-CoV-2 genomic and variants surveillance program | Female | 77 | Live | unknown | National SARS-CoV-2 genomic and variants surveillance program | BA.5.2.1 | GRA |
| hCoV-19/Algeria/1273/2022 | EPI_ISL_17675208 | 02/08/2022 | Africa / Algeria / Setif | Human | National SARS-CoV-2 genomic and variants surveillance program | Female | 86 | Live | unknown | National SARS-CoV-2 genomic and variants surveillance program | BA.5.1.22 | GRA |
| hCoV-19/Algeria/1275/2022 | EPI_ISL_17675209 | 02/08/2022 | Africa / Algeria / Setif | Human | National SARS-CoV-2 genomic and variants surveillance program | Female | 88 | Live | unknown | National SARS-CoV-2 genomic and variants surveillance program | BE.1.1 | GRA |
| hCoV-19/Algeria/1276/2022 | EPI_ISL_17675210 | 02/08/2022 | Africa / Algeria / Setif | Human | National SARS-CoV-2 genomic and variants surveillance program | Male | 75 | Live | unknown | National SARS-CoV-2 genomic and variants surveillance program | BA.5.2 | GRA |
| hCoV-19/Algeria/1287/2022 | EPI_ISL_17675211 | 04/08/2022 | Africa / Algeria / Setif | Human | National SARS-CoV-2 genomic and variants surveillance program | Female | 74 | Live | unknown | National SARS-CoV-2 genomic and variants surveillance program | BA.5.2.1 | GRA |
| hCoV-19/Algeria/5167/2023 | EPI_ISL_17985280 | 08/05/2023 | Africa / Algeria / Medea | Human | National SARS-CoV-2 genomic and variants surveillance program | Female | 38 | Live | unknown | National SARS-CoV-2 genomic and variants surveillance program | XBB.1.9.1 | GRA |
| hCoV-19/Algeria/28932/2023 | EPI_ISL_17985281 | 03/04/2023 | Africa / Algeria / Oran | Human | National SARS-CoV-2 genomic and variants surveillance program | Female | 32 | Live | unknown | National SARS-CoV-2 genomic and variants surveillance program | XBB.1.5.14 | GRA |
| hCoV-19/Algeria/28935/2023 | EPI_ISL_17985282 | 09/04/2023 | Africa / Algeria / Oran | Human | National SARS-CoV-2 genomic and variants surveillance program | Female | 29 | Live | unknown | National SARS-CoV-2 genomic and variants surveillance program | XBB.1.9.1 | GRA |
| hCoV-19/Algeria/28938/2023 | EPI_ISL_17985283 | 09/04/2023 | Africa / Algeria / Oran | Human | National SARS-CoV-2 genomic and variants surveillance program | Female | 47 | Live | unknown | National SARS-CoV-2 genomic and variants surveillance program | XBB.1.5.63 | GRA |
| hCoV-19/Algeria/28960/2023 | EPI_ISL_17985284 | 18/04/2023 | Africa / Algeria / Oran | Human | National SARS-CoV-2 genomic and variants surveillance program | Female | 34 | Live | unknown | National SARS-CoV-2 genomic and variants surveillance program | FL.10 | GRA |
| hCoV-19/Algeria/28978/2023 | EPI_ISL_17985285 | 25/04/2023 | Africa / Algeria / Oran | Human | National SARS-CoV-2 genomic and variants surveillance program | Female | 40 | Live | unknown | National SARS-CoV-2 genomic and variants surveillance program | BQ.1.1 | GRA |
| hCoV-19/Algeria/28979/2023 | EPI_ISL_17985286 | 25/04/2023 | Africa / Algeria / Oran | Human | National SARS-CoV-2 genomic and variants surveillance program | Female | 26 | Live | unknown | National SARS-CoV-2 genomic and variants surveillance program | XBB.1.5.63 | GRA |
| hCoV-19/Algeria/28989/2023 | EPI_ISL_17985287 | 27/04/2023 | Africa / Algeria / Oran | Human | National SARS-CoV-2 genomic and variants surveillance program | Female | 30 | Live | unknown | National SARS-CoV-2 genomic and variants surveillance program | XBB.1.9.2 | GRA |
| hCoV-19/Algeria/28991/2023 | EPI_ISL_17985288 | 29/04/2023 | Africa / Algeria / Oran | Human | National SARS-CoV-2 genomic and variants surveillance program | Female | 87 | Live | unknown | National SARS-CoV-2 genomic and variants surveillance program | XBB.1.28.1 | GRA |
| hCoV-19/Algeria/28993/2023 | EPI_ISL_17985289 | 29/04/2023 | Africa / Algeria / Oran | Human | National SARS-CoV-2 genomic and variants surveillance program | Female | 36 | Live | unknown | National SARS-CoV-2 genomic and variants surveillance program | FL.10 | GRA |
| hCoV-19/Algeria/29008/2023 | EPI_ISL_17985290 | 05/04/2023 | Africa / Algeria / Oran | Human | National SARS-CoV-2 genomic and variants surveillance program | Female | 23 | Live | unknown | National SARS-CoV-2 genomic and variants surveillance program | FL.10 | GRA |
| hCoV-19/Algeria/28950/2023 | EPI_ISL_17985291 | 15/04/2023 | Africa / Algeria / Oran | Human | National SARS-CoV-2 genomic and variants surveillance program | unknown | 36 | Live | unknown | National SARS-CoV-2 genomic and variants surveillance program | XBB.1.5 | GRA |
| hCoV-19/Algeria/28957/2023 | EPI_ISL_17985292 | 17/04/2023 | Africa / Algeria / Oran | Human | National SARS-CoV-2 genomic and variants surveillance program | unknown | 38 | Live | unknown | National SARS-CoV-2 genomic and variants surveillance program | XBV | GRA |
| hCoV-19/Algeria/28959/2023 | EPI_ISL_17985293 | 17/04/2023 | Africa / Algeria / Oran | Human | National SARS-CoV-2 genomic and variants surveillance program | unknown | unknown | Live | unknown | National SARS-CoV-2 genomic and variants surveillance program | XBB.1.9.1 | GRA |
| hCoV-19/Algeria/28964/2023 | EPI_ISL_17985294 | 18/04/2023 | Africa / Algeria / Oran | Human | National SARS-CoV-2 genomic and variants surveillance program | unknown | unknown | Live | unknown | National SARS-CoV-2 genomic and variants surveillance program | XBB.1.5.63 | GRA |
| hCoV-19/Algeria/28983/2023 | EPI_ISL_17985295 | 26/04/2023 | Africa / Algeria / Oran | Human | National SARS-CoV-2 genomic and variants surveillance program | unknown | 31 | Live | unknown | National SARS-CoV-2 genomic and variants surveillance program | XBB.1.5 | GRA |
| hCoV-19/Algeria/29005/2023 | EPI_ISL_17985296 | 04/05/2023 | Africa / Algeria / Oran | Human | National SARS-CoV-2 genomic and variants surveillance program | unknown | 52 | Live | unknown | National SARS-CoV-2 genomic and variants surveillance program | XBB.1.5.24 | GRA |
| hCoV-19/Algeria/4452/2023 | EPI_ISL_17986356 | 18/04/2023 | Africa / Algeria / Blida | Human | National SARS-CoV-2 genomic and variants surveillance program | Female | 43 | Live | unknown | National SARS-CoV-2 genomic and variants surveillance program | XBB.1.17.1 | GRA |
| hCoV-19/Algeria/4535/2023 | EPI_ISL_17986357 | 24/04/2023 | Africa / Algeria / Tissemsilt | Human | National SARS-CoV-2 genomic and variants surveillance program | Male | 57 | Live | unknown | National SARS-CoV-2 genomic and variants surveillance program | XBB.1.28.1 | GRA |
| hCoV-19/Algeria/4758/2023 | EPI_ISL_17986358 | 30/04/2023 | Africa / Algeria / Medea | Human | National SARS-CoV-2 genomic and variants surveillance program | Male | 83 | Live | unknown | National SARS-CoV-2 genomic and variants surveillance program | FL.1 | GRA |
| hCoV-19/Algeria/4757/2023 | EPI_ISL_17986359 | 29/04/2023 | Africa / Algeria / Medea | Human | National SARS-CoV-2 genomic and variants surveillance program | Male | 61 | Live | unknown | National SARS-CoV-2 genomic and variants surveillance program | XBB.1.9.1 | GRA |
| hCoV-19/Algeria/5076/2023 | EPI_ISL_17986360 | 03/05/2023 | Africa / Algeria / Blida | Human | National SARS-CoV-2 genomic and variants surveillance program | Male | 57 | Live | unknown | National SARS-CoV-2 genomic and variants surveillance program | XBB.1.9.1 | GRA |
| hCoV-19/Algeria/3479/2023 | EPI_ISL_17986361 | 20/03/2023 | Africa / Algeria / Tebessa | Human | National SARS-CoV-2 genomic and variants surveillance program | Female | 54 | Live | unknown | National SARS-CoV-2 genomic and variants surveillance program | EG.4 | GRA |
| hCoV-19/Algeria/3530/2023 | EPI_ISL_17986362 | 26/03/2023 | Africa / Algeria / Algiers | Human | National SARS-CoV-2 genomic and variants surveillance program | Male | unknown | Live | unknown | National SARS-CoV-2 genomic and variants surveillance program | XBB.1.5.63 | GRA |
| hCoV-19/Algeria/3863/2023 | EPI_ISL_17986363 | 01/04/2023 | Africa / Algeria / Algiers | Human | National SARS-CoV-2 genomic and variants surveillance program | Female | 80 | Live | unknown | National SARS-CoV-2 genomic and variants surveillance program | XBB.1.9.1 | GRA |
| hCoV-19/Algeria/3866/2023 | EPI_ISL_17986364 | 04/04/2023 | Africa / Algeria / Algiers | Human | National SARS-CoV-2 genomic and variants surveillance program | Male | unknown | Live | unknown | National SARS-CoV-2 genomic and variants surveillance program | FL.4 | GRA |
| hCoV-19/Algeria/3484/2023 | EPI_ISL_18090021 | 23/03/2023 | Africa / Algeria / Algiers | Human | National SARS-CoV-2 genomic and variants surveillance program | Male | 55 | Live | unknown | National SARS-CoV-2 genomic and variants surveillance program | XBB.1 | GRA |
| hCoV-19/Algeria/4759/2023 | EPI_ISL_18090022 | 30/04/2023 | Africa / Algeria / Medea | Human | National SARS-CoV-2 genomic and variants surveillance program | Female | 61 | Live | unknown | National SARS-CoV-2 genomic and variants surveillance program | XBB.1.9.1 | GRA |
| hCoV-19/Algeria/4840/2023 | EPI_ISL_18090023 | 26/04/2023 | Africa / Algeria / Tebessa | Human | National SARS-CoV-2 genomic and variants surveillance program | Female | 28 | Live | unknown | National SARS-CoV-2 genomic and variants surveillance program | XBB.1.9.2 | GRA |
| hCoV-19/Algeria/4647/2023 | EPI_ISL_18090024 | 26/04/2023 | Africa / Algeria / Algiers | Human | National SARS-CoV-2 genomic and variants surveillance program | Male | 22 | Live | unknown | National SARS-CoV-2 genomic and variants surveillance program | FL.4 | GRA |
| hCoV-19/Algeria/3659/2023 | EPI_ISL_18090025 | 29/03/2023 | Africa / Algeria / Algiers | Human | National SARS-CoV-2 genomic and variants surveillance program | Female | 83 | Live | unknown | National SARS-CoV-2 genomic and variants surveillance program | XBB.1.9.1 | GRA |
| hCoV-19/Algeria/5414/2023 | EPI_ISL_18090026 | 10/05/2023 | Africa / Algeria / Algiers | Human | National SARS-CoV-2 genomic and variants surveillance program | Female | 2 months | Live | unknown | National SARS-CoV-2 genomic and variants surveillance program | XBB.1.5.43 | GRA |
| hCoV-19/Algeria/5757/2023 | EPI_ISL_18090027 | 19/05/2023 | Africa / Algeria / Algiers | Human | National SARS-CoV-2 genomic and variants surveillance program | Female | 28 | Live | unknown | National SARS-CoV-2 genomic and variants surveillance program | XBB.1.28.1 | GRA |
| hCoV-19/Algeria/28915/2023 | EPI_ISL_18090028 | 26/03/2023 | Africa / Algeria / Oran | Human | National SARS-CoV-2 genomic and variants surveillance program | Female | 28 | Live | unknown | National SARS-CoV-2 genomic and variants surveillance program | XBB.1.5.63 | GRA |
| hCoV-19/Algeria/28952/2023 | EPI_ISL_18090029 | 16/04/2023 | Africa / Algeria / Oran | Human | National SARS-CoV-2 genomic and variants surveillance program | Male | 32 | Live | unknown | National SARS-CoV-2 genomic and variants surveillance program | XBB.1.9.1 | GRA |
| hCoV-19/Algeria/28943/2023 | EPI_ISL_18090030 | 11/04/2023 | Africa / Algeria / Oran | Human | National SARS-CoV-2 genomic and variants surveillance program | Male | unknown | Live | unknown | National SARS-CoV-2 genomic and variants surveillance program | FL.1 | GRA |
| hCoV-19/Algeria/28988/2023 | EPI_ISL_18090031 | 27/04/2023 | Africa / Algeria / Oran | Human | National SARS-CoV-2 genomic and variants surveillance program | Female | 42 | Live | unknown | National SARS-CoV-2 genomic and variants surveillance program | FL.10 | GRA |
| hCoV-19/Algeria/29016/2023 | EPI_ISL_18090032 | 06/05/2023 | Africa / Algeria / Algiers | Human | National SARS-CoV-2 genomic and variants surveillance program | unknown | unknown | Live | unknown | National SARS-CoV-2 genomic and variants surveillance program | FL.10 | GRA |
| hCoV-19/Algeria/36421/2021 | EPI_ISL_3161806 | 10/05/2021 | Africa / Algeria / Ouargla | Human | unknown | Male | 45 | unknown | unknown | unknown | B.1.617.2 | GK |
| hCoV-19/Algeria/36420/2021 | EPI_ISL_3161807 | 10/05/2021 | Africa / Algeria / Ouargla | Human | unknown | Male | 38 | unknown | unknown | unknown | B.1.617.2 | GK |
| hCoV-19/Algeria/36417/2021 | EPI_ISL_3161808 | 10/05/2021 | Africa / Algeria / Ouargla | Human | unknown | Male | 43 | unknown | unknown | unknown | B.1.617.2 | GK |
| hCoV-19/Algeria/48875/2021 | EPI_ISL_3161809 | 21/06/2021 | Africa / Algeria / Algiers | Human | unknown | Male | 16 | unknown | unknown | unknown | B.1.617.2 | GK |
| hCoV-19/Algeria/48372/2021 | EPI_ISL_3161810 | 20/06/2021 | Africa / Algeria / Algiers | Human | unknown | Male | 58 | unknown | unknown | unknown | B.1.617.2 | GK |
| hCoV-19/Algeria/SMII/2021 | EPI_ISL_3161811 | 23/06/2021 | Africa / Algeria / Algiers | Human | unknown | Male | 45 | unknown | unknown | unknown | AY.116 | GK |
| hCoV-19/Algeria/46836/2021 | EPI_ISL_3375624 | 14/06/2021 | Africa / Algeria / Medea | Human | unknown | Male | 67 | unknown | unknown | unknown | B.1.617.2 | GK |
| hCoV-19/Algeria/46851/2021 | EPI_ISL_3375625 | 14/06/2021 | Africa / Algeria / Algiers | Human | unknown | Female | 22 | unknown | unknown | unknown | B.1.617.2 | GK |
| hCoV-19/Algeria/47640/2021 | EPI_ISL_3375626 | 16/06/2021 | Africa / Algeria / Bouira | Human | unknown | Male | 62 | unknown | unknown | unknown | B.1.617.2 | GK |
| hCoV-19/Algeria/48915/2021 | EPI_ISL_3375627 | 22/06/2021 | Africa / Algeria / Algiers | Human | unknown | Male | 78 | unknown | unknown | unknown | B.1.617.2 | GK |
| hCoV-19/Algeria/49227/2021 | EPI_ISL_3375628 | 22/06/2021 | Africa / Algeria / Algiers | Human | unknown | Female | 52 | unknown | unknown | unknown | B.1.617.2 | GK |
| hCoV-19/Algeria/49926/2021 | EPI_ISL_3375629 | 24/06/2021 | Africa / Algeria / Laghouat | Human | unknown | Male | 35 | unknown | unknown | unknown | B.1.617.2 | GK |
| hCoV-19/Algeria/50100/2021 | EPI_ISL_3375630 | 24/06/2021 | Africa / Algeria / Blida | Human | unknown | Male | 27 | unknown | unknown | unknown | B.1.617.2 | GK |
| hCoV-19/Algeria/50291/2021 | EPI_ISL_3375631 | 27/06/2021 | Africa / Algeria / Blida | Human | unknown | Female | 29 | unknown | unknown | unknown | B.1.617.2 | GK |
| hCoV-19/Algeria/51743/2021 | EPI_ISL_3375632 | 30/06/2021 | Africa / Algeria / Algiers | Human | unknown | Male | 52 | unknown | unknown | unknown | AY.122 | GK |
| hCoV-19/Algeria/57433/2021 | EPI_ISL_3375633 | 13/07/2021 | Africa / Algeria / Algiers | Human | unknown | Female | 62 | unknown | unknown | unknown | B.1.617.2 | GK |
| hCoV-19/Algeria/57453/2021 | EPI_ISL_3375634 | 13/07/2021 | Africa / Algeria / Algiers | Human | unknown | Female | 52 | unknown | unknown | unknown | B.1.617.2 | GK |
| hCoV-19/Algeria/SJAA1081/2021 | EPI_ISL_3690016 | 28/07/2021 | Africa / Algeria / Eddis | Human | Breakthrough infection | Female | 45 | Ambulatory | AstraZeneca: 27/4 & 6/6/2021 | Breakthrough infection | B.1.617.2 | GK |
| hCoV-19/Algeria/43053/2021 | EPI_ISL_3718527 | 02/06/2021 | Africa / Algeria / Algiers | Human | unknown | Female | 86 | unknown | unknown | unknown | B.1.1.7 | GRY |
| hCoV-19/Algeria/43106/2021 | EPI_ISL_3718528 | 02/06/2021 | Africa / Algeria / Blida | Human | unknown | Female | 81 | unknown | unknown | unknown | B.1.1.7 | GRY |
| hCoV-19/Algeria/46472/2021 | EPI_ISL_3718529 | 14/06/2021 | Africa / Algeria / Bouira | Human | unknown | Male | 67 | unknown | unknown | unknown | B.1.1.7 | GRY |
| hCoV-19/Algeria/28629/2021 | EPI_ISL_3718530 | 12/04/2021 | Africa / Algeria / Algiers | Human | unknown | Male | 26 | unknown | unknown | unknown | B.1 | G |
| hCoV-19/Algeria/28624/2021 | EPI_ISL_3718531 | 12/04/2021 | Africa / Algeria / Algiers | Human | unknown | Female | 37 | unknown | unknown | unknown | B.1.1.7 | GRY |
| hCoV-19/Algeria/46955/2021 | EPI_ISL_3946135 | 14/06/2021 | Africa / Algeria / Bouira | Human | unknown | Male | 66 | unknown | unknown | unknown | A.27 | S |
| hCoV-19/Algeria/58/2021 | EPI_ISL_4004796 | 22/01/2021 | Africa / Algeria / Sidi Bel Abbes | Human | unknown | Male | 79 | unknown | unknown | unknown | B.1.160 | GH |
| hCoV-19/Algeria/512/2021 | EPI_ISL_4004797 | 17/03/2021 | Africa / Algeria / Sidi Bel Abbes | Human | unknown | Male | 74 | unknown | unknown | unknown | B.1.160 | GH |
| hCoV-19/Algeria/G0638_2264/2020 | EPI_ISL_418241 | 02/03/2020 | Africa / Algeria / Boufarik | Human | unknown | Female | 28 | Hospitalized | unknown | unknown | B.1 | GH |
| hCoV-19/Algeria/G0640_2265/2020 | EPI_ISL_418242 | 08/03/2020 | Africa / Algeria / Blida | Human | unknown | Male | 87 | Hospitalized | unknown | unknown | B.1 | GH |
| hCoV-19/Algeria/G0860_2262/2020 | EPI_ISL_420037 | 02/03/2020 | Africa / Algeria / Boufarik | Human | unknown | Male | 41 | Hospitalized | unknown | unknown | B.1 | GH |
| hCoV-19/Algeria/58229/2021 | EPI_ISL_5052196 | 14/07/2021 | Africa / Algeria / Algiers | Human | unknown | Female | 33 | unknown | unknown | unknown | B.1.525 | G |
| hCoV-19/Algeria/57568/2021 | EPI_ISL_5052197 | 08/07/2021 | Africa / Algeria / Tissemsilt | Human | unknown | Female | 50 | unknown | unknown | unknown | B.1.525 | G |
| hCoV-19/Algeria/56621/2021 | EPI_ISL_5052198 | 11/07/2021 | Africa / Algeria / Laghouat | Human | unknown | Male | 37 | unknown | unknown | unknown | B.1.525 | G |
| hCoV-19/Algeria/59015/2021 | EPI_ISL_5052199 | 12/07/2021 | Africa / Algeria / Bouira | Human | unknown | Male | 33 | unknown | unknown | unknown | B.1.1.7 | GRY |
| hCoV-19/Algeria/58641/2021 | EPI_ISL_5052200 | 28/07/2021 | Africa / Algeria / Djelfa | Human | unknown | Female | 39 | unknown | unknown | unknown | B.1.1.7 | GRY |
| hCoV-19/Algeria/55577/2021 | EPI_ISL_5052201 | 14/07/2021 | Africa / Algeria / Laghouat | Human | unknown | Female | 28 | unknown | unknown | unknown | B.1.1.7 | GRY |
| hCoV-19/Algeria/01064/2021 | EPI_ISL_5052202 | 15/06/2021 | Africa / Algeria / Sidi Bel Abbes | Human | unknown | Male | 79 | unknown | unknown | unknown | B.1.1.7 | GRY |
| hCoV-19/Algeria/61403/2021 | EPI_ISL_5052203 | 22/07/2021 | Africa / Algeria / Algiers | Human | unknown | Female | 36 | unknown | unknown | unknown | B.1.1.7 | GRY |
| hCoV-19/Algeria/60136/2021 | EPI_ISL_5052204 | 17/07/2021 | Africa / Algeria / El Oued | Human | unknown | Male | 55 | unknown | unknown | unknown | B.1.1.7 | GRY |
| hCoV-19/Algeria/17416/2021 | EPI_ISL_5052205 | 08/07/2021 | Africa / Algeria / Oran | Human | unknown | Male | 67 | unknown | unknown | unknown | B.1.1.7 | GR |
| hCoV-19/Algeria/71632/2021 | EPI_ISL_5052206 | 12/08/2021 | Africa / Algeria / Tindouf | Human | unknown | Male | 75 | unknown | unknown | unknown | B.1.617.2 | GK |
| hCoV-19/Algeria/00018/2021 | EPI_ISL_5052207 | 30/07/2021 | Africa / Algeria / Touggourt | Human | unknown | Female | 59 | unknown | unknown | unknown | B.1.617.2 | GK |
| hCoV-19/Algeria/00003/2021 | EPI_ISL_5052208 | 01/08/2021 | Africa / Algeria / Ouargla | Human | unknown | Female | 39 | unknown | unknown | unknown | B.1.617.2 | GK |
| hCoV-19/Algeria/03594/2021 | EPI_ISL_5052209 | 14/08/2021 | Africa / Algeria / Biskra | Human | unknown | Male | 55 | unknown | unknown | unknown | B.1.617.2 | GK |
| hCoV-19/Algeria/02615/2021 | EPI_ISL_5052210 | 11/07/2021 | Africa / Algeria / Sidi Bel Abbes | Human | unknown | Male | 59 | unknown | unknown | unknown | B.1.617.2 | GK |
| hCoV-19/Algeria/60317/2021 | EPI_ISL_5052211 | 19/07/2021 | Africa / Algeria / Blida | Human | unknown | Female | 22 | unknown | unknown | unknown | B.1.617.2 | GK |
| hCoV-19/Algeria/76465/2021 | EPI_ISL_5052212 | 01/09/2021 | Africa / Algeria / Batna | Human | unknown | Female | 3 | unknown | unknown | unknown | B.1.617.2 | GK |
| hCoV-19/Algeria/95495-46/2021 | EPI_ISL_7661152 | 10/12/2021 | Africa / Algeria / Algiers | Human | unknown | Male | 37 | Live | unknown | unknown | BA.1.1 | GRA |
| hCoV-19/Algeria/95495_44/2021 | EPI_ISL_7661153 | 10/12/2021 | Africa / Algeria / Algiers | Human | unknown | Male | 37 | Live | unknown | unknown | Unassigned | O |
| hCoV-19/Algeria/G41498-8846/2020 | EPI_ISL_766861 | 29/06/2020 | Africa / Algeria / Alger | Human | unknown | Female | 26 | unknown | unknown | unknown | B.1.597 | G |
| hCoV-19/Algeria/G42452-8847/2020 | EPI_ISL_766862 | 24/06/2020 | Africa / Algeria / Bouira | Human | unknown | Male | 88 | unknown | unknown | unknown | B.1.597 | GH |
| hCoV-19/Algeria/G37318-8849/2020 | EPI_ISL_766863 | 15/06/2020 | Africa / Algeria / Tipaza | Human | unknown | Female | 82 | unknown | unknown | unknown | B.1.36 | GH |
| hCoV-19/Algeria/G35155-8850/2020 | EPI_ISL_766864 | 15/07/2020 | Africa / Algeria / Bouira | Human | unknown | Male | 62 | unknown | unknown | unknown | B.1.1 | GR |
| hCoV-19/Algeria/G38218-8852/2020 | EPI_ISL_766865 | 19/06/2020 | Africa / Algeria / SÃ©tif | Human | unknown | Female | 38 | unknown | unknown | unknown | B.1.1 | GR |
| hCoV-19/Algeria/G38882-8853/2020 | EPI_ISL_766866 | 20/06/2020 | Africa / Algeria / Bouira | Human | unknown | Male | 52 | unknown | unknown | unknown | B.1.597 | GH |
| hCoV-19/Algeria/G37138-8854/2020 | EPI_ISL_766867 | 18/06/2020 | Africa / Algeria / Alger | Human | unknown | Male | 79 | unknown | unknown | unknown | B.1.597 | GH |
| hCoV-19/Algeria/G33030-8855/2020 | EPI_ISL_766868 | 2020-06 | Africa / Algeria / Alger | Human | unknown | Female | 70 | unknown | unknown | unknown | B.1 | G |
| hCoV-19/Algeria/G35014-8856/2020 | EPI_ISL_766869 | 09/06/2020 | Africa / Algeria / El Oued | Human | unknown | Male | 70 | unknown | unknown | unknown | B.1.1 | GR |
| hCoV-19/Algeria/G35081-8858/2020 | EPI_ISL_766870 | 15/06/2020 | Africa / Algeria / Tizi-Ouzou | Human | unknown | Female | 31 | unknown | unknown | unknown | B.1 | G |
| hCoV-19/Algeria/G38599-8859/2020 | EPI_ISL_766871 | 20/06/2020 | Africa / Algeria / Laghouat | Human | unknown | Female | 24 | unknown | unknown | unknown | B.1.1 | GR |
| hCoV-19/Algeria/G38029-8861/2020 | EPI_ISL_766872 | 19/06/2020 | Africa / Algeria / Bouira | Human | unknown | Male | 66 | unknown | unknown | unknown | B.1 | G |
| hCoV-19/Algeria/G40009-8862/2020 | EPI_ISL_766873 | 22/06/2020 | Africa / Algeria / Bordj-Bou-Arreridj | Human | unknown | Male | 51 | unknown | unknown | unknown | B.1 | GH |
| hCoV-19/Algeria/G43521-8863/2020 | EPI_ISL_766874 | 21/06/2020 | Africa / Algeria / Adrar | Human | unknown | Male | 42 | unknown | unknown | unknown | B.1 | GH |
| hCoV-19/Algeria/G37308-8864/2020 | EPI_ISL_766875 | 17/06/2020 | Africa / Algeria / Ouargla | Human | unknown | Female | 30 | unknown | unknown | unknown | B.1 | G |
| hCoV-19/Algeria/92827/2021 | EPI_ISL_7880242 | 30/11/2021 | Africa / Algeria / Algiers | Human | unknown | Male | 39 | Live | unknown | unknown | BA.1 | GRA |
| hCoV-19/Algeria/90942/2021 | EPI_ISL_8035611 | 20/11/2021 | Africa / Algeria / Laghouat | Human | unknown | Male | 36 | unknown | unknown | unknown | B.1.617.2 | GK |
| hCoV-19/Algeria/90943/2021 | EPI_ISL_8035612 | 20/11/2021 | Africa / Algeria / Laghouat | Human | unknown | Female | 85 | unknown | unknown | unknown | B.1.617.2 | GK |
| hCoV-19/Algeria/90944/2021 | EPI_ISL_8035613 | 20/11/2021 | Africa / Algeria / Laghouat | Human | unknown | Male | 53 | unknown | unknown | unknown | B.1.617.2 | GK |
| hCoV-19/Algeria/79788/2021 | EPI_ISL_8421063 | 17/09/2021 | Africa / Algeria / Medea | Human | Non-sentinel-surveillance (hospital) | Male | 55 | unknown | unknown | Non-sentinel-surveillance (hospital) | B.1.617.2 | GK |
| hCoV-19/Algeria/79735/2021 | EPI_ISL_8421064 | 10/09/2021 | Africa / Algeria / Bejaia | Human | Non-sentinel-surveillance (hospital) | Male | 50 | unknown | unknown | Non-sentinel-surveillance (hospital) | B.1.617.2 | GK |
| hCoV-19/Algeria/79727/2021 | EPI_ISL_8421065 | 10/09/2021 | Africa / Algeria / Bejaia | Human | Non-sentinel-surveillance (hospital) | Female | 41 | unknown | unknown | Non-sentinel-surveillance (hospital) | B.1.617.2 | GK |
| hCoV-19/Algeria/79234/2021 | EPI_ISL_8421066 | 12/09/2021 | Africa / Algeria / Blida | Human | Non-sentinel-surveillance (hospital) | Male | 40 | unknown | unknown | Non-sentinel-surveillance (hospital) | B.1.617.2 | GK |
| hCoV-19/Algeria/78620/2021 | EPI_ISL_8421067 | 12/09/2021 | Africa / Algeria / Bouira | Human | Non-sentinel-surveillance (hospital) | Female | 72 | unknown | unknown | Non-sentinel-surveillance (hospital) | B.1.617.2 | GK |
| hCoV-19/Algeria/77525/2021 | EPI_ISL_8421068 | 07/09/2021 | Africa / Algeria / Blida | Human | Non-sentinel-surveillance (hospital) | Female | 58 | unknown | unknown | Non-sentinel-surveillance (hospital) | B.1.617.2 | GK |
| hCoV-19/Algeria/76595/2021 | EPI_ISL_8421069 | 04/09/2021 | Africa / Algeria / Bouira | Human | Non-sentinel-surveillance (hospital) | Female | 67 | unknown | unknown | Non-sentinel-surveillance (hospital) | B.1.617.2 | GK |
