# Supplementary material for: Genomic surveillance of SARS-CoV-2 in North Africa: 4 years of GISAID data sharing
Source: IJID Reg. 2024 Mar 19;11:100356. doi: 10.1016/j.ijregi.2024.100356 (PMC11035039; doi:10.1016/j.ijregi.2024.100356)
Supplement: Supplementary file 6 [file mmc6.docx]

Table S5. Comprehensive Virus Data from Morocco Including Virus Name, Accession Number, and Clinical Attributes (based on data downloaded from GISAID per 15 September 2023)

| Virus name | Accession ID | Collection date | Location | Host | Sampling strategy | Gender | Patient age (years) | Patient status | Last vaccinated | Sampling strategy | Lineage | Clade |
| --- | --- | --- | --- | --- | --- | --- | --- | --- | --- | --- | --- | --- |
| hCoV-19/Morocco/IPM20386918/2021 | EPI_ISL_8523915 | 15/12/2021 | Africa / Morocco / Casablanca | Human | unknown | Female | 32 | unknown | unknown | unknown | B.1.1.529 | GRA |
| hCoV-19/Morocco/6896/2020 | EPI_ISL_459974 | 20/03/2020 | Africa / Morocco | Human | unknown | unknown | unknown | unknown | unknown | unknown | B.1 | G |
| hCoV-19/Morocco/FMP-239/2021 | EPI_ISL_5126276 | 24/03/2021 | Africa / Morocco / Rabat | Human | unknown | Male | 69 | unknown | unknown | unknown | B.1 | G |
| hCoV-19/Morocco/904/2022 | EPI_ISL_13259131 | 19/05/2022 | Africa / Morocco / Casablanca | Human | unknown | Male | 51 | Released | unknown | unknown | BA.2.9.3 | GRA |
| hCoV-19/Morocco/IPM20386510/2021 | EPI_ISL_8523913 | 13/12/2021 | Africa / Morocco / Casablanca | Human | unknown | Male | 62 | unknown | unknown | unknown | B.1.1.529 | GRA |
| hCoV-19/Morocco/894/2022 | EPI_ISL_13259123 | 20/05/2022 | Africa / Morocco / Casablanca | Human | unknown | Female | 25 | Released | unknown | unknown | BA.2 | GRA |
| hCoV-19/Morocco/890/2022 | EPI_ISL_13259120 | 18/05/2022 | Africa / Morocco / Casablanca | Human | unknown | Male | 44 | Released | unknown | unknown | BA.2 | GRA |
| hCoV-19/Morocco/891/2022 | EPI_ISL_13259121 | 20/05/2022 | Africa / Morocco / Casablanca | Human | unknown | Male | 15 | Released | unknown | unknown | BA.2.5 | GRA |
| hCoV-19/Morocco/895/2022 | EPI_ISL_13259127 | 20/05/2022 | Africa / Morocco / Rabat | Human | unknown | Female | 32 | Released | unknown | unknown | BA.5.2 | GRA |
| hCoV-19/Morocco/905/2022 | EPI_ISL_13259132 | 19/05/2022 | Africa / Morocco / Casablanca | Human | unknown | Male | 31 | Released | unknown | unknown | BA.2.9.3 | GRA |
| hCoV-19/Morocco/758/2022 | EPI_ISL_13289775 | 18/03/2022 | Africa / Morocco / Al Houceima | Human | unknown | Female | unknown | Intensive care | unknown | unknown | BA.1 | GRA |
| hCoV-19/Morocco/754/2022 | EPI_ISL_13289782 | 14/03/2022 | Africa / Morocco / Al Houceima | Human | unknown | Female | unknown | Intensive care | unknown | unknown | BA.1 | GRA |
| hCoV-19/Morocco/789/2022 | EPI_ISL_13289772 | 05/04/2022 | Africa / Morocco / Casablanca | Human | unknown | Female | 58 | Released | unknown | unknown | BA.2 | GRA |
| hCoV-19/Morocco/737/2022 | EPI_ISL_13289774 | 30/03/2022 | Africa / Morocco / Rabat | Human | unknown | Male | 31 | Hospitalized | unknown | unknown | B.1.177 | GV |
| hCoV-19/Morocco/772/2022 | EPI_ISL_13289771 | 31/03/2022 | Africa / Morocco / Casablanca | Human | unknown | Female | unknown | Released | unknown | unknown | BA.2 | GRA |
| hCoV-19/Morocco/756/2022 | EPI_ISL_13289779 | 16/03/2022 | Africa / Morocco / Al Houceima | Human | unknown | Female | unknown | Intensive care | unknown | unknown | BA.1 | GRA |
| hCoV-19/Morocco/765/2022 | EPI_ISL_13289773 | 30/03/2022 | Africa / Morocco / Fkih ben salah | Human | unknown | Female | 30 | Released | unknown | unknown | BA.1 | GRA |
| hCoV-19/Morocco/769/2022 | EPI_ISL_13289776 | 30/01/2022 | Africa / Morocco / Azilal | Human | unknown | Male | 40 | Intensive care | unknown | unknown | AY.33 | GK |
| hCoV-19/Morocco/790/2022 | EPI_ISL_13289778 | 08/04/2022 | Africa / Morocco / Casablanca | Human | unknown | Male | 71 | Released | unknown | unknown | BA.2 | GRA |
| hCoV-19/Morocco/770/2022 | EPI_ISL_13289783 | 30/01/2022 | Africa / Morocco / Azilal | Human | unknown | Male | 61 | Intensive care | unknown | unknown | AY.34 | GK |
| hCoV-19/Morocco/782/2022 | EPI_ISL_13289785 | 31/03/2022 | Africa / Morocco / Casablanca | Human | unknown | Male | 34 | Released | unknown | unknown | BA.2 | GRA |
| hCoV-19/Morocco/776/2022 | EPI_ISL_13289786 | 06/04/2022 | Africa / Morocco / Casablanca | Human | unknown | Female | 53 | Released | unknown | unknown | BA.2 | GRA |
| hCoV-19/Morocco/788/2022 | EPI_ISL_13289787 | 10/04/2022 | Africa / Morocco / Casablanca | Human | unknown | Male | 16 | Released | unknown | unknown | BA.2 | GRA |
| hCoV-19/Morocco/759/2022 | EPI_ISL_13289801 | 14/03/2022 | Africa / Morocco / Al Houceima | Human | unknown | Male | unknown | Intensive care | unknown | unknown | BA.1 | GRA |
| hCoV-19/Morocco/796/2022 | EPI_ISL_13289788 | 11/04/2022 | Africa / Morocco / Casablanca | Human | unknown | Female | 36 | Released | unknown | unknown | BA.2 | GRA |
| hCoV-19/Morocco/781/2022 | EPI_ISL_13289789 | 06/04/2022 | Africa / Morocco / Casablanca | Human | unknown | Female | 62 | Released | unknown | unknown | BA.2 | GRA |
| hCoV-19/Morocco/736/2022 | EPI_ISL_13289792 | 30/03/2022 | Africa / Morocco / Rabat | Human | unknown | Male | 91 | Hospitalized | unknown | unknown | BA.2 | GRA |
| hCoV-19/Morocco/793/2022 | EPI_ISL_13289793 | 09/04/2022 | Africa / Morocco / Casablanca | Human | unknown | Male | 70 | Released | unknown | unknown | BA.2 | GRA |
| hCoV-19/Morocco/798/2022 | EPI_ISL_13289794 | 11/04/2022 | Africa / Morocco / Casablanca | Human | unknown | Female | 41 | Released | unknown | unknown | BA.2 | GRA |
| hCoV-19/Morocco/755/2022 | EPI_ISL_13289795 | 15/03/2022 | Africa / Morocco / Al Houceima | Human | unknown | Female | unknown | Intensive care | unknown | unknown | BA.1 | GRA |
| hCoV-19/Morocco/757/2022 | EPI_ISL_13289796 | 17/03/2022 | Africa / Morocco / Al Houceima | Human | unknown | Male | unknown | Intensive care | unknown | unknown | BA.1 | GRA |
| hCoV-19/Morocco/724/2022 | EPI_ISL_13289800 | 25/03/2022 | Africa / Morocco / Casablanca | Human | unknown | Female | 35 | Released | unknown | unknown | BA.2 | GRA |
| hCoV-19/Morocco/728/2022 | EPI_ISL_13289797 | 30/03/2022 | Africa / Morocco / Casablanca | Human | unknown | Female | unknown | Released | unknown | unknown | BA.2 | GRA |
| hCoV-19/Morocco/771/2022 | EPI_ISL_13289798 | 30/03/2022 | Africa / Morocco / Mohammedia | Human | unknown | Female | 8 | Released | unknown | unknown | BA.2 | GRA |
| hCoV-19/Morocco/794/2022 | EPI_ISL_13289799 | 09/04/2022 | Africa / Morocco / Casablanca | Human | unknown | Male | 55 | Released | unknown | unknown | BA.2.3 | GRA |
| hCoV-19/Morocco/GR196/2021 | EPI_ISL_9439343 | 29/12/2021 | Africa / Morocco | Human | Active surveillance | Male | unknown | Released | unknown | Active surveillance | B.1.1.529 | GRA |
| hCoV-19/Morocco/831/2022 | EPI_ISL_13408039 | 18/04/2022 | Africa / Morocco / Casablanca | Human | unknown | Female | 77 | Live | unknown | unknown | BA.2 | GRA |
| hCoV-19/Morocco/880/2022 | EPI_ISL_13408035 | 10/05/2022 | Africa / Morocco / Casablanca | Human | unknown | Male | unknown | Live | unknown | unknown | BA.2 | GRA |
| hCoV-19/Morocco/882/2022 | EPI_ISL_13408043 | 06/05/2022 | Africa / Morocco / Casablanca | Human | unknown | Male | 27 | Live | unknown | unknown | BA.2.49 | GRA |
| hCoV-19/Morocco/879/2022 | EPI_ISL_13408040 | 07/05/2022 | Africa / Morocco / Casablanca | Human | unknown | Male | unknown | Live | unknown | unknown | BA.2 | GRA |
| hCoV-19/Morocco/881/2022 | EPI_ISL_13408042 | 06/05/2022 | Africa / Morocco / Casablanca | Human | unknown | Female | 54 | Live | unknown | unknown | BA.2 | GRA |
| hCoV-19/Morocco/876/2022 | EPI_ISL_13408036 | 09/05/2022 | Africa / Morocco / Casablanca | Human | unknown | Female | 67 | Live | unknown | unknown | BA.2.10 | GRA |
| hCoV-19/Morocco/826/2022 | EPI_ISL_13408033 | 21/04/2022 | Africa / Morocco / Casablanca | Human | unknown | Female | 22 | Live | unknown | unknown | BA.2 | GRA |
| hCoV-19/Morocco/828/2022 | EPI_ISL_13408032 | 25/04/2022 | Africa / Morocco / Casablanca | Human | unknown | Female | 34 | Live | unknown | unknown | BA.2 | GRA |
| hCoV-19/Morocco/833/2022 | EPI_ISL_13408041 | 09/05/2022 | Africa / Morocco / Casablanca | Human | unknown | Male | unknown | Live | unknown | unknown | BA.2 | GRA |
| hCoV-19/Morocco/884/2022 | EPI_ISL_13408044 | 09/05/2022 | Africa / Morocco / Casablanca | Human | unknown | Female | 28 | Live | unknown | unknown | BA.2.49 | GRA |
| hCoV-19/Morocco/595/2022 | EPI_ISL_13421937 | 16/01/2022 | Africa / Morocco / Temara | Human | unknown | Male | 36 | Intensive care | unknown | unknown | BA.1 | GRA |
| hCoV-19/Morocco/573/2022 | EPI_ISL_13421930 | 18/01/2022 | Africa / Morocco / Rabat | Human | unknown | Male | unknown | Intensive care | unknown | unknown | BA.1 | GRA |
| hCoV-19/Morocco/577/2022 | EPI_ISL_13421932 | 21/01/2022 | Africa / Morocco / Rabat | Human | unknown | Male | unknown | Intensive care | unknown | unknown | BA.1 | GRA |
| hCoV-19/Morocco/579/2022 | EPI_ISL_13421933 | 31/01/2022 | Africa / Morocco / Rabat | Human | unknown | Male | unknown | Intensive care | unknown | unknown | BA.1 | GRA |
| hCoV-19/Morocco/585/2022 | EPI_ISL_13421936 | 25/01/2022 | Africa / Morocco / Beni Mellal | Human | unknown | Male | 76 | Intensive care | unknown | unknown | BA.1 | GRA |
| hCoV-19/Morocco/596/2022 | EPI_ISL_13421938 | 16/01/2022 | Africa / Morocco / Temara | Human | unknown | Male | 31 | Intensive care | unknown | unknown | BA.1 | GRA |
| hCoV-19/Morocco/598/2022 | EPI_ISL_13421939 | 16/01/2022 | Africa / Morocco / Temara | Human | unknown | Male | 62 | Intensive care | unknown | unknown | BA.1 | GRA |
| hCoV-19/Morocco/604/2022 | EPI_ISL_13421941 | 13/01/2022 | Africa / Morocco / Khemisset | Human | unknown | Male | 51 | Intensive care | unknown | unknown | BA.1 | GRA |
| hCoV-19/Morocco/606/2022 | EPI_ISL_13421942 | 13/01/2022 | Africa / Morocco / Khemisset | Human | unknown | Male | 52 | Intensive care | unknown | unknown | BA.1 | GRA |
| hCoV-19/Morocco/619/2022 | EPI_ISL_13421946 | 13/02/2022 | Africa / Morocco / Fes | Human | unknown | Female | 35 | Intensive care | unknown | unknown | BA.1.1.1 | GRA |
| hCoV-19/Morocco/611/2022 | EPI_ISL_13421944 | 08/01/2022 | Africa / Morocco / Beni Mellal | Human | unknown | Female | 65 | Intensive care | unknown | unknown | BA.1 | GRA |
| hCoV-19/Morocco/IPM20387041/2021 | EPI_ISL_9417728 | 15/12/2021 | Africa / Morocco / Casablanca | Human | unknown | Male | 9 | unknown | unknown | unknown | B.1.617.2 | GK |
| hCoV-19/Morocco/IPM20387072/2021 | EPI_ISL_9417727 | 15/12/2021 | Africa / Morocco / Casablanca | Human | unknown | Male | 43 | unknown | unknown | unknown | B.1.617.2 | GK |
| hCoV-19/Morocco/IPM20386933/2021 | EPI_ISL_9417726 | 15/12/2021 | Africa / Morocco / Casablanca | Human | unknown | Female | 58 | unknown | unknown | unknown | B.1.617.2 | GK |
| hCoV-19/Morocco/IPM20386930/2021 | EPI_ISL_9417725 | 15/12/2021 | Africa / Morocco / Casablanca | Human | unknown | Male | 22 | unknown | unknown | unknown | AY.33 | GK |
| hCoV-19/Morocco/IPM20386824/2021 | EPI_ISL_9417723 | 15/12/2021 | Africa / Morocco / Casablanca | Human | unknown | Male | 58 | unknown | unknown | unknown | B.1.617.2 | GK |
| hCoV-19/Morocco/IPM20386674/2021 | EPI_ISL_9417721 | 14/12/2021 | Africa / Morocco / Casablanca | Human | unknown | Female | 48 | unknown | unknown | unknown | AY.33 | GK |
| hCoV-19/Morocco/6904/2020 | EPI_ISL_459982 | 18/04/2020 | Africa / Morocco | Human | unknown | unknown | unknown | unknown | unknown | unknown | B.1 | GH |
| hCoV-19/Morocco/FMP-340/2021 | EPI_ISL_4573207 | 13/07/2021 | Africa / Morocco / Agadir | Human | unknown | Female | unknown | unknown | unknown | unknown | AY.51 | GK |
| hCoV-19/Morocco/IPM20377700/2021 | EPI_ISL_6107655 | 15/10/2021 | Africa / Morocco / Mohammadia | Human | unknown | Female | 55 | unknown | unknown | unknown | B.1.617.2 | GK |
| hCoV-19/Morocco/59cc/2021 | EPI_ISL_9018763 | 13/07/2021 | Africa / Morocco | Human | Active surveillance | Male | unknown | Released | unknown | Active surveillance | AY.33 | GK |
| hCoV-19/Morocco/14CC/2021 | EPI_ISL_9019543 | 12/07/2021 | Africa / Morocco | Human | Active surveillance | Male | unknown | Released | unknown | Active surveillance | AY.33 | GK |
| hCoV-19/Morocco/IPM20382572/2021 | EPI_ISL_8186760 | 09/11/2021 | Africa / Morocco / Casablanca | Human | unknown | Female | 46 | unknown | unknown | unknown | AY.33 | GK |
| hCoV-19/Morocco/IPM20382808/2021 | EPI_ISL_8186758 | 10/11/2021 | Africa / Morocco / Casablanca | Human | unknown | Male | 62 | unknown | unknown | unknown | B.1.617.2 | GK |
| hCoV-19/Morocco/FMP-326/2021 | EPI_ISL_5118276 | 12/07/2021 | Africa / Morocco / Ouarzazate | Human | unknown | Male | unknown | unknown | unknown | unknown | B.1.1.7 | GRY |
| hCoV-19/Morocco/IPM20382988/2021 | EPI_ISL_8186756 | 11/11/2021 | Africa / Morocco / Casablanca | Human | unknown | Female | 30 | unknown | unknown | unknown | B.1.617.2 | GK |
| hCoV-19/Morocco/IPM20382805/2021 | EPI_ISL_8186755 | 10/11/2021 | Africa / Morocco / Casablanca | Human | unknown | Female | 65 | unknown | unknown | unknown | B.1.617.2 | GK |
| hCoV-19/Morocco/IPM20382591/2021 | EPI_ISL_8186754 | 09/11/2021 | Africa / Morocco / Casablanca | Human | unknown | Male | 29 | unknown | unknown | unknown | AY.33 | GK |
| hCoV-19/Morocco/IPM20382542/2021 | EPI_ISL_8186753 | 09/11/2021 | Africa / Morocco / Casablanca | Human | unknown | Female | 63 | unknown | unknown | unknown | AY.33 | GK |
| hCoV-19/Morocco/IPM20383893/2021 | EPI_ISL_8186749 | 18/11/2021 | Africa / Morocco / Casablanca | Human | unknown | Male | 67 | unknown | unknown | unknown | AY.34.1.1 | GK |
| hCoV-19/Morocco/IPM20383438/2021 | EPI_ISL_8186745 | 15/11/2021 | Africa / Morocco / Mohammadia | Human | unknown | Male | 56 | unknown | unknown | unknown | AY.33 | GK |
| hCoV-19/Morocco/CNRST-INH5900/2021 | EPI_ISL_6002900 | 08/07/2021 | Africa / Morocco / Casablanca | Human | unknown | Female | 60 | Released | unknown | unknown | AY.33 | GK |
| hCoV-19/Morocco/IPM20383359/2021 | EPI_ISL_8186744 | 15/11/2021 | Africa / Morocco / Mohammadia | Human | unknown | Female | 49 | unknown | unknown | unknown | AY.51 | GK |
| hCoV-19/Morocco/IPM20386249/2021 | EPI_ISL_8186742 | 10/12/2021 | Africa / Morocco / Casablanca | Human | unknown | Female | 18 | unknown | unknown | unknown | AY.33 | GK |
| hCoV-19/Morocco/CNRST-INH5858/2021 | EPI_ISL_6002899 | 07/07/2021 | Africa / Morocco / Casablanca | Human | unknown | Male | 55 | Released | unknown | unknown | AY.33 | GK |
| hCoV-19/Morocco/RMPS-04/2020 | EPI_ISL_469052 | 30/03/2020 | Africa / Morocco | Human | unknown | unknown | unknown | unknown | unknown | unknown | B.1 | GH |
| hCoV-19/Morocco/413/2022 | EPI_ISL_10019529 | 03/01/2022 | Africa / Morocco / Sale | Human | unknown | Male | 75 | Live - Intensive Care Unit | 06/03/2021 | unknown | AY.112 | GK |
| hCoV-19/Morocco/CNRST_CHU16/2021 | EPI_ISL_8629702 | 24/10/2021 | Africa / Morocco / Rabat | Human | unknown | Male | 14 | released | unknown | unknown | B.1.1.7 | GRY |
| hCoV-19/Morocco/RA62/2021 | EPI_ISL_8164977 | 20/09/2021 | Africa / Morocco | Human | Active surveillance | Male | unknown | Released | unknown | Active surveillance | AY.33 | GK |
| hCoV-19/Morocco/FMP-11/2020 | EPI_ISL_728291 | 2020-11 | Africa / Morocco / Rabat | Human | unknown | unknown | unknown | unknown | unknown | unknown | B.1.1 | GR |
| hCoV-19/Morocco/160/2021 | EPI_ISL_8135033 | 20/12/2021 | Africa / Morocco / Rabat | Human | unknown | Male | unknown | Live | unknown | unknown | BA.1 | GRA |
| hCoV-19/Morocco/FMP-396/2021 | EPI_ISL_8568500 | 2021 | Africa / Morocco / Fes | Human | unknown | unknown | unknown | unknown | unknown | unknown | BA.1.1 | GRA |
| hCoV-19/Morocco/FMP-393/2021 | EPI_ISL_8568495 | 2021 | Africa / Morocco / Rabat | Human | unknown | unknown | unknown | unknown | unknown | unknown | BA.1 | GRA |
| hCoV-19/Morocco/FMP-391/2021 | EPI_ISL_8568493 | 2021 | Africa / Morocco / Rabat | Human | unknown | unknown | unknown | unknown | unknown | unknown | BA.1 | GRA |
| hCoV-19/Morocco/FMP-390/2021 | EPI_ISL_8568490 | 2021 | Africa / Morocco / Rabat | Human | unknown | unknown | unknown | unknown | unknown | unknown | BA.1 | GRA |
| hCoV-19/Morocco/FMP-389/2021 | EPI_ISL_8568488 | 2021 | Africa / Morocco / Rabat | Human | unknown | unknown | unknown | unknown | unknown | unknown | BA.1 | GRA |
| hCoV-19/Morocco/119/2021 | EPI_ISL_8144254 | 22/12/2021 | Africa / Morocco / Tanger | Human | unknown | Male | unknown | Live | unknown | unknown | BA.1.1 | GRA |
| hCoV-19/Morocco/113/2021 | EPI_ISL_8144257 | 22/12/2021 | Africa / Morocco / Tanger | Human | unknown | Male | unknown | Live | unknown | unknown | BA.1.1 | GRA |
| hCoV-19/Morocco/147/2021 | EPI_ISL_8144259 | 22/12/2021 | Africa / Morocco / Kenitra | Human | unknown | Female | unknown | Live | unknown | unknown | BA.1 | GRA |
| hCoV-19/Morocco/98/2021 | EPI_ISL_8144260 | 21/12/2021 | Africa / Morocco / Fes | Human | unknown | Female | unknown | Live | unknown | unknown | BA.1.18 | GRA |
| hCoV-19/Morocco/166/2021 | EPI_ISL_8144262 | 24/12/2021 | Africa / Morocco / Tanger | Human | unknown | Male | unknown | Live | unknown | unknown | BA.1.1 | GRA |
| hCoV-19/Morocco/99/2021 | EPI_ISL_8144263 | 21/12/2021 | Africa / Morocco / Fes | Human | unknown | Male | unknown | Live | unknown | unknown | BA.1.1 | GRA |
| hCoV-19/Morocco/FMP-387/2021 | EPI_ISL_8543252 | 2021 | Africa / Morocco / Rabat | Human | unknown | unknown | unknown | unknown | unknown | unknown | BA.1.1 (consensus call) | GRA |
| hCoV-19/Morocco/SCS62/2021 | EPI_ISL_8132259 | 20/09/2021 | Africa / Morocco | Human | Active surveillance | Male | unknown | Released | unknown | Active surveillance | AY.33 | GK |
| hCoV-19/Morocco/CNRST_CHU14/2021 | EPI_ISL_8629700 | 23/10/2021 | Africa / Morocco / Rabat | Human | unknown | Female | 39 | released | unknown | unknown | B.1.1.7 | GRY |
| hCoV-19/Morocco/CNRST_CHU11/2021 | EPI_ISL_8629698 | 21/10/2021 | Africa / Morocco / Rabat | Human | unknown | Female | 41 | released | unknown | unknown | AY.33 | GK |
| hCoV-19/Morocco/CNRST_CHU01/2021 | EPI_ISL_8629695 | 15/10/2021 | Africa / Morocco / Rabat | Human | unknown | Female | 27 | released | unknown | unknown | B.1.1.7 | GRY |
| hCoV-19/Morocco/IPM20377157/2021 | EPI_ISL_5924607 | 13/10/2021 | Africa / Morocco / Casablanca | Human | unknown | Female | 57 | unknown | unknown | unknown | B.1.617.2 | GK |
| hCoV-19/Morocco/CNRST_HM36/2021 | EPI_ISL_8074123 | 22/12/2021 | Africa / Morocco / Rabat | Human | S gene dropout | Female | 32 | Released | Two doses Sinopharm, Last injection 23 August 2021 | S gene dropout | BA.1 | GRA |
| hCoV-19/Morocco/IPM20377092/2021 | EPI_ISL_5924605 | 13/10/2021 | Africa / Morocco / Casablanca | Human | unknown | Female | 60 | unknown | unknown | unknown | B.1.617.2 | GK |
| hCoV-19/Morocco/IPM20376776/2021 | EPI_ISL_5924601 | 12/10/2021 | Africa / Morocco / Casablanca | Human | unknown | Male | 49 | unknown | unknown | unknown | B.1.617.2 | GK |
| hCoV-19/Morocco/IPM20376509/2021 | EPI_ISL_5924596 | 12/10/2021 | Africa / Morocco / Casablanca | Human | unknown | Female | 32 | unknown | unknown | unknown | B.1.617.2 | GK |
| hCoV-19/Morocco/IPM20376587/2021 | EPI_ISL_5924598 | 12/10/2021 | Africa / Morocco / Mohammadia | Human | unknown | Male | 28 | unknown | unknown | unknown | B.1.617.2 | GK |
| hCoV-19/Morocco/20355473/2021 | EPI_ISL_4741156 | 31/08/2021 | Africa / Morocco / Casablanca | Human | unknown | Female | 17 | unknown | unknown | unknown | AY.33 | GK |
| hCoV-19/Morocco/IPM20376181/2021 | EPI_ISL_5924591 | 11/10/2021 | Africa / Morocco / Casablanca | Human | unknown | Female | 39 | unknown | unknown | unknown | B.1.617.2 | GK |
| hCoV-19/Morocco/20355435/2021 | EPI_ISL_4741155 | 30/08/2021 | Africa / Morocco / Casablanca | Human | unknown | Male | 38 | unknown | unknown | unknown | AY.33 | GK |
| hCoV-19/Morocco/20356364/2021 | EPI_ISL_4741162 | 31/08/2021 | Africa / Morocco / Casablanca | Human | unknown | Female | 55 | unknown | unknown | unknown | AY.73 | GK |
| hCoV-19/Morocco/71/2021 | EPI_ISL_8308356 | 16/12/2021 | Africa / Morocco / Sale | Human | unknown | Male | 51 | Live | 06/03/2021 | unknown | BA.1 | GRA |
| hCoV-19/Morocco/20356163/2021 | EPI_ISL_4741161 | 31/08/2021 | Africa / Morocco / Casablanca | Human | unknown | Male | 28 | unknown | unknown | unknown | AY.33 | GK |
| hCoV-19/Morocco/47/2021 | EPI_ISL_8308347 | 22/12/2021 | Africa / Morocco / Rabat | Human | unknown | Female | unknown | Live | unknown | unknown | BA.1 | GRA |
| hCoV-19/Morocco/72/2021 | EPI_ISL_8308342 | 14/12/2021 | Africa / Morocco / Sale | Human | unknown | Male | unknown | Live | unknown | unknown | BA.1 | GRA |
| hCoV-19/Morocco/43/2021 | EPI_ISL_8308345 | 15/12/2021 | Africa / Morocco / Rabat | Human | unknown | Male | unknown | Live | unknown | unknown | AY.33 | GK |
| hCoV-19/Morocco/70/2021 | EPI_ISL_8308351 | 16/12/2021 | Africa / Morocco / Sale | Human | unknown | Female | 79 | Live | 03/03/2021 | unknown | BA.1 | GRA |
| hCoV-19/Morocco/52/2021 | EPI_ISL_8308344 | 15/12/2021 | Africa / Morocco / Rabat | Human | unknown | Female | unknown | Live | unknown | unknown | BA.1 | GRA |
| hCoV-19/Morocco/51/2021 | EPI_ISL_8308346 | 14/12/2021 | Africa / Morocco / Rabat | Human | unknown | Male | unknown | Live | unknown | unknown | AY.133 | GK |
| hCoV-19/Morocco/44/2021 | EPI_ISL_8308341 | 15/12/2021 | Africa / Morocco / Sale | Human | unknown | Male | unknown | Live | unknown | unknown | AY.33 | GK |
| hCoV-19/Morocco/69/2021 | EPI_ISL_8308352 | 15/12/2021 | Africa / Morocco / Sale | Human | unknown | Female | 60 | Live | 03/03/2021 | unknown | BA.1 | GRA |
| hCoV-19/Morocco/63/2021 | EPI_ISL_8308354 | 04/12/2021 | Africa / Morocco / Casablanca | Human | unknown | Male | 59 | Live | unknown | unknown | AY.122 | GK |
| hCoV-19/Morocco/IPM20153056/2020 | EPI_ISL_17650192 | 19/08/2020 | Africa / Morocco / Casablanca | Human | unknown | Female | unknown | unknown | unknown | unknown | B.1 | G |
| hCoV-19/Morocco/IPM20435946/2023 | EPI_ISL_17650226 | 30/03/2023 | Africa / Morocco / Fes | Human | unknown | Female | 57 | unknown | unknown | unknown | EG.3 | GRA |
| hCoV-19/Morocco/249/2021 | EPI_ISL_10019535 | 29/12/2021 | Africa / Morocco / Rabat | Human | unknown | Female | unknown | Live | unknown | unknown | BA.1 | GRA |
| hCoV-19/Morocco/257/2021 | EPI_ISL_10019534 | 28/12/2021 | Africa / Morocco / Casablanca | Human | unknown | Female | 48 | Live | unknown | unknown | BA.1 | GRA |
| hCoV-19/Morocco/FMP-114/2021 | EPI_ISL_2259440 | 2021 | Africa / Morocco / Tinghir | Human | Baseline surveillance | unknown | unknown | unknown | unknown | Baseline surveillance | B.1.36 | GH |
| hCoV-19/Morocco/1117/2022 | EPI_ISL_14980808 | 01/07/2022 | Africa / Morocco / Casablanca | Human | unknown | Female | 67 | unknown | unknown | unknown | BA.5.2.20 | GRA |
| hCoV-19/Morocco/1109/2022 | EPI_ISL_14980801 | 15/08/2022 | Africa / Morocco / Casablanca | Human | unknown | Male | 32 | unknown | unknown | unknown | BA.5.2.20 | GRA |
| hCoV-19/Morocco/1111/2022 | EPI_ISL_14980803 | 10/08/2022 | Africa / Morocco / Casablanca | Human | unknown | Male | 32 | unknown | unknown | unknown | BA.5.1 | GRA |
| hCoV-19/Morocco/1110/2022 | EPI_ISL_14980802 | 11/08/2022 | Africa / Morocco / Casablanca | Human | unknown | Male | 35 | unknown | unknown | unknown | BA.5.2.1 | GRA |
| hCoV-19/Morocco/1112/2022 | EPI_ISL_14980804 | 17/08/2022 | Africa / Morocco / Casablanca | Human | unknown | Female | 30 | unknown | unknown | unknown | BA.5.2 | GRA |
| hCoV-19/Morocco/1114/2022 | EPI_ISL_14980806 | 03/07/2022 | Africa / Morocco / Casablanca | Human | unknown | Male | 53 | unknown | unknown | unknown | BA.5.2.20 | GRA |
| hCoV-19/Morocco/1118/2022 | EPI_ISL_14980809 | 27/06/2022 | Africa / Morocco / Casablanca | Human | unknown | Male | 70 | unknown | unknown | unknown | BA.2 | GRA |
| hCoV-19/Morocco/1113/2022 | EPI_ISL_14980805 | 13/08/2022 | Africa / Morocco / Casablanca | Human | unknown | Female | 28 | unknown | unknown | unknown | BA.5.2.20 | GRA |
| hCoV-19/Morocco/934/2022 | EPI_ISL_15111825 | 01/06/2022 | Africa / Morocco / Casablanca | Human | unknown | Male | 29 years | Live | unknown | unknown | BA.5.1 | GRA |
| hCoV-19/Morocco/920/2022 | EPI_ISL_15111817 | 30/05/2022 | Africa / Morocco / Casablanca | Human | unknown | Male | 31 years | Live | unknown | unknown | BA.2 | GRA |
| hCoV-19/Morocco/923/2022 | EPI_ISL_15111820 | 30/05/2022 | Africa / Morocco / Casablanca | Human | unknown | Female | 45 years | Live | unknown | unknown | BA.2 | GRA |
| hCoV-19/Morocco/943/2022 | EPI_ISL_15111830 | 07/06/2022 | Africa / Morocco / Rabat | Human | unknown | Female | 36 years | Live | unknown | unknown | BA.2 | GRA |
| hCoV-19/Morocco/921/2022 | EPI_ISL_15111818 | 30/05/2022 | Africa / Morocco / Casablanca | Human | unknown | Female | 48 years | Live | unknown | unknown | BA.5.2.20 | GRA |
| hCoV-19/Morocco/927/2022 | EPI_ISL_15111822 | 31/05/2022 | Africa / Morocco / Casablanca | Human | unknown | Male | 22 years | Live | unknown | unknown | XW | GRA |
| hCoV-19/Morocco/938/2022 | EPI_ISL_15111826 | 01/06/2022 | Africa / Morocco / Casablanca | Human | unknown | Male | 07 years | Live | unknown | unknown | BA.2 | GRA |
| hCoV-19/Morocco/947/2022 | EPI_ISL_15111832 | 09/06/2022 | Africa / Morocco / Rabat | Human | unknown | Female | 63 years | Live | unknown | unknown | BE.1 | GRA |
| hCoV-19/Morocco/941/2022 | EPI_ISL_15111828 | 30/05/2022 | Africa / Morocco / Essaouira | Human | unknown | Female | unknown | Live | unknown | unknown | BA.2.9.3 | GRA |
| hCoV-19/Morocco/914/2022 | EPI_ISL_15111813 | 06/06/2022 | Africa / Morocco / Rabat | Human | unknown | Male | 49 years | Live | unknown | unknown | BA.2 | GRA |
| hCoV-19/Morocco/957/2022 | EPI_ISL_15111834 | 24/05/2022 | Africa / Morocco / Marrakech | Human | unknown | Female | 53 years | Live | unknown | unknown | BA.2.9 | GRA |
| hCoV-19/Morocco/930/2022 | EPI_ISL_15111823 | 31/05/2022 | Africa / Morocco / Casablanca | Human | unknown | Male | 27 years | Live | unknown | unknown | BA.2 | GRA |
| hCoV-19/Morocco/962/2022 | EPI_ISL_15111835 | 07/06/2022 | Africa / Morocco / Rabat | Human | unknown | Male | unknown | Live | unknown | unknown | BF.5 | GRA |
| hCoV-19/Morocco/1017/2022 | EPI_ISL_15139474 | 30/06/2022 | Africa / Morocco / Rabat | Human | unknown | Male | 34 years | Live | unknown | unknown | BA.5.2.20 | GRA |
| hCoV-19/Morocco/1001/2022 | EPI_ISL_15139481 | 25/06/2022 | Africa / Morocco / Rabat | Human | unknown | Female | 30 years | Live | unknown | unknown | BA.5.2 | GRA |
| hCoV-19/Morocco/1011/2022 | EPI_ISL_15139472 | 29/06/2022 | Africa / Morocco / Rabat | Human | unknown | Female | 58 years | Live | unknown | unknown | BA.5.2 | GRA |
| hCoV-19/Morocco/996/2022 | EPI_ISL_15139469 | 06/06/2022 | Africa / Morocco / Casablanca | Human | unknown | Female | 13 years | Live | unknown | unknown | BA.5.2.1 | GRA |
| hCoV-19/Morocco/1000/2022 | EPI_ISL_15139470 | 10/06/2022 | Africa / Morocco / Casablanca | Human | unknown | Male | 51 years | Live | unknown | unknown | BA.2.12.1 | GRA |
| hCoV-19/Morocco/1012/2022 | EPI_ISL_15139484 | 29/06/2022 | Africa / Morocco / Kenitra | Human | unknown | Male | 40 years | Live | unknown | unknown | BA.5.2.20 | GRA |
| hCoV-19/Morocco/1005/2022 | EPI_ISL_15139471 | 30/06/2022 | Africa / Morocco / Kenitra | Human | unknown | Male | 57 years | Live | unknown | unknown | BA.5.2.20 | GRA |
| hCoV-19/Morocco/976/2022 | EPI_ISL_15139489 | 13/06/2022 | Africa / Morocco / Rabat | Human | unknown | Female | 40 years | Live | unknown | unknown | BA.2 | GRA |
| hCoV-19/Morocco/1013/2022 | EPI_ISL_15139473 | 28/06/2022 | Africa / Morocco / Sale | Human | unknown | Female | 21 years | Live | unknown | unknown | BA.5.2.20 | GRA |
| hCoV-19/Morocco/982/2022 | EPI_ISL_15139478 | 17/06/2022 | Africa / Morocco / Kenitra | Human | unknown | Male | 56 years | Live | unknown | unknown | BA.5.2.20 | GRA |
| hCoV-19/Morocco/1018/2022 | EPI_ISL_15139475 | 01/07/2022 | Africa / Morocco / Rabat | Human | unknown | Male | 09 years | Live | unknown | unknown | BA.2.40.1 | GRA |
| hCoV-19/Morocco/968/2022 | EPI_ISL_15139476 | 15/06/2022 | Africa / Morocco / Rabat | Human | unknown | Female | 51 years | Live | unknown | unknown | BA.5.2.20 | GRA |
| hCoV-19/Morocco/998/2022 | EPI_ISL_15139480 | 07/06/2022 | Africa / Morocco / Casablanca | Human | unknown | Male | 52 years | Live | unknown | unknown | BA.5.2.20 | GRA |
| hCoV-19/Morocco/991/2022 | EPI_ISL_15139479 | 17/06/2022 | Africa / Morocco / Marrakech | Human | unknown | Female | 29 years | Live | unknown | unknown | BA.2.9.3 | GRA |
| hCoV-19/Morocco/1015/2022 | EPI_ISL_15139485 | 30/06/2022 | Africa / Morocco / Rabat | Human | unknown | Female | 38 years | Live | unknown | unknown | BA.5.2.20 | GRA |
| hCoV-19/Morocco/1020/2022 | EPI_ISL_15139487 | 27/06/2022 | Africa / Morocco / Laayoun | Human | unknown | Male | 40 years | Live | unknown | unknown | BA.5.2.20 | GRA |
| hCoV-19/Morocco/1006/2022 | EPI_ISL_15139483 | 29/06/2022 | Africa / Morocco / Kenitra | Human | unknown | Female | 60 years | Live | unknown | unknown | BA.5.1 | GRA |
| hCoV-19/Morocco/1002/2022 | EPI_ISL_15139482 | 22/06/2022 | Africa / Morocco / Sale | Human | unknown | Female | 45 years | Live | unknown | unknown | BA.5.2.20 | GRA |
| hCoV-19/Morocco/977/2022 | EPI_ISL_15139490 | 13/06/2022 | Africa / Morocco / Rabat | Human | unknown | Male | 53 years | Live | unknown | unknown | BA.4 | GRA |
| hCoV-19/Morocco/974/2022 | EPI_ISL_15139492 | 17/06/2022 | Africa / Morocco / Rabat | Human | unknown | Female | 39 years | Live | unknown | unknown | BA.5.2.20 | GRA |
| hCoV-19/Morocco/1019/2022 | EPI_ISL_15139486 | 27/06/2022 | Africa / Morocco / Laayoun | Human | unknown | Female | 45 years | Live | unknown | unknown | BA.5.2.20 | GRA |
| hCoV-19/Morocco/1021/2022 | EPI_ISL_15139488 | 27/06/2022 | Africa / Morocco / Laayoun | Human | unknown | Male | 35 years | Live | unknown | unknown | BA.5.2.20 | GRA |
| hCoV-19/Morocco/1031/2022 | EPI_ISL_15139499 | 27/06/2022 | Africa / Morocco / Tetouan | Human | unknown | Female | unknow | Live | unknown | unknown | BA.5.2.20 | GRA |
| hCoV-19/Morocco/980/2022 | EPI_ISL_15139493 | 17/06/2022 | Africa / Morocco / Kenitra | Human | unknown | Female | 29 years | Live | unknown | unknown | BA.5.2.20 | GRA |
| hCoV-19/Morocco/1032/2022 | EPI_ISL_15139500 | 25/06/2022 | Africa / Morocco / Tetouan | Human | unknown | Male | unknow | Live | unknown | unknown | BA.2 | GRA |
| hCoV-19/Morocco/978/2022 | EPI_ISL_15139491 | 13/06/2022 | Africa / Morocco / Rabat | Human | unknown | Male | 28 years | Live | unknown | unknown | BA.5.2 | GRA |
| hCoV-19/Morocco/985/2022 | EPI_ISL_15139505 | 17/06/2022 | Africa / Morocco / Kenitra | Human | unknown | Female | 29 years | Live | unknown | unknown | BA.5.1 | GRA |
| hCoV-19/Morocco/1026/2022 | EPI_ISL_15139494 | 24/06/2022 | Africa / Morocco / Laayoun | Human | unknown | Male | 26 years | Live | unknown | unknown | BA.2.9.3 | GRA |
| hCoV-19/Morocco/983/2022 | EPI_ISL_15139504 | 17/06/2022 | Africa / Morocco / Sale | Human | unknown | Female | 46 years | Live | unknown | unknown | BA.5.2.20 | GRA |
| hCoV-19/Morocco/1027/2022 | EPI_ISL_15139495 | 28/06/2022 | Africa / Morocco / Tetouan | Human | unknown | Female | unknow | Live | unknown | unknown | BA.5.1.23 | GRA |
| hCoV-19/Morocco/989/2022 | EPI_ISL_15139503 | 17/06/2022 | Africa / Morocco / Sale | Human | unknown | Male | 34 years | Live | unknown | unknown | BA.5.2.20 | GRA |
| hCoV-19/Morocco/986/2022 | EPI_ISL_15139506 | 17/06/2022 | Africa / Morocco / Sale | Human | unknown | Male | 58 years | Live | unknown | unknown | BA.5.2.20 | GRA |
| hCoV-19/Morocco/967/2022 | EPI_ISL_15385200 | 15/06/2022 | Africa / Morocco / Rabat | Human | unknown | Female | 40 | Live | unknown | unknown | BA.2 | GRA |
| hCoV-19/Morocco/965/2022 | EPI_ISL_15139502 | 15/06/2022 | Africa / Morocco / Rabat | Human | unknown | Female | 58 years | Live | unknown | unknown | BA.2.40.1 | GRA |
| hCoV-19/Morocco/984/2022 | EPI_ISL_15385196 | 17/06/2022 | Africa / Morocco / Sale | Human | unknown | Male | 40 | Live | unknown | unknown | BA.5 | GRA |
| hCoV-19/Morocco/1039/2022 | EPI_ISL_15385501 | 02/07/2022 | Africa / Morocco / Tanger | Human | unknown | Female | 27 | Live | unknown | unknown | BA.5.2 | GRA |
| hCoV-19/Morocco/1042/2022 | EPI_ISL_15385506 | 05/07/2022 | Africa / Morocco / Tanger | Human | unknown | Male | 42 | Live | unknown | unknown | BA.4 | GRA |
| hCoV-19/Morocco/1041/2022 | EPI_ISL_15385508 | 05/07/2022 | Africa / Morocco / Tanger | Human | unknown | Female | 32 | Live | unknown | unknown | BA.5.1 | GRA |
| hCoV-19/Morocco/1057/2022 | EPI_ISL_15385497 | 20/07/2022 | Africa / Morocco / Kenitra | Human | unknown | Female | 28 | Live | unknown | unknown | BA.5.2.20 | GRA |
| hCoV-19/Morocco/1025/2022 | EPI_ISL_15385198 | 24/06/2022 | Africa / Morocco / Laayoun | Human | unknown | Female | 51 | Live | unknown | unknown | BA.2.9.3 | GRA |
| hCoV-19/Morocco/1043/2022 | EPI_ISL_15385495 | 05/07/2022 | Africa / Morocco / Tanger | Human | unknown | Female | 67 | Live | unknown | unknown | BA.5.2 | GRA |
| hCoV-19/Morocco/1054/2022 | EPI_ISL_15385499 | 20/07/2022 | Africa / Morocco / Kenitra | Human | unknown | Male | 58 | Live | unknown | unknown | BA.5.2.20 | GRA |
| hCoV-19/Morocco/1058/2022 | EPI_ISL_15385504 | 20/07/2022 | Africa / Morocco / Kenitra | Human | unknown | Male | 48 | Live | unknown | unknown | BA.5.1 | GRA |
| hCoV-19/Morocco/1044/2022 | EPI_ISL_15385502 | 05/07/2022 | Africa / Morocco / Tanger | Human | unknown | Male | 59 | Live | unknown | unknown | BA.4 | GRA |
| hCoV-19/Morocco/1037/2022 | EPI_ISL_15385494 | 01/07/2022 | Africa / Morocco / Tanger | Human | unknown | Female | 35 | Live | unknown | unknown | BA.5.2 | GRA |
| hCoV-19/Morocco/FMP-98/2021 | EPI_ISL_2116539 | 2021 | Africa / Morocco / Mohammedia | Human | Baseline surveillance | unknown | unknown | unknown | unknown | Baseline surveillance | B.1 | G |
| hCoV-19/Morocco/IPM20379238/2021 | EPI_ISL_6436654 | 22/10/2021 | Africa / Morocco / Mohammadia | Human | unknown | Male | 47 | unknown | unknown | unknown | AY.43 | GK |
| hCoV-19/Morocco/IPM20379442/2021 | EPI_ISL_6436659 | 23/10/2021 | Africa / Morocco / Casablanca | Human | unknown | Female | 38 | unknown | unknown | unknown | AY.33 | GK |
| hCoV-19/Morocco/IPM20379418/2021 | EPI_ISL_6436657 | 23/10/2021 | Africa / Morocco / Casablanca | Human | unknown | Female | 50 | unknown | unknown | unknown | AY.33 | GK |
| hCoV-19/Morocco/IPM20379276/2021 | EPI_ISL_6436655 | 22/10/2021 | Africa / Morocco / Casablanca | Human | unknown | Male | 22 | unknown | unknown | unknown | B.1.617.2 | GK |
| hCoV-19/Morocco/IPM20379042/2021 | EPI_ISL_6436651 | 22/10/2021 | Africa / Morocco / Casablanca | Human | unknown | Male | 61 | unknown | unknown | unknown | B.1.617.2 | GK |
| hCoV-19/Morocco/159/2021 | EPI_ISL_8144253 | 22/12/2021 | Africa / Morocco / Rabat | Human | unknown | Male | unknown | Live | unknown | unknown | BA.1 | GRA |
| hCoV-19/Morocco/FMP-208/2020 | EPI_ISL_5123575 | 02/11/2020 | Africa / Morocco / Harhoura | Human | unknown | Female | 60 | unknown | unknown | unknown | B.1.1 | GR |
| hCoV-19/Morocco/148/2021 | EPI_ISL_8144258 | 22/12/2021 | Africa / Morocco / Kenitra | Human | unknown | Male | unknown | Live | unknown | unknown | BA.1 | GRA |
| hCoV-19/Morocco/IPM20379230/2021 | EPI_ISL_6436653 | 22/10/2021 | Africa / Morocco / Mohammadia | Human | unknown | Female | 40 | unknown | unknown | unknown | AY.43 | GK |
| hCoV-19/Morocco/528/2022 | EPI_ISL_10260259 | 08/01/2022 | Africa / Morocco / Marrakech | Human | unknown | Female | 58 | Live | unknown | unknown | BA.1 | GRA |
| hCoV-19/Morocco/IPM20360110/2021 | EPI_ISL_5511705 | 06/09/2021 | Africa / Morocco / Casablanca | Human | unknown | Female | 32 | unknown | unknown | unknown | AY.33 | GK |
| hCoV-19/Morocco/IPM20361774/2021 | EPI_ISL_5511704 | 08/09/2021 | Africa / Morocco / Casablanca | Human | unknown | Female | 56 | unknown | unknown | unknown | B.1.617.2 | GK |
| hCoV-19/Morocco/IPM20361857/2021 | EPI_ISL_5511700 | 08/09/2021 | Africa / Morocco / Casablanca | Human | unknown | Female | 52 | unknown | unknown | unknown | B.1.617.2 | GK |
| hCoV-19/Morocco/IPM20360864/2021 | EPI_ISL_5501140 | 07/09/2021 | Africa / Morocco / Casablanca | Human | unknown | Female | 25 | unknown | unknown | unknown | AY.33 | GK |
| hCoV-19/Morocco/20353637/2021 | EPI_ISL_4430766 | 27/08/2021 | Africa / Morocco / Casablanca | Human | unknown | Female | 55 | unknown | unknown | unknown | AY.33 | GK |
| hCoV-19/Morocco/FMP-225/2021 | EPI_ISL_1913079 | 19/03/2021 | Africa / Morocco / Kenitra | Human | unknown | unknown | unknown | unknown | unknown | unknown | B.1.1.7 | G |
| hCoV-19/Morocco/GR96/2021 | EPI_ISL_9439417 | 29/12/2021 | Africa / Morocco | Human | Active surveillance | Male | unknown | Released | unknown | Active surveillance | BA.1.15 | GRA |
| hCoV-19/Morocco/FMP-282/2021 | EPI_ISL_5133529 | 07/04/2021 | Africa / Morocco | Human | unknown | unknown | unknown | unknown | unknown | unknown | B.1.1.7 | GRY |
| hCoV-19/Morocco/FMP-238/2021 | EPI_ISL_5123577 | 24/03/2021 | Africa / Morocco / Rabat | Human | unknown | Male | 7 months | unknown | unknown | unknown | B.1.575 | GH |
| hCoV-19/Morocco/FMP-206/2021 | EPI_ISL_5123551 | 24/03/2021 | Africa / Morocco / Khemisset | Human | unknown | Female | 70 | unknown | unknown | unknown | B.1 | G |
| hCoV-19/Morocco/FMP-205/2021 | EPI_ISL_5123547 | 24/03/2021 | Africa / Morocco / Rabat | Human | unknown | Male | 69 | unknown | unknown | unknown | B.1 | GH |
| hCoV-19/Morocco/FMP-199/2020 | EPI_ISL_5123542 | 22/12/2020 | Africa / Morocco / Harhoura | Human | unknown | Female | 60 | unknown | unknown | unknown | B.1.597 | GH |
| hCoV-19/Morocco/FMP-347/2021 | EPI_ISL_5122537 | 26/07/2021 | Africa / Morocco / Rabat | Human | unknown | Male | unknown | unknown | unknown | unknown | B.1.1.7 | GRY |
| hCoV-19/Morocco/FMP-341/2021 | EPI_ISL_5121443 | 05/06/2021 | Africa / Morocco / Inezgane | Human | unknown | Female | unknown | unknown | unknown | unknown | B.1.1.7 | GRY |
| hCoV-19/Morocco/FMP_337/2021 | EPI_ISL_5121065 | 13/07/2021 | Africa / Morocco / Agadir | Human | unknown | Male | unknown | unknown | unknown | unknown | AY.73 | GK |
| hCoV-19/Morocco/FMP-336/2021 | EPI_ISL_5119883 | 19/05/2021 | Africa / Morocco | Human | unknown | Male | 50 | unknown | unknown | unknown | B.1.1.7 | GRY |
| hCoV-19/Morocco/FMP-335/2021 | EPI_ISL_5118980 | 13/07/2021 | Africa / Morocco / Rabat | Human | unknown | Male | unknown | unknown | unknown | unknown | AY.112 | GK |
| hCoV-19/Morocco/FMP-333/2021 | EPI_ISL_5117973 | 22/07/2021 | Africa / Morocco / Rabat | Human | unknown | Male | unknown | unknown | unknown | unknown | B.1.1.7 | GRY |
| hCoV-19/Morocco/FMP-329/2021 | EPI_ISL_5116329 | 22/07/2021 | Africa / Morocco / Agadir | Human | unknown | Male | unknown | unknown | unknown | unknown | B.1.1.7 | GRY |
| hCoV-19/Morocco/FMP-320/2021 | EPI_ISL_5116328 | 2021-04 | Africa / Morocco / Harhoura | Human | unknown | Female | 35 | unknown | unknown | unknown | B.1.1.7 | GRY |
| hCoV-19/Morocco/FMP-319/2021 | EPI_ISL_5116227 | 2021-05 | Africa / Morocco / Rabat | Human | unknown | unknown | unknown | unknown | unknown | unknown | B.1.1.7 | GRY |
| hCoV-19/Morocco/FMP-315/2021 | EPI_ISL_5103520 | 2021-05 | Africa / Morocco / Kenitra | Human | unknown | unknown | unknown | unknown | unknown | unknown | B.1.1.7 | G |
| hCoV-19/Morocco/FMP-312/2021 | EPI_ISL_5102829 | 2021-05 | Africa / Morocco / Kenitra | Human | unknown | unknown | unknown | unknown | unknown | unknown | B.1.1.7 | GRY |
| hCoV-19/Morocco/FMP-311/2021 | EPI_ISL_5102141 | 2021-05 | Africa / Morocco / Kenitra | Human | unknown | unknown | unknown | unknown | unknown | unknown | B.1.1.7 | GRY |
| hCoV-19/Morocco/FMP-308/2021 | EPI_ISL_5100559 | 2021-05 | Africa / Morocco / Rabat | Human | unknown | unknown | unknown | unknown | unknown | unknown | B.1.1.7 | GRY |
| hCoV-19/Morocco/FMP-307/2021 | EPI_ISL_5100386 | 2021-05 | Africa / Morocco / Sale | Human | unknown | unknown | unkonwn | unknown | unknown | unknown | B.1.525 | G |
| hCoV-19/Morocco/FMP-306/2021 | EPI_ISL_5100047 | 2021-05 | Africa / Morocco / Kenitra | Human | unknown | unknown | unknown | unknown | unknown | unknown | B.1.1.7 | GRY |
| hCoV-19/Morocco/FMP-304/2021 | EPI_ISL_5096112 | 2021-05 | Africa / Morocco / Rabat | Human | unknown | unknown | unknown | unknown | unknown | unknown | B.1.1.7 | GRY |
| hCoV-19/Morocco/FMP-302/2021 | EPI_ISL_5096106 | 2021-05 | Africa / Morocco / Rabat | Human | unknown | unknown | unknown | unknown | unknown | unknown | B.1.1.7 | GRY |
| hCoV-19/Morocco/FMP-301/2021 | EPI_ISL_5096103 | 2021-05 | Africa / Morocco / Kenitra | Human | unknown | unknown | unknown | unknown | unknown | unknown | B.1.1.7 | GRY |
| hCoV-19/Morocco/FMP-300/2021 | EPI_ISL_5096096 | 2021-05 | Africa / Morocco / Kenitra | Human | unknown | unknown | unknown | unknown | unknown | unknown | B.1.1.7 | GRY |
| hCoV-19/Morocco/FMP-299/2021 | EPI_ISL_5096093 | 2021-05 | Africa / Morocco / Kenitra | Human | unknown | unknown | unknown | unknown | unknown | unknown | B.1.1.7 | GRY |
| hCoV-19/Morocco/FMP-297/2021 | EPI_ISL_5096085 | 2021-05 | Africa / Morocco / Sale | Human | unknown | unknown | unknown | unknown | unknown | unknown | B.1.1.7 | GRY |
| hCoV-19/Morocco/FMP-296/2021 | EPI_ISL_5096084 | 2021-05 | Africa / Morocco / Sale | Human | unknown | unknown | unknown | unknown | unknown | unknown | B.1.1.7 | GRY |
| hCoV-19/Morocco/FMP-295/2021 | EPI_ISL_5096080 | 2021-05 | Africa / Morocco / Sidi Slimane | Human | unknown | unknown | unknown | unknown | unknown | unknown | B.1.525 | G |
| hCoV-19/Morocco/FMP-294/2021 | EPI_ISL_5096075 | 2021-05 | Africa / Morocco / Sidi Slimane | Human | unknown | unknown | unknown | unknown | unknown | unknown | B.1.1.7 | GRY |
| hCoV-19/Morocco/FMP-293/2021 | EPI_ISL_5096071 | 2021-05 | Africa / Morocco / Sale | Human | unknown | unknown | unknown | unknown | unknown | unknown | B.1.525 | G |
| hCoV-19/Morocco/FMP-292/2021 | EPI_ISL_5096069 | 2021-05 | Africa / Morocco / Kenitra | Human | unknown | unknown | unknown | unknown | unknown | unknown | B.1.1.7 | GRY |
| hCoV-19/Morocco/FMP-322/2021 | EPI_ISL_5096060 | 12/07/2021 | Africa / Morocco / Ouarzazate | Human | unknown | Male | unknown | unknown | unknown | unknown | B.1.1.7 | GRY |
| hCoV-19/Morocco/FMP-290/2021 | EPI_ISL_5096055 | 2021-04 | Africa / Morocco / Temara | Human | unknown | Male | 64 | unknown | unknown | unknown | B.1.1.7 | GRY |
| hCoV-19/Morocco/FMP-321/2021 | EPI_ISL_5096053 | 10/07/2021 | Africa / Morocco / Inezgane | Human | unknown | Female | unknown | unknown | unknown | unknown | AY.33 | GK |
| hCoV-19/Morocco/FMP-289/2021 | EPI_ISL_5096051 | 2021-04 | Africa / Morocco / Temara | Human | unknown | Female | 54 | unknown | unknown | unknown | B.1.1.7 | GRY |
| hCoV-19/Morocco/IPM20405121/2022 | EPI_ISL_10070733 | 19/01/2022 | Africa / Morocco / Casablanca | Human | unknown | Female | 68 | unknown | unknown | unknown | BA.1.17.1 | GRA |
| hCoV-19/Morocco/IPM20405120/2022 | EPI_ISL_10070732 | 19/01/2022 | Africa / Morocco / Casablanca | Human | unknown | Male | 38 | unknown | unknown | unknown | BA.1 | GRA |
| hCoV-19/Morocco/IPM20405118/2022 | EPI_ISL_10070730 | 19/01/2022 | Africa / Morocco / Casablanca | Human | unknown | Male | 90 | unknown | unknown | unknown | BA.1 | GRA |
| hCoV-19/Morocco/IPM20404449/2022 | EPI_ISL_10070725 | 18/01/2022 | Africa / Morocco / Casablanca | Human | unknown | Female | 79 | unknown | unknown | unknown | BA.1 | GRA |
| hCoV-19/Morocco/IPM20402120/2022 | EPI_ISL_10070724 | 12/01/2022 | Africa / Morocco / Casablanca | Human | unknown | Male | 99 | unknown | unknown | unknown | BA.1 | GRA |
| hCoV-19/Morocco/IPM20402115/2022 | EPI_ISL_10070721 | 12/01/2022 | Africa / Morocco / Casablanca | Human | unknown | Male | 92 | unknown | unknown | unknown | BA.1 | GRA |
| hCoV-19/Morocco/IPM20401713/2022 | EPI_ISL_10070719 | 12/01/2022 | Africa / Morocco / Casablanca | Human | unknown | Male | 71 | unknown | unknown | unknown | BA.1 | GRA |
| hCoV-19/Morocco/IPM20401712/2022 | EPI_ISL_10070718 | 12/01/2022 | Africa / Morocco / Casablanca | Human | unknown | Male | 50 | unknown | unknown | unknown | BA.1 | GRA |
| hCoV-19/Morocco/IPM20401710/2022 | EPI_ISL_10070717 | 12/01/2022 | Africa / Morocco / Casablanca | Human | unknown | Female | 81 | unknown | unknown | unknown | AY.72 | GK |
| hCoV-19/Morocco/IPM20400730/2022 | EPI_ISL_10070713 | 10/01/2022 | Africa / Morocco / Casablanca | Human | unknown | Female | 33 | unknown | unknown | unknown | B.1.617.2 | GK |
| hCoV-19/Morocco/IPM20400728/2022 | EPI_ISL_10070711 | 10/01/2022 | Africa / Morocco / Casablanca | Human | unknown | Female | 27 | unknown | unknown | unknown | BA.1 | GRA |
| hCoV-19/Morocco/IPM20400727/2022 | EPI_ISL_10070710 | 10/01/2022 | Africa / Morocco / Casablanca | Human | unknown | Female | 64 | unknown | unknown | unknown | BA.1 | GRA |
| hCoV-19/Morocco/FMP-2/2021 | EPI_ISL_5061885 | 02/01/2021 | Africa / Morocco / Sidi Lahcen | Human | unknown | Male | 40 | unknown | unknown | unknown | B.1 | GH |
| hCoV-19/Morocco/RA207/2021 | EPI_ISL_3253426 | 20/06/2021 | Africa / Morocco | Human | unknown | Male | unknown | Released | unknown | unknown | AY.33 | GK |
| hCoV-19/Morocco/RA112-3C/2021 | EPI_ISL_3244531 | 06/07/2021 | Africa / Morocco | Human | Active surveillance | Female | unknown | Released | unknown | Active surveillance | B | L |
| hCoV-19/Morocco/IPM2095960/2020 | EPI_ISL_17650173 | 06/07/2020 | Africa / Morocco / Casablanca | Human | unknown | Male | 5 | unknown | unknown | unknown | B.1 | G |
| hCoV-19/Morocco/IPM20149828/2020 | EPI_ISL_17650191 | 16/08/2020 | Africa / Morocco / Casablanca | Human | unknown | Male | unknown | unknown | unknown | unknown | B.1 | G |
| hCoV-19/Morocco/IPM20131054/2020 | EPI_ISL_17650181 | 26/07/2020 | Africa / Morocco / Casablanca | Human | unknown | Female | unknown | unknown | unknown | unknown | B.1 | G |
| hCoV-19/Morocco/IPM20100159/2020 | EPI_ISL_17650174 | 08/07/2020 | Africa / Morocco / Casablanca | Human | unknown | Male | 63 | unknown | unknown | unknown | B.1 | G |
| hCoV-19/Morocco/IPM2032357/2020 | EPI_ISL_17650159 | 25/05/2020 | Africa / Morocco / Casablanca | Human | unknown | Female | unknown | unknown | unknown | unknown | B.1 | G |
| hCoV-19/Morocco/IPM2027930/2020 | EPI_ISL_17650158 | 19/05/2020 | Africa / Morocco / Casablanca | Human | unknown | Female | 26 | unknown | unknown | unknown | B.1 | G |
| hCoV-19/Morocco/IPM20138732/2020 | EPI_ISL_17650184 | 04/08/2020 | Africa / Morocco / Casablanca | Human | unknown | Female | unknown | unknown | unknown | unknown | B.1 | GH |
| hCoV-19/Morocco/IPM2007000/2020 | EPI_ISL_17650146 | 18/04/2020 | Africa / Morocco / Casablanca | Human | unknown | Female | unknown | unknown | unknown | unknown | B.1.1 | GR |
| hCoV-19/Morocco/IPM20101882/2020 | EPI_ISL_17650175 | 09/07/2020 | Africa / Morocco / Casablanca | Human | unknown | Male | unknown | unknown | unknown | unknown | B.1.1 | GR |
| hCoV-19/Morocco/IPM2077348/2020 | EPI_ISL_17650170 | 26/06/2020 | Africa / Morocco / Casablanca | Human | unknown | Female | unknown | unknown | unknown | unknown | B.1 | G |
| hCoV-19/Morocco/IPM2005210/2020 | EPI_ISL_17650141 | 14/04/2020 | Africa / Morocco / Casablanca | Human | unknown | Female | 31 | unknown | unknown | unknown | B.1.1 | GR |
| hCoV-19/Morocco/IPM2000693/2020 | EPI_ISL_17650133 | 26/03/2020 | Africa / Morocco / Casablanca | Human | unknown | Female | 38 | unknown | unknown | unknown | B.1.1 | GR |
| hCoV-19/Morocco/IPM2000802/2020 | EPI_ISL_17650134 | 27/03/2020 | Africa / Morocco / Casablanca | Human | unknown | Female | 72 | unknown | unknown | unknown | B.1.1 | GR |
| hCoV-19/Morocco/IPM2002730/2020 | EPI_ISL_17650135 | 04/04/2020 | Africa / Morocco / Casablanca | Human | unknown | Female | 23 | unknown | unknown | unknown | B.1.1 | GR |
| hCoV-19/Morocco/IPM20146063/2020 | EPI_ISL_17650187 | 11/08/2020 | Africa / Morocco / Casablanca | Human | unknown | Male | unknown | unknown | unknown | unknown | B.1 | G |
| hCoV-19/Morocco/Sett44/2020 | EPI_ISL_3204262 | 03/05/2020 | Africa / Morocco | Human | Outbreak investigation | Female | 23 | unknown | unknown | Outbreak investigation | B.1 | G |
| hCoV-19/Morocco/211/2021 | EPI_ISL_10019531 | 26/12/2021 | Africa / Morocco / Guelmim | Human | unknown | Male | 56 | Live | 03/03/2021 | unknown | BA.1 | GRA |
| hCoV-19/Morocco/255/2021 | EPI_ISL_10019526 | 28/12/2021 | Africa / Morocco / Tetouan | Human | unknown | Male | 65 | Live | unknown | unknown | AY.33 | GK |
| hCoV-19/Morocco/369/2022 | EPI_ISL_10019537 | 02/01/2022 | Africa / Morocco / Casablanca | Human | unknown | Female | 22 | Live | 03/03/2021 | unknown | BA.1 | GRA |
| hCoV-19/Morocco/251/2021 | EPI_ISL_10019536 | 29/12/2021 | Africa / Morocco / Sale | Human | unknown | Male | unknown | Live | unknown | unknown | BA.1 | GRA |
| hCoV-19/Morocco/205/2021 | EPI_ISL_10019524 | 25/12/2021 | Africa / Morocco / Rabat | Human | unknown | Female | unknown | Live | unknown | unknown | AY.43 | GK |
| hCoV-19/Morocco/261/2021 | EPI_ISL_10019525 | 28/12/2021 | Africa / Morocco / Sale | Human | unknown | Male | 47 | Live | unknown | unknown | AY.5 | GK |
| hCoV-19/Morocco/258/2021 | EPI_ISL_10019527 | 28/12/2021 | Africa / Morocco / Casablanca | Human | unknown | Male | 39 | Live | unknown | unknown | AY.126 | GK |
| hCoV-19/Morocco/411/2022 | EPI_ISL_10019528 | 03/01/2022 | Africa / Morocco / Rabat | Human | unknown | Female | 68 | Live - Intensive Care Unit | unknown | unknown | AY.112 | GK |
| hCoV-19/Morocco/256/2021 | EPI_ISL_10019542 | 28/12/2021 | Africa / Morocco / Tetouan | Human | unknown | Female | 56 | Live | unknown | unknown | BA.1.18 | GRA |
| hCoV-19/Morocco/202/2021 | EPI_ISL_10019532 | 27/12/2021 | Africa / Morocco / Sale | Human | unknown | Female | unknown | Live | unknown | unknown | BA.1 | GRA |
| hCoV-19/Morocco/248/2021 | EPI_ISL_10019533 | 28/12/2021 | Africa / Morocco / Rabat | Human | unknown | Male | unknown | Live | unknown | unknown | BA.1 | GRA |
| hCoV-19/Morocco/410/2022 | EPI_ISL_10019538 | 03/01/2022 | Africa / Morocco / Sale | Human | unknown | Male | 59 | Live - Intensive Care Unit | unknown | unknown | BA.1 | GRA |
| hCoV-19/Morocco/250/2022 | EPI_ISL_10019540 | 04/01/2022 | Africa / Morocco / Casablanca | Human | unknown | Female | unknown | Live | unknown | unknown | BA.1 | GRA |
| hCoV-19/Morocco/409/2021 | EPI_ISL_10019541 | 07/12/2021 | Africa / Morocco / Rabat | Human | unknown | Female | unknown | Live - Intensive Care Unit | unknown | unknown | BA.1 | GRA |
| hCoV-19/Morocco/FMP-352/2021 | EPI_ISL_4945861 | 22/07/2021 | Africa / Morocco / Tetouan | Human | unknown | Female | unknown | unknown | unknown | unknown | AY.33 | GK |
| hCoV-19/Morocco/FMP-346/2021 | EPI_ISL_4945675 | 13/07/2021 | Africa / Morocco / Agadir | Human | unknown | Female | unknown | unknown | unknown | unknown | AY.112 | GK |
| hCoV-19/Morocco/INH-109/2020 | EPI_ISL_4899911 | 02/02/2020 | Africa / Morocco / Rabat | Human | unknown | Male | 62 | Released | unknown | unknown | B.1.177 | GV |
| hCoV-19/Morocco/INH-106/2020 | EPI_ISL_4899892 | 02/02/2020 | Africa / Morocco / Rabat | Human | unknown | Male | 46 | Released | unknown | unknown | B.1.160 | GH |
| hCoV-19/Morocco/INH-103/2020 | EPI_ISL_4899870 | 02/02/2020 | Africa / Morocco / Rabat | Human | unknown | Female | 63 | Released | unknown | unknown | B.1 | G |
| hCoV-19/Morocco/INH-101/2020 | EPI_ISL_4899863 | 02/02/2020 | Africa / Morocco / Rabat | Human | unknown | Male | 60 | Released | unknown | unknown | B.1.221 | G |
| hCoV-19/Morocco/ION_CODE_4/2021 | EPI_ISL_3155339 | 17/06/2021 | Africa / Morocco / Casablanca | Human | unknown | Male | 23 | unknown | unknown | unknown | AY.51 | GK |
| hCoV-19/Morocco/20356934/2021 | EPI_ISL_4798130 | 01/09/2021 | Africa / Morocco / Casablanca | Human | unknown | Female | 33 | unknown | unknown | unknown | AY.33 | GK |
| hCoV-19/Morocco/FMP-13/2020 | EPI_ISL_728297 | 2020-11 | Africa / Morocco / Rabat | Human | unknown | unknown | unknown | unknown | unknown | unknown | B.1 | G |
| hCoV-19/Morocco/INH-4811/2021 | EPI_ISL_18210270 | 05/06/2021 | Africa / Morocco / Sale | Human | unknown | Female | 68 years | Live | unknown | unknown | B.1.619 | G |
| hCoV-19/Morocco/INH-4765/2021 | EPI_ISL_18210273 | 21/05/2021 | Africa / Morocco / Casablanca | Human | unknown | Male | 38 years | Live | unknown | unknown | C.17 | GR |
| hCoV-19/Morocco/20358921/2021 | EPI_ISL_4741173 | 03/09/2021 | Africa / Morocco / Casablanca | Human | unknown | Male | 50 | unknown | unknown | unknown | AY.33 | GK |
| hCoV-19/Morocco/20356767/2021 | EPI_ISL_4741164 | 01/09/2021 | Africa / Morocco / Casablanca | Human | unknown | Female | 72 | unknown | unknown | unknown | AY.33 | GK |
| hCoV-19/Morocco/20356384/2021 | EPI_ISL_4741163 | 31/08/2021 | Africa / Morocco / Casablanca | Human | unknown | Female | 24 | unknown | unknown | unknown | AY.33 | GK |
| hCoV-19/Morocco/20356070/2021 | EPI_ISL_4741160 | 31/08/2021 | Africa / Morocco / Casablanca | Human | unknown | Male | 33 | unknown | unknown | unknown | AY.33 | GK |
| hCoV-19/Morocco/20355868/2021 | EPI_ISL_4741158 | 31/08/2021 | Africa / Morocco / Casablanca | Human | unknown | Female | 15 | unknown | unknown | unknown | AY.33 | GK |
| hCoV-19/Morocco/20355845/2021 | EPI_ISL_4741157 | 31/08/2021 | Africa / Morocco / Casablanca | Human | unknown | Female | 66 | unknown | unknown | unknown | AY.112 | GK |
| hCoV-19/Morocco/20355301/2021 | EPI_ISL_4741154 | 30/08/2021 | Africa / Morocco / Casablanca | Human | unknown | Female | 54 | unknown | unknown | unknown | AY.37 | GK |
| hCoV-19/Morocco/INH-4809/2021 | EPI_ISL_18210269 | 05/06/2021 | Africa / Morocco / Sale | Human | unknown | Female | 70 years | Live | unknown | unknown | B.1.619 | G |
| hCoV-19/Morocco/INH-4388/2021 | EPI_ISL_18210262 | 15/05/2021 | Africa / Morocco / Kenitra | Human | unknown | Female | 18 years | Live | unknown | unknown | B.1.525 | G |
| hCoV-19/Morocco/INH-4801/2021 | EPI_ISL_18210267 | 05/06/2021 | Africa / Morocco / Rabat | Human | unknown | Female | 57 years | Live | unknown | unknown | B.1.619 | G |
| hCoV-19/Morocco/INH-4691/2021 | EPI_ISL_18210263 | 25/05/2021 | Africa / Morocco / Rabat | Human | unknown | Male | 72 years | Live | unknown | unknown | B.1.619 | G |
| hCoV-19/Morocco/INH-4826/2021 | EPI_ISL_18210274 | 02/06/2021 | Africa / Morocco / Rabat | Human | unknown | Male | 62 years | Live | unknown | unknown | B.1.619 | G |
| hCoV-19/Morocco/INH-4779/2021 | EPI_ISL_18210271 | 23/05/2021 | Africa / Morocco / Fes | Human | unknown | Male | 47 years | Live | unknown | unknown | B.1.619 | G |
| hCoV-19/Morocco/INH-4756/2021 | EPI_ISL_18210272 | 20/05/2021 | Africa / Morocco / Tanger | Human | unknown | Female | 43 years | Live | unknown | unknown | B.1.617.2 | GK |
| hCoV-19/Morocco/INH-4800/2021 | EPI_ISL_18210266 | 05/06/2021 | Africa / Morocco / Rabat | Human | unknown | Male | 62 years | Live | unknown | unknown | B.1.619 | G |
| hCoV-19/Morocco/INH-4701/2021 | EPI_ISL_18210264 | 25/05/2021 | Africa / Morocco / Casablanca | Human | unknown | Female | 33 years | Live | unknown | unknown | B.1.1.318 | GR |
| hCoV-19/Morocco/INH-4841/2021 | EPI_ISL_18210275 | 02/06/2021 | Africa / Morocco / Rabat | Human | unknown | Female | 50 years | Live | unknown | unknown | B.1.351 | GH |
| hCoV-19/Morocco/INH-4798/2021 | EPI_ISL_18210265 | 24/05/2021 | Africa / Morocco / Rabat | Human | unknown | Male | 66 years | Live | unknown | unknown | B.1.619 | G |
| hCoV-19/Morocco/INH-4802/2021 | EPI_ISL_18210268 | 05/06/2021 | Africa / Morocco / Sale | Human | unknown | Female | 45 years | Live | unknown | unknown | B.1.619 | G |
| hCoV-19/Morocco/INH-4904/2021 | EPI_ISL_18210276 | 02/06/2021 | Africa / Morocco / Casablanca | Human | unknown | Male | 43 years | Live | unknown | unknown | B.1.351 | GH |
| hCoV-19/Morocco/CP457-INH/2020 | EPI_ISL_18210314 | 15/03/2020 | Africa / Morocco / Rabat | Human | unknown | Femele | 36 years | Live | unknown | unknown | B.1 | GH |
| hCoV-19/Morocco/CP6946-INH/2020 | EPI_ISL_18210316 | 13/05/2020 | Africa / Morocco / Tanger | Human | unknown | Femele | 37 years | Live | unknown | unknown | B.1 | G |
| hCoV-19/Morocco/CP19147-INH/2020 | EPI_ISL_18210328 | 05/09/2020 | Africa / Morocco / Sale | Human | unknown | Femele | 34 years | Live | unknown | unknown | B.1 | G |
| hCoV-19/Morocco/CP12312-INH/2020 | EPI_ISL_18210342 | 17/11/2020 | Africa / Morocco / Larache | Human | unknown | Femele | 33 years | Live | unknown | unknown | B.1 | G |
| hCoV-19/Morocco/CP12378-INH/2020 | EPI_ISL_18210346 | 19/11/2020 | Africa / Morocco / Ouarzazate | Human | unknown | Male | 25 years | Live | unknown | unknown | B.1.528 | G |
| hCoV-19/Morocco/CP10855-INH/2020 | EPI_ISL_18210339 | 14/10/2020 | Africa / Morocco / Ouarzazate | Human | unknown | Femele | 50 years | Live | unknown | unknown | B.1 | G |
| hCoV-19/Morocco/CP10888-INH/2020 | EPI_ISL_18210340 | 14/10/2020 | Africa / Morocco / Ouarzazate | Human | unknown | Femele | 48 years | Live | unknown | unknown | B.1 | G |
| hCoV-19/Morocco/CP12335-INH/2020 | EPI_ISL_18210343 | 18/11/2020 | Africa / Morocco / Ouarzazate | Human | unknown | Male | 36 years | Live | unknown | unknown | B.1.528 | G |
| hCoV-19/Morocco/CP12340-INH/2020 | EPI_ISL_18210344 | 18/11/2020 | Africa / Morocco / Ouarzazate | Human | unknown | Male | 44 years | Live | unknown | unknown | B.1.528 | G |
| hCoV-19/Morocco/CP10941-INH/2020 | EPI_ISL_18210341 | 16/10/2020 | Africa / Morocco / Larache | Human | unknown | Femele | 32 years | Live | unknown | unknown | B.1 | G |
| hCoV-19/Morocco/255789-INH/2020 | EPI_ISL_18210347 | 29/05/2020 | Africa / Morocco / Rabat | Human | unknown | Male | 18 years | Live | unknown | unknown | B.1.36 | GH |
| hCoV-19/Morocco/INH-3298/2021 | EPI_ISL_18210348 | 10/05/2021 | Africa / Morocco / Casablanca | Human | unknown | Femele | 40 years | Live | unknown | unknown | B.1.1.7 | GRY |
| hCoV-19/Morocco/CP12355-INH/2020 | EPI_ISL_18210345 | 18/11/2020 | Africa / Morocco / Ouarzazate | Human | unknown | Male | 32 years | Live | unknown | unknown | B.1.528 | G |
| hCoV-19/Morocco/INH-3905/2021 | EPI_ISL_18210349 | 16/05/2021 | Africa / Morocco / Casablanca | Human | unknown | Male | 56 years | Live | unknown | unknown | B.1.1.7 | GRY |
| hCoV-19/Morocco/INH-4546/2021 | EPI_ISL_18210350 | 19/05/2021 | Africa / Morocco / Kenitra | Human | unknown | Male | 50 years | Live | unknown | unknown | B.1.617.2 | GK |
| hCoV-19/Morocco/INH-4547/2021 | EPI_ISL_18210351 | 20/05/2021 | Africa / Morocco / Rabat | Human | unknown | Femele | 34 years | Live | unknown | unknown | B.1.617.2 | GK |
| hCoV-19/Morocco/INH-4948/2021 | EPI_ISL_18210354 | 28/05/2021 | Africa / Morocco / Kenitra | Human | unknown | Male | 35 years | Live | unknown | unknown | B.1.1.7 | GRY |
| hCoV-19/Morocco/INH-4953/2021 | EPI_ISL_18210355 | 28/05/2021 | Africa / Morocco / Kenitra | Human | unknown | Femele | 32 years | Live | unknown | unknown | B.1.619 | G |
| hCoV-19/Morocco/INH-4549/2021 | EPI_ISL_18210352 | 21/05/2021 | Africa / Morocco / Rabat | Human | unknown | Male | 25 years | Live | unknown | unknown | B.1.617.2 | GK |
| hCoV-19/Morocco/INH-4553/2021 | EPI_ISL_18210353 | 24/05/2021 | Africa / Morocco / Sale | Human | unknown | Femele | 18 years | Live | unknown | unknown | B.1.1.7 | GRY |
| hCoV-19/Morocco/INH-1106/2021 | EPI_ISL_18210358 | 25/02/2021 | Africa / Morocco / Casablanca | Human | unknown | Male | 24 years | Live | unknown | unknown | B.1.1.7 | GRY |
| hCoV-19/Morocco/CP407-INH/2020 | EPI_ISL_18210315 | 12/03/2020 | Africa / Morocco / Rabat | Human | unknown | Femele | 26 years | Live | unknown | unknown | B.1.1 | GR |
| hCoV-19/Morocco/CP15015-INH/2020 | EPI_ISL_18210323 | 11/08/2020 | Africa / Morocco / Sale | Human | unknown | Male | 36 years | Live | unknown | unknown | B.1 | G |
| hCoV-19/Morocco/INH-1233/2021 | EPI_ISL_18210359 | 26/02/2021 | Africa / Morocco / Rabat | Human | unknown | Male | 45 years | Live | unknown | unknown | B.1.1.7 | GRY |
| hCoV-19/Morocco/INH-1082/2021 | EPI_ISL_18210356 | 22/02/2021 | Africa / Morocco / Casablanca | Human | unknown | Femele | 39 years | Live | unknown | unknown | B.1.1.7 | GRY |
| hCoV-19/Morocco/INH-1087/2021 | EPI_ISL_18210357 | 17/02/2021 | Africa / Morocco / Casablanca | Human | unknown | Femele | 35 years | Live | unknown | unknown | B.1.1.7 | GRY |
| hCoV-19/Morocco/CP411-INH/2020 | EPI_ISL_18210318 | 16/03/2020 | Africa / Morocco / Sale | Human | unknown | Male | 47 years | Live | unknown | unknown | A.5 | S |
| hCoV-19/Morocco/INH-1238/2021 | EPI_ISL_18210360 | 25/02/2021 | Africa / Morocco / Rabat | Human | unknown | Male | 37 years | Live | unknown | unknown | B.1.1.7 | GRY |
| hCoV-19/Morocco/CP14567-INH/2020 | EPI_ISL_18210331 | 05/08/2020 | Africa / Morocco / Tanger | Human | unknown | Male | 42 years | Live | unknown | unknown | B.1 | G |
| hCoV-19/Morocco/CP3858-INH/2020 | EPI_ISL_18210319 | 17/04/2020 | Africa / Morocco / Berkane | Human | unknown | Femele | 55 years | Live | unknown | unknown | B.1 | GH |
| hCoV-19/Morocco/CP19143-INH/2020 | EPI_ISL_18210326 | 05/09/2020 | Africa / Morocco / Sale | Human | unknown | Male | 37 years | Live | unknown | unknown | B.1 | G |
| hCoV-19/Morocco/CP425-INH/2020 | EPI_ISL_18210317 | 15/03/2020 | Africa / Morocco / Sale | Human | unknown | Male | 23 years | Live | unknown | unknown | B.1.1 | GR |
| hCoV-19/Morocco/INH-1590/2021 | EPI_ISL_18210361 | 02/03/2021 | Africa / Morocco / Rabat | Human | unknown | Male | 36 years | Live | unknown | unknown | B.1.1.7 | GRY |
| hCoV-19/Morocco/CP388-INH/2020 | EPI_ISL_18210321 | 20/03/2020 | Africa / Morocco / Rabat | Human | unknown | Femele | 67 years | Live | unknown | unknown | B.1.356 | GH |
| hCoV-19/Morocco/CP14473-INH/2020 | EPI_ISL_18210322 | 10/08/2020 | Africa / Morocco / Tetouan | Human | unknown | Femele | 65 years | Live | unknown | unknown | B.1 | G |
| hCoV-19/Morocco/CP2370-INH/2020 | EPI_ISL_18210320 | 18/04/2020 | Africa / Morocco / Rabat | Human | unknown | Male | 43 years | Live | unknown | unknown | B.1 | G |
| hCoV-19/Morocco/CP14942-INH/2020 | EPI_ISL_18210330 | 11/08/2020 | Africa / Morocco / Fes | Human | unknown | Male | 29 years | Live | unknown | unknown | B.1 | G |
| hCoV-19/Morocco/CP17545-INH/2020 | EPI_ISL_18210325 | 20/08/2020 | Africa / Morocco / Rabat | Human | unknown | Femele | 41 years | Live | unknown | unknown | B.1 | G |
| hCoV-19/Morocco/CP14529-INH/2020 | EPI_ISL_18210334 | 05/08/2020 | Africa / Morocco / Tanger | Human | unknown | Male | 38 years | Live | unknown | unknown | B.1 | G |
| hCoV-19/Morocco/CP14927-INH/2020 | EPI_ISL_18210324 | 11/08/2020 | Africa / Morocco / Fes | Human | unknown | Femele | 35 years | Live | unknown | unknown | B.1 | G |
| hCoV-19/Morocco/CP19214-INH/2020 | EPI_ISL_18210329 | 06/09/2020 | Africa / Morocco / Sale | Human | unknown | Femele | 32 years | Live | unknown | unknown | B.1 | G |
| hCoV-19/Morocco/CP21348-INH/2020 | EPI_ISL_18210327 | 20/09/2020 | Africa / Morocco / Sale | Human | unknown | Male | 50 years | Live | unknown | unknown | B.1 | G |
| hCoV-19/Morocco/CP14537-INH/2020 | EPI_ISL_18210332 | 05/08/2020 | Africa / Morocco / Larache | Human | unknown | Male | 32 years | Live | unknown | unknown | B.1 | G |
| hCoV-19/Morocco/CP14535-INH/2020 | EPI_ISL_18210333 | 05/08/2020 | Africa / Morocco / Tanger | Human | unknown | Femele | 27 years | Live | unknown | unknown | B.1 | G |
| hCoV-19/Morocco/CP3728-INH/2020 | EPI_ISL_18210336 | 10/06/2020 | Africa / Morocco / Tanger | Human | unknown | Femele | 68 years | Live | unknown | unknown | B.1.1 | GR |
| hCoV-19/Morocco/CP6434-INH/2020 | EPI_ISL_18210337 | 28/06/2020 | Africa / Morocco / Tanger | Human | unknown | Femele | 36 years | Live | unknown | unknown | B.1 | G |
| hCoV-19/Morocco/CP21463-INH/2020 | EPI_ISL_18210335 | 03/08/2020 | Africa / Morocco / Larache | Human | unknown | Femele | 35 years | Live | unknown | unknown | B.1 | G |
| hCoV-19/Morocco/CP6994-INH/2020 | EPI_ISL_18210338 | 30/06/2020 | Africa / Morocco / Tanger | Human | unknown | Femele | 56 years | Live | unknown | unknown | B.1 | G |
| hCoV-19/Morocco/FMP-112/2021 | EPI_ISL_2127072 | 2021 | Africa / Morocco / Sidi Kacem | Human | Baseline surveillance | unknown | unknown | unknown | unknown | Baseline surveillance | B.1.177.77 | GV |
| hCoV-19/Morocco/FMP-105/2021 | EPI_ISL_2127528 | 2021 | Africa / Morocco / Casablanca | Human | Baseline surveillance | unknown | unknown | unknown | unknown | Baseline surveillance | B.1.1 | GR |
| hCoV-19/Morocco/FMP-109/2021 | EPI_ISL_2125511 | 2021 | Africa / Morocco / Casablanca | Human | Baseline surveillance | unknown | unknown | unknown | unknown | Baseline surveillance | B.1.177 | GV |
| hCoV-19/Morocco/FMP-110/2021 | EPI_ISL_2126028 | 2021 | Africa / Morocco / Beni Mellal | Human | Baseline surveillance | unknown | unknown | unknown | unknown | Baseline surveillance | B.1.36 | GH |
| hCoV-19/Morocco/FMP-108/2021 | EPI_ISL_2125207 | 2021 | Africa / Morocco / Casablanca | Human | Baseline surveillance | unknown | unknown | unknown | unknown | Baseline surveillance | B.1 | G |
| hCoV-19/Morocco/FMP-332/2021 | EPI_ISL_4572442 | 13/07/2021 | Africa / Morocco / Awrir | Human | unknown | Male | unknown | unknown | unknown | unknown | AY.112 | GK |
| hCoV-19/Morocco/FMP-330/2021 | EPI_ISL_4572416 | 12/07/2021 | Africa / Morocco / Zagora | Human | unknown | Female | unknown | unknown | unknown | unknown | AY.73 | GK |
| hCoV-19/Morocco/FMP-327/2021 | EPI_ISL_4572272 | 12/07/2021 | Africa / Morocco / Rabat | Human | unknown | Male | unknown | unknown | unknown | unknown | AY.51 | GK |
| hCoV-19/Morocco/FMP-323/2021 | EPI_ISL_4572233 | 12/07/2021 | Africa / Morocco / Ouarzazate | Human | unknown | Male | unknown | unknown | unknown | unknown | B.1.617.2 | GK |
| hCoV-19/Morocco/FMP-106/2021 | EPI_ISL_2124861 | 2021 | Africa / Morocco / Casablanca | Human | Baseline surveillance | unknown | unknown | unknown | unknown | Baseline surveillance | B.1 | G |
| hCoV-19/Morocco/526/2022 | EPI_ISL_10260251 | 11/01/2022 | Africa / Morocco / Marrakech | Human | unknown | Male | unknown | Live | unknown | unknown | BA.1 | GRA |
| hCoV-19/Morocco/553/2022 | EPI_ISL_10260258 | 10/01/2022 | Africa / Morocco / Beni Mellal | Human | unknown | Male | unknown | Live | unknown | unknown | BA.1 | GRA |
| hCoV-19/Morocco/530/2022 | EPI_ISL_10260261 | 08/01/2022 | Africa / Morocco / Marrakech | Human | unknown | Male | 60 | Live | unknown | unknown | AY.112 | GK |
| hCoV-19/Morocco/533/2022 | EPI_ISL_10260253 | 13/01/2022 | Africa / Morocco / Beni Mellal | Human | unknown | Male | unknown | Live | unknown | unknown | BA.1 | GRA |
| hCoV-19/Morocco/527/2022 | EPI_ISL_10260252 | 10/01/2022 | Africa / Morocco / Marrakech | Human | unknown | Female | 55 | Live | unknown | unknown | AY.4 | GK |
| hCoV-19/Morocco/532/2022 | EPI_ISL_10260264 | 04/01/2022 | Africa / Morocco / Marrakech | Human | unknown | Male | unknown | Live | unknown | unknown | BA.1 | GRA |
| hCoV-19/Morocco/554/2022 | EPI_ISL_10260265 | 14/01/2022 | Africa / Morocco / Beni Mellal | Human | unknown | Male | unknown | Live | unknown | unknown | BA.1 | GRA |
| hCoV-19/Morocco/540/2022 | EPI_ISL_10260256 | 13/01/2022 | Africa / Morocco / Rabat | Human | unknown | Male | 60 | Live | unknown | unknown | BA.1 | GRA |
| hCoV-19/Morocco/529/2022 | EPI_ISL_10260260 | 08/01/2022 | Africa / Morocco / Marrakech | Human | unknown | Female | unknown | Live | unknown | unknown | BA.1 | GRA |
| hCoV-19/Morocco/535/2022 | EPI_ISL_10260262 | 08/01/2022 | Africa / Morocco / Fes | Human | unknown | Male | unknown | Live | unknown | unknown | BA.1 | GRA |
| hCoV-19/Morocco/544/2022 | EPI_ISL_10260263 | 14/01/2022 | Africa / Morocco / Rabat | Human | unknown | Male | unknown | Live | unknown | unknown | BA.1 | GRA |
| hCoV-19/Morocco/20352205/2021 | EPI_ISL_4511388 | 25/08/2021 | Africa / Morocco / Casablanca | Human | unknown | Female | 21 | unknown | unknown | unknown | AY.34.1 | GK |
| hCoV-19/Morocco/20350834/2021 | EPI_ISL_4511386 | 24/08/2021 | Africa / Morocco / Casablanca | Human | unknown | Male | 59 | unknown | unknown | unknown | AY.34 | GK |
| hCoV-19/Morocco/ION-CODE-101/2021 | EPI_ISL_3071138 | 08/07/2021 | Africa / Morocco / Casablanca | Human | unknown | Male | 29 | Released | unknown | unknown | B.1.1.7 | GRY |
| hCoV-19/Morocco/FMP-104/2021 | EPI_ISL_2122010 | 2021 | Africa / Morocco / Casablanca | Human | Baseline surveillance | unknown | unknown | unknown | unknown | Baseline surveillance | B.1.1 | GR |
| hCoV-19/Morocco/FMP-103/2021 | EPI_ISL_2121485 | 2021 | Africa / Morocco / Casablanca | Human | Baseline surveillance | unknown | unknown | unknown | unknown | Baseline surveillance | B.1 | G |
| hCoV-19/Morocco/20353650/2021 | EPI_ISL_4430769 | 27/08/2021 | Africa / Morocco / Casablanca | Human | unknown | Female | 57 | unknown | unknown | unknown | AY.33 | GK |
| hCoV-19/Morocco/20353540/2021 | EPI_ISL_4430764 | 27/08/2021 | Africa / Morocco / Casablanca | Human | unknown | Female | 33 | unknown | unknown | unknown | AY.33 | GK |
| hCoV-19/Morocco/20352914/2021 | EPI_ISL_4430761 | 26/08/2021 | Africa / Morocco / Casablanca | Human | unknown | Male | 38 | unknown | unknown | unknown | AY.33 | GK |
| hCoV-19/Morocco/20352644/2021 | EPI_ISL_4430751 | 26/08/2021 | Africa / Morocco / Casablanca | Human | unknown | Male | 13 | unknown | unknown | unknown | AY.33 | GK |
| hCoV-19/Morocco/20352164/2021 | EPI_ISL_4430747 | 25/08/2021 | Africa / Morocco / Casablanca | Human | unknown | Female | 46 | unknown | unknown | unknown | AY.122 | GK |
| hCoV-19/Morocco/20352030/2021 | EPI_ISL_4430741 | 25/08/2021 | Africa / Morocco / Casablanca | Human | unknown | Male | 38 | unknown | unknown | unknown | AY.33 | GK |
| hCoV-19/Morocco/20351081/2021 | EPI_ISL_4430739 | 24/08/2021 | Africa / Morocco / Casablanca | Human | unknown | Female | 52 | unknown | unknown | unknown | AY.33 | GK |
| hCoV-19/Morocco/20350021/2021 | EPI_ISL_4430718 | 23/08/2021 | Africa / Morocco / Casablanca | Human | unknown | Male | 47 | unknown | unknown | unknown | AY.33 | GK |
| hCoV-19/Morocco/FMP-101/2021 | EPI_ISL_2119583 | 2021 | Africa / Morocco / Casablanca | Human | Baseline surveillance | unknown | unknown | unknown | unknown | Baseline surveillance | B.1.1 | GR |
| hCoV-19/Morocco/FMP-100/2021 | EPI_ISL_2118134 | 2021 | Africa / Morocco / Casablanca | Human | Baseline surveillance | unknown | unknown | unknown | unknown | Baseline surveillance | B.1.160 | GH |
| hCoV-19/Morocco/FMP-97/2021 | EPI_ISL_2117168 | 2021 | Africa / Morocco / Mohammedia | Human | Baseline surveillance | unknown | unknown | unknown | unknown | Baseline surveillance | B.1.1.447 | GR |
| hCoV-19/Morocco/1178-NIH/2022 | EPI_ISL_15856357 | 07/11/2022 | Africa / Morocco / Rabat | Human | unknown | Male | 45 years | Live | unknown | unknown | BN.1.2 | GRA |
| hCoV-19/Morocco/FMP-107/2021 | EPI_ISL_2124573 | 2021 | Africa / Morocco / Casablanca | Human | Baseline surveillance | unknown | unknown | unknown | unknown | Baseline surveillance | B.1.1 | GR |
| hCoV-19/Morocco/RA2C/2021 | EPI_ISL_4309589 | 04/07/2021 | Africa / Morocco | Human | unknown | unknown | unknown | unknown | unknown | unknown | B.1.621 | GH |
| hCoV-19/Morocco/RA16/2021 | EPI_ISL_4306000 | 20/06/2021 | Africa / Morocco | Human | unknown | unknown | unknown | Released | unknown | unknown | B.1 | G |
| hCoV-19/Morocco/FMP-278/2021 | EPI_ISL_2111158 | 09/04/2021 | Africa / Morocco / Casablanca | Human | Baseline surveillance | unknown | unknown | unknown | unknown | Baseline surveillance | B.1 | G |
| hCoV-19/Morocco/nCoV20326324/2021 | EPI_ISL_4300683 | 12/07/2021 | Africa / Morocco / Casablanca | Human | unknown | Female | 21 | unknown | unknown | unknown | AY.112 | GK |
| hCoV-19/Morocco/nCoV20326292/2021 | EPI_ISL_4300681 | 12/07/2021 | Africa / Morocco / Casablanca | Human | unknown | Male | 37 | unknown | unknown | unknown | B.1.1.7 | GRY |
| hCoV-19/Morocco/CNRST-IND01/2021 | EPI_ISL_2110643 | 22/04/2021 | Africa / Morocco / Casablanca | Human | Same-patient sampling strategy | Male | 47 | Released | unknown | Same-patient sampling strategy | B.1.617.2 | GK |
| hCoV-19/Morocco/TANG183/2020 | EPI_ISL_4299861 | 27/04/2020 | Africa / Morocco | Human | unknown | Female | 42 | unknown | unknown | unknown | B.1 | G |
| hCoV-19/Morocco/HMIMV-279CCC/2020 | EPI_ISL_2968068 | 07/12/2020 | Africa / Morocco / Rabat | Human | unknown | Male | 55 | Released | unknown | unknown | B.1 | GH |
| hCoV-19/Morocco/HMIMV-FATP1/2020 | EPI_ISL_2968066 | 07/12/2020 | Africa / Morocco / Rabat | Human | unknown | Female | 60 | Released | unknown | unknown | B.1.22 | GH |
| hCoV-19/Morocco/HMIMV-IKRP1/2020 | EPI_ISL_2968065 | 07/12/2020 | Africa / Morocco / Rabat | Human | unknown | Male | 51 | Released | unknown | unknown | B.1 | G |
| hCoV-19/Morocco/HMIMV-00037/2020 | EPI_ISL_2968061 | 07/12/2020 | Africa / Morocco / Rabat | Human | unknown | Male | 65 | Released | unknown | unknown | B.1.1 | GR |
| hCoV-19/Morocco/HMIMV-00079/2020 | EPI_ISL_2968060 | 07/12/2020 | Africa / Morocco / Rabat | Human | unknown | Male | 44 | Released | unknown | unknown | B.1 | GH |
| hCoV-19/Morocco/HMIMV-00242/2020 | EPI_ISL_2968056 | 07/12/2020 | Africa / Morocco / Rabat | Human | unknown | Female | 38 | Released | unknown | unknown | B.1 | G |
| hCoV-19/Morocco/HMIMV-00249/2020 | EPI_ISL_2968054 | 07/12/2020 | Africa / Morocco / Rabat | Human | unknown | Male | 55 | Released | unknown | unknown | B.1 | G |
| hCoV-19/Morocco/HMIMV-16N/2020 | EPI_ISL_2968052 | 18/07/2020 | Africa / Morocco / Rabat | Human | unknown | Male | 76 | Released | unknown | unknown | B.1 | G |
| hCoV-19/Morocco/HMIMV-19N/2020 | EPI_ISL_2968051 | 18/07/2020 | Africa / Morocco / Rabat | Human | unknown | Female | 33 | Released | unknown | unknown | B.1 | G |
| hCoV-19/Morocco/HMIMV-20N/2020 | EPI_ISL_2968050 | 18/07/2020 | Africa / Morocco / Rabat | Human | unknown | Female | 45 | Released | unknown | unknown | B.1 | G |
| hCoV-19/Morocco/HMIMV-5N/2020 | EPI_ISL_2968049 | 18/07/2020 | Africa / Morocco / Rabat | Human | unknown | Male | 76 | Released | unknown | unknown | B.1 | G |
| hCoV-19/Morocco/HMIMV-113N/2020 | EPI_ISL_2968048 | 18/07/2020 | Africa / Morocco / Rabat | Human | unknown | Female | 66 | Released | unknown | unknown | B.1 | G |
| hCoV-19/Morocco/HMIMV-3N/2020 | EPI_ISL_2968046 | 18/07/2020 | Africa / Morocco / Rabat | Human | unknown | Male | 68 | Released | unknown | unknown | B.1 | GH |
| hCoV-19/Morocco/HMIMV-106E/2020 | EPI_ISL_2968045 | 18/07/2020 | Africa / Morocco / Rabat | Human | unknown | Male | 64 | Released | unknown | unknown | B.1 | G |
| hCoV-19/Morocco/HMIMV-16E/2020 | EPI_ISL_2968042 | 18/07/2020 | Africa / Morocco / Rabat | Human | unknown | Male | 46 | Released | unknown | unknown | B.1 | G |
| hCoV-19/Morocco/HMIMV-121300/2020 | EPI_ISL_2968041 | 18/07/2020 | Africa / Morocco / Rabat | Human | unknown | Female | 53 | Released | unknown | unknown | B.1 | G |
| hCoV-19/Morocco/HMIMV-62E/2020 | EPI_ISL_2968040 | 18/07/2020 | Africa / Morocco / Rabat | Human | unknown | Female | 66 | Released | unknown | unknown | B.1 | G |
| hCoV-19/Morocco/HMIMV-39E/2020 | EPI_ISL_2968039 | 18/07/2020 | Africa / Morocco / Rabat | Human | unknown | Female | 45 | Released | unknown | unknown | B.1 | G |
| hCoV-19/Morocco/HMIMV-15EN/2020 | EPI_ISL_2968037 | 18/07/2020 | Africa / Morocco / Rabat | Human | unknown | Male | 55 | Released | unknown | unknown | B | G |
| hCoV-19/Morocco/HMIMV-21EN/2020 | EPI_ISL_2968036 | 18/07/2020 | Africa / Morocco / Rabat | Human | unknown | Male | 65 | Released | unknown | unknown | B.1 | G |
| hCoV-19/Morocco/HMIMV-585E/2020 | EPI_ISL_2968035 | 18/07/2020 | Africa / Morocco / Rabat | Human | unknown | Male | 60 | Released | unknown | unknown | B | G |
| hCoV-19/Morocco/CNRST-IND02/2021 | EPI_ISL_2966236 | 22/04/2021 | Africa / Morocco / Casablanca | Human | unknown | Male | 50 | Released | unknown | unknown | B.1.617.1 | GK |
| hCoV-19/Morocco/49/2021 | EPI_ISL_8308343 | 21/12/2021 | Africa / Morocco / Sale | Human | unknown | Male | unknown | Live | unknown | unknown | AY.122 | GK |
| hCoV-19/Morocco/587/2022 | EPI_ISL_10752412 | 29/01/2022 | Africa / Morocco / Beni Mellal | Human | unknown | Female | unknown | Hospitalized | unknown | unknown | BA.1 | GRA |
| hCoV-19/Morocco/570/2022 | EPI_ISL_10752409 | 22/01/2022 | Africa / Morocco / Beni Mellal | Human | unknown | Female | unknown | Hospitalized | unknown | unknown | BA.1 | GRA |
| hCoV-19/Morocco/567/2022 | EPI_ISL_10752416 | 18/01/2022 | Africa / Morocco / Kenitra | Human | unknown | Female | unknown | Live | unknown | unknown | B.1.1.529 | GRA |
| hCoV-19/Morocco/602/2022 | EPI_ISL_10752421 | 13/01/2022 | Africa / Morocco / Rabat | Human | unknown | Male | 29 | Hospitalized | unknown | unknown | BA.1 | GRA |
| hCoV-19/Morocco/575/2022 | EPI_ISL_10752424 | 18/01/2022 | Africa / Morocco / Rabat | Human | unknown | Male | 58 | Hospitalized | unknown | unknown | BA.1 | GRA |
| hCoV-19/Morocco/582/2022 | EPI_ISL_10752410 | 29/01/2022 | Africa / Morocco / Beni Mellal | Human | unknown | Female | 51 | Hospitalized | unknown | unknown | BA.1 | GRA |
| hCoV-19/Morocco/586/2022 | EPI_ISL_10752411 | 29/01/2022 | Africa / Morocco / Beni Mellal | Human | unknown | Male | 78 | Hospitalized | unknown | unknown | BA.1 | GRA |
| hCoV-19/Morocco/592/2022 | EPI_ISL_10752413 | 16/01/2022 | Africa / Morocco / Rabat | Human | unknown | Male | unknown | Hospitalized | unknown | unknown | BA.1 | GRA |
| hCoV-19/Morocco/597/2022 | EPI_ISL_10752414 | 16/01/2022 | Africa / Morocco / Rabat | Human | unknown | Male | 12 | Hospitalized | unknown | unknown | BA.1 | GRA |
| hCoV-19/Morocco/569/2022 | EPI_ISL_10752420 | 22/01/2022 | Africa / Morocco / Beni Mellal | Human | unknown | Female | unknown | Hospitalized | unknown | unknown | BA.1 | GRA |
| hCoV-19/Morocco/605/2022 | EPI_ISL_10752415 | 13/01/2022 | Africa / Morocco / Temara | Human | unknown | Female | 42 | Hospitalized | unknown | unknown | BA.1 | GRA |
| hCoV-19/Morocco/600/2022 | EPI_ISL_10752417 | 15/01/2022 | Africa / Morocco / Khemisset | Human | unknown | Female | 47 | Hospitalized | unknown | unknown | BA.1 | GRA |
| hCoV-19/Morocco/571/2022 | EPI_ISL_10752418 | 22/01/2022 | Africa / Morocco / Sale | Human | unknown | Female | unknown | Hospitalized | unknown | unknown | BA.1 | GRA |
| hCoV-19/Morocco/593/2022 | EPI_ISL_10752419 | 12/01/2022 | Africa / Morocco / Rabat | Human | unknown | Male | 60 | Hospitalized | unknown | unknown | BA.1 | GRA |
| hCoV-19/Morocco/584/2022 | EPI_ISL_10752422 | 29/01/2022 | Africa / Morocco / Beni Mellal | Human | unknown | Male | 70 | Hospitalized | unknown | unknown | BA.1 | GRA |
| hCoV-19/Morocco/FMP-54/2020 | EPI_ISL_775252 | 2020-11 | Africa / Morocco / Harhoura | Human | unknown | Male | 85 | unknown | unknown | unknown | B.1.1 | GR |
| hCoV-19/Morocco/IPM20361246/2021 | EPI_ISL_5501095 | 07/09/2021 | Africa / Morocco / Casablanca | Human | unknown | Female | 43 | unknown | unknown | unknown | AY.33 | GK |
| hCoV-19/Morocco/IPM20362391/2021 | EPI_ISL_5511706 | 09/09/2021 | Africa / Morocco / Casablanca | Human | unknown | Female | 61 | unknown | unknown | unknown | B.1.617.2 | GK |
| hCoV-19/Morocco/IPM20361716/2021 | EPI_ISL_5511703 | 08/09/2021 | Africa / Morocco / Casablanca | Human | unknown | Female | 32 | unknown | unknown | unknown | AY.33 | GK |
| hCoV-19/Morocco/IPM20361076/2021 | EPI_ISL_5511702 | 07/09/2021 | Africa / Morocco / Mohammadia | Human | unknown | Female | 38 | unknown | unknown | unknown | AY.73 | GK |
| hCoV-19/Morocco/IPM20360784/2021 | EPI_ISL_5511701 | 07/09/2021 | Africa / Morocco / Casablanca | Human | unknown | Male | 27 | unknown | unknown | unknown | B.1.617.2 | GK |
| hCoV-19/Morocco/IPM20379384/2021 | EPI_ISL_6436656 | 23/10/2021 | Africa / Morocco / Casablanca | Human | unknown | Male | 51 | unknown | unknown | unknown | AY.33 | GK |
| hCoV-19/Morocco/IPM20379227/2021 | EPI_ISL_6436652 | 22/10/2021 | Africa / Morocco / Mohammadia | Human | unknown | Male | 73 | unknown | unknown | unknown | AY.122 | GK |
| hCoV-19/Morocco/IPM20378880/2021 | EPI_ISL_6436650 | 21/10/2021 | Africa / Morocco / Casablanca | Human | unknown | Male | 45 | unknown | unknown | unknown | AY.112 | GK |
| hCoV-19/Morocco/IPM20379439/2021 | EPI_ISL_6436658 | 23/10/2021 | Africa / Morocco / Casablanca | Human | unknown | Female | 12 | unknown | unknown | unknown | AY.33 | GK |
| hCoV-19/Morocco/IPM20362474/2021 | EPI_ISL_5511699 | 09/09/2021 | Africa / Morocco / Mohammadia | Human | unknown | Female | 60 | unknown | unknown | unknown | AY.43 | GK |
| hCoV-19/Morocco/IPM20378061/2021 | EPI_ISL_6332500 | 18/10/2021 | Africa / Morocco / Casablanca | Human | unknown | Female | 41 | unknown | unknown | unknown | AY.112 | GK |
| hCoV-19/Morocco/IPM20378282/2021 | EPI_ISL_6332507 | 18/10/2021 | Africa / Morocco / Mohammadia | Human | unknown | Male | 57 | unknown | unknown | unknown | AY.33 | GK |
| hCoV-19/Morocco/IPM20378490/2021 | EPI_ISL_6332511 | 21/10/2021 | Africa / Morocco / Casablanca | Human | unknown | Male | 52 | unknown | unknown | unknown | AY.122 | GK |
| hCoV-19/Morocco/IPM20378191/2021 | EPI_ISL_6332506 | 18/10/2021 | Africa / Morocco / Casablanca | Human | unknown | Female | 28 | unknown | unknown | unknown | AY.33 | GK |
| hCoV-19/Morocco/IPM20378300/2021 | EPI_ISL_6332508 | 18/10/2021 | Africa / Morocco / Mohammadia | Human | unknown | Male | 38 | unknown | unknown | unknown | AY.33 | GK |
| hCoV-19/Morocco/IPM20378401/2021 | EPI_ISL_6332510 | 20/10/2021 | Africa / Morocco / Casablanca | Human | unknown | Female | 30 | unknown | unknown | unknown | AY.126 | GK |
| hCoV-19/Morocco/IPM20377246/2021 | EPI_ISL_5924608 | 14/10/2021 | Africa / Morocco / Casablanca | Human | unknown | Female | 54 | unknown | unknown | unknown | B.1.617.2 | GK |
| hCoV-19/Morocco/IPM20376705/2021 | EPI_ISL_5924600 | 12/10/2021 | Africa / Morocco / Casablanca | Human | unknown | Male | 62 | unknown | unknown | unknown | B.1.617.2 | GK |
| hCoV-19/Morocco/IPM20377001/2021 | EPI_ISL_5924604 | 13/10/2021 | Africa / Morocco / Casablanca | Human | unknown | Male | 53 | unknown | unknown | unknown | B.1.617.2 | GK |
| hCoV-19/Morocco/IPM20377475/2021 | EPI_ISL_5924611 | 14/10/2021 | Africa / Morocco / Casablanca | Human | unknown | Male | 66 | unknown | unknown | unknown | B.1.617.2 | GK |
| hCoV-19/Morocco/IPM20377546/2021 | EPI_ISL_5924612 | 14/10/2021 | Africa / Morocco / Mohammadia | Human | unknown | Female | 52 | unknown | unknown | unknown | B.1.617.2 | GK |
| hCoV-19/Morocco/IPM20377396/2021 | EPI_ISL_5924610 | 14/10/2021 | Africa / Morocco / Casablanca | Human | unknown | Male | 48 | unknown | unknown | unknown | B.1.617.2 | GK |
| hCoV-19/Morocco/IPM20377563/2021 | EPI_ISL_5924613 | 14/10/2021 | Africa / Morocco / Mohammadia | Human | unknown | Female | 76 | unknown | unknown | unknown | B.1.617.2 | GK |
| hCoV-19/Morocco/IPM20377665/2021 | EPI_ISL_5924614 | 15/10/2021 | Africa / Morocco / Casablanca | Human | unknown | Female | 47 | unknown | unknown | unknown | B.1.617.2 | GK |
| hCoV-19/Morocco/IPM20376349/2021 | EPI_ISL_5924594 | 11/10/2021 | Africa / Morocco / Casablanca | Human | unknown | Male | 12 | unknown | unknown | unknown | B.1.617.2 | GK |
| hCoV-19/Morocco/IPM20376416/2021 | EPI_ISL_5924595 | 11/10/2021 | Africa / Morocco / Casablanca | Human | unknown | Female | 49 | unknown | unknown | unknown | B.1.617.2 | GK |
| hCoV-19/Morocco/IPM20376214/2021 | EPI_ISL_5924592 | 11/10/2021 | Africa / Morocco / Casablanca | Human | unknown | Female | 20 | unknown | unknown | unknown | B.1.617.2 | GK |
| hCoV-19/Morocco/IPM20376297/2021 | EPI_ISL_5924593 | 11/10/2021 | Africa / Morocco / Casablanca | Human | unknown | Female | 64 | unknown | unknown | unknown | B.1.617.2 | GK |
| hCoV-19/Morocco/IPM20376656/2021 | EPI_ISL_5924599 | 12/10/2021 | Africa / Morocco / Casablanca | Human | unknown | Male | 55 | unknown | unknown | unknown | B.1.617.2 | GK |
| hCoV-19/Morocco/IPM20377691/2021 | EPI_ISL_6107654 | 15/10/2021 | Africa / Morocco / Mohammadia | Human | unknown | Female | 48 | unknown | unknown | unknown | AY.122 | GK |
| hCoV-19/Morocco/IPM20386830/2021 | EPI_ISL_9417724 | 15/12/2021 | Africa / Morocco / Casablanca | Human | unknown | Female | 25 | unknown | unknown | unknown | B.1.617.2 | GK |
| hCoV-19/Morocco/IPM20386353/2021 | EPI_ISL_9417720 | 11/12/2021 | Africa / Morocco / Casablanca | Human | unknown | Female | 73 | unknown | unknown | unknown | B.1.617.2 | GK |
| hCoV-19/Morocco/IPM20360522/2021 | EPI_ISL_5501132 | 07/09/2021 | Africa / Morocco / Mohammadia | Human | unknown | Male | 59 | unknown | unknown | unknown | AY.51 | GK |
| hCoV-19/Morocco/IPM20361572/2021 | EPI_ISL_5501117 | 08/09/2021 | Africa / Morocco / Mohammadia | Human | unknown | Male | 32 | unknown | unknown | unknown | AY.112 | GK |
| hCoV-19/Morocco/IPM20361487/2021 | EPI_ISL_5501110 | 08/09/2021 | Africa / Morocco / Casablanca | Human | unknown | Male | 73 | unknown | unknown | unknown | AY.33 | GK |
| hCoV-19/Morocco/IPM20383855/2021 | EPI_ISL_8186748 | 17/11/2021 | Africa / Morocco / Casablanca | Human | unknown | Female | 22 | unknown | unknown | unknown | B.1.617.2 | GK |
| hCoV-19/Morocco/IPM20386307/2021 | EPI_ISL_8186743 | 10/12/2021 | Africa / Morocco / Casablanca | Human | unknown | Female | 54 | unknown | unknown | unknown | AY.33 | GK |
| hCoV-19/Morocco/IPM20383968/2021 | EPI_ISL_8186750 | 19/11/2021 | Africa / Morocco / Casablanca | Human | unknown | Female | 68 | unknown | unknown | unknown | AY.33 | GK |
| hCoV-19/Morocco/IPM20382745/2021 | EPI_ISL_8186751 | 10/11/2021 | Africa / Morocco / Casablanca | Human | unknown | Male | 24 | unknown | unknown | unknown | AY.33 | GK |
| hCoV-19/Morocco/IPM20383429/2021 | EPI_ISL_8186752 | 15/11/2021 | Africa / Morocco / Casablanca | Human | unknown | Female | 26 | unknown | unknown | unknown | AY.33 | GK |
| hCoV-19/Morocco/IPM20383993/2021 | EPI_ISL_8186757 | 19/11/2021 | Africa / Morocco / Casablanca | Human | unknown | Female | 40 | unknown | unknown | unknown | AY.33 | GK |
| hCoV-19/Morocco/IPM20382933/2021 | EPI_ISL_8186759 | 11/11/2021 | Africa / Morocco / Casablanca | Human | unknown | Female | 47 | unknown | unknown | unknown | AY.4 | GK |
| hCoV-19/Morocco/IPM20361976/2021 | EPI_ISL_5501154 | 08/09/2021 | Africa / Morocco / Casablanca | Human | unknown | Male | 50 | unknown | unknown | unknown | B.1.617.2 | GK |
| hCoV-19/Morocco/IPM20386310/2021 | EPI_ISL_8421377 | 10/12/2021 | Africa / Morocco / Casablanca | Human | unknown | Female | 31 | unknown | unknown | unknown | B.1.1.529 | GRA |
| hCoV-19/Morocco/IPM20386910/2021 | EPI_ISL_8523914 | 15/12/2021 | Africa / Morocco / Casablanca | Human | unknown | Male | 36 | unknown | unknown | unknown | B.1.1.529 | GRA |
| hCoV-19/Morocco/IPM20386919/2021 | EPI_ISL_8523916 | 15/12/2021 | Africa / Morocco / Casablanca | Human | unknown | Female | 30 | unknown | unknown | unknown | B.1.1.529 | GRA |
| hCoV-19/Morocco/IPM20386978/2021 | EPI_ISL_8523917 | 15/12/2021 | Africa / Morocco / Casablanca | Human | unknown | Male | 12 | unknown | unknown | unknown | BA.1 | GRA |
| hCoV-19/Morocco/IPM20386345/2021 | EPI_ISL_8523908 | 11/12/2021 | Africa / Morocco / Casablanca | Human | unknown | Female | 52 | unknown | unknown | unknown | B.1.1.529 | GRA |
| hCoV-19/Morocco/IPM20386343/2021 | EPI_ISL_8523906 | 11/12/2021 | Africa / Morocco / Casablanca | Human | unknown | Female | 61 | unknown | unknown | unknown | B.1.1.529 | GRA |
| hCoV-19/Morocco/IPM20386344/2021 | EPI_ISL_8523907 | 11/12/2021 | Africa / Morocco / Casablanca | Human | unknown | Male | 1 | unknown | unknown | unknown | B.1.1.529 | GRA |
| hCoV-19/Morocco/IPM20386346/2021 | EPI_ISL_8523909 | 11/12/2021 | Africa / Morocco / Casablanca | Human | unknown | Female | 4 | unknown | unknown | unknown | B.1.1.529 | GRA |
| hCoV-19/Morocco/IPM20386355/2021 | EPI_ISL_8523910 | 11/12/2021 | Africa / Morocco / Casablanca | Human | unknown | Female | 13 | unknown | unknown | unknown | B.1.1.529 | GRA |
| hCoV-19/Morocco/IPM20386504/2021 | EPI_ISL_8523912 | 13/12/2021 | Africa / Morocco / Casablanca | Human | unknown | Male | 85 | unknown | unknown | unknown | B.1.1.529 | GRA |
| hCoV-19/Morocco/IPM20386486/2021 | EPI_ISL_8523911 | 13/12/2021 | Africa / Morocco / Casablanca | Human | unknown | Female | 41 | unknown | unknown | unknown | B.1.1.529 | GRA |
| hCoV-19/Morocco/LCAM-mohammedia_ELF-R_12/2020 | EPI_ISL_2835004 | 07/12/2020 | Africa / Morocco / Mohammedia | Human | unknown | Male | 25 | Live | unknown | unknown | B.1.1 | GR |
| hCoV-19/Morocco/LCAM-mohammedia_HAW-A_12/2020 | EPI_ISL_2835003 | 07/12/2020 | Africa / Morocco / Mohammedia | Human | unknown | Female | 35 | Live | unknown | unknown | B.1.1 | GR |
| hCoV-19/Morocco/LCAM-mohammedia_ELF-A-12/2020 | EPI_ISL_2835002 | 07/12/2020 | Africa / Morocco / Mohammedia | Human | unknown | Male | 20 | Live | unknown | unknown | B.1 | G |
| hCoV-19/Morocco/ouar384/2020 | EPI_ISL_978543 | 05/05/2020 | Africa / Morocco / Ouarzazate | Human | unknown | Male | 51 | unknown | unknown | unknown | B.1.528 | G |
| hCoV-19/Morocco/FMP-331/2021 | EPI_ISL_5116787 | 13/07/2021 | Africa / Morocco / Agadir | Human | unknown | Female | unknown | unknown | unknown | unknown | B.1.1.7 | GRY |
| hCoV-19/Morocco/FMP-394/2021 | EPI_ISL_8568497 | 2021 | Africa / Morocco / Fes | Human | unknown | unknown | unknown | unknown | unknown | unknown | BA.1.1 | GRA |
| hCoV-19/Morocco/FMP-395/2021 | EPI_ISL_8568498 | 2021 | Africa / Morocco / Fes | Human | unknown | unknown | unknown | unknown | unknown | unknown | BA.1.1 | GRA |
| hCoV-19/Morocco/INH-SEQ-3299/2021 | EPI_ISL_2695785 | 07/04/2021 | Africa / Morocco / Inzegane | Human | Active surveillance | Male | 45 | Live | unknown | Active surveillance | B.1.1.7 | GRY |
| hCoV-19/Morocco/CNRST_CHU15/2021 | EPI_ISL_8629701 | 24/10/2021 | Africa / Morocco / Rabat | Human | unknown | Male | 56 | released | unknown | unknown | AY.33 | GK |
| hCoV-19/Morocco/CNRST_CHU09/2021 | EPI_ISL_8629697 | 19/10/2021 | Africa / Morocco / Rabat | Human | unknown | Female | 77 | released | unknown | unknown | AY.33 | GK |
| hCoV-19/Morocco/FMP-184/2021 | EPI_ISL_2318899 | 07/01/2021 | Africa / Morocco / Rabat | Human | unknown | Female | 74 | unknown | unknown | unknown | B.1.597 | GH |
| hCoV-19/Morocco/FMP-183/2020 | EPI_ISL_2318037 | 08/08/2020 | Africa / Morocco / Sidi Lahcen | Human | unknown | Male | 59 | unknown | unknown | unknown | B.1 | G |
| hCoV-19/Morocco/FMP-182/2021 | EPI_ISL_2316770 | 04/01/2021 | Africa / Morocco / Sidi Lahcen | Human | unknown | Female | 35 | unknown | unknown | unknown | B.1 | GH |
| hCoV-19/Morocco/FMP-111/2021 | EPI_ISL_2126645 | 2021 | Africa / Morocco / Kenitra | Human | Baseline surveillance | unknown | unknown | unknown | unknown | Baseline surveillance | B.1.177.73 | GV |
| hCoV-19/Morocco/FMP-128/2021 | EPI_ISL_2313116 | 2021 | Africa / Morocco / Kenitra | Human | unknown | unknown | unknown | unknown | unknown | unknown | B.1.160 | GH |
| hCoV-19/Morocco/FMP-127/2021 | EPI_ISL_2313113 | 2021 | Africa / Morocco / Kenitra | Human | unknown | unknown | unknown | unknown | unknown | unknown | B.1.221 | G |
| hCoV-19/Morocco/FMP-126/2021 | EPI_ISL_2313085 | 2021 | Africa / Morocco / Kenitra | Human | unknown | unknown | unknown | unknown | unknown | unknown | B.1.177.73 | GV |
| hCoV-19/Morocco/FMP-125/2021 | EPI_ISL_2313083 | 2021 | Africa / Morocco / Kenitra | Human | unknown | unknown | unknown | unknown | unknown | unknown | B.1 | G |
| hCoV-19/Morocco/FMP-121/2021 | EPI_ISL_2313077 | 2021 | Africa / Morocco / Casablanca | Human | unknown | unknown | unknown | unknown | unknown | unknown | B.1.1.7 | GRY |
| hCoV-19/Morocco/FMP-119/2021 | EPI_ISL_2313075 | 2021 | Africa / Morocco / Beni Mellal | Human | unknown | unknown | unknown | unknown | unknown | unknown | B.1.221 | G |
| hCoV-19/Morocco/FMP-117/2021 | EPI_ISL_2313072 | 2021 | Africa / Morocco / Tinghir | Human | unknown | unknown | unknown | unknown | unknown | unknown | B.1.160 | GH |
| hCoV-19/Morocco/FMP-116/2021 | EPI_ISL_2313071 | 2021 | Africa / Morocco / Tinghir | Human | unknown | unknown | unknown | unknown | unknown | unknown | B.1.1 | GR |
| hCoV-19/Morocco/FMP-115/2021 | EPI_ISL_2313070 | 2021 | Africa / Morocco / Tinghir | Human | unknown | unknown | unknown | unknown | unknown | unknown | B.1.177 | GV |
| hCoV-19/Morocco/FMP-248/2021 | EPI_ISL_1914289 | 25/02/2021 | Africa / Morocco / Casablanca | Human | unknown | unknown | unknown | unknown | unknown | unknown | B.1.1.7 | GRY |
| hCoV-19/Morocco/FMP-246/2021 | EPI_ISL_1914284 | 14/03/2021 | Africa / Morocco / Casablanca | Human | unknown | unknown | unknown | unknown | unknown | unknown | B.1.1.7 | GRY |
| hCoV-19/Morocco/FMP-245/2021 | EPI_ISL_1914276 | 2021 | Africa / Morocco / Tinghir | Human | unknown | unknown | unknown | unknown | unknown | unknown | B.1.1.7 | GRY |
| hCoV-19/Morocco/FMP-244/2021 | EPI_ISL_1914274 | 2021 | Africa / Morocco / Tinghir | Human | unknown | unknown | unknown | unknown | unknown | unknown | B.1.1.7 | GRY |
| hCoV-19/Morocco/FMP-243/2021 | EPI_ISL_1914155 | 2021 | Africa / Morocco / Tinghir | Human | unknown | unknown | unknown | unknown | unknown | unknown | B.1.1.7 | GRY |
| hCoV-19/Morocco/FMP-241/2021 | EPI_ISL_1913268 | 2021 | Africa / Morocco / Tinghir | Human | unknown | unknown | unknown | unknown | unknown | unknown | B.1.1.7 | GRY |
| hCoV-19/Morocco/FMP-236/2021 | EPI_ISL_1913213 | 25/03/2021 | Africa / Morocco / Dakhla | Human | unknown | unknown | unknown | unknown | unknown | unknown | B.1.1.7 | GRY |
| hCoV-19/Morocco/FMP-235/2021 | EPI_ISL_1913165 | 19/03/2021 | Africa / Morocco / Kenitra | Human | unknown | unknown | unknown | unknown | unknown | unknown | B.1.1.7 | GRY |
| hCoV-19/Morocco/FMP-233/2021 | EPI_ISL_1913084 | 15/03/2021 | Africa / Morocco / Barchid | Human | unknown | unknown | unknown | unknown | unknown | unknown | B.1.1.7 | GRY |
| hCoV-19/Morocco/FMP-230/2021 | EPI_ISL_1913083 | 25/03/2021 | Africa / Morocco / Dakhla | Human | unknown | unknown | unknown | unknown | unknown | unknown | B.1.1.7 | GRY |
| hCoV-19/Morocco/FMP-227/2021 | EPI_ISL_1913082 | 19/03/2021 | Africa / Morocco / Sale | Human | unknown | unknown | unknown | unknown | unknown | unknown | B.1.1.7 | GRY |
| hCoV-19/Morocco/FMP-226/2021 | EPI_ISL_1913081 | 17/03/2021 | Africa / Morocco / Sale | Human | unknown | unknown | unknown | unknown | unknown | unknown | B.1.1.7 | GRY |
| hCoV-19/Morocco/FMP-224/2021 | EPI_ISL_1913077 | 15/03/2021 | Africa / Morocco / Barchid | Human | unknown | unknown | unknown | unknown | unknown | unknown | B.1.1.7 | GRY |
| hCoV-19/Morocco/FMP-223/2021 | EPI_ISL_1913074 | 17/03/2021 | Africa / Morocco / Temara | Human | unknown | unknown | unknown | unknown | unknown | unknown | B.1.1.7 | G |
| hCoV-19/Morocco/FMP-222/2021 | EPI_ISL_1913072 | 15/03/2021 | Africa / Morocco / Settat | Human | unknown | unknown | unknown | unknown | unknown | unknown | B.1.1.7 | GRY |
| hCoV-19/Morocco/FMP-221/2021 | EPI_ISL_1913069 | 19/03/2021 | Africa / Morocco / Sale | Human | unknown | unknown | unknown | unknown | unknown | unknown | B.1.1.7 | GRY |
| hCoV-19/Morocco/FMP-219/2021 | EPI_ISL_1913064 | 2021 | Africa / Morocco / Casablanca | Human | unknown | unknown | unknown | unknown | unknown | unknown | B.1.1.7 | GRY |
| hCoV-19/Morocco/FMP-218/2021 | EPI_ISL_1913062 | 14/03/2021 | Africa / Morocco / Casablanca | Human | unknown | unknown | unknown | unknown | unknown | unknown | B.1.1.7 | GRY |
| hCoV-19/Morocco/FMP-217/2021 | EPI_ISL_1913061 | 14/03/2021 | Africa / Morocco / Casablanca | Human | unknown | unknown | unknown | unknown | unknown | unknown | B.1.1.7 | GRY |
| hCoV-19/Morocco/FMP-216/2021 | EPI_ISL_1913059 | 25/03/2021 | Africa / Morocco / Dakhla | Human | unknown | unknown | unknown | unknown | unknown | unknown | B.1.1.7 | GRY |
| hCoV-19/Morocco/FMP-214/2021 | EPI_ISL_1913056 | 25/03/2021 | Africa / Morocco / Dakhla | Human | unknown | unknown | unknown | unknown | unknown | unknown | B.1.1.7 | GRY |
| hCoV-19/Morocco/FMP-213/2021 | EPI_ISL_1913054 | 2021 | Africa / Morocco / Tinghir | Human | unknown | unknown | unknown | unknown | unknown | unknown | B.1.1.7 | GRY |
| hCoV-19/Morocco/FMP-212/2021 | EPI_ISL_1913053 | 19/03/2021 | Africa / Morocco / Sale | Human | unknown | unknown | unknown | unknown | unknown | unknown | B.1.1.7 | GRY |
| hCoV-19/Morocco/FMP-211/2021 | EPI_ISL_1913051 | 19/03/2021 | Africa / Morocco / Sale | Human | unknown | unknown | unknown | unknown | unknown | unknown | B.1.1.7 | GRY |
| hCoV-19/Morocco/FMP-181/2021 | EPI_ISL_1913033 | 01/03/2021 | Africa / Morocco / Casablanca | Human | unknown | unknown | unknown | unknown | unknown | unknown | B.1.1.7 | GRY |
| hCoV-19/Morocco/FMP-179/2021 | EPI_ISL_1913031 | 2021 | Africa / Morocco / Casablanca | Human | unknown | unknown | unknown | unknown | unknown | unknown | B.1.1.7 | GRY |
| hCoV-19/Morocco/FMP-178/2021 | EPI_ISL_1913029 | 01/03/2021 | Africa / Morocco / Casablanca | Human | unknown | unknown | unknown | unknown | unknown | unknown | B.1.1.7 | GRY |
| hCoV-19/Morocco/FMP-177/2021 | EPI_ISL_1913028 | 27/02/2021 | Africa / Morocco / Casablanca | Human | unknown | unknown | unknown | unknown | unknown | unknown | B.1.1.7 | GRY |
| hCoV-19/Morocco/FMP-176/2021 | EPI_ISL_1913026 | 26/02/2021 | Africa / Morocco / Casablanca | Human | unknown | unknown | unknown | unknown | unknown | unknown | B.1.1.7 | GRY |
| hCoV-19/Morocco/FMP-174/2021 | EPI_ISL_1913019 | 02/03/2021 | Africa / Morocco / Mohammedia | Human | unknown | unknown | unknown | unknown | unknown | unknown | B.1.1.7 | GRY |
| hCoV-19/Morocco/FMP-172/2021 | EPI_ISL_1913018 | 06/02/2021 | Africa / Morocco / Casablanca | Human | unknown | unknown | unknown | unknown | unknown | unknown | B.1.1.7 | GRY |
| hCoV-19/Morocco/FMP-171/2021 | EPI_ISL_1913017 | 02/03/2021 | Africa / Morocco / casablanca | Human | unknown | unknown | unknown | unknown | unknown | unknown | B.1.1.7 | G |
| hCoV-19/Morocco/FMP-170/2021 | EPI_ISL_1913015 | 01/03/2021 | Africa / Morocco / Casablanca | Human | unknown | unknown | unknown | unknown | unknown | unknown | B.1.1.7 | GRY |
| hCoV-19/Morocco/FMP-169/2021 | EPI_ISL_1913014 | 24/02/2021 | Africa / Morocco / Casablanca | Human | unknown | unknown | unknown | unknown | unknown | unknown | B.1.1.7 | GRY |
| hCoV-19/Morocco/FMP-168/2021 | EPI_ISL_1913009 | 25/02/2021 | Africa / Morocco / Casablanca | Human | unknown | unknown | unknown | unknown | unknown | unknown | B.1.1.7 | GRY |
| hCoV-19/Morocco/FMP-166/2021 | EPI_ISL_1912937 | 23/02/2021 | Africa / Morocco / Casablanca | Human | unknown | unknown | unknown | unknown | unknown | unknown | B.1.1.7 | GRY |
| hCoV-19/Morocco/FMP-165/2021 | EPI_ISL_1912936 | 26/02/2021 | Africa / Morocco / Casablanca | Human | unknown | unknown | unknown | unknown | unknown | unknown | B.1.1.7 | GRY |
| hCoV-19/Morocco/FMP-163/2021 | EPI_ISL_1912790 | 25/02/2021 | Africa / Morocco / Casablanca | Human | unknown | unknown | unknown | unknown | unknown | unknown | B.1.1.7 | GRY |
| hCoV-19/Morocco/FMP-162/2021 | EPI_ISL_1909254 | 02/03/2021 | Africa / Morocco / Casablanca | Human | unknown | unknown | unknown | unknown | unknown | unknown | B.1.1.7 | GRY |
| hCoV-19/Morocco/FMP-287/2021 | EPI_ISL_1909252 | 09/04/2021 | Africa / Morocco / Casablanca | Human | unknown | unknown | unknown | unknown | unknown | unknown | B.1.1.7 | GRY |
| hCoV-19/Morocco/FMP-284/2021 | EPI_ISL_1909249 | 16/04/2021 | Africa / Morocco / Casablanca | Human | unknown | unknown | unknown | unknown | unknown | unknown | B.1.1.7 | G |
| hCoV-19/Morocco/FMP-283/2021 | EPI_ISL_1909247 | 09/04/2021 | Africa / Morocco / Casablanca | Human | unknown | unknown | unknown | unknown | unknown | unknown | B.1.1.7 | GRY |
| hCoV-19/Morocco/FMP-279/2021 | EPI_ISL_1909244 | 13/04/2021 | Africa / Morocco / Meknes | Human | unknown | unknown | unknown | unknown | unknown | unknown | B.1.1.7 | GRY |
| hCoV-19/Morocco/FMP-277/2021 | EPI_ISL_1909243 | 21/04/2021 | Africa / Morocco / Sale | Human | unknown | unknown | unknown | unknown | unknown | unknown | B.1.1.7 | GRY |
| hCoV-19/Morocco/FMP-276/2021 | EPI_ISL_1909242 | 07/04/2021 | Africa / Morocco / Casablanca | Human | unknown | unknown | unknown | unknown | unknown | unknown | B.1.1.7 | GRY |
| hCoV-19/Morocco/FMP-272/2021 | EPI_ISL_1909121 | 22/04/2021 | Africa / Morocco / Casablanca | Human | unknown | unknown | unknown | unknown | unknown | unknown | B.1.1.7 | GRY |
| hCoV-19/Morocco/FMP-271/2021 | EPI_ISL_1909099 | 17/04/2021 | Africa / Morocco / Casablanca | Human | unknown | unknown | unknown | unknown | unknown | unknown | B.1.1.7 | GRY |
| hCoV-19/Morocco/FMP-269/2021 | EPI_ISL_1908967 | 09/04/2021 | Africa / Morocco / Casablanca | Human | unknown | unknown | unknown | unknown | unknown | unknown | B.1.1.7 | GRY |
| hCoV-19/Morocco/FMP-265/2021 | EPI_ISL_1908735 | 08/04/2021 | Africa / Morocco / Kenitra | Human | unknown | unknown | unknown | unknown | unknown | unknown | B.1.1.7 | GRY |
| hCoV-19/Morocco/FMP-264/2021 | EPI_ISL_1908158 | 21/04/2021 | Africa / Morocco / Sale | Human | unknown | unknown | unknown | unknown | unknown | unknown | B.1.1.7 | GRY |
| hCoV-19/Morocco/FMP-263/2021 | EPI_ISL_1908156 | 2021 | Africa / Morocco / Casablanca | Human | unknown | unknown | unknown | unknown | unknown | unknown | B.1.1.7 | GRY |
| hCoV-19/Morocco/FMP-262/2021 | EPI_ISL_1908155 | 16/04/2021 | Africa / Morocco / Casablanca | Human | unknown | unknown | unknown | unknown | unknown | unknown | B.1.1.7 | GRY |
| hCoV-19/Morocco/FMP-260/2021 | EPI_ISL_1908149 | 2021 | Africa / Morocco / Casablanca | Human | unknown | unknown | unknown | unknown | unknown | unknown | B.1.1.7 | GRY |
| hCoV-19/Morocco/FMP-288/2021 | EPI_ISL_1905079 | 09/04/2021 | Africa / Morocco | Human | unknown | Female | 28 | Live | unknown | unknown | B.1.1.7 | GRY |
| hCoV-19/Morocco/FMP-250/2021 | EPI_ISL_1904884 | 15/03/2021 | Africa / Morocco / Rabat | Human | unknown | Female | 32 | unknown | unknown | unknown | B.1.1.7 | GRY |
| hCoV-19/Morocco/FMP-240/2021 | EPI_ISL_1904876 | 22/03/2021 | Africa / Morocco / Rabat | Human | unknown | Female | 37 | unknown | unknown | unknown | B.1.1.7 | GRY |
| hCoV-19/Morocco/FMP-210/2021 | EPI_ISL_1904599 | 19/03/2021 | Africa / Morocco / SalÃ© | Human | unknown | unknown | unknown | unknown | unknown | unknown | B.1.1.7 | GRY |
| hCoV-19/Morocco/FMP-209/2021 | EPI_ISL_1904561 | 15/03/2021 | Africa / Morocco / Casablanca | Human | unknown | unknown | unknown | unknown | unknown | unknown | B.1.1.7 | G |
| hCoV-19/Morocco/FMP-202/2021 | EPI_ISL_1896699 | 17/03/2021 | Africa / Morocco / Sidi Lahcen | Human | unknown | Male | 73 | unknown | unknown | unknown | B.1.1.7 | GRY |
| hCoV-19/Morocco/FMP-198/2021 | EPI_ISL_1896698 | 01/03/2021 | Africa / Morocco / Casablanca | Human | unknown | unknown | unknown | unknown | unknown | unknown | B.1.1.7 | GRY |
| hCoV-19/Morocco/FMP-197/2021 | EPI_ISL_1896670 | 24/02/2021 | Africa / Morocco / Casablanca | Human | unknown | unknown | unknown | unknown | unknown | unknown | B.1.1.7 | GRY |
| hCoV-19/Morocco/FMP-196/2021 | EPI_ISL_1896669 | 2021 | Africa / Morocco / Casablanca | Human | unknown | unknown | unknown | unknown | unknown | unknown | B.1.1.7 | GRY |
| hCoV-19/Morocco/FMP-195/2021 | EPI_ISL_1896668 | 16/03/2021 | Africa / Morocco / Sidi Lahcen | Human | unknown | Female | 17 | unknown | unknown | unknown | B.1.1.7 | GRY |
| hCoV-19/Morocco/FMP-194/2021 | EPI_ISL_1896158 | 16/03/2021 | Africa / Morocco / Rabat | Human | unknown | Female | 20 | unknown | unknown | unknown | B.1.1.7 | GRY |
| hCoV-19/Morocco/FMP-95/2021 | EPI_ISL_1811221 | 07/01/2021 | Africa / Morocco / Rabat | Human | unknown | Male | 38 | unknown | unknown | unknown | B.1.1 | GR |
| hCoV-19/Morocco/FMP-94/2021 | EPI_ISL_1810949 | 07/01/2021 | Africa / Morocco / Rabat | Human | unknown | Female | 23 | unknown | unknown | unknown | B.1.597 | GH |
| hCoV-19/Morocco/FMP-91/2021 | EPI_ISL_1810947 | 02/01/2021 | Africa / Morocco / Rabat | Human | unknown | Male | 22 | unknown | unknown | unknown | B.1 | G |
| hCoV-19/Morocco/FMP-90/2021 | EPI_ISL_1810938 | 02/01/2021 | Africa / Morocco / Rabat | Human | unknown | Male | 44 | unknown | unknown | unknown | B.1.1 | GR |
| hCoV-19/Morocco/FMP-88/2021 | EPI_ISL_1810936 | 07/01/2021 | Africa / Morocco / Sidi Lahcen | Human | unknown | Female | 82 | unknown | unknown | unknown | B.1 | GH |
| hCoV-19/Morocco/FMP-86/2021 | EPI_ISL_1810934 | 07/01/2021 | Africa / Morocco / Rabat | Human | unknown | Female | 57 | unknown | unknown | unknown | B.1.177.77 | GV |
| hCoV-19/Morocco/FMP-85/2021 | EPI_ISL_1810933 | 06/01/2021 | Africa / Morocco / Sidi Lahcen | Human | unknown | Female | 25 | unknown | unknown | unknown | B.1.1 | GR |
| hCoV-19/Morocco/6906/2020 | EPI_ISL_459984 | 06/04/2020 | Africa / Morocco | Human | unknown | unknown | unknown | unknown | unknown | unknown | B.1.1 | GR |
| hCoV-19/Morocco/6895/2020 | EPI_ISL_459973 | 20/03/2020 | Africa / Morocco | Human | unknown | unknown | unknown | unknown | unknown | unknown | B.1.1 | GR |
| hCoV-19/Morocco/FMP-123/2021 | EPI_ISL_2313079 | 2021 | Africa / Morocco / Tinghir | Human | unknown | unknown | unknown | unknown | unknown | unknown | B.1.1 | GR |
| hCoV-19/Morocco/FMP-27/2020 | EPI_ISL_728355 | 2020-12 | Africa / Morocco / Harhoura | Human | unknown | Female | 35 | unknown | unknown | unknown | B.1 | GH |
| hCoV-19/Morocco/FMP-25/2020 | EPI_ISL_728352 | 2020-12 | Africa / Morocco / Temara | Human | unknown | Female | 40 | unknown | unknown | unknown | B.1.1 | GR |
| hCoV-19/Morocco/FMP-40/2020 | EPI_ISL_768839 | 2020-11 | Africa / Morocco | Human | unknown | unknown | unknown | unknown | unknown | unknown | B.1 | G |
| hCoV-19/Morocco/1185/2022 | EPI_ISL_16098027 | 21/11/2022 | Africa / Morocco / Sale | Human | unknown | Male | 46 years | Live | unknown | unknown | XBB.1 | GRA |
| hCoV-19/Morocco/1168/2022 | EPI_ISL_16098032 | 01/11/2022 | Africa / Morocco / Rabat | Human | unknown | Male | 55 years | Live | unknown | unknown | BQ.1.1.69 | GRA |
| hCoV-19/Morocco/1186/2022 | EPI_ISL_16098028 | 11/11/2022 | Africa / Morocco / Rabat | Human | unknown | Male | 60 years | Live | unknown | unknown | BQ.1.1 | GRA |
| hCoV-19/Morocco/1187/2022 | EPI_ISL_16098029 | 11/11/2022 | Africa / Morocco / Sale | Human | unknown | Male | 44 years | Live | unknown | unknown | BQ.1.1 | GRA |
| hCoV-19/Morocco/1189/2022 | EPI_ISL_16098030 | 14/11/2022 | Africa / Morocco / Rabat | Human | unknown | Male | unknown | Live | unknown | unknown | BQ.1.1 | GRA |
| hCoV-19/Morocco/1195/2022 | EPI_ISL_16098031 | 22/11/2022 | Africa / Morocco / Rabat | Human | unknown | Female | 54 years | Live | unknown | unknown | BQ.1.1 | GRA |
| hCoV-19/Morocco/1202/2022 | EPI_ISL_16098033 | 28/11/2022 | Africa / Morocco / Sale | Human | unknown | Male | 44 years | Live | unknown | unknown | BQ.1.1 | GRA |
| hCoV-19/Morocco/FMP-84/2021 | EPI_ISL_1159700 | 06/01/2021 | Africa / Morocco | Human | unknown | Female | 24 | unknown | unknown | unknown | B.1 | GH |
| hCoV-19/Morocco/FMP-83/2021 | EPI_ISL_1159699 | 04/01/2021 | Africa / Morocco / Sidi Lahcen | Human | unknown | Male | 41 | unknown | unknown | unknown | B.1.177 | GV |
| hCoV-19/Morocco/FMP-82/2021 | EPI_ISL_1159698 | 02/01/2021 | Africa / Morocco / My Youssef | Human | unknown | Female | 30 | unknown | unknown | unknown | B.1.1 | GR |
| hCoV-19/Morocco/FMP-81/2021 | EPI_ISL_1159697 | 02/01/2021 | Africa / Morocco / My Youssef | Human | unknown | Female | 49 | unknown | unknown | unknown | B.1 | G |
| hCoV-19/Morocco/FMP80/2021 | EPI_ISL_1137621 | 08/01/2021 | Africa / Morocco / Sidi Lahcen | Human | unknown | Female | 48 | unknown | unknown | unknown | B.1.1.7 | GRY |
| hCoV-19/Morocco/FMP-79/2021 | EPI_ISL_1120732 | 06/01/2021 | Africa / Morocco / Sidi Lahcen | Human | unknown | Male | 12 | unknown | unknown | unknown | B.1.1 | GR |
| hCoV-19/Morocco/FMP-78/2021 | EPI_ISL_1117289 | 06/01/2021 | Africa / Morocco / Sidi Lahcen | Human | unknown | Male | 33 | unknown | unknown | unknown | B.1 | G |
| hCoV-19/Morocco/FMP_77/2021 | EPI_ISL_1116592 | 06/01/2021 | Africa / Morocco | Human | unknown | Female | 76 | unknown | unknown | unknown | B.1.1 | GR |
| hCoV-19/Morocco/FMP-76/2021 | EPI_ISL_1116555 | 04/01/2021 | Africa / Morocco / Sidi Lahcen | Human | unknown | Male | 52 | unknown | unknown | unknown | B.1 | GH |
| hCoV-19/Morocco/FMP-75/2021 | EPI_ISL_1116470 | 04/02/2021 | Africa / Morocco / My Youssef | Human | unknown | Female | 25 | unknown | unknown | unknown | B.1.1 | GR |
| hCoV-19/Morocco/FMP-74/2021 | EPI_ISL_1116466 | 04/02/2021 | Africa / Morocco / Sidi Lahcen | Human | unknown | Male | 26 | unknown | unknown | unknown | B.1 | G |
| hCoV-19/Morocco/FMP-73/2021 | EPI_ISL_1116465 | 02/01/2021 | Africa / Morocco / Sidi Lahcen | Human | unknown | Female | 50 | unknown | unknown | unknown | B.1 | G |
| hCoV-19/Morocco/FMP-234/2021 | EPI_ISL_1913086 | 11/03/2021 | Africa / Morocco / Casablanca | Human | unknown | unknown | unknown | unknown | unknown | unknown | B.1.1.7 | GRY |
| hCoV-19/Morocco/FMP-71/2021 | EPI_ISL_1109622 | 07/01/2021 | Africa / Morocco / Sidi Lahcen | Human | unknown | Male | 57 | unknown | unknown | unknown | B.1 | GH |
| hCoV-19/Morocco/FMP-70/2021 | EPI_ISL_1107189 | 07/01/2021 | Africa / Morocco / Sidi Lahcen | Human | unknown | Female | 30 | unknown | unknown | unknown | B.1 | G |
| hCoV-19/Morocco/HMIMV-00111/2020 | EPI_ISL_2968059 | 07/12/2020 | Africa / Morocco / Rabat | Human | unknown | Male | 66 | Released | unknown | unknown | B.1 | G |
| hCoV-19/Morocco/FMP-69/2021 | EPI_ISL_1105946 | 06/01/2021 | Africa / Morocco / Sidi Lahcen | Human | unknown | Female | 20 | unknown | unknown | unknown | B.1 | GH |
| hCoV-19/Morocco/NIH-1193/2022 | EPI_ISL_16201189 | 22/11/2022 | Africa / Morocco / Rabat | Human | unknown | Male | 60 | Live | unknown | unknown | BQ.1.1 | GRA |
| hCoV-19/Morocco/NIH-1188/2022 | EPI_ISL_16201187 | 10/11/2022 | Africa / Morocco / Rabat | Human | unknown | Female | 21 | Live | unknown | unknown | BQ.1.23 | GRA |
| hCoV-19/Morocco/NIH-1197/2022 | EPI_ISL_16201192 | 25/11/2022 | Africa / Morocco / Sale | Human | unknown | Male | 45 | Live | unknown | unknown | BQ.1.1 | GRA |
| hCoV-19/Morocco/NIH/1181/2022 | EPI_ISL_16201246 | 15/11/2022 | Africa / Morocco / Rabat | Human | unknown | Male | 50 Years | Live | unknown | unknown | BQ.1.1 | GRA |
| hCoV-19/Morocco/NIH-1191/2022 | EPI_ISL_16201186 | 22/11/2022 | Africa / Morocco / Sale | Human | unknown | Male | 50 | Live | unknown | unknown | BQ.1.1 | GRA |
| hCoV-19/Morocco/NIH-1190/2022 | EPI_ISL_16201188 | 22/11/2022 | Africa / Morocco / Sale | Human | unknown | Male | 32 | Live | unknown | unknown | BQ.1.1 | GRA |
| hCoV-19/Morocco/NIH-1194/2022 | EPI_ISL_16201190 | 22/11/2022 | Africa / Morocco / Rabat | Human | unknown | Male | 60 | Live | unknown | unknown | BQ.1.31 | GRA |
| hCoV-19/Morocco/NIH-1196/2022 | EPI_ISL_16201191 | 26/11/2022 | Africa / Morocco / Rabat | Human | unknown | Male | 36 | Live | unknown | unknown | BQ.1.1 | GRA |
| hCoV-19/Morocco/NIH/1182/2022 | EPI_ISL_16201247 | 15/11/2022 | Africa / Morocco / Rabat | Human | unknown | Male | Unknew | Live | unknown | unknown | BQ.1.1 | GRA |
| hCoV-19/Morocco/NIH/1183/2022 | EPI_ISL_16201248 | 15/11/2022 | Africa / Morocco / Sale | Human | unknown | Male | Unknew | Live | unknown | unknown | BQ.1.1 | GRA |
| hCoV-19/Morocco/NIH/1192/2022 | EPI_ISL_16201249 | 22/11/2022 | Africa / Morocco / Rabat | Human | unknown | Male | 57 years | Live | unknown | unknown | BQ.1.1 | GRA |
| hCoV-19/Morocco/NIH/1198/2022 | EPI_ISL_16201250 | 22/11/2022 | Africa / Morocco / Rabat | Human | unknown | Male | 44 years | Live | unknown | unknown | BQ.1.1 | GRA |
| hCoV-19/Morocco/FMP-68/2021 | EPI_ISL_1103584 | 05/01/2021 | Africa / Morocco / Sidi Lahcen | Human | unknown | Female | 37 | unknown | unknown | unknown | B.1.177 | GV |
| hCoV-19/Morocco/FMP-67/2021 | EPI_ISL_1103579 | 05/01/2021 | Africa / Morocco | Human | unknown | Female | 61 | unknown | unknown | unknown | B.1 | GH |
| hCoV-19/Morocco/FMP-66/2021 | EPI_ISL_1103576 | 04/02/2021 | Africa / Morocco / Sidi Lahcen | Human | unknown | Female | 27 | unknown | unknown | unknown | B.1 | G |
| hCoV-19/Morocco/FMP-65/2021 | EPI_ISL_1103569 | 02/01/2021 | Africa / Morocco / Sidi Lahcen | Human | unknown | Male | 40 | Unknown | unknown | unknown | B.1 | G |
| hCoV-19/Morocco/NIH/1240/2022 | EPI_ISL_16359850 | 26/12/2022 | Africa / Morocco / Rabat | Human | unknown | Female | 62 | Live | unknown | unknown | BQ.1.1 | GRA |
| hCoV-19/Morocco/NIH/1236/2022 | EPI_ISL_16359847 | 25/12/2022 | Africa / Morocco / Rabat | Human | unknown | Male | unknown | Live | unknown | unknown | BQ.1.1 | GRA |
| hCoV-19/Morocco/NIH/1238/2022 | EPI_ISL_16359849 | 27/12/2022 | Africa / Morocco / Kenitra | Human | unknown | Female | 20 | Live | unknown | unknown | BQ.1.1 | GRA |
| hCoV-19/Morocco/NIH/1234/2022 | EPI_ISL_16359853 | 10/12/2022 | Africa / Morocco / Beni Mellal | Human | unknown | Female | 21 | Live | unknown | unknown | BQ.1.1 | GRA |
| hCoV-19/Morocco/NIH/1235/2022 | EPI_ISL_16359854 | 10/12/2022 | Africa / Morocco / Beni Mellal | Human | unknown | Male | 62 | Live | unknown | unknown | BQ.1.1 | GRA |
| hCoV-19/Morocco/NIH/1206/2022 | EPI_ISL_16359852 | 12/12/2022 | Africa / Morocco / Rabat | Human | unknown | Male | unknown | Live | unknown | unknown | BQ.1.1 | GRA |
| hCoV-19/Morocco/NIH/1239/2022 | EPI_ISL_16359855 | 27/12/2022 | Africa / Morocco / Kenitra | Human | unknown | Male | 21 | Live | unknown | unknown | BQ.1.5 | GRA |
| hCoV-19/Morocco/NIH/1230/2022 | EPI_ISL_16359846 | 22/12/2022 | Africa / Morocco / Rabat | Human | unknown | Male | 23 | Live | unknown | unknown | BQ.1.1 | GRA |
| hCoV-19/Morocco/NIH/1233/2022 | EPI_ISL_16359845 | 30/11/2022 | Africa / Morocco / Rabat | Human | unknown | Female | 25 | Live | unknown | unknown | BQ.1 | GRA |
| hCoV-19/Morocco/NIH/1237/2022 | EPI_ISL_16359848 | 27/12/2022 | Africa / Morocco / Kenitra | Human | unknown | Male | 23 | Live | unknown | unknown | BQ.1.1 | GRA |
| hCoV-19/Morocco/NIH/1241/2022 | EPI_ISL_16359851 | 28/12/2022 | Africa / Morocco / Rabat | Human | unknown | Female | 57 | Live | unknown | unknown | BQ.1.1 | GRA |
| hCoV-19/Morocco/NIH-1218/2022 | EPI_ISL_16378348 | 12/12/2022 | Africa / Morocco / Sale | Human | unknown | Male | 45 | Live | unknown | unknown | DB.1 | GRA |
| hCoV-19/Morocco/NIH-1226/2022 | EPI_ISL_16378350 | 15/12/2022 | Africa / Morocco / Rabat | Human | unknown | Female | 51 | Live | unknown | unknown | BQ.1.1.7 (consensus call) | GRA |
| hCoV-19/Morocco/NIH-1220/2022 | EPI_ISL_16378340 | 12/12/2022 | Africa / Morocco / Rabat | Human | unknown | Male | unknown | Live | unknown | unknown | BQ.1.1 | GRA |
| hCoV-19/Morocco/NIH-1229/2022 | EPI_ISL_16378342 | 19/12/2022 | Africa / Morocco / Rabat | Human | unknown | Male | unknown | Live | unknown | unknown | BQ.1.1.18 | GRA |
| hCoV-19/Morocco/NIH-1208/2022 | EPI_ISL_16378345 | 05/12/2022 | Africa / Morocco / Rabat | Human | unknown | Female | unknown | Live | unknown | unknown | BQ.1.1 | GRA |
| hCoV-19/Morocco/NIH-1207/2022 | EPI_ISL_16378346 | 05/12/2022 | Africa / Morocco / Rabat | Human | unknown | Female | unknown | Live | unknown | unknown | BQ.1.1.18 | GRA |
| hCoV-19/Morocco/NIH-1224/2022 | EPI_ISL_16378341 | 15/12/2022 | Africa / Morocco / Sale | Human | unknown | Female | 28 | Live | unknown | unknown | BQ.1.1.58 | GRA |
| hCoV-19/Morocco/NIH-1215/2022 | EPI_ISL_16378347 | 12/12/2022 | Africa / Morocco / Rabat | Human | unknown | Female | unknown | Live | unknown | unknown | BQ.1.1.18 | GRA |
| hCoV-19/Morocco/NIH-1210/2022 | EPI_ISL_16378339 | 02/12/2022 | Africa / Morocco / Sale | Human | unknown | Male | unknown | Live | unknown | unknown | BQ.1.1 | GRA |
| hCoV-19/Morocco/NIH-1228/2022 | EPI_ISL_16378352 | 16/12/2022 | Africa / Morocco / Kenitra | Human | unknown | Female | 25 | Live | unknown | unknown | XBB.1 | GRA |
| hCoV-19/Morocco/NIH-1232/2022 | EPI_ISL_16378343 | 09/12/2022 | Africa / Morocco / Sale | Human | unknown | Male | 58 | Live | unknown | unknown | BQ.1.1 | GRA |
| hCoV-19/Morocco/NIH-1209/2022 | EPI_ISL_16378344 | 02/12/2022 | Africa / Morocco / Rabat | Human | unknown | Female | 61 | Live | unknown | unknown | BQ.1.1.4 | GRA |
| hCoV-19/Morocco/NIH-1219/2022 | EPI_ISL_16378349 | 12/12/2022 | Africa / Morocco / Sale | Human | unknown | Male | 45 | Live | unknown | unknown | BQ.1.1 | GRA |
| hCoV-19/Morocco/NIH-1227/2022 | EPI_ISL_16378351 | 16/12/2022 | Africa / Morocco / Rabat | Human | unknown | Female | 61 | Live | unknown | unknown | BQ.1.1 | GRA |
| hCoV-19/Morocco/NIH-1222/2022 | EPI_ISL_16378353 | 13/12/2022 | Africa / Morocco / Kenitra | Human | unknown | Male | unknown | Live | unknown | unknown | BQ.1.1 | GRA |
| hCoV-19/Morocco/FMP-124/2021 | EPI_ISL_2313080 | 2021 | Africa / Morocco / Kenitra | Human | unknown | unknown | unknown | unknown | unknown | unknown | B.1.160 | GH |
| hCoV-19/Morocco/IPM20376980/2021 | EPI_ISL_5924603 | 13/10/2021 | Africa / Morocco / Casablanca | Human | unknown | Female | 24 | unknown | unknown | unknown | B.1.617.2 | GK |
| hCoV-19/Morocco/ouar246bis/2020 | EPI_ISL_978552 | 05/05/2020 | Africa / Morocco / Ouarzazate | Human | unknown | Male | 55 | unknown | unknown | unknown | B.1.528 | G |
| hCoV-19/Morocco/ouar246/2020 | EPI_ISL_978551 | 05/05/2020 | Africa / Morocco / Ouarzazate | Human | unknown | Male | 55 | unknown | unknown | unknown | B.1.528 | G |
| hCoV-19/Morocco/ouar475bis/2020 | EPI_ISL_978550 | 05/05/2020 | Africa / Morocco / Ouarzazate | Human | unknown | Male | 57 | unknown | unknown | unknown | B.1.528 | G |
| hCoV-19/Morocco/ouar397/2020 | EPI_ISL_978548 | 05/05/2020 | Africa / Morocco / Ouarzazate | Human | unknown | Male | 48 | unknown | unknown | unknown | B.1.528 | G |
| hCoV-19/Morocco/ouar396bis/2020 | EPI_ISL_978547 | 05/05/2020 | Africa / Morocco / Ouarzazate | Human | unknown | Male | 33 | unknown | unknown | unknown | B.1.528 | G |
| hCoV-19/Morocco/ouar396/2020 | EPI_ISL_978546 | 05/05/2020 | Africa / Morocco / Ouarzazate | Human | unknown | Male | 38 | unknown | unknown | unknown | B.1.528 | G |
| hCoV-19/Morocco/ouar377bis/2020 | EPI_ISL_978542 | 05/05/2020 | Africa / Morocco / Ouarzazate | Human | unknown | Male | 46 | unknown | unknown | unknown | B.1.528 | G |
| hCoV-19/Morocco/ouar377/2020 | EPI_ISL_978541 | 05/05/2020 | Africa / Morocco / Ouarzazate | Human | unknown | Male | 30 | unknown | unknown | unknown | B.1.528 | G |
| hCoV-19/Morocco/ouar372/2020 | EPI_ISL_978540 | 05/05/2020 | Africa / Morocco / Ouarzazate | Human | unknown | Male | 65 | unknown | unknown | unknown | B.1.528 | G |
| hCoV-19/Morocco/ouar371/2020 | EPI_ISL_978539 | 05/05/2020 | Africa / Morocco / Ouarzazate | Human | unknown | Male | 33 | unknown | unknown | unknown | B.1.528 | G |
| hCoV-19/Morocco/ouar366/2020 | EPI_ISL_978538 | 05/05/2020 | Africa / Morocco / Ouarzazate | Human | unknown | Male | 32 | unknown | unknown | unknown | B.1.528 | G |
| hCoV-19/Morocco/ouar269/2020 | EPI_ISL_978537 | 05/05/2020 | Africa / Morocco / Ouarzazate | Human | unknown | Male | 61 | unknown | unknown | unknown | B.1.528 | G |
| hCoV-19/Morocco/ouar254/2020 | EPI_ISL_978536 | 05/05/2020 | Africa / Morocco / Ouarzazate | Human | unknown | Male | 65 | unknown | unknown | unknown | B.1.528 | G |
| hCoV-19/Morocco/ouar224/2020 | EPI_ISL_978534 | 05/05/2020 | Africa / Morocco / Ouarzazate | Human | unknown | Male | 43 | unknown | unknown | unknown | B.1.528 | G |
| hCoV-19/Morocco/HMIMV-279CC/2020 | EPI_ISL_966940 | 01/07/2020 | Africa / Morocco | Human | unknown | unknown | unknown | unknown | unknown | unknown | B.1 | GH |
| hCoV-19/Morocco/FMP-99/2021 | EPI_ISL_2117459 | 2021 | Africa / Morocco / Mohammedia | Human | Baseline surveillance | unknown | unknown | unknown | unknown | Baseline surveillance | B.1.1.317 | GR |
| hCoV-19/env/Morocco/SW5/2020 | EPI_ISL_933719 | 14/09/2020 | Africa / Morocco / Rabat | Human | unknown | NA | NA | NA | unknown | unknown | B.1 | G |
| hCoV-19/env/Morocco/SW4/2020 | EPI_ISL_933715 | 14/09/2020 | Africa / Morocco / Rabat | unknown | unknown | NA | NA | NA | unknown | unknown | B.1 | G |
| hCoV-19/Morocco/nCoV20326490/2021 | EPI_ISL_4300688 | 12/07/2021 | Africa / Morocco / Casablanca | Human | unknown | Male | 16 | unknown | unknown | unknown | B.1.617.2 | GK |
| hCoV-19/Morocco/nCoV20326487/2021 | EPI_ISL_4300687 | 12/07/2021 | Africa / Morocco / Casablanca | Human | unknown | Female | 61 | unknown | unknown | unknown | B.1.1.7 | GRY |
| hCoV-19/Morocco/nCoV20326297/2021 | EPI_ISL_4300682 | 12/07/2021 | Africa / Morocco / Casablanca | Human | unknown | Female | 65 | unknown | unknown | unknown | B.1.1.7 | GRY |
| hCoV-19/Morocco/nCoV20326349/2021 | EPI_ISL_4300684 | 12/07/2021 | Africa / Morocco / Casablanca | Human | unknown | Female | 16 | unknown | unknown | unknown | B.1.617.2 | GK |
| hCoV-19/Morocco/nCoV20326470/2021 | EPI_ISL_4300686 | 12/07/2021 | Africa / Morocco / Casablanca | Human | unknown | Female | 63 | unknown | unknown | unknown | AY.33 | GK |
| hCoV-19/Morocco/nCoV20326467/2021 | EPI_ISL_4300685 | 12/07/2021 | Africa / Morocco / Casablanca | Human | unknown | Female | 11 | unknown | unknown | unknown | B.1.617.2 | GK |
| hCoV-19/Morocco/FMP-253/2021 | EPI_ISL_1904888 | 23/03/2021 | Africa / Morocco / Rabat | Human | unknown | Female | 26 | unknown | unknown | unknown | B.1.1.7 | GRY |
| hCoV-19/Morocco/6903/2020 | EPI_ISL_459981 | 19/04/2020 | Africa / Morocco | Human | unknown | unknown | unknown | unknown | unknown | unknown | B.1 | G |
| hCoV-19/Morocco/6899/2020 | EPI_ISL_459977 | 21/04/2020 | Africa / Morocco | Human | unknown | unknown | unknown | unknown | unknown | unknown | B.1.1 | GR |
| hCoV-19/Morocco/6894/2020 | EPI_ISL_459972 | 20/03/2020 | Africa / Morocco | Human | unknown | unknown | unknown | unknown | unknown | unknown | B.1 | G |
| hCoV-19/Morocco/IPM20434267/2023 | EPI_ISL_16649944 | 04/01/2023 | Africa / Morocco / Casablanca | Human | unknown | Female | 57 | unknown | unknown | unknown | BA.4 | GRA |
| hCoV-19/Morocco/IPM20434222/2023 | EPI_ISL_16649943 | 04/01/2023 | Africa / Morocco / Casablanca | Human | unknown | Female | 41 | unknown | unknown | unknown | BA.2 | GRA |
| hCoV-19/Morocco/IPM20434188/2023 | EPI_ISL_16649942 | 04/01/2023 | Africa / Morocco / Casablanca | Human | unknown | Male | 30 | unknown | unknown | unknown | BA.2 | GRA |
| hCoV-19/Morocco/IPM20434038/2023 | EPI_ISL_16649941 | 03/01/2023 | Africa / Morocco / Casablanca | Human | unknown | Male | 28 | unknown | unknown | unknown | BA.2 | GRA |
| hCoV-19/Morocco/IPM20434020/2023 | EPI_ISL_16649940 | 02/01/2023 | Africa / Morocco / Casablanca | Human | unknown | Male | 60 | unknown | unknown | unknown | BQ.1.1 | GRA |
| hCoV-19/Morocco/IPM20434016/2023 | EPI_ISL_16649939 | 02/01/2023 | Africa / Morocco / Casablanca | Human | unknown | Male | 57 | unknown | unknown | unknown | BQ.1.1.56 | GRA |
| hCoV-19/Morocco/IPM20433993/2023 | EPI_ISL_16649938 | 02/01/2023 | Africa / Morocco / Casablanca | Human | unknown | Female | 27 | unknown | unknown | unknown | BA.5.3.1 | GRA |
| hCoV-19/Morocco/IPM20433968/2022 | EPI_ISL_16649937 | 30/12/2022 | Africa / Morocco / Casablanca | Human | unknown | Female | 20 | unknown | unknown | unknown | BA.2 | GRA |
| hCoV-19/Morocco/IPM20433952/2022 | EPI_ISL_16649936 | 30/12/2022 | Africa / Morocco / Casablanca | Human | unknown | Female | 30 | unknown | unknown | unknown | BQ.1.1.56 | GRA |
| hCoV-19/Morocco/IPM20433934/2022 | EPI_ISL_16649935 | 29/12/2022 | Africa / Morocco / Casablanca | Human | unknown | Male | 29 | unknown | unknown | unknown | BA.2 | GRA |
| hCoV-19/Morocco/IPM20433918/2022 | EPI_ISL_16649934 | 29/12/2022 | Africa / Morocco / Casablanca | Human | unknown | Male | 33 | unknown | unknown | unknown | BQ.1.1 | GRA |
| hCoV-19/Morocco/IPM20433896/2022 | EPI_ISL_16649933 | 29/12/2022 | Africa / Morocco / Casablanca | Human | unknown | Female | 58 | unknown | unknown | unknown | BA.2 | GRA |
| hCoV-19/Morocco/IPM20433881/2022 | EPI_ISL_16649932 | 29/12/2022 | Africa / Morocco / Casablanca | Human | unknown | Male | 30 | unknown | unknown | unknown | BQ.1 | GRA |
| hCoV-19/Morocco/IPM20433875/2022 | EPI_ISL_16649931 | 29/12/2022 | Africa / Morocco / Casablanca | Human | unknown | Male | 29 | unknown | unknown | unknown | BQ.1.1 | GRA |
| hCoV-19/Morocco/IPM20433849/2022 | EPI_ISL_16649930 | 28/12/2022 | Africa / Morocco / Casablanca | Human | unknown | Female | 49 | unknown | unknown | unknown | BA.5.3.1 | GRA |
| hCoV-19/Morocco/IPM20433848/2022 | EPI_ISL_16649929 | 28/12/2022 | Africa / Morocco / Casablanca | Human | unknown | Male | 63 | unknown | unknown | unknown | BQ.1.1 | GRA |
| hCoV-19/Morocco/IPM20433785/2022 | EPI_ISL_16649928 | 28/12/2022 | Africa / Morocco / Casablanca | Human | unknown | Female | 26 | unknown | unknown | unknown | BQ.1.1 | GRA |
| hCoV-19/Morocco/IPM20433783/2022 | EPI_ISL_16649927 | 28/12/2022 | Africa / Morocco / Casablanca | Human | unknown | Male | 54 | unknown | unknown | unknown | BQ.1 | GRA |
| hCoV-19/Morocco/IPM20433695/2022 | EPI_ISL_16649926 | 27/12/2022 | Africa / Morocco / Casablanca | Human | unknown | Male | 58 | unknown | unknown | unknown | BQ.1.1 | GRA |
| hCoV-19/Morocco/IPM20433694/2022 | EPI_ISL_16649925 | 27/12/2022 | Africa / Morocco / Casablanca | Human | unknown | Male | 61 | unknown | unknown | unknown | BA.4 | GRA |
| hCoV-19/Morocco/IPM20433693/2022 | EPI_ISL_16649924 | 27/12/2022 | Africa / Morocco / Casablanca | Human | unknown | Male | 56 | unknown | unknown | unknown | CR.1 | GRA |
| hCoV-19/Morocco/IPM20433667/2022 | EPI_ISL_16649923 | 26/12/2022 | Africa / Morocco / Casablanca | Human | unknown | Male | 42 | unknown | unknown | unknown | BQ.1.1.58 | GRA |
| hCoV-19/Morocco/IPM20433666/2022 | EPI_ISL_16649922 | 26/12/2022 | Africa / Morocco / Casablanca | Human | unknown | Male | 42 | unknown | unknown | unknown | BQ.1 | GRA |
| hCoV-19/Morocco/IPM20433664/2022 | EPI_ISL_16649921 | 26/12/2022 | Africa / Morocco / Casablanca | Human | unknown | Male | 46 | unknown | unknown | unknown | BQ.1.1.58 | GRA |
| hCoV-19/Morocco/IPM20433652/2022 | EPI_ISL_16649920 | 23/12/2022 | Africa / Morocco / Casablanca | Human | unknown | Female | 31 | unknown | unknown | unknown | BA.5.2 | GRA |
| hCoV-19/Morocco/IPM20433616/2022 | EPI_ISL_16649919 | 22/12/2022 | Africa / Morocco / Casablanca | Human | unknown | Male | 29 | unknown | unknown | unknown | BQ.1 | GRA |
| hCoV-19/Morocco/IPM20433614/2022 | EPI_ISL_16649918 | 22/12/2022 | Africa / Morocco / Casablanca | Human | unknown | Female | 34 | unknown | unknown | unknown | BQ.1.1 | GRA |
| hCoV-19/Morocco/IPM20433591/2022 | EPI_ISL_16649917 | 21/12/2022 | Africa / Morocco / Casablanca | Human | unknown | Female | 35 | unknown | unknown | unknown | BE.1.1 | GRA |
| hCoV-19/Morocco/IPM20433583/2022 | EPI_ISL_16649916 | 21/12/2022 | Africa / Morocco / Casablanca | Human | unknown | Male | 38 | unknown | unknown | unknown | BQ.1 | GRA |
| hCoV-19/Morocco/IPM20433579/2022 | EPI_ISL_16649915 | 21/12/2022 | Africa / Morocco / Casablanca | Human | unknown | Male | 61 | unknown | unknown | unknown | BQ.1 | GRA |
| hCoV-19/Morocco/IPM20433571/2022 | EPI_ISL_16649914 | 21/12/2022 | Africa / Morocco / Casablanca | Human | unknown | Female | 25 | unknown | unknown | unknown | BA.2 | GRA |
| hCoV-19/Morocco/IPM20433493/2022 | EPI_ISL_16649913 | 20/12/2022 | Africa / Morocco / Casablanca | Human | unknown | Male | 29 | unknown | unknown | unknown | DB.1 | GRA |
| hCoV-19/Morocco/IPM20433491/2022 | EPI_ISL_16649912 | 19/12/2022 | Africa / Morocco / Casablanca | Human | unknown | Male | 36 | unknown | unknown | unknown | BA.2.75.5 | GRA |
| hCoV-19/Morocco/IPM20433481/2022 | EPI_ISL_16649911 | 19/12/2022 | Africa / Morocco / Casablanca | Human | unknown | Male | 36 | unknown | unknown | unknown | BQ.1 | GRA |
| hCoV-19/Morocco/IPM20433479/2022 | EPI_ISL_16649910 | 19/12/2022 | Africa / Morocco / Casablanca | Human | unknown | Female | 27 | unknown | unknown | unknown | BA.2.75.5 | GRA |
| hCoV-19/Morocco/IPM20433476/2022 | EPI_ISL_16649909 | 19/12/2022 | Africa / Morocco / Casablanca | Human | unknown | Male | 66 | unknown | unknown | unknown | BA.2 | GRA |
| hCoV-19/Morocco/IPM20433464/2022 | EPI_ISL_16649908 | 19/12/2022 | Africa / Morocco / Casablanca | Human | unknown | Male | 26 | unknown | unknown | unknown | BA.2 | GRA |
| hCoV-19/Morocco/IPM20433357/2022 | EPI_ISL_16649907 | 15/12/2022 | Africa / Morocco / Casablanca | Human | unknown | Female | 29 | unknown | unknown | unknown | BQ.1.1 | GRA |
| hCoV-19/Morocco/IPM20433353/2022 | EPI_ISL_16649906 | 15/12/2022 | Africa / Morocco / Casablanca | Human | unknown | Male | 37 | unknown | unknown | unknown | BQ.1.1 | GRA |
| hCoV-19/Morocco/IPM20433328/2022 | EPI_ISL_16649905 | 14/12/2022 | Africa / Morocco / Casablanca | Human | unknown | Female | 47 | unknown | unknown | unknown | BA.2 | GRA |
| hCoV-19/Morocco/IPM20433252/2022 | EPI_ISL_16649904 | 13/12/2022 | Africa / Morocco / Casablanca | Human | unknown | Male | 33 | unknown | unknown | unknown | BA.2.10.1 | GRA |
| hCoV-19/Morocco/IPM20433242/2022 | EPI_ISL_16649903 | 13/12/2022 | Africa / Morocco / Casablanca | Human | unknown | Male | 34 | unknown | unknown | unknown | BQ.1 | GRA |
| hCoV-19/Morocco/IPM20433218/2022 | EPI_ISL_16649902 | 12/12/2022 | Africa / Morocco / Casablanca | Human | unknown | Female | 69 | unknown | unknown | unknown | BQ.1 | GRA |
| hCoV-19/Morocco/IPM20433206/2022 | EPI_ISL_16649901 | 12/12/2022 | Africa / Morocco / Casablanca | Human | unknown | Male | 35 | unknown | unknown | unknown | BE.1 | GRA |
| hCoV-19/Morocco/IPM20433198/2022 | EPI_ISL_16649900 | 12/12/2022 | Africa / Morocco / Casablanca | Human | unknown | Female | 52 | unknown | unknown | unknown | BQ.1.1 | GRA |
| hCoV-19/Morocco/IPM20433177/2022 | EPI_ISL_16649899 | 12/12/2022 | Africa / Morocco / Casablanca | Human | unknown | Male | 51 | unknown | unknown | unknown | BQ.1 | GRA |
| hCoV-19/Morocco/IPM20433167/2022 | EPI_ISL_16649898 | 09/12/2022 | Africa / Morocco / Casablanca | Human | unknown | Female | 30 | unknown | unknown | unknown | BA.2 | GRA |
| hCoV-19/Morocco/IPM20433157/2022 | EPI_ISL_16649897 | 09/12/2022 | Africa / Morocco / Casablanca | Human | unknown | Male | 60 | unknown | unknown | unknown | BQ.1 | GRA |
| hCoV-19/Morocco/IPM20433131/2022 | EPI_ISL_16649896 | 07/12/2022 | Africa / Morocco / Casablanca | Human | unknown | Male | 27 | unknown | unknown | unknown | BA.4 | GRA |
| hCoV-19/Morocco/IPM20433050/2022 | EPI_ISL_16649895 | 07/12/2022 | Africa / Morocco / Casablanca | Human | unknown | Male | 48 | unknown | unknown | unknown | BE.1.1 | GRA |
| hCoV-19/Morocco/IPM20435994/2023 | EPI_ISL_17797611 | 28/04/2023 | Africa / Morocco / Casablanca | Human | unknown | Male | 54 | unknown | unknown | unknown | XBB.1.5 | GRA |
| hCoV-19/Morocco/IPM20435975/2023 | EPI_ISL_17797601 | 21/04/2023 | Africa / Morocco / Casablanca | Human | unknown | Male | 38 | unknown | unknown | unknown | XBB.1.5 | GRA |
| hCoV-19/Morocco/IPM20435973/2023 | EPI_ISL_17797600 | 17/04/2023 | Africa / Morocco / Casablanca | Human | unknown | Male | 51 | unknown | unknown | unknown | XBB.1.5 | GRA |
| hCoV-19/Morocco/IPM20435978/2023 | EPI_ISL_17797602 | 25/04/2023 | Africa / Morocco / Casablanca | Human | unknown | Female | 64 | unknown | unknown | unknown | XBB.1.9.1 | GRA |
| hCoV-19/Morocco/117/2023 | EPI_ISL_17685328 | 01/05/2023 | Africa / Morocco / Rabat | Human | unknown | Male | 43 | Released | unknown | unknown | EF.1 | GRA |
| hCoV-19/Morocco/IPM20435955/2023 | EPI_ISL_17650231 | 04/04/2023 | Africa / Morocco / Casablanca | Human | unknown | unknown | unknown | unknown | unknown | unknown | XBB.1.5 | GRA |
| hCoV-19/Morocco/IPM20435954/2023 | EPI_ISL_17650230 | 04/04/2023 | Africa / Morocco / Casablanca | Human | unknown | unknown | unknown | unknown | unknown | unknown | FL.2 | GRA |
| hCoV-19/Morocco/IPM20435953/2023 | EPI_ISL_17650229 | 03/04/2023 | Africa / Morocco / Casablanca | Human | unknown | unknown | unknown | unknown | unknown | unknown | XBB.1.5 | GRA |
| hCoV-19/Morocco/IPM20435948/2023 | EPI_ISL_17650228 | 30/03/2023 | Africa / Morocco / Meknes | Human | unknown | Female | 27 | unknown | unknown | unknown | XBB.2.4 | GRA |
| hCoV-19/Morocco/IPM20435947/2023 | EPI_ISL_17650227 | 30/03/2023 | Africa / Morocco / Meknes | Human | unknown | Male | 23 | unknown | unknown | unknown | XBB.1.5.23 | GRA |
| hCoV-19/Morocco/IPM20435937/2023 | EPI_ISL_17650225 | 22/03/2023 | Africa / Morocco / Casablanca | Human | unknown | unknown | unknown | unknown | unknown | unknown | XBB.1.5 | GRA |
| hCoV-19/Morocco/IPM20435890/2023 | EPI_ISL_17650224 | 14/03/2023 | Africa / Morocco / Casablanca | Human | unknown | unknown | unknown | unknown | unknown | unknown | FL.5 | GRA |
| hCoV-19/Morocco/IPM20435884/2023 | EPI_ISL_17650223 | 14/03/2023 | Africa / Morocco / Casablanca | Human | unknown | unknown | unknown | unknown | unknown | unknown | XBB.1.5 | GRA |
| hCoV-19/Morocco/IPM20435883/2023 | EPI_ISL_17650222 | 13/03/2023 | Africa / Morocco / Casablanca | Human | unknown | unknown | unknown | unknown | unknown | unknown | XBB.1.5 | GRA |
| hCoV-19/Morocco/IPM20435768/2023 | EPI_ISL_17650221 | 14/03/2023 | Africa / Morocco / Casablanca | Human | unknown | Female | 19 | unknown | unknown | unknown | XBB.1.5 | GRA |
| hCoV-19/Morocco/IPM20435574/2023 | EPI_ISL_17650220 | 07/03/2023 | Africa / Morocco / Casablanca | Human | unknown | Male | 51 | unknown | unknown | unknown | XBB.1.5 | GRA |
| hCoV-19/Morocco/IPM20435499/2023 | EPI_ISL_17650219 | 27/02/2023 | Africa / Morocco / Casablanca | Human | unknown | Female | 33 | unknown | unknown | unknown | FL.10 | GRA |
| hCoV-19/Morocco/IPM20435480/2023 | EPI_ISL_17650218 | 23/02/2023 | Africa / Morocco / Casablanca | Human | unknown | Male | 41 | unknown | unknown | unknown | FL.10 | GRA |
| hCoV-19/Morocco/IPM20435479/2023 | EPI_ISL_17650217 | 23/02/2023 | Africa / Morocco / Casablanca | Human | unknown | Female | 37 | unknown | unknown | unknown | BA.2.56 | GRA |
| hCoV-19/Morocco/IPM20435471/2023 | EPI_ISL_17650215 | 23/02/2023 | Africa / Morocco / Casablanca | Human | unknown | Female | 25 | unknown | unknown | unknown | BQ.1.1.47 | GRA |
| hCoV-19/Morocco/IPM20435449/2023 | EPI_ISL_17650214 | 22/02/2023 | Africa / Morocco / Casablanca | Human | unknown | Male | 24 | unknown | unknown | unknown | BA.2 | GRA |
| hCoV-19/Morocco/IPM20435287/2023 | EPI_ISL_17650213 | 13/02/2023 | Africa / Morocco / Casablanca | Human | unknown | Male | 63 | unknown | unknown | unknown | BQ.1 | GRA |
| hCoV-19/Morocco/IPM20435284/2023 | EPI_ISL_17650212 | 10/02/2023 | Africa / Morocco / Casablanca | Human | unknown | Male | 27 | unknown | unknown | unknown | BA.2 | GRA |
| hCoV-19/Morocco/IPM20435273/2023 | EPI_ISL_17650211 | 09/02/2023 | Africa / Morocco / Casablanca | Human | unknown | Male | 24 | unknown | unknown | unknown | BA.2 | GRA |
| hCoV-19/Morocco/IPM20435266/2023 | EPI_ISL_17650210 | 09/02/2023 | Africa / Morocco / Casablanca | Human | unknown | Male | unknown | unknown | unknown | unknown | XBB.1.5.24 | GRA |
| hCoV-19/Morocco/IPM20435233/2023 | EPI_ISL_17650209 | 08/02/2023 | Africa / Morocco / Casablanca | Human | unknown | Female | 30 | unknown | unknown | unknown | BA.2 | GRA |
| hCoV-19/Morocco/IPM20435164/2023 | EPI_ISL_17650208 | 06/02/2023 | Africa / Morocco / Casablanca | Human | unknown | Female | 28 | unknown | unknown | unknown | BA.2 | GRA |
| hCoV-19/Morocco/IPM20435110/2023 | EPI_ISL_17650207 | 31/01/2023 | Africa / Morocco / Casablanca | Human | unknown | Male | 24 | unknown | unknown | unknown | XBB | GRA |
| hCoV-19/Morocco/IPM20434995/2023 | EPI_ISL_17650206 | 27/01/2023 | Africa / Morocco / Casablanca | Human | unknown | Male | 41 | unknown | unknown | unknown | XBB.1.5.23 | GRA |
| hCoV-19/Morocco/IPM20434916/2023 | EPI_ISL_17650205 | 26/01/2023 | Africa / Morocco / Casablanca | Human | unknown | Male | 39 | unknown | unknown | unknown | BA.2 | GRA |
| hCoV-19/Morocco/IPM20434868/2023 | EPI_ISL_17650204 | 25/01/2023 | Africa / Morocco / Casablanca | Human | unknown | Male | 29 | unknown | unknown | unknown | BQ.1.1 | GRA |
| hCoV-19/Morocco/IPM20434786/2023 | EPI_ISL_17650203 | 20/01/2023 | Africa / Morocco / Casablanca | Human | unknown | Male | 30 | unknown | unknown | unknown | XBB.1.5 | GRA |
| hCoV-19/Morocco/IPM20434700/2023 | EPI_ISL_17650202 | 18/01/2023 | Africa / Morocco / Casablanca | Human | unknown | Male | 43 | unknown | unknown | unknown | BQ.1.1.58 | GRA |
| hCoV-19/Morocco/IPM20434585/2023 | EPI_ISL_17650201 | 16/01/2023 | Africa / Morocco / Casablanca | Human | unknown | Male | 24 | unknown | unknown | unknown | BA.2 | GRA |
| hCoV-19/Morocco/IPM20434572/2023 | EPI_ISL_17650200 | 16/01/2023 | Africa / Morocco / Casablanca | Human | unknown | Male | 34 | unknown | unknown | unknown | BA.2 | GRA |
| hCoV-19/Morocco/IPM20434451/2023 | EPI_ISL_17650199 | 10/01/2023 | Africa / Morocco / Casablanca | Human | unknown | Female | 39 | unknown | unknown | unknown | BA.5 | GRA |
| hCoV-19/Morocco/IPM20434440/2023 | EPI_ISL_17650198 | 10/01/2023 | Africa / Morocco / Casablanca | Human | unknown | Male | 46 | unknown | unknown | unknown | BA.2 | GRA |
| hCoV-19/Morocco/IPM20434420/2023 | EPI_ISL_17650197 | 09/01/2023 | Africa / Morocco / Casablanca | Human | unknown | Female | 27 | unknown | unknown | unknown | BQ.1.1 | GRA |
| hCoV-19/Morocco/IPM20434399/2023 | EPI_ISL_17650196 | 09/01/2023 | Africa / Morocco / Casablanca | Human | unknown | Male | 50 | unknown | unknown | unknown | BA.5.2 | GR |
| hCoV-19/Morocco/IPM20163761/2020 | EPI_ISL_17650195 | 29/08/2020 | Africa / Morocco / Casablanca | Human | unknown | Female | unknown | unknown | unknown | unknown | B.1 | GH |
| hCoV-19/Morocco/IPM20157410/2020 | EPI_ISL_17650194 | 23/08/2020 | Africa / Morocco / Casablanca | Human | unknown | Female | unknown | unknown | unknown | unknown | B.1.1 | GR |
| hCoV-19/Morocco/IPM20155760/2020 | EPI_ISL_17650193 | 21/08/2020 | Africa / Morocco / Casablanca | Human | unknown | Female | unknown | unknown | unknown | unknown | B.1 | G |
| hCoV-19/Morocco/IPM20149351/2020 | EPI_ISL_17650190 | 15/08/2020 | Africa / Morocco / Casablanca | Human | unknown | Female | unknown | unknown | unknown | unknown | B.1 | G |
| hCoV-19/Morocco/IPM20148318/2020 | EPI_ISL_17650189 | 14/08/2020 | Africa / Morocco / Casablanca | Human | unknown | Male | unknown | unknown | unknown | unknown | B.1.1 | GR |
| hCoV-19/Morocco/IPM20147770/2020 | EPI_ISL_17650188 | 13/08/2020 | Africa / Morocco / Casablanca | Human | unknown | Female | unknown | unknown | unknown | unknown | B.1 | G |
| hCoV-19/Morocco/IPM20145414/2020 | EPI_ISL_17650186 | 10/08/2020 | Africa / Morocco / Casablanca | Human | unknown | Female | unknown | unknown | unknown | unknown | B.1.1 | GR |
| hCoV-19/Morocco/IPM20140690/2020 | EPI_ISL_17650185 | 06/08/2020 | Africa / Morocco / Casablanca | Human | unknown | Female | unknown | unknown | unknown | unknown | B.1 | G |
| hCoV-19/Morocco/IPM20137668/2020 | EPI_ISL_17650183 | 01/08/2020 | Africa / Morocco / Casablanca | Human | unknown | Male | unknown | unknown | unknown | unknown | B.1 | G |
| hCoV-19/Morocco/IPM20136508/2020 | EPI_ISL_17650182 | 29/07/2020 | Africa / Morocco / Casablanca | Human | unknown | Female | unknown | unknown | unknown | unknown | B.1 | G |
| hCoV-19/Morocco/IPM20125708/2020 | EPI_ISL_17650180 | 25/07/2020 | Africa / Morocco / Casablanca | Human | unknown | Female | unknown | unknown | unknown | unknown | B.1.1 | GR |
| hCoV-19/Morocco/IPM20118337/2020 | EPI_ISL_17650177 | 20/07/2020 | Africa / Morocco / Casablanca | Human | unknown | Female | unknown | unknown | unknown | unknown | B.1 | G |
| hCoV-19/Morocco/IPM20116215/2020 | EPI_ISL_17650176 | 17/07/2020 | Africa / Morocco / Casablanca | Human | unknown | Male | unknown | unknown | unknown | unknown | B.1 | G |
| hCoV-19/Morocco/IPM2076824/2020 | EPI_ISL_17650169 | 25/06/2020 | Africa / Morocco / Casablanca | Human | unknown | Female | 36 | unknown | unknown | unknown | B.1 | G |
| hCoV-19/Morocco/IPM2065693/2020 | EPI_ISL_17650168 | 19/06/2020 | Africa / Morocco / Casablanca | Human | unknown | Male | unknown | unknown | unknown | unknown | B.1 | G |
| hCoV-19/Morocco/IPM2057245/2020 | EPI_ISL_17650167 | 15/06/2020 | Africa / Morocco / Casablanca | Human | unknown | Female | unknown | unknown | unknown | unknown | B.1 | G |
| hCoV-19/Morocco/IPM2052745/2020 | EPI_ISL_17650166 | 12/06/2020 | Africa / Morocco / Casablanca | Human | unknown | Male | unknown | unknown | unknown | unknown | B.1 | G |
| hCoV-19/Morocco/IPM2048904/2020 | EPI_ISL_17650165 | 10/06/2020 | Africa / Morocco / Casablanca | Human | unknown | Male | unknown | unknown | unknown | unknown | B.1 | G |
| hCoV-19/Morocco/IPM2039146/2020 | EPI_ISL_17650164 | 04/06/2020 | Africa / Morocco / Casablanca | Human | unknown | Male | 42 | unknown | unknown | unknown | B.1 | G |
| hCoV-19/Morocco/IPM2034941/2020 | EPI_ISL_17650162 | 28/05/2020 | Africa / Morocco / Casablanca | Human | unknown | Male | unknown | unknown | unknown | unknown | B.1.1 | GR |
| hCoV-19/Morocco/IPM2033123/2020 | EPI_ISL_17650161 | 26/05/2020 | Africa / Morocco / Casablanca | Human | unknown | Female | unknown | unknown | unknown | unknown | B.1 | G |
| hCoV-19/Morocco/IPM2032545/2020 | EPI_ISL_17650160 | 25/05/2020 | Africa / Morocco / Casablanca | Human | unknown | Female | unknown | unknown | unknown | unknown | B.1 | G |
| hCoV-19/Morocco/IPM2023832/2020 | EPI_ISL_17650157 | 14/05/2020 | Africa / Morocco / Casablanca | Human | unknown | Female | 34 | unknown | unknown | unknown | B.1 | G |
| hCoV-19/Morocco/IPM2019560/2020 | EPI_ISL_17650156 | 08/05/2020 | Africa / Morocco / Casablanca | Human | unknown | Female | 24 | unknown | unknown | unknown | B.1.1 | GR |
| hCoV-19/Morocco/IPM2015184/2020 | EPI_ISL_17650155 | 03/05/2020 | Africa / Morocco / Casablanca | Human | unknown | Male | 62 | unknown | unknown | unknown | B.1.1 | GR |
| hCoV-19/Morocco/IPM2012180/2020 | EPI_ISL_17650154 | 28/04/2020 | Africa / Morocco / Casablanca | Human | unknown | Female | 85 | unknown | unknown | unknown | B.1.1 | GR |
| hCoV-19/Morocco/IPM2010760/2020 | EPI_ISL_17650152 | 25/04/2020 | Africa / Morocco / Casablanca | Human | unknown | Male | unknown | unknown | unknown | unknown | B.1.1 | GR |
| hCoV-19/Morocco/IPM2009757/2020 | EPI_ISL_17650151 | 24/04/2020 | Africa / Morocco / Casablanca | Human | unknown | Male | 25 | unknown | unknown | unknown | B.1.1 | GR |
| hCoV-19/Morocco/IPM2009377/2020 | EPI_ISL_17650150 | 23/04/2020 | Africa / Morocco / Casablanca | Human | unknown | Male | unknown | unknown | unknown | unknown | B.1.1 | GR |
| hCoV-19/Morocco/IPM2008255/2020 | EPI_ISL_17650149 | 21/04/2020 | Africa / Morocco / Casablanca | Human | unknown | Female | 43 | unknown | unknown | unknown | B.1.1 | GR |
| hCoV-19/Morocco/IPM2008205/2020 | EPI_ISL_17650148 | 21/04/2020 | Africa / Morocco / Casablanca | Human | unknown | Female | 50 | unknown | unknown | unknown | B.1.1 | GR |
| hCoV-19/Morocco/IPM2007621/2020 | EPI_ISL_17650147 | 19/04/2020 | Africa / Morocco / Casablanca | Human | unknown | Female | 27 | unknown | unknown | unknown | B.1.1 | GR |
| hCoV-19/Morocco/IPM2005610/2020 | EPI_ISL_17650144 | 15/04/2020 | Africa / Morocco / Casablanca | Human | unknown | Female | unknown | unknown | unknown | unknown | B.1.1 | GR |
| hCoV-19/Morocco/IPM2005500/2020 | EPI_ISL_17650143 | 15/04/2020 | Africa / Morocco / Casablanca | Human | unknown | Male | 3 | unknown | unknown | unknown | B.1.1 | GR |
| hCoV-19/Morocco/IPM2005420/2020 | EPI_ISL_17650142 | 14/04/2020 | Africa / Morocco / Casablanca | Human | unknown | Female | unknown | unknown | unknown | unknown | B.1.1 | GR |
| hCoV-19/Morocco/IPM2004570/2020 | EPI_ISL_17650140 | 12/04/2020 | Africa / Morocco / Casablanca | Human | unknown | Male | 21 | unknown | unknown | unknown | B.1 | GH |
| hCoV-19/Morocco/IPM2004208/2020 | EPI_ISL_17650139 | 10/04/2020 | Africa / Morocco / Casablanca | Human | unknown | Male | 52 | unknown | unknown | unknown | B.1.356 | GH |
| hCoV-19/Morocco/IPM2003201/2020 | EPI_ISL_17650137 | 06/04/2020 | Africa / Morocco / Casablanca | Human | unknown | Male | 56 | unknown | unknown | unknown | B.1.1 | GR |
| hCoV-19/Morocco/IPM2003100/2020 | EPI_ISL_17650136 | 06/04/2020 | Africa / Morocco / Casablanca | Human | unknown | Female | 59 | unknown | unknown | unknown | B.1.1 | GR |
| hCoV-19/Morocco/IPM2000600/2020 | EPI_ISL_17650132 | 25/03/2020 | Africa / Morocco / Casablanca | Human | unknown | Male | 45 | unknown | unknown | unknown | B.1.1 | GR |
| hCoV-19/Morocco/IPM2000592/2020 | EPI_ISL_17650131 | 25/03/2020 | Africa / Morocco / Casablanca | Human | unknown | Female | 72 | unknown | unknown | unknown | B.1.1 | GR |
| hCoV-19/Morocco/IPM2000471/2020 | EPI_ISL_17650130 | 24/03/2020 | Africa / Morocco / Casablanca | Human | unknown | Female | 72 | unknown | unknown | unknown | B.1 | GH |
| hCoV-19/Morocco/IPM2000441/2020 | EPI_ISL_17650129 | 24/03/2020 | Africa / Morocco / Casablanca | Human | unknown | Male | 75 | unknown | unknown | unknown | B.1.1 | GR |
| hCoV-19/Morocco/HMIMV_279CC/2020 | EPI_ISL_971451 | 01/07/2020 | Africa / Morocco / Rabat | Human | unknown | Female | 48 | Released | unknown | unknown | B.1 | GH |
| hCoV-19/Morocco/FMP604/2022 | EPI_ISL_17383428 | 24/11/2022 | Africa / Morocco / Rabat | Human | unknown | Male | 58 | unknown | unknown | unknown | XBB | GRA |
| hCoV-19/Morocco/FMP578/2022 | EPI_ISL_17304059 | 12/10/2022 | Africa / Morocco / Sale | Human | unknown | Male | 1 | Live | unknown | unknown | XBB.6 | GRA |
| hCoV-19/Morocco/FMP577/2022 | EPI_ISL_17304047 | 11/10/2022 | Africa / Morocco / Sale | Human | unknown | Male | 82 | Live | unknown | unknown | XBB.6 | GRA |
| hCoV-19/Morocco/FMP565/2022 | EPI_ISL_17304044 | 05/10/2022 | Africa / Morocco / Sale | Human | unknown | Female | 38 | Live | unknown | unknown | XBB.6 | GRA |
| hCoV-19/Morocco/FMP-64/2020 | EPI_ISL_778870 | 2020 | Africa / Morocco | Human | unknown | unknown | unknown | unknown | unknown | unknown | B.1.1 | GR |
| hCoV-19/Morocco/MA-12-HMIMV-P3-Jouh/2022 | EPI_ISL_17179367 | 02/12/2022 | Africa / Morocco / Rabat | unknown | unknown | unknown | unknown | unknown | unknown | unknown | BA.5.2.20 | GRA |
| hCoV-19/Morocco/FMP-63/2020 | EPI_ISL_775265 | 2020-11 | Africa / Morocco | Human | unknown | unknown | unknown | unknown | unknown | unknown | B.1 | G |
| hCoV-19/Morocco/FMP-62/2020 | EPI_ISL_775264 | 2020-11 | Africa / Morocco | Human | unknown | unknown | unknown | unknown | unknown | unknown | B.1 | G |
| hCoV-19/Morocco/FMP-61/2020 | EPI_ISL_775263 | 21/12/2020 | Africa / Morocco / Rabat | Human | unknown | Male | 33 | unknown | unknown | unknown | B.1.36 | GH |
| hCoV-19/Morocco/FMP-60/2020 | EPI_ISL_775262 | 21/12/2020 | Africa / Morocco / Rabat | Human | unknown | Male | 30 | unknown | unknown | unknown | B.1.416 | G |
| hCoV-19/Morocco/FMP-59/2020 | EPI_ISL_775260 | 21/12/2020 | Africa / Morocco / Temara | Human | unknown | Female | 45 | unknown | unknown | unknown | B.1.1 | GR |
| hCoV-19/Morocco/FMP-58/2020 | EPI_ISL_775258 | 21/12/2020 | Africa / Morocco / Temara | Human | unknown | Female | 17 | unknown | unknown | unknown | B.1 | G |
| hCoV-19/Morocco/FMP-57/2020 | EPI_ISL_775256 | 21/12/2020 | Africa / Morocco / Temara | Human | unknown | Female | 29 | unknown | unknown | unknown | B.1.177 | GV |
| hCoV-19/Morocco/FMP-56/2020 | EPI_ISL_775255 | 2020 | Africa / Morocco / Temara | Human | unknown | Male | 39 | unknown | unknown | unknown | B.1 | G |
| hCoV-19/Morocco/FMP-55/2020 | EPI_ISL_775254 | 2020-12 | Africa / Morocco / Ain Aouda | Human | unknown | Female | 71 | unknown | unknown | unknown | B.1 | G |
| hCoV-19/Morocco/FMP-53/2020 | EPI_ISL_775250 | 2020-11 | Africa / Morocco / Sale | Human | unknown | Female | 13 | unknown | unknown | unknown | B.1 | GH |
| hCoV-19/Morocco/FMP-52/2020 | EPI_ISL_775249 | 2020-12 | Africa / Morocco / Rabat | Human | unknown | Male | 32 | unknown | unknown | unknown | B.1.1 | GR |
| hCoV-19/Morocco/FMP-51/2020 | EPI_ISL_775247 | 2020-12 | Africa / Morocco / Temara | Human | unknown | Male | 31 | unknown | unknown | unknown | B.1.1 | GR |
| hCoV-19/Morocco/FMP-50/2020 | EPI_ISL_775226 | 2020-12 | Africa / Morocco / Rabat | Human | unknown | Female | 32 | unknown | unknown | unknown | B.1.160 | GH |
| hCoV-19/Morocco/FMP-49/2020 | EPI_ISL_775225 | 14/12/2020 | Africa / Morocco / Temara | Human | unknown | Female | 74 | unknown | unknown | unknown | B.1 | G |
| hCoV-19/Morocco/FMP-48/2020 | EPI_ISL_775224 | 2020-12 | Africa / Morocco / Sale | Human | unknown | Male | 29 | unknown | unknown | unknown | B.1.1 | GR |
| hCoV-19/Morocco/FMP-46/2020 | EPI_ISL_775222 | 16/12/2020 | Africa / Morocco / Sale | Human | unknown | Female | 53 | unknown | unknown | unknown | B.1 | G |
| hCoV-19/Morocco/FMP-45/2020 | EPI_ISL_775221 | 15/12/2020 | Africa / Morocco / Temara | Human | unknown | Female | 69 | unknown | unknown | unknown | B.1.160 | GH |
| hCoV-19/Morocco/FMP-44/2020 | EPI_ISL_775220 | 15/12/2020 | Africa / Morocco / Temara | Human | unknown | Male | 28 | unknown | unknown | unknown | B.1.1 | GR |
| hCoV-19/Morocco/FMP-43/2020 | EPI_ISL_775219 | 14/12/2020 | Africa / Morocco / Temara | Human | unknown | Female | 69 | unknown | unknown | unknown | B.1 | G |
| hCoV-19/Morocco/MA-05-HMIMV-P3/2022 | EPI_ISL_17167251 | 05/05/2022 | Africa / Morocco / Rabat | unknown | unknown | unknown | unknown | unknown | unknown | unknown | AY.33 | GK |
| hCoV-19/Morocco/LDB-01/2020 | EPI_ISL_723469 | 2020-11 | Africa / Morocco / Rabat | Human | unknown | unknown | unknown | unknown | unknown | unknown | B.1.177 | GV |
| hCoV-19/Morocco/FMP-42/2020 | EPI_ISL_769863 | 14/12/2020 | Africa / Morocco / Casablanca | Human | unknown | Male | 43 | unknown | unknown | unknown | B.1 | G |
| hCoV-19/Morocco/HMIMV-P3-ALV/2022 | EPI_ISL_17155879 | 27/03/2022 | Africa / Morocco / Rabat | unknown | unknown | unknown | unknown | unknown | unknown | unknown | B.1.1.7 | GR |
| hCoV-19/Morocco/MN908947_259/2022 | EPI_ISL_17154594 | 14/05/2022 | Africa / Morocco / Rabat | Human | Baseline surveillance | Male | 43 | unknown | unknown | Baseline surveillance | BA.2 | GRA |
| hCoV-19/Morocco/FMP-41/2020 | EPI_ISL_768840 | 17/12/2020 | Africa / Morocco / Rabat | Human | unknown | Male | 67 | unknown | unknown | unknown | B.1.177 | GV |
| hCoV-19/Morocco/FMP-39/2020 | EPI_ISL_768838 | 2020 | Africa / Morocco | Human | unknown | unknown | unknown | unknown | unknown | unknown | B.1.597 | GH |
| hCoV-19/Morocco/FMP-38/2020 | EPI_ISL_768837 | 2020-12 | Africa / Morocco / Temara | Human | unknown | Female | 55 | unknown | unknown | unknown | B.1.1.118 | GR |
| hCoV-19/Morocco/FMP-37/2020 | EPI_ISL_768836 | 2020-12 | Africa / Morocco / Rabat | Human | unknown | Female | 10 | unknown | unknown | unknown | B.1 | G |
| hCoV-19/Morocco/FMP-36/2020 | EPI_ISL_768835 | 2020-12 | Africa / Morocco | Human | unknown | Female | 44 | unknown | unknown | unknown | B.1 | GH |
| hCoV-19/Morocco/FMP-35/2020 | EPI_ISL_768834 | 2020-12 | Africa / Morocco / Temara | Human | unknown | Female | unknown | unknown | unknown | unknown | B.1.1 | GR |
| hCoV-19/Morocco/FMP-33/2020 | EPI_ISL_768831 | 2020-12 | Africa / Morocco / SalÃ© | Human | unknown | Female | 38 | unknown | unknown | unknown | B.1.597 | GH |
| hCoV-19/Morocco/CNRST-0000002/2022 | EPI_ISL_17082268 | 10/06/2022 | Africa / Morocco | Human | unknown | unknown | unknown | unknown | unknown | unknown | BA.5.2.20 | GRA |
| hCoV-19/Morocco/HMIMV-07/2022 | EPI_ISL_17082267 | 02/09/2022 | Africa / Morocco | Human | unknown | unknown | unknown | unknown | unknown | unknown | BA.5.2.20 | GRA |
| hCoV-19/Morocco/FMP-32/2020 | EPI_ISL_728367 | 2020-12 | Africa / Morocco | Human | unknown | Female | unknown | unknown | unknown | unknown | B.1.1 | GR |
| hCoV-19/Morocco/FMP-29/2020 | EPI_ISL_728360 | 2020-12 | Africa / Morocco / Temara | Human | unknown | Female | 9 months | unknown | unknown | unknown | B.1.1 | GR |
| hCoV-19/Morocco/FMP-28/2020 | EPI_ISL_728359 | 2020-12 | Africa / Morocco / Rabat | Human | unknown | Male | 45 | unknown | unknown | unknown | B.1.597 | GH |
| hCoV-19/Morocco/FMP-26/2020 | EPI_ISL_728353 | 2020-12 | Africa / Morocco / Harhoura | Human | unknown | Male | 64 | unknown | unknown | unknown | B.1.258 | G |
| hCoV-19/Morocco/FMP-24/2020 | EPI_ISL_728349 | 2020-12 | Africa / Morocco / Rabat | Human | unknown | Male | 9 | unknown | unknown | unknown | B.1.597 | GH |
| hCoV-19/Morocco/FMP-21/2020 | EPI_ISL_728342 | 2020-12 | Africa / Morocco / Temara | Human | unknown | Male | 17 | unknown | unknown | unknown | B.1.1 | GR |
| hCoV-19/Morocco/FMP-19/2020 | EPI_ISL_728339 | 2020-12 | Africa / Morocco / Temara | Human | unknown | Female | 27 | unknown | unknown | unknown | B.1.597 | GH |
| hCoV-19/Morocco/FMP-18/2020 | EPI_ISL_728336 | 2020-12 | Africa / Morocco / Rabat | Human | unknown | Male | 62 | unknown | unknown | unknown | B.1.1 | GR |
| hCoV-19/Morocco/FMP-17/2020 | EPI_ISL_728334 | 2020-12 | Africa / Morocco / Rabat | Human | unknown | Male | 54 | unknown | unknown | unknown | B.1.1.219 | GR |
| hCoV-19/Morocco/FMP-16/2020 | EPI_ISL_728332 | 2020-11 | Africa / Morocco / Rabat | Human | unknown | unknown | unknown | unknown | unknown | unknown | B.1.597 | GH |
| hCoV-19/Morocco/FMP-15/2020 | EPI_ISL_728322 | 2020-11 | Africa / Morocco / Rabat | Human | unknown | unknown | unknown | unknown | unknown | unknown | B.1.1 | GR |
| hCoV-19/Morocco/FMP-14/2020 | EPI_ISL_728301 | 2020-11 | Africa / Morocco / Rabat | Human | unknown | unknown | unknown | unknown | unknown | unknown | B.1.1 | GR |
| hCoV-19/Morocco/FMP-12/2020 | EPI_ISL_728295 | 2020-11 | Africa / Morocco / Rabat | Human | unknown | unknown | unknown | unknown | unknown | unknown | B.1.1 | GR |
| hCoV-19/Morocco/FMP-10/2020 | EPI_ISL_728290 | 2020-11 | Africa / Morocco / Rabat | Human | unknown | unknown | unknown | unknown | unknown | unknown | B.1 | G |
| hCoV-19/Morocco/FMP-9/2020 | EPI_ISL_728289 | 2020-11 | Africa / Morocco / Rabat | Human | unknown | unknown | unknown | unknown | unknown | unknown | B.1 | G |
| hCoV-19/Morocco/FMP-7/2020 | EPI_ISL_728276 | 2020-11 | Africa / Morocco / Rabat | Human | unknown | unknown | unknown | unknown | unknown | unknown | B.1.597 | GH |
| hCoV-19/Morocco/FMP-6/2020 | EPI_ISL_728275 | 2020-11 | Africa / Morocco / Rabat | Human | unknown | unknown | unknown | unknown | unknown | unknown | B.1.597 | GH |
| hCoV-19/Morocco/FMP-5/2020 | EPI_ISL_728274 | 2020-11 | Africa / Morocco / Rabat | Human | unknown | unknown | unknown | unknown | unknown | unknown | B.1.1 | GR |
| hCoV-19/Morocco/FMP-4/2020 | EPI_ISL_728273 | 2020-11 | Africa / Morocco / Rabat | Human | unknown | unknown | unknown | unknown | unknown | unknown | B.1 | G |
| hCoV-19/Morocco/FMP-3/2020 | EPI_ISL_728272 | 2020-11 | Africa / Morocco / Rabat | Human | unknown | unknown | unknown | unknown | unknown | unknown | B.1 | G |
| hCoV-19/Morocco/RMPS-25/2020 | EPI_ISL_728239 | 2020 | Africa / Morocco | Human | unknown | unknown | unknown | unknown | unknown | unknown | B.1 | GH |
| hCoV-19/Morocco/RMPS-23/2020 | EPI_ISL_728237 | 01/04/2020 | Africa / Morocco | Human | unknown | Male | unknown | unknown | unknown | unknown | B.1 | G |
| hCoV-19/Morocco/RMPS-22/2020 | EPI_ISL_728236 | 01/04/2020 | Africa / Morocco | Human | unknown | Female | unknown | unknown | unknown | unknown | B.1.1 | GR |
| hCoV-19/Morocco/RMPS-21/2020 | EPI_ISL_728235 | 01/04/2020 | Africa / Morocco | Human | unknown | Female | unknown | unknown | unknown | unknown | B.1 | G |
| hCoV-19/Morocco/RMPS-17/2020 | EPI_ISL_728221 | 31/03/2020 | Africa / Morocco | Human | unknown | Female | unknown | unknown | unknown | unknown | Unassigned | G |
| hCoV-19/Morocco/LDB-02/2020 | EPI_ISL_728219 | 2020-11 | Africa / Morocco / Rabat | Human | unknown | unknown | unknown | unknown | unknown | unknown | B.1 | GH |
| hCoV-19/Morocco/IPM20433027/2022 | EPI_ISL_16259329 | 06/12/2022 | Africa / Morocco / Casablanca | Human | unknown | Male | 74 | unknown | unknown | unknown | BQ.1 | GRA |
| hCoV-19/Morocco/IPM20433010/2022 | EPI_ISL_16259328 | 05/12/2022 | Africa / Morocco / Casablanca | Human | unknown | Male | 48 | unknown | unknown | unknown | BQ.1.1 | GRA |
| hCoV-19/Morocco/IPM20432986/2022 | EPI_ISL_16259327 | 05/12/2022 | Africa / Morocco / Casablanca | Human | unknown | Male | 57 | unknown | unknown | unknown | BQ.1 | GRA |
| hCoV-19/Morocco/IPM20432983/2022 | EPI_ISL_16259326 | 05/12/2022 | Africa / Morocco / Casablanca | Human | unknown | Female | 37 | unknown | unknown | unknown | BQ.1 | GRA |
| hCoV-19/Morocco/IPM20432981/2022 | EPI_ISL_16259325 | 05/12/2022 | Africa / Morocco / Casablanca | Human | unknown | Female | 51 | unknown | unknown | unknown | BQ.1.13.1 | GRA |
| hCoV-19/Morocco/IPM20432979/2022 | EPI_ISL_16259324 | 05/12/2022 | Africa / Morocco / Casablanca | Human | unknown | Female | 51 | unknown | unknown | unknown | BQ.1 | GRA |
| hCoV-19/Morocco/IPM20432971/2022 | EPI_ISL_16259323 | 02/12/2022 | Africa / Morocco / Casablanca | Human | unknown | Male | 1 | unknown | unknown | unknown | BQ.1 | GRA |
| hCoV-19/Morocco/IPM20432970/2022 | EPI_ISL_16259322 | 02/12/2022 | Africa / Morocco / Casablanca | Human | unknown | Female | 36 | unknown | unknown | unknown | BQ.1.1 | GRA |
| hCoV-19/Morocco/IPM20432967/2022 | EPI_ISL_16259321 | 02/12/2022 | Africa / Morocco / Casablanca | Human | unknown | Male | 41 | unknown | unknown | unknown | BQ.1.1 | GRA |
| hCoV-19/Morocco/IPM20432945/2022 | EPI_ISL_16259320 | 01/12/2022 | Africa / Morocco / Casablanca | Human | unknown | Female | 64 | unknown | unknown | unknown | BQ.1.1 | GRA |
| hCoV-19/Morocco/IPM20432934/2022 | EPI_ISL_16259319 | 01/12/2022 | Africa / Morocco / Casablanca | Human | unknown | Male | 70 | unknown | unknown | unknown | BA.2 | GRA |
| hCoV-19/Morocco/IPM20432925/2022 | EPI_ISL_16259318 | 01/12/2022 | Africa / Morocco / Casablanca | Human | unknown | Male | 29 | unknown | unknown | unknown | BQ.1 | GRA |
| hCoV-19/Morocco/IPM20432838/2022 | EPI_ISL_16259317 | 30/11/2022 | Africa / Morocco / Casablanca | Human | unknown | Female | 22 | unknown | unknown | unknown | CJ.1.1 | GRA |
| hCoV-19/Morocco/IPM20432826/2022 | EPI_ISL_16259316 | 29/11/2022 | Africa / Morocco / Casablanca | Human | unknown | Male | 34 | unknown | unknown | unknown | BQ.1.1 | GRA |
| hCoV-19/Morocco/IPM20432822/2022 | EPI_ISL_16259315 | 29/11/2022 | Africa / Morocco / Casablanca | Human | unknown | Female | 53 | unknown | unknown | unknown | BQ.1.1 | GRA |
| hCoV-19/Morocco/IPM20432804/2022 | EPI_ISL_16259314 | 29/11/2022 | Africa / Morocco / Casablanca | Human | unknown | Female | 81 | unknown | unknown | unknown | BQ.1.1 | GRA |
| hCoV-19/Morocco/IPM20432802/2022 | EPI_ISL_16259313 | 29/11/2022 | Africa / Morocco / Casablanca | Human | unknown | Female | 49 | unknown | unknown | unknown | BQ.1.1 | GRA |
| hCoV-19/Morocco/IPM20432798/2022 | EPI_ISL_16259312 | 29/11/2022 | Africa / Morocco / Casablanca | Human | unknown | Female | 25 | unknown | unknown | unknown | BQ.1.1 | GRA |
| hCoV-19/Morocco/IPM20432797/2022 | EPI_ISL_16259311 | 29/11/2022 | Africa / Morocco / Casablanca | Human | unknown | Female | 38 | unknown | unknown | unknown | BQ.1.1 | GRA |
| hCoV-19/Morocco/IPM20432744/2022 | EPI_ISL_16259310 | 28/11/2022 | Africa / Morocco / Casablanca | Human | unknown | Female | 42 | unknown | unknown | unknown | XBB.1.38 | GRA |
| hCoV-19/Morocco/IPM20432740/2022 | EPI_ISL_16259309 | 28/11/2022 | Africa / Morocco / Casablanca | Human | unknown | Female | 59 | unknown | unknown | unknown | BQ.1.1 | GRA |
| hCoV-19/Morocco/IPM20432737/2022 | EPI_ISL_16259308 | 28/11/2022 | Africa / Morocco / Casablanca | Human | unknown | Female | 56 | unknown | unknown | unknown | XBB.1 | GRA |
| hCoV-19/Morocco/IPM20432734/2022 | EPI_ISL_16259307 | 28/11/2022 | Africa / Morocco / Casablanca | Human | unknown | Female | 33 | unknown | unknown | unknown | BQ.1 | GRA |
| hCoV-19/Morocco/IPM20432700/2022 | EPI_ISL_16259306 | 25/11/2022 | Africa / Morocco / Casablanca | Human | unknown | Male | 45 | unknown | unknown | unknown | BQ.1 | GRA |
| hCoV-19/Morocco/IPM20432679/2022 | EPI_ISL_16259305 | 25/11/2022 | Africa / Morocco / Casablanca | Human | unknown | Female | 69 | unknown | unknown | unknown | BQ.1.1 | GRA |
| hCoV-19/Morocco/IPM20432677/2022 | EPI_ISL_16259304 | 25/11/2022 | Africa / Morocco / Casablanca | Human | unknown | Female | 38 | unknown | unknown | unknown | BQ.1.1.18 | GRA |
| hCoV-19/Morocco/IPM20432663/2022 | EPI_ISL_16259303 | 24/11/2022 | Africa / Morocco / Casablanca | Human | unknown | Female | 35 | unknown | unknown | unknown | BQ.1.1 | GRA |
| hCoV-19/Morocco/IPM20432652/2022 | EPI_ISL_16259302 | 23/11/2022 | Africa / Morocco / Casablanca | Human | unknown | Female | 59 | unknown | unknown | unknown | BQ.1 | GRA |
| hCoV-19/Morocco/IPM20432650/2022 | EPI_ISL_16259301 | 23/11/2022 | Africa / Morocco / Casablanca | Human | unknown | Male | 40 | unknown | unknown | unknown | BQ.1.1 | GRA |
| hCoV-19/Morocco/IPM20432577/2022 | EPI_ISL_16259300 | 22/11/2022 | Africa / Morocco / Casablanca | Human | unknown | Female | 26 | unknown | unknown | unknown | BQ.1.1 | GRA |
| hCoV-19/Morocco/IPM20432554/2022 | EPI_ISL_16259299 | 21/11/2022 | Africa / Morocco / Casablanca | Human | unknown | Male | 23 | unknown | unknown | unknown | BQ.1 | GRA |
| hCoV-19/Morocco/IPM20432544/2022 | EPI_ISL_16259298 | 21/11/2022 | Africa / Morocco / Casablanca | Human | unknown | Female | 28 | unknown | unknown | unknown | BQ.1.1 | GRA |
| hCoV-19/Morocco/IPM20432542/2022 | EPI_ISL_16259297 | 21/11/2022 | Africa / Morocco / Casablanca | Human | unknown | Female | 31 | unknown | unknown | unknown | BQ.1 | GRA |
| hCoV-19/Morocco/IPM20432534/2022 | EPI_ISL_16259296 | 21/11/2022 | Africa / Morocco / Casablanca | Human | unknown | Female | 48 | unknown | unknown | unknown | BQ.1.5 | GRA |
| hCoV-19/Morocco/IPM20432524/2022 | EPI_ISL_16259295 | 21/11/2022 | Africa / Morocco / Casablanca | Human | unknown | Female | 74 | unknown | unknown | unknown | BQ.1 | GRA |
| hCoV-19/Morocco/IPM20432522/2022 | EPI_ISL_16259294 | 17/11/2022 | Africa / Morocco / Casablanca | Human | unknown | Male | 43 | unknown | unknown | unknown | BQ.1.1 | GRA |
| hCoV-19/Morocco/IPM20432487/2022 | EPI_ISL_16259293 | 16/11/2022 | Africa / Morocco / Casablanca | Human | unknown | Male | 30 | unknown | unknown | unknown | BQ.1.1 | GRA |
| hCoV-19/Morocco/IPM20432468/2022 | EPI_ISL_16259292 | 16/11/2022 | Africa / Morocco / Casablanca | Human | unknown | Male | 60 | unknown | unknown | unknown | BQ.1.1 | GRA |
| hCoV-19/Morocco/IPM20432388/2022 | EPI_ISL_16259291 | 14/11/2022 | Africa / Morocco / Casablanca | Human | unknown | Male | 68 | unknown | unknown | unknown | BQ.1.1 | GRA |
| hCoV-19/Morocco/IPM20432362/2022 | EPI_ISL_16259290 | 11/11/2022 | Africa / Morocco / Casablanca | Human | unknown | Male | 58 | unknown | unknown | unknown | BQ.1.1 | GRA |
| hCoV-19/Morocco/IPM20432353/2022 | EPI_ISL_16259289 | 11/11/2022 | Africa / Morocco / Casablanca | Human | unknown | Male | 56 | unknown | unknown | unknown | CR.1 | GRA |
| hCoV-19/Morocco/IPM20432349/2022 | EPI_ISL_16259288 | 11/11/2022 | Africa / Morocco / Casablanca | Human | unknown | Female | 50 | unknown | unknown | unknown | BQ.1.1 | GRA |
| hCoV-19/Morocco/IPM20432330/2022 | EPI_ISL_16259287 | 10/11/2022 | Africa / Morocco / Casablanca | Human | unknown | Female | 49 | unknown | unknown | unknown | BQ.1 | GRA |
| hCoV-19/Morocco/IPM20432322/2022 | EPI_ISL_16259286 | 10/11/2022 | Africa / Morocco / Casablanca | Human | unknown | Male | 27 | unknown | unknown | unknown | BQ.1 | GRA |
| hCoV-19/Morocco/IPM20432259/2022 | EPI_ISL_16259285 | 08/11/2022 | Africa / Morocco / Casablanca | Human | unknown | Male | 41 | unknown | unknown | unknown | BQ.1 | GRA |
| hCoV-19/Morocco/FMP573/2021 | EPI_ISL_15912224 | 27/11/2021 | Africa / Morocco | Human | unknown | Male | 51 | unknown | unknown | unknown | B.1.177 | GV |
| hCoV-19/Morocco/FMP551/2022 | EPI_ISL_15912223 | 19/02/2022 | Africa / Morocco | Human | unknown | Male | 51 | unknown | unknown | unknown | B.1.177 | GV |
| hCoV-19/Morocco/FMP450/2021 | EPI_ISL_15912222 | 17/12/2021 | Africa / Morocco | Human | unknown | Male | 51 | unknown | unknown | unknown | B.1.177 | GV |
| hCoV-19/Morocco/FMP449/2021 | EPI_ISL_15912221 | 16/11/2021 | Africa / Morocco | Human | unknown | Male | 51 | unknown | unknown | unknown | B.1.177 | GV |
| hCoV-19/Morocco/IPM20424300/2022 | EPI_ISL_15897568 | 30/06/2022 | Africa / Morocco / Casablanca | Human | unknown | Female | 49 | unknown | unknown | unknown | BA.5.2 | GRA |
| hCoV-19/Morocco/IPM20424514/2022 | EPI_ISL_15897567 | 30/06/2022 | Africa / Morocco / Casablanca | Human | unknown | Female | 48 | unknown | unknown | unknown | BA.5.2.20 | GRA |
| hCoV-19/Morocco/IPM20425020/2022 | EPI_ISL_15897566 | 02/07/2022 | Africa / Morocco / Casablanca | Human | unknown | Female | 34 | unknown | unknown | unknown | BA.5 | GRA |
| hCoV-19/Morocco/IPM20424804/2022 | EPI_ISL_15897565 | 02/07/2022 | Africa / Morocco / Casablanca | Human | unknown | Male | 60 | unknown | unknown | unknown | BA.5.2.20 | GRA |
| hCoV-19/Morocco/IPM20425311/2022 | EPI_ISL_15897564 | 02/07/2022 | Africa / Morocco / Casablanca | Human | unknown | Female | 61 | unknown | unknown | unknown | BA.5.1 | GRA |
| hCoV-19/Morocco/IPM20426103/2022 | EPI_ISL_15897563 | 04/07/2022 | Africa / Morocco / Casablanca | Human | unknown | Male | 51 | unknown | unknown | unknown | BA.5.2.20 | GRA |
| hCoV-19/Morocco/IPM20424428/2022 | EPI_ISL_15897562 | 30/06/2022 | Africa / Morocco / Casablanca | Human | unknown | Female | 9 | unknown | unknown | unknown | BA.5.2 | GRA |
| hCoV-19/Morocco/IPM20425277/2022 | EPI_ISL_15897561 | 02/07/2022 | Africa / Morocco / Casablanca | Human | unknown | Male | 50 | unknown | unknown | unknown | BA.2 | GRA |
| hCoV-19/Morocco/IPM20424464/2022 | EPI_ISL_15897560 | 30/06/2022 | Africa / Morocco / Casablanca | Human | unknown | Male | 27 | unknown | unknown | unknown | BA.5.2 | GRA |
| hCoV-19/Morocco/IPM20426397/2022 | EPI_ISL_15897559 | 05/07/2022 | Africa / Morocco / Casablanca | Human | unknown | Male | 55 | unknown | unknown | unknown | BA.5.2.20 | GRA |
| hCoV-19/Morocco/IPM20424315/2022 | EPI_ISL_15897558 | 30/06/2022 | Africa / Morocco / Casablanca | Human | unknown | Female | 52 | unknown | unknown | unknown | BA.5.2.1 | GRA |
| hCoV-19/Morocco/IPM20425202/2022 | EPI_ISL_15897557 | 02/07/2022 | Africa / Morocco / Casablanca | Human | unknown | Female | 25 | unknown | unknown | unknown | BA.5.2 | GRA |
| hCoV-19/Morocco/IPM20424476/2022 | EPI_ISL_15897556 | 30/06/2022 | Africa / Morocco / Casablanca | Human | unknown | Male | 51 | unknown | unknown | unknown | BA.5.2.20 | GRA |
| hCoV-19/Morocco/IPM20425324/2022 | EPI_ISL_15897555 | 02/07/2022 | Africa / Morocco / Casablanca | Human | unknown | Female | 23 | unknown | unknown | unknown | BA.5.2 | GRA |
| hCoV-19/Morocco/IPM20426273/2022 | EPI_ISL_15897554 | 04/07/2022 | Africa / Morocco / Casablanca | Human | unknown | Female | 54 | unknown | unknown | unknown | BA.5.2.20 | GRA |
| hCoV-19/Morocco/IPM20425289/2022 | EPI_ISL_15897553 | 02/07/2022 | Africa / Morocco / Casablanca | Human | unknown | Female | 41 | unknown | unknown | unknown | BA.5.2.20 | GRA |
| hCoV-19/Morocco/IPM20425921/2022 | EPI_ISL_15897552 | 02/07/2022 | Africa / Morocco / Casablanca | Human | unknown | Male | 48 | unknown | unknown | unknown | BA.2.12.1 | GRA |
| hCoV-19/Morocco/IPM20424325/2022 | EPI_ISL_15897551 | 30/06/2022 | Africa / Morocco / Casablanca | Human | unknown | Male | 50 | unknown | unknown | unknown | BF.5 | GRA |
| hCoV-19/Morocco/IPM20424485/2022 | EPI_ISL_15897550 | 30/06/2022 | Africa / Morocco / Casablanca | Human | unknown | Male | 68 | unknown | unknown | unknown | Unassigned | GR |
| hCoV-19/Morocco/IPM20424397/2022 | EPI_ISL_15897549 | 30/06/2022 | Africa / Morocco / Casablanca | Human | unknown | Male | 46 | unknown | unknown | unknown | BA.5.2 | GRA |
| hCoV-19/Morocco/IPM20424366/2022 | EPI_ISL_15897548 | 30/06/2022 | Africa / Morocco / Casablanca | Human | unknown | Male | 29 | unknown | unknown | unknown | BA.5.1 | GRA |
| hCoV-19/Morocco/IPM20425769/2022 | EPI_ISL_15897547 | 02/07/2022 | Africa / Morocco / Casablanca | Human | unknown | Female | 77 | unknown | unknown | unknown | BA.5.2 | GRA |
| hCoV-19/Morocco/IPM20424174/2022 | EPI_ISL_15897546 | 29/06/2022 | Africa / Morocco / Casablanca | Human | unknown | Female | 83 | unknown | unknown | unknown | BA.5.2.20 | GRA |
| hCoV-19/Morocco/IPM20426333/2022 | EPI_ISL_15897545 | 04/07/2022 | Africa / Morocco / Casablanca | Human | unknown | Male | 56 | unknown | unknown | unknown | BA.2.12.1 | GRA |
| hCoV-19/Morocco/IPM20425726/2022 | EPI_ISL_15897544 | 02/07/2022 | Africa / Morocco / Casablanca | Human | unknown | Male | 29 | unknown | unknown | unknown | BA.5.2 | GRA |
| hCoV-19/Morocco/IPM20424472/2022 | EPI_ISL_15897543 | 30/06/2022 | Africa / Morocco / Casablanca | Human | unknown | Male | 62 | unknown | unknown | unknown | BA.4 | GRA |
| hCoV-19/Morocco/IPM20425461/2022 | EPI_ISL_15897542 | 02/07/2022 | Africa / Morocco / Casablanca | Human | unknown | Male | 99 | unknown | unknown | unknown | BA.5.2.20 | GRA |
| hCoV-19/Morocco/IPM20426227/2022 | EPI_ISL_15897541 | 04/07/2022 | Africa / Morocco / Casablanca | Human | unknown | Male | 72 | unknown | unknown | unknown | BA.5.2 | GRA |
| hCoV-19/Morocco/IPM20426220/2022 | EPI_ISL_15897540 | 04/07/2022 | Africa / Morocco / Casablanca | Human | unknown | Male | 81 | unknown | unknown | unknown | BA.5.2 | GRA |
| hCoV-19/Morocco/IPM20426163/2022 | EPI_ISL_15897539 | 04/07/2022 | Africa / Morocco / Casablanca | Human | unknown | Female | 35 | unknown | unknown | unknown | BA.5.2.20 | GRA |
| hCoV-19/Morocco/IPM20426149/2022 | EPI_ISL_15897538 | 04/07/2022 | Africa / Morocco / Casablanca | Human | unknown | Male | 61 | unknown | unknown | unknown | BA.5.2 | GRA |
| hCoV-19/Morocco/IPM20426084/2022 | EPI_ISL_15897537 | 04/07/2022 | Africa / Morocco / Casablanca | Human | unknown | Male | 39 | unknown | unknown | unknown | BF.5 | GRA |
| hCoV-19/Morocco/IPM20426018/2022 | EPI_ISL_15897536 | 02/07/2022 | Africa / Morocco / Casablanca | Human | unknown | Male | 60 | unknown | unknown | unknown | BF.5 | GRA |
| hCoV-19/Morocco/IPM20425948/2022 | EPI_ISL_15897535 | 02/07/2022 | Africa / Morocco / Casablanca | Human | unknown | Male | 51 | unknown | unknown | unknown | BA.5.2.20 | GRA |
| hCoV-19/Morocco/IPM20425892/2022 | EPI_ISL_15897534 | 02/07/2022 | Africa / Morocco / Casablanca | Human | unknown | Female | 64 | unknown | unknown | unknown | BA.5.2 | GRA |
| hCoV-19/Morocco/IPM20425738/2022 | EPI_ISL_15897533 | 02/07/2022 | Africa / Morocco / Casablanca | Human | unknown | Female | 54 | unknown | unknown | unknown | BE.3 | GRA |
| hCoV-19/Morocco/IPM20425585/2022 | EPI_ISL_15897532 | 02/07/2022 | Africa / Morocco / Casablanca | Human | unknown | Male | 32 | unknown | unknown | unknown | BA.5.2 | GRA |
| hCoV-19/Morocco/IPM20425569/2022 | EPI_ISL_15897531 | 02/07/2022 | Africa / Morocco / Casablanca | Human | unknown | Female | 30 | unknown | unknown | unknown | BA.5.2.20 | GRA |
| hCoV-19/Morocco/IPM20425535/2022 | EPI_ISL_15897530 | 02/07/2022 | Africa / Morocco / Casablanca | Human | unknown | Female | 49 | unknown | unknown | unknown | BA.5.2.20 | GRA |
| hCoV-19/Morocco/IPM20425516/2022 | EPI_ISL_15897529 | 02/07/2022 | Africa / Morocco / Casablanca | Human | unknown | Male | 35 | unknown | unknown | unknown | BA.5.2 | GRA |
| hCoV-19/Morocco/IPM20425488/2022 | EPI_ISL_15897528 | 02/07/2022 | Africa / Morocco / Casablanca | Human | unknown | Female | 40 | unknown | unknown | unknown | BA.5.2 | GRA |
| hCoV-19/Morocco/IPM20425408/2022 | EPI_ISL_15897527 | 02/07/2022 | Africa / Morocco / Casablanca | Human | unknown | Female | 40 | unknown | unknown | unknown | BA.5.2 | GRA |
| hCoV-19/Morocco/IPM20425374/2022 | EPI_ISL_15897526 | 02/07/2022 | Africa / Morocco / Casablanca | Human | unknown | Female | 18 | unknown | unknown | unknown | BA.5.2 | GRA |
| hCoV-19/Morocco/IPM20425365/2022 | EPI_ISL_15897525 | 02/07/2022 | Africa / Morocco / Casablanca | Human | unknown | Female | 50 | unknown | unknown | unknown | BA.5.2.1 | GRA |
| hCoV-19/Morocco/IPM20425342/2022 | EPI_ISL_15897524 | 02/07/2022 | Africa / Morocco / Casablanca | Human | unknown | Male | 31 | unknown | unknown | unknown | BA.5.2.1 | GRA |
| hCoV-19/Morocco/IPM20425302/2022 | EPI_ISL_15897523 | 02/07/2022 | Africa / Morocco / Casablanca | Human | unknown | Male | 22 | unknown | unknown | unknown | BA.5.2.20 | GRA |
| hCoV-19/Morocco/IPM20425262/2022 | EPI_ISL_15897522 | 02/07/2022 | Africa / Morocco / Casablanca | Human | unknown | Female | 48 | unknown | unknown | unknown | BA.5.2.20 | GRA |
| hCoV-19/Morocco/IPM20425252/2022 | EPI_ISL_15897521 | 02/07/2022 | Africa / Morocco / Casablanca | Human | unknown | Female | 54 | unknown | unknown | unknown | BA.5.2.20 | GRA |
| hCoV-19/Morocco/IPM20425183/2022 | EPI_ISL_15897520 | 02/07/2022 | Africa / Morocco / Casablanca | Human | unknown | Female | 76 | unknown | unknown | unknown | BA.5.2.20 | GRA |
| hCoV-19/Morocco/IPM20425143/2022 | EPI_ISL_15897519 | 02/07/2022 | Africa / Morocco / Casablanca | Human | unknown | Male | 28 | unknown | unknown | unknown | BA.5.2 | GRA |
| hCoV-19/Morocco/IPM20425096/2022 | EPI_ISL_15897518 | 02/07/2022 | Africa / Morocco / Casablanca | Human | unknown | Female | 38 | unknown | unknown | unknown | BA.5.2.20 | GRA |
| hCoV-19/Morocco/IPM20424694/2022 | EPI_ISL_15897517 | 02/07/2022 | Africa / Morocco / Casablanca | Human | unknown | Female | 22 | unknown | unknown | unknown | BA.5.2 | GRA |
| hCoV-19/Morocco/IPM20424625/2022 | EPI_ISL_15897516 | 02/07/2022 | Africa / Morocco / Casablanca | Human | unknown | Female | 37 | unknown | unknown | unknown | BA.5.1 | GRA |
| hCoV-19/Morocco/IPM20424621/2022 | EPI_ISL_15897515 | 02/07/2022 | Africa / Morocco / Casablanca | Human | unknown | Female | 47 | unknown | unknown | unknown | BA.5.1 | GRA |
| hCoV-19/Morocco/IPM20424614/2022 | EPI_ISL_15897514 | 01/07/2022 | Africa / Morocco / Casablanca | Human | unknown | Female | 44 | unknown | unknown | unknown | BA.5.2 | GRA |
| hCoV-19/Morocco/IPM20424607/2022 | EPI_ISL_15897513 | 01/07/2022 | Africa / Morocco / Casablanca | Human | unknown | Female | 60 | unknown | unknown | unknown | BA.5.2.20 | GRA |
| hCoV-19/Morocco/IPM20424495/2022 | EPI_ISL_15897512 | 30/06/2022 | Africa / Morocco / Casablanca | Human | unknown | Female | 38 | unknown | unknown | unknown | BA.5.2 | GRA |
| hCoV-19/Morocco/IPM20424490/2022 | EPI_ISL_15897511 | 30/06/2022 | Africa / Morocco / Casablanca | Human | unknown | Female | 64 | unknown | unknown | unknown | BA.5.1 | GRA |
| hCoV-19/Morocco/IPM20424481/2022 | EPI_ISL_15897510 | 30/06/2022 | Africa / Morocco / Casablanca | Human | unknown | Male | 24 | unknown | unknown | unknown | BA.5.2 | GRA |
| hCoV-19/Morocco/IPM20424465/2022 | EPI_ISL_15897509 | 30/06/2022 | Africa / Morocco / Casablanca | Human | unknown | Male | 71 | unknown | unknown | unknown | BA.5.2.20 | GRA |
| hCoV-19/Morocco/IPM20424458/2022 | EPI_ISL_15897508 | 30/06/2022 | Africa / Morocco / Casablanca | Human | unknown | Female | 63 | unknown | unknown | unknown | BA.5.1.22 | GRA |
| hCoV-19/Morocco/IPM20424433/2022 | EPI_ISL_15897507 | 30/06/2022 | Africa / Morocco / Casablanca | Human | unknown | Female | 25 | unknown | unknown | unknown | BA.5.2 | GRA |
| hCoV-19/Morocco/IPM20424430/2022 | EPI_ISL_15897506 | 30/06/2022 | Africa / Morocco / Casablanca | Human | unknown | Female | 44 | unknown | unknown | unknown | BA.5.2 | GRA |
| hCoV-19/Morocco/IPM20424422/2022 | EPI_ISL_15897505 | 30/06/2022 | Africa / Morocco / Casablanca | Human | unknown | Female | 67 | unknown | unknown | unknown | BA.5.2.20 | GRA |
| hCoV-19/Morocco/IPM20424388/2022 | EPI_ISL_15897504 | 30/06/2022 | Africa / Morocco / Casablanca | Human | unknown | Female | 50 | unknown | unknown | unknown | BA.5.2.20 | GRA |
| hCoV-19/Morocco/IPM20424292/2022 | EPI_ISL_15897503 | 30/06/2022 | Africa / Morocco / Casablanca | Human | unknown | Female | 59 | unknown | unknown | unknown | BA.5.2 | GRA |
| hCoV-19/Morocco/IPM20424237/2022 | EPI_ISL_15897502 | 29/06/2022 | Africa / Morocco / Casablanca | Human | unknown | Male | 50 | unknown | unknown | unknown | BA.5.2 | GRA |
| hCoV-19/Morocco/IPM20424190/2022 | EPI_ISL_15897501 | 29/06/2022 | Africa / Morocco / Casablanca | Human | unknown | Female | 61 | unknown | unknown | unknown | BA.5.2.20 | GRA |
| hCoV-19/Morocco/IPM20426048/2022 | EPI_ISL_15897500 | 02/07/2022 | Africa / Morocco / Casablanca | Human | unknown | Female | 51 | unknown | unknown | unknown | BA.2.9.3 | GRA |
| hCoV-19/Morocco/IPM20424977/2022 | EPI_ISL_15897499 | 02/07/2022 | Africa / Morocco / Casablanca | Human | unknown | Male | 69 | unknown | unknown | unknown | BA.2.12.1 | GRA |
| hCoV-19/Morocco/IPM20424297/2022 | EPI_ISL_15897498 | 30/06/2022 | Africa / Morocco / Casablanca | Human | unknown | Female | 40 | unknown | unknown | unknown | BA.2.12.1 | GRA |
| hCoV-19/Morocco/IPM20424874/2022 | EPI_ISL_15897497 | 02/07/2022 | Africa / Morocco / Casablanca | Human | unknown | Female | 40 | unknown | unknown | unknown | BF.5 | GRA |
| hCoV-19/Morocco/IPM20432252/2022 | EPI_ISL_15889734 | 07/11/2022 | Africa / Morocco / Casablanca | Human | unknown | Female | 54 | unknown | unknown | unknown | BQ.1.1 | GRA |
| hCoV-19/Morocco/IPM20432224/2022 | EPI_ISL_15889733 | 04/11/2022 | Africa / Morocco / Casablanca | Human | unknown | Female | 54 | unknown | unknown | unknown | BA.4 | GRA |
| hCoV-19/Morocco/IPM20432198/2022 | EPI_ISL_15889732 | 04/11/2022 | Africa / Morocco / Casablanca | Human | unknown | unknown | unknown | unknown | unknown | unknown | BQ.1.1 | GRA |
| hCoV-19/Morocco/IPM20432169/2022 | EPI_ISL_15889731 | 02/11/2022 | Africa / Morocco / Casablanca | Human | unknown | Female | 73 | unknown | unknown | unknown | BQ.1.1 | GRA |
| hCoV-19/Morocco/IPM20431387/2022 | EPI_ISL_15889730 | 29/09/2022 | Africa / Morocco / Casablanca | Human | unknown | Female | 46 | unknown | unknown | unknown | BQ.1.1 | GRA |
| hCoV-19/Morocco/IPM20431292/2022 | EPI_ISL_15889729 | 27/09/2022 | Africa / Morocco / Casablanca | Human | unknown | Male | 50 | unknown | unknown | unknown | BQ.1.1 | GRA |
| hCoV-19/Morocco/IPM20431264/2022 | EPI_ISL_15889728 | 26/09/2022 | Africa / Morocco / Casablanca | Human | unknown | Male | 57 | unknown | unknown | unknown | BQ.1.1 | GRA |
| hCoV-19/Morocco/IPM20432135/2022 | EPI_ISL_15889727 | 01/11/2022 | Africa / Morocco / Casablanca | Human | unknown | Female | 49 | unknown | unknown | unknown | BQ.1.1 | GRA |
| hCoV-19/Morocco/IPM20432116/2022 | EPI_ISL_15889726 | 31/10/2022 | Africa / Morocco / Casablanca | Human | unknown | Female | 40 | unknown | unknown | unknown | BQ.1.1 | GRA |
| hCoV-19/Morocco/IPM20432063/2022 | EPI_ISL_15889725 | 28/10/2022 | Africa / Morocco / Casablanca | Human | unknown | Female | 28 | unknown | unknown | unknown | BA.4 | GRA |
| hCoV-19/Morocco/IPM20432031/2022 | EPI_ISL_15889724 | 26/10/2022 | Africa / Morocco / Casablanca | Human | unknown | Female | 26 | unknown | unknown | unknown | BQ.1.1 | GRA |
| hCoV-19/Morocco/IPM20432020/2022 | EPI_ISL_15889723 | 26/10/2022 | Africa / Morocco / Casablanca | Human | unknown | Female | 50 | unknown | unknown | unknown | BQ.1.1 | GRA |
| hCoV-19/Morocco/IPM20431909/2022 | EPI_ISL_15889722 | 21/10/2022 | Africa / Morocco / Casablanca | Human | unknown | Male | 36 | unknown | unknown | unknown | BA.4 | GRA |
| hCoV-19/Morocco/IPM20431881/2022 | EPI_ISL_15889721 | 20/10/2022 | Africa / Morocco / Casablanca | Human | unknown | Male | 58 | unknown | unknown | unknown | BQ.1.1 | GRA |
| hCoV-19/Morocco/IPM20431877/2022 | EPI_ISL_15889720 | 20/10/2022 | Africa / Morocco / Casablanca | Human | unknown | Male | 35 | unknown | unknown | unknown | BA.5.2.1 | GRA |
| hCoV-19/Morocco/IPM20431825/2022 | EPI_ISL_15889719 | 18/10/2022 | Africa / Morocco / Casablanca | Human | unknown | Male | 31 | unknown | unknown | unknown | BQ.1.1 | GRA |
| hCoV-19/Morocco/IPM20431808/2022 | EPI_ISL_15889718 | 18/10/2022 | Africa / Morocco / Casablanca | Human | unknown | Male | 40 | unknown | unknown | unknown | BA.5.2 | GRA |
| hCoV-19/Morocco/IPM20431767/2022 | EPI_ISL_15889717 | 14/10/2022 | Africa / Morocco / Casablanca | Human | unknown | Female | 40 | unknown | unknown | unknown | BA.5.2 | GRA |
| hCoV-19/Morocco/IPM20431765/2022 | EPI_ISL_15889716 | 14/10/2022 | Africa / Morocco / Casablanca | Human | unknown | Male | 44 | unknown | unknown | unknown | BQ.1.1 | GRA |
| hCoV-19/Morocco/IPM20431748/2022 | EPI_ISL_15889715 | 14/10/2022 | Africa / Morocco / Casablanca | Human | unknown | Male | 32 | unknown | unknown | unknown | BA.5.2 | GRA |
| hCoV-19/Morocco/IPM20431653/2022 | EPI_ISL_15889714 | 08/10/2022 | Africa / Morocco / Casablanca | Human | unknown | Female | 57 | unknown | unknown | unknown | BA.4 | GRA |
| hCoV-19/Morocco/IPM20431617/2022 | EPI_ISL_15889713 | 06/10/2022 | Africa / Morocco / Casablanca | Human | unknown | Male | 38 | unknown | unknown | unknown | BQ.1.1 | GRA |
| hCoV-19/Morocco/IPM20431616/2022 | EPI_ISL_15889712 | 06/10/2022 | Africa / Morocco / Casablanca | Human | unknown | Female | 24 | unknown | unknown | unknown | BQ.1.1 | GRA |
| hCoV-19/Morocco/IPM20431489/2022 | EPI_ISL_15889711 | 03/10/2022 | Africa / Morocco / Casablanca | Human | unknown | Male | 58 | unknown | unknown | unknown | BQ.1.1 | GRA |
| hCoV-19/Morocco/IPM20431439/2022 | EPI_ISL_15889710 | 30/09/2022 | Africa / Morocco / Casablanca | Human | unknown | Female | 50 | unknown | unknown | unknown | BQ.1.1 | GRA |
| hCoV-19/Morocco/IPM20431406/2022 | EPI_ISL_15889709 | 30/09/2022 | Africa / Morocco / Casablanca | Human | unknown | unknown | unknown | unknown | unknown | unknown | BA.5.2 | GRA |
| hCoV-19/Morocco/20352937/2021 | EPI_ISL_4511389 | 26/08/2021 | Africa / Morocco / Casablanca | Human | unknown | Male | 41 | unknown | unknown | unknown | AY.122 | GK |
| hCoV-19/Morocco/RMPS-15/2020 | EPI_ISL_482740 | 26/05/2020 | Africa / Morocco | Human | unknown | Male | unknown | unknown | unknown | unknown | B.1 | G |
| hCoV-19/Morocco/RMPS-12/2020 | EPI_ISL_482737 | 08/05/2020 | Africa / Morocco | Human | unknown | Male | unknown | unknown | unknown | unknown | B.1.1 | GR |
| hCoV-19/Morocco/RMPS-11/2020 | EPI_ISL_482736 | 24/04/2020 | Africa / Morocco | Human | unknown | unknown | unknown | unknown | unknown | unknown | B.1 | GH |
| hCoV-19/Morocco/RMPS-10/2020 | EPI_ISL_482735 | 22/04/2020 | Africa / Morocco | Human | unknown | Female | unknown | unknown | unknown | unknown | B.1 | GH |
| hCoV-19/Morocco/RMPS-09/2020 | EPI_ISL_482734 | 01/04/2020 | Africa / Morocco | Human | unknown | Female | unknown | unknown | unknown | unknown | B.1 | GH |
| hCoV-19/Morocco/RMPS-08/2020 | EPI_ISL_482733 | 01/04/2020 | Africa / Morocco | Human | unknown | Female | unknown | unknown | unknown | unknown | B.1 | GH |
| hCoV-19/Morocco/refstage1/2020 | EPI_ISL_476559 | 27/02/2020 | Africa / Morocco | Human | unknown | unknown | unknown | unknown | unknown | unknown | B.1 | G |
| hCoV-19/Morocco/AZ183/2020 | EPI_ISL_476026 | 11/05/2020 | Africa / Morocco | Human | unknown | Female | unknown | unknown | unknown | unknown | B.1.1 | GR |
| hCoV-19/Morocco/CA24/2020 | EPI_ISL_476025 | 02/05/2020 | Africa / Morocco | Human | unknown | Male | 39 | unknown | unknown | unknown | B.1.1 | GR |
| hCoV-19/Morocco/HMIMV-14N/2020 | EPI_ISL_2968038 | 18/07/2020 | Africa / Morocco / Rabat | Human | unknown | Male | 67 | Released | unknown | unknown | B.1 | G |
| hCoV-19/Morocco/HMIMV-Rabat1462-04/2020 | EPI_ISL_471460 | 19/04/2020 | Africa / Morocco / Rabat | Human | unknown | Male | 40 | Released | unknown | unknown | B.1 | G |
| hCoV-19/Morocco/HMIMV-Rabat1435-04/2020 | EPI_ISL_471459 | 19/04/2020 | Africa / Morocco / Rabat | Human | unknown | Male | 32 | Released | unknown | unknown | B.1 | G |
| hCoV-19/Morocco/HMIMV-Rabat1429-04/2020 | EPI_ISL_471458 | 19/04/2020 | Africa / Morocco / Rabat | Human | unknown | Male | 36 | Released | unknown | unknown | B.1 | G |
| hCoV-19/Morocco/HMIMV-Rabat1025-04/2020 | EPI_ISL_471457 | 13/04/2020 | Africa / Morocco / Rabat | Human | unknown | Male | 28 | Released | unknown | unknown | B.1 | G |
| hCoV-19/Morocco/HMIMV-Rabat102-03/2020 | EPI_ISL_471456 | 31/03/2020 | Africa / Morocco / Rabat | Human | unknown | Male | 48 | Released | unknown | unknown | B.1 | G |
| hCoV-19/Morocco/HMIMV-00210/2020 | EPI_ISL_2968058 | 07/12/2020 | Africa / Morocco / Rabat | Human | unknown | Female | 53 | Released | unknown | unknown | B.1.1 | GR |
| hCoV-19/Morocco/RMPS-06/2020 | EPI_ISL_469054 | 01/04/2020 | Africa / Morocco | Human | unknown | unknown | unknown | unknown | unknown | unknown | B.1 | GH |
| hCoV-19/Morocco/RMPS-05/2020 | EPI_ISL_469053 | 30/03/2020 | Africa / Morocco | Human | unknown | unknown | unknown | unknown | unknown | unknown | B.1 | GH |
| hCoV-19/Morocco/RMPS-02/2020 | EPI_ISL_469049 | 30/03/2020 | Africa / Morocco | Human | unknown | unknown | unknown | unknown | unknown | unknown | B.1 | GH |
| hCoV-19/Morocco/RMPS-01/2020 | EPI_ISL_469017 | 13/04/2020 | Africa / Morocco | Human | unknown | unknown | unknown | unknown | unknown | unknown | B.1 | GH |
| hCoV-19/Morocco/RA85/2020 | EPI_ISL_467299 | 21/05/2020 | Africa / Morocco | Human | unknown | Male | 25 | Live | unknown | unknown | B.1 | G |
| hCoV-19/Morocco/6905/2020 | EPI_ISL_459983 | 21/04/2020 | Africa / Morocco | Human | unknown | unknown | unknown | unknown | unknown | unknown | B.1.1 | GR |
| hCoV-19/Morocco/6902/2020 | EPI_ISL_459980 | 19/04/2020 | Africa / Morocco | Human | unknown | unknown | unknown | unknown | unknown | unknown | B.1.1 | GR |
| hCoV-19/Morocco/6900/2020 | EPI_ISL_459978 | 20/04/2020 | Africa / Morocco | Human | unknown | unknown | unknown | unknown | unknown | unknown | B.1.1 | GR |
| hCoV-19/Morocco/6898/2020 | EPI_ISL_459976 | 16/03/2020 | Africa / Morocco | Human | unknown | unknown | unknown | unknown | unknown | unknown | B.1 | GH |
| hCoV-19/Morocco/6892/2020 | EPI_ISL_459970 | 17/03/2020 | Africa / Morocco | Human | unknown | unknown | unknown | unknown | unknown | unknown | B.1 | G |
| hCoV-19/Morocco/6891/2020 | EPI_ISL_459969 | 20/03/2020 | Africa / Morocco | Human | unknown | unknown | unknown | unknown | unknown | unknown | B.1 | GH |
| hCoV-19/Morocco/6890/2020 | EPI_ISL_459968 | 17/03/2020 | Africa / Morocco | Human | unknown | unknown | unknown | unknown | unknown | unknown | B.1 | G |
| hCoV-19/Morocco/6889/2020 | EPI_ISL_459967 | 15/03/2020 | Africa / Morocco | Human | unknown | unknown | unknown | unknown | unknown | unknown | B.1 | G |
| hCoV-19/Morocco/6887/2020 | EPI_ISL_459965 | 03/03/2020 | Africa / Morocco | Human | unknown | unknown | unknown | unknown | unknown | unknown | B.1.1 | GR |
| hCoV-19/Morocco/15N/2020 | EPI_ISL_458150 | 15/05/2020 | Africa / Morocco / Casablanca | Human | unknown | Male | 46 | unknown | unknown | unknown | B.1.1 | GR |
| hCoV-19/Morocco/FMP-324/2021 | EPI_ISL_4572251 | 13/07/2021 | Africa / Morocco / Inezgane | Human | unknown | Female | unknown | unknown | unknown | unknown | AY.33 | GK |
| hCoV-19/Morocco/FMP-203/2021 | EPI_ISL_1904295 | 17/03/2021 | Africa / Morocco / Sidi Lahcen | Human | unknown | Female | 36 | unknown | unknown | unknown | B.1.1.7 | GRY |
| hCoV-19/Morocco/OUA677-19/2020 | EPI_ISL_451400 | 23/04/2020 | Africa / Morocco / Ouarzazate | Human | unknown | Male | unknown | unknown | unknown | unknown | B.1.528 | G |
| hCoV-19/Morocco/1047/2022 | EPI_ISL_17497716 | 12/07/2022 | Africa / Morocco / Casablanca | Human | unknown | Female | 12 | Live | unknown | unknown | BA.5 | GRA |
| hCoV-19/Morocco/1061/2022 | EPI_ISL_17497714 | 25/07/2022 | Africa / Morocco / Sale | Human | unknown | Male | 49 | Live | unknown | unknown | BA.5 | GRA |
| hCoV-19/Morocco/1046/2022 | EPI_ISL_17497715 | 12/07/2022 | Africa / Morocco / Casablanca | Human | unknown | Female | 39 | Live | unknown | unknown | BA.5 | GRA |
| hCoV-19/Morocco/INH-1286/2023 | EPI_ISL_17660880 | 08/03/2023 | Africa / Morocco / Sale | Human | unknown | Female | 42 | Live | unknown | unknown | XBB.1.5 | GRA |
| hCoV-19/Morocco/NIH/1254/2023 | EPI_ISL_17667715 | 05/01/2023 | Africa / Morocco / Rabat | Human | unknown | Female | unknown | Live | unknown | unknown | BQ.1.1 | GRA |
| hCoV-19/Morocco/NIH/1251/2023 | EPI_ISL_17667712 | 03/01/2023 | Africa / Morocco / Rabat | Human | unknown | Female | 30 | Live | unknown | unknown | BQ.1.1 | GRA |
| hCoV-19/Morocco/NIH/1245/2023 | EPI_ISL_17667710 | 02/01/2023 | Africa / Morocco / Rabat | Human | unknown | Male | 58 | Live | unknown | unknown | BQ.1.1.56 | GRA |
| hCoV-19/Morocco/NIH/1255/2023 | EPI_ISL_17667714 | 02/01/2023 | Africa / Morocco / Sale | Human | unknown | Male | 45 | Live | unknown | unknown | BQ.1.1.72 | GRA |
| hCoV-19/Morocco/NIH/1247/2023 | EPI_ISL_17667711 | 02/01/2023 | Africa / Morocco / Rabat | Human | unknown | Female | 57 | Live | unknown | unknown | BQ.1.1.15 | GRA |
| hCoV-19/Morocco/NIH/1248/2023 | EPI_ISL_17667718 | 02/01/2023 | Africa / Morocco / Rabat | Human | unknown | Female | 76 | Live | unknown | unknown | BQ.1.1.6 | GRA |
| hCoV-19/Morocco/NIH/1246/2023 | EPI_ISL_17667717 | 02/01/2023 | Africa / Morocco / Rabat | Human | unknown | Male | 66 | Live | unknown | unknown | BQ.1.1 | GRA |
| hCoV-19/Morocco/NIH/1259/2023 | EPI_ISL_17667721 | 09/01/2023 | Africa / Morocco / Kenitra | Human | unknown | Female | 33 | Live | unknown | unknown | BQ.1.1 | GRA |
| hCoV-19/Morocco/NIH/1256/2023 | EPI_ISL_17667719 | 09/01/2023 | Africa / Morocco / Rabat | Human | unknown | Female | 60 | Live | unknown | unknown | BQ.1.1 | GRA |
| hCoV-19/Morocco/NIH/1244/2023 | EPI_ISL_17667709 | 02/01/2023 | Africa / Morocco / Rabat | Human | unknown | Male | 60 | Live | unknown | unknown | XBB.1 | GRA |
| hCoV-19/Morocco/NIH/1252/2023 | EPI_ISL_17667713 | 02/01/2023 | Africa / Morocco / Kenitra | Human | unknown | Male | 33 | Live | unknown | unknown | BQ.1.1 | GRA |
| hCoV-19/Morocco/NIH/1253/2023 | EPI_ISL_17667716 | 02/01/2023 | Africa / Morocco / Rabat | Human | unknown | Female | unknown | Live | unknown | unknown | BQ.1.1 | GRA |
| hCoV-19/Morocco/NIH/1258/2023 | EPI_ISL_17667720 | 09/01/2023 | Africa / Morocco / Rabat | Human | unknown | Male | unknown | Live | unknown | unknown | BQ.1.1 | GRA |
| hCoV-19/Morocco/NIH/1288/2023 | EPI_ISL_17699790 | 15/05/2023 | Africa / Morocco / Rabat | Human | unknown | Female | 62Years | Live | unknown | unknown | CH.1.1.11 | GRA |
| hCoV-19/Morocco/NIH/1290/2023 | EPI_ISL_17699791 | 16/05/2023 | Africa / Morocco / Rabat | Human | unknown | Male | unknown | Live | unknown | unknown | XBB.1.5.6 | GRA |
| hCoV-19/Morocco/INH-1283/2023 | EPI_ISL_17711384 | 20/01/2023 | Africa / Morocco / Kenitra | Human | unknown | Male | 26 | Live | unknown | unknown | BQ.1.1 | GRA |
| hCoV-19/Morocco/INH-1282/2023 | EPI_ISL_17711383 | 12/01/2023 | Africa / Morocco / Kenitra | Human | unknown | Female | 26 | Live | unknown | unknown | BQ.1.1 | GRA |
| hCoV-19/Morocco/INH-1280/2023 | EPI_ISL_17711382 | 12/01/2023 | Africa / Morocco / Kenitra | Human | unknown | Female | 27 | Live | unknown | unknown | BQ.1.1 | GRA |
| hCoV-19/Morocco/INH-256638/2021 | EPI_ISL_17765536 | 01/01/2021 | Africa / Morocco / Sidi Kacem | Human | unknown | Female | 61 | Live | unknown | unknown | B.1 | G |
| hCoV-19/Morocco/INH-1043/2021 | EPI_ISL_17765538 | 18/02/2021 | Africa / Morocco / Casablanca | Human | unknown | Female | 50 | Live | unknown | unknown | B.1.1.7 | GRY |
| hCoV-19/Morocco/INH-4957/2021 | EPI_ISL_17765539 | 29/05/2021 | Africa / Morocco / Rabat | Human | unknown | Female | 50 | Live | unknown | unknown | B.1.619 | G |
| hCoV-19/Morocco/INH-1062/2021 | EPI_ISL_17765537 | 17/02/2021 | Africa / Morocco / Casablanca | Human | unknown | Male | 57 | Live | unknown | unknown | B.1.1.7 | GRY |
| hCoV-19/Morocco/INH-256563/2021 | EPI_ISL_17765535 | 01/01/2021 | Africa / Morocco / Sale | Human | unknown | Male | 72 | Live | unknown | unknown | B.1 | G |
| hCoV-19/Morocco/INH-4984/2021 | EPI_ISL_17766949 | 19/05/2021 | Africa / Morocco / Casablanca | Human | unknown | Female | 52 | Live | unknown | unknown | B.1.351 | GH |
| hCoV-19/Morocco/INH-4941/2021 | EPI_ISL_17766948 | 26/05/2021 | Africa / Morocco / Sale | Human | unknown | Male | 35 | Live | unknown | unknown | B.1.621 | GH |
| hCoV-19/Morocco/INH-ELH5/2021 | EPI_ISL_17766979 | 21/01/2021 | Africa / Morocco / Al hoceima | Human | unknown | Male | 16 | Live | unknown | unknown | B.1.177.77 | GV |
| hCoV-19/Morocco/INH-256806/2021 | EPI_ISL_17766972 | 01/01/2021 | Africa / Morocco / Sidi Slimane | Human | unknown | Female | 14 | Live | unknown | unknown | B.1 | G |
| hCoV-19/Morocco/INH-264520/2021 | EPI_ISL_17766975 | 31/01/2021 | Africa / Morocco / Sidi Slimane | Human | unknown | Male | 55 | Live | unknown | unknown | B.1.221 | G |
| hCoV-19/Morocco/INH-5019/2021 | EPI_ISL_17766950 | 27/05/2021 | Africa / Morocco / Rabat | Human | unknown | Female | 27 | Live | unknown | unknown | AY.72 | GK |
| hCoV-19/Morocco/INH-264293//2021 | EPI_ISL_17766976 | 07/01/2021 | Africa / Morocco / Sidi Slimane | Human | unknown | Male | 47 | Live | unknown | unknown | B.1.221 | G |
| hCoV-19/Morocco/INH-261305/2021 | EPI_ISL_17766977 | 15/01/2021 | Africa / Morocco / Sale | Human | unknown | Male | 60 | Live | unknown | unknown | B.1.1 | G |
| hCoV-19/Morocco/INH-5020/2021 | EPI_ISL_17766951 | 27/05/2021 | Africa / Morocco / Rabat | Human | unknown | Female | 61 | Live | unknown | unknown | AY.72 | GK |
| hCoV-19/env/Morocco/INH-13/2023 | EPI_ISL_17766982 | 16/02/2023 | Africa / Morocco / step Sidi moussa | unknown | unknown | unknown | unknown | unknown | unknown | unknown | XBB.1.5 | GRA |
| hCoV-19/Morocco/INH-1100/2021 | EPI_ISL_17766967 | 16/01/2021 | Africa / Morocco / Mohammedia | Human | unknown | Female | 32 | Live | unknown | unknown | B.1.1.7 | GRY |
| hCoV-19/Morocco/INH-256571/2021 | EPI_ISL_17766968 | 01/01/2021 | Africa / Morocco / Sale | Human | unknown | Female | 43 | Live | unknown | unknown | B.1.1 | G |
| hCoV-19/Morocco/INH-259640/2021 | EPI_ISL_17766971 | 11/01/2021 | Africa / Morocco / Kenitra | Human | unknown | Male | 20 | Live | unknown | unknown | B.1.177 | GV |
| hCoV-19/Morocco/INH-264187/2021 | EPI_ISL_17766978 | 17/02/2021 | Africa / Morocco / Kenitra | Human | unknown | Male | 72 | Live | unknown | unknown | B.1.1.112 | G |
| hCoV-19/Morocco/INH-264133/2021 | EPI_ISL_17766974 | 15/01/2021 | Africa / Morocco / Kenitra | Human | unknown | Female | 24 | Live | unknown | unknown | B.1.160 | GH |
| hCoV-19/env/Morocco/INH-12/2023 | EPI_ISL_17766981 | 10/02/2023 | Africa / Morocco / step El Yousofia | unknown | unknown | unknown | unknown | unknown | unknown | unknown | XBB.1.9 | GRA |
| hCoV-19/env/Morocco/INH-14/2023 | EPI_ISL_17766983 | 16/02/2023 | Africa / Morocco / step El Yousofia | unknown | unknown | unknown | unknown | unknown | unknown | unknown | XBB.1 | GRA |
| hCoV-19/env/Morocco/INH-17/2023 | EPI_ISL_17766984 | 14/03/2023 | Africa / Morocco / step Sidi moussa | unknown | unknown | unknown | unknown | unknown | unknown | unknown | XBB.1.22 | GRA |
| hCoV-19/Morocco/IPM20436066/2023 | EPI_ISL_17982452 | 01/06/2023 | Africa / Morocco / Casablanca | Human | unknown | Male | 27 | unknown | unknown | unknown | XBB.1.5 | GRA |
| hCoV-19/Morocco/IPM20436060/2023 | EPI_ISL_17982451 | 30/05/2023 | Africa / Morocco / Casablanca | Human | unknown | Male | 33 | unknown | unknown | unknown | XBB.1.5 (consensus call) | GRA |
| hCoV-19/Morocco/IPM20436017/2023 | EPI_ISL_17982446 | 10/05/2023 | Africa / Morocco / Casablanca | Human | unknown | Male | 51 | unknown | unknown | unknown | FL.10 | GRA |
| hCoV-19/Morocco/IPM20436054/2023 | EPI_ISL_17982449 | 24/05/2023 | Africa / Morocco / Casablanca | Human | unknown | Female | 78 | unknown | unknown | unknown | XBB.1.5 | GRA |
| hCoV-19/Morocco/IPM20436040/2023 | EPI_ISL_17982448 | 18/05/2023 | Africa / Morocco / Casablanca | Human | unknown | Male | 29 | unknown | unknown | unknown | XBB.1.9.1 | GRA |
| hCoV-19/Morocco/IPM20436055/2023 | EPI_ISL_17982450 | 24/05/2023 | Africa / Morocco / Casablanca | Human | unknown | Female | 27 | unknown | unknown | unknown | XBB.1.5 | GRA |
| hCoV-19/Morocco/IPM20435997/2023 | EPI_ISL_17982442 | 02/05/2023 | Africa / Morocco / Casablanca | Human | unknown | Male | 47 | unknown | unknown | unknown | FL.10 | GRA |
| hCoV-19/Morocco/IPM20435998/2023 | EPI_ISL_17982443 | 02/05/2023 | Africa / Morocco / Casablanca | Human | unknown | Male | 31 | unknown | unknown | unknown | FL.10 | GRA |
| hCoV-19/Morocco/IPM20436005/2023 | EPI_ISL_17982445 | 05/05/2023 | Africa / Morocco / Casablanca | Human | unknown | Female | 29 | unknown | unknown | unknown | XBB.1.16 | GRA |
| hCoV-19/Morocco/IPM20436002/2023 | EPI_ISL_17982444 | 04/05/2023 | Africa / Morocco / Casablanca | Human | unknown | Female | 24 | unknown | unknown | unknown | XBB.1.5 | GRA |
| hCoV-19/Morocco/IPM20436037/2023 | EPI_ISL_17982447 | 17/05/2023 | Africa / Morocco / Casablanca | Human | unknown | Male | 58 | unknown | unknown | unknown | FL.10 | GRA |
| hCoV-19/Morocco/IPM20435993/2023 | EPI_ISL_17797610 | 28/04/2023 | Africa / Morocco / Casablanca | Human | unknown | Female | 51 | unknown | unknown | unknown | XBB.1.9.1 | GRA |
| hCoV-19/Morocco/IPM20435990/2023 | EPI_ISL_17797609 | 28/04/2023 | Africa / Morocco / Casablanca | Human | unknown | Male | 72 | unknown | unknown | unknown | XBB.1.9.1 | GRA |
| hCoV-19/Morocco/IPM20435988/2023 | EPI_ISL_17797608 | 27/04/2023 | Africa / Morocco / Casablanca | Human | unknown | Female | 33 | unknown | unknown | unknown | XBB.1.9.1 | GRA |
| hCoV-19/Morocco/IPM20435987/2023 | EPI_ISL_17797607 | 27/04/2023 | Africa / Morocco / Casablanca | Human | unknown | Female | 68 | unknown | unknown | unknown | XBB.1.9.1 | GRA |
| hCoV-19/Morocco/IPM20435986/2023 | EPI_ISL_17797606 | 27/04/2023 | Africa / Morocco / Casablanca | Human | unknown | Female | 45 | unknown | unknown | unknown | XBB.1.9.1 | GRA |
| hCoV-19/Morocco/IPM20435983/2023 | EPI_ISL_17797604 | 26/04/2023 | Africa / Morocco / Casablanca | Human | unknown | Female | 46 | unknown | unknown | unknown | XBB.1.9.1 | GRA |
| hCoV-19/Morocco/IPM20435982/2023 | EPI_ISL_17797603 | 26/04/2023 | Africa / Morocco / Casablanca | Human | unknown | Female | 82 | unknown | unknown | unknown | XBB.1.9.1 | GRA |
| hCoV-19/env/Morocco/INH-18/2023 | EPI_ISL_17766985 | 14/03/2023 | Africa / Morocco / step El Yousofia | unknown | unknown | unknown | unknown | unknown | unknown | unknown | XBB.1.9.1 | GRA |
| hCoV-19/Morocco/NIH/1293/2023 | EPI_ISL_17699794 | 08/05/2023 | Africa / Morocco / Rabat | Human | unknown | Male | 32 years | Live | unknown | unknown | XBB.1.9.1 | GRA |
| hCoV-19/Morocco/NIH/1292/2023 | EPI_ISL_17699793 | 08/05/2023 | Africa / Morocco / Rabat | Human | unknown | Male | 65 years | Live | unknown | unknown | XBB.1.9.2 | GRA |
| hCoV-19/Morocco/NIH/1291/2023 | EPI_ISL_17699792 | 10/05/2023 | Africa / Morocco / Rabat | Human | unknown | Male | 48 Years | Live | unknown | unknown | XBB.1.9.2 | GRA |
| hCoV-19/Morocco/INH-1287/2023 | EPI_ISL_17660881 | 04/05/2023 | Africa / Morocco / Rabat | Human | unknown | Male | 58 | Live | unknown | unknown | XBB.1.9.1 | GRA |
| hCoV-19/Morocco/48/2021 | EPI_ISL_8308349 | 21/12/2021 | Africa / Morocco / Rabat | Human | unknown | Female | unknown | Live | unknown | unknown | AY.126 | GK |
| hCoV-19/Morocco/62/2021 | EPI_ISL_8308355 | 05/12/2021 | Africa / Morocco / Casablanca | Human | unknown | Female | 58 | Live | unknown | unknown | AY.122 | GK |
| hCoV-19/Morocco/53/2021 | EPI_ISL_8308357 | 04/12/2021 | Africa / Morocco / Casablanca | Human | unknown | Female | 40 | Live | unknown | unknown | AY.33 | GK |
| hCoV-19/Morocco/45/2021 | EPI_ISL_8308348 | 22/12/2021 | Africa / Morocco / Rabat | Human | unknown | Male | unknown | Live | unknown | unknown | B.1.617.2 | GK |
| hCoV-19/Morocco/46/2021 | EPI_ISL_8308350 | 22/12/2021 | Africa / Morocco / Casablanca | Human | unknown | Male | unknown | Live | unknown | unknown | B.1.617.2 | GK |
| hCoV-19/Morocco/20349663/2021 | EPI_ISL_4430715 | 23/08/2021 | Africa / Morocco / Casablanca | Human | unknown | Female | 16 | unknown | unknown | unknown | AY.33 | GK |
| hCoV-19/Morocco/20352116/2021 | EPI_ISL_4430745 | 25/08/2021 | Africa / Morocco / Casablanca | Human | unknown | Male | 40 | unknown | unknown | unknown | AY.33 | GK |
| hCoV-19/Morocco/20352418/2021 | EPI_ISL_4430749 | 26/08/2021 | Africa / Morocco / Casablanca | Human | unknown | Female | 39 | unknown | unknown | unknown | AY.33 | GK |
| hCoV-19/Morocco/20352711/2021 | EPI_ISL_4430758 | 26/08/2021 | Africa / Morocco / Casablanca | Human | unknown | Female | 22 | unknown | unknown | unknown | AY.73 | GK |
| hCoV-19/Morocco/INH-1294/2023 | EPI_ISL_18044673 | 20/07/2023 | Africa / Morocco / Rabat | Human | Sentinel surveillance (ILI) | Male | 53 | Live | unknown | Sentinel surveillance (ILI) | XBB.1.16 | GRA |
| hCoV-19/Morocco/MA-10-1-delta/2021 | EPI_ISL_18048770 | 22/10/2021 | Africa / Morocco / Rabat | unknown | unknown | unknown | unknown | unknown | unknown | unknown | AY.33 | GK |
| hCoV-19/Morocco/MA-8-3-6-delta/2021 | EPI_ISL_18048769 | 10/08/2021 | Africa / Morocco / Rabat | unknown | unknown | unknown | unknown | unknown | unknown | unknown | AY.33 | GK |
| hCoV-19/Morocco/MA-11-2-Jouh/2022 | EPI_ISL_18048737 | 29/11/2022 | Africa / Morocco / Rabat | unknown | unknown | unknown | unknown | unknown | unknown | unknown | BA.5.2.20 | GRA |
| hCoV-19/Morocco/MA-8-2-6-delta/2021 | EPI_ISL_18048768 | 06/08/2021 | Africa / Morocco / Rabat | unknown | unknown | unknown | unknown | unknown | unknown | unknown | AY.33 | GK |
| hCoV-19/Morocco/MA-10-2-delta/2021 | EPI_ISL_18048772 | 28/10/2021 | Africa / Morocco / Rabat | unknown | unknown | unknown | unknown | unknown | unknown | unknown | AY.33 | GK |
| hCoV-19/Morocco/MA-11-HMIMV-1-Jouh/2022 | EPI_ISL_18048712 | 22/11/2022 | Africa / Morocco / Rabat | unknown | unknown | unknown | unknown | unknown | unknown | unknown | BA.5.2.20 | GRA |
| hCoV-19/Morocco/ION_CODE_3/2021 | EPI_ISL_3155074 | 17/06/2021 | Africa / Morocco / Casablanca | Human | unknown | Female | 47 | Released | unknown | unknown | B.1.351 | GH |
| hCoV-19/Morocco/40CC/2021 | EPI_ISL_8920489 | 13/07/2021 | Africa / Morocco | Human | unknown | Male | unknown | Released | unknown | unknown | AY.33 | GK |
| hCoV-19/Morocco/INH-P641/2020 | EPI_ISL_18145898 | 17/04/2020 | Africa / Morocco / Sale | Human | unknown | Female | 73 | Live | unknown | unknown | B.1 | G |
| hCoV-19/Morocco/INH-P790/2020 | EPI_ISL_18145899 | 25/04/2020 | Africa / Morocco / Tanger | Human | unknown | Female | 25 | Live | unknown | unknown | B.1 | G |
| hCoV-19/Morocco/INH-P529/2020 | EPI_ISL_18145897 | 16/04/2020 | Africa / Morocco / Rabat | Human | unknown | Male | 78 | Live | unknown | unknown | B.1 | G |
| hCoV-19/Morocco/INH-P802/2020 | EPI_ISL_18145900 | 10/05/2020 | Africa / Morocco / Rabat | Human | unknown | Male | 68 | Live | unknown | unknown | B.1 | GH |
| hCoV-19/Morocco/INH-P911/2020 | EPI_ISL_18145901 | 11/05/2020 | Africa / Morocco / Meknes | Human | unknown | Male | 86 | Live | unknown | unknown | B.1 | G |
| hCoV-19/Morocco/INH-P769/2020 | EPI_ISL_18145915 | 24/04/2020 | Africa / Morocco / Rabat | Human | unknown | Male | 23 | Live | unknown | unknown | B.1.1 | GR |
| hCoV-19/Morocco/INH-P520/2020 | EPI_ISL_18145896 | 15/04/2020 | Africa / Morocco / Rabat | Human | unknown | Male | 47 | Live | unknown | unknown | B.39 | V |
| hCoV-19/Morocco/INH-P519/2020 | EPI_ISL_18145912 | 19/03/2020 | Africa / Morocco / Rabat | Human | unknown | Female | 27 | Live | unknown | unknown | B.1 | G |
| hCoV-19/Morocco/INH-P647/2020 | EPI_ISL_18145922 | 24/04/2020 | Africa / Morocco / Rabat | Human | unknown | Male | 47 | Live | unknown | unknown | B.1 | G |
| hCoV-19/Morocco/INH-P778/2020 | EPI_ISL_18145918 | 25/04/2020 | Africa / Morocco / Rabat | Human | unknown | Female | 41 | Live | unknown | unknown | B.1 | G |
| hCoV-19/Morocco/INH-P673/2020 | EPI_ISL_18145914 | 24/04/2020 | Africa / Morocco / Meknes | Human | unknown | Male | 78 | Live | unknown | unknown | B.1 | GH |
| hCoV-19/Morocco/INH-P779/2020 | EPI_ISL_18145920 | 25/04/2020 | Africa / Morocco / Tanger | Human | unknown | Female | 63 | Live | unknown | unknown | B.1 | GH |
| hCoV-19/Morocco/INH-P478/2020 | EPI_ISL_18145911 | 18/03/2020 | Africa / Morocco / Rabat | Human | unknown | Male | 32 | Live | unknown | unknown | B.1 | G |
| hCoV-19/Morocco/INH-P770/2020 | EPI_ISL_18145917 | 25/04/2020 | Africa / Morocco / Rabat | Human | unknown | Male | 34 | Live | unknown | unknown | B.1 | G |
| hCoV-19/Morocco/INH-P654/2020 | EPI_ISL_18145913 | 18/04/2020 | Africa / Morocco / Kenitra | Human | unknown | Female | 68 | Live | unknown | unknown | B.1 | G |
| hCoV-19/Morocco/INH-P885/2020 | EPI_ISL_18145925 | 28/04/2020 | Africa / Morocco / Fes | Human | unknown | Male | 69 | Live | unknown | unknown | B.1 | GH |
| hCoV-19/Morocco/INH-P806/2020 | EPI_ISL_18145924 | 28/04/2020 | Africa / Morocco / Meknes | Human | unknown | Female | 66 | Live | unknown | unknown | B.1 | GH |
| hCoV-19/Morocco/INH-P921/2020 | EPI_ISL_18145926 | 30/05/2020 | Africa / Morocco / Meknes | Human | unknown | Male | 68 | Live | unknown | unknown | B.1 | GH |
| hCoV-19/Morocco/INH-P484/2020 | EPI_ISL_18145923 | 19/03/2020 | Africa / Morocco / Rabat | Human | unknown | Female | 29 | Live | unknown | unknown | B.1 | G |
| hCoV-19/Morocco/INH-P783/2020 | EPI_ISL_18145919 | 25/04/2020 | Africa / Morocco / Rabat | Human | unknown | Male | 63 | Live | unknown | unknown | B.1 | G |
| hCoV-19/Morocco/INH-86/2021 | EPI_ISL_18145905 | 13/12/2021 | Africa / Morocco / Casablanca | Human | unknown | Male | 52 | Live | unknown | unknown | B.1.617.2 | GK |
| hCoV-19/Morocco/INH-P671/2020 | EPI_ISL_18145902 | 24/04/2020 | Africa / Morocco / Meknes | Human | unknown | Male | 68 | Live | unknown | unknown | B.1 | GH |
| hCoV-19/Morocco/INH-P943/2020 | EPI_ISL_18145903 | 20/05/2020 | Africa / Morocco / Meknes | Human | unknown | Female | 67 | Live | unknown | unknown | B.1 | GH |
| hCoV-19/Morocco/INH-P487/2020 | EPI_ISL_18145921 | 15/03/2020 | Africa / Morocco / Rabat | Human | unknown | Male | 47 | Live | unknown | unknown | A.5 | S |
| hCoV-19/Morocco/INH-84/2021 | EPI_ISL_18145904 | 11/12/2021 | Africa / Morocco / Casablanca | Human | unknown | Female | 38 | Live | unknown | unknown | AY.112 | GK |
| hCoV-19/Morocco/INH-95/2021 | EPI_ISL_18145909 | 17/12/2021 | Africa / Morocco / Casablanca | Human | unknown | Male | 47 | Live | unknown | unknown | AY.33 | GK |
| hCoV-19/Morocco/INH-87/2021 | EPI_ISL_18145906 | 13/12/2021 | Africa / Morocco / Casablanca | Human | unknown | Female | 63 | Live | unknown | unknown | AY.33 | GK |
| hCoV-19/Morocco/INH-80/2021 | EPI_ISL_18145907 | 14/12/2021 | Africa / Morocco / Casablanca | Human | unknown | Female | 40 | Live | unknown | unknown | B.1.617.2 | GK |
| hCoV-19/Morocco/INH-85/2021 | EPI_ISL_18145908 | 14/12/2021 | Africa / Morocco / Casablanca | Human | unknown | Male | 61 | Live | unknown | unknown | AY.112 | GK |
| hCoV-19/Morocco/INH-P677/2020 | EPI_ISL_18145916 | 25/04/2020 | Africa / Morocco / Taourirt | Human | unknown | Female | 65 | Live | unknown | unknown | B.1 | GH |
| hCoV-19/Morocco/INH-92/2021 | EPI_ISL_18145910 | 18/12/2021 | Africa / Morocco / Casablanca | Human | unknown | Female | 53 | Live | unknown | unknown | AY.5 | GK |
| hCoV-19/Morocco/20352059/2021 | EPI_ISL_4511387 | 25/08/2021 | Africa / Morocco / Casablanca | Human | unknown | Male | 51 | unknown | unknown | unknown | B.1.617.2 | GK |
| hCoV-19/Morocco/RA47/2021 | EPI_ISL_3231138 | 01/03/2021 | Africa / Morocco | Human | unknown | Male | 25 | unknown | unknown | unknown | B.1.1.7 | GR |
| hCoV-19/Morocco/FMP-386/2021 | EPI_ISL_8543192 | 2021 | Africa / Morocco / Fes | Human | unknown | unknown | unknown | unknown | unknown | unknown | AY.33 | GK |
| hCoV-19/Morocco/NIH-1169/2022 | EPI_ISL_16201193 | 02/11/2022 | Africa / Morocco / Rabat | Human | unknown | Male | 20 | Live | unknown | unknown | BQ.1.1.69 | GRA |
| hCoV-19/Morocco/FMP-377/2021 | EPI_ISL_13961937 | 21/10/2021 | Africa / Morocco | Human | unknown | Female | 41 | unknown | unknown | unknown | AY.112 | GK |
| hCoV-19/Morocco/FMP381/2021 | EPI_ISL_13961936 | 27/10/2021 | Africa / Morocco | Human | unknown | Male | 44 | unknown | unknown | unknown | AY.73 | GK |
| hCoV-19/Morocco/FMP362/2021 | EPI_ISL_13961935 | 05/10/2021 | Africa / Morocco | Human | unknown | Female | 30 | unknown | unknown | unknown | AY.33 | GK |
| hCoV-19/Morocco/FMP-431/2021 | EPI_ISL_13961934 | 28/12/2021 | Africa / Morocco | Human | unknown | Male | 59 | unknown | unknown | unknown | BA.1 | GRA |
| hCoV-19/Morocco/FMP424/2021 | EPI_ISL_13961933 | 28/12/2021 | Africa / Morocco | Human | unknown | Female | 25 | unknown | unknown | unknown | BA.1 | GRA |
| hCoV-19/Morocco/FMP447/2021 | EPI_ISL_13961859 | 28/12/2021 | Africa / Morocco | Human | unknown | Female | 48 | unknown | unknown | unknown | BA.1 | GRA |
| hCoV-19/Morocco/RA212/2021 | EPI_ISL_3259350 | 20/06/2021 | Africa / Morocco | Human | Active surveillance | Male | unknown | Released | unknown | Active surveillance | B.1.1.7 | GR |
| hCoV-19/Morocco/FMP446/2021 | EPI_ISL_13961177 | 28/12/2021 | Africa / Morocco | Human | unknown | Female | 62 | unknown | unknown | unknown | BA.1 | GRA |
| hCoV-19/Morocco/FMP-325/2021 | EPI_ISL_4572255 | 13/07/2021 | Africa / Morocco / Agadir | Human | unknown | Male | unknown | unknown | unknown | unknown | AY.112 | GK |
| hCoV-19/Morocco/FMP321-90/2021 | EPI_ISL_4572229 | 10/07/2021 | Africa / Morocco / Inezgane | Human | unknown | Female | unknown | unknown | unknown | unknown | AY.33 | GK |
| hCoV-19/Morocco/FMP-338/2021 | EPI_ISL_4572736 | 13/07/2021 | Africa / Morocco / Chtouka | Human | unknown | Male | unknown | unknown | unknown | unknown | AY.33 | GK |
| hCoV-19/Morocco/FMP-337/2021 | EPI_ISL_4572536 | 13/07/2021 | Africa / Morocco / Agadir | Human | unknown | Male | unknown | unknown | unknown | unknown | AY.73 | GK |
| hCoV-19/Morocco/OUA11/2020 | EPI_ISL_4300209 | 23/04/2020 | Africa / Morocco | Human | unknown | unknown | unknown | unknown | unknown | unknown | B.1 | G |
| hCoV-19/Morocco/FMP-339/2021 | EPI_ISL_4572814 | 13/07/2021 | Africa / Morocco / Tiznit | Human | unknown | Male | unknown | unknown | unknown | unknown | AY.33 | GK |
| hCoV-19/Morocco/IPM20383466/2021 | EPI_ISL_8186746 | 16/11/2021 | Africa / Morocco / Mohammadia | Human | unknown | Female | 62 | unknown | unknown | unknown | AY.73 | GK |
| hCoV-19/Morocco/165/2021 | EPI_ISL_8144256 | 25/12/2021 | Africa / Morocco / Tanger | Human | unknown | Female | unknown | Live | unknown | unknown | AY.98 | GK |
| hCoV-19/Morocco/IPM20378155/2021 | EPI_ISL_6332505 | 18/10/2021 | Africa / Morocco / Casablanca | Human | unknown | Female | 29 | unknown | unknown | unknown | AY.33 | GK |
| hCoV-19/Morocco/IPM20378322/2021 | EPI_ISL_6332509 | 18/10/2021 | Africa / Morocco / Casablanca | Human | unknown | Female | 68 | unknown | unknown | unknown | AY.112 | GK |
| hCoV-19/Morocco/IPM20378661/2021 | EPI_ISL_6332512 | 21/10/2021 | Africa / Morocco / Mohammadia | Human | unknown | Female | 33 | unknown | unknown | unknown | AY.33 | GK |
| hCoV-19/Morocco/FMP408/2021 | EPI_ISL_13947433 | 28/12/2021 | Africa / Morocco | Human | unknown | Male | 51 | unknown | unknown | unknown | BA.1 | GRA |
| hCoV-19/Morocco/IPM20378767/2021 | EPI_ISL_6332513 | 21/10/2021 | Africa / Morocco / Casablanca | Human | unknown | Male | 57 | unknown | unknown | unknown | AY.112 | GK |
| hCoV-19/Morocco/RMPS-18/2020 | EPI_ISL_728222 | 01/04/2020 | Africa / Morocco | Human | unknown | Male | unknown | unknown | unknown | unknown | Unassigned | GH |
| hCoV-19/Morocco/RMPS-19/2020 | EPI_ISL_728223 | 01/04/2020 | Africa / Morocco | Human | unknown | Male | unknown | unknown | unknown | unknown | Unassigned | GH |
| hCoV-19/Morocco/FMP-102/2021 | EPI_ISL_5123044 | 2021-02 | Africa / Morocco | Human | unknown | unknown | unknown | unknown | unknown | unknown | Unassigned | G |
| hCoV-19/Morocco/Fes86/2021 | EPI_ISL_4300282 | 20/06/2021 | Africa / Morocco | Human | unknown | Male | unknown | unknown | unknown | unknown | Unassigned | G |
| hCoV-19/Morocco/FMP-193/2021 | EPI_ISL_1895101 | 22/03/2021 | Africa / Morocco / Rabat | Human | unknown | Male | 69 | unknown | unknown | unknown | B.1.1.7 | GRY |
| hCoV-19/Morocco/FMP-204/2021 | EPI_ISL_1904297 | 16/03/2021 | Africa / Morocco / Sidi Lahcen | Human | unknown | Female | 22 | unknown | unknown | unknown | B.1.1.7 | GRY |
| hCoV-19/Morocco/FMP-231/2021 | EPI_ISL_1904692 | 23/03/2021 | Africa / Morocco / Rabat | Human | unknown | Male | 45 | unknown | unknown | unknown | B.1.1.7 | GRY |
| hCoV-19/Morocco/FMP-207/2021 | EPI_ISL_1904298 | 19/03/2021 | Africa / Morocco / Sidi Lahcen | Human | unknown | Male | 16 | unknown | unknown | unknown | B.1.1.7 | GRY |
| hCoV-19/Morocco/OUA677_19/2020 | EPI_ISL_458287 | 23/04/2020 | Africa / Morocco / Rabat | Human | unknown | unknown | unknown | unknown | unknown | unknown | B.1.528 | G |
| hCoV-19/Morocco/FMP-252/2021 | EPI_ISL_1904885 | 23/03/2021 | Africa / Morocco / Rabat | Human | unknown | Male | 44 | unknown | unknown | unknown | B.1.1.7 | GRY |
| hCoV-19/Morocco/FMP-232/2021 | EPI_ISL_1904846 | 22/03/2021 | Africa / Morocco / Sidi Lahcen | Human | unknown | Female | 69 | unknown | unknown | unknown | B.1.1.7 | GRY |
| hCoV-19/Morocco/FMP-237/2021 | EPI_ISL_1904875 | 18/03/2021 | Africa / Morocco / Rabat | Human | unknown | Female | 53 | unknown | unknown | unknown | B.1.1.7 | GRY |
| hCoV-19/Morocco/FMP-280/2021 | EPI_ISL_1905060 | 23/03/2021 | Africa / Morocco | Human | unknown | Female | 68 | unknown | unknown | unknown | B.1.1.7 | GRY |
| hCoV-19/Morocco/FMP-256/2021 | EPI_ISL_1904887 | 20/03/2021 | Africa / Morocco / Khemisset | Human | unknown | Male | 23 | unknown | unknown | unknown | B.1.1.7 | GRY |
| hCoV-19/Morocco/FMP-249/2021 | EPI_ISL_1904877 | 15/03/2021 | Africa / Morocco / Rabat | Human | unknown | Female | 58 | unknown | unknown | unknown | B.1.1.7 | GRY |
| hCoV-19/Morocco/FMP-255/2021 | EPI_ISL_1904886 | 16/03/2021 | Africa / Morocco / sidi Lahcen | Human | unknown | Female | 43 | unknown | unknown | unknown | B.1.1.7 | GRY |
| hCoV-19/Morocco/RMPS-13/2020 | EPI_ISL_482738 | 10/04/2020 | Africa / Morocco | Human | unknown | unknown | unknown | unknown | unknown | unknown | B.1 | GH |
| hCoV-19/Morocco/20356985/2021 | EPI_ISL_4741165 | 01/09/2021 | Africa / Morocco / Casablanca | Human | unknown | Male | 37 | unknown | unknown | unknown | B.1.617.2 | GK |
| hCoV-19/Morocco/20355935/2021 | EPI_ISL_4741159 | 31/08/2021 | Africa / Morocco / Casablanca | Human | unknown | Male | 56 | unknown | unknown | unknown | AY.112 | GK |
| hCoV-19/Morocco/20357851/2021 | EPI_ISL_4741166 | 02/09/2021 | Africa / Morocco / Casablanca | Human | unknown | Male | 65 | unknown | unknown | unknown | AY.33 | GK |
| hCoV-19/Morocco/20358215/2021 | EPI_ISL_4741168 | 03/09/2021 | Africa / Morocco / Casablanca | Human | unknown | Female | 50 | unknown | unknown | unknown | AY.46 | GK |
| hCoV-19/Morocco/20358089/2021 | EPI_ISL_4741167 | 03/09/2021 | Africa / Morocco / Casablanca | Human | unknown | Female | 61 | unknown | unknown | unknown | AY.33 | GK |
| hCoV-19/Morocco/20358496/2021 | EPI_ISL_4741169 | 03/09/2021 | Africa / Morocco / Casablanca | Human | unknown | Female | 48 | unknown | unknown | unknown | B.1.617.2 | GK |
| hCoV-19/Morocco/20358732/2021 | EPI_ISL_4741170 | 03/09/2021 | Africa / Morocco / Casablanca | Human | unknown | Female | 71 | unknown | unknown | unknown | AY.33 | GK |
| hCoV-19/Morocco/20358839/2021 | EPI_ISL_4741171 | 03/09/2021 | Africa / Morocco / Casablanca | Human | unknown | Male | 25 | unknown | unknown | unknown | AY.33 | GK |
| hCoV-19/Morocco/20358915/2021 | EPI_ISL_4741172 | 03/09/2021 | Africa / Morocco / Casablanca | Human | unknown | Male | 52 | unknown | unknown | unknown | AY.33 | GK |
| hCoV-19/Morocco/FMP-259/2021 | EPI_ISL_1908148 | 16/04/2021 | Africa / Morocco / Kenitra | Human | unknown | unknown | unknown | unknown | unknown | unknown | B.1.1.7 | GRY |
| hCoV-19/Morocco/FMP-258/2021 | EPI_ISL_1908147 | 03/04/2021 | Africa / Morocco / Kenitra | Human | unknown | unknown | unknown | unknown | unknown | unknown | B.1.1.7 | GRY |
| hCoV-19/Morocco/FMP-266/2021 | EPI_ISL_1908787 | 08/04/2021 | Africa / Morocco / Sidi Kacem | Human | unknown | unknown | unknown | unknown | unknown | unknown | B.1.1.7 | GRY |
| hCoV-19/Morocco/FMP-267/2021 | EPI_ISL_1908805 | 08/04/2021 | Africa / Morocco / Sidi Kacem | Human | unknown | unknown | unknown | unknown | unknown | unknown | B.1.1.7 | GRY |
| hCoV-19/Morocco/FMP-268/2021 | EPI_ISL_1908896 | 08/04/2021 | Africa / Morocco / Sidi Kacem | Human | unknown | unknown | unknown | unknown | unknown | unknown | B.1.1.7 | GRY |
| hCoV-19/Morocco/FMP-261/2021 | EPI_ISL_1908153 | 2021 | Africa / Morocco / Casablanca | Human | unknown | unknown | unknown | unknown | unknown | unknown | B.1.1.7 | GRY |
| hCoV-19/Morocco/RA6/2021 | EPI_ISL_9020397 | 03/12/2021 | Africa / Morocco | Human | unknown | unknown | unknown | Released | unknown | unknown | AY.33 | GK |
| hCoV-19/Morocco/FMP-257/2021 | EPI_ISL_1907961 | 03/04/2021 | Africa / Morocco / Kenitra | Human | unknown | unknown | unknown | unknown | unknown | unknown | B.1.1.7 | GRY |
| hCoV-19/Morocco/FMP-275/2021 | EPI_ISL_1909241 | 16/04/2021 | Africa / Morocco / Casablanca | Human | unknown | unknown | unknown | unknown | unknown | unknown | B.1.1.7 | GRY |
| hCoV-19/Morocco/FMP-161/2021 | EPI_ISL_1909253 | 27/02/2021 | Africa / Morocco / Casablanca | Human | unknown | unknown | unknown | unknown | unknown | unknown | B.1.1.7 | GRY |
| hCoV-19/Morocco/FMP-286/2021 | EPI_ISL_1909251 | 22/04/2021 | Africa / Morocco / Casablanca | Human | unknown | unknown | unknown | unknown | unknown | unknown | B.1.1.7 | GRY |
| hCoV-19/Morocco/FMP-273/2021 | EPI_ISL_1909211 | 06/04/2021 | Africa / Morocco / Kenitra | Human | unknown | unknown | unknown | unknown | unknown | unknown | B.1.1.7 | GRY |
| hCoV-19/Morocco/FMP-270/2021 | EPI_ISL_1909088 | 10/04/2021 | Africa / Morocco / Casablanca | Human | unknown | unknown | unknown | unknown | unknown | unknown | B.1.1.7 | GRY |
| hCoV-19/Morocco/FMP-285/2021 | EPI_ISL_1909250 | 09/04/2021 | Africa / Morocco / Casablanca | Human | unknown | unknown | unknown | unknown | unknown | unknown | B.1.1.7 | GRY |
| hCoV-19/Morocco/FMP-281/2021 | EPI_ISL_1909246 | 03/04/2021 | Africa / Morocco / Kenitra | Human | unknown | unknown | unknown | unknown | unknown | unknown | B.1.1.7 | G |
| hCoV-19/Morocco/FMP-167/2021 | EPI_ISL_1913005 | 2021 | Africa / Morocco / Casablanca | Human | unknown | unknown | unknown | unknown | unknown | unknown | B.1.1.7 | GRY |
| hCoV-19/Morocco/FMP-215/2021 | EPI_ISL_1913057 | 25/03/2021 | Africa / Morocco / Dakhla | Human | unknown | unknown | unknown | unknown | unknown | unknown | B.1.1.7 | GRY |
| hCoV-19/Morocco/FMP-175/2021 | EPI_ISL_1913024 | 24/02/2021 | Africa / Morocco / Casablanca | Human | unknown | unknown | unknown | unknown | unknown | unknown | B.1.1.7 | GRY |
| hCoV-19/Morocco/FMP-180/2021 | EPI_ISL_1913032 | 2021 | Africa / Morocco / Casablanca | Human | unknown | unknown | unknown | unknown | unknown | unknown | B.1.1.7 | GRY |
| hCoV-19/Morocco/FMP-220/2021 | EPI_ISL_1913067 | 14/03/2021 | Africa / Morocco / Casablanca | Human | unknown | unknown | unknown | unknown | unknown | unknown | B.1.1.7 | GRY |
| hCoV-19/Morocco/IPM20400733/2022 | EPI_ISL_10070715 | 10/01/2022 | Africa / Morocco / Casablanca | Human | unknown | Male | 68 | unknown | unknown | unknown | BA.1 | GRA |
| hCoV-19/Morocco/IPM20400729/2022 | EPI_ISL_10070712 | 10/01/2022 | Africa / Morocco / Casablanca | Human | unknown | Male | 52 | unknown | unknown | unknown | B.1.617.2 | GK |
| hCoV-19/Morocco/IPM20400732/2022 | EPI_ISL_10070714 | 10/01/2022 | Africa / Morocco / Casablanca | Human | unknown | Male | 79 | unknown | unknown | unknown | BA.1 | GRA |
| hCoV-19/Morocco/IPM20402116/2022 | EPI_ISL_10070722 | 12/01/2022 | Africa / Morocco / Casablanca | Human | unknown | Female | 51 | unknown | unknown | unknown | BA.1 | GRA |
| hCoV-19/Morocco/IPM20405836/2022 | EPI_ISL_10070735 | 20/01/2022 | Africa / Morocco / Casablanca | Human | unknown | Male | 81 | unknown | unknown | unknown | B.1.617.2 | GK |
| hCoV-19/Morocco/IPM20401715/2022 | EPI_ISL_10070720 | 12/01/2022 | Africa / Morocco / Casablanca | Human | unknown | Male | 66 | unknown | unknown | unknown | B.1.617.2 | GK |
| hCoV-19/Morocco/IPM20405837/2022 | EPI_ISL_10070736 | 20/01/2022 | Africa / Morocco / Casablanca | Human | unknown | Male | 62 | unknown | unknown | unknown | BA.1 | GRA |
| hCoV-19/Morocco/IPM20404451/2022 | EPI_ISL_10070727 | 18/01/2022 | Africa / Morocco / Casablanca | Human | unknown | Male | 83 | unknown | unknown | unknown | BA.1 | GRA |
| hCoV-19/Morocco/IPM20406691/2022 | EPI_ISL_10070738 | 21/01/2022 | Africa / Morocco / Casablanca | Human | unknown | Female | 82 | unknown | unknown | unknown | AY.43 | GK |
| hCoV-19/Morocco/IPM20406693/2022 | EPI_ISL_10070739 | 21/01/2022 | Africa / Morocco / Casablanca | Human | unknown | Male | 80 | unknown | unknown | unknown | B.1.617.2 | GK |
| hCoV-19/Morocco/IPM20404452/2022 | EPI_ISL_10070728 | 18/01/2022 | Africa / Morocco / Casablanca | Human | unknown | Male | 67 | unknown | unknown | unknown | BA.1 | GRA |
| hCoV-19/Morocco/IPM20405835/2022 | EPI_ISL_10070734 | 20/01/2022 | Africa / Morocco / Casablanca | Human | unknown | Male | 80 | unknown | unknown | unknown | BA.1 | GRA |
| hCoV-19/Morocco/FMP-242/2021 | EPI_ISL_1913590 | 2021 | Africa / Morocco / Tinghir | Human | unknown | unknown | unknown | unknown | unknown | unknown | B.1.1.7 | GRY |
| hCoV-19/Morocco/FMP-385/2021 | EPI_ISL_8543177 | 2021 | Africa / Morocco / Rabat | Human | unknown | unknown | unknown | unknown | unknown | unknown | BA.1 | GRA |
| hCoV-19/Morocco/549/2022 | EPI_ISL_10260257 | 11/01/2022 | Africa / Morocco / Rabat | Human | unknown | Female | unknown | Live | unknown | unknown | BA.1 | GRA |
| hCoV-19/Morocco/414/2022 | EPI_ISL_10019539 | 03/01/2022 | Africa / Morocco / Rabat | Human | unknown | Female | unknown | Live - Intensive Care Unit | unknown | unknown | BA.1 | GRA |
| hCoV-19/Morocco/538/2022 | EPI_ISL_10260255 | 13/01/2022 | Africa / Morocco / Rabat | Human | unknown | Female | 86 | Live | unknown | unknown | BA.1 | GRA |
| hCoV-19/Morocco/IPM20435984/2023 | EPI_ISL_17797605 | 26/04/2023 | Africa / Morocco / Casablanca | Human | unknown | Female | 58 | unknown | unknown | unknown | XBB.1.9.1 | GRA |
| hCoV-19/Morocco/IPM20422438/2022 | EPI_ISL_13695674 | 23/06/2022 | Africa / Morocco / Casablanca | Human | unknown | unknown | unknown | unknown | unknown | unknown | BA.2.40.1 | GRA |
| hCoV-19/Morocco/IPM20422343/2022 | EPI_ISL_13695672 | 23/06/2022 | Africa / Morocco / Casablanca | Human | unknown | unknown | unknown | unknown | unknown | unknown | BA.5.2.1 | GRA |
| hCoV-19/Morocco/IPM20422334/2022 | EPI_ISL_13695671 | 23/06/2022 | Africa / Morocco / Casablanca | Human | unknown | unknown | unknown | unknown | unknown | unknown | BA.5.2.20 | GRA |
| hCoV-19/Morocco/IPM20422329/2022 | EPI_ISL_13695669 | 23/06/2022 | Africa / Morocco / Casablanca | Human | unknown | unknown | unknown | unknown | unknown | unknown | BA.5.2 | GRA |
| hCoV-19/Morocco/IPM20419754/2022 | EPI_ISL_13695668 | 08/06/2022 | Africa / Morocco / Casablanca | Human | unknown | unknown | unknown | unknown | unknown | unknown | BA.2.9.3 | GRA |
| hCoV-19/Morocco/IPM20419608/2022 | EPI_ISL_13695667 | 07/06/2022 | Africa / Morocco / Casablanca | Human | unknown | unknown | unknown | unknown | unknown | unknown | BA.2.9 | GRA |
| hCoV-19/Morocco/IPM20419540/2022 | EPI_ISL_13695666 | 06/06/2022 | Africa / Morocco / Casablanca | Human | unknown | unknown | unknown | unknown | unknown | unknown | BA.5.2.1 | GRA |
| hCoV-19/Morocco/IPM20422415/2022 | EPI_ISL_13695665 | 23/06/2022 | Africa / Morocco / Casablanca | Human | unknown | Female | 72 | unknown | unknown | unknown | BA.5.2 | GRA |
| hCoV-19/Morocco/IPM20422447/2022 | EPI_ISL_13695664 | 23/06/2022 | Africa / Morocco / Casablanca | Human | unknown | Male | 71 | unknown | unknown | unknown | BA.5.2.20 | GRA |
| hCoV-19/Morocco/IPM20422450/2022 | EPI_ISL_13695661 | 23/06/2022 | Africa / Morocco / Casablanca | Human | unknown | Female | 62 | unknown | unknown | unknown | BA.5.2 | GRA |
| hCoV-19/Morocco/IPM20419708/2022 | EPI_ISL_13695660 | 08/06/2022 | Africa / Morocco / Casablanca | Human | unknown | Male | 61 | unknown | unknown | unknown | BA.5.2 | GRA |
| hCoV-19/Morocco/IPM20419630/2022 | EPI_ISL_13695659 | 08/06/2022 | Africa / Morocco / Casablanca | Human | unknown | Female | 60 | unknown | unknown | unknown | BA.2 | GRA |
| hCoV-19/Morocco/IPM20419566/2022 | EPI_ISL_13695658 | 07/06/2022 | Africa / Morocco / Casablanca | Human | unknown | Female | 60 | unknown | unknown | unknown | BA.2.12.1 | GRA |
| hCoV-19/Morocco/IPM20419563/2022 | EPI_ISL_13695657 | 07/06/2022 | Africa / Morocco / Casablanca | Human | unknown | Male | 60 | unknown | unknown | unknown | BA.5.2 | GRA |
| hCoV-19/Morocco/IPM20419778/2022 | EPI_ISL_13695656 | 09/06/2022 | Africa / Morocco / Casablanca | Human | unknown | Female | 58 | unknown | unknown | unknown | BA.5.2.1 | GRA |
| hCoV-19/Morocco/IPM20422384/2022 | EPI_ISL_13695655 | 23/06/2022 | Africa / Morocco / Casablanca | Human | unknown | Female | 55 | unknown | unknown | unknown | BA.5.2.20 | GRA |
| hCoV-19/Morocco/IPM20419712/2022 | EPI_ISL_13695654 | 08/06/2022 | Africa / Morocco / Casablanca | Human | unknown | Male | 53 | unknown | unknown | unknown | BA.5.2 | GRA |
| hCoV-19/Morocco/IPM20419604/2022 | EPI_ISL_13695652 | 07/06/2022 | Africa / Morocco / Casablanca | Human | unknown | Female | 51 | unknown | unknown | unknown | BA.5.2 | GRA |
| hCoV-19/Morocco/IPM20422418/2022 | EPI_ISL_13695651 | 23/06/2022 | Africa / Morocco / Casablanca | Human | unknown | Male | 47 | unknown | unknown | unknown | BA.5.2 | GRA |
| hCoV-19/Morocco/IPM20419713/2022 | EPI_ISL_13695650 | 08/06/2022 | Africa / Morocco / Casablanca | Human | unknown | Male | 47 | unknown | unknown | unknown | BA.5.2 | GRA |
| hCoV-19/Morocco/IPM20419784/2022 | EPI_ISL_13695649 | 09/06/2022 | Africa / Morocco / Casablanca | Human | unknown | Female | 46 | unknown | unknown | unknown | BA.2.12.1 | GRA |
| hCoV-19/Morocco/IPM20422431/2022 | EPI_ISL_13695648 | 23/06/2022 | Africa / Morocco / Casablanca | Human | unknown | Male | 45 | unknown | unknown | unknown | BA.5.2.20 | GRA |
| hCoV-19/Morocco/IPM20422416/2022 | EPI_ISL_13695647 | 23/06/2022 | Africa / Morocco / Casablanca | Human | unknown | Female | 45 | unknown | unknown | unknown | BA.5.2 | GRA |
| hCoV-19/Morocco/IPM20419574/2022 | EPI_ISL_13695646 | 07/06/2022 | Africa / Morocco / Casablanca | Human | unknown | Male | 42 | unknown | unknown | unknown | BA.5.2 | GRA |
| hCoV-19/Morocco/IPM20419711/2022 | EPI_ISL_13695645 | 08/06/2022 | Africa / Morocco / Casablanca | Human | unknown | Female | 39 | unknown | unknown | unknown | BA.5.2 | GRA |
| hCoV-19/Morocco/IPM20422428/2022 | EPI_ISL_13695644 | 23/06/2022 | Africa / Morocco / Casablanca | Human | unknown | Female | 38 | unknown | unknown | unknown | BA.5.2 | GRA |
| hCoV-19/Morocco/IPM20419767/2022 | EPI_ISL_13695643 | 09/06/2022 | Africa / Morocco / Casablanca | Human | unknown | Male | 38 | unknown | unknown | unknown | BA.5.2 | GRA |
| hCoV-19/Morocco/IPM20419559/2022 | EPI_ISL_13695641 | 07/06/2022 | Africa / Morocco / Casablanca | Human | unknown | Female | 33 | unknown | unknown | unknown | BA.5.2.20 | GRA |
| hCoV-19/Morocco/IPM20422422/2022 | EPI_ISL_13695640 | 23/06/2022 | Africa / Morocco / Casablanca | Human | unknown | Male | 30 | unknown | unknown | unknown | BA.2.40.1 | GRA |
| hCoV-19/Morocco/IPM20419725/2022 | EPI_ISL_13695639 | 08/06/2022 | Africa / Morocco / Casablanca | Human | unknown | Male | 28 | unknown | unknown | unknown | BA.2.11 | GRA |
| hCoV-19/Morocco/IPM20419615/2022 | EPI_ISL_13695637 | 08/06/2022 | Africa / Morocco / Mohammedia | Human | unknown | Female | 27 | unknown | unknown | unknown | BA.2.9.3 | GRA |
| hCoV-19/Morocco/IPM20419587/2022 | EPI_ISL_13695636 | 07/06/2022 | Africa / Morocco / Casablanca | Human | unknown | Male | 27 | unknown | unknown | unknown | BA.5.2 | GRA |
| hCoV-19/Morocco/IPM20419558/2022 | EPI_ISL_13695635 | 07/06/2022 | Africa / Morocco / Casablanca | Human | unknown | Male | 26 | unknown | unknown | unknown | BA.5.2.1 | GRA |
| hCoV-19/Morocco/IPM20422391/2022 | EPI_ISL_13695634 | 23/06/2022 | Africa / Morocco / Casablanca | Human | unknown | Female | 23 | unknown | unknown | unknown | BA.5.2 | GRA |
| hCoV-19/Morocco/IPM20422452/2022 | EPI_ISL_13695633 | 23/06/2022 | Africa / Morocco / Casablanca | Human | unknown | Female | 22 | unknown | unknown | unknown | BA.5.2 | GRA |
| hCoV-19/Morocco/IPM20422362/2022 | EPI_ISL_13695632 | 23/06/2022 | Africa / Morocco / Casablanca | Human | unknown | Male | 22 | unknown | unknown | unknown | BA.5.2.20 | GRA |
| hCoV-19/Morocco/IPM20422381/2022 | EPI_ISL_13695629 | 23/06/2022 | Africa / Morocco / Casablanca | Human | unknown | Male | 15 | unknown | unknown | unknown | BA.5.1 | GRA |
| hCoV-19/Morocco/RA42C/2021 | EPI_ISL_8178768 | 04/07/2021 | Africa / Morocco | Human | Active surveillance | Male | unknown | Released | unknown | Active surveillance | B.1.621 | GH |
| hCoV-19/Morocco/154/2021 | EPI_ISL_8144255 | 22/12/2021 | Africa / Morocco / Rabat | Human | unknown | Male | unknown | Live | unknown | unknown | BA.1 | GRA |
| hCoV-19/Morocco/IPM20419612/2022 | EPI_ISL_13695630 | 08/06/2022 | Africa / Morocco / Casablanca | Human | unknown | Female | 17 | unknown | unknown | unknown | BA.5.2.20 | GRA |
| hCoV-19/Morocco/IPM20419531/2022 | EPI_ISL_13695638 | 06/06/2022 | Africa / Morocco / Casablanca | Human | unknown | Male | 27 | unknown | unknown | unknown | BA.2.11 | GRA |
| hCoV-19/Morocco/IPM20419599/2022 | EPI_ISL_13695631 | 07/06/2022 | Africa / Morocco / Casablanca | Human | unknown | Female | 19 | unknown | unknown | unknown | BA.5.2 | GRA |
| hCoV-19/Morocco/IPM20419530/2022 | EPI_ISL_13695628 | 06/06/2022 | Africa / Morocco / Casablanca | Human | unknown | Male | 11 | unknown | unknown | unknown | BA.2.12.1 | GRA |
| hCoV-19/Morocco/IPM20422344/2022 | EPI_ISL_13695673 | 23/06/2022 | Africa / Morocco / Casablanca | Human | unknown | unknown | unknown | unknown | unknown | unknown | BA.5.2 | GRA |
| hCoV-19/Morocco/IPM20422459/2022 | EPI_ISL_13695663 | 23/06/2022 | Africa / Morocco / Casablanca | Human | unknown | Female | 69 | unknown | unknown | unknown | BA.5.2.20 | GRA |
| hCoV-19/Morocco/IPM20419520/2022 | EPI_ISL_13526818 | 06/06/2022 | Africa / Morocco / Casablanca | Human | unknown | Female | 36 | unknown | unknown | unknown | BA.2 | GRA |
| hCoV-19/Morocco/IPM202266/2022 | EPI_ISL_13526820 | 06/06/2022 | Africa / Morocco / Casablanca | Human | unknown | Male | unknown | unknown | unknown | unknown | BA.5.2 | GRA |
| hCoV-19/Morocco/IPM202265/2022 | EPI_ISL_13526819 | 06/06/2022 | Africa / Morocco / Casablanca | Human | unknown | Female | unknown | unknown | unknown | unknown | BA.5.2 | GRA |
| hCoV-19/Morocco/IPM20419494/2022 | EPI_ISL_13526817 | 06/06/2022 | Africa / Morocco / Casablanca | Human | unknown | Female | 17 | unknown | unknown | unknown | BA.2 | GRA |
| hCoV-19/Morocco/IPM20419455/2022 | EPI_ISL_13526816 | 06/06/2022 | Africa / Morocco / Casablanca | Human | unknown | unknown | unknown | unknown | unknown | unknown | BA.2 | GRA |
| hCoV-19/Morocco/IPM20419411/2022 | EPI_ISL_13526815 | 03/06/2022 | Africa / Morocco / Casablanca | Human | unknown | Male | 33 | unknown | unknown | unknown | BA.5.2 | GRA |
| hCoV-19/Morocco/IPM20419404/2022 | EPI_ISL_13526814 | 03/06/2022 | Africa / Morocco / Casablanca | Human | unknown | Male | 61 | unknown | unknown | unknown | BA.5.2 | GRA |
| hCoV-19/Morocco/IPM20419318/2022 | EPI_ISL_13526812 | 02/06/2022 | Africa / Morocco / Casablanca | Human | unknown | Female | 46 | unknown | unknown | unknown | BA.5.2 | GRA |
| hCoV-19/Morocco/IPM20419304/2022 | EPI_ISL_13526811 | 02/06/2022 | Africa / Morocco / Casablanca | Human | unknown | Female | 36 | unknown | unknown | unknown | BA.5.2.1 | GRA |
| hCoV-19/Morocco/IPM20419271/2022 | EPI_ISL_13526810 | 01/06/2022 | Africa / Morocco / Casablanca | Human | unknown | Female | 70 | unknown | unknown | unknown | BA.2.11 | GRA |
| hCoV-19/Morocco/IPM20419246/2022 | EPI_ISL_13526809 | 01/06/2022 | Africa / Morocco / Casablanca | Human | unknown | Male | 44 | unknown | unknown | unknown | BA.5.2 | GRA |
| hCoV-19/Morocco/IPM20419167/2022 | EPI_ISL_13526808 | 01/06/2022 | Africa / Morocco / Casablanca | Human | unknown | Male | 30 | unknown | unknown | unknown | BA.2 | GRA |
| hCoV-19/Morocco/IPM20419154/2022 | EPI_ISL_13526807 | 01/06/2022 | Africa / Morocco / Casablanca | Human | unknown | unknown | unknown | unknown | unknown | unknown | BA.2.12.1 | GRA |
| hCoV-19/Morocco/IPM20419125/2022 | EPI_ISL_13526806 | 31/05/2022 | Africa / Morocco / Casablanca | Human | unknown | Male | 45 | unknown | unknown | unknown | BA.2 | GRA |
| hCoV-19/Morocco/IPM20419115/2022 | EPI_ISL_13526805 | 31/05/2022 | Africa / Morocco / Casablanca | Human | unknown | Female | 30 | unknown | unknown | unknown | BA.5.2.20 | GRA |
| hCoV-19/Morocco/IPM20419068/2022 | EPI_ISL_13526804 | 30/05/2022 | Africa / Morocco / Casablanca | Human | unknown | Female | 24 | unknown | unknown | unknown | BA.5.2 | GRA |
| hCoV-19/Morocco/IPM20419023/2022 | EPI_ISL_13526803 | 28/05/2022 | Africa / Morocco / Casablanca | Human | unknown | Female | 17 | unknown | unknown | unknown | BA.5.2.20 | GRA |
| hCoV-19/Morocco/IPM20418995/2022 | EPI_ISL_13526802 | 28/05/2022 | Africa / Morocco / Casablanca | Human | unknown | Female | 82 | unknown | unknown | unknown | BA.2 | GRA |
| hCoV-19/Morocco/IPM20418965/2022 | EPI_ISL_13526801 | 26/05/2022 | Africa / Morocco / Casablanca | Human | unknown | Male | 51 | unknown | unknown | unknown | BA.2.9 | GRA |
| hCoV-19/Morocco/IPM20418871/2022 | EPI_ISL_13526799 | 26/05/2022 | Africa / Morocco / Casablanca | Human | unknown | Female | 62 | unknown | unknown | unknown | BA.2.9 | GRA |
| hCoV-19/Morocco/IPM20418668/2022 | EPI_ISL_13526798 | 23/05/2022 | Africa / Morocco / Casablanca | Human | unknown | unknown | unknown | unknown | unknown | unknown | BA.2 | GRA |
| hCoV-19/Morocco/IPM20418592/2022 | EPI_ISL_13526797 | 20/05/2022 | Africa / Morocco / Casablanca | Human | unknown | Female | 88 | unknown | unknown | unknown | BA.2 | GRA |
| hCoV-19/Morocco/IPM20418529/2022 | EPI_ISL_13526796 | 19/05/2022 | Africa / Morocco / Casablanca | Human | unknown | Male | 64 | unknown | unknown | unknown | BA.2 | GRA |
| hCoV-19/Morocco/IPM20418505/2022 | EPI_ISL_13526795 | 19/05/2022 | Africa / Morocco / Casablanca | Human | unknown | Male | 20 | unknown | unknown | unknown | BA.2 | GRA |
| hCoV-19/Morocco/IPM20418402/2022 | EPI_ISL_13526794 | 18/05/2022 | Africa / Morocco / Casablanca | Human | unknown | Female | 50 | unknown | unknown | unknown | BA.2 | GRA |
| hCoV-19/Morocco/IPM20418325/2022 | EPI_ISL_13526792 | 16/05/2022 | Africa / Morocco / Casablanca | Human | unknown | Female | 42 | unknown | unknown | unknown | BA.2 | GRA |
| hCoV-19/Morocco/IPM20418261/2022 | EPI_ISL_13526791 | 14/05/2022 | Africa / Morocco / Casablanca | Human | unknown | Male | 50 | unknown | unknown | unknown | BA.2 | GRA |
| hCoV-19/Morocco/IPM20418163/2022 | EPI_ISL_13526790 | 12/05/2022 | Africa / Morocco / Casablanca | Human | unknown | Female | 37 | unknown | unknown | unknown | BA.2 | GRA |
| hCoV-19/Morocco/IPM20418023/2022 | EPI_ISL_13526789 | 11/05/2022 | Africa / Morocco / Casablanca | Human | unknown | Male | 26 | unknown | unknown | unknown | BA.2 | GRA |
| hCoV-19/Morocco/IPM20417993/2022 | EPI_ISL_13526788 | 10/05/2022 | Africa / Morocco / Casablanca | Human | unknown | Male | 39 | unknown | unknown | unknown | BA.2.3 | GRA |
| hCoV-19/Morocco/IPM20417875/2022 | EPI_ISL_13526787 | 09/05/2022 | Africa / Morocco / Casablanca | Human | unknown | Male | 55 | unknown | unknown | unknown | BA.2 | GRA |
| hCoV-19/Morocco/IPM20417843/2022 | EPI_ISL_13526786 | 07/05/2022 | Africa / Morocco / Casablanca | Human | unknown | Male | 99 | unknown | unknown | unknown | BA.2 | GRA |
| hCoV-19/Morocco/IPM20417801/2022 | EPI_ISL_13526785 | 06/05/2022 | Africa / Morocco / Casablanca | Human | unknown | Male | 25 | unknown | unknown | unknown | BA.1 | GRA |
| hCoV-19/Morocco/IPM20417796/2022 | EPI_ISL_13526784 | 06/05/2022 | Africa / Morocco / Casablanca | Human | unknown | Female | 40 | unknown | unknown | unknown | BA.2 | GRA |
| hCoV-19/Morocco/IPM20417653/2022 | EPI_ISL_13526783 | 04/05/2022 | Africa / Morocco / Casablanca | Human | unknown | Female | 49 | unknown | unknown | unknown | BA.2 | GRA |
| hCoV-19/Morocco/IPM20417529/2022 | EPI_ISL_13526782 | 29/04/2022 | Africa / Morocco / Casablanca | Human | unknown | Female | 49 | unknown | unknown | unknown | BA.2 | GRA |
| hCoV-19/Morocco/IPM20417525/2022 | EPI_ISL_13526781 | 29/04/2022 | Africa / Morocco / Casablanca | Human | unknown | Male | 59 | unknown | unknown | unknown | BA.2 | GRA |
| hCoV-19/Morocco/IPM20417384/2022 | EPI_ISL_13526780 | 26/04/2022 | Africa / Morocco / Casablanca | Human | unknown | Male | 35 | unknown | unknown | unknown | BA.2 | GRA |
| hCoV-19/Morocco/IPM20417378/2022 | EPI_ISL_13526779 | 26/04/2022 | Africa / Morocco / Casablanca | Human | unknown | Male | 59 | unknown | unknown | unknown | BA.2 | GRA |
| hCoV-19/Morocco/IPM20417285/2022 | EPI_ISL_13526777 | 22/04/2022 | Africa / Morocco / Mohammedia | Human | unknown | Male | 60 | unknown | unknown | unknown | BA.2 | GRA |
| hCoV-19/Morocco/IPM20417153/2022 | EPI_ISL_13526776 | 18/04/2022 | Africa / Morocco / Casablanca | Human | unknown | Female | 50 | unknown | unknown | unknown | BA.2.3 | GRA |
| hCoV-19/Morocco/IPM20417149/2022 | EPI_ISL_13526775 | 18/04/2022 | Africa / Morocco / Casablanca | Human | unknown | Male | 59 | unknown | unknown | unknown | BA.2 | GRA |
| hCoV-19/Morocco/IPM20417121/2022 | EPI_ISL_13526774 | 16/04/2022 | Africa / Morocco / Casablanca | Human | unknown | Female | 24 | unknown | unknown | unknown | BA.2 | GRA |
| hCoV-19/Morocco/IPM20417120/2022 | EPI_ISL_13526773 | 16/04/2022 | Africa / Morocco / Casablanca | Human | unknown | Female | 23 | unknown | unknown | unknown | BA.2 | GRA |
| hCoV-19/Morocco/IPM20417096/2022 | EPI_ISL_13526772 | 15/04/2022 | Africa / Morocco / Casablanca | Human | unknown | Female | 43 | unknown | unknown | unknown | BA.2 | GRA |
| hCoV-19/Morocco/IPM20417066/2022 | EPI_ISL_13526771 | 14/04/2022 | Africa / Morocco / Casablanca | Human | unknown | Female | 26 | unknown | unknown | unknown | BA.2 | GRA |
| hCoV-19/Morocco/IPM20416910/2022 | EPI_ISL_13526770 | 11/04/2022 | Africa / Morocco / Casablanca | Human | unknown | Female | 56 | unknown | unknown | unknown | BA.2 | GRA |
| hCoV-19/Morocco/IPM20416667/2022 | EPI_ISL_13526769 | 02/04/2022 | Africa / Morocco / Mohammedia | Human | unknown | Female | 56 | unknown | unknown | unknown | BA.2 | GRA |
| hCoV-19/Morocco/IPM20416608/2022 | EPI_ISL_13526768 | 01/04/2022 | Africa / Morocco / Casablanca | Human | unknown | Male | 26 | unknown | unknown | unknown | BA.1 | GRA |
| hCoV-19/Morocco/IPM20416605/2022 | EPI_ISL_13526767 | 01/04/2022 | Africa / Morocco / Casablanca | Human | unknown | Male | 26 | unknown | unknown | unknown | BA.1 | GRA |
| hCoV-19/Morocco/IPM20416598/2022 | EPI_ISL_13526766 | 01/04/2022 | Africa / Morocco / Casablanca | Human | unknown | Female | 20 | unknown | unknown | unknown | BA.1 | GRA |
| hCoV-19/Morocco/IPM20416596/2022 | EPI_ISL_13526765 | 01/04/2022 | Africa / Morocco / Casablanca | Human | unknown | Female | 74 | unknown | unknown | unknown | B.1.617.2 | GK |
| hCoV-19/Morocco/IPM20416590/2022 | EPI_ISL_13526764 | 01/04/2022 | Africa / Morocco / Casablanca | Human | unknown | Male | 51 | unknown | unknown | unknown | BA.1 | GRA |
| hCoV-19/Morocco/IPM20386811/2021 | EPI_ISL_9417722 | 15/12/2021 | Africa / Morocco / Casablanca | Human | unknown | Male | 52 | unknown | unknown | unknown | B.1.617.2 | GK |
| hCoV-19/Morocco/CNRST_HMIMV_AS_59/2021 | EPI_ISL_13445003 | 2021-07 | Africa / Morocco / Rabat | Human | unknown | Male | 65 | Released | unknown | unknown | AY.33 | GK |
| hCoV-19/Morocco/CNRST_HMIMV_AS_64/2021 | EPI_ISL_13445004 | 2021-08 | Africa / Morocco / Rabat | Human | unknown | Male | 54 | Released | unknown | unknown | B.1.1.7 | GRY |
| hCoV-19/Morocco/CNRST_HMIMV_AS_51/2021 | EPI_ISL_13445001 | 2021-07 | Africa / Morocco / Rabat | Human | unknown | Male | 76 | Released | unknown | unknown | B.1.1.7 | GRY |
| hCoV-19/Morocco/CNRST_HMIMV_AS_48/2021 | EPI_ISL_13445000 | 2021-07 | Africa / Morocco / Rabat | Human | unknown | Male | 76 | Released | unknown | unknown | AY.33 | GK |
| hCoV-19/Morocco/CNRST_HMIMV_AS_35/2021 | EPI_ISL_13444999 | 2021-07 | Africa / Morocco / Rabat | Human | unknown | Female | 66 | Released | unknown | unknown | B.1.1.7 | GRY |
| hCoV-19/Morocco/CNRST_HMIMV_AS_33/2021 | EPI_ISL_13444998 | 2021-07 | Africa / Morocco / Rabat | Human | unknown | Male | 65 | Released | unknown | unknown | AY.33 | GK |
| hCoV-19/Morocco/CNRST_HMIMV_AS_29/2021 | EPI_ISL_13444997 | 2021-07 | Africa / Morocco / Rabat | Human | unknown | Male | 54 | Released | unknown | unknown | B.1.525 | G |
| hCoV-19/Morocco/CNRST_HMIMV_AS_28/2021 | EPI_ISL_13444996 | 2021-07 | Africa / Morocco / Rabat | Human | unknown | Male | 54 | Released | unknown | unknown | AY.33 | GK |
| hCoV-19/Morocco/CNRST_HMIMV_AS_24/2021 | EPI_ISL_13444995 | 2021-07 | Africa / Morocco / Rabat | Human | unknown | Male | 59 | Released | unknown | unknown | AY.33 | GK |
| hCoV-19/Morocco/CNRST_HMIMV_AS_22/2021 | EPI_ISL_13444994 | 2021-07 | Africa / Morocco / Rabat | Human | unknown | Male | 69 | Released | unknown | unknown | AY.33 | GK |
| hCoV-19/Morocco/CNRST_HMIMV_AS_19/2021 | EPI_ISL_13444993 | 2021-07 | Africa / Morocco / Rabat | Human | unknown | Male | 65 | Released | unknown | unknown | AY.33 | GK |
| hCoV-19/Morocco/CNRST_HMIMV_AS_16/2021 | EPI_ISL_13444992 | 2021-07 | Africa / Morocco / Rabat | Human | unknown | Male | 49 | Released | unknown | unknown | B.1.1.7 | GRY |
| hCoV-19/Morocco/CNRST_HMIMV_AS_15/2021 | EPI_ISL_13444991 | 2021-07 | Africa / Morocco / Rabat | Human | unknown | Male | 45 | Released | unknown | unknown | AY.122 | GK |
| hCoV-19/Morocco/CNRST_HMIMV_AS_12/2021 | EPI_ISL_13444990 | 2021-07 | Africa / Morocco / Rabat | Human | unknown | Female | 38 | Released | unknown | unknown | B.1.1.7 | GRY |
| hCoV-19/Morocco/CNRST_HMIMV_AS_54/2021 | EPI_ISL_13445002 | 2021-07 | Africa / Morocco / Rabat | Human | unknown | Male | 76 | Released | unknown | unknown | B.1.1.7 | GRY |
| hCoV-19/Morocco/CNRST_HMIMV_AS_7/2021 | EPI_ISL_13444989 | 2021-07 | Africa / Morocco / Rabat | Human | unknown | Male | 37 | Released | unknown | unknown | AY.33 | GK |
| hCoV-19/Morocco/CNRST_HMIMV_AS_5/2021 | EPI_ISL_13444988 | 2021-07 | Africa / Morocco / Rabat | Human | unknown | Male | 40 | Released | unknown | unknown | AY.33 | GK |
| hCoV-19/Morocco/CNRST_HMIMV_KHAT_1/2021 | EPI_ISL_13444987 | 2021-07 | Africa / Morocco / Rabat | Human | unknown | Female | 28 | Released | unknown | unknown | AY.33 | GK |
| hCoV-19/Morocco/GR92/2021 | EPI_ISL_9449575 | 29/12/2021 | Africa / Morocco | Human | Active surveillance | Male | unknown | Released | unknown | Active surveillance | B.1.1.529 | GRA |
| hCoV-19/Morocco/CNRST_LCAM_520/2021 | EPI_ISL_13444986 | 2021-07 | Africa / Morocco / Rabat | Human | unknown | Male | 66 | Released | unknown | unknown | B.1.351 | GH |
| hCoV-19/Morocco/629/2022 | EPI_ISL_13421948 | 22/02/2022 | Africa / Morocco / Rabat | Human | unknown | Male | 72 | Intensive care | unknown | unknown | BA.1 | GRA |
| hCoV-19/Morocco/613/2022 | EPI_ISL_13421945 | 02/01/2022 | Africa / Morocco / Beni Mellal | Human | unknown | Male | 74 | Intensive care | unknown | unknown | BA.1 | GRA |
| hCoV-19/Morocco/608/2022 | EPI_ISL_13421943 | 07/02/2022 | Africa / Morocco / Beni Mellal | Human | unknown | Male | 78 | Intensive care | unknown | unknown | BA.1 | GRA |
| hCoV-19/Morocco/583/2022 | EPI_ISL_13421935 | 24/01/2022 | Africa / Morocco / Beni Mellal | Human | unknown | Female | unknown | Intensive care | unknown | unknown | BA.1 | GRA |
| hCoV-19/Morocco/603/2022 | EPI_ISL_13421940 | 13/01/2022 | Africa / Morocco / Temara | Human | unknown | Male | 40 | Intensive care | unknown | unknown | BA.1 | GRA |
| hCoV-19/Morocco/581/2022 | EPI_ISL_13421934 | 19/01/2022 | Africa / Morocco / Khouribga | Human | unknown | Male | unknown | Intensive care | unknown | unknown | BA.1 | GRA |
| hCoV-19/Morocco/566/2022 | EPI_ISL_13421929 | 16/01/2022 | Africa / Morocco / Kenitra | Human | unknown | Male | unknown | Live | unknown | unknown | AY.121 | GK |
| hCoV-19/Morocco/574/2022 | EPI_ISL_13421931 | 18/01/2022 | Africa / Morocco / Rabat | Human | unknown | Female | unknown | Intensive care | unknown | unknown | BA.1 | GRA |
| hCoV-19/Morocco/MA-10-HMIMV-P8/2022 | EPI_ISL_17167250 | 24/10/2022 | Africa / Morocco / Rabat | unknown | unknown | unknown | unknown | unknown | unknown | unknown | B.1 | GH |
| hCoV-19/Morocco/848/2022 | EPI_ISL_13408053 | 13/05/2022 | Africa / Morocco / Rabat | Human | unknown | Female | 62 | Live | unknown | unknown | BA.5.1 | GRA |
| hCoV-19/Morocco/883/2022 | EPI_ISL_13408052 | 06/05/2022 | Africa / Morocco / Casablanca | Human | unknown | Male | 26 | Live | unknown | unknown | BA.2.49 | GRA |
| hCoV-19/Morocco/878/2022 | EPI_ISL_13408051 | 05/05/2022 | Africa / Morocco / Casablanca | Human | unknown | Female | 60 | Live | unknown | unknown | BA.2 | GRA |
| hCoV-19/Morocco/840/2022 | EPI_ISL_13408050 | 30/04/2022 | Africa / Morocco / Casablanca | Human | unknown | Female | 71 | Live | unknown | unknown | BA.2 | GRA |
| hCoV-19/Morocco/836/2022 | EPI_ISL_13408049 | 25/04/2022 | Africa / Morocco / Casablanca | Human | unknown | Female | 58 | Live | unknown | unknown | BA.2 | GRA |
| hCoV-19/Morocco/834/2022 | EPI_ISL_13408047 | 09/05/2022 | Africa / Morocco / Casablanca | Human | unknown | Male | unknown | Live | unknown | unknown | BA.5.2 | GRA |
| hCoV-19/Morocco/835/2022 | EPI_ISL_13408048 | 05/05/2022 | Africa / Morocco / Casablanca | Human | unknown | Female | 78 | Live | unknown | unknown | BA.2 | GRA |
| hCoV-19/Morocco/847/2022 | EPI_ISL_13408046 | 13/05/2022 | Africa / Morocco / Rabat | Human | unknown | Male | 7 | Live | unknown | unknown | BA.2 | GRA |
| hCoV-19/Morocco/820/2022 | EPI_ISL_13408038 | 24/04/2022 | Africa / Morocco / Casablanca | Human | unknown | Female | 30 | Live | unknown | unknown | BA.2 | GRA |
| hCoV-19/Morocco/885/2022 | EPI_ISL_13408045 | 04/05/2022 | Africa / Morocco / Casablanca | Human | unknown | Female | 57 | Live | unknown | unknown | BA.2 | GRA |
| hCoV-19/Morocco/830/2022 | EPI_ISL_13408034 | 20/04/2022 | Africa / Morocco / Casablanca | Human | unknown | Male | 43 | Live | unknown | unknown | BA.2 | GRA |
| hCoV-19/Morocco/877/2022 | EPI_ISL_13408037 | 09/05/2022 | Africa / Morocco / Casablanca | Human | unknown | Female | 60 | Live | unknown | unknown | BA.2 | GRA |
| hCoV-19/Morocco/6901/2020 | EPI_ISL_459979 | 19/04/2020 | Africa / Morocco | Human | unknown | unknown | unknown | unknown | unknown | unknown | B.1 | GH |
| hCoV-19/Morocco/RMPS-14/2020 | EPI_ISL_482739 | 22/04/2020 | Africa / Morocco | Human | unknown | Female | unknown | unknown | unknown | unknown | B.1 | GH |
| hCoV-19/Morocco/822/2022 | EPI_ISL_13408031 | 24/04/2022 | Africa / Morocco / Casablanca | Human | unknown | Female | 24 | Live | unknown | unknown | BA.2.9 | GRA |
| hCoV-19/Morocco/795/2022 | EPI_ISL_13330999 | 12/04/2022 | Africa / Morocco / Casablanca | Human | unknown | Male | unknown | Released | unknown | unknown | BA.2.3.9 | GRA |
| hCoV-19/Morocco/725/2022 | EPI_ISL_13331000 | 30/03/2022 | Africa / Morocco / Rabat | Human | unknown | Male | unknown | Released | unknown | unknown | BA.2 | GRA |
| hCoV-19/Morocco/783/2022 | EPI_ISL_13289791 | 02/04/2022 | Africa / Morocco / Casablanca | Human | unknown | Male | 44 | Released | unknown | unknown | BA.2 | GRA |
| hCoV-19/Morocco/727/2022 | EPI_ISL_13289790 | 30/03/2022 | Africa / Morocco / Rabat | Human | unknown | Female | unknown | Released | unknown | unknown | BA.2 | GRA |
| hCoV-19/Morocco/786/2022 | EPI_ISL_13289784 | 11/04/2022 | Africa / Morocco / Sale | Human | unknown | Male | unknown | Intensive care | unknown | unknown | BA.2 | GRA |
| hCoV-19/Morocco/779/2022 | EPI_ISL_13289781 | 30/03/2022 | Africa / Morocco / Casablanca | Human | unknown | Male | 33 | Released | unknown | unknown | BA.2 | GRA |
| hCoV-19/Morocco/777/2022 | EPI_ISL_13289780 | 06/04/2022 | Africa / Morocco / Casablanca | Human | unknown | Male | 66 | Released | unknown | unknown | BA.2 | GRA |
| hCoV-19/Morocco/797/2022 | EPI_ISL_13289770 | 12/04/2022 | Africa / Morocco / Casablanca | Human | unknown | Female | 60 | Released | unknown | unknown | BA.2 | GRA |
| hCoV-19/Morocco/RA92C/2020 | EPI_ISL_4299860 | 17/06/2020 | Africa / Morocco | Human | Outbreak investigation | Female | 45 | unknown | unknown | Outbreak investigation | B | L |
| hCoV-19/Morocco/INH-4910/2021 | EPI_ISL_17766952 | 26/05/2021 | Africa / Morocco / Kenitra | Human | unknown | Female | 46 | Live | unknown | unknown | B.1 | G |
| hCoV-19/Morocco/INH-263236/2021 | EPI_ISL_17766970 | 15/01/2021 | Africa / Morocco / Sidi Kacem | Human | unknown | Female | 19 | Live | unknown | unknown | B.1 | G |
| hCoV-19/Morocco/INH-256804/2021 | EPI_ISL_17766969 | 01/01/2021 | Africa / Morocco / Sidi Slimane | Human | unknown | Female | 18 | Live | unknown | unknown | B.1 | G |
| hCoV-19/Morocco/INH-256813/2021 | EPI_ISL_17766973 | 01/01/2021 | Africa / Morocco / Sidi Slimane | Human | unknown | Male | 13 | Live | unknown | unknown | B.1 | G |
| hCoV-19/Morocco/FMP-164/2021 | EPI_ISL_1912917 | 02/03/2021 | Africa / Morocco / Casablanca | Human | unknown | unknown | unknown | unknown | unknown | unknown | B.1.1.7 | G |
| hCoV-19/Morocco/903/2022 | EPI_ISL_13259130 | 18/05/2022 | Africa / Morocco / Casablanca | Human | unknown | Male | 33 | Released | unknown | unknown | BA.2 | GRA |
| hCoV-19/Morocco/898/2022 | EPI_ISL_13259129 | 13/05/2022 | Africa / Morocco / Casablanca | Human | unknown | Male | 41 | Released | unknown | unknown | BA.2 | GRA |
| hCoV-19/Morocco/896/2022 | EPI_ISL_13259128 | 20/05/2022 | Africa / Morocco / Rabat | Human | unknown | Male | 18 | Released | unknown | unknown | BA.5.2 | GRA |
| hCoV-19/Morocco/901/2022 | EPI_ISL_13259126 | 17/05/2022 | Africa / Morocco / Casablanca | Human | unknown | Male | 29 | Released | unknown | unknown | BA.2.9.3 | GRA |
| hCoV-19/Morocco/902/2022 | EPI_ISL_13259125 | 17/05/2022 | Africa / Morocco / Casablanca | Human | unknown | Female | 36 | Released | unknown | unknown | BA.2.9.3 | GRA |
| hCoV-19/Morocco/897/2022 | EPI_ISL_13259124 | 27/05/2022 | Africa / Morocco / Casablanca | Human | unknown | Female | 58 | Released | unknown | unknown | BA.2.56 | GRA |
| hCoV-19/Morocco/892/2022 | EPI_ISL_13259122 | 26/05/2022 | Africa / Morocco / Rabat | Human | unknown | Male | 27 | Released | unknown | unknown | BA.2 | GRA |
| hCoV-19/Morocco/IPM20383474/2021 | EPI_ISL_8186747 | 16/11/2021 | Africa / Morocco / Casablanca | Human | unknown | Female | 53 | unknown | unknown | unknown | AY.73 | GK |
| hCoV-19/Morocco/FMP-22/2020 | EPI_ISL_728344 | 2020-12 | Africa / Morocco / Rabat | Human | unknown | Male | 73 | unknown | unknown | unknown | B.1.1 | GR |
| hCoV-19/Morocco/IPM2003261/2020 | EPI_ISL_17650138 | 07/04/2020 | Africa / Morocco / Casablanca | Human | unknown | Female | 10 | unknown | unknown | unknown | B.1.1 | GR |
| hCoV-19/Morocco/INH-108/2020 | EPI_ISL_4899903 | 02/02/2020 | Africa / Morocco / Rabat | Human | unknown | Female | 76 | Released | unknown | unknown | B.1.177 | GV |
| hCoV-19/Morocco/CNRST_CHU08/2021 | EPI_ISL_8629696 | 19/10/2021 | Africa / Morocco / Rabat | Human | unknown | Female | 33 | released | unknown | unknown | AY.33 | GK |
| hCoV-19/Morocco/moh100/2020 | EPI_ISL_476024 | 05/05/2020 | Africa / Morocco | Human | unknown | Male | 18 | unknown | unknown | unknown | B.1.1 | GR |
| hCoV-19/Morocco/FMP-87/2021 | EPI_ISL_1810935 | 07/01/2021 | Africa / Morocco / Sidi Lahcen | Human | unknown | Female | 40 | unknown | unknown | unknown | B.1.1 | GR |
| hCoV-19/Morocco/FMP-298/2021 | EPI_ISL_5096088 | 2021-05 | Africa / Morocco / Sale | Human | unknown | unknown | unknown | unknown | unknown | unknown | B.1.1.7 | GRY |
| hCoV-19/Morocco/FMP-20/2020 | EPI_ISL_728340 | 2020-12 | Africa / Morocco / Mohammedia | Human | unknown | Female | 64 | unknown | unknown | unknown | B.1.1 | GR |
| hCoV-19/Morocco/FMP-93/2021 | EPI_ISL_1810948 | 04/01/2021 | Africa / Morocco / Sidi Lahcen | Human | unknown | Female | 61 | unknown | unknown | unknown | B.1 | G |
| hCoV-19/Morocco/FMP-89/2021 | EPI_ISL_1810937 | 02/01/2021 | Africa / Morocco / Sidi Lahcen | Human | unknown | Male | 51 | unknown | unknown | unknown | B.1.1 | GR |
| hCoV-19/Morocco/FMP-30/2020 | EPI_ISL_728363 | 2020-12 | Africa / Morocco / Rabat | Human | unknown | Male | 18 | unknown | unknown | unknown | B.1 | GH |
| hCoV-19/Morocco/HMIMV-P4279CCA/2020 | EPI_ISL_2968067 | 07/12/2020 | Africa / Morocco / Rabat | Human | unknown | Male | 51 | Released | unknown | unknown | B.1 | GH |
| hCoV-19/Morocco/HMIMV-ALT-G/2020 | EPI_ISL_2968062 | 07/12/2020 | Africa / Morocco / Rabat | Human | unknown | Female | 55 | Released | unknown | unknown | B.1 | G |
| hCoV-19/Morocco/6893/2020 | EPI_ISL_459971 | 18/03/2020 | Africa / Morocco | Human | unknown | unknown | unknown | unknown | unknown | unknown | B.1.153 | G |
| hCoV-19/Morocco/FMP-1/2021 | EPI_ISL_5061472 | 02/01/2021 | Africa / Morocco / Khemisset | Human | unknown | Female | 65 | unknown | unknown | unknown | B.1.177 | GV |
| hCoV-19/Morocco/HMIMV-2N/2020 | EPI_ISL_2968047 | 18/07/2020 | Africa / Morocco / Rabat | Human | unknown | Female | 44 | Released | unknown | unknown | B.1.78 | G |
| hCoV-19/Morocco/LRA211/2021 | EPI_ISL_8132278 | 22/07/2021 | Africa / Morocco | Human | Active surveillance | Male | unknown | Released | unknown | Active surveillance | B.1.1.7 | GRY |
| hCoV-19/Morocco/126/2021 | EPI_ISL_8134525 | 20/12/2021 | Africa / Morocco / Marrakech | Human | unknown | Female | unknown | Live | unknown | unknown | BA.1.1 | GRA |
| hCoV-19/Morocco/127/2021 | EPI_ISL_8135334 | 20/12/2021 | Africa / Morocco / Marrakech | Human | unknown | Female | unknown | Live | unknown | unknown | BA.1.1 | GRA |
| hCoV-19/Morocco/163/2021 | EPI_ISL_8135547 | 20/12/2021 | Africa / Morocco / Tangier | Human | unknown | Female | unknown | Live | unknown | unknown | BA.1.1 | GRA |
| hCoV-19/Morocco/Ion_code_2/2021 | EPI_ISL_3155073 | 17/06/2021 | Africa / Morocco / casablanca | Human | unknown | Male | 40 | Released | unknown | unknown | B.1.1.7 | GRY |
| hCoV-19/Morocco/101/2021 | EPI_ISL_8144261 | 21/12/2021 | Africa / Morocco / Fes | Human | unknown | Male | unknown | Live | unknown | unknown | BA.1 | GRA |
| hCoV-19/Morocco/IPM20435477/2023 | EPI_ISL_17650216 | 23/02/2023 | Africa / Morocco / Casablanca | Human | unknown | Female | 61 | unknown | unknown | unknown | BQ.1.1.47 | GRA |
| hCoV-19/Morocco/IPM20125322/2020 | EPI_ISL_17650179 | 24/07/2020 | Africa / Morocco / Casablanca | Human | unknown | Male | unknown | unknown | unknown | unknown | B.1.1 | GR |
| hCoV-19/Morocco/IPM20124438/2020 | EPI_ISL_17650178 | 23/07/2020 | Africa / Morocco / Casablanca | Human | unknown | Female | unknown | unknown | unknown | unknown | B.1.1 | GR |
| hCoV-19/Morocco/IPM2095777/2020 | EPI_ISL_17650172 | 06/07/2020 | Africa / Morocco / Casablanca | Human | unknown | Male | unknown | unknown | unknown | unknown | B.1 | G |
| hCoV-19/Morocco/IPM2083722/2020 | EPI_ISL_17650171 | 29/06/2020 | Africa / Morocco / Casablanca | Human | unknown | Male | unknown | unknown | unknown | unknown | B.1 | G |
| hCoV-19/Morocco/IPM2037181/2020 | EPI_ISL_17650163 | 01/06/2020 | Africa / Morocco / Casablanca | Human | unknown | Male | unknown | unknown | unknown | unknown | B.1 | G |
| hCoV-19/Morocco/FMP-31/2020 | EPI_ISL_728366 | 2020-12 | Africa / Morocco / Temara | Human | unknown | Female | 57 | unknown | unknown | unknown | B.1.177.51 | GV |
| hCoV-19/Morocco/6888/2020 | EPI_ISL_459966 | 15/03/2020 | Africa / Morocco | Human | unknown | unknown | unknown | unknown | unknown | unknown | B.1.1 | GR |
| hCoV-19/Morocco/INH-105/2020 | EPI_ISL_4899888 | 02/02/2020 | Africa / Morocco / Rabat | Human | unknown | Male | 65 | Released | unknown | unknown | B.1.221 | G |
| hCoV-19/Morocco/INH-104/2020 | EPI_ISL_4899881 | 02/02/2020 | Africa / Morocco / Rabat | Human | unknown | Female | 57 | Released | unknown | unknown | B.1 | G |
| hCoV-19/Morocco/INH-107/2020 | EPI_ISL_4899898 | 02/02/2020 | Africa / Morocco / Rabat | Human | unknown | Male | 55 | Released | unknown | unknown | B.1.177 | GV |
| hCoV-19/Morocco/Khouri101/2020 | EPI_ISL_3200860 | 06/05/2020 | Africa / Morocco | Human | Outbreak investigation | Female | 66 | unknown | unknown | Outbreak investigation | B.1 | G |
| hCoV-19/Morocco/INH-MN908947/2020 | EPI_ISL_4899917 | 02/02/2020 | Africa / Morocco / Rabat | Human | unknown | Female | 66 | Released | unknown | unknown | B.1 | G |
| hCoV-19/Morocco/HMIMV-279CCB/2020 | EPI_ISL_2968069 | 07/12/2020 | Africa / Morocco / Rabat | Human | unknown | Female | 60 | Released | unknown | unknown | B.1 | GH |
| hCoV-19/Morocco/734/2022 | EPI_ISL_13289777 | 30/03/2022 | Africa / Morocco / Rabat | Human | unknown | Male | unknown | Hospitalized | unknown | unknown | BA.1 | GRA |
| hCoV-19/Morocco/FMP-118/2021 | EPI_ISL_2313073 | 2021 | Africa / Morocco / Tinghir | Human | unknown | unknown | unknown | unknown | unknown | unknown | B.1.160 | GH |
| hCoV-19/Morocco/534/2022 | EPI_ISL_10260254 | 03/01/2022 | Africa / Morocco / Khouribga | Human | unknown | Male | unknown | Live | unknown | unknown | BA.1 | GRA |
| hCoV-19/Morocco/FMP-350/2021 | EPI_ISL_4945816 | 22/07/2021 | Africa / Morocco / Agadir | Human | unknown | Female | unknown | unknown | unknown | unknown | AY.51 | GK |
| hCoV-19/Morocco/FMP-344/2021 | EPI_ISL_4945298 | 12/07/2021 | Africa / Morocco / Ouarzazate | Human | unknown | Female | unknown | unknown | unknown | unknown | B.1.617.2 | GK |
| hCoV-19/Morocco/FMP-343/2021 | EPI_ISL_4945008 | 13/07/2021 | Africa / Morocco / Chtouka | Human | unknown | Female | unknown | unknown | unknown | unknown | AY.33 | GK |
| hCoV-19/Morocco/FMP-351/2021 | EPI_ISL_4945821 | 22/07/2021 | Africa / Morocco / Agadir | Human | unknown | Female | unknown | unknown | unknown | unknown | AY.51 | GK |
| hCoV-19/Morocco/HMIMV-10N/2020 | EPI_ISL_2968043 | 18/07/2020 | Africa / Morocco / Rabat | Human | unknown | Female | 80 | Released | unknown | unknown | B.39 | V |
| hCoV-19/Morocco/6897/2020 | EPI_ISL_459975 | 21/03/2020 | Africa / Morocco | Human | unknown | unknown | unknown | unknown | unknown | unknown | B.1 | G |
| hCoV-19/Morocco/FMP-345/2021 | EPI_ISL_4945669 | 22/07/2021 | Africa / Morocco / Agadir | Human | unknown | Female | unknown | unknown | unknown | unknown | B.1.617.2 | GK |
| hCoV-19/Morocco/IPM20418944/2022 | EPI_ISL_13526800 | 26/05/2022 | Africa / Morocco / Casablanca | Human | unknown | Male | 60 | unknown | unknown | unknown | BA.2.3 | GRA |
| hCoV-19/Morocco/IPM20376571/2021 | EPI_ISL_5924597 | 12/10/2021 | Africa / Morocco / Mohammadia | Human | unknown | Male | 59 | unknown | unknown | unknown | B.1.617.2 | GK |
| hCoV-19/Morocco/IPM20419401/2022 | EPI_ISL_13526813 | 03/06/2022 | Africa / Morocco / Casablanca | Human | unknown | unknown | unknown | unknown | unknown | unknown | BA.5.2.1 | GRA |
| hCoV-19/Morocco/IPM20377351/2021 | EPI_ISL_5924609 | 14/10/2021 | Africa / Morocco / Casablanca | Human | unknown | Male | 18 | unknown | unknown | unknown | B.1.617.2 | GK |
| hCoV-19/Morocco/IPM20359950/2021 | EPI_ISL_5501137 | 06/09/2021 | Africa / Morocco / Casablanca | Human | unknown | Female | 25 | unknown | unknown | unknown | AY.33 | GK |
| hCoV-19/Morocco/IPM20377097/2021 | EPI_ISL_5924606 | 13/10/2021 | Africa / Morocco / Mohammadia | Human | unknown | Male | 42 | unknown | unknown | unknown | B.1.617.2 | GK |
| hCoV-19/Morocco/IPM20376882/2021 | EPI_ISL_5924602 | 13/10/2021 | Africa / Morocco / Casablanca | Human | unknown | Male | 8 | unknown | unknown | unknown | B.1.617.2 | GK |
| hCoV-19/Morocco/IPM20418357/2022 | EPI_ISL_13526793 | 17/05/2022 | Africa / Morocco / Casablanca | Human | unknown | Female | 47 | unknown | unknown | unknown | BA.2 | GRA |
| hCoV-19/Morocco/FMP-349/2021 | EPI_ISL_4945787 | 17/07/2021 | Africa / Morocco / Beni Mellal | Human | unknown | Male | unknown | unknown | unknown | unknown | B.1.617.2 | GK |
| hCoV-19/Morocco/FMP-348/2021 | EPI_ISL_4945749 | 10/07/2021 | Africa / Morocco / Inezgane | Human | unknown | Female | unknown | unknown | unknown | unknown | AY.33 | GK |
| hCoV-19/Morocco/IPM20417318/2022 | EPI_ISL_13526778 | 22/04/2022 | Africa / Morocco / Casablanca | Human | unknown | Male | 20 | unknown | unknown | unknown | BA.1 | GRA |
| hCoV-19/Morocco/IPM20422385/2022 | EPI_ISL_13695642 | 23/06/2022 | Africa / Morocco / Casablanca | Human | unknown | Female | 34 | unknown | unknown | unknown | BA.5.2.20 | GRA |
| hCoV-19/Morocco/IPM20400736/2022 | EPI_ISL_10070716 | 10/01/2022 | Africa / Morocco / Casablanca | Human | unknown | Male | 80 | unknown | unknown | unknown | B.1.617.2 | GK |
| hCoV-19/Morocco/IPM20404450/2022 | EPI_ISL_10070726 | 18/01/2022 | Africa / Morocco / Casablanca | Human | unknown | Female | 84 | unknown | unknown | unknown | BA.1 | GRA |
| hCoV-19/Morocco/IPM20422354/2022 | EPI_ISL_13695653 | 23/06/2022 | Africa / Morocco / Casablanca | Human | unknown | Male | 52 | unknown | unknown | unknown | BA.5.2 | GRA |
| hCoV-19/Morocco/IPM20402117/2022 | EPI_ISL_10070723 | 12/01/2022 | Africa / Morocco / Casablanca | Human | unknown | Male | 65 | unknown | unknown | unknown | AY.33 | GK |
| hCoV-19/Morocco/IPM20405119/2022 | EPI_ISL_10070731 | 19/01/2022 | Africa / Morocco / Casablanca | Human | unknown | Male | 78 | unknown | unknown | unknown | BA.1 | GRA |
| hCoV-19/Morocco/IPM20404453/2022 | EPI_ISL_10070729 | 18/01/2022 | Africa / Morocco / Casablanca | Human | unknown | Female | 64 | unknown | unknown | unknown | BA.1 | GRA |
| hCoV-19/Morocco/IPM20422333/2022 | EPI_ISL_13695670 | 23/06/2022 | Africa / Morocco / Casablanca | Human | unknown | unknown | unknown | unknown | unknown | unknown | BA.5.2 | GRA |
| hCoV-19/Morocco/IPM20422488/2022 | EPI_ISL_13695662 | 23/06/2022 | Africa / Morocco / Casablanca | Human | unknown | Female | 63 | unknown | unknown | unknown | BA.2 | GRA |
| hCoV-19/Morocco/IPM20405838/2022 | EPI_ISL_10070737 | 20/01/2022 | Africa / Morocco / Casablanca | Human | unknown | Male | 75 | unknown | unknown | unknown | BA.1 | GRA |
| hCoV-19/Morocco/HMIMV-1001EN/2020 | EPI_ISL_2968044 | 18/07/2020 | Africa / Morocco / Rabat | Human | unknown | Male | 58 | Released | unknown | unknown | B.1 | G |
| hCoV-19/Morocco/FMP-120/2021 | EPI_ISL_2313076 | 2021 | Africa / Morocco / Casablanca | Human | unknown | unknown | unknown | unknown | unknown | unknown | B.1.1 | GR |
| hCoV-19/Morocco/FMP-8/2020 | EPI_ISL_728277 | 2020-11 | Africa / Morocco / Rabat | Human | unknown | unknown | unknown | unknown | unknown | unknown | B.1.1 | GR |
| hCoV-19/Morocco/112CC/2021 | EPI_ISL_8920479 | 06/07/2021 | Africa / Morocco | Human | Active surveillance | Female | unknown | Released | unknown | Active surveillance | AY.33 | GK |
| hCoV-19/Morocco/RA166/2021 | EPI_ISL_3241420 | 03/03/2021 | Africa / Morocco | Human | unknown | Female | 71 | unknown | unknown | unknown | B.1.1.7 | GRY |
| hCoV-19/Morocco/RA156-1C/2021 | EPI_ISL_3239896 | 20/06/2021 | Africa / Morocco | Human | Active surveillance | Male | unknown | Released | unknown | Active surveillance | B.1.1.7 | GR |
| hCoV-19/Morocco/RA12CC-73/2020 | EPI_ISL_3250687 | 13/08/2020 | Africa / Morocco | Human | Active surveillance | Male | unknown | Released | unknown | Active surveillance | B.1 | G |
| hCoV-19/Morocco/20350355/2021 | EPI_ISL_4430721 | 24/08/2021 | Africa / Morocco / Casablanca | Human | unknown | Male | 26 | unknown | unknown | unknown | B.1.617.2 | GK |
| hCoV-19/Morocco/20350480/2021 | EPI_ISL_4430727 | 24/08/2021 | Africa / Morocco / Casablanca | Human | unknown | Male | 36 | unknown | unknown | unknown | AY.34.1.1 | GK |
| hCoV-19/Morocco/20350409/2021 | EPI_ISL_4430724 | 24/08/2021 | Africa / Morocco / Casablanca | Human | unknown | Male | 49 | unknown | unknown | unknown | AY.33 | GK |
| hCoV-19/Morocco/20350900/2021 | EPI_ISL_4430733 | 24/08/2021 | Africa / Morocco / Casablanca | Human | unknown | Male | 35 | unknown | unknown | unknown | AY.33 | GK |
| hCoV-19/Morocco/20350832/2021 | EPI_ISL_4430730 | 24/08/2021 | Africa / Morocco / Casablanca | Human | unknown | Male | 33 | unknown | unknown | unknown | AY.33 | GK |
| hCoV-19/Morocco/20352672/2021 | EPI_ISL_4430754 | 26/08/2021 | Africa / Morocco / Casablanca | Human | unknown | Male | 18 | unknown | unknown | unknown | B.1.617.2 | GK |
| hCoV-19/Morocco/20350922/2021 | EPI_ISL_4430736 | 24/08/2021 | Africa / Morocco / Casablanca | Human | unknown | Female | 23 | unknown | unknown | unknown | AY.33 | GK |
| hCoV-19/Morocco/IPM2006257/2020 | EPI_ISL_17650145 | 16/04/2020 | Africa / Morocco / Casablanca | Human | unknown | Female | unknown | unknown | unknown | unknown | B.1.1 | GR |
| hCoV-19/Morocco/IPM2010950/2020 | EPI_ISL_17650153 | 26/04/2020 | Africa / Morocco / Casablanca | Human | unknown | Male | unknown | unknown | unknown | unknown | B.1 | G |
| hCoV-19/Morocco/625/2022 | EPI_ISL_13421947 | 12/02/2022 | Africa / Morocco / Fes | Human | unknown | Male | 26 | Intensive care | unknown | unknown | AY.33 | GK |
| hCoV-19/Morocco/CNRST_CHU12/2021 | EPI_ISL_8629699 | 22/10/2021 | Africa / Morocco / Rabat | Human | unknown | Female | 17 | released | unknown | unknown | AY.33 | GK |
| hCoV-19/Morocco/MA-07-HMIMV-P2-Jouh/2022 | EPI_ISL_16683687 | 09/02/2022 | Africa / Morocco / Rabat | Human | unknown | unknown | unknown | Released | unknown | unknown | BA.5.2.20 | GRA |
| hCoV-19/Morocco/208/2021 | EPI_ISL_10019530 | 26/12/2021 | Africa / Morocco / Fnidek | Human | unknown | Female | unknown | Live | unknown | unknown | BA.1 | GRA |
| hCoV-19/Morocco/MA-07-HMIMV-Jouh/2022 | EPI_ISL_16683686 | 06/10/2022 | Africa / Morocco / Rabat | Human | unknown | Female | 25 | Released | unknown | unknown | BA.5.2.20 | GRA |
| hCoV-19/Morocco/FMP536/2022 | EPI_ISL_15654663 | 14/06/2022 | Africa / Morocco / Sidi Lahcen | Human | unknown | Female | 45 | unknown | unknown | unknown | BA.5.2.20 | GRA |
| hCoV-19/Morocco/FMP535/2022 | EPI_ISL_15654662 | 14/06/2022 | Africa / Morocco / Sidi Lahcen | Human | unknown | Male | 56 | unknown | unknown | unknown | BA.5.2 | GRA |
| hCoV-19/Morocco/FMP534/2022 | EPI_ISL_15654661 | 14/06/2022 | Africa / Morocco / Sidi Lahcen | Human | unknown | Female | 40 | unknown | unknown | unknown | BA.1 | GRA |
| hCoV-19/Morocco/FMP533/2022 | EPI_ISL_15654639 | 14/06/2022 | Africa / Morocco / Sidi Lahcen | Human | unknown | Male | 46 | unknown | unknown | unknown | BA.5.2.20 | GRA |
| hCoV-19/Morocco/FMP-173/2021 | EPI_ISL_2111464 | 02/03/2021 | Africa / Morocco / Casablanca | Human | Baseline surveillance | unknown | unknown | unknown | unknown | Baseline surveillance | B.1.525 | G |
| hCoV-19/Morocco/FMP530/2022 | EPI_ISL_15651092 | 05/06/2022 | Africa / Morocco | Human | unknown | Male | 34 | unknown | unknown | unknown | BA.5.2.20 | GRA |
| hCoV-19/Morocco/FMP529/2022 | EPI_ISL_15650797 | 02/06/2022 | Africa / Morocco | Human | unknown | Male | 28 | unknown | unknown | unknown | BA.5.2 | GRA |
| hCoV-19/Morocco/FMP528/2022 | EPI_ISL_15650796 | 14/06/2022 | Africa / Morocco / Sidi Lahcen | Human | unknown | Male | 23 | unknown | unknown | unknown | BA.1 | GRA |
| hCoV-19/Morocco/AFMP527/2022 | EPI_ISL_15650790 | 14/06/2022 | Africa / Morocco / Sidi Lahcen | Human | unknown | Female | 30 | unknown | unknown | unknown | BA.5.2.20 | GRA |
| hCoV-19/Morocco/FMP526/2022 | EPI_ISL_15650597 | 14/06/2022 | Africa / Morocco / Sidi Lahcen | Human | unknown | Male | 32 | unknown | unknown | unknown | BA.5.2.20 | GRA |
| hCoV-19/Morocco/FMP525/2022 | EPI_ISL_15650509 | 14/06/2022 | Africa / Morocco / Sidi Lahcen | Human | unknown | Male | 37 | unknown | unknown | unknown | BA.5.1.22 | GRA |
| hCoV-19/Morocco/FMP532/2022 | EPI_ISL_15654638 | 14/06/2022 | Africa / Morocco / Sidi Lahcen | Human | unknown | Female | 47 | unknown | unknown | unknown | BA.5.2.20 | GRA |
| hCoV-19/Morocco/FMP531/2022 | EPI_ISL_15654637 | 06/06/2022 | Africa / Morocco | Human | unknown | Male | 51 | unknown | unknown | unknown | BA.5.2.20 | GRA |
| hCoV-19/Morocco/FMP523/2022 | EPI_ISL_15650346 | 14/06/2022 | Africa / Morocco | Human | unknown | Male | 39 | unknown | unknown | unknown | BA.5.1 | GRA |
| hCoV-19/Morocco/FMP524/2022 | EPI_ISL_15650423 | 14/06/2022 | Africa / Morocco / Sidi Lahcen | Human | unknown | Female | 34 | unknown | unknown | unknown | BA.5.2.20 | GRA |
| hCoV-19/Morocco/FMP521/2022 | EPI_ISL_15650192 | 06/06/2022 | Africa / Morocco | Human | unknown | Female | 35 | unknown | unknown | unknown | BA.5.2.20 | GRA |
| hCoV-19/Morocco/FMP522/2022 | EPI_ISL_15650196 | 06/06/2022 | Africa / Morocco | Human | unknown | Female | unknown | unknown | unknown | unknown | BA.1 | GRA |
| hCoV-19/Morocco/FMP520/2022 | EPI_ISL_15650188 | 28/05/2022 | Africa / Morocco | Human | unknown | Male | unknown | unknown | unknown | unknown | B.1 | G |
| hCoV-19/Morocco/FMP519/2022 | EPI_ISL_15650091 | 14/06/2022 | Africa / Morocco / Sidi Lahcen | Human | unknown | Female | 67 | unknown | unknown | unknown | BA.5.2.20 | GRA |
| hCoV-19/Morocco/FMP518/2022 | EPI_ISL_15650086 | 06/06/2022 | Africa / Morocco | Human | unknown | Female | 55 | unknown | unknown | unknown | BA.5.1 | GRA |
| hCoV-19/Morocco/FMP516/2022 | EPI_ISL_15649948 | 02/06/2022 | Africa / Morocco | Human | unknown | Female | 50 | unknown | unknown | unknown | BA.5.2.20 | GRA |
| hCoV-19/Morocco/FMP517/2022 | EPI_ISL_15649998 | 06/06/2022 | Africa / Morocco | Human | unknown | Female | 33 | unknown | unknown | unknown | BA.5.2.20 | GRA |
| hCoV-19/Morocco/FMP515/2022 | EPI_ISL_15649778 | 02/06/2022 | Africa / Morocco | Human | unknown | Female | 45 | unknown | unknown | unknown | BA.5.2.20 | GRA |
| hCoV-19/Morocco/FMP513/2022 | EPI_ISL_15649635 | 01/06/2022 | Africa / Morocco | Human | unknown | Male | 35 | unknown | unknown | unknown | BA.5.2.20 | GRA |
| hCoV-19/Morocco/FMP514/2022 | EPI_ISL_15649688 | 02/06/2022 | Africa / Morocco | Human | unknown | Female | 27 | unknown | unknown | unknown | BA.5.2.20 | GRA |
| hCoV-19/Morocco/FMP187/2021 | EPI_ISL_15635655 | 02/01/2021 | Africa / Morocco | Human | unknown | Male | 60 | unknown | unknown | unknown | B.1 | G |
| hCoV-19/Morocco/FMP503/2022 | EPI_ISL_15635030 | 05/06/2022 | Africa / Morocco | Human | unknown | Male | 34 | unknown | unknown | unknown | BA.5.2.20 | GRA |
| hCoV-19/Morocco/FMP491/2022 | EPI_ISL_15634610 | 14/06/2022 | Africa / Morocco / Sidi Lahcen | Human | unknown | Male | 37 | unknown | unknown | unknown | BA.5 | GRA |
| hCoV-19/Morocco/FMP497/2022 | EPI_ISL_15634836 | 06/06/2022 | Africa / Morocco | Human | unknown | Female | 35 | unknown | unknown | unknown | BA.2.12.1 | GRA |
| hCoV-19/Morocco/FMP486/2022 | EPI_ISL_15634141 | 06/06/2022 | Africa / Morocco | Human | unknown | Female | 54 | unknown | unknown | unknown | BA.5.1 | GRA |
| hCoV-19/Morocco/FMP489/2022 | EPI_ISL_15634385 | 06/06/2022 | Africa / Morocco | Human | unknown | Female | unknown | unknown | unknown | unknown | BA.5.2.20 | GRA |
| hCoV-19/Morocco/FMP485/2022 | EPI_ISL_15633907 | 06/06/2022 | Africa / Morocco | Human | unknown | Female | 33 | unknown | unknown | unknown | BA.5.2 | GRA |
| hCoV-19/Morocco/FMP484/2022 | EPI_ISL_15633599 | 02/06/2022 | Africa / Morocco | Human | unknown | Female | 50 | unknown | unknown | unknown | BA.2 | GRA |
| hCoV-19/Morocco/FMP483/2022 | EPI_ISL_15633279 | 02/06/2022 | Africa / Morocco | Human | unknown | Female | 45 | unknown | unknown | unknown | BA.5.2 | GRA |
| hCoV-19/Morocco/FMP482/2022 | EPI_ISL_15633032 | 06/06/2022 | Africa / Morocco | Human | unknown | Male | 51 | unknown | unknown | unknown | BA.5.2.20 | GRA |
| hCoV-19/Morocco/FMP481/2022 | EPI_ISL_15632814 | 02/06/2022 | Africa / Morocco | Human | unknown | Male | 28 | unknown | unknown | unknown | BA.2.12.1 | GRA |
| hCoV-19/Morocco/FMP479/2022 | EPI_ISL_15632469 | 14/01/2022 | Africa / Morocco | Human | unknown | Male | 2 | unknown | unknown | unknown | BA.1 | GRA |
| hCoV-19/Morocco/FMP477/2022 | EPI_ISL_15632243 | 17/01/2022 | Africa / Morocco | Human | unknown | Female | 28 | unknown | unknown | unknown | BA.1 | GRA |
| hCoV-19/Morocco/FMP476/2022 | EPI_ISL_15632007 | 17/01/2022 | Africa / Morocco | Human | unknown | Male | 57 | unknown | unknown | unknown | BA.1 | GRA |
| hCoV-19/Morocco/FMP474/2022 | EPI_ISL_15631792 | 18/01/2022 | Africa / Morocco | Human | unknown | Female | 34 | unknown | unknown | unknown | B.1 | G |
| hCoV-19/Morocco/FMP470/2022 | EPI_ISL_15631709 | 17/01/2022 | Africa / Morocco | Human | unknown | Female | 56 | unknown | unknown | unknown | BA.1 | GRA |
| hCoV-19/Morocco/FMP472/2022 | EPI_ISL_15631711 | 16/01/2022 | Africa / Morocco | Human | unknown | Female | 51 | unknown | unknown | unknown | BA.1 | GRA |
| hCoV-19/Morocco/FMP471/2022 | EPI_ISL_15631710 | 16/01/2022 | Africa / Morocco | Human | unknown | Male | 12 | unknown | unknown | unknown | BA.1 | GRA |
| hCoV-19/Morocco/FMP469/2022 | EPI_ISL_15631631 | 12/01/2022 | Africa / Morocco | Human | unknown | Female | 26 | unknown | unknown | unknown | BA.1 | GRA |
| hCoV-19/Morocco/FMP468/2022 | EPI_ISL_15631546 | 14/01/2022 | Africa / Morocco | Human | unknown | Female | 64 | unknown | unknown | unknown | BA.1 | GRA |
| hCoV-19/Morocco/FMP467/2022 | EPI_ISL_15631538 | 09/02/2022 | Africa / Morocco | Human | unknown | Female | 34 | unknown | unknown | unknown | BA.1 | GRA |
| hCoV-19/Morocco/FMP464/2022 | EPI_ISL_15631492 | 19/01/2022 | Africa / Morocco | Human | unknown | Male | 88 | unknown | unknown | unknown | BA.1 | GRA |
| hCoV-19/Morocco/FMP465/2021 | EPI_ISL_15631537 | 18/10/2021 | Africa / Morocco | Human | unknown | Male | 51 | unknown | unknown | unknown | B.1.177 | GV |
| hCoV-19/Morocco/FMP463/2022 | EPI_ISL_15631386 | 18/01/2022 | Africa / Morocco | Human | unknown | Male | 61 | unknown | unknown | unknown | BA.1 | GRA |
| hCoV-19/Morocco/FMP461/2022 | EPI_ISL_15631385 | 12/01/2022 | Africa / Morocco | Human | unknown | Female | 38 | unknown | unknown | unknown | BA.1 | GRA |
| hCoV-19/Morocco/FMP459/2022 | EPI_ISL_15630610 | 12/01/2022 | Africa / Morocco | Human | unknown | Female | 59 | unknown | unknown | unknown | BA.1 | GRA |
| hCoV-19/Morocco/FMP460/2022 | EPI_ISL_15630647 | 19/01/2022 | Africa / Morocco | Human | unknown | Female | 43 | unknown | unknown | unknown | BA.1 | GRA |
| hCoV-19/Morocco/FMP458/2022 | EPI_ISL_15630486 | 24/01/2022 | Africa / Morocco | Human | unknown | Male | 65 | unknown | unknown | unknown | BA.1 | GRA |
| hCoV-19/Morocco/FMP456/2022 | EPI_ISL_15630485 | 12/01/2022 | Africa / Morocco | Human | unknown | Female | 29 | unknown | unknown | unknown | BA.1 | GRA |
| hCoV-19/Morocco/FMP455/2022 | EPI_ISL_15630405 | 12/01/2022 | Africa / Morocco | Human | unknown | Female | 33 | unknown | unknown | unknown | BA.1 | GRA |
| hCoV-19/Morocco/FMP452/2022 | EPI_ISL_15630194 | 24/01/2022 | Africa / Morocco / Rabat | Human | unknown | Female | 69 | unknown | unknown | unknown | BA.1 | GRA |
| hCoV-19/Morocco/FMP454/2022 | EPI_ISL_15630296 | 15/01/2022 | Africa / Morocco | Human | unknown | Female | 77 | unknown | unknown | unknown | BA.1 | GRA |
| hCoV-19/Morocco/FMP453/2022 | EPI_ISL_15630294 | 07/02/2022 | Africa / Morocco | Human | unknown | Male | 85 | unknown | unknown | unknown | BA.1 | GRA |
| hCoV-19/Morocco/FMP-313/2021 | EPI_ISL_5103517 | 2021-05 | Africa / Morocco / Kenitra | Human | unknown | unknown | unknown | unknown | unknown | unknown | B.1.1.7 | GRY |
| hCoV-19/Morocco/FMP-314/2021 | EPI_ISL_5103519 | 2021-05 | Africa / Morocco / Kenitra | Human | unknown | unknown | unknown | unknown | unknown | unknown | B.1.1.7 | GRY |
| hCoV-19/Morocco/FMP448/2021 | EPI_ISL_15389649 | 19/12/2021 | Africa / Morocco | Human | unknown | Female | 63 | unknown | unknown | unknown | BA.1 | GRA |
| hCoV-19/Morocco/FMP445/2021 | EPI_ISL_15389646 | 28/12/2021 | Africa / Morocco | Human | unknown | Female | 60 | unknown | unknown | unknown | BA.1 | GRA |
| hCoV-19/Morocco/FMP443/2021 | EPI_ISL_15389254 | 27/12/2021 | Africa / Morocco | Human | unknown | Female | 59 | unknown | unknown | unknown | BA.1 | GRA |
| hCoV-19/Morocco/FMP442/2021 | EPI_ISL_15389250 | 27/12/2021 | Africa / Morocco | Human | unknown | Female | 54 | unknown | unknown | unknown | BA.1 | GRA |
| hCoV-19/Morocco/FMP440/2021 | EPI_ISL_15389073 | 23/12/2021 | Africa / Morocco | Human | unknown | Female | 44 | unknown | unknown | unknown | BA.1 | GRA |
| hCoV-19/Morocco/FMP439/2021 | EPI_ISL_15389072 | 23/12/2021 | Africa / Morocco | Human | unknown | Male | 67 | unknown | unknown | unknown | BA.1 | GRA |
| hCoV-19/Morocco/FMP438/2021 | EPI_ISL_15389071 | 23/12/2021 | Africa / Morocco | Human | unknown | Female | 46 | unknown | unknown | unknown | BA.1 | GRA |
| hCoV-19/Morocco/FMP437/2021 | EPI_ISL_15389070 | 27/12/2021 | Africa / Morocco | Human | unknown | Female | 25 | unknown | unknown | unknown | BA.1 | GRA |
| hCoV-19/Morocco/FMP436/2022 | EPI_ISL_15389069 | 14/01/2022 | Africa / Morocco | Human | unknown | Male | 29 | unknown | unknown | unknown | BA.1 | GRA |
| hCoV-19/Morocco/FMP435/2021 | EPI_ISL_15389062 | 27/12/2021 | Africa / Morocco | Human | unknown | Female | 32 | unknown | unknown | unknown | BA.1 | GRA |
| hCoV-19/Morocco/FMP434/2022 | EPI_ISL_15389061 | 14/01/2022 | Africa / Morocco | Human | unknown | Female | 33 | unknown | unknown | unknown | BA.1 | GRA |
| hCoV-19/Morocco/FMP433/2021 | EPI_ISL_15389060 | 29/12/2021 | Africa / Morocco | Human | unknown | Male | 55 | unknown | unknown | unknown | BA.1 | GRA |
| hCoV-19/Morocco/FMP432/2022 | EPI_ISL_15389059 | 10/01/2022 | Africa / Morocco | Human | unknown | Female | 56 | unknown | unknown | unknown | BA.1 | GRA |
| hCoV-19/Morocco/FMP430/2021 | EPI_ISL_15388973 | 27/12/2021 | Africa / Morocco | Human | unknown | Male | 51 | unknown | unknown | unknown | BA.1 | GRA |
| hCoV-19/Morocco/FMP429/2021 | EPI_ISL_15388893 | 27/12/2021 | Africa / Morocco | Human | unknown | Female | 63 | unknown | unknown | unknown | BA.1 | GRA |
| hCoV-19/Morocco/FMP428/2022 | EPI_ISL_15388888 | 18/01/2022 | Africa / Morocco | Human | unknown | Female | 61 | unknown | unknown | unknown | BA.1 | GRA |
| hCoV-19/Morocco/FMP427/2022 | EPI_ISL_15388887 | 16/01/2022 | Africa / Morocco | Human | unknown | Female | 23 | unknown | unknown | unknown | BA.1 | GRA |
| hCoV-19/Morocco/FMP426/2021 | EPI_ISL_15388886 | 29/12/2021 | Africa / Morocco | Human | unknown | Female | 74 | unknown | unknown | unknown | BA.1.8 | GRA |
| hCoV-19/Morocco/FMP423/2021 | EPI_ISL_15388832 | 27/12/2021 | Africa / Morocco | Human | unknown | Female | 23 | unknown | unknown | unknown | BA.1 | GRA |
| hCoV-19/Morocco/FMP422/2021 | EPI_ISL_15388804 | 27/12/2021 | Africa / Morocco | Human | unknown | Female | 24 | unknown | unknown | unknown | BA.1 | GRA |
| hCoV-19/Morocco/FMP421/2021 | EPI_ISL_15388803 | 27/12/2021 | Africa / Morocco | Human | unknown | Male | 64 | unknown | unknown | unknown | BA.1 | GRA |
| hCoV-19/Morocco/FMP420/2022 | EPI_ISL_15388689 | 19/01/2022 | Africa / Morocco | Human | unknown | Male | 64 | unknown | unknown | unknown | BA.1 | GRA |
| hCoV-19/Morocco/FMP419/2021 | EPI_ISL_15388688 | 30/12/2021 | Africa / Morocco | Human | unknown | Female | 68 | unknown | unknown | unknown | BA.1 | GRA |
| hCoV-19/Morocco/FMP418/2022 | EPI_ISL_15388687 | 18/01/2022 | Africa / Morocco | Human | unknown | Male | 56 | unknown | unknown | unknown | BA.1 | GRA |
| hCoV-19/Morocco/FMP416/2021 | EPI_ISL_15388676 | 23/12/2021 | Africa / Morocco / Sidi Lahcen | Human | unknown | Female | 38 | unknown | unknown | unknown | BA.1 | GRA |
| hCoV-19/Morocco/FMP415/2021 | EPI_ISL_15388599 | 23/12/2021 | Africa / Morocco | Human | unknown | Male | 51 | unknown | unknown | unknown | BA.1 | GRA |
| hCoV-19/Morocco/FMP414/2021 | EPI_ISL_15388526 | 27/12/2021 | Africa / Morocco | Human | unknown | Male | 43 | unknown | unknown | unknown | BA.1 | GRA |
| hCoV-19/Morocco/FMP413/2021 | EPI_ISL_15388525 | 27/12/2021 | Africa / Morocco | Human | unknown | Female | 33 | unknown | unknown | unknown | BA.1 | GRA |
| hCoV-19/Morocco/FMP411/2021 | EPI_ISL_15388070 | 27/12/2021 | Africa / Morocco / Rabat | Human | unknown | unknown | unknown | unknown | unknown | unknown | BA.1 | GRA |
| hCoV-19/Morocco/FMP412/2021 | EPI_ISL_15388165 | 27/12/2021 | Africa / Morocco / Rabat | Human | unknown | unknown | unknown | unknown | unknown | unknown | BA.1 | GRA |
| hCoV-19/Morocco/FMP410/2021 | EPI_ISL_15388060 | 27/12/2021 | Africa / Morocco / Fes | Human | unknown | unknown | unknown | unknown | unknown | unknown | BA.1.1 | GRA |
| hCoV-19/Morocco/FMP404/2021 | EPI_ISL_15387278 | 27/12/2021 | Africa / Morocco / Fes | Human | unknown | unknown | unknown | unknown | unknown | unknown | BA.1.1 | GRA |
| hCoV-19/Morocco/FMP400/2021 | EPI_ISL_15387267 | 27/12/2021 | Africa / Morocco / Fes | Human | unknown | unknown | unknown | unknown | unknown | unknown | BA.1.1 | GRA |
| hCoV-19/Morocco/FMP396/2021 | EPI_ISL_15387266 | 27/12/2021 | Africa / Morocco / Fes | Human | unknown | unknown | unknown | unknown | unknown | unknown | BA.1.1 | GRA |
| hCoV-19/Morocco/FMP395/2021 | EPI_ISL_15387263 | 27/12/2021 | Africa / Morocco / Fes | Human | unknown | unknown | unknown | unknown | unknown | unknown | BA.1.1 | GRA |
| hCoV-19/Morocco/FMP394/2021 | EPI_ISL_15387262 | 27/12/2021 | Africa / Morocco / Fes | Human | unknown | unknown | unknown | unknown | unknown | unknown | BA.1.1 | GRA |
| hCoV-19/Morocco/FMP393/2021 | EPI_ISL_15387233 | 27/12/2021 | Africa / Morocco / Rabat | Human | unknown | unknown | unknown | unknown | unknown | unknown | BA.1 | GRA |
| hCoV-19/Morocco/FMP391/2021 | EPI_ISL_15387019 | 27/12/2021 | Africa / Morocco / Rabat | Human | unknown | unknown | unknown | unknown | unknown | unknown | BA.1 | GRA |
| hCoV-19/Morocco/FMP409/2021 | EPI_ISL_15388042 | 27/12/2021 | Africa / Morocco / Rabat | Human | unknown | unknown | unknown | unknown | unknown | unknown | BA.1 | GRA |
| hCoV-19/Morocco/FMP389/2021 | EPI_ISL_15386569 | 27/12/2021 | Africa / Morocco / Rabat | Human | unknown | unknown | unknown | unknown | unknown | unknown | BA.1 | GRA |
| hCoV-19/Morocco/FMP390/2021 | EPI_ISL_15386801 | 27/12/2021 | Africa / Morocco / Rabat | Human | unknown | unknown | unknown | unknown | unknown | unknown | BA.1 | GRA |
| hCoV-19/Morocco/FMP387/2021 | EPI_ISL_15385908 | 27/12/2021 | Africa / Morocco / Rabat | Human | unknown | unknown | unknown | unknown | unknown | unknown | BA.1.1 (consensus call) | GRA |
| hCoV-19/Morocco/FMP388/2021 | EPI_ISL_15386289 | 27/12/2021 | Africa / Morocco / Rabat | Human | unknown | unknown | unknown | unknown | unknown | unknown | BA.1 | GRA |
| hCoV-19/Morocco/FMP385/2021 | EPI_ISL_15385535 | 27/12/2021 | Africa / Morocco / Rabat | Human | unknown | unknown | unknown | unknown | unknown | unknown | BA.1 | GRA |
| hCoV-19/Morocco/1045/2022 | EPI_ISL_15385505 | 05/07/2022 | Africa / Morocco / Tanger | Human | unknown | Female | 42 | Live | unknown | unknown | BA.5.2 | GRA |
| hCoV-19/Morocco/1060/2022 | EPI_ISL_15385507 | 25/07/2022 | Africa / Morocco / Sale | Human | unknown | Female | 36 | Live | unknown | unknown | BA.5.2.20 | GRA |
| hCoV-19/Morocco/1038/2022 | EPI_ISL_15385500 | 01/07/2022 | Africa / Morocco / Tanger | Human | unknown | Female | 56 | Live | unknown | unknown | BA.5.2 | GRA |
| hCoV-19/Morocco/1049/2022 | EPI_ISL_15385503 | 16/07/2022 | Africa / Morocco / Casablanca | Human | unknown | Male | 43 | Live | unknown | unknown | BA.5.2.20 | GRA |
| hCoV-19/Morocco/1056/2022 | EPI_ISL_15385498 | 20/07/2022 | Africa / Morocco / Kenitra | Human | unknown | Male | 27 | Live | unknown | unknown | BA.5.2 | GRA |
| hCoV-19/Morocco/1036/2022 | EPI_ISL_15385493 | 01/07/2022 | Africa / Morocco / Tanger | Human | unknown | Female | 73 | Live | unknown | unknown | BA.5.1 | GRA |
| hCoV-19/Morocco/1050/2022 | EPI_ISL_15385496 | 16/07/2022 | Africa / Morocco / Casablanca | Human | unknown | Female | 22 | Live | unknown | unknown | BA.5.2.1 | GRA |
| hCoV-19/Morocco/1035/2022 | EPI_ISL_15385492 | 01/07/2022 | Africa / Morocco / Tanger | Human | unknown | Male | 57 | Live | unknown | unknown | BA.5.1.23 | GRA |
| hCoV-19/Morocco/1024/2022 | EPI_ISL_15385199 | 26/06/2022 | Africa / Morocco / Laayoun | Human | unknown | Male | 37 | Live | unknown | unknown | BA.5 | GRA |
| hCoV-19/Morocco/1023/2022 | EPI_ISL_15385197 | 27/06/2022 | Africa / Morocco / Laayoun | Human | unknown | Male | 27 | Live | unknown | unknown | BA.5 | GRA |
| hCoV-19/Morocco/999/2022 | EPI_ISL_15385194 | 10/06/2022 | Africa / Morocco / Casablanca | Human | unknown | Female | 59 | Live | unknown | unknown | BA.2.12.1 | GRA |
| hCoV-19/Morocco/1009/2022 | EPI_ISL_15385195 | 30/06/2022 | Africa / Morocco / Rabat | Human | unknown | Female | 31 | Live | unknown | unknown | BA.5 | GRA |
| hCoV-19/Morocco/FMP-96/2021 | EPI_ISL_1811222 | 10/01/2021 | Africa / Morocco / Rabat | Human | unknown | Female | 42 | unknown | unknown | unknown | B.1.177 | GV |
| hCoV-19/Morocco/IPM20424150/2022 | EPI_ISL_15291916 | 29/06/2022 | Africa / Morocco / Casablanca | Human | unknown | Male | 19 | unknown | unknown | unknown | BA.5.2.1 | GRA |
| hCoV-19/Morocco/IPM20424135/2022 | EPI_ISL_15291915 | 29/06/2022 | Africa / Morocco / Casablanca | Human | unknown | Female | 28 | unknown | unknown | unknown | BF.5 | GRA |
| hCoV-19/Morocco/IPM20424129/2022 | EPI_ISL_15291914 | 29/06/2022 | Africa / Morocco / Casablanca | Human | unknown | Female | 49 | unknown | unknown | unknown | BA.5.2.20 | GRA |
| hCoV-19/Morocco/IPM20424111/2022 | EPI_ISL_15291913 | 29/06/2022 | Africa / Morocco / Casablanca | Human | unknown | Female | 41 | unknown | unknown | unknown | BA.5.2.20 | GRA |
| hCoV-19/Morocco/IPM20424088/2022 | EPI_ISL_15291912 | 29/06/2022 | Africa / Morocco / Casablanca | Human | unknown | Female | 60 | unknown | unknown | unknown | BA.5.2.20 | GRA |
| hCoV-19/Morocco/IPM20424074/2022 | EPI_ISL_15291911 | 29/06/2022 | Africa / Morocco / Casablanca | Human | unknown | Male | 21 | unknown | unknown | unknown | BA.5.1 | GRA |
| hCoV-19/Morocco/IPM20424006/2022 | EPI_ISL_15291908 | 28/06/2022 | Africa / Morocco / Casablanca | Human | unknown | Female | 73 | unknown | unknown | unknown | BA.5.2.20 | GRA |
| hCoV-19/Morocco/IPM20423988/2022 | EPI_ISL_15291907 | 28/06/2022 | Africa / Morocco / Casablanca | Human | unknown | Male | 33 | unknown | unknown | unknown | BA.5.2.20 | GRA |
| hCoV-19/Morocco/IPM20423953/2022 | EPI_ISL_15291906 | 28/06/2022 | Africa / Morocco / Casablanca | Human | unknown | Male | 77 | unknown | unknown | unknown | BA.2.9.3 | GRA |
| hCoV-19/Morocco/IPM20424037/2022 | EPI_ISL_15291910 | 28/06/2022 | Africa / Morocco / Casablanca | Human | unknown | Female | 58 | unknown | unknown | unknown | BA.4 | GRA |
| hCoV-19/Morocco/IPM20424019/2022 | EPI_ISL_15291909 | 28/06/2022 | Africa / Morocco / Casablanca | Human | unknown | Male | 55 | unknown | unknown | unknown | BA.5.2 | GRA |
| hCoV-19/Morocco/IPM20423938/2022 | EPI_ISL_15291905 | 28/06/2022 | Africa / Morocco / Casablanca | Human | unknown | Female | 78 | unknown | unknown | unknown | BA.5.1 | GRA |
| hCoV-19/Morocco/IPM20423846/2022 | EPI_ISL_15291901 | 28/06/2022 | Africa / Morocco / Casablanca | Human | unknown | Female | 41 | unknown | unknown | unknown | BA.5.2 | GRA |
| hCoV-19/Morocco/IPM20423904/2022 | EPI_ISL_15291904 | 28/06/2022 | Africa / Morocco / Casablanca | Human | unknown | Female | 69 | unknown | unknown | unknown | BA.5.2 | GRA |
| hCoV-19/Morocco/IPM20423876/2022 | EPI_ISL_15291903 | 28/06/2022 | Africa / Morocco / Casablanca | Human | unknown | Female | 50 | unknown | unknown | unknown | BA.5.1 | GRA |
| hCoV-19/Morocco/IPM20423855/2022 | EPI_ISL_15291902 | 28/06/2022 | Africa / Morocco / Casablanca | Human | unknown | Male | 26 | unknown | unknown | unknown | BA.5.2.20 | GRA |
| hCoV-19/Morocco/IPM20423825/2022 | EPI_ISL_15291900 | 28/06/2022 | Africa / Morocco / Casablanca | Human | unknown | Male | 24 | unknown | unknown | unknown | BA.5.2 | GRA |
| hCoV-19/Morocco/IPM20423773/2022 | EPI_ISL_15291897 | 28/06/2022 | Africa / Morocco / Casablanca | Human | unknown | Female | 63 | unknown | unknown | unknown | BA.5.2.20 | GRA |
| hCoV-19/Morocco/IPM20423818/2022 | EPI_ISL_15291899 | 28/06/2022 | Africa / Morocco / Casablanca | Human | unknown | Female | 45 | unknown | unknown | unknown | BA.5.2 | GRA |
| hCoV-19/Morocco/IPM20423781/2022 | EPI_ISL_15291898 | 28/06/2022 | Africa / Morocco / Casablanca | Human | unknown | Female | 79 | unknown | unknown | unknown | BA.5.1 | GRA |
| hCoV-19/Morocco/IPM20423737/2022 | EPI_ISL_15291896 | 28/06/2022 | Africa / Morocco / Casablanca | Human | unknown | Female | 72 | unknown | unknown | unknown | BA.5 | GRA |
| hCoV-19/Morocco/IPM20423729/2022 | EPI_ISL_15291895 | 28/06/2022 | Africa / Morocco / Casablanca | Human | unknown | Female | 90 | unknown | unknown | unknown | BA.5.1 | GRA |
| hCoV-19/Morocco/IPM20423442/2022 | EPI_ISL_15291891 | 27/06/2022 | Africa / Morocco / Casablanca | Human | unknown | Male | 32 | unknown | unknown | unknown | BF.5 | GRA |
| hCoV-19/Morocco/IPM20423713/2022 | EPI_ISL_15291894 | 28/06/2022 | Africa / Morocco / Casablanca | Human | unknown | Female | 52 | unknown | unknown | unknown | BF.5 | GRA |
| hCoV-19/Morocco/IPM20423697/2022 | EPI_ISL_15291893 | 28/06/2022 | Africa / Morocco / Casablanca | Human | unknown | Female | 61 | unknown | unknown | unknown | BA.5.2.20 | GRA |
| hCoV-19/Morocco/IPM20423476/2022 | EPI_ISL_15291892 | 27/06/2022 | Africa / Morocco / Casablanca | Human | unknown | Male | 57 | unknown | unknown | unknown | BA.5.2.20 | GRA |
| hCoV-19/Morocco/IPM20423418/2022 | EPI_ISL_15291890 | 27/06/2022 | Africa / Morocco / Casablanca | Human | unknown | Male | 54 | unknown | unknown | unknown | BA.5.2.20 | GRA |
| hCoV-19/Morocco/IPM20423400/2022 | EPI_ISL_15291889 | 27/06/2022 | Africa / Morocco / Casablanca | Human | unknown | Male | 26 | unknown | unknown | unknown | BA.5.2.1 | GRA |
| hCoV-19/Morocco/IPM20423343/2022 | EPI_ISL_15291886 | 27/06/2022 | Africa / Morocco / Casablanca | Human | unknown | Male | 62 | unknown | unknown | unknown | BA.5.2.20 | GRA |
| hCoV-19/Morocco/IPM20423384/2022 | EPI_ISL_15291888 | 27/06/2022 | Africa / Morocco / Casablanca | Human | unknown | Male | 30 | unknown | unknown | unknown | BA.5.2.20 | GRA |
| hCoV-19/Morocco/IPM20423369/2022 | EPI_ISL_15291887 | 27/06/2022 | Africa / Morocco / Casablanca | Human | unknown | Male | 24 | unknown | unknown | unknown | BA.5.2.1 | GRA |
| hCoV-19/Morocco/IPM20423313/2022 | EPI_ISL_15291885 | 27/06/2022 | Africa / Morocco / Casablanca | Human | unknown | Female | 71 | unknown | unknown | unknown | BA.5.2.20 | GRA |
| hCoV-19/Morocco/IPM20423307/2022 | EPI_ISL_15291884 | 27/06/2022 | Africa / Morocco / Casablanca | Human | unknown | Female | 39 | unknown | unknown | unknown | BV.2 | GRA |
| hCoV-19/Morocco/IPM20423165/2022 | EPI_ISL_15291881 | 25/06/2022 | Africa / Morocco / Casablanca | Human | unknown | Male | 57 | unknown | unknown | unknown | BA.5.2.20 | GRA |
| hCoV-19/Morocco/IPM20423288/2022 | EPI_ISL_15291883 | 27/06/2022 | Africa / Morocco / Casablanca | Human | unknown | Female | 61 | unknown | unknown | unknown | BA.5.2.20 | GRA |
| hCoV-19/Morocco/IPM20423176/2022 | EPI_ISL_15291882 | 25/06/2022 | Africa / Morocco / Casablanca | Human | unknown | Male | 36 | unknown | unknown | unknown | BA.5.2.20 | GRA |
| hCoV-19/Morocco/IPM20423151/2022 | EPI_ISL_15291880 | 25/06/2022 | Africa / Morocco / Casablanca | Human | unknown | Male | 75 | unknown | unknown | unknown | BA.5.2.20 | GRA |
| hCoV-19/Morocco/IPM20423106/2022 | EPI_ISL_15291877 | 25/06/2022 | Africa / Morocco / Casablanca | Human | unknown | Female | 52 | unknown | unknown | unknown | BA.5.2.20 | GRA |
| hCoV-19/Morocco/IPM20423123/2022 | EPI_ISL_15291879 | 25/06/2022 | Africa / Morocco / Casablanca | Human | unknown | Male | 66 | unknown | unknown | unknown | BF.5 | GRA |
| hCoV-19/Morocco/IPM20423112/2022 | EPI_ISL_15291878 | 25/06/2022 | Africa / Morocco / Casablanca | Human | unknown | Female | 30 | unknown | unknown | unknown | BA.5.2.20 | GRA |
| hCoV-19/Morocco/IPM20423082/2022 | EPI_ISL_15291876 | 25/06/2022 | Africa / Morocco / Casablanca | Human | unknown | Female | 43 | unknown | unknown | unknown | BA.5.2.20 | GRA |
| hCoV-19/Morocco/IPM20422797/2022 | EPI_ISL_15291873 | 24/06/2022 | Africa / Morocco / Casablanca | Human | unknown | Female | 75 | unknown | unknown | unknown | BA.5.2.20 | GRA |
| hCoV-19/Morocco/IPM20423074/2022 | EPI_ISL_15291875 | 25/06/2022 | Africa / Morocco / Casablanca | Human | unknown | Female | 12 | unknown | unknown | unknown | BA.5.2.20 | GRA |
| hCoV-19/Morocco/IPM20422819/2022 | EPI_ISL_15291874 | 24/06/2022 | Africa / Morocco / Casablanca | Human | unknown | Female | 28 | unknown | unknown | unknown | BA.5.2.20 | GRA |
| hCoV-19/Morocco/IPM20422783/2022 | EPI_ISL_15291872 | 24/06/2022 | Africa / Morocco / Casablanca | Human | unknown | Male | 38 | unknown | unknown | unknown | BA.5.2.20 | GRA |
| hCoV-19/Morocco/IPM20422782/2022 | EPI_ISL_15291871 | 24/06/2022 | Africa / Morocco / Casablanca | Human | unknown | Female | 71 | unknown | unknown | unknown | BA.5.2 | GRA |
| hCoV-19/Morocco/IPM20422772/2022 | EPI_ISL_15291870 | 24/06/2022 | Africa / Morocco / Casablanca | Human | unknown | Female | 54 | unknown | unknown | unknown | BA.5.2 | GRA |
| hCoV-19/Morocco/IPM20422763/2022 | EPI_ISL_15291869 | 24/06/2022 | Africa / Morocco / Casablanca | Human | unknown | Female | 32 | unknown | unknown | unknown | BA.5.1 | GRA |
| hCoV-19/Morocco/IPM20422734/2022 | EPI_ISL_15291866 | 24/06/2022 | Africa / Morocco / Casablanca | Human | unknown | Female | 71 | unknown | unknown | unknown | BA.5.1 | GRA |
| hCoV-19/Morocco/IPM20422756/2022 | EPI_ISL_15291868 | 24/06/2022 | Africa / Morocco / Casablanca | Human | unknown | Female | 49 | unknown | unknown | unknown | BA.4.1 | GRA |
| hCoV-19/Morocco/IPM20422748/2022 | EPI_ISL_15291867 | 24/06/2022 | Africa / Morocco / Casablanca | Human | unknown | Female | 71 | unknown | unknown | unknown | BA.2 | GRA |
| hCoV-19/Morocco/IPM20422714/2022 | EPI_ISL_15291864 | 24/06/2022 | Africa / Morocco / Casablanca | Human | unknown | Male | 43 | unknown | unknown | unknown | BA.5.2 | GRA |
| hCoV-19/Morocco/IPM20422702/2022 | EPI_ISL_15291863 | 24/06/2022 | Africa / Morocco / Casablanca | Human | unknown | Male | 62 | unknown | unknown | unknown | BA.5.2 | GRA |
| hCoV-19/Morocco/IPM20422691/2022 | EPI_ISL_15291862 | 24/06/2022 | Africa / Morocco / Casablanca | Human | unknown | Female | 48 | unknown | unknown | unknown | BA.5.2.20 | GRA |
| hCoV-19/Morocco/IPM20422687/2022 | EPI_ISL_15291861 | 24/06/2022 | Africa / Morocco / Casablanca | Human | unknown | Female | 22 | unknown | unknown | unknown | BA.5.2.20 | GRA |
| hCoV-19/Morocco/IPM20422652/2022 | EPI_ISL_15291860 | 24/06/2022 | Africa / Morocco / Casablanca | Human | unknown | Female | 22 | unknown | unknown | unknown | BA.5.2.20 | GRA |
| hCoV-19/Morocco/IPM20422650/2022 | EPI_ISL_15291859 | 24/06/2022 | Africa / Morocco / Casablanca | Human | unknown | Male | 44 | unknown | unknown | unknown | BA.5.2.20 | GRA |
| hCoV-19/Morocco/IPM20422641/2022 | EPI_ISL_15291858 | 24/06/2022 | Africa / Morocco / Casablanca | Human | unknown | Female | 12 | unknown | unknown | unknown | BA.5.2.20 | GRA |
| hCoV-19/Morocco/IPM20422632/2022 | EPI_ISL_15291857 | 24/06/2022 | Africa / Morocco / Casablanca | Human | unknown | Female | 30 | unknown | unknown | unknown | BA.5.2.20 | GRA |
| hCoV-19/Morocco/IPM20422726/2022 | EPI_ISL_15291865 | 24/06/2022 | Africa / Morocco / Casablanca | Human | unknown | Male | 53 | unknown | unknown | unknown | BA.2.12.1 | GRA |
| hCoV-19/Morocco/IPM20422553/2022 | EPI_ISL_15291853 | 24/06/2022 | Africa / Morocco / Casablanca | Human | unknown | Male | 53 | unknown | unknown | unknown | BA.5.2.20 | GRA |
| hCoV-19/Morocco/IPM20422627/2022 | EPI_ISL_15291856 | 24/06/2022 | Africa / Morocco / Casablanca | Human | unknown | Female | 40 | unknown | unknown | unknown | BA.5.2.20 | GRA |
| hCoV-19/Morocco/IPM20422564/2022 | EPI_ISL_15291855 | 24/06/2022 | Africa / Morocco / Casablanca | Human | unknown | Female | 1 | unknown | unknown | unknown | BA.5.2.20 | GRA |
| hCoV-19/Morocco/IPM20422560/2022 | EPI_ISL_15291854 | 24/06/2022 | Africa / Morocco / Casablanca | Human | unknown | Male | 34 | unknown | unknown | unknown | BA.2.79 | GRA |
| hCoV-19/Morocco/IPM20422545/2022 | EPI_ISL_15291852 | 23/06/2022 | Africa / Morocco / Casablanca | Human | unknown | Female | 40 | unknown | unknown | unknown | BA.5.2.20 | GRA |
| hCoV-19/Morocco/IPM20422526/2022 | EPI_ISL_15291851 | 23/06/2022 | Africa / Morocco / Casablanca | Human | unknown | Male | 56 | unknown | unknown | unknown | BA.5.2.20 | GRA |
| hCoV-19/Morocco/IPM20422499/2022 | EPI_ISL_15291848 | 23/06/2022 | Africa / Morocco / Casablanca | Human | unknown | Male | 71 | unknown | unknown | unknown | BA.5.2.1 | GRA |
| hCoV-19/Morocco/IPM20422521/2022 | EPI_ISL_15291850 | 23/06/2022 | Africa / Morocco / Casablanca | Human | unknown | Male | 38 | unknown | unknown | unknown | BA.5.2 | GRA |
| hCoV-19/Morocco/IPM20422503/2022 | EPI_ISL_15291849 | 23/06/2022 | Africa / Morocco / Casablanca | Human | unknown | Male | 46 | unknown | unknown | unknown | BA.5.1 | GRA |
| hCoV-19/Morocco/IPM20422406/2022 | EPI_ISL_15291847 | 23/06/2022 | Africa / Morocco / Casablanca | Human | unknown | Male | 34 | unknown | unknown | unknown | BA.5.2.20 | GRA |
| hCoV-19/Morocco/IPM20422201/2022 | EPI_ISL_15291846 | 22/06/2022 | Africa / Morocco / Casablanca | Human | unknown | Male | 27 | unknown | unknown | unknown | BA.5.2.20 | GRA |
| hCoV-19/Morocco/IPM20422173/2022 | EPI_ISL_15291845 | 22/06/2022 | Africa / Morocco / Casablanca | Human | unknown | Female | 62 | unknown | unknown | unknown | BA.5.2 | GRA |
| hCoV-19/Morocco/IPM20422143/2022 | EPI_ISL_15291842 | 22/06/2022 | Africa / Morocco / Casablanca | Human | unknown | Male | 19 | unknown | unknown | unknown | BA.2 | GRA |
| hCoV-19/Morocco/IPM20422153/2022 | EPI_ISL_15291844 | 22/06/2022 | Africa / Morocco / Casablanca | Human | unknown | Male | 24 | unknown | unknown | unknown | BA.5.2.1 | GRA |
| hCoV-19/Morocco/IPM20422146/2022 | EPI_ISL_15291843 | 22/06/2022 | Africa / Morocco / Casablanca | Human | unknown | Male | 54 | unknown | unknown | unknown | BA.5.2 | GRA |
| hCoV-19/Morocco/IPM20422136/2022 | EPI_ISL_15291841 | 22/06/2022 | Africa / Morocco / Casablanca | Human | unknown | Male | 24 | unknown | unknown | unknown | BA.5.2.20 | GRA |
| hCoV-19/Morocco/IPM20422127/2022 | EPI_ISL_15291840 | 22/06/2022 | Africa / Morocco / Casablanca | Human | unknown | Female | 22 | unknown | unknown | unknown | BA.5.2.20 | GRA |
| hCoV-19/Morocco/IPM20422103/2022 | EPI_ISL_15291837 | 22/06/2022 | Africa / Morocco / Casablanca | Human | unknown | Female | 32 | unknown | unknown | unknown | BA.5.2.20 | GRA |
| hCoV-19/Morocco/IPM20422122/2022 | EPI_ISL_15291839 | 22/06/2022 | Africa / Morocco / Casablanca | Human | unknown | Female | 41 | unknown | unknown | unknown | BA.5.2.20 | GRA |
| hCoV-19/Morocco/IPM20422111/2022 | EPI_ISL_15291838 | 22/06/2022 | Africa / Morocco / Casablanca | Human | unknown | Male | 69 | unknown | unknown | unknown | BA.5.2 | GRA |
| hCoV-19/Morocco/IPM20422088/2022 | EPI_ISL_15291836 | 22/06/2022 | Africa / Morocco / Casablanca | Human | unknown | Male | 71 | unknown | unknown | unknown | BA.2 | GRA |
| hCoV-19/Morocco/IPM20422075/2022 | EPI_ISL_15291835 | 22/06/2022 | Africa / Morocco / Casablanca | Human | unknown | Male | 59 | unknown | unknown | unknown | BA.2.9.3 | GRA |
| hCoV-19/Morocco/IPM20422044/2022 | EPI_ISL_15291832 | 22/06/2022 | Africa / Morocco / Casablanca | Human | unknown | Male | 52 | unknown | unknown | unknown | BA.5.2.20 | GRA |
| hCoV-19/Morocco/IPM20422062/2022 | EPI_ISL_15291834 | 22/06/2022 | Africa / Morocco / Casablanca | Human | unknown | Female | 51 | unknown | unknown | unknown | BA.5.2.20 | GRA |
| hCoV-19/Morocco/IPM20422050/2022 | EPI_ISL_15291833 | 22/06/2022 | Africa / Morocco / Casablanca | Human | unknown | Female | 55 | unknown | unknown | unknown | BA.5.2.20 | GRA |
| hCoV-19/Morocco/IPM20422039/2022 | EPI_ISL_15291831 | 22/06/2022 | Africa / Morocco / Casablanca | Human | unknown | Female | 66 | unknown | unknown | unknown | BA.5.2 | GRA |
| hCoV-19/Morocco/IPM20422009/2022 | EPI_ISL_15291828 | 22/06/2022 | Africa / Morocco / Casablanca | Human | unknown | Male | 50 | unknown | unknown | unknown | BA.5.2.20 | GRA |
| hCoV-19/Morocco/IPM20422027/2022 | EPI_ISL_15291830 | 22/06/2022 | Africa / Morocco / Casablanca | Human | unknown | Female | 50 | unknown | unknown | unknown | BA.5.2 | GRA |
| hCoV-19/Morocco/IPM20422019/2022 | EPI_ISL_15291829 | 22/06/2022 | Africa / Morocco / Casablanca | Human | unknown | Male | 42 | unknown | unknown | unknown | BA.5.1.23 | GRA |
| hCoV-19/Morocco/IPM20421994/2022 | EPI_ISL_15291827 | 22/06/2022 | Africa / Morocco / Casablanca | Human | unknown | Male | 36 | unknown | unknown | unknown | BA.5.1 | GRA |
| hCoV-19/Morocco/IPM20421971/2022 | EPI_ISL_15291824 | 22/06/2022 | Africa / Morocco / Casablanca | Human | unknown | Male | 16 | unknown | unknown | unknown | BA.5.2 | GRA |
| hCoV-19/Morocco/IPM20421983/2022 | EPI_ISL_15291826 | 22/06/2022 | Africa / Morocco / Casablanca | Human | unknown | Male | 28 | unknown | unknown | unknown | BF.5 | GRA |
| hCoV-19/Morocco/IPM20421975/2022 | EPI_ISL_15291825 | 22/06/2022 | Africa / Morocco / Casablanca | Human | unknown | Female | 36 | unknown | unknown | unknown | BA.5.2.20 | GRA |
| hCoV-19/Morocco/IPM20421960/2022 | EPI_ISL_15291823 | 22/06/2022 | Africa / Morocco / Casablanca | Human | unknown | Female | 35 | unknown | unknown | unknown | BA.5.2.20 | GRA |
| hCoV-19/Morocco/IPM20421941/2022 | EPI_ISL_15291821 | 22/06/2022 | Africa / Morocco / Casablanca | Human | unknown | Male | 63 | unknown | unknown | unknown | BA.2 | GRA |
| hCoV-19/Morocco/IPM20421924/2022 | EPI_ISL_15291820 | 22/06/2022 | Africa / Morocco / Casablanca | Human | unknown | Female | 58 | unknown | unknown | unknown | BA.5.2 | GRA |
| hCoV-19/Morocco/IPM20421919/2022 | EPI_ISL_15291819 | 22/06/2022 | Africa / Morocco / Casablanca | Human | unknown | Male | 52 | unknown | unknown | unknown | BA.2.9.3 | GRA |
| hCoV-19/Morocco/IPM20421913/2022 | EPI_ISL_15291818 | 22/06/2022 | Africa / Morocco / Casablanca | Human | unknown | Female | 64 | unknown | unknown | unknown | BA.5.2.20 | GRA |
| hCoV-19/Morocco/IPM20421900/2022 | EPI_ISL_15291817 | 22/06/2022 | Africa / Morocco / Casablanca | Human | unknown | Female | 41 | unknown | unknown | unknown | BA.5.2 | GRA |
| hCoV-19/Morocco/IPM20421898/2022 | EPI_ISL_15291816 | 22/06/2022 | Africa / Morocco / Casablanca | Human | unknown | Male | 33 | unknown | unknown | unknown | BA.5.2 | GRA |
| hCoV-19/Morocco/IPM20421849/2022 | EPI_ISL_15291815 | 22/06/2022 | Africa / Morocco / Casablanca | Human | unknown | Male | 59 | unknown | unknown | unknown | BA.5.2.20 | GRA |
| hCoV-19/Morocco/IPM20421954/2022 | EPI_ISL_15291822 | 22/06/2022 | Africa / Morocco / Casablanca | Human | unknown | Female | 22 | unknown | unknown | unknown | BA.5.2.20 | GRA |
| hCoV-19/Morocco/IPM20421813/2022 | EPI_ISL_15291811 | 22/06/2022 | Africa / Morocco / Casablanca | Human | unknown | Female | 70 | unknown | unknown | unknown | BA.5.2.20 | GRA |
| hCoV-19/Morocco/IPM20421840/2022 | EPI_ISL_15291814 | 22/06/2022 | Africa / Morocco / Casablanca | Human | unknown | Female | 38 | unknown | unknown | unknown | BA.5.2 | GRA |
| hCoV-19/Morocco/IPM20421831/2022 | EPI_ISL_15291813 | 22/06/2022 | Africa / Morocco / Casablanca | Human | unknown | Male | 62 | unknown | unknown | unknown | BA.2 | GRA |
| hCoV-19/Morocco/IPM20421820/2022 | EPI_ISL_15291812 | 22/06/2022 | Africa / Morocco / Casablanca | Human | unknown | Male | 49 | unknown | unknown | unknown | BA.4 | GRA |
| hCoV-19/Morocco/IPM20421802/2022 | EPI_ISL_15291810 | 22/06/2022 | Africa / Morocco / Casablanca | Human | unknown | Male | 99 | unknown | unknown | unknown | BA.5.2 | GRA |
| hCoV-19/Morocco/IPM20421765/2022 | EPI_ISL_15291807 | 21/06/2022 | Africa / Morocco / Casablanca | Human | unknown | Male | 61 | unknown | unknown | unknown | BA.5.2.20 | GRA |
| hCoV-19/Morocco/IPM20421793/2022 | EPI_ISL_15291809 | 22/06/2022 | Africa / Morocco / Casablanca | Human | unknown | Female | 65 | unknown | unknown | unknown | BA.5.2 | GRA |
| hCoV-19/Morocco/IPM20421778/2022 | EPI_ISL_15291808 | 22/06/2022 | Africa / Morocco / Casablanca | Human | unknown | Male | 37 | unknown | unknown | unknown | BA.5.2.20 | GRA |
| hCoV-19/Morocco/IPM20421754/2022 | EPI_ISL_15291806 | 21/06/2022 | Africa / Morocco / Casablanca | Human | unknown | Male | 34 | unknown | unknown | unknown | BA.5.2.20 | GRA |
| hCoV-19/Morocco/IPM20421696/2022 | EPI_ISL_15291802 | 21/06/2022 | Africa / Morocco / Casablanca | Human | unknown | Female | 60 | unknown | unknown | unknown | BF.5 | GRA |
| hCoV-19/Morocco/IPM20421746/2022 | EPI_ISL_15291805 | 21/06/2022 | Africa / Morocco / Casablanca | Human | unknown | Male | 59 | unknown | unknown | unknown | BA.5.2 | GRA |
| hCoV-19/Morocco/IPM20421731/2022 | EPI_ISL_15291804 | 21/06/2022 | Africa / Morocco / Casablanca | Human | unknown | Male | 23 | unknown | unknown | unknown | BF.5 | GRA |
| hCoV-19/Morocco/IPM20421699/2022 | EPI_ISL_15291803 | 21/06/2022 | Africa / Morocco / Casablanca | Human | unknown | Female | 37 | unknown | unknown | unknown | BA.5.2.20 | GRA |
| hCoV-19/Morocco/IPM20421676/2022 | EPI_ISL_15291801 | 21/06/2022 | Africa / Morocco / Casablanca | Human | unknown | Female | 27 | unknown | unknown | unknown | BA.5.2 | GRA |
| hCoV-19/Morocco/IPM20421663/2022 | EPI_ISL_15291800 | 21/06/2022 | Africa / Morocco / Casablanca | Human | unknown | Female | 87 | unknown | unknown | unknown | BA.5.2 | GRA |
| hCoV-19/Morocco/IPM20421622/2022 | EPI_ISL_15291796 | 21/06/2022 | Africa / Morocco / Casablanca | Human | unknown | Male | 80 | unknown | unknown | unknown | BA.5.2 | GRA |
| hCoV-19/Morocco/IPM20421655/2022 | EPI_ISL_15291799 | 21/06/2022 | Africa / Morocco / Casablanca | Human | unknown | Male | 19 | unknown | unknown | unknown | BA.5.2 | GRA |
| hCoV-19/Morocco/IPM20421651/2022 | EPI_ISL_15291798 | 21/06/2022 | Africa / Morocco / Casablanca | Human | unknown | Female | 47 | unknown | unknown | unknown | BF.19 | GRA |
| hCoV-19/Morocco/IPM20421625/2022 | EPI_ISL_15291797 | 21/06/2022 | Africa / Morocco / Casablanca | Human | unknown | Female | 29 | unknown | unknown | unknown | BA.5.2 | GRA |
| hCoV-19/Morocco/IPM20421594/2022 | EPI_ISL_15291793 | 21/06/2022 | Africa / Morocco / Casablanca | Human | unknown | Female | 64 | unknown | unknown | unknown | BA.5.2 | GRA |
| hCoV-19/Morocco/IPM20421584/2022 | EPI_ISL_15291792 | 21/06/2022 | Africa / Morocco / Casablanca | Human | unknown | Male | 66 | unknown | unknown | unknown | BA.5.2 | GRA |
| hCoV-19/Morocco/IPM20421554/2022 | EPI_ISL_15291791 | 20/06/2022 | Africa / Morocco / Casablanca | Human | unknown | Male | 15 | unknown | unknown | unknown | BA.5.2 | GRA |
| hCoV-19/Morocco/IPM20421548/2022 | EPI_ISL_15291790 | 20/06/2022 | Africa / Morocco / Casablanca | Human | unknown | Female | 99 | unknown | unknown | unknown | BA.5.2 | GRA |
| hCoV-19/Morocco/IPM20421618/2022 | EPI_ISL_15291795 | 21/06/2022 | Africa / Morocco / Casablanca | Human | unknown | Male | 56 | unknown | unknown | unknown | BA.5.1 | GRA |
| hCoV-19/Morocco/IPM20421609/2022 | EPI_ISL_15291794 | 21/06/2022 | Africa / Morocco / Casablanca | Human | unknown | Male | 48 | unknown | unknown | unknown | BA.5.2 | GRA |
| hCoV-19/Morocco/IPM20421516/2022 | EPI_ISL_15291786 | 20/06/2022 | Africa / Morocco / Casablanca | Human | unknown | Female | 67 | unknown | unknown | unknown | BA.2 | GRA |
| hCoV-19/Morocco/IPM20421539/2022 | EPI_ISL_15291789 | 20/06/2022 | Africa / Morocco / Casablanca | Human | unknown | Male | 65 | unknown | unknown | unknown | BA.2.36 | GRA |
| hCoV-19/Morocco/IPM20421525/2022 | EPI_ISL_15291788 | 20/06/2022 | Africa / Morocco / Casablanca | Human | unknown | Female | 59 | unknown | unknown | unknown | BA.5.1 | GRA |
| hCoV-19/Morocco/IPM20421520/2022 | EPI_ISL_15291787 | 20/06/2022 | Africa / Morocco / Casablanca | Human | unknown | Female | 68 | unknown | unknown | unknown | BA.5.2 | GRA |
| hCoV-19/Morocco/IPM20421489/2022 | EPI_ISL_15291785 | 20/06/2022 | Africa / Morocco / Casablanca | Human | unknown | Male | 34 | unknown | unknown | unknown | B.1.617.2 | GK |
| hCoV-19/Morocco/IPM20421419/2022 | EPI_ISL_15291782 | 20/06/2022 | Africa / Morocco / Casablanca | Human | unknown | Female | 30 | unknown | unknown | unknown | BA.5.2.20 | GRA |
| hCoV-19/Morocco/IPM20421456/2022 | EPI_ISL_15291784 | 20/06/2022 | Africa / Morocco / Casablanca | Human | unknown | Male | 24 | unknown | unknown | unknown | BA.2 | GRA |
| hCoV-19/Morocco/IPM20421431/2022 | EPI_ISL_15291783 | 20/06/2022 | Africa / Morocco / Casablanca | Human | unknown | Male | 37 | unknown | unknown | unknown | BA.5.2 | GRA |
| hCoV-19/Morocco/IPM20421411/2022 | EPI_ISL_15291781 | 20/06/2022 | Africa / Morocco / Casablanca | Human | unknown | Male | 36 | unknown | unknown | unknown | BA.4 | GRA |
| hCoV-19/Morocco/IPM20421404/2022 | EPI_ISL_15291780 | 20/06/2022 | Africa / Morocco / Casablanca | Human | unknown | Male | 40 | unknown | unknown | unknown | BA.5.2.20 | GRA |
| hCoV-19/Morocco/IPM20421383/2022 | EPI_ISL_15291776 | 20/06/2022 | Africa / Morocco / Casablanca | Human | unknown | Female | 76 | unknown | unknown | unknown | BA.5.2.20 | GRA |
| hCoV-19/Morocco/IPM20421393/2022 | EPI_ISL_15291779 | 20/06/2022 | Africa / Morocco / Casablanca | Human | unknown | Female | 50 | unknown | unknown | unknown | BA.5.2.20 | GRA |
| hCoV-19/Morocco/IPM20421388/2022 | EPI_ISL_15291778 | 20/06/2022 | Africa / Morocco / Casablanca | Human | unknown | Female | 88 | unknown | unknown | unknown | BA.5.2 | GRA |
| hCoV-19/Morocco/IPM20421385/2022 | EPI_ISL_15291777 | 20/06/2022 | Africa / Morocco / Casablanca | Human | unknown | Male | 51 | unknown | unknown | unknown | BA.5.2.1 | GRA |
| hCoV-19/Morocco/IPM20421360/2022 | EPI_ISL_15291774 | 20/06/2022 | Africa / Morocco / Casablanca | Human | unknown | Male | 31 | unknown | unknown | unknown | BA.2 | GRA |
| hCoV-19/Morocco/IPM20421357/2022 | EPI_ISL_15291773 | 20/06/2022 | Africa / Morocco / Casablanca | Human | unknown | Male | 50 | unknown | unknown | unknown | BA.5.1 | GRA |
| hCoV-19/Morocco/IPM20421371/2022 | EPI_ISL_15291775 | 20/06/2022 | Africa / Morocco / Casablanca | Human | unknown | Male | 65 | unknown | unknown | unknown | BA.5.2 | GRA |
| hCoV-19/Morocco/IPM20421343/2022 | EPI_ISL_15291770 | 20/06/2022 | Africa / Morocco / Casablanca | Human | unknown | Female | 49 | unknown | unknown | unknown | BA.2 | GRA |
| hCoV-19/Morocco/IPM20421353/2022 | EPI_ISL_15291772 | 20/06/2022 | Africa / Morocco / Casablanca | Human | unknown | Male | 35 | unknown | unknown | unknown | BA.2.36 | GRA |
| hCoV-19/Morocco/IPM20421348/2022 | EPI_ISL_15291771 | 20/06/2022 | Africa / Morocco / Casablanca | Human | unknown | Male | 39 | unknown | unknown | unknown | BA.5.1.23 | GRA |
| hCoV-19/Morocco/IPM20421332/2022 | EPI_ISL_15291769 | 20/06/2022 | Africa / Morocco / Casablanca | Human | unknown | Male | 40 | unknown | unknown | unknown | BA.5.2 | GRA |
| hCoV-19/Morocco/IPM20421299/2022 | EPI_ISL_15291768 | 20/06/2022 | Africa / Morocco / Casablanca | Human | unknown | Female | 38 | unknown | unknown | unknown | BA.5.2 | GRA |
| hCoV-19/Morocco/IPM20421292/2022 | EPI_ISL_15291767 | 20/06/2022 | Africa / Morocco / Casablanca | Human | unknown | Female | 65 | unknown | unknown | unknown | BA.2 | GRA |
| hCoV-19/Morocco/IPM20421291/2022 | EPI_ISL_15291766 | 20/06/2022 | Africa / Morocco / Casablanca | Human | unknown | Female | 22 | unknown | unknown | unknown | BA.5.2.20 | GRA |
| hCoV-19/Morocco/IPM20421267/2022 | EPI_ISL_15291765 | 20/06/2022 | Africa / Morocco / Casablanca | Human | unknown | Female | 25 | unknown | unknown | unknown | BA.5.2.20 | GRA |
| hCoV-19/Morocco/IPM20421262/2022 | EPI_ISL_15291764 | 20/06/2022 | Africa / Morocco / Casablanca | Human | unknown | Male | 45 | unknown | unknown | unknown | BA.5.1.22 | GRA |
| hCoV-19/Morocco/IPM20421252/2022 | EPI_ISL_15291763 | 20/06/2022 | Africa / Morocco / Casablanca | Human | unknown | Male | 51 | unknown | unknown | unknown | BA.5.2.20 | GRA |
| hCoV-19/Morocco/IPM20421244/2022 | EPI_ISL_15291762 | 20/06/2022 | Africa / Morocco / Casablanca | Human | unknown | Female | 26 | unknown | unknown | unknown | BA.5.2.20 | GRA |
| hCoV-19/Morocco/IPM20421241/2022 | EPI_ISL_15291761 | 20/06/2022 | Africa / Morocco / Casablanca | Human | unknown | Male | 46 | unknown | unknown | unknown | BA.5.2 | GRA |
| hCoV-19/Morocco/IPM20421227/2022 | EPI_ISL_15291760 | 20/06/2022 | Africa / Morocco / Casablanca | Human | unknown | Female | 40 | unknown | unknown | unknown | BA.5.2 | GRA |
| hCoV-19/Morocco/IPM20421222/2022 | EPI_ISL_15291759 | 20/06/2022 | Africa / Morocco / Casablanca | Human | unknown | Female | 26 | unknown | unknown | unknown | BA.5.2 | GRA |
| hCoV-19/Morocco/IPM20421216/2022 | EPI_ISL_15291758 | 20/06/2022 | Africa / Morocco / Casablanca | Human | unknown | Male | 47 | unknown | unknown | unknown | BF.5 | GRA |
| hCoV-19/Morocco/IPM20421213/2022 | EPI_ISL_15291757 | 20/06/2022 | Africa / Morocco / Casablanca | Human | unknown | Male | 37 | unknown | unknown | unknown | BA.5.2.20 | GRA |
| hCoV-19/Morocco/IPM20421209/2022 | EPI_ISL_15291756 | 20/06/2022 | Africa / Morocco / Casablanca | Human | unknown | Male | 27 | unknown | unknown | unknown | BA.5.2.20 | GRA |
| hCoV-19/Morocco/IPM20421107/2022 | EPI_ISL_15291755 | 18/06/2022 | Africa / Morocco / Casablanca | Human | unknown | Female | 49 | unknown | unknown | unknown | BA.5.2.20 | GRA |
| hCoV-19/Morocco/IPM20421103/2022 | EPI_ISL_15291754 | 18/06/2022 | Africa / Morocco / Casablanca | Human | unknown | Female | 39 | unknown | unknown | unknown | BA.2.9.3 | GRA |
| hCoV-19/Morocco/IPM20421094/2022 | EPI_ISL_15291753 | 18/06/2022 | Africa / Morocco / Casablanca | Human | unknown | Female | 21 | unknown | unknown | unknown | BA.5.2.20 | GRA |
| hCoV-19/Morocco/IPM20421092/2022 | EPI_ISL_15291752 | 18/06/2022 | Africa / Morocco / Casablanca | Human | unknown | Male | 31 | unknown | unknown | unknown | BA.5.2.20 | GRA |
| hCoV-19/Morocco/IPM20421086/2022 | EPI_ISL_15291751 | 18/06/2022 | Africa / Morocco / Casablanca | Human | unknown | Female | 22 | unknown | unknown | unknown | BA.5.2.20 | GRA |
| hCoV-19/Morocco/IPM20421076/2022 | EPI_ISL_15291750 | 18/06/2022 | Africa / Morocco / Casablanca | Human | unknown | Male | 52 | unknown | unknown | unknown | BA.5.2.20 | GRA |
| hCoV-19/Morocco/IPM20421063/2022 | EPI_ISL_15291749 | 18/06/2022 | Africa / Morocco / Casablanca | Human | unknown | Female | 18 | unknown | unknown | unknown | BA.5.2 | GRA |
| hCoV-19/Morocco/IPM20421024/2022 | EPI_ISL_15291748 | 18/06/2022 | Africa / Morocco / Casablanca | Human | unknown | Male | 69 | unknown | unknown | unknown | BA.5.2.20 | GRA |
| hCoV-19/Morocco/IPM20421021/2022 | EPI_ISL_15291747 | 18/06/2022 | Africa / Morocco / Casablanca | Human | unknown | Female | 22 | unknown | unknown | unknown | BA.5.2 | GRA |
| hCoV-19/Morocco/IPM20421001/2022 | EPI_ISL_15291744 | 17/06/2022 | Africa / Morocco / Casablanca | Human | unknown | Female | 24 | unknown | unknown | unknown | BA.5.1 | GRA |
| hCoV-19/Morocco/IPM20421014/2022 | EPI_ISL_15291746 | 18/06/2022 | Africa / Morocco / Casablanca | Human | unknown | Male | 70 | unknown | unknown | unknown | BA.2.9.3 | GRA |
| hCoV-19/Morocco/IPM20421006/2022 | EPI_ISL_15291745 | 17/06/2022 | Africa / Morocco / Casablanca | Human | unknown | Male | 37 | unknown | unknown | unknown | BA.5.2.20 | GRA |
| hCoV-19/Morocco/IPM20420984/2022 | EPI_ISL_15291743 | 17/06/2022 | Africa / Morocco / Casablanca | Human | unknown | Male | 79 | unknown | unknown | unknown | BA.5.2.20 | GRA |
| hCoV-19/Morocco/IPM20420976/2022 | EPI_ISL_15291742 | 17/06/2022 | Africa / Morocco / Casablanca | Human | unknown | Male | 41 | unknown | unknown | unknown | BA.5.2.20 | GRA |
| hCoV-19/Morocco/IPM20420973/2022 | EPI_ISL_15291741 | 17/06/2022 | Africa / Morocco / Casablanca | Human | unknown | Female | 30 | unknown | unknown | unknown | BA.5.2.20 | GRA |
| hCoV-19/Morocco/IPM20420972/2022 | EPI_ISL_15291740 | 17/06/2022 | Africa / Morocco / Casablanca | Human | unknown | Male | 32 | unknown | unknown | unknown | BA.5.1 | GRA |
| hCoV-19/Morocco/IPM20420955/2022 | EPI_ISL_15291737 | 17/06/2022 | Africa / Morocco / Casablanca | Human | unknown | Male | 23 | unknown | unknown | unknown | BA.5.2.20 | GRA |
| hCoV-19/Morocco/IPM20420970/2022 | EPI_ISL_15291739 | 17/06/2022 | Africa / Morocco / Casablanca | Human | unknown | Male | 33 | unknown | unknown | unknown | BA.5.2.20 | GRA |
| hCoV-19/Morocco/IPM20420964/2022 | EPI_ISL_15291738 | 17/06/2022 | Africa / Morocco / Casablanca | Human | unknown | Female | 33 | unknown | unknown | unknown | BA.5.2.20 | GRA |
| hCoV-19/Morocco/IPM20420930/2022 | EPI_ISL_15291735 | 17/06/2022 | Africa / Morocco / Casablanca | Human | unknown | Male | 57 | unknown | unknown | unknown | BA.5.2.20 | GRA |
| hCoV-19/Morocco/IPM20420919/2022 | EPI_ISL_15291734 | 17/06/2022 | Africa / Morocco / Casablanca | Human | unknown | Female | 28 | unknown | unknown | unknown | BA.5.1.23 | GRA |
| hCoV-19/Morocco/IPM20420893/2022 | EPI_ISL_15291733 | 17/06/2022 | Africa / Morocco / Casablanca | Human | unknown | Female | 25 | unknown | unknown | unknown | BA.5.2.20 | GRA |
| hCoV-19/Morocco/IPM20420888/2022 | EPI_ISL_15291732 | 17/06/2022 | Africa / Morocco / Casablanca | Human | unknown | Male | 23 | unknown | unknown | unknown | BA.5.2.20 | GRA |
| hCoV-19/Morocco/IPM20420938/2022 | EPI_ISL_15291736 | 17/06/2022 | Africa / Morocco / Casablanca | Human | unknown | Male | 59 | unknown | unknown | unknown | BA.5.2.1 | GRA |
| hCoV-19/Morocco/IPM20420876/2022 | EPI_ISL_15291729 | 17/06/2022 | Africa / Morocco / Casablanca | Human | unknown | Male | 28 | unknown | unknown | unknown | BA.5.2 | GRA |
| hCoV-19/Morocco/IPM20420874/2022 | EPI_ISL_15291728 | 17/06/2022 | Africa / Morocco / Casablanca | Human | unknown | Male | 39 | unknown | unknown | unknown | BA.5.2.20 | GRA |
| hCoV-19/Morocco/IPM20420871/2022 | EPI_ISL_15291727 | 17/06/2022 | Africa / Morocco / Casablanca | Human | unknown | Female | 56 | unknown | unknown | unknown | BA.5.2 | GRA |
| hCoV-19/Morocco/IPM20420884/2022 | EPI_ISL_15291731 | 17/06/2022 | Africa / Morocco / Casablanca | Human | unknown | Male | 33 | unknown | unknown | unknown | BA.5.2.1 | GRA |
| hCoV-19/Morocco/IPM20420878/2022 | EPI_ISL_15291730 | 17/06/2022 | Africa / Morocco / Casablanca | Human | unknown | Male | 29 | unknown | unknown | unknown | BA.5.2 | GRA |
| hCoV-19/Morocco/IPM20420870/2022 | EPI_ISL_15291726 | 17/06/2022 | Africa / Morocco / Casablanca | Human | unknown | Male | 34 | unknown | unknown | unknown | BA.5.2.20 | GRA |
| hCoV-19/Morocco/IPM20420857/2022 | EPI_ISL_15291723 | 17/06/2022 | Africa / Morocco / Casablanca | Human | unknown | Male | 24 | unknown | unknown | unknown | B.1.617.2 | GK |
| hCoV-19/Morocco/IPM20420866/2022 | EPI_ISL_15291725 | 17/06/2022 | Africa / Morocco / Casablanca | Human | unknown | Male | 55 | unknown | unknown | unknown | BA.5.2.20 | GRA |
| hCoV-19/Morocco/IPM20420861/2022 | EPI_ISL_15291724 | 17/06/2022 | Africa / Morocco / Casablanca | Human | unknown | Male | 56 | unknown | unknown | unknown | BA.5.2.20 | GRA |
| hCoV-19/Morocco/IPM20420847/2022 | EPI_ISL_15291722 | 17/06/2022 | Africa / Morocco / Casablanca | Human | unknown | Male | 23 | unknown | unknown | unknown | BA.5.2.20 | GRA |
| hCoV-19/Morocco/IPM20420836/2022 | EPI_ISL_15291721 | 17/06/2022 | Africa / Morocco / Casablanca | Human | unknown | Female | 58 | unknown | unknown | unknown | BA.2.9.3 | GRA |
| hCoV-19/Morocco/IPM20420814/2022 | EPI_ISL_15291718 | 17/06/2022 | Africa / Morocco / Casablanca | Human | unknown | Female | 85 | unknown | unknown | unknown | BA.5.2.20 | GRA |
| hCoV-19/Morocco/IPM20420811/2022 | EPI_ISL_15291717 | 17/06/2022 | Africa / Morocco / Casablanca | Human | unknown | Male | 17 | unknown | unknown | unknown | BA.5.1 | GRA |
| hCoV-19/Morocco/IPM20420806/2022 | EPI_ISL_15291716 | 17/06/2022 | Africa / Morocco / Casablanca | Human | unknown | Male | 35 | unknown | unknown | unknown | BA.5.2 | GRA |
| hCoV-19/Morocco/IPM20420830/2022 | EPI_ISL_15291720 | 17/06/2022 | Africa / Morocco / Casablanca | Human | unknown | Female | 43 | unknown | unknown | unknown | BA.5.2 | GRA |
| hCoV-19/Morocco/IPM20420818/2022 | EPI_ISL_15291719 | 17/06/2022 | Africa / Morocco / Casablanca | Human | unknown | Female | 57 | unknown | unknown | unknown | BA.5.2.20 | GRA |
| hCoV-19/Morocco/IPM20420789/2022 | EPI_ISL_15291715 | 17/06/2022 | Africa / Morocco / Casablanca | Human | unknown | Male | 69 | unknown | unknown | unknown | BA.5.2.20 | GRA |
| hCoV-19/Morocco/IPM20420781/2022 | EPI_ISL_15291714 | 17/06/2022 | Africa / Morocco / Casablanca | Human | unknown | Male | 29 | unknown | unknown | unknown | BA.2.11 | GRA |
| hCoV-19/Morocco/IPM20420760/2022 | EPI_ISL_15291713 | 17/06/2022 | Africa / Morocco / Casablanca | Human | unknown | Female | 26 | unknown | unknown | unknown | BA.2.9.3 | GRA |
| hCoV-19/Morocco/IPM20420750/2022 | EPI_ISL_15291712 | 17/06/2022 | Africa / Morocco / Casablanca | Human | unknown | Male | 33 | unknown | unknown | unknown | BA.2.12.1 | GRA |
| hCoV-19/Morocco/IPM20420734/2022 | EPI_ISL_15291711 | 17/06/2022 | Africa / Morocco / Casablanca | Human | unknown | Male | 50 | unknown | unknown | unknown | BA.5.2 | GRA |
| hCoV-19/Morocco/IPM20420721/2022 | EPI_ISL_15291710 | 17/06/2022 | Africa / Morocco / Casablanca | Human | unknown | Male | 19 | unknown | unknown | unknown | BA.5.2.20 | GRA |
| hCoV-19/Morocco/IPM20420657/2022 | EPI_ISL_15291707 | 16/06/2022 | Africa / Morocco / Casablanca | Human | unknown | Female | 56 | unknown | unknown | unknown | BA.5.2 | GRA |
| hCoV-19/Morocco/IPM20420692/2022 | EPI_ISL_15291709 | 16/06/2022 | Africa / Morocco / Casablanca | Human | unknown | Female | 21 | unknown | unknown | unknown | BA.5.2.20 | GRA |
| hCoV-19/Morocco/IPM20420689/2022 | EPI_ISL_15291708 | 16/06/2022 | Africa / Morocco / Casablanca | Human | unknown | Female | 64 | unknown | unknown | unknown | BA.5.2.20 | GRA |
| hCoV-19/Morocco/IPM20420656/2022 | EPI_ISL_15291706 | 16/06/2022 | Africa / Morocco / Casablanca | Human | unknown | Female | 59 | unknown | unknown | unknown | BA.5.2.20 | GRA |
| hCoV-19/Morocco/IPM20420637/2022 | EPI_ISL_15291705 | 16/06/2022 | Africa / Morocco / Casablanca | Human | unknown | Female | 25 | unknown | unknown | unknown | BA.4.3 | GRA |
| hCoV-19/Morocco/IPM20420616/2022 | EPI_ISL_15291701 | 16/06/2022 | Africa / Morocco / Casablanca | Human | unknown | Female | 41 | unknown | unknown | unknown | BA.5.2.1 | GRA |
| hCoV-19/Morocco/IPM20420635/2022 | EPI_ISL_15291704 | 16/06/2022 | Africa / Morocco / Casablanca | Human | unknown | Female | 72 | unknown | unknown | unknown | BA.5.2.20 | GRA |
| hCoV-19/Morocco/IPM20420632/2022 | EPI_ISL_15291703 | 16/06/2022 | Africa / Morocco / Casablanca | Human | unknown | Male | 24 | unknown | unknown | unknown | BA.5.2.20 | GRA |
| hCoV-19/Morocco/IPM20420624/2022 | EPI_ISL_15291702 | 16/06/2022 | Africa / Morocco / Casablanca | Human | unknown | Female | 35 | unknown | unknown | unknown | BA.2.9.3 | GRA |
| hCoV-19/Morocco/IPM20420598/2022 | EPI_ISL_15291699 | 16/06/2022 | Africa / Morocco / Casablanca | Human | unknown | Female | 54 | unknown | unknown | unknown | BA.2.9.3 | GRA |
| hCoV-19/Morocco/IPM20420591/2022 | EPI_ISL_15291698 | 16/06/2022 | Africa / Morocco / Casablanca | Human | unknown | Female | 25 | unknown | unknown | unknown | BA.5.1.2 | GRA |
| hCoV-19/Morocco/IPM20420604/2022 | EPI_ISL_15291700 | 16/06/2022 | Africa / Morocco / Casablanca | Human | unknown | Female | 58 | unknown | unknown | unknown | BA.2.79 | GRA |
| hCoV-19/Morocco/IPM20420517/2022 | EPI_ISL_15291695 | 15/06/2022 | Africa / Morocco / Casablanca | Human | unknown | Female | 41 | unknown | unknown | unknown | BA.2 | GRA |
| hCoV-19/Morocco/IPM20420525/2022 | EPI_ISL_15291697 | 15/06/2022 | Africa / Morocco / Casablanca | Human | unknown | Female | 42 | unknown | unknown | unknown | BA.5.2.20 | GRA |
| hCoV-19/Morocco/IPM20420520/2022 | EPI_ISL_15291696 | 15/06/2022 | Africa / Morocco / Casablanca | Human | unknown | Male | 26 | unknown | unknown | unknown | BA.5.2.20 | GRA |
| hCoV-19/Morocco/IPM20420492/2022 | EPI_ISL_15291694 | 15/06/2022 | Africa / Morocco / Casablanca | Human | unknown | Male | 79 | unknown | unknown | unknown | BA.5.2.1 | GRA |
| hCoV-19/Morocco/IPM20420472/2022 | EPI_ISL_15291691 | 15/06/2022 | Africa / Morocco / Casablanca | Human | unknown | Female | 36 | unknown | unknown | unknown | BA.5.2 | GRA |
| hCoV-19/Morocco/IPM20420484/2022 | EPI_ISL_15291693 | 15/06/2022 | Africa / Morocco / Casablanca | Human | unknown | Female | 15 | unknown | unknown | unknown | BA.2 | GRA |
| hCoV-19/Morocco/IPM20420480/2022 | EPI_ISL_15291692 | 15/06/2022 | Africa / Morocco / Casablanca | Human | unknown | Female | 68 | unknown | unknown | unknown | BA.5.2 | GRA |
| hCoV-19/Morocco/IPM20420463/2022 | EPI_ISL_15291690 | 15/06/2022 | Africa / Morocco / Casablanca | Human | unknown | Female | 54 | unknown | unknown | unknown | BA.5.2 | GRA |
| hCoV-19/Morocco/IPM20420391/2022 | EPI_ISL_15291687 | 14/06/2022 | Africa / Morocco / Casablanca | Human | unknown | Male | 37 | unknown | unknown | unknown | BA.5.2.20 | GRA |
| hCoV-19/Morocco/IPM20420440/2022 | EPI_ISL_15291689 | 15/06/2022 | Africa / Morocco / Casablanca | Human | unknown | Female | 29 | unknown | unknown | unknown | BA.5.2.1 | GRA |
| hCoV-19/Morocco/IPM20420417/2022 | EPI_ISL_15291688 | 15/06/2022 | Africa / Morocco / Casablanca | Human | unknown | Male | 36 | unknown | unknown | unknown | BA.5.2.1 | GRA |
| hCoV-19/Morocco/IPM20420384/2022 | EPI_ISL_15291686 | 14/06/2022 | Africa / Morocco / Casablanca | Human | unknown | Female | 40 | unknown | unknown | unknown | BA.5.2.20 | GRA |
| hCoV-19/Morocco/IPM20420351/2022 | EPI_ISL_15291683 | 14/06/2022 | Africa / Morocco / Casablanca | Human | unknown | Female | 52 | unknown | unknown | unknown | BA.5.2.20 | GRA |
| hCoV-19/Morocco/IPM20420374/2022 | EPI_ISL_15291685 | 14/06/2022 | Africa / Morocco / Casablanca | Human | unknown | Female | 42 | unknown | unknown | unknown | BA.5 | GRA |
| hCoV-19/Morocco/IPM20420368/2022 | EPI_ISL_15291684 | 14/06/2022 | Africa / Morocco / Casablanca | Human | unknown | Male | 62 | unknown | unknown | unknown | BF.5 | GRA |
| hCoV-19/Morocco/IPM20420345/2022 | EPI_ISL_15291682 | 14/06/2022 | Africa / Morocco / Casablanca | Human | unknown | Male | 41 | unknown | unknown | unknown | BA.5.2.20 | GRA |
| hCoV-19/Morocco/IPM20420318/2022 | EPI_ISL_15291681 | 14/06/2022 | Africa / Morocco / Casablanca | Human | unknown | Female | 23 | unknown | unknown | unknown | BA.5.2 | GRA |
| hCoV-19/Morocco/IPM20420264/2022 | EPI_ISL_15291677 | 14/06/2022 | Africa / Morocco / Casablanca | Human | unknown | Female | 28 | unknown | unknown | unknown | BA.5.2.20 | GRA |
| hCoV-19/Morocco/IPM20420308/2022 | EPI_ISL_15291680 | 14/06/2022 | Africa / Morocco / Casablanca | Human | unknown | Female | 26 | unknown | unknown | unknown | BA.5.2.20 | GRA |
| hCoV-19/Morocco/IPM20420295/2022 | EPI_ISL_15291679 | 14/06/2022 | Africa / Morocco / Casablanca | Human | unknown | Female | 66 | unknown | unknown | unknown | BA.5.2 | GRA |
| hCoV-19/Morocco/IPM20420284/2022 | EPI_ISL_15291678 | 14/06/2022 | Africa / Morocco / Casablanca | Human | unknown | Female | 57 | unknown | unknown | unknown | BA.5.2 | GRA |
| hCoV-19/Morocco/IPM20420246/2022 | EPI_ISL_15291675 | 14/06/2022 | Africa / Morocco / Casablanca | Human | unknown | Male | 56 | unknown | unknown | unknown | BA.2.9.3 | GRA |
| hCoV-19/Morocco/IPM20420238/2022 | EPI_ISL_15291674 | 14/06/2022 | Africa / Morocco / Casablanca | Human | unknown | Male | 63 | unknown | unknown | unknown | BA.2.9.3 | GRA |
| hCoV-19/Morocco/IPM20420251/2022 | EPI_ISL_15291676 | 14/06/2022 | Africa / Morocco / Casablanca | Human | unknown | Female | 71 | unknown | unknown | unknown | BA.5.2.20 | GRA |
| hCoV-19/Morocco/IPM20420176/2022 | EPI_ISL_15291669 | 14/06/2022 | Africa / Morocco / Casablanca | Human | unknown | Female | 35 | unknown | unknown | unknown | BA.5.2 | GRA |
| hCoV-19/Morocco/IPM20420223/2022 | EPI_ISL_15291673 | 14/06/2022 | Africa / Morocco / Casablanca | Human | unknown | Male | 31 | unknown | unknown | unknown | BA.5 | GRA |
| hCoV-19/Morocco/IPM20420195/2022 | EPI_ISL_15291672 | 14/06/2022 | Africa / Morocco / Casablanca | Human | unknown | Male | 75 | unknown | unknown | unknown | BA.5.2 | GRA |
| hCoV-19/Morocco/IPM20420188/2022 | EPI_ISL_15291671 | 14/06/2022 | Africa / Morocco / Casablanca | Human | unknown | Male | 2 | unknown | unknown | unknown | BA.5.2 | GRA |
| hCoV-19/Morocco/IPM20420183/2022 | EPI_ISL_15291670 | 14/06/2022 | Africa / Morocco / Casablanca | Human | unknown | Male | 26 | unknown | unknown | unknown | BA.5.2.1 | GRA |
| hCoV-19/Morocco/IPM20420171/2022 | EPI_ISL_15291668 | 14/06/2022 | Africa / Morocco / Casablanca | Human | unknown | Female | 54 | unknown | unknown | unknown | BA.5.2 | GRA |
| hCoV-19/Morocco/IPM20420135/2022 | EPI_ISL_15291664 | 13/06/2022 | Africa / Morocco / Casablanca | Human | unknown | Female | 30 | unknown | unknown | unknown | BA.5.2.20 | GRA |
| hCoV-19/Morocco/IPM20420167/2022 | EPI_ISL_15291667 | 14/06/2022 | Africa / Morocco / Casablanca | Human | unknown | Female | 49 | unknown | unknown | unknown | BA.2.40.1 | GRA |
| hCoV-19/Morocco/IPM20420152/2022 | EPI_ISL_15291666 | 13/06/2022 | Africa / Morocco / Casablanca | Human | unknown | Female | 31 | unknown | unknown | unknown | BA.5.2.20 | GRA |
| hCoV-19/Morocco/IPM20420144/2022 | EPI_ISL_15291665 | 13/06/2022 | Africa / Morocco / Casablanca | Human | unknown | Male | 45 | unknown | unknown | unknown | BA.5.2.20 | GRA |
| hCoV-19/Morocco/IPM20420094/2022 | EPI_ISL_15291663 | 13/06/2022 | Africa / Morocco / Casablanca | Human | unknown | Female | 70 | unknown | unknown | unknown | BA.2.3 | GRA |
| hCoV-19/Morocco/IPM20420073/2022 | EPI_ISL_15291660 | 13/06/2022 | Africa / Morocco / Casablanca | Human | unknown | Male | 62 | unknown | unknown | unknown | BA.2.12.1 | GRA |
| hCoV-19/Morocco/IPM20420083/2022 | EPI_ISL_15291662 | 13/06/2022 | Africa / Morocco / Casablanca | Human | unknown | Female | 66 | unknown | unknown | unknown | BA.2.36 | GRA |
| hCoV-19/Morocco/IPM20420078/2022 | EPI_ISL_15291661 | 13/06/2022 | Africa / Morocco / Casablanca | Human | unknown | Female | 38 | unknown | unknown | unknown | BA.5.2 | GRA |
| hCoV-19/Morocco/IPM20420070/2022 | EPI_ISL_15291659 | 13/06/2022 | Africa / Morocco / Casablanca | Human | unknown | Male | 45 | unknown | unknown | unknown | BA.5.2.20 | GRA |
| hCoV-19/Morocco/IPM20420060/2022 | EPI_ISL_15291658 | 13/06/2022 | Africa / Morocco / Casablanca | Human | unknown | Female | 21 | unknown | unknown | unknown | BA.2 | GRA |
| hCoV-19/Morocco/IPM20420000/2022 | EPI_ISL_15291655 | 11/06/2022 | Africa / Morocco / Casablanca | Human | unknown | Female | 80 | unknown | unknown | unknown | BA.2.9.3 | GRA |
| hCoV-19/Morocco/IPM20420049/2022 | EPI_ISL_15291657 | 13/06/2022 | Africa / Morocco / Casablanca | Human | unknown | Female | 25 | unknown | unknown | unknown | BA.5.2.20 | GRA |
| hCoV-19/Morocco/IPM20420038/2022 | EPI_ISL_15291656 | 13/06/2022 | Africa / Morocco / Casablanca | Human | unknown | Male | 37 | unknown | unknown | unknown | BA.5.2.20 | GRA |
| hCoV-19/Morocco/IPM20419982/2022 | EPI_ISL_15291654 | 10/06/2022 | Africa / Morocco / Casablanca | Human | unknown | Female | 41 | unknown | unknown | unknown | BA.2.9.3 | GRA |
| hCoV-19/Morocco/IPM20419960/2022 | EPI_ISL_15291653 | 10/06/2022 | Africa / Morocco / Casablanca | Human | unknown | Male | 33 | unknown | unknown | unknown | BA.5.2.20 | GRA |
| hCoV-19/Morocco/IPM20419930/2022 | EPI_ISL_15291649 | 10/06/2022 | Africa / Morocco / Casablanca | Human | unknown | Female | 57 | unknown | unknown | unknown | BA.2 | GRA |
| hCoV-19/Morocco/IPM20419927/2022 | EPI_ISL_15291648 | 10/06/2022 | Africa / Morocco / Casablanca | Human | unknown | Male | 54 | unknown | unknown | unknown | BA.5 | GRA |
| hCoV-19/Morocco/IPM20419904/2022 | EPI_ISL_15291647 | 10/06/2022 | Africa / Morocco / Casablanca | Human | unknown | Female | 42 | unknown | unknown | unknown | BA.5.2.20 | GRA |
| hCoV-19/Morocco/IPM20419949/2022 | EPI_ISL_15291652 | 10/06/2022 | Africa / Morocco / Casablanca | Human | unknown | Female | 29 | unknown | unknown | unknown | BA.2 | GRA |
| hCoV-19/Morocco/IPM20419945/2022 | EPI_ISL_15291651 | 10/06/2022 | Africa / Morocco / Mohammedia | Human | unknown | Female | 30 | unknown | unknown | unknown | BA.5.2 | GRA |
| hCoV-19/Morocco/IPM20419934/2022 | EPI_ISL_15291650 | 10/06/2022 | Africa / Morocco / Casablanca | Human | unknown | Female | 61 | unknown | unknown | unknown | BA.5.2.20 | GRA |
| hCoV-19/Morocco/IPM20419879/2022 | EPI_ISL_15291646 | 09/06/2022 | Africa / Morocco / Casablanca | Human | unknown | Male | 71 | unknown | unknown | unknown | BA.5.2.1 | GRA |
| hCoV-19/Morocco/IPM20419844/2022 | EPI_ISL_15291643 | 09/06/2022 | Africa / Morocco / Casablanca | Human | unknown | Female | 57 | unknown | unknown | unknown | BA.2 | GRA |
| hCoV-19/Morocco/IPM20419863/2022 | EPI_ISL_15291645 | 09/06/2022 | Africa / Morocco / Casablanca | Human | unknown | Female | 21 | unknown | unknown | unknown | BA.2 | GRA |
| hCoV-19/Morocco/IPM20419856/2022 | EPI_ISL_15291644 | 09/06/2022 | Africa / Morocco / Casablanca | Human | unknown | Male | 23 | unknown | unknown | unknown | BA.2.12.1 | GRA |
| hCoV-19/Morocco/IPM20419833/2022 | EPI_ISL_15291642 | 09/06/2022 | Africa / Morocco / Casablanca | Human | unknown | Female | 41 | unknown | unknown | unknown | BA.5.2 | GRA |
| hCoV-19/Morocco/IPM20419813/2022 | EPI_ISL_15291641 | 09/06/2022 | Africa / Morocco / Casablanca | Human | unknown | Male | 40 | unknown | unknown | unknown | BF.5 | GRA |
| hCoV-19/Morocco/IPM20419809/2022 | EPI_ISL_15291640 | 09/06/2022 | Africa / Morocco / Casablanca | Human | unknown | Male | 63 | unknown | unknown | unknown | BA.2.14 | GRA |
| hCoV-19/Morocco/FMP-47/2020 | EPI_ISL_775223 | 2020-12 | Africa / Morocco / Temara | Human | unknown | Male | 40 | unknown | unknown | unknown | B.1.1 | GR |
| hCoV-19/Morocco/IPM20419796/2022 | EPI_ISL_15291639 | 09/06/2022 | Africa / Morocco / Casablanca | Human | unknown | Male | 27 | unknown | unknown | unknown | BA.2.9.3 | GRA |
| hCoV-19/Morocco/IPM20419788/2022 | EPI_ISL_15291638 | 09/06/2022 | Africa / Morocco / Casablanca | Human | unknown | Male | 58 | unknown | unknown | unknown | BA.2.72 | GRA |
| hCoV-19/Morocco/RMPS-20/2020 | EPI_ISL_728224 | 01/04/2020 | Africa / Morocco | Human | unknown | Male | unknown | unknown | unknown | unknown | B.1 | GH |
| hCoV-19/Morocco/FMP-72/2021 | EPI_ISL_1109623 | 11/01/2021 | Africa / Morocco | Human | unknown | Male | 22 | unknown | unknown | unknown | B.1.597 | GH |
| hCoV-19/Morocco/IPM20430982/2022 | EPI_ISL_15251990 | 16/09/2022 | Africa / Morocco / Casablanca | Human | unknown | Female | 80 | unknown | unknown | unknown | BA.5.2.3 | GRA |
| hCoV-19/Morocco/IPM20430755/2022 | EPI_ISL_15251986 | 12/09/2022 | Africa / Morocco / Casablanca | Human | unknown | Male | 29 | unknown | unknown | unknown | BA.5.6 | GRA |
| hCoV-19/Morocco/IPM20430891/2022 | EPI_ISL_15251989 | 15/09/2022 | Africa / Morocco / Casablanca | Human | unknown | Female | 56 | unknown | unknown | unknown | BA.4 | GRA |
| hCoV-19/Morocco/IPM20430888/2022 | EPI_ISL_15251988 | 15/09/2022 | Africa / Morocco / Casablanca | Human | unknown | Female | 27 | unknown | unknown | unknown | BA.5.6 | GRA |
| hCoV-19/Morocco/IPM20430817/2022 | EPI_ISL_15251987 | 13/09/2022 | Africa / Morocco / Casablanca | Human | unknown | Female | 47 | unknown | unknown | unknown | BA.5.2.1 | GRA |
| hCoV-19/Morocco/IPM20430709/2022 | EPI_ISL_15251984 | 12/09/2022 | Africa / Morocco / Casablanca | Human | unknown | Female | 47 | unknown | unknown | unknown | BA.5 | GRA |
| hCoV-19/Morocco/IPM20430328/2022 | EPI_ISL_15251983 | 02/09/2022 | Africa / Morocco / Casablanca | Human | unknown | Male | 99 | unknown | unknown | unknown | BA.5.2.20 | GRA |
| hCoV-19/Morocco/IPM20430257/2022 | EPI_ISL_15251982 | 01/09/2022 | Africa / Morocco / Casablanca | Human | unknown | Female | 44 | unknown | unknown | unknown | BA.5.2.20 | GRA |
| hCoV-19/Morocco/IPM20430710/2022 | EPI_ISL_15251985 | 12/09/2022 | Africa / Morocco / Casablanca | Human | unknown | Male | 50 | unknown | unknown | unknown | BA.5 | GRA |
| hCoV-19/Morocco/IPM20430057/2022 | EPI_ISL_15251978 | 29/08/2022 | Africa / Morocco / Casablanca | Human | unknown | Female | 45 | unknown | unknown | unknown | BA.5.1.5 | GRA |
| hCoV-19/Morocco/IPM20430185/2022 | EPI_ISL_15251981 | 31/08/2022 | Africa / Morocco / Casablanca | Human | unknown | Female | 49 | unknown | unknown | unknown | BA.5.1 | GRA |
| hCoV-19/Morocco/IPM20430182/2022 | EPI_ISL_15251980 | 31/08/2022 | Africa / Morocco / Casablanca | Human | unknown | Male | 21 | unknown | unknown | unknown | BF.5 | GRA |
| hCoV-19/Morocco/IPM20430110/2022 | EPI_ISL_15251979 | 30/08/2022 | Africa / Morocco / Casablanca | Human | unknown | Male | 57 | unknown | unknown | unknown | BA.2 | GRA |
| hCoV-19/Morocco/IPM20429999/2022 | EPI_ISL_15251977 | 29/08/2022 | Africa / Morocco / Casablanca | Human | unknown | Male | 99 | unknown | unknown | unknown | BA.5.2.20 | GRA |
| hCoV-19/Morocco/IPM20429757/2022 | EPI_ISL_15251976 | 24/08/2022 | Africa / Morocco / Casablanca | Human | unknown | Female | 43 | unknown | unknown | unknown | BA.5.1.22 | GRA |
| hCoV-19/Morocco/IPM20429703/2022 | EPI_ISL_15251973 | 23/08/2022 | Africa / Morocco / Casablanca | Human | unknown | Male | 55 | unknown | unknown | unknown | BA.5.1 | GRA |
| hCoV-19/Morocco/IPM20429756/2022 | EPI_ISL_15251975 | 24/08/2022 | Africa / Morocco / Casablanca | Human | unknown | Female | 32 | unknown | unknown | unknown | BA.2 | GRA |
| hCoV-19/Morocco/IPM20429722/2022 | EPI_ISL_15251974 | 24/08/2022 | Africa / Morocco / Casablanca | Human | unknown | Female | 57 | unknown | unknown | unknown | BA.5.2.20 | GRA |
| hCoV-19/Morocco/IPM20429660/2022 | EPI_ISL_15251972 | 23/08/2022 | Africa / Morocco / Casablanca | Human | unknown | Male | 35 | unknown | unknown | unknown | BA.5.2.20 | GRA |
| hCoV-19/Morocco/IPM20429539/2022 | EPI_ISL_15251969 | 19/08/2022 | Africa / Morocco / Casablanca | Human | unknown | Female | 16 | unknown | unknown | unknown | BA.5.1 | GRA |
| hCoV-19/Morocco/IPM20429619/2022 | EPI_ISL_15251971 | 22/08/2022 | Africa / Morocco / Casablanca | Human | unknown | Female | 52 | unknown | unknown | unknown | BA.2 | GRA |
| hCoV-19/Morocco/IPM20429606/2022 | EPI_ISL_15251970 | 22/08/2022 | Africa / Morocco / Casablanca | Human | unknown | Male | 99 | unknown | unknown | unknown | BA.5.2.20 | GRA |
| hCoV-19/Morocco/IPM20429492/2022 | EPI_ISL_15251968 | 19/08/2022 | Africa / Morocco / Casablanca | Human | unknown | Female | 52 | unknown | unknown | unknown | BF.15 | GRA |
| hCoV-19/Morocco/IPM20429145/2022 | EPI_ISL_15251963 | 13/08/2022 | Africa / Morocco / Casablanca | Human | unknown | Female | 61 | unknown | unknown | unknown | BF.10 | GRA |
| hCoV-19/Morocco/IPM20429402/2022 | EPI_ISL_15251967 | 18/08/2022 | Africa / Morocco / Casablanca | Human | unknown | Male | 34 | unknown | unknown | unknown | BA.5.2 | GRA |
| hCoV-19/Morocco/IPM20429339/2022 | EPI_ISL_15251966 | 17/08/2022 | Africa / Morocco / Casablanca | Human | unknown | Male | 99 | unknown | unknown | unknown | BA.5.2.20 | GRA |
| hCoV-19/Morocco/IPM20429271/2022 | EPI_ISL_15251965 | 16/08/2022 | Africa / Morocco / Casablanca | Human | unknown | Male | 38 | unknown | unknown | unknown | BA.5.2.20 | GRA |
| hCoV-19/Morocco/IPM20429176/2022 | EPI_ISL_15251964 | 15/08/2022 | Africa / Morocco / Casablanca | Human | unknown | Female | 40 | unknown | unknown | unknown | BA.5.2.20 | GRA |
| hCoV-19/Morocco/IPM20429015/2022 | EPI_ISL_15251960 | 11/08/2022 | Africa / Morocco / Casablanca | Human | unknown | Male | 81 | unknown | unknown | unknown | BA.5.2 | GRA |
| hCoV-19/Morocco/IPM20428908/2022 | EPI_ISL_15251959 | 10/08/2022 | Africa / Morocco / Casablanca | Human | unknown | Female | 28 | unknown | unknown | unknown | BA.5.2 | GRA |
| hCoV-19/Morocco/IPM20428897/2022 | EPI_ISL_15251958 | 10/08/2022 | Africa / Morocco / Mohammedia | Human | unknown | Female | 33 | unknown | unknown | unknown | BA.5.2 | GRA |
| hCoV-19/Morocco/IPM20428858/2022 | EPI_ISL_15251957 | 09/08/2022 | Africa / Morocco / Casablanca | Human | unknown | Female | 23 | unknown | unknown | unknown | BA.5.2.20 | GRA |
| hCoV-19/Morocco/IPM20428845/2022 | EPI_ISL_15251956 | 09/08/2022 | Africa / Morocco / Casablanca | Human | unknown | Female | 73 | unknown | unknown | unknown | BA.5.2.20 | GRA |
| hCoV-19/Morocco/IPM20428787/2022 | EPI_ISL_15251955 | 08/08/2022 | Africa / Morocco / Casablanca | Human | unknown | Female | 80 | unknown | unknown | unknown | BA.5.1 | GRA |
| hCoV-19/Morocco/IPM20429123/2022 | EPI_ISL_15251962 | 13/08/2022 | Africa / Morocco / Casablanca | Human | unknown | Male | 99 | unknown | unknown | unknown | BA.5.2.20 | GRA |
| hCoV-19/Morocco/IPM20429050/2022 | EPI_ISL_15251961 | 12/08/2022 | Africa / Morocco / Casablanca | Human | unknown | Male | 30 | unknown | unknown | unknown | BF.10 | GRA |
| hCoV-19/Morocco/IPM20428539/2022 | EPI_ISL_15251951 | 03/08/2022 | Africa / Morocco / Casablanca | Human | unknown | Male | 26 | unknown | unknown | unknown | BA.5.2.1 | GRA |
| hCoV-19/Morocco/IPM20428681/2022 | EPI_ISL_15251954 | 05/08/2022 | Africa / Morocco / Casablanca | Human | unknown | Female | 58 | unknown | unknown | unknown | BA.5.2 | GRA |
| hCoV-19/Morocco/IPM20428655/2022 | EPI_ISL_15251953 | 05/08/2022 | Africa / Morocco / Casablanca | Human | unknown | Male | 99 | unknown | unknown | unknown | BA.5.2.20 | GRA |
| hCoV-19/Morocco/IPM20428598/2022 | EPI_ISL_15251952 | 04/08/2022 | Africa / Morocco / Casablanca | Human | unknown | Female | 64 | unknown | unknown | unknown | BA.5.2.20 | GRA |
| hCoV-19/Morocco/IPM20428510/2022 | EPI_ISL_15251950 | 03/08/2022 | Africa / Morocco / Casablanca | Human | unknown | Male | 40 | unknown | unknown | unknown | BA.5.2.20 | GRA |
| hCoV-19/Morocco/IPM20428439/2022 | EPI_ISL_15251946 | 02/08/2022 | Africa / Morocco / Casablanca | Human | unknown | Female | 29 | unknown | unknown | unknown | BF.5 | GRA |
| hCoV-19/Morocco/IPM20428489/2022 | EPI_ISL_15251949 | 02/08/2022 | Africa / Morocco / Casablanca | Human | unknown | Female | 51 | unknown | unknown | unknown | BA.5.2.20 | GRA |
| hCoV-19/Morocco/IPM20428477/2022 | EPI_ISL_15251948 | 02/08/2022 | Africa / Morocco / Casablanca | Human | unknown | Male | 26 | unknown | unknown | unknown | BA.5.2.20 | GRA |
| hCoV-19/Morocco/IPM20428448/2022 | EPI_ISL_15251947 | 02/08/2022 | Africa / Morocco / Casablanca | Human | unknown | Male | 64 | unknown | unknown | unknown | BA.2 | GRA |
| hCoV-19/Morocco/IPM20428278/2022 | EPI_ISL_15251945 | 02/08/2022 | Africa / Morocco / Casablanca | Human | unknown | Male | unknown | unknown | unknown | unknown | BA.2 | GRA |
| hCoV-19/Morocco/RMPS-07/2020 | EPI_ISL_482732 | 04/04/2020 | Africa / Morocco | Human | unknown | Male | unknown | unknown | unknown | unknown | B.1 | GH |
| hCoV-19/Morocco/FMP-34/2020 | EPI_ISL_768832 | 2020-12 | Africa / Morocco / Temara | Human | unknown | Male | 44 | unknown | unknown | unknown | B.1 | G |
| hCoV-19/Morocco/FMP-113/2021 | EPI_ISL_2259026 | 2021 | Africa / Morocco / Casablanca | Human | Baseline surveillance | unknown | unknown | unknown | unknown | Baseline surveillance | B.1.177 | GV |
| hCoV-19/Morocco/1034/2022 | EPI_ISL_15139501 | 22/06/2022 | Africa / Morocco / Tetouan | Human | unknown | Male | unknow | Live | unknown | unknown | BE.1 | GRA |
| hCoV-19/Morocco/ouar231/2020 | EPI_ISL_978535 | 05/05/2020 | Africa / Morocco / Ouarzazate | Human | unknown | Male | 35 | unknown | unknown | unknown | B.1.528 | G |
| hCoV-19/Morocco/1029/2022 | EPI_ISL_15139497 | 27/06/2022 | Africa / Morocco / Chefchaoun | Human | unknown | Male | unknow | Live | unknown | unknown | BA.5.2 | GRA |
| hCoV-19/Morocco/1030/2022 | EPI_ISL_15139498 | 27/06/2022 | Africa / Morocco / Tetouan | Human | unknown | Male | unknow | Live | unknown | unknown | BA.2 | GRA |
| hCoV-19/Morocco/1028/2022 | EPI_ISL_15139496 | 27/06/2022 | Africa / Morocco / Tetouan | Human | unknown | Male | unknow | Live | unknown | unknown | BA.2 | GRA |
| hCoV-19/Morocco/964/2022 | EPI_ISL_15111836 | 07/06/2022 | Africa / Morocco / Rabat | Human | unknown | Male | unknown | Live | unknown | unknown | BA.5.2.20 | GRA |
| hCoV-19/Morocco/969/2022 | EPI_ISL_15139477 | 13/06/2022 | Africa / Morocco / Rabat | Human | unknown | Female | 35 years | Live | unknown | unknown | BA.5.2.20 | GRA |
| hCoV-19/Morocco/946/2022 | EPI_ISL_15111831 | 03/06/2022 | Africa / Morocco / Rabat | Human | unknown | Female | 43 years | Live | unknown | unknown | BA.5.2 | GRA |
| hCoV-19/Morocco/954/2022 | EPI_ISL_15111833 | 07/06/2022 | Africa / Morocco / Casablanca | Human | unknown | Male | unknown | Live | unknown | unknown | BA.1 | GRA |
| hCoV-19/Morocco/940/2022 | EPI_ISL_15111827 | 01/06/2022 | Africa / Morocco / Casablanca | Human | unknown | Male | 48 years | Live | unknown | unknown | BA.5.1 | GRA |
| hCoV-19/Morocco/942/2022 | EPI_ISL_15111829 | 01/06/2022 | Africa / Morocco / Essaouira | Human | unknown | Male | unknown | Live | unknown | unknown | BA.2.12.1 | GRA |
| hCoV-19/Morocco/925/2022 | EPI_ISL_15111821 | 30/05/2022 | Africa / Morocco / Casablanca | Human | unknown | Male | 50 years | Live | unknown | unknown | BA.2.40.1 | GRA |
| hCoV-19/Morocco/932/2022 | EPI_ISL_15111824 | 31/05/2022 | Africa / Morocco / Casablanca | Human | unknown | Female | 43 years | Live | unknown | unknown | BA.2 | GRA |
| hCoV-19/Morocco/918/2022 | EPI_ISL_15111816 | 30/05/2022 | Africa / Morocco / Casablanca | Human | unknown | Male | 49 years | Live | unknown | unknown | BA.5.1 | GRA |
| hCoV-19/Morocco/922/2022 | EPI_ISL_15111819 | 30/05/2022 | Africa / Morocco / Casablanca | Human | unknown | Female | 32 years | Live | unknown | unknown | BA.2 | GRA |
| hCoV-19/Morocco/912/2022 | EPI_ISL_15111812 | 06/06/2022 | Africa / Morocco / Kenitra | Human | unknown | Male | unknown | Live | unknown | unknown | BA.5.2.20 | GRA |
| hCoV-19/Morocco/917/2022 | EPI_ISL_15111815 | 03/06/2022 | Africa / Morocco / Casablanca | Human | unknown | Male | 39 years | Live | unknown | unknown | BA.5.2 | GRA |
| hCoV-19/Morocco/915/2022 | EPI_ISL_15111814 | 03/06/2022 | Africa / Morocco / Rabat | Human | unknown | Male | 57 years | Live | unknown | unknown | BA.2 | GRA |
| hCoV-19/Morocco/Marrak-1/2020 | EPI_ISL_728240 | 2020 | Africa / Morocco | Human | unknown | unknown | unknown | unknown | unknown | unknown | B.1.1 | GR |
| hCoV-19/Morocco/ouar384bis/2020 | EPI_ISL_978544 | 05/05/2020 | Africa / Morocco / Ouarzazate | Human | unknown | Male | 32 | unknown | unknown | unknown | B.1.528 | G |
| hCoV-19/Morocco/FMP-92/2021 | EPI_ISL_1810944 | 02/01/2021 | Africa / Morocco / Rabat | Human | unknown | Male | 57 | unknown | unknown | unknown | B.1.416 | G |
| hCoV-19/Morocco/580/2022 | EPI_ISL_15111439 | 18/01/2022 | Africa / Morocco / Beni Mellal | Human | unknown | Female | 30 | Intensive care | unknown | unknown | BA.1 | GRA |
| hCoV-19/Morocco/ouar385/2020 | EPI_ISL_978545 | 05/05/2020 | Africa / Morocco / Ouarzazate | Human | unknown | Male | 55 | unknown | unknown | unknown | B.1.528 | G |
| hCoV-19/Morocco/FMP-23/2020 | EPI_ISL_728347 | 2020-12 | Africa / Morocco / Temara | Human | unknown | Female | 26 | unknown | unknown | unknown | B.1 | G |
| hCoV-19/Morocco/RMPS-03/2020 | EPI_ISL_469051 | 03/04/2020 | Africa / Morocco | Human | unknown | unknown | unknown | unknown | unknown | unknown | B.1 | G |
| hCoV-19/Morocco/1116/2022 | EPI_ISL_14980807 | 03/07/2022 | Africa / Morocco / Casablanca | Human | unknown | Male | 79 | unknown | unknown | unknown | BA.5.2.20 | GRA |
| hCoV-19/Morocco/ouar475/2020 | EPI_ISL_978549 | 05/05/2020 | Africa / Morocco / Ouarzazate | Human | unknown | Male | 44 | unknown | unknown | unknown | B.1.528 | G |
| hCoV-19/Morocco/1104/2022 | EPI_ISL_14980799 | 12/08/2022 | Africa / Morocco / Casablanca | Human | unknown | Female | 54 | unknown | unknown | unknown | BA.5.2.1 | GRA |
| hCoV-19/Morocco/1107/2022 | EPI_ISL_14980800 | 10/08/2022 | Africa / Morocco / Casablanca | Human | unknown | Male | 5 | unknown | unknown | unknown | BA.5.2.1 | GRA |
| hCoV-19/Morocco/HMIMV-00318/2020 | EPI_ISL_2968055 | 07/12/2020 | Africa / Morocco / Rabat | Human | unknown | Male | 77 | Released | unknown | unknown | B.1 | G |
| hCoV-19/Morocco/HMIMV-00317/2020 | EPI_ISL_2968057 | 07/12/2020 | Africa / Morocco / Rabat | Human | unknown | Female | 63 | Released | unknown | unknown | B.1 | G |
| hCoV-19/Morocco/IPM20428396/2022 | EPI_ISL_14846139 | 01/08/2022 | Africa / Morocco / Casablanca | Human | unknown | Female | 30 | unknown | unknown | unknown | BF.5 | GRA |
| hCoV-19/Morocco/HMIMV-17E/2020 | EPI_ISL_2968053 | 18/07/2020 | Africa / Morocco / Rabat | Human | unknown | Male | 54 | Released | unknown | unknown | B.1 | G |
| hCoV-19/Morocco/IPM20428341/2022 | EPI_ISL_14846138 | 01/08/2022 | Africa / Morocco / Casablanca | Human | unknown | Female | 53 | unknown | unknown | unknown | BA.5.2.1 | GRA |
| hCoV-19/Morocco/IPM20428159/2022 | EPI_ISL_14846135 | 28/07/2022 | Africa / Morocco / Casablanca | Human | unknown | Female | 83 | unknown | unknown | unknown | BA.5.2.20 | GRA |
| hCoV-19/Morocco/IPM20428315/2022 | EPI_ISL_14846137 | 01/08/2022 | Africa / Morocco / Casablanca | Human | unknown | Male | 54 | unknown | unknown | unknown | BA.5.2.20 | GRA |
| hCoV-19/Morocco/IPM20428192/2022 | EPI_ISL_14846136 | 29/07/2022 | Africa / Morocco / Casablanca | Human | unknown | Female | 36 | unknown | unknown | unknown | BA.5.2 | GRA |
| hCoV-19/Morocco/IPM20428107/2022 | EPI_ISL_14846134 | 28/07/2022 | Africa / Morocco / Casablanca | Human | unknown | Female | 27 | unknown | unknown | unknown | BA.5.2 | GRA |
| hCoV-19/Morocco/IPM20428032/2022 | EPI_ISL_14846133 | 27/07/2022 | Africa / Morocco / Casablanca | Human | unknown | Female | 21 | unknown | unknown | unknown | BE.2 | GRA |
| hCoV-19/Morocco/IPM20427846/2022 | EPI_ISL_14846128 | 26/07/2022 | Africa / Morocco / Casablanca | Human | unknown | Male | 73 | unknown | unknown | unknown | BA.5.2.20 | GRA |
| hCoV-19/Morocco/IPM20427963/2022 | EPI_ISL_14846132 | 27/07/2022 | Africa / Morocco / Casablanca | Human | unknown | Female | 19 | unknown | unknown | unknown | BA.5.2.20 | GRA |
| hCoV-19/Morocco/IPM20427961/2022 | EPI_ISL_14846131 | 27/07/2022 | Africa / Morocco / Casablanca | Human | unknown | Female | 47 | unknown | unknown | unknown | BF.5 | GRA |
| hCoV-19/Morocco/IPM20427902/2022 | EPI_ISL_14846130 | 26/07/2022 | Africa / Morocco / Casablanca | Human | unknown | Male | 31 | unknown | unknown | unknown | BA.5.2 | GRA |
| hCoV-19/Morocco/IPM20427860/2022 | EPI_ISL_14846129 | 26/07/2022 | Africa / Morocco / Casablanca | Human | unknown | Female | 35 | unknown | unknown | unknown | BA.5.2 | GRA |
| hCoV-19/Morocco/IPM20427756/2022 | EPI_ISL_14846126 | 25/07/2022 | Africa / Morocco / Casablanca | Human | unknown | Male | 31 | unknown | unknown | unknown | BA.5.2.1 | GRA |
| hCoV-19/Morocco/IPM20427740/2022 | EPI_ISL_14846125 | 25/07/2022 | Africa / Morocco / Casablanca | Human | unknown | Female | 60 | unknown | unknown | unknown | BA.5.1 | GRA |
| hCoV-19/Morocco/IPM20427660/2022 | EPI_ISL_14846124 | 22/07/2022 | Africa / Morocco / Casablanca | Human | unknown | Female | 63 | unknown | unknown | unknown | BA.5.2.20 | GRA |
| hCoV-19/Morocco/IPM20427657/2022 | EPI_ISL_14846123 | 22/07/2022 | Africa / Morocco / Casablanca | Human | unknown | Female | 59 | unknown | unknown | unknown | BA.5.2.20 | GRA |
| hCoV-19/Morocco/IPM20427623/2022 | EPI_ISL_14846122 | 22/07/2022 | Africa / Morocco / Casablanca | Human | unknown | Male | 47 | unknown | unknown | unknown | BA.5.2.20 | GRA |
| hCoV-19/Morocco/IPM20427575/2022 | EPI_ISL_14846121 | 21/07/2022 | Africa / Morocco / Casablanca | Human | unknown | Male | 41 | unknown | unknown | unknown | BA.5.2.20 | GRA |
| hCoV-19/Morocco/IPM20427553/2022 | EPI_ISL_14846120 | 21/07/2022 | Africa / Morocco / Casablanca | Human | unknown | Male | 58 | unknown | unknown | unknown | BA.5.2.20 | GRA |
| hCoV-19/Morocco/IPM20427544/2022 | EPI_ISL_14846119 | 21/07/2022 | Africa / Morocco / Casablanca | Human | unknown | Male | 44 | unknown | unknown | unknown | BE.1.1 | GRA |
| hCoV-19/Morocco/IPM20427414/2022 | EPI_ISL_14846118 | 20/07/2022 | Africa / Morocco / Casablanca | Human | unknown | Female | 37 | unknown | unknown | unknown | BA.5.2 | GRA |
| hCoV-19/Morocco/IPM20427408/2022 | EPI_ISL_14846117 | 20/07/2022 | Africa / Morocco / Casablanca | Human | unknown | Female | 22 | unknown | unknown | unknown | BA.5.2.20 | GRA |
| hCoV-19/Morocco/IPM20427401/2022 | EPI_ISL_14846116 | 20/07/2022 | Africa / Morocco / Casablanca | Human | unknown | Male | 47 | unknown | unknown | unknown | BA.5.2.20 | GRA |
| hCoV-19/Morocco/IPM20427346/2022 | EPI_ISL_14846115 | 19/07/2022 | Africa / Morocco / Casablanca | Human | unknown | Male | 47 | unknown | unknown | unknown | BA.5.2.20 | GRA |
| hCoV-19/Morocco/IPM20427322/2022 | EPI_ISL_14846114 | 19/07/2022 | Africa / Morocco / Casablanca | Human | unknown | Male | 29 | unknown | unknown | unknown | BA.5.2.20 | GRA |
| hCoV-19/Morocco/IPM20427284/2022 | EPI_ISL_14846113 | 19/07/2022 | Africa / Morocco / Casablanca | Human | unknown | Female | 43 | unknown | unknown | unknown | BA.2 | GRA |
| hCoV-19/Morocco/IPM20427158/2022 | EPI_ISL_14846112 | 18/07/2022 | Africa / Morocco / Casablanca | Human | unknown | Female | 52 | unknown | unknown | unknown | BA.5.1 | GRA |
| hCoV-19/Morocco/IPM20427090/2022 | EPI_ISL_14846111 | 18/07/2022 | Africa / Morocco / Casablanca | Human | unknown | Female | 62 | unknown | unknown | unknown | BA.5.2 | GRA |
| hCoV-19/Morocco/IPM20427066/2022 | EPI_ISL_14846110 | 18/07/2022 | Africa / Morocco / Casablanca | Human | unknown | Female | 47 | unknown | unknown | unknown | BA.5.2.20 | GRA |
| hCoV-19/Morocco/IPM20427060/2022 | EPI_ISL_14846109 | 18/07/2022 | Africa / Morocco / Casablanca | Human | unknown | Female | 25 | unknown | unknown | unknown | BF.5 | GRA |
| hCoV-19/Morocco/IPM20427807/2022 | EPI_ISL_14846127 | 25/07/2022 | Africa / Morocco / Casablanca | Human | unknown | Male | 57 | unknown | unknown | unknown | BA.5.2.20 | GRA |
| hCoV-19/Morocco/IPM20426891/2022 | EPI_ISL_14846106 | 15/07/2022 | Africa / Morocco / Casablanca | Human | unknown | Male | 22 | unknown | unknown | unknown | BA.5.2.20 | GRA |
| hCoV-19/Morocco/IPM20426999/2022 | EPI_ISL_14846108 | 16/07/2022 | Africa / Morocco / Casablanca | Human | unknown | Male | 38 | unknown | unknown | unknown | BA.5.2.20 | GRA |
| hCoV-19/Morocco/IPM20426924/2022 | EPI_ISL_14846107 | 15/07/2022 | Africa / Morocco / Casablanca | Human | unknown | Female | 28 | unknown | unknown | unknown | BA.5.2.20 | GRA |
| hCoV-19/Morocco/IPM20426859/2022 | EPI_ISL_14846105 | 15/07/2022 | Africa / Morocco / Casablanca | Human | unknown | Male | 39 | unknown | unknown | unknown | BA.5.2.20 | GRA |
| hCoV-19/Morocco/IPM20426776/2022 | EPI_ISL_14846102 | 14/07/2022 | Africa / Morocco / Casablanca | Human | unknown | Male | 41 | unknown | unknown | unknown | BA.2 | GRA |
| hCoV-19/Morocco/IPM20426786/2022 | EPI_ISL_14846104 | 14/07/2022 | Africa / Morocco / Casablanca | Human | unknown | Female | 57 | unknown | unknown | unknown | BA.5.2.20 | GRA |
| hCoV-19/Morocco/IPM20426780/2022 | EPI_ISL_14846103 | 14/07/2022 | Africa / Morocco / Casablanca | Human | unknown | Male | 63 | unknown | unknown | unknown | BA.5.1 | GRA |
| hCoV-19/Morocco/IPM20426643/2022 | EPI_ISL_14846100 | 13/07/2022 | Africa / Morocco / Casablanca | Human | unknown | Female | 62 | unknown | unknown | unknown | BA.5.2.20 | GRA |
| hCoV-19/Morocco/IPM20426639/2022 | EPI_ISL_14846099 | 13/07/2022 | Africa / Morocco / Casablanca | Human | unknown | Female | 32 | unknown | unknown | unknown | BA.5.2.20 | GRA |
| hCoV-19/Morocco/IPM20426686/2022 | EPI_ISL_14846101 | 14/07/2022 | Africa / Morocco / Casablanca | Human | unknown | Male | 56 | unknown | unknown | unknown | BA.5.2.1 | GRA |
| hCoV-19/Morocco/IPM20426548/2022 | EPI_ISL_14846094 | 13/07/2022 | Africa / Morocco / Casablanca | Human | unknown | Female | 28 | unknown | unknown | unknown | BA.2.9 | GRA |
| hCoV-19/Morocco/IPM20426621/2022 | EPI_ISL_14846098 | 13/07/2022 | Africa / Morocco / Casablanca | Human | unknown | Female | 44 | unknown | unknown | unknown | BA.5.1 | GRA |
| hCoV-19/Morocco/IPM20426598/2022 | EPI_ISL_14846097 | 13/07/2022 | Africa / Morocco / Casablanca | Human | unknown | Male | 38 | unknown | unknown | unknown | BA.5.2.20 | GRA |
| hCoV-19/Morocco/IPM20426559/2022 | EPI_ISL_14846096 | 13/07/2022 | Africa / Morocco / Casablanca | Human | unknown | Male | 44 | unknown | unknown | unknown | BF.5 | GRA |
| hCoV-19/Morocco/IPM20426554/2022 | EPI_ISL_14846095 | 13/07/2022 | Africa / Morocco / Casablanca | Human | unknown | Female | 48 | unknown | unknown | unknown | BA.5.2 | GRA |
| hCoV-19/Morocco/IPM20426512/2022 | EPI_ISL_14846093 | 13/07/2022 | Africa / Morocco / Casablanca | Human | unknown | Female | 40 | unknown | unknown | unknown | BA.5.2.20 | GRA |
| hCoV-19/Morocco/HMIMV-00299/2020 | EPI_ISL_2968064 | 07/12/2020 | Africa / Morocco / Rabat | Human | unknown | Male | 40 | Released | unknown | unknown | B.1.1 | GR |
| hCoV-19/Morocco/IPM20416872/2022 | EPI_ISL_12590791 | 08/04/2022 | Africa / Morocco / Casablanca | Human | unknown | Female | 82 | unknown | unknown | unknown | BA.2.3 | GRA |
| hCoV-19/Morocco/HMIMV-00300/2020 | EPI_ISL_2968063 | 07/12/2020 | Africa / Morocco / Rabat | Human | unknown | Female | 64 | Released | unknown | unknown | B.1 | G |
| hCoV-19/Morocco/IPM20416758/2022 | EPI_ISL_12590788 | 05/04/2022 | Africa / Morocco / Casablanca | Human | unknown | Male | 75 | unknown | unknown | unknown | BA.2 | GRA |
| hCoV-19/Morocco/IPM20416752/2022 | EPI_ISL_12590787 | 05/04/2022 | Africa / Morocco / Casablanca | Human | unknown | Female | 31 | unknown | unknown | unknown | BA.2 | GRA |
| hCoV-19/Morocco/IPM20416747/2022 | EPI_ISL_12590786 | 05/04/2022 | Africa / Morocco / Casablanca | Human | unknown | Male | 62 | unknown | unknown | unknown | BA.2.3 | GRA |
| hCoV-19/Morocco/IPM20416365/2022 | EPI_ISL_12590785 | 30/03/2022 | Africa / Morocco / Casablanca | Human | unknown | Female | 47 | unknown | unknown | unknown | BA.2 | GRA |
| hCoV-19/Morocco/IPM20416301/2022 | EPI_ISL_12590784 | 29/03/2022 | Africa / Morocco / Casablanca | Human | unknown | unknown | unknown | unknown | unknown | unknown | BA.2 | GRA |
| hCoV-19/Morocco/IPM20416251/2022 | EPI_ISL_12590783 | 28/03/2022 | Africa / Morocco / Casablanca | Human | unknown | Female | 23 | unknown | unknown | unknown | BA.2 | GRA |
| hCoV-19/Morocco/IPM20416177/2022 | EPI_ISL_12590782 | 26/03/2022 | Africa / Morocco / Casablanca | Human | unknown | Female | 31 | unknown | unknown | unknown | BA.2 | GRA |
| hCoV-19/Morocco/IPM20416128/2022 | EPI_ISL_12590781 | 25/03/2022 | Africa / Morocco / Casablanca | Human | unknown | Male | 38 | unknown | unknown | unknown | BA.2 | GRA |
| hCoV-19/Morocco/IPM20416015/2022 | EPI_ISL_12590780 | 24/03/2022 | Africa / Morocco / Casablanca | Human | unknown | Female | 47 | unknown | unknown | unknown | BA.2 | GRA |
| hCoV-19/Morocco/IPM20415890/2022 | EPI_ISL_12590779 | 23/03/2022 | Africa / Morocco / Casablanca | Human | unknown | Male | 54 | unknown | unknown | unknown | BA.2 | GRA |
| hCoV-19/Morocco/IPM20414938/2022 | EPI_ISL_12590778 | 10/03/2022 | Africa / Morocco / Casablanca | Human | unknown | Male | 49 | unknown | unknown | unknown | BA.1 | GRA |
| hCoV-19/Morocco/IPM20414862/2022 | EPI_ISL_12590777 | 09/03/2022 | Africa / Morocco / Casablanca | Human | unknown | Male | 35 | unknown | unknown | unknown | B.1.617.2 | GK |
| hCoV-19/Morocco/IPM20416797/2022 | EPI_ISL_12590790 | 06/04/2022 | Africa / Morocco / Casablanca | Human | unknown | Female | 47 | unknown | unknown | unknown | BA.2 | GRA |
| hCoV-19/Morocco/IPM20416789/2022 | EPI_ISL_12590789 | 06/04/2022 | Africa / Morocco / Casablanca | Human | unknown | Male | 17 | unknown | unknown | unknown | BA.2 | GRA |
| hCoV-19/Morocco/IPM20414745/2022 | EPI_ISL_12590776 | 08/03/2022 | Africa / Morocco / Casablanca | Human | unknown | Male | 28 | unknown | unknown | unknown | BA.1 | GRA |
| hCoV-19/Morocco/IPM20414421/2022 | EPI_ISL_12590775 | 03/03/2022 | Africa / Morocco / Casablanca | Human | unknown | Female | 58 | unknown | unknown | unknown | BA.1 | GRA |
| hCoV-19/Morocco/IPM20414163/2022 | EPI_ISL_12590774 | 01/03/2022 | Africa / Morocco / Casablanca | Human | unknown | Male | 25 | unknown | unknown | unknown | BA.2 | GRA |
| hCoV-19/Morocco/IPM20414088/2022 | EPI_ISL_12590773 | 28/02/2022 | Africa / Morocco / Mohammedia | Human | unknown | Female | 52 | unknown | unknown | unknown | BA.1 | GRA |
| hCoV-19/Morocco/IPM20412629/2022 | EPI_ISL_12590768 | 17/02/2022 | Africa / Morocco / Casablanca | Human | unknown | unknown | unknown | unknown | unknown | unknown | BA.1 | GRA |
| hCoV-19/Morocco/IPM20414011/2022 | EPI_ISL_12590772 | 28/02/2022 | Africa / Morocco / Casablanca | Human | unknown | Male | 52 | unknown | unknown | unknown | BA.1 | GRA |
| hCoV-19/Morocco/IPM20413470/2022 | EPI_ISL_12590771 | 23/02/2022 | Africa / Morocco / Mohammedia | Human | unknown | Male | 26 | unknown | unknown | unknown | BA.2 | GRA |
| hCoV-19/Morocco/IPM20413132/2022 | EPI_ISL_12590770 | 21/02/2022 | Africa / Morocco / Mohammedia | Human | unknown | Female | 61 | unknown | unknown | unknown | BA.1 | GRA |
| hCoV-19/Morocco/IPM20412896/2022 | EPI_ISL_12590769 | 19/02/2022 | Africa / Morocco / Casablanca | Human | unknown | Female | 87 | unknown | unknown | unknown | BA.1 | GRA |
| hCoV-19/Morocco/IPM20412350/2022 | EPI_ISL_12590767 | 16/02/2022 | Africa / Morocco / Mohammedia | Human | unknown | Male | 70 | unknown | unknown | unknown | BA.1 | GRA |
| hCoV-19/Morocco/IPM20412230/2022 | EPI_ISL_12590766 | 15/02/2022 | Africa / Morocco / Casablanca | Human | unknown | Male | 17 | unknown | unknown | unknown | BA.1 | GRA |
| hCoV-19/Morocco/IPM20412183/2022 | EPI_ISL_12590765 | 15/02/2022 | Africa / Morocco / Casablanca | Human | unknown | Male | 55 | unknown | unknown | unknown | BA.1 | GRA |
| hCoV-19/Morocco/IPM20411804/2022 | EPI_ISL_12590760 | 11/02/2022 | Africa / Morocco / Casablanca | Human | unknown | Male | 28 | unknown | unknown | unknown | BA.1 | GRA |
| hCoV-19/Morocco/IPM20412160/2022 | EPI_ISL_12590764 | 15/02/2022 | Africa / Morocco / Mohammedia | Human | unknown | Male | 44 | unknown | unknown | unknown | BA.1 | GRA |
| hCoV-19/Morocco/IPM20412096/2022 | EPI_ISL_12590763 | 14/02/2022 | Africa / Morocco / Casablanca | Human | unknown | Male | 35 | unknown | unknown | unknown | BA.1 | GRA |
| hCoV-19/Morocco/IPM20411932/2022 | EPI_ISL_12590762 | 12/02/2022 | Africa / Morocco / Casablanca | Human | unknown | Male | 61 | unknown | unknown | unknown | BA.1 | GRA |
| hCoV-19/Morocco/IPM20411836/2022 | EPI_ISL_12590761 | 11/02/2022 | Africa / Morocco / Casablanca | Human | unknown | Female | 99 | unknown | unknown | unknown | BA.1.1 | GRA |
| hCoV-19/Morocco/IPM20411287/2022 | EPI_ISL_12590756 | 09/02/2022 | Africa / Morocco / Casablanca | Human | unknown | Female | 40 | unknown | unknown | unknown | BA.1.1 | GRA |
| hCoV-19/Morocco/IPM20370941/2021 | EPI_ISL_12590755 | 25/09/2021 | Africa / Morocco / Casablanca | Human | unknown | Male | 59 | unknown | unknown | unknown | B.1.617.2 | GK |
| hCoV-19/Morocco/IPM20416871/2022 | EPI_ISL_12590754 | 08/04/2022 | Africa / Morocco / Casablanca | Human | unknown | Male | 22 | unknown | unknown | unknown | BA.2 | GRA |
| hCoV-19/Morocco/IPM20416828/2022 | EPI_ISL_12590753 | 07/04/2022 | Africa / Morocco / Casablanca | Human | unknown | Female | 50 | unknown | unknown | unknown | BA.2 | GRA |
| hCoV-19/Morocco/IPM20411758/2022 | EPI_ISL_12590759 | 11/02/2022 | Africa / Morocco / Casablanca | Human | unknown | Female | 70 | unknown | unknown | unknown | BA.1 | GRA |
| hCoV-19/Morocco/IPM20411671/2022 | EPI_ISL_12590758 | 11/02/2022 | Africa / Morocco / Casablanca | Human | unknown | Male | 49 | unknown | unknown | unknown | BA.1 | GRA |
| hCoV-19/Morocco/IPM20411310/2022 | EPI_ISL_12590757 | 09/02/2022 | Africa / Morocco / Casablanca | Human | unknown | Female | 42 | unknown | unknown | unknown | BA.1.1 | GRA |
| hCoV-19/Morocco/IPM20411641/2022 | EPI_ISL_12590748 | 10/02/2022 | Africa / Morocco / Casablanca | Human | unknown | Female | 70 | unknown | unknown | unknown | B.1.1.529 | GRA |
| hCoV-19/Morocco/IPM20416794/2022 | EPI_ISL_12590752 | 06/04/2022 | Africa / Morocco / Casablanca | Human | unknown | Female | 22 | unknown | unknown | unknown | BA.2 | GRA |
| hCoV-19/Morocco/IPM20416400/2022 | EPI_ISL_12590751 | 30/03/2022 | Africa / Morocco / Casablanca | Human | unknown | Male | 10 | unknown | unknown | unknown | BA.2 | GRA |
| hCoV-19/Morocco/IPM20416369/2022 | EPI_ISL_12590750 | 30/03/2022 | Africa / Morocco / Casablanca | Human | unknown | Male | 57 | unknown | unknown | unknown | BA.2 | GRA |
| hCoV-19/Morocco/IPM20416306/2022 | EPI_ISL_12590749 | 29/03/2022 | Africa / Morocco / Casablanca | Human | unknown | Female | 55 | unknown | unknown | unknown | BA.2 | GRA |
| hCoV-19/Morocco/IPM20411622/2022 | EPI_ISL_12590747 | 10/02/2022 | Africa / Morocco / Mohammedia | Human | unknown | Female | 47 | unknown | unknown | unknown | B.1.1.529 | GRA |
| hCoV-19/Morocco/IPM20411533/2022 | EPI_ISL_12590746 | 10/02/2022 | Africa / Morocco / Casablanca | Human | unknown | Male | 52 | unknown | unknown | unknown | B.1.1.529 | GRA |
| hCoV-19/Morocco/IPM20386211/2021 | EPI_ISL_12590745 | 09/12/2021 | Africa / Morocco / Casablanca | Human | unknown | Female | 78 | unknown | unknown | unknown | AY.33 | GK |
| hCoV-19/env/Morocco/F11/2022 | EPI_ISL_11020420 | 03/02/2022 | Africa / Morocco / Fes | unknown | unknown | unknown | unknown | unknown | unknown | Wastewater testing | BA.1.15 | GRA |
| hCoV-19/env/Morocco/F03/2021 | EPI_ISL_11020424 | 09/12/2021 | Africa / Morocco / Fes | unknown | unknown | unknown | unknown | unknown | unknown | Wastewater testing | AY.33 | GK |
| hCoV-19/Morocco/IPM20411220/2022 | EPI_ISL_10862807 | 08/02/2022 | Africa / Morocco / Casablanca | Human | unknown | Female | 60 | unknown | unknown | unknown | BA.1 | GRA |
| hCoV-19/Morocco/IPM20411219/2022 | EPI_ISL_10862806 | 08/02/2022 | Africa / Morocco / Casablanca | Human | unknown | Female | 53 | unknown | unknown | unknown | BA.1 | GRA |
| hCoV-19/Morocco/IPM20411173/2022 | EPI_ISL_10862805 | 08/02/2022 | Africa / Morocco / Casablanca | Human | unknown | Female | 29 | unknown | unknown | unknown | BA.1 | GRA |
| hCoV-19/Morocco/IPM20411134/2022 | EPI_ISL_10862804 | 08/02/2022 | Africa / Morocco / Casablanca | Human | unknown | Female | 26 | unknown | unknown | unknown | BA.1 | GRA |
| hCoV-19/Morocco/IPM20411122/2022 | EPI_ISL_10862803 | 08/02/2022 | Africa / Morocco / Casablanca | Human | unknown | Male | 40 | unknown | unknown | unknown | BA.1 | GRA |
| hCoV-19/Morocco/IPM20411110/2022 | EPI_ISL_10862802 | 08/02/2022 | Africa / Morocco / Casablanca | Human | unknown | Female | 45 | unknown | unknown | unknown | BA.1 | GRA |
| hCoV-19/Morocco/IPM20411057/2022 | EPI_ISL_10862801 | 08/02/2022 | Africa / Morocco / Casablanca | Human | unknown | Male | 61 | unknown | unknown | unknown | BA.1 | GRA |
| hCoV-19/Morocco/IPM20411050/2022 | EPI_ISL_10862800 | 08/02/2022 | Africa / Morocco / Casablanca | Human | unknown | Male | 25 | unknown | unknown | unknown | BA.1 | GRA |
| hCoV-19/Morocco/IPM20410785/2022 | EPI_ISL_10862799 | 07/02/2022 | Africa / Morocco / Casablanca | Human | unknown | Male | 68 | unknown | unknown | unknown | BA.1 | GRA |
| hCoV-19/Morocco/IPM20410783/2022 | EPI_ISL_10862798 | 07/02/2022 | Africa / Morocco / Casablanca | Human | unknown | Female | 72 | unknown | unknown | unknown | B.1.617.2 | GK |
| hCoV-19/Morocco/IPM20410573/2022 | EPI_ISL_10862797 | 04/02/2022 | Africa / Morocco / Casablanca | Human | unknown | Female | 58 | unknown | unknown | unknown | B.1.1.529 | GRA |
| hCoV-19/Morocco/IPM20409682/2022 | EPI_ISL_10862796 | 01/02/2022 | Africa / Morocco / Casablanca | Human | unknown | Male | 83 | unknown | unknown | unknown | BA.1 | GRA |
| hCoV-19/Morocco/IPM20409681/2022 | EPI_ISL_10862795 | 01/02/2022 | Africa / Morocco / Casablanca | Human | unknown | Male | 84 | unknown | unknown | unknown | B.1.1.529 | GRA |
| hCoV-19/Morocco/IPM20409679/2022 | EPI_ISL_10862794 | 01/02/2022 | Africa / Morocco / Casablanca | Human | unknown | Male | 78 | unknown | unknown | unknown | BA.1 | GRA |
| hCoV-19/Morocco/IPM20409677/2022 | EPI_ISL_10862793 | 01/02/2022 | Africa / Morocco / Casablanca | Human | unknown | Male | 83 | unknown | unknown | unknown | B.1.1.529 | GRA |
| hCoV-19/Morocco/IPM20409676/2022 | EPI_ISL_10862792 | 01/02/2022 | Africa / Morocco / Casablanca | Human | unknown | Female | 25 | unknown | unknown | unknown | BA.1 | GRA |
| hCoV-19/Morocco/IPM20409675/2022 | EPI_ISL_10862791 | 01/02/2022 | Africa / Morocco / Casablanca | Human | unknown | Male | 86 | unknown | unknown | unknown | BA.1 | GRA |
| hCoV-19/Morocco/IPM20409325/2022 | EPI_ISL_10862786 | 31/01/2022 | Africa / Morocco / Casablanca | Human | unknown | Male | 84 | unknown | unknown | unknown | BA.1 | GRA |
| hCoV-19/Morocco/IPM20409330/2022 | EPI_ISL_10862789 | 31/01/2022 | Africa / Morocco / Casablanca | Human | unknown | Male | 77 | unknown | unknown | unknown | BA.1 | GRA |
| hCoV-19/Morocco/IPM20409328/2022 | EPI_ISL_10862788 | 31/01/2022 | Africa / Morocco / Casablanca | Human | unknown | Male | 94 | unknown | unknown | unknown | BA.1 | GRA |
| hCoV-19/Morocco/IPM20409327/2022 | EPI_ISL_10862787 | 31/01/2022 | Africa / Morocco / Casablanca | Human | unknown | Female | 88 | unknown | unknown | unknown | BA.1 | GRA |
| hCoV-19/Morocco/IPM20408866/2022 | EPI_ISL_10862785 | 28/01/2022 | Africa / Morocco / Casablanca | Human | unknown | Female | 33 | unknown | unknown | unknown | BA.1 | GRA |
| hCoV-19/Morocco/IPM20408699/2022 | EPI_ISL_10862784 | 27/01/2022 | Africa / Morocco / Casablanca | Human | unknown | Female | 74 | unknown | unknown | unknown | BA.1 | GRA |
| hCoV-19/Morocco/IPM20408695/2022 | EPI_ISL_10862783 | 27/01/2022 | Africa / Morocco / Casablanca | Human | unknown | Male | unkonwn | unknown | unknown | unknown | BA.1 | GRA |
| hCoV-19/Morocco/IPM20407612/2022 | EPI_ISL_10862778 | 25/01/2022 | Africa / Morocco / Casablanca | Human | unknown | Female | 44 | unknown | unknown | unknown | BA.1.1 | GRA |
| hCoV-19/Morocco/IPM20407611/2022 | EPI_ISL_10862777 | 25/01/2022 | Africa / Morocco / Casablanca | Human | unknown | Male | 85 | unknown | unknown | unknown | B.1.1.529 | GRA |
| hCoV-19/Morocco/IPM20407610/2022 | EPI_ISL_10862776 | 25/01/2022 | Africa / Morocco / Casablanca | Human | unknown | Female | 77 | unknown | unknown | unknown | BA.1 | GRA |
| hCoV-19/Morocco/IPM20407504/2022 | EPI_ISL_10862775 | 25/01/2022 | Africa / Morocco / Casablanca | Human | unknown | Female | 81 | unknown | unknown | unknown | BA.1 | GRA |
| hCoV-19/Morocco/IPM20408533/2022 | EPI_ISL_10862782 | 27/01/2022 | Africa / Morocco / Casablanca | Human | unknown | Female | 59 | unknown | unknown | unknown | BA.1 | GRA |
| hCoV-19/Morocco/IPM20408532/2022 | EPI_ISL_10862781 | 27/01/2022 | Africa / Morocco / Casablanca | Human | unknown | Male | 86 | unknown | unknown | unknown | BA.1 | GRA |
| hCoV-19/Morocco/IPM20408530/2022 | EPI_ISL_10862780 | 27/01/2022 | Africa / Morocco / Casablanca | Human | unknown | Male | 88 | unknown | unknown | unknown | BA.1 | GRA |
| hCoV-19/Morocco/IPM20408528/2022 | EPI_ISL_10862779 | 27/01/2022 | Africa / Morocco / Casablanca | Human | unknown | Male | 65 | unknown | unknown | unknown | BA.1 | GRA |
| hCoV-19/Morocco/IPM20407400/2022 | EPI_ISL_10862770 | 24/01/2022 | Africa / Morocco / Casablanca | Human | unknown | Male | 68 | unknown | unknown | unknown | BA.1 | GRA |
| hCoV-19/Morocco/IPM20407503/2022 | EPI_ISL_10862774 | 25/01/2022 | Africa / Morocco / Casablanca | Human | unknown | Male | 94 | unknown | unknown | unknown | BA.1 | GRA |
| hCoV-19/Morocco/IPM20407502/2022 | EPI_ISL_10862773 | 25/01/2022 | Africa / Morocco / Casablanca | Human | unknown | Female | 68 | unknown | unknown | unknown | BA.1.13 | GRA |
| hCoV-19/Morocco/IPM20407500/2022 | EPI_ISL_10862772 | 25/01/2022 | Africa / Morocco / Casablanca | Human | unknown | Male | 86 | unknown | unknown | unknown | BA.1 | GRA |
| hCoV-19/Morocco/IPM20407402/2022 | EPI_ISL_10862771 | 24/01/2022 | Africa / Morocco / Casablanca | Human | unknown | Female | 77 | unknown | unknown | unknown | BA.1 | GRA |
| hCoV-19/Morocco/IPM20407391/2022 | EPI_ISL_10862766 | 24/01/2022 | Africa / Morocco / Casablanca | Human | unknown | Male | 77 | unknown | unknown | unknown | BA.1 | GRA |
| hCoV-19/Morocco/IPM20407389/2022 | EPI_ISL_10862765 | 24/01/2022 | Africa / Morocco / Casablanca | Human | unknown | Male | 77 | unknown | unknown | unknown | BA.1 | GRA |
| hCoV-19/Morocco/IPM20406698/2022 | EPI_ISL_10862763 | 21/01/2022 | Africa / Morocco / Casablanca | Human | unknown | Female | 83 | unknown | unknown | unknown | BA.1 | GRA |
| hCoV-19/Morocco/IPM20407398/2022 | EPI_ISL_10862769 | 24/01/2022 | Africa / Morocco / Casablanca | Human | unknown | Male | 85 | unknown | unknown | unknown | BA.1 | GRA |
| hCoV-19/Morocco/IPM20407396/2022 | EPI_ISL_10862768 | 24/01/2022 | Africa / Morocco / Casablanca | Human | unknown | Male | 70 | unknown | unknown | unknown | BA.1 | GRA |
| hCoV-19/Morocco/IPM20407393/2022 | EPI_ISL_10862767 | 24/01/2022 | Africa / Morocco / Casablanca | Human | unknown | Male | 68 | unknown | unknown | unknown | BA.1 | GRA |
| hCoV-19/Morocco/IPM20406696/2022 | EPI_ISL_10862762 | 21/01/2022 | Africa / Morocco / Casablanca | Human | unknown | Male | 80 | unknown | unknown | unknown | BA.1 | GRA |
