# Supplementary material for: Genomic surveillance of SARS-CoV-2 in North Africa: 4 years of GISAID data sharing
Source: IJID Reg. 2024 Mar 19;11:100356. doi: 10.1016/j.ijregi.2024.100356 (PMC11035039; doi:10.1016/j.ijregi.2024.100356)
Supplement: Supplementary file 7 [file mmc7.docx]

Table S6. Comprehensive Virus Data from Mauritania Including Virus Name, Accession Number, and Clinical Attributes (based on data downloaded from GISAID per 15 September 2023)

| Virus name | Accession ID | Collection date | Location | Host | Sampling strategy | Gender | Patient age (years) | Patient status | Last vaccinated | Sampling strategy | Lineage | Clade |
| --- | --- | --- | --- | --- | --- | --- | --- | --- | --- | --- | --- | --- |
| hCoV-19/Mauritania/CT740/2021 | EPI_ISL_11033239 | 15/03/2021 | Africa / Mauritania / Nouackchott | Human | unknown | Female | 23 | Released | unknown | unknown | B.1.1.318 | GR |
| hCoV-19/Mauritania/N033859/2021 | EPI_ISL_11033240 | 16/03/2021 | Africa / Mauritania / Nouackchott | Human | unknown | Male | 42 | Live | unknown | unknown | AY.34.1 | GK |
| hCoV-19/Mauritania/N035803/2021 | EPI_ISL_11033241 | 22/03/2021 | Africa / Mauritania / Nouackchott | Human | unknown | Male | 37 | Live | unknown | unknown | AY.34.1 | GK |
| hCoV-19/Mauritania/N045937/2021 | EPI_ISL_11033242 | 22/04/2021 | Africa / Mauritania / Nouackchott | Human | unknown | Male | 39 | Live | unknown | unknown | B.1.617.2 | GK |
| hCoV-19/Mauritania/N043313/2021 | EPI_ISL_11033243 | 22/04/2021 | Africa / Mauritania / Nouackchott | Human | unknown | Female | 8 | Live | unknown | unknown | AY.34.1 | GK |
| hCoV-19/Mauritania/INRSP55844/2021 | EPI_ISL_11033244 | 29/05/2021 | Africa / Mauritania / Nouackchott | Human | unknown | Male | 41 | Live | unknown | unknown | B.1.620 | G |
| hCoV-19/Mauritania/INRSP56433/2021 | EPI_ISL_11033245 | 31/05/2021 | Africa / Mauritania / Nouackchott | Human | unknown | Male | 33 | Live | unknown | unknown | B.1.525 | G |
| hCoV-19/Mauritania/INRSP56576/2021 | EPI_ISL_11033246 | 31/05/2021 | Africa / Mauritania / Nouackchott | Human | unknown | Male | 7 months | Live | unknown | unknown | B.1.620 | G |
| hCoV-19/Mauritania/INRSP56580/2021 | EPI_ISL_11033247 | 31/05/2021 | Africa / Mauritania / Nouackchott | Human | unknown | Male | 46 | Live | unknown | unknown | B.1.525 | G |
| hCoV-19/Mauritania/INRSP56964/2021 | EPI_ISL_11033248 | 02/06/2021 | Africa / Mauritania / Nouackchott | Human | unknown | Male | 29 | Live | unknown | unknown | B.1.1.7 | GRY |
| hCoV-19/Mauritania/INRSP57037/2021 | EPI_ISL_11033249 | 02/06/2021 | Africa / Mauritania / Nouackchott | Human | unknown | Male | 57 | Live | unknown | unknown | B.1.525 | G |
| hCoV-19/Mauritania/CT3965/2021 | EPI_ISL_11033250 | 16/10/2021 | Africa / Mauritania / Nouackchott | Human | unknown | Female | 41 | Released | unknown | unknown | AY.34.1 | GK |
| hCoV-19/Mauritania/397353/2021 | EPI_ISL_11033251 | 23/10/2021 | Africa / Mauritania / Nouackchott | Human | unknown | Male | 48 | Hospitalized | unknown | unknown | AY.34.1 | GK |
| hCoV-19/Mauritania/CT213/2021 | EPI_ISL_11268633 | 18/02/2021 | Africa / Mauritania / Nouackchott | Human | unknown | Male | 46 | Released | unknown | unknown | B.1.1.7 | GRY |
| hCoV-19/Mauritania/N034647/2021 | EPI_ISL_11268634 | 05/09/2021 | Africa / Mauritania / Nouackchott | Human | unknown | Female | 42 | Live | unknown | unknown | AY.34.1 | GK |
| hCoV-19/Mauritanie/INRSP56062/2021 | EPI_ISL_11380682 | 29/05/2021 | Africa / Mauritania / Nouackchott | Human | unknown | Male | 62 | Live | unknown | unknown | B.1.525 | G |
| hCoV-19/Mauritanie/INRSP55933/2021 | EPI_ISL_11380683 | 29/05/2021 | Africa / Mauritania / Nouackchott | Human | unknown | Male | 11 | Live | unknown | unknown | B.1.1.318 | GR |
| hCoV-19/Mauritanie/INRSP52000/2021 | EPI_ISL_11380684 | 16/05/2021 | Africa / Mauritania / Nouackchott | Human | unknown | Female | 44 | Live | unknown | unknown | B.1.620 | G |
| hCoV-19/Mauritanie/CT552/2021 | EPI_ISL_11380685 | 28/02/2021 | Africa / Mauritania / Nouackchott | Human | unknown | Female | 44 | Released | unknown | unknown | B.1.620 | G |
| hCoV-19/Mauritanie/389154/2021 | EPI_ISL_11380686 | 30/05/2021 | Africa / Mauritania / Nouackchott | Human | unknown | Female | 34 | hospitalized | unknown | unknown | B.1.525 | G |
| hCoV-19/Mauritanie/395978/2021 | EPI_ISL_11380687 | 06/09/2021 | Africa / Mauritania / Nouackchott | Human | unknown | Male | 35 | hospitalized | unknown | unknown | AY.34.1 | GK |
| hCoV-19/Mauritanie/395968/2021 | EPI_ISL_11380688 | 07/09/2021 | Africa / Mauritania / Nouackchott | Human | unknown | Male | 39 | Hospitalized | unknown | unknown | AY.34.1 | GK |
| hCoV-19/Mauritanie/N035010/2021 | EPI_ISL_11380689 | 06/09/2021 | Africa / Mauritania / Nouackchott | Human | unknown | Female | 8 | Live | unknown | unknown | AY.34.1 | GK |
| hCoV-19/Mauritanie/397322/2021 | EPI_ISL_11380690 | 22/10/2021 | Africa / Mauritania / Nouackchott | Human | unknown | Female | 18 | Hospitalized | unknown | unknown | AY.34.1 | GK |
| hCoV-19/Mauritanie/397325/2021 | EPI_ISL_11380691 | 22/10/2021 | Africa / Mauritania / Nouackchott | Human | unknown | Male | 42 | Hospitalized | unknown | unknown | AY.34.1 | GK |
| hCoV-19/Mauritanie/396993/2021 | EPI_ISL_11380692 | 06/10/2021 | Africa / Mauritania / Nouackchott | Human | unknown | Male | 88 | Hospitalized | unknown | unknown | AY.34.1 | GK |
| hCoV-19/Mauritanie/N042493/2021 | EPI_ISL_11380693 | 06/10/2021 | Africa / Mauritania / Nouackchott | Human | unknown | Male | 27 | Live | unknown | unknown | AY.34.1 | GK |
| hCoV-19/Mauritanie/N038086/2021 | EPI_ISL_11380694 | 28/03/2021 | Africa / Mauritania / Nouackchott | Human | unknown | Female | 17 | Live | unknown | unknown | B.1.1.7 | GRY |
| hCoV-19/Mauritanie/INRSP51943/2021 | EPI_ISL_11380695 | 16/05/2021 | Africa / Mauritania / Nouackchott | Human | unknown | Male | 43 | Live | unknown | unknown | B.1.1.7 | GRY |
| hCoV-19/Mauritanie/INRSP57072/2021 | EPI_ISL_11380696 | 25/05/2021 | Africa / Mauritania / Nouackchott | Human | unknown | Male | 71 | Live | unknown | unknown | B.1.525 | G |
| hCoV-19/Mauritanie/397497/2021 | EPI_ISL_11380697 | 14/05/2021 | Africa / Mauritania / Nouackchott | Human | unknown | Male | 37 | Hospitalized | unknown | unknown | AY.34 | GK |
| hCoV-19/Mauritanie/389166/2021 | EPI_ISL_12316578 | 31/05/2021 | Africa / Mauritania / Nouackchott | Human | unknown | Female | 27 | Live | unknown | unknown | B.1.1.7 | GRY |
| hCoV-19/Mauritanie/388708/2021 | EPI_ISL_12316579 | 24/05/2021 | Africa / Mauritania / Nouackchott | Human | unknown | Male | 60 | Live | unknown | unknown | B.1.525 | G |
| hCoV-19/Mauritanie/387840/2021 | EPI_ISL_12316580 | 05/05/2021 | Africa / Mauritania / Guidimakha | Human | unknown | Female | 46 | Live | unknown | unknown | B.1.525 | G |
| hCoV-19/Mauritanie/387836/2021 | EPI_ISL_12316581 | 05/05/2021 | Africa / Mauritania / Guidimakha | Human | unknown | Male | 35 | Live | unknown | unknown | B.1.525 | G |
| hCoV-19/Mauritanie/387832/2021 | EPI_ISL_12316582 | 05/05/2021 | Africa / Mauritania / Guidimakha | Human | unknown | Male | 19 | Live | unknown | unknown | B.1.525 | G |
| hCoV-19/Mauritanie/394430/2021 | EPI_ISL_12316583 | 13/08/2021 | Africa / Mauritania / Nouackchott | Human | unknown | Female | 51 | Live | unknown | unknown | AY.34.1 | GK |
| hCoV-19/Mauritanie/395964/2021 | EPI_ISL_12316584 | 06/09/2021 | Africa / Mauritania / Nouackchott | Human | unknown | Male | 80 | Live | unknown | unknown | AY.34.1 | GK |
| hCoV-19/Mauritanie/388711/2021 | EPI_ISL_12316585 | 24/05/2021 | Africa / Mauritania / Nouackchott | Human | unknown | Male | 60 | Live | unknown | unknown | B.1.525 | G |
| hCoV-19/Mauritanie/387835/2021 | EPI_ISL_12316586 | 05/05/2021 | Africa / Mauritania / Guidimakha | Human | unknown | Female | 43 | Live | unknown | unknown | B.1.525 | G |
| hCoV-19/Mauritanie/387248/2021 | EPI_ISL_12316587 | 22/04/2021 | Africa / Mauritania / Nouackchott | Human | unknown | Male | 36 | Live | unknown | unknown | B.1.617.2 | GK |
| hCoV-19/Mauritanie/CT4368/2021 | EPI_ISL_12316588 | 05/11/2021 | Africa / Mauritania / Nouackchott | Human | unknown | Male | 45 | Live | unknown | unknown | AY.121 | GK |
| hCoV-19/Mauritanie/396382/2021 | EPI_ISL_12316589 | 15/09/2021 | Africa / Mauritania / Nouackchott | Human | unknown | Male | 90 | Live | unknown | unknown | AY.34.1 | GK |
| hCoV-19/Mauritanie/389153/2021 | EPI_ISL_12316590 | 30/05/2021 | Africa / Mauritania / Nouackchott | Human | unknown | Female | 61 | Live | unknown | unknown | B.1.525 | G |
| hCoV-19/Mauritanie/387742/2021 | EPI_ISL_12316591 | 03/05/2021 | Africa / Mauritania / Nouackchott | Human | unknown | Female | 49 | Live | unknown | unknown | B.1.620 | G |
| hCoV-19/Mauritanie/388131/2021 | EPI_ISL_12316592 | 11/05/2021 | Africa / Mauritania / Nouackchott | Human | unknown | Female | 47 | Live | unknown | unknown | B.1.525 | G |
| hCoV-19/Mauritanie/387833/2021 | EPI_ISL_12316593 | 05/05/2021 | Africa / Mauritania / Guidimakha | Human | unknown | Female | 60 | Live | unknown | unknown | B.1.525 | G |
| hCoV-19/Mauritanie/387817/2021 | EPI_ISL_12316594 | 04/05/2021 | Africa / Mauritania / Nouackchott | Human | unknown | Female | 66 | Live | unknown | unknown | B.1.525 | G |
| hCoV-19/Mauritanie/387741/2021 | EPI_ISL_12316595 | 03/05/2021 | Africa / Mauritania / Nouackchott | Human | unknown | Male | 3 | Live | unknown | unknown | B.1.620 | G |
| hCoV-19/Mauritanie/387454/2021 | EPI_ISL_12316596 | 27/04/2021 | Africa / Mauritania / Nouackchott | Human | unknown | Female | 59 | Live | unknown | unknown | B.1.525 | G |
| hCoV-19/Mauritanie/389673/2021 | EPI_ISL_12316597 | 08/06/2021 | Africa / Mauritania / Nouackchott | Human | unknown | Male | 52 | Live | unknown | unknown | B.1.525 | G |
| hCoV-19/Mauritanie/401093/2022 | EPI_ISL_14195951 | 10/05/2022 | Africa / Mauritania / GUI / ghabou | Human | unknown | Male | 51 | unknown | unknown | unknown | Unassigned | GRA |
| hCoV-19/Mauritanie/401094/2022 | EPI_ISL_14195952 | 12/05/2022 | Africa / Mauritania / ASS / Kiffa | Human | unknown | Male | 19 | unknown | unknown | unknown | BA.2 | GRA |
| hCoV-19/Mauritanie/401096/2022 | EPI_ISL_14195953 | 12/05/2022 | Africa / Mauritania / GUI / Ould Yenge | Human | unknown | Male | 4 | unknown | unknown | unknown | Unassigned | GRA |
| hCoV-19/Mauritanie/401095/2022 | EPI_ISL_14195954 | 12/05/2022 | Africa / Mauritania / ASS / Kiffa | Human | unknown | Male | 55 | unknown | unknown | unknown | Unassigned | GRA |
| hCoV-19/Mauritanie/401105/2022 | EPI_ISL_14195955 | 12/05/2022 | Africa / Mauritania / GUI / Ould Yenge | Human | unknown | Male | 14 | unknown | unknown | unknown | BA.2 | GRA |
| hCoV-19/Mauritanie/401104/2022 | EPI_ISL_14195956 | 12/05/2022 | Africa / Mauritania / GUI / Ould Yenge | Human | unknown | Male | 42 | unknown | unknown | unknown | Unassigned | GRA |
| hCoV-19/Mauritanie/401109/2022 | EPI_ISL_14195957 | 12/05/2022 | Africa / Mauritania / GUI / Ould Yenge | Human | unknown | Female | 50 | unknown | unknown | unknown | BA.2 | GRA |
